# Supplementary material for: Construction of an Exudative Age-Related Macular Degeneration Diagnostic and Therapeutic Molecular Network Using Multi-Layer Network Analysis, a Fuzzy Logic Model, and Deep Learning Techniques: Are Retinal and Brain Neurodegenerative Disorders Related?
Source: Pharmaceuticals (Basel). 2023 Nov 2;16(11):1555. doi: 10.3390/ph16111555 (PMC10674956; doi:10.3390/ph16111555)
Supplement: Supplementary file 1 [file pharmaceuticals-16-01555-s001.zip › Supplementary Tables.pdf]

Supplementary Table S1: the first list of genes involved in nAMD pathogenesis were identified from the MuST and DIAMOnD algorithms	
ABCA4	ATP binding cassette subfamily A member 4
VEGFA	vascular endothelial growth factor A
RAB1A	RAB1A, member RAS oncogene family
TNFRSF10A	tumor necrosis factor receptor superfamily member 10a
ERCC6	ERCC excision repair 6, chromatin remodeling factor
C1QTNF5	C1q and tumor necrosis factor related protein 5
MYC	v-myc avian myelocytomatosis viral oncogene homolog
NEFM	neurofilament, medium polypeptide
C9	complement component 9
IRAK1	interleukin 1 receptor associated kinase 1
C2	complement component 2
C3	complement component 3
NPM1	nucleophosmin (nucleolar phosphoprotein B23, numatrin)
TLR4	toll like receptor 4
SLC16A8	solute carrier family 16 member 8
HIF1A	hypoxia inducible factor 1 alpha subunit
TIMP3	TIMP metallopeptidase inhibitor 3
IQCB1	IQ motif containing B1
UBA52	ubiquitin A-52 residue ribosomal protein fusion product 1
RPGR	retinitis pigmentosa GTPase regulator
VWCE	von Willebrand factor C and EGF domains
CFH	complement factor H
NMNAT1	nicotinamide nucleotide adenylyltransferase 1
TGM2	transglutaminase 2
CFI	complement factor I
APOE	apolipoprotein E
COPS6	COP9 signalosome subunit 6
SQSTM1	sequestosome 1
SLC2A4	solute carrier family 2 member 4
CFB	complement factor B
KDM5B	lysine demethylase 5B
LTBP1	latent transforming growth factor beta binding protein 1
SERPINA1	serpin family A member 1
CD46	CD46 molecule
VCAN	versican
ANTXR2	anthrax toxin receptor 2
SNX27	sorting nexin family member 27
APP	amyloid beta precursor protein
P3H3	prolyl 3-hydroxylase 3
KLK3	kallikrein related peptidase 3

LAMA1	laminin subunit alpha 1
ADAMTS1	ADAM metallopeptidase with thrombospondin type 1 motif 1
SERPINA3	serpin family A member 3
VWF	von Willebrand factor
PDGFA	platelet derived growth factor subunit A
PDGFB	platelet derived growth factor subunit B
CST3	cystatin C
SERPINE2	serpin family E member 2
A2M	alpha-2-macroglobulin
PXN	paxillin
VTN	vitronectin
SERPINB6	serpin family B member 6
CX3CL1	C-X3-C motif chemokine ligand 1
CPB2	carboxypeptidase B2
COL18A1	collagen type XVIII alpha 1 chain
CP	ceruloplasmin
AMBP	alpha-1-microglobulin/bikunin precursor
FBN2	fibrillin 2
FBN1	fibrillin 1
CALR	calreticulin
SERPINE1	serpin family E member 1
CXCL12	C-X-C motif chemokine ligand 12
ITGA2	integrin subunit alpha 2
FCN2	ficolin 2
CTGF (CCN2)	connective tissue growth factor
FN1	fibronectin 1
DPT	dermatopontin
CD36	CD36 molecule
FCN1	ficolin 1
ACHE	acetylcholinesterase (Cartwright blood group)
MAG	myelin associated glycoprotein
ITGAM	integrin subunit alpha M
DEFA1B	defensin alpha 1B
COLGALT2	collagen beta(1-O)galactosyltransferase 2
CR1	complement component 3b/4b receptor 1 (Knops blood group)
SERPING1	serpin family G member 1
C1QA	complement component 1, q subcomponent, A chain
ITGA2B	integrin subunit alpha 2b
C1QB	complement component 1, q subcomponent, B chain
IGLL5	immunoglobulin lambda like polypeptide 5
C1QC	complement component 1, q subcomponent, C chain

C1R	complement C1r subcomponent
DDX19B	DEAD-box helicase 19B
C1S	complement component 1, s subcomponent
ITGA5	integrin subunit alpha 5
COL14A1	collagen type XIV alpha 1 chain
PF4	platelet factor 4
ITGB3	integrin subunit beta 3
CFP	complement factor properdin
SAA1	serum amyloid A1
FGA	fibrinogen alpha chain
C1QTNF2	C1q and tumor necrosis factor related protein 2
TNFAIP6	TNF alpha induced protein 6
KLK13	kallikrein related peptidase 13
MASP2	mannan binding lectin serine peptidase 2
CD44	CD44 molecule (Indian blood group)
C4A	complement component 4A (Rodgers blood group)
C4B	complement component 4B (Chido blood group)
C4BPA	complement component 4 binding protein alpha
MYOC	myocilin
ITGAV	integrin subunit alpha V
PROC	protein C, inactivator of coagulation factors Va and VIIIa
ITGB1	integrin subunit beta 1
C5	complement component 5
PROS1	protein S (alpha)
TFAP2C	transcription factor AP-2 gamma
COL4A4	collagen type IV alpha 4 chain
COL4A3	collagen type IV alpha 3 chain
COL4A2	collagen type IV alpha 2
FGF7	fibroblast growth factor 7
COL4A1	collagen type IV alpha 1 chain
COL3A1	collagen type III alpha 1 chain
COL2A1	collagen type II alpha 1 chain
HSPG2	heparan sulfate proteoglycan 2
ELN	elastin
FGF2	fibroblast growth factor 2
COL1A2	collagen type I alpha 2 chain
COL1A1	collagen type I alpha 1
FGB	fibrinogen beta chain
TF	transferrin
TFPI	tissue factor pathway inhibitor
TTR	transthyretin

KLK6	kallikrein related peptidase 6
COL9A1	collagen type IX alpha 1
COL8A2	collagen type VIII alpha 2
COL7A1	collagen type VII alpha 1
COL6A2	collagen type VI alpha 2
COL6A1	collagen type VI alpha 1
COL5A1	collagen type V alpha 1
COL4A6	collagen type IV alpha 6 chain
COL4A5	collagen type IV alpha 5 chain
LAMA3	laminin subunit alpha 3
MMP1	matrix metallopeptidase 1
MMP2	matrix metallopeptidase 2
MMP3	matrix metallopeptidase 3
MMP7	matrix metallopeptidase 7
MASP1	mannan binding lectin serine peptidase 1
MMP9	matrix metallopeptidase 9
MMP10	matrix metallopeptidase 10
TGFBI	transforming growth factor beta induced
DDX31	DEAD-box helicase 31
IGFBP3	insulin like growth factor binding protein 3
IGFBP2	insulin like growth factor binding protein 2
IGFBP1	insulin like growth factor binding protein 1
F5	coagulation factor V
HP	haptoglobin
TGFB1	transforming growth factor beta 1
IGF2	insulin like growth factor 2
AHSG	alpha 2-HS glycoprotein
LAMC1	laminin subunit gamma 1
F2	coagulation factor II, thrombin
BGN	biglycan
FGG	fibrinogen gamma chain
LAMA5	laminin subunit alpha 5
LAMB1	laminin subunit beta 1
THBS1	thrombospondin 1
THBD	thrombomodulin
PTPRK	protein tyrosine phosphatase, receptor type K
F13A1	coagulation factor XIII A chain
VKORC1	vitamin K epoxide reductase complex subunit 1
F11	coagulation factor XI
MIS12	MIS12, kinetochore complex component
C4B_2	complement component 4B (Chido blood group), copy 2

F10	coagulation factor X
IGFBP5	insulin like growth factor binding protein 5
F8	coagulation factor VIII
CA8	carbonic anhydrase 8
CD93	CD93 molecule
BMP1	bone morphogenetic protein 1
PRELP	proline and arginine rich end leucine rich repeat protein
PITX3	paired like homeodomain 3
CTRB1	chymotrypsinogen B1
GDPD1	glycerophosphodiester phosphodiesterase domain containing 1
PLA2G2A	phospholipase A2 group IIA
HPX	hemopexin
CTSL	cathepsin L
DCN	decorin
KLK1	kallikrein 1
CTSG	cathepsin G
KLK2	kallikrein related peptidase 2
ELANE	elastase, neutrophil expressed
MEP1A	meprin A subunit alpha
LCAT	lecithin-cholesterol acyltransferase
TIMP1	TIMP metallopeptidase inhibitor 1
ZNF558	zinc finger protein 558
HRG	histidine rich glycoprotein
HAPLN1	hyaluronan and proteoglycan link protein 1
KNG1	kininogen 1
CRP	C-reactive protein, pentraxin-related
HABP2	hyaluronan binding protein 2
MFAP2	microfibrillar associated protein 2
PLAT	plasminogen activator, tissue type
PLAU	plasminogen activator, urokinase
ECE1	endothelin converting enzyme 1
PLG	plasminogen
SERPINF2	serpin family F member 2
FBLN2	fibulin 2
COLQ	collagen-like tail subunit (single strand of homotrimer) of asymmetric acetylcholinesterase
NID2	nidogen 2
FBLN1	fibulin 1
LGALS3	galectin 3
FAM20C	family with sequence similarity 20 member C
PLOD1	procollagen-lysine,2-oxoglutarate 5-dioxygenase 1
MAPK6	mitogen-activated protein kinase 6

SERPIND1	serpin family D member 1
BCAN	brevican
DEFA1	defensin alpha 1
APCS	amyloid P component, serum
SPARC	secreted protein acidic and cysteine rich
SERPINA5	serpin family A member 5
LOX	lysyl oxidase
NID1	nidogen 1
LGALS8	galectin 8
LGALS9	galectin 9
MBL2	mannose binding lectin 2
SPP1	secreted phosphoprotein 1
LRP1	LDL receptor related protein 1
ORM1	orosomucoid 1
C1QTNF9	C1q and tumor necrosis factor related protein 9
ALB	albumin
APOA1	apolipoprotein A1
COLEC12	collectin subfamily member 12
APOA2	apolipoprotein A2
ALDH2	aldehyde dehydrogenase 2 family (mitochondrial)
MATN2	matrilin 2
PCOLCE	procollagen C-endopeptidase enhancer
APOC1	apolipoprotein C1
SERPINC1	serpin family C member 1
PTX3	pentraxin 3
OSM	oncostatin M

Supplementary Table S2: the second list of genes involved in nAMD disease were identified from the the valid databases	
VEGFA	vascular endothelial growth factor A [Source:HGNC Symbol;Acc:HGNC:12680]
CFB	complement factor B [Source:HGNC Symbol;Acc:HGNC:1037]
C3	complement component 3 [Source:HGNC Symbol;Acc:HGNC:1318]
ARMS2	age-related maculopathy susceptibility 2 [Source:HGNC Symbol;Acc:HGNC:32685]
C2	complement component 2 [Source:HGNC Symbol;Acc:HGNC:1248]
CFHR1	complement factor H related 1 [Source:HGNC Symbol;Acc:HGNC:4888]
APOE	apolipoprotein E [Source:HGNC Symbol;Acc:HGNC:613]
HTRA1	HtrA serine peptidase 1 [Source:HGNC Symbol;Acc:HGNC:9476]
CFI	complement factor I [Source:HGNC Symbol;Acc:HGNC:5394]
CFH	complement factor H [Source:HGNC Symbol;Acc:HGNC:4883]
TIMP3	TIMP metallopeptidase inhibitor 3 [Source:HGNC Symbol;Acc:HGNC:11822]
HMCN1	hemicentin 1 [Source:HGNC Symbol;Acc:HGNC:19194]
CFHR3	complement factor H related 3 [Source:HGNC Symbol;Acc:HGNC:16980]
RPGR	retinitis pigmentosa GTPase regulator [Source:HGNC Symbol;Acc:HGNC:10295]
C9	complement component 9 [Source:HGNC Symbol;Acc:HGNC:1358]
CFHR4	complement factor H related 4 [Source:HGNC Symbol;Acc:HGNC:16979]
SLC16A8	solute carrier family 16 member 8 [Source:HGNC Symbol;Acc:HGNC:16270]
CFHR2	complement factor H related 2 [Source:HGNC Symbol;Acc:HGNC:4890]
RLBP1	retinaldehyde binding protein 1 [Source:HGNC Symbol;Acc:HGNC:10024]
RAX2	retina and anterior neural fold homeobox 2 [Source:HGNC Symbol;Acc:HGNC:18286]
C1QTNF5	C1q and tumor necrosis factor related protein 5 [Source:HGNC Symbol;Acc:HGNC:14344]
TLR4	toll like receptor 4 [Source:HGNC Symbol;Acc:HGNC:11850]
FBLN5	fibulin 5 [Source:HGNC Symbol;Acc:HGNC:3602]
SQSTM1	sequestosome 1 [Source:HGNC Symbol;Acc:HGNC:11280]
ERCC6	ERCC excision repair 6, chromatin remodeling factor [Source:HGNC Symbol;Acc:HGNC:3438]
TNFRSF10A	tumor necrosis factor receptor superfamily member 10a [Source:HGNC Symbol;Acc:HGNC:11904]
PARP12	poly(ADP-ribose) polymerase family member 12 [Source:HGNC Symbol;Acc:HGNC:21919]
NMNAT1	nicotinamide nucleotide adenylyltransferase 1 [Source:HGNC Symbol;Acc:HGNC:17877]
HIC1	hypermethylated in cancer 1 [Source:HGNC Symbol;Acc:HGNC:4909]
CCR2	C-C motif chemokine receptor 2 [Source:HGNC Symbol;Acc:HGNC:1603]
CD46	CD46 molecule [Source:HGNC Symbol;Acc:HGNC:6953]
MDM1	Mdm1 nuclear protein [Source:HGNC Symbol;Acc:HGNC:29917]
ABCA1	ATP binding cassette subfamily A member 1 [Source:HGNC Symbol;Acc:HGNC:29]
LIPC	lipase C, hepatic type [Source:HGNC Symbol;Acc:HGNC:6619]
CCL13	C-C motif chemokine ligand 13 [Source:HGNC Symbol;Acc:HGNC:10611]
CETP	cholesteryl ester transfer protein [Source:HGNC Symbol;Acc:HGNC:1869]
PLEKHA1	pleckstrin homology domain containing A1 [Source:HGNC Symbol;Acc:HGNC:14335]
SKIV2L	Ski2 like RNA helicase [Source:HGNC Symbol;Acc:HGNC:10898]
CST3	cystatin C [Source:HGNC Symbol;Acc:HGNC:2475]

COL18A1	collagen type XVIII alpha 1 chain [Source:HGNC Symbol;Acc:HGNC:2195]
RAD51B	RAD51 paralog B [Source:HGNC Symbol;Acc:HGNC:9822]
TNXB	tenascin XB [Source:HGNC Symbol;Acc:HGNC:11976]
COL8A1	collagen type VIII alpha 1 [Source:HGNC Symbol;Acc:HGNC:2215]
ALDH3A2	aldehyde dehydrogenase 3 family member A2 [Source:HGNC Symbol;Acc:HGNC:403]
TGFBR1	transforming growth factor beta receptor 1 [Source:HGNC Symbol;Acc:HGNC:11772]
TOMM40	translocase of outer mitochondrial membrane 40 [Source:HGNC Symbol;Acc:HGNC:18001]
RDH5	retinol dehydrogenase 5 [Source:HGNC Symbol;Acc:HGNC:9940]
CFHR5	complement factor H related 5 [Source:HGNC Symbol;Acc:HGNC:24668]
B3GLCT	beta 3-glucosyltransferase [Source:HGNC Symbol;Acc:HGNC:20207]
REST	RE1 silencing transcription factor [Source:HGNC Symbol;Acc:HGNC:9966]
NELFE	negative elongation factor complex member E [Source:HGNC Symbol;Acc:HGNC:13974]
ATXN7	ataxin 7 [Source:HGNC Symbol;Acc:HGNC:10560]
SCAPER	S-phase cyclin A associated protein in the ER [Source:HGNC Symbol;Acc:HGNC:13081]
PILRA	paired immunoglobin like type 2 receptor alpha [Source:HGNC Symbol;Acc:HGNC:20396]
ACAD10	acyl-CoA dehydrogenase family member 10 [Source:HGNC Symbol;Acc:HGNC:21597]
PITPNM3	PITPNM family member 3 [Source:HGNC Symbol;Acc:HGNC:21043]
SYN3	synapsin III [Source:HGNC Symbol;Acc:HGNC:11496]
FBN2	fibrillin 2 [Source:HGNC Symbol;Acc:HGNC:3604]
ERCC2	ERCC excision repair 2, TFIIH core complex helicase subunit [Source:HGNC Symbol;Acc:HGNC:3434]
COL10A1	collagen type X alpha 1 chain [Source:HGNC Symbol;Acc:HGNC:2185]
TRPM1	transient receptor potential cation channel subfamily M member 1 [Source:HGNC Symbol;Acc:HGNC:7146]
NOTCH4	notch 4 [Source:HGNC Symbol;Acc:HGNC:7884]
RNF113A	ring finger protein 113A [Source:HGNC Symbol;Acc:HGNC:12974]
SPG11	spastic paraplegia 11 (autosomal recessive) [Source:HGNC Symbol;Acc:HGNC:11226]
PLXNA2	plexin A2 [Source:HGNC Symbol;Acc:HGNC:9100]
TRAF3IP1	TRAF3 interacting protein 1 [Source:HGNC Symbol;Acc:HGNC:17861]
AMD1	adenosylmethionine decarboxylase 1 [Source:HGNC Symbol;Acc:HGNC:457]
PPT1	palmitoyl-protein thioesterase 1 [Source:HGNC Symbol;Acc:HGNC:9325]
FUT6	fucosyltransferase 6 [Source:HGNC Symbol;Acc:HGNC:4017]
PRCD	progressive rod-cone degeneration [Source:HGNC Symbol;Acc:HGNC:32528]
ZFYVE26	zinc finger FYVE-type containing 26 [Source:HGNC Symbol;Acc:HGNC:20761]
SRPK2	SRSF protein kinase 2 [Source:HGNC Symbol;Acc:HGNC:11306]
DPF3	double PHD fingers 3 [Source:HGNC Symbol;Acc:HGNC:17427]
PLA2G12A	phospholipase A2 group XIIA [Source:HGNC Symbol;Acc:HGNC:18554]
ANO10	anoctamin 10 [Source:HGNC Symbol;Acc:HGNC:25519]
NT5DC1	5'-nucleotidase domain containing 1 [Source:HGNC Symbol;Acc:HGNC:21556]
EFEMP1	EGF containing fibulin like extracellular matrix protein 1 [Source:HGNC Symbol;Acc:HGNC:3218]
RPGRIP1	retinitis pigmentosa GTPase regulator interacting protein 1 [Source:HGNC Symbol;Acc:HGNC:13436]

SERPINF1	serpin family F member 1 [Source:HGNC Symbol;Acc:HGNC:8824]
BBS9	Bardet-Biedl syndrome 9 [Source:HGNC Symbol;Acc:HGNC:30000]
ABCC6	ATP binding cassette subfamily C member 6 [Source:HGNC Symbol;Acc:HGNC:57]
KDR	kinase insert domain receptor [Source:HGNC Symbol;Acc:HGNC:6307]
HS3ST4	heparan sulfate-glucosamine 3-sulfotransferase 4 [Source:HGNC Symbol;Acc:HGNC:5200]
NECTIN2	nectin cell adhesion molecule 2 [Source:HGNC Symbol;Acc:HGNC:9707]
SEMA4A	semaphorin 4A [Source:HGNC Symbol;Acc:HGNC:10729]
GTF2H5	general transcription factor IIH subunit 5 [Source:HGNC Symbol;Acc:HGNC:21157]
RPE	ribulose-5-phosphate-3-epimerase [Source:HGNC Symbol;Acc:HGNC:10293]
XYLT1	xylosyltransferase 1 [Source:HGNC Symbol;Acc:HGNC:15516]
RREB1	ras responsive element binding protein 1 [Source:HGNC Symbol;Acc:HGNC:10449]
CHST6	carbohydrate sulfotransferase 6 [Source:HGNC Symbol;Acc:HGNC:6938]
CXCL8	C-X-C motif chemokine ligand 8 [Source:HGNC Symbol;Acc:HGNC:6025]
ARHGAP21	Rho GTPase activating protein 21 [Source:HGNC Symbol;Acc:HGNC:23725]
MARK4	microtubule affinity regulating kinase 4 [Source:HGNC Symbol;Acc:HGNC:13538]
TMEM97	transmembrane protein 97 [Source:HGNC Symbol;Acc:HGNC:28106]
TST	thiosulfate sulfurtransferase [Source:HGNC Symbol;Acc:HGNC:12388]
IL6	interleukin 6 [Source:HGNC Symbol;Acc:HGNC:6018]
GTF2E2	general transcription factor IIE subunit 2 [Source:HGNC Symbol;Acc:HGNC:4651]
SERPING1	serpin family G member 1 [Source:HGNC Symbol;Acc:HGNC:1228]
XYLT2	xylosyltransferase 2 [Source:HGNC Symbol;Acc:HGNC:15517]
PBX2	PBX homeobox 2 [Source:HGNC Symbol;Acc:HGNC:8633]
NFE2L2	nuclear factor, erythroid 2 like 2 [Source:HGNC Symbol;Acc:HGNC:7782]
KCNT2	potassium sodium-activated channel subfamily T member 2 [Source:HGNC Symbol;Acc:HGNC:18866]
MYO5A	myosin VA [Source:HGNC Symbol;Acc:HGNC:7602]
CCL2	C-C motif chemokine ligand 2 [Source:HGNC Symbol;Acc:HGNC:10618]
FTCD	formimidoyltransferase cyclodeaminase [Source:HGNC Symbol;Acc:HGNC:3974]
CNN2	calponin 2 [Source:HGNC Symbol;Acc:HGNC:2156]
MPLKIP	M-phase specific PLK1 interacting protein [Source:HGNC Symbol;Acc:HGNC:16002]
ABHD2	abhydrolase domain containing 2 [Source:HGNC Symbol;Acc:HGNC:18717]
TRPM3	transient receptor potential cation channel subfamily M member 3 [Source:HGNC Symbol;Acc:HGNC:17992]
CLN3	ceroid-lipofuscinosis, neuronal 3 [Source:HGNC Symbol;Acc:HGNC:2074]
CX3CR1	C-X3-C motif chemokine receptor 1 [Source:HGNC Symbol;Acc:HGNC:2558]
NPLOC4	NPL4 homolog, ubiquitin recognition factor [Source:HGNC Symbol;Acc:HGNC:18261]
CYP21A2	cytochrome P450 family 21 subfamily A member 2 [Source:HGNC Symbol;Acc:HGNC:2600]
HERC1	HECT and RLD domain containing E3 ubiquitin protein ligase family member 1 [Source:HGNC Symbol;Acc:HGNC:4867]
ATP6V0D1	ATPase H+ transporting V0 subunit d1 [Source:HGNC Symbol;Acc:HGNC:13724]
SYCE1	synaptonemal complex central element protein 1 [Source:HGNC Symbol;Acc:HGNC:28852]
BBS5	Bardet-Biedl syndrome 5 [Source:HGNC Symbol;Acc:HGNC:970]

TTC8	tetratricopeptide repeat domain 8 [Source:HGNC Symbol;Acc:HGNC:20087]
CRP	C-reactive protein, pentraxin-related [Source:HGNC Symbol;Acc:HGNC:2367]
ERCC3	ERCC excision repair 3, TFIIH core complex helicase subunit [Source:HGNC Symbol;Acc:HGNC:3435]
CEP78	centrosomal protein 78 [Source:HGNC Symbol;Acc:HGNC:25740]
STK19	serine/threonine kinase 19 [Source:HGNC Symbol;Acc:HGNC:11398]
NLRP3	NLR family pyrin domain containing 3 [Source:HGNC Symbol;Acc:HGNC:16400]
EXOC3L2	exocyst complex component 3-like 2 [Source:HGNC Symbol;Acc:HGNC:30162]
COL4A3	collagen type IV alpha 3 chain [Source:HGNC Symbol;Acc:HGNC:2204]
ALDH1A2	aldehyde dehydrogenase 1 family member A2 [Source:HGNC Symbol;Acc:HGNC:15472]
TLR3	toll like receptor 3 [Source:HGNC Symbol;Acc:HGNC:11849]
GABPA	GA binding protein transcription factor alpha subunit [Source:HGNC Symbol;Acc:HGNC:4071]
MMP2	matrix metallopeptidase 2 [Source:HGNC Symbol;Acc:HGNC:7166]
IL1B	interleukin 1 beta [Source:HGNC Symbol;Acc:HGNC:5992]
HIF1A	hypoxia inducible factor 1 alpha subunit [Source:HGNC Symbol;Acc:HGNC:4910]
FHL1	four and a half LIM domains 1 [Source:HGNC Symbol;Acc:HGNC:3702]
LPL	lipoprotein lipase [Source:HGNC Symbol;Acc:HGNC:6677]
FLT1	fms related tyrosine kinase 1 [Source:HGNC Symbol;Acc:HGNC:3763]
SOD2	superoxide dismutase 2, mitochondrial [Source:HGNC Symbol;Acc:HGNC:11180]
TNF	tumor necrosis factor [Source:HGNC Symbol;Acc:HGNC:11892]
APOB	apolipoprotein B [Source:HGNC Symbol;Acc:HGNC:603]
PON1	paraoxonase 1 [Source:HGNC Symbol;Acc:HGNC:9204]
MMP9	matrix metallopeptidase 9 [Source:HGNC Symbol;Acc:HGNC:7176]
PRPH	peripherin [Source:HGNC Symbol;Acc:HGNC:9461]
GSTM1	glutathione S-transferase mu 1 [Source:HGNC Symbol;Acc:HGNC:4632]
CD36	CD36 molecule [Source:HGNC Symbol;Acc:HGNC:1663]
AKR1A1	aldo-keto reductase family 1 member A1 [Source:HGNC Symbol;Acc:HGNC:380]
IL17A	interleukin 17A [Source:HGNC Symbol;Acc:HGNC:5981]
ACE	angiotensin I converting enzyme [Source:HGNC Symbol;Acc:HGNC:2707]
SOD1	superoxide dismutase 1, soluble [Source:HGNC Symbol;Acc:HGNC:11179]
ARMC9	armadillo repeat containing 9 [Source:HGNC Symbol;Acc:HGNC:20730]
GSTT1	glutathione S-transferase theta 1 [Source:HGNC Symbol;Acc:HGNC:4641]
RS1	retinoschisin 1 [Source:HGNC Symbol;Acc:HGNC:10457]
TLR2	toll like receptor 2 [Source:HGNC Symbol;Acc:HGNC:11848]
PPARGC1A	PPARG coactivator 1 alpha [Source:HGNC Symbol;Acc:HGNC:9237]
CP	ceruloplasmin [Source:HGNC Symbol;Acc:HGNC:2295]
MME	membrane metallo-endopeptidase [Source:HGNC Symbol;Acc:HGNC:7154]
FGF2	fibroblast growth factor 2 [Source:HGNC Symbol;Acc:HGNC:3676]
IL18	interleukin 18 [Source:HGNC Symbol;Acc:HGNC:5986]
SMUG1	single-strand-selective monofunctional uracil-DNA glycosylase 1 [Source:HGNC Symbol;Acc:HGNC:17148]
PPARA	peroxisome proliferator activated receptor alpha [Source:HGNC Symbol;Acc:HGNC:9232]

PGF	placental growth factor [Source:HGNC Symbol;Acc:HGNC:8893]
CD59	CD59 molecule [Source:HGNC Symbol;Acc:HGNC:1689]
SIRT1	sirtuin 1 [Source:HGNC Symbol;Acc:HGNC:14929]
SLC2A1	solute carrier family 2 member 1 [Source:HGNC Symbol;Acc:HGNC:11005]
ABCG1	ATP binding cassette subfamily G member 1 [Source:HGNC Symbol;Acc:HGNC:73]
ADM	adrenomedullin [Source:HGNC Symbol;Acc:HGNC:259]
AHR	aryl hydrocarbon receptor [Source:HGNC Symbol;Acc:HGNC:348]
CRX	cone-rod homeobox [Source:HGNC Symbol;Acc:HGNC:2383]
CRYAB	crystallin alpha B [Source:HGNC Symbol;Acc:HGNC:2389]
SGCD	sarcoglycan delta [Source:HGNC Symbol;Acc:HGNC:10807]
ELN	elastin [Source:HGNC Symbol;Acc:HGNC:3327]
SUCLA2	succinate-CoA ligase ADP-forming beta subunit [Source:HGNC Symbol;Acc:HGNC:11448]
IGF1	insulin like growth factor 1 [Source:HGNC Symbol;Acc:HGNC:5464]
MAPK3	mitogen-activated protein kinase 3 [Source:HGNC Symbol;Acc:HGNC:6877]
GSTP1	glutathione S-transferase pi 1 [Source:HGNC Symbol;Acc:HGNC:4638]
TF	transferrin [Source:HGNC Symbol;Acc:HGNC:11740]
HPGDS	hematopoietic prostaglandin D synthase [Source:HGNC Symbol;Acc:HGNC:17890]
IL17RC	interleukin 17 receptor C [Source:HGNC Symbol;Acc:HGNC:18358]
BDNF	brain derived neurotrophic factor [Source:HGNC Symbol;Acc:HGNC:1033]
MBL2	mannose binding lectin 2 [Source:HGNC Symbol;Acc:HGNC:6922]
OGG1	8-oxoguanine DNA glycosylase [Source:HGNC Symbol;Acc:HGNC:8125]
CXCL10	C-X-C motif chemokine ligand 10 [Source:HGNC Symbol;Acc:HGNC:10637]
CCR3	C-C motif chemokine receptor 3 [Source:HGNC Symbol;Acc:HGNC:1604]
F3	coagulation factor III, tissue factor [Source:HGNC Symbol;Acc:HGNC:3541]
RENBP	renin binding protein [Source:HGNC Symbol;Acc:HGNC:9959]
ADIPOQ	adiponectin, C1Q and collagen domain containing [Source:HGNC Symbol;Acc:HGNC:13633]
HLA-C	major histocompatibility complex, class I, C [Source:HGNC Symbol;Acc:HGNC:4933]
BMP4	bone morphogenetic protein 4 [Source:HGNC Symbol;Acc:HGNC:1071]
PTX3	pentraxin 3 [Source:HGNC Symbol;Acc:HGNC:9692]
P2RX7	purinergic receptor P2X 7 [Source:HGNC Symbol;Acc:HGNC:8537]
SCARB1	scavenger receptor class B member 1 [Source:HGNC Symbol;Acc:HGNC:1664]
CX3CL1	C-X3-C motif chemokine ligand 1 [Source:HGNC Symbol;Acc:HGNC:10647]
DICER1	dicer 1, ribonuclease III [Source:HGNC Symbol;Acc:HGNC:17098]
GSTK1	glutathione S-transferase kappa 1 [Source:HGNC Symbol;Acc:HGNC:16906]
PIK3CB	phosphatidylinositol-4,5-bisphosphate 3-kinase catalytic subunit beta [Source:HGNC Symbol;Acc:HGNC:8976]
IL10	interleukin 10 [Source:HGNC Symbol;Acc:HGNC:5962]
ACAT1	acetyl-CoA acetyltransferase 1 [Source:HGNC Symbol;Acc:HGNC:93]
IL33	interleukin 33 [Source:HGNC Symbol;Acc:HGNC:16028]
TNMD	tenomodulin [Source:HGNC Symbol;Acc:HGNC:17757]
NUP62	nucleoporin 62 [Source:HGNC Symbol;Acc:HGNC:8066]
LAMP1	lysosomal associated membrane protein 1 [Source:HGNC Symbol;Acc:HGNC:6499]

FTMT	ferritin mitochondrial [Source:HGNC Symbol;Acc:HGNC:17345]
MUTYH	mutY DNA glycosylase [Source:HGNC Symbol;Acc:HGNC:7527]
ATM	ATM serine/threonine kinase [Source:HGNC Symbol;Acc:HGNC:795]
CLU	clusterin [Source:HGNC Symbol;Acc:HGNC:2095]
OCA2	OCA2 melanosomal transmembrane protein [Source:HGNC Symbol;Acc:HGNC:8101]
OTX2	orthodenticle homeobox 2 [Source:HGNC Symbol;Acc:HGNC:8522]
SERPINE1	serpin family E member 1 [Source:HGNC Symbol;Acc:HGNC:8583]
DCTN4	dynactin subunit 4 [Source:HGNC Symbol;Acc:HGNC:15518]
PNPLA2	patatin like phospholipase domain containing 2 [Source:HGNC Symbol;Acc:HGNC:30802]
CFP	complement factor properdin [Source:HGNC Symbol;Acc:HGNC:8864]
PIK3CA	phosphatidylinositol-4,5-bisphosphate 3-kinase catalytic subunit alpha [Source:HGNC Symbol;Acc:HGNC:8975]
PIK3CD	phosphatidylinositol-4,5-bisphosphate 3-kinase catalytic subunit delta [Source:HGNC Symbol;Acc:HGNC:8977]
PIK3CG	phosphatidylinositol-4,5-bisphosphate 3-kinase catalytic subunit gamma [Source:HGNC Symbol;Acc:HGNC:8978]
SUCNR1	succinate receptor 1 [Source:HGNC Symbol;Acc:HGNC:4542]
PLG	plasminogen [Source:HGNC Symbol;Acc:HGNC:9071]
POLR2B	polymerase (RNA) II subunit B [Source:HGNC Symbol;Acc:HGNC:9188]
MAPK1	mitogen-activated protein kinase 1 [Source:HGNC Symbol;Acc:HGNC:6871]
MT2A	metallothionein 2A [Source:HGNC Symbol;Acc:HGNC:7406]
RGS10	regulator of G-protein signaling 10 [Source:HGNC Symbol;Acc:HGNC:9992]
RORA	RAR related orphan receptor A [Source:HGNC Symbol;Acc:HGNC:10258]
GDF6	growth differentiation factor 6 [Source:HGNC Symbol;Acc:HGNC:4221]
MYOM2	myomesin 2 [Source:HGNC Symbol;Acc:HGNC:7614]
CHI3L1	chitinase 3 like 1 [Source:HGNC Symbol;Acc:HGNC:1932]
CXCR5	C-X-C motif chemokine receptor 5 [Source:HGNC Symbol;Acc:HGNC:1060]
LRP6	LDL receptor related protein 6 [Source:HGNC Symbol;Acc:HGNC:6698]
DAPL1	death associated protein like 1 [Source:HGNC Symbol;Acc:HGNC:21490]
SELE	selectin E [Source:HGNC Symbol;Acc:HGNC:10718]
FKBPL	FK506 binding protein like [Source:HGNC Symbol;Acc:HGNC:13949]
CXCL12	C-X-C motif chemokine ligand 12 [Source:HGNC Symbol;Acc:HGNC:10672]
CXCL11	C-X-C motif chemokine ligand 11 [Source:HGNC Symbol;Acc:HGNC:10638]
MT-ND2	mitochondrially encoded NADH:ubiquinone oxidoreductase core subunit 2 [Source:HGNC Symbol;Acc:HGNC:7456]
MAN2B1	mannosidase alpha class 2B member 1 [Source:HGNC Symbol;Acc:HGNC:6826]
KHDRBS1	KH RNA binding domain containing, signal transduction associated 1 [Source:HGNC Symbol;Acc:HGNC:18116]
POSTN	periostin [Source:HGNC Symbol;Acc:HGNC:16953]
MEFV	Mediterranean fever [Source:HGNC Symbol;Acc:HGNC:6998]
PPARG	peroxisome proliferator activated receptor gamma [Source:HGNC Symbol;Acc:HGNC:9236]
IGFBP7	insulin like growth factor binding protein 7 [Source:HGNC Symbol;Acc:HGNC:5476]
AGBL4	ATP/GTP binding protein-like 4 [Source:HGNC Symbol;Acc:HGNC:25892]

SETD2	SET domain containing 2 [Source:HGNC Symbol;Acc:HGNC:18420]
DECR1	2,4-dienoyl-CoA reductase 1, mitochondrial [Source:HGNC Symbol;Acc:HGNC:2753]
PDE5A	phosphodiesterase 5A [Source:HGNC Symbol;Acc:HGNC:8784]
GSTM5	glutathione S-transferase mu 5 [Source:HGNC Symbol;Acc:HGNC:4637]
AIF1	allograft inflammatory factor 1 [Source:HGNC Symbol;Acc:HGNC:352]
GTF2H1	general transcription factor IIH subunit 1 [Source:HGNC Symbol;Acc:HGNC:4655]
NOA1	nitric oxide associated 1 [Source:HGNC Symbol;Acc:HGNC:28473]
SLCO6A1	solute carrier organic anion transporter family member 6A1 [Source:HGNC Symbol;Acc:HGNC:23613]
HLA-B	major histocompatibility complex, class I, B [Source:HGNC Symbol;Acc:HGNC:4932]
VTN	vitronectin [Source:HGNC Symbol;Acc:HGNC:12724]
ANPEP	alanyl aminopeptidase, membrane [Source:HGNC Symbol;Acc:HGNC:500]
VLDLR	very low density lipoprotein receptor [Source:HGNC Symbol;Acc:HGNC:12698]
CAT	catalase [Source:HGNC Symbol;Acc:HGNC:1516]
PAGR1	PAXIP1 associated glutamate rich protein 1 [Source:HGNC Symbol;Acc:HGNC:28707]
AGER	advanced glycosylation end product-specific receptor [Source:HGNC Symbol;Acc:HGNC:320]
CTRB1	chymotrypsinogen B1 [Source:HGNC Symbol;Acc:HGNC:2521]
UNG	uracil DNA glycosylase [Source:HGNC Symbol;Acc:HGNC:12572]
CYP27A1	cytochrome P450 family 27 subfamily A member 1 [Source:HGNC Symbol;Acc:HGNC:2605]
ANG	angiogenin [Source:HGNC Symbol;Acc:HGNC:483]
GAS6	growth arrest specific 6 [Source:HGNC Symbol;Acc:HGNC:4168]
CD55	CD55 molecule (Cromer blood group) [Source:HGNC Symbol;Acc:HGNC:2665]
FGFR1	fibroblast growth factor receptor 1 [Source:HGNC Symbol;Acc:HGNC:3688]
NAT2	N-acetyltransferase 2 (arylamine N-acetyltransferase) [Source:HGNC Symbol;Acc:HGNC:7646]
VWF	von Willebrand factor [Source:HGNC Symbol;Acc:HGNC:12726]
TSPO	translocator protein [Source:HGNC Symbol;Acc:HGNC:1158]
HP	haptoglobin [Source:HGNC Symbol;Acc:HGNC:5141]
TGFB1	transforming growth factor beta 1 [Source:HGNC Symbol;Acc:HGNC:11766]
ALB	albumin [Source:HGNC Symbol;Acc:HGNC:399]
MFRP	membrane frizzled-related protein [Source:HGNC Symbol;Acc:HGNC:18121]
XRCC1	X-ray repair cross complementing 1 [Source:HGNC Symbol;Acc:HGNC:12828]
CFD	complement factor D [Source:HGNC Symbol;Acc:HGNC:2771]
DIAPH2	diaphanous related formin 2 [Source:HGNC Symbol;Acc:HGNC:2877]
TFR2	transferrin receptor 2 [Source:HGNC Symbol;Acc:HGNC:11762]
HMOX2	heme oxygenase 2 [Source:HGNC Symbol;Acc:HGNC:5014]
CASP1	caspase 1 [Source:HGNC Symbol;Acc:HGNC:1499]
HLA-DQB1	major histocompatibility complex, class II, DQ beta 1 [Source:HGNC Symbol;Acc:HGNC:4944]
PEA15	phosphoprotein enriched in astrocytes 15 [Source:HGNC Symbol;Acc:HGNC:8822]
HLA-G	major histocompatibility complex, class I, G [Source:HGNC Symbol;Acc:HGNC:4964]
GALNS	galactosamine (N-acetyl)-6-sulfatase [Source:HGNC Symbol;Acc:HGNC:4122]
TIMP1	TIMP metallopeptidase inhibitor 1 [Source:HGNC Symbol;Acc:HGNC:11820]
EPHX1	epoxide hydrolase 1 [Source:HGNC Symbol;Acc:HGNC:3401]

FSCN2	fascin actin-bundling protein 2, retinal [Source:HGNC Symbol;Acc:HGNC:3960]
GAST	gastrin [Source:HGNC Symbol;Acc:HGNC:4164]
IL21	interleukin 21 [Source:HGNC Symbol;Acc:HGNC:6005]
AOC3	amine oxidase, copper containing 3 [Source:HGNC Symbol;Acc:HGNC:550]
CACNA1C	calcium voltage-gated channel subunit alpha1 C [Source:HGNC Symbol;Acc:HGNC:1390]
GRAP2	GRB2-related adaptor protein 2 [Source:HGNC Symbol;Acc:HGNC:4563]
ADIPOR2	adiponectin receptor 2 [Source:HGNC Symbol;Acc:HGNC:24041]
CD34	CD34 molecule [Source:HGNC Symbol;Acc:HGNC:1662]
EDF1	endothelial differentiation related factor 1 [Source:HGNC Symbol;Acc:HGNC:3164]
ALDH5A1	aldehyde dehydrogenase 5 family member A1 [Source:HGNC Symbol;Acc:HGNC:408]
CXCR4	C-X-C motif chemokine receptor 4 [Source:HGNC Symbol;Acc:HGNC:2561]
ABCG2	ATP binding cassette subfamily G member 2 (Junior blood group) [Source:HGNC Symbol;Acc:HGNC:74]
ITM2B	integral membrane protein 2B [Source:HGNC Symbol;Acc:HGNC:6174]
REG1A	regenerating family member 1 alpha [Source:HGNC Symbol;Acc:HGNC:9951]
RFC2	replication factor C subunit 2 [Source:HGNC Symbol;Acc:HGNC:9970]
BCL2	B-cell CLL/lymphoma 2 [Source:HGNC Symbol;Acc:HGNC:990]
FADS2	fatty acid desaturase 2 [Source:HGNC Symbol;Acc:HGNC:3575]
IL27RA	interleukin 27 receptor subunit alpha [Source:HGNC Symbol;Acc:HGNC:17290]
RAP1A	RAP1A, member of RAS oncogene family [Source:HGNC Symbol;Acc:HGNC:9855]
AIMP2	aminoacyl tRNA synthetase complex interacting multifunctional protein 2 [Source:HGNC Symbol;Acc:HGNC:20609]
HDAC11	histone deacetylase 11 [Source:HGNC Symbol;Acc:HGNC:19086]
TLR10	toll like receptor 10 [Source:HGNC Symbol;Acc:HGNC:15634]
CAPG	capping actin protein, gelsolin like [Source:HGNC Symbol;Acc:HGNC:1474]
HDAC9	histone deacetylase 9 [Source:HGNC Symbol;Acc:HGNC:14065]
MASP1	mannan binding lectin serine peptidase 1 [Source:HGNC Symbol;Acc:HGNC:6901]
KEAP1	kelch like ECH associated protein 1 [Source:HGNC Symbol;Acc:HGNC:23177]
XCL1	X-C motif chemokine ligand 1 [Source:HGNC Symbol;Acc:HGNC:10645]
FZD4	frizzled class receptor 4 [Source:HGNC Symbol;Acc:HGNC:4042]
MYDGF	myeloid derived growth factor [Source:HGNC Symbol;Acc:HGNC:16948]
MAPK8	mitogen-activated protein kinase 8 [Source:HGNC Symbol;Acc:HGNC:6881]
SESN2	sestrin 2 [Source:HGNC Symbol;Acc:HGNC:20746]
CASP5	caspase 5 [Source:HGNC Symbol;Acc:HGNC:1506]
APOM	apolipoprotein M [Source:HGNC Symbol;Acc:HGNC:13916]
ZEB2	zinc finger E-box binding homeobox 2 [Source:HGNC Symbol;Acc:HGNC:14881]
CASP8	caspase 8 [Source:HGNC Symbol;Acc:HGNC:1509]
FGD6	FYVE, RhoGEF and PH domain containing 6 [Source:HGNC Symbol;Acc:HGNC:21740]
HEPH	hephaestin [Source:HGNC Symbol;Acc:HGNC:4866]
PROK1	prokineticin 1 [Source:HGNC Symbol;Acc:HGNC:18454]
FNDC1	fibronectin type III domain containing 1 [Source:HGNC Symbol;Acc:HGNC:21184]
RAD51	RAD51 recombinase [Source:HGNC Symbol;Acc:HGNC:9817]

CALCR	calcitonin receptor [Source:HGNC Symbol;Acc:HGNC:1440]
SCD5	stearoyl-CoA desaturase 5 [Source:HGNC Symbol;Acc:HGNC:21088]
CTNNB1	catenin beta 1 [Source:HGNC Symbol;Acc:HGNC:2514]
TBPL1	TATA-box binding protein like 1 [Source:HGNC Symbol;Acc:HGNC:11589]
HAMP	hepcidin antimicrobial peptide [Source:HGNC Symbol;Acc:HGNC:15598]
CD40	CD40 molecule [Source:HGNC Symbol;Acc:HGNC:11919]
LRRTM4	leucine rich repeat transmembrane neuronal 4 [Source:HGNC Symbol;Acc:HGNC:19411]
GEMIN2	gem nuclear organelle associated protein 2 [Source:HGNC Symbol;Acc:HGNC:10884]
KIDINS220	kinase D-interacting substrate 220kDa [Source:HGNC Symbol;Acc:HGNC:29508]
PTEN	phosphatase and tensin homolog [Source:HGNC Symbol;Acc:HGNC:9588]
SLURP1	secreted LY6/PLAUR domain containing 1 [Source:HGNC Symbol;Acc:HGNC:18746]
WDCP	WD repeat and coiled coil containing [Source:HGNC Symbol;Acc:HGNC:26157]
SEMA3E	semaphorin 3E [Source:HGNC Symbol;Acc:HGNC:10727]
BBX	BBX, HMG-box containing [Source:HGNC Symbol;Acc:HGNC:14422]
CALR	calreticulin [Source:HGNC Symbol;Acc:HGNC:1455]
SKI	SKI proto-oncogene [Source:HGNC Symbol;Acc:HGNC:10896]
ZFP36	ZFP36 ring finger protein [Source:HGNC Symbol;Acc:HGNC:12862]
TERF2	telomeric repeat binding factor 2 [Source:HGNC Symbol;Acc:HGNC:11729]
SLC6A8	solute carrier family 6 member 8 [Source:HGNC Symbol;Acc:HGNC:11055]
TSPAN7	tetraspanin 7 [Source:HGNC Symbol;Acc:HGNC:11854]
SCAF11	SR-related CTD associated factor 11 [Source:HGNC Symbol;Acc:HGNC:10784]
SPHK1	sphingosine kinase 1 [Source:HGNC Symbol;Acc:HGNC:11240]
TP53	tumor protein p53 [Source:HGNC Symbol;Acc:HGNC:11998]
TRAF6	TNF receptor associated factor 6 [Source:HGNC Symbol;Acc:HGNC:12036]
C3AR1	complement component 3a receptor 1 [Source:HGNC Symbol;Acc:HGNC:1319]
TRA2B	transformer 2 beta homolog (Drosophila) [Source:HGNC Symbol;Acc:HGNC:10781]
C4A	complement component 4A (Rodgers blood group) [Source:HGNC Symbol;Acc:HGNC:1323]
C4BPA	complement component 4 binding protein alpha [Source:HGNC Symbol;Acc:HGNC:1325]
XPR1	xenotropic and polytropic retrovirus receptor 1 [Source:HGNC Symbol;Acc:HGNC:12827]
C4BPB	complement component 4 binding protein beta [Source:HGNC Symbol;Acc:HGNC:1328]
SET	SET nuclear proto-oncogene [Source:HGNC Symbol;Acc:HGNC:10760]
CAPN5	calpain 5 [Source:HGNC Symbol;Acc:HGNC:1482]
BMP6	bone morphogenetic protein 6 [Source:HGNC Symbol;Acc:HGNC:1073]
HGS	hepatocyte growth factor-regulated tyrosine kinase substrate [Source:HGNC Symbol;Acc:HGNC:4897]
TLR1	toll like receptor 1 [Source:HGNC Symbol;Acc:HGNC:11847]
TEP1	telomerase associated protein 1 [Source:HGNC Symbol;Acc:HGNC:11726]
PPP1R11	protein phosphatase 1 regulatory inhibitor subunit 11 [Source:HGNC Symbol;Acc:HGNC:9285]
TFRC	transferrin receptor [Source:HGNC Symbol;Acc:HGNC:11763]
KLF5	Kruppel like factor 5 [Source:HGNC Symbol;Acc:HGNC:6349]
UBE3D	ubiquitin protein ligase E3D [Source:HGNC Symbol;Acc:HGNC:21381]
STC1	stanniocalcin 1 [Source:HGNC Symbol;Acc:HGNC:11373]

TGFB2	transforming growth factor beta 2 [Source:HGNC Symbol;Acc:HGNC:11768]
STAT3	signal transducer and activator of transcription 3 [Source:HGNC Symbol;Acc:HGNC:11364]
TGFBR2	transforming growth factor beta receptor 2 [Source:HGNC Symbol;Acc:HGNC:11773]
THBD	thrombomodulin [Source:HGNC Symbol;Acc:HGNC:11784]
THBS1	thrombospondin 1 [Source:HGNC Symbol;Acc:HGNC:11785]
SRM	spermidine synthase [Source:HGNC Symbol;Acc:HGNC:11296]
USP6	ubiquitin specific peptidase 6 [Source:HGNC Symbol;Acc:HGNC:12629]
TIMP2	TIMP metallopeptidase inhibitor 2 [Source:HGNC Symbol;Acc:HGNC:11821]
SLPI	secretory leukocyte peptidase inhibitor [Source:HGNC Symbol;Acc:HGNC:11092]
CD5L	CD5 molecule like [Source:HGNC Symbol;Acc:HGNC:1690]
SST	somatostatin [Source:HGNC Symbol;Acc:HGNC:11329]
VEGFC	vascular endothelial growth factor C [Source:HGNC Symbol;Acc:HGNC:12682]
EZR	ezrin [Source:HGNC Symbol;Acc:HGNC:12691]
VIM	vimentin [Source:HGNC Symbol;Acc:HGNC:12692]
RPE65	retinal pigment epithelium-specific protein 65kDa [Source:HGNC Symbol;Acc:HGNC:10294]
SLC9A3R2	SLC9A3 regulator 2 [Source:HGNC Symbol;Acc:HGNC:11076]
ACVRL1	activin A receptor like type 1 [Source:HGNC Symbol;Acc:HGNC:175]
WNT7A	Wnt family member 7A [Source:HGNC Symbol;Acc:HGNC:12786]
ROS1	ROS proto-oncogene 1, receptor tyrosine kinase [Source:HGNC Symbol;Acc:HGNC:10261]
WNT7B	Wnt family member 7B [Source:HGNC Symbol;Acc:HGNC:12787]
ROM1	retinal outer segment membrane protein 1 [Source:HGNC Symbol;Acc:HGNC:10254]
ROBO1	roundabout guidance receptor 1 [Source:HGNC Symbol;Acc:HGNC:10249]
CD28	CD28 molecule [Source:HGNC Symbol;Acc:HGNC:1653]
RHO	rhodopsin [Source:HGNC Symbol;Acc:HGNC:10012]
ACTB	actin beta [Source:HGNC Symbol;Acc:HGNC:132]
MMP20	matrix metallopeptidase 20 [Source:HGNC Symbol;Acc:HGNC:7167]
DLGAP1	DLG associated protein 1 [Source:HGNC Symbol;Acc:HGNC:2905]
SRL	sarcalumenin [Source:HGNC Symbol;Acc:HGNC:11295]
SELP	selectin P [Source:HGNC Symbol;Acc:HGNC:10721]
SELL	selectin L [Source:HGNC Symbol;Acc:HGNC:10720]
C5	complement component 5 [Source:HGNC Symbol;Acc:HGNC:1331]
TTN	titin [Source:HGNC Symbol;Acc:HGNC:12403]
TTPA	alpha tocopherol transfer protein [Source:HGNC Symbol;Acc:HGNC:12404]
CAV1	caveolin 1 [Source:HGNC Symbol;Acc:HGNC:1527]
TNFSF14	tumor necrosis factor superfamily member 14 [Source:HGNC Symbol;Acc:HGNC:11930]
TXN	thioredoxin [Source:HGNC Symbol;Acc:HGNC:12435]
UGT2B17	UDP glucuronosyltransferase family 2 member B17 [Source:HGNC Symbol;Acc:HGNC:12547]
CXCL5	C-X-C motif chemokine ligand 5 [Source:HGNC Symbol;Acc:HGNC:10642]
CXCL6	C-X-C motif chemokine ligand 6 [Source:HGNC Symbol;Acc:HGNC:10643]
CCL22	C-C motif chemokine ligand 22 [Source:HGNC Symbol;Acc:HGNC:10621]
CDHR1	cadherin related family member 1 [Source:HGNC Symbol;Acc:HGNC:14550]

CCL11	C-C motif chemokine ligand 11 [Source:HGNC Symbol;Acc:HGNC:10610]
RIPK1	receptor interacting serine/threonine kinase 1 [Source:HGNC Symbol;Acc:HGNC:10019]
RGR	retinal G protein coupled receptor [Source:HGNC Symbol;Acc:HGNC:9990]
ELAVL2	ELAV like neuron-specific RNA binding protein 2 [Source:HGNC Symbol;Acc:HGNC:3313]
FECH	ferrochelatase [Source:HGNC Symbol;Acc:HGNC:3647]
FGFR2	fibroblast growth factor receptor 2 [Source:HGNC Symbol;Acc:HGNC:3689]
VEGFD	vascular endothelial growth factor D [Source:HGNC Symbol;Acc:HGNC:3708]
NLRP1	NLR family pyrin domain containing 1 [Source:HGNC Symbol;Acc:HGNC:14374]
CD93	CD93 molecule [Source:HGNC Symbol;Acc:HGNC:15855]
DKK1	dickkopf WNT signaling pathway inhibitor 1 [Source:HGNC Symbol;Acc:HGNC:2891]
FLT4	fms related tyrosine kinase 4 [Source:HGNC Symbol;Acc:HGNC:3767]
FMOD	fibromodulin [Source:HGNC Symbol;Acc:HGNC:3774]
FN1	fibronectin 1 [Source:HGNC Symbol;Acc:HGNC:3778]
CRB1	crumbs 1, cell polarity complex component [Source:HGNC Symbol;Acc:HGNC:2343]
ANGPTL2	angiopoietin like 2 [Source:HGNC Symbol;Acc:HGNC:490]
FOSB	FosB proto-oncogene, AP-1 transcription factor subunit [Source:HGNC Symbol;Acc:HGNC:3797]
FPR1	formyl peptide receptor 1 [Source:HGNC Symbol;Acc:HGNC:3826]
FCN2	ficolin 2 [Source:HGNC Symbol;Acc:HGNC:3624]
FCGR3B	Fc fragment of IgG receptor IIIb [Source:HGNC Symbol;Acc:HGNC:3620]
C6orf223	chromosome 6 open reading frame 223 [Source:HGNC Symbol;Acc:HGNC:28692]
EPAS1	endothelial PAS domain protein 1 [Source:HGNC Symbol;Acc:HGNC:3374]
EPO	erythropoietin [Source:HGNC Symbol;Acc:HGNC:3415]
ERG	ERG, ETS transcription factor [Source:HGNC Symbol;Acc:HGNC:3446]
AKT2	AKT serine/threonine kinase 2 [Source:HGNC Symbol;Acc:HGNC:392]
ESR1	estrogen receptor 1 [Source:HGNC Symbol;Acc:HGNC:3467]
ETS1	ETS proto-oncogene 1, transcription factor [Source:HGNC Symbol;Acc:HGNC:3488]
F2	coagulation factor II, thrombin [Source:HGNC Symbol;Acc:HGNC:3535]
F9	coagulation factor IX [Source:HGNC Symbol;Acc:HGNC:3551]
F10	coagulation factor X [Source:HGNC Symbol;Acc:HGNC:3528]
FABP5	fatty acid binding protein 5 [Source:HGNC Symbol;Acc:HGNC:3560]
HYLS1	HYLS1, centriolar and ciliogenesis associated [Source:HGNC Symbol;Acc:HGNC:26558]
FCGR2A	Fc fragment of IgG receptor IIa [Source:HGNC Symbol;Acc:HGNC:3616]
FCGR3A	Fc fragment of IgG receptor IIIa [Source:HGNC Symbol;Acc:HGNC:3619]
OSBP2	oxysterol binding protein 2 [Source:HGNC Symbol;Acc:HGNC:8504]
IL17RA	interleukin 17 receptor A [Source:HGNC Symbol;Acc:HGNC:5985]
GEM	GTP binding protein overexpressed in skeletal muscle [Source:HGNC Symbol;Acc:HGNC:4234]
GHSR	growth hormone secretagogue receptor [Source:HGNC Symbol;Acc:HGNC:4267]
CLUL1	clusterin like 1 [Source:HGNC Symbol;Acc:HGNC:2096]
FAM155B	family with sequence similarity 155 member B [Source:HGNC Symbol;Acc:HGNC:30701]
GCLC	glutamate-cysteine ligase catalytic subunit [Source:HGNC Symbol;Acc:HGNC:4311]
GCLM	glutamate-cysteine ligase modifier subunit [Source:HGNC Symbol;Acc:HGNC:4312]

BHLHE22	basic helix-loop-helix family member e22 [Source:HGNC Symbol;Acc:HGNC:11963]
SGSM3	small G protein signaling modulator 3 [Source:HGNC Symbol;Acc:HGNC:25228]
GNB3	G protein subunit beta 3 [Source:HGNC Symbol;Acc:HGNC:4400]
GNGT2	G protein subunit gamma transducin 2 [Source:HGNC Symbol;Acc:HGNC:4412]
ANGPT1	angiopoietin 1 [Source:HGNC Symbol;Acc:HGNC:484]
ANGPT2	angiopoietin 2 [Source:HGNC Symbol;Acc:HGNC:485]
GRK5	G protein-coupled receptor kinase 5 [Source:HGNC Symbol;Acc:HGNC:4544]
GDF2	growth differentiation factor 2 [Source:HGNC Symbol;Acc:HGNC:4217]
FGF21	fibroblast growth factor 21 [Source:HGNC Symbol;Acc:HGNC:3678]
OPTC	opticin [Source:HGNC Symbol;Acc:HGNC:8158]
FRK	fyn related Src family tyrosine kinase [Source:HGNC Symbol;Acc:HGNC:3955]
PELI3	pellino E3 ubiquitin protein ligase family member 3 [Source:HGNC Symbol;Acc:HGNC:30010]
IL27	interleukin 27 [Source:HGNC Symbol;Acc:HGNC:19157]
MTOR	mechanistic target of rapamycin [Source:HGNC Symbol;Acc:HGNC:3942]
G6PD	glucose-6-phosphate dehydrogenase [Source:HGNC Symbol;Acc:HGNC:4057]
RNF19A	ring finger protein 19A, RBR E3 ubiquitin protein ligase [Source:HGNC Symbol;Acc:HGNC:13432]
MYRIP	myosin VIIA and Rab interacting protein [Source:HGNC Symbol;Acc:HGNC:19156]
ASPM	abnormal spindle microtubule assembly [Source:HGNC Symbol;Acc:HGNC:19048]
CHMP2B	charged multivesicular body protein 2B [Source:HGNC Symbol;Acc:HGNC:24537]
PCDHGA12	protocadherin gamma subfamily A, 12 [Source:HGNC Symbol;Acc:HGNC:8699]
POLDIP2	polymerase (DNA) delta interacting protein 2 [Source:HGNC Symbol;Acc:HGNC:23781]
PRPF31	pre-mRNA processing factor 31 [Source:HGNC Symbol;Acc:HGNC:15446]
GPX3	glutathione peroxidase 3 [Source:HGNC Symbol;Acc:HGNC:4555]
NR2E3	nuclear receptor subfamily 2 group E member 3 [Source:HGNC Symbol;Acc:HGNC:7974]
ARIH2	ariadne RBR E3 ubiquitin protein ligase 2 [Source:HGNC Symbol;Acc:HGNC:690]
MERTK	MER proto-oncogene, tyrosine kinase [Source:HGNC Symbol;Acc:HGNC:7027]
NOP56	NOP56 ribonucleoprotein [Source:HGNC Symbol;Acc:HGNC:15911]
ATG7	autophagy related 7 [Source:HGNC Symbol;Acc:HGNC:16935]
CXCL13	C-X-C motif chemokine ligand 13 [Source:HGNC Symbol;Acc:HGNC:10639]
AHSA1	activator of Hsp90 ATPase activity 1 [Source:HGNC Symbol;Acc:HGNC:1189]
SLCO1B1	solute carrier organic anion transporter family member 1B1 [Source:HGNC Symbol;Acc:HGNC:10959]
TXNIP	thioredoxin interacting protein [Source:HGNC Symbol;Acc:HGNC:16952]
CGRRF1	cell growth regulator with ring finger domain 1 [Source:HGNC Symbol;Acc:HGNC:15528]
HSPH1	heat shock protein family H (Hsp110) member 1 [Source:HGNC Symbol;Acc:HGNC:16969]
CYP46A1	cytochrome P450 family 46 subfamily A member 1 [Source:HGNC Symbol;Acc:HGNC:2641]
GPR75	G protein-coupled receptor 75 [Source:HGNC Symbol;Acc:HGNC:4526]
METAP2	methionyl aminopeptidase 2 [Source:HGNC Symbol;Acc:HGNC:16672]
SEMA3A	semaphorin 3A [Source:HGNC Symbol;Acc:HGNC:10723]
CACNG3	calcium voltage-gated channel auxiliary subunit gamma 3 [Source:HGNC Symbol;Acc:HGNC:1407]

ABCA7	ATP binding cassette subfamily A member 7 [Source:HGNC Symbol;Acc:HGNC:37]
TEC	tec protein tyrosine kinase [Source:HGNC Symbol;Acc:HGNC:11719]
CDH7	cadherin 7 [Source:HGNC Symbol;Acc:HGNC:1766]
ABCB6	ATP binding cassette subfamily B member 6 (Langereis blood group) [Source:HGNC Symbol;Acc:HGNC:47]
NR1H3	nuclear receptor subfamily 1 group H member 3 [Source:HGNC Symbol;Acc:HGNC:7966]
LRPPRC	leucine rich pentatricopeptide repeat containing [Source:HGNC Symbol;Acc:HGNC:15714]
ATP6AP2	ATPase H+ transporting accessory protein 2 [Source:HGNC Symbol;Acc:HGNC:18305]
TSHZ1	teashirt zinc finger homeobox 1 [Source:HGNC Symbol;Acc:HGNC:10669]
CDKN1C	cyclin dependent kinase inhibitor 1C [Source:HGNC Symbol;Acc:HGNC:1786]
CDKN2A	cyclin dependent kinase inhibitor 2A [Source:HGNC Symbol;Acc:HGNC:1787]
CDKN2D	cyclin dependent kinase inhibitor 2D [Source:HGNC Symbol;Acc:HGNC:1790]
KERA	keratocan [Source:HGNC Symbol;Acc:HGNC:6309]
MAP4K1	mitogen-activated protein kinase kinase kinase kinase 1 [Source:HGNC Symbol;Acc:HGNC:6863]
CYP2C19	cytochrome P450 family 2 subfamily C member 19 [Source:HGNC Symbol;Acc:HGNC:2621]
CYP24A1	cytochrome P450 family 24 subfamily A member 1 [Source:HGNC Symbol;Acc:HGNC:2602]
NQO1	NAD(P)H quinone dehydrogenase 1 [Source:HGNC Symbol;Acc:HGNC:2874]
DMBT1	deleted in malignant brain tumors 1 [Source:HGNC Symbol;Acc:HGNC:2926]
DMD	dystrophin [Source:HGNC Symbol;Acc:HGNC:2928]
DMRT1	doublesex and mab-3 related transcription factor 1 [Source:HGNC Symbol;Acc:HGNC:2934]
DNASE1	deoxyribonuclease I [Source:HGNC Symbol;Acc:HGNC:2956]
AGT	angiotensinogen [Source:HGNC Symbol;Acc:HGNC:333]
AGTR1	angiotensin II receptor type 1 [Source:HGNC Symbol;Acc:HGNC:336]
E2F2	E2F transcription factor 2 [Source:HGNC Symbol;Acc:HGNC:3114]
ECM1	extracellular matrix protein 1 [Source:HGNC Symbol;Acc:HGNC:3153]
EGF	epidermal growth factor [Source:HGNC Symbol;Acc:HGNC:3229]
CYP1A2	cytochrome P450 family 1 subfamily A member 2 [Source:HGNC Symbol;Acc:HGNC:2596]
CTNNA1	catenin alpha 1 [Source:HGNC Symbol;Acc:HGNC:2509]
MAPK14	mitogen-activated protein kinase 14 [Source:HGNC Symbol;Acc:HGNC:6876]
FILIP1L	filamin A interacting protein 1 like [Source:HGNC Symbol;Acc:HGNC:24589]
CYP2R1	cytochrome P450 family 2 subfamily R member 1 [Source:HGNC Symbol;Acc:HGNC:20580]
TDRD9	tudor domain containing 9 [Source:HGNC Symbol;Acc:HGNC:20122]
CCR1	C-C motif chemokine receptor 1 [Source:HGNC Symbol;Acc:HGNC:1602]
LTB4R	leukotriene B4 receptor [Source:HGNC Symbol;Acc:HGNC:6713]
CNR1	cannabinoid receptor 1 (brain) [Source:HGNC Symbol;Acc:HGNC:2159]
CNTF	ciliary neurotrophic factor [Source:HGNC Symbol;Acc:HGNC:2169]
RBM45	RNA binding motif protein 45 [Source:HGNC Symbol;Acc:HGNC:24468]
CRK	v-crk avian sarcoma virus CT10 oncogene homolog [Source:HGNC Symbol;Acc:HGNC:2362]
CRYBB1	crystallin beta B1 [Source:HGNC Symbol;Acc:HGNC:2397]
CRYBB2	crystallin beta B2 [Source:HGNC Symbol;Acc:HGNC:2398]
PARP1	poly(ADP-ribose) polymerase 1 [Source:HGNC Symbol;Acc:HGNC:270]

DCP1B	decapping mRNA 1B [Source:HGNC Symbol;Acc:HGNC:24451]
TBK1	TANK binding kinase 1 [Source:HGNC Symbol;Acc:HGNC:11584]
CXCL9	C-X-C motif chemokine ligand 9 [Source:HGNC Symbol;Acc:HGNC:7098]
MMP1	matrix metallopeptidase 1 [Source:HGNC Symbol;Acc:HGNC:7155]
MMP3	matrix metallopeptidase 3 [Source:HGNC Symbol;Acc:HGNC:7173]
MMP7	matrix metallopeptidase 7 [Source:HGNC Symbol;Acc:HGNC:7174]
MMP13	matrix metallopeptidase 13 [Source:HGNC Symbol;Acc:HGNC:7159]
MMP14	matrix metallopeptidase 14 [Source:HGNC Symbol;Acc:HGNC:7160]
MNAT1	MNAT1, CDK activating kinase assembly factor [Source:HGNC Symbol;Acc:HGNC:7181]
CD200	CD200 molecule [Source:HGNC Symbol;Acc:HGNC:7203]
CTRB2	chymotrypsinogen B2 [Source:HGNC Symbol;Acc:HGNC:2522]
MTTP	microsomal triglyceride transfer protein [Source:HGNC Symbol;Acc:HGNC:7467]
MYO7A	myosin VIIA [Source:HGNC Symbol;Acc:HGNC:7606]
MYOG	myogenin [Source:HGNC Symbol;Acc:HGNC:7612]
MGST1	microsomal glutathione S-transferase 1 [Source:HGNC Symbol;Acc:HGNC:7061]
MFGE8	milk fat globule-EGF factor 8 protein [Source:HGNC Symbol;Acc:HGNC:7036]
MDM2	MDM2 proto-oncogene [Source:HGNC Symbol;Acc:HGNC:6973]
MAP2	microtubule associated protein 2 [Source:HGNC Symbol;Acc:HGNC:6839]
NCAM1	neural cell adhesion molecule 1 [Source:HGNC Symbol;Acc:HGNC:7656]
ACO1	aconitase 1 [Source:HGNC Symbol;Acc:HGNC:117]
PEX1	peroxisomal biogenesis factor 1 [Source:HGNC Symbol;Acc:HGNC:8850]
SERPINA1	serpin family A member 1 [Source:HGNC Symbol;Acc:HGNC:8941]
SERPINB8	serpin family B member 8 [Source:HGNC Symbol;Acc:HGNC:8952]
IL17D	interleukin 17D [Source:HGNC Symbol;Acc:HGNC:5984]
PLCG2	phospholipase C gamma 2 [Source:HGNC Symbol;Acc:HGNC:9066]
PLEK	pleckstrin [Source:HGNC Symbol;Acc:HGNC:9070]
PLTP	phospholipid transfer protein [Source:HGNC Symbol;Acc:HGNC:9093]
BCO1	beta-carotene oxygenase 1 [Source:HGNC Symbol;Acc:HGNC:13815]
TREM2	triggering receptor expressed on myeloid cells 2 [Source:HGNC Symbol;Acc:HGNC:17761]
PON2	paraoxonase 2 [Source:HGNC Symbol;Acc:HGNC:9205]
DDIT4	DNA damage inducible transcript 4 [Source:HGNC Symbol;Acc:HGNC:24944]
PPARD	peroxisome proliferator activated receptor delta [Source:HGNC Symbol;Acc:HGNC:9235]
P4HTM	prolyl 4-hydroxylase, transmembrane [Source:HGNC Symbol;Acc:HGNC:28858]
RTEL1	regulator of telomere elongation helicase 1 [Source:HGNC Symbol;Acc:HGNC:15888]
GHRL	ghrelin/obestatin prepropeptide [Source:HGNC Symbol;Acc:HGNC:18129]
IL23A	interleukin 23 subunit alpha [Source:HGNC Symbol;Acc:HGNC:15488]
NGF	nerve growth factor [Source:HGNC Symbol;Acc:HGNC:7808]
NOS2	nitric oxide synthase 2 [Source:HGNC Symbol;Acc:HGNC:7873]
NPHP1	nephrocystin 1 [Source:HGNC Symbol;Acc:HGNC:7905]
SLC11A2	solute carrier family 11 member 2 [Source:HGNC Symbol;Acc:HGNC:10908]
P2RX4	purinergic receptor P2X 4 [Source:HGNC Symbol;Acc:HGNC:8535]

IL22	interleukin 22 [Source:HGNC Symbol;Acc:HGNC:14900]
RDH8	retinol dehydrogenase 8 (all-trans) [Source:HGNC Symbol;Acc:HGNC:14423]
ADIPOR1	adiponectin receptor 1 [Source:HGNC Symbol;Acc:HGNC:24040]
ANGPTL4	angiopoietin like 4 [Source:HGNC Symbol;Acc:HGNC:16039]
TLR7	toll like receptor 7 [Source:HGNC Symbol;Acc:HGNC:15631]
ZCWPW1	zinc finger CW-type and PWWP domain containing 1 [Source:HGNC Symbol;Acc:HGNC:23486]
PRSS50	protease, serine 50 [Source:HGNC Symbol;Acc:HGNC:17910]
XIAP	X-linked inhibitor of apoptosis [Source:HGNC Symbol;Acc:HGNC:592]
HSPG2	heparan sulfate proteoglycan 2 [Source:HGNC Symbol;Acc:HGNC:5273]
APOA1	apolipoprotein A1 [Source:HGNC Symbol;Acc:HGNC:600]
TNC	tenascin C [Source:HGNC Symbol;Acc:HGNC:5318]
ICAM1	intercellular adhesion molecule 1 [Source:HGNC Symbol;Acc:HGNC:5344]
IFNG	interferon, gamma [Source:HGNC Symbol;Acc:HGNC:5438]
EYS	eyes shut homolog (Drosophila) [Source:HGNC Symbol;Acc:HGNC:21555]
IGF1R	insulin like growth factor 1 receptor [Source:HGNC Symbol;Acc:HGNC:5465]
APP	amyloid beta precursor protein [Source:HGNC Symbol;Acc:HGNC:620]
IL1A	interleukin 1 alpha [Source:HGNC Symbol;Acc:HGNC:5991]
IL2	interleukin 2 [Source:HGNC Symbol;Acc:HGNC:6001]
IL2RA	interleukin 2 receptor subunit alpha [Source:HGNC Symbol;Acc:HGNC:6008]
IL2RG	interleukin 2 receptor subunit gamma [Source:HGNC Symbol;Acc:HGNC:6010]
HSPA4	heat shock protein family A (Hsp70) member 4 [Source:HGNC Symbol;Acc:HGNC:5237]
HSD11B1	hydroxysteroid 11-beta dehydrogenase 1 [Source:HGNC Symbol;Acc:HGNC:5208]
HMOX1	heme oxygenase 1 [Source:HGNC Symbol;Acc:HGNC:5013]
CXCL1	C-X-C motif chemokine ligand 1 [Source:HGNC Symbol;Acc:HGNC:4602]
GSR	glutathione reductase [Source:HGNC Symbol;Acc:HGNC:4623]
GSTM2	glutathione S-transferase mu 2 (muscle) [Source:HGNC Symbol;Acc:HGNC:4634]
GSTZ1	glutathione S-transferase zeta 1 [Source:HGNC Symbol;Acc:HGNC:4643]
GTF2H4	general transcription factor IIH subunit 4 [Source:HGNC Symbol;Acc:HGNC:4658]
GUCA1A	guanylate cyclase activator 1A [Source:HGNC Symbol;Acc:HGNC:4678]
ICOS	inducible T-cell costimulator [Source:HGNC Symbol;Acc:HGNC:5351]
VSX1	visual system homeobox 1 [Source:HGNC Symbol;Acc:HGNC:12723]
HLA-A	major histocompatibility complex, class I, A [Source:HGNC Symbol;Acc:HGNC:4931]
HLA-DPB1	major histocompatibility complex, class II, DP beta 1 [Source:HGNC Symbol;Acc:HGNC:4940]
HLA-DQA1	major histocompatibility complex, class II, DQ alpha 1 [Source:HGNC Symbol;Acc:HGNC:4942]
HLA-DRB1	major histocompatibility complex, class II, DR beta 1 [Source:HGNC Symbol;Acc:HGNC:4948]
HMGB1	high mobility group box 1 [Source:HGNC Symbol;Acc:HGNC:4983]
IL6ST	interleukin 6 signal transducer [Source:HGNC Symbol;Acc:HGNC:6021]
AQP1	aquaporin 1 (Colton blood group) [Source:HGNC Symbol;Acc:HGNC:633]
LCN2	lipocalin 2 [Source:HGNC Symbol;Acc:HGNC:6526]
LGALS1	galectin 1 [Source:HGNC Symbol;Acc:HGNC:6561]
FADS1	fatty acid desaturase 1 [Source:HGNC Symbol;Acc:HGNC:3574]

LOXL1	lysyl oxidase like 1 [Source:HGNC Symbol;Acc:HGNC:6665]
LPA	lipoprotein(a) [Source:HGNC Symbol;Acc:HGNC:6667]
LRP5	LDL receptor related protein 5 [Source:HGNC Symbol;Acc:HGNC:6697]
ARNT	aryl hydrocarbon receptor nuclear translocator [Source:HGNC Symbol;Acc:HGNC:700]
LCN9	lipocalin 9 [Source:HGNC Symbol;Acc:HGNC:17442]
LAMP2	lysosomal associated membrane protein 2 [Source:HGNC Symbol;Acc:HGNC:6501]
LAMA3	laminin subunit alpha 3 [Source:HGNC Symbol;Acc:HGNC:6483]
IL13	interleukin 13 [Source:HGNC Symbol;Acc:HGNC:5973]
IL15	interleukin 15 [Source:HGNC Symbol;Acc:HGNC:5977]
IMPDH1	IMP (inosine 5'-monophosphate) dehydrogenase 1 [Source:HGNC Symbol;Acc:HGNC:6052]
ISG20	interferon stimulated exonuclease gene 20 [Source:HGNC Symbol;Acc:HGNC:6130]
ITGAM	integrin subunit alpha M [Source:HGNC Symbol;Acc:HGNC:6149]
MIA3	melanoma inhibitory activity family member 3 [Source:HGNC Symbol;Acc:HGNC:24008]
CA13	carbonic anhydrase 13 [Source:HGNC Symbol;Acc:HGNC:14914]
KIR2DS1	killer cell immunoglobulin like receptor, two Ig domains and short cytoplasmic tail 1 [Source:HGNC Symbol;Acc:HGNC:6333]
KIR3DL1	killer cell immunoglobulin like receptor, three Ig domains and long cytoplasmic tail 1 [Source:HGNC Symbol;Acc:HGNC:6338]
KIR3DL2	killer cell immunoglobulin like receptor, three Ig domains and long cytoplasmic tail 2 [Source:HGNC Symbol;Acc:HGNC:6339]
KRT8	keratin 8 [Source:HGNC Symbol;Acc:HGNC:6446]
ACAT2	acetyl-CoA acetyltransferase 2 [Source:HGNC Symbol;Acc:HGNC:94]
ADAMTS9	ADAM metallopeptidase with thrombospondin type 1 motif 9 [Source:HGNC Symbol;Acc:HGNC:13202]
A2M	alpha-2-macroglobulin [Source:HGNC Symbol;Acc:HGNC:7]
ABCA10	ATP binding cassette subfamily A member 10 [Source:HGNC Symbol;Acc:HGNC:30]
ABCA12	ATP binding cassette subfamily A member 12 [Source:HGNC Symbol;Acc:HGNC:14637]
ABCA13	ATP binding cassette subfamily A member 13 [Source:HGNC Symbol;Acc:HGNC:14638]
ABCA2	ATP binding cassette subfamily A member 2 [Source:HGNC Symbol;Acc:HGNC:32]
ABCA3	ATP binding cassette subfamily A member 3 [Source:HGNC Symbol;Acc:HGNC:33]
ABCA5	ATP binding cassette subfamily A member 5 [Source:HGNC Symbol;Acc:HGNC:35]
ABCA6	ATP binding cassette subfamily A member 6 [Source:HGNC Symbol;Acc:HGNC:36]
ABCB1	ATP binding cassette subfamily B member 1 [Source:HGNC Symbol;Acc:HGNC:40]
ABCC5	ATP binding cassette subfamily C member 5 [Source:HGNC Symbol;Acc:HGNC:56]
ABCG5	ATP binding cassette subfamily G member 5 [Source:HGNC Symbol;Acc:HGNC:13886]
ABCG8	ATP binding cassette subfamily G member 8 [Source:HGNC Symbol;Acc:HGNC:13887]
ACAN	aggrecan [Source:HGNC Symbol;Acc:HGNC:319]
ACKR1	atypical chemokine receptor 1 (Duffy blood group) [Source:HGNC Symbol;Acc:HGNC:4035]
ACTG1	actin gamma 1 [Source:HGNC Symbol;Acc:HGNC:144]
ADI1	acireductone dioxygenase 1 [Source:HGNC Symbol;Acc:HGNC:30576]
ADORA1	adenosine A1 receptor [Source:HGNC Symbol;Acc:HGNC:262]
ADORA2A	adenosine A2a receptor [Source:HGNC Symbol;Acc:HGNC:263]
ADORA2B	adenosine A2b receptor [Source:HGNC Symbol;Acc:HGNC:264]

ADORA3	adenosine A3 receptor [Source:HGNC Symbol;Acc:HGNC:268]
ADRB2	adrenoceptor beta 2 [Source:HGNC Symbol;Acc:HGNC:286]
AGBL1	ATP/GTP binding protein-like 1 [Source:HGNC Symbol;Acc:HGNC:26504]
AGBL3	ATP/GTP binding protein-like 3 [Source:HGNC Symbol;Acc:HGNC:27981]
AGO2	argonaute 2, RISC catalytic component [Source:HGNC Symbol;Acc:HGNC:3263]
AIM2	absent in melanoma 2 [Source:HGNC Symbol;Acc:HGNC:357]
AIPL1	aryl hydrocarbon receptor interacting protein like 1 [Source:HGNC Symbol;Acc:HGNC:359]
AKR1B1	aldo-keto reductase family 1 member B [Source:HGNC Symbol;Acc:HGNC:381]
AKT1	AKT serine/threonine kinase 1 [Source:HGNC Symbol;Acc:HGNC:391]
ALDH2	aldehyde dehydrogenase 2 family (mitochondrial) [Source:HGNC Symbol;Acc:HGNC:404]
ALOX15	arachidonate 15-lipoxygenase [Source:HGNC Symbol;Acc:HGNC:433]
ANXA2	annexin A2 [Source:HGNC Symbol;Acc:HGNC:537]
ANXA5	annexin A5 [Source:HGNC Symbol;Acc:HGNC:543]
ANXA8	annexin A8 [Source:HGNC Symbol;Acc:HGNC:546]
AP5M1	adaptor related protein complex 5 mu 1 subunit [Source:HGNC Symbol;Acc:HGNC:20192]
APCS	amyloid P component, serum [Source:HGNC Symbol;Acc:HGNC:584]
APOA2	apolipoprotein A2 [Source:HGNC Symbol;Acc:HGNC:601]
APOC1	apolipoprotein C1 [Source:HGNC Symbol;Acc:HGNC:607]
APOC2	apolipoprotein C2 [Source:HGNC Symbol;Acc:HGNC:609]
APOC3	apolipoprotein C3 [Source:HGNC Symbol;Acc:HGNC:610]
APOC4	apolipoprotein C4 [Source:HGNC Symbol;Acc:HGNC:611]
AQP4	aquaporin 4 [Source:HGNC Symbol;Acc:HGNC:637]
ARID3A	AT-rich interaction domain 3A [Source:HGNC Symbol;Acc:HGNC:3031]
ARR3	arrestin 3 retinal (X-arrestin) [Source:HGNC Symbol;Acc:HGNC:710]
ASCL1	achaete-scute family bHLH transcription factor 1 [Source:HGNC Symbol;Acc:HGNC:738]
ATF4	activating transcription factor 4 [Source:HGNC Symbol;Acc:HGNC:786]
ATF6	activating transcription factor 6 [Source:HGNC Symbol;Acc:HGNC:791]
ATG12	autophagy related 12 [Source:HGNC Symbol;Acc:HGNC:588]
ATG5	autophagy related 5 [Source:HGNC Symbol;Acc:HGNC:589]
ATOH7	atonal bHLH transcription factor 7 [Source:HGNC Symbol;Acc:HGNC:13907]
ATP12A	ATPase H+/K+ transporting non-gastric alpha2 subunit [Source:HGNC Symbol;Acc:HGNC:13816]
ATP4A	ATPase H+/K+ transporting alpha subunit [Source:HGNC Symbol;Acc:HGNC:819]
ATRAID	all-trans retinoic acid induced differentiation factor [Source:HGNC Symbol;Acc:HGNC:24090]
ATXN2	ataxin 2 [Source:HGNC Symbol;Acc:HGNC:10555]
AXL	AXL receptor tyrosine kinase [Source:HGNC Symbol;Acc:HGNC:905]
B2M	beta-2-microglobulin [Source:HGNC Symbol;Acc:HGNC:914]
BACE1	beta-secretase 1 [Source:HGNC Symbol;Acc:HGNC:933]
BAHCC1	BAH domain and coiled-coil containing 1 [Source:HGNC Symbol;Acc:HGNC:29279]
BAIAP2L2	BAI1 associated protein 2 like 2 [Source:HGNC Symbol;Acc:HGNC:26203]
BBS1	Bardet-Biedl syndrome 1 [Source:HGNC Symbol;Acc:HGNC:966]
BCAM	basal cell adhesion molecule (Lutheran blood group) [Source:HGNC Symbol;Acc:HGNC:6722]

BCL2L1	BCL2 like 1 [Source:HGNC Symbol;Acc:HGNC:992]
BCO2	beta-carotene oxygenase 2 [Source:HGNC Symbol;Acc:HGNC:18503]
BECN1	beclin 1 [Source:HGNC Symbol;Acc:HGNC:1034]
BFSP1	beaded filament structural protein 1 [Source:HGNC Symbol;Acc:HGNC:1040]
BGN	biglycan [Source:HGNC Symbol;Acc:HGNC:1044]
BMP1	bone morphogenetic protein 1 [Source:HGNC Symbol;Acc:HGNC:1067]
BMP7	bone morphogenetic protein 7 [Source:HGNC Symbol;Acc:HGNC:1074]
BMP8B	bone morphogenetic protein 8b [Source:HGNC Symbol;Acc:HGNC:1075]
BRAP	BRCA1 associated protein [Source:HGNC Symbol;Acc:HGNC:1099]
BRCA1	BRCA1, DNA repair associated [Source:HGNC Symbol;Acc:HGNC:1100]
BSG	basigin (Ok blood group) [Source:HGNC Symbol;Acc:HGNC:1116]
BSN	bassoon presynaptic cytomatrix protein [Source:HGNC Symbol;Acc:HGNC:1117]
C18orf8	chromosome 18 open reading frame 8 [Source:HGNC Symbol;Acc:HGNC:24326]
C1QA	complement component 1, q subcomponent, A chain [Source:HGNC Symbol;Acc:HGNC:1241]
C1QB	complement component 1, q subcomponent, B chain [Source:HGNC Symbol;Acc:HGNC:1242]
C1QC	complement component 1, q subcomponent, C chain [Source:HGNC Symbol;Acc:HGNC:1245]
C1R	complement C1r subcomponent [Source:HGNC Symbol;Acc:HGNC:1246]
C1RL	complement C1r subcomponent like [Source:HGNC Symbol;Acc:HGNC:21265]
C1S	complement component 1, s subcomponent [Source:HGNC Symbol;Acc:HGNC:1247]
C20orf85	chromosome 20 open reading frame 85 [Source:HGNC Symbol;Acc:HGNC:16216]
C2orf71	chromosome 2 open reading frame 71 [Source:HGNC Symbol;Acc:HGNC:34383]
C4B	complement component 4B (Chido blood group) [Source:HGNC Symbol;Acc:HGNC:1324]
C5AR1	complement component 5a receptor 1 [Source:HGNC Symbol;Acc:HGNC:1338]
C5AR2	complement component 5a receptor 2 [Source:HGNC Symbol;Acc:HGNC:4527]
C7orf61	chromosome 7 open reading frame 61 [Source:HGNC Symbol;Acc:HGNC:22135]
C8B	complement component 8, beta polypeptide [Source:HGNC Symbol;Acc:HGNC:1353]
CA4	carbonic anhydrase 4 [Source:HGNC Symbol;Acc:HGNC:1375]
CACNA1F	calcium voltage-gated channel subunit alpha1 F [Source:HGNC Symbol;Acc:HGNC:1393]
CACNA2D4	calcium voltage-gated channel auxiliary subunit alpha2delta 4 [Source:HGNC Symbol;Acc:HGNC:20202]
CALB1	calbindin 1 [Source:HGNC Symbol;Acc:HGNC:1434]
CALB2	calbindin 2 [Source:HGNC Symbol;Acc:HGNC:1435]
CAPN2	calpain 2 [Source:HGNC Symbol;Acc:HGNC:1479]
CASP2	caspase 2 [Source:HGNC Symbol;Acc:HGNC:1503]
CASP3	caspase 3 [Source:HGNC Symbol;Acc:HGNC:1504]
CASP4	caspase 4 [Source:HGNC Symbol;Acc:HGNC:1505]
CASP6	caspase 6 [Source:HGNC Symbol;Acc:HGNC:1507]
CASP9	caspase 9 [Source:HGNC Symbol;Acc:HGNC:1511]
CATSPERD	cation channel sperm associated auxiliary subunit delta [Source:HGNC Symbol;Acc:HGNC:28598]
CAV2	caveolin 2 [Source:HGNC Symbol;Acc:HGNC:1528]
CBSL	cystathionine-beta-synthase like [Source:HGNC Symbol;Acc:HGNC:51829]

CCDC109B	coiled-coil domain containing 109B [Source:HGNC Symbol;Acc:HGNC:26076]
CCDC172	coiled-coil domain containing 172 [Source:HGNC Symbol;Acc:HGNC:30524]
CCDC96	coiled-coil domain containing 96 [Source:HGNC Symbol;Acc:HGNC:26900]
CCL24	C-C motif chemokine ligand 24 [Source:HGNC Symbol;Acc:HGNC:10623]
CCL26	C-C motif chemokine ligand 26 [Source:HGNC Symbol;Acc:HGNC:10625]
CCL3	C-C motif chemokine ligand 3 [Source:HGNC Symbol;Acc:HGNC:10627]
CCL4	C-C motif chemokine ligand 4 [Source:HGNC Symbol;Acc:HGNC:10630]
CCL5	C-C motif chemokine ligand 5 [Source:HGNC Symbol;Acc:HGNC:10632]
CCL7	C-C motif chemokine ligand 7 [Source:HGNC Symbol;Acc:HGNC:10634]
CCND1	cyclin D1 [Source:HGNC Symbol;Acc:HGNC:1582]
CCR5	C-C motif chemokine receptor 5 (gene/pseudogene) [Source:HGNC Symbol;Acc:HGNC:1606]
CD163	CD163 molecule [Source:HGNC Symbol;Acc:HGNC:1631]
CD19	CD19 molecule [Source:HGNC Symbol;Acc:HGNC:1633]
CD200R1	CD200 receptor 1 [Source:HGNC Symbol;Acc:HGNC:24235]
CD4	CD4 molecule [Source:HGNC Symbol;Acc:HGNC:1678]
CD44	CD44 molecule (Indian blood group) [Source:HGNC Symbol;Acc:HGNC:1681]
CD47	CD47 molecule [Source:HGNC Symbol;Acc:HGNC:1682]
CD63	CD63 molecule [Source:HGNC Symbol;Acc:HGNC:1692]
CD68	CD68 molecule [Source:HGNC Symbol;Acc:HGNC:1693]
CD81	CD81 molecule [Source:HGNC Symbol;Acc:HGNC:1701]
CD8A	CD8a molecule [Source:HGNC Symbol;Acc:HGNC:1706]
CDC42	cell division cycle 42 [Source:HGNC Symbol;Acc:HGNC:1736]
CDH1	cadherin 1 [Source:HGNC Symbol;Acc:HGNC:1748]
CDH17	cadherin 17 [Source:HGNC Symbol;Acc:HGNC:1756]
CDH2	cadherin 2 [Source:HGNC Symbol;Acc:HGNC:1759]
CDH23	cadherin related 23 [Source:HGNC Symbol;Acc:HGNC:13733]
CDH5	cadherin 5 [Source:HGNC Symbol;Acc:HGNC:1764]
CDKN1A	cyclin dependent kinase inhibitor 1A [Source:HGNC Symbol;Acc:HGNC:1784]
CEP290	centrosomal protein 290 [Source:HGNC Symbol;Acc:HGNC:29021]
CERKL	ceramide kinase like [Source:HGNC Symbol;Acc:HGNC:21699]
CFDP1	craniofacial development protein 1 [Source:HGNC Symbol;Acc:HGNC:1873]
CFTR	cystic fibrosis transmembrane conductance regulator [Source:HGNC Symbol;Acc:HGNC:1884]
CHM	CHM, Rab escort protein 1 [Source:HGNC Symbol;Acc:HGNC:1940]
CHMP7	charged multivesicular body protein 7 [Source:HGNC Symbol;Acc:HGNC:28439]
CLDN19	claudin 19 [Source:HGNC Symbol;Acc:HGNC:2040]
CLDN5	claudin 5 [Source:HGNC Symbol;Acc:HGNC:2047]
CLPTM1	CLPTM1, transmembrane protein [Source:HGNC Symbol;Acc:HGNC:2087]
CLRN1	clarin 1 [Source:HGNC Symbol;Acc:HGNC:12605]
CMSS1	cms1 ribosomal small subunit homolog (yeast) [Source:HGNC Symbol;Acc:HGNC:28666]
CNGA1	cyclic nucleotide gated channel alpha 1 [Source:HGNC Symbol;Acc:HGNC:2148]
CNGA3	cyclic nucleotide gated channel alpha 3 [Source:HGNC Symbol;Acc:HGNC:2150]

CNGB1	cyclic nucleotide gated channel beta 1 [Source:HGNC Symbol;Acc:HGNC:2151]
COL11A1	collagen type XI alpha 1 chain [Source:HGNC Symbol;Acc:HGNC:2186]
COL15A1	collagen type XV alpha 1 chain [Source:HGNC Symbol;Acc:HGNC:2192]
COL1A1	collagen type I alpha 1 [Source:HGNC Symbol;Acc:HGNC:2197]
COL4A1	collagen type IV alpha 1 chain [Source:HGNC Symbol;Acc:HGNC:2202]
COL4A2	collagen type IV alpha 2 [Source:HGNC Symbol;Acc:HGNC:2203]
COL4A4	collagen type IV alpha 4 chain [Source:HGNC Symbol;Acc:HGNC:2206]
COL6A2	collagen type VI alpha 2 [Source:HGNC Symbol;Acc:HGNC:2212]
COL8A2	collagen type VIII alpha 2 [Source:HGNC Symbol;Acc:HGNC:2216]
COLEC11	collectin subfamily member 11 [Source:HGNC Symbol;Acc:HGNC:17213]
CR1	complement component 3b/4b receptor 1 (Knops blood group) [Source:HGNC Symbol;Acc:HGNC:2334]
CREB1	cAMP responsive element binding protein 1 [Source:HGNC Symbol;Acc:HGNC:2345]
CRYAA	crystallin alpha A [Source:HGNC Symbol;Acc:HGNC:2388]
CRYBA1	crystallin beta A1 [Source:HGNC Symbol;Acc:HGNC:2394]
CRYGB	crystallin gamma B [Source:HGNC Symbol;Acc:HGNC:2409]
CRYGC	crystallin gamma C [Source:HGNC Symbol;Acc:HGNC:2410]
CRYGN	crystallin gamma N [Source:HGNC Symbol;Acc:HGNC:20458]
CRYGS	crystallin gamma S [Source:HGNC Symbol;Acc:HGNC:2417]
CSF1R	colony stimulating factor 1 receptor [Source:HGNC Symbol;Acc:HGNC:2433]
CSF2	colony stimulating factor 2 [Source:HGNC Symbol;Acc:HGNC:2434]
CSF3	colony stimulating factor 3 [Source:HGNC Symbol;Acc:HGNC:2438]
CTBP2	C-terminal binding protein 2 [Source:HGNC Symbol;Acc:HGNC:2495]
CTGF	connective tissue growth factor [Source:HGNC Symbol;Acc:HGNC:2500]
CTLA4	cytotoxic T-lymphocyte associated protein 4 [Source:HGNC Symbol;Acc:HGNC:2505]
CTRL	chymotrypsin like [Source:HGNC Symbol;Acc:HGNC:2524]
CTSB	cathepsin B [Source:HGNC Symbol;Acc:HGNC:2527]
CTSD	cathepsin D [Source:HGNC Symbol;Acc:HGNC:2529]
CTSL	cathepsin L [Source:HGNC Symbol;Acc:HGNC:2537]
CUL3	cullin 3 [Source:HGNC Symbol;Acc:HGNC:2553]
CYBA	cytochrome b-245 alpha chain [Source:HGNC Symbol;Acc:HGNC:2577]
CYBB	cytochrome b-245 beta chain [Source:HGNC Symbol;Acc:HGNC:2578]
CYCS	cytochrome c, somatic [Source:HGNC Symbol;Acc:HGNC:19986]
CYP1B1	cytochrome P450 family 1 subfamily B member 1 [Source:HGNC Symbol;Acc:HGNC:2597]
CYP2C9	cytochrome P450 family 2 subfamily C member 9 [Source:HGNC Symbol;Acc:HGNC:2623]
CYP4V2	cytochrome P450 family 4 subfamily V member 2 [Source:HGNC Symbol;Acc:HGNC:23198]
DAB2	DAB2, clathrin adaptor protein [Source:HGNC Symbol;Acc:HGNC:2662]
DCN	decorin [Source:HGNC Symbol;Acc:HGNC:2705]
DCT	dopachrome tautomerase [Source:HGNC Symbol;Acc:HGNC:2709]
DDIT3	DNA damage inducible transcript 3 [Source:HGNC Symbol;Acc:HGNC:2726]
DDR1	discoidin domain receptor tyrosine kinase 1 [Source:HGNC Symbol;Acc:HGNC:2730]
DDX58	DEXD/H-box helicase 58 [Source:HGNC Symbol;Acc:HGNC:19102]

DEGS1	delta(4)-desaturase, sphingolipid 1 [Source:HGNC Symbol;Acc:HGNC:13709]
WHRN	whirlin [Source:HGNC Symbol;Acc:HGNC:16361]
DGCR8	DGCR8 microprocessor complex subunit [Source:HGNC Symbol;Acc:HGNC:2847]
DLG4	discs large MAGUK scaffold protein 4 [Source:HGNC Symbol;Acc:HGNC:2903]
DLL4	delta like canonical Notch ligand 4 [Source:HGNC Symbol;Acc:HGNC:2910]
DNMT1	DNA (cytosine-5-)-methyltransferase 1 [Source:HGNC Symbol;Acc:HGNC:2976]
DNTT	DNA nucleotidylexotransferase [Source:HGNC Symbol;Acc:HGNC:2983]
DROSHA	drosha ribonuclease III [Source:HGNC Symbol;Acc:HGNC:17904]
DUOX1	dual oxidase 1 [Source:HGNC Symbol;Acc:HGNC:3062]
DUS3L	dihydrouridine synthase 3 like [Source:HGNC Symbol;Acc:HGNC:26920]
EDN1	endothelin 1 [Source:HGNC Symbol;Acc:HGNC:3176]
EDN2	endothelin 2 [Source:HGNC Symbol;Acc:HGNC:3177]
EFNB2	ephrin B2 [Source:HGNC Symbol;Acc:HGNC:3227]
EGFR	epidermal growth factor receptor [Source:HGNC Symbol;Acc:HGNC:3236]
EGR1	early growth response 1 [Source:HGNC Symbol;Acc:HGNC:3238]
EIF2AK3	eukaryotic translation initiation factor 2 alpha kinase 3 [Source:HGNC Symbol;Acc:HGNC:3255]
EIF2S1	eukaryotic translation initiation factor 2 subunit alpha [Source:HGNC Symbol;Acc:HGNC:3265]
ELANE	elastase, neutrophil expressed [Source:HGNC Symbol;Acc:HGNC:3309]
ELOVL1	ELOVL fatty acid elongase 1 [Source:HGNC Symbol;Acc:HGNC:14418]
ELOVL2	ELOVL fatty acid elongase 2 [Source:HGNC Symbol;Acc:HGNC:14416]
ELOVL5	ELOVL fatty acid elongase 5 [Source:HGNC Symbol;Acc:HGNC:21308]
EMC1	ER membrane protein complex subunit 1 [Source:HGNC Symbol;Acc:HGNC:28957]
ENG	endoglin [Source:HGNC Symbol;Acc:HGNC:3349]
ENO1	enolase 1 [Source:HGNC Symbol;Acc:HGNC:3350]
CBS	cystathionine-beta-synthase [Source:HGNC Symbol;Acc:HGNC:1550]
XXbac-BPG246D15.9	
XXbac-BPG116M5.17	
RP11-330H6.5	
RP11-566K11.2	
OPN1MW3	opsin 1 (cone pigments), medium-wave-sensitive 3 [Source:HGNC Symbol;Acc:HGNC:51831]
OPN1MW	opsin 1 (cone pigments), medium-wave-sensitive [Source:HGNC Symbol;Acc:HGNC:4206]
TBC1D3I	TBC1 domain family member 3I [Source:HGNC Symbol;Acc:HGNC:32709]
EPHA2	EPH receptor A2 [Source:HGNC Symbol;Acc:HGNC:3386]
ERBB2	erb-b2 receptor tyrosine kinase 2 [Source:HGNC Symbol;Acc:HGNC:3430]
ERMN	ermin [Source:HGNC Symbol;Acc:HGNC:29208]
ERN1	endoplasmic reticulum to nucleus signaling 1 [Source:HGNC Symbol;Acc:HGNC:3449]
ERP29	endoplasmic reticulum protein 29 [Source:HGNC Symbol;Acc:HGNC:13799]
F13B	coagulation factor XIII B chain [Source:HGNC Symbol;Acc:HGNC:3534]
FAM161A	family with sequence similarity 161 member A [Source:HGNC Symbol;Acc:HGNC:25808]
FASLG	Fas ligand [Source:HGNC Symbol;Acc:HGNC:11936]
FBLN1	fibulin 1 [Source:HGNC Symbol;Acc:HGNC:3600]

FBLN2	fibulin 2 [Source:HGNC Symbol;Acc:HGNC:3601]
FBLN7	fibulin 7 [Source:HGNC Symbol;Acc:HGNC:26740]
FBN1	fibrillin 1 [Source:HGNC Symbol;Acc:HGNC:3603]
FCGR1A	Fc fragment of IgG receptor Ia [Source:HGNC Symbol;Acc:HGNC:3613]
FCGR2B	Fc fragment of IgG receptor IIb [Source:HGNC Symbol;Acc:HGNC:3618]
FCGRT	Fc fragment of IgG receptor and transporter [Source:HGNC Symbol;Acc:HGNC:3621]
FCN1	ficolin 1 [Source:HGNC Symbol;Acc:HGNC:3623]
FCN3	ficolin 3 [Source:HGNC Symbol;Acc:HGNC:3625]
FGF1	fibroblast growth factor 1 [Source:HGNC Symbol;Acc:HGNC:3665]
FGF13	fibroblast growth factor 13 [Source:HGNC Symbol;Acc:HGNC:3670]
FGF5	fibroblast growth factor 5 [Source:HGNC Symbol;Acc:HGNC:3683]
FOLH1	folate hydrolase (prostate-specific membrane antigen) 1 [Source:HGNC Symbol;Acc:HGNC:3788]
FOS	Fos proto-oncogene, AP-1 transcription factor subunit [Source:HGNC Symbol;Acc:HGNC:3796]
FOXC1	forkhead box C1 [Source:HGNC Symbol;Acc:HGNC:3800]
FOXO1	forkhead box O1 [Source:HGNC Symbol;Acc:HGNC:3819]
FOXO3	forkhead box O3 [Source:HGNC Symbol;Acc:HGNC:3821]
FTO	fat mass and obesity associated [Source:HGNC Symbol;Acc:HGNC:24678]
FURIN	furin, paired basic amino acid cleaving enzyme [Source:HGNC Symbol;Acc:HGNC:8568]
FUT3	fucosyltransferase 3 (Lewis blood group) [Source:HGNC Symbol;Acc:HGNC:4014]
FUT5	fucosyltransferase 5 [Source:HGNC Symbol;Acc:HGNC:4016]
FYB	FYN binding protein [Source:HGNC Symbol;Acc:HGNC:4036]
GADD45G	growth arrest and DNA damage inducible gamma [Source:HGNC Symbol;Acc:HGNC:4097]
GAPDH	glyceraldehyde-3-phosphate dehydrogenase [Source:HGNC Symbol;Acc:HGNC:4141]
GDNF	glial cell derived neurotrophic factor [Source:HGNC Symbol;Acc:HGNC:4232]
GFAP	glial fibrillary acidic protein [Source:HGNC Symbol;Acc:HGNC:4235]
GJA1	gap junction protein alpha 1 [Source:HGNC Symbol;Acc:HGNC:4274]
GJD2	gap junction protein delta 2 [Source:HGNC Symbol;Acc:HGNC:19154]
GLIS1	GLIS family zinc finger 1 [Source:HGNC Symbol;Acc:HGNC:29525]
GLUL	glutamate-ammonia ligase [Source:HGNC Symbol;Acc:HGNC:4341]
GNAT1	G protein subunit alpha transducin 1 [Source:HGNC Symbol;Acc:HGNC:4393]
GNAT2	G protein subunit alpha transducin 2 [Source:HGNC Symbol;Acc:HGNC:4394]
GPR143	G protein-coupled receptor 143 [Source:HGNC Symbol;Acc:HGNC:20145]
GPR179	G protein-coupled receptor 179 [Source:HGNC Symbol;Acc:HGNC:31371]
GPX1	glutathione peroxidase 1 [Source:HGNC Symbol;Acc:HGNC:4553]
GPX4	glutathione peroxidase 4 [Source:HGNC Symbol;Acc:HGNC:4556]
GRIN3B	glutamate ionotropic receptor NMDA type subunit 3B [Source:HGNC Symbol;Acc:HGNC:16768]
GRK1	G protein-coupled receptor kinase 1 [Source:HGNC Symbol;Acc:HGNC:10013]
GRK7	G protein-coupled receptor kinase 7 [Source:HGNC Symbol;Acc:HGNC:17031]
GRM6	glutamate metabotropic receptor 6 [Source:HGNC Symbol;Acc:HGNC:4598]
GSDMD	gasdermin D [Source:HGNC Symbol;Acc:HGNC:25697]
GSK3B	glycogen synthase kinase 3 beta [Source:HGNC Symbol;Acc:HGNC:4617]

GUCA1B	guanylate cyclase activator 1B [Source:HGNC Symbol;Acc:HGNC:4679]
GUCA1C	guanylate cyclase activator 1C [Source:HGNC Symbol;Acc:HGNC:4680]
GUCY2D	guanylate cyclase 2D, retinal [Source:HGNC Symbol;Acc:HGNC:4689]
HBEGF	heparin binding EGF like growth factor [Source:HGNC Symbol;Acc:HGNC:3059]
HDAC6	histone deacetylase 6 [Source:HGNC Symbol;Acc:HGNC:14064]
HERPUD1	homocysteine inducible ER protein with ubiquitin like domain 1 [Source:HGNC Symbol;Acc:HGNC:13744]
HFE	hemochromatosis [Source:HGNC Symbol;Acc:HGNC:4886]
HFE2	hemochromatosis type 2 (juvenile) [Source:HGNC Symbol;Acc:HGNC:4887]
HGF	hepatocyte growth factor [Source:HGNC Symbol;Acc:HGNC:4893]
HIST2H3PS2	histone cluster 2, H3, pseudogene 2 [Source:HGNC Symbol;Acc:HGNC:32060]
HMGCR	3-hydroxy-3-methylglutaryl-CoA reductase [Source:HGNC Symbol;Acc:HGNC:5006]
ARHGAP45	Rho GTPase activating protein 45 [Source:HGNC Symbol;Acc:HGNC:17102]
HN1	hematological and neurological expressed 1 [Source:HGNC Symbol;Acc:HGNC:14569]
HPRT1	hypoxanthine phosphoribosyltransferase 1 [Source:HGNC Symbol;Acc:HGNC:5157]
HSP90AA1	heat shock protein 90kDa alpha family class A member 1 [Source:HGNC Symbol;Acc:HGNC:5253]
HSPA5	heat shock protein family A (Hsp70) member 5 [Source:HGNC Symbol;Acc:HGNC:5238]
HSPA8	heat shock protein family A (Hsp70) member 8 [Source:HGNC Symbol;Acc:HGNC:5241]
HSPB1	heat shock protein family B (small) member 1 [Source:HGNC Symbol;Acc:HGNC:5246]
HSPB2	heat shock protein family B (small) member 2 [Source:HGNC Symbol;Acc:HGNC:5247]
HSPB3	heat shock protein family B (small) member 3 [Source:HGNC Symbol;Acc:HGNC:5248]
HTRA2	HtrA serine peptidase 2 [Source:HGNC Symbol;Acc:HGNC:14348]
HTRA4	HtrA serine peptidase 4 [Source:HGNC Symbol;Acc:HGNC:26909]
HTT	huntingtin [Source:HGNC Symbol;Acc:HGNC:4851]
ICAM2	intercellular adhesion molecule 2 [Source:HGNC Symbol;Acc:HGNC:5345]
IDH3B	isocitrate dehydrogenase 3 (NAD(+)) beta [Source:HGNC Symbol;Acc:HGNC:5385]
IER3	immediate early response 3 [Source:HGNC Symbol;Acc:HGNC:5392]
IFIH1	interferon induced with helicase C domain 1 [Source:HGNC Symbol;Acc:HGNC:18873]
IFNA1	interferon, alpha 1 [Source:HGNC Symbol;Acc:HGNC:5417]
IFNB1	interferon beta 1 [Source:HGNC Symbol;Acc:HGNC:5434]
IGF2	insulin like growth factor 2 [Source:HGNC Symbol;Acc:HGNC:5466]
IGFBP2	insulin like growth factor binding protein 2 [Source:HGNC Symbol;Acc:HGNC:5471]
IL1R1	interleukin 1 receptor type 1 [Source:HGNC Symbol;Acc:HGNC:5993]
IL23R	interleukin 23 receptor [Source:HGNC Symbol;Acc:HGNC:19100]
IL4	interleukin 4 [Source:HGNC Symbol;Acc:HGNC:6014]
IL5	interleukin 5 [Source:HGNC Symbol;Acc:HGNC:6016]
IL7	interleukin 7 [Source:HGNC Symbol;Acc:HGNC:6023]
IL9	interleukin 9 [Source:HGNC Symbol;Acc:HGNC:6029]
INS	insulin [Source:HGNC Symbol;Acc:HGNC:6081]
IQCB1	IQ motif containing B1 [Source:HGNC Symbol;Acc:HGNC:28949]
IRAK1	interleukin 1 receptor associated kinase 1 [Source:HGNC Symbol;Acc:HGNC:6112]

ISL1	ISL LIM homeobox 1 [Source:HGNC Symbol;Acc:HGNC:6132]
ITGA7	integrin subunit alpha 7 [Source:HGNC Symbol;Acc:HGNC:6143]
ITGAV	integrin subunit alpha V [Source:HGNC Symbol;Acc:HGNC:6150]
ITIH4	inter-alpha-trypsin inhibitor heavy chain family member 4 [Source:HGNC Symbol;Acc:HGNC:6169]
JAG1	jagged 1 [Source:HGNC Symbol;Acc:HGNC:6188]
JUN	Jun proto-oncogene, AP-1 transcription factor subunit [Source:HGNC Symbol;Acc:HGNC:6204]
KBTBD12	kelch repeat and BTB domain containing 12 [Source:HGNC Symbol;Acc:HGNC:25731]
KCNJ10	potassium voltage-gated channel subfamily J member 10 [Source:HGNC Symbol;Acc:HGNC:6256]
KCNJ13	potassium voltage-gated channel subfamily J member 13 [Source:HGNC Symbol;Acc:HGNC:6259]
KCNV2	potassium voltage-gated channel modifier subfamily V member 2 [Source:HGNC Symbol;Acc:HGNC:19698]
KCTD10	potassium channel tetramerization domain containing 10 [Source:HGNC Symbol;Acc:HGNC:23236]
KIT	KIT proto-oncogene receptor tyrosine kinase [Source:HGNC Symbol;Acc:HGNC:6342]
KITLG	KIT ligand [Source:HGNC Symbol;Acc:HGNC:6343]
KLF4	Kruppel like factor 4 [Source:HGNC Symbol;Acc:HGNC:6348]
KLHL7	kelch like family member 7 [Source:HGNC Symbol;Acc:HGNC:15646]
KLK4	kallikrein related peptidase 4 [Source:HGNC Symbol;Acc:HGNC:6365]
KLKB1	kallikrein B1 [Source:HGNC Symbol;Acc:HGNC:6371]
KMT2E	lysine methyltransferase 2E [Source:HGNC Symbol;Acc:HGNC:18541]
KNG1	kininogen 1 [Source:HGNC Symbol;Acc:HGNC:6383]
KRT12	keratin 12 [Source:HGNC Symbol;Acc:HGNC:6414]
KRT18	keratin 18 [Source:HGNC Symbol;Acc:HGNC:6430]
KRTAP13-4	keratin associated protein 13-4 [Source:HGNC Symbol;Acc:HGNC:18926]
LCA5	LCA5, lebercilin [Source:HGNC Symbol;Acc:HGNC:31923]
LEFTY2	left-right determination factor 2 [Source:HGNC Symbol;Acc:HGNC:3122]
LEP	leptin [Source:HGNC Symbol;Acc:HGNC:6553]
LGALS3	galectin 3 [Source:HGNC Symbol;Acc:HGNC:6563]
LHX2	LIM homeobox 2 [Source:HGNC Symbol;Acc:HGNC:6594]
LIF	leukemia inhibitory factor [Source:HGNC Symbol;Acc:HGNC:6596]
LIN28A	lin-28 homolog A [Source:HGNC Symbol;Acc:HGNC:15986]
LMNA	lamin A/C [Source:HGNC Symbol;Acc:HGNC:6636]
LOX	lysyl oxidase [Source:HGNC Symbol;Acc:HGNC:6664]
LOXL2	lysyl oxidase like 2 [Source:HGNC Symbol;Acc:HGNC:6666]
LPCAT3	lysophosphatidylcholine acyltransferase 3 [Source:HGNC Symbol;Acc:HGNC:30244]
LRAT	lecithin retinol acyltransferase (phosphatidylcholine--retinol O-acyltransferase) [Source:HGNC Symbol;Acc:HGNC:6685]
LRIT3	leucine rich repeat, Ig-like and transmembrane domains 3 [Source:HGNC Symbol;Acc:HGNC:24783]
LRRK2	leucine rich repeat kinase 2 [Source:HGNC Symbol;Acc:HGNC:18618]
LTA	lymphotoxin alpha [Source:HGNC Symbol;Acc:HGNC:6709]

LTBP2	latent transforming growth factor beta binding protein 2 [Source:HGNC Symbol;Acc:HGNC:6715]
LTBP3	latent transforming growth factor beta binding protein 3 [Source:HGNC Symbol;Acc:HGNC:6716]
LUM	lumican [Source:HGNC Symbol;Acc:HGNC:6724]
MAF	MAF bZIP transcription factor [Source:HGNC Symbol;Acc:HGNC:6776]
MAK	male germ cell associated kinase [Source:HGNC Symbol;Acc:HGNC:6816]
MAP1LC3A	microtubule associated protein 1 light chain 3 alpha [Source:HGNC Symbol;Acc:HGNC:6838]
MAP1LC3B	microtubule associated protein 1 light chain 3 beta [Source:HGNC Symbol;Acc:HGNC:13352]
MAP2K1	mitogen-activated protein kinase kinase 1 [Source:HGNC Symbol;Acc:HGNC:6840]
MAP3K5	mitogen-activated protein kinase kinase kinase 5 [Source:HGNC Symbol;Acc:HGNC:6857]
MAPT	microtubule associated protein tau [Source:HGNC Symbol;Acc:HGNC:6893]
MASP2	mannan binding lectin serine peptidase 2 [Source:HGNC Symbol;Acc:HGNC:6902]
MEPCE	methylphosphate capping enzyme [Source:HGNC Symbol;Acc:HGNC:20247]
MET	MET proto-oncogene, receptor tyrosine kinase [Source:HGNC Symbol;Acc:HGNC:7029]
METTL7B	methyltransferase like 7B [Source:HGNC Symbol;Acc:HGNC:28276]
MFF	mitochondrial fission factor [Source:HGNC Symbol;Acc:HGNC:24858]
MIF	macrophage migration inhibitory factor (glycosylation-inhibiting factor) [Source:HGNC Symbol;Acc:HGNC:7097]
MITF	melanogenesis associated transcription factor [Source:HGNC Symbol;Acc:HGNC:7105]
MPO	myeloperoxidase [Source:HGNC Symbol;Acc:HGNC:7218]
MRPL10	mitochondrial ribosomal protein L10 [Source:HGNC Symbol;Acc:HGNC:14055]
MRPL54	mitochondrial ribosomal protein L54 [Source:HGNC Symbol;Acc:HGNC:16685]
MSRA	methionine sulfoxide reductase A [Source:HGNC Symbol;Acc:HGNC:7377]
MTHFR	methylenetetrahydrofolate reductase (NAD(P)H) [Source:HGNC Symbol;Acc:HGNC:7436]
MT-ND4	mitochondrially encoded NADH:ubiquinone oxidoreductase core subunit 4 [Source:HGNC Symbol;Acc:HGNC:7459]
MYBPH	myosin binding protein H [Source:HGNC Symbol;Acc:HGNC:7552]
MYC	v-myc avian myelocytomatosis viral oncogene homolog [Source:HGNC Symbol;Acc:HGNC:7553]
MYD88	myeloid differentiation primary response 88 [Source:HGNC Symbol;Acc:HGNC:7562]
MYOC	myocilin [Source:HGNC Symbol;Acc:HGNC:7610]
NANOG	Nanog homeobox [Source:HGNC Symbol;Acc:HGNC:20857]
NCL	nucleolin [Source:HGNC Symbol;Acc:HGNC:7667]
NDP	NDP, norrin cystine knot growth factor [Source:HGNC Symbol;Acc:HGNC:7678]
NDUFA11	NADH:ubiquinone oxidoreductase subunit A11 [Source:HGNC Symbol;Acc:HGNC:20371]
NEFL	neurofilament, light polypeptide [Source:HGNC Symbol;Acc:HGNC:7739]
NES	nestin [Source:HGNC Symbol;Acc:HGNC:7756]
NEUROD1	neuronal differentiation 1 [Source:HGNC Symbol;Acc:HGNC:7762]
NFAT5	nuclear factor of activated T-cells 5, tonicity-responsive [Source:HGNC Symbol;Acc:HGNC:7774]
NFKBIA	NFKB inhibitor alpha [Source:HGNC Symbol;Acc:HGNC:7797]
NID1	nidogen 1 [Source:HGNC Symbol;Acc:HGNC:7821]
NIPSNAP3B	nipsnap homolog 3B [Source:HGNC Symbol;Acc:HGNC:23641]
NLRC4	NLR family CARD domain containing 4 [Source:HGNC Symbol;Acc:HGNC:16412]

NLRC5	NLR family CARD domain containing 5 [Source:HGNC Symbol;Acc:HGNC:29933]
NOD2	nucleotide binding oligomerization domain containing 2 [Source:HGNC Symbol;Acc:HGNC:5331]
NOG	noggin [Source:HGNC Symbol;Acc:HGNC:7866]
NOS1	nitric oxide synthase 1 [Source:HGNC Symbol;Acc:HGNC:7872]
NOS1AP	nitric oxide synthase 1 adaptor protein [Source:HGNC Symbol;Acc:HGNC:16859]
NOS3	nitric oxide synthase 3 [Source:HGNC Symbol;Acc:HGNC:7876]
NOTCH1	notch 1 [Source:HGNC Symbol;Acc:HGNC:7881]
NOX1	NADPH oxidase 1 [Source:HGNC Symbol;Acc:HGNC:7889]
NOX4	NADPH oxidase 4 [Source:HGNC Symbol;Acc:HGNC:7891]
NPIPA2	nuclear pore complex interacting protein family member A2 [Source:HGNC Symbol;Acc:HGNC:41979]
NPIPA3	nuclear pore complex interacting protein family member A3 [Source:HGNC Symbol;Acc:HGNC:41978]
NRL	neural retina leucine zipper [Source:HGNC Symbol;Acc:HGNC:8002]
NRP1	neuropilin 1 [Source:HGNC Symbol;Acc:HGNC:8004]
NRP2	neuropilin 2 [Source:HGNC Symbol;Acc:HGNC:8005]
NRTN	neurturin [Source:HGNC Symbol;Acc:HGNC:8007]
NT5DC3	5'-nucleotidase domain containing 3 [Source:HGNC Symbol;Acc:HGNC:30826]
NT5E	5'-nucleotidase ecto [Source:HGNC Symbol;Acc:HGNC:8021]
NTF3	neurotrophin 3 [Source:HGNC Symbol;Acc:HGNC:8023]
NTF4	neurotrophin 4 [Source:HGNC Symbol;Acc:HGNC:8024]
NTRK1	neurotrophic receptor tyrosine kinase 1 [Source:HGNC Symbol;Acc:HGNC:8031]
NTRK2	neurotrophic receptor tyrosine kinase 2 [Source:HGNC Symbol;Acc:HGNC:8032]
NXNL1	nucleoredoxin-like 1 [Source:HGNC Symbol;Acc:HGNC:25179]
NYAP1	neuronal tyrosine phosphorylated phosphoinositide-3-kinase adaptor 1 [Source:HGNC Symbol;Acc:HGNC:22009]
NYX	nyctalopin [Source:HGNC Symbol;Acc:HGNC:8082]
OCLN	occludin [Source:HGNC Symbol;Acc:HGNC:8104]
OLR1	oxidized low density lipoprotein receptor 1 [Source:HGNC Symbol;Acc:HGNC:8133]
OPN1LW	opsin 1 (cone pigments), long-wave-sensitive [Source:HGNC Symbol;Acc:HGNC:9936]
OPN1SW	opsin 1 (cone pigments), short-wave-sensitive [Source:HGNC Symbol;Acc:HGNC:1012]
OPN4	opsin 4 [Source:HGNC Symbol;Acc:HGNC:14449]
OPTN	optineurin [Source:HGNC Symbol;Acc:HGNC:17142]
OR10P1	olfactory receptor family 10 subfamily P member 1 [Source:HGNC Symbol;Acc:HGNC:15378]
P4HB	prolyl 4-hydroxylase subunit beta [Source:HGNC Symbol;Acc:HGNC:8548]
PARK2	parkin RBR E3 ubiquitin protein ligase [Source:HGNC Symbol;Acc:HGNC:8607]
PARK7	Parkinsonism associated deglycase [Source:HGNC Symbol;Acc:HGNC:16369]
PAX6	paired box 6 [Source:HGNC Symbol;Acc:HGNC:8620]
PCDH15	protocadherin related 15 [Source:HGNC Symbol;Acc:HGNC:14674]
PCSK9	proprotein convertase subtilisin/kexin type 9 [Source:HGNC Symbol;Acc:HGNC:20001]
PDC	phosducin [Source:HGNC Symbol;Acc:HGNC:8759]
PDE6A	phosphodiesterase 6A [Source:HGNC Symbol;Acc:HGNC:8785]

PDE6B	phosphodiesterase 6B [Source:HGNC Symbol;Acc:HGNC:8786]
PDE6C	phosphodiesterase 6C [Source:HGNC Symbol;Acc:HGNC:8787]
PDE6G	phosphodiesterase 6G [Source:HGNC Symbol;Acc:HGNC:8789]
PDE6H	phosphodiesterase 6H [Source:HGNC Symbol;Acc:HGNC:8790]
PDGFA	platelet derived growth factor subunit A [Source:HGNC Symbol;Acc:HGNC:8799]
PDGFB	platelet derived growth factor subunit B [Source:HGNC Symbol;Acc:HGNC:8800]
PDGFC	platelet derived growth factor C [Source:HGNC Symbol;Acc:HGNC:8801]
PDGFRB	platelet derived growth factor receptor beta [Source:HGNC Symbol;Acc:HGNC:8804]
PECAM1	platelet and endothelial cell adhesion molecule 1 [Source:HGNC Symbol;Acc:HGNC:8823]
PI3	peptidase inhibitor 3 [Source:HGNC Symbol;Acc:HGNC:8947]
PICALM	phosphatidylinositol binding clathrin assembly protein [Source:HGNC Symbol;Acc:HGNC:15514]
PICK1	protein interacting with PRKCA 1 [Source:HGNC Symbol;Acc:HGNC:9394]
PILRB	paired immunoglobin-like type 2 receptor beta [Source:HGNC Symbol;Acc:HGNC:18297]
PINK1	PTEN induced putative kinase 1 [Source:HGNC Symbol;Acc:HGNC:14581]
PIWIL1	piwi like RNA-mediated gene silencing 1 [Source:HGNC Symbol;Acc:HGNC:9007]
PIWIL4	piwi like RNA-mediated gene silencing 4 [Source:HGNC Symbol;Acc:HGNC:18444]
PKM	pyruvate kinase, muscle [Source:HGNC Symbol;Acc:HGNC:9021]
PKN3	protein kinase N3 [Source:HGNC Symbol;Acc:HGNC:17999]
PLA2G1B	phospholipase A2 group IB [Source:HGNC Symbol;Acc:HGNC:9030]
PLA2G6	phospholipase A2 group VI [Source:HGNC Symbol;Acc:HGNC:9039]
PLAT	plasminogen activator, tissue type [Source:HGNC Symbol;Acc:HGNC:9051]
PLAUR	plasminogen activator, urokinase receptor [Source:HGNC Symbol;Acc:HGNC:9053]
PLK1	polo like kinase 1 [Source:HGNC Symbol;Acc:HGNC:9077]
PLVAP	plasmalemma vesicle associated protein [Source:HGNC Symbol;Acc:HGNC:13635]
PLXNA4	plexin A4 [Source:HGNC Symbol;Acc:HGNC:9102]
PMEL	premelanosome protein [Source:HGNC Symbol;Acc:HGNC:10880]
POLR2E	polymerase (RNA) II subunit E [Source:HGNC Symbol;Acc:HGNC:9192]
POLR2F	polymerase (RNA) II subunit F [Source:HGNC Symbol;Acc:HGNC:9193]
POTEF	POTE ankyrin domain family member F [Source:HGNC Symbol;Acc:HGNC:33905]
POU4F1	POU class 4 homeobox 1 [Source:HGNC Symbol;Acc:HGNC:9218]
POU4F2	POU class 4 homeobox 2 [Source:HGNC Symbol;Acc:HGNC:9219]
POU5F1	POU class 5 homeobox 1 [Source:HGNC Symbol;Acc:HGNC:9221]
PPIA	peptidylprolyl isomerase A [Source:HGNC Symbol;Acc:HGNC:9253]
PPIG	peptidylprolyl isomerase G [Source:HGNC Symbol;Acc:HGNC:14650]
PRKCA	protein kinase C alpha [Source:HGNC Symbol;Acc:HGNC:9393]
PRKCB	protein kinase C beta [Source:HGNC Symbol;Acc:HGNC:9395]
PRPF3	pre-mRNA processing factor 3 [Source:HGNC Symbol;Acc:HGNC:17348]
PRPF8	pre-mRNA processing factor 8 [Source:HGNC Symbol;Acc:HGNC:17340]
PSEN1	presenilin 1 [Source:HGNC Symbol;Acc:HGNC:9508]
PTGS2	prostaglandin-endoperoxide synthase 2 [Source:HGNC Symbol;Acc:HGNC:9605]
PTK2	protein tyrosine kinase 2 [Source:HGNC Symbol;Acc:HGNC:9611]

PTK7	protein tyrosine kinase 7 (inactive) [Source:HGNC Symbol;Acc:HGNC:9618]
PTPN22	protein tyrosine phosphatase, non-receptor type 22 [Source:HGNC Symbol;Acc:HGNC:9652]
PTPRB	protein tyrosine phosphatase, receptor type B [Source:HGNC Symbol;Acc:HGNC:9665]
PTPRC	protein tyrosine phosphatase, receptor type C [Source:HGNC Symbol;Acc:HGNC:9666]
RAB28	RAB28, member RAS oncogene family [Source:HGNC Symbol;Acc:HGNC:9768]
RAB5A	RAB5A, member RAS oncogene family [Source:HGNC Symbol;Acc:HGNC:9783]
RAB7A	RAB7A, member RAS oncogene family [Source:HGNC Symbol;Acc:HGNC:9788]
RANBP3	RAN binding protein 3 [Source:HGNC Symbol;Acc:HGNC:9850]
RAPGEF1	Rap guanine nucleotide exchange factor 1 [Source:HGNC Symbol;Acc:HGNC:4568]
RASIP1	Ras interacting protein 1 [Source:HGNC Symbol;Acc:HGNC:24716]
RB1CC1	RB1 inducible coiled-coil 1 [Source:HGNC Symbol;Acc:HGNC:15574]
RBFOX3	RNA binding protein, fox-1 homolog 3 [Source:HGNC Symbol;Acc:HGNC:27097]
RBM8A	RNA binding motif protein 8A [Source:HGNC Symbol;Acc:HGNC:9905]
RBMS1	RNA binding motif single stranded interacting protein 1 [Source:HGNC Symbol;Acc:HGNC:9907]
RBMS2	RNA binding motif single stranded interacting protein 2 [Source:HGNC Symbol;Acc:HGNC:9909]
RBP1	retinol binding protein 1 [Source:HGNC Symbol;Acc:HGNC:9919]
RBP3	retinol binding protein 3 [Source:HGNC Symbol;Acc:HGNC:9921]
RBP4	retinol binding protein 4 [Source:HGNC Symbol;Acc:HGNC:9922]
RBPMS	RNA binding protein with multiple splicing [Source:HGNC Symbol;Acc:HGNC:19097]
RCVRN	recoverin [Source:HGNC Symbol;Acc:HGNC:9937]
RDH10	retinol dehydrogenase 10 (all-trans) [Source:HGNC Symbol;Acc:HGNC:19975]
RDH11	retinol dehydrogenase 11 (all-trans/9-cis/11-cis) [Source:HGNC Symbol;Acc:HGNC:17964]
RDH12	retinol dehydrogenase 12 (all-trans/9-cis/11-cis) [Source:HGNC Symbol;Acc:HGNC:19977]
RDH14	retinol dehydrogenase 14 (all-trans/9-cis/11-cis) [Source:HGNC Symbol;Acc:HGNC:19979]
REEP6	receptor accessory protein 6 [Source:HGNC Symbol;Acc:HGNC:30078]
REL	REL proto-oncogene, NF-kB subunit [Source:HGNC Symbol;Acc:HGNC:9954]
RELA	RELA proto-oncogene, NF-kB subunit [Source:HGNC Symbol;Acc:HGNC:9955]
RELB	RELB proto-oncogene, NF-kB subunit [Source:HGNC Symbol;Acc:HGNC:9956]
REN	renin [Source:HGNC Symbol;Acc:HGNC:9958]
RGS9BP	regulator of G-protein signaling 9 binding protein [Source:HGNC Symbol;Acc:HGNC:30304]
RHOA	ras homolog family member A [Source:HGNC Symbol;Acc:HGNC:667]
RIPK3	receptor interacting serine/threonine kinase 3 [Source:HGNC Symbol;Acc:HGNC:10021]
ROBO4	roundabout guidance receptor 4 [Source:HGNC Symbol;Acc:HGNC:17985]
RORB	RAR related orphan receptor B [Source:HGNC Symbol;Acc:HGNC:10259]
RP9	retinitis pigmentosa 9 (autosomal dominant) [Source:HGNC Symbol;Acc:HGNC:10288]
RRH	retinal pigment epithelium-derived rhodopsin homolog [Source:HGNC Symbol;Acc:HGNC:10450]
RTBDN	retbindin [Source:HGNC Symbol;Acc:HGNC:30310]
S1PR2	sphingosine-1-phosphate receptor 2 [Source:HGNC Symbol;Acc:HGNC:3169]
SAG	S-antigen; retina and pineal gland (arrestin) [Source:HGNC Symbol;Acc:HGNC:10521]
SARM1	sterile alpha and TIR motif containing 1 [Source:HGNC Symbol;Acc:HGNC:17074]
SARNP	SAP domain containing ribonucleoprotein [Source:HGNC Symbol;Acc:HGNC:24432]

SBNO2	strawberry notch homolog 2 (Drosophila) [Source:HGNC Symbol;Acc:HGNC:29158]
SCIN	scinderin [Source:HGNC Symbol;Acc:HGNC:21695]
SDR9C7	short chain dehydrogenase/reductase family 9C, member 7 [Source:HGNC Symbol;Acc:HGNC:29958]
SEMA3B	semaphorin 3B [Source:HGNC Symbol;Acc:HGNC:10724]
SEMA3C	semaphorin 3C [Source:HGNC Symbol;Acc:HGNC:10725]
SEMA3D	semaphorin 3D [Source:HGNC Symbol;Acc:HGNC:10726]
SEMA3F	semaphorin 3F [Source:HGNC Symbol;Acc:HGNC:10728]
SEMA3G	semaphorin 3G [Source:HGNC Symbol;Acc:HGNC:30400]
SEMA4B	semaphorin 4B [Source:HGNC Symbol;Acc:HGNC:10730]
SEMA4C	semaphorin 4C [Source:HGNC Symbol;Acc:HGNC:10731]
SEMA4D	semaphorin 4D [Source:HGNC Symbol;Acc:HGNC:10732]
SEMA4F	ssemaphorin 4F [Source:HGNC Symbol;Acc:HGNC:10734]
SEMA4G	semaphorin 4G [Source:HGNC Symbol;Acc:HGNC:10735]
SEMA5A	semaphorin 5A [Source:HGNC Symbol;Acc:HGNC:10736]
SEMA5B	semaphorin 5B [Source:HGNC Symbol;Acc:HGNC:10737]
SEMA6A	semaphorin 6A [Source:HGNC Symbol;Acc:HGNC:10738]
SEMA6B	semaphorin 6B [Source:HGNC Symbol;Acc:HGNC:10739]
SEMA6C	semaphorin 6C [Source:HGNC Symbol;Acc:HGNC:10740]
SEMA6D	semaphorin 6D [Source:HGNC Symbol;Acc:HGNC:16770]
SEMA7A	semaphorin 7A (John Milton Hagen blood group) [Source:HGNC Symbol;Acc:HGNC:10741]
SFTA3	surfactant associated 3 [Source:HGNC Symbol;Acc:HGNC:18387]
SH2D2A	SH2 domain containing 2A [Source:HGNC Symbol;Acc:HGNC:10821]
SHH	sonic hedgehog [Source:HGNC Symbol;Acc:HGNC:10848]
SIRT2	sirtuin 2 [Source:HGNC Symbol;Acc:HGNC:10886]
SIRT3	sirtuin 3 [Source:HGNC Symbol;Acc:HGNC:14931]
SIRT4	sirtuin 4 [Source:HGNC Symbol;Acc:HGNC:14932]
SIRT5	sirtuin 5 [Source:HGNC Symbol;Acc:HGNC:14933]
SIRT6	sirtuin 6 [Source:HGNC Symbol;Acc:HGNC:14934]
SIRT7	sirtuin 7 [Source:HGNC Symbol;Acc:HGNC:14935]
SIX3	SIX homeobox 3 [Source:HGNC Symbol;Acc:HGNC:10889]
SIX6	SIX homeobox 6 [Source:HGNC Symbol;Acc:HGNC:10892]
SLC12A3	solute carrier family 12 member 3 [Source:HGNC Symbol;Acc:HGNC:10912]
SLC16A3	solute carrier family 16 member 3 [Source:HGNC Symbol;Acc:HGNC:10924]
SLC1A3	solute carrier family 1 member 3 [Source:HGNC Symbol;Acc:HGNC:10941]
SLC1A7	solute carrier family 1 member 7 [Source:HGNC Symbol;Acc:HGNC:10945]
SLC24A5	solute carrier family 24 member 5 [Source:HGNC Symbol;Acc:HGNC:20611]
SLC46A1	solute carrier family 46 member 1 [Source:HGNC Symbol;Acc:HGNC:30521]
SLC4A11	solute carrier family 4 member 11 [Source:HGNC Symbol;Acc:HGNC:16438]
SMAD2	SMAD family member 2 [Source:HGNC Symbol;Acc:HGNC:6768]
SMAD3	SMAD family member 3 [Source:HGNC Symbol;Acc:HGNC:6769]
SMAD9	SMAD family member 9 [Source:HGNC Symbol;Acc:HGNC:6774]

SNAI1	snail family transcriptional repressor 1 [Source:HGNC Symbol;Acc:HGNC:11128]
SNCA	synuclein alpha [Source:HGNC Symbol;Acc:HGNC:11138]
SNCG	synuclein gamma [Source:HGNC Symbol;Acc:HGNC:11141]
SNRNP200	small nuclear ribonucleoprotein U5 subunit 200 [Source:HGNC Symbol;Acc:HGNC:30859]
SOCS3	suppressor of cytokine signaling 3 [Source:HGNC Symbol;Acc:HGNC:19391]
SOD3	superoxide dismutase 3, extracellular [Source:HGNC Symbol;Acc:HGNC:11181]
SORT1	sortilin 1 [Source:HGNC Symbol;Acc:HGNC:11186]
SOX10	SRY-box 10 [Source:HGNC Symbol;Acc:HGNC:11190]
SOX2	SRY-box 2 [Source:HGNC Symbol;Acc:HGNC:11195]
SPARC	secreted protein acidic and cysteine rich [Source:HGNC Symbol;Acc:HGNC:11219]
SPATA7	spermatogenesis associated 7 [Source:HGNC Symbol;Acc:HGNC:20423]
SPDYE3	speedy/RINGO cell cycle regulator family member E3 [Source:HGNC Symbol;Acc:HGNC:35462]
SPEF2	sperm flagellar 2 [Source:HGNC Symbol;Acc:HGNC:26293]
SPINK2	serine peptidase inhibitor, Kazal type 2 [Source:HGNC Symbol;Acc:HGNC:11245]
SPP1	secreted phosphoprotein 1 [Source:HGNC Symbol;Acc:HGNC:11255]
SRC	SRC proto-oncogene, non-receptor tyrosine kinase [Source:HGNC Symbol;Acc:HGNC:11283]
SRPK1	SRSF protein kinase 1 [Source:HGNC Symbol;Acc:HGNC:11305]
STARD3	StAR related lipid transfer domain containing 3 [Source:HGNC Symbol;Acc:HGNC:17579]
STAT1	signal transducer and activator of transcription 1 [Source:HGNC Symbol;Acc:HGNC:11362]
STRA6	stimulated by retinoic acid 6 [Source:HGNC Symbol;Acc:HGNC:30650]
STX3	syntaxin 3 [Source:HGNC Symbol;Acc:HGNC:11438]
SYP	synaptophysin [Source:HGNC Symbol;Acc:HGNC:11506]
TBL1X	transducin (beta)-like 1X-linked [Source:HGNC Symbol;Acc:HGNC:11585]
TCF7L2	transcription factor 7 like 2 [Source:HGNC Symbol;Acc:HGNC:11641]
TEK	TEK receptor tyrosine kinase [Source:HGNC Symbol;Acc:HGNC:11724]
TERT	telomerase reverse transcriptase [Source:HGNC Symbol;Acc:HGNC:11730]
TFAM	transcription factor A, mitochondrial [Source:HGNC Symbol;Acc:HGNC:11741]
TFEB	transcription factor EB [Source:HGNC Symbol;Acc:HGNC:11753]
TGFB3	transforming growth factor beta 3 [Source:HGNC Symbol;Acc:HGNC:11769]
TGFBI	transforming growth factor beta induced [Source:HGNC Symbol;Acc:HGNC:11771]
TH	tyrosine hydroxylase [Source:HGNC Symbol;Acc:HGNC:11782]
THBS2	thrombospondin 2 [Source:HGNC Symbol;Acc:HGNC:11786]
THY1	Thy-1 cell surface antigen [Source:HGNC Symbol;Acc:HGNC:11801]
TIE1	tyrosine kinase with immunoglobulin like and EGF like domains 1 [Source:HGNC Symbol;Acc:HGNC:11809]
TJP1	tight junction protein 1 [Source:HGNC Symbol;Acc:HGNC:11827]
TLR6	toll like receptor 6 [Source:HGNC Symbol;Acc:HGNC:16711]
TLR8	toll like receptor 8 [Source:HGNC Symbol;Acc:HGNC:15632]
TLR9	toll like receptor 9 [Source:HGNC Symbol;Acc:HGNC:15633]
TMCO1	transmembrane and coiled-coil domains 1 [Source:HGNC Symbol;Acc:HGNC:18188]
TMEM199	transmembrane protein 199 [Source:HGNC Symbol;Acc:HGNC:18085]
TMEM259	transmembrane protein 259 [Source:HGNC Symbol;Acc:HGNC:17039]

TNFAIP1	TNF alpha induced protein 1 [Source:HGNC Symbol;Acc:HGNC:11894]
TNFAIP6	TNF alpha induced protein 6 [Source:HGNC Symbol;Acc:HGNC:11898]
TNFRSF1A	tumor necrosis factor receptor superfamily member 1A [Source:HGNC Symbol;Acc:HGNC:11916]
TOPORS	TOP1 binding arginine/serine rich protein [Source:HGNC Symbol;Acc:HGNC:21653]
TRIP10	thyroid hormone receptor interactor 10 [Source:HGNC Symbol;Acc:HGNC:12304]
TSC22D4	TSC22 domain family member 4 [Source:HGNC Symbol;Acc:HGNC:21696]
TSPAN10	tetraspanin 10 [Source:HGNC Symbol;Acc:HGNC:29942]
TSPAN12	tetraspanin 12 [Source:HGNC Symbol;Acc:HGNC:21641]
TTR	transthyretin [Source:HGNC Symbol;Acc:HGNC:12405]
TUB	tubby bipartite transcription factor [Source:HGNC Symbol;Acc:HGNC:12406]
TUBB4A	tubulin beta 4A class IVa [Source:HGNC Symbol;Acc:HGNC:20774]
TULP1	tubby like protein 1 [Source:HGNC Symbol;Acc:HGNC:12423]
TYR	tyrosinase [Source:HGNC Symbol;Acc:HGNC:12442]
TYRP1	tyrosinase related protein 1 [Source:HGNC Symbol;Acc:HGNC:12450]
UCP2	uncoupling protein 2 [Source:HGNC Symbol;Acc:HGNC:12518]
USH2A	usherin [Source:HGNC Symbol;Acc:HGNC:12601]
VAV1	vav guanine nucleotide exchange factor 1 [Source:HGNC Symbol;Acc:HGNC:12657]
VAX2	ventral anterior homeobox 2 [Source:HGNC Symbol;Acc:HGNC:12661]
VCAM1	vascular cell adhesion molecule 1 [Source:HGNC Symbol;Acc:HGNC:12663]
VCAN	versican [Source:HGNC Symbol;Acc:HGNC:2464]
VEGFB	vascular endothelial growth factor B [Source:HGNC Symbol;Acc:HGNC:12681]
VIP	vasoactive intestinal peptide [Source:HGNC Symbol;Acc:HGNC:12693]
VKORC1	vitamin K epoxide reductase complex subunit 1 [Source:HGNC Symbol;Acc:HGNC:23663]
VMAC	vimentin-type intermediate filament associated coiled-coil protein [Source:HGNC Symbol;Acc:HGNC:33803]
VSIG4	V-set and immunoglobulin domain containing 4 [Source:HGNC Symbol;Acc:HGNC:17032]
VSX2	visual system homeobox 2 [Source:HGNC Symbol;Acc:HGNC:1975]
WDR18	WD repeat domain 18 [Source:HGNC Symbol;Acc:HGNC:17956]
WDR36	WD repeat domain 36 [Source:HGNC Symbol;Acc:HGNC:30696]
WNT3A	Wnt family member 3A [Source:HGNC Symbol;Acc:HGNC:15983]
XBP1	X-box binding protein 1 [Source:HGNC Symbol;Acc:HGNC:12801]
XPO5	exportin 5 [Source:HGNC Symbol;Acc:HGNC:17675]
ZC3H12A	zinc finger CCCH-type containing 12A [Source:HGNC Symbol;Acc:HGNC:26259]
ZPR1	ZPR1 zinc finger [Source:HGNC Symbol;Acc:HGNC:13051]
CD14	CD14 molecule [Source:HGNC Symbol;Acc:HGNC:1628]
RASGRP1	RAS guanyl releasing protein 1 [Source:HGNC Symbol;Acc:HGNC:9878]
GAS1	growth arrest specific 1 [Source:HGNC Symbol;Acc:HGNC:4165]
SLC39A8	solute carrier family 39 member 8 [Source:HGNC Symbol;Acc:HGNC:20862]
TNFSF10	tumor necrosis factor superfamily member 10 [Source:HGNC Symbol;Acc:HGNC:11925]
E2F8	E2F transcription factor 8 [Source:HGNC Symbol;Acc:HGNC:24727]
CD74	CD74 molecule [Source:HGNC Symbol;Acc:HGNC:1697]

CXCL2	C-X-C motif chemokine ligand 2 [Source:HGNC Symbol;Acc:HGNC:4603]
PPP1R16B	protein phosphatase 1 regulatory subunit 16B [Source:HGNC Symbol;Acc:HGNC:15850]
SLC2A14	solute carrier family 2 member 14 [Source:HGNC Symbol;Acc:HGNC:18301]
PLA2G4A	phospholipase A2 group IVA [Source:HGNC Symbol;Acc:HGNC:9035]
LSM3	LSM3 homolog, U6 small nuclear RNA and mRNA degradation associated [Source:HGNC Symbol;Acc:HGNC:17874]
ZNF711	zinc finger protein 711 [Source:HGNC Symbol;Acc:HGNC:13128]
AKR1B10	aldo-keto reductase family 1 member B10 [Source:HGNC Symbol;Acc:HGNC:382]
S100A9	S100 calcium binding protein A9 [Source:HGNC Symbol;Acc:HGNC:10499]
TTC9	tetratricopeptide repeat domain 9 [Source:HGNC Symbol;Acc:HGNC:20267]
GPX2	glutathione peroxidase 2 [Source:HGNC Symbol;Acc:HGNC:4554]
GPT	glutamic-pyruvate transaminase (alanine aminotransferase) [Source:HGNC Symbol;Acc:HGNC:4552]
AREG	amphiregulin [Source:HGNC Symbol;Acc:HGNC:651]
PAK3	p21 (RAC1) activated kinase 3 [Source:HGNC Symbol;Acc:HGNC:8592]
PDK4	pyruvate dehydrogenase kinase 4 [Source:HGNC Symbol;Acc:HGNC:8812]
PIK3R1	phosphoinositide-3-kinase regulatory subunit 1 [Source:HGNC Symbol;Acc:HGNC:8979]
PRY	PTPN13-like, Y-linked [Source:HGNC Symbol;Acc:HGNC:14024]
JCHAIN	joining chain of multimeric IgA and IgM [Source:HGNC Symbol;Acc:HGNC:5713]
MYL2	myosin light chain 2 [Source:HGNC Symbol;Acc:HGNC:7583]
SRR	serine racemase [Source:HGNC Symbol;Acc:HGNC:14398]
ALPL	alkaline phosphatase, liver/bone/kidney [Source:HGNC Symbol;Acc:HGNC:438]
MAOA	monoamine oxidase A [Source:HGNC Symbol;Acc:HGNC:6833]
NME4	NME/NM23 nucleoside diphosphate kinase 4 [Source:HGNC Symbol;Acc:HGNC:7852]
PLXDC2	plexin domain containing 2 [Source:HGNC Symbol;Acc:HGNC:21013]
IGFBP3	insulin like growth factor binding protein 3 [Source:HGNC Symbol;Acc:HGNC:5472]
MTF1	metal-regulatory transcription factor 1 [Source:HGNC Symbol;Acc:HGNC:7428]
RPL17	ribosomal protein L17 [Source:HGNC Symbol;Acc:HGNC:10307]
MAP3K14	mitogen-activated protein kinase kinase kinase 14 [Source:HGNC Symbol;Acc:HGNC:6853]
IRF6	interferon regulatory factor 6 [Source:HGNC Symbol;Acc:HGNC:6121]
NCOA7	nuclear receptor coactivator 7 [Source:HGNC Symbol;Acc:HGNC:21081]
IFI44	interferon induced protein 44 [Source:HGNC Symbol;Acc:HGNC:16938]
ACACA	acetyl-CoA carboxylase alpha [Source:HGNC Symbol;Acc:HGNC:84]
STEAP4	STEAP4 metalloreductase [Source:HGNC Symbol;Acc:HGNC:21923]
GPRC5A	G protein-coupled receptor class C group 5 member A [Source:HGNC Symbol;Acc:HGNC:9836]
CNN3	calponin 3 [Source:HGNC Symbol;Acc:HGNC:2157]
STK11	serine/threonine kinase 11 [Source:HGNC Symbol;Acc:HGNC:11389]
SLC7A7	solute carrier family 7 member 7 [Source:HGNC Symbol;Acc:HGNC:11065]
EGLN1	egl-9 family hypoxia inducible factor 1 [Source:HGNC Symbol;Acc:HGNC:1232]
PRSS8	protease, serine 8 [Source:HGNC Symbol;Acc:HGNC:9491]
BST2	bone marrow stromal cell antigen 2 [Source:HGNC Symbol;Acc:HGNC:1119]

TRPC6	transient receptor potential cation channel subfamily C member 6 [Source:HGNC Symbol;Acc:HGNC:12338]
IHH	indian hedgehog [Source:HGNC Symbol;Acc:HGNC:5956]
L1CAM	L1 cell adhesion molecule [Source:HGNC Symbol;Acc:HGNC:6470]
ETV5	ETS variant 5 [Source:HGNC Symbol;Acc:HGNC:3494]
SMC4	structural maintenance of chromosomes 4 [Source:HGNC Symbol;Acc:HGNC:14013]
LAMB3	laminin subunit beta 3 [Source:HGNC Symbol;Acc:HGNC:6490]
NPC1	NPC intracellular cholesterol transporter 1 [Source:HGNC Symbol;Acc:HGNC:7897]
NR5A1	nuclear receptor subfamily 5 group A member 1 [Source:HGNC Symbol;Acc:HGNC:7983]
DLD	dihydrolipoamide dehydrogenase [Source:HGNC Symbol;Acc:HGNC:2898]
RAC2	ras-related C3 botulinum toxin substrate 2 (rho family, small GTP binding protein Rac2) [Source:HGNC Symbol;Acc:HGNC:9802]
TP63	tumor protein p63 [Source:HGNC Symbol;Acc:HGNC:15979]
TPD52L1	tumor protein D52-like 1 [Source:HGNC Symbol;Acc:HGNC:12006]
SCP2	sterol carrier protein 2 [Source:HGNC Symbol;Acc:HGNC:10606]
RSAD2	radical S-adenosyl methionine domain containing 2 [Source:HGNC Symbol;Acc:HGNC:30908]
SLC5A1	solute carrier family 5 member 1 [Source:HGNC Symbol;Acc:HGNC:11036]
KRT5	keratin 5 [Source:HGNC Symbol;Acc:HGNC:6442]
TGIF1	TGFB induced factor homeobox 1 [Source:HGNC Symbol;Acc:HGNC:11776]
MSH6	mutS homolog 6 [Source:HGNC Symbol;Acc:HGNC:7329]
TES	testin LIM domain protein [Source:HGNC Symbol;Acc:HGNC:14620]
NDUFS1	NADH:ubiquinone oxidoreductase core subunit S1 [Source:HGNC Symbol;Acc:HGNC:7707]
PTGDS	prostaglandin D2 synthase [Source:HGNC Symbol;Acc:HGNC:9592]
CTSH	cathepsin H [Source:HGNC Symbol;Acc:HGNC:2535]
MAP1B	microtubule associated protein 1B [Source:HGNC Symbol;Acc:HGNC:6836]
VCL	vinculin [Source:HGNC Symbol;Acc:HGNC:12665]
ACSL5	acyl-CoA synthetase long-chain family member 5 [Source:HGNC Symbol;Acc:HGNC:16526]
MUC1	mucin 1, cell surface associated [Source:HGNC Symbol;Acc:HGNC:7508]
ACSL3	acyl-CoA synthetase long-chain family member 3 [Source:HGNC Symbol;Acc:HGNC:3570]
MGST3	microsomal glutathione S-transferase 3 [Source:HGNC Symbol;Acc:HGNC:7064]
S100P	S100 calcium binding protein P [Source:HGNC Symbol;Acc:HGNC:10504]
ACACB	acetyl-CoA carboxylase beta [Source:HGNC Symbol;Acc:HGNC:85]
PPP1R1B	protein phosphatase 1 regulatory inhibitor subunit 1B [Source:HGNC Symbol;Acc:HGNC:9287]
KYNU	kynureninase [Source:HGNC Symbol;Acc:HGNC:6469]
SDHA	succinate dehydrogenase complex flavoprotein subunit A [Source:HGNC Symbol;Acc:HGNC:10680]
NFATC1	nuclear factor of activated T-cells 1 [Source:HGNC Symbol;Acc:HGNC:7775]
SULF2	sulfatase 2 [Source:HGNC Symbol;Acc:HGNC:20392]
GPAM	glycerol-3-phosphate acyltransferase, mitochondrial [Source:HGNC Symbol;Acc:HGNC:24865]
CXCL14	C-X-C motif chemokine ligand 14 [Source:HGNC Symbol;Acc:HGNC:10640]
HSF1	heat shock transcription factor 1 [Source:HGNC Symbol;Acc:HGNC:5224]
TPM3	tropomyosin 3 [Source:HGNC Symbol;Acc:HGNC:12012]

NEDD9	neural precursor cell expressed, developmentally down-regulated 9 [Source:HGNC Symbol;Acc:HGNC:7733]
CCDC175	coiled-coil domain containing 175 [Source:HGNC Symbol;Acc:HGNC:19847]
WEE1	WEE1 G2 checkpoint kinase [Source:HGNC Symbol;Acc:HGNC:12761]
BAX	BCL2 associated X protein [Source:HGNC Symbol;Acc:HGNC:959]
PRKAA1	protein kinase AMP-activated catalytic subunit alpha 1 [Source:HGNC Symbol;Acc:HGNC:9376]
SPANXD	SPANX family member D [Source:HGNC Symbol;Acc:HGNC:14332]
ISG15	ISG15 ubiquitin-like modifier [Source:HGNC Symbol;Acc:HGNC:4053]
CST4	cystatin S [Source:HGNC Symbol;Acc:HGNC:2476]
MCM4	minichromosome maintenance complex component 4 [Source:HGNC Symbol;Acc:HGNC:6947]
DNAJB4	DnaJ heat shock protein family (Hsp40) member B4 [Source:HGNC Symbol;Acc:HGNC:14886]
ATP1B1	ATPase Na+/K+ transporting subunit beta 1 [Source:HGNC Symbol;Acc:HGNC:804]
CDKN2B	cyclin dependent kinase inhibitor 2B [Source:HGNC Symbol;Acc:HGNC:1788]
CYP26A1	cytochrome P450 family 26 subfamily A member 1 [Source:HGNC Symbol;Acc:HGNC:2603]
BTG1	B-cell translocation gene 1, anti-proliferative [Source:HGNC Symbol;Acc:HGNC:1130]
S100A6	S100 calcium binding protein A6 [Source:HGNC Symbol;Acc:HGNC:10496]
TPTE	transmembrane phosphatase with tensin homology [Source:HGNC Symbol;Acc:HGNC:12023]
GSN	gelsolin [Source:HGNC Symbol;Acc:HGNC:4620]
VDAC1	voltage dependent anion channel 1 [Source:HGNC Symbol;Acc:HGNC:12669]
FST	follistatin [Source:HGNC Symbol;Acc:HGNC:3971]
G0S2	G0/G1 switch 2 [Source:HGNC Symbol;Acc:HGNC:30229]
SLC40A1	solute carrier family 40 member 1 [Source:HGNC Symbol;Acc:HGNC:10909]
MGLL	monoglyceride lipase [Source:HGNC Symbol;Acc:HGNC:17038]
ANXA3	annexin A3 [Source:HGNC Symbol;Acc:HGNC:541]
DNAJB1	DnaJ heat shock protein family (Hsp40) member B1 [Source:HGNC Symbol;Acc:HGNC:5270]
CPO	carboxypeptidase O [Source:HGNC Symbol;Acc:HGNC:21011]
HMGCS2	3-hydroxy-3-methylglutaryl-CoA synthase 2 [Source:HGNC Symbol;Acc:HGNC:5008]
KRT19	keratin 19 [Source:HGNC Symbol;Acc:HGNC:6436]
RAC1	ras-related C3 botulinum toxin substrate 1 (rho family, small GTP binding protein Rac1) [Source:HGNC Symbol;Acc:HGNC:9801]
CHUK	conserved helix-loop-helix ubiquitous kinase [Source:HGNC Symbol;Acc:HGNC:1974]
MRPL44	mitochondrial ribosomal protein L44 [Source:HGNC Symbol;Acc:HGNC:16650]
CCNE2	cyclin E2 [Source:HGNC Symbol;Acc:HGNC:1590]
EPGN	epithelial mitogen [Source:HGNC Symbol;Acc:HGNC:17470]
NRG1	neuregulin 1 [Source:HGNC Symbol;Acc:HGNC:7997]
NUPR1	nuclear protein 1, transcriptional regulator [Source:HGNC Symbol;Acc:HGNC:29990]
NT5M	5',3'-nucleotidase, mitochondrial [Source:HGNC Symbol;Acc:HGNC:15769]
ZNF91	zinc finger protein 91 [Source:HGNC Symbol;Acc:HGNC:13166]
MAOB	monoamine oxidase B [Source:HGNC Symbol;Acc:HGNC:6834]
NDUFA4L2	NADH dehydrogenase (ubiquinone) 1 alpha subcomplex, 4-like 2 [Source:HGNC Symbol;Acc:HGNC:29836]
SDR16C5	short chain dehydrogenase/reductase family 16C, member 5 [Source:HGNC Symbol;Acc:HGNC:30311]

E2F1	E2F transcription factor 1 [Source:HGNC Symbol;Acc:HGNC:3113]
ZNF33B	zinc finger protein 33B [Source:HGNC Symbol;Acc:HGNC:13097]
PPP1R15A	protein phosphatase 1 regulatory subunit 15A [Source:HGNC Symbol;Acc:HGNC:14375]
SUSD5	sushi domain containing 5 [Source:HGNC Symbol;Acc:HGNC:29061]
ANXA1	annexin A1 [Source:HGNC Symbol;Acc:HGNC:533]
GSTM3	glutathione S-transferase mu 3 (brain) [Source:HGNC Symbol;Acc:HGNC:4635]
MSANTD4	Myb/SANT DNA binding domain containing 4 with coiled-coils [Source:HGNC Symbol;Acc:HGNC:29383]
RRM2	ribonucleotide reductase regulatory subunit M2 [Source:HGNC Symbol;Acc:HGNC:10452]
MXRA5	matrix remodeling associated 5 [Source:HGNC Symbol;Acc:HGNC:7539]
SP1	Sp1 transcription factor [Source:HGNC Symbol;Acc:HGNC:11205]
CD209	CD209 molecule [Source:HGNC Symbol;Acc:HGNC:1641]
TDP2	tyrosyl-DNA phosphodiesterase 2 [Source:HGNC Symbol;Acc:HGNC:17768]
KLK7	kallikrein related peptidase 7 [Source:HGNC Symbol;Acc:HGNC:6368]
FTH1	ferritin heavy chain 1 [Source:HGNC Symbol;Acc:HGNC:3976]
DSG1	desmoglein 1 [Source:HGNC Symbol;Acc:HGNC:3048]
POU1F1	POU class 1 homeobox 1 [Source:HGNC Symbol;Acc:HGNC:9210]
SCD	stearoyl-CoA desaturase [Source:HGNC Symbol;Acc:HGNC:10571]
CSTF2T	cleavage stimulation factor subunit 2, tau variant [Source:HGNC Symbol;Acc:HGNC:17086]
GADD45B	growth arrest and DNA damage inducible beta [Source:HGNC Symbol;Acc:HGNC:4096]
KRAS	KRAS proto-oncogene, GTPase [Source:HGNC Symbol;Acc:HGNC:6407]
MGMT	O-6-methylguanine-DNA methyltransferase [Source:HGNC Symbol;Acc:HGNC:7059]
PIGU	phosphatidylinositol glycan anchor biosynthesis class U [Source:HGNC Symbol;Acc:HGNC:15791]
DOK7	docking protein 7 [Source:HGNC Symbol;Acc:HGNC:26594]
FANCF	Fanconi anemia complementation group F [Source:HGNC Symbol;Acc:HGNC:3587]
AKR1C3	aldo-keto reductase family 1 member C3 [Source:HGNC Symbol;Acc:HGNC:386]
ALOX5	arachidonate 5-lipoxygenase [Source:HGNC Symbol;Acc:HGNC:435]
ATG101	autophagy related 101 [Source:HGNC Symbol;Acc:HGNC:25679]
ATP6V1B1	ATPase H+ transporting V1 subunit B1 [Source:HGNC Symbol;Acc:HGNC:853]
IKBKB	inhibitor of kappa light polypeptide gene enhancer in B-cells, kinase beta [Source:HGNC Symbol;Acc:HGNC:5960]
GSTA4	glutathione S-transferase alpha 4 [Source:HGNC Symbol;Acc:HGNC:4629]
INTS5	integrator complex subunit 5 [Source:HGNC Symbol;Acc:HGNC:29352]
TMEM60	transmembrane protein 60 [Source:HGNC Symbol;Acc:HGNC:21754]
IL1RN	interleukin 1 receptor antagonist [Source:HGNC Symbol;Acc:HGNC:6000]
TMPRSS3	transmembrane protease, serine 3 [Source:HGNC Symbol;Acc:HGNC:11877]
YIF1B	Yip1 interacting factor homolog B, membrane trafficking protein [Source:HGNC Symbol;Acc:HGNC:30511]
ULBP2	UL16 binding protein 2 [Source:HGNC Symbol;Acc:HGNC:14894]
ZNF467	zinc finger protein 467 [Source:HGNC Symbol;Acc:HGNC:23154]
HES4	hes family bHLH transcription factor 4 [Source:HGNC Symbol;Acc:HGNC:24149]
ARID3B	AT-rich interaction domain 3B [Source:HGNC Symbol;Acc:HGNC:14350]

CPNE7	copine 7 [Source:HGNC Symbol;Acc:HGNC:2320]
NUP62CL	nucleoporin 62 C-terminal like [Source:HGNC Symbol;Acc:HGNC:25960]
POLR2D	polymerase (RNA) II subunit D [Source:HGNC Symbol;Acc:HGNC:9191]
SCML2	sex comb on midleg-like 2 (Drosophila) [Source:HGNC Symbol;Acc:HGNC:10581]
ZNF462	zinc finger protein 462 [Source:HGNC Symbol;Acc:HGNC:21684]
NEIL2	nei like DNA glycosylase 2 [Source:HGNC Symbol;Acc:HGNC:18956]
OLAH	oleoyl-ACP hydrolase [Source:HGNC Symbol;Acc:HGNC:25625]
DCTN6	dynactin subunit 6 [Source:HGNC Symbol;Acc:HGNC:16964]
NPAS3	neuronal PAS domain protein 3 [Source:HGNC Symbol;Acc:HGNC:19311]
ZNF385B	zinc finger protein 385B [Source:HGNC Symbol;Acc:HGNC:26332]
ARL6	ADP ribosylation factor like GTPase 6 [Source:HGNC Symbol;Acc:HGNC:13210]
LRRC34	leucine rich repeat containing 34 [Source:HGNC Symbol;Acc:HGNC:28408]
PRSS16	protease, serine 16 [Source:HGNC Symbol;Acc:HGNC:9480]
DCTN3	dynactin subunit 3 [Source:HGNC Symbol;Acc:HGNC:2713]
LY6K	lymphocyte antigen 6 complex, locus K [Source:HGNC Symbol;Acc:HGNC:24225]
PRR15L	proline rich 15 like [Source:HGNC Symbol;Acc:HGNC:28149]
UPK1B	uroplakin 1B [Source:HGNC Symbol;Acc:HGNC:12578]
SLC26A11	solute carrier family 26 member 11 [Source:HGNC Symbol;Acc:HGNC:14471]
DERL2	derlin 2 [Source:HGNC Symbol;Acc:HGNC:17943]
MS4A6A	membrane spanning 4-domains A6A [Source:HGNC Symbol;Acc:HGNC:13375]
BTC	betacellulin [Source:HGNC Symbol;Acc:HGNC:1121]
SCEL	sciellin [Source:HGNC Symbol;Acc:HGNC:10573]
CISD2	CDGSH iron sulfur domain 2 [Source:HGNC Symbol;Acc:HGNC:24212]
MRPS16	mitochondrial ribosomal protein S16 [Source:HGNC Symbol;Acc:HGNC:14048]
MSI1	musashi RNA binding protein 1 [Source:HGNC Symbol;Acc:HGNC:7330]
WBP11	WW domain binding protein 11 [Source:HGNC Symbol;Acc:HGNC:16461]
HOXC6	homeobox C6 [Source:HGNC Symbol;Acc:HGNC:5128]
WAS	Wiskott-Aldrich syndrome [Source:HGNC Symbol;Acc:HGNC:12731]
GXYLT1	glucoside xylosyltransferase 1 [Source:HGNC Symbol;Acc:HGNC:27482]
KLK10	kallikrein related peptidase 10 [Source:HGNC Symbol;Acc:HGNC:6358]
KRT6A	keratin 6A [Source:HGNC Symbol;Acc:HGNC:6443]
OGFOD1	2-oxoglutarate and iron dependent oxygenase domain containing 1 [Source:HGNC Symbol;Acc:HGNC:25585]
CHRND	cholinergic receptor nicotinic delta subunit [Source:HGNC Symbol;Acc:HGNC:1965]
PLAU	plasminogen activator, urokinase [Source:HGNC Symbol;Acc:HGNC:9052]
GTF3A	general transcription factor IIIA [Source:HGNC Symbol;Acc:HGNC:4662]
MYF5	myogenic factor 5 [Source:HGNC Symbol;Acc:HGNC:7565]
RRAGA	Ras related GTP binding A [Source:HGNC Symbol;Acc:HGNC:16963]
PARVB	parvin beta [Source:HGNC Symbol;Acc:HGNC:14653]
PKP3	plakophilin 3 [Source:HGNC Symbol;Acc:HGNC:9025]
SAMD5	sterile alpha motif domain containing 5 [Source:HGNC Symbol;Acc:HGNC:21180]
AP1S3	adaptor related protein complex 1 sigma 3 subunit [Source:HGNC Symbol;Acc:HGNC:18971]

ADH1B	alcohol dehydrogenase 1B (class I), beta polypeptide [Source:HGNC Symbol;Acc:HGNC:250]
TMSB15A	thymosin beta 15a [Source:HGNC Symbol;Acc:HGNC:30744]
CHST8	carbohydrate sulfotransferase 8 [Source:HGNC Symbol;Acc:HGNC:15993]
WWP2	WW domain containing E3 ubiquitin protein ligase 2 [Source:HGNC Symbol;Acc:HGNC:16804]
CNOT7	CCR4-NOT transcription complex subunit 7 [Source:HGNC Symbol;Acc:HGNC:14101]
RBM34	RNA binding motif protein 34 [Source:HGNC Symbol;Acc:HGNC:28965]
CYB5R2	cytochrome b5 reductase 2 [Source:HGNC Symbol;Acc:HGNC:24376]
GIT2	GIT ArfGAP 2 [Source:HGNC Symbol;Acc:HGNC:4273]
NDUFB7	NADH:ubiquinone oxidoreductase subunit B7 [Source:HGNC Symbol;Acc:HGNC:7702]
FUT1	fucosyltransferase 1 (H blood group) [Source:HGNC Symbol;Acc:HGNC:4012]
CAPZA2	capping actin protein of muscle Z-line alpha subunit 2 [Source:HGNC Symbol;Acc:HGNC:1490]
MANSC1	MANSC domain containing 1 [Source:HGNC Symbol;Acc:HGNC:25505]
KRR1	KRR1, small subunit processome component homolog [Source:HGNC Symbol;Acc:HGNC:5176]
GSTA2	glutathione S-transferase alpha 2 [Source:HGNC Symbol;Acc:HGNC:4627]
NUDT16L1	nudix hydrolase 16 like 1 [Source:HGNC Symbol;Acc:HGNC:28154]
ORMDL2	ORMDL sphingolipid biosynthesis regulator 2 [Source:HGNC Symbol;Acc:HGNC:16037]
HYI	hydroxypyruvate isomerase (putative) [Source:HGNC Symbol;Acc:HGNC:26948]
MPV17L	MPV17 mitochondrial inner membrane protein like [Source:HGNC Symbol;Acc:HGNC:26827]
CPT1A	carnitine palmitoyltransferase 1A [Source:HGNC Symbol;Acc:HGNC:2328]
IL12B	interleukin 12B [Source:HGNC Symbol;Acc:HGNC:5970]
RPS6KB1	ribosomal protein S6 kinase B1 [Source:HGNC Symbol;Acc:HGNC:10436]
CYYR1	cysteine and tyrosine rich 1 [Source:HGNC Symbol;Acc:HGNC:16274]
SERTAD4	SERTA domain containing 4 [Source:HGNC Symbol;Acc:HGNC:25236]
ALYREF	Aly/REF export factor [Source:HGNC Symbol;Acc:HGNC:19071]
LRR1	leucine rich repeat protein 1 [Source:HGNC Symbol;Acc:HGNC:19742]
TSPAN31	tetraspanin 31 [Source:HGNC Symbol;Acc:HGNC:10539]
FN3K	fructosamine 3 kinase [Source:HGNC Symbol;Acc:HGNC:24822]
SLC30A4	solute carrier family 30 member 4 [Source:HGNC Symbol;Acc:HGNC:11015]
GSTA1	glutathione S-transferase alpha 1 [Source:HGNC Symbol;Acc:HGNC:4626]
AP2A1	adaptor related protein complex 2 alpha 1 subunit [Source:HGNC Symbol;Acc:HGNC:561]
SLC39A1	solute carrier family 39 member 1 [Source:HGNC Symbol;Acc:HGNC:12876]
OGDHL	oxoglutarate dehydrogenase-like [Source:HGNC Symbol;Acc:HGNC:25590]
COMMD10	COMM domain containing 10 [Source:HGNC Symbol;Acc:HGNC:30201]
NEIL1	nei like DNA glycosylase 1 [Source:HGNC Symbol;Acc:HGNC:18448]
HSPA1A	heat shock protein family A (Hsp70) member 1A [Source:HGNC Symbol;Acc:HGNC:5232]
SF3B3	splicing factor 3b subunit 3 [Source:HGNC Symbol;Acc:HGNC:10770]
PPP1R14C	protein phosphatase 1 regulatory inhibitor subunit 14C [Source:HGNC Symbol;Acc:HGNC:14952]
RAB25	RAB25, member RAS oncogene family [Source:HGNC Symbol;Acc:HGNC:18238]
CYBRD1	cytochrome b reductase 1 [Source:HGNC Symbol;Acc:HGNC:20797]
FNTB	farnesyltransferase, CAAX box, beta [Source:HGNC Symbol;Acc:HGNC:3785]
MRTO4	MRT4 homolog, ribosome maturation factor [Source:HGNC Symbol;Acc:HGNC:18477]

TWIST2	twist family bHLH transcription factor 2 [Source:HGNC Symbol;Acc:HGNC:20670]
SPP2	secreted phosphoprotein 2 [Source:HGNC Symbol;Acc:HGNC:11256]
OASL	2'-5'-oligoadenylate synthetase like [Source:HGNC Symbol;Acc:HGNC:8090]
GDPD3	glycerophosphodiester phosphodiesterase domain containing 3 [Source:HGNC Symbol;Acc:HGNC:28638]
PLEKHF2	pleckstrin homology and FYVE domain containing 2 [Source:HGNC Symbol;Acc:HGNC:20757]
SEC14L1	SEC14 like lipid binding 1 [Source:HGNC Symbol;Acc:HGNC:10698]
TRIM13	tripartite motif containing 13 [Source:HGNC Symbol;Acc:HGNC:9976]
ADH7	alcohol dehydrogenase 7 (class IV), mu or sigma polypeptide [Source:HGNC Symbol;Acc:HGNC:256]
FAHD1	fumarylacetoacetate hydrolase domain containing 1 [Source:HGNC Symbol;Acc:HGNC:14169]
FAM102B	family with sequence similarity 102 member B [Source:HGNC Symbol;Acc:HGNC:27637]
CERS4	ceramide synthase 4 [Source:HGNC Symbol;Acc:HGNC:23747]
DNASE2	deoxyribonuclease II, lysosomal [Source:HGNC Symbol;Acc:HGNC:2960]
GLMP	glycosylated lysosomal membrane protein [Source:HGNC Symbol;Acc:HGNC:29436]
HLA-F	major histocompatibility complex, class I, F [Source:HGNC Symbol;Acc:HGNC:4963]
TMX4	thioredoxin related transmembrane protein 4 [Source:HGNC Symbol;Acc:HGNC:25237]
RAB11FIP1	RAB11 family interacting protein 1 [Source:HGNC Symbol;Acc:HGNC:30265]
GABARAP	GABA type A receptor-associated protein [Source:HGNC Symbol;Acc:HGNC:4067]
RAC3	ras-related C3 botulinum toxin substrate 3 (rho family, small GTP binding protein Rac3) [Source:HGNC Symbol;Acc:HGNC:9803]
SPATA20	spermatogenesis associated 20 [Source:HGNC Symbol;Acc:HGNC:26125]
CD3D	CD3d molecule [Source:HGNC Symbol;Acc:HGNC:1673]
ALDH1A1	aldehyde dehydrogenase 1 family member A1 [Source:HGNC Symbol;Acc:HGNC:402]
GP1BB	glycoprotein Ib platelet beta subunit [Source:HGNC Symbol;Acc:HGNC:4440]
HNRNPH2	heterogeneous nuclear ribonucleoprotein H2 (H') [Source:HGNC Symbol;Acc:HGNC:5042]
SYTL5	synaptotagmin like 5 [Source:HGNC Symbol;Acc:HGNC:15589]
TSEN15	tRNA splicing endonuclease subunit 15 [Source:HGNC Symbol;Acc:HGNC:16791]
SLC35F2	solute carrier family 35 member F2 [Source:HGNC Symbol;Acc:HGNC:23615]
FABP6	fatty acid binding protein 6 [Source:HGNC Symbol;Acc:HGNC:3561]
PIK3C2B	phosphatidylinositol-4-phosphate 3-kinase catalytic subunit type 2 beta [Source:HGNC Symbol;Acc:HGNC:8972]
BEX4	brain expressed X-linked 4 [Source:HGNC Symbol;Acc:HGNC:25475]
COX11	COX11 cytochrome c oxidase copper chaperone [Source:HGNC Symbol;Acc:HGNC:2261]
SLC6A14	solute carrier family 6 member 14 [Source:HGNC Symbol;Acc:HGNC:11047]
SYTL4	synaptotagmin like 4 [Source:HGNC Symbol;Acc:HGNC:15588]
TIMM23	translocase of inner mitochondrial membrane 23 [Source:HGNC Symbol;Acc:HGNC:17312]
KLK8	kallikrein related peptidase 8 [Source:HGNC Symbol;Acc:HGNC:6369]
SERPINA5	serpin family A member 5 [Source:HGNC Symbol;Acc:HGNC:8723]
BCHE	butyrylcholinesterase [Source:HGNC Symbol;Acc:HGNC:983]
AIF1L	allograft inflammatory factor 1 like [Source:HGNC Symbol;Acc:HGNC:28904]
ARPC4	actin related protein 2/3 complex subunit 4 [Source:HGNC Symbol;Acc:HGNC:707]
ASH1L	ASH1 like histone lysine methyltransferase [Source:HGNC Symbol;Acc:HGNC:19088]

DDX18	DEAD-box helicase 18 [Source:HGNC Symbol;Acc:HGNC:2741]
HRG	histidine rich glycoprotein [Source:HGNC Symbol;Acc:HGNC:5181]
MCCC1	methylcrotonoyl-CoA carboxylase 1 [Source:HGNC Symbol;Acc:HGNC:6936]
ST6GALNAC2	ST6 N-acetylgalactosaminide alpha-2,6-sialyltransferase 2 [Source:HGNC Symbol;Acc:HGNC:10867]
TMC5	transmembrane channel like 5 [Source:HGNC Symbol;Acc:HGNC:22999]
RNF19B	ring finger protein 19B [Source:HGNC Symbol;Acc:HGNC:26886]
GPM6B	glycoprotein M6B [Source:HGNC Symbol;Acc:HGNC:4461]
FGF4	fibroblast growth factor 4 [Source:HGNC Symbol;Acc:HGNC:3682]
MRPL13	mitochondrial ribosomal protein L13 [Source:HGNC Symbol;Acc:HGNC:14278]
MRPL4	mitochondrial ribosomal protein L4 [Source:HGNC Symbol;Acc:HGNC:14276]
USP47	ubiquitin specific peptidase 47 [Source:HGNC Symbol;Acc:HGNC:20076]
VAMP5	vesicle associated membrane protein 5 [Source:HGNC Symbol;Acc:HGNC:12646]
DMKN	dermokine [Source:HGNC Symbol;Acc:HGNC:25063]
ERMP1	endoplasmic reticulum metallopeptidase 1 [Source:HGNC Symbol;Acc:HGNC:23703]
FLOT1	flotillin 1 [Source:HGNC Symbol;Acc:HGNC:3757]
PDHX	pyruvate dehydrogenase complex component X [Source:HGNC Symbol;Acc:HGNC:21350]
SAC3D1	SAC3 domain containing 1 [Source:HGNC Symbol;Acc:HGNC:30179]
SCAMP5	secretory carrier membrane protein 5 [Source:HGNC Symbol;Acc:HGNC:30386]
SNRPA	small nuclear ribonucleoprotein polypeptide A [Source:HGNC Symbol;Acc:HGNC:11151]
TNK2	tyrosine kinase non receptor 2 [Source:HGNC Symbol;Acc:HGNC:19297]
REEP1	receptor accessory protein 1 [Source:HGNC Symbol;Acc:HGNC:25786]
PTGS1	prostaglandin-endoperoxide synthase 1 [Source:HGNC Symbol;Acc:HGNC:9604]
C1QTNF6	C1q and tumor necrosis factor related protein 6 [Source:HGNC Symbol;Acc:HGNC:14343]
PAG1	phosphoprotein membrane anchor with glycosphingolipid microdomains 1 [Source:HGNC Symbol;Acc:HGNC:30043]
NDUFB5	NADH:ubiquinone oxidoreductase subunit B5 [Source:HGNC Symbol;Acc:HGNC:7700]
FXN	frataxin [Source:HGNC Symbol;Acc:HGNC:3951]
NDUFV1	NADH:ubiquinone oxidoreductase core subunit V1 [Source:HGNC Symbol;Acc:HGNC:7716]
OAS1	2'-5'-oligoadenylate synthetase 1 [Source:HGNC Symbol;Acc:HGNC:8086]
COLEC12	collectin subfamily member 12 [Source:HGNC Symbol;Acc:HGNC:16016]
PDZD2	PDZ domain containing 2 [Source:HGNC Symbol;Acc:HGNC:18486]
TMEM47	transmembrane protein 47 [Source:HGNC Symbol;Acc:HGNC:18515]
GALNT7	polypeptide N-acetylgalactosaminyltransferase 7 [Source:HGNC Symbol;Acc:HGNC:4129]
FAM20C	family with sequence similarity 20 member C [Source:HGNC Symbol;Acc:HGNC:22140]
PHTF2	putative homeodomain transcription factor 2 [Source:HGNC Symbol;Acc:HGNC:13411]
CCNT2	cyclin T2 [Source:HGNC Symbol;Acc:HGNC:1600]
EIF2B4	eukaryotic translation initiation factor 2B subunit delta [Source:HGNC Symbol;Acc:HGNC:3260]
ABCC1	ATP binding cassette subfamily C member 1 [Source:HGNC Symbol;Acc:HGNC:51]
MASTL	microtubule associated serine/threonine kinase like [Source:HGNC Symbol;Acc:HGNC:19042]
WNT5B	Wnt family member 5B [Source:HGNC Symbol;Acc:HGNC:16265]
SKA1	spindle and kinetochore associated complex subunit 1 [Source:HGNC Symbol;Acc:HGNC:28109]

COX7A1	cytochrome c oxidase subunit 7A1 [Source:HGNC Symbol;Acc:HGNC:2287]
PLPP2	phospholipid phosphatase 2 [Source:HGNC Symbol;Acc:HGNC:9230]
ELF1	E74 like ETS transcription factor 1 [Source:HGNC Symbol;Acc:HGNC:3316]
AP2B1	adaptor related protein complex 2 beta 1 subunit [Source:HGNC Symbol;Acc:HGNC:563]
DAXX	death-domain associated protein [Source:HGNC Symbol;Acc:HGNC:2681]
FBXO3	F-box protein 3 [Source:HGNC Symbol;Acc:HGNC:13582]
KDM6A	lysine demethylase 6A [Source:HGNC Symbol;Acc:HGNC:12637]
TCF7L1	transcription factor 7 like 1 [Source:HGNC Symbol;Acc:HGNC:11640]
BIN1	bridging integrator 1 [Source:HGNC Symbol;Acc:HGNC:1052]
DNER	delta/notch like EGF repeat containing [Source:HGNC Symbol;Acc:HGNC:24456]
RTP4	receptor transporter protein 4 [Source:HGNC Symbol;Acc:HGNC:23992]
ANTXR1	anthrax toxin receptor 1 [Source:HGNC Symbol;Acc:HGNC:21014]
GAS2L3	growth arrest specific 2 like 3 [Source:HGNC Symbol;Acc:HGNC:27475]
NDUFB3	NADH:ubiquinone oxidoreductase subunit B3 [Source:HGNC Symbol;Acc:HGNC:7698]
PCP4	Purkinje cell protein 4 [Source:HGNC Symbol;Acc:HGNC:8742]
GRHL1	grainyhead like transcription factor 1 [Source:HGNC Symbol;Acc:HGNC:17923]
ACP1	acid phosphatase 1, soluble [Source:HGNC Symbol;Acc:HGNC:122]
BLVRA	biliverdin reductase A [Source:HGNC Symbol;Acc:HGNC:1062]
COX7A2	cytochrome c oxidase subunit 7A2 [Source:HGNC Symbol;Acc:HGNC:2288]
DHRS4	dehydrogenase/reductase 4 [Source:HGNC Symbol;Acc:HGNC:16985]
RNF4	ring finger protein 4 [Source:HGNC Symbol;Acc:HGNC:10067]
RPLP2	ribosomal protein lateral stalk subunit P2 [Source:HGNC Symbol;Acc:HGNC:10377]
GALNT3	polypeptide N-acetylgalactosaminyltransferase 3 [Source:HGNC Symbol;Acc:HGNC:4125]
SCT	secretin [Source:HGNC Symbol;Acc:HGNC:10607]
DSG2	desmoglein 2 [Source:HGNC Symbol;Acc:HGNC:3049]
ELOVL7	ELOVL fatty acid elongase 7 [Source:HGNC Symbol;Acc:HGNC:26292]
HILPDA	hypoxia inducible lipid droplet associated [Source:HGNC Symbol;Acc:HGNC:28859]
RASA3	RAS p21 protein activator 3 [Source:HGNC Symbol;Acc:HGNC:20331]
CFL2	cofilin 2 [Source:HGNC Symbol;Acc:HGNC:1875]
COBL	cordon-bleu WH2 repeat protein [Source:HGNC Symbol;Acc:HGNC:22199]
CTHRC1	collagen triple helix repeat containing 1 [Source:HGNC Symbol;Acc:HGNC:18831]
IRF3	interferon regulatory factor 3 [Source:HGNC Symbol;Acc:HGNC:6118]
MYH14	myosin, heavy chain 14, non-muscle [Source:HGNC Symbol;Acc:HGNC:23212]
SLC16A12	solute carrier family 16 member 12 [Source:HGNC Symbol;Acc:HGNC:23094]
TNFRSF10D	tumor necrosis factor receptor superfamily member 10d [Source:HGNC Symbol;Acc:HGNC:11907]
SLC10A2	solute carrier family 10 member 2 [Source:HGNC Symbol;Acc:HGNC:10906]
CCDC88A	coiled-coil domain containing 88A [Source:HGNC Symbol;Acc:HGNC:25523]
FOXC2	forkhead box C2 [Source:HGNC Symbol;Acc:HGNC:3801]
ITGB3BP	integrin subunit beta 3 binding protein [Source:HGNC Symbol;Acc:HGNC:6157]
PCCB	propionyl-CoA carboxylase beta subunit [Source:HGNC Symbol;Acc:HGNC:8654]
PPID	peptidylprolyl isomerase D [Source:HGNC Symbol;Acc:HGNC:9257]

SLC26A2	solute carrier family 26 member 2 [Source:HGNC Symbol;Acc:HGNC:10994]
THPO	thrombopoietin [Source:HGNC Symbol;Acc:HGNC:11795]
TMTC2	transmembrane and tetratricopeptide repeat containing 2 [Source:HGNC Symbol;Acc:HGNC:25440]
KCNK5	potassium two pore domain channel subfamily K member 5 [Source:HGNC Symbol;Acc:HGNC:6280]
MLLT11	myeloid/lymphoid or mixed-lineage leukemia; translocated to, 11 [Source:HGNC Symbol;Acc:HGNC:16997]
NR5A2	nuclear receptor subfamily 5 group A member 2 [Source:HGNC Symbol;Acc:HGNC:7984]
CTF1	cardiotrophin 1 [Source:HGNC Symbol;Acc:HGNC:2499]
KIF14	kinesin family member 14 [Source:HGNC Symbol;Acc:HGNC:19181]
PCSK1	proprotein convertase subtilisin/kexin type 1 [Source:HGNC Symbol;Acc:HGNC:8743]
SMTN	smoothelin [Source:HGNC Symbol;Acc:HGNC:11126]
TINF2	TERF1 interacting nuclear factor 2 [Source:HGNC Symbol;Acc:HGNC:11824]
SESN3	sestrin 3 [Source:HGNC Symbol;Acc:HGNC:23060]
ACKR3	atypical chemokine receptor 3 [Source:HGNC Symbol;Acc:HGNC:23692]
BTF3	basic transcription factor 3 [Source:HGNC Symbol;Acc:HGNC:1125]
CLCA1	chloride channel accessory 1 [Source:HGNC Symbol;Acc:HGNC:2015]
DGKA	diacylglycerol kinase alpha [Source:HGNC Symbol;Acc:HGNC:2849]
KDM5B	lysine demethylase 5B [Source:HGNC Symbol;Acc:HGNC:18039]
NMU	neuromedin U [Source:HGNC Symbol;Acc:HGNC:7859]
TRA2A	transformer 2 alpha homolog [Source:HGNC Symbol;Acc:HGNC:16645]
BACE2	beta-site APP-cleaving enzyme 2 [Source:HGNC Symbol;Acc:HGNC:934]
EPB41L5	erythrocyte membrane protein band 4.1 like 5 [Source:HGNC Symbol;Acc:HGNC:19819]
CEL	carboxyl ester lipase [Source:HGNC Symbol;Acc:HGNC:1848]
DSTN	destrin, actin depolymerizing factor [Source:HGNC Symbol;Acc:HGNC:15750]
NDUFA1	NADH:ubiquinone oxidoreductase subunit A1 [Source:HGNC Symbol;Acc:HGNC:7683]
SRSF11	serine and arginine rich splicing factor 11 [Source:HGNC Symbol;Acc:HGNC:10782]
FBLIM1	filamin binding LIM protein 1 [Source:HGNC Symbol;Acc:HGNC:24686]
DUSP3	dual specificity phosphatase 3 [Source:HGNC Symbol;Acc:HGNC:3069]
CAPNS1	calpain small subunit 1 [Source:HGNC Symbol;Acc:HGNC:1481]
CENPM	centromere protein M [Source:HGNC Symbol;Acc:HGNC:18352]
MT-CO3	mitochondrially encoded cytochrome c oxidase III [Source:HGNC Symbol;Acc:HGNC:7422]
ECI2	enoyl-CoA delta isomerase 2 [Source:HGNC Symbol;Acc:HGNC:14601]
IDS	iduronate 2-sulfatase [Source:HGNC Symbol;Acc:HGNC:5389]
MTA1	metastasis associated 1 [Source:HGNC Symbol;Acc:HGNC:7410]
POLD3	polymerase (DNA) delta 3, accessory subunit [Source:HGNC Symbol;Acc:HGNC:20932]
TOPBP1	topoisomerase (DNA) II binding protein 1 [Source:HGNC Symbol;Acc:HGNC:17008]
AZGP1	alpha-2-glycoprotein 1, zinc-binding [Source:HGNC Symbol;Acc:HGNC:910]
OAS2	2'-5'-oligoadenylate synthetase 2 [Source:HGNC Symbol;Acc:HGNC:8087]
PRF1	perforin 1 [Source:HGNC Symbol;Acc:HGNC:9360]
EXOSC2	exosome component 2 [Source:HGNC Symbol;Acc:HGNC:17097]

NDE1	nudE neurodevelopment protein 1 [Source:HGNC Symbol;Acc:HGNC:17619]
RBM25	RNA binding motif protein 25 [Source:HGNC Symbol;Acc:HGNC:23244]
RNF144B	ring finger protein 144B [Source:HGNC Symbol;Acc:HGNC:21578]
G2E3	G2/M-phase specific E3 ubiquitin protein ligase [Source:HGNC Symbol;Acc:HGNC:20338]
NEBL	nebulette [Source:HGNC Symbol;Acc:HGNC:16932]
MAPK9	mitogen-activated protein kinase 9 [Source:HGNC Symbol;Acc:HGNC:6886]
RAB32	RAB32, member RAS oncogene family [Source:HGNC Symbol;Acc:HGNC:9772]
RPL32	ribosomal protein L32 [Source:HGNC Symbol;Acc:HGNC:10336]
DNAJA4	DnaJ heat shock protein family (Hsp40) member A4 [Source:HGNC Symbol;Acc:HGNC:14885]
HIBADH	3-hydroxyisobutyrate dehydrogenase [Source:HGNC Symbol;Acc:HGNC:4907]
YPEL2	yippee like 2 [Source:HGNC Symbol;Acc:HGNC:18326]
PSMD14	proteasome 26S subunit, non-ATPase 14 [Source:HGNC Symbol;Acc:HGNC:16889]
SAA2	serum amyloid A2 [Source:HGNC Symbol;Acc:HGNC:10514]
SNAP23	synaptosome associated protein 23 [Source:HGNC Symbol;Acc:HGNC:11131]
MAP3K7	mitogen-activated protein kinase kinase kinase 7 [Source:HGNC Symbol;Acc:HGNC:6859]
LGR5	leucine rich repeat containing G protein-coupled receptor 5 [Source:HGNC Symbol;Acc:HGNC:4504]
NFE2L3	nuclear factor, erythroid 2 like 3 [Source:HGNC Symbol;Acc:HGNC:7783]
SYTL2	synaptotagmin like 2 [Source:HGNC Symbol;Acc:HGNC:15585]
POLB	polymerase (DNA) beta [Source:HGNC Symbol;Acc:HGNC:9174]
ATF3	activating transcription factor 3 [Source:HGNC Symbol;Acc:HGNC:785]
TAP2	transporter 2, ATP-binding cassette, sub-family B (MDR/TAP) [Source:HGNC Symbol;Acc:HGNC:44]
LRIG1	leucine rich repeats and immunoglobulin like domains 1 [Source:HGNC Symbol;Acc:HGNC:17360]
MPST	mercaptopyruvate sulfurtransferase [Source:HGNC Symbol;Acc:HGNC:7223]
PMVK	phosphomevalonate kinase [Source:HGNC Symbol;Acc:HGNC:9141]
SKAP2	src kinase associated phosphoprotein 2 [Source:HGNC Symbol;Acc:HGNC:15687]
ADAM9	ADAM metallopeptidase domain 9 [Source:HGNC Symbol;Acc:HGNC:216]
ALPI	alkaline phosphatase, intestinal [Source:HGNC Symbol;Acc:HGNC:437]
CIT	citron rho-interacting serine/threonine kinase [Source:HGNC Symbol;Acc:HGNC:1985]
GSPT1	G1 to S phase transition 1 [Source:HGNC Symbol;Acc:HGNC:4621]
CSTA	cystatin A [Source:HGNC Symbol;Acc:HGNC:2481]
LAPTM5	lysosomal protein transmembrane 5 [Source:HGNC Symbol;Acc:HGNC:29612]
UGP2	UDP-glucose pyrophosphorylase 2 [Source:HGNC Symbol;Acc:HGNC:12527]
ARHGEF2	Rho/Rac guanine nucleotide exchange factor 2 [Source:HGNC Symbol;Acc:HGNC:682]
AUH	AU RNA binding protein/enoyl-CoA hydratase [Source:HGNC Symbol;Acc:HGNC:890]
CLEC7A	C-type lectin domain family 7 member A [Source:HGNC Symbol;Acc:HGNC:14558]
PLEKHF1	pleckstrin homology and FYVE domain containing 1 [Source:HGNC Symbol;Acc:HGNC:20764]
PNN	pinin, desmosome associated protein [Source:HGNC Symbol;Acc:HGNC:9162]
RHBDF1	rhomboid 5 homolog 1 [Source:HGNC Symbol;Acc:HGNC:20561]
S1PR3	sphingosine-1-phosphate receptor 3 [Source:HGNC Symbol;Acc:HGNC:3167]

TSPAN8	tetraspanin 8 [Source:HGNC Symbol;Acc:HGNC:11855]
ATP2A3	ATPase sarcoplasmic/endoplasmic reticulum Ca2+ transporting 3 [Source:HGNC Symbol;Acc:HGNC:813]
GCAT	glycine C-acetyltransferase [Source:HGNC Symbol;Acc:HGNC:4188]
HBA1	hemoglobin subunit alpha 1 [Source:HGNC Symbol;Acc:HGNC:4823]
KMO	kynurenine 3-monooxygenase [Source:HGNC Symbol;Acc:HGNC:6381]
NUDT1	nudix hydrolase 1 [Source:HGNC Symbol;Acc:HGNC:8048]
NUP210	nucleoporin 210 [Source:HGNC Symbol;Acc:HGNC:30052]
PAK2	p21 (RAC1) activated kinase 2 [Source:HGNC Symbol;Acc:HGNC:8591]
SH3BGRL	SH3 domain binding glutamate rich protein like [Source:HGNC Symbol;Acc:HGNC:10823]
SREBF1	sterol regulatory element binding transcription factor 1 [Source:HGNC Symbol;Acc:HGNC:11289]
PRICKLE1	prickle planar cell polarity protein 1 [Source:HGNC Symbol;Acc:HGNC:17019]
CYP19A1	cytochrome P450 family 19 subfamily A member 1 [Source:HGNC Symbol;Acc:HGNC:2594]
AK2	adenylate kinase 2 [Source:HGNC Symbol;Acc:HGNC:362]
ATP1B3	ATPase Na+/K+ transporting subunit beta 3 [Source:HGNC Symbol;Acc:HGNC:806]
PAX3	paired box 3 [Source:HGNC Symbol;Acc:HGNC:8617]
PBX1	PBX homeobox 1 [Source:HGNC Symbol;Acc:HGNC:8632]
RAB30	RAB30, member RAS oncogene family [Source:HGNC Symbol;Acc:HGNC:9770]
TNPO1	transportin 1 [Source:HGNC Symbol;Acc:HGNC:6401]
UBB	ubiquitin B [Source:HGNC Symbol;Acc:HGNC:12463]
OXTR	oxytocin receptor [Source:HGNC Symbol;Acc:HGNC:8529]
SRD5A2	steroid 5 alpha-reductase 2 [Source:HGNC Symbol;Acc:HGNC:11285]
SUCLG1	succinate-CoA ligase alpha subunit [Source:HGNC Symbol;Acc:HGNC:11449]
TNFAIP2	TNF alpha induced protein 2 [Source:HGNC Symbol;Acc:HGNC:11895]
TXN2	thioredoxin 2 [Source:HGNC Symbol;Acc:HGNC:17772]
ADGRG1	adhesion G protein-coupled receptor G1 [Source:HGNC Symbol;Acc:HGNC:4512]
DLC1	DLC1 Rho GTPase activating protein [Source:HGNC Symbol;Acc:HGNC:2897]
ARF1	ADP ribosylation factor 1 [Source:HGNC Symbol;Acc:HGNC:652]
EFHD1	EF-hand domain family member D1 [Source:HGNC Symbol;Acc:HGNC:29556]
ELAVL1	ELAV like RNA binding protein 1 [Source:HGNC Symbol;Acc:HGNC:3312]
RIOK3	RIO kinase 3 [Source:HGNC Symbol;Acc:HGNC:11451]
CTNNAL1	catenin alpha like 1 [Source:HGNC Symbol;Acc:HGNC:2512]
FAM214A	family with sequence similarity 214 member A [Source:HGNC Symbol;Acc:HGNC:25609]
RBBP8	RB binding protein 8, endonuclease [Source:HGNC Symbol;Acc:HGNC:9891]
SPDL1	spindle apparatus coiled-coil protein 1 [Source:HGNC Symbol;Acc:HGNC:26010]
CD40LG	CD40 ligand [Source:HGNC Symbol;Acc:HGNC:11935]
F13A1	coagulation factor XIII A chain [Source:HGNC Symbol;Acc:HGNC:3531]
HDAC3	histone deacetylase 3 [Source:HGNC Symbol;Acc:HGNC:4854]
JADE1	jade family PHD finger 1 [Source:HGNC Symbol;Acc:HGNC:30027]
ST8SIA4	ST8 alpha-N-acetyl-neuraminide alpha-2,8-sialyltransferase 4 [Source:HGNC Symbol;Acc:HGNC:10871]

LAMA1	laminin subunit alpha 1 [Source:HGNC Symbol;Acc:HGNC:6481]
FIGNL1	fidgetin like 1 [Source:HGNC Symbol;Acc:HGNC:13286]
PCSK6	proprotein convertase subtilisin/kexin type 6 [Source:HGNC Symbol;Acc:HGNC:8569]
ANTXR2	anthrax toxin receptor 2 [Source:HGNC Symbol;Acc:HGNC:21732]
CA12	carbonic anhydrase 12 [Source:HGNC Symbol;Acc:HGNC:1371]
GSK3A	glycogen synthase kinase 3 alpha [Source:HGNC Symbol;Acc:HGNC:4616]
KLF13	Kruppel like factor 13 [Source:HGNC Symbol;Acc:HGNC:13672]
MYL1	myosin light chain 1 [Source:HGNC Symbol;Acc:HGNC:7582]
OIP5	Opa interacting protein 5 [Source:HGNC Symbol;Acc:HGNC:20300]
TCF7	transcription factor 7 (T-cell specific, HMG-box) [Source:HGNC Symbol;Acc:HGNC:11639]
SDC1	syndecan 1 [Source:HGNC Symbol;Acc:HGNC:10658]
FGF7	fibroblast growth factor 7 [Source:HGNC Symbol;Acc:HGNC:3685]
BAG2	BCL2 associated athanogene 2 [Source:HGNC Symbol;Acc:HGNC:938]
F8	coagulation factor VIII [Source:HGNC Symbol;Acc:HGNC:3546]
KRT15	keratin 15 [Source:HGNC Symbol;Acc:HGNC:6421]
MAP4	microtubule associated protein 4 [Source:HGNC Symbol;Acc:HGNC:6862]
MARCKSL1	MARCKS like 1 [Source:HGNC Symbol;Acc:HGNC:7142]
SLC16A7	solute carrier family 16 member 7 [Source:HGNC Symbol;Acc:HGNC:10928]
SNX10	sorting nexin 10 [Source:HGNC Symbol;Acc:HGNC:14974]
TPM2	tropomyosin 2 (beta) [Source:HGNC Symbol;Acc:HGNC:12011]
UTRN	utrophin [Source:HGNC Symbol;Acc:HGNC:12635]
CLDN11	claudin 11 [Source:HGNC Symbol;Acc:HGNC:8514]
CHAF1A	chromatin assembly factor 1 subunit A [Source:HGNC Symbol;Acc:HGNC:1910]
NCAPG2	non-SMC condensin II complex subunit G2 [Source:HGNC Symbol;Acc:HGNC:21904]
PPBP	pro-platelet basic protein [Source:HGNC Symbol;Acc:HGNC:9240]
UBE2D3	ubiquitin conjugating enzyme E2 D3 [Source:HGNC Symbol;Acc:HGNC:12476]
CREG1	cellular repressor of E1A stimulated genes 1 [Source:HGNC Symbol;Acc:HGNC:2351]
HOXA1	homeobox A1 [Source:HGNC Symbol;Acc:HGNC:5099]
WFDC2	WAP four-disulfide core domain 2 [Source:HGNC Symbol;Acc:HGNC:15939]
XPO1	exportin 1 [Source:HGNC Symbol;Acc:HGNC:12825]
ACTR3	ARP3 actin-related protein 3 homolog (yeast) [Source:HGNC Symbol;Acc:HGNC:170]
DTYMK	deoxythymidylate kinase [Source:HGNC Symbol;Acc:HGNC:3061]
GPC1	glypican 1 [Source:HGNC Symbol;Acc:HGNC:4449]
LBR	lamin B receptor [Source:HGNC Symbol;Acc:HGNC:6518]
PTPRG	protein tyrosine phosphatase, receptor type G [Source:HGNC Symbol;Acc:HGNC:9671]
RBM39	RNA binding motif protein 39 [Source:HGNC Symbol;Acc:HGNC:15923]
RPA2	replication protein A2 [Source:HGNC Symbol;Acc:HGNC:10290]
CD274	CD274 molecule [Source:HGNC Symbol;Acc:HGNC:17635]
CLDN2	claudin 2 [Source:HGNC Symbol;Acc:HGNC:2041]
ACADSB	acyl-CoA dehydrogenase, short/branched chain [Source:HGNC Symbol;Acc:HGNC:91]
CHGA	chromogranin A [Source:HGNC Symbol;Acc:HGNC:1929]

CLCF1	cardiotrophin-like cytokine factor 1 [Source:HGNC Symbol;Acc:HGNC:17412]
G3BP1	G3BP stress granule assembly factor 1 [Source:HGNC Symbol;Acc:HGNC:30292]
MARCKS	myristoylated alanine rich protein kinase C substrate [Source:HGNC Symbol;Acc:HGNC:6759]
PKP2	plakophilin 2 [Source:HGNC Symbol;Acc:HGNC:9024]
TAX1BP3	Tax1 binding protein 3 [Source:HGNC Symbol;Acc:HGNC:30684]
ALDH4A1	aldehyde dehydrogenase 4 family member A1 [Source:HGNC Symbol;Acc:HGNC:406]
PSMB7	proteasome subunit beta 7 [Source:HGNC Symbol;Acc:HGNC:9544]
SP7	Sp7 transcription factor [Source:HGNC Symbol;Acc:HGNC:17321]
SERPINA3	serpin family A member 3 [Source:HGNC Symbol;Acc:HGNC:16]
BCL2L2	BCL2 like 2 [Source:HGNC Symbol;Acc:HGNC:995]
COL14A1	collagen type XIV alpha 1 chain [Source:HGNC Symbol;Acc:HGNC:2191]
DHRS3	dehydrogenase/reductase 3 [Source:HGNC Symbol;Acc:HGNC:17693]
EEF2K	eukaryotic elongation factor 2 kinase [Source:HGNC Symbol;Acc:HGNC:24615]
SLC25A5	solute carrier family 25 member 5 [Source:HGNC Symbol;Acc:HGNC:10991]
PLSCR1	phospholipid scramblase 1 [Source:HGNC Symbol;Acc:HGNC:9092]
SLC39A14	solute carrier family 39 member 14 [Source:HGNC Symbol;Acc:HGNC:20858]
CLGN	calmegin [Source:HGNC Symbol;Acc:HGNC:2060]
CRELD2	cysteine rich with EGF like domains 2 [Source:HGNC Symbol;Acc:HGNC:28150]
DAG1	dystroglycan 1 [Source:HGNC Symbol;Acc:HGNC:2666]
DBN1	drebrin 1 [Source:HGNC Symbol;Acc:HGNC:2695]
PIK3C3	phosphatidylinositol 3-kinase catalytic subunit type 3 [Source:HGNC Symbol;Acc:HGNC:8974]
STARD4	StAR related lipid transfer domain containing 4 [Source:HGNC Symbol;Acc:HGNC:18058]
SUCLG2	succinate-CoA ligase GDP-forming beta subunit [Source:HGNC Symbol;Acc:HGNC:11450]
SULF1	sulfatase 1 [Source:HGNC Symbol;Acc:HGNC:20391]
TMSB4X	thymosin beta 4, X-linked [Source:HGNC Symbol;Acc:HGNC:11881]
ELF3	E74 like ETS transcription factor 3 [Source:HGNC Symbol;Acc:HGNC:3318]
FASN	fatty acid synthase [Source:HGNC Symbol;Acc:HGNC:3594]
EIF4B	eukaryotic translation initiation factor 4B [Source:HGNC Symbol;Acc:HGNC:3285]
IVD	isovaleryl-CoA dehydrogenase [Source:HGNC Symbol;Acc:HGNC:6186]
P4HA1	prolyl 4-hydroxylase subunit alpha 1 [Source:HGNC Symbol;Acc:HGNC:8546]
PLN	phospholamban [Source:HGNC Symbol;Acc:HGNC:9080]
SERPINA6	serpin family A member 6 [Source:HGNC Symbol;Acc:HGNC:1540]
ANP32E	acidic nuclear phosphoprotein 32 family member E [Source:HGNC Symbol;Acc:HGNC:16673]
JDP2	Jun dimerization protein 2 [Source:HGNC Symbol;Acc:HGNC:17546]
TPT1	tumor protein, translationally-controlled 1 [Source:HGNC Symbol;Acc:HGNC:12022]
DSP	desmoplakin [Source:HGNC Symbol;Acc:HGNC:3052]
LTF	lactotransferrin [Source:HGNC Symbol;Acc:HGNC:6720]
HSPA1L	heat shock protein family A (Hsp70) member 1 like [Source:HGNC Symbol;Acc:HGNC:5234]
PNPLA3	patatin like phospholipase domain containing 3 [Source:HGNC Symbol;Acc:HGNC:18590]
CEP55	centrosomal protein 55 [Source:HGNC Symbol;Acc:HGNC:1161]
GINS1	GINS complex subunit 1 [Source:HGNC Symbol;Acc:HGNC:28980]

METTL7A	methyltransferase like 7A [Source:HGNC Symbol;Acc:HGNC:24550]
PRKAR1A	protein kinase cAMP-dependent type I regulatory subunit alpha [Source:HGNC Symbol;Acc:HGNC:9388]
SLC37A4	solute carrier family 37 member 4 [Source:HGNC Symbol;Acc:HGNC:4061]
SDPR	serum deprivation response [Source:HGNC Symbol;Acc:HGNC:10690]
EEF1A2	eukaryotic translation elongation factor 1 alpha 2 [Source:HGNC Symbol;Acc:HGNC:3192]
FANCI	Fanconi anemia complementation group I [Source:HGNC Symbol;Acc:HGNC:25568]
MT1H	metallothionein 1H [Source:HGNC Symbol;Acc:HGNC:7400]
NPY1R	neuropeptide Y receptor Y1 [Source:HGNC Symbol;Acc:HGNC:7956]
PDHA1	pyruvate dehydrogenase (lipoamide) alpha 1 [Source:HGNC Symbol;Acc:HGNC:8806]
ARRDC3	arrestin domain containing 3 [Source:HGNC Symbol;Acc:HGNC:29263]
HCK	HCK proto-oncogene, Src family tyrosine kinase [Source:HGNC Symbol;Acc:HGNC:4840]
SDHB	succinate dehydrogenase complex iron sulfur subunit B [Source:HGNC Symbol;Acc:HGNC:10681]
SLC2A5	solute carrier family 2 member 5 [Source:HGNC Symbol;Acc:HGNC:11010]
MVP	major vault protein [Source:HGNC Symbol;Acc:HGNC:7531]
RYR1	ryanodine receptor 1 [Source:HGNC Symbol;Acc:HGNC:10483]
COX5A	cytochrome c oxidase subunit 5A [Source:HGNC Symbol;Acc:HGNC:2267]
ENDOG	endonuclease G [Source:HGNC Symbol;Acc:HGNC:3346]
TMSB10	thymosin beta 10 [Source:HGNC Symbol;Acc:HGNC:11879]
RAB31	RAB31, member RAS oncogene family [Source:HGNC Symbol;Acc:HGNC:9771]
ABLIM1	actin binding LIM protein 1 [Source:HGNC Symbol;Acc:HGNC:78]
BBOX1	gamma-butyrobetaine hydroxylase 1 [Source:HGNC Symbol;Acc:HGNC:964]
CDCA5	cell division cycle associated 5 [Source:HGNC Symbol;Acc:HGNC:14626]
IFIT3	interferon induced protein with tetratricopeptide repeats 3 [Source:HGNC Symbol;Acc:HGNC:5411]
MSR1	macrophage scavenger receptor 1 [Source:HGNC Symbol;Acc:HGNC:7376]
OLFML3	olfactomedin like 3 [Source:HGNC Symbol;Acc:HGNC:24956]
PAK1	p21 (RAC1) activated kinase 1 [Source:HGNC Symbol;Acc:HGNC:8590]
PSRC1	proline and serine rich coiled-coil 1 [Source:HGNC Symbol;Acc:HGNC:24472]
RRBP1	ribosome binding protein 1 [Source:HGNC Symbol;Acc:HGNC:10448]
ETFA	electron transfer flavoprotein alpha subunit [Source:HGNC Symbol;Acc:HGNC:3481]
MAPK13	mitogen-activated protein kinase 13 [Source:HGNC Symbol;Acc:HGNC:6875]
KRT7	keratin 7 [Source:HGNC Symbol;Acc:HGNC:6445]
CD38	CD38 molecule [Source:HGNC Symbol;Acc:HGNC:1667]
DSC2	desmocollin 2 [Source:HGNC Symbol;Acc:HGNC:3036]
GPD2	glycerol-3-phosphate dehydrogenase 2 [Source:HGNC Symbol;Acc:HGNC:4456]
HDAC2	histone deacetylase 2 [Source:HGNC Symbol;Acc:HGNC:4853]
KNSTRN	kinetochore-localized astrin/SPAG5 binding protein [Source:HGNC Symbol;Acc:HGNC:30767]
PPIF	peptidylprolyl isomerase F [Source:HGNC Symbol;Acc:HGNC:9259]
CAMK4	calcium/calmodulin dependent protein kinase IV [Source:HGNC Symbol;Acc:HGNC:1464]
FZD2	frizzled class receptor 2 [Source:HGNC Symbol;Acc:HGNC:4040]

IFI30	IFI30, lysosomal thiol reductase [Source:HGNC Symbol;Acc:HGNC:5398]
RETN	resistin [Source:HGNC Symbol;Acc:HGNC:20389]
SLC22A4	solute carrier family 22 member 4 [Source:HGNC Symbol;Acc:HGNC:10968]
E2F7	E2F transcription factor 7 [Source:HGNC Symbol;Acc:HGNC:23820]
BCL2A1	BCL2 related protein A1 [Source:HGNC Symbol;Acc:HGNC:991]
ALDH1A3	aldehyde dehydrogenase 1 family member A3 [Source:HGNC Symbol;Acc:HGNC:409]
CD24	CD24 molecule [Source:HGNC Symbol;Acc:HGNC:1645]
IL24	interleukin 24 [Source:HGNC Symbol;Acc:HGNC:11346]
NPC2	NPC intracellular cholesterol transporter 2 [Source:HGNC Symbol;Acc:HGNC:14537]
PXN	paxillin [Source:HGNC Symbol;Acc:HGNC:9718]
LTBP1	latent transforming growth factor beta binding protein 1 [Source:HGNC Symbol;Acc:HGNC:6714]
EIF2A	eukaryotic translation initiation factor 2A [Source:HGNC Symbol;Acc:HGNC:3254]
KIF15	kinesin family member 15 [Source:HGNC Symbol;Acc:HGNC:17273]
BACH1	BTB domain and CNC homolog 1 [Source:HGNC Symbol;Acc:HGNC:935]
DST	dystonin [Source:HGNC Symbol;Acc:HGNC:1090]
RASSF1	Ras association domain family member 1 [Source:HGNC Symbol;Acc:HGNC:9882]
CACYBP	calcyclin binding protein [Source:HGNC Symbol;Acc:HGNC:30423]
TNFRSF25	tumor necrosis factor receptor superfamily member 25 [Source:HGNC Symbol;Acc:HGNC:11910]
BOK	BCL2-related ovarian killer [Source:HGNC Symbol;Acc:HGNC:1087]
CKS1B	CDC28 protein kinase regulatory subunit 1B [Source:HGNC Symbol;Acc:HGNC:19083]
CYGB	cytoglobin [Source:HGNC Symbol;Acc:HGNC:16505]
MCAM	melanoma cell adhesion molecule [Source:HGNC Symbol;Acc:HGNC:6934]
TG	thyroglobulin [Source:HGNC Symbol;Acc:HGNC:11764]
PTH	parathyroid hormone [Source:HGNC Symbol;Acc:HGNC:9606]
GREB1	growth regulation by estrogen in breast cancer 1 [Source:HGNC Symbol;Acc:HGNC:24885]
ITGA3	integrin subunit alpha 3 [Source:HGNC Symbol;Acc:HGNC:6139]
KL	klotho [Source:HGNC Symbol;Acc:HGNC:6344]
LMCD1	LIM and cysteine rich domains 1 [Source:HGNC Symbol;Acc:HGNC:6633]
MYH10	myosin, heavy chain 10, non-muscle [Source:HGNC Symbol;Acc:HGNC:7568]
NCAPG	non-SMC condensin I complex subunit G [Source:HGNC Symbol;Acc:HGNC:24304]
CIRBP	cold inducible RNA binding protein [Source:HGNC Symbol;Acc:HGNC:1982]
ALDH1L2	aldehyde dehydrogenase 1 family member L2 [Source:HGNC Symbol;Acc:HGNC:26777]
ARHGDIA	Rho GDP dissociation inhibitor alpha [Source:HGNC Symbol;Acc:HGNC:678]
KAT2B	lysine acetyltransferase 2B [Source:HGNC Symbol;Acc:HGNC:8638]
CDCA2	cell division cycle associated 2 [Source:HGNC Symbol;Acc:HGNC:14623]
FEN1	flap structure-specific endonuclease 1 [Source:HGNC Symbol;Acc:HGNC:3650]
GRB2	growth factor receptor bound protein 2 [Source:HGNC Symbol;Acc:HGNC:4566]
IFITM3	interferon induced transmembrane protein 3 [Source:HGNC Symbol;Acc:HGNC:5414]
FAS	Fas cell surface death receptor [Source:HGNC Symbol;Acc:HGNC:11920]
TGM1	transglutaminase 1 [Source:HGNC Symbol;Acc:HGNC:11777]

HYOU1	hypoxia up-regulated 1 [Source:HGNC Symbol;Acc:HGNC:16931]
IRAK2	interleukin 1 receptor associated kinase 2 [Source:HGNC Symbol;Acc:HGNC:6113]
NABP1	nucleic acid binding protein 1 [Source:HGNC Symbol;Acc:HGNC:26232]
PDPK1	3-phosphoinositide dependent protein kinase 1 [Source:HGNC Symbol;Acc:HGNC:8816]
ATP7A	ATPase copper transporting alpha [Source:HGNC Symbol;Acc:HGNC:869]
BIK	BCL2 interacting killer [Source:HGNC Symbol;Acc:HGNC:1051]
DDX17	DEAD-box helicase 17 [Source:HGNC Symbol;Acc:HGNC:2740]
GRB10	growth factor receptor bound protein 10 [Source:HGNC Symbol;Acc:HGNC:4564]
HADHB	hydroxyacyl-CoA dehydrogenase/3-ketoacyl-CoA thiolase/enoyl-CoA hydratase (trifunctional protein), beta subunit [Source:HGNC Symbol;Acc:HGNC:4803]
PAH	phenylalanine hydroxylase [Source:HGNC Symbol;Acc:HGNC:8582]
UGT2B15	UDP glucuronosyltransferase family 2 member B15 [Source:HGNC Symbol;Acc:HGNC:12546]
RGCC	regulator of cell cycle [Source:HGNC Symbol;Acc:HGNC:20369]
CALD1	caldesmon 1 [Source:HGNC Symbol;Acc:HGNC:1441]
PDLIM1	PDZ and LIM domain 1 [Source:HGNC Symbol;Acc:HGNC:2067]
SHMT2	serine hydroxymethyltransferase 2 [Source:HGNC Symbol;Acc:HGNC:10852]
ATP7B	ATPase copper transporting beta [Source:HGNC Symbol;Acc:HGNC:870]
DGAT1	diacylglycerol O-acyltransferase 1 [Source:HGNC Symbol;Acc:HGNC:2843]
AHRR	aryl-hydrocarbon receptor repressor [Source:HGNC Symbol;Acc:HGNC:346]
EIF5A	eukaryotic translation initiation factor 5A [Source:HGNC Symbol;Acc:HGNC:3300]
FBXO5	F-box protein 5 [Source:HGNC Symbol;Acc:HGNC:13584]
PAICS	phosphoribosylaminoimidazole carboxylase; phosphoribosylaminoimidazolesuccinocarboxamide synthase [Source:HGNC Symbol;Acc:HGNC:8587]
PPL	periplakin [Source:HGNC Symbol;Acc:HGNC:9273]
ROCK1	Rho associated coiled-coil containing protein kinase 1 [Source:HGNC Symbol;Acc:HGNC:10251]
MB	myoglobin [Source:HGNC Symbol;Acc:HGNC:6915]
PTCH1	patched 1 [Source:HGNC Symbol;Acc:HGNC:9585]
CROT	carnitine O-octanoyltransferase [Source:HGNC Symbol;Acc:HGNC:2366]
ITGA6	integrin subunit alpha 6 [Source:HGNC Symbol;Acc:HGNC:6142]
ANKRD1	ankyrin repeat domain 1 [Source:HGNC Symbol;Acc:HGNC:15819]
CLK1	CDC like kinase 1 [Source:HGNC Symbol;Acc:HGNC:2068]
FKBP4	FK506 binding protein 4 [Source:HGNC Symbol;Acc:HGNC:3720]
ACO2	aconitase 2 [Source:HGNC Symbol;Acc:HGNC:118]
ITGB4	integrin subunit beta 4 [Source:HGNC Symbol;Acc:HGNC:6158]
CPE	carboxypeptidase E [Source:HGNC Symbol;Acc:HGNC:2303]
GTSE1	G2 and S-phase expressed 1 [Source:HGNC Symbol;Acc:HGNC:13698]
HMGA2	high mobility group AT-hook 2 [Source:HGNC Symbol;Acc:HGNC:5009]
PNP	purine nucleoside phosphorylase [Source:HGNC Symbol;Acc:HGNC:7892]
TCF19	transcription factor 19 [Source:HGNC Symbol;Acc:HGNC:11629]
USP18	ubiquitin specific peptidase 18 [Source:HGNC Symbol;Acc:HGNC:12616]
MT3	metallothionein 3 [Source:HGNC Symbol;Acc:HGNC:7408]

SORL1	sortilin-related receptor, L(DLR class) A repeats containing [Source:HGNC Symbol;Acc:HGNC:11185]
COL6A3	collagen type VI alpha 3 chain [Source:HGNC Symbol;Acc:HGNC:2213]
NRIP1	nuclear receptor interacting protein 1 [Source:HGNC Symbol;Acc:HGNC:8001]
NUCB2	nucleobindin 2 [Source:HGNC Symbol;Acc:HGNC:8044]
JUP	junction plakoglobin [Source:HGNC Symbol;Acc:HGNC:6207]
NUF2	NUF2, NDC80 kinetochore complex component [Source:HGNC Symbol;Acc:HGNC:14621]
PEBP1	phosphatidylethanolamine binding protein 1 [Source:HGNC Symbol;Acc:HGNC:8630]
PMP22	peripheral myelin protein 22 [Source:HGNC Symbol;Acc:HGNC:9118]
SLC25A4	solute carrier family 25 member 4 [Source:HGNC Symbol;Acc:HGNC:10990]
MX1	MX dynamin like GTPase 1 [Source:HGNC Symbol;Acc:HGNC:7532]
ANLN	anillin actin binding protein [Source:HGNC Symbol;Acc:HGNC:14082]
CHAT	choline O-acetyltransferase [Source:HGNC Symbol;Acc:HGNC:1912]
CYP26B1	cytochrome P450 family 26 subfamily B member 1 [Source:HGNC Symbol;Acc:HGNC:20581]
MEF2C	myocyte enhancer factor 2C [Source:HGNC Symbol;Acc:HGNC:6996]
OGDH	oxoglutarate dehydrogenase [Source:HGNC Symbol;Acc:HGNC:8124]
SFRP1	secreted frizzled related protein 1 [Source:HGNC Symbol;Acc:HGNC:10776]
SPC25	SPC25, NDC80 kinetochore complex component [Source:HGNC Symbol;Acc:HGNC:24031]
ACADL	acyl-CoA dehydrogenase, long chain [Source:HGNC Symbol;Acc:HGNC:88]
RASD1	ras related dexamethasone induced 1 [Source:HGNC Symbol;Acc:HGNC:15828]
HELLS	helicase, lymphoid-specific [Source:HGNC Symbol;Acc:HGNC:4861]
HIPK2	homeodomain interacting protein kinase 2 [Source:HGNC Symbol;Acc:HGNC:14402]
TNFRSF9	tumor necrosis factor receptor superfamily member 9 [Source:HGNC Symbol;Acc:HGNC:11924]
BCL3	B-cell CLL/lymphoma 3 [Source:HGNC Symbol;Acc:HGNC:998]
EXO1	exonuclease 1 [Source:HGNC Symbol;Acc:HGNC:3511]
ALDH6A1	aldehyde dehydrogenase 6 family member A1 [Source:HGNC Symbol;Acc:HGNC:7179]
KPNA2	karyopherin subunit alpha 2 [Source:HGNC Symbol;Acc:HGNC:6395]
LHCGR	luteinizing hormone/choriogonadotropin receptor [Source:HGNC Symbol;Acc:HGNC:6585]
RHOC	ras homolog family member C [Source:HGNC Symbol;Acc:HGNC:669]
SERPINC1	serpin family C member 1 [Source:HGNC Symbol;Acc:HGNC:775]
TNFRSF21	tumor necrosis factor receptor superfamily member 21 [Source:HGNC Symbol;Acc:HGNC:13469]
COL12A1	collagen type XII alpha 1 chain [Source:HGNC Symbol;Acc:HGNC:2188]
ECI1	enoyl-CoA delta isomerase 1 [Source:HGNC Symbol;Acc:HGNC:2703]
IL7R	interleukin 7 receptor [Source:HGNC Symbol;Acc:HGNC:6024]
LBP	lipopolysaccharide binding protein [Source:HGNC Symbol;Acc:HGNC:6517]
EMP1	epithelial membrane protein 1 [Source:HGNC Symbol;Acc:HGNC:3333]
GSTA5	glutathione S-transferase alpha 5 [Source:HGNC Symbol;Acc:HGNC:19662]
PTK2B	protein tyrosine kinase 2 beta [Source:HGNC Symbol;Acc:HGNC:9612]
LCAT	lecithin-cholesterol acyltransferase [Source:HGNC Symbol;Acc:HGNC:6522]
MRC1	mannose receptor, C type 1 [Source:HGNC Symbol;Acc:HGNC:7228]
ROCK2	Rho associated coiled-coil containing protein kinase 2 [Source:HGNC Symbol;Acc:HGNC:10252]

SHC1	SHC adaptor protein 1 [Source:HGNC Symbol;Acc:HGNC:10840]
CENPA	centromere protein A [Source:HGNC Symbol;Acc:HGNC:1851]
LIG1	DNA ligase 1 [Source:HGNC Symbol;Acc:HGNC:6598]
LITAF	lipopolysaccharide induced TNF factor [Source:HGNC Symbol;Acc:HGNC:16841]
NEK2	NIMA related kinase 2 [Source:HGNC Symbol;Acc:HGNC:7745]
UBC	ubiquitin C [Source:HGNC Symbol;Acc:HGNC:12468]
UGCG	UDP-glucose ceramide glucosyltransferase [Source:HGNC Symbol;Acc:HGNC:12524]
CDCA8	cell division cycle associated 8 [Source:HGNC Symbol;Acc:HGNC:14629]
CENPE	centromere protein E [Source:HGNC Symbol;Acc:HGNC:1856]
COX4I1	cytochrome c oxidase subunit 4I1 [Source:HGNC Symbol;Acc:HGNC:2265]
HPX	hemopexin [Source:HGNC Symbol;Acc:HGNC:5171]
YWHAZ	tyrosine 3-monooxygenase/tryptophan 5-monooxygenase activation protein zeta [Source:HGNC Symbol;Acc:HGNC:12855]
AHCY	adenosylhomocysteinase [Source:HGNC Symbol;Acc:HGNC:343]
CDCA3	cell division cycle associated 3 [Source:HGNC Symbol;Acc:HGNC:14624]
HSPA4L	heat shock protein family A (Hsp70) member 4 like [Source:HGNC Symbol;Acc:HGNC:17041]
RACGAP1	Rac GTPase activating protein 1 [Source:HGNC Symbol;Acc:HGNC:9804]
VASP	vasodilator-stimulated phosphoprotein [Source:HGNC Symbol;Acc:HGNC:12652]
FTL	ferritin, light polypeptide [Source:HGNC Symbol;Acc:HGNC:3999]
IL1R2	interleukin 1 receptor type 2 [Source:HGNC Symbol;Acc:HGNC:5994]
TFPI	tissue factor pathway inhibitor [Source:HGNC Symbol;Acc:HGNC:11760]
CCNA1	cyclin A1 [Source:HGNC Symbol;Acc:HGNC:1577]
KLF2	Kruppel like factor 2 [Source:HGNC Symbol;Acc:HGNC:6347]
DLGAP5	DLG associated protein 5 [Source:HGNC Symbol;Acc:HGNC:16864]
EBP	emopamil binding protein (sterol isomerase) [Source:HGNC Symbol;Acc:HGNC:3133]
TPX2	TPX2, microtubule nucleation factor [Source:HGNC Symbol;Acc:HGNC:1249]
DUSP10	dual specificity phosphatase 10 [Source:HGNC Symbol;Acc:HGNC:3065]
GPER1	G protein-coupled estrogen receptor 1 [Source:HGNC Symbol;Acc:HGNC:4485]
ITGA2	integrin subunit alpha 2 [Source:HGNC Symbol;Acc:HGNC:6137]
MUC5AC	mucin 5AC, oligomeric mucus/gel-forming [Source:HGNC Symbol;Acc:HGNC:7515]
SPTBN1	spectrin beta, non-erythrocytic 1 [Source:HGNC Symbol;Acc:HGNC:11275]
COL6A1	collagen type VI alpha 1 [Source:HGNC Symbol;Acc:HGNC:2211]
HSD17B7	hydroxysteroid 17-beta dehydrogenase 7 [Source:HGNC Symbol;Acc:HGNC:5215]
ALDOC	aldolase, fructose-bisphosphate C [Source:HGNC Symbol;Acc:HGNC:418]
CCNF	cyclin F [Source:HGNC Symbol;Acc:HGNC:1591]
CXCL3	C-X-C motif chemokine ligand 3 [Source:HGNC Symbol;Acc:HGNC:4604]
HNRNPA1	heterogeneous nuclear ribonucleoprotein A1 [Source:HGNC Symbol;Acc:HGNC:5031]
EZH2	enhancer of zeste 2 polycomb repressive complex 2 subunit [Source:HGNC Symbol;Acc:HGNC:3527]
MSH2	mutS homolog 2 [Source:HGNC Symbol;Acc:HGNC:7325]
PLA2G2A	phospholipase A2 group IIA [Source:HGNC Symbol;Acc:HGNC:9031]
SOX4	SRY-box 4 [Source:HGNC Symbol;Acc:HGNC:11200]

SPTAN1	spectrin alpha, non-erythrocytic 1 [Source:HGNC Symbol;Acc:HGNC:11273]

NDC80	NDC80, kinetochore complex component [Source:HGNC Symbol;Acc:HGNC:16909]
SLC5A5	solute carrier family 5 member 5 [Source:HGNC Symbol;Acc:HGNC:11040]
IL1RL1	interleukin 1 receptor like 1 [Source:HGNC Symbol;Acc:HGNC:5998]
PGD	phosphogluconate dehydrogenase [Source:HGNC Symbol;Acc:HGNC:8891]
CSRP1	cysteine and glycine rich protein 1 [Source:HGNC Symbol;Acc:HGNC:2469]
GPD1	glycerol-3-phosphate dehydrogenase 1 [Source:HGNC Symbol;Acc:HGNC:4455]
IGF2R	insulin like growth factor 2 receptor [Source:HGNC Symbol;Acc:HGNC:5467]
MAD2L1	MAD2 mitotic arrest deficient-like 1 (yeast) [Source:HGNC Symbol;Acc:HGNC:6763]
RGS4	regulator of G-protein signaling 4 [Source:HGNC Symbol;Acc:HGNC:10000]
SULT1E1	sulfotransferase family 1E member 1 [Source:HGNC Symbol;Acc:HGNC:11377]
GSS	glutathione synthetase [Source:HGNC Symbol;Acc:HGNC:4624]
SRD5A1	steroid 5 alpha-reductase 1 [Source:HGNC Symbol;Acc:HGNC:11284]
IGFBP4	insulin like growth factor binding protein 4 [Source:HGNC Symbol;Acc:HGNC:5473]
MAP2K6	mitogen-activated protein kinase kinase 6 [Source:HGNC Symbol;Acc:HGNC:6846]
ZEB1	zinc finger E-box binding homeobox 1 [Source:HGNC Symbol;Acc:HGNC:11642]
DGAT2	diacylglycerol O-acyltransferase 2 [Source:HGNC Symbol;Acc:HGNC:16940]
CLDN1	claudin 1 [Source:HGNC Symbol;Acc:HGNC:2032]
DHCR7	7-dehydrocholesterol reductase [Source:HGNC Symbol;Acc:HGNC:2860]
HEXB	hexosaminidase subunit beta [Source:HGNC Symbol;Acc:HGNC:4879]
ITGA5	integrin subunit alpha 5 [Source:HGNC Symbol;Acc:HGNC:6141]
KIF2C	kinesin family member 2C [Source:HGNC Symbol;Acc:HGNC:6393]
SLC6A6	solute carrier family 6 member 6 [Source:HGNC Symbol;Acc:HGNC:11052]
FBXO32	F-box protein 32 [Source:HGNC Symbol;Acc:HGNC:16731]
KIF23	kinesin family member 23 [Source:HGNC Symbol;Acc:HGNC:6392]
SLC27A2	solute carrier family 27 member 2 [Source:HGNC Symbol;Acc:HGNC:10996]
TCF4	transcription factor 4 [Source:HGNC Symbol;Acc:HGNC:11634]
NFIL3	nuclear factor, interleukin 3 regulated [Source:HGNC Symbol;Acc:HGNC:7787]
CAMK2A	calcium/calmodulin dependent protein kinase II alpha [Source:HGNC Symbol;Acc:HGNC:1460]
NRF1	nuclear respiratory factor 1 [Source:HGNC Symbol;Acc:HGNC:7996]
SERPINH1	serpin family H member 1 [Source:HGNC Symbol;Acc:HGNC:1546]
TKT	transketolase [Source:HGNC Symbol;Acc:HGNC:11834]
TPM4	tropomyosin 4 [Source:HGNC Symbol;Acc:HGNC:12013]
PLD1	phospholipase D1 [Source:HGNC Symbol;Acc:HGNC:9067]
ATP2A2	ATPase sarcoplasmic/endoplasmic reticulum Ca2+ transporting 2 [Source:HGNC Symbol;Acc:HGNC:812]
FABP3	fatty acid binding protein 3 [Source:HGNC Symbol;Acc:HGNC:3557]
LRP1	LDL receptor related protein 1 [Source:HGNC Symbol;Acc:HGNC:6692]
NGFR	nerve growth factor receptor [Source:HGNC Symbol;Acc:HGNC:7809]

RAN	RAN, member RAS oncogene family [Source:HGNC Symbol;Acc:HGNC:9846]
ACP5	acid phosphatase 5, tartrate resistant [Source:HGNC Symbol;Acc:HGNC:124]
DES	desmin [Source:HGNC Symbol;Acc:HGNC:2770]
GSTO1	glutathione S-transferase omega 1 [Source:HGNC Symbol;Acc:HGNC:13312]
PIM1	Pim-1 proto-oncogene, serine/threonine kinase [Source:HGNC Symbol;Acc:HGNC:8986]
PRKACA	protein kinase cAMP-activated catalytic subunit alpha [Source:HGNC Symbol;Acc:HGNC:9380]
UPP1	uridine phosphorylase 1 [Source:HGNC Symbol;Acc:HGNC:12576]
EFNA1	ephrin A1 [Source:HGNC Symbol;Acc:HGNC:3221]
LEF1	lymphoid enhancer binding factor 1 [Source:HGNC Symbol;Acc:HGNC:6551]
SERPINB2	serpin family B member 2 [Source:HGNC Symbol;Acc:HGNC:8584]
SKP2	S-phase kinase-associated protein 2, E3 ubiquitin protein ligase [Source:HGNC Symbol;Acc:HGNC:10901]
MT1X	metallothionein 1X [Source:HGNC Symbol;Acc:HGNC:7405]
ACAA2	acetyl-CoA acyltransferase 2 [Source:HGNC Symbol;Acc:HGNC:83]
BRAF	B-Raf proto-oncogene, serine/threonine kinase [Source:HGNC Symbol;Acc:HGNC:1097]
KIF11	kinesin family member 11 [Source:HGNC Symbol;Acc:HGNC:6388]
MFN2	mitofusin 2 [Source:HGNC Symbol;Acc:HGNC:16877]
STIP1	stress induced phosphoprotein 1 [Source:HGNC Symbol;Acc:HGNC:11387]
TIPARP	TCDD inducible poly(ADP-ribose) polymerase [Source:HGNC Symbol;Acc:HGNC:23696]
PRNP	prion protein [Source:HGNC Symbol;Acc:HGNC:9449]
FHL2	four and a half LIM domains 2 [Source:HGNC Symbol;Acc:HGNC:3703]
HAVCR1	hepatitis A virus cellular receptor 1 [Source:HGNC Symbol;Acc:HGNC:17866]
NEFH	neurofilament, heavy polypeptide [Source:HGNC Symbol;Acc:HGNC:7737]
PTGER4	prostaglandin E receptor 4 [Source:HGNC Symbol;Acc:HGNC:9596]
EPCAM	epithelial cell adhesion molecule [Source:HGNC Symbol;Acc:HGNC:11529]
ASS1	argininosuccinate synthase 1 [Source:HGNC Symbol;Acc:HGNC:758]
CFL1	cofilin 1 [Source:HGNC Symbol;Acc:HGNC:1874]
COL5A1	collagen type V alpha 1 [Source:HGNC Symbol;Acc:HGNC:2209]
HBB	hemoglobin subunit beta [Source:HGNC Symbol;Acc:HGNC:4827]
KIF20A	kinesin family member 20A [Source:HGNC Symbol;Acc:HGNC:9787]
DRD1	dopamine receptor D1 [Source:HGNC Symbol;Acc:HGNC:3020]
GAP43	growth associated protein 43 [Source:HGNC Symbol;Acc:HGNC:4140]
GATA3	GATA binding protein 3 [Source:HGNC Symbol;Acc:HGNC:4172]
HADH	hydroxyacyl-CoA dehydrogenase [Source:HGNC Symbol;Acc:HGNC:4799]
UGT1A7	UDP glucuronosyltransferase family 1 member A7 [Source:HGNC Symbol;Acc:HGNC:12539]
BAG3	BCL2 associated athanogene 3 [Source:HGNC Symbol;Acc:HGNC:939]
CBR1	carbonyl reductase 1 [Source:HGNC Symbol;Acc:HGNC:1548]
NUSAP1	nucleolar and spindle associated protein 1 [Source:HGNC Symbol;Acc:HGNC:18538]

ACTA1	actin, alpha 1, skeletal muscle [Source:HGNC Symbol;Acc:HGNC:129]
BUB1B	BUB1 mitotic checkpoint serine/threonine kinase B [Source:HGNC Symbol;Acc:HGNC:1149]
CDKN2C	cyclin dependent kinase inhibitor 2C [Source:HGNC Symbol;Acc:HGNC:1789]
NFKB2	nuclear factor kappa B subunit 2 [Source:HGNC Symbol;Acc:HGNC:7795]
ERBB3	erb-b2 receptor tyrosine kinase 3 [Source:HGNC Symbol;Acc:HGNC:3431]
ERRFI1	ERBB receptor feedback inhibitor 1 [Source:HGNC Symbol;Acc:HGNC:18185]
PBK	PDZ binding kinase [Source:HGNC Symbol;Acc:HGNC:18282]
DNMT3B	DNA (cytosine-5-)-methyltransferase 3 beta [Source:HGNC Symbol;Acc:HGNC:2979]
EEF1A1	eukaryotic translation elongation factor 1 alpha 1 [Source:HGNC Symbol;Acc:HGNC:3189]
GOT1	glutamic-oxaloacetic transaminase 1 [Source:HGNC Symbol;Acc:HGNC:4432]
CTSK	cathepsin K [Source:HGNC Symbol;Acc:HGNC:2536]
ATF2	activating transcription factor 2 [Source:HGNC Symbol;Acc:HGNC:784]
GHR	growth hormone receptor [Source:HGNC Symbol;Acc:HGNC:4263]
HSPE1	heat shock protein family E (Hsp10) member 1 [Source:HGNC Symbol;Acc:HGNC:5269]
ITGB2	integrin subunit beta 2 [Source:HGNC Symbol;Acc:HGNC:6155]
BUB1	BUB1 mitotic checkpoint serine/threonine kinase [Source:HGNC Symbol;Acc:HGNC:1148]
HSD17B4	hydroxysteroid 17-beta dehydrogenase 4 [Source:HGNC Symbol;Acc:HGNC:5213]
SOCS2	suppressor of cytokine signaling 2 [Source:HGNC Symbol;Acc:HGNC:19382]
ATP1A1	ATPase Na+/K+ transporting subunit alpha 1 [Source:HGNC Symbol;Acc:HGNC:799]
HSPB8	heat shock protein family B (small) member 8 [Source:HGNC Symbol;Acc:HGNC:30171]
ACHE	acetylcholinesterase (Cartwright blood group) [Source:HGNC Symbol;Acc:HGNC:108]
CFLAR	CASP8 and FADD like apoptosis regulator [Source:HGNC Symbol;Acc:HGNC:1876]
DNAJA1	DnaJ heat shock protein family (Hsp40) member A1 [Source:HGNC Symbol;Acc:HGNC:5229]
GCH1	GTP cyclohydrolase 1 [Source:HGNC Symbol;Acc:HGNC:4193]
SELENBP1	selenium binding protein 1 [Source:HGNC Symbol;Acc:HGNC:10719]
TSHB	thyroid stimulating hormone beta [Source:HGNC Symbol;Acc:HGNC:12372]
CREM	cAMP responsive element modulator [Source:HGNC Symbol;Acc:HGNC:2352]
KLK3	kallikrein related peptidase 3 [Source:HGNC Symbol;Acc:HGNC:6364]
ORM1	orosomucoid 1 [Source:HGNC Symbol;Acc:HGNC:8498]
PRDX6	peroxiredoxin 6 [Source:HGNC Symbol;Acc:HGNC:16753]
RGS2	regulator of G-protein signaling 2 [Source:HGNC Symbol;Acc:HGNC:9998]
SOX9	SRY-box 9 [Source:HGNC Symbol;Acc:HGNC:11204]
PER2	period circadian clock 2 [Source:HGNC Symbol;Acc:HGNC:8846]
AQP3	aquaporin 3 (Gill blood group) [Source:HGNC Symbol;Acc:HGNC:636]
BCL6	B-cell CLL/lymphoma 6 [Source:HGNC Symbol;Acc:HGNC:1001]
ENC1	ectodermal-neural cortex 1 [Source:HGNC Symbol;Acc:HGNC:3345]
LPIN1	lipin 1 [Source:HGNC Symbol;Acc:HGNC:13345]

MGP	matrix Gla protein [Source:HGNC Symbol;Acc:HGNC:7060]
ADRB1	adrenoceptor beta 1 [Source:HGNC Symbol;Acc:HGNC:285]
CDKN3	cyclin dependent kinase inhibitor 3 [Source:HGNC Symbol;Acc:HGNC:1791]
TFF1	trefoil factor 1 [Source:HGNC Symbol;Acc:HGNC:11755]
TPI1	triosephosphate isomerase 1 [Source:HGNC Symbol;Acc:HGNC:12009]
CENPF	centromere protein F [Source:HGNC Symbol;Acc:HGNC:1857]
EEF2	eukaryotic translation elongation factor 2 [Source:HGNC Symbol;Acc:HGNC:3214]
IRS2	insulin receptor substrate 2 [Source:HGNC Symbol;Acc:HGNC:6126]
APOA4	apolipoprotein A4 [Source:HGNC Symbol;Acc:HGNC:602]
GGT1	gamma-glutamyltransferase 1 [Source:HGNC Symbol;Acc:HGNC:4250]
PGK1	phosphoglycerate kinase 1 [Source:HGNC Symbol;Acc:HGNC:8896]
ENPP2	ectonucleotide pyrophosphatase/phosphodiesterase 2 [Source:HGNC Symbol;Acc:HGNC:3357]
PDIA3	protein disulfide isomerase family A member 3 [Source:HGNC Symbol;Acc:HGNC:4606]
ENO2	enolase 2 [Source:HGNC Symbol;Acc:HGNC:3353]
IDI1	isopentenyl-diphosphate delta isomerase 1 [Source:HGNC Symbol;Acc:HGNC:5387]
LEPR	leptin receptor [Source:HGNC Symbol;Acc:HGNC:6554]
MCM5	minichromosome maintenance complex component 5 [Source:HGNC Symbol;Acc:HGNC:6948]
PTTG1	pituitary tumor-transforming 1 [Source:HGNC Symbol;Acc:HGNC:9690]
UBE2C	ubiquitin conjugating enzyme E2 C [Source:HGNC Symbol;Acc:HGNC:15937]
SREBF2	sterol regulatory element binding transcription factor 2 [Source:HGNC Symbol;Acc:HGNC:11290]
MYH7	myosin, heavy chain 7, cardiac muscle, beta [Source:HGNC Symbol;Acc:HGNC:7577]
SLC2A3	solute carrier family 2 member 3 [Source:HGNC Symbol;Acc:HGNC:11007]
IFRD1	interferon related developmental regulator 1 [Source:HGNC Symbol;Acc:HGNC:5456]
SAT1	spermidine/spermine N1-acetyltransferase 1 [Source:HGNC Symbol;Acc:HGNC:10540]
ACADM	acyl-CoA dehydrogenase, C-4 to C-12 straight chain [Source:HGNC Symbol;Acc:HGNC:89]
EP300	E1A binding protein p300 [Source:HGNC Symbol;Acc:HGNC:3373]
MCM6	minichromosome maintenance complex component 6 [Source:HGNC Symbol;Acc:HGNC:6949]
PDK1	pyruvate dehydrogenase kinase 1 [Source:HGNC Symbol;Acc:HGNC:8809]
SLC16A1	solute carrier family 16 member 1 [Source:HGNC Symbol;Acc:HGNC:10922]
ACSL1	acyl-CoA synthetase long-chain family member 1 [Source:HGNC Symbol;Acc:HGNC:3569]
PSAT1	phosphoserine aminotransferase 1 [Source:HGNC Symbol;Acc:HGNC:19129]
HSPA9	heat shock protein family A (Hsp70) member 9 [Source:HGNC Symbol;Acc:HGNC:5244]
MAP2K2	mitogen-activated protein kinase kinase 2 [Source:HGNC Symbol;Acc:HGNC:6842]
TGFA	transforming growth factor alpha [Source:HGNC Symbol;Acc:HGNC:11765]

CYP2E1	cytochrome P450 family 2 subfamily E member 1 [Source:HGNC Symbol;Acc:HGNC:2631]
ITPR1	inositol 1,4,5-trisphosphate receptor type 1 [Source:HGNC Symbol;Acc:HGNC:6180]
MCM2	minichromosome maintenance complex component 2 [Source:HGNC Symbol;Acc:HGNC:6944]
PDGFRA	platelet derived growth factor receptor alpha [Source:HGNC Symbol;Acc:HGNC:8803]
BNIP3	BCL2/adenovirus E1B 19kDa interacting protein 3 [Source:HGNC Symbol;Acc:HGNC:1084]
DNAJB9	DnaJ heat shock protein family (Hsp40) member B9 [Source:HGNC Symbol;Acc:HGNC:6968]
DNMT3A	DNA (cytosine-5-)-methyltransferase 3 alpha [Source:HGNC Symbol;Acc:HGNC:2978]
CHEK2	checkpoint kinase 2 [Source:HGNC Symbol;Acc:HGNC:16627]
SLC7A11	solute carrier family 7 member 11 [Source:HGNC Symbol;Acc:HGNC:11059]
TK1	thymidine kinase 1 [Source:HGNC Symbol;Acc:HGNC:11830]
HK2	hexokinase 2 [Source:HGNC Symbol;Acc:HGNC:4923]
INHBA	inhibin beta A subunit [Source:HGNC Symbol;Acc:HGNC:6066]
ALDOA	aldolase, fructose-bisphosphate A [Source:HGNC Symbol;Acc:HGNC:414]
APEX1	apurinic/apyrimidinic endodeoxyribonuclease 1 [Source:HGNC Symbol;Acc:HGNC:587]
JUND	JunD proto-oncogene, AP-1 transcription factor subunit [Source:HGNC Symbol;Acc:HGNC:6206]
MT1A	metallothionein 1A [Source:HGNC Symbol;Acc:HGNC:7393]
AURKA	aurora kinase A [Source:HGNC Symbol;Acc:HGNC:11393]
VDR	vitamin D (1,25- dihydroxyvitamin D3) receptor [Source:HGNC Symbol;Acc:HGNC:12679]
RARA	retinoic acid receptor alpha [Source:HGNC Symbol;Acc:HGNC:9864]
SLC22A1	solute carrier family 22 member 1 [Source:HGNC Symbol;Acc:HGNC:10963]
FABP1	fatty acid binding protein 1 [Source:HGNC Symbol;Acc:HGNC:3555]
SLC2A2	solute carrier family 2 member 2 [Source:HGNC Symbol;Acc:HGNC:11006]
ME1	malic enzyme 1 [Source:HGNC Symbol;Acc:HGNC:6983]
NR1H4	nuclear receptor subfamily 1 group H member 4 [Source:HGNC Symbol;Acc:HGNC:7967]
IL12A	interleukin 12A [Source:HGNC Symbol;Acc:HGNC:5969]
PRDX2	peroxiredoxin 2 [Source:HGNC Symbol;Acc:HGNC:9353]
BGLAP	bone gamma-carboxyglutamate protein [Source:HGNC Symbol;Acc:HGNC:1043]
BIRC2	baculoviral IAP repeat containing 2 [Source:HGNC Symbol;Acc:HGNC:590]
INSR	insulin receptor [Source:HGNC Symbol;Acc:HGNC:6091]
NDRG1	N-myc downstream regulated 1 [Source:HGNC Symbol;Acc:HGNC:7679]
DUSP6	dual specificity phosphatase 6 [Source:HGNC Symbol;Acc:HGNC:3072]
IFT172	intraflagellar transport 172 [Source:HGNC Symbol;Acc:HGNC:30391]
CCND3	cyclin D3 [Source:HGNC Symbol;Acc:HGNC:1585]
HSD3B1	hydroxy-delta-5-steroid dehydrogenase, 3 beta- and steroid delta-isomerase 1 [Source:HGNC Symbol;Acc:HGNC:5217]

IDH2	isocitrate dehydrogenase (NADP(+)) 2, mitochondrial [Source:HGNC Symbol;Acc:HGNC:5383]
MSMO1	methylsterol monooxygenase 1 [Source:HGNC Symbol;Acc:HGNC:10545]
SQLE	squalene epoxidase [Source:HGNC Symbol;Acc:HGNC:11279]
STMN1	stathmin 1 [Source:HGNC Symbol;Acc:HGNC:6510]
BMP2	bone morphogenetic protein 2 [Source:HGNC Symbol;Acc:HGNC:1069]
S100A8	S100 calcium binding protein A8 [Source:HGNC Symbol;Acc:HGNC:10498]
AKR1C2	aldo-keto reductase family 1 member C2 [Source:HGNC Symbol;Acc:HGNC:385]
PLIN2	perilipin 2 [Source:HGNC Symbol;Acc:HGNC:248]
HSP90B1	heat shock protein 90kDa beta family member 1 [Source:HGNC Symbol;Acc:HGNC:12028]
ITGB1	integrin subunit beta 1 [Source:HGNC Symbol;Acc:HGNC:6153]
HSP90AB1	heat shock protein 90kDa alpha family class B member 1 [Source:HGNC Symbol;Acc:HGNC:5258]
THRA	thyroid hormone receptor, alpha [Source:HGNC Symbol;Acc:HGNC:11796]
HSPA1B	heat shock protein family A (Hsp70) member 1B [Source:HGNC Symbol;Acc:HGNC:5233]
TNFRSF11B	tumor necrosis factor receptor superfamily member 11b [Source:HGNC Symbol;Acc:HGNC:11909]
TNFSF11	tumor necrosis factor superfamily member 11 [Source:HGNC Symbol;Acc:HGNC:11926]
CCNB2	cyclin B2 [Source:HGNC Symbol;Acc:HGNC:1580]
NCF1	neutrophil cytosolic factor 1 [Source:HGNC Symbol;Acc:HGNC:7660]
COL2A1	collagen type II alpha 1 chain [Source:HGNC Symbol;Acc:HGNC:2200]
AGAP5	ArfGAP with GTPase domain, ankyrin repeat and PH domain 5 [Source:HGNC Symbol;Acc:HGNC:23467]
B3GAT3	beta-1,3-glucuronyltransferase 3 [Source:HGNC Symbol;Acc:HGNC:923]
OR4F16	olfactory receptor family 4 subfamily F member 16 [Source:HGNC Symbol;Acc:HGNC:15079]
SMARCB1	SWI/SNF related, matrix associated, actin dependent regulator of chromatin, subfamily b, member 1 [Source:HGNC Symbol;Acc:HGNC:11103]
PLRG1	pleiotropic regulator 1 [Source:HGNC Symbol;Acc:HGNC:9089]
SLC35F6	solute carrier family 35 member F6 [Source:HGNC Symbol;Acc:HGNC:26055]
MAFF	MAF bZIP transcription factor F [Source:HGNC Symbol;Acc:HGNC:6780]
RPS6	ribosomal protein S6 [Source:HGNC Symbol;Acc:HGNC:10429]
TNFAIP3	TNF alpha induced protein 3 [Source:HGNC Symbol;Acc:HGNC:11896]
CCND2	cyclin D2 [Source:HGNC Symbol;Acc:HGNC:1583]
FOSL1	FOS like 1, AP-1 transcription factor subunit [Source:HGNC Symbol;Acc:HGNC:13718]
PCK1	phosphoenolpyruvate carboxykinase 1 [Source:HGNC Symbol;Acc:HGNC:8724]
HRAS	Harvey rat sarcoma viral oncogene homolog [Source:HGNC Symbol;Acc:HGNC:5173]
TYMS	thymidylate synthetase [Source:HGNC Symbol;Acc:HGNC:12441]
NR4A1	nuclear receptor subfamily 4 group A member 1 [Source:HGNC Symbol;Acc:HGNC:7980]
CHEK1	checkpoint kinase 1 [Source:HGNC Symbol;Acc:HGNC:1925]

CALCA	calcitonin related polypeptide alpha [Source:HGNC Symbol;Acc:HGNC:1437]
BAK1	BCL2 antagonist/killer 1 [Source:HGNC Symbol;Acc:HGNC:949]
DRD2	dopamine receptor D2 [Source:HGNC Symbol;Acc:HGNC:3023]
ACOX1	acyl-CoA oxidase 1 [Source:HGNC Symbol;Acc:HGNC:119]
PRDX1	peroxiredoxin 1 [Source:HGNC Symbol;Acc:HGNC:9352]
IRS1	insulin receptor substrate 1 [Source:HGNC Symbol;Acc:HGNC:6125]
PRKCD	protein kinase C delta [Source:HGNC Symbol;Acc:HGNC:9399]
EIF4EBP1	eukaryotic translation initiation factor 4E binding protein 1 [Source:HGNC Symbol;Acc:HGNC:3288]
ODC1	ornithine decarboxylase 1 [Source:HGNC Symbol;Acc:HGNC:8109]
RAF1	Raf-1 proto-oncogene, serine/threonine kinase [Source:HGNC Symbol;Acc:HGNC:9829]
PMAIP1	phorbol-12-myristate-13-acetate-induced protein 1 [Source:HGNC Symbol;Acc:HGNC:9108]
THRB	thyroid hormone receptor beta [Source:HGNC Symbol;Acc:HGNC:11799]
COL3A1	collagen type III alpha 1 chain [Source:HGNC Symbol;Acc:HGNC:2201]
RUNX2	runt related transcription factor 2 [Source:HGNC Symbol;Acc:HGNC:10472]
BAD	BCL2 associated agonist of cell death [Source:HGNC Symbol;Acc:HGNC:936]
DCANP1	dendritic cell-associated nuclear protein [Source:HGNC Symbol;Acc:HGNC:24459]
AIFM1	apoptosis inducing factor, mitochondria associated 1 [Source:HGNC Symbol;Acc:HGNC:8768]
CYP2D6	cytochrome P450 family 2 subfamily D member 6 [Source:HGNC Symbol;Acc:HGNC:2625]
BIRC3	baculoviral IAP repeat containing 3 [Source:HGNC Symbol;Acc:HGNC:591]
TNFRSF10B	tumor necrosis factor receptor superfamily member 10b [Source:HGNC Symbol;Acc:HGNC:11905]
COL1A2	collagen type I alpha 2 chain [Source:HGNC Symbol;Acc:HGNC:2198]
CLC	Charcot-Leyden crystal galectin [Source:HGNC Symbol;Acc:HGNC:2014]
JUNB	JunB proto-oncogene, AP-1 transcription factor subunit [Source:HGNC Symbol;Acc:HGNC:6205]
CEBPA	CCAAT/enhancer binding protein alpha [Source:HGNC Symbol;Acc:HGNC:1833]
GSTA3	glutathione S-transferase alpha 3 [Source:HGNC Symbol;Acc:HGNC:4628]
HSPD1	heat shock protein family D (Hsp60) member 1 [Source:HGNC Symbol;Acc:HGNC:5261]
POR	cytochrome p450 oxidoreductase [Source:HGNC Symbol;Acc:HGNC:9208]
ARGFX	arginine-fifty homeobox [Source:HGNC Symbol;Acc:HGNC:30146]
GAGE12B	G antigen 12B [Source:HGNC Symbol;Acc:HGNC:26779]
GAGE12J	G antigen 12J [Source:HGNC Symbol;Acc:HGNC:17778]
GOLGA6L10	golgin A6 family-like 10 [Source:HGNC Symbol;Acc:HGNC:37228]
OR10H3	olfactory receptor family 10 subfamily H member 3 [Source:HGNC Symbol;Acc:HGNC:8174]
OR1D5	olfactory receptor family 1 subfamily D member 5 [Source:HGNC Symbol;Acc:HGNC:8186]

OR1L3	olfactory receptor family 1 subfamily L member 3 [Source:HGNC Symbol;Acc:HGNC:8215]
OR2T34	olfactory receptor family 2 subfamily T member 34 [Source:HGNC Symbol;Acc:HGNC:31256]
OR4D5	olfactory receptor family 4 subfamily D member 5 [Source:HGNC Symbol;Acc:HGNC:14852]
OR4F21	olfactory receptor family 4 subfamily F member 21 [Source:HGNC Symbol;Acc:HGNC:19583]
OR5AN1	olfactory receptor family 5 subfamily AN member 1 [Source:HGNC Symbol;Acc:HGNC:15255]
OR8B3	olfactory receptor family 8 subfamily B member 3 [Source:HGNC Symbol;Acc:HGNC:8472]
PRAMEF14	PRAME family member 14 [Source:HGNC Symbol;Acc:HGNC:13576]
PRR20A	proline rich 20A [Source:HGNC Symbol;Acc:HGNC:24754]
TNFRSF17	tumor necrosis factor receptor superfamily member 17 [Source:HGNC Symbol;Acc:HGNC:11913]
FSHB	follicle stimulating hormone beta subunit [Source:HGNC Symbol;Acc:HGNC:3964]
NPPA	natriuretic peptide A [Source:HGNC Symbol;Acc:HGNC:7939]
LDLR	low density lipoprotein receptor [Source:HGNC Symbol;Acc:HGNC:6547]
PRL	prolactin [Source:HGNC Symbol;Acc:HGNC:9445]
ELL	elongation factor for RNA polymerase II [Source:HGNC Symbol;Acc:HGNC:23114]
ENTPD3	ectonucleoside triphosphate diphosphohydrolase 3 [Source:HGNC Symbol;Acc:HGNC:3365]
SYNJ2BP-COX16	SYNJ2BP-COX16 readthrough [Source:HGNC Symbol;Acc:HGNC:48350]
CYP11A1	cytochrome P450 family 11 subfamily A member 1 [Source:HGNC Symbol;Acc:HGNC:2590]
FABP4	fatty acid binding protein 4 [Source:HGNC Symbol;Acc:HGNC:3559]
GDF15	growth differentiation factor 15 [Source:HGNC Symbol;Acc:HGNC:30142]
DEFA6	defensin alpha 6 [Source:HGNC Symbol;Acc:HGNC:2765]
UXT	ubiquitously expressed prefoldin like chaperone [Source:HGNC Symbol;Acc:HGNC:12641]
CDK4	cyclin dependent kinase 4 [Source:HGNC Symbol;Acc:HGNC:1773]
XDH	xanthine dehydrogenase [Source:HGNC Symbol;Acc:HGNC:12805]
GPX6	glutathione peroxidase 6 [Source:HGNC Symbol;Acc:HGNC:4558]
ITK	IL2 inducible T-cell kinase [Source:HGNC Symbol;Acc:HGNC:6171]
DUSP1	dual specificity phosphatase 1 [Source:HGNC Symbol;Acc:HGNC:3064]
MCL1	myeloid cell leukemia 1 [Source:HGNC Symbol;Acc:HGNC:6943]
CDRT4	CMT1A duplicated region transcript 4 [Source:HGNC Symbol;Acc:HGNC:14383]
LGALS16	galectin 16 [Source:HGNC Symbol;Acc:HGNC:40039]
OR8J3	olfactory receptor family 8 subfamily J member 3 [Source:HGNC Symbol;Acc:HGNC:15312]
TMCO5A	transmembrane and coiled-coil domains 5A [Source:HGNC Symbol;Acc:HGNC:28558]
TPSD1	tryptase delta 1 [Source:HGNC Symbol;Acc:HGNC:14118]

C11orf40	chromosome 11 open reading frame 40 [Source:HGNC Symbol;Acc:HGNC:23986]
CT45A2	cancer/testis antigen family 45, member A2 [Source:HGNC Symbol;Acc:HGNC:28400]
CT45A6	cancer/testis antigen family 45, member A6 [Source:HGNC Symbol;Acc:HGNC:33271]
DEFB108B	defensin beta 108B [Source:HGNC Symbol;Acc:HGNC:29966]
HBS1L	HBS1 like translational GTPase [Source:HGNC Symbol;Acc:HGNC:4834]
GAGE12G	G antigen 12G [Source:HGNC Symbol;Acc:HGNC:31907]
GOLGA6L1	golgin A6 family-like 1 [Source:HGNC Symbol;Acc:HGNC:37444]
KIR2DL2	killer cell immunoglobulin like receptor, two Ig domains and long cytoplasmic tail 2 [Source:HGNC Symbol;Acc:HGNC:6330]
NBPF6	neuroblastoma breakpoint family member 6 [Source:HGNC Symbol;Acc:HGNC:31988]
OR6C75	olfactory receptor family 6 subfamily C member 75 [Source:HGNC Symbol;Acc:HGNC:31304]
OR6S1	olfactory receptor family 6 subfamily S member 1 [Source:HGNC Symbol;Acc:HGNC:15363]
RECQL4	RecQ like helicase 4 [Source:HGNC Symbol;Acc:HGNC:9949]
SPDYE5	speedy/RINGO cell cycle regulator family member E5 [Source:HGNC Symbol;Acc:HGNC:35464]
ADH4	alcohol dehydrogenase 4 (class II), pi polypeptide [Source:HGNC Symbol;Acc:HGNC:252]
ABCC3	ATP binding cassette subfamily C member 3 [Source:HGNC Symbol;Acc:HGNC:54]
ADH1A	alcohol dehydrogenase 1A (class I), alpha polypeptide [Source:HGNC Symbol;Acc:HGNC:249]
SLAMF7	SLAM family member 7 [Source:HGNC Symbol;Acc:HGNC:21394]
TRIM21	tripartite motif containing 21 [Source:HGNC Symbol;Acc:HGNC:11312]
LHB	luteinizing hormone beta polypeptide [Source:HGNC Symbol;Acc:HGNC:6584]
SSX3	SSX family member 3 [Source:HGNC Symbol;Acc:HGNC:11337]
DEFA3	defensin alpha 3 [Source:HGNC Symbol;Acc:HGNC:2762]
MS4A1	membrane spanning 4-domains A1 [Source:HGNC Symbol;Acc:HGNC:7315]
PRG3	proteoglycan 3 [Source:HGNC Symbol;Acc:HGNC:9363]
MAVS	mitochondrial antiviral signaling protein [Source:HGNC Symbol;Acc:HGNC:29233]
SLC9A3R1	SLC9A3 regulator 1 [Source:HGNC Symbol;Acc:HGNC:11075]
CD27	CD27 molecule [Source:HGNC Symbol;Acc:HGNC:11922]
MCEMP1	mast cell-expressed membrane protein 1 [Source:HGNC Symbol;Acc:HGNC:27291]
TREML2	triggering receptor expressed on myeloid cells like 2 [Source:HGNC Symbol;Acc:HGNC:21092]
ABCB4	ATP binding cassette subfamily B member 4 [Source:HGNC Symbol;Acc:HGNC:45]
POMC	proopiomelanocortin [Source:HGNC Symbol;Acc:HGNC:9201]
HNRNPM	heterogeneous nuclear ribonucleoprotein M [Source:HGNC Symbol;Acc:HGNC:5046]
KIR2DS2	killer cell immunoglobulin like receptor, two Ig domains and short cytoplasmic tail 2 [Source:HGNC Symbol;Acc:HGNC:6334]
KRTAP2-1	keratin associated protein 2-1 [Source:HGNC Symbol;Acc:HGNC:16775]
OR2AT4	olfactory receptor family 2 subfamily AT member 4 [Source:HGNC Symbol;Acc:HGNC:19620]

OR2T3	olfactory receptor family 2 subfamily T member 3 [Source:HGNC Symbol;Acc:HGNC:14727]
OR2Y1	olfactory receptor family 2 subfamily Y member 1 [Source:HGNC Symbol;Acc:HGNC:14837]
OR3A1	olfactory receptor family 3 subfamily A member 1 [Source:HGNC Symbol;Acc:HGNC:8282]
OR5B3	olfactory receptor family 5 subfamily B member 3 [Source:HGNC Symbol;Acc:HGNC:8324]
OR8B2	olfactory receptor family 8 subfamily B member 2 [Source:HGNC Symbol;Acc:HGNC:8471]
PRAMEF1	PRAME family member 1 [Source:HGNC Symbol;Acc:HGNC:28840]
TRIM49	tripartite motif containing 49 [Source:HGNC Symbol;Acc:HGNC:13431]
TSPAN16	tetraspanin 16 [Source:HGNC Symbol;Acc:HGNC:30725]
ZIM3	zinc finger imprinted 3 [Source:HGNC Symbol;Acc:HGNC:16366]
BID	BH3 interacting domain death agonist [Source:HGNC Symbol;Acc:HGNC:1050]
CCNE1	cyclin E1 [Source:HGNC Symbol;Acc:HGNC:1589]
ALDH3A1	aldehyde dehydrogenase 3 family member A1 [Source:HGNC Symbol;Acc:HGNC:405]
IFI44L	interferon induced protein 44 like [Source:HGNC Symbol;Acc:HGNC:17817]
MKI67	marker of proliferation Ki-67 [Source:HGNC Symbol;Acc:HGNC:7107]
STAR	steroidogenic acute regulatory protein [Source:HGNC Symbol;Acc:HGNC:11359]
IRF4	interferon regulatory factor 4 [Source:HGNC Symbol;Acc:HGNC:6119]
CCNA2	cyclin A2 [Source:HGNC Symbol;Acc:HGNC:1578]
CYP17A1	cytochrome P450 family 17 subfamily A member 1 [Source:HGNC Symbol;Acc:HGNC:2593]
H2AFX	H2A histone family member X [Source:HGNC Symbol;Acc:HGNC:4739]
TXNRD1	thioredoxin reductase 1 [Source:HGNC Symbol;Acc:HGNC:12437]
CSNK1A1L	casein kinase 1 alpha 1 like [Source:HGNC Symbol;Acc:HGNC:20289]
REG1B	regenerating family member 1 beta [Source:HGNC Symbol;Acc:HGNC:9952]
APOL5	apolipoprotein L5 [Source:HGNC Symbol;Acc:HGNC:14869]
CT45A3	cancer/testis antigen family 45, member A3 [Source:HGNC Symbol;Acc:HGNC:33268]
MAP3K1	mitogen-activated protein kinase kinase kinase 1 [Source:HGNC Symbol;Acc:HGNC:6848]
MROH9	maestro heat like repeat family member 9 [Source:HGNC Symbol;Acc:HGNC:26287]
NBPF4	neuroblastoma breakpoint family member 4 [Source:HGNC Symbol;Acc:HGNC:26550]
OR1J1	olfactory receptor family 1 subfamily J member 1 [Source:HGNC Symbol;Acc:HGNC:8208]
OR4C46	olfactory receptor family 4 subfamily C member 46 [Source:HGNC Symbol;Acc:HGNC:31271]
OR4X1	olfactory receptor family 4 subfamily X member 1 (gene/pseudogene) [Source:HGNC Symbol;Acc:HGNC:14854]
PAGE2	PAGE family member 2 [Source:HGNC Symbol;Acc:HGNC:31804]
PRAMEF5	PRAME family member 5 [Source:HGNC Symbol;Acc:HGNC:27995]
TBC1D3	TBC1 domain family member 3 [Source:HGNC Symbol;Acc:HGNC:19031]
GBP1	guanylate binding protein 1 [Source:HGNC Symbol;Acc:HGNC:4182]

GPR34	G protein-coupled receptor 34 [Source:HGNC Symbol;Acc:HGNC:4490]
LIPA	lipase A, lysosomal acid type [Source:HGNC Symbol;Acc:HGNC:6617]
RIPK2	receptor interacting serine/threonine kinase 2 [Source:HGNC Symbol;Acc:HGNC:10020]
SPON1	spondin 1 [Source:HGNC Symbol;Acc:HGNC:11252]
GKN2	gastrokine 2 [Source:HGNC Symbol;Acc:HGNC:24588]
FOLR3	folate receptor 3 (gamma) [Source:HGNC Symbol;Acc:HGNC:3795]
C17orf64	chromosome 17 open reading frame 64 [Source:HGNC Symbol;Acc:HGNC:26990]
C20orf144	chromosome 20 open reading frame 144 [Source:HGNC Symbol;Acc:HGNC:16137]
KIR2DS4	killer cell immunoglobulin like receptor, two Ig domains and short cytoplasmic tail 4 [Source:HGNC Symbol;Acc:HGNC:6336]
KRTAP10-6	keratin associated protein 10-6 [Source:HGNC Symbol;Acc:HGNC:20523]
MS4A12	membrane spanning 4-domains A12 [Source:HGNC Symbol;Acc:HGNC:13370]
OR10J1	olfactory receptor family 10 subfamily J member 1 [Source:HGNC Symbol;Acc:HGNC:8175]
OR1L8	olfactory receptor family 1 subfamily L member 8 [Source:HGNC Symbol;Acc:HGNC:15110]
OR4K1	olfactory receptor family 4 subfamily K member 1 [Source:HGNC Symbol;Acc:HGNC:14726]
OR52D1	olfactory receptor family 52 subfamily D member 1 [Source:HGNC Symbol;Acc:HGNC:15212]
TRIM51	tripartite motif-containing 51 [Source:HGNC Symbol;Acc:HGNC:19023]
TRAF3	TNF receptor associated factor 3 [Source:HGNC Symbol;Acc:HGNC:12033]
IL10RA	interleukin 10 receptor subunit alpha [Source:HGNC Symbol;Acc:HGNC:5964]
ANKRD30B	ankyrin repeat domain 30B [Source:HGNC Symbol;Acc:HGNC:24165]
NFATC2	nuclear factor of activated T-cells 2 [Source:HGNC Symbol;Acc:HGNC:7776]
ZNF845	zinc finger protein 845 [Source:HGNC Symbol;Acc:HGNC:25112]
GKN1	gastrokine 1 [Source:HGNC Symbol;Acc:HGNC:23217]
LAMP5	lysosomal associated membrane protein family member 5 [Source:HGNC Symbol;Acc:HGNC:16097]
CLPS	colipase [Source:HGNC Symbol;Acc:HGNC:2085]
KLHDC7B	kelch domain containing 7B [Source:HGNC Symbol;Acc:HGNC:25145]
PGR	progesterone receptor [Source:HGNC Symbol;Acc:HGNC:8910]
ARL14EPL	ADP ribosylation factor like GTPase 14 effector protein like [Source:HGNC Symbol;Acc:HGNC:44201]
FRG2C	FSHD region gene 2 family member C [Source:HGNC Symbol;Acc:HGNC:33626]
KIR3DL3	killer cell immunoglobulin like receptor, three Ig domains and long cytoplasmic tail 3 [Source:HGNC Symbol;Acc:HGNC:16312]
KRTAP5-2	keratin associated protein 5-2 [Source:HGNC Symbol;Acc:HGNC:23597]
LRRC25	leucine rich repeat containing 25 [Source:HGNC Symbol;Acc:HGNC:29806]
MUC7	mucin 7, secreted [Source:HGNC Symbol;Acc:HGNC:7518]
OR4Q3	olfactory receptor family 4 subfamily Q member 3 [Source:HGNC Symbol;Acc:HGNC:15426]
PRDM1	PR domain 1 [Source:HGNC Symbol;Acc:HGNC:9346]

CYP1A1	cytochrome P450 family 1 subfamily A member 1 [Source:HGNC Symbol;Acc:HGNC:2595]
GADD45A	growth arrest and DNA damage inducible alpha [Source:HGNC Symbol;Acc:HGNC:4095]
PCNA	proliferating cell nuclear antigen [Source:HGNC Symbol;Acc:HGNC:8729]
CDK1	cyclin dependent kinase 1 [Source:HGNC Symbol;Acc:HGNC:1722]
CDKN1B	cyclin dependent kinase inhibitor 1B [Source:HGNC Symbol;Acc:HGNC:1785]
ASCL3	achaete-scute family bHLH transcription factor 3 [Source:HGNC Symbol;Acc:HGNC:740]
C3orf80	chromosome 3 open reading frame 80 [Source:HGNC Symbol;Acc:HGNC:40048]
C7orf25	chromosome 7 open reading frame 25 [Source:HGNC Symbol;Acc:HGNC:21703]
HSBP1L1	heat shock factor binding protein 1-like 1 [Source:HGNC Symbol;Acc:HGNC:37243]
IQCD	IQ motif containing D [Source:HGNC Symbol;Acc:HGNC:25168]
LRRC37A3	leucine rich repeat containing 37 member A3 [Source:HGNC Symbol;Acc:HGNC:32427]
TAF7L	TATA-box binding protein associated factor 7 like [Source:HGNC Symbol;Acc:HGNC:11548]
TGM7	transglutaminase 7 [Source:HGNC Symbol;Acc:HGNC:30790]
ZNF134	zinc finger protein 134 [Source:HGNC Symbol;Acc:HGNC:12918]
ZNF496	zinc finger protein 496 [Source:HGNC Symbol;Acc:HGNC:23713]
ZNF611	zinc finger protein 611 [Source:HGNC Symbol;Acc:HGNC:28766]
ACTA2	actin, alpha 2, smooth muscle, aorta [Source:HGNC Symbol;Acc:HGNC:130]
MMP10	matrix metallopeptidase 10 [Source:HGNC Symbol;Acc:HGNC:7156]
PIWIL3	piwi like RNA-mediated gene silencing 3 [Source:HGNC Symbol;Acc:HGNC:18443]
PLPP3	phospholipid phosphatase 3 [Source:HGNC Symbol;Acc:HGNC:9229]
SAA1	serum amyloid A1 [Source:HGNC Symbol;Acc:HGNC:10513]
BEX5	brain expressed X-linked 5 [Source:HGNC Symbol;Acc:HGNC:27990]
C5orf51	chromosome 5 open reading frame 51 [Source:HGNC Symbol;Acc:HGNC:27750]
CLEC2B	C-type lectin domain family 2 member B [Source:HGNC Symbol;Acc:HGNC:2053]
ETV7	ETS variant 7 [Source:HGNC Symbol;Acc:HGNC:18160]
ITLN1	intelectin 1 [Source:HGNC Symbol;Acc:HGNC:18259]
LCK	LCK proto-oncogene, Src family tyrosine kinase [Source:HGNC Symbol;Acc:HGNC:6524]
PSG3	pregnancy specific beta-1-glycoprotein 3 [Source:HGNC Symbol;Acc:HGNC:9520]
SP6	Sp6 transcription factor [Source:HGNC Symbol;Acc:HGNC:14530]
VSIG10L	V-set and immunoglobulin domain containing 10 like [Source:HGNC Symbol;Acc:HGNC:27111]
CSTL1	cystatin like 1 [Source:HGNC Symbol;Acc:HGNC:15958]
FAM177B	family with sequence similarity 177 member B [Source:HGNC Symbol;Acc:HGNC:34395]
ALG1	ALG1, chitobiosyldiphosphodolichol beta-mannosyltransferase [Source:HGNC Symbol;Acc:HGNC:18294]
ENOPH1	enolase-phosphatase 1 [Source:HGNC Symbol;Acc:HGNC:24599]

NXF5	nuclear RNA export factor 5 [Source:HGNC Symbol;Acc:HGNC:8075]
OCM2	oncomodulin 2 [Source:HGNC Symbol;Acc:HGNC:34396]
SPDYE2	speedy/RINGO cell cycle regulator family member E2 [Source:HGNC Symbol;Acc:HGNC:33841]
USP50	ubiquitin specific peptidase 50 [Source:HGNC Symbol;Acc:HGNC:20079]
CCSAP	centriole, cilia and spindle associated protein [Source:HGNC Symbol;Acc:HGNC:29578]
NR3C1	nuclear receptor subfamily 3 group C member 1 [Source:HGNC Symbol;Acc:HGNC:7978]
AHSP	alpha hemoglobin stabilizing protein [Source:HGNC Symbol;Acc:HGNC:18075]
CATSPER1	cation channel sperm associated 1 [Source:HGNC Symbol;Acc:HGNC:17116]
CFAP161	cilia and flagella associated protein 161 [Source:HGNC Symbol;Acc:HGNC:26782]
ZNF416	zinc finger protein 416 [Source:HGNC Symbol;Acc:HGNC:20645]
ZNF585A	zinc finger protein 585A [Source:HGNC Symbol;Acc:HGNC:26305]
ZNF649	zinc finger protein 649 [Source:HGNC Symbol;Acc:HGNC:25741]
CASP7	caspase 7 [Source:HGNC Symbol;Acc:HGNC:1508]
SERPINB13	serpin family B member 13 [Source:HGNC Symbol;Acc:HGNC:8944]
ZNF35	zinc finger protein 35 [Source:HGNC Symbol;Acc:HGNC:13099]
ZNF548	zinc finger protein 548 [Source:HGNC Symbol;Acc:HGNC:26561]
ZNF675	zinc finger protein 675 [Source:HGNC Symbol;Acc:HGNC:30768]
ADGRE3	adhesion G protein-coupled receptor E3 [Source:HGNC Symbol;Acc:HGNC:23647]
CT45A5	cancer/testis antigen family 45, member A5 [Source:HGNC Symbol;Acc:HGNC:33270]
IFNA13	interferon, alpha 13 [Source:HGNC Symbol;Acc:HGNC:5419]
KIR2DL3	killer cell immunoglobulin like receptor, two Ig domains and long cytoplasmic tail 3 [Source:HGNC Symbol;Acc:HGNC:6331]
MAGEB18	MAGE family member B18 [Source:HGNC Symbol;Acc:HGNC:28515]
PLGLB2	plasminogen-like B2 [Source:HGNC Symbol;Acc:HGNC:9073]
PRSS38	protease, serine 38 [Source:HGNC Symbol;Acc:HGNC:29625]
PYURF	PIGY upstream reading frame [Source:HGNC Symbol;Acc:HGNC:44317]
RBMXL2	RNA binding motif protein, X-linked-like 2 [Source:HGNC Symbol;Acc:HGNC:17886]
SFTA2	surfactant associated 2 [Source:HGNC Symbol;Acc:HGNC:18386]
SLFN12L	schlafen family member 12 like [Source:HGNC Symbol;Acc:HGNC:33920]
SSX2	SSX family member 2 [Source:HGNC Symbol;Acc:HGNC:11336]
TBC1D3B	TBC1 domain family member 3B [Source:HGNC Symbol;Acc:HGNC:27011]
TCEAL6	transcription elongation factor A like 6 [Source:HGNC Symbol;Acc:HGNC:24553]
WFDC13	WAP four-disulfide core domain 13 [Source:HGNC Symbol;Acc:HGNC:16131]
ZNF90	zinc finger protein 90 [Source:HGNC Symbol;Acc:HGNC:13165]
ADGRF2	adhesion G protein-coupled receptor F2 [Source:HGNC Symbol;Acc:HGNC:18991]
C11orf91	chromosome 11 open reading frame 91 [Source:HGNC Symbol;Acc:HGNC:34444]
PHOSPHO1	phosphoethanolamine/phosphocholine phosphatase [Source:HGNC Symbol;Acc:HGNC:16815]
UBE2E1	ubiquitin conjugating enzyme E2 E1 [Source:HGNC Symbol;Acc:HGNC:12477]

CDIPT	CDP-diacylglycerol--inositol 3-phosphatidyltransferase [Source:HGNC Symbol;Acc:HGNC:1769]
CKMT1B	creatine kinase, mitochondrial 1B [Source:HGNC Symbol;Acc:HGNC:1995]
KLK5	kallikrein related peptidase 5 [Source:HGNC Symbol;Acc:HGNC:6366]
PNLIPRP1	pancreatic lipase related protein 1 [Source:HGNC Symbol;Acc:HGNC:9156]
ZNF225	zinc finger protein 225 [Source:HGNC Symbol;Acc:HGNC:13018]
ZNF415	zinc finger protein 415 [Source:HGNC Symbol;Acc:HGNC:20636]
TMPRSS6	transmembrane protease, serine 6 [Source:HGNC Symbol;Acc:HGNC:16517]
C1orf131	chromosome 1 open reading frame 131 [Source:HGNC Symbol;Acc:HGNC:25332]
CDK15	cyclin dependent kinase 15 [Source:HGNC Symbol;Acc:HGNC:14434]
PLD4	phospholipase D family member 4 [Source:HGNC Symbol;Acc:HGNC:23792]
TMEM252	transmembrane protein 252 [Source:HGNC Symbol;Acc:HGNC:28537]
TMEM92	transmembrane protein 92 [Source:HGNC Symbol;Acc:HGNC:26579]
CELA2B	chymotrypsin like elastase family member 2B [Source:HGNC Symbol;Acc:HGNC:29995]
FBXW5	F-box and WD repeat domain containing 5 [Source:HGNC Symbol;Acc:HGNC:13613]
FRG2B	FSHD region gene 2 family member B [Source:HGNC Symbol;Acc:HGNC:33518]
HNRNPA1L2	heterogeneous nuclear ribonucleoprotein A1-like 2 [Source:HGNC Symbol;Acc:HGNC:27067]
SSX4	SSX family member 4 [Source:HGNC Symbol;Acc:HGNC:11338]
TBC1D3F	TBC1 domain family member 3F [Source:HGNC Symbol;Acc:HGNC:18257]
USP17L2	ubiquitin specific peptidase 17-like family member 2 [Source:HGNC Symbol;Acc:HGNC:34434]
ZNF286B	zinc finger protein 286B [Source:HGNC Symbol;Acc:HGNC:33241]
ESR2	estrogen receptor 2 [Source:HGNC Symbol;Acc:HGNC:3468]
CCNB1	cyclin B1 [Source:HGNC Symbol;Acc:HGNC:1579]
CD200R1L	CD200 receptor 1 like [Source:HGNC Symbol;Acc:HGNC:24665]
GPR142	G protein-coupled receptor 142 [Source:HGNC Symbol;Acc:HGNC:20088]
GPR89B	G protein-coupled receptor 89B [Source:HGNC Symbol;Acc:HGNC:13840]
IL31RA	interleukin 31 receptor A [Source:HGNC Symbol;Acc:HGNC:18969]
KCNRG	potassium channel regulator [Source:HGNC Symbol;Acc:HGNC:18893]
ZNF43	zinc finger protein 43 [Source:HGNC Symbol;Acc:HGNC:13109]
ZNF714	zinc finger protein 714 [Source:HGNC Symbol;Acc:HGNC:27124]
ZNF805	zinc finger protein 805 [Source:HGNC Symbol;Acc:HGNC:23272]
NFKB1	nuclear factor kappa B subunit 1 [Source:HGNC Symbol;Acc:HGNC:7794]
CD300A	CD300a molecule [Source:HGNC Symbol;Acc:HGNC:19319]
TMEM154	transmembrane protein 154 [Source:HGNC Symbol;Acc:HGNC:26489]
ABCC2	ATP binding cassette subfamily C member 2 [Source:HGNC Symbol;Acc:HGNC:53]
AFP	alpha fetoprotein [Source:HGNC Symbol;Acc:HGNC:317]
GSDMB	gasdermin B [Source:HGNC Symbol;Acc:HGNC:23690]
OSBPL11	oxysterol binding protein like 11 [Source:HGNC Symbol;Acc:HGNC:16397]
TTC7A	tetratricopeptide repeat domain 7A [Source:HGNC Symbol;Acc:HGNC:19750]

ZNF273	zinc finger protein 273 [Source:HGNC Symbol;Acc:HGNC:13067]
AGAP4	ArfGAP with GTPase domain, ankyrin repeat and PH domain 4 [Source:HGNC Symbol;Acc:HGNC:23459]
GOLGA6L9	golgin A6 family-like 9 [Source:HGNC Symbol;Acc:HGNC:37229]
GSC2	goosecoid homeobox 2 [Source:HGNC Symbol;Acc:HGNC:4613]
IRF7	interferon regulatory factor 7 [Source:HGNC Symbol;Acc:HGNC:6122]
LY75	lymphocyte antigen 75 [Source:HGNC Symbol;Acc:HGNC:6729]
OR51B4	olfactory receptor family 51 subfamily B member 4 [Source:HGNC Symbol;Acc:HGNC:14708]
SPDYE1	speedy/RINGO cell cycle regulator family member E1 [Source:HGNC Symbol;Acc:HGNC:16408]
ZNF316	zinc finger protein 316 [Source:HGNC Symbol;Acc:HGNC:13843]
ACTRT2	actin related protein T2 [Source:HGNC Symbol;Acc:HGNC:24026]
FAM117B	family with sequence similarity 117 member B [Source:HGNC Symbol;Acc:HGNC:14440]
PCDHB5	protocadherin beta 5 [Source:HGNC Symbol;Acc:HGNC:8690]
POLN	polymerase (DNA) nu [Source:HGNC Symbol;Acc:HGNC:18870]
ROPN1L	rhophilin associated tail protein 1 like [Source:HGNC Symbol;Acc:HGNC:24060]
SNAPC1	small nuclear RNA activating complex polypeptide 1 [Source:HGNC Symbol;Acc:HGNC:11134]
TSHZ3	teashirt zinc finger homeobox 3 [Source:HGNC Symbol;Acc:HGNC:30700]
ZNF142	zinc finger protein 142 [Source:HGNC Symbol;Acc:HGNC:12927]
ZNF382	zinc finger protein 382 [Source:HGNC Symbol;Acc:HGNC:17409]
CTNNBIP1	catenin beta interacting protein 1 [Source:HGNC Symbol;Acc:HGNC:16913]
OPN3	opsin 3 [Source:HGNC Symbol;Acc:HGNC:14007]
CASP8AP2	caspase 8 associated protein 2 [Source:HGNC Symbol;Acc:HGNC:1510]
ULBP3	UL16 binding protein 3 [Source:HGNC Symbol;Acc:HGNC:14895]
ACTRT3	actin related protein T3 [Source:HGNC Symbol;Acc:HGNC:24022]
CD58	CD58 molecule [Source:HGNC Symbol;Acc:HGNC:1688]
NGB	neuroglobin [Source:HGNC Symbol;Acc:HGNC:14077]
SPR	sepiapterin reductase (7,8-dihydrobiopterin:NADP+ oxidoreductase) [Source:HGNC Symbol;Acc:HGNC:11257]
TIGD7	tigger transposable element derived 7 [Source:HGNC Symbol;Acc:HGNC:18331]
TINCR	tissue differentiation-inducing non-protein coding RNA [Source:HGNC Symbol;Acc:HGNC:14607]
ZNF138	zinc finger protein 138 [Source:HGNC Symbol;Acc:HGNC:12922]
ZNF567	zinc finger protein 567 [Source:HGNC Symbol;Acc:HGNC:28696]
ZNF780A	zinc finger protein 780A [Source:HGNC Symbol;Acc:HGNC:27603]
HELT	helt bHLH transcription factor [Source:HGNC Symbol;Acc:HGNC:33783]
KBTBD13	kelch repeat and BTB domain containing 13 [Source:HGNC Symbol;Acc:HGNC:37227]
LRRC37A2	leucine rich repeat containing 37 member A2 [Source:HGNC Symbol;Acc:HGNC:32404]
MS4A7	membrane spanning 4-domains A7 [Source:HGNC Symbol;Acc:HGNC:13378]

RGSL1	regulator of G-protein signaling like 1 [Source:HGNC Symbol;Acc:HGNC:18636]
EXOC6	exocyst complex component 6 [Source:HGNC Symbol;Acc:HGNC:23196]
JAZF1	JAZF zinc finger 1 [Source:HGNC Symbol;Acc:HGNC:28917]
LIG3	DNA ligase 3 [Source:HGNC Symbol;Acc:HGNC:6600]
LRRC46	leucine rich repeat containing 46 [Source:HGNC Symbol;Acc:HGNC:25047]
MTMR1	myotubularin related protein 1 [Source:HGNC Symbol;Acc:HGNC:7449]
PIP5K1B	phosphatidylinositol-4-phosphate 5-kinase type 1 beta [Source:HGNC Symbol;Acc:HGNC:8995]
PPP1R42	protein phosphatase 1 regulatory subunit 42 [Source:HGNC Symbol;Acc:HGNC:33732]
RXFP2	relaxin/insulin like family peptide receptor 2 [Source:HGNC Symbol;Acc:HGNC:17318]
ZNF469	zinc finger protein 469 [Source:HGNC Symbol;Acc:HGNC:23216]
MCOLN1	mucolipin 1 [Source:HGNC Symbol;Acc:HGNC:13356]
NR1I3	nuclear receptor subfamily 1 group I member 3 [Source:HGNC Symbol;Acc:HGNC:7969]
ANKRD22	ankyrin repeat domain 22 [Source:HGNC Symbol;Acc:HGNC:28321]
NUDT17	nudix hydrolase 17 [Source:HGNC Symbol;Acc:HGNC:26618]
PRTN3	proteinase 3 [Source:HGNC Symbol;Acc:HGNC:9495]
SNX22	sorting nexin 22 [Source:HGNC Symbol;Acc:HGNC:16315]
SYT14	synaptotagmin 14 [Source:HGNC Symbol;Acc:HGNC:23143]
TRMT2B	tRNA methyltransferase 2 homolog B [Source:HGNC Symbol;Acc:HGNC:25748]
VGLL1	vestigial like family member 1 [Source:HGNC Symbol;Acc:HGNC:20985]
ZNF239	zinc finger protein 239 [Source:HGNC Symbol;Acc:HGNC:13031]
ZNF791	zinc finger protein 791 [Source:HGNC Symbol;Acc:HGNC:26895]
ZNF92	zinc finger protein 92 [Source:HGNC Symbol;Acc:HGNC:13168]
GJA10	gap junction protein alpha 10 [Source:HGNC Symbol;Acc:HGNC:16995]
ZNF740	zinc finger protein 740 [Source:HGNC Symbol;Acc:HGNC:27465]
C7orf31	chromosome 7 open reading frame 31 [Source:HGNC Symbol;Acc:HGNC:21722]
CDR1	cerebellar degeneration related protein 1 [Source:HGNC Symbol;Acc:HGNC:1798]
CLEC3A	C-type lectin domain family 3 member A [Source:HGNC Symbol;Acc:HGNC:2052]
MUC12	mucin 12, cell surface associated [Source:HGNC Symbol;Acc:HGNC:7510]
MUC3A	mucin 3A, cell surface associated [Source:HGNC Symbol;Acc:HGNC:7513]
MZT2A	mitotic spindle organizing protein 2A [Source:HGNC Symbol;Acc:HGNC:33187]
NKX2-4	NK2 homeobox 4 [Source:HGNC Symbol;Acc:HGNC:7837]
SPZ1	spermatogenic leucine zipper 1 [Source:HGNC Symbol;Acc:HGNC:30721]
TMEM114	transmembrane protein 114 [Source:HGNC Symbol;Acc:HGNC:33227]
TPPP3	tubulin polymerization promoting protein family member 3 [Source:HGNC Symbol;Acc:HGNC:24162]
ZNF404	zinc finger protein 404 [Source:HGNC Symbol;Acc:HGNC:19417]
ZNF568	zinc finger protein 568 [Source:HGNC Symbol;Acc:HGNC:25392]
ZNF709	zinc finger protein 709 [Source:HGNC Symbol;Acc:HGNC:20629]
C5orf24	chromosome 5 open reading frame 24 [Source:HGNC Symbol;Acc:HGNC:26746]
CENPC	centromere protein C [Source:HGNC Symbol;Acc:HGNC:1854]

PCGF5	polycomb group ring finger 5 [Source:HGNC Symbol;Acc:HGNC:28264]
UPK3B	uroplakin 3B [Source:HGNC Symbol;Acc:HGNC:21444]
ZIK1	zinc finger protein interacting with K protein 1 [Source:HGNC Symbol;Acc:HGNC:33104]
ZNF493	zinc finger protein 493 [Source:HGNC Symbol;Acc:HGNC:23708]
CD109	CD109 molecule [Source:HGNC Symbol;Acc:HGNC:21685]
FAM83B	family with sequence similarity 83 member B [Source:HGNC Symbol;Acc:HGNC:21357]
GANC	glucosidase alpha, neutral C [Source:HGNC Symbol;Acc:HGNC:4139]
MIOX	myo-inositol oxygenase [Source:HGNC Symbol;Acc:HGNC:14522]
MSH4	mutS homolog 4 [Source:HGNC Symbol;Acc:HGNC:7327]
PRRT3	proline rich transmembrane protein 3 [Source:HGNC Symbol;Acc:HGNC:26591]
TRAM1L1	translocation associated membrane protein 1-like 1 [Source:HGNC Symbol;Acc:HGNC:28371]
WDR17	WD repeat domain 17 [Source:HGNC Symbol;Acc:HGNC:16661]
C8orf82	chromosome 8 open reading frame 82 [Source:HGNC Symbol;Acc:HGNC:33826]
CALML5	calmodulin like 5 [Source:HGNC Symbol;Acc:HGNC:18180]
EDDM3B	epididymal protein 3B [Source:HGNC Symbol;Acc:HGNC:19223]
FAM209B	family with sequence similarity 209 member B [Source:HGNC Symbol;Acc:HGNC:16101]
FER1L6	fer-1 like family member 6 [Source:HGNC Symbol;Acc:HGNC:28065]
HDAC8	histone deacetylase 8 [Source:HGNC Symbol;Acc:HGNC:13315]
SBK2	SH3 domain binding kinase family member 2 [Source:HGNC Symbol;Acc:HGNC:34416]
SMR3A	submaxillary gland androgen regulated protein 3A [Source:HGNC Symbol;Acc:HGNC:19216]
SYCE3	synaptonemal complex central element protein 3 [Source:HGNC Symbol;Acc:HGNC:35245]
ZNF358	zinc finger protein 358 [Source:HGNC Symbol;Acc:HGNC:16838]
TMSB4Y	thymosin beta 4, Y-linked [Source:HGNC Symbol;Acc:HGNC:11882]
UQCR11	ubiquinol-cytochrome c reductase, complex III subunit XI [Source:HGNC Symbol;Acc:HGNC:30862]
ZNF135	zinc finger protein 135 [Source:HGNC Symbol;Acc:HGNC:12919]
ALG11	ALG11, alpha-1,2-mannosyltransferase [Source:HGNC Symbol;Acc:HGNC:32456]
ANKRD36B	ankyrin repeat domain 36B [Source:HGNC Symbol;Acc:HGNC:29333]
BUD13	BUD13 homolog [Source:HGNC Symbol;Acc:HGNC:28199]
GPSM3	G-protein signaling modulator 3 [Source:HGNC Symbol;Acc:HGNC:13945]
JADE3	jade family PHD finger 3 [Source:HGNC Symbol;Acc:HGNC:22982]
PLPP1	phospholipid phosphatase 1 [Source:HGNC Symbol;Acc:HGNC:9228]
PRDM6	PR domain 6 [Source:HGNC Symbol;Acc:HGNC:9350]
THAP6	THAP domain containing 6 [Source:HGNC Symbol;Acc:HGNC:23189]
TMEM86A	transmembrane protein 86A [Source:HGNC Symbol;Acc:HGNC:26890]
TRIM27	tripartite motif containing 27 [Source:HGNC Symbol;Acc:HGNC:9975]
FAM47E	family with sequence similarity 47 member E [Source:HGNC Symbol;Acc:HGNC:34343]

PNLIP	pancreatic lipase [Source:HGNC Symbol;Acc:HGNC:9155]
POLR3E	polymerase (RNA) III subunit E [Source:HGNC Symbol;Acc:HGNC:30347]
ZNF44	zinc finger protein 44 [Source:HGNC Symbol;Acc:HGNC:13110]
ACBD3	acyl-CoA binding domain containing 3 [Source:HGNC Symbol;Acc:HGNC:15453]
FCAMR	Fc fragment of IgA and IgM receptor [Source:HGNC Symbol;Acc:HGNC:24692]
GAR1	GAR1 ribonucleoprotein [Source:HGNC Symbol;Acc:HGNC:14264]
AJUBA	ajuba LIM protein [Source:HGNC Symbol;Acc:HGNC:20250]
NUP210L	nucleoporin 210 like [Source:HGNC Symbol;Acc:HGNC:29915]
TMEM31	transmembrane protein 31 [Source:HGNC Symbol;Acc:HGNC:28601]
ZNF554	zinc finger protein 554 [Source:HGNC Symbol;Acc:HGNC:26629]
ZNF747	zinc finger protein 747 [Source:HGNC Symbol;Acc:HGNC:28350]
CYTIP	cytohesin 1 interacting protein [Source:HGNC Symbol;Acc:HGNC:9506]
DOCK10	dedicator of cytokinesis 10 [Source:HGNC Symbol;Acc:HGNC:23479]
KCNE3	potassium voltage-gated channel subfamily E regulatory subunit 3 [Source:HGNC Symbol;Acc:HGNC:6243]
PCNX4	pecanex homolog 4 (Drosophila) [Source:HGNC Symbol;Acc:HGNC:20349]
SNPH	syntaphilin [Source:HGNC Symbol;Acc:HGNC:15931]
SOX15	SRY-box 15 [Source:HGNC Symbol;Acc:HGNC:11196]
TMEM126B	transmembrane protein 126B [Source:HGNC Symbol;Acc:HGNC:30883]
ZNF697	zinc finger protein 697 [Source:HGNC Symbol;Acc:HGNC:32034]
CFAP45	cilia and flagella associated protein 45 [Source:HGNC Symbol;Acc:HGNC:17229]
GPN1	GPN-loop GTPase 1 [Source:HGNC Symbol;Acc:HGNC:17030]
ORMDL1	ORMDL sphingolipid biosynthesis regulator 1 [Source:HGNC Symbol;Acc:HGNC:16036]
RASL10A	RAS like family 10 member A [Source:HGNC Symbol;Acc:HGNC:16954]
SPTSSB	serine palmitoyltransferase small subunit B [Source:HGNC Symbol;Acc:HGNC:24045]
STEAP2	STEAP2 metalloreductase [Source:HGNC Symbol;Acc:HGNC:17885]
ADGRG7	adhesion G protein-coupled receptor G7 [Source:HGNC Symbol;Acc:HGNC:19241]
CD70	CD70 molecule [Source:HGNC Symbol;Acc:HGNC:11937]
GP6	glycoprotein VI platelet [Source:HGNC Symbol;Acc:HGNC:14388]
MAGEB16	MAGE family member B16 [Source:HGNC Symbol;Acc:HGNC:21188]
MRI1	methylthioribose-1-phosphate isomerase 1 [Source:HGNC Symbol;Acc:HGNC:28469]
NLGN4X	neuroligin 4, X-linked [Source:HGNC Symbol;Acc:HGNC:14287]
PGLYRP3	peptidoglycan recognition protein 3 [Source:HGNC Symbol;Acc:HGNC:30014]
POM121L2	POM121 transmembrane nucleoporin like 2 [Source:HGNC Symbol;Acc:HGNC:13973]
SAMD1	sterile alpha motif domain containing 1 [Source:HGNC Symbol;Acc:HGNC:17958]
SLC11A1	solute carrier family 11 member 1 [Source:HGNC Symbol;Acc:HGNC:10907]
TMEM99	transmembrane protein 99 [Source:HGNC Symbol;Acc:HGNC:28305]
TXNDC2	thioredoxin domain containing 2 [Source:HGNC Symbol;Acc:HGNC:16470]
UROD	uroporphyrinogen decarboxylase [Source:HGNC Symbol;Acc:HGNC:12591]
ZNF816	zinc finger protein 816 [Source:HGNC Symbol;Acc:HGNC:26995]
CRYBB3	crystallin beta B3 [Source:HGNC Symbol;Acc:HGNC:2400]

FAM83A	family with sequence similarity 83 member A [Source:HGNC Symbol;Acc:HGNC:28210]
GTPBP10	GTP binding protein 10 [Source:HGNC Symbol;Acc:HGNC:25106]
RPL37	ribosomal protein L37 [Source:HGNC Symbol;Acc:HGNC:10347]
SIM1	single-minded family bHLH transcription factor 1 [Source:HGNC Symbol;Acc:HGNC:10882]
TCEAL7	transcription elongation factor A like 7 [Source:HGNC Symbol;Acc:HGNC:28336]
TMEM167A	transmembrane protein 167A [Source:HGNC Symbol;Acc:HGNC:28330]
TMEM45B	transmembrane protein 45B [Source:HGNC Symbol;Acc:HGNC:25194]
UNK	unkempt family zinc finger [Source:HGNC Symbol;Acc:HGNC:29369]
ZFP14	ZFP14 zinc finger protein [Source:HGNC Symbol;Acc:HGNC:29312]
ARMCX4	armadillo repeat containing, X-linked 4 [Source:HGNC Symbol;Acc:HGNC:28615]
ASB16	ankyrin repeat and SOCS box containing 16 [Source:HGNC Symbol;Acc:HGNC:19768]
CDS1	CDP-diacylglycerol synthase 1 [Source:HGNC Symbol;Acc:HGNC:1800]
DNAJC17	DnaJ heat shock protein family (Hsp40) member C17 [Source:HGNC Symbol;Acc:HGNC:25556]
LYPLA1	lysophospholipase I [Source:HGNC Symbol;Acc:HGNC:6737]
SLC6A1	solute carrier family 6 member 1 [Source:HGNC Symbol;Acc:HGNC:11042]
SYT15	synaptotagmin 15 [Source:HGNC Symbol;Acc:HGNC:17167]
TAF1B	TATA-box binding protein associated factor, RNA polymerase I subunit B [Source:HGNC Symbol;Acc:HGNC:11533]
C15orf65	chromosome 15 open reading frame 65 [Source:HGNC Symbol;Acc:HGNC:44654]
C1orf216	chromosome 1 open reading frame 216 [Source:HGNC Symbol;Acc:HGNC:26800]
CCL15	C-C motif chemokine ligand 15 [Source:HGNC Symbol;Acc:HGNC:10613]
KPNA7	karyopherin subunit alpha 7 [Source:HGNC Symbol;Acc:HGNC:21839]
NLRP7	NLR family pyrin domain containing 7 [Source:HGNC Symbol;Acc:HGNC:22947]
NMT2	N-myristoyltransferase 2 [Source:HGNC Symbol;Acc:HGNC:7858]
OR8B8	olfactory receptor family 8 subfamily B member 8 [Source:HGNC Symbol;Acc:HGNC:8477]
RSPH10B	radial spoke head 10 homolog B [Source:HGNC Symbol;Acc:HGNC:27362]
ZNF282	zinc finger protein 282 [Source:HGNC Symbol;Acc:HGNC:13076]
ATP8A2	ATPase phospholipid transporting 8A2 [Source:HGNC Symbol;Acc:HGNC:13533]
CD163L1	CD163 molecule like 1 [Source:HGNC Symbol;Acc:HGNC:30375]
DDX47	DEAD-box helicase 47 [Source:HGNC Symbol;Acc:HGNC:18682]
DENND1C	DENN domain containing 1C [Source:HGNC Symbol;Acc:HGNC:26225]
HOXB4	homeobox B4 [Source:HGNC Symbol;Acc:HGNC:5115]
MCTP1	multiple C2 and transmembrane domain containing 1 [Source:HGNC Symbol;Acc:HGNC:26183]
MUC20	mucin 20, cell surface associated [Source:HGNC Symbol;Acc:HGNC:23282]
PLAA	phospholipase A2 activating protein [Source:HGNC Symbol;Acc:HGNC:9043]
RBP7	retinol binding protein 7 [Source:HGNC Symbol;Acc:HGNC:30316]
SMIM3	small integral membrane protein 3 [Source:HGNC Symbol;Acc:HGNC:30248]
TMEM161B	transmembrane protein 161B [Source:HGNC Symbol;Acc:HGNC:28483]

APLF	aprataxin and PNKP like factor [Source:HGNC Symbol;Acc:HGNC:28724]
APOH	apolipoprotein H [Source:HGNC Symbol;Acc:HGNC:616]
KLC3	kinesin light chain 3 [Source:HGNC Symbol;Acc:HGNC:20717]
QSOX2	quiescin sulfhydryl oxidase 2 [Source:HGNC Symbol;Acc:HGNC:30249]
RNF139	ring finger protein 139 [Source:HGNC Symbol;Acc:HGNC:17023]
TTC38	tetratricopeptide repeat domain 38 [Source:HGNC Symbol;Acc:HGNC:26082]
U2AF1L4	U2 small nuclear RNA auxiliary factor 1-like 4 [Source:HGNC Symbol;Acc:HGNC:23020]
ZFYVE9	zinc finger FYVE-type containing 9 [Source:HGNC Symbol;Acc:HGNC:6775]
ZNF430	zinc finger protein 430 [Source:HGNC Symbol;Acc:HGNC:20808]
ZNF624	zinc finger protein 624 [Source:HGNC Symbol;Acc:HGNC:29254]
LCN1	lipocalin 1 [Source:HGNC Symbol;Acc:HGNC:6525]
TSSK6	testis specific serine kinase 6 [Source:HGNC Symbol;Acc:HGNC:30410]
ADIG	adipogenin [Source:HGNC Symbol;Acc:HGNC:28606]
GZMH	granzyme H [Source:HGNC Symbol;Acc:HGNC:4710]
ACSF2	acyl-CoA synthetase family member 2 [Source:HGNC Symbol;Acc:HGNC:26101]
HOXA3	homeobox A3 [Source:HGNC Symbol;Acc:HGNC:5104]
NIPSNAP3A	nipsnap homolog 3A [Source:HGNC Symbol;Acc:HGNC:23619]
RBM48	RNA binding motif protein 48 [Source:HGNC Symbol;Acc:HGNC:21785]
SAMD12	sterile alpha motif domain containing 12 [Source:HGNC Symbol;Acc:HGNC:31750]
SCO1	SCO1 cytochrome c oxidase assembly protein [Source:HGNC Symbol;Acc:HGNC:10603]
TRO	trophinin [Source:HGNC Symbol;Acc:HGNC:12326]
ZNF146	zinc finger protein 146 [Source:HGNC Symbol;Acc:HGNC:12931]
ANGPTL7	angiopoietin like 7 [Source:HGNC Symbol;Acc:HGNC:24078]
CRISPLD1	cysteine rich secretory protein LCCL domain containing 1 [Source:HGNC Symbol;Acc:HGNC:18206]
DLK2	delta like non-canonical Notch ligand 2 [Source:HGNC Symbol;Acc:HGNC:21113]
IGHMBP2	immunoglobulin mu binding protein 2 [Source:HGNC Symbol;Acc:HGNC:5542]
LYPD6B	LY6/PLAUR domain containing 6B [Source:HGNC Symbol;Acc:HGNC:27018]
MTMR9	myotubularin related protein 9 [Source:HGNC Symbol;Acc:HGNC:14596]
OVOL2	ovo like zinc finger 2 [Source:HGNC Symbol;Acc:HGNC:15804]
STEAP3	STEAP3 metalloreductase [Source:HGNC Symbol;Acc:HGNC:24592]
ZNF24	zinc finger protein 24 [Source:HGNC Symbol;Acc:HGNC:13032]
ANKIB1	ankyrin repeat and IBR domain containing 1 [Source:HGNC Symbol;Acc:HGNC:22215]
ASPHD1	aspartate beta-hydroxylase domain containing 1 [Source:HGNC Symbol;Acc:HGNC:27380]
CCPG1	cell cycle progression 1 [Source:HGNC Symbol;Acc:HGNC:24227]
DSG3	desmoglein 3 [Source:HGNC Symbol;Acc:HGNC:3050]
GPR119	G protein-coupled receptor 119 [Source:HGNC Symbol;Acc:HGNC:19060]
GPR135	G protein-coupled receptor 135 [Source:HGNC Symbol;Acc:HGNC:19991]
GPR21	G protein-coupled receptor 21 [Source:HGNC Symbol;Acc:HGNC:4476]
GRHL2	grainyhead like transcription factor 2 [Source:HGNC Symbol;Acc:HGNC:2799]

HRNR	hornerin [Source:HGNC Symbol;Acc:HGNC:20846]
KCNMB1	potassium calcium-activated channel subfamily M regulatory beta subunit 1 [Source:HGNC Symbol;Acc:HGNC:6285]
KRT6C	keratin 6C [Source:HGNC Symbol;Acc:HGNC:20406]
NAP1L3	nucleosome assembly protein 1 like 3 [Source:HGNC Symbol;Acc:HGNC:7639]
OSTN	osteocrin [Source:HGNC Symbol;Acc:HGNC:29961]
OVGP1	oviductal glycoprotein 1 [Source:HGNC Symbol;Acc:HGNC:8524]
TRAF3IP3	TRAF3 interacting protein 3 [Source:HGNC Symbol;Acc:HGNC:30766]
XKR9	XK related 9 [Source:HGNC Symbol;Acc:HGNC:20937]
ZXDA	zinc finger, X-linked, duplicated A [Source:HGNC Symbol;Acc:HGNC:13198]
COMMD6	COMM domain containing 6 [Source:HGNC Symbol;Acc:HGNC:24015]
ELMO3	engulfment and cell motility 3 [Source:HGNC Symbol;Acc:HGNC:17289]
HRH4	histamine receptor H4 [Source:HGNC Symbol;Acc:HGNC:17383]
PITHD1	PITH domain containing 1 [Source:HGNC Symbol;Acc:HGNC:25022]
SYDE2	synapse defective Rho GTPase homolog 2 [Source:HGNC Symbol;Acc:HGNC:25841]
TBX5	T-box 5 [Source:HGNC Symbol;Acc:HGNC:11604]
C21orf33	chromosome 21 open reading frame 33 [Source:HGNC Symbol;Acc:HGNC:1273]
FSD1	fibronectin type III and SPRY domain containing 1 [Source:HGNC Symbol;Acc:HGNC:13745]
AKAP13	A-kinase anchoring protein 13 [Source:HGNC Symbol;Acc:HGNC:371]
CA8	carbonic anhydrase 8 [Source:HGNC Symbol;Acc:HGNC:1382]
CMTM4	CKLF like MARVEL transmembrane domain containing 4 [Source:HGNC Symbol;Acc:HGNC:19175]
CNIH1	cornichon family AMPA receptor auxiliary protein 1 [Source:HGNC Symbol;Acc:HGNC:19431]
KANSL1L	KAT8 regulatory NSL complex subunit 1 like [Source:HGNC Symbol;Acc:HGNC:26310]
KRT24	keratin 24 [Source:HGNC Symbol;Acc:HGNC:18527]
MORN2	MORN repeat containing 2 [Source:HGNC Symbol;Acc:HGNC:30166]
NPSR1	neuropeptide S receptor 1 [Source:HGNC Symbol;Acc:HGNC:23631]
OVOS2	
PPIAL4A	peptidylprolyl isomerase A like 4A [Source:HGNC Symbol;Acc:HGNC:24369]
PROP1	PROP paired-like homeobox 1 [Source:HGNC Symbol;Acc:HGNC:9455]
RBM7	RNA binding motif protein 7 [Source:HGNC Symbol;Acc:HGNC:9904]
SPAG6	sperm associated antigen 6 [Source:HGNC Symbol;Acc:HGNC:11215]
SYTL1	synaptotagmin like 1 [Source:HGNC Symbol;Acc:HGNC:15584]
TAPBPL	TAP binding protein like [Source:HGNC Symbol;Acc:HGNC:30683]
TMEM156	transmembrane protein 156 [Source:HGNC Symbol;Acc:HGNC:26260]
TMEM87A	transmembrane protein 87A [Source:HGNC Symbol;Acc:HGNC:24522]
TMIGD1	transmembrane and immunoglobulin domain containing 1 [Source:HGNC Symbol;Acc:HGNC:32431]
UPK2	uroplakin 2 [Source:HGNC Symbol;Acc:HGNC:12579]
GAS7	growth arrest specific 7 [Source:HGNC Symbol;Acc:HGNC:4169]

KDM3A	lysine demethylase 3A [Source:HGNC Symbol;Acc:HGNC:20815]
LETM2	leucine zipper and EF-hand containing transmembrane protein 2 [Source:HGNC Symbol;Acc:HGNC:14648]
MMP28	matrix metallopeptidase 28 [Source:HGNC Symbol;Acc:HGNC:14366]
PCTP	phosphatidylcholine transfer protein [Source:HGNC Symbol;Acc:HGNC:8752]
PLEKHA7	pleckstrin homology domain containing A7 [Source:HGNC Symbol;Acc:HGNC:27049]
ADGB	androglobin [Source:HGNC Symbol;Acc:HGNC:21212]
AK6	adenylate kinase 6 [Source:HGNC Symbol;Acc:HGNC:49151]
C1QL2	complement component 1, q subcomponent-like 2 [Source:HGNC Symbol;Acc:HGNC:24181]
CCP110	centriolar coiled-coil protein 110 [Source:HGNC Symbol;Acc:HGNC:24342]
UBE2U	ubiquitin conjugating enzyme E2 U (putative) [Source:HGNC Symbol;Acc:HGNC:28559]
ABCC11	ATP binding cassette subfamily C member 11 [Source:HGNC Symbol;Acc:HGNC:14639]
ADAMTS3	ADAM metallopeptidase with thrombospondin type 1 motif 3 [Source:HGNC Symbol;Acc:HGNC:219]
AZI2	5-azacytidine induced 2 [Source:HGNC Symbol;Acc:HGNC:24002]
C11orf80	chromosome 11 open reading frame 80 [Source:HGNC Symbol;Acc:HGNC:26197]
CCDC170	coiled-coil domain containing 170 [Source:HGNC Symbol;Acc:HGNC:21177]
DCD	dermcidin [Source:HGNC Symbol;Acc:HGNC:14669]
FGD3	FYVE, RhoGEF and PH domain containing 3 [Source:HGNC Symbol;Acc:HGNC:16027]
GPR26	G protein-coupled receptor 26 [Source:HGNC Symbol;Acc:HGNC:4481]
OCM	oncomodulin [Source:HGNC Symbol;Acc:HGNC:8105]
PALM	paralemmin [Source:HGNC Symbol;Acc:HGNC:8594]
RBAK	RB associated KRAB zinc finger [Source:HGNC Symbol;Acc:HGNC:17680]
RGS1	regulator of G-protein signaling 1 [Source:HGNC Symbol;Acc:HGNC:9991]
SEC14L4	SEC14 like lipid binding 4 [Source:HGNC Symbol;Acc:HGNC:20627]
SYT3	synaptotagmin 3 [Source:HGNC Symbol;Acc:HGNC:11511]
TCF24	transcription factor 24 [Source:HGNC Symbol;Acc:HGNC:32275]
TIGD5	tigger transposable element derived 5 [Source:HGNC Symbol;Acc:HGNC:18336]
ZFYVE28	zinc finger FYVE-type containing 28 [Source:HGNC Symbol;Acc:HGNC:29334]
ZNF107	zinc finger protein 107 [Source:HGNC Symbol;Acc:HGNC:12887]
ZNF831	zinc finger protein 831 [Source:HGNC Symbol;Acc:HGNC:16167]
APBA2	amyloid beta precursor protein binding family A member 2 [Source:HGNC Symbol;Acc:HGNC:579]
ARHGAP8	Rho GTPase activating protein 8 [Source:HGNC Symbol;Acc:HGNC:677]
ATAD1	ATPase family, AAA domain containing 1 [Source:HGNC Symbol;Acc:HGNC:25903]
CRB3	crumbs 3, cell polarity complex component [Source:HGNC Symbol;Acc:HGNC:20237]
IREB2	iron responsive element binding protein 2 [Source:HGNC Symbol;Acc:HGNC:6115]
IRF8	interferon regulatory factor 8 [Source:HGNC Symbol;Acc:HGNC:5358]
MAGIX	MAGI family member, X-linked [Source:HGNC Symbol;Acc:HGNC:30006]

POMGNT2	protein O-linked mannose N-acetylglucosaminyltransferase 2 (beta 1,4-) [Source:HGNC Symbol;Acc:HGNC:25902]
SHTN1	shootin 1 [Source:HGNC Symbol;Acc:HGNC:29319]
SLC23A2	solute carrier family 23 member 2 [Source:HGNC Symbol;Acc:HGNC:10973]
TMEM139	transmembrane protein 139 [Source:HGNC Symbol;Acc:HGNC:22058]
TTLL3	tubulin tyrosine ligase like 3 [Source:HGNC Symbol;Acc:HGNC:24483]
ADAMTS12	ADAM metallopeptidase with thrombospondin type 1 motif 12 [Source:HGNC Symbol;Acc:HGNC:14605]
ADCK2	aarF domain containing kinase 2 [Source:HGNC Symbol;Acc:HGNC:19039]
ANKRD2	ankyrin repeat domain 2 [Source:HGNC Symbol;Acc:HGNC:495]
APOL6	apolipoprotein L6 [Source:HGNC Symbol;Acc:HGNC:14870]
BTN2A1	butyrophilin subfamily 2 member A1 [Source:HGNC Symbol;Acc:HGNC:1136]
CA14	carbonic anhydrase 14 [Source:HGNC Symbol;Acc:HGNC:1372]
CD1A	CD1a molecule [Source:HGNC Symbol;Acc:HGNC:1634]
CIB3	calcium and integrin binding family member 3 [Source:HGNC Symbol;Acc:HGNC:24580]
CLDN15	claudin 15 [Source:HGNC Symbol;Acc:HGNC:2036]
LRIF1	ligand dependent nuclear receptor interacting factor 1 [Source:HGNC Symbol;Acc:HGNC:30299]
MACC1	metastasis associated in colon cancer 1 [Source:HGNC Symbol;Acc:HGNC:30215]
MUL1	mitochondrial E3 ubiquitin protein ligase 1 [Source:HGNC Symbol;Acc:HGNC:25762]
NMNAT3	nicotinamide nucleotide adenylyltransferase 3 [Source:HGNC Symbol;Acc:HGNC:20989]
NOL9	nucleolar protein 9 [Source:HGNC Symbol;Acc:HGNC:26265]
NXPE2	neurexophilin and PC-esterase domain family member 2 [Source:HGNC Symbol;Acc:HGNC:26331]
RPP38	ribonuclease P/MRP subunit p38 [Source:HGNC Symbol;Acc:HGNC:30329]
SLC30A8	solute carrier family 30 member 8 [Source:HGNC Symbol;Acc:HGNC:20303]
SLITRK5	SLIT and NTRK like family member 5 [Source:HGNC Symbol;Acc:HGNC:20295]
SPRR1B	small proline rich protein 1B [Source:HGNC Symbol;Acc:HGNC:11260]
TM2D1	TM2 domain containing 1 [Source:HGNC Symbol;Acc:HGNC:24142]
TMEM125	transmembrane protein 125 [Source:HGNC Symbol;Acc:HGNC:28275]
VARS	valyl-tRNA synthetase [Source:HGNC Symbol;Acc:HGNC:12651]
ZNF114	zinc finger protein 114 [Source:HGNC Symbol;Acc:HGNC:12894]
ZNF286A	zinc finger protein 286A [Source:HGNC Symbol;Acc:HGNC:13501]
ZNF644	zinc finger protein 644 [Source:HGNC Symbol;Acc:HGNC:29222]
GLOD5	glyoxalase domain containing 5 [Source:HGNC Symbol;Acc:HGNC:33358]
IL17C	interleukin 17C [Source:HGNC Symbol;Acc:HGNC:5983]
KRT76	keratin 76 [Source:HGNC Symbol;Acc:HGNC:24430]
ARHGEF5	Rho guanine nucleotide exchange factor 5 [Source:HGNC Symbol;Acc:HGNC:13209]
GRID1	glutamate ionotropic receptor delta type subunit 1 [Source:HGNC Symbol;Acc:HGNC:4575]
LRRC1	leucine rich repeat containing 1 [Source:HGNC Symbol;Acc:HGNC:14307]

MRAP2	melanocortin 2 receptor accessory protein 2 [Source:HGNC Symbol;Acc:HGNC:21232]
SRCAP	Snf2-related CREBBP activator protein [Source:HGNC Symbol;Acc:HGNC:16974]
SVIP	small VCP/p97-interacting protein [Source:HGNC Symbol;Acc:HGNC:25238]
TRIM29	tripartite motif containing 29 [Source:HGNC Symbol;Acc:HGNC:17274]
ABHD17B	abhydrolase domain containing 17B [Source:HGNC Symbol;Acc:HGNC:24278]
ACSBG1	acyl-CoA synthetase bubblegum family member 1 [Source:HGNC Symbol;Acc:HGNC:29567]
B3GNT7	UDP-GlcNAc:betaGal beta-1,3-N-acetylglucosaminyltransferase 7 [Source:HGNC Symbol;Acc:HGNC:18811]
CAAP1	caspase activity and apoptosis inhibitor 1 [Source:HGNC Symbol;Acc:HGNC:25834]
FAM72D	family with sequence similarity 72 member D [Source:HGNC Symbol;Acc:HGNC:33593]
GABBR2	gamma-aminobutyric acid type B receptor subunit 2 [Source:HGNC Symbol;Acc:HGNC:4507]
IL1RAPL2	interleukin 1 receptor accessory protein like 2 [Source:HGNC Symbol;Acc:HGNC:5997]
MAML2	mastermind like transcriptional coactivator 2 [Source:HGNC Symbol;Acc:HGNC:16259]
NMD3	NMD3 ribosome export adaptor [Source:HGNC Symbol;Acc:HGNC:24250]
NPIPB3	nuclear pore complex interacting protein family member B3 [Source:HGNC Symbol;Acc:HGNC:28989]
NXPE3	neurexophilin and PC-esterase domain family member 3 [Source:HGNC Symbol;Acc:HGNC:28238]
PFKFB4	6-phosphofructo-2-kinase/fructose-2,6-biphosphatase 4 [Source:HGNC Symbol;Acc:HGNC:8875]
PLET1	placenta expressed transcript 1 [Source:HGNC Symbol;Acc:HGNC:30053]
PNMA2	paraneoplastic Ma antigen 2 [Source:HGNC Symbol;Acc:HGNC:9159]
PPP1R27	protein phosphatase 1 regulatory subunit 27 [Source:HGNC Symbol;Acc:HGNC:16813]
R3HCC1L	R3H domain and coiled-coil containing 1 like [Source:HGNC Symbol;Acc:HGNC:23512]
SLC34A2	solute carrier family 34 member 2 [Source:HGNC Symbol;Acc:HGNC:11020]
SLC35A3	solute carrier family 35 member A3 [Source:HGNC Symbol;Acc:HGNC:11023]
ZNF443	zinc finger protein 443 [Source:HGNC Symbol;Acc:HGNC:20878]
ZNF552	zinc finger protein 552 [Source:HGNC Symbol;Acc:HGNC:26135]
ZSWIM3	zinc finger SWIM-type containing 3 [Source:HGNC Symbol;Acc:HGNC:16157]
ANKRD37	ankyrin repeat domain 37 [Source:HGNC Symbol;Acc:HGNC:29593]
DOCK2	dedicator of cytokinesis 2 [Source:HGNC Symbol;Acc:HGNC:2988]
FZD10	frizzled class receptor 10 [Source:HGNC Symbol;Acc:HGNC:4039]
GPR87	G protein-coupled receptor 87 [Source:HGNC Symbol;Acc:HGNC:4538]
HOXA9	homeobox A9 [Source:HGNC Symbol;Acc:HGNC:5109]
IAH1	isoamyl acetate-hydrolyzing esterase 1 homolog [Source:HGNC Symbol;Acc:HGNC:27696]
KCTD14	potassium channel tetramerization domain containing 14 [Source:HGNC Symbol;Acc:HGNC:23295]
MUC13	mucin 13, cell surface associated [Source:HGNC Symbol;Acc:HGNC:7511]
PALB2	partner and localizer of BRCA2 [Source:HGNC Symbol;Acc:HGNC:26144]

PAM	peptidylglycine alpha-amidating monooxygenase [Source:HGNC Symbol;Acc:HGNC:8596]
SAMD11	sterile alpha motif domain containing 11 [Source:HGNC Symbol;Acc:HGNC:28706]
SPECC1	sperm antigen with calponin homology and coiled-coil domains 1 [Source:HGNC Symbol;Acc:HGNC:30615]
TRIM8	tripartite motif containing 8 [Source:HGNC Symbol;Acc:HGNC:15579]
TRIP4	thyroid hormone receptor interactor 4 [Source:HGNC Symbol;Acc:HGNC:12310]
WIPI1	WD repeat domain, phosphoinositide interacting 1 [Source:HGNC Symbol;Acc:HGNC:25471]
YAF2	YY1 associated factor 2 [Source:HGNC Symbol;Acc:HGNC:17363]
IFNA2	interferon, alpha 2 [Source:HGNC Symbol;Acc:HGNC:5423]
NOX3	NADPH oxidase 3 [Source:HGNC Symbol;Acc:HGNC:7890]
INTU	inturned planar cell polarity protein [Source:HGNC Symbol;Acc:HGNC:29239]
LRRC40	leucine rich repeat containing 40 [Source:HGNC Symbol;Acc:HGNC:26004]
MAP7D2	MAP7 domain containing 2 [Source:HGNC Symbol;Acc:HGNC:25899]
OVOL1	ovo like transcriptional repressor 1 [Source:HGNC Symbol;Acc:HGNC:8525]
PAOX	polyamine oxidase (exo-N4-amino) [Source:HGNC Symbol;Acc:HGNC:20837]
PAX5	paired box 5 [Source:HGNC Symbol;Acc:HGNC:8619]
RABL3	RAB, member of RAS oncogene family like 3 [Source:HGNC Symbol;Acc:HGNC:18072]
SCML1	sex comb on midleg-like 1 (Drosophila) [Source:HGNC Symbol;Acc:HGNC:10580]
ALAS2	5'-aminolevulinate synthase 2 [Source:HGNC Symbol;Acc:HGNC:397]
CHRNA9	cholinergic receptor nicotinic alpha 9 subunit [Source:HGNC Symbol;Acc:HGNC:14079]
DZANK1	double zinc ribbon and ankyrin repeat domains 1 [Source:HGNC Symbol;Acc:HGNC:15858]
ESRP2	epithelial splicing regulatory protein 2 [Source:HGNC Symbol;Acc:HGNC:26152]
FAM167A	family with sequence similarity 167 member A [Source:HGNC Symbol;Acc:HGNC:15549]
HOOK1	hook microtubule-tethering protein 1 [Source:HGNC Symbol;Acc:HGNC:19884]
IBTK	inhibitor of Bruton tyrosine kinase [Source:HGNC Symbol;Acc:HGNC:17853]
LPAR1	lysophosphatidic acid receptor 1 [Source:HGNC Symbol;Acc:HGNC:3166]
OMP	olfactory marker protein [Source:HGNC Symbol;Acc:HGNC:8136]
PORCN	porcupine homolog (Drosophila) [Source:HGNC Symbol;Acc:HGNC:17652]
TAC3	tachykinin 3 [Source:HGNC Symbol;Acc:HGNC:11521]
TCN1	transcobalamin 1 [Source:HGNC Symbol;Acc:HGNC:11652]
TP53I11	tumor protein p53 inducible protein 11 [Source:HGNC Symbol;Acc:HGNC:16842]
TP53I13	tumor protein p53 inducible protein 13 [Source:HGNC Symbol;Acc:HGNC:25102]
VPS53	VPS53, GARP complex subunit [Source:HGNC Symbol;Acc:HGNC:25608]
ZNF256	zinc finger protein 256 [Source:HGNC Symbol;Acc:HGNC:13049]
ZP1	zona pellucida glycoprotein 1 [Source:HGNC Symbol;Acc:HGNC:13187]
ANKRD18A	ankyrin repeat domain 18A [Source:HGNC Symbol;Acc:HGNC:23643]
QARS	glutaminyl-tRNA synthetase [Source:HGNC Symbol;Acc:HGNC:9751]

VSIG2	V-set and immunoglobulin domain containing 2 [Source:HGNC Symbol;Acc:HGNC:17149]
YARS	tyrosyl-tRNA synthetase [Source:HGNC Symbol;Acc:HGNC:12840]
BICDL1	BICD family like cargo adaptor 1 [Source:HGNC Symbol;Acc:HGNC:28095]
FABP2	fatty acid binding protein 2 [Source:HGNC Symbol;Acc:HGNC:3556]
HCLS1	hematopoietic cell-specific Lyn substrate 1 [Source:HGNC Symbol;Acc:HGNC:4844]
LRRN1	leucine rich repeat neuronal 1 [Source:HGNC Symbol;Acc:HGNC:20980]
LSM11	LSM11, U7 small nuclear RNA associated [Source:HGNC Symbol;Acc:HGNC:30860]
MARVELD2	MARVEL domain containing 2 [Source:HGNC Symbol;Acc:HGNC:26401]
PPM1L	protein phosphatase, Mg2+/Mn2+ dependent 1L [Source:HGNC Symbol;Acc:HGNC:16381]
PRSS1	protease, serine 1 [Source:HGNC Symbol;Acc:HGNC:9475]
RASSF8	Ras association domain family member 8 [Source:HGNC Symbol;Acc:HGNC:13232]
TMEM209	transmembrane protein 209 [Source:HGNC Symbol;Acc:HGNC:21898]
TRPV3	transient receptor potential cation channel subfamily V member 3 [Source:HGNC Symbol;Acc:HGNC:18084]
USP31	ubiquitin specific peptidase 31 [Source:HGNC Symbol;Acc:HGNC:20060]
AATK	apoptosis associated tyrosine kinase [Source:HGNC Symbol;Acc:HGNC:21]
ARHGAP28	Rho GTPase activating protein 28 [Source:HGNC Symbol;Acc:HGNC:25509]
CDO1	cysteine dioxygenase type 1 [Source:HGNC Symbol;Acc:HGNC:1795]
CLIC6	chloride intracellular channel 6 [Source:HGNC Symbol;Acc:HGNC:2065]
FAM89B	family with sequence similarity 89 member B [Source:HGNC Symbol;Acc:HGNC:16708]
FMN2	formin 2 [Source:HGNC Symbol;Acc:HGNC:14074]
HOXB2	homeobox B2 [Source:HGNC Symbol;Acc:HGNC:5113]
MFSD6	major facilitator superfamily domain containing 6 [Source:HGNC Symbol;Acc:HGNC:24711]
MCAT	malonyl-CoA-acyl carrier protein transacylase [Source:HGNC Symbol;Acc:HGNC:29622]
PUS7	pseudouridylate synthase 7 (putative) [Source:HGNC Symbol;Acc:HGNC:26033]
RBM4	RNA binding motif protein 4 [Source:HGNC Symbol;Acc:HGNC:9901]
RNF24	ring finger protein 24 [Source:HGNC Symbol;Acc:HGNC:13779]
ST6GALNAC5	ST6 N-acetylgalactosaminide alpha-2,6-sialyltransferase 5 [Source:HGNC Symbol;Acc:HGNC:19342]
A4GALT	alpha 1,4-galactosyltransferase [Source:HGNC Symbol;Acc:HGNC:18149]
MMGT1	membrane magnesium transporter 1 [Source:HGNC Symbol;Acc:HGNC:28100]
SPOCK2	sparc/osteonectin, cwcv and kazal-like domains proteoglycan (testican) 2 [Source:HGNC Symbol;Acc:HGNC:13564]
NKX1-2	NK1 homeobox 2 [Source:HGNC Symbol;Acc:HGNC:31652]
B4GALNT1	beta-1,4-N-acetyl-galactosaminyltransferase 1 [Source:HGNC Symbol;Acc:HGNC:4117]
BMX	BMX non-receptor tyrosine kinase [Source:HGNC Symbol;Acc:HGNC:1079]
CNEP1R1	CTD nuclear envelope phosphatase 1 regulatory subunit 1 [Source:HGNC Symbol;Acc:HGNC:26759]

DCAF8	DDB1 and CUL4 associated factor 8 [Source:HGNC Symbol;Acc:HGNC:24891]
HCFC1R1	host cell factor C1 regulator 1 [Source:HGNC Symbol;Acc:HGNC:21198]
NABP2	nucleic acid binding protein 2 [Source:HGNC Symbol;Acc:HGNC:28412]
NHLRC2	NHL repeat containing 2 [Source:HGNC Symbol;Acc:HGNC:24731]
SDHAF2	succinate dehydrogenase complex assembly factor 2 [Source:HGNC Symbol;Acc:HGNC:26034]
SH3YL1	SH3 and SYLF domain containing 1 [Source:HGNC Symbol;Acc:HGNC:29546]
SLAMF8	SLAM family member 8 [Source:HGNC Symbol;Acc:HGNC:21391]
SNAPC2	small nuclear RNA activating complex polypeptide 2 [Source:HGNC Symbol;Acc:HGNC:11135]
TBX4	T-box 4 [Source:HGNC Symbol;Acc:HGNC:11603]
TDRD3	tudor domain containing 3 [Source:HGNC Symbol;Acc:HGNC:20612]
VPS45	vacuolar protein sorting 45 homolog [Source:HGNC Symbol;Acc:HGNC:14579]
ABCA9	ATP binding cassette subfamily A member 9 [Source:HGNC Symbol;Acc:HGNC:39]
ATP6V1C2	ATPase H+ transporting V1 subunit C2 [Source:HGNC Symbol;Acc:HGNC:18264]
DISC1	disrupted in schizophrenia 1 [Source:HGNC Symbol;Acc:HGNC:2888]
EPB41L4A	erythrocyte membrane protein band 4.1 like 4A [Source:HGNC Symbol;Acc:HGNC:13278]
FOXN1	forkhead box N1 [Source:HGNC Symbol;Acc:HGNC:12765]
HPR	haptoglobin-related protein [Source:HGNC Symbol;Acc:HGNC:5156]
KRIT1	KRIT1, ankyrin repeat containing [Source:HGNC Symbol;Acc:HGNC:1573]
LGALS7	galectin 7 [Source:HGNC Symbol;Acc:HGNC:6568]
MED25	mediator complex subunit 25 [Source:HGNC Symbol;Acc:HGNC:28845]
RBKS	ribokinase [Source:HGNC Symbol;Acc:HGNC:30325]
RPUSD1	RNA pseudouridylate synthase domain containing 1 [Source:HGNC Symbol;Acc:HGNC:14173]
SNCAIP	synuclein alpha interacting protein [Source:HGNC Symbol;Acc:HGNC:11139]
SNX11	sorting nexin 11 [Source:HGNC Symbol;Acc:HGNC:14975]
SYT17	synaptotagmin 17 [Source:HGNC Symbol;Acc:HGNC:24119]
VKORC1L1	vitamin K epoxide reductase complex subunit 1 like 1 [Source:HGNC Symbol;Acc:HGNC:21492]
ATP5G2	ATP synthase, H+ transporting, mitochondrial Fo complex subunit C2 (subunit 9) [Source:HGNC Symbol;Acc:HGNC:842]
C2orf69	chromosome 2 open reading frame 69 [Source:HGNC Symbol;Acc:HGNC:26799]
STRIP1	striatin interacting protein 1 [Source:HGNC Symbol;Acc:HGNC:25916]
TBATA	thymus, brain and testes associated [Source:HGNC Symbol;Acc:HGNC:23511]
WARS	tryptophanyl-tRNA synthetase [Source:HGNC Symbol;Acc:HGNC:12729]
ESRP1	epithelial splicing regulatory protein 1 [Source:HGNC Symbol;Acc:HGNC:25966]
GJA3	gap junction protein alpha 3 [Source:HGNC Symbol;Acc:HGNC:4277]
ITPRIPL2	inositol 1,4,5-trisphosphate receptor interacting protein-like 2 [Source:HGNC Symbol;Acc:HGNC:27257]
KCNE2	potassium voltage-gated channel subfamily E regulatory subunit 2 [Source:HGNC Symbol;Acc:HGNC:6242]

MYEF2	myelin expression factor 2 [Source:HGNC Symbol;Acc:HGNC:17940]
MYO9A	myosin IXA [Source:HGNC Symbol;Acc:HGNC:7608]
OTUD4	OTU deubiquitinase 4 [Source:HGNC Symbol;Acc:HGNC:24949]
RHOD	ras homolog family member D [Source:HGNC Symbol;Acc:HGNC:670]
SMG1	SMG1, nonsense mediated mRNA decay associated PI3K related kinase [Source:HGNC Symbol;Acc:HGNC:30045]
TMEM204	transmembrane protein 204 [Source:HGNC Symbol;Acc:HGNC:14158]
ACSS3	acyl-CoA synthetase short-chain family member 3 [Source:HGNC Symbol;Acc:HGNC:24723]
AMELX	amelogenin, X-linked [Source:HGNC Symbol;Acc:HGNC:461]
ATXN7L3B	ataxin 7 like 3B [Source:HGNC Symbol;Acc:HGNC:37931]
BTBD6	BTB domain containing 6 [Source:HGNC Symbol;Acc:HGNC:19897]
CDC14A	cell division cycle 14A [Source:HGNC Symbol;Acc:HGNC:1718]
CERCAM	cerebral endothelial cell adhesion molecule [Source:HGNC Symbol;Acc:HGNC:23723]
CHST2	carbohydrate sulfotransferase 2 [Source:HGNC Symbol;Acc:HGNC:1970]
CLDN23	claudin 23 [Source:HGNC Symbol;Acc:HGNC:17591]
DIP2C	disco interacting protein 2 homolog C [Source:HGNC Symbol;Acc:HGNC:29150]
EPHX4	epoxide hydrolase 4 [Source:HGNC Symbol;Acc:HGNC:23758]
INPP5K	inositol polyphosphate-5-phosphatase K [Source:HGNC Symbol;Acc:HGNC:33882]
NDUFAF7	NADH:ubiquinone oxidoreductase complex assembly factor 7 [Source:HGNC Symbol;Acc:HGNC:28816]
NKD2	naked cuticle homolog 2 [Source:HGNC Symbol;Acc:HGNC:17046]
PCLO	piccolo presynaptic cytomatrix protein [Source:HGNC Symbol;Acc:HGNC:13406]
PPTC7	PTC7 protein phosphatase homolog [Source:HGNC Symbol;Acc:HGNC:30695]
RAB3IP	RAB3A interacting protein [Source:HGNC Symbol;Acc:HGNC:16508]
RNF208	ring finger protein 208 [Source:HGNC Symbol;Acc:HGNC:25420]
SUSD3	sushi domain containing 3 [Source:HGNC Symbol;Acc:HGNC:28391]
TADA3	transcriptional adaptor 3 [Source:HGNC Symbol;Acc:HGNC:19422]
TREML1	triggering receptor expressed on myeloid cells like 1 [Source:HGNC Symbol;Acc:HGNC:20434]
VAMP4	vesicle associated membrane protein 4 [Source:HGNC Symbol;Acc:HGNC:12645]
ABCA8	ATP binding cassette subfamily A member 8 [Source:HGNC Symbol;Acc:HGNC:38]
ARHGEF16	Rho guanine nucleotide exchange factor 16 [Source:HGNC Symbol;Acc:HGNC:15515]
ARL5A	ADP ribosylation factor like GTPase 5A [Source:HGNC Symbol;Acc:HGNC:696]
ARMCX2	armadillo repeat containing, X-linked 2 [Source:HGNC Symbol;Acc:HGNC:16869]
BCCIP	BRCA2 and CDKN1A interacting protein [Source:HGNC Symbol;Acc:HGNC:978]
BICD1	BICD cargo adaptor 1 [Source:HGNC Symbol;Acc:HGNC:1049]
C1GALT1	core 1 synthase, glycoprotein-N-acetylgalactosamine 3-beta-galactosyltransferase 1 [Source:HGNC Symbol;Acc:HGNC:24337]
CELSR1	cadherin EGF LAG seven-pass G-type receptor 1 [Source:HGNC Symbol;Acc:HGNC:1850]
CHMP4C	charged multivesicular body protein 4C [Source:HGNC Symbol;Acc:HGNC:30599]
EIF5A2	eukaryotic translation initiation factor 5A2 [Source:HGNC Symbol;Acc:HGNC:3301]

GJB3	gap junction protein beta 3 [Source:HGNC Symbol;Acc:HGNC:4285]
HDDC3	HD domain containing 3 [Source:HGNC Symbol;Acc:HGNC:30522]
JPH1	junctophilin 1 [Source:HGNC Symbol;Acc:HGNC:14201]
KCNJ16	potassium voltage-gated channel subfamily J member 16 [Source:HGNC Symbol;Acc:HGNC:6262]
LLGL2	LLGL2, scribble cell polarity complex component [Source:HGNC Symbol;Acc:HGNC:6629]
METRN	meteorin, glial cell differentiation regulator [Source:HGNC Symbol;Acc:HGNC:14151]
NECTIN4	nectin cell adhesion molecule 4 [Source:HGNC Symbol;Acc:HGNC:19688]
PAK6	p21 (RAC1) activated kinase 6 [Source:HGNC Symbol;Acc:HGNC:16061]
PDCD6	programmed cell death 6 [Source:HGNC Symbol;Acc:HGNC:8765]
PHF6	PHD finger protein 6 [Source:HGNC Symbol;Acc:HGNC:18145]
PPP2R3A	protein phosphatase 2 regulatory subunit B''alpha [Source:HGNC Symbol;Acc:HGNC:9307]
PSTPIP2	proline-serine-threonine phosphatase interacting protein 2 [Source:HGNC Symbol;Acc:HGNC:9581]
RASEF	RAS and EF-hand domain containing [Source:HGNC Symbol;Acc:HGNC:26464]
RNASE4	ribonuclease A family member 4 [Source:HGNC Symbol;Acc:HGNC:10047]
SBSPON	somatomedin B and thrombospondin type 1 domain containing [Source:HGNC Symbol;Acc:HGNC:30362]
TFB2M	transcription factor B2, mitochondrial [Source:HGNC Symbol;Acc:HGNC:18559]
RAB9A	RAB9A, member RAS oncogene family [Source:HGNC Symbol;Acc:HGNC:9792]
TMEM181	transmembrane protein 181 [Source:HGNC Symbol;Acc:HGNC:20958]
ZKSCAN2	zinc finger with KRAB and SCAN domains 2 [Source:HGNC Symbol;Acc:HGNC:25677]
ACTR1B	ARP1 actin-related protein 1 homolog B, centractin beta [Source:HGNC Symbol;Acc:HGNC:168]
ADGRE2	adhesion G protein-coupled receptor E2 [Source:HGNC Symbol;Acc:HGNC:3337]
ALPP	alkaline phosphatase, placental [Source:HGNC Symbol;Acc:HGNC:439]
CCDC97	coiled-coil domain containing 97 [Source:HGNC Symbol;Acc:HGNC:28289]
CREB3L1	cAMP responsive element binding protein 3 like 1 [Source:HGNC Symbol;Acc:HGNC:18856]
DGKG	diacylglycerol kinase gamma [Source:HGNC Symbol;Acc:HGNC:2853]
EREG	epiregulin [Source:HGNC Symbol;Acc:HGNC:3443]
FBRS	fibrosin [Source:HGNC Symbol;Acc:HGNC:20442]
GALNT12	polypeptide N-acetylgalactosaminyltransferase 12 [Source:HGNC Symbol;Acc:HGNC:19877]
HES3	hes family bHLH transcription factor 3 [Source:HGNC Symbol;Acc:HGNC:26226]
HES7	hes family bHLH transcription factor 7 [Source:HGNC Symbol;Acc:HGNC:15977]
LRP11	LDL receptor related protein 11 [Source:HGNC Symbol;Acc:HGNC:16936]
MAN1A1	mannosidase alpha class 1A member 1 [Source:HGNC Symbol;Acc:HGNC:6821]
PLEKHG3	pleckstrin homology and RhoGEF domain containing G3 [Source:HGNC Symbol;Acc:HGNC:20364]
RAB42	RAB42, member RAS oncogene family [Source:HGNC Symbol;Acc:HGNC:28702]

RASAL3	RAS protein activator like 3 [Source:HGNC Symbol;Acc:HGNC:26129]
RTF1	RTF1 homolog, Paf1/RNA polymerase II complex component [Source:HGNC Symbol;Acc:HGNC:28996]
SLC35D3	solute carrier family 35 member D3 [Source:HGNC Symbol;Acc:HGNC:15621]
THAP3	THAP domain containing 3 [Source:HGNC Symbol;Acc:HGNC:20855]
TOP3B	topoisomerase (DNA) III beta [Source:HGNC Symbol;Acc:HGNC:11993]
TOX	thymocyte selection associated high mobility group box [Source:HGNC Symbol;Acc:HGNC:18988]
TRIM17	tripartite motif containing 17 [Source:HGNC Symbol;Acc:HGNC:13430]
ZBTB1	zinc finger and BTB domain containing 1 [Source:HGNC Symbol;Acc:HGNC:20259]
BATF	basic leucine zipper ATF-like transcription factor [Source:HGNC Symbol;Acc:HGNC:958]
DYNC1I1	dynein cytoplasmic 1 intermediate chain 1 [Source:HGNC Symbol;Acc:HGNC:2963]
ENPP5	ectonucleotide pyrophosphatase/phosphodiesterase 5 (putative) [Source:HGNC Symbol;Acc:HGNC:13717]
GALNT15	polypeptide N-acetylgalactosaminyltransferase 15 [Source:HGNC Symbol;Acc:HGNC:21531]
GPBP1	GC-rich promoter binding protein 1 [Source:HGNC Symbol;Acc:HGNC:29520]
PLXNB1	plexin B1 [Source:HGNC Symbol;Acc:HGNC:9103]
VGLL4	vestigial like family member 4 [Source:HGNC Symbol;Acc:HGNC:28966]
ZCCHC24	zinc finger CCHC-type containing 24 [Source:HGNC Symbol;Acc:HGNC:26911]
RAD9B	RAD9 checkpoint clamp component B [Source:HGNC Symbol;Acc:HGNC:21700]
TUBGCP4	tubulin gamma complex associated protein 4 [Source:HGNC Symbol;Acc:HGNC:16691]
AMY2A	amylase, alpha 2A (pancreatic) [Source:HGNC Symbol;Acc:HGNC:477]
C1QL3	complement component 1, q subcomponent-like 3 [Source:HGNC Symbol;Acc:HGNC:19359]
C15orf57	chromosome 15 open reading frame 57 [Source:HGNC Symbol;Acc:HGNC:28295]
MB21D1	Mab-21 domain containing 1 [Source:HGNC Symbol;Acc:HGNC:21367]
EHF	ETS homologous factor [Source:HGNC Symbol;Acc:HGNC:3246]
FSTL5	follistatin like 5 [Source:HGNC Symbol;Acc:HGNC:21386]
HSH2D	hematopoietic SH2 domain containing [Source:HGNC Symbol;Acc:HGNC:24920]
IL1RL2	interleukin 1 receptor like 2 [Source:HGNC Symbol;Acc:HGNC:5999]
KCNK4	potassium two pore domain channel subfamily K member 4 [Source:HGNC Symbol;Acc:HGNC:6279]
KLK6	kallikrein related peptidase 6 [Source:HGNC Symbol;Acc:HGNC:6367]
NEGR1	neuronal growth regulator 1 [Source:HGNC Symbol;Acc:HGNC:17302]
NPIPA1	nuclear pore complex interacting protein family member A1 [Source:HGNC Symbol;Acc:HGNC:7909]
SLC12A2	solute carrier family 12 member 2 [Source:HGNC Symbol;Acc:HGNC:10911]
STON2	stonin 2 [Source:HGNC Symbol;Acc:HGNC:30652]
SULT6B1	sulfotransferase family 6B member 1 [Source:HGNC Symbol;Acc:HGNC:33433]
TC2N	tandem C2 domains, nuclear [Source:HGNC Symbol;Acc:HGNC:19859]

TMTC3	transmembrane and tetratricopeptide repeat containing 3 [Source:HGNC Symbol;Acc:HGNC:26899]
ZRANB2	zinc finger RANBP2-type containing 2 [Source:HGNC Symbol;Acc:HGNC:13058]
ATMIN	ATM interactor [Source:HGNC Symbol;Acc:HGNC:29034]
DYRK3	dual specificity tyrosine phosphorylation regulated kinase 3 [Source:HGNC Symbol;Acc:HGNC:3094]
EPB41L4B	erythrocyte membrane protein band 4.1 like 4B [Source:HGNC Symbol;Acc:HGNC:19818]
GIPR	gastric inhibitory polypeptide receptor [Source:HGNC Symbol;Acc:HGNC:4271]
HECTD3	HECT domain E3 ubiquitin protein ligase 3 [Source:HGNC Symbol;Acc:HGNC:26117]
IL20RB	interleukin 20 receptor subunit beta [Source:HGNC Symbol;Acc:HGNC:6004]
IPO7	importin 7 [Source:HGNC Symbol;Acc:HGNC:9852]
KCTD1	potassium channel tetramerization domain containing 1 [Source:HGNC Symbol;Acc:HGNC:18249]
KLHL29	kelch like family member 29 [Source:HGNC Symbol;Acc:HGNC:29404]
KRT9	keratin 9 [Source:HGNC Symbol;Acc:HGNC:6447]
MEIOB	meiosis specific with OB domains [Source:HGNC Symbol;Acc:HGNC:28569]
MPP7	membrane palmitoylated protein 7 [Source:HGNC Symbol;Acc:HGNC:26542]
MT1HL1	metallothionein 1H-like 1 [Source:HGNC Symbol;Acc:HGNC:31864]
MYRFL	myelin regulatory factor-like [Source:HGNC Symbol;Acc:HGNC:26316]
OSR2	odd-skipped related transciption factor 2 [Source:HGNC Symbol;Acc:HGNC:15830]
PLPP4	phospholipid phosphatase 4 [Source:HGNC Symbol;Acc:HGNC:23531]
POLR3GL	polymerase (RNA) III subunit G like [Source:HGNC Symbol;Acc:HGNC:28466]
PSCA	prostate stem cell antigen [Source:HGNC Symbol;Acc:HGNC:9500]
RING1	ring finger protein 1 [Source:HGNC Symbol;Acc:HGNC:10018]
RNF135	ring finger protein 135 [Source:HGNC Symbol;Acc:HGNC:21158]
SFTPA1	surfactant protein A1 [Source:HGNC Symbol;Acc:HGNC:10798]
TMEM30B	transmembrane protein 30B [Source:HGNC Symbol;Acc:HGNC:27254]
TNPO3	transportin 3 [Source:HGNC Symbol;Acc:HGNC:17103]
TRIM22	tripartite motif containing 22 [Source:HGNC Symbol;Acc:HGNC:16379]
C9orf40	chromosome 9 open reading frame 40 [Source:HGNC Symbol;Acc:HGNC:23433]
CCAR2	cell cycle and apoptosis regulator 2 [Source:HGNC Symbol;Acc:HGNC:23360]
T	T brachyury transcription factor [Source:HGNC Symbol;Acc:HGNC:11515]
AFF3	AF4/FMR2 family member 3 [Source:HGNC Symbol;Acc:HGNC:6473]
EGLN3	egl-9 family hypoxia inducible factor 3 [Source:HGNC Symbol;Acc:HGNC:14661]
EVA1A	eva-1 homolog A, regulator of programmed cell death [Source:HGNC Symbol;Acc:HGNC:25816]
HERC6	HECT and RLD domain containing E3 ubiquitin protein ligase family member 6 [Source:HGNC Symbol;Acc:HGNC:26072]
NAP1L5	nucleosome assembly protein 1 like 5 [Source:HGNC Symbol;Acc:HGNC:19968]
NOVA1	NOVA alternative splicing regulator 1 [Source:HGNC Symbol;Acc:HGNC:7886]
SPINT1	serine peptidase inhibitor, Kunitz type 1 [Source:HGNC Symbol;Acc:HGNC:11246]

AP1M2	adaptor related protein complex 1 mu 2 subunit [Source:HGNC Symbol;Acc:HGNC:558]
B4GALT6	beta-1,4-galactosyltransferase 6 [Source:HGNC Symbol;Acc:HGNC:929]
BOP1	block of proliferation 1 [Source:HGNC Symbol;Acc:HGNC:15519]
BTBD17	BTB domain containing 17 [Source:HGNC Symbol;Acc:HGNC:33758]
COBLL1	cordon-bleu WH2 repeat protein like 1 [Source:HGNC Symbol;Acc:HGNC:23571]
CPEB3	cytoplasmic polyadenylation element binding protein 3 [Source:HGNC Symbol;Acc:HGNC:21746]
FA2H	fatty acid 2-hydroxylase [Source:HGNC Symbol;Acc:HGNC:21197]
FEZ2	fasciculation and elongation protein zeta 2 [Source:HGNC Symbol;Acc:HGNC:3660]
HEMGN	hemogen [Source:HGNC Symbol;Acc:HGNC:17509]
HOXA5	homeobox A5 [Source:HGNC Symbol;Acc:HGNC:5106]
LAMC2	laminin subunit gamma 2 [Source:HGNC Symbol;Acc:HGNC:6493]
LGSN	lengsin, lens protein with glutamine synthetase domain [Source:HGNC Symbol;Acc:HGNC:21016]
MAST4	microtubule associated serine/threonine kinase family member 4 [Source:HGNC Symbol;Acc:HGNC:19037]
MOB3B	MOB kinase activator 3B [Source:HGNC Symbol;Acc:HGNC:23825]
OLFM4	olfactomedin 4 [Source:HGNC Symbol;Acc:HGNC:17190]
PHF11	PHD finger protein 11 [Source:HGNC Symbol;Acc:HGNC:17024]
THOC5	THO complex 5 [Source:HGNC Symbol;Acc:HGNC:19074]
TRIM16L	tripartite motif containing 16-like [Source:HGNC Symbol;Acc:HGNC:32670]
CARD16	caspase recruitment domain family member 16 [Source:HGNC Symbol;Acc:HGNC:33701]
FAM222B	family with sequence similarity 222 member B [Source:HGNC Symbol;Acc:HGNC:25563]
KCNS1	potassium voltage-gated channel modifier subfamily S member 1 [Source:HGNC Symbol;Acc:HGNC:6300]
TRABD2A	TraB domain containing 2A [Source:HGNC Symbol;Acc:HGNC:27013]
ZNF25	zinc finger protein 25 [Source:HGNC Symbol;Acc:HGNC:13043]
ATP5D	ATP synthase, H+ transporting, mitochondrial F1 complex, delta subunit [Source:HGNC Symbol;Acc:HGNC:837]
ATP8B4	ATPase phospholipid transporting 8B4 (putative) [Source:HGNC Symbol;Acc:HGNC:13536]
EPDR1	ependymin related 1 [Source:HGNC Symbol;Acc:HGNC:17572]
FBXO43	F-box protein 43 [Source:HGNC Symbol;Acc:HGNC:28521]
FRMD4B	FERM domain containing 4B [Source:HGNC Symbol;Acc:HGNC:24886]
FXYD3	FXYD domain containing ion transport regulator 3 [Source:HGNC Symbol;Acc:HGNC:4027]
GFOD1	glucose-fructose oxidoreductase domain containing 1 [Source:HGNC Symbol;Acc:HGNC:21096]
GTPBP6	GTP binding protein 6 (putative) [Source:HGNC Symbol;Acc:HGNC:30189]
IFT80	intraflagellar transport 80 [Source:HGNC Symbol;Acc:HGNC:29262]
KMT2C	lysine methyltransferase 2C [Source:HGNC Symbol;Acc:HGNC:13726]

KRT78	keratin 78 [Source:HGNC Symbol;Acc:HGNC:28926]
LAD1	ladinin 1 [Source:HGNC Symbol;Acc:HGNC:6472]
LIMK2	LIM domain kinase 2 [Source:HGNC Symbol;Acc:HGNC:6614]
MAPK8IP2	mitogen-activated protein kinase 8 interacting protein 2 [Source:HGNC Symbol;Acc:HGNC:6883]
MITD1	microtubule interacting and trafficking domain containing 1 [Source:HGNC Symbol;Acc:HGNC:25207]
MVD	mevalonate diphosphate decarboxylase [Source:HGNC Symbol;Acc:HGNC:7529]
MYCL	v-myc avian myelocytomatosis viral oncogene lung carcinoma derived homolog [Source:HGNC Symbol;Acc:HGNC:7555]
MYO5C	myosin VC [Source:HGNC Symbol;Acc:HGNC:7604]
MYO6	myosin VI [Source:HGNC Symbol;Acc:HGNC:7605]
PDE8B	phosphodiesterase 8B [Source:HGNC Symbol;Acc:HGNC:8794]
PLA2G2E	phospholipase A2 group IIE [Source:HGNC Symbol;Acc:HGNC:13414]
PPP3CC	protein phosphatase 3 catalytic subunit gamma [Source:HGNC Symbol;Acc:HGNC:9316]
RHAG	Rh-associated glycoprotein [Source:HGNC Symbol;Acc:HGNC:10006]
S100A7	S100 calcium binding protein A7 [Source:HGNC Symbol;Acc:HGNC:10497]
SLC24A3	solute carrier family 24 member 3 [Source:HGNC Symbol;Acc:HGNC:10977]
SMG5	SMG5, nonsense mediated mRNA decay factor [Source:HGNC Symbol;Acc:HGNC:24644]
SPATS2L	spermatogenesis associated serine rich 2 like [Source:HGNC Symbol;Acc:HGNC:24574]
SPOCK1	sparc/osteonectin, cwcv and kazal-like domains proteoglycan (testican) 1 [Source:HGNC Symbol;Acc:HGNC:11251]
ST3GAL4	ST3 beta-galactoside alpha-2,3-sialyltransferase 4 [Source:HGNC Symbol;Acc:HGNC:10864]
TELO2	telomere maintenance 2 [Source:HGNC Symbol;Acc:HGNC:29099]
TEX19	testis expressed 19 [Source:HGNC Symbol;Acc:HGNC:33802]
TMCC3	transmembrane and coiled-coil domain family 3 [Source:HGNC Symbol;Acc:HGNC:29199]
TUSC2	tumor suppressor candidate 2 [Source:HGNC Symbol;Acc:HGNC:17034]
TUSC3	tumor suppressor candidate 3 [Source:HGNC Symbol;Acc:HGNC:30242]
HTRA3	HtrA serine peptidase 3 [Source:HGNC Symbol;Acc:HGNC:30406]
IFNAR2	interferon alpha and beta receptor subunit 2 [Source:HGNC Symbol;Acc:HGNC:5433]
RGS17	regulator of G-protein signaling 17 [Source:HGNC Symbol;Acc:HGNC:14088]
SCLY	selenocysteine lyase [Source:HGNC Symbol;Acc:HGNC:18161]
ATP5G3	ATP synthase, H+ transporting, mitochondrial Fo complex subunit C3 (subunit 9) [Source:HGNC Symbol;Acc:HGNC:843]
EDC4	enhancer of mRNA decapping 4 [Source:HGNC Symbol;Acc:HGNC:17157]
EXOSC1	exosome component 1 [Source:HGNC Symbol;Acc:HGNC:17286]
KANSL3	KAT8 regulatory NSL complex subunit 3 [Source:HGNC Symbol;Acc:HGNC:25473]
MAK16	MAK16 homolog [Source:HGNC Symbol;Acc:HGNC:13703]
RSBN1L	round spermatid basic protein 1 like [Source:HGNC Symbol;Acc:HGNC:24765]

TGM5	transglutaminase 5 [Source:HGNC Symbol;Acc:HGNC:11781]
C1orf54	chromosome 1 open reading frame 54 [Source:HGNC Symbol;Acc:HGNC:26258]
CDK14	cyclin dependent kinase 14 [Source:HGNC Symbol;Acc:HGNC:8883]
CHPF2	chondroitin polymerizing factor 2 [Source:HGNC Symbol;Acc:HGNC:29270]
EFNA5	ephrin A5 [Source:HGNC Symbol;Acc:HGNC:3225]
FBXO15	F-box protein 15 [Source:HGNC Symbol;Acc:HGNC:13617]
FBXW7	F-box and WD repeat domain containing 7 [Source:HGNC Symbol;Acc:HGNC:16712]
LY9	lymphocyte antigen 9 [Source:HGNC Symbol;Acc:HGNC:6730]
MED26	mediator complex subunit 26 [Source:HGNC Symbol;Acc:HGNC:2376]
NSRP1	nuclear speckle splicing regulatory protein 1 [Source:HGNC Symbol;Acc:HGNC:25305]
PEX2	peroxisomal biogenesis factor 2 [Source:HGNC Symbol;Acc:HGNC:9717]
PLXND1	plexin D1 [Source:HGNC Symbol;Acc:HGNC:9107]
PRRG4	proline rich and Gla domain 4 [Source:HGNC Symbol;Acc:HGNC:30799]
S100A14	S100 calcium binding protein A14 [Source:HGNC Symbol;Acc:HGNC:18901]
SORD	sorbitol dehydrogenase [Source:HGNC Symbol;Acc:HGNC:11184]
TEX15	testis expressed 15 [Source:HGNC Symbol;Acc:HGNC:11738]
TTC19	tetratricopeptide repeat domain 19 [Source:HGNC Symbol;Acc:HGNC:26006]
AMY1A	amylase, alpha 1A (salivary) [Source:HGNC Symbol;Acc:HGNC:474]
ANKRD10	ankyrin repeat domain 10 [Source:HGNC Symbol;Acc:HGNC:20265]
CD22	CD22 molecule [Source:HGNC Symbol;Acc:HGNC:1643]
DHDDS	dehydrodolichyl diphosphate synthase subunit [Source:HGNC Symbol;Acc:HGNC:20603]
EP400	E1A binding protein p400 [Source:HGNC Symbol;Acc:HGNC:11958]
HEY2	hes related family bHLH transcription factor with YRPW motif 2 [Source:HGNC Symbol;Acc:HGNC:4881]
HOXA7	homeobox A7 [Source:HGNC Symbol;Acc:HGNC:5108]
INTS6L	integrator complex subunit 6 like [Source:HGNC Symbol;Acc:HGNC:27334]
JAG2	jagged 2 [Source:HGNC Symbol;Acc:HGNC:6189]
PLAC1	placenta specific 1 [Source:HGNC Symbol;Acc:HGNC:9044]
SLC13A1	solute carrier family 13 member 1 [Source:HGNC Symbol;Acc:HGNC:10916]
SLC23A3	solute carrier family 23 member 3 [Source:HGNC Symbol;Acc:HGNC:20601]
SLC27A6	solute carrier family 27 member 6 [Source:HGNC Symbol;Acc:HGNC:11000]
YIPF6	Yip1 domain family member 6 [Source:HGNC Symbol;Acc:HGNC:28304]
ZNHIT2	zinc finger HIT-type containing 2 [Source:HGNC Symbol;Acc:HGNC:1177]
AHSA2	AHA1, activator of heat shock 90kDa protein ATPase homolog 2 (yeast) [Source:HGNC Symbol;Acc:HGNC:20437]
ATPAF2	ATP synthase mitochondrial F1 complex assembly factor 2 [Source:HGNC Symbol;Acc:HGNC:18802]
CDH12	cadherin 12 [Source:HGNC Symbol;Acc:HGNC:1751]
GEMIN7	gem nuclear organelle associated protein 7 [Source:HGNC Symbol;Acc:HGNC:20045]
HM13	histocompatibility (minor) 13 [Source:HGNC Symbol;Acc:HGNC:16435]
LYRM4	LYR motif containing 4 [Source:HGNC Symbol;Acc:HGNC:21365]

NT5C3A	5'-nucleotidase, cytosolic IIIA [Source:HGNC Symbol;Acc:HGNC:17820]
REG3A	regenerating family member 3 alpha [Source:HGNC Symbol;Acc:HGNC:8601]
RPS19BP1	ribosomal protein S19 binding protein 1 [Source:HGNC Symbol;Acc:HGNC:28749]
TPRG1L	tumor protein p63 regulated 1-like [Source:HGNC Symbol;Acc:HGNC:27007]
CLIC3	chloride intracellular channel 3 [Source:HGNC Symbol;Acc:HGNC:2064]
CSF3R	colony stimulating factor 3 receptor [Source:HGNC Symbol;Acc:HGNC:2439]
GPRC5B	G protein-coupled receptor class C group 5 member B [Source:HGNC Symbol;Acc:HGNC:13308]
IMPA1	inositol monophosphatase 1 [Source:HGNC Symbol;Acc:HGNC:6050]
IRX3	iroquois homeobox 3 [Source:HGNC Symbol;Acc:HGNC:14360]
KRT13	keratin 13 [Source:HGNC Symbol;Acc:HGNC:6415]
NFATC4	nuclear factor of activated T-cells 4 [Source:HGNC Symbol;Acc:HGNC:7778]
PCOLCE2	procollagen C-endopeptidase enhancer 2 [Source:HGNC Symbol;Acc:HGNC:8739]
SAMD9L	sterile alpha motif domain containing 9 like [Source:HGNC Symbol;Acc:HGNC:1349]
SVEP1	sushi, von Willebrand factor type A, EGF and pentraxin domain containing 1 [Source:HGNC Symbol;Acc:HGNC:15985]
TRIM14	tripartite motif containing 14 [Source:HGNC Symbol;Acc:HGNC:16283]
TSPAN13	tetraspanin 13 [Source:HGNC Symbol;Acc:HGNC:21643]
UPF3B	UPF3 regulator of nonsense transcripts homolog B (yeast) [Source:HGNC Symbol;Acc:HGNC:20439]
C1orf115	chromosome 1 open reading frame 115 [Source:HGNC Symbol;Acc:HGNC:25873]
C4orf46	chromosome 4 open reading frame 46 [Source:HGNC Symbol;Acc:HGNC:27320]
NKX2-5	NK2 homeobox 5 [Source:HGNC Symbol;Acc:HGNC:2488]
SMN2	survival of motor neuron 2, centromeric [Source:HGNC Symbol;Acc:HGNC:11118]
TACO1	translational activator of cytochrome c oxidase I [Source:HGNC Symbol;Acc:HGNC:24316]
ZNF84	zinc finger protein 84 [Source:HGNC Symbol;Acc:HGNC:13159]
ZSWIM8	zinc finger SWIM-type containing 8 [Source:HGNC Symbol;Acc:HGNC:23528]
ATF7	activating transcription factor 7 [Source:HGNC Symbol;Acc:HGNC:792]
ELP3	elongator acetyltransferase complex subunit 3 [Source:HGNC Symbol;Acc:HGNC:20696]
GNPTG	N-acetylglucosamine-1-phosphate transferase gamma subunit [Source:HGNC Symbol;Acc:HGNC:23026]
GPR4	G protein-coupled receptor 4 [Source:HGNC Symbol;Acc:HGNC:4497]
IFT46	intraflagellar transport 46 [Source:HGNC Symbol;Acc:HGNC:26146]
MINK1	misshapen like kinase 1 [Source:HGNC Symbol;Acc:HGNC:17565]
REV1	REV1, DNA directed polymerase [Source:HGNC Symbol;Acc:HGNC:14060]
RIPK4	receptor interacting serine/threonine kinase 4 [Source:HGNC Symbol;Acc:HGNC:496]
RNF8	ring finger protein 8 [Source:HGNC Symbol;Acc:HGNC:10071]
SATB2	SATB homeobox 2 [Source:HGNC Symbol;Acc:HGNC:21637]
SLC26A10	solute carrier family 26 member 10 [Source:HGNC Symbol;Acc:HGNC:14470]
SLC45A1	solute carrier family 45 member 1 [Source:HGNC Symbol;Acc:HGNC:17939]
TAOK1	TAO kinase 1 [Source:HGNC Symbol;Acc:HGNC:29259]

TIMM17A	translocase of inner mitochondrial membrane 17 homolog A (yeast) [Source:HGNC Symbol;Acc:HGNC:17315]
TLE3	transducin like enhancer of split 3 [Source:HGNC Symbol;Acc:HGNC:11839]
VAMP7	vesicle associated membrane protein 7 [Source:HGNC Symbol;Acc:HGNC:11486]
ZNF296	zinc finger protein 296 [Source:HGNC Symbol;Acc:HGNC:15981]
ARHGAP30	Rho GTPase activating protein 30 [Source:HGNC Symbol;Acc:HGNC:27414]
CPOX	coproporphyrinogen oxidase [Source:HGNC Symbol;Acc:HGNC:2321]
DPYSL4	dihydropyrimidinase like 4 [Source:HGNC Symbol;Acc:HGNC:3016]
EPPK1	epiplakin 1 [Source:HGNC Symbol;Acc:HGNC:15577]
EVI2A	ecotropic viral integration site 2A [Source:HGNC Symbol;Acc:HGNC:3499]
FBF1	Fas binding factor 1 [Source:HGNC Symbol;Acc:HGNC:24674]
FGF6	fibroblast growth factor 6 [Source:HGNC Symbol;Acc:HGNC:3684]
GABRA1	gamma-aminobutyric acid type A receptor alpha1 subunit [Source:HGNC Symbol;Acc:HGNC:4075]
GNRH2	gonadotropin releasing hormone 2 [Source:HGNC Symbol;Acc:HGNC:4420]
GPR155	G protein-coupled receptor 155 [Source:HGNC Symbol;Acc:HGNC:22951]
IL37	interleukin 37 [Source:HGNC Symbol;Acc:HGNC:15563]
KANK1	KN motif and ankyrin repeat domains 1 [Source:HGNC Symbol;Acc:HGNC:19309]
MAP7	microtubule associated protein 7 [Source:HGNC Symbol;Acc:HGNC:6869]
MCC	mutated in colorectal cancers [Source:HGNC Symbol;Acc:HGNC:6935]
PIGH	phosphatidylinositol glycan anchor biosynthesis class H [Source:HGNC Symbol;Acc:HGNC:8964]
RBM47	RNA binding motif protein 47 [Source:HGNC Symbol;Acc:HGNC:30358]
STXBP6	syntaxin binding protein 6 [Source:HGNC Symbol;Acc:HGNC:19666]
SYPL2	synaptophysin like 2 [Source:HGNC Symbol;Acc:HGNC:27638]
UBE2D4	ubiquitin conjugating enzyme E2 D4 (putative) [Source:HGNC Symbol;Acc:HGNC:21647]
CAPSL	calcyphosine like [Source:HGNC Symbol;Acc:HGNC:28375]
CERS1	ceramide synthase 1 [Source:HGNC Symbol;Acc:HGNC:14253]
CFAP36	cilia and flagella associated protein 36 [Source:HGNC Symbol;Acc:HGNC:30540]
DDX55	DEAD-box helicase 55 [Source:HGNC Symbol;Acc:HGNC:20085]
IFT43	intraflagellar transport 43 [Source:HGNC Symbol;Acc:HGNC:29669]
PNPLA5	patatin like phospholipase domain containing 5 [Source:HGNC Symbol;Acc:HGNC:24888]
RSPRY1	ring finger and SPRY domain containing 1 [Source:HGNC Symbol;Acc:HGNC:29420]
SLC25A40	solute carrier family 25 member 40 [Source:HGNC Symbol;Acc:HGNC:29680]
TBCCD1	TBCC domain containing 1 [Source:HGNC Symbol;Acc:HGNC:25546]
TMEM26	transmembrane protein 26 [Source:HGNC Symbol;Acc:HGNC:28550]
ZNF507	zinc finger protein 507 [Source:HGNC Symbol;Acc:HGNC:23783]
AMPD1	adenosine monophosphate deaminase 1 [Source:HGNC Symbol;Acc:HGNC:468]
ARMC1	armadillo repeat containing 1 [Source:HGNC Symbol;Acc:HGNC:17684]
ATAT1	alpha tubulin acetyltransferase 1 [Source:HGNC Symbol;Acc:HGNC:21186]

ATOH8	atonal bHLH transcription factor 8 [Source:HGNC Symbol;Acc:HGNC:24126]
CHRAC1	chromatin accessibility complex 1 [Source:HGNC Symbol;Acc:HGNC:13544]
CNIH3	cornichon family AMPA receptor auxiliary protein 3 [Source:HGNC Symbol;Acc:HGNC:26802]
COG7	component of oligomeric golgi complex 7 [Source:HGNC Symbol;Acc:HGNC:18622]
COL4A6	collagen type IV alpha 6 chain [Source:HGNC Symbol;Acc:HGNC:2208]
CYP4F11	cytochrome P450 family 4 subfamily F member 11 [Source:HGNC Symbol;Acc:HGNC:13265]
KANK4	KN motif and ankyrin repeat domains 4 [Source:HGNC Symbol;Acc:HGNC:27263]
KCNJ3	potassium voltage-gated channel subfamily J member 3 [Source:HGNC Symbol;Acc:HGNC:6264]
LRRC47	leucine rich repeat containing 47 [Source:HGNC Symbol;Acc:HGNC:29207]
LSR	lipolysis stimulated lipoprotein receptor [Source:HGNC Symbol;Acc:HGNC:29572]
MECOM	MDS1 and EVI1 complex locus [Source:HGNC Symbol;Acc:HGNC:3498]
NAV1	neuron navigator 1 [Source:HGNC Symbol;Acc:HGNC:15989]
PIP	prolactin induced protein [Source:HGNC Symbol;Acc:HGNC:8993]
RNF183	ring finger protein 183 [Source:HGNC Symbol;Acc:HGNC:28721]
SGSH	N-sulfoglucosamine sulfohydrolase [Source:HGNC Symbol;Acc:HGNC:10818]
SLC35F1	solute carrier family 35 member F1 [Source:HGNC Symbol;Acc:HGNC:21483]
USF1	upstream transcription factor 1 [Source:HGNC Symbol;Acc:HGNC:12593]
VSTM2L	V-set and transmembrane domain containing 2 like [Source:HGNC Symbol;Acc:HGNC:16096]
ZMYND19	zinc finger MYND-type containing 19 [Source:HGNC Symbol;Acc:HGNC:21146]
CPM	carboxypeptidase M [Source:HGNC Symbol;Acc:HGNC:2311]
FZD6	frizzled class receptor 6 [Source:HGNC Symbol;Acc:HGNC:4044]
KRT4	keratin 4 [Source:HGNC Symbol;Acc:HGNC:6441]
MAN2A1	mannosidase alpha class 2A member 1 [Source:HGNC Symbol;Acc:HGNC:6824]
PPP1R1A	protein phosphatase 1 regulatory inhibitor subunit 1A [Source:HGNC Symbol;Acc:HGNC:9286]
PROX1	prospero homeobox 1 [Source:HGNC Symbol;Acc:HGNC:9459]
SPINT2	serine peptidase inhibitor, Kunitz type, 2 [Source:HGNC Symbol;Acc:HGNC:11247]
AGR3	anterior gradient 3, protein disulphide isomerase family member [Source:HGNC Symbol;Acc:HGNC:24167]
DDX49	DEAD-box helicase 49 [Source:HGNC Symbol;Acc:HGNC:18684]
DEFB4A	defensin beta 4A [Source:HGNC Symbol;Acc:HGNC:2767]
GTF3C3	general transcription factor IIIC subunit 3 [Source:HGNC Symbol;Acc:HGNC:4666]
IL20	interleukin 20 [Source:HGNC Symbol;Acc:HGNC:6002]
KCNH8	potassium voltage-gated channel subfamily H member 8 [Source:HGNC Symbol;Acc:HGNC:18864]
POLDIP3	polymerase (DNA) delta interacting protein 3 [Source:HGNC Symbol;Acc:HGNC:23782]
POLR2J	polymerase (RNA) II subunit J [Source:HGNC Symbol;Acc:HGNC:9197]
RAET1E	retinoic acid early transcript 1E [Source:HGNC Symbol;Acc:HGNC:16793]

RIBC2	RIB43A domain with coiled-coils 2 [Source:HGNC Symbol;Acc:HGNC:13241]
SDR39U1	short chain dehydrogenase/reductase family 39U member 1 [Source:HGNC Symbol;Acc:HGNC:20275]
TRAPPC5	trafficking protein particle complex 5 [Source:HGNC Symbol;Acc:HGNC:23067]
UGT2B11	UDP glucuronosyltransferase family 2 member B11 [Source:HGNC Symbol;Acc:HGNC:12545]
UNC119B	unc-119 lipid binding chaperone B [Source:HGNC Symbol;Acc:HGNC:16488]
ACSS1	acyl-CoA synthetase short-chain family member 1 [Source:HGNC Symbol;Acc:HGNC:16091]
ATP2C2	ATPase secretory pathway Ca2+ transporting 2 [Source:HGNC Symbol;Acc:HGNC:29103]
B4GALNT4	beta-1,4-N-acetyl-galactosaminyltransferase 4 [Source:HGNC Symbol;Acc:HGNC:26315]
CALML3	calmodulin like 3 [Source:HGNC Symbol;Acc:HGNC:1452]
COL16A1	collagen type XVI alpha 1 chain [Source:HGNC Symbol;Acc:HGNC:2193]
CPNE8	copine 8 [Source:HGNC Symbol;Acc:HGNC:23498]
CYB561D2	cytochrome b561 family member D2 [Source:HGNC Symbol;Acc:HGNC:30253]
DHX35	DEAH-box helicase 35 [Source:HGNC Symbol;Acc:HGNC:15861]
DNALI1	dynein axonemal light intermediate chain 1 [Source:HGNC Symbol;Acc:HGNC:14353]
EID2	EP300 interacting inhibitor of differentiation 2 [Source:HGNC Symbol;Acc:HGNC:28292]
FCF1	FCF1 rRNA-processing protein [Source:HGNC Symbol;Acc:HGNC:20220]
FLI1	Fli-1 proto-oncogene, ETS transcription factor [Source:HGNC Symbol;Acc:HGNC:3749]
FSTL4	follistatin like 4 [Source:HGNC Symbol;Acc:HGNC:21389]
GPKOW	G-patch domain and KOW motifs [Source:HGNC Symbol;Acc:HGNC:30677]
GTPBP3	GTP binding protein 3 (mitochondrial) [Source:HGNC Symbol;Acc:HGNC:14880]
HBA2	hemoglobin subunit alpha 2 [Source:HGNC Symbol;Acc:HGNC:4824]
HGH1	HGH1 homolog [Source:HGNC Symbol;Acc:HGNC:24161]
IPO8	importin 8 [Source:HGNC Symbol;Acc:HGNC:9853]
JAML	junction adhesion molecule like [Source:HGNC Symbol;Acc:HGNC:19084]
KNOP1	lysine rich nucleolar protein 1 [Source:HGNC Symbol;Acc:HGNC:34404]
LRRC32	leucine rich repeat containing 32 [Source:HGNC Symbol;Acc:HGNC:4161]
MAB21L3	mab-21 like 3 [Source:HGNC Symbol;Acc:HGNC:26787]
MARCO	macrophage receptor with collagenous structure [Source:HGNC Symbol;Acc:HGNC:6895]
MEX3D	mex-3 RNA binding family member D [Source:HGNC Symbol;Acc:HGNC:16734]
MPL	MPL proto-oncogene, thrombopoietin receptor [Source:HGNC Symbol;Acc:HGNC:7217]
PADI2	peptidyl arginine deiminase 2 [Source:HGNC Symbol;Acc:HGNC:18341]
PDCD2L	programmed cell death 2-like [Source:HGNC Symbol;Acc:HGNC:28194]
PDIA2	protein disulfide isomerase family A member 2 [Source:HGNC Symbol;Acc:HGNC:14180]
PGBD5	piggyBac transposable element derived 5 [Source:HGNC Symbol;Acc:HGNC:19405]

PHAX	phosphorylated adaptor for RNA export [Source:HGNC Symbol;Acc:HGNC:10241]
PIK3C2A	phosphatidylinositol-4-phosphate 3-kinase catalytic subunit type 2 alpha [Source:HGNC Symbol;Acc:HGNC:8971]
SH3PXD2A	SH3 and PX domains 2A [Source:HGNC Symbol;Acc:HGNC:23664]
SHISA2	shisa family member 2 [Source:HGNC Symbol;Acc:HGNC:20366]
SMCR8	Smith-Magenis syndrome chromosome region, candidate 8 [Source:HGNC Symbol;Acc:HGNC:17921]
SRPX	sushi repeat containing protein, X-linked [Source:HGNC Symbol;Acc:HGNC:11309]
TIMM50	translocase of inner mitochondrial membrane 50 [Source:HGNC Symbol;Acc:HGNC:23656]
YTHDF3	YTH N6-methyladenosine RNA binding protein 3 [Source:HGNC Symbol;Acc:HGNC:26465]
ZSCAN26	zinc finger and SCAN domain containing 26 [Source:HGNC Symbol;Acc:HGNC:12978]
ATG13	autophagy related 13 [Source:HGNC Symbol;Acc:HGNC:29091]
CCNYL1	cyclin Y like 1 [Source:HGNC Symbol;Acc:HGNC:26868]
CSN2	casein beta [Source:HGNC Symbol;Acc:HGNC:2447]
DDX4	DEAD-box helicase 4 [Source:HGNC Symbol;Acc:HGNC:18700]
FAN1	FANCD2/FANCI-associated nuclease 1 [Source:HGNC Symbol;Acc:HGNC:29170]
FLRT2	fibronectin leucine rich transmembrane protein 2 [Source:HGNC Symbol;Acc:HGNC:3761]
KIF1C	kinesin family member 1C [Source:HGNC Symbol;Acc:HGNC:6317]
MAFA	MAF bZIP transcription factor A [Source:HGNC Symbol;Acc:HGNC:23145]
MRAS	muscle RAS oncogene homolog [Source:HGNC Symbol;Acc:HGNC:7227]
PPP1R9A	protein phosphatase 1 regulatory subunit 9A [Source:HGNC Symbol;Acc:HGNC:14946]
RHOB	ras homolog family member B [Source:HGNC Symbol;Acc:HGNC:668]
SLITRK6	SLIT and NTRK like family member 6 [Source:HGNC Symbol;Acc:HGNC:23503]
TRUB2	TruB pseudouridine synthase family member 2 [Source:HGNC Symbol;Acc:HGNC:17170]
ZBTB17	zinc finger and BTB domain containing 17 [Source:HGNC Symbol;Acc:HGNC:12936]
ATP5O	ATP synthase, H+ transporting, mitochondrial F1 complex, O subunit [Source:HGNC Symbol;Acc:HGNC:850]
COMMD7	COMM domain containing 7 [Source:HGNC Symbol;Acc:HGNC:16223]
EMC2	ER membrane protein complex subunit 2 [Source:HGNC Symbol;Acc:HGNC:28963]
FLT3LG	fms related tyrosine kinase 3 ligand [Source:HGNC Symbol;Acc:HGNC:3766]
HS1BP3	HCLS1 binding protein 3 [Source:HGNC Symbol;Acc:HGNC:24979]
NOM1	nucleolar protein with MIF4G domain 1 [Source:HGNC Symbol;Acc:HGNC:13244]
PSMG3	proteasome assembly chaperone 3 [Source:HGNC Symbol;Acc:HGNC:22420]
SYAP1	synapse associated protein 1 [Source:HGNC Symbol;Acc:HGNC:16273]
ABLIM3	actin binding LIM protein family member 3 [Source:HGNC Symbol;Acc:HGNC:29132]
BRIP1	BRCA1 interacting protein C-terminal helicase 1 [Source:HGNC Symbol;Acc:HGNC:20473]
CAMK1D	calcium/calmodulin dependent protein kinase ID [Source:HGNC Symbol;Acc:HGNC:19341]

CD52	CD52 molecule [Source:HGNC Symbol;Acc:HGNC:1804]
DZIP1	DAZ interacting zinc finger protein 1 [Source:HGNC Symbol;Acc:HGNC:20908]
FRMD4A	FERM domain containing 4A [Source:HGNC Symbol;Acc:HGNC:25491]
GALNT14	polypeptide N-acetylgalactosaminyltransferase 14 [Source:HGNC Symbol;Acc:HGNC:22946]
GPR107	G protein-coupled receptor 107 [Source:HGNC Symbol;Acc:HGNC:17830]
GPR27	G protein-coupled receptor 27 [Source:HGNC Symbol;Acc:HGNC:4482]
GPX5	glutathione peroxidase 5 [Source:HGNC Symbol;Acc:HGNC:4557]
GRTP1	growth hormone regulated TBC protein 1 [Source:HGNC Symbol;Acc:HGNC:20310]
HOXC9	homeobox C9 [Source:HGNC Symbol;Acc:HGNC:5130]
HOXD10	homeobox D10 [Source:HGNC Symbol;Acc:HGNC:5133]
INTS10	integrator complex subunit 10 [Source:HGNC Symbol;Acc:HGNC:25548]
LY6G6C	lymphocyte antigen 6 complex, locus G6C [Source:HGNC Symbol;Acc:HGNC:13936]
LZTR1	leucine-zipper-like transcription regulator 1 [Source:HGNC Symbol;Acc:HGNC:6742]
MAPK12	mitogen-activated protein kinase 12 [Source:HGNC Symbol;Acc:HGNC:6874]
MYBPC2	myosin binding protein C, fast type [Source:HGNC Symbol;Acc:HGNC:7550]
NEK3	NIMA related kinase 3 [Source:HGNC Symbol;Acc:HGNC:7746]
NEMF	nuclear export mediator factor [Source:HGNC Symbol;Acc:HGNC:10663]
PATJ	PATJ, crumbs cell polarity complex component [Source:HGNC Symbol;Acc:HGNC:28881]
PHOSPHO2	phosphatase, orphan 2 [Source:HGNC Symbol;Acc:HGNC:28316]
PMF1	polyamine-modulated factor 1 [Source:HGNC Symbol;Acc:HGNC:9112]
RELT	RELT tumor necrosis factor receptor [Source:HGNC Symbol;Acc:HGNC:13764]
SLC46A3	solute carrier family 46 member 3 [Source:HGNC Symbol;Acc:HGNC:27501]
TAF1D	TATA-box binding protein associated factor, RNA polymerase I subunit D [Source:HGNC Symbol;Acc:HGNC:28759]
TCF20	transcription factor 20 (AR1) [Source:HGNC Symbol;Acc:HGNC:11631]
DARS	aspartyl-tRNA synthetase [Source:HGNC Symbol;Acc:HGNC:2678]
FAM72A	family with sequence similarity 72 member A [Source:HGNC Symbol;Acc:HGNC:24044]
FOXE1	forkhead box E1 [Source:HGNC Symbol;Acc:HGNC:3806]
HBG2	hemoglobin subunit gamma 2 [Source:HGNC Symbol;Acc:HGNC:4832]
LCLAT1	lysocardiolipin acyltransferase 1 [Source:HGNC Symbol;Acc:HGNC:26756]
C12orf10	chromosome 12 open reading frame 10 [Source:HGNC Symbol;Acc:HGNC:17590]
PLPPR2	phospholipid phosphatase related 2 [Source:HGNC Symbol;Acc:HGNC:29566]
VCPKMT	valosin containing protein lysine methyltransferase [Source:HGNC Symbol;Acc:HGNC:20352]
CCNG2	cyclin G2 [Source:HGNC Symbol;Acc:HGNC:1593]
CH25H	cholesterol 25-hydroxylase [Source:HGNC Symbol;Acc:HGNC:1907]
CXXC5	CXXC finger protein 5 [Source:HGNC Symbol;Acc:HGNC:26943]
CYB5R4	cytochrome b5 reductase 4 [Source:HGNC Symbol;Acc:HGNC:20147]
EIF1B	eukaryotic translation initiation factor 1B [Source:HGNC Symbol;Acc:HGNC:30792]

ETV1	ETS variant 1 [Source:HGNC Symbol;Acc:HGNC:3490]
GALK1	galactokinase 1 [Source:HGNC Symbol;Acc:HGNC:4118]
GTF3C6	general transcription factor IIIC subunit 6 [Source:HGNC Symbol;Acc:HGNC:20872]
HAUS6	HAUS augmin like complex subunit 6 [Source:HGNC Symbol;Acc:HGNC:25948]
HPS6	HPS6, biogenesis of lysosomal organelles complex 2 subunit 3 [Source:HGNC Symbol;Acc:HGNC:18817]
INPP5J	inositol polyphosphate-5-phosphatase J [Source:HGNC Symbol;Acc:HGNC:8956]
KRT2	keratin 2 [Source:HGNC Symbol;Acc:HGNC:6439]
LAMA4	laminin subunit alpha 4 [Source:HGNC Symbol;Acc:HGNC:6484]
LAMTOR5	late endosomal/lysosomal adaptor, MAPK and MTOR activator 5 [Source:HGNC Symbol;Acc:HGNC:17955]
MICU3	mitochondrial calcium uptake family member 3 [Source:HGNC Symbol;Acc:HGNC:27820]
MOCOS	molybdenum cofactor sulfurase [Source:HGNC Symbol;Acc:HGNC:18234]
MXD4	MAX dimerization protein 4 [Source:HGNC Symbol;Acc:HGNC:13906]
NAA20	N(alpha)-acetyltransferase 20, NatB catalytic subunit [Source:HGNC Symbol;Acc:HGNC:15908]
NOSIP	nitric oxide synthase interacting protein [Source:HGNC Symbol;Acc:HGNC:17946]
RESP18	regulated endocrine specific protein 18 [Source:HGNC Symbol;Acc:HGNC:33762]
RNF187	ring finger protein 187 [Source:HGNC Symbol;Acc:HGNC:27146]
SHPRH	SNF2 histone linker PHD RING helicase [Source:HGNC Symbol;Acc:HGNC:19336]
SNAI3	snail family transcriptional repressor 3 [Source:HGNC Symbol;Acc:HGNC:18411]
SNRNP35	small nuclear ribonucleoprotein U11/U12 subunit 35 [Source:HGNC Symbol;Acc:HGNC:30852]
SYT1	synaptotagmin 1 [Source:HGNC Symbol;Acc:HGNC:11509]
TAF12	TATA-box binding protein associated factor 12 [Source:HGNC Symbol;Acc:HGNC:11545]
TMEM132A	transmembrane protein 132A [Source:HGNC Symbol;Acc:HGNC:31092]
TRIM24	tripartite motif containing 24 [Source:HGNC Symbol;Acc:HGNC:11812]
ZNF165	zinc finger protein 165 [Source:HGNC Symbol;Acc:HGNC:12953]
ADAM19	ADAM metallopeptidase domain 19 [Source:HGNC Symbol;Acc:HGNC:197]
C1D	C1D nuclear receptor corepressor [Source:HGNC Symbol;Acc:HGNC:29911]
CNTN4	contactin 4 [Source:HGNC Symbol;Acc:HGNC:2174]
COQ6	coenzyme Q6, monooxygenase [Source:HGNC Symbol;Acc:HGNC:20233]
CYHR1	cysteine and histidine rich 1 [Source:HGNC Symbol;Acc:HGNC:17806]
DCBLD2	discoidin, CUB and LCCL domain containing 2 [Source:HGNC Symbol;Acc:HGNC:24627]
FBXO6	F-box protein 6 [Source:HGNC Symbol;Acc:HGNC:13585]
HNRNPA0	heterogeneous nuclear ribonucleoprotein A0 [Source:HGNC Symbol;Acc:HGNC:5030]
KYAT1	kynurenine aminotransferase 1 [Source:HGNC Symbol;Acc:HGNC:1564]
LSM2	LSM2 homolog, U6 small nuclear RNA and mRNA degradation associated [Source:HGNC Symbol;Acc:HGNC:13940]
MATN2	matrilin 2 [Source:HGNC Symbol;Acc:HGNC:6908]
MMP8	matrix metallopeptidase 8 [Source:HGNC Symbol;Acc:HGNC:7175]

PFN2	profilin 2 [Source:HGNC Symbol;Acc:HGNC:8882]
PLD6	phospholipase D family member 6 [Source:HGNC Symbol;Acc:HGNC:30447]
RBP2	retinol binding protein 2 [Source:HGNC Symbol;Acc:HGNC:9920]
SCRIB	scribbled planar cell polarity protein [Source:HGNC Symbol;Acc:HGNC:30377]
SH2B2	SH2B adaptor protein 2 [Source:HGNC Symbol;Acc:HGNC:17381]
SIX5	SIX homeobox 5 [Source:HGNC Symbol;Acc:HGNC:10891]
SLC38A7	solute carrier family 38 member 7 [Source:HGNC Symbol;Acc:HGNC:25582]
SLC7A8	solute carrier family 7 member 8 [Source:HGNC Symbol;Acc:HGNC:11066]
ST3GAL6	ST3 beta-galactoside alpha-2,3-sialyltransferase 6 [Source:HGNC Symbol;Acc:HGNC:18080]
TFCP2	transcription factor CP2 [Source:HGNC Symbol;Acc:HGNC:11748]
TRIB2	tribbles pseudokinase 2 [Source:HGNC Symbol;Acc:HGNC:30809]
TRPM4	transient receptor potential cation channel subfamily M member 4 [Source:HGNC Symbol;Acc:HGNC:17993]
UBE2R2	ubiquitin conjugating enzyme E2 R2 [Source:HGNC Symbol;Acc:HGNC:19907]
UCK1	uridine-cytidine kinase 1 [Source:HGNC Symbol;Acc:HGNC:14859]
ZDHHC9	zinc finger DHHC-type containing 9 [Source:HGNC Symbol;Acc:HGNC:18475]
BRF1	BRF1, RNA polymerase III transcription initiation factor 90 kDa subunit [Source:HGNC Symbol;Acc:HGNC:11551]
EN1	engrailed homeobox 1 [Source:HGNC Symbol;Acc:HGNC:3342]
FAM161B	family with sequence similarity 161 member B [Source:HGNC Symbol;Acc:HGNC:19854]
IL19	interleukin 19 [Source:HGNC Symbol;Acc:HGNC:5990]
POLR2K	polymerase (RNA) II subunit K [Source:HGNC Symbol;Acc:HGNC:9198]
RPRD1B	regulation of nuclear pre-mRNA domain containing 1B [Source:HGNC Symbol;Acc:HGNC:16209]
SF3B6	splicing factor 3b subunit 6 [Source:HGNC Symbol;Acc:HGNC:30096]
SFR1	SWI5 dependent homologous recombination repair protein 1 [Source:HGNC Symbol;Acc:HGNC:29574]
ASPHD2	aspartate beta-hydroxylase domain containing 2 [Source:HGNC Symbol;Acc:HGNC:30437]
BMI1	BMI1 proto-oncogene, polycomb ring finger [Source:HGNC Symbol;Acc:HGNC:1066]
CAPN15	calpain 15 [Source:HGNC Symbol;Acc:HGNC:11182]
COX10	COX10 heme A:farnesyltransferase cytochrome c oxidase assembly factor [Source:HGNC Symbol;Acc:HGNC:2260]
CSF2RB	colony stimulating factor 2 receptor beta common subunit [Source:HGNC Symbol;Acc:HGNC:2436]
CXCL16	C-X-C motif chemokine ligand 16 [Source:HGNC Symbol;Acc:HGNC:16642]
DSCAML1	DS cell adhesion molecule like 1 [Source:HGNC Symbol;Acc:HGNC:14656]
EMP3	epithelial membrane protein 3 [Source:HGNC Symbol;Acc:HGNC:3335]
FCRLA	Fc receptor like A [Source:HGNC Symbol;Acc:HGNC:18504]
FEZ1	fasciculation and elongation protein zeta 1 [Source:HGNC Symbol;Acc:HGNC:3659]
GPR161	G protein-coupled receptor 161 [Source:HGNC Symbol;Acc:HGNC:23694]

GRB14	growth factor receptor bound protein 14 [Source:HGNC Symbol;Acc:HGNC:4565]
HARS	histidyl-tRNA synthetase [Source:HGNC Symbol;Acc:HGNC:4816]
PCSK1N	proprotein convertase subtilisin/kexin type 1 inhibitor [Source:HGNC Symbol;Acc:HGNC:17301]
PRR7	proline rich 7 (synaptic) [Source:HGNC Symbol;Acc:HGNC:28130]
PSMB2	proteasome subunit beta 2 [Source:HGNC Symbol;Acc:HGNC:9539]
RAB3B	RAB3B, member RAS oncogene family [Source:HGNC Symbol;Acc:HGNC:9778]
RNASE6	ribonuclease A family member k6 [Source:HGNC Symbol;Acc:HGNC:10048]
RNLS	renalase, FAD dependent amine oxidase [Source:HGNC Symbol;Acc:HGNC:25641]
RUFY1	RUN and FYVE domain containing 1 [Source:HGNC Symbol;Acc:HGNC:19760]
SERPINI1	serpin family I member 1 [Source:HGNC Symbol;Acc:HGNC:8943]
SLC39A9	solute carrier family 39 member 9 [Source:HGNC Symbol;Acc:HGNC:20182]
TMEM79	transmembrane protein 79 [Source:HGNC Symbol;Acc:HGNC:28196]
TSSC4	tumor suppressing subtransferable candidate 4 [Source:HGNC Symbol;Acc:HGNC:12386]
TTC36	tetratricopeptide repeat domain 36 [Source:HGNC Symbol;Acc:HGNC:33708]
UBA6	ubiquitin like modifier activating enzyme 6 [Source:HGNC Symbol;Acc:HGNC:25581]
VIT	vitrin [Source:HGNC Symbol;Acc:HGNC:12697]
WDSUB1	WD repeat, sterile alpha motif and U-box domain containing 1 [Source:HGNC Symbol;Acc:HGNC:26697]
ZNF207	zinc finger protein 207 [Source:HGNC Symbol;Acc:HGNC:12998]
ZNF503	zinc finger protein 503 [Source:HGNC Symbol;Acc:HGNC:23589]
C1orf116	chromosome 1 open reading frame 116 [Source:HGNC Symbol;Acc:HGNC:28667]
CCDC14	coiled-coil domain containing 14 [Source:HGNC Symbol;Acc:HGNC:25766]
CMC2	C-X9-C motif containing 2 [Source:HGNC Symbol;Acc:HGNC:24447]
COMMD8	COMM domain containing 8 [Source:HGNC Symbol;Acc:HGNC:26036]
DBR1	debranching RNA lariats 1 [Source:HGNC Symbol;Acc:HGNC:15594]
DNAAF5	dynein (axonemal) assembly factor 5 [Source:HGNC Symbol;Acc:HGNC:26013]
E4F1	E4F transcription factor 1 [Source:HGNC Symbol;Acc:HGNC:3121]
IFT22	intraflagellar transport 22 [Source:HGNC Symbol;Acc:HGNC:21895]
KLRG2	killer cell lectin like receptor G2 [Source:HGNC Symbol;Acc:HGNC:24778]
LYRM9	LYR motif containing 9 [Source:HGNC Symbol;Acc:HGNC:27314]
MRPS15	mitochondrial ribosomal protein S15 [Source:HGNC Symbol;Acc:HGNC:14504]
RDH13	retinol dehydrogenase 13 (all-trans/9-cis) [Source:HGNC Symbol;Acc:HGNC:19978]
RNF185	ring finger protein 185 [Source:HGNC Symbol;Acc:HGNC:26783]
UBE4A	ubiquitination factor E4A [Source:HGNC Symbol;Acc:HGNC:12499]
USE1	unconventional SNARE in the ER 1 [Source:HGNC Symbol;Acc:HGNC:30882]
XAB2	XPA binding protein 2 [Source:HGNC Symbol;Acc:HGNC:14089]
ZDHHC7	zinc finger DHHC-type containing 7 [Source:HGNC Symbol;Acc:HGNC:18459]
ZNF148	zinc finger protein 148 [Source:HGNC Symbol;Acc:HGNC:12933]
EMB	embigin [Source:HGNC Symbol;Acc:HGNC:30465]

GCNT2	glucosaminyl (N-acetyl) transferase 2, I-branching enzyme (I blood group) [Source:HGNC Symbol;Acc:HGNC:4204]
GNG11	G protein subunit gamma 11 [Source:HGNC Symbol;Acc:HGNC:4403]
KCNK1	potassium two pore domain channel subfamily K member 1 [Source:HGNC Symbol;Acc:HGNC:6272]
LAMP3	lysosomal associated membrane protein 3 [Source:HGNC Symbol;Acc:HGNC:14582]
PTGIS	prostaglandin I2 (prostacyclin) synthase [Source:HGNC Symbol;Acc:HGNC:9603]
ST14	suppression of tumorigenicity 14 [Source:HGNC Symbol;Acc:HGNC:11344]
STEAP1	six transmembrane epithelial antigen of the prostate 1 [Source:HGNC Symbol;Acc:HGNC:11378]
SYNJ2	synaptojanin 2 [Source:HGNC Symbol;Acc:HGNC:11504]
TACSTD2	tumor-associated calcium signal transducer 2 [Source:HGNC Symbol;Acc:HGNC:11530]
AR	androgen receptor [Source:HGNC Symbol;Acc:HGNC:644]
ATP9B	ATPase phospholipid transporting 9B (putative) [Source:HGNC Symbol;Acc:HGNC:13541]
C1orf112	chromosome 1 open reading frame 112 [Source:HGNC Symbol;Acc:HGNC:25565]
CCDC137	coiled-coil domain containing 137 [Source:HGNC Symbol;Acc:HGNC:33451]
CLTC	clathrin heavy chain [Source:HGNC Symbol;Acc:HGNC:2092]
HNRNPUL2	heterogeneous nuclear ribonucleoprotein U-like 2 [Source:HGNC Symbol;Acc:HGNC:25451]
INTS4	integrator complex subunit 4 [Source:HGNC Symbol;Acc:HGNC:25048]
KRI1	KRI1 homolog [Source:HGNC Symbol;Acc:HGNC:25769]
MAD2L1BP	MAD2L1 binding protein [Source:HGNC Symbol;Acc:HGNC:21059]
MTX1	metaxin 1 [Source:HGNC Symbol;Acc:HGNC:7504]
SLC25A19	solute carrier family 25 member 19 [Source:HGNC Symbol;Acc:HGNC:14409]
NOL12	nucleolar protein 12 [Source:HGNC Symbol;Acc:HGNC:28585]
NYNRIN	NYN domain and retroviral integrase containing [Source:HGNC Symbol;Acc:HGNC:20165]
PAF1	PAF1 homolog, Paf1/RNA polymerase II complex component [Source:HGNC Symbol;Acc:HGNC:25459]
PSMD10	proteasome 26S subunit, non-ATPase 10 [Source:HGNC Symbol;Acc:HGNC:9555]
SEC14L3	SEC14 like lipid binding 3 [Source:HGNC Symbol;Acc:HGNC:18655]
TNFAIP8L2	TNF alpha induced protein 8 like 2 [Source:HGNC Symbol;Acc:HGNC:26277]
UBE2QL1	ubiquitin conjugating enzyme E2 Q family like 1 [Source:HGNC Symbol;Acc:HGNC:37269]
VPS25	vacuolar protein sorting 25 homolog [Source:HGNC Symbol;Acc:HGNC:28122]
AAMP	angio associated migratory cell protein [Source:HGNC Symbol;Acc:HGNC:18]
ADAP1	ArfGAP with dual PH domains 1 [Source:HGNC Symbol;Acc:HGNC:16486]
FEM1B	fem-1 homolog B [Source:HGNC Symbol;Acc:HGNC:3649]
FLNC	filamin C [Source:HGNC Symbol;Acc:HGNC:3756]
LZTS2	leucine zipper, putative tumor suppressor 2 [Source:HGNC Symbol;Acc:HGNC:29381]
MAL2	mal, T-cell differentiation protein 2 (gene/pseudogene) [Source:HGNC Symbol;Acc:HGNC:13634]

MLH3	mutL homolog 3 [Source:HGNC Symbol;Acc:HGNC:7128]
NEK6	NIMA related kinase 6 [Source:HGNC Symbol;Acc:HGNC:7749]
POLR2L	polymerase (RNA) II subunit L [Source:HGNC Symbol;Acc:HGNC:9199]
PYGB	phosphorylase, glycogen; brain [Source:HGNC Symbol;Acc:HGNC:9723]
SH3TC2	SH3 domain and tetratricopeptide repeats 2 [Source:HGNC Symbol;Acc:HGNC:29427]
SSBP4	single stranded DNA binding protein 4 [Source:HGNC Symbol;Acc:HGNC:15676]
UBE2D1	ubiquitin conjugating enzyme E2 D1 [Source:HGNC Symbol;Acc:HGNC:12474]
ACAD8	acyl-CoA dehydrogenase family member 8 [Source:HGNC Symbol;Acc:HGNC:87]
B4GALT7	beta-1,4-galactosyltransferase 7 [Source:HGNC Symbol;Acc:HGNC:930]
CASKIN1	CASK interacting protein 1 [Source:HGNC Symbol;Acc:HGNC:20879]
CEP83	centrosomal protein 83 [Source:HGNC Symbol;Acc:HGNC:17966]
CFAP20	cilia and flagella associated protein 20 [Source:HGNC Symbol;Acc:HGNC:29523]
COL22A1	collagen type XXII alpha 1 [Source:HGNC Symbol;Acc:HGNC:22989]
CXADR	coxsackie virus and adenovirus receptor [Source:HGNC Symbol;Acc:HGNC:2559]
FSCN1	fascin actin-bundling protein 1 [Source:HGNC Symbol;Acc:HGNC:11148]
HIRA	histone cell cycle regulator [Source:HGNC Symbol;Acc:HGNC:4916]
MESP1	mesoderm posterior bHLH transcription factor 1 [Source:HGNC Symbol;Acc:HGNC:29658]
MRPS14	mitochondrial ribosomal protein S14 [Source:HGNC Symbol;Acc:HGNC:14049]
GBAS	glioblastoma amplified sequence [Source:HGNC Symbol;Acc:HGNC:4179]
PATZ1	POZ/BTB and AT hook containing zinc finger 1 [Source:HGNC Symbol;Acc:HGNC:13071]
PCNP	PEST proteolytic signal containing nuclear protein [Source:HGNC Symbol;Acc:HGNC:30023]
PIGC	phosphatidylinositol glycan anchor biosynthesis class C [Source:HGNC Symbol;Acc:HGNC:8960]
QKI	QKI, KH domain containing, RNA binding [Source:HGNC Symbol;Acc:HGNC:21100]
SAMHD1	SAM and HD domain containing deoxynucleoside triphosphate triphosphohydrolase 1 [Source:HGNC Symbol;Acc:HGNC:15925]
SLC6A3	solute carrier family 6 member 3 [Source:HGNC Symbol;Acc:HGNC:11049]
TFAP2B	transcription factor AP-2 beta [Source:HGNC Symbol;Acc:HGNC:11743]
VILL	villin-like [Source:HGNC Symbol;Acc:HGNC:30906]
WWOX	WW domain containing oxidoreductase [Source:HGNC Symbol;Acc:HGNC:12799]
CDX1	caudal type homeobox 1 [Source:HGNC Symbol;Acc:HGNC:1805]
DNAJB14	DnaJ heat shock protein family (Hsp40) member B14 [Source:HGNC Symbol;Acc:HGNC:25881]
EDARADD	EDAR-associated death domain [Source:HGNC Symbol;Acc:HGNC:14341]
IPO13	importin 13 [Source:HGNC Symbol;Acc:HGNC:16853]
MED28	mediator complex subunit 28 [Source:HGNC Symbol;Acc:HGNC:24628]
MEMO1	mediator of cell motility 1 [Source:HGNC Symbol;Acc:HGNC:14014]
MEP1A	meprin A subunit alpha [Source:HGNC Symbol;Acc:HGNC:7015]
MRPL27	mitochondrial ribosomal protein L27 [Source:HGNC Symbol;Acc:HGNC:14483]
MRPL36	mitochondrial ribosomal protein L36 [Source:HGNC Symbol;Acc:HGNC:14490]

NDUFAF3	NADH:ubiquinone oxidoreductase complex assembly factor 3 [Source:HGNC Symbol;Acc:HGNC:29918]
NECAP2	NECAP endocytosis associated 2 [Source:HGNC Symbol;Acc:HGNC:25528]
RCCD1	RCC1 domain containing 1 [Source:HGNC Symbol;Acc:HGNC:30457]
TGM3	transglutaminase 3 [Source:HGNC Symbol;Acc:HGNC:11779]
TRABD	TraB domain containing [Source:HGNC Symbol;Acc:HGNC:28805]
TRAPPC3	trafficking protein particle complex 3 [Source:HGNC Symbol;Acc:HGNC:19942]
ZKSCAN3	zinc finger with KRAB and SCAN domains 3 [Source:HGNC Symbol;Acc:HGNC:13853]
ZNRF2	zinc and ring finger 2, E3 ubiquitin protein ligase [Source:HGNC Symbol;Acc:HGNC:22316]
CCL28	C-C motif chemokine ligand 28 [Source:HGNC Symbol;Acc:HGNC:17700]
CLMN	calmin (calponin-like, transmembrane) [Source:HGNC Symbol;Acc:HGNC:19972]
COL23A1	collagen type XXIII alpha 1 chain [Source:HGNC Symbol;Acc:HGNC:22990]
DOK5	docking protein 5 [Source:HGNC Symbol;Acc:HGNC:16173]
KRT6B	keratin 6B [Source:HGNC Symbol;Acc:HGNC:6444]
MARS2	methionyl-tRNA synthetase 2, mitochondrial [Source:HGNC Symbol;Acc:HGNC:25133]
OSBP	oxysterol binding protein [Source:HGNC Symbol;Acc:HGNC:8503]
SIGIRR	single immunoglobulin and toll-interleukin 1 receptor (TIR) domain [Source:HGNC Symbol;Acc:HGNC:30575]
STAC2	SH3 and cysteine rich domain 2 [Source:HGNC Symbol;Acc:HGNC:23990]
SYNE2	spectrin repeat containing nuclear envelope protein 2 [Source:HGNC Symbol;Acc:HGNC:17084]
TRNP1	TMF1-regulated nuclear protein 1 [Source:HGNC Symbol;Acc:HGNC:34348]
TXNDC17	thioredoxin domain containing 17 [Source:HGNC Symbol;Acc:HGNC:28218]
UCN	urocortin [Source:HGNC Symbol;Acc:HGNC:12516]
ACSM2A	acyl-CoA synthetase medium-chain family member 2A [Source:HGNC Symbol;Acc:HGNC:32017]
ATP6V1G2	ATPase H+ transporting V1 subunit G2 [Source:HGNC Symbol;Acc:HGNC:862]
BIRC7	baculoviral IAP repeat containing 7 [Source:HGNC Symbol;Acc:HGNC:13702]
C3orf52	chromosome 3 open reading frame 52 [Source:HGNC Symbol;Acc:HGNC:26255]
CERS2	ceramide synthase 2 [Source:HGNC Symbol;Acc:HGNC:14076]
DCAF4	DDB1 and CUL4 associated factor 4 [Source:HGNC Symbol;Acc:HGNC:20229]
DCLRE1B	DNA cross-link repair 1B [Source:HGNC Symbol;Acc:HGNC:17641]
DHX16	DEAH-box helicase 16 [Source:HGNC Symbol;Acc:HGNC:2739]
EXOSC10	exosome component 10 [Source:HGNC Symbol;Acc:HGNC:9138]
KLK2	kallikrein related peptidase 2 [Source:HGNC Symbol;Acc:HGNC:6363]
LSM8	LSM8 homolog, U6 small nuclear RNA associated [Source:HGNC Symbol;Acc:HGNC:20471]
MNDA	myeloid cell nuclear differentiation antigen [Source:HGNC Symbol;Acc:HGNC:7183]
MZT1	mitotic spindle organizing protein 1 [Source:HGNC Symbol;Acc:HGNC:33830]
NEMP1	nuclear envelope integral membrane protein 1 [Source:HGNC Symbol;Acc:HGNC:29001]

NTMT1	N-terminal Xaa-Pro-Lys N-methyltransferase 1 [Source:HGNC Symbol;Acc:HGNC:23373]
NVL	nuclear VCP-like [Source:HGNC Symbol;Acc:HGNC:8070]
RAB4B	RAB4B, member RAS oncogene family [Source:HGNC Symbol;Acc:HGNC:9782]
RNPC3	RNA binding region (RNP1, RRM) containing 3 [Source:HGNC Symbol;Acc:HGNC:18666]
SMU1	DNA replication regulator and spliceosomal factor [Source:HGNC Symbol;Acc:HGNC:18247]
SNRNP48	small nuclear ribonucleoprotein U11/U12 subunit 48 [Source:HGNC Symbol;Acc:HGNC:21368]
SPRYD4	SPRY domain containing 4 [Source:HGNC Symbol;Acc:HGNC:27468]
TBCA	tubulin folding cofactor A [Source:HGNC Symbol;Acc:HGNC:11579]
TM4SF5	transmembrane 4 L six family member 5 [Source:HGNC Symbol;Acc:HGNC:11857]
ZC3H14	zinc finger CCCH-type containing 14 [Source:HGNC Symbol;Acc:HGNC:20509]
BHLHB9	basic helix-loop-helix domain containing, class B, 9 [Source:HGNC Symbol;Acc:HGNC:29353]
FOXL2	forkhead box L2 [Source:HGNC Symbol;Acc:HGNC:1092]
IRX2	iroquois homeobox 2 [Source:HGNC Symbol;Acc:HGNC:14359]
KCNC1	potassium voltage-gated channel subfamily C member 1 [Source:HGNC Symbol;Acc:HGNC:6233]
LMO7	LIM domain 7 [Source:HGNC Symbol;Acc:HGNC:6646]
MYO1G	myosin IG [Source:HGNC Symbol;Acc:HGNC:13880]
NUP35	nucleoporin 35 [Source:HGNC Symbol;Acc:HGNC:29797]
PAX2	paired box 2 [Source:HGNC Symbol;Acc:HGNC:8616]
PCYOX1L	prenylcysteine oxidase 1 like [Source:HGNC Symbol;Acc:HGNC:28477]
RAD1	RAD1 checkpoint DNA exonuclease [Source:HGNC Symbol;Acc:HGNC:9806]
RHOV	ras homolog family member V [Source:HGNC Symbol;Acc:HGNC:18313]
SLC39A7	solute carrier family 39 member 7 [Source:HGNC Symbol;Acc:HGNC:4927]
SLC9A8	solute carrier family 9 member A8 [Source:HGNC Symbol;Acc:HGNC:20728]
SURF6	surfeit 6 [Source:HGNC Symbol;Acc:HGNC:11478]
UBR5	ubiquitin protein ligase E3 component n-recognin 5 [Source:HGNC Symbol;Acc:HGNC:16806]
USP39	ubiquitin specific peptidase 39 [Source:HGNC Symbol;Acc:HGNC:20071]
VAT1	vesicle amine transport 1 [Source:HGNC Symbol;Acc:HGNC:16919]
WAPL	WAPL cohesin release factor [Source:HGNC Symbol;Acc:HGNC:23293]
ACOT8	acyl-CoA thioesterase 8 [Source:HGNC Symbol;Acc:HGNC:15919]
ADGRF1	adhesion G protein-coupled receptor F1 [Source:HGNC Symbol;Acc:HGNC:18990]
B3GAT2	beta-1,3-glucuronyltransferase 2 [Source:HGNC Symbol;Acc:HGNC:922]
DBNDD2	dysbindin domain containing 2 [Source:HGNC Symbol;Acc:HGNC:15881]
EXOC2	exocyst complex component 2 [Source:HGNC Symbol;Acc:HGNC:24968]
F11R	F11 receptor [Source:HGNC Symbol;Acc:HGNC:14685]
GNPDA2	glucosamine-6-phosphate deaminase 2 [Source:HGNC Symbol;Acc:HGNC:21526]
HRK	harakiri, BCL2 interacting protein [Source:HGNC Symbol;Acc:HGNC:5185]

KMT5A	lysine methyltransferase 5A [Source:HGNC Symbol;Acc:HGNC:29489]
LSM10	LSM10, U7 small nuclear RNA associated [Source:HGNC Symbol;Acc:HGNC:17562]
MGAT3	mannosyl (beta-1,4-)-glycoprotein beta-1,4-N-acetylglucosaminyltransferase [Source:HGNC Symbol;Acc:HGNC:7046]
MRPL23	mitochondrial ribosomal protein L23 [Source:HGNC Symbol;Acc:HGNC:10322]
NEDD8	neural precursor cell expressed, developmentally down-regulated 8 [Source:HGNC Symbol;Acc:HGNC:7732]
SORBS1	sorbin and SH3 domain containing 1 [Source:HGNC Symbol;Acc:HGNC:14565]
SULT1C2	sulfotransferase family 1C member 2 [Source:HGNC Symbol;Acc:HGNC:11456]
TBCB	tubulin folding cofactor B [Source:HGNC Symbol;Acc:HGNC:1989]
UGT1A6	UDP glucuronosyltransferase family 1 member A6 [Source:HGNC Symbol;Acc:HGNC:12538]
BCL2L12	BCL2 like 12 [Source:HGNC Symbol;Acc:HGNC:13787]
COX4I2	cytochrome c oxidase subunit 4I2 [Source:HGNC Symbol;Acc:HGNC:16232]
DCLRE1A	DNA cross-link repair 1A [Source:HGNC Symbol;Acc:HGNC:17660]
DCUN1D5	defective in cullin neddylation 1 domain containing 5 [Source:HGNC Symbol;Acc:HGNC:28409]
MEAF6	MYST/Esa1 associated factor 6 [Source:HGNC Symbol;Acc:HGNC:25674]
MED29	mediator complex subunit 29 [Source:HGNC Symbol;Acc:HGNC:23074]
MTNR1B	melatonin receptor 1B [Source:HGNC Symbol;Acc:HGNC:7464]
PYGO2	pygopus family PHD finger 2 [Source:HGNC Symbol;Acc:HGNC:30257]
RAB33B	RAB33B, member RAS oncogene family [Source:HGNC Symbol;Acc:HGNC:16075]
RGS13	regulator of G-protein signaling 13 [Source:HGNC Symbol;Acc:HGNC:9995]
RPRD2	regulation of nuclear pre-mRNA domain containing 2 [Source:HGNC Symbol;Acc:HGNC:29039]
SBF1	SET binding factor 1 [Source:HGNC Symbol;Acc:HGNC:10542]
SCAF1	SR-related CTD associated factor 1 [Source:HGNC Symbol;Acc:HGNC:30403]
STX4	syntaxin 4 [Source:HGNC Symbol;Acc:HGNC:11439]
TMEM11	transmembrane protein 11 [Source:HGNC Symbol;Acc:HGNC:16823]
TOMM22	translocase of outer mitochondrial membrane 22 [Source:HGNC Symbol;Acc:HGNC:18002]
TTC9C	tetratricopeptide repeat domain 9C [Source:HGNC Symbol;Acc:HGNC:28432]
AKIRIN2	akirin 2 [Source:HGNC Symbol;Acc:HGNC:21407]
ARHGAP35	Rho GTPase activating protein 35 [Source:HGNC Symbol;Acc:HGNC:4591]
CASP14	caspase 14 [Source:HGNC Symbol;Acc:HGNC:1502]
CCL17	C-C motif chemokine ligand 17 [Source:HGNC Symbol;Acc:HGNC:10615]
CLCA2	chloride channel accessory 2 [Source:HGNC Symbol;Acc:HGNC:2016]
CLDN7	claudin 7 [Source:HGNC Symbol;Acc:HGNC:2049]
D2HGDH	D-2-hydroxyglutarate dehydrogenase [Source:HGNC Symbol;Acc:HGNC:28358]
DUSP13	dual specificity phosphatase 13 [Source:HGNC Symbol;Acc:HGNC:19681]
FOXQ1	forkhead box Q1 [Source:HGNC Symbol;Acc:HGNC:20951]
GDA	guanine deaminase [Source:HGNC Symbol;Acc:HGNC:4212]
NDN	necdin, MAGE family member [Source:HGNC Symbol;Acc:HGNC:7675]

NPRL3	NPR3 like, GATOR1 complex subunit [Source:HGNC Symbol;Acc:HGNC:14124]
PGC	progastricsin [Source:HGNC Symbol;Acc:HGNC:8890]
PIGQ	phosphatidylinositol glycan anchor biosynthesis class Q [Source:HGNC Symbol;Acc:HGNC:14135]
RNF39	ring finger protein 39 [Source:HGNC Symbol;Acc:HGNC:18064]
SH3KBP1	SH3 domain containing kinase binding protein 1 [Source:HGNC Symbol;Acc:HGNC:13867]
SLC15A4	solute carrier family 15 member 4 [Source:HGNC Symbol;Acc:HGNC:23090]
SLC5A2	solute carrier family 5 member 2 [Source:HGNC Symbol;Acc:HGNC:11037]
TREM1	triggering receptor expressed on myeloid cells 1 [Source:HGNC Symbol;Acc:HGNC:17760]
ALG9	ALG9, alpha-1,2-mannosyltransferase [Source:HGNC Symbol;Acc:HGNC:15672]
ATP13A1	ATPase 13A1 [Source:HGNC Symbol;Acc:HGNC:24215]
ATP5E	ATP synthase, H+ transporting, mitochondrial F1 complex, epsilon subunit [Source:HGNC Symbol;Acc:HGNC:838]
CCDC12	coiled-coil domain containing 12 [Source:HGNC Symbol;Acc:HGNC:28332]
CCDC88B	coiled-coil domain containing 88B [Source:HGNC Symbol;Acc:HGNC:26757]
DGUOK	deoxyguanosine kinase [Source:HGNC Symbol;Acc:HGNC:2858]
DHX38	DEAH-box helicase 38 [Source:HGNC Symbol;Acc:HGNC:17211]
LRRC39	leucine rich repeat containing 39 [Source:HGNC Symbol;Acc:HGNC:28228]
MRPS11	mitochondrial ribosomal protein S11 [Source:HGNC Symbol;Acc:HGNC:14050]
NKX2-2	NK2 homeobox 2 [Source:HGNC Symbol;Acc:HGNC:7835]
NKX6-1	NK6 homeobox 1 [Source:HGNC Symbol;Acc:HGNC:7839]
PIGZ	phosphatidylinositol glycan anchor biosynthesis class Z [Source:HGNC Symbol;Acc:HGNC:30596]
PTCHD4	patched domain containing 4 [Source:HGNC Symbol;Acc:HGNC:21345]
SEC11A	SEC11 homolog A, signal peptidase complex subunit [Source:HGNC Symbol;Acc:HGNC:17718]
SNX18	sorting nexin 18 [Source:HGNC Symbol;Acc:HGNC:19245]
TBC1D7	TBC1 domain family member 7 [Source:HGNC Symbol;Acc:HGNC:21066]
ASCL2	achaete-scute family bHLH transcription factor 2 [Source:HGNC Symbol;Acc:HGNC:739]
CADM3	cell adhesion molecule 3 [Source:HGNC Symbol;Acc:HGNC:17601]
CYP2A6	cytochrome P450 family 2 subfamily A member 6 [Source:HGNC Symbol;Acc:HGNC:2610]
DAZL	deleted in azoospermia like [Source:HGNC Symbol;Acc:HGNC:2685]
DENND2C	DENN domain containing 2C [Source:HGNC Symbol;Acc:HGNC:24748]
DIS3	DIS3 homolog, exosome endoribonuclease and 3'-5' exoribonuclease [Source:HGNC Symbol;Acc:HGNC:20604]
FAXDC2	fatty acid hydroxylase domain containing 2 [Source:HGNC Symbol;Acc:HGNC:1334]
FRMD3	FERM domain containing 3 [Source:HGNC Symbol;Acc:HGNC:24125]
GLIPR1	GLI pathogenesis related 1 [Source:HGNC Symbol;Acc:HGNC:17001]
GRM8	glutamate metabotropic receptor 8 [Source:HGNC Symbol;Acc:HGNC:4600]

LSP1	lymphocyte-specific protein 1 [Source:HGNC Symbol;Acc:HGNC:6707]
MED30	mediator complex subunit 30 [Source:HGNC Symbol;Acc:HGNC:23032]
OSTF1	osteoclast stimulating factor 1 [Source:HGNC Symbol;Acc:HGNC:8510]
RAB34	RAB34, member RAS oncogene family [Source:HGNC Symbol;Acc:HGNC:16519]
RABGGTA	Rab geranylgeranyltransferase alpha subunit [Source:HGNC Symbol;Acc:HGNC:9795]
RARG	retinoic acid receptor gamma [Source:HGNC Symbol;Acc:HGNC:9866]
RMDN1	regulator of microtubule dynamics 1 [Source:HGNC Symbol;Acc:HGNC:24285]
RUNDC3B	RUN domain containing 3B [Source:HGNC Symbol;Acc:HGNC:30286]
SCTR	secretin receptor [Source:HGNC Symbol;Acc:HGNC:10608]
ABCD4	ATP binding cassette subfamily D member 4 [Source:HGNC Symbol;Acc:HGNC:68]
ATP5C1	ATP synthase, H+ transporting, mitochondrial F1 complex, gamma polypeptide 1 [Source:HGNC Symbol;Acc:HGNC:833]
CCR9	C-C motif chemokine receptor 9 [Source:HGNC Symbol;Acc:HGNC:1610]
COL21A1	collagen type XXI alpha 1 chain [Source:HGNC Symbol;Acc:HGNC:17025]
CPSF1	cleavage and polyadenylation specific factor 1 [Source:HGNC Symbol;Acc:HGNC:2324]
DCTN5	dynactin subunit 5 [Source:HGNC Symbol;Acc:HGNC:24594]
EDRF1	erythroid differentiation regulatory factor 1 [Source:HGNC Symbol;Acc:HGNC:24640]
ELP5	elongator acetyltransferase complex subunit 5 [Source:HGNC Symbol;Acc:HGNC:30617]
EMC8	ER membrane protein complex subunit 8 [Source:HGNC Symbol;Acc:HGNC:7864]
FAM149B1	family with sequence similarity 149 member B1 [Source:HGNC Symbol;Acc:HGNC:29162]
FCER1A	Fc fragment of IgE receptor Ia [Source:HGNC Symbol;Acc:HGNC:3609]
GLRX5	glutaredoxin 5 [Source:HGNC Symbol;Acc:HGNC:20134]
GPRIN1	G protein regulated inducer of neurite outgrowth 1 [Source:HGNC Symbol;Acc:HGNC:24835]
HCCS	holocytochrome c synthase [Source:HGNC Symbol;Acc:HGNC:4837]
INTS2	integrator complex subunit 2 [Source:HGNC Symbol;Acc:HGNC:29241]
INTS8	integrator complex subunit 8 [Source:HGNC Symbol;Acc:HGNC:26048]
IRGM	immunity-related GTPase M [Source:HGNC Symbol;Acc:HGNC:29597]
LMAN2	lectin, mannose binding 2 [Source:HGNC Symbol;Acc:HGNC:16986]
LMF2	lipase maturation factor 2 [Source:HGNC Symbol;Acc:HGNC:25096]
NIM1K	NIM1 serine/threonine protein kinase [Source:HGNC Symbol;Acc:HGNC:28646]
RAB2B	RAB2B, member RAS oncogene family [Source:HGNC Symbol;Acc:HGNC:20246]
SLC45A2	solute carrier family 45 member 2 [Source:HGNC Symbol;Acc:HGNC:16472]
SNRPB2	small nuclear ribonucleoprotein polypeptide B2 [Source:HGNC Symbol;Acc:HGNC:11155]
SYNE4	spectrin repeat containing nuclear envelope family member 4 [Source:HGNC Symbol;Acc:HGNC:26703]
TMEM40	transmembrane protein 40 [Source:HGNC Symbol;Acc:HGNC:25620]
TOMM34	translocase of outer mitochondrial membrane 34 [Source:HGNC Symbol;Acc:HGNC:15746]
ANXA6	annexin A6 [Source:HGNC Symbol;Acc:HGNC:544]

DCAF6	DDB1 and CUL4 associated factor 6 [Source:HGNC Symbol;Acc:HGNC:30002]
EIF2B5	eukaryotic translation initiation factor 2B subunit epsilon [Source:HGNC Symbol;Acc:HGNC:3261]
FKBP9	FK506 binding protein 9 [Source:HGNC Symbol;Acc:HGNC:3725]
GREM1	gremlin 1, DAN family BMP antagonist [Source:HGNC Symbol;Acc:HGNC:2001]
HS3ST2	heparan sulfate-glucosamine 3-sulfotransferase 2 [Source:HGNC Symbol;Acc:HGNC:5195]
IL1RAPL1	interleukin 1 receptor accessory protein like 1 [Source:HGNC Symbol;Acc:HGNC:5996]
IMP3	IMP3, U3 small nucleolar ribonucleoprotein [Source:HGNC Symbol;Acc:HGNC:14497]
LDHC	lactate dehydrogenase C [Source:HGNC Symbol;Acc:HGNC:6544]
LUZP1	leucine zipper protein 1 [Source:HGNC Symbol;Acc:HGNC:14985]
MED20	mediator complex subunit 20 [Source:HGNC Symbol;Acc:HGNC:16840]
MMP17	matrix metallopeptidase 17 [Source:HGNC Symbol;Acc:HGNC:7163]
MTHFD2L	methylenetetrahydrofolate dehydrogenase (NADP+ dependent) 2-like [Source:HGNC Symbol;Acc:HGNC:31865]
NOTCH3	notch 3 [Source:HGNC Symbol;Acc:HGNC:7883]
PPIL4	peptidylprolyl isomerase like 4 [Source:HGNC Symbol;Acc:HGNC:15702]
SERPINA4	serpin family A member 4 [Source:HGNC Symbol;Acc:HGNC:8948]
SLC5A12	solute carrier family 5 member 12 [Source:HGNC Symbol;Acc:HGNC:28750]
THEM4	thioesterase superfamily member 4 [Source:HGNC Symbol;Acc:HGNC:17947]
TSPYL4	TSPY-like 4 [Source:HGNC Symbol;Acc:HGNC:21559]
ATP5G1	ATP synthase, H+ transporting, mitochondrial Fo complex subunit C1 (subunit 9) [Source:HGNC Symbol;Acc:HGNC:841]
CENPO	centromere protein O [Source:HGNC Symbol;Acc:HGNC:28152]
COPS3	COP9 signalosome subunit 3 [Source:HGNC Symbol;Acc:HGNC:2239]
EIF3L	eukaryotic translation initiation factor 3 subunit L [Source:HGNC Symbol;Acc:HGNC:18138]
FOXRED1	FAD dependent oxidoreductase domain containing 1 [Source:HGNC Symbol;Acc:HGNC:26927]
GALNT4	polypeptide N-acetylgalactosaminyltransferase 4 [Source:HGNC Symbol;Acc:HGNC:4126]
GRAMD1A	GRAM domain containing 1A [Source:HGNC Symbol;Acc:HGNC:29305]
INTS3	integrator complex subunit 3 [Source:HGNC Symbol;Acc:HGNC:26153]
KATNAL1	katanin catalytic subunit A1 like 1 [Source:HGNC Symbol;Acc:HGNC:28361]
KCNK12	potassium two pore domain channel subfamily K member 12 [Source:HGNC Symbol;Acc:HGNC:6274]
MED24	mediator complex subunit 24 [Source:HGNC Symbol;Acc:HGNC:22963]
MGA	MGA, MAX dimerization protein [Source:HGNC Symbol;Acc:HGNC:14010]
MORF4L1	mortality factor 4 like 1 [Source:HGNC Symbol;Acc:HGNC:16989]
MRPL11	mitochondrial ribosomal protein L11 [Source:HGNC Symbol;Acc:HGNC:14042]
NBEA	neurobeachin [Source:HGNC Symbol;Acc:HGNC:7648]
PBDC1	polysaccharide biosynthesis domain containing 1 [Source:HGNC Symbol;Acc:HGNC:28790]

PIP4K2C	phosphatidylinositol-5-phosphate 4-kinase type 2 gamma [Source:HGNC Symbol;Acc:HGNC:23786]
PPP2R5D	protein phosphatase 2 regulatory subunit B'delta [Source:HGNC Symbol;Acc:HGNC:9312]
PQBP1	polyglutamine binding protein 1 [Source:HGNC Symbol;Acc:HGNC:9330]
PROM2	prominin 2 [Source:HGNC Symbol;Acc:HGNC:20685]
TBX6	T-box 6 [Source:HGNC Symbol;Acc:HGNC:11605]
TNFRSF10C	tumor necrosis factor receptor superfamily member 10c [Source:HGNC Symbol;Acc:HGNC:11906]
TSN	translin [Source:HGNC Symbol;Acc:HGNC:12379]
ARHGAP23	Rho GTPase activating protein 23 [Source:HGNC Symbol;Acc:HGNC:29293]
ATPAF1	ATP synthase mitochondrial F1 complex assembly factor 1 [Source:HGNC Symbol;Acc:HGNC:18803]
BCAS1	breast carcinoma amplified sequence 1 [Source:HGNC Symbol;Acc:HGNC:974]
CACNA1E	calcium voltage-gated channel subunit alpha1 E [Source:HGNC Symbol;Acc:HGNC:1392]
CIART	circadian associated repressor of transcription [Source:HGNC Symbol;Acc:HGNC:25200]
CORIN	corin, serine peptidase [Source:HGNC Symbol;Acc:HGNC:19012]
CYP4F3	cytochrome P450 family 4 subfamily F member 3 [Source:HGNC Symbol;Acc:HGNC:2646]
ERBIN	erbb2 interacting protein [Source:HGNC Symbol;Acc:HGNC:15842]
EXOSC4	exosome component 4 [Source:HGNC Symbol;Acc:HGNC:18189]
FRMD8	FERM domain containing 8 [Source:HGNC Symbol;Acc:HGNC:25462]
GABRA6	gamma-aminobutyric acid type A receptor alpha6 subunit [Source:HGNC Symbol;Acc:HGNC:4080]
METTL1	methyltransferase like 1 [Source:HGNC Symbol;Acc:HGNC:7030]
MT1B	metallothionein 1B [Source:HGNC Symbol;Acc:HGNC:7394]
MT4	metallothionein 4 [Source:HGNC Symbol;Acc:HGNC:18705]
MTSS1	metastasis suppressor 1 [Source:HGNC Symbol;Acc:HGNC:20443]
MYH4	myosin, heavy chain 4, skeletal muscle [Source:HGNC Symbol;Acc:HGNC:7574]
NELFA	negative elongation factor complex member A [Source:HGNC Symbol;Acc:HGNC:12768]
NMNAT2	nicotinamide nucleotide adenylyltransferase 2 [Source:HGNC Symbol;Acc:HGNC:16789]
PAX7	paired box 7 [Source:HGNC Symbol;Acc:HGNC:8621]
SLC19A3	solute carrier family 19 member 3 [Source:HGNC Symbol;Acc:HGNC:16266]
SLC22A17	solute carrier family 22 member 17 [Source:HGNC Symbol;Acc:HGNC:23095]
SLC25A32	solute carrier family 25 member 32 [Source:HGNC Symbol;Acc:HGNC:29683]
SNX25	sorting nexin 25 [Source:HGNC Symbol;Acc:HGNC:21883]
SP110	SP110 nuclear body protein [Source:HGNC Symbol;Acc:HGNC:5401]
SPRY4	sprouty RTK signaling antagonist 4 [Source:HGNC Symbol;Acc:HGNC:15533]
UGT8	UDP glycosyltransferase 8 [Source:HGNC Symbol;Acc:HGNC:12555]
USP16	ubiquitin specific peptidase 16 [Source:HGNC Symbol;Acc:HGNC:12614]

VANGL2	VANGL planar cell polarity protein 2 [Source:HGNC Symbol;Acc:HGNC:15511]
VTCN1	V-set domain containing T cell activation inhibitor 1 [Source:HGNC Symbol;Acc:HGNC:28873]
WSCD1	WSC domain containing 1 [Source:HGNC Symbol;Acc:HGNC:29060]
ALDH3B2	aldehyde dehydrogenase 3 family member B2 [Source:HGNC Symbol;Acc:HGNC:411]
ALKBH2	alkB homolog 2, alpha-ketoglutarate dependent dioxygenase [Source:HGNC Symbol;Acc:HGNC:32487]
ANAPC16	anaphase promoting complex subunit 16 [Source:HGNC Symbol;Acc:HGNC:26976]
BCL2L15	BCL2 like 15 [Source:HGNC Symbol;Acc:HGNC:33624]
CD72	CD72 molecule [Source:HGNC Symbol;Acc:HGNC:1696]
COQ2	coenzyme Q2, polyprenyltransferase [Source:HGNC Symbol;Acc:HGNC:25223]
CTR9	CTR9 homolog, Paf1/RNA polymerase II complex component [Source:HGNC Symbol;Acc:HGNC:16850]
DPM1	dolichyl-phosphate mannosyltransferase polypeptide 1, catalytic subunit [Source:HGNC Symbol;Acc:HGNC:3005]
EXOSC7	exosome component 7 [Source:HGNC Symbol;Acc:HGNC:28112]
FAM135A	family with sequence similarity 135 member A [Source:HGNC Symbol;Acc:HGNC:21084]
GDF1	growth differentiation factor 1 [Source:HGNC Symbol;Acc:HGNC:4214]
GGNBP2	gametogenetin binding protein 2 [Source:HGNC Symbol;Acc:HGNC:19357]
GPAA1	glycosylphosphatidylinositol anchor attachment 1 [Source:HGNC Symbol;Acc:HGNC:4446]
HAUS8	HAUS augmin like complex subunit 8 [Source:HGNC Symbol;Acc:HGNC:30532]
KLRK1	killer cell lectin like receptor K1 [Source:HGNC Symbol;Acc:HGNC:18788]
LDAH	lipid droplet associated hydrolase [Source:HGNC Symbol;Acc:HGNC:26145]
MAGOHB	mago homolog B, exon junction complex core component [Source:HGNC Symbol;Acc:HGNC:25504]
MRPL34	mitochondrial ribosomal protein L34 [Source:HGNC Symbol;Acc:HGNC:14488]
NOL11	nucleolar protein 11 [Source:HGNC Symbol;Acc:HGNC:24557]
NUBP2	nucleotide binding protein 2 [Source:HGNC Symbol;Acc:HGNC:8042]
NUDCD2	NudC domain containing 2 [Source:HGNC Symbol;Acc:HGNC:30535]
NUDT16	nudix hydrolase 16 [Source:HGNC Symbol;Acc:HGNC:26442]
PELP1	proline, glutamate and leucine rich protein 1 [Source:HGNC Symbol;Acc:HGNC:30134]
PRPF4	pre-mRNA processing factor 4 [Source:HGNC Symbol;Acc:HGNC:17349]
PWP1	PWP1 homolog, endonuclein [Source:HGNC Symbol;Acc:HGNC:17015]
RHOT1	ras homolog family member T1 [Source:HGNC Symbol;Acc:HGNC:21168]
RRP1	ribosomal RNA processing 1 [Source:HGNC Symbol;Acc:HGNC:18785]
SMNDC1	survival motor neuron domain containing 1 [Source:HGNC Symbol;Acc:HGNC:16900]
SSH1	slingshot protein phosphatase 1 [Source:HGNC Symbol;Acc:HGNC:30579]
SUSD1	sushi domain containing 1 [Source:HGNC Symbol;Acc:HGNC:25413]
TIMM8A	translocase of inner mitochondrial membrane 8 homolog A (yeast) [Source:HGNC Symbol;Acc:HGNC:11817]

TRPC5	transient receptor potential cation channel subfamily C member 5 [Source:HGNC Symbol;Acc:HGNC:12337]
TSPYL1	TSPY-like 1 [Source:HGNC Symbol;Acc:HGNC:12382]
UTP11	UTP11, small subunit processome component homolog (S. cerevisiae) [Source:HGNC Symbol;Acc:HGNC:24329]
A1BG	alpha-1-B glycoprotein [Source:HGNC Symbol;Acc:HGNC:5]
ADRA2B	adrenoceptor alpha 2B [Source:HGNC Symbol;Acc:HGNC:282]
AGRP	agouti related neuropeptide [Source:HGNC Symbol;Acc:HGNC:330]
ARHGAP19	Rho GTPase activating protein 19 [Source:HGNC Symbol;Acc:HGNC:23724]
COPS4	COP9 signalosome subunit 4 [Source:HGNC Symbol;Acc:HGNC:16702]
GAL3ST1	galactose-3-O-sulfotransferase 1 [Source:HGNC Symbol;Acc:HGNC:24240]
GFER	growth factor, augmenter of liver regeneration [Source:HGNC Symbol;Acc:HGNC:4236]
MAP4K5	mitogen-activated protein kinase kinase kinase kinase 5 [Source:HGNC Symbol;Acc:HGNC:6867]
NRCAM	neuronal cell adhesion molecule [Source:HGNC Symbol;Acc:HGNC:7994]
NTM	neurotrimin [Source:HGNC Symbol;Acc:HGNC:17941]
RASAL1	RAS protein activator like 1 [Source:HGNC Symbol;Acc:HGNC:9873]
RXRB	retinoid X receptor beta [Source:HGNC Symbol;Acc:HGNC:10478]
S100A2	S100 calcium binding protein A2 [Source:HGNC Symbol;Acc:HGNC:10492]
SLC7A9	solute carrier family 7 member 9 [Source:HGNC Symbol;Acc:HGNC:11067]
STK26	serine/threonine protein kinase 26 [Source:HGNC Symbol;Acc:HGNC:18174]
THEM6	thioesterase superfamily member 6 [Source:HGNC Symbol;Acc:HGNC:29656]
TMEM229B	transmembrane protein 229B [Source:HGNC Symbol;Acc:HGNC:20130]
ARHGAP20	Rho GTPase activating protein 20 [Source:HGNC Symbol;Acc:HGNC:18357]
ATP2C1	ATPase secretory pathway Ca2+ transporting 1 [Source:HGNC Symbol;Acc:HGNC:13211]
BANP	BTG3 associated nuclear protein [Source:HGNC Symbol;Acc:HGNC:13450]
BPHL	biphenyl hydrolase-like (serine hydrolase) [Source:HGNC Symbol;Acc:HGNC:1094]
EXPH5	exophilin 5 [Source:HGNC Symbol;Acc:HGNC:30578]
FBXL5	F-box and leucine rich repeat protein 5 [Source:HGNC Symbol;Acc:HGNC:13602]
FOXF2	forkhead box F2 [Source:HGNC Symbol;Acc:HGNC:3810]
GLDC	glycine decarboxylase [Source:HGNC Symbol;Acc:HGNC:4313]
IFI27	interferon alpha inducible protein 27 [Source:HGNC Symbol;Acc:HGNC:5397]
MALL	mal, T-cell differentiation protein like [Source:HGNC Symbol;Acc:HGNC:6818]
MRPL1	mitochondrial ribosomal protein L1 [Source:HGNC Symbol;Acc:HGNC:14275]
PAQR4	progestin and adipoQ receptor family member 4 [Source:HGNC Symbol;Acc:HGNC:26386]
PLEKHH2	pleckstrin homology, MyTH4 and FERM domain containing H2 [Source:HGNC Symbol;Acc:HGNC:30506]
RAB3C	RAB3C, member RAS oncogene family [Source:HGNC Symbol;Acc:HGNC:30269]
UNC5B	unc-5 netrin receptor B [Source:HGNC Symbol;Acc:HGNC:12568]
UNC93B1	unc-93 homolog B1 (C. elegans) [Source:HGNC Symbol;Acc:HGNC:13481]

VPS37B	VPS37B, ESCRT-I subunit [Source:HGNC Symbol;Acc:HGNC:25754]
ALG8	ALG8, alpha-1,3-glucosyltransferase [Source:HGNC Symbol;Acc:HGNC:23161]
ARL2	ADP ribosylation factor like GTPase 2 [Source:HGNC Symbol;Acc:HGNC:693]
ATP5L	ATP synthase, H+ transporting, mitochondrial Fo complex subunit G [Source:HGNC Symbol;Acc:HGNC:14247]
CHODL	chondrolectin [Source:HGNC Symbol;Acc:HGNC:17807]
KCTD20	potassium channel tetramerization domain containing 20 [Source:HGNC Symbol;Acc:HGNC:21052]
LRRC49	leucine rich repeat containing 49 [Source:HGNC Symbol;Acc:HGNC:25965]
MPHOSPH10	M-phase phosphoprotein 10 [Source:HGNC Symbol;Acc:HGNC:7213]
MRPL48	mitochondrial ribosomal protein L48 [Source:HGNC Symbol;Acc:HGNC:16653]
MRPS17	mitochondrial ribosomal protein S17 [Source:HGNC Symbol;Acc:HGNC:14047]
MRPS2	mitochondrial ribosomal protein S2 [Source:HGNC Symbol;Acc:HGNC:14495]
PARN	poly(A)-specific ribonuclease [Source:HGNC Symbol;Acc:HGNC:8609]
PDCD7	programmed cell death 7 [Source:HGNC Symbol;Acc:HGNC:8767]
PIN4	peptidylprolyl cis/trans isomerase, NIMA-interacting 4 [Source:HGNC Symbol;Acc:HGNC:8992]
PPM1E	protein phosphatase, Mg2+/Mn2+ dependent 1E [Source:HGNC Symbol;Acc:HGNC:19322]
PRPF6	pre-mRNA processing factor 6 [Source:HGNC Symbol;Acc:HGNC:15860]
SLC41A3	solute carrier family 41 member 3 [Source:HGNC Symbol;Acc:HGNC:31046]
SPSB3	splA/ryanodine receptor domain and SOCS box containing 3 [Source:HGNC Symbol;Acc:HGNC:30629]
SRP19	signal recognition particle 19kDa [Source:HGNC Symbol;Acc:HGNC:11300]
SSH2	slingshot protein phosphatase 2 [Source:HGNC Symbol;Acc:HGNC:30580]
TOMM70	translocase of outer mitochondrial membrane 70 [Source:HGNC Symbol;Acc:HGNC:11985]
UPK1A	uroplakin 1A [Source:HGNC Symbol;Acc:HGNC:12577]
ZNF703	zinc finger protein 703 [Source:HGNC Symbol;Acc:HGNC:25883]
ACTR2	ARP2 actin-related protein 2 homolog (yeast) [Source:HGNC Symbol;Acc:HGNC:169]
ADH6	alcohol dehydrogenase 6 (class V) [Source:HGNC Symbol;Acc:HGNC:255]
ARHGAP17	Rho GTPase activating protein 17 [Source:HGNC Symbol;Acc:HGNC:18239]
ECHDC3	enoyl-CoA hydratase domain containing 3 [Source:HGNC Symbol;Acc:HGNC:23489]
EGFL6	EGF like domain multiple 6 [Source:HGNC Symbol;Acc:HGNC:3235]
KISS1R	KISS1 receptor [Source:HGNC Symbol;Acc:HGNC:4510]
LAMA5	laminin subunit alpha 5 [Source:HGNC Symbol;Acc:HGNC:6485]
LARGE1	LARGE xylosyl- and glucuronyltransferase 1 [Source:HGNC Symbol;Acc:HGNC:6511]
NPDC1	neural proliferation, differentiation and control, 1 [Source:HGNC Symbol;Acc:HGNC:7899]
OAZ2	ornithine decarboxylase antizyme 2 [Source:HGNC Symbol;Acc:HGNC:8096]
PPP1R13L	protein phosphatase 1 regulatory subunit 13 like [Source:HGNC Symbol;Acc:HGNC:18838]
PROCR	protein C receptor [Source:HGNC Symbol;Acc:HGNC:9452]

RASGEF1B	RasGEF domain family member 1B [Source:HGNC Symbol;Acc:HGNC:24881]
RPL18A	ribosomal protein L18a [Source:HGNC Symbol;Acc:HGNC:10311]
RTN4IP1	reticulon 4 interacting protein 1 [Source:HGNC Symbol;Acc:HGNC:18647]
VWA5A	von Willebrand factor A domain containing 5A [Source:HGNC Symbol;Acc:HGNC:6658]
WDR46	WD repeat domain 46 [Source:HGNC Symbol;Acc:HGNC:13923]
BTF3L4	basic transcription factor 3-like 4 [Source:HGNC Symbol;Acc:HGNC:30547]
CAPN9	calpain 9 [Source:HGNC Symbol;Acc:HGNC:1486]
CSRNP2	cysteine and serine rich nuclear protein 2 [Source:HGNC Symbol;Acc:HGNC:16006]
DDX54	DEAD-box helicase 54 [Source:HGNC Symbol;Acc:HGNC:20084]
FOXRED2	FAD dependent oxidoreductase domain containing 2 [Source:HGNC Symbol;Acc:HGNC:26264]
MEDAG	mesenteric estrogen dependent adipogenesis [Source:HGNC Symbol;Acc:HGNC:25926]
METTL18	methyltransferase like 18 [Source:HGNC Symbol;Acc:HGNC:28793]
MOS	v-mos Moloney murine sarcoma viral oncogene homolog [Source:HGNC Symbol;Acc:HGNC:7199]
MRE11A	MRE11 homolog A, double strand break repair nuclease [Source:HGNC Symbol;Acc:HGNC:7230]
MRPL22	mitochondrial ribosomal protein L22 [Source:HGNC Symbol;Acc:HGNC:14480]
NINL	ninein like [Source:HGNC Symbol;Acc:HGNC:29163]
NKAIN1	Na+/K+ transporting ATPase interacting 1 [Source:HGNC Symbol;Acc:HGNC:25743]
RBM28	RNA binding motif protein 28 [Source:HGNC Symbol;Acc:HGNC:21863]
RCBTB1	RCC1 and BTB domain containing protein 1 [Source:HGNC Symbol;Acc:HGNC:18243]
RFX2	regulatory factor X2 [Source:HGNC Symbol;Acc:HGNC:9983]
RMI1	RecQ mediated genome instability 1 [Source:HGNC Symbol;Acc:HGNC:25764]
SERPINA12	serpin family A member 12 [Source:HGNC Symbol;Acc:HGNC:18359]
TMEM164	transmembrane protein 164 [Source:HGNC Symbol;Acc:HGNC:26217]
ZCCHC11	zinc finger CCHC-type containing 11 [Source:HGNC Symbol;Acc:HGNC:28981]
USP4	ubiquitin specific peptidase 4 [Source:HGNC Symbol;Acc:HGNC:12627]
AGXT2	alanine--glyoxylate aminotransferase 2 [Source:HGNC Symbol;Acc:HGNC:14412]
AMDHD2	amidohydrolase domain containing 2 [Source:HGNC Symbol;Acc:HGNC:24262]
AVIL	advillin [Source:HGNC Symbol;Acc:HGNC:14188]
BCL11B	B-cell CLL/lymphoma 11B [Source:HGNC Symbol;Acc:HGNC:13222]
COX6B2	cytochrome c oxidase subunit 6B2 [Source:HGNC Symbol;Acc:HGNC:24380]
CUBN	cubilin [Source:HGNC Symbol;Acc:HGNC:2548]
EIF2AK1	eukaryotic translation initiation factor 2 alpha kinase 1 [Source:HGNC Symbol;Acc:HGNC:24921]
IFIT2	interferon induced protein with tetratricopeptide repeats 2 [Source:HGNC Symbol;Acc:HGNC:5409]
LGALS3BP	galectin 3 binding protein [Source:HGNC Symbol;Acc:HGNC:6564]
LRRC8C	leucine rich repeat containing 8 family member C [Source:HGNC Symbol;Acc:HGNC:25075]

NDUFA8	NADH:ubiquinone oxidoreductase subunit A8 [Source:HGNC Symbol;Acc:HGNC:7692]
NPR3	natriuretic peptide receptor 3 [Source:HGNC Symbol;Acc:HGNC:7945]
OS9	OS9, endoplasmic reticulum lectin [Source:HGNC Symbol;Acc:HGNC:16994]
RHEBL1	Ras homolog enriched in brain like 1 [Source:HGNC Symbol;Acc:HGNC:21166]
SLC2A13	solute carrier family 2 member 13 [Source:HGNC Symbol;Acc:HGNC:15956]
SLC39A5	solute carrier family 39 member 5 [Source:HGNC Symbol;Acc:HGNC:20502]
AATF	apoptosis antagonizing transcription factor [Source:HGNC Symbol;Acc:HGNC:19235]
BCAS2	breast carcinoma amplified sequence 2 [Source:HGNC Symbol;Acc:HGNC:975]
BRD7	bromodomain containing 7 [Source:HGNC Symbol;Acc:HGNC:14310]
CDKL5	cyclin dependent kinase like 5 [Source:HGNC Symbol;Acc:HGNC:11411]
KIAA1524	KIAA1524 [Source:HGNC Symbol;Acc:HGNC:29302]
CRYZL1	crystallin zeta like 1 [Source:HGNC Symbol;Acc:HGNC:2420]

DDX27	DEAD-box helicase 27 [Source:HGNC Symbol;Acc:HGNC:15837]
DDX50	DEAD-box helicase 50 [Source:HGNC Symbol;Acc:HGNC:17906]
INSM1	INSM transcriptional repressor 1 [Source:HGNC Symbol;Acc:HGNC:6090]
IQCK	IQ motif containing K [Source:HGNC Symbol;Acc:HGNC:28556]
KIF7	kinesin family member 7 [Source:HGNC Symbol;Acc:HGNC:30497]
LIN54	lin-54 DREAM MuvB core complex component [Source:HGNC Symbol;Acc:HGNC:25397]
MRPL15	mitochondrial ribosomal protein L15 [Source:HGNC Symbol;Acc:HGNC:14054]
MRPL16	mitochondrial ribosomal protein L16 [Source:HGNC Symbol;Acc:HGNC:14476]
MRPL40	mitochondrial ribosomal protein L40 [Source:HGNC Symbol;Acc:HGNC:14491]
PWP2	PWP2 periodic tryptophan protein homolog (yeast) [Source:HGNC Symbol;Acc:HGNC:9711]
RHNO1	RAD9-HUS1-RAD1 interacting nuclear orphan 1 [Source:HGNC Symbol;Acc:HGNC:28206]
SETMAR	SET domain and mariner transposase fusion gene [Source:HGNC Symbol;Acc:HGNC:10762]
SLC30A6	solute carrier family 30 member 6 [Source:HGNC Symbol;Acc:HGNC:19305]
SYNE3	spectrin repeat containing nuclear envelope family member 3 [Source:HGNC Symbol;Acc:HGNC:19861]
TAF5	TATA-box binding protein associated factor 5 [Source:HGNC Symbol;Acc:HGNC:11539]
TMEM41B	transmembrane protein 41B [Source:HGNC Symbol;Acc:HGNC:28948]
WDR5	WD repeat domain 5 [Source:HGNC Symbol;Acc:HGNC:12757]
ZFP62	ZFP62 zinc finger protein [Source:HGNC Symbol;Acc:HGNC:23241]
ZNF367	zinc finger protein 367 [Source:HGNC Symbol;Acc:HGNC:18320]
AFM	afamin [Source:HGNC Symbol;Acc:HGNC:316]
ATP13A2	ATPase 13A2 [Source:HGNC Symbol;Acc:HGNC:30213]
CBFA2T3	CBFA2/RUNX1 translocation partner 3 [Source:HGNC Symbol;Acc:HGNC:1537]
EDA2R	ectodysplasin A2 receptor [Source:HGNC Symbol;Acc:HGNC:17756]

EYA2	EYA transcriptional coactivator and phosphatase 2 [Source:HGNC Symbol;Acc:HGNC:3520]
FCMR	Fc fragment of IgM receptor [Source:HGNC Symbol;Acc:HGNC:14315]
FOXP1	forkhead box P1 [Source:HGNC Symbol;Acc:HGNC:3823]
GATA6	GATA binding protein 6 [Source:HGNC Symbol;Acc:HGNC:4174]
GPAT3	glycerol-3-phosphate acyltransferase 3 [Source:HGNC Symbol;Acc:HGNC:28157]
GPR37	G protein-coupled receptor 37 [Source:HGNC Symbol;Acc:HGNC:4494]
IMPACT	impact RWD domain protein [Source:HGNC Symbol;Acc:HGNC:20387]
KYAT3	kynurenine aminotransferase 3 [Source:HGNC Symbol;Acc:HGNC:33238]
LSM6	LSM6 homolog, U6 small nuclear RNA and mRNA degradation associated [Source:HGNC Symbol;Acc:HGNC:17017]
PCF11	PCF11 cleavage and polyadenylation factor subunit [Source:HGNC Symbol;Acc:HGNC:30097]
PHACTR1	phosphatase and actin regulator 1 [Source:HGNC Symbol;Acc:HGNC:20990]
SGPP1	sphingosine-1-phosphate phosphatase 1 [Source:HGNC Symbol;Acc:HGNC:17720]
ST6GAL1	ST6 beta-galactoside alpha-2,6-sialyltransferase 1 [Source:HGNC Symbol;Acc:HGNC:10860]
WNT4	Wnt family member 4 [Source:HGNC Symbol;Acc:HGNC:12783]
WNT6	Wnt family member 6 [Source:HGNC Symbol;Acc:HGNC:12785]
CTDSPL2	CTD small phosphatase like 2 [Source:HGNC Symbol;Acc:HGNC:26936]
DDX52	DEAD-box helicase 52 [Source:HGNC Symbol;Acc:HGNC:20038]
EXOSC6	exosome component 6 [Source:HGNC Symbol;Acc:HGNC:19055]
EYA3	EYA transcriptional coactivator and phosphatase 3 [Source:HGNC Symbol;Acc:HGNC:3521]
ILVBL	ilvB (bacterial acetolactate synthase)-like [Source:HGNC Symbol;Acc:HGNC:6041]
IMMP1L	inner mitochondrial membrane peptidase subunit 1 [Source:HGNC Symbol;Acc:HGNC:26317]
IWS1	IWS1, SUPT6H interacting protein [Source:HGNC Symbol;Acc:HGNC:25467]
LST1	leukocyte specific transcript 1 [Source:HGNC Symbol;Acc:HGNC:14189]
MPPED2	metallophosphoesterase domain containing 2 [Source:HGNC Symbol;Acc:HGNC:1180]
MRPS12	mitochondrial ribosomal protein S12 [Source:HGNC Symbol;Acc:HGNC:10380]
REPS1	RALBP1 associated Eps domain containing 1 [Source:HGNC Symbol;Acc:HGNC:15578]
RTCA	RNA 3'-terminal phosphate cyclase [Source:HGNC Symbol;Acc:HGNC:17981]
RUNDC3A	RUN domain containing 3A [Source:HGNC Symbol;Acc:HGNC:16984]
TIMM22	translocase of inner mitochondrial membrane 22 homolog (yeast) [Source:HGNC Symbol;Acc:HGNC:17317]
TMEM70	transmembrane protein 70 [Source:HGNC Symbol;Acc:HGNC:26050]
TSFM	Ts translation elongation factor, mitochondrial [Source:HGNC Symbol;Acc:HGNC:12367]
TSTD1	thiosulfate sulfurtransferase like domain containing 1 [Source:HGNC Symbol;Acc:HGNC:35410]
UROS	uroporphyrinogen III synthase [Source:HGNC Symbol;Acc:HGNC:12592]
WNK2	WNK lysine deficient protein kinase 2 [Source:HGNC Symbol;Acc:HGNC:14542]

WRN	Werner syndrome RecQ like helicase [Source:HGNC Symbol;Acc:HGNC:12791]
ADAM15	ADAM metallopeptidase domain 15 [Source:HGNC Symbol;Acc:HGNC:193]
ARHGEF6	Rac/Cdc42 guanine nucleotide exchange factor 6 [Source:HGNC Symbol;Acc:HGNC:685]
C11orf96	chromosome 11 open reading frame 96 [Source:HGNC Symbol;Acc:HGNC:38675]
C8G	complement component 8, gamma polypeptide [Source:HGNC Symbol;Acc:HGNC:1354]
CADPS	calcium dependent secretion activator [Source:HGNC Symbol;Acc:HGNC:1426]
CDK2AP2	cyclin dependent kinase 2 associated protein 2 [Source:HGNC Symbol;Acc:HGNC:30833]
CPA4	carboxypeptidase A4 [Source:HGNC Symbol;Acc:HGNC:15740]
CPNE3	copine 3 [Source:HGNC Symbol;Acc:HGNC:2316]
DOT1L	DOT1 like histone lysine methyltransferase [Source:HGNC Symbol;Acc:HGNC:24948]
ERF	ETS2 repressor factor [Source:HGNC Symbol;Acc:HGNC:3444]
ERP44	endoplasmic reticulum protein 44 [Source:HGNC Symbol;Acc:HGNC:18311]
F7	coagulation factor VII [Source:HGNC Symbol;Acc:HGNC:3544]
FAF1	Fas associated factor 1 [Source:HGNC Symbol;Acc:HGNC:3578]
FAT3	FAT atypical cadherin 3 [Source:HGNC Symbol;Acc:HGNC:23112]
HMGN5	high mobility group nucleosome binding domain 5 [Source:HGNC Symbol;Acc:HGNC:8013]
HOGA1	4-hydroxy-2-oxoglutarate aldolase 1 [Source:HGNC Symbol;Acc:HGNC:25155]
HS6ST2	heparan sulfate 6-O-sulfotransferase 2 [Source:HGNC Symbol;Acc:HGNC:19133]
MOCS1	molybdenum cofactor synthesis 1 [Source:HGNC Symbol;Acc:HGNC:7190]
MYL12A	myosin light chain 12A [Source:HGNC Symbol;Acc:HGNC:16701]
PAWR	pro-apoptotic WT1 regulator [Source:HGNC Symbol;Acc:HGNC:8614]
PCDH8	protocadherin 8 [Source:HGNC Symbol;Acc:HGNC:8660]
PGLS	6-phosphogluconolactonase [Source:HGNC Symbol;Acc:HGNC:8903]
RAB40B	RAB40B, member RAS oncogene family [Source:HGNC Symbol;Acc:HGNC:18284]
RGS20	regulator of G-protein signaling 20 [Source:HGNC Symbol;Acc:HGNC:14600]
RPL7A	ribosomal protein L7a [Source:HGNC Symbol;Acc:HGNC:10364]
SCN3A	sodium voltage-gated channel alpha subunit 3 [Source:HGNC Symbol;Acc:HGNC:10590]
SH3GL3	SH3 domain containing GRB2 like endophilin A3 [Source:HGNC Symbol;Acc:HGNC:10832]
SLC16A11	solute carrier family 16 member 11 [Source:HGNC Symbol;Acc:HGNC:23093]
SLC17A3	solute carrier family 17 member 3 [Source:HGNC Symbol;Acc:HGNC:10931]
SLC25A24	solute carrier family 25 member 24 [Source:HGNC Symbol;Acc:HGNC:20662]
STX2	syntaxin 2 [Source:HGNC Symbol;Acc:HGNC:3403]
TTPAL	alpha tocopherol transfer protein like [Source:HGNC Symbol;Acc:HGNC:16114]
ARPC5	actin related protein 2/3 complex subunit 5 [Source:HGNC Symbol;Acc:HGNC:708]
ASH2L	ASH2 like histone lysine methyltransferase complex subunit [Source:HGNC Symbol;Acc:HGNC:744]

ATP5A1	ATP synthase, H+ transporting, mitochondrial F1 complex, alpha subunit 1, cardiac muscle [Source:HGNC Symbol;Acc:HGNC:823]
ATP6V1F	ATPase H+ transporting V1 subunit F [Source:HGNC Symbol;Acc:HGNC:16832]
CORO1B	coronin 1B [Source:HGNC Symbol;Acc:HGNC:2253]
CSTF1	cleavage stimulation factor subunit 1 [Source:HGNC Symbol;Acc:HGNC:2483]
DDX42	DEAD-box helicase 42 [Source:HGNC Symbol;Acc:HGNC:18676]
DYNC2H1	dynein cytoplasmic 2 heavy chain 1 [Source:HGNC Symbol;Acc:HGNC:2962]
EMC9	ER membrane protein complex subunit 9 [Source:HGNC Symbol;Acc:HGNC:20273]
HNRNPLL	heterogeneous nuclear ribonucleoprotein L like [Source:HGNC Symbol;Acc:HGNC:25127]
MICA	MHC class I polypeptide-related sequence A [Source:HGNC Symbol;Acc:HGNC:7090]
MRPS31	mitochondrial ribosomal protein S31 [Source:HGNC Symbol;Acc:HGNC:16632]
NCOA5	nuclear receptor coactivator 5 [Source:HGNC Symbol;Acc:HGNC:15909]
NTPCR	nucleoside-triphosphatase, cancer-related [Source:HGNC Symbol;Acc:HGNC:28204]
NUP107	nucleoporin 107 [Source:HGNC Symbol;Acc:HGNC:29914]
PINX1	PIN2/TERF1 interacting, telomerase inhibitor 1 [Source:HGNC Symbol;Acc:HGNC:30046]
POLR2I	polymerase (RNA) II subunit I [Source:HGNC Symbol;Acc:HGNC:9196]
RAB21	RAB21, member RAS oncogene family [Source:HGNC Symbol;Acc:HGNC:18263]
RSBN1	round spermatid basic protein 1 [Source:HGNC Symbol;Acc:HGNC:25642]
SBF2	SET binding factor 2 [Source:HGNC Symbol;Acc:HGNC:2135]
TMEM109	transmembrane protein 109 [Source:HGNC Symbol;Acc:HGNC:28771]
YIF1A	Yip1 interacting factor homolog A, membrane trafficking protein [Source:HGNC Symbol;Acc:HGNC:16688]
ZBTB2	zinc finger and BTB domain containing 2 [Source:HGNC Symbol;Acc:HGNC:20868]
CISH	cytokine inducible SH2 containing protein [Source:HGNC Symbol;Acc:HGNC:1984]
CPA1	carboxypeptidase A1 [Source:HGNC Symbol;Acc:HGNC:2296]
DUSP9	dual specificity phosphatase 9 [Source:HGNC Symbol;Acc:HGNC:3076]
EXOC3	exocyst complex component 3 [Source:HGNC Symbol;Acc:HGNC:30378]
GABPB1	GA binding protein transcription factor beta subunit 1 [Source:HGNC Symbol;Acc:HGNC:4074]
GRHPR	glyoxylate reductase/hydroxypyruvate reductase [Source:HGNC Symbol;Acc:HGNC:4570]
NDUFS7	NADH:ubiquinone oxidoreductase core subunit S7 [Source:HGNC Symbol;Acc:HGNC:7714]
PCYT1A	phosphate cytidylyltransferase 1, choline, alpha [Source:HGNC Symbol;Acc:HGNC:8754]
PRR13	proline rich 13 [Source:HGNC Symbol;Acc:HGNC:24528]
RPL34	ribosomal protein L34 [Source:HGNC Symbol;Acc:HGNC:10340]
SH3GL2	SH3 domain containing GRB2 like 2, endophilin A1 [Source:HGNC Symbol;Acc:HGNC:10831]
SNTG2	syntrophin gamma 2 [Source:HGNC Symbol;Acc:HGNC:13741]
SRRM1	serine and arginine repetitive matrix 1 [Source:HGNC Symbol;Acc:HGNC:16638]

WWP1	WW domain containing E3 ubiquitin protein ligase 1 [Source:HGNC Symbol;Acc:HGNC:17004]
XPC	XPC complex subunit, DNA damage recognition and repair factor [Source:HGNC Symbol;Acc:HGNC:12816]
ARHGAP9	Rho GTPase activating protein 9 [Source:HGNC Symbol;Acc:HGNC:14130]
CCL21	C-C motif chemokine ligand 21 [Source:HGNC Symbol;Acc:HGNC:10620]
APITD1	apoptosis-inducing, TAF9-like domain 1 [Source:HGNC Symbol;Acc:HGNC:23163]
FICD	FIC domain containing [Source:HGNC Symbol;Acc:HGNC:18416]
FYTTD1	forty-two-three domain containing 1 [Source:HGNC Symbol;Acc:HGNC:25407]
HINT2	histidine triad nucleotide binding protein 2 [Source:HGNC Symbol;Acc:HGNC:18344]
LYPD6	LY6/PLAUR domain containing 6 [Source:HGNC Symbol;Acc:HGNC:28751]
MBOAT7	membrane bound O-acyltransferase domain containing 7 [Source:HGNC Symbol;Acc:HGNC:15505]
MRPS33	mitochondrial ribosomal protein S33 [Source:HGNC Symbol;Acc:HGNC:16634]
NUFIP2	NUFIP2, FMR1 interacting protein 2 [Source:HGNC Symbol;Acc:HGNC:17634]
POLR2G	polymerase (RNA) II subunit G [Source:HGNC Symbol;Acc:HGNC:9194]
RBM22	RNA binding motif protein 22 [Source:HGNC Symbol;Acc:HGNC:25503]
SF3B5	splicing factor 3b subunit 5 [Source:HGNC Symbol;Acc:HGNC:21083]
TMC4	transmembrane channel like 4 [Source:HGNC Symbol;Acc:HGNC:22998]
TWF2	twinfilin actin binding protein 2 [Source:HGNC Symbol;Acc:HGNC:9621]
ZMPSTE24	zinc metallopeptidase STE24 [Source:HGNC Symbol;Acc:HGNC:12877]
ADAMTSL4	ADAMTS like 4 [Source:HGNC Symbol;Acc:HGNC:19706]
ATP6V0B	ATPase H+ transporting V0 subunit b [Source:HGNC Symbol;Acc:HGNC:861]
CLEC11A	C-type lectin domain family 11 member A [Source:HGNC Symbol;Acc:HGNC:10576]
CTBP1	C-terminal binding protein 1 [Source:HGNC Symbol;Acc:HGNC:2494]
LGALS4	galectin 4 [Source:HGNC Symbol;Acc:HGNC:6565]
LRIG3	leucine rich repeats and immunoglobulin like domains 3 [Source:HGNC Symbol;Acc:HGNC:30991]
MTMR11	myotubularin related protein 11 [Source:HGNC Symbol;Acc:HGNC:24307]
PNKD	paroxysmal nonkinesigenic dyskinesia [Source:HGNC Symbol;Acc:HGNC:9153]
RSPO3	R-spondin 3 [Source:HGNC Symbol;Acc:HGNC:20866]
SLC17A1	solute carrier family 17 member 1 [Source:HGNC Symbol;Acc:HGNC:10929]
SLC26A1	solute carrier family 26 member 1 [Source:HGNC Symbol;Acc:HGNC:10993]
UBE2D2	ubiquitin conjugating enzyme E2 D2 [Source:HGNC Symbol;Acc:HGNC:12475]
ACP6	acid phosphatase 6, lysophosphatidic [Source:HGNC Symbol;Acc:HGNC:29609]
BCAP31	B-cell receptor-associated protein 31 [Source:HGNC Symbol;Acc:HGNC:16695]
CDC123	cell division cycle 123 [Source:HGNC Symbol;Acc:HGNC:16827]
CUL5	cullin 5 [Source:HGNC Symbol;Acc:HGNC:2556]
EED	embryonic ectoderm development [Source:HGNC Symbol;Acc:HGNC:3188]
ERLIN2	ER lipid raft associated 2 [Source:HGNC Symbol;Acc:HGNC:1356]
G6PC3	glucose-6-phosphatase catalytic subunit 3 [Source:HGNC Symbol;Acc:HGNC:24861]
GNPNAT1	glucosamine-phosphate N-acetyltransferase 1 [Source:HGNC Symbol;Acc:HGNC:19980]

NMRAL1	NmrA-like family domain containing 1 [Source:HGNC Symbol;Acc:HGNC:24987]
POMP	proteasome maturation protein [Source:HGNC Symbol;Acc:HGNC:20330]
PRKAG1	protein kinase AMP-activated non-catalytic subunit gamma 1 [Source:HGNC Symbol;Acc:HGNC:9385]
SIAH1	siah E3 ubiquitin protein ligase 1 [Source:HGNC Symbol;Acc:HGNC:10857]
SNX3	sorting nexin 3 [Source:HGNC Symbol;Acc:HGNC:11174]
SRP68	signal recognition particle 68 [Source:HGNC Symbol;Acc:HGNC:11302]
STT3B	STT3B, catalytic subunit of the oligosaccharyltransferase complex [Source:HGNC Symbol;Acc:HGNC:30611]
SULT4A1	sulfotransferase family 4A member 1 [Source:HGNC Symbol;Acc:HGNC:14903]
TMEM263	transmembrane protein 263 [Source:HGNC Symbol;Acc:HGNC:28281]
UBE2V1	ubiquitin conjugating enzyme E2 V1 [Source:HGNC Symbol;Acc:HGNC:12494]
AEBP1	AE binding protein 1 [Source:HGNC Symbol;Acc:HGNC:303]
CAB39L	calcium binding protein 39 like [Source:HGNC Symbol;Acc:HGNC:20290]
CDC42EP2	CDC42 effector protein 2 [Source:HGNC Symbol;Acc:HGNC:16263]
CIDEB	cell death-inducing DFFA-like effector b [Source:HGNC Symbol;Acc:HGNC:1977]
CRLF1	cytokine receptor like factor 1 [Source:HGNC Symbol;Acc:HGNC:2364]
CYP4B1	cytochrome P450 family 4 subfamily B member 1 [Source:HGNC Symbol;Acc:HGNC:2644]
DEXI	Dexi homolog [Source:HGNC Symbol;Acc:HGNC:13267]
F2RL1	F2R like trypsin receptor 1 [Source:HGNC Symbol;Acc:HGNC:3538]
FBXL20	F-box and leucine rich repeat protein 20 [Source:HGNC Symbol;Acc:HGNC:24679]
FSTL3	follistatin like 3 [Source:HGNC Symbol;Acc:HGNC:3973]
GNS	glucosamine (N-acetyl)-6-sulfatase [Source:HGNC Symbol;Acc:HGNC:4422]
GREM2	gremlin 2, DAN family BMP antagonist [Source:HGNC Symbol;Acc:HGNC:17655]
KANK2	KN motif and ankyrin repeat domains 2 [Source:HGNC Symbol;Acc:HGNC:29300]
LHPP	phospholysine phosphohistidine inorganic pyrophosphate phosphatase [Source:HGNC Symbol;Acc:HGNC:30042]
MT1M	metallothionein 1M [Source:HGNC Symbol;Acc:HGNC:14296]
MYO1C	myosin IC [Source:HGNC Symbol;Acc:HGNC:7597]
NDUFS8	NADH:ubiquinone oxidoreductase core subunit S8 [Source:HGNC Symbol;Acc:HGNC:7715]
PDLIM4	PDZ and LIM domain 4 [Source:HGNC Symbol;Acc:HGNC:16501]
PER3	period circadian clock 3 [Source:HGNC Symbol;Acc:HGNC:8847]
PRR11	proline rich 11 [Source:HGNC Symbol;Acc:HGNC:25619]
PTP4A3	protein tyrosine phosphatase type IVA, member 3 [Source:HGNC Symbol;Acc:HGNC:9636]
RAB4A	RAB4A, member RAS oncogene family [Source:HGNC Symbol;Acc:HGNC:9781]
RALGPS2	Ral GEF with PH domain and SH3 binding motif 2 [Source:HGNC Symbol;Acc:HGNC:30279]
RNMT	RNA guanine-7 methyltransferase [Source:HGNC Symbol;Acc:HGNC:10075]
SBNO1	strawberry notch homolog 1 (Drosophila) [Source:HGNC Symbol;Acc:HGNC:22973]
SCRN1	secernin 1 [Source:HGNC Symbol;Acc:HGNC:22192]

SIAE	sialic acid acetylesterase [Source:HGNC Symbol;Acc:HGNC:18187]
SLC16A4	solute carrier family 16 member 4 [Source:HGNC Symbol;Acc:HGNC:10925]
SYT11	synaptotagmin 11 [Source:HGNC Symbol;Acc:HGNC:19239]
TBX3	T-box 3 [Source:HGNC Symbol;Acc:HGNC:11602]
TCEAL9	transcription elongation factor A like 9 [Source:HGNC Symbol;Acc:HGNC:30084]
TNFSF13	tumor necrosis factor superfamily member 13 [Source:HGNC Symbol;Acc:HGNC:11928]
ZBTB4	zinc finger and BTB domain containing 4 [Source:HGNC Symbol;Acc:HGNC:23847]
ABHD15	abhydrolase domain containing 15 [Source:HGNC Symbol;Acc:HGNC:26971]
AMFR	autocrine motility factor receptor [Source:HGNC Symbol;Acc:HGNC:463]
AMOTL1	angiomotin like 1 [Source:HGNC Symbol;Acc:HGNC:17811]
ARF5	ADP ribosylation factor 5 [Source:HGNC Symbol;Acc:HGNC:658]
CLTA	clathrin light chain A [Source:HGNC Symbol;Acc:HGNC:2090]
ENPP4	ectonucleotide pyrophosphatase/phosphodiesterase 4 (putative) [Source:HGNC Symbol;Acc:HGNC:3359]
FCHO2	FCH domain only 2 [Source:HGNC Symbol;Acc:HGNC:25180]
FES	FES proto-oncogene, tyrosine kinase [Source:HGNC Symbol;Acc:HGNC:3657]
HAVCR2	hepatitis A virus cellular receptor 2 [Source:HGNC Symbol;Acc:HGNC:18437]
ILDR2	immunoglobulin like domain containing receptor 2 [Source:HGNC Symbol;Acc:HGNC:18131]
KRT20	keratin 20 [Source:HGNC Symbol;Acc:HGNC:20412]
MMD2	monocyte to macrophage differentiation associated 2 [Source:HGNC Symbol;Acc:HGNC:30133]
MRPL24	mitochondrial ribosomal protein L24 [Source:HGNC Symbol;Acc:HGNC:14037]
MRPS18A	mitochondrial ribosomal protein S18A [Source:HGNC Symbol;Acc:HGNC:14515]
MYLPF	myosin light chain, phosphorylatable, fast skeletal muscle [Source:HGNC Symbol;Acc:HGNC:29824]
NUDT12	nudix hydrolase 12 [Source:HGNC Symbol;Acc:HGNC:18826]
PYCR2	pyrroline-5-carboxylate reductase family member 2 [Source:HGNC Symbol;Acc:HGNC:30262]
RNFT2	ring finger protein, transmembrane 2 [Source:HGNC Symbol;Acc:HGNC:25905]
SEC22B	SEC22 homolog B, vesicle trafficking protein (gene/pseudogene) [Source:HGNC Symbol;Acc:HGNC:10700]
SLF1	SMC5-SMC6 complex localization factor 1 [Source:HGNC Symbol;Acc:HGNC:25408]
STOML2	stomatin like 2 [Source:HGNC Symbol;Acc:HGNC:14559]
SULT1A2	sulfotransferase family 1A member 2 [Source:HGNC Symbol;Acc:HGNC:11454]
TEX30	testis expressed 30 [Source:HGNC Symbol;Acc:HGNC:25188]
TFAP4	transcription factor AP-4 (activating enhancer binding protein 4) [Source:HGNC Symbol;Acc:HGNC:11745]
TMED4	transmembrane p24 trafficking protein 4 [Source:HGNC Symbol;Acc:HGNC:22301]
TOMM7	translocase of outer mitochondrial membrane 7 [Source:HGNC Symbol;Acc:HGNC:21648]
ZBTB7A	zinc finger and BTB domain containing 7A [Source:HGNC Symbol;Acc:HGNC:18078]
COL5A2	collagen type V alpha 2 chain [Source:HGNC Symbol;Acc:HGNC:2210]

GDPD1	glycerophosphodiester phosphodiesterase domain containing 1 [Source:HGNC Symbol;Acc:HGNC:20883]
KPNA4	karyopherin subunit alpha 4 [Source:HGNC Symbol;Acc:HGNC:6397]
MSH3	mutS homolog 3 [Source:HGNC Symbol;Acc:HGNC:7326]
PDE1C	phosphodiesterase 1C [Source:HGNC Symbol;Acc:HGNC:8776]
PPCS	phosphopantothenoylcysteine synthetase [Source:HGNC Symbol;Acc:HGNC:25686]
PSMD6	proteasome 26S subunit, non-ATPase 6 [Source:HGNC Symbol;Acc:HGNC:9564]
SLC30A2	solute carrier family 30 member 2 [Source:HGNC Symbol;Acc:HGNC:11013]
SREK1	splicing regulatory glutamic acid and lysine rich protein 1 [Source:HGNC Symbol;Acc:HGNC:17882]
TAF9B	TATA-box binding protein associated factor 9b [Source:HGNC Symbol;Acc:HGNC:17306]
TSPAN33	tetraspanin 33 [Source:HGNC Symbol;Acc:HGNC:28743]
BNIP1	BCL2/adenovirus E1B 19kDa interacting protein 1 [Source:HGNC Symbol;Acc:HGNC:1082]
BRI3BP	BRI3 binding protein [Source:HGNC Symbol;Acc:HGNC:14251]
CCDC113	coiled-coil domain containing 113 [Source:HGNC Symbol;Acc:HGNC:25002]
CHCHD2	coiled-coil-helix-coiled-coil-helix domain containing 2 [Source:HGNC Symbol;Acc:HGNC:21645]
CNST	consortin, connexin sorting protein [Source:HGNC Symbol;Acc:HGNC:26486]
DHRS1	dehydrogenase/reductase 1 [Source:HGNC Symbol;Acc:HGNC:16445]
DOK4	docking protein 4 [Source:HGNC Symbol;Acc:HGNC:19868]
GSG2	germ cell associated 2, haspin [Source:HGNC Symbol;Acc:HGNC:19682]
HYKK	hydroxylysine kinase [Source:HGNC Symbol;Acc:HGNC:34403]
IPCEF1	interaction protein for cytohesin exchange factors 1 [Source:HGNC Symbol;Acc:HGNC:21204]
MRPL46	mitochondrial ribosomal protein L46 [Source:HGNC Symbol;Acc:HGNC:1192]
NCBP1	nuclear cap binding protein subunit 1 [Source:HGNC Symbol;Acc:HGNC:7658]
P2RY12	purinergic receptor P2Y12 [Source:HGNC Symbol;Acc:HGNC:18124]
PAQR5	progestin and adipoQ receptor family member 5 [Source:HGNC Symbol;Acc:HGNC:29645]
PTPMT1	protein tyrosine phosphatase, mitochondrial 1 [Source:HGNC Symbol;Acc:HGNC:26965]
RCOR1	REST corepressor 1 [Source:HGNC Symbol;Acc:HGNC:17441]
REEP4	receptor accessory protein 4 [Source:HGNC Symbol;Acc:HGNC:26176]
RTN3	reticulon 3 [Source:HGNC Symbol;Acc:HGNC:10469]
SCX	scleraxis bHLH transcription factor [Source:HGNC Symbol;Acc:HGNC:32322]
SGCG	sarcoglycan gamma [Source:HGNC Symbol;Acc:HGNC:10809]
SNX4	sorting nexin 4 [Source:HGNC Symbol;Acc:HGNC:11175]
SPDEF	SAM pointed domain containing ETS transcription factor [Source:HGNC Symbol;Acc:HGNC:17257]
STX8	syntaxin 8 [Source:HGNC Symbol;Acc:HGNC:11443]
SYNGR3	synaptogyrin 3 [Source:HGNC Symbol;Acc:HGNC:11501]

TMEM237	transmembrane protein 237 [Source:HGNC Symbol;Acc:HGNC:14432]
TSG101	tumor susceptibility 101 [Source:HGNC Symbol;Acc:HGNC:15971]
VDAC3	voltage dependent anion channel 3 [Source:HGNC Symbol;Acc:HGNC:12674]
WNT10A	Wnt family member 10A [Source:HGNC Symbol;Acc:HGNC:13829]
GABRD	gamma-aminobutyric acid type A receptor delta subunit [Source:HGNC Symbol;Acc:HGNC:4084]
GDI1	GDP dissociation inhibitor 1 [Source:HGNC Symbol;Acc:HGNC:4226]
GLCCI1	glucocorticoid induced 1 [Source:HGNC Symbol;Acc:HGNC:18713]
IL18R1	interleukin 18 receptor 1 [Source:HGNC Symbol;Acc:HGNC:5988]
PLA2G5	phospholipase A2 group V [Source:HGNC Symbol;Acc:HGNC:9038]
PPP3CB	protein phosphatase 3 catalytic subunit beta [Source:HGNC Symbol;Acc:HGNC:9315]
QDPR	quinoid dihydropteridine reductase [Source:HGNC Symbol;Acc:HGNC:9752]
RYBP	RING1 and YY1 binding protein [Source:HGNC Symbol;Acc:HGNC:10480]
SDHC	succinate dehydrogenase complex subunit C [Source:HGNC Symbol;Acc:HGNC:10682]
SNRPF	small nuclear ribonucleoprotein polypeptide F [Source:HGNC Symbol;Acc:HGNC:11162]
SSB	Sjogren syndrome antigen B [Source:HGNC Symbol;Acc:HGNC:11316]
SYT7	synaptotagmin 7 [Source:HGNC Symbol;Acc:HGNC:11514]
USP25	ubiquitin specific peptidase 25 [Source:HGNC Symbol;Acc:HGNC:12624]
APBB1	amyloid beta precursor protein binding family B member 1 [Source:HGNC Symbol;Acc:HGNC:581]
ARFGEF3	ARFGEF family member 3 [Source:HGNC Symbol;Acc:HGNC:21213]
ARPC5L	actin related protein 2/3 complex subunit 5 like [Source:HGNC Symbol;Acc:HGNC:23366]
BCS1L	BCS1 homolog, ubiquinol-cytochrome c reductase complex chaperone [Source:HGNC Symbol;Acc:HGNC:1020]
CCDC117	coiled-coil domain containing 117 [Source:HGNC Symbol;Acc:HGNC:26599]
CCNK	cyclin K [Source:HGNC Symbol;Acc:HGNC:1596]
CDC5L	cell division cycle 5 like [Source:HGNC Symbol;Acc:HGNC:1743]
CENPL	centromere protein L [Source:HGNC Symbol;Acc:HGNC:17879]
CREB3L4	cAMP responsive element binding protein 3 like 4 [Source:HGNC Symbol;Acc:HGNC:18854]
CRTC2	CREB regulated transcription coactivator 2 [Source:HGNC Symbol;Acc:HGNC:27301]
DDX23	DEAD-box helicase 23 [Source:HGNC Symbol;Acc:HGNC:17347]
ERC2	ELKS/RAB6-interacting/CAST family member 2 [Source:HGNC Symbol;Acc:HGNC:31922]
HAUS1	HAUS augmin like complex subunit 1 [Source:HGNC Symbol;Acc:HGNC:25174]
IL18BP	interleukin 18 binding protein [Source:HGNC Symbol;Acc:HGNC:5987]
INPP4A	inositol polyphosphate-4-phosphatase type I A [Source:HGNC Symbol;Acc:HGNC:6074]
KCNIP3	potassium voltage-gated channel interacting protein 3 [Source:HGNC Symbol;Acc:HGNC:15523]
MC1R	melanocortin 1 receptor [Source:HGNC Symbol;Acc:HGNC:6929]
MRPS18C	mitochondrial ribosomal protein S18C [Source:HGNC Symbol;Acc:HGNC:16633]

MTNR1A	melatonin receptor 1A [Source:HGNC Symbol;Acc:HGNC:7463]
NAIP	NLR family apoptosis inhibitory protein [Source:HGNC Symbol;Acc:HGNC:7634]
NDUFC1	NADH:ubiquinone oxidoreductase subunit C1 [Source:HGNC Symbol;Acc:HGNC:7705]
NDUFS6	NADH:ubiquinone oxidoreductase subunit S6 [Source:HGNC Symbol;Acc:HGNC:7713]
PRMT6	protein arginine methyltransferase 6 [Source:HGNC Symbol;Acc:HGNC:18241]
RASA2	RAS p21 protein activator 2 [Source:HGNC Symbol;Acc:HGNC:9872]
SEC61G	Sec61 translocon gamma subunit [Source:HGNC Symbol;Acc:HGNC:18277]
SNX24	sorting nexin 24 [Source:HGNC Symbol;Acc:HGNC:21533]
TIMM44	translocase of inner mitochondrial membrane 44 [Source:HGNC Symbol;Acc:HGNC:17316]
TXNL4B	thioredoxin like 4B [Source:HGNC Symbol;Acc:HGNC:26041]
VPS29	VPS29, retromer complex component [Source:HGNC Symbol;Acc:HGNC:14340]
ZDHHC23	zinc finger DHHC-type containing 23 [Source:HGNC Symbol;Acc:HGNC:28654]
ZKSCAN1	zinc finger with KRAB and SCAN domains 1 [Source:HGNC Symbol;Acc:HGNC:13101]
CAP1	adenylate cyclase associated protein 1 [Source:HGNC Symbol;Acc:HGNC:20040]
CD3G	CD3g molecule [Source:HGNC Symbol;Acc:HGNC:1675]
CLSPN	claspin [Source:HGNC Symbol;Acc:HGNC:19715]
DRD5	dopamine receptor D5 [Source:HGNC Symbol;Acc:HGNC:3026]
EIF3I	eukaryotic translation initiation factor 3 subunit I [Source:HGNC Symbol;Acc:HGNC:3272]
F11	coagulation factor XI [Source:HGNC Symbol;Acc:HGNC:3529]
FGGY	FGGY carbohydrate kinase domain containing [Source:HGNC Symbol;Acc:HGNC:25610]
GLRA1	glycine receptor alpha 1 [Source:HGNC Symbol;Acc:HGNC:4326]
NAA38	N(alpha)-acetyltransferase 38, NatC auxiliary subunit [Source:HGNC Symbol;Acc:HGNC:28212]
NRN1	neuritin 1 [Source:HGNC Symbol;Acc:HGNC:17972]
OLFML2A	olfactomedin like 2A [Source:HGNC Symbol;Acc:HGNC:27270]
PGRMC2	progesterone receptor membrane component 2 [Source:HGNC Symbol;Acc:HGNC:16089]
PIK3AP1	phosphoinositide-3-kinase adaptor protein 1 [Source:HGNC Symbol;Acc:HGNC:30034]
PITX2	paired like homeodomain 2 [Source:HGNC Symbol;Acc:HGNC:9005]
PRG4	proteoglycan 4 [Source:HGNC Symbol;Acc:HGNC:9364]
PTPRU	protein tyrosine phosphatase, receptor type U [Source:HGNC Symbol;Acc:HGNC:9683]
RFFL	ring finger and FYVE-like domain containing E3 ubiquitin protein ligase [Source:HGNC Symbol;Acc:HGNC:24821]
RPL28	ribosomal protein L28 [Source:HGNC Symbol;Acc:HGNC:10330]
RPN1	ribophorin I [Source:HGNC Symbol;Acc:HGNC:10381]
RPS17	ribosomal protein S17 [Source:HGNC Symbol;Acc:HGNC:10397]
RUNX1	runt related transcription factor 1 [Source:HGNC Symbol;Acc:HGNC:10471]
SFXN3	sideroflexin 3 [Source:HGNC Symbol;Acc:HGNC:16087]
SLC29A2	solute carrier family 29 member 2 [Source:HGNC Symbol;Acc:HGNC:11004]
TFCP2L1	transcription factor CP2-like 1 [Source:HGNC Symbol;Acc:HGNC:17925]

TNFSF15	tumor necrosis factor superfamily member 15 [Source:HGNC Symbol;Acc:HGNC:11931]
UBE2J1	ubiquitin conjugating enzyme E2 J1 [Source:HGNC Symbol;Acc:HGNC:17598]
YRDC	yrdC N6-threonylcarbamoyltransferase domain containing [Source:HGNC Symbol;Acc:HGNC:28905]
AP2A2	adaptor related protein complex 2 alpha 2 subunit [Source:HGNC Symbol;Acc:HGNC:562]
ARFGEF1	ADP ribosylation factor guanine nucleotide exchange factor 1 [Source:HGNC Symbol;Acc:HGNC:15772]
ATE1	arginyltransferase 1 [Source:HGNC Symbol;Acc:HGNC:782]
BET1	Bet1 golgi vesicular membrane trafficking protein [Source:HGNC Symbol;Acc:HGNC:14562]
BTK	Bruton tyrosine kinase [Source:HGNC Symbol;Acc:HGNC:1133]
BYSL	bystin like [Source:HGNC Symbol;Acc:HGNC:1157]
DCTN1	dynactin subunit 1 [Source:HGNC Symbol;Acc:HGNC:2711]
DERA	deoxyribose-phosphate aldolase [Source:HGNC Symbol;Acc:HGNC:24269]
EHBP1L1	EH domain binding protein 1 like 1 [Source:HGNC Symbol;Acc:HGNC:30682]
FBXO11	F-box protein 11 [Source:HGNC Symbol;Acc:HGNC:13590]
GAPVD1	GTPase activating protein and VPS9 domains 1 [Source:HGNC Symbol;Acc:HGNC:23375]
IFT27	intraflagellar transport 27 [Source:HGNC Symbol;Acc:HGNC:18626]
LSAMP	limbic system-associated membrane protein [Source:HGNC Symbol;Acc:HGNC:6705]
MATN3	matrilin 3 [Source:HGNC Symbol;Acc:HGNC:6909]
MC2R	melanocortin 2 receptor [Source:HGNC Symbol;Acc:HGNC:6930]
MPHOSPH9	M-phase phosphoprotein 9 [Source:HGNC Symbol;Acc:HGNC:7215]
MRPS28	mitochondrial ribosomal protein S28 [Source:HGNC Symbol;Acc:HGNC:14513]
MRPS7	mitochondrial ribosomal protein S7 [Source:HGNC Symbol;Acc:HGNC:14499]
NMRK1	nicotinamide riboside kinase 1 [Source:HGNC Symbol;Acc:HGNC:26057]
NOC2L	NOC2 like nucleolar associated transcriptional repressor [Source:HGNC Symbol;Acc:HGNC:24517]
PKN2	protein kinase N2 [Source:HGNC Symbol;Acc:HGNC:9406]
PLXDC1	plexin domain containing 1 [Source:HGNC Symbol;Acc:HGNC:20945]
PMPCB	peptidase, mitochondrial processing beta subunit [Source:HGNC Symbol;Acc:HGNC:9119]
RAB5B	RAB5B, member RAS oncogene family [Source:HGNC Symbol;Acc:HGNC:9784]
RNF26	ring finger protein 26 [Source:HGNC Symbol;Acc:HGNC:14646]
SIL1	SIL1 nucleotide exchange factor [Source:HGNC Symbol;Acc:HGNC:24624]
SNRPD3	small nuclear ribonucleoprotein D3 polypeptide [Source:HGNC Symbol;Acc:HGNC:11160]
TP53BP2	tumor protein p53 binding protein 2 [Source:HGNC Symbol;Acc:HGNC:12000]
UBE2G2	ubiquitin conjugating enzyme E2 G2 [Source:HGNC Symbol;Acc:HGNC:12483]
UQCR10	ubiquinol-cytochrome c reductase, complex III subunit X [Source:HGNC Symbol;Acc:HGNC:30863]
CALCRL	calcitonin receptor like receptor [Source:HGNC Symbol;Acc:HGNC:16709]

CD302	CD302 molecule [Source:HGNC Symbol;Acc:HGNC:30843]
FOLR2	folate receptor beta [Source:HGNC Symbol;Acc:HGNC:3793]
KRT80	keratin 80 [Source:HGNC Symbol;Acc:HGNC:27056]
LRPAP1	LDL receptor related protein associated protein 1 [Source:HGNC Symbol;Acc:HGNC:6701]
MLC1	megalencephalic leukoencephalopathy with subcortical cysts 1 [Source:HGNC Symbol;Acc:HGNC:17082]
MRC2	mannose receptor C type 2 [Source:HGNC Symbol;Acc:HGNC:16875]
MST1	macrophage stimulating 1 [Source:HGNC Symbol;Acc:HGNC:7380]
PLD3	phospholipase D family member 3 [Source:HGNC Symbol;Acc:HGNC:17158]
RPL14	ribosomal protein L14 [Source:HGNC Symbol;Acc:HGNC:10305]
SLC18A2	solute carrier family 18 member A2 [Source:HGNC Symbol;Acc:HGNC:10935]
SLC25A15	solute carrier family 25 member 15 [Source:HGNC Symbol;Acc:HGNC:10985]
TBC1D15	TBC1 domain family member 15 [Source:HGNC Symbol;Acc:HGNC:25694]
TRPA1	transient receptor potential cation channel subfamily A member 1 [Source:HGNC Symbol;Acc:HGNC:497]
AFTPH	aftiphilin [Source:HGNC Symbol;Acc:HGNC:25951]
AKAP8	A-kinase anchoring protein 8 [Source:HGNC Symbol;Acc:HGNC:378]
ALG14	ALG14, UDP-N-acetylglucosaminyltransferase subunit [Source:HGNC Symbol;Acc:HGNC:28287]
AP3M1	adaptor related protein complex 3 mu 1 subunit [Source:HGNC Symbol;Acc:HGNC:569]
BRCC3	BRCA1/BRCA2-containing complex subunit 3 [Source:HGNC Symbol;Acc:HGNC:24185]
FANCL	Fanconi anemia complementation group L [Source:HGNC Symbol;Acc:HGNC:20748]
MARK2	microtubule affinity regulating kinase 2 [Source:HGNC Symbol;Acc:HGNC:3332]
MBD3	methyl-CpG binding domain protein 3 [Source:HGNC Symbol;Acc:HGNC:6918]
MCEE	methylmalonyl-CoA epimerase [Source:HGNC Symbol;Acc:HGNC:16732]
MORC3	MORC family CW-type zinc finger 3 [Source:HGNC Symbol;Acc:HGNC:23572]
MRPL49	mitochondrial ribosomal protein L49 [Source:HGNC Symbol;Acc:HGNC:1176]
MTFP1	mitochondrial fission process 1 [Source:HGNC Symbol;Acc:HGNC:26945]
MTMR7	myotubularin related protein 7 [Source:HGNC Symbol;Acc:HGNC:7454]
MYO18A	myosin XVIIIA [Source:HGNC Symbol;Acc:HGNC:31104]
ORMDL3	ORMDL sphingolipid biosynthesis regulator 3 [Source:HGNC Symbol;Acc:HGNC:16038]
PBRM1	polybromo 1 [Source:HGNC Symbol;Acc:HGNC:30064]
PHF5A	PHD finger protein 5A [Source:HGNC Symbol;Acc:HGNC:18000]
PTRH2	peptidyl-tRNA hydrolase 2 [Source:HGNC Symbol;Acc:HGNC:24265]
PUF60	poly(U) binding splicing factor 60KDa [Source:HGNC Symbol;Acc:HGNC:17042]
RANBP2	RAN binding protein 2 [Source:HGNC Symbol;Acc:HGNC:9848]
RAP2A	RAP2A, member of RAS oncogene family [Source:HGNC Symbol;Acc:HGNC:9861]
SCAP	SREBF chaperone [Source:HGNC Symbol;Acc:HGNC:30634]
SPIN4	spindlin family member 4 [Source:HGNC Symbol;Acc:HGNC:27040]
TGIF2	TGFB induced factor homeobox 2 [Source:HGNC Symbol;Acc:HGNC:15764]
THOC1	THO complex 1 [Source:HGNC Symbol;Acc:HGNC:19070]

ULBP1	UL16 binding protein 1 [Source:HGNC Symbol;Acc:HGNC:14893]
YIPF5	Yip1 domain family member 5 [Source:HGNC Symbol;Acc:HGNC:24877]
ZFP91	ZFP91 zinc finger protein [Source:HGNC Symbol;Acc:HGNC:14983]
ANGPTL3	angiopoietin like 3 [Source:HGNC Symbol;Acc:HGNC:491]
BACH2	BTB domain and CNC homolog 2 [Source:HGNC Symbol;Acc:HGNC:14078]
BPTF	bromodomain PHD finger transcription factor [Source:HGNC Symbol;Acc:HGNC:3581]
CAPN6	calpain 6 [Source:HGNC Symbol;Acc:HGNC:1483]
COX15	COX15 cytochrome c oxidase assembly homolog [Source:HGNC Symbol;Acc:HGNC:2263]
EFEMP2	EGF containing fibulin like extracellular matrix protein 2 [Source:HGNC Symbol;Acc:HGNC:3219]
FGF23	fibroblast growth factor 23 [Source:HGNC Symbol;Acc:HGNC:3680]
GALNT6	polypeptide N-acetylgalactosaminyltransferase 6 [Source:HGNC Symbol;Acc:HGNC:4128]
GLRX3	glutaredoxin 3 [Source:HGNC Symbol;Acc:HGNC:15987]
HEBP2	heme binding protein 2 [Source:HGNC Symbol;Acc:HGNC:15716]
HES5	hes family bHLH transcription factor 5 [Source:HGNC Symbol;Acc:HGNC:19764]
IBSP	integrin binding sialoprotein [Source:HGNC Symbol;Acc:HGNC:5341]
PKDCC	protein kinase domain containing, cytoplasmic [Source:HGNC Symbol;Acc:HGNC:25123]
PRKCSH	protein kinase C substrate 80K-H [Source:HGNC Symbol;Acc:HGNC:9411]
PTP4A1	protein tyrosine phosphatase type IVA, member 1 [Source:HGNC Symbol;Acc:HGNC:9634]
RAPGEF3	Rap guanine nucleotide exchange factor 3 [Source:HGNC Symbol;Acc:HGNC:16629]
RPL4	ribosomal protein L4 [Source:HGNC Symbol;Acc:HGNC:10353]
RPS12	ribosomal protein S12 [Source:HGNC Symbol;Acc:HGNC:10385]
SCGB1A1	secretoglobin family 1A member 1 [Source:HGNC Symbol;Acc:HGNC:12523]
ADGRD1	adhesion G protein-coupled receptor D1 [Source:HGNC Symbol;Acc:HGNC:19893]
BAG6	BCL2 associated athanogene 6 [Source:HGNC Symbol;Acc:HGNC:13919]
CDC73	cell division cycle 73 [Source:HGNC Symbol;Acc:HGNC:16783]
CDK5RAP3	CDK5 regulatory subunit associated protein 3 [Source:HGNC Symbol;Acc:HGNC:18673]
CHP1	calcineurin like EF-hand protein 1 [Source:HGNC Symbol;Acc:HGNC:17433]
ETNK2	ethanolamine kinase 2 [Source:HGNC Symbol;Acc:HGNC:25575]
FAM110B	family with sequence similarity 110 member B [Source:HGNC Symbol;Acc:HGNC:28587]
FKBP3	FK506 binding protein 3 [Source:HGNC Symbol;Acc:HGNC:3719]
GNL2	G protein nucleolar 2 [Source:HGNC Symbol;Acc:HGNC:29925]
GPAT4	glycerol-3-phosphate acyltransferase 4 [Source:HGNC Symbol;Acc:HGNC:20880]
GRHL3	grainyhead like transcription factor 3 [Source:HGNC Symbol;Acc:HGNC:25839]
GYPA	glycophorin A (MNS blood group) [Source:HGNC Symbol;Acc:HGNC:4702]
HIPK1	homeodomain interacting protein kinase 1 [Source:HGNC Symbol;Acc:HGNC:19006]
MRPL3	mitochondrial ribosomal protein L3 [Source:HGNC Symbol;Acc:HGNC:10379]
MRPS27	mitochondrial ribosomal protein S27 [Source:HGNC Symbol;Acc:HGNC:14512]

NDUFB4	NADH:ubiquinone oxidoreductase subunit B4 [Source:HGNC Symbol;Acc:HGNC:7699]
NDUFB9	NADH:ubiquinone oxidoreductase subunit B9 [Source:HGNC Symbol;Acc:HGNC:7704]
NFS1	NFS1 cysteine desulfurase [Source:HGNC Symbol;Acc:HGNC:15910]
NKX2-1	NK2 homeobox 1 [Source:HGNC Symbol;Acc:HGNC:11825]
NLRP12	NLR family pyrin domain containing 12 [Source:HGNC Symbol;Acc:HGNC:22938]
NOD1	nucleotide binding oligomerization domain containing 1 [Source:HGNC Symbol;Acc:HGNC:16390]
OGFR	opioid growth factor receptor [Source:HGNC Symbol;Acc:HGNC:15768]
POLD2	polymerase (DNA) delta 2, accessory subunit [Source:HGNC Symbol;Acc:HGNC:9176]
SMC6	structural maintenance of chromosomes 6 [Source:HGNC Symbol;Acc:HGNC:20466]
SNRPA1	small nuclear ribonucleoprotein polypeptide A' [Source:HGNC Symbol;Acc:HGNC:11152]
SNRPD2	small nuclear ribonucleoprotein D2 polypeptide [Source:HGNC Symbol;Acc:HGNC:11159]
SPAG1	sperm associated antigen 1 [Source:HGNC Symbol;Acc:HGNC:11212]
TOR1B	torsin family 1 member B [Source:HGNC Symbol;Acc:HGNC:11995]
TSPAN1	tetraspanin 1 [Source:HGNC Symbol;Acc:HGNC:20657]
UST	uronyl 2-sulfotransferase [Source:HGNC Symbol;Acc:HGNC:17223]
YARS2	tyrosyl-tRNA synthetase 2 [Source:HGNC Symbol;Acc:HGNC:24249]
ZNF292	zinc finger protein 292 [Source:HGNC Symbol;Acc:HGNC:18410]
DEDD2	death effector domain containing 2 [Source:HGNC Symbol;Acc:HGNC:24450]
HMGCL	3-hydroxymethyl-3-methylglutaryl-CoA lyase [Source:HGNC Symbol;Acc:HGNC:5005]
PKP4	plakophilin 4 [Source:HGNC Symbol;Acc:HGNC:9026]
RPS27A	ribosomal protein S27a [Source:HGNC Symbol;Acc:HGNC:10417]
SLC44A4	solute carrier family 44 member 4 [Source:HGNC Symbol;Acc:HGNC:13941]
TNRC6B	trinucleotide repeat containing 6B [Source:HGNC Symbol;Acc:HGNC:29190]
AMMECR1	Alport syndrome, mental retardation, midface hypoplasia and elliptocytosis chromosomal region gene 1 [Source:HGNC Symbol;Acc:HGNC:467]
APPBP2	amyloid beta precursor protein binding protein 2 [Source:HGNC Symbol;Acc:HGNC:622]
ARID4A	AT-rich interaction domain 4A [Source:HGNC Symbol;Acc:HGNC:9885]
ARL3	ADP ribosylation factor like GTPase 3 [Source:HGNC Symbol;Acc:HGNC:694]
BNIP2	BCL2/adenovirus E1B 19kDa interacting protein 2 [Source:HGNC Symbol;Acc:HGNC:1083]
CD3E	CD3e molecule [Source:HGNC Symbol;Acc:HGNC:1674]
CDCA4	cell division cycle associated 4 [Source:HGNC Symbol;Acc:HGNC:14625]
CEP128	centrosomal protein 128 [Source:HGNC Symbol;Acc:HGNC:20359]
CEP152	centrosomal protein 152 [Source:HGNC Symbol;Acc:HGNC:29298]
COQ7	coenzyme Q7, hydroxylase [Source:HGNC Symbol;Acc:HGNC:2244]
DCPS	decapping enzyme, scavenger [Source:HGNC Symbol;Acc:HGNC:29812]
DCX	doublecortin [Source:HGNC Symbol;Acc:HGNC:2714]
DERL1	derlin 1 [Source:HGNC Symbol;Acc:HGNC:28454]
DHX15	DEAH-box helicase 15 [Source:HGNC Symbol;Acc:HGNC:2738]

DNA2	DNA replication helicase/nuclease 2 [Source:HGNC Symbol;Acc:HGNC:2939]
FITM2	fat storage inducing transmembrane protein 2 [Source:HGNC Symbol;Acc:HGNC:16135]
IK	IK cytokine, down-regulator of HLA II [Source:HGNC Symbol;Acc:HGNC:5958]
ILF2	interleukin enhancer binding factor 2 [Source:HGNC Symbol;Acc:HGNC:6037]
INTS7	integrator complex subunit 7 [Source:HGNC Symbol;Acc:HGNC:24484]
L2HGDH	L-2-hydroxyglutarate dehydrogenase [Source:HGNC Symbol;Acc:HGNC:20499]
LCOR	ligand dependent nuclear receptor corepressor [Source:HGNC Symbol;Acc:HGNC:29503]
LSM4	LSM4 homolog, U6 small nuclear RNA and mRNA degradation associated [Source:HGNC Symbol;Acc:HGNC:17259]
MAT2B	methionine adenosyltransferase 2B [Source:HGNC Symbol;Acc:HGNC:6905]
MBTPS1	membrane bound transcription factor peptidase, site 1 [Source:HGNC Symbol;Acc:HGNC:15456]
MGAT4A	mannosyl (alpha-1,3-)-glycoprotein beta-1,4-N-acetylglucosaminyltransferase, isozyme A [Source:HGNC Symbol;Acc:HGNC:7047]
MIA	melanoma inhibitory activity [Source:HGNC Symbol;Acc:HGNC:7076]
NDUFA6	NADH:ubiquinone oxidoreductase subunit A6 [Source:HGNC Symbol;Acc:HGNC:7690]
NOP16	NOP16 nucleolar protein [Source:HGNC Symbol;Acc:HGNC:26934]
NUCB1	nucleobindin 1 [Source:HGNC Symbol;Acc:HGNC:8043]
PDCD2	programmed cell death 2 [Source:HGNC Symbol;Acc:HGNC:8762]
PDXP	pyridoxal phosphatase [Source:HGNC Symbol;Acc:HGNC:30259]
PNPO	pyridoxamine 5'-phosphate oxidase [Source:HGNC Symbol;Acc:HGNC:30260]
PYROXD2	pyridine nucleotide-disulphide oxidoreductase domain 2 [Source:HGNC Symbol;Acc:HGNC:23517]
RAB10	RAB10, member RAS oncogene family [Source:HGNC Symbol;Acc:HGNC:9759]
RAB11B	RAB11B, member RAS oncogene family [Source:HGNC Symbol;Acc:HGNC:9761]
RAB18	RAB18, member RAS oncogene family [Source:HGNC Symbol;Acc:HGNC:14244]
RAB2A	RAB2A, member RAS oncogene family [Source:HGNC Symbol;Acc:HGNC:9763]
RAB8A	RAB8A, member RAS oncogene family [Source:HGNC Symbol;Acc:HGNC:7007]
RBM15	RNA binding motif protein 15 [Source:HGNC Symbol;Acc:HGNC:14959]
RMI2	RecQ mediated genome instability 2 [Source:HGNC Symbol;Acc:HGNC:28349]
RNF144A	ring finger protein 144A [Source:HGNC Symbol;Acc:HGNC:20457]
RRP1B	ribosomal RNA processing 1B [Source:HGNC Symbol;Acc:HGNC:23818]
RSRC1	arginine and serine rich coiled-coil 1 [Source:HGNC Symbol;Acc:HGNC:24152]
SDHD	succinate dehydrogenase complex subunit D [Source:HGNC Symbol;Acc:HGNC:10683]
SEC11C	SEC11 homolog C, signal peptidase complex subunit [Source:HGNC Symbol;Acc:HGNC:23400]
SENP7	SUMO1/sentrin specific peptidase 7 [Source:HGNC Symbol;Acc:HGNC:30402]
SF3A1	splicing factor 3a subunit 1 [Source:HGNC Symbol;Acc:HGNC:10765]
SGCB	sarcoglycan beta [Source:HGNC Symbol;Acc:HGNC:10806]
SH3BGRL2	SH3 domain binding glutamate rich protein like 2 [Source:HGNC Symbol;Acc:HGNC:15567]

SKA3	spindle and kinetochore associated complex subunit 3 [Source:HGNC Symbol;Acc:HGNC:20262]
SLC25A11	solute carrier family 25 member 11 [Source:HGNC Symbol;Acc:HGNC:10981]
SOX3	SRY-box 3 [Source:HGNC Symbol;Acc:HGNC:11199]
SPEN	spen family transcriptional repressor [Source:HGNC Symbol;Acc:HGNC:17575]
STRN3	striatin 3 [Source:HGNC Symbol;Acc:HGNC:15720]
TAF1	TATA-box binding protein associated factor 1 [Source:HGNC Symbol;Acc:HGNC:11535]
TEX264	testis expressed 264 [Source:HGNC Symbol;Acc:HGNC:30247]
THRAP3	thyroid hormone receptor associated protein 3 [Source:HGNC Symbol;Acc:HGNC:22964]
TMEM107	transmembrane protein 107 [Source:HGNC Symbol;Acc:HGNC:28128]
AMOTL2	angiomotin like 2 [Source:HGNC Symbol;Acc:HGNC:17812]
ASGR1	asialoglycoprotein receptor 1 [Source:HGNC Symbol;Acc:HGNC:742]
AVPI1	arginine vasopressin induced 1 [Source:HGNC Symbol;Acc:HGNC:30898]
CLDN4	claudin 4 [Source:HGNC Symbol;Acc:HGNC:2046]
HYAL1	hyaluronoglucosaminidase 1 [Source:HGNC Symbol;Acc:HGNC:5320]
KIFC3	kinesin family member C3 [Source:HGNC Symbol;Acc:HGNC:6326]
MGAT2	mannosyl (alpha-1,6-)-glycoprotein beta-1,2-N-acetylglucosaminyltransferase [Source:HGNC Symbol;Acc:HGNC:7045]
MPEG1	macrophage expressed 1 [Source:HGNC Symbol;Acc:HGNC:29619]
PIGR	polymeric immunoglobulin receptor [Source:HGNC Symbol;Acc:HGNC:8968]
PPP2R1B	protein phosphatase 2 scaffold subunit Abeta [Source:HGNC Symbol;Acc:HGNC:9303]
PZP	PZP, alpha-2-macroglobulin like [Source:HGNC Symbol;Acc:HGNC:9750]
RAPGEF2	Rap guanine nucleotide exchange factor 2 [Source:HGNC Symbol;Acc:HGNC:16854]
RHBG	Rh family B glycoprotein (gene/pseudogene) [Source:HGNC Symbol;Acc:HGNC:14572]
XRCC4	X-ray repair cross complementing 4 [Source:HGNC Symbol;Acc:HGNC:12831]
ABHD11	abhydrolase domain containing 11 [Source:HGNC Symbol;Acc:HGNC:16407]
ADCY4	adenylate cyclase 4 [Source:HGNC Symbol;Acc:HGNC:235]
BBS2	Bardet-Biedl syndrome 2 [Source:HGNC Symbol;Acc:HGNC:967]
C15orf48	chromosome 15 open reading frame 48 [Source:HGNC Symbol;Acc:HGNC:29898]
CCKAR	cholecystokinin A receptor [Source:HGNC Symbol;Acc:HGNC:1570]
CDK5RAP2	CDK5 regulatory subunit associated protein 2 [Source:HGNC Symbol;Acc:HGNC:18672]
DONSON	downstream neighbor of SON [Source:HGNC Symbol;Acc:HGNC:2993]
EFTUD2	elongation factor Tu GTP binding domain containing 2 [Source:HGNC Symbol;Acc:HGNC:30858]
FAM111B	family with sequence similarity 111 member B [Source:HGNC Symbol;Acc:HGNC:24200]
FAM171B	family with sequence similarity 171 member B [Source:HGNC Symbol;Acc:HGNC:29412]
IGSF6	immunoglobulin superfamily member 6 [Source:HGNC Symbol;Acc:HGNC:5953]
IPO9	importin 9 [Source:HGNC Symbol;Acc:HGNC:19425]
MED13	mediator complex subunit 13 [Source:HGNC Symbol;Acc:HGNC:22474]

MFSD4A	major facilitator superfamily domain containing 4A [Source:HGNC Symbol;Acc:HGNC:25433]
MLYCD	malonyl-CoA decarboxylase [Source:HGNC Symbol;Acc:HGNC:7150]
MTFR2	mitochondrial fission regulator 2 [Source:HGNC Symbol;Acc:HGNC:21115]
NCOA6	nuclear receptor coactivator 6 [Source:HGNC Symbol;Acc:HGNC:15936]
NRM	nurim (nuclear envelope membrane protein) [Source:HGNC Symbol;Acc:HGNC:8003]
SNRNP25	small nuclear ribonucleoprotein U11/U12 subunit 25 [Source:HGNC Symbol;Acc:HGNC:14161]
SSRP1	structure specific recognition protein 1 [Source:HGNC Symbol;Acc:HGNC:11327]
SURF4	surfeit 4 [Source:HGNC Symbol;Acc:HGNC:11476]
TBX1	T-box 1 [Source:HGNC Symbol;Acc:HGNC:11592]
TLK1	tousled like kinase 1 [Source:HGNC Symbol;Acc:HGNC:11841]
TMEM135	transmembrane protein 135 [Source:HGNC Symbol;Acc:HGNC:26167]
TMEM64	transmembrane protein 64 [Source:HGNC Symbol;Acc:HGNC:25441]
VAPA	VAMP associated protein A [Source:HGNC Symbol;Acc:HGNC:12648]
CHRDL1	chordin-like 1 [Source:HGNC Symbol;Acc:HGNC:29861]
EEF1B2	eukaryotic translation elongation factor 1 beta 2 [Source:HGNC Symbol;Acc:HGNC:3208]
HINT1	histidine triad nucleotide binding protein 1 [Source:HGNC Symbol;Acc:HGNC:4912]
KCNK2	potassium two pore domain channel subfamily K member 2 [Source:HGNC Symbol;Acc:HGNC:6277]
KRT16	keratin 16 [Source:HGNC Symbol;Acc:HGNC:6423]
MMP11	matrix metallopeptidase 11 [Source:HGNC Symbol;Acc:HGNC:7157]
PELO	pelota homolog (Drosophila) [Source:HGNC Symbol;Acc:HGNC:8829]
PHLDB1	pleckstrin homology like domain family B member 1 [Source:HGNC Symbol;Acc:HGNC:23697]
SLC22A12	solute carrier family 22 member 12 [Source:HGNC Symbol;Acc:HGNC:17989]
SPTBN2	spectrin beta, non-erythrocytic 2 [Source:HGNC Symbol;Acc:HGNC:11276]
SSBP2	single stranded DNA binding protein 2 [Source:HGNC Symbol;Acc:HGNC:15831]
UCHL1	ubiquitin C-terminal hydrolase L1 [Source:HGNC Symbol;Acc:HGNC:12513]
ACYP1	acylphosphatase 1 [Source:HGNC Symbol;Acc:HGNC:179]
AGPS	alkylglycerone phosphate synthase [Source:HGNC Symbol;Acc:HGNC:327]
AGTRAP	angiotensin II receptor associated protein [Source:HGNC Symbol;Acc:HGNC:13539]
ARPP21	cAMP regulated phosphoprotein 21 [Source:HGNC Symbol;Acc:HGNC:16968]
COMMD3	COMM domain containing 3 [Source:HGNC Symbol;Acc:HGNC:23332]
DMXL2	Dmx like 2 [Source:HGNC Symbol;Acc:HGNC:2938]
HAS1	hyaluronan synthase 1 [Source:HGNC Symbol;Acc:HGNC:4818]
HAX1	HCLS1 associated protein X-1 [Source:HGNC Symbol;Acc:HGNC:16915]
IFT122	intraflagellar transport 122 [Source:HGNC Symbol;Acc:HGNC:13556]
IRF2BPL	interferon regulatory factor 2 binding protein like [Source:HGNC Symbol;Acc:HGNC:14282]
MRPL19	mitochondrial ribosomal protein L19 [Source:HGNC Symbol;Acc:HGNC:14052]
MRPL33	mitochondrial ribosomal protein L33 [Source:HGNC Symbol;Acc:HGNC:14487]

MTMR4	myotubularin related protein 4 [Source:HGNC Symbol;Acc:HGNC:7452]
PHF14	PHD finger protein 14 [Source:HGNC Symbol;Acc:HGNC:22203]
PHTF1	putative homeodomain transcription factor 1 [Source:HGNC Symbol;Acc:HGNC:8939]
PRRC2A	proline rich coiled-coil 2A [Source:HGNC Symbol;Acc:HGNC:13918]
RAB43	RAB43, member RAS oncogene family [Source:HGNC Symbol;Acc:HGNC:19983]
RBM19	RNA binding motif protein 19 [Source:HGNC Symbol;Acc:HGNC:29098]
SALL4	spalt like transcription factor 4 [Source:HGNC Symbol;Acc:HGNC:15924]
STK38L	serine/threonine kinase 38 like [Source:HGNC Symbol;Acc:HGNC:17848]
THSD4	thrombospondin type 1 domain containing 4 [Source:HGNC Symbol;Acc:HGNC:25835]
ZC2HC1A	zinc finger C2HC-type containing 1A [Source:HGNC Symbol;Acc:HGNC:24277]
ACAD11	acyl-CoA dehydrogenase family member 11 [Source:HGNC Symbol;Acc:HGNC:30211]
ADGRG2	adhesion G protein-coupled receptor G2 [Source:HGNC Symbol;Acc:HGNC:4516]
CAMK2G	calcium/calmodulin dependent protein kinase II gamma [Source:HGNC Symbol;Acc:HGNC:1463]
CELA1	chymotrypsin like elastase family member 1 [Source:HGNC Symbol;Acc:HGNC:3308]
CLIP4	CAP-Gly domain containing linker protein family member 4 [Source:HGNC Symbol;Acc:HGNC:26108]
CPN1	carboxypeptidase N subunit 1 [Source:HGNC Symbol;Acc:HGNC:2312]
MT-CYB	mitochondrially encoded cytochrome b [Source:HGNC Symbol;Acc:HGNC:7427]
DLX5	distal-less homeobox 5 [Source:HGNC Symbol;Acc:HGNC:2918]
DPEP1	dipeptidase 1 (renal) [Source:HGNC Symbol;Acc:HGNC:3002]
EDN3	endothelin 3 [Source:HGNC Symbol;Acc:HGNC:3178]
F12	coagulation factor XII [Source:HGNC Symbol;Acc:HGNC:3530]
FADS3	fatty acid desaturase 3 [Source:HGNC Symbol;Acc:HGNC:3576]
GCGR	glucagon receptor [Source:HGNC Symbol;Acc:HGNC:4192]
HRH2	histamine receptor H2 [Source:HGNC Symbol;Acc:HGNC:5183]
IRAK3	interleukin 1 receptor associated kinase 3 [Source:HGNC Symbol;Acc:HGNC:17020]
ITIH2	inter-alpha-trypsin inhibitor heavy chain 2 [Source:HGNC Symbol;Acc:HGNC:6167]
MAGI1	membrane associated guanylate kinase, WW and PDZ domain containing 1 [Source:HGNC Symbol;Acc:HGNC:946]
NRGN	neurogranin [Source:HGNC Symbol;Acc:HGNC:8000]
ONECUT1	one cut homeobox 1 [Source:HGNC Symbol;Acc:HGNC:8138]
PIR	pirin [Source:HGNC Symbol;Acc:HGNC:30048]
SATB1	SATB homeobox 1 [Source:HGNC Symbol;Acc:HGNC:10541]
SULT2B1	sulfotransferase family 2B member 1 [Source:HGNC Symbol;Acc:HGNC:11459]
AHCTF1	AT-hook containing transcription factor 1 [Source:HGNC Symbol;Acc:HGNC:24618]
AP3D1	adaptor related protein complex 3 delta 1 subunit [Source:HGNC Symbol;Acc:HGNC:568]
BUB3	BUB3, mitotic checkpoint protein [Source:HGNC Symbol;Acc:HGNC:1151]
CERS6	ceramide synthase 6 [Source:HGNC Symbol;Acc:HGNC:23826]
CSNK1G1	casein kinase 1 gamma 1 [Source:HGNC Symbol;Acc:HGNC:2454]
CTPS1	CTP synthase 1 [Source:HGNC Symbol;Acc:HGNC:2519]

FAM110C	family with sequence similarity 110 member C [Source:HGNC Symbol;Acc:HGNC:33340]
GHRH	growth hormone releasing hormone [Source:HGNC Symbol;Acc:HGNC:4265]
GPD1L	glycerol-3-phosphate dehydrogenase 1-like [Source:HGNC Symbol;Acc:HGNC:28956]
GRPEL2	GrpE like 2, mitochondrial [Source:HGNC Symbol;Acc:HGNC:21060]
HACD2	3-hydroxyacyl-CoA dehydratase 2 [Source:HGNC Symbol;Acc:HGNC:9640]
KAT5	lysine acetyltransferase 5 [Source:HGNC Symbol;Acc:HGNC:5275]
MAP1A	microtubule associated protein 1A [Source:HGNC Symbol;Acc:HGNC:6835]
MICB	MHC class I polypeptide-related sequence B [Source:HGNC Symbol;Acc:HGNC:7091]
MTPN	myotrophin [Source:HGNC Symbol;Acc:HGNC:15667]
N4BP1	NEDD4 binding protein 1 [Source:HGNC Symbol;Acc:HGNC:29850]
NDC1	NDC1 transmembrane nucleoporin [Source:HGNC Symbol;Acc:HGNC:25525]
PDSS1	prenyl (decaprenyl) diphosphate synthase, subunit 1 [Source:HGNC Symbol;Acc:HGNC:17759]
PTGES2	prostaglandin E synthase 2 [Source:HGNC Symbol;Acc:HGNC:17822]
PTPRA	protein tyrosine phosphatase, receptor type A [Source:HGNC Symbol;Acc:HGNC:9664]
RHOJ	ras homolog family member J [Source:HGNC Symbol;Acc:HGNC:688]
RPL19	ribosomal protein L19 [Source:HGNC Symbol;Acc:HGNC:10312]
RPL39	ribosomal protein L39 [Source:HGNC Symbol;Acc:HGNC:10350]
SAR1A	secretion associated Ras related GTPase 1A [Source:HGNC Symbol;Acc:HGNC:10534]
SH3PXD2B	SH3 and PX domains 2B [Source:HGNC Symbol;Acc:HGNC:29242]
SNRPE	small nuclear ribonucleoprotein polypeptide E [Source:HGNC Symbol;Acc:HGNC:11161]
SRGAP3	SLIT-ROBO Rho GTPase activating protein 3 [Source:HGNC Symbol;Acc:HGNC:19744]
SURF1	surfeit 1 [Source:HGNC Symbol;Acc:HGNC:11474]
TBC1D5	TBC1 domain family member 5 [Source:HGNC Symbol;Acc:HGNC:19166]
TIMM13	translocase of inner mitochondrial membrane 13 [Source:HGNC Symbol;Acc:HGNC:11816]
TMEM117	transmembrane protein 117 [Source:HGNC Symbol;Acc:HGNC:25308]
TMEM33	transmembrane protein 33 [Source:HGNC Symbol;Acc:HGNC:25541]
COX6C	cytochrome c oxidase subunit 6C [Source:HGNC Symbol;Acc:HGNC:2285]
CRABP1	cellular retinoic acid binding protein 1 [Source:HGNC Symbol;Acc:HGNC:2338]
EYA1	EYA transcriptional coactivator and phosphatase 1 [Source:HGNC Symbol;Acc:HGNC:3519]
FXYD6	FXYD domain containing ion transport regulator 6 [Source:HGNC Symbol;Acc:HGNC:4030]
KIF18B	kinesin family member 18B [Source:HGNC Symbol;Acc:HGNC:27102]
MCCC2	methylcrotonoyl-CoA carboxylase 2 [Source:HGNC Symbol;Acc:HGNC:6937]
PKMYT1	protein kinase, membrane associated tyrosine/threonine 1 [Source:HGNC Symbol;Acc:HGNC:29650]
SLC20A2	solute carrier family 20 member 2 [Source:HGNC Symbol;Acc:HGNC:10947]
ZMIZ1	zinc finger MIZ-type containing 1 [Source:HGNC Symbol;Acc:HGNC:16493]
API5	apoptosis inhibitor 5 [Source:HGNC Symbol;Acc:HGNC:594]

ARL8B	ADP ribosylation factor like GTPase 8B [Source:HGNC Symbol;Acc:HGNC:25564]
ARSG	arylsulfatase G [Source:HGNC Symbol;Acc:HGNC:24102]
ATL3	atlastin GTPase 3 [Source:HGNC Symbol;Acc:HGNC:24526]
ATP8B2	ATPase phospholipid transporting 8B2 [Source:HGNC Symbol;Acc:HGNC:13534]
BCAS3	breast carcinoma amplified sequence 3 [Source:HGNC Symbol;Acc:HGNC:14347]
BUD31	BUD31 homolog [Source:HGNC Symbol;Acc:HGNC:29629]
CLCN7	chloride voltage-gated channel 7 [Source:HGNC Symbol;Acc:HGNC:2025]
CLEC4F	C-type lectin domain family 4 member F [Source:HGNC Symbol;Acc:HGNC:25357]
CPSF6	cleavage and polyadenylation specific factor 6 [Source:HGNC Symbol;Acc:HGNC:13871]
CTSV	cathepsin V [Source:HGNC Symbol;Acc:HGNC:2538]
FAIM	Fas apoptotic inhibitory molecule [Source:HGNC Symbol;Acc:HGNC:18703]
FOXD1	forkhead box D1 [Source:HGNC Symbol;Acc:HGNC:3802]
GBA	glucosylceramidase beta [Source:HGNC Symbol;Acc:HGNC:4177]
GDPD5	glycerophosphodiester phosphodiesterase domain containing 5 [Source:HGNC Symbol;Acc:HGNC:28804]
GHITM	growth hormone inducible transmembrane protein [Source:HGNC Symbol;Acc:HGNC:17281]
GPC6	glypican 6 [Source:HGNC Symbol;Acc:HGNC:4454]
HIRIP3	HIRA interacting protein 3 [Source:HGNC Symbol;Acc:HGNC:4917]
IGF2BP2	insulin like growth factor 2 mRNA binding protein 2 [Source:HGNC Symbol;Acc:HGNC:28867]
ISOC1	isochorismatase domain containing 1 [Source:HGNC Symbol;Acc:HGNC:24254]
KCNH1	potassium voltage-gated channel subfamily H member 1 [Source:HGNC Symbol;Acc:HGNC:6250]
MANEA	mannosidase endo-alpha [Source:HGNC Symbol;Acc:HGNC:21072]
MRPL20	mitochondrial ribosomal protein L20 [Source:HGNC Symbol;Acc:HGNC:14478]
NANOS1	nanos C2HC-type zinc finger 1 [Source:HGNC Symbol;Acc:HGNC:23044]
NDUFA4	NDUFA4, mitochondrial complex associated [Source:HGNC Symbol;Acc:HGNC:7687]
PDZK1IP1	PDZK1 interacting protein 1 [Source:HGNC Symbol;Acc:HGNC:16887]
PIAS3	protein inhibitor of activated STAT 3 [Source:HGNC Symbol;Acc:HGNC:16861]
PPP2R5A	protein phosphatase 2 regulatory subunit B'alpha [Source:HGNC Symbol;Acc:HGNC:9309]
PREB	prolactin regulatory element binding [Source:HGNC Symbol;Acc:HGNC:9356]
PRODH2	proline dehydrogenase 2 [Source:HGNC Symbol;Acc:HGNC:17325]
PRPSAP1	phosphoribosyl pyrophosphate synthetase-associated protein 1 [Source:HGNC Symbol;Acc:HGNC:9466]
QPRT	quinolinate phosphoribosyltransferase [Source:HGNC Symbol;Acc:HGNC:9755]
RASGRF1	Ras protein specific guanine nucleotide releasing factor 1 [Source:HGNC Symbol;Acc:HGNC:9875]
RNF6	ring finger protein 6 [Source:HGNC Symbol;Acc:HGNC:10069]
RNH1	ribonuclease/angiogenin inhibitor 1 [Source:HGNC Symbol;Acc:HGNC:10074]
RPS21	ribosomal protein S21 [Source:HGNC Symbol;Acc:HGNC:10409]
S100A16	S100 calcium binding protein A16 [Source:HGNC Symbol;Acc:HGNC:20441]

SH3BP4	SH3 domain binding protein 4 [Source:HGNC Symbol;Acc:HGNC:10826]
SKA2	spindle and kinetochore associated complex subunit 2 [Source:HGNC Symbol;Acc:HGNC:28006]
SMARCA5	SWI/SNF related, matrix associated, actin dependent regulator of chromatin, subfamily a, member 5 [Source:HGNC Symbol;Acc:HGNC:11101]
SNX5	sorting nexin 5 [Source:HGNC Symbol;Acc:HGNC:14969]
SOX13	SRY-box 13 [Source:HGNC Symbol;Acc:HGNC:11192]
SP4	Sp4 transcription factor [Source:HGNC Symbol;Acc:HGNC:11209]
SPI1	Spi-1 proto-oncogene [Source:HGNC Symbol;Acc:HGNC:11241]
SPSB4	splA/ryanodine receptor domain and SOCS box containing 4 [Source:HGNC Symbol;Acc:HGNC:30630]
STT3A	STT3A, catalytic subunit of the oligosaccharyltransferase complex [Source:HGNC Symbol;Acc:HGNC:6172]
TLK2	tousled like kinase 2 [Source:HGNC Symbol;Acc:HGNC:11842]
TM6SF1	transmembrane 6 superfamily member 1 [Source:HGNC Symbol;Acc:HGNC:11860]
TMEM38B	transmembrane protein 38B [Source:HGNC Symbol;Acc:HGNC:25535]
TNNT3	troponin T3, fast skeletal type [Source:HGNC Symbol;Acc:HGNC:11950]
TRAPPC6A	trafficking protein particle complex 6A [Source:HGNC Symbol;Acc:HGNC:23069]
TTF2	transcription termination factor 2 [Source:HGNC Symbol;Acc:HGNC:12398]
ZHX2	zinc fingers and homeoboxes 2 [Source:HGNC Symbol;Acc:HGNC:18513]
ASF1A	anti-silencing function 1A histone chaperone [Source:HGNC Symbol;Acc:HGNC:20995]
CST6	cystatin E/M [Source:HGNC Symbol;Acc:HGNC:2478]
EPHB2	EPH receptor B2 [Source:HGNC Symbol;Acc:HGNC:3393]
HSPB6	heat shock protein family B (small) member 6 [Source:HGNC Symbol;Acc:HGNC:26511]
ITIH1	inter-alpha-trypsin inhibitor heavy chain 1 [Source:HGNC Symbol;Acc:HGNC:6166]
LPAR6	lysophosphatidic acid receptor 6 [Source:HGNC Symbol;Acc:HGNC:15520]
MAPK6	mitogen-activated protein kinase 6 [Source:HGNC Symbol;Acc:HGNC:6879]
MND1	meiotic nuclear divisions 1 [Source:HGNC Symbol;Acc:HGNC:24839]
NPR2	natriuretic peptide receptor 2 [Source:HGNC Symbol;Acc:HGNC:7944]
NTN1	netrin 1 [Source:HGNC Symbol;Acc:HGNC:8029]
PALMD	palmdelphin [Source:HGNC Symbol;Acc:HGNC:15846]
PITPNC1	phosphatidylinositol transfer protein, cytoplasmic 1 [Source:HGNC Symbol;Acc:HGNC:21045]
PYCR1	pyrroline-5-carboxylate reductase 1 [Source:HGNC Symbol;Acc:HGNC:9721]
SCG2	secretogranin II [Source:HGNC Symbol;Acc:HGNC:10575]
SPAG9	sperm associated antigen 9 [Source:HGNC Symbol;Acc:HGNC:14524]
TRIM16	tripartite motif containing 16 [Source:HGNC Symbol;Acc:HGNC:17241]
WNT11	Wnt family member 11 [Source:HGNC Symbol;Acc:HGNC:12776]
BEX2	brain expressed X-linked 2 [Source:HGNC Symbol;Acc:HGNC:30933]
CENPQ	centromere protein Q [Source:HGNC Symbol;Acc:HGNC:21347]
CREBZF	CREB/ATF bZIP transcription factor [Source:HGNC Symbol;Acc:HGNC:24905]
DSN1	DSN1 homolog, MIS12 kinetochore complex component [Source:HGNC Symbol;Acc:HGNC:16165]

HOMER2	homer scaffolding protein 2 [Source:HGNC Symbol;Acc:HGNC:17513]
KDM5A	lysine demethylase 5A [Source:HGNC Symbol;Acc:HGNC:9886]
NADK2	NAD kinase 2, mitochondrial [Source:HGNC Symbol;Acc:HGNC:26404]
PTGES3	prostaglandin E synthase 3 [Source:HGNC Symbol;Acc:HGNC:16049]
PTPRR	protein tyrosine phosphatase, receptor type R [Source:HGNC Symbol;Acc:HGNC:9680]
RAB11A	RAB11A, member RAS oncogene family [Source:HGNC Symbol;Acc:HGNC:9760]
SHB	SH2 domain containing adaptor protein B [Source:HGNC Symbol;Acc:HGNC:10838]
SP100	SP100 nuclear antigen [Source:HGNC Symbol;Acc:HGNC:11206]
SRRT	serrate, RNA effector molecule [Source:HGNC Symbol;Acc:HGNC:24101]
TIMM10	translocase of inner mitochondrial membrane 10 homolog (yeast) [Source:HGNC Symbol;Acc:HGNC:11814]
TRMT6	tRNA methyltransferase 6 [Source:HGNC Symbol;Acc:HGNC:20900]
USP10	ubiquitin specific peptidase 10 [Source:HGNC Symbol;Acc:HGNC:12608]
WNT1	Wnt family member 1 [Source:HGNC Symbol;Acc:HGNC:12774]
ABL2	ABL proto-oncogene 2, non-receptor tyrosine kinase [Source:HGNC Symbol;Acc:HGNC:77]
MT-ATP6	mitochondrially encoded ATP synthase 6 [Source:HGNC Symbol;Acc:HGNC:7414]
CADPS2	calcium dependent secretion activator 2 [Source:HGNC Symbol;Acc:HGNC:16018]
EFNB1	ephrin B1 [Source:HGNC Symbol;Acc:HGNC:3226]
FLG	filaggrin [Source:HGNC Symbol;Acc:HGNC:3748]
GPNMB	glycoprotein nmb [Source:HGNC Symbol;Acc:HGNC:4462]
ILK	integrin linked kinase [Source:HGNC Symbol;Acc:HGNC:6040]
MOXD1	monooxygenase DBH like 1 [Source:HGNC Symbol;Acc:HGNC:21063]
PDE4B	phosphodiesterase 4B [Source:HGNC Symbol;Acc:HGNC:8781]
RPL22	ribosomal protein L22 [Source:HGNC Symbol;Acc:HGNC:10315]
SERPINB1	serpin family B member 1 [Source:HGNC Symbol;Acc:HGNC:3311]
TNIK	TRAF2 and NCK interacting kinase [Source:HGNC Symbol;Acc:HGNC:30765]
UBE2N	ubiquitin conjugating enzyme E2 N [Source:HGNC Symbol;Acc:HGNC:12492]
CAPZB	capping actin protein of muscle Z-line beta subunit [Source:HGNC Symbol;Acc:HGNC:1491]
CENPN	centromere protein N [Source:HGNC Symbol;Acc:HGNC:30873]
CHRNB1	cholinergic receptor nicotinic beta 1 subunit [Source:HGNC Symbol;Acc:HGNC:1961]
GINS3	GINS complex subunit 3 [Source:HGNC Symbol;Acc:HGNC:25851]
HNF1B	HNF1 homeobox B [Source:HGNC Symbol;Acc:HGNC:11630]
LY6D	lymphocyte antigen 6 complex, locus D [Source:HGNC Symbol;Acc:HGNC:13348]
MDFIC	MyoD family inhibitor domain containing [Source:HGNC Symbol;Acc:HGNC:28870]
MYOCD	myocardin [Source:HGNC Symbol;Acc:HGNC:16067]
NDUFA13	NADH:ubiquinone oxidoreductase subunit A13 [Source:HGNC Symbol;Acc:HGNC:17194]
NEO1	neogenin 1 [Source:HGNC Symbol;Acc:HGNC:7754]
NUP155	nucleoporin 155 [Source:HGNC Symbol;Acc:HGNC:8063]
PDCD11	programmed cell death 11 [Source:HGNC Symbol;Acc:HGNC:13408]

PLCB3	phospholipase C beta 3 [Source:HGNC Symbol;Acc:HGNC:9056]
RAB5C	RAB5C, member RAS oncogene family [Source:HGNC Symbol;Acc:HGNC:9785]
SLC15A3	solute carrier family 15 member 3 [Source:HGNC Symbol;Acc:HGNC:18068]
SRPRB	SRP receptor beta subunit [Source:HGNC Symbol;Acc:HGNC:24085]
TMX1	thioredoxin related transmembrane protein 1 [Source:HGNC Symbol;Acc:HGNC:15487]
UACA	uveal autoantigen with coiled-coil domains and ankyrin repeats [Source:HGNC Symbol;Acc:HGNC:15947]
WNT2	Wnt family member 2 [Source:HGNC Symbol;Acc:HGNC:12780]
ZSWIM6	zinc finger SWIM-type containing 6 [Source:HGNC Symbol;Acc:HGNC:29316]
EIF3B	eukaryotic translation initiation factor 3 subunit B [Source:HGNC Symbol;Acc:HGNC:3280]
HIBCH	3-hydroxyisobutyryl-CoA hydrolase [Source:HGNC Symbol;Acc:HGNC:4908]
KIF4A	kinesin family member 4A [Source:HGNC Symbol;Acc:HGNC:13339]
NECTIN3	nectin cell adhesion molecule 3 [Source:HGNC Symbol;Acc:HGNC:17664]
SLC47A2	solute carrier family 47 member 2 [Source:HGNC Symbol;Acc:HGNC:26439]
TRPV6	transient receptor potential cation channel subfamily V member 6 [Source:HGNC Symbol;Acc:HGNC:14006]
WDR1	WD repeat domain 1 [Source:HGNC Symbol;Acc:HGNC:12754]
BSCL2	BSCL2, seipin lipid droplet biogenesis associated [Source:HGNC Symbol;Acc:HGNC:15832]
CDK8	cyclin dependent kinase 8 [Source:HGNC Symbol;Acc:HGNC:1779]
CHAC2	ChaC cation transport regulator homolog 2 [Source:HGNC Symbol;Acc:HGNC:32363]
CHCHD3	coiled-coil-helix-coiled-coil-helix domain containing 3 [Source:HGNC Symbol;Acc:HGNC:21906]
DDOST	dolichyl-diphosphooligosaccharide--protein glycosyltransferase non-catalytic subunit [Source:HGNC Symbol;Acc:HGNC:2728]
EPS15	epidermal growth factor receptor pathway substrate 15 [Source:HGNC Symbol;Acc:HGNC:3419]
EZH1	enhancer of zeste 1 polycomb repressive complex 2 subunit [Source:HGNC Symbol;Acc:HGNC:3526]
GTF2B	general transcription factor IIB [Source:HGNC Symbol;Acc:HGNC:4648]
MAX	MYC associated factor X [Source:HGNC Symbol;Acc:HGNC:6913]
MIS18BP1	MIS18 binding protein 1 [Source:HGNC Symbol;Acc:HGNC:20190]
PARP2	poly(ADP-ribose) polymerase 2 [Source:HGNC Symbol;Acc:HGNC:272]
PARPBP	PARP1 binding protein [Source:HGNC Symbol;Acc:HGNC:26074]
PRKX	protein kinase, X-linked [Source:HGNC Symbol;Acc:HGNC:9441]
RAB23	RAB23, member RAS oncogene family [Source:HGNC Symbol;Acc:HGNC:14263]
STK17A	serine/threonine kinase 17a [Source:HGNC Symbol;Acc:HGNC:11395]
SULT1A3	sulfotransferase family 1A member 3 [Source:HGNC Symbol;Acc:HGNC:11455]
TAF15	TATA-box binding protein associated factor 15 [Source:HGNC Symbol;Acc:HGNC:11547]
TMEM106C	transmembrane protein 106C [Source:HGNC Symbol;Acc:HGNC:28775]
TOB2	transducer of ERBB2, 2 [Source:HGNC Symbol;Acc:HGNC:11980]

VPS13B	vacuolar protein sorting 13 homolog B [Source:HGNC Symbol;Acc:HGNC:2183]
VRK1	vaccinia related kinase 1 [Source:HGNC Symbol;Acc:HGNC:12718]
ZMYM2	zinc finger MYM-type containing 2 [Source:HGNC Symbol;Acc:HGNC:12989]
ACAA1	acetyl-CoA acyltransferase 1 [Source:HGNC Symbol;Acc:HGNC:82]
CASR	calcium sensing receptor [Source:HGNC Symbol;Acc:HGNC:1514]
COX5B	cytochrome c oxidase subunit 5B [Source:HGNC Symbol;Acc:HGNC:2269]
GPM6A	glycoprotein M6A [Source:HGNC Symbol;Acc:HGNC:4460]
NUCKS1	nuclear casein kinase and cyclin dependent kinase substrate 1 [Source:HGNC Symbol;Acc:HGNC:29923]
SMPDL3A	sphingomyelin phosphodiesterase acid like 3A [Source:HGNC Symbol;Acc:HGNC:17389]
ABCB9	ATP binding cassette subfamily B member 9 [Source:HGNC Symbol;Acc:HGNC:50]
ABCF2	ATP binding cassette subfamily F member 2 [Source:HGNC Symbol;Acc:HGNC:71]
ACMSD	aminocarboxymuconate semialdehyde decarboxylase [Source:HGNC Symbol;Acc:HGNC:19288]
ARF4	ADP ribosylation factor 4 [Source:HGNC Symbol;Acc:HGNC:655]
ARID1B	AT-rich interaction domain 1B [Source:HGNC Symbol;Acc:HGNC:18040]
ARL1	ADP ribosylation factor like GTPase 1 [Source:HGNC Symbol;Acc:HGNC:692]
AUTS2	autism susceptibility candidate 2 [Source:HGNC Symbol;Acc:HGNC:14262]
CAMP	cathelicidin antimicrobial peptide [Source:HGNC Symbol;Acc:HGNC:1472]
CBX1	chromobox 1 [Source:HGNC Symbol;Acc:HGNC:1551]
CCR4	C-C motif chemokine receptor 4 [Source:HGNC Symbol;Acc:HGNC:1605]
CDKAL1	CDK5 regulatory subunit associated protein 1 like 1 [Source:HGNC Symbol;Acc:HGNC:21050]
CLCN5	chloride voltage-gated channel 5 [Source:HGNC Symbol;Acc:HGNC:2023]
COX8A	cytochrome c oxidase subunit 8A [Source:HGNC Symbol;Acc:HGNC:2294]
CTNND2	catenin delta 2 [Source:HGNC Symbol;Acc:HGNC:2516]
CYB5R1	cytochrome b5 reductase 1 [Source:HGNC Symbol;Acc:HGNC:13397]
DDX10	DEAD-box helicase 10 [Source:HGNC Symbol;Acc:HGNC:2735]
DERL3	derlin 3 [Source:HGNC Symbol;Acc:HGNC:14236]
FAR1	fatty acyl-CoA reductase 1 [Source:HGNC Symbol;Acc:HGNC:26222]
GLRX2	glutaredoxin 2 [Source:HGNC Symbol;Acc:HGNC:16065]
H3F3B	H3 histone, family 3B (H3.3B) [Source:HGNC Symbol;Acc:HGNC:4765]
KHSRP	KH-type splicing regulatory protein [Source:HGNC Symbol;Acc:HGNC:6316]
LYAR	Ly1 antibody reactive [Source:HGNC Symbol;Acc:HGNC:26021]
MAP3K4	mitogen-activated protein kinase kinase kinase 4 [Source:HGNC Symbol;Acc:HGNC:6856]
NDUFA3	NADH:ubiquinone oxidoreductase subunit A3 [Source:HGNC Symbol;Acc:HGNC:7686]
NDUFS3	NADH:ubiquinone oxidoreductase core subunit S3 [Source:HGNC Symbol;Acc:HGNC:7710]
NDUFS5	NADH:ubiquinone oxidoreductase subunit S5 [Source:HGNC Symbol;Acc:HGNC:7712]
NPPC	natriuretic peptide C [Source:HGNC Symbol;Acc:HGNC:7941]

PARM1	prostate androgen-regulated mucin-like protein 1 [Source:HGNC Symbol;Acc:HGNC:24536]
PGP	phosphoglycolate phosphatase [Source:HGNC Symbol;Acc:HGNC:8909]
POLI	polymerase (DNA) iota [Source:HGNC Symbol;Acc:HGNC:9182]
RERG	RAS like estrogen regulated growth inhibitor [Source:HGNC Symbol;Acc:HGNC:15980]
RPRM	reprimo, TP53 dependent G2 arrest mediator candidate [Source:HGNC Symbol;Acc:HGNC:24201]
SRP72	signal recognition particle 72kDa [Source:HGNC Symbol;Acc:HGNC:11303]
ST3GAL3	ST3 beta-galactoside alpha-2,3-sialyltransferase 3 [Source:HGNC Symbol;Acc:HGNC:10866]
SUZ12	SUZ12 polycomb repressive complex 2 subunit [Source:HGNC Symbol;Acc:HGNC:17101]
SYBU	syntabulin [Source:HGNC Symbol;Acc:HGNC:26011]
TERF1	telomeric repeat binding factor 1 [Source:HGNC Symbol;Acc:HGNC:11728]
TFB1M	transcription factor B1, mitochondrial [Source:HGNC Symbol;Acc:HGNC:17037]
TIGAR	TP53 induced glycolysis regulatory phosphatase [Source:HGNC Symbol;Acc:HGNC:1185]
TRERF1	transcriptional regulating factor 1 [Source:HGNC Symbol;Acc:HGNC:18273]
TRIM63	tripartite motif containing 63 [Source:HGNC Symbol;Acc:HGNC:16007]
USP1	ubiquitin specific peptidase 1 [Source:HGNC Symbol;Acc:HGNC:12607]
ADRA1D	adrenoceptor alpha 1D [Source:HGNC Symbol;Acc:HGNC:280]
AIFM2	apoptosis inducing factor, mitochondria associated 2 [Source:HGNC Symbol;Acc:HGNC:21411]
ARRDC2	arrestin domain containing 2 [Source:HGNC Symbol;Acc:HGNC:25225]
DEPDC1B	DEP domain containing 1B [Source:HGNC Symbol;Acc:HGNC:24902]
DHCR24	24-dehydrocholesterol reductase [Source:HGNC Symbol;Acc:HGNC:2859]
GPCPD1	glycerophosphocholine phosphodiesterase 1 [Source:HGNC Symbol;Acc:HGNC:26957]
H6PD	hexose-6-phosphate dehydrogenase/glucose 1-dehydrogenase [Source:HGNC Symbol;Acc:HGNC:4795]
MEST	mesoderm specific transcript [Source:HGNC Symbol;Acc:HGNC:7028]
RPL10	ribosomal protein L10 [Source:HGNC Symbol;Acc:HGNC:10298]
RPL7	ribosomal protein L7 [Source:HGNC Symbol;Acc:HGNC:10363]
VHL	von Hippel-Lindau tumor suppressor [Source:HGNC Symbol;Acc:HGNC:12687]
ACOT13	acyl-CoA thioesterase 13 [Source:HGNC Symbol;Acc:HGNC:20999]
AGPAT4	1-acylglycerol-3-phosphate O-acyltransferase 4 [Source:HGNC Symbol;Acc:HGNC:20885]
ATG3	autophagy related 3 [Source:HGNC Symbol;Acc:HGNC:20962]
ATL2	atlastin GTPase 2 [Source:HGNC Symbol;Acc:HGNC:24047]
B3GNT5	UDP-GlcNAc:betaGal beta-1,3-N-acetylglucosaminyltransferase 5 [Source:HGNC Symbol;Acc:HGNC:15684]
CAV3	caveolin 3 [Source:HGNC Symbol;Acc:HGNC:1529]
CDK12	cyclin dependent kinase 12 [Source:HGNC Symbol;Acc:HGNC:24224]
CENPH	centromere protein H [Source:HGNC Symbol;Acc:HGNC:17268]

CTTNBP2NL	CTTNBP2 N-terminal like [Source:HGNC Symbol;Acc:HGNC:25330]
FMO4	flavin containing monooxygenase 4 [Source:HGNC Symbol;Acc:HGNC:3772]
GOLIM4	golgi integral membrane protein 4 [Source:HGNC Symbol;Acc:HGNC:15448]
MAMDC2	MAM domain containing 2 [Source:HGNC Symbol;Acc:HGNC:23673]
MECR	mitochondrial trans-2-enoyl-CoA reductase [Source:HGNC Symbol;Acc:HGNC:19691]
NACA	nascent polypeptide-associated complex alpha subunit [Source:HGNC Symbol;Acc:HGNC:7629]
PI4K2B	phosphatidylinositol 4-kinase type 2 beta [Source:HGNC Symbol;Acc:HGNC:18215]
SGO2	shugoshin 2 [Source:HGNC Symbol;Acc:HGNC:30812]
SLBP	stem-loop binding protein [Source:HGNC Symbol;Acc:HGNC:10904]
SLC27A3	solute carrier family 27 member 3 [Source:HGNC Symbol;Acc:HGNC:10997]
SLC9A3	solute carrier family 9 member A3 [Source:HGNC Symbol;Acc:HGNC:11073]
SRD5A3	steroid 5 alpha-reductase 3 [Source:HGNC Symbol;Acc:HGNC:25812]
TIMM8B	translocase of inner mitochondrial membrane 8 homolog B (yeast) [Source:HGNC Symbol;Acc:HGNC:11818]
TSC22D2	TSC22 domain family member 2 [Source:HGNC Symbol;Acc:HGNC:29095]
CA2	carbonic anhydrase 2 [Source:HGNC Symbol;Acc:HGNC:1373]
EHMT2	euchromatic histone lysine methyltransferase 2 [Source:HGNC Symbol;Acc:HGNC:14129]
EPB41L1	erythrocyte membrane protein band 4.1 like 1 [Source:HGNC Symbol;Acc:HGNC:3378]
FGA	fibrinogen alpha chain [Source:HGNC Symbol;Acc:HGNC:3661]
KRT14	keratin 14 [Source:HGNC Symbol;Acc:HGNC:6416]
MSX1	msh homeobox 1 [Source:HGNC Symbol;Acc:HGNC:7391]
RHEB	Ras homolog enriched in brain [Source:HGNC Symbol;Acc:HGNC:10011]
RPL13A	ribosomal protein L13a [Source:HGNC Symbol;Acc:HGNC:10304]
SOX18	SRY-box 18 [Source:HGNC Symbol;Acc:HGNC:11194]
TMEM37	transmembrane protein 37 [Source:HGNC Symbol;Acc:HGNC:18216]
ABI1	abl interactor 1 [Source:HGNC Symbol;Acc:HGNC:11320]
ANAPC1	anaphase promoting complex subunit 1 [Source:HGNC Symbol;Acc:HGNC:19988]
ARAF	A-Raf proto-oncogene, serine/threonine kinase [Source:HGNC Symbol;Acc:HGNC:646]
ATPIF1	ATPase inhibitory factor 1 [Source:HGNC Symbol;Acc:HGNC:871]
BEX3	brain expressed X-linked 3 [Source:HGNC Symbol;Acc:HGNC:13388]
BMP2K	BMP2 inducible kinase [Source:HGNC Symbol;Acc:HGNC:18041]
CBFB	core-binding factor, beta subunit [Source:HGNC Symbol;Acc:HGNC:1539]
CCT4	chaperonin containing TCP1 subunit 4 [Source:HGNC Symbol;Acc:HGNC:1617]
CELSR2	cadherin EGF LAG seven-pass G-type receptor 2 [Source:HGNC Symbol;Acc:HGNC:3231]
CHGB	chromogranin B [Source:HGNC Symbol;Acc:HGNC:1930]
CLYBL	citrate lyase beta like [Source:HGNC Symbol;Acc:HGNC:18355]
CPB2	carboxypeptidase B2 [Source:HGNC Symbol;Acc:HGNC:2300]
CYB561	cytochrome b561 [Source:HGNC Symbol;Acc:HGNC:2571]

CYP4F2	cytochrome P450 family 4 subfamily F member 2 [Source:HGNC Symbol;Acc:HGNC:2645]
DCAKD	dephospho-CoA kinase domain containing [Source:HGNC Symbol;Acc:HGNC:26238]
DYNLT3	dynein light chain Tctex-type 3 [Source:HGNC Symbol;Acc:HGNC:11694]
DYRK1A	dual specificity tyrosine phosphorylation regulated kinase 1A [Source:HGNC Symbol;Acc:HGNC:3091]
EEF1E1	eukaryotic translation elongation factor 1 epsilon 1 [Source:HGNC Symbol;Acc:HGNC:3212]
FMNL2	formin like 2 [Source:HGNC Symbol;Acc:HGNC:18267]
GMPPB	GDP-mannose pyrophosphorylase B [Source:HGNC Symbol;Acc:HGNC:22932]
HOXB9	homeobox B9 [Source:HGNC Symbol;Acc:HGNC:5120]
IFRD2	interferon-related developmental regulator 2 [Source:HGNC Symbol;Acc:HGNC:5457]
LMNB2	lamin B2 [Source:HGNC Symbol;Acc:HGNC:6638]
MCM8	minichromosome maintenance 8 homologous recombination repair factor [Source:HGNC Symbol;Acc:HGNC:16147]
MFAP5	microfibrillar associated protein 5 [Source:HGNC Symbol;Acc:HGNC:29673]
MPRIP	myosin phosphatase Rho interacting protein [Source:HGNC Symbol;Acc:HGNC:30321]
MSI2	musashi RNA binding protein 2 [Source:HGNC Symbol;Acc:HGNC:18585]
NAB1	NGFI-A binding protein 1 [Source:HGNC Symbol;Acc:HGNC:7626]
NDUFA7	NADH:ubiquinone oxidoreductase subunit A7 [Source:HGNC Symbol;Acc:HGNC:7691]
NOC3L	NOC3 like DNA replication regulator [Source:HGNC Symbol;Acc:HGNC:24034]
NPHS2	NPHS2 podocin [Source:HGNC Symbol;Acc:HGNC:13394]
NUP98	nucleoporin 98 [Source:HGNC Symbol;Acc:HGNC:8068]
PGPEP1	pyroglutamyl-peptidase I [Source:HGNC Symbol;Acc:HGNC:13568]
PKD2	polycystin 2, transient receptor potential cation channel [Source:HGNC Symbol;Acc:HGNC:9009]
PLEKHA5	pleckstrin homology domain containing A5 [Source:HGNC Symbol;Acc:HGNC:30036]
PLOD3	procollagen-lysine,2-oxoglutarate 5-dioxygenase 3 [Source:HGNC Symbol;Acc:HGNC:9083]
RAB1A	RAB1A, member RAS oncogene family [Source:HGNC Symbol;Acc:HGNC:9758]
RASGRP2	RAS guanyl releasing protein 2 [Source:HGNC Symbol;Acc:HGNC:9879]
RBM33	RNA binding motif protein 33 [Source:HGNC Symbol;Acc:HGNC:27223]
RNF128	ring finger protein 128, E3 ubiquitin protein ligase [Source:HGNC Symbol;Acc:HGNC:21153]
SLC17A5	solute carrier family 17 member 5 [Source:HGNC Symbol;Acc:HGNC:10933]
SMC3	structural maintenance of chromosomes 3 [Source:HGNC Symbol;Acc:HGNC:2468]
STIL	SCL/TAL1 interrupting locus [Source:HGNC Symbol;Acc:HGNC:10879]
TBC1D2	TBC1 domain family member 2 [Source:HGNC Symbol;Acc:HGNC:18026]
TCOF1	treacle ribosome biogenesis factor 1 [Source:HGNC Symbol;Acc:HGNC:11654]
TECR	trans-2,3-enoyl-CoA reductase [Source:HGNC Symbol;Acc:HGNC:4551]
TMED3	transmembrane p24 trafficking protein 3 [Source:HGNC Symbol;Acc:HGNC:28889]
TRAM1	translocation associated membrane protein 1 [Source:HGNC Symbol;Acc:HGNC:20568]
UBE2L3	ubiquitin conjugating enzyme E2 L3 [Source:HGNC Symbol;Acc:HGNC:12488]

UQCRB	ubiquinol-cytochrome c reductase binding protein [Source:HGNC Symbol;Acc:HGNC:12582]
WASL	Wiskott-Aldrich syndrome like [Source:HGNC Symbol;Acc:HGNC:12735]
WDR43	WD repeat domain 43 [Source:HGNC Symbol;Acc:HGNC:28945]
ZMYM3	zinc finger MYM-type containing 3 [Source:HGNC Symbol;Acc:HGNC:13054]
ADH5	alcohol dehydrogenase 5 (class III), chi polypeptide [Source:HGNC Symbol;Acc:HGNC:253]
APLP2	amyloid beta precursor like protein 2 [Source:HGNC Symbol;Acc:HGNC:598]
BAG1	BCL2 associated athanogene 1 [Source:HGNC Symbol;Acc:HGNC:937]
CENPK	centromere protein K [Source:HGNC Symbol;Acc:HGNC:29479]
DRD4	dopamine receptor D4 [Source:HGNC Symbol;Acc:HGNC:3025]
EEF1D	eukaryotic translation elongation factor 1 delta [Source:HGNC Symbol;Acc:HGNC:3211]
EIF3A	eukaryotic translation initiation factor 3 subunit A [Source:HGNC Symbol;Acc:HGNC:3271]
FXYD5	FXYD domain containing ion transport regulator 5 [Source:HGNC Symbol;Acc:HGNC:4029]
GM2A	GM2 ganglioside activator [Source:HGNC Symbol;Acc:HGNC:4367]
GNAO1	G protein subunit alpha o1 [Source:HGNC Symbol;Acc:HGNC:4389]
HRH3	histamine receptor H3 [Source:HGNC Symbol;Acc:HGNC:5184]
MAPKAPK3	mitogen-activated protein kinase-activated protein kinase 3 [Source:HGNC Symbol;Acc:HGNC:6888]
ORM2	orosomucoid 2 [Source:HGNC Symbol;Acc:HGNC:8499]
PDGFRL	platelet derived growth factor receptor like [Source:HGNC Symbol;Acc:HGNC:8805]
PELI1	pellino E3 ubiquitin protein ligase 1 [Source:HGNC Symbol;Acc:HGNC:8827]
PHLDA1	pleckstrin homology like domain family A member 1 [Source:HGNC Symbol;Acc:HGNC:8933]
PSPH	phosphoserine phosphatase [Source:HGNC Symbol;Acc:HGNC:9577]
PTMS	parathymosin [Source:HGNC Symbol;Acc:HGNC:9629]
VMP1	vacuole membrane protein 1 [Source:HGNC Symbol;Acc:HGNC:29559]
ACIN1	apoptotic chromatin condensation inducer 1 [Source:HGNC Symbol;Acc:HGNC:17066]
APOBEC3B	apolipoprotein B mRNA editing enzyme catalytic subunit 3B [Source:HGNC Symbol;Acc:HGNC:17352]
CDC42BPA	CDC42 binding protein kinase alpha [Source:HGNC Symbol;Acc:HGNC:1737]
CEP57	centrosomal protein 57 [Source:HGNC Symbol;Acc:HGNC:30794]
CREB3L2	cAMP responsive element binding protein 3 like 2 [Source:HGNC Symbol;Acc:HGNC:23720]
DLST	dihydrolipoamide S-succinyltransferase [Source:HGNC Symbol;Acc:HGNC:2911]
ESPN	espin [Source:HGNC Symbol;Acc:HGNC:13281]
FOXO4	forkhead box O4 [Source:HGNC Symbol;Acc:HGNC:7139]
LACTB2	lactamase beta 2 [Source:HGNC Symbol;Acc:HGNC:18512]
MADD	MAP kinase activating death domain [Source:HGNC Symbol;Acc:HGNC:6766]
MAPK7	mitogen-activated protein kinase 7 [Source:HGNC Symbol;Acc:HGNC:6880]
MTCH2	mitochondrial carrier 2 [Source:HGNC Symbol;Acc:HGNC:17587]

PAX8	paired box 8 [Source:HGNC Symbol;Acc:HGNC:8622]
PLCD1	phospholipase C delta 1 [Source:HGNC Symbol;Acc:HGNC:9060]
PPP1R2	protein phosphatase 1 regulatory inhibitor subunit 2 [Source:HGNC Symbol;Acc:HGNC:9288]
PTPRN2	protein tyrosine phosphatase, receptor type N2 [Source:HGNC Symbol;Acc:HGNC:9677]
RAB6A	RAB6A, member RAS oncogene family [Source:HGNC Symbol;Acc:HGNC:9786]
S100A1	S100 calcium binding protein A1 [Source:HGNC Symbol;Acc:HGNC:10486]
SF3B1	splicing factor 3b subunit 1 [Source:HGNC Symbol;Acc:HGNC:10768]
SLA	Src-like-adaptor [Source:HGNC Symbol;Acc:HGNC:10902]
SMAD5	SMAD family member 5 [Source:HGNC Symbol;Acc:HGNC:6771]
SMARCE1	SWI/SNF related, matrix associated, actin dependent regulator of chromatin, subfamily e, member 1 [Source:HGNC Symbol;Acc:HGNC:11109]
SRF	serum response factor [Source:HGNC Symbol;Acc:HGNC:11291]
SRSF10	serine and arginine rich splicing factor 10 [Source:HGNC Symbol;Acc:HGNC:16713]
TDP1	tyrosyl-DNA phosphodiesterase 1 [Source:HGNC Symbol;Acc:HGNC:18884]
TUBG1	tubulin gamma 1 [Source:HGNC Symbol;Acc:HGNC:12417]
USP36	ubiquitin specific peptidase 36 [Source:HGNC Symbol;Acc:HGNC:20062]
WWC1	WW and C2 domain containing 1 [Source:HGNC Symbol;Acc:HGNC:29435]
ARNT2	aryl hydrocarbon receptor nuclear translocator 2 [Source:HGNC Symbol;Acc:HGNC:16876]
CHD2	chromodomain helicase DNA binding protein 2 [Source:HGNC Symbol;Acc:HGNC:1917]
DLL1	delta like canonical Notch ligand 1 [Source:HGNC Symbol;Acc:HGNC:2908]
EDNRA	endothelin receptor type A [Source:HGNC Symbol;Acc:HGNC:3179]
KIF22	kinesin family member 22 [Source:HGNC Symbol;Acc:HGNC:6391]
MBP	myelin basic protein [Source:HGNC Symbol;Acc:HGNC:6925]
MCF2L	MCF.2 cell line derived transforming sequence like [Source:HGNC Symbol;Acc:HGNC:14576]
PDE10A	phosphodiesterase 10A [Source:HGNC Symbol;Acc:HGNC:8772]
PECR	peroxisomal trans-2-enoyl-CoA reductase [Source:HGNC Symbol;Acc:HGNC:18281]
RARB	retinoic acid receptor beta [Source:HGNC Symbol;Acc:HGNC:9865]
SERPIND1	serpin family D member 1 [Source:HGNC Symbol;Acc:HGNC:4838]
SIRPA	signal regulatory protein alpha [Source:HGNC Symbol;Acc:HGNC:9662]
SSTR2	somatostatin receptor 2 [Source:HGNC Symbol;Acc:HGNC:11331]
TRPM7	transient receptor potential cation channel subfamily M member 7 [Source:HGNC Symbol;Acc:HGNC:17994]
VSNL1	visinin like 1 [Source:HGNC Symbol;Acc:HGNC:12722]
ACER2	alkaline ceramidase 2 [Source:HGNC Symbol;Acc:HGNC:23675]
ACTN2	actinin alpha 2 [Source:HGNC Symbol;Acc:HGNC:164]
ADAMTS4	ADAM metallopeptidase with thrombospondin type 1 motif 4 [Source:HGNC Symbol;Acc:HGNC:220]
ANO1	anoctamin 1 [Source:HGNC Symbol;Acc:HGNC:21625]

B3GALNT1	beta-1,3-N-acetylgalactosaminyltransferase 1 (globoside blood group) [Source:HGNC Symbol;Acc:HGNC:918]
BCAT2	branched chain amino acid transaminase 2 [Source:HGNC Symbol;Acc:HGNC:977]
CDC27	cell division cycle 27 [Source:HGNC Symbol;Acc:HGNC:1728]
CNBP	CCHC-type zinc finger nucleic acid binding protein [Source:HGNC Symbol;Acc:HGNC:13164]
COX6A1	cytochrome c oxidase subunit 6A1 [Source:HGNC Symbol;Acc:HGNC:2277]
DTNA	dystrobrevin alpha [Source:HGNC Symbol;Acc:HGNC:3057]
EPB41L2	erythrocyte membrane protein band 4.1 like 2 [Source:HGNC Symbol;Acc:HGNC:3379]
FOXA3	forkhead box A3 [Source:HGNC Symbol;Acc:HGNC:5023]
GPX8	glutathione peroxidase 8 (putative) [Source:HGNC Symbol;Acc:HGNC:33100]
HLCS	holocarboxylase synthetase [Source:HGNC Symbol;Acc:HGNC:4976]
HNRNPR	heterogeneous nuclear ribonucleoprotein R [Source:HGNC Symbol;Acc:HGNC:5047]
HR	hair growth associated [Source:HGNC Symbol;Acc:HGNC:5172]
LIMK1	LIM domain kinase 1 [Source:HGNC Symbol;Acc:HGNC:6613]
LTC4S	leukotriene C4 synthase [Source:HGNC Symbol;Acc:HGNC:6719]
MMP16	matrix metallopeptidase 16 [Source:HGNC Symbol;Acc:HGNC:7162]
NUMA1	nuclear mitotic apparatus protein 1 [Source:HGNC Symbol;Acc:HGNC:8059]
PEG3	paternally expressed 3 [Source:HGNC Symbol;Acc:HGNC:8826]
PHF19	PHD finger protein 19 [Source:HGNC Symbol;Acc:HGNC:24566]
RAB38	RAB38, member RAS oncogene family [Source:HGNC Symbol;Acc:HGNC:9776]
RAP1B	RAP1B, member of RAS oncogene family [Source:HGNC Symbol;Acc:HGNC:9857]
SEPHS2	selenophosphate synthetase 2 [Source:HGNC Symbol;Acc:HGNC:19686]
SLC25A22	solute carrier family 25 member 22 [Source:HGNC Symbol;Acc:HGNC:19954]
SUV39H1	suppressor of variegation 3-9 homolog 1 [Source:HGNC Symbol;Acc:HGNC:11479]
SUV39H2	suppressor of variegation 3-9 homolog 2 [Source:HGNC Symbol;Acc:HGNC:17287]
TMED5	transmembrane p24 trafficking protein 5 [Source:HGNC Symbol;Acc:HGNC:24251]
UQCRH	ubiquinol-cytochrome c reductase hinge protein [Source:HGNC Symbol;Acc:HGNC:12590]
WASF2	WAS protein family member 2 [Source:HGNC Symbol;Acc:HGNC:12733]
WFS1	wolframin ER transmembrane glycoprotein [Source:HGNC Symbol;Acc:HGNC:12762]
CHPT1	choline phosphotransferase 1 [Source:HGNC Symbol;Acc:HGNC:17852]
EDEM1	ER degradation enhancing alpha-mannosidase like protein 1 [Source:HGNC Symbol;Acc:HGNC:18967]
EEF1G	eukaryotic translation elongation factor 1 gamma [Source:HGNC Symbol;Acc:HGNC:3213]
ERCC4	ERCC excision repair 4, endonuclease catalytic subunit [Source:HGNC Symbol;Acc:HGNC:3436]
GAA	glucosidase alpha, acid [Source:HGNC Symbol;Acc:HGNC:4065]
MAPK11	mitogen-activated protein kinase 11 [Source:HGNC Symbol;Acc:HGNC:6873]
PPP3R1	protein phosphatase 3 regulatory subunit B, alpha [Source:HGNC Symbol;Acc:HGNC:9317]
RPL10A	ribosomal protein L10a [Source:HGNC Symbol;Acc:HGNC:10299]

SGK2	SGK2, serine/threonine kinase 2 [Source:HGNC Symbol;Acc:HGNC:13900]
AMH	anti-Mullerian hormone [Source:HGNC Symbol;Acc:HGNC:464]
ATXN3	ataxin 3 [Source:HGNC Symbol;Acc:HGNC:7106]
BCOR	BCL6 corepressor [Source:HGNC Symbol;Acc:HGNC:20893]
BCR	BCR, RhoGEF and GTPase activating protein [Source:HGNC Symbol;Acc:HGNC:1014]
BMPR1B	bone morphogenetic protein receptor type 1B [Source:HGNC Symbol;Acc:HGNC:1077]
BZW1	basic leucine zipper and W2 domains 1 [Source:HGNC Symbol;Acc:HGNC:18380]
DDIAS	DNA damage induced apoptosis suppressor [Source:HGNC Symbol;Acc:HGNC:26351]
DDX1	DEAD/H-box helicase 1 [Source:HGNC Symbol;Acc:HGNC:2734]
DNAJC9	DnaJ heat shock protein family (Hsp40) member C9 [Source:HGNC Symbol;Acc:HGNC:19123]
EIF4G2	eukaryotic translation initiation factor 4 gamma 2 [Source:HGNC Symbol;Acc:HGNC:3297]
EWSR1	EWS RNA binding protein 1 [Source:HGNC Symbol;Acc:HGNC:3508]
MBD1	methyl-CpG binding domain protein 1 [Source:HGNC Symbol;Acc:HGNC:6916]
PPAN	peter pan homolog (Drosophila) [Source:HGNC Symbol;Acc:HGNC:9227]
PTPN12	protein tyrosine phosphatase, non-receptor type 12 [Source:HGNC Symbol;Acc:HGNC:9645]
PTPN21	protein tyrosine phosphatase, non-receptor type 21 [Source:HGNC Symbol;Acc:HGNC:9651]
PXMP4	peroxisomal membrane protein 4 [Source:HGNC Symbol;Acc:HGNC:15920]
RAB3D	RAB3D, member RAS oncogene family [Source:HGNC Symbol;Acc:HGNC:9779]
RBM5	RNA binding motif protein 5 [Source:HGNC Symbol;Acc:HGNC:9902]
RPTOR	regulatory associated protein of MTOR complex 1 [Source:HGNC Symbol;Acc:HGNC:30287]
SH3GLB1	SH3 domain containing GRB2 like endophilin B1 [Source:HGNC Symbol;Acc:HGNC:10833]
SLC7A6	solute carrier family 7 member 6 [Source:HGNC Symbol;Acc:HGNC:11064]
STAMBPL1	STAM binding protein like 1 [Source:HGNC Symbol;Acc:HGNC:24105]
TICAM1	toll like receptor adaptor molecule 1 [Source:HGNC Symbol;Acc:HGNC:18348]
TIFA	TRAF interacting protein with forkhead associated domain [Source:HGNC Symbol;Acc:HGNC:19075]
WNT10B	Wnt family member 10B [Source:HGNC Symbol;Acc:HGNC:12775]
NOV	nephroblastoma overexpressed [Source:HGNC Symbol;Acc:HGNC:7885]
COX17	COX17 cytochrome c oxidase copper chaperone [Source:HGNC Symbol;Acc:HGNC:2264]
IQGAP1	IQ motif containing GTPase activating protein 1 [Source:HGNC Symbol;Acc:HGNC:6110]
KRT1	keratin 1 [Source:HGNC Symbol;Acc:HGNC:6412]
NET1	neuroepithelial cell transforming 1 [Source:HGNC Symbol;Acc:HGNC:14592]
RPLP0	ribosomal protein lateral stalk subunit P0 [Source:HGNC Symbol;Acc:HGNC:10371]
SLIT3	slit guidance ligand 3 [Source:HGNC Symbol;Acc:HGNC:11087]
VDAC2	voltage dependent anion channel 2 [Source:HGNC Symbol;Acc:HGNC:12672]

VGF	VGF nerve growth factor inducible [Source:HGNC Symbol;Acc:HGNC:12684]
ABHD6	abhydrolase domain containing 6 [Source:HGNC Symbol;Acc:HGNC:21398]
ABTB2	ankyrin repeat and BTB domain containing 2 [Source:HGNC Symbol;Acc:HGNC:23842]
ANXA11	annexin A11 [Source:HGNC Symbol;Acc:HGNC:535]
BMP5	bone morphogenetic protein 5 [Source:HGNC Symbol;Acc:HGNC:1072]
BORA	bora, aurora kinase A activator [Source:HGNC Symbol;Acc:HGNC:24724]
CAP2	CAP, adenylate cyclase-associated protein, 2 (yeast) [Source:HGNC Symbol;Acc:HGNC:20039]
CDCA7L	cell division cycle associated 7 like [Source:HGNC Symbol;Acc:HGNC:30777]
CKAP2L	cytoskeleton associated protein 2 like [Source:HGNC Symbol;Acc:HGNC:26877]
DSCC1	DNA replication and sister chromatid cohesion 1 [Source:HGNC Symbol;Acc:HGNC:24453]
EIF6	eukaryotic translation initiation factor 6 [Source:HGNC Symbol;Acc:HGNC:6159]
FGF10	fibroblast growth factor 10 [Source:HGNC Symbol;Acc:HGNC:3666]
FXYD2	FXYD domain containing ion transport regulator 2 [Source:HGNC Symbol;Acc:HGNC:4026]
GALNT10	polypeptide N-acetylgalactosaminyltransferase 10 [Source:HGNC Symbol;Acc:HGNC:19873]
GRINA	glutamate ionotropic receptor NMDA type subunit associated protein 1 [Source:HGNC Symbol;Acc:HGNC:4589]
INTS6	integrator complex subunit 6 [Source:HGNC Symbol;Acc:HGNC:14879]
KCNA5	potassium voltage-gated channel subfamily A member 5 [Source:HGNC Symbol;Acc:HGNC:6224]
LPGAT1	lysophosphatidylglycerol acyltransferase 1 [Source:HGNC Symbol;Acc:HGNC:28985]
MBD2	methyl-CpG binding domain protein 2 [Source:HGNC Symbol;Acc:HGNC:6917]
NELL2	neural EGFL like 2 [Source:HGNC Symbol;Acc:HGNC:7751]
NUP153	nucleoporin 153 [Source:HGNC Symbol;Acc:HGNC:8062]
PGM2L1	phosphoglucomutase 2-like 1 [Source:HGNC Symbol;Acc:HGNC:20898]
PRPF4B	pre-mRNA processing factor 4B [Source:HGNC Symbol;Acc:HGNC:17346]
PUS1	pseudouridylate synthase 1 [Source:HGNC Symbol;Acc:HGNC:15508]
RAB14	RAB14, member RAS oncogene family [Source:HGNC Symbol;Acc:HGNC:16524]
RAD54B	RAD54 homolog B (S. cerevisiae) [Source:HGNC Symbol;Acc:HGNC:17228]
RBM24	RNA binding motif protein 24 [Source:HGNC Symbol;Acc:HGNC:21539]
RPF2	ribosome production factor 2 homolog [Source:HGNC Symbol;Acc:HGNC:20870]
SCARA5	scavenger receptor class A member 5 [Source:HGNC Symbol;Acc:HGNC:28701]
SEC31A	SEC31 homolog A, COPII coat complex component [Source:HGNC Symbol;Acc:HGNC:17052]
SLC12A7	solute carrier family 12 member 7 [Source:HGNC Symbol;Acc:HGNC:10915]
SLC27A4	solute carrier family 27 member 4 [Source:HGNC Symbol;Acc:HGNC:10998]
SLC2A9	solute carrier family 2 member 9 [Source:HGNC Symbol;Acc:HGNC:13446]
SRI	sorcin [Source:HGNC Symbol;Acc:HGNC:11292]
SYVN1	synoviolin 1 [Source:HGNC Symbol;Acc:HGNC:20738]
TMED10	transmembrane p24 trafficking protein 10 [Source:HGNC Symbol;Acc:HGNC:16998]

TRIM28	tripartite motif containing 28 [Source:HGNC Symbol;Acc:HGNC:16384]
UBA7	ubiquitin like modifier activating enzyme 7 [Source:HGNC Symbol;Acc:HGNC:12471]
UBE3A	ubiquitin protein ligase E3A [Source:HGNC Symbol;Acc:HGNC:12496]
ZC3HAV1	zinc finger CCCH-type containing, antiviral 1 [Source:HGNC Symbol;Acc:HGNC:23721]
CCT5	chaperonin containing TCP1 subunit 5 [Source:HGNC Symbol;Acc:HGNC:1618]
CSAD	cysteine sulfinic acid decarboxylase [Source:HGNC Symbol;Acc:HGNC:18966]
DBH	dopamine beta-hydroxylase [Source:HGNC Symbol;Acc:HGNC:2689]
EDNRB	endothelin receptor type B [Source:HGNC Symbol;Acc:HGNC:3180]
GART	phosphoribosylglycinamide formyltransferase, phosphoribosylglycinamide synthetase, phosphoribosylaminoimidazole synthetase [Source:HGNC Symbol;Acc:HGNC:4163]
HSDL2	hydroxysteroid dehydrogenase like 2 [Source:HGNC Symbol;Acc:HGNC:18572]
IFITM2	interferon induced transmembrane protein 2 [Source:HGNC Symbol;Acc:HGNC:5413]
JMJD6	arginine demethylase and lysine hydroxylase [Source:HGNC Symbol;Acc:HGNC:19355]
LRG1	leucine rich alpha-2-glycoprotein 1 [Source:HGNC Symbol;Acc:HGNC:29480]
MDH2	malate dehydrogenase 2 [Source:HGNC Symbol;Acc:HGNC:6971]
PSMA4	proteasome subunit alpha 4 [Source:HGNC Symbol;Acc:HGNC:9533]
ABCD2	ATP binding cassette subfamily D member 2 [Source:HGNC Symbol;Acc:HGNC:66]
ACOT9	acyl-CoA thioesterase 9 [Source:HGNC Symbol;Acc:HGNC:17152]
ACTN4	actinin alpha 4 [Source:HGNC Symbol;Acc:HGNC:166]
ADGRE5	adhesion G protein-coupled receptor E5 [Source:HGNC Symbol;Acc:HGNC:1711]
CYC1	cytochrome c1 [Source:HGNC Symbol;Acc:HGNC:2579]
ENDOD1	endonuclease domain containing 1 [Source:HGNC Symbol;Acc:HGNC:29129]
FRMD6	FERM domain containing 6 [Source:HGNC Symbol;Acc:HGNC:19839]
GJA4	gap junction protein alpha 4 [Source:HGNC Symbol;Acc:HGNC:4278]
GLIPR2	GLI pathogenesis related 2 [Source:HGNC Symbol;Acc:HGNC:18007]
MRPL12	mitochondrial ribosomal protein L12 [Source:HGNC Symbol;Acc:HGNC:10378]
NDUFA2	NADH:ubiquinone oxidoreductase subunit A2 [Source:HGNC Symbol;Acc:HGNC:7685]
NDUFB8	NADH:ubiquinone oxidoreductase subunit B8 [Source:HGNC Symbol;Acc:HGNC:7703]
PLIN4	perilipin 4 [Source:HGNC Symbol;Acc:HGNC:29393]
RNASEH2A	ribonuclease H2 subunit A [Source:HGNC Symbol;Acc:HGNC:18518]
SGK3	serum/glucocorticoid regulated kinase family member 3 [Source:HGNC Symbol;Acc:HGNC:10812]
ADCY7	adenylate cyclase 7 [Source:HGNC Symbol;Acc:HGNC:238]
ADK	adenosine kinase [Source:HGNC Symbol;Acc:HGNC:257]
ELL2	elongation factor for RNA polymerase II 2 [Source:HGNC Symbol;Acc:HGNC:17064]
FBXO30	F-box protein 30 [Source:HGNC Symbol;Acc:HGNC:15600]
PSMC5	proteasome 26S subunit, ATPase 5 [Source:HGNC Symbol;Acc:HGNC:9552]
RAMP1	receptor activity modifying protein 1 [Source:HGNC Symbol;Acc:HGNC:9843]
SGMS1	sphingomyelin synthase 1 [Source:HGNC Symbol;Acc:HGNC:29799]
TNS3	tensin 3 [Source:HGNC Symbol;Acc:HGNC:21616]
AKT1S1	AKT1 substrate 1 [Source:HGNC Symbol;Acc:HGNC:28426]

ASAP1	ArfGAP with SH3 domain, ankyrin repeat and PH domain 1 [Source:HGNC Symbol;Acc:HGNC:2720]
ATF1	activating transcription factor 1 [Source:HGNC Symbol;Acc:HGNC:783]
ATOX1	antioxidant 1 copper chaperone [Source:HGNC Symbol;Acc:HGNC:798]
BAP1	BRCA1 associated protein 1 [Source:HGNC Symbol;Acc:HGNC:950]
CCL1	C-C motif chemokine ligand 1 [Source:HGNC Symbol;Acc:HGNC:10609]
CDV3	CDV3 homolog [Source:HGNC Symbol;Acc:HGNC:26928]
COX6B1	cytochrome c oxidase subunit 6B1 [Source:HGNC Symbol;Acc:HGNC:2280]
COX7B	cytochrome c oxidase subunit 7B [Source:HGNC Symbol;Acc:HGNC:2291]
DEPDC1	DEP domain containing 1 [Source:HGNC Symbol;Acc:HGNC:22949]
DHODH	dihydroorotate dehydrogenase (quinone) [Source:HGNC Symbol;Acc:HGNC:2867]
DHRS2	dehydrogenase/reductase 2 [Source:HGNC Symbol;Acc:HGNC:18349]
FANCG	Fanconi anemia complementation group G [Source:HGNC Symbol;Acc:HGNC:3588]
FUCA1	fucosidase, alpha-L- 1, tissue [Source:HGNC Symbol;Acc:HGNC:4006]
GPSM2	G-protein signaling modulator 2 [Source:HGNC Symbol;Acc:HGNC:29501]
GYS1	glycogen synthase 1 [Source:HGNC Symbol;Acc:HGNC:4706]
HDGF	hepatoma-derived growth factor [Source:HGNC Symbol;Acc:HGNC:4856]
HSD17B12	hydroxysteroid 17-beta dehydrogenase 12 [Source:HGNC Symbol;Acc:HGNC:18646]
IGF2BP3	insulin like growth factor 2 mRNA binding protein 3 [Source:HGNC Symbol;Acc:HGNC:28868]
KCNB1	potassium voltage-gated channel subfamily B member 1 [Source:HGNC Symbol;Acc:HGNC:6231]
MAG	myelin associated glycoprotein [Source:HGNC Symbol;Acc:HGNC:6783]
MPZL2	myelin protein zero like 2 [Source:HGNC Symbol;Acc:HGNC:3496]
MYOD1	myogenic differentiation 1 [Source:HGNC Symbol;Acc:HGNC:7611]
NDUFA5	NADH:ubiquinone oxidoreductase subunit A5 [Source:HGNC Symbol;Acc:HGNC:7688]
NDUFV2	NADH:ubiquinone oxidoreductase core subunit V2 [Source:HGNC Symbol;Acc:HGNC:7717]
NTN4	netrin 4 [Source:HGNC Symbol;Acc:HGNC:13658]
PIDD1	p53-induced death domain protein 1 [Source:HGNC Symbol;Acc:HGNC:16491]
PKIB	protein kinase (cAMP-dependent, catalytic) inhibitor beta [Source:HGNC Symbol;Acc:HGNC:9018]
PMS1	PMS1 homolog 1, mismatch repair system component [Source:HGNC Symbol;Acc:HGNC:9121]
PRKCI	protein kinase C iota [Source:HGNC Symbol;Acc:HGNC:9404]
SLC12A4	solute carrier family 12 member 4 [Source:HGNC Symbol;Acc:HGNC:10913]
SSX2IP	SSX family member 2 interacting protein [Source:HGNC Symbol;Acc:HGNC:16509]
TIMP4	TIMP metallopeptidase inhibitor 4 [Source:HGNC Symbol;Acc:HGNC:11823]
TIPIN	TIMELESS interacting protein [Source:HGNC Symbol;Acc:HGNC:30750]
XRCC2	X-ray repair cross complementing 2 [Source:HGNC Symbol;Acc:HGNC:12829]
ZCCHC7	zinc finger CCHC-type containing 7 [Source:HGNC Symbol;Acc:HGNC:26209]
ZFR	zinc finger RNA binding protein [Source:HGNC Symbol;Acc:HGNC:17277]
CDA	cytidine deaminase [Source:HGNC Symbol;Acc:HGNC:1712]

CRYL1	crystallin lambda 1 [Source:HGNC Symbol;Acc:HGNC:18246]
HIGD1A	HIG1 hypoxia inducible domain family member 1A [Source:HGNC Symbol;Acc:HGNC:29527]
IGFALS	insulin like growth factor binding protein acid labile subunit [Source:HGNC Symbol;Acc:HGNC:5468]
LASP1	LIM and SH3 protein 1 [Source:HGNC Symbol;Acc:HGNC:6513]
PTAFR	platelet activating factor receptor [Source:HGNC Symbol;Acc:HGNC:9582]
SLC5A3	solute carrier family 5 member 3 [Source:HGNC Symbol;Acc:HGNC:11038]
SLCO4A1	solute carrier organic anion transporter family member 4A1 [Source:HGNC Symbol;Acc:HGNC:10953]
TM4SF1	transmembrane 4 L six family member 1 [Source:HGNC Symbol;Acc:HGNC:11853]
APRT	adenine phosphoribosyltransferase [Source:HGNC Symbol;Acc:HGNC:626]
BLNK	B-cell linker [Source:HGNC Symbol;Acc:HGNC:14211]
WISP1	WNT1 inducible signaling pathway protein 1 [Source:HGNC Symbol;Acc:HGNC:12769]
CCR6	C-C motif chemokine receptor 6 [Source:HGNC Symbol;Acc:HGNC:1607]
CSNK2A2	casein kinase 2 alpha 2 [Source:HGNC Symbol;Acc:HGNC:2459]
DDAH2	dimethylarginine dimethylaminohydrolase 2 [Source:HGNC Symbol;Acc:HGNC:2716]
DNM2	dynamin 2 [Source:HGNC Symbol;Acc:HGNC:2974]
FGF8	fibroblast growth factor 8 [Source:HGNC Symbol;Acc:HGNC:3686]
GAB1	GRB2 associated binding protein 1 [Source:HGNC Symbol;Acc:HGNC:4066]
GPLD1	glycosylphosphatidylinositol specific phospholipase D1 [Source:HGNC Symbol;Acc:HGNC:4459]
HJURP	Holliday junction recognition protein [Source:HGNC Symbol;Acc:HGNC:25444]
NEIL3	nei like DNA glycosylase 3 [Source:HGNC Symbol;Acc:HGNC:24573]
PAPSS1	3'-phosphoadenosine 5'-phosphosulfate synthase 1 [Source:HGNC Symbol;Acc:HGNC:8603]
PIM3	Pim-3 proto-oncogene, serine/threonine kinase [Source:HGNC Symbol;Acc:HGNC:19310]
PLP2	proteolipid protein 2 [Source:HGNC Symbol;Acc:HGNC:9087]
PSEN2	presenilin 2 [Source:HGNC Symbol;Acc:HGNC:9509]
PTGER1	prostaglandin E receptor 1 [Source:HGNC Symbol;Acc:HGNC:9593]
RAB8B	RAB8B, member RAS oncogene family [Source:HGNC Symbol;Acc:HGNC:30273]
RAPH1	Ras association (RalGDS/AF-6) and pleckstrin homology domains 1 [Source:HGNC Symbol;Acc:HGNC:14436]
RFTN1	raftlin, lipid raft linker 1 [Source:HGNC Symbol;Acc:HGNC:30278]
SACS	sacsin molecular chaperone [Source:HGNC Symbol;Acc:HGNC:10519]
SEL1L	SEL1L ERAD E3 ligase adaptor subunit [Source:HGNC Symbol;Acc:HGNC:10717]
TAPBP	TAP binding protein (tapasin) [Source:HGNC Symbol;Acc:HGNC:11566]
TRIM59	tripartite motif containing 59 [Source:HGNC Symbol;Acc:HGNC:30834]
TUFM	Tu translation elongation factor, mitochondrial [Source:HGNC Symbol;Acc:HGNC:12420]
UAP1	UDP-N-acetylglucosamine pyrophosphorylase 1 [Source:HGNC Symbol;Acc:HGNC:12457]

ZFAND5	zinc finger AN1-type containing 5 [Source:HGNC Symbol;Acc:HGNC:13008]
DTL	denticleless E3 ubiquitin protein ligase homolog [Source:HGNC Symbol;Acc:HGNC:30288]
HSD17B10	hydroxysteroid 17-beta dehydrogenase 10 [Source:HGNC Symbol;Acc:HGNC:4800]
LY6E	lymphocyte antigen 6 complex, locus E [Source:HGNC Symbol;Acc:HGNC:6727]
MAFK	MAF bZIP transcription factor K [Source:HGNC Symbol;Acc:HGNC:6782]
MAP3K6	mitogen-activated protein kinase kinase kinase 6 [Source:HGNC Symbol;Acc:HGNC:6858]
PSMA1	proteasome subunit alpha 1 [Source:HGNC Symbol;Acc:HGNC:9530]
SLC39A10	solute carrier family 39 member 10 [Source:HGNC Symbol;Acc:HGNC:20861]
SRSF2	serine and arginine rich splicing factor 2 [Source:HGNC Symbol;Acc:HGNC:10783]
XPA	XPA, DNA damage recognition and repair factor [Source:HGNC Symbol;Acc:HGNC:12814]
ADAM10	ADAM metallopeptidase domain 10 [Source:HGNC Symbol;Acc:HGNC:188]
ALOX12	arachidonate 12-lipoxygenase, 12S type [Source:HGNC Symbol;Acc:HGNC:429]
ARHGAP5	Rho GTPase activating protein 5 [Source:HGNC Symbol;Acc:HGNC:675]
CORO1C	coronin 1C [Source:HGNC Symbol;Acc:HGNC:2254]
CXCR3	C-X-C motif chemokine receptor 3 [Source:HGNC Symbol;Acc:HGNC:4540]
FAM107B	family with sequence similarity 107 member B [Source:HGNC Symbol;Acc:HGNC:23726]
FGD4	FYVE, RhoGEF and PH domain containing 4 [Source:HGNC Symbol;Acc:HGNC:19125]
INPP4B	inositol polyphosphate-4-phosphatase type II B [Source:HGNC Symbol;Acc:HGNC:6075]
NKX3-1	NK3 homeobox 1 [Source:HGNC Symbol;Acc:HGNC:7838]
PDX1	pancreatic and duodenal homeobox 1 [Source:HGNC Symbol;Acc:HGNC:6107]
PID1	phosphotyrosine interaction domain containing 1 [Source:HGNC Symbol;Acc:HGNC:26084]
PLXNB2	plexin B2 [Source:HGNC Symbol;Acc:HGNC:9104]
PPAT	phosphoribosyl pyrophosphate amidotransferase [Source:HGNC Symbol;Acc:HGNC:9238]
PRKAR2A	protein kinase cAMP-dependent type II regulatory subunit alpha [Source:HGNC Symbol;Acc:HGNC:9391]
PRPS2	phosphoribosyl pyrophosphate synthetase 2 [Source:HGNC Symbol;Acc:HGNC:9465]
RAB20	RAB20, member RAS oncogene family [Source:HGNC Symbol;Acc:HGNC:18260]
RAD54L	RAD54-like (S. cerevisiae) [Source:HGNC Symbol;Acc:HGNC:9826]
RHOBTB1	Rho related BTB domain containing 1 [Source:HGNC Symbol;Acc:HGNC:18738]
RPA3	replication protein A3 [Source:HGNC Symbol;Acc:HGNC:10291]
SDS	serine dehydratase [Source:HGNC Symbol;Acc:HGNC:10691]
STK39	serine/threonine kinase 39 [Source:HGNC Symbol;Acc:HGNC:17717]
STRBP	spermatid perinuclear RNA binding protein [Source:HGNC Symbol;Acc:HGNC:16462]
TCF12	transcription factor 12 [Source:HGNC Symbol;Acc:HGNC:11623]
TLR5	toll like receptor 5 [Source:HGNC Symbol;Acc:HGNC:11851]
TROAP	trophinin associated protein [Source:HGNC Symbol;Acc:HGNC:12327]
VAMP2	vesicle associated membrane protein 2 [Source:HGNC Symbol;Acc:HGNC:12643]

ZWILCH	zwilch kinetochore protein [Source:HGNC Symbol;Acc:HGNC:25468]
CYP3A5	cytochrome P450 family 3 subfamily A member 5 [Source:HGNC Symbol;Acc:HGNC:2638]
NCAPD2	non-SMC condensin I complex subunit D2 [Source:HGNC Symbol;Acc:HGNC:24305]
ZFP36L2	ZFP36 ring finger protein-like 2 [Source:HGNC Symbol;Acc:HGNC:1108]
ADCY9	adenylate cyclase 9 [Source:HGNC Symbol;Acc:HGNC:240]
CD53	CD53 molecule [Source:HGNC Symbol;Acc:HGNC:1686]
CDC34	cell division cycle 34 [Source:HGNC Symbol;Acc:HGNC:1734]
CENPW	centromere protein W [Source:HGNC Symbol;Acc:HGNC:21488]
CHRNA5	cholinergic receptor nicotinic alpha 5 subunit [Source:HGNC Symbol;Acc:HGNC:1959]
CORO2A	coronin 2A [Source:HGNC Symbol;Acc:HGNC:2255]
CUX1	cut like homeobox 1 [Source:HGNC Symbol;Acc:HGNC:2557]
DPP7	dipeptidyl peptidase 7 [Source:HGNC Symbol;Acc:HGNC:14892]
EPHA1	EPH receptor A1 [Source:HGNC Symbol;Acc:HGNC:3385]
FAM107A	family with sequence similarity 107 member A [Source:HGNC Symbol;Acc:HGNC:30827]
FGR	FGR proto-oncogene, Src family tyrosine kinase [Source:HGNC Symbol;Acc:HGNC:3697]
FOXN3	forkhead box N3 [Source:HGNC Symbol;Acc:HGNC:1928]
GFPT2	glutamine-fructose-6-phosphate transaminase 2 [Source:HGNC Symbol;Acc:HGNC:4242]
HMGN2	high mobility group nucleosomal binding domain 2 [Source:HGNC Symbol;Acc:HGNC:4986]
MRPS18B	mitochondrial ribosomal protein S18B [Source:HGNC Symbol;Acc:HGNC:14516]
PFKFB1	6-phosphofructo-2-kinase/fructose-2,6-biphosphatase 1 [Source:HGNC Symbol;Acc:HGNC:8872]
PHLDB2	pleckstrin homology like domain family B member 2 [Source:HGNC Symbol;Acc:HGNC:29573]
PPM1A	protein phosphatase, Mg2+/Mn2+ dependent 1A [Source:HGNC Symbol;Acc:HGNC:9275]
PRDM16	PR domain 16 [Source:HGNC Symbol;Acc:HGNC:14000]
PSMC3IP	PSMC3 interacting protein [Source:HGNC Symbol;Acc:HGNC:17928]
PTPRE	protein tyrosine phosphatase, receptor type E [Source:HGNC Symbol;Acc:HGNC:9669]
PXDN	peroxidasin [Source:HGNC Symbol;Acc:HGNC:14966]
RAD18	RAD18, E3 ubiquitin protein ligase [Source:HGNC Symbol;Acc:HGNC:18278]
REEP5	receptor accessory protein 5 [Source:HGNC Symbol;Acc:HGNC:30077]
SAMD4A	sterile alpha motif domain containing 4A [Source:HGNC Symbol;Acc:HGNC:23023]
SFTPB	surfactant protein B [Source:HGNC Symbol;Acc:HGNC:10801]
SFXN2	sideroflexin 2 [Source:HGNC Symbol;Acc:HGNC:16086]
STBD1	starch binding domain 1 [Source:HGNC Symbol;Acc:HGNC:24854]
TJP3	tight junction protein 3 [Source:HGNC Symbol;Acc:HGNC:11829]
TNFRSF11A	tumor necrosis factor receptor superfamily member 11a [Source:HGNC Symbol;Acc:HGNC:11908]
UBE2H	ubiquitin conjugating enzyme E2 H [Source:HGNC Symbol;Acc:HGNC:12484]

USP7	ubiquitin specific peptidase 7 (herpes virus-associated) [Source:HGNC Symbol;Acc:HGNC:12630]
CNN1	calponin 1 [Source:HGNC Symbol;Acc:HGNC:2155]
DNAJB6	DnaJ heat shock protein family (Hsp40) member B6 [Source:HGNC Symbol;Acc:HGNC:14888]
GC	GC, vitamin D binding protein [Source:HGNC Symbol;Acc:HGNC:4187]
HES6	hes family bHLH transcription factor 6 [Source:HGNC Symbol;Acc:HGNC:18254]
HHEX	hematopoietically expressed homeobox [Source:HGNC Symbol;Acc:HGNC:4901]
LRP2	LDL receptor related protein 2 [Source:HGNC Symbol;Acc:HGNC:6694]
MYLIP	myosin regulatory light chain interacting protein [Source:HGNC Symbol;Acc:HGNC:21155]
OPRD1	opioid receptor delta 1 [Source:HGNC Symbol;Acc:HGNC:8153]
PSMA2	proteasome subunit alpha 2 [Source:HGNC Symbol;Acc:HGNC:9531]
RSRP1	arginine and serine rich protein 1 [Source:HGNC Symbol;Acc:HGNC:25234]
SFRP2	secreted frizzled related protein 2 [Source:HGNC Symbol;Acc:HGNC:10777]
SPON2	spondin 2 [Source:HGNC Symbol;Acc:HGNC:11253]
TNFAIP8	TNF alpha induced protein 8 [Source:HGNC Symbol;Acc:HGNC:17260]
AGR2	anterior gradient 2, protein disulphide isomerase family member [Source:HGNC Symbol;Acc:HGNC:328]
ALDH9A1	aldehyde dehydrogenase 9 family member A1 [Source:HGNC Symbol;Acc:HGNC:412]
CSE1L	chromosome segregation 1 like [Source:HGNC Symbol;Acc:HGNC:2431]
DBT	dihydrolipoamide branched chain transacylase E2 [Source:HGNC Symbol;Acc:HGNC:2698]
DOCK8	dedicator of cytokinesis 8 [Source:HGNC Symbol;Acc:HGNC:19191]
EI24	EI24, autophagy associated transmembrane protein [Source:HGNC Symbol;Acc:HGNC:13276]
ERCC6L	ERCC excision repair 6 like, spindle assembly checkpoint helicase [Source:HGNC Symbol;Acc:HGNC:20794]
IL17RB	interleukin 17 receptor B [Source:HGNC Symbol;Acc:HGNC:18015]
IRF2BP2	interferon regulatory factor 2 binding protein 2 [Source:HGNC Symbol;Acc:HGNC:21729]
KCNJ11	potassium voltage-gated channel subfamily J member 11 [Source:HGNC Symbol;Acc:HGNC:6257]
LONP1	lon peptidase 1, mitochondrial [Source:HGNC Symbol;Acc:HGNC:9479]
LPP	LIM domain containing preferred translocation partner in lipoma [Source:HGNC Symbol;Acc:HGNC:6679]
MATR3	matrin 3 [Source:HGNC Symbol;Acc:HGNC:6912]
MRPS6	mitochondrial ribosomal protein S6 [Source:HGNC Symbol;Acc:HGNC:14051]
MYO10	myosin X [Source:HGNC Symbol;Acc:HGNC:7593]
NCOA4	nuclear receptor coactivator 4 [Source:HGNC Symbol;Acc:HGNC:7671]
NTRK3	neurotrophic receptor tyrosine kinase 3 [Source:HGNC Symbol;Acc:HGNC:8033]
OSBPL3	oxysterol binding protein like 3 [Source:HGNC Symbol;Acc:HGNC:16370]
RAB27B	RAB27B, member RAS oncogene family [Source:HGNC Symbol;Acc:HGNC:9767]
RPS3A	ribosomal protein S3A [Source:HGNC Symbol;Acc:HGNC:10421]

SF1	splicing factor 1 [Source:HGNC Symbol;Acc:HGNC:12950]
SGO1	shugoshin 1 [Source:HGNC Symbol;Acc:HGNC:25088]
SLC41A2	solute carrier family 41 member 2 [Source:HGNC Symbol;Acc:HGNC:31045]
SP3	Sp3 transcription factor [Source:HGNC Symbol;Acc:HGNC:11208]
ST13	suppression of tumorigenicity 13 (colon carcinoma) (Hsp70 interacting protein) [Source:HGNC Symbol;Acc:HGNC:11343]
TBX21	T-box 21 [Source:HGNC Symbol;Acc:HGNC:11599]
TCP11L2	t-complex 11 like 2 [Source:HGNC Symbol;Acc:HGNC:28627]
TSC1	tuberous sclerosis 1 [Source:HGNC Symbol;Acc:HGNC:12362]
YBX1	Y-box binding protein 1 [Source:HGNC Symbol;Acc:HGNC:8014]
ACTG2	actin, gamma 2, smooth muscle, enteric [Source:HGNC Symbol;Acc:HGNC:145]
AMBP	alpha-1-microglobulin/bikunin precursor [Source:HGNC Symbol;Acc:HGNC:453]
CRAT	carnitine O-acetyltransferase [Source:HGNC Symbol;Acc:HGNC:2342]
CSRP2	cysteine and glycine rich protein 2 [Source:HGNC Symbol;Acc:HGNC:2470]
KRT17	keratin 17 [Source:HGNC Symbol;Acc:HGNC:6427]
LGMN	legumain [Source:HGNC Symbol;Acc:HGNC:9472]
MT1G	metallothionein 1G [Source:HGNC Symbol;Acc:HGNC:7399]
OSMR	oncostatin M receptor [Source:HGNC Symbol;Acc:HGNC:8507]
PVR	poliovirus receptor [Source:HGNC Symbol;Acc:HGNC:9705]
RPL13	ribosomal protein L13 [Source:HGNC Symbol;Acc:HGNC:10303]
SLC27A1	solute carrier family 27 member 1 [Source:HGNC Symbol;Acc:HGNC:10995]
ACOT4	acyl-CoA thioesterase 4 [Source:HGNC Symbol;Acc:HGNC:19748]
ACOT7	acyl-CoA thioesterase 7 [Source:HGNC Symbol;Acc:HGNC:24157]
AKT3	AKT serine/threonine kinase 3 [Source:HGNC Symbol;Acc:HGNC:393]
CLIP1	CAP-Gly domain containing linker protein 1 [Source:HGNC Symbol;Acc:HGNC:10461]
COMTD1	catechol-O-methyltransferase domain containing 1 [Source:HGNC Symbol;Acc:HGNC:26309]
CRIM1	cysteine rich transmembrane BMP regulator 1 (chordin-like) [Source:HGNC Symbol;Acc:HGNC:2359]
ELOVL3	ELOVL fatty acid elongase 3 [Source:HGNC Symbol;Acc:HGNC:18047]
ENTPD1	ectonucleoside triphosphate diphosphohydrolase 1 [Source:HGNC Symbol;Acc:HGNC:3363]
FGL1	fibrinogen like 1 [Source:HGNC Symbol;Acc:HGNC:3695]
GGH	gamma-glutamyl hydrolase [Source:HGNC Symbol;Acc:HGNC:4248]
HHIP	hedgehog interacting protein [Source:HGNC Symbol;Acc:HGNC:14866]
KNTC1	kinetochore associated 1 [Source:HGNC Symbol;Acc:HGNC:17255]
MAP2K5	mitogen-activated protein kinase kinase 5 [Source:HGNC Symbol;Acc:HGNC:6845]
MYBBP1A	MYB binding protein 1a [Source:HGNC Symbol;Acc:HGNC:7546]
MYH11	myosin, heavy chain 11, smooth muscle [Source:HGNC Symbol;Acc:HGNC:7569]
NFE2L1	nuclear factor, erythroid 2 like 1 [Source:HGNC Symbol;Acc:HGNC:7781]
PPP2CB	protein phosphatase 2 catalytic subunit beta [Source:HGNC Symbol;Acc:HGNC:9300]
PRDM2	PR domain 2 [Source:HGNC Symbol;Acc:HGNC:9347]

PRKAB2	protein kinase AMP-activated non-catalytic subunit beta 2 [Source:HGNC Symbol;Acc:HGNC:9379]
PRKCG	protein kinase C gamma [Source:HGNC Symbol;Acc:HGNC:9402]
PRKD1	protein kinase D1 [Source:HGNC Symbol;Acc:HGNC:9407]
PSMC6	proteasome 26S subunit, ATPase 6 [Source:HGNC Symbol;Acc:HGNC:9553]
PSME4	proteasome activator subunit 4 [Source:HGNC Symbol;Acc:HGNC:20635]
RFC5	replication factor C subunit 5 [Source:HGNC Symbol;Acc:HGNC:9973]
RHOQ	ras homolog family member Q [Source:HGNC Symbol;Acc:HGNC:17736]
SCPEP1	serine carboxypeptidase 1 [Source:HGNC Symbol;Acc:HGNC:29507]
SRRM2	serine/arginine repetitive matrix 2 [Source:HGNC Symbol;Acc:HGNC:16639]
TFAP2C	transcription factor AP-2 gamma [Source:HGNC Symbol;Acc:HGNC:11744]
TOMM20	translocase of outer mitochondrial membrane 20 [Source:HGNC Symbol;Acc:HGNC:20947]
TPMT	thiopurine S-methyltransferase [Source:HGNC Symbol;Acc:HGNC:12014]
YPEL5	yippee like 5 [Source:HGNC Symbol;Acc:HGNC:18329]
ZDHHC2	zinc finger DHHC-type containing 2 [Source:HGNC Symbol;Acc:HGNC:18469]
AKR1D1	aldo-keto reductase family 1 member D1 [Source:HGNC Symbol;Acc:HGNC:388]
ATP2B1	ATPase plasma membrane Ca2+ transporting 1 [Source:HGNC Symbol;Acc:HGNC:814]
ECHS1	enoyl-CoA hydratase, short chain, 1, mitochondrial [Source:HGNC Symbol;Acc:HGNC:3151]
FKBP1A	FK506 binding protein 1A [Source:HGNC Symbol;Acc:HGNC:3711]
PLS3	plastin 3 [Source:HGNC Symbol;Acc:HGNC:9091]
SRSF7	serine and arginine rich splicing factor 7 [Source:HGNC Symbol;Acc:HGNC:10789]
AACS	acetoacetyl-CoA synthetase [Source:HGNC Symbol;Acc:HGNC:21298]
CALU	calumenin [Source:HGNC Symbol;Acc:HGNC:1458]
CKAP4	cytoskeleton-associated protein 4 [Source:HGNC Symbol;Acc:HGNC:16991]
CTNND1	catenin delta 1 [Source:HGNC Symbol;Acc:HGNC:2515]
DRAM1	DNA damage regulated autophagy modulator 1 [Source:HGNC Symbol;Acc:HGNC:25645]
FAM111A	family with sequence similarity 111 member A [Source:HGNC Symbol;Acc:HGNC:24725]
FANCA	Fanconi anemia complementation group A [Source:HGNC Symbol;Acc:HGNC:3582]
FNDC3B	fibronectin type III domain containing 3B [Source:HGNC Symbol;Acc:HGNC:24670]
HIVEP1	human immunodeficiency virus type I enhancer binding protein 1 [Source:HGNC Symbol;Acc:HGNC:4920]
IFI6	interferon alpha inducible protein 6 [Source:HGNC Symbol;Acc:HGNC:4054]
ILF3	interleukin enhancer binding factor 3 [Source:HGNC Symbol;Acc:HGNC:6038]
LTBR	lymphotoxin beta receptor [Source:HGNC Symbol;Acc:HGNC:6718]
NDUFS4	NADH:ubiquinone oxidoreductase subunit S4 [Source:HGNC Symbol;Acc:HGNC:7711]
PRIM2	primase (DNA) subunit 2 [Source:HGNC Symbol;Acc:HGNC:9370]
PSMC3	proteasome 26S subunit, ATPase 3 [Source:HGNC Symbol;Acc:HGNC:9549]
RAD51C	RAD51 paralog C [Source:HGNC Symbol;Acc:HGNC:9820]
RANGAP1	Ran GTPase activating protein 1 [Source:HGNC Symbol;Acc:HGNC:9854]

RHOU	ras homolog family member U [Source:HGNC Symbol;Acc:HGNC:17794]
RPL3	ribosomal protein L3 [Source:HGNC Symbol;Acc:HGNC:10332]
SOS1	SOS Ras/Rac guanine nucleotide exchange factor 1 [Source:HGNC Symbol;Acc:HGNC:11187]
TPR	translocated promoter region, nuclear basket protein [Source:HGNC Symbol;Acc:HGNC:12017]
UCK2	uridine-cytidine kinase 2 [Source:HGNC Symbol;Acc:HGNC:12562]
WDHD1	WD repeat and HMG-box DNA binding protein 1 [Source:HGNC Symbol;Acc:HGNC:23170]
YBX3	Y-box binding protein 3 [Source:HGNC Symbol;Acc:HGNC:2428]
ZBTB10	zinc finger and BTB domain containing 10 [Source:HGNC Symbol;Acc:HGNC:30953]
ACADVL	acyl-CoA dehydrogenase, very long chain [Source:HGNC Symbol;Acc:HGNC:92]
FLNB	filamin B [Source:HGNC Symbol;Acc:HGNC:3755]
GABRB3	gamma-aminobutyric acid type A receptor beta3 subunit [Source:HGNC Symbol;Acc:HGNC:4083]
ID2	inhibitor of DNA binding 2, HLH protein [Source:HGNC Symbol;Acc:HGNC:5361]
IL1RAP	interleukin 1 receptor accessory protein [Source:HGNC Symbol;Acc:HGNC:5995]
MT1F	metallothionein 1F [Source:HGNC Symbol;Acc:HGNC:7398]
RETSAT	retinol saturase [Source:HGNC Symbol;Acc:HGNC:25991]
SERTAD1	SERTA domain containing 1 [Source:HGNC Symbol;Acc:HGNC:17932]
TACR1	tachykinin receptor 1 [Source:HGNC Symbol;Acc:HGNC:11526]
TRADD	TNFRSF1A associated via death domain [Source:HGNC Symbol;Acc:HGNC:12030]
ULK1	unc-51 like autophagy activating kinase 1 [Source:HGNC Symbol;Acc:HGNC:12558]
CCL8	C-C motif chemokine ligand 8 [Source:HGNC Symbol;Acc:HGNC:10635]
ERBB4	erb-b2 receptor tyrosine kinase 4 [Source:HGNC Symbol;Acc:HGNC:3432]
FAM13A	family with sequence similarity 13 member A [Source:HGNC Symbol;Acc:HGNC:19367]
GFRA2	GDNF family receptor alpha 2 [Source:HGNC Symbol;Acc:HGNC:4244]
GLA	galactosidase alpha [Source:HGNC Symbol;Acc:HGNC:4296]
GYS2	glycogen synthase 2 [Source:HGNC Symbol;Acc:HGNC:4707]
MID1IP1	MID1 interacting protein 1 [Source:HGNC Symbol;Acc:HGNC:20715]
PDHB	pyruvate dehydrogenase (lipoamide) beta [Source:HGNC Symbol;Acc:HGNC:8808]
PPP1CA	protein phosphatase 1 catalytic subunit alpha [Source:HGNC Symbol;Acc:HGNC:9281]
PSIP1	PC4 and SFRS1 interacting protein 1 [Source:HGNC Symbol;Acc:HGNC:9527]
APOD	apolipoprotein D [Source:HGNC Symbol;Acc:HGNC:612]
CAMK2D	calcium/calmodulin dependent protein kinase II delta [Source:HGNC Symbol;Acc:HGNC:1462]
CCR7	C-C motif chemokine receptor 7 [Source:HGNC Symbol;Acc:HGNC:1608]
FUS	FUS RNA binding protein [Source:HGNC Symbol;Acc:HGNC:4010]
PAPSS2	3'-phosphoadenosine 5'-phosphosulfate synthase 2 [Source:HGNC Symbol;Acc:HGNC:8604]
PPARGC1B	PPARG coactivator 1 beta [Source:HGNC Symbol;Acc:HGNC:30022]
ABCC8	ATP binding cassette subfamily C member 8 [Source:HGNC Symbol;Acc:HGNC:59]
ABCF1	ATP binding cassette subfamily F member 1 [Source:HGNC Symbol;Acc:HGNC:70]

AFF4	AF4/FMR2 family member 4 [Source:HGNC Symbol;Acc:HGNC:17869]
WISP2	WNT1 inducible signaling pathway protein 2 [Source:HGNC Symbol;Acc:HGNC:12770]
CES3	carboxylesterase 3 [Source:HGNC Symbol;Acc:HGNC:1865]
CLIC1	chloride intracellular channel 1 [Source:HGNC Symbol;Acc:HGNC:2062]
CSRP3	cysteine and glycine rich protein 3 [Source:HGNC Symbol;Acc:HGNC:2472]
DIAPH3	diaphanous related formin 3 [Source:HGNC Symbol;Acc:HGNC:15480]
DKC1	dyskerin pseudouridine synthase 1 [Source:HGNC Symbol;Acc:HGNC:2890]
DLAT	dihydrolipoamide S-acetyltransferase [Source:HGNC Symbol;Acc:HGNC:2896]
DNAJB11	DnaJ heat shock protein family (Hsp40) member B11 [Source:HGNC Symbol;Acc:HGNC:14889]
ETFB	electron transfer flavoprotein beta subunit [Source:HGNC Symbol;Acc:HGNC:3482]
ETHE1	ETHE1, persulfide dioxygenase [Source:HGNC Symbol;Acc:HGNC:23287]
FAM43A	family with sequence similarity 43 member A [Source:HGNC Symbol;Acc:HGNC:26888]
FDX1	ferredoxin 1 [Source:HGNC Symbol;Acc:HGNC:3638]
ITGA2B	integrin subunit alpha 2b [Source:HGNC Symbol;Acc:HGNC:6138]
LRP8	LDL receptor related protein 8 [Source:HGNC Symbol;Acc:HGNC:6700]
MR1	major histocompatibility complex, class I-related [Source:HGNC Symbol;Acc:HGNC:4975]
MTHFD1	methylenetetrahydrofolate dehydrogenase, cyclohydrolase and formyltetrahydrofolate synthetase 1 [Source:HGNC Symbol;Acc:HGNC:7432]
NUDT7	nudix hydrolase 7 [Source:HGNC Symbol;Acc:HGNC:8054]
PERP	PERP, TP53 apoptosis effector [Source:HGNC Symbol;Acc:HGNC:17637]
PFKM	phosphofructokinase, muscle [Source:HGNC Symbol;Acc:HGNC:8877]
POLA2	polymerase (DNA) alpha 2, accessory subunit [Source:HGNC Symbol;Acc:HGNC:30073]
PPP1CB	protein phosphatase 1 catalytic subunit beta [Source:HGNC Symbol;Acc:HGNC:9282]
PRKCQ	protein kinase C theta [Source:HGNC Symbol;Acc:HGNC:9410]
RABGAP1L	RAB GTPase activating protein 1-like [Source:HGNC Symbol;Acc:HGNC:24663]
SPRY1	sprouty RTK signaling antagonist 1 [Source:HGNC Symbol;Acc:HGNC:11269]
SUMO1	small ubiquitin-like modifier 1 [Source:HGNC Symbol;Acc:HGNC:12502]
TIMELESS	timeless circadian clock [Source:HGNC Symbol;Acc:HGNC:11813]
TNIP1	TNFAIP3 interacting protein 1 [Source:HGNC Symbol;Acc:HGNC:16903]
TP53I3	tumor protein p53 inducible protein 3 [Source:HGNC Symbol;Acc:HGNC:19373]
YWHAH	tyrosine 3-monooxygenase/tryptophan 5-monooxygenase activation protein eta [Source:HGNC Symbol;Acc:HGNC:12853]
ADGRE1	adhesion G protein-coupled receptor E1 [Source:HGNC Symbol;Acc:HGNC:3336]
ALDH7A1	aldehyde dehydrogenase 7 family member A1 [Source:HGNC Symbol;Acc:HGNC:877]
HNRNPH1	heterogeneous nuclear ribonucleoprotein H1 (H) [Source:HGNC Symbol;Acc:HGNC:5041]
KMT2A	lysine methyltransferase 2A [Source:HGNC Symbol;Acc:HGNC:7132]
KRT10	keratin 10 [Source:HGNC Symbol;Acc:HGNC:6413]
LY96	lymphocyte antigen 96 [Source:HGNC Symbol;Acc:HGNC:17156]
MT1E	metallothionein 1E [Source:HGNC Symbol;Acc:HGNC:7397]

PROC	protein C, inactivator of coagulation factors Va and VIIIa [Source:HGNC Symbol;Acc:HGNC:9451]
RACK1	receptor for activated C kinase 1 [Source:HGNC Symbol;Acc:HGNC:4399]
RRAD	RRAD, Ras related glycolysis inhibitor and calcium channel regulator [Source:HGNC Symbol;Acc:HGNC:10446]
TCP1	t-complex 1 [Source:HGNC Symbol;Acc:HGNC:11655]
TNFSF9	tumor necrosis factor superfamily member 9 [Source:HGNC Symbol;Acc:HGNC:11939]
CCDC80	coiled-coil domain containing 80 [Source:HGNC Symbol;Acc:HGNC:30649]
CCNL1	cyclin L1 [Source:HGNC Symbol;Acc:HGNC:20569]
CDC7	cell division cycle 7 [Source:HGNC Symbol;Acc:HGNC:1745]
CDK5	cyclin dependent kinase 5 [Source:HGNC Symbol;Acc:HGNC:1774]
CENPU	centromere protein U [Source:HGNC Symbol;Acc:HGNC:21348]
CRIP2	cysteine rich protein 2 [Source:HGNC Symbol;Acc:HGNC:2361]
CRYZ	crystallin zeta [Source:HGNC Symbol;Acc:HGNC:2419]
DBF4	DBF4 zinc finger [Source:HGNC Symbol;Acc:HGNC:17364]
DHX9	DEAH-box helicase 9 [Source:HGNC Symbol;Acc:HGNC:2750]
HNRNPAB	heterogeneous nuclear ribonucleoprotein A/B [Source:HGNC Symbol;Acc:HGNC:5034]
IDE	insulin degrading enzyme [Source:HGNC Symbol;Acc:HGNC:5381]
IMPDH2	IMP (inosine 5'-monophosphate) dehydrogenase 2 [Source:HGNC Symbol;Acc:HGNC:6053]
IQGAP2	IQ motif containing GTPase activating protein 2 [Source:HGNC Symbol;Acc:HGNC:6111]
ITIH3	inter-alpha-trypsin inhibitor heavy chain 3 [Source:HGNC Symbol;Acc:HGNC:6168]
KLF11	Kruppel like factor 11 [Source:HGNC Symbol;Acc:HGNC:11811]
LIN7A	lin-7 homolog A, crumbs cell polarity complex component [Source:HGNC Symbol;Acc:HGNC:17787]
LPIN2	lipin 2 [Source:HGNC Symbol;Acc:HGNC:14450]
METRNL	meteorin, glial cell differentiation regulator-like [Source:HGNC Symbol;Acc:HGNC:27584]
MMD	monocyte to macrophage differentiation associated [Source:HGNC Symbol;Acc:HGNC:7153]
NAT8	N-acetyltransferase 8 (putative) [Source:HGNC Symbol;Acc:HGNC:18069]
NID2	nidogen 2 [Source:HGNC Symbol;Acc:HGNC:13389]
PAPPA	pappalysin 1 [Source:HGNC Symbol;Acc:HGNC:8602]
PRKAR2B	protein kinase cAMP-dependent type II regulatory subunit beta [Source:HGNC Symbol;Acc:HGNC:9392]
SPC24	SPC24, NDC80 kinetochore complex component [Source:HGNC Symbol;Acc:HGNC:26913]
TNNC1	troponin C1, slow skeletal and cardiac type [Source:HGNC Symbol;Acc:HGNC:11943]
TRIO	trio Rho guanine nucleotide exchange factor [Source:HGNC Symbol;Acc:HGNC:12303]
YWHAQ	tyrosine 3-monooxygenase/tryptophan 5-monooxygenase activation protein theta [Source:HGNC Symbol;Acc:HGNC:12854]
MYH9	myosin, heavy chain 9, non-muscle [Source:HGNC Symbol;Acc:HGNC:7579]
BLM	Bloom syndrome RecQ like helicase [Source:HGNC Symbol;Acc:HGNC:1058]

CAMK2N1	calcium/calmodulin dependent protein kinase II inhibitor 1 [Source:HGNC Symbol;Acc:HGNC:24190]
CAPRIN1	cell cycle associated protein 1 [Source:HGNC Symbol;Acc:HGNC:6743]
CBX5	chromobox 5 [Source:HGNC Symbol;Acc:HGNC:1555]
CPEB2	cytoplasmic polyadenylation element binding protein 2 [Source:HGNC Symbol;Acc:HGNC:21745]
CRIP1	cysteine rich protein 1 [Source:HGNC Symbol;Acc:HGNC:2360]
DDR2	discoidin domain receptor tyrosine kinase 2 [Source:HGNC Symbol;Acc:HGNC:2731]
EPB41L3	erythrocyte membrane protein band 4.1 like 3 [Source:HGNC Symbol;Acc:HGNC:3380]
FIS1	fission, mitochondrial 1 [Source:HGNC Symbol;Acc:HGNC:21689]
GLS2	glutaminase 2 [Source:HGNC Symbol;Acc:HGNC:29570]
HNRNPA3	heterogeneous nuclear ribonucleoprotein A3 [Source:HGNC Symbol;Acc:HGNC:24941]
HOPX	HOP homeobox [Source:HGNC Symbol;Acc:HGNC:24961]
IDO1	indoleamine 2,3-dioxygenase 1 [Source:HGNC Symbol;Acc:HGNC:6059]
KIF18A	kinesin family member 18A [Source:HGNC Symbol;Acc:HGNC:29441]
KPNB1	karyopherin subunit beta 1 [Source:HGNC Symbol;Acc:HGNC:6400]
LXN	latexin [Source:HGNC Symbol;Acc:HGNC:13347]
WHSC1	Wolf-Hirschhorn syndrome candidate 1 [Source:HGNC Symbol;Acc:HGNC:12766]
OLFM1	olfactomedin 1 [Source:HGNC Symbol;Acc:HGNC:17187]
PHYH	phytanoyl-CoA 2-hydroxylase [Source:HGNC Symbol;Acc:HGNC:8940]
SERPINB5	serpin family B member 5 [Source:HGNC Symbol;Acc:HGNC:8949]
SIAH2	siah E3 ubiquitin protein ligase 2 [Source:HGNC Symbol;Acc:HGNC:10858]
TBL1XR1	transducin (beta)-like 1 X-linked receptor 1 [Source:HGNC Symbol;Acc:HGNC:29529]
TFF3	trefoil factor 3 [Source:HGNC Symbol;Acc:HGNC:11757]
TP73	tumor protein p73 [Source:HGNC Symbol;Acc:HGNC:12003]
TRAP1	TNF receptor associated protein 1 [Source:HGNC Symbol;Acc:HGNC:16264]
TSHR	thyroid stimulating hormone receptor [Source:HGNC Symbol;Acc:HGNC:12373]
ARHGDIB	Rho GDP dissociation inhibitor beta [Source:HGNC Symbol;Acc:HGNC:679]
BCAT1	branched chain amino acid transaminase 1 [Source:HGNC Symbol;Acc:HGNC:976]
IL13RA1	interleukin 13 receptor subunit alpha 1 [Source:HGNC Symbol;Acc:HGNC:5974]
MXD1	MAX dimerization protein 1 [Source:HGNC Symbol;Acc:HGNC:6761]
SPINK1	serine peptidase inhibitor, Kazal type 1 [Source:HGNC Symbol;Acc:HGNC:11244]
ZFAND2A	zinc finger AN1-type containing 2A [Source:HGNC Symbol;Acc:HGNC:28073]
ABCC9	ATP binding cassette subfamily C member 9 [Source:HGNC Symbol;Acc:HGNC:60]
ALDH18A1	aldehyde dehydrogenase 18 family member A1 [Source:HGNC Symbol;Acc:HGNC:9722]
CACNA2D1	calcium voltage-gated channel auxiliary subunit alpha2delta 1 [Source:HGNC Symbol;Acc:HGNC:1399]
CARHSP1	calcium regulated heat stable protein 1 [Source:HGNC Symbol;Acc:HGNC:17150]
CBL	Cbl proto-oncogene [Source:HGNC Symbol;Acc:HGNC:1541]
CDK5R1	cyclin dependent kinase 5 regulatory subunit 1 [Source:HGNC Symbol;Acc:HGNC:1775]
DACT1	dishevelled binding antagonist of beta catenin 1 [Source:HGNC Symbol;Acc:HGNC:17748]

DFFA	DNA fragmentation factor subunit alpha [Source:HGNC Symbol;Acc:HGNC:2772]
DHRS7	dehydrogenase/reductase 7 [Source:HGNC Symbol;Acc:HGNC:21524]
ENPEP	glutamyl aminopeptidase [Source:HGNC Symbol;Acc:HGNC:3355]
EPOR	erythropoietin receptor [Source:HGNC Symbol;Acc:HGNC:3416]
ETV4	ETS variant 4 [Source:HGNC Symbol;Acc:HGNC:3493]
FKBP11	FK506 binding protein 11 [Source:HGNC Symbol;Acc:HGNC:18624]
HNRNPDL	heterogeneous nuclear ribonucleoprotein D like [Source:HGNC Symbol;Acc:HGNC:5037]
ITGAL	integrin subunit alpha L [Source:HGNC Symbol;Acc:HGNC:6148]
IVNS1ABP	influenza virus NS1A binding protein [Source:HGNC Symbol;Acc:HGNC:16951]
MKNK2	MAP kinase interacting serine/threonine kinase 2 [Source:HGNC Symbol;Acc:HGNC:7111]
NT5DC2	5'-nucleotidase domain containing 2 [Source:HGNC Symbol;Acc:HGNC:25717]
ORC1	origin recognition complex subunit 1 [Source:HGNC Symbol;Acc:HGNC:8487]
PSMD12	proteasome 26S subunit, non-ATPase 12 [Source:HGNC Symbol;Acc:HGNC:9557]
RAD23B	RAD23 homolog B, nucleotide excision repair protein [Source:HGNC Symbol;Acc:HGNC:9813]
SLC15A1	solute carrier family 15 member 1 [Source:HGNC Symbol;Acc:HGNC:10920]
STAT4	signal transducer and activator of transcription 4 [Source:HGNC Symbol;Acc:HGNC:11365]
STOM	stomatin [Source:HGNC Symbol;Acc:HGNC:3383]
TGFBR3	transforming growth factor beta receptor 3 [Source:HGNC Symbol;Acc:HGNC:11774]
TYROBP	TYRO protein tyrosine kinase binding protein [Source:HGNC Symbol;Acc:HGNC:12449]
UQCRC2	ubiquinol-cytochrome c reductase core protein II [Source:HGNC Symbol;Acc:HGNC:12586]
YWHAG	tyrosine 3-monooxygenase/tryptophan 5-monooxygenase activation protein gamma [Source:HGNC Symbol;Acc:HGNC:12852]
BLVRB	biliverdin reductase B [Source:HGNC Symbol;Acc:HGNC:1063]
DCXR	dicarbonyl/L-xylulose reductase [Source:HGNC Symbol;Acc:HGNC:18985]
HSD17B3	hydroxysteroid 17-beta dehydrogenase 3 [Source:HGNC Symbol;Acc:HGNC:5212]
ACSM3	acyl-CoA synthetase medium-chain family member 3 [Source:HGNC Symbol;Acc:HGNC:10522]
AKAP9	A-kinase anchoring protein 9 [Source:HGNC Symbol;Acc:HGNC:379]
ARPC1B	actin related protein 2/3 complex subunit 1B [Source:HGNC Symbol;Acc:HGNC:704]
BCL10	B-cell CLL/lymphoma 10 [Source:HGNC Symbol;Acc:HGNC:989]
BMPR2	bone morphogenetic protein receptor type 2 [Source:HGNC Symbol;Acc:HGNC:1078]
CAD	carbamoyl-phosphate synthetase 2, aspartate transcarbamylase, and dihydroorotase [Source:HGNC Symbol;Acc:HGNC:1424]
CCKBR	cholecystokinin B receptor [Source:HGNC Symbol;Acc:HGNC:1571]
CORO1A	coronin 1A [Source:HGNC Symbol;Acc:HGNC:2252]
DCK	deoxycytidine kinase [Source:HGNC Symbol;Acc:HGNC:2704]
DEK	DEK proto-oncogene [Source:HGNC Symbol;Acc:HGNC:2768]
DYNLL1	dynein light chain LC8-type 1 [Source:HGNC Symbol;Acc:HGNC:15476]

E2F3	E2F transcription factor 3 [Source:HGNC Symbol;Acc:HGNC:3115]
EIF4A2	eukaryotic translation initiation factor 4A2 [Source:HGNC Symbol;Acc:HGNC:3284]
FCER1G	Fc fragment of IgE receptor Ig [Source:HGNC Symbol;Acc:HGNC:3611]
GNB1	G protein subunit beta 1 [Source:HGNC Symbol;Acc:HGNC:4396]
HNF1A	HNF1 homeobox A [Source:HGNC Symbol;Acc:HGNC:11621]
INCENP	inner centromere protein [Source:HGNC Symbol;Acc:HGNC:6058]
ITGA1	integrin subunit alpha 1 [Source:HGNC Symbol;Acc:HGNC:6134]
LIMA1	LIM domain and actin binding 1 [Source:HGNC Symbol;Acc:HGNC:24636]
NBN	nibrin [Source:HGNC Symbol;Acc:HGNC:7652]
NPAS2	neuronal PAS domain protein 2 [Source:HGNC Symbol;Acc:HGNC:7895]
OXCT1	3-oxoacid CoA-transferase 1 [Source:HGNC Symbol;Acc:HGNC:8527]
PCSK5	proprotein convertase subtilisin/kexin type 5 [Source:HGNC Symbol;Acc:HGNC:8747]
PDE2A	phosphodiesterase 2A [Source:HGNC Symbol;Acc:HGNC:8777]
PLEC	plectin [Source:HGNC Symbol;Acc:HGNC:9069]
PNRC1	proline rich nuclear receptor coactivator 1 [Source:HGNC Symbol;Acc:HGNC:17278]
PPP1R10	protein phosphatase 1 regulatory subunit 10 [Source:HGNC Symbol;Acc:HGNC:9284]
RPA1	replication protein A1 [Source:HGNC Symbol;Acc:HGNC:10289]
ST3GAL1	ST3 beta-galactoside alpha-2,3-sialyltransferase 1 [Source:HGNC Symbol;Acc:HGNC:10862]
STK17B	serine/threonine kinase 17b [Source:HGNC Symbol;Acc:HGNC:11396]
TFDP1	transcription factor Dp-1 [Source:HGNC Symbol;Acc:HGNC:11749]
UCP3	uncoupling protein 3 [Source:HGNC Symbol;Acc:HGNC:12519]
VAV3	vav guanine nucleotide exchange factor 3 [Source:HGNC Symbol;Acc:HGNC:12659]
ALCAM	activated leukocyte cell adhesion molecule [Source:HGNC Symbol;Acc:HGNC:400]
DBI	diazepam binding inhibitor, acyl-CoA binding protein [Source:HGNC Symbol;Acc:HGNC:2690]
ETS2	ETS proto-oncogene 2, transcription factor [Source:HGNC Symbol;Acc:HGNC:3489]
GK	glycerol kinase [Source:HGNC Symbol;Acc:HGNC:4289]
INSIG2	insulin induced gene 2 [Source:HGNC Symbol;Acc:HGNC:20452]
AHSG	alpha 2-HS glycoprotein [Source:HGNC Symbol;Acc:HGNC:349]
APLN	apelin [Source:HGNC Symbol;Acc:HGNC:16665]
ASPH	aspartate beta-hydroxylase [Source:HGNC Symbol;Acc:HGNC:757]
CASP10	caspase 10 [Source:HGNC Symbol;Acc:HGNC:1500]
CCT2	chaperonin containing TCP1 subunit 2 [Source:HGNC Symbol;Acc:HGNC:1615]
CGB3	chorionic gonadotropin beta subunit 3 [Source:HGNC Symbol;Acc:HGNC:1886]
DEPTOR	DEP domain containing MTOR-interacting protein [Source:HGNC Symbol;Acc:HGNC:22953]
DUSP8	dual specificity phosphatase 8 [Source:HGNC Symbol;Acc:HGNC:3074]
FMR1	fragile X mental retardation 1 [Source:HGNC Symbol;Acc:HGNC:3775]
GCHFR	GTP cyclohydrolase I feedback regulator [Source:HGNC Symbol;Acc:HGNC:4194]
GZMB	granzyme B [Source:HGNC Symbol;Acc:HGNC:4709]
HAO2	hydroxyacid oxidase 2 [Source:HGNC Symbol;Acc:HGNC:4810]

KCNN4	potassium calcium-activated channel subfamily N member 4 [Source:HGNC Symbol;Acc:HGNC:6293]
CASC5	cancer susceptibility candidate 5 [Source:HGNC Symbol;Acc:HGNC:24054]
LRRFIP1	leucine rich repeat (in FLII) interacting protein 1 [Source:HGNC Symbol;Acc:HGNC:6702]
ORC6	origin recognition complex subunit 6 [Source:HGNC Symbol;Acc:HGNC:17151]
PRMT1	protein arginine methyltransferase 1 [Source:HGNC Symbol;Acc:HGNC:5187]
RARRES1	retinoic acid receptor responder 1 [Source:HGNC Symbol;Acc:HGNC:9867]
RECK	reversion inducing cysteine rich protein with kazal motifs [Source:HGNC Symbol;Acc:HGNC:11345]
RPS19	ribosomal protein S19 [Source:HGNC Symbol;Acc:HGNC:10402]
SLC25A10	solute carrier family 25 member 10 [Source:HGNC Symbol;Acc:HGNC:10980]
SMC1A	structural maintenance of chromosomes 1A [Source:HGNC Symbol;Acc:HGNC:11111]
SRSF5	serine and arginine rich splicing factor 5 [Source:HGNC Symbol;Acc:HGNC:10787]
TOP2B	topoisomerase (DNA) II beta [Source:HGNC Symbol;Acc:HGNC:11990]
TRAF2	TNF receptor associated factor 2 [Source:HGNC Symbol;Acc:HGNC:12032]
WSB1	WD repeat and SOCS box containing 1 [Source:HGNC Symbol;Acc:HGNC:19221]
YWHAB	tyrosine 3-monooxygenase/tryptophan 5-monooxygenase activation protein beta [Source:HGNC Symbol;Acc:HGNC:12849]
BNIP3L	BCL2/adenovirus E1B 19kDa interacting protein 3-like [Source:HGNC Symbol;Acc:HGNC:1085]
DRD3	dopamine receptor D3 [Source:HGNC Symbol;Acc:HGNC:3024]
DUSP4	dual specificity phosphatase 4 [Source:HGNC Symbol;Acc:HGNC:3070]
FLNA	filamin A [Source:HGNC Symbol;Acc:HGNC:3754]
GNRH1	gonadotropin releasing hormone 1 [Source:HGNC Symbol;Acc:HGNC:4419]
PRKCZ	protein kinase C zeta [Source:HGNC Symbol;Acc:HGNC:9412]
SIK1	salt inducible kinase 1 [Source:HGNC Symbol;Acc:HGNC:11142]
TAT	tyrosine aminotransferase [Source:HGNC Symbol;Acc:HGNC:11573]
BARD1	BRCA1 associated RING domain 1 [Source:HGNC Symbol;Acc:HGNC:952]
CDCA7	cell division cycle associated 7 [Source:HGNC Symbol;Acc:HGNC:14628]
CRABP2	cellular retinoic acid binding protein 2 [Source:HGNC Symbol;Acc:HGNC:2339]
EPS8	epidermal growth factor receptor pathway substrate 8 [Source:HGNC Symbol;Acc:HGNC:3420]
FAH	fumarylacetoacetate hydrolase (fumarylacetoacetase) [Source:HGNC Symbol;Acc:HGNC:3579]
FDXR	ferredoxin reductase [Source:HGNC Symbol;Acc:HGNC:3642]
GINS2	GINS complex subunit 2 [Source:HGNC Symbol;Acc:HGNC:24575]
IL3	interleukin 3 [Source:HGNC Symbol;Acc:HGNC:6011]
ISYNA1	inositol-3-phosphate synthase 1 [Source:HGNC Symbol;Acc:HGNC:29821]
LAMC1	laminin subunit gamma 1 [Source:HGNC Symbol;Acc:HGNC:6492]
NNMT	nicotinamide N-methyltransferase [Source:HGNC Symbol;Acc:HGNC:7861]
NRAS	neuroblastoma RAS viral oncogene homolog [Source:HGNC Symbol;Acc:HGNC:7989]

PGRMC1	progesterone receptor membrane component 1 [Source:HGNC Symbol;Acc:HGNC:16090]
PSMB10	proteasome subunit beta 10 [Source:HGNC Symbol;Acc:HGNC:9538]
PSMB5	proteasome subunit beta 5 [Source:HGNC Symbol;Acc:HGNC:9542]
PTMA	prothymosin, alpha [Source:HGNC Symbol;Acc:HGNC:9623]
RASSF4	Ras association domain family member 4 [Source:HGNC Symbol;Acc:HGNC:20793]
RPL6	ribosomal protein L6 [Source:HGNC Symbol;Acc:HGNC:10362]
RPS6KA1	ribosomal protein S6 kinase A1 [Source:HGNC Symbol;Acc:HGNC:10430]
SHCBP1	SHC binding and spindle associated 1 [Source:HGNC Symbol;Acc:HGNC:29547]
SLC25A37	solute carrier family 25 member 37 [Source:HGNC Symbol;Acc:HGNC:29786]
SMC2	structural maintenance of chromosomes 2 [Source:HGNC Symbol;Acc:HGNC:14011]
SMO	smoothened, frizzled class receptor [Source:HGNC Symbol;Acc:HGNC:11119]
SYNE1	spectrin repeat containing nuclear envelope protein 1 [Source:HGNC Symbol;Acc:HGNC:17089]
UQCRC1	ubiquinol-cytochrome c reductase core protein I [Source:HGNC Symbol;Acc:HGNC:12585]
HSPA6	heat shock protein family A (Hsp70) member 6 [Source:HGNC Symbol;Acc:HGNC:5239]
INHBB	inhibin beta B subunit [Source:HGNC Symbol;Acc:HGNC:6067]
OPRK1	opioid receptor kappa 1 [Source:HGNC Symbol;Acc:HGNC:8154]
PTHLH	parathyroid hormone-like hormone [Source:HGNC Symbol;Acc:HGNC:9607]
RGS16	regulator of G-protein signaling 16 [Source:HGNC Symbol;Acc:HGNC:9997]
SLC25A20	solute carrier family 25 member 20 [Source:HGNC Symbol;Acc:HGNC:1421]
ADD3	adducin 3 [Source:HGNC Symbol;Acc:HGNC:245]
CEBPG	CCAAT/enhancer binding protein gamma [Source:HGNC Symbol;Acc:HGNC:1837]
DDX21	DEAD-box helicase 21 [Source:HGNC Symbol;Acc:HGNC:2744]
DLG1	discs large MAGUK scaffold protein 1 [Source:HGNC Symbol;Acc:HGNC:2900]
EIF1	eukaryotic translation initiation factor 1 [Source:HGNC Symbol;Acc:HGNC:3249]
EPHA4	EPH receptor A4 [Source:HGNC Symbol;Acc:HGNC:3388]
HDAC5	histone deacetylase 5 [Source:HGNC Symbol;Acc:HGNC:14068]
IRF9	interferon regulatory factor 9 [Source:HGNC Symbol;Acc:HGNC:6131]
MELK	maternal embryonic leucine zipper kinase [Source:HGNC Symbol;Acc:HGNC:16870]
MFN1	mitofusin 1 [Source:HGNC Symbol;Acc:HGNC:18262]
MTUS1	microtubule associated tumor suppressor 1 [Source:HGNC Symbol;Acc:HGNC:29789]
NASP	nuclear autoantigenic sperm protein [Source:HGNC Symbol;Acc:HGNC:7644]
POLD1	polymerase (DNA) delta 1, catalytic subunit [Source:HGNC Symbol;Acc:HGNC:9175]
SLCO2A1	solute carrier organic anion transporter family member 2A1 [Source:HGNC Symbol;Acc:HGNC:10955]
SRSF1	serine and arginine rich splicing factor 1 [Source:HGNC Symbol;Acc:HGNC:10780]
TDO2	tryptophan 2,3-dioxygenase [Source:HGNC Symbol;Acc:HGNC:11708]
TRAF1	TNF receptor associated factor 1 [Source:HGNC Symbol;Acc:HGNC:12031]
AKAP12	A-kinase anchoring protein 12 [Source:HGNC Symbol;Acc:HGNC:370]

SLC29A1	solute carrier family 29 member 1 (Augustine blood group) [Source:HGNC Symbol;Acc:HGNC:11003]
ATAD2	ATPase family, AAA domain containing 2 [Source:HGNC Symbol;Acc:HGNC:30123]
CTTN	cortactin [Source:HGNC Symbol;Acc:HGNC:3338]
DDB2	damage specific DNA binding protein 2 [Source:HGNC Symbol;Acc:HGNC:2718]
ESPL1	extra spindle pole bodies like 1, separase [Source:HGNC Symbol;Acc:HGNC:16856]
FOXP3	forkhead box P3 [Source:HGNC Symbol;Acc:HGNC:6106]
FSTL1	follistatin like 1 [Source:HGNC Symbol;Acc:HGNC:3972]
LYZ	lysozyme [Source:HGNC Symbol;Acc:HGNC:6740]
NEDD4	neural precursor cell expressed, developmentally down-regulated 4, E3 ubiquitin protein ligase [Source:HGNC Symbol;Acc:HGNC:7727]
PDZK1	PDZ domain containing 1 [Source:HGNC Symbol;Acc:HGNC:8821]
PIK3R3	phosphoinositide-3-kinase regulatory subunit 3 [Source:HGNC Symbol;Acc:HGNC:8981]
RBM3	RNA binding motif (RNP1, RRM) protein 3 [Source:HGNC Symbol;Acc:HGNC:9900]
RFC3	replication factor C subunit 3 [Source:HGNC Symbol;Acc:HGNC:9971]
TNS1	tensin 1 [Source:HGNC Symbol;Acc:HGNC:11973]
TP53INP1	tumor protein p53 inducible nuclear protein 1 [Source:HGNC Symbol;Acc:HGNC:18022]
ABAT	4-aminobutyrate aminotransferase [Source:HGNC Symbol;Acc:HGNC:23]
F5	coagulation factor V [Source:HGNC Symbol;Acc:HGNC:3542]
GAL	galanin and GMAP prepropeptide [Source:HGNC Symbol;Acc:HGNC:4114]
SCNN1A	sodium channel epithelial 1 alpha subunit [Source:HGNC Symbol;Acc:HGNC:10599]
SLC47A1	solute carrier family 47 member 1 [Source:HGNC Symbol;Acc:HGNC:25588]
TUBB2A	tubulin beta 2A class IIa [Source:HGNC Symbol;Acc:HGNC:12412]
UGDH	UDP-glucose 6-dehydrogenase [Source:HGNC Symbol;Acc:HGNC:12525]
ACADS	acyl-CoA dehydrogenase, C-2 to C-3 short chain [Source:HGNC Symbol;Acc:HGNC:90]
CMBL	carboxymethylenebutenolidase homolog (Pseudomonas) [Source:HGNC Symbol;Acc:HGNC:25090]
EIF4A1	eukaryotic translation initiation factor 4A1 [Source:HGNC Symbol;Acc:HGNC:3282]
EIF4G1	eukaryotic translation initiation factor 4 gamma 1 [Source:HGNC Symbol;Acc:HGNC:3296]
FOXA1	forkhead box A1 [Source:HGNC Symbol;Acc:HGNC:5021]
HNRNPD	heterogeneous nuclear ribonucleoprotein D [Source:HGNC Symbol;Acc:HGNC:5036]
KLF15	Kruppel like factor 15 [Source:HGNC Symbol;Acc:HGNC:14536]
MTHFD1L	methylenetetrahydrofolate dehydrogenase (NADP+ dependent) 1-like [Source:HGNC Symbol;Acc:HGNC:21055]
NOLC1	nucleolar and coiled-body phosphoprotein 1 [Source:HGNC Symbol;Acc:HGNC:15608]
NPHS1	NPHS1 nephrin [Source:HGNC Symbol;Acc:HGNC:7908]
POLA1	polymerase (DNA) alpha 1, catalytic subunit [Source:HGNC Symbol;Acc:HGNC:9173]
PPP1R3B	protein phosphatase 1 regulatory subunit 3B [Source:HGNC Symbol;Acc:HGNC:14942]
PRKACB	protein kinase cAMP-activated catalytic subunit beta [Source:HGNC Symbol;Acc:HGNC:9381]
RFC4	replication factor C subunit 4 [Source:HGNC Symbol;Acc:HGNC:9972]
RYR2	ryanodine receptor 2 [Source:HGNC Symbol;Acc:HGNC:10484]

SLC4A1	solute carrier family 4 member 1 (Diego blood group) [Source:HGNC Symbol;Acc:HGNC:11027]
SLC4A4	solute carrier family 4 member 4 [Source:HGNC Symbol;Acc:HGNC:11030]
SOX17	SRY-box 17 [Source:HGNC Symbol;Acc:HGNC:18122]
SPAG5	sperm associated antigen 5 [Source:HGNC Symbol;Acc:HGNC:13452]
SYK	spleen tyrosine kinase [Source:HGNC Symbol;Acc:HGNC:11491]
TXNL1	thioredoxin like 1 [Source:HGNC Symbol;Acc:HGNC:12436]
PRC1	protein regulator of cytokinesis 1 [Source:HGNC Symbol;Acc:HGNC:9341]
RND3	Rho family GTPase 3 [Source:HGNC Symbol;Acc:HGNC:671]
TRIB1	tribbles pseudokinase 1 [Source:HGNC Symbol;Acc:HGNC:16891]
ADAM17	ADAM metallopeptidase domain 17 [Source:HGNC Symbol;Acc:HGNC:195]
ASF1B	anti-silencing function 1B histone chaperone [Source:HGNC Symbol;Acc:HGNC:20996]
CD69	CD69 molecule [Source:HGNC Symbol;Acc:HGNC:1694]
CRISPLD2	cysteine rich secretory protein LCCL domain containing 2 [Source:HGNC Symbol;Acc:HGNC:25248]
CYB5R3	cytochrome b5 reductase 3 [Source:HGNC Symbol;Acc:HGNC:2873]
GLB1	galactosidase beta 1 [Source:HGNC Symbol;Acc:HGNC:4298]
GLI1	GLI family zinc finger 1 [Source:HGNC Symbol;Acc:HGNC:4317]
HNRNPK	heterogeneous nuclear ribonucleoprotein K [Source:HGNC Symbol;Acc:HGNC:5044]
MECP2	methyl-CpG binding protein 2 [Source:HGNC Symbol;Acc:HGNC:6990]
NCAPH	non-SMC condensin I complex subunit H [Source:HGNC Symbol;Acc:HGNC:1112]
PLK4	polo like kinase 4 [Source:HGNC Symbol;Acc:HGNC:11397]
PPP2CA	protein phosphatase 2 catalytic subunit alpha [Source:HGNC Symbol;Acc:HGNC:9299]
RGS5	regulator of G-protein signaling 5 [Source:HGNC Symbol;Acc:HGNC:10001]
SHMT1	serine hydroxymethyltransferase 1 [Source:HGNC Symbol;Acc:HGNC:10850]
SLC9A1	solute carrier family 9 member A1 [Source:HGNC Symbol;Acc:HGNC:11071]
TALDO1	transaldolase 1 [Source:HGNC Symbol;Acc:HGNC:11559]
TPO	thyroid peroxidase [Source:HGNC Symbol;Acc:HGNC:12015]
HSD17B1	hydroxysteroid 17-beta dehydrogenase 1 [Source:HGNC Symbol;Acc:HGNC:5210]
OSGIN1	oxidative stress induced growth inhibitor 1 [Source:HGNC Symbol;Acc:HGNC:30093]
S100A11	S100 calcium binding protein A11 [Source:HGNC Symbol;Acc:HGNC:10488]
SLC30A1	solute carrier family 30 member 1 [Source:HGNC Symbol;Acc:HGNC:11012]
SLC38A2	solute carrier family 38 member 2 [Source:HGNC Symbol;Acc:HGNC:13448]
BCAR3	breast cancer anti-estrogen resistance 3 [Source:HGNC Symbol;Acc:HGNC:973]
COTL1	coactosin like F-actin binding protein 1 [Source:HGNC Symbol;Acc:HGNC:18304]
ERO1A	endoplasmic reticulum oxidoreductase alpha [Source:HGNC Symbol;Acc:HGNC:13280]
FOXA2	forkhead box A2 [Source:HGNC Symbol;Acc:HGNC:5022]
G6PC	glucose-6-phosphatase catalytic subunit [Source:HGNC Symbol;Acc:HGNC:4056]
GFRA1	GDNF family receptor alpha 1 [Source:HGNC Symbol;Acc:HGNC:4243]
GLO1	glyoxalase I [Source:HGNC Symbol;Acc:HGNC:4323]
IKBKG	inhibitor of kappa light polypeptide gene enhancer in B-cells, kinase gamma [Source:HGNC Symbol;Acc:HGNC:5961]

MYO1B	myosin IB [Source:HGNC Symbol;Acc:HGNC:7596]
OPA1	OPA1, mitochondrial dynamin like GTPase [Source:HGNC Symbol;Acc:HGNC:8140]
PMEPA1	prostate transmembrane protein, androgen induced 1 [Source:HGNC Symbol;Acc:HGNC:14107]
PPP1R12A	protein phosphatase 1 regulatory subunit 12A [Source:HGNC Symbol;Acc:HGNC:7618]
PSMA5	proteasome subunit alpha 5 [Source:HGNC Symbol;Acc:HGNC:9534]
RRM2B	ribonucleotide reductase regulatory TP53 inducible subunit M2B [Source:HGNC Symbol;Acc:HGNC:17296]
SMAD1	SMAD family member 1 [Source:HGNC Symbol;Acc:HGNC:6767]
SMARCA4	SWI/SNF related, matrix associated, actin dependent regulator of chromatin, subfamily a, member 4 [Source:HGNC Symbol;Acc:HGNC:11100]
TWIST1	twist family bHLH transcription factor 1 [Source:HGNC Symbol;Acc:HGNC:12428]
YWHAE	tyrosine 3-monooxygenase/tryptophan 5-monooxygenase activation protein epsilon [Source:HGNC Symbol;Acc:HGNC:12851]
CALM1	calmodulin 1 (phosphorylase kinase, delta) [Source:HGNC Symbol;Acc:HGNC:1442]
IFIT1	interferon induced protein with tetratricopeptide repeats 1 [Source:HGNC Symbol;Acc:HGNC:5407]
IL11	interleukin 11 [Source:HGNC Symbol;Acc:HGNC:5966]
CGA	glycoprotein hormones, alpha polypeptide [Source:HGNC Symbol;Acc:HGNC:1885]
DCLK1	doublecortin like kinase 1 [Source:HGNC Symbol;Acc:HGNC:2700]
IER2	immediate early response 2 [Source:HGNC Symbol;Acc:HGNC:28871]
ITPR3	inositol 1,4,5-trisphosphate receptor type 3 [Source:HGNC Symbol;Acc:HGNC:6182]
MYBL2	MYB proto-oncogene like 2 [Source:HGNC Symbol;Acc:HGNC:7548]
NR2F2	nuclear receptor subfamily 2 group F member 2 [Source:HGNC Symbol;Acc:HGNC:7976]
PF4	platelet factor 4 [Source:HGNC Symbol;Acc:HGNC:8861]
POLE2	polymerase (DNA) epsilon 2, accessory subunit [Source:HGNC Symbol;Acc:HGNC:9178]
PRIM1	primase (DNA) subunit 1 [Source:HGNC Symbol;Acc:HGNC:9369]
PRKAA2	protein kinase AMP-activated catalytic subunit alpha 2 [Source:HGNC Symbol;Acc:HGNC:9377]
SLC7A2	solute carrier family 7 member 2 [Source:HGNC Symbol;Acc:HGNC:11060]
TFAP2A	transcription factor AP-2 alpha [Source:HGNC Symbol;Acc:HGNC:11742]
TMPRSS2	transmembrane protease, serine 2 [Source:HGNC Symbol;Acc:HGNC:11876]
TRH	thyrotropin releasing hormone [Source:HGNC Symbol;Acc:HGNC:12298]
UBE2T	ubiquitin conjugating enzyme E2 T [Source:HGNC Symbol;Acc:HGNC:25009]
HRH1	histamine receptor H1 [Source:HGNC Symbol;Acc:HGNC:5182]
PER1	period circadian clock 1 [Source:HGNC Symbol;Acc:HGNC:8845]
STC2	stanniocalcin 2 [Source:HGNC Symbol;Acc:HGNC:11374]
ALOX5AP	arachidonate 5-lipoxygenase activating protein [Source:HGNC Symbol;Acc:HGNC:436]
BDH1	3-hydroxybutyrate dehydrogenase, type 1 [Source:HGNC Symbol;Acc:HGNC:1027]
CEACAM1	carcinoembryonic antigen related cell adhesion molecule 1 [Source:HGNC Symbol;Acc:HGNC:1814]
DUT	deoxyuridine triphosphatase [Source:HGNC Symbol;Acc:HGNC:3078]

ECE1	endothelin converting enzyme 1 [Source:HGNC Symbol;Acc:HGNC:3146]
EGR3	early growth response 3 [Source:HGNC Symbol;Acc:HGNC:3240]
EIF2AK2	eukaryotic translation initiation factor 2 alpha kinase 2 [Source:HGNC Symbol;Acc:HGNC:9437]
ENPP1	ectonucleotide pyrophosphatase/phosphodiesterase 1 [Source:HGNC Symbol;Acc:HGNC:3356]
FABP7	fatty acid binding protein 7 [Source:HGNC Symbol;Acc:HGNC:3562]
FANCD2	Fanconi anemia complementation group D2 [Source:HGNC Symbol;Acc:HGNC:3585]
LIPE	lipase E, hormone sensitive type [Source:HGNC Symbol;Acc:HGNC:6621]
NFIX	nuclear factor I X [Source:HGNC Symbol;Acc:HGNC:7788]
NOTCH2	notch 2 [Source:HGNC Symbol;Acc:HGNC:7882]
PC	pyruvate carboxylase [Source:HGNC Symbol;Acc:HGNC:8636]
PTPRF	protein tyrosine phosphatase, receptor type F [Source:HGNC Symbol;Acc:HGNC:9670]
RTN4	reticulon 4 [Source:HGNC Symbol;Acc:HGNC:14085]
SDC2	syndecan 2 [Source:HGNC Symbol;Acc:HGNC:10659]
TACC3	transforming acidic coiled-coil containing protein 3 [Source:HGNC Symbol;Acc:HGNC:11524]
TSC2	tuberous sclerosis 2 [Source:HGNC Symbol;Acc:HGNC:12363]
SERPINE2	serpin family E member 2 [Source:HGNC Symbol;Acc:HGNC:8951]
TUBB3	tubulin beta 3 class III [Source:HGNC Symbol;Acc:HGNC:20772]
ARRB2	arrestin beta 2 [Source:HGNC Symbol;Acc:HGNC:712]
CDC45	cell division cycle 45 [Source:HGNC Symbol;Acc:HGNC:1739]
CDT1	chromatin licensing and DNA replication factor 1 [Source:HGNC Symbol;Acc:HGNC:24576]
CKAP2	cytoskeleton associated protein 2 [Source:HGNC Symbol;Acc:HGNC:1990]
ESRRA	estrogen related receptor alpha [Source:HGNC Symbol;Acc:HGNC:3471]
GMNN	geminin, DNA replication inhibitor [Source:HGNC Symbol;Acc:HGNC:17493]
NCOA3	nuclear receptor coactivator 3 [Source:HGNC Symbol;Acc:HGNC:7670]
PSMB8	proteasome subunit beta 8 [Source:HGNC Symbol;Acc:HGNC:9545]
SDF2L1	stromal cell derived factor 2 like 1 [Source:HGNC Symbol;Acc:HGNC:10676]
SMAD4	SMAD family member 4 [Source:HGNC Symbol;Acc:HGNC:6770]
TUBA1C	tubulin alpha 1c [Source:HGNC Symbol;Acc:HGNC:20768]
FOSL2	FOS like 2, AP-1 transcription factor subunit [Source:HGNC Symbol;Acc:HGNC:3798]
SHBG	sex hormone binding globulin [Source:HGNC Symbol;Acc:HGNC:10839]
ALDH1B1	aldehyde dehydrogenase 1 family member B1 [Source:HGNC Symbol;Acc:HGNC:407]
ASAH1	N-acylsphingosine amidohydrolase (acid ceramidase) 1 [Source:HGNC Symbol;Acc:HGNC:735]
MCM10	minichromosome maintenance 10 replication initiation factor [Source:HGNC Symbol;Acc:HGNC:18043]
NCOR2	nuclear receptor corepressor 2 [Source:HGNC Symbol;Acc:HGNC:7673]
PRKG1	protein kinase, cGMP-dependent, type I [Source:HGNC Symbol;Acc:HGNC:9414]
RAD51AP1	RAD51 associated protein 1 [Source:HGNC Symbol;Acc:HGNC:16956]
TFPI2	tissue factor pathway inhibitor 2 [Source:HGNC Symbol;Acc:HGNC:11761]

TRIP13	thyroid hormone receptor interactor 13 [Source:HGNC Symbol;Acc:HGNC:12307]
ZBTB16	zinc finger and BTB domain containing 16 [Source:HGNC Symbol;Acc:HGNC:12930]
GLRX	glutaredoxin [Source:HGNC Symbol;Acc:HGNC:4330]
IDH1	isocitrate dehydrogenase (NADP(+)) 1, cytosolic [Source:HGNC Symbol;Acc:HGNC:5382]
BMF	Bcl2 modifying factor [Source:HGNC Symbol;Acc:HGNC:24132]
CBR3	carbonyl reductase 3 [Source:HGNC Symbol;Acc:HGNC:1549]
CIDEA	cell death-inducing DFFA-like effector a [Source:HGNC Symbol;Acc:HGNC:1976]
EIF5	eukaryotic translation initiation factor 5 [Source:HGNC Symbol;Acc:HGNC:3299]
ELK1	ELK1, ETS transcription factor [Source:HGNC Symbol;Acc:HGNC:3321]
HNRNPA2B1	heterogeneous nuclear ribonucleoprotein A2/B1 [Source:HGNC Symbol;Acc:HGNC:5033]
ID4	inhibitor of DNA binding 4, HLH protein [Source:HGNC Symbol;Acc:HGNC:5363]
IDH3A	isocitrate dehydrogenase 3 (NAD(+)) alpha [Source:HGNC Symbol;Acc:HGNC:5384]
MAP2K3	mitogen-activated protein kinase kinase 3 [Source:HGNC Symbol;Acc:HGNC:6843]
NQO2	NAD(P)H quinone dehydrogenase 2 [Source:HGNC Symbol;Acc:HGNC:7856]
NTS	neurotensin [Source:HGNC Symbol;Acc:HGNC:8038]
PDE4D	phosphodiesterase 4D [Source:HGNC Symbol;Acc:HGNC:8783]
PGAM1	phosphoglycerate mutase 1 [Source:HGNC Symbol;Acc:HGNC:8888]
PTPN11	protein tyrosine phosphatase, non-receptor type 11 [Source:HGNC Symbol;Acc:HGNC:9644]
SERPINA7	serpin family A member 7 [Source:HGNC Symbol;Acc:HGNC:11583]
TAGLN2	transgelin 2 [Source:HGNC Symbol;Acc:HGNC:11554]
TUBB	tubulin beta class I [Source:HGNC Symbol;Acc:HGNC:20778]
USP2	ubiquitin specific peptidase 2 [Source:HGNC Symbol;Acc:HGNC:12618]
CPT2	carnitine palmitoyltransferase 2 [Source:HGNC Symbol;Acc:HGNC:2330]
PRLR	prolactin receptor [Source:HGNC Symbol;Acc:HGNC:9446]
ABL1	ABL proto-oncogene 1, non-receptor tyrosine kinase [Source:HGNC Symbol;Acc:HGNC:76]
ATR	ATR serine/threonine kinase [Source:HGNC Symbol;Acc:HGNC:882]
AXIN2	axin 2 [Source:HGNC Symbol;Acc:HGNC:904]
CANX	calnexin [Source:HGNC Symbol;Acc:HGNC:1473]
IFITM1	interferon induced transmembrane protein 1 [Source:HGNC Symbol;Acc:HGNC:5412]
MAT2A	methionine adenosyltransferase 2A [Source:HGNC Symbol;Acc:HGNC:6904]
MYCN	v-myc avian myelocytomatosis viral oncogene neuroblastoma derived homolog [Source:HGNC Symbol;Acc:HGNC:7559]
NEDD4L	neural precursor cell expressed, developmentally down-regulated 4-like, E3 ubiquitin protein ligase [Source:HGNC Symbol;Acc:HGNC:7728]
PDYN	prodynorphin [Source:HGNC Symbol;Acc:HGNC:8820]
RAD50	RAD50 double strand break repair protein [Source:HGNC Symbol;Acc:HGNC:9816]
STAT6	signal transducer and activator of transcription 6 [Source:HGNC Symbol;Acc:HGNC:11368]
TUBB4B	tubulin beta 4B class IVb [Source:HGNC Symbol;Acc:HGNC:20771]

TUBB6	tubulin beta 6 class V [Source:HGNC Symbol;Acc:HGNC:20776]
ZWINT	ZW10 interacting kinetochore protein [Source:HGNC Symbol;Acc:HGNC:13195]
CHAC1	ChaC glutathione specific gamma-glutamylcyclotransferase 1 [Source:HGNC Symbol;Acc:HGNC:28680]
PENK	proenkephalin [Source:HGNC Symbol;Acc:HGNC:8831]
CA3	carbonic anhydrase 3 [Source:HGNC Symbol;Acc:HGNC:1374]
CCK	cholecystokinin [Source:HGNC Symbol;Acc:HGNC:1569]
CS	citrate synthase [Source:HGNC Symbol;Acc:HGNC:2422]
GATM	glycine amidinotransferase [Source:HGNC Symbol;Acc:HGNC:4175]
LIFR	leukemia inhibitory factor receptor alpha [Source:HGNC Symbol;Acc:HGNC:6597]
MYH6	myosin, heavy chain 6, cardiac muscle, alpha [Source:HGNC Symbol;Acc:HGNC:7576]
SLC1A5	solute carrier family 1 member 5 [Source:HGNC Symbol;Acc:HGNC:10943]
SLC31A1	solute carrier family 31 member 1 [Source:HGNC Symbol;Acc:HGNC:11016]
SLC8A1	solute carrier family 8 member A1 [Source:HGNC Symbol;Acc:HGNC:11068]
TAP1	transporter 1, ATP-binding cassette, sub-family B (MDR/TAP) [Source:HGNC Symbol;Acc:HGNC:43]
TMPO	thymopoietin [Source:HGNC Symbol;Acc:HGNC:11875]
TOP1	topoisomerase (DNA) I [Source:HGNC Symbol;Acc:HGNC:11986]
TTK	TTK protein kinase [Source:HGNC Symbol;Acc:HGNC:12401]
TUBA4A	tubulin alpha 4a [Source:HGNC Symbol;Acc:HGNC:12407]
UGT1A10	UDP glucuronosyltransferase family 1 member A10 [Source:HGNC Symbol;Acc:HGNC:12531]
UGT1A3	UDP glucuronosyltransferase family 1 member A3 [Source:HGNC Symbol;Acc:HGNC:12535]
UGT1A8	UDP glucuronosyltransferase family 1 member A8 [Source:HGNC Symbol;Acc:HGNC:12540]
SLC6A4	solute carrier family 6 member 4 [Source:HGNC Symbol;Acc:HGNC:11050]
ACOT2	acyl-CoA thioesterase 2 [Source:HGNC Symbol;Acc:HGNC:18431]
ACSL4	acyl-CoA synthetase long-chain family member 4 [Source:HGNC Symbol;Acc:HGNC:3571]
ACTN1	actinin alpha 1 [Source:HGNC Symbol;Acc:HGNC:163]
ARC	activity-regulated cytoskeleton-associated protein [Source:HGNC Symbol;Acc:HGNC:648]
ATRX	ATRX, chromatin remodeler [Source:HGNC Symbol;Acc:HGNC:886]
ECT2	epithelial cell transforming 2 [Source:HGNC Symbol;Acc:HGNC:3155]
ENO3	enolase 3 [Source:HGNC Symbol;Acc:HGNC:3354]
HK1	hexokinase 1 [Source:HGNC Symbol;Acc:HGNC:4922]
NME1	NME/NM23 nucleoside diphosphate kinase 1 [Source:HGNC Symbol;Acc:HGNC:7849]
PLOD2	procollagen-lysine,2-oxoglutarate 5-dioxygenase 2 [Source:HGNC Symbol;Acc:HGNC:9082]
PML	promyelocytic leukemia [Source:HGNC Symbol;Acc:HGNC:9113]
RCAN1	regulator of calcineurin 1 [Source:HGNC Symbol;Acc:HGNC:3040]
SIGMAR1	sigma non-opioid intracellular receptor 1 [Source:HGNC Symbol;Acc:HGNC:8157]

SLC1A4	solute carrier family 1 member 4 [Source:HGNC Symbol;Acc:HGNC:10942]
SOCS1	suppressor of cytokine signaling 1 [Source:HGNC Symbol;Acc:HGNC:19383]
BDKRB2	bradykinin receptor B2 [Source:HGNC Symbol;Acc:HGNC:1030]
HSD17B2	hydroxysteroid 17-beta dehydrogenase 2 [Source:HGNC Symbol;Acc:HGNC:5211]
PLCG1	phospholipase C gamma 1 [Source:HGNC Symbol;Acc:HGNC:9065]
S100B	S100 calcium binding protein B [Source:HGNC Symbol;Acc:HGNC:10500]
STAT5A	signal transducer and activator of transcription 5A [Source:HGNC Symbol;Acc:HGNC:11366]
VNN1	vanin 1 [Source:HGNC Symbol;Acc:HGNC:12705]
CKB	creatine kinase B [Source:HGNC Symbol;Acc:HGNC:1991]
AHNAK	AHNAK nucleoprotein [Source:HGNC Symbol;Acc:HGNC:347]
GRIA1	glutamate ionotropic receptor AMPA type subunit 1 [Source:HGNC Symbol;Acc:HGNC:4571]
GUSB	glucuronidase beta [Source:HGNC Symbol;Acc:HGNC:4696]
HMMR	hyaluronan mediated motility receptor [Source:HGNC Symbol;Acc:HGNC:5012]
MCM7	minichromosome maintenance complex component 7 [Source:HGNC Symbol;Acc:HGNC:6950]
NCF2	neutrophil cytosolic factor 2 [Source:HGNC Symbol;Acc:HGNC:7661]
NCOR1	nuclear receptor corepressor 1 [Source:HGNC Symbol;Acc:HGNC:7672]
PFKP	phosphofructokinase, platelet [Source:HGNC Symbol;Acc:HGNC:8878]
PRSS23	protease, serine 23 [Source:HGNC Symbol;Acc:HGNC:14370]
PYGL	phosphorylase, glycogen, liver [Source:HGNC Symbol;Acc:HGNC:9725]
RELN	reelin [Source:HGNC Symbol;Acc:HGNC:9957]
RGN	regucalcin [Source:HGNC Symbol;Acc:HGNC:9989]
SESN1	sestrin 1 [Source:HGNC Symbol;Acc:HGNC:21595]
SLC1A1	solute carrier family 1 member 1 [Source:HGNC Symbol;Acc:HGNC:10939]
SLC22A7	solute carrier family 22 member 7 [Source:HGNC Symbol;Acc:HGNC:10971]
SMAD7	SMAD family member 7 [Source:HGNC Symbol;Acc:HGNC:6773]
CRH	corticotropin releasing hormone [Source:HGNC Symbol;Acc:HGNC:2355]
DUSP5	dual specificity phosphatase 5 [Source:HGNC Symbol;Acc:HGNC:3071]
EGR2	early growth response 2 [Source:HGNC Symbol;Acc:HGNC:3239]
PRKCE	protein kinase C epsilon [Source:HGNC Symbol;Acc:HGNC:9401]
GJB2	gap junction protein beta 2 [Source:HGNC Symbol;Acc:HGNC:4284]
LIPG	lipase G, endothelial type [Source:HGNC Symbol;Acc:HGNC:6623]
MTHFD2	methylenetetrahydrofolate dehydrogenase (NADP+ dependent) 2, methenyltetrahydrofolate cyclohydrolase [Source:HGNC Symbol;Acc:HGNC:7434]
NAT1	N-acetyltransferase 1 (arylamine N-acetyltransferase) [Source:HGNC Symbol;Acc:HGNC:7645]
PROS1	protein S (alpha) [Source:HGNC Symbol;Acc:HGNC:9456]
RRM1	ribonucleotide reductase catalytic subunit M1 [Source:HGNC Symbol;Acc:HGNC:10451]
SFPQ	splicing factor proline and glutamine rich [Source:HGNC Symbol;Acc:HGNC:10774]
TNNT2	troponin T2, cardiac type [Source:HGNC Symbol;Acc:HGNC:11949]

WNT5A	Wnt family member 5A [Source:HGNC Symbol;Acc:HGNC:12784]
HSD11B2	hydroxysteroid 11-beta dehydrogenase 2 [Source:HGNC Symbol;Acc:HGNC:5209]
BHMT	betaine--homocysteine S-methyltransferase [Source:HGNC Symbol;Acc:HGNC:1047]
CHRM2	cholinergic receptor muscarinic 2 [Source:HGNC Symbol;Acc:HGNC:1951]
CHRM3	cholinergic receptor muscarinic 3 [Source:HGNC Symbol;Acc:HGNC:1952]
CKS2	CDC28 protein kinase regulatory subunit 2 [Source:HGNC Symbol;Acc:HGNC:2000]
EIF4E	eukaryotic translation initiation factor 4E [Source:HGNC Symbol;Acc:HGNC:3287]
FBP1	fructose-bisphosphatase 1 [Source:HGNC Symbol;Acc:HGNC:3606]
FOXM1	forkhead box M1 [Source:HGNC Symbol;Acc:HGNC:3818]
LDHB	lactate dehydrogenase B [Source:HGNC Symbol;Acc:HGNC:6541]
PDIA4	protein disulfide isomerase family A member 4 [Source:HGNC Symbol;Acc:HGNC:30167]
CES1	carboxylesterase 1 [Source:HGNC Symbol;Acc:HGNC:1863]
TNFRSF12A	tumor necrosis factor receptor superfamily member 12A [Source:HGNC Symbol;Acc:HGNC:18152]
BRCA2	BRCA2, DNA repair associated [Source:HGNC Symbol;Acc:HGNC:1101]
CHRM1	cholinergic receptor muscarinic 1 [Source:HGNC Symbol;Acc:HGNC:1950]
DAPK1	death associated protein kinase 1 [Source:HGNC Symbol;Acc:HGNC:2674]
ERCC1	ERCC excision repair 1, endonuclease non-catalytic subunit [Source:HGNC Symbol;Acc:HGNC:3433]
GRN	granulin [Source:HGNC Symbol;Acc:HGNC:4601]
MCM3	minichromosome maintenance complex component 3 [Source:HGNC Symbol;Acc:HGNC:6945]
NFKBIB	NFKB inhibitor beta [Source:HGNC Symbol;Acc:HGNC:7798]
PFKFB3	6-phosphofructo-2-kinase/fructose-2,6-biphosphatase 3 [Source:HGNC Symbol;Acc:HGNC:8874]
PPP1R3C	protein phosphatase 1 regulatory subunit 3C [Source:HGNC Symbol;Acc:HGNC:9293]
ALAD	aminolevulinate dehydratase [Source:HGNC Symbol;Acc:HGNC:395]
NR4A2	nuclear receptor subfamily 4 group A member 2 [Source:HGNC Symbol;Acc:HGNC:7981]
TPM1	tropomyosin 1 (alpha) [Source:HGNC Symbol;Acc:HGNC:12010]
ANXA4	annexin A4 [Source:HGNC Symbol;Acc:HGNC:542]
CDC25B	cell division cycle 25B [Source:HGNC Symbol;Acc:HGNC:1726]
CPT1B	carnitine palmitoyltransferase 1B [Source:HGNC Symbol;Acc:HGNC:2329]
CTSS	cathepsin S [Source:HGNC Symbol;Acc:HGNC:2545]
CYP8B1	cytochrome P450 family 8 subfamily B member 1 [Source:HGNC Symbol;Acc:HGNC:2653]
DIABLO	diablo IAP-binding mitochondrial protein [Source:HGNC Symbol;Acc:HGNC:21528]
FYN	FYN proto-oncogene, Src family tyrosine kinase [Source:HGNC Symbol;Acc:HGNC:4037]
NFKBIZ	NFKB inhibitor zeta [Source:HGNC Symbol;Acc:HGNC:29805]
NREP	neuronal regeneration related protein [Source:HGNC Symbol;Acc:HGNC:16834]
PTPN1	protein tyrosine phosphatase, non-receptor type 1 [Source:HGNC Symbol;Acc:HGNC:9642]

SC5D	sterol-C5-desaturase [Source:HGNC Symbol;Acc:HGNC:10547]
SLC20A1	solute carrier family 20 member 1 [Source:HGNC Symbol;Acc:HGNC:10946]
UGT2B7	UDP glucuronosyltransferase family 2 member B7 [Source:HGNC Symbol;Acc:HGNC:12554]
OPRM1	opioid receptor mu 1 [Source:HGNC Symbol;Acc:HGNC:8156]
CD80	CD80 molecule [Source:HGNC Symbol;Acc:HGNC:1700]
GCK	glucokinase [Source:HGNC Symbol;Acc:HGNC:4195]
HSPA2	heat shock protein family A (Hsp70) member 2 [Source:HGNC Symbol;Acc:HGNC:5235]
UHRF1	ubiquitin like with PHD and ring finger domains 1 [Source:HGNC Symbol;Acc:HGNC:12556]
CDC20	cell division cycle 20 [Source:HGNC Symbol;Acc:HGNC:1723]
KLF6	Kruppel like factor 6 [Source:HGNC Symbol;Acc:HGNC:2235]
CDC25A	cell division cycle 25A [Source:HGNC Symbol;Acc:HGNC:1725]
CDC6	cell division cycle 6 [Source:HGNC Symbol;Acc:HGNC:1744]
CYP51A1	cytochrome P450 family 51 subfamily A member 1 [Source:HGNC Symbol;Acc:HGNC:2649]
CYP7B1	cytochrome P450 family 7 subfamily B member 1 [Source:HGNC Symbol;Acc:HGNC:2652]
FADD	Fas associated via death domain [Source:HGNC Symbol;Acc:HGNC:3573]
FGFR3	fibroblast growth factor receptor 3 [Source:HGNC Symbol;Acc:HGNC:3690]
ITGB3	integrin subunit beta 3 [Source:HGNC Symbol;Acc:HGNC:6156]
RET	ret proto-oncogene [Source:HGNC Symbol;Acc:HGNC:9967]
TNFRSF1B	tumor necrosis factor receptor superfamily member 1B [Source:HGNC Symbol;Acc:HGNC:11917]
UCP1	uncoupling protein 1 [Source:HGNC Symbol;Acc:HGNC:12517]
CTH	cystathionine gamma-lyase [Source:HGNC Symbol;Acc:HGNC:2501]
DNM1L	dynamin 1-like [Source:HGNC Symbol;Acc:HGNC:2973]
ECH1	enoyl-CoA hydratase 1, peroxisomal [Source:HGNC Symbol;Acc:HGNC:3149]
HPGD	hydroxyprostaglandin dehydrogenase 15-(NAD) [Source:HGNC Symbol;Acc:HGNC:5154]
JAK1	Janus kinase 1 [Source:HGNC Symbol;Acc:HGNC:6190]
LDHA	lactate dehydrogenase A [Source:HGNC Symbol;Acc:HGNC:6535]
NPY	neuropeptide Y [Source:HGNC Symbol;Acc:HGNC:7955]
LMNB1	lamin B1 [Source:HGNC Symbol;Acc:HGNC:6637]
PDCD4	programmed cell death 4 (neoplastic transformation inhibitor) [Source:HGNC Symbol;Acc:HGNC:8763]
STAT5B	signal transducer and activator of transcription 5B [Source:HGNC Symbol;Acc:HGNC:11367]
SLC3A2	solute carrier family 3 member 2 [Source:HGNC Symbol;Acc:HGNC:11026]
ARG2	arginase 2 [Source:HGNC Symbol;Acc:HGNC:664]
DDC	dopa decarboxylase [Source:HGNC Symbol;Acc:HGNC:2719]
GLS	glutaminase [Source:HGNC Symbol;Acc:HGNC:4331]
GNAS	GNAS complex locus [Source:HGNC Symbol;Acc:HGNC:4392]

HDAC1	histone deacetylase 1 [Source:HGNC Symbol;Acc:HGNC:4852]
HMGB2	high mobility group box 2 [Source:HGNC Symbol;Acc:HGNC:5000]
MYLK	myosin light chain kinase [Source:HGNC Symbol;Acc:HGNC:7590]
NCOA1	nuclear receptor coactivator 1 [Source:HGNC Symbol;Acc:HGNC:7668]
NR1D1	nuclear receptor subfamily 1 group D member 1 [Source:HGNC Symbol;Acc:HGNC:7962]
PCK2	phosphoenolpyruvate carboxykinase 2, mitochondrial [Source:HGNC Symbol;Acc:HGNC:8725]
SNAI2	snail family transcriptional repressor 2 [Source:HGNC Symbol;Acc:HGNC:11094]
AOX1	aldehyde oxidase 1 [Source:HGNC Symbol;Acc:HGNC:553]
FMO1	flavin containing monooxygenase 1 [Source:HGNC Symbol;Acc:HGNC:3769]
GH1	growth hormone 1 [Source:HGNC Symbol;Acc:HGNC:4261]
HNF4A	hepatocyte nuclear factor 4 alpha [Source:HGNC Symbol;Acc:HGNC:5024]
KLF9	Kruppel like factor 9 [Source:HGNC Symbol;Acc:HGNC:1123]
CSF1	colony stimulating factor 1 [Source:HGNC Symbol;Acc:HGNC:2432]
CYB5A	cytochrome b5 type A [Source:HGNC Symbol;Acc:HGNC:2570]
NCOA2	nuclear receptor coactivator 2 [Source:HGNC Symbol;Acc:HGNC:7669]
PTGES	prostaglandin E synthase [Source:HGNC Symbol;Acc:HGNC:9599]
TUBA1A	tubulin alpha 1a [Source:HGNC Symbol;Acc:HGNC:20766]
CHRNA4	cholinergic receptor nicotinic alpha 4 subunit [Source:HGNC Symbol;Acc:HGNC:1958]
DHFR	dihydrofolate reductase [Source:HGNC Symbol;Acc:HGNC:2861]
ELOVL6	ELOVL fatty acid elongase 6 [Source:HGNC Symbol;Acc:HGNC:15829]
IRF1	interferon regulatory factor 1 [Source:HGNC Symbol;Acc:HGNC:6116]
S100A4	S100 calcium binding protein A4 [Source:HGNC Symbol;Acc:HGNC:10494]
SLC1A2	solute carrier family 1 member 2 [Source:HGNC Symbol;Acc:HGNC:10940]
SLC22A6	solute carrier family 22 member 6 [Source:HGNC Symbol;Acc:HGNC:10970]
F2R	coagulation factor II thrombin receptor [Source:HGNC Symbol;Acc:HGNC:3537]
GRIN2B	glutamate ionotropic receptor NMDA type subunit 2B [Source:HGNC Symbol;Acc:HGNC:4586]
PYCARD	PYD and CARD domain containing [Source:HGNC Symbol;Acc:HGNC:16608]
TSC22D1	TSC22 domain family member 1 [Source:HGNC Symbol;Acc:HGNC:16826]
SGK1	serum/glucocorticoid regulated kinase 1 [Source:HGNC Symbol;Acc:HGNC:10810]
FDPS	farnesyl diphosphate synthase [Source:HGNC Symbol;Acc:HGNC:3631]
GRIN1	glutamate ionotropic receptor NMDA type subunit 1 [Source:HGNC Symbol;Acc:HGNC:4584]
NAMPT	nicotinamide phosphoribosyltransferase [Source:HGNC Symbol;Acc:HGNC:30092]
THRSP	thyroid hormone responsive [Source:HGNC Symbol;Acc:HGNC:11800]
ACLY	ATP citrate lyase [Source:HGNC Symbol;Acc:HGNC:115]
AURKB	aurora kinase B [Source:HGNC Symbol;Acc:HGNC:11390]
CREBBP	CREB binding protein [Source:HGNC Symbol;Acc:HGNC:2348]
CYP2C8	cytochrome P450 family 2 subfamily C member 8 [Source:HGNC Symbol;Acc:HGNC:2622]

IGFBP5	insulin like growth factor binding protein 5 [Source:HGNC Symbol;Acc:HGNC:5474]
SLC7A5	solute carrier family 7 member 5 [Source:HGNC Symbol;Acc:HGNC:11063]
AKR1C1	aldo-keto reductase family 1 member C1 [Source:HGNC Symbol;Acc:HGNC:384]
UGT1A9	UDP glucuronosyltransferase family 1 member A9 [Source:HGNC Symbol;Acc:HGNC:12541]
BHLHE40	basic helix-loop-helix family member e40 [Source:HGNC Symbol;Acc:HGNC:1046]
DIO1	deiodinase, iodothyronine type I [Source:HGNC Symbol;Acc:HGNC:2883]
HES1	hes family bHLH transcription factor 1 [Source:HGNC Symbol;Acc:HGNC:5192]
SLC22A8	solute carrier family 22 member 8 [Source:HGNC Symbol;Acc:HGNC:10972]
TAGLN	transgelin [Source:HGNC Symbol;Acc:HGNC:11553]
SRXN1	sulfiredoxin 1 [Source:HGNC Symbol;Acc:HGNC:16132]
TRIB3	tribbles pseudokinase 3 [Source:HGNC Symbol;Acc:HGNC:16228]
FDFT1	farnesyl-diphosphate farnesyltransferase 1 [Source:HGNC Symbol;Acc:HGNC:3629]
TSC22D3	TSC22 domain family member 3 [Source:HGNC Symbol;Acc:HGNC:3051]
CCL20	C-C motif chemokine ligand 20 [Source:HGNC Symbol;Acc:HGNC:10619]
CCNG1	cyclin G1 [Source:HGNC Symbol;Acc:HGNC:1592]
CEBPD	CCAAT/enhancer binding protein delta [Source:HGNC Symbol;Acc:HGNC:1835]
FKBP5	FK506 binding protein 5 [Source:HGNC Symbol;Acc:HGNC:3721]
ID3	inhibitor of DNA binding 3, HLH protein [Source:HGNC Symbol;Acc:HGNC:5362]
CDC25C	cell division cycle 25C [Source:HGNC Symbol;Acc:HGNC:1727]
DIO2	deiodinase, iodothyronine, type II [Source:HGNC Symbol;Acc:HGNC:2884]
IGFBP1	insulin like growth factor binding protein 1 [Source:HGNC Symbol;Acc:HGNC:5469]
EPHX2	epoxide hydrolase 2 [Source:HGNC Symbol;Acc:HGNC:3402]
ID1	inhibitor of DNA binding 1, HLH protein [Source:HGNC Symbol;Acc:HGNC:5360]
NR0B2	nuclear receptor subfamily 0 group B member 2 [Source:HGNC Symbol;Acc:HGNC:7961]
APAF1	apoptotic peptidase activating factor 1 [Source:HGNC Symbol;Acc:HGNC:576]
CDK6	cyclin dependent kinase 6 [Source:HGNC Symbol;Acc:HGNC:1777]
JAK2	Janus kinase 2 [Source:HGNC Symbol;Acc:HGNC:6192]
NPPB	natriuretic peptide B [Source:HGNC Symbol;Acc:HGNC:7940]
BBC3	BCL2 binding component 3 [Source:HGNC Symbol;Acc:HGNC:17868]
BTG2	BTG family member 2 [Source:HGNC Symbol;Acc:HGNC:1131]
ARG1	arginase 1 [Source:HGNC Symbol;Acc:HGNC:663]
INSIG1	insulin induced gene 1 [Source:HGNC Symbol;Acc:HGNC:6083]
ASNS	asparagine synthetase (glutamine-hydrolyzing) [Source:HGNC Symbol;Acc:HGNC:753]
BCL2L11	BCL2 like 11 [Source:HGNC Symbol;Acc:HGNC:994]
COMT	catechol-O-methyltransferase [Source:HGNC Symbol;Acc:HGNC:2228]
SLC2A4	solute carrier family 2 member 4 [Source:HGNC Symbol;Acc:HGNC:11009]
RXRA	retinoid X receptor alpha [Source:HGNC Symbol;Acc:HGNC:10477]
SLC22A2	solute carrier family 22 member 2 [Source:HGNC Symbol;Acc:HGNC:10966]
HMGCS1	3-hydroxy-3-methylglutaryl-CoA synthase 1 [Source:HGNC Symbol;Acc:HGNC:5007]
ABCC4	ATP binding cassette subfamily C member 4 [Source:HGNC Symbol;Acc:HGNC:55]

CD86	CD86 molecule [Source:HGNC Symbol;Acc:HGNC:1705]
CYP7A1	cytochrome P450 family 7 subfamily A member 1 [Source:HGNC Symbol;Acc:HGNC:2651]
SULT1A1	sulfotransferase family 1A member 1 [Source:HGNC Symbol;Acc:HGNC:11453]
TOP2A	topoisomerase (DNA) II alpha [Source:HGNC Symbol;Acc:HGNC:11989]
CEBPB	CCAAT/enhancer binding protein beta [Source:HGNC Symbol;Acc:HGNC:1834]
UGT1A1	UDP glucuronosyltransferase family 1 member A1 [Source:HGNC Symbol;Acc:HGNC:12530]
CDK2	cyclin dependent kinase 2 [Source:HGNC Symbol;Acc:HGNC:1771]
KCNH2	potassium voltage-gated channel subfamily H member 2 [Source:HGNC Symbol;Acc:HGNC:6251]
RB1	RB transcriptional corepressor 1 [Source:HGNC Symbol;Acc:HGNC:9884]
BIRC5	baculoviral IAP repeat containing 5 [Source:HGNC Symbol;Acc:HGNC:593]
NR1I2	nuclear receptor subfamily 1 group I member 2 [Source:HGNC Symbol;Acc:HGNC:7968]
CYP3A4	cytochrome P450 family 3 subfamily A member 4 [Source:HGNC Symbol;Acc:HGNC:2637]
EPC1	enhancer of polycomb homolog 1 [Source:HGNC Symbol;Acc:HGNC:19876]
HUS1	HUS1 checkpoint clamp component [Source:HGNC Symbol;Acc:HGNC:5309]
OTUD6B	OTU domain containing 6B [Source:HGNC Symbol;Acc:HGNC:24281]
WWC2	WW and C2 domain containing 2 [Source:HGNC Symbol;Acc:HGNC:24148]
CDR2	cerebellar degeneration related protein 2 [Source:HGNC Symbol;Acc:HGNC:1799]
PEX5	peroxisomal biogenesis factor 5 [Source:HGNC Symbol;Acc:HGNC:9719]
CREB5	cAMP responsive element binding protein 5 [Source:HGNC Symbol;Acc:HGNC:16844]
CTSG	cathepsin G [Source:HGNC Symbol;Acc:HGNC:2532]
DACT2	dishevelled binding antagonist of beta catenin 2 [Source:HGNC Symbol;Acc:HGNC:21231]
GABARAPL2	GABA type A receptor associated protein like 2 [Source:HGNC Symbol;Acc:HGNC:13291]
HIP1	huntingtin interacting protein 1 [Source:HGNC Symbol;Acc:HGNC:4913]
MAML1	mastermind like transcriptional coactivator 1 [Source:HGNC Symbol;Acc:HGNC:13632]
MAML3	mastermind like transcriptional coactivator 3 [Source:HGNC Symbol;Acc:HGNC:16272]
PPP1CC	protein phosphatase 1 catalytic subunit gamma [Source:HGNC Symbol;Acc:HGNC:9283]
PPP2R2A	protein phosphatase 2 regulatory subunit Balpha [Source:HGNC Symbol;Acc:HGNC:9304]
SMAD6	SMAD family member 6 [Source:HGNC Symbol;Acc:HGNC:6772]
SMURF1	SMAD specific E3 ubiquitin protein ligase 1 [Source:HGNC Symbol;Acc:HGNC:16807]
SMURF2	SMAD specific E3 ubiquitin protein ligase 2 [Source:HGNC Symbol;Acc:HGNC:16809]
UCHL5	ubiquitin C-terminal hydrolase L5 [Source:HGNC Symbol;Acc:HGNC:19678]
ZMYND8	zinc finger MYND-type containing 8 [Source:HGNC Symbol;Acc:HGNC:9397]
GABARAPL1	GABA type A receptor associated protein like 1 [Source:HGNC Symbol;Acc:HGNC:4068]
NBR1	NBR1, autophagy cargo receptor [Source:HGNC Symbol;Acc:HGNC:6746]
CD226	CD226 molecule [Source:HGNC Symbol;Acc:HGNC:16961]

CARTPT	CART prepropeptide [Source:HGNC Symbol;Acc:HGNC:24323]
SCN2A	sodium voltage-gated channel alpha subunit 2 [Source:HGNC Symbol;Acc:HGNC:10588]
PIK3R2	phosphoinositide-3-kinase regulatory subunit 2 [Source:HGNC Symbol;Acc:HGNC:8980]
MAP3K8	mitogen-activated protein kinase kinase kinase 8 [Source:HGNC Symbol;Acc:HGNC:6860]
TIRAP	toll-interleukin 1 receptor (TIR) domain containing adaptor protein [Source:HGNC Symbol;Acc:HGNC:17192]
TICAM2	toll like receptor adaptor molecule 2 [Source:HGNC Symbol;Acc:HGNC:21354]
VPS39	VPS39, HOPS complex subunit [Source:HGNC Symbol;Acc:HGNC:20593]
MTRNR2L13	MT-RNR2-like 13 [Source:HGNC Symbol;Acc:HGNC:37170]
CLIC5	chloride intracellular channel 5 [Source:HGNC Symbol;Acc:HGNC:13517]
ADGRL3	adhesion G protein-coupled receptor L3 [Source:HGNC Symbol;Acc:HGNC:20974]
ACTR6	ARP6 actin-related protein 6 homolog [Source:HGNC Symbol;Acc:HGNC:24025]
GLI2	GLI family zinc finger 2 [Source:HGNC Symbol;Acc:HGNC:4318]
GLI3	GLI family zinc finger 3 [Source:HGNC Symbol;Acc:HGNC:4319]
FAM84B	family with sequence similarity 84 member B [Source:HGNC Symbol;Acc:HGNC:24166]
TRPC4	transient receptor potential cation channel subfamily C member 4 [Source:HGNC Symbol;Acc:HGNC:12336]
NMRK2	nicotinamide riboside kinase 2 [Source:HGNC Symbol;Acc:HGNC:17871]
DAPK3	death associated protein kinase 3 [Source:HGNC Symbol;Acc:HGNC:2676]
TDRP	testis development related protein [Source:HGNC Symbol;Acc:HGNC:26951]
WTAP	Wilms tumor 1 associated protein [Source:HGNC Symbol;Acc:HGNC:16846]
DNAJC27	DnaJ heat shock protein family (Hsp40) member C27 [Source:HGNC Symbol;Acc:HGNC:30290]
OTOS	otospiralin [Source:HGNC Symbol;Acc:HGNC:22644]
RHBDD1	rhomboid domain containing 1 [Source:HGNC Symbol;Acc:HGNC:23081]
ZNF341	zinc finger protein 341 [Source:HGNC Symbol;Acc:HGNC:15992]
OR7G3	olfactory receptor family 7 subfamily G member 3 [Source:HGNC Symbol;Acc:HGNC:8467]
WBSCR17	Williams-Beuren syndrome chromosome region 17 [Source:HGNC Symbol;Acc:HGNC:16347]
ICAM3	intercellular adhesion molecule 3 [Source:HGNC Symbol;Acc:HGNC:5346]
LSM5	LSM5 homolog, U6 small nuclear RNA and mRNA degradation associated [Source:HGNC Symbol;Acc:HGNC:17162]
NALCN	sodium leak channel, non-selective [Source:HGNC Symbol;Acc:HGNC:19082]
ZNF700	zinc finger protein 700 [Source:HGNC Symbol;Acc:HGNC:25292]
GLIS3	GLIS family zinc finger 3 [Source:HGNC Symbol;Acc:HGNC:28510]
DGKI	diacylglycerol kinase iota [Source:HGNC Symbol;Acc:HGNC:2855]
MSH5	mutS homolog 5 [Source:HGNC Symbol;Acc:HGNC:7328]
RNF123	ring finger protein 123 [Source:HGNC Symbol;Acc:HGNC:21148]
EMILIN2	elastin microfibril interfacer 2 [Source:HGNC Symbol;Acc:HGNC:19881]
CSNK2B	casein kinase 2 beta [Source:HGNC Symbol;Acc:HGNC:2460]

FAM46A	family with sequence similarity 46 member A [Source:HGNC Symbol;Acc:HGNC:18345]
YTHDC1	YTH domain containing 1 [Source:HGNC Symbol;Acc:HGNC:30626]
ERGIC3	ERGIC and golgi 3 [Source:HGNC Symbol;Acc:HGNC:15927]
NAALADL2	N-acetylated alpha-linked acidic dipeptidase-like 2 [Source:HGNC Symbol;Acc:HGNC:23219]
SLC35C2	solute carrier family 35 member C2 [Source:HGNC Symbol;Acc:HGNC:17117]
LAMC3	laminin subunit gamma 3 [Source:HGNC Symbol;Acc:HGNC:6494]
CACNB3	calcium voltage-gated channel auxiliary subunit beta 3 [Source:HGNC Symbol;Acc:HGNC:1403]
CDK11A	cyclin dependent kinase 11A [Source:HGNC Symbol;Acc:HGNC:1730]
GALNT13	polypeptide N-acetylgalactosaminyltransferase 13 [Source:HGNC Symbol;Acc:HGNC:23242]
PMPCA	peptidase, mitochondrial processing alpha subunit [Source:HGNC Symbol;Acc:HGNC:18667]
SNRPB	small nuclear ribonucleoprotein polypeptides B and B1 [Source:HGNC Symbol;Acc:HGNC:11153]
TSSK2	testis specific serine kinase 2 [Source:HGNC Symbol;Acc:HGNC:11401]
XRN1	5'-3' exoribonuclease 1 [Source:HGNC Symbol;Acc:HGNC:30654]
ITGAX	integrin subunit alpha X [Source:HGNC Symbol;Acc:HGNC:6152]
TGM2	transglutaminase 2 [Source:HGNC Symbol;Acc:HGNC:11778]
ANKH	ANKH inorganic pyrophosphate transport regulator [Source:HGNC Symbol;Acc:HGNC:15492]
ARFGAP1	ADP ribosylation factor GTPase activating protein 1 [Source:HGNC Symbol;Acc:HGNC:15852]
CDC37	cell division cycle 37 [Source:HGNC Symbol;Acc:HGNC:1735]
CSNK2A1	casein kinase 2 alpha 1 [Source:HGNC Symbol;Acc:HGNC:2457]
ECSIT	ECSIT signalling integrator [Source:HGNC Symbol;Acc:HGNC:29548]
EPN2	epsin 2 [Source:HGNC Symbol;Acc:HGNC:18639]
FARSA	phenylalanyl-tRNA synthetase alpha subunit [Source:HGNC Symbol;Acc:HGNC:3592]
FBXL12	F-box and leucine rich repeat protein 12 [Source:HGNC Symbol;Acc:HGNC:13611]
FXYD7	FXYD domain containing ion transport regulator 7 [Source:HGNC Symbol;Acc:HGNC:4034]
GCDH	glutaryl-CoA dehydrogenase [Source:HGNC Symbol;Acc:HGNC:4189]
IFIT5	interferon induced protein with tetratricopeptide repeats 5 [Source:HGNC Symbol;Acc:HGNC:13328]
IQSEC1	IQ motif and Sec7 domain 1 [Source:HGNC Symbol;Acc:HGNC:29112]
LOXL4	lysyl oxidase like 4 [Source:HGNC Symbol;Acc:HGNC:17171]
MAST1	microtubule associated serine/threonine kinase 1 [Source:HGNC Symbol;Acc:HGNC:19034]
NEFM	neurofilament, medium polypeptide [Source:HGNC Symbol;Acc:HGNC:7734]
PCMT1	protein-L-isoaspartate (D-aspartate) O-methyltransferase [Source:HGNC Symbol;Acc:HGNC:8728]
PLEKHA6	pleckstrin homology domain containing A6 [Source:HGNC Symbol;Acc:HGNC:17053]
PRAM1	PML-RARA regulated adaptor molecule 1 [Source:HGNC Symbol;Acc:HGNC:30091]

RNF32	ring finger protein 32 [Source:HGNC Symbol;Acc:HGNC:17118]
TMCC2	transmembrane and coiled-coil domain family 2 [Source:HGNC Symbol;Acc:HGNC:24239]
TYRO3	TYRO3 protein tyrosine kinase [Source:HGNC Symbol;Acc:HGNC:12446]
ZNF558	zinc finger protein 558 [Source:HGNC Symbol;Acc:HGNC:26422]
C9orf72	chromosome 9 open reading frame 72 [Source:HGNC Symbol;Acc:HGNC:28337]
MORF4L2	mortality factor 4 like 2 [Source:HGNC Symbol;Acc:HGNC:16849]
PIAS2	protein inhibitor of activated STAT 2 [Source:HGNC Symbol;Acc:HGNC:17311]
ADHFE1	alcohol dehydrogenase, iron containing 1 [Source:HGNC Symbol;Acc:HGNC:16354]
ADAMTS1	ADAM metallopeptidase with thrombospondin type 1 motif 1 [Source:HGNC Symbol;Acc:HGNC:217]
LYVE1	lymphatic vessel endothelial hyaluronan receptor 1 [Source:HGNC Symbol;Acc:HGNC:14687]
U2AF1	U2 small nuclear RNA auxiliary factor 1 [Source:HGNC Symbol;Acc:HGNC:12453]
VPS35	VPS35, retromer complex component [Source:HGNC Symbol;Acc:HGNC:13487]
ARAP1	ArfGAP with RhoGAP domain, ankyrin repeat and PH domain 1 [Source:HGNC Symbol;Acc:HGNC:16925]
DAP3	death associated protein 3 [Source:HGNC Symbol;Acc:HGNC:2673]
ARAP3	ArfGAP with RhoGAP domain, ankyrin repeat and PH domain 3 [Source:HGNC Symbol;Acc:HGNC:24097]
ARMC5	armadillo repeat containing 5 [Source:HGNC Symbol;Acc:HGNC:25781]
C11orf1	chromosome 11 open reading frame 1 [Source:HGNC Symbol;Acc:HGNC:1163]
C19orf54	chromosome 19 open reading frame 54 [Source:HGNC Symbol;Acc:HGNC:24758]
C22orf39	chromosome 22 open reading frame 39 [Source:HGNC Symbol;Acc:HGNC:27012]
C9orf24	chromosome 9 open reading frame 24 [Source:HGNC Symbol;Acc:HGNC:19919]
CYSRT1	cysteine rich tail 1 [Source:HGNC Symbol;Acc:HGNC:30529]
DAZAP2	DAZ associated protein 2 [Source:HGNC Symbol;Acc:HGNC:2684]
FAM86C1	family with sequence similarity 86 member C1 [Source:HGNC Symbol;Acc:HGNC:25561]
FBXO34	F-box protein 34 [Source:HGNC Symbol;Acc:HGNC:20201]
FRS3	fibroblast growth factor receptor substrate 3 [Source:HGNC Symbol;Acc:HGNC:16970]
GLYR1	glyoxylate reductase 1 homolog [Source:HGNC Symbol;Acc:HGNC:24434]
INCA1	inhibitor of CDK, cyclin A1 interacting protein 1 [Source:HGNC Symbol;Acc:HGNC:32224]
KLHL12	kelch like family member 12 [Source:HGNC Symbol;Acc:HGNC:19360]
KLHL20	kelch like family member 20 [Source:HGNC Symbol;Acc:HGNC:25056]
KRT40	keratin 40 [Source:HGNC Symbol;Acc:HGNC:26707]
LIMS1	LIM zinc finger domain containing 1 [Source:HGNC Symbol;Acc:HGNC:6616]
NADSYN1	NAD synthetase 1 [Source:HGNC Symbol;Acc:HGNC:29832]
NOTCH2NL	notch 2 N-terminal like [Source:HGNC Symbol;Acc:HGNC:31862]
RHBDD2	rhomboid domain containing 2 [Source:HGNC Symbol;Acc:HGNC:23082]
SLC35A2	solute carrier family 35 member A2 [Source:HGNC Symbol;Acc:HGNC:11022]
TMEM256	transmembrane protein 256 [Source:HGNC Symbol;Acc:HGNC:28618]

TRIM35	tripartite motif containing 35 [Source:HGNC Symbol;Acc:HGNC:16285]
UBQLN2	ubiquilin 2 [Source:HGNC Symbol;Acc:HGNC:12509]
UBQLN4	ubiquilin 4 [Source:HGNC Symbol;Acc:HGNC:1237]
VAC14	Vac14, PIKFYVE complex component [Source:HGNC Symbol;Acc:HGNC:25507]
YPEL3	yippee like 3 [Source:HGNC Symbol;Acc:HGNC:18327]
ZNF720	zinc finger protein 720 [Source:HGNC Symbol;Acc:HGNC:26987]
ZNF774	zinc finger protein 774 [Source:HGNC Symbol;Acc:HGNC:33108]
ZNHIT1	zinc finger HIT-type containing 1 [Source:HGNC Symbol;Acc:HGNC:21688]
ACVR1	activin A receptor type 1 [Source:HGNC Symbol;Acc:HGNC:171]
APPL1	adaptor protein, phosphotyrosine interacting with PH domain and leucine zipper 1 [Source:HGNC Symbol;Acc:HGNC:24035]
BAMBI	BMP and activin membrane bound inhibitor [Source:HGNC Symbol;Acc:HGNC:30251]
FNTA	farnesyltransferase, CAAX box, alpha [Source:HGNC Symbol;Acc:HGNC:3782]
OTUB1	OTU deubiquitinase, ubiquitin aldehyde binding 1 [Source:HGNC Symbol;Acc:HGNC:23077]
RANBP9	RAN binding protein 9 [Source:HGNC Symbol;Acc:HGNC:13727]
ADAMTS18	ADAM metallopeptidase with thrombospondin type 1 motif 18 [Source:HGNC Symbol;Acc:HGNC:17110]
ADAMTSL1	ADAMTS like 1 [Source:HGNC Symbol;Acc:HGNC:14632]
MEOX2	mesenchyme homeobox 2 [Source:HGNC Symbol;Acc:HGNC:7014]
RPAIN	RPA interacting protein [Source:HGNC Symbol;Acc:HGNC:28641]
CACTIN	cactin, spliceosome C complex subunit [Source:HGNC Symbol;Acc:HGNC:29938]
CCDC57	coiled-coil domain containing 57 [Source:HGNC Symbol;Acc:HGNC:27564]
MTUS2	microtubule associated tumor suppressor candidate 2 [Source:HGNC Symbol;Acc:HGNC:20595]
NELFB	negative elongation factor complex member B [Source:HGNC Symbol;Acc:HGNC:24324]
NELFCD	negative elongation factor complex member C/D [Source:HGNC Symbol;Acc:HGNC:15934]
VAMP3	vesicle associated membrane protein 3 [Source:HGNC Symbol;Acc:HGNC:12644]
GZMK	granzyme K [Source:HGNC Symbol;Acc:HGNC:4711]
UFD1L	ubiquitin fusion degradation 1 like (yeast) [Source:HGNC Symbol;Acc:HGNC:12520]
VCP	valosin containing protein [Source:HGNC Symbol;Acc:HGNC:12666]
YOD1	YOD1 deubiquitinase [Source:HGNC Symbol;Acc:HGNC:25035]
DNPH1	2'-deoxynucleoside 5'-phosphate N-hydrolase 1 [Source:HGNC Symbol;Acc:HGNC:21218]
PTPN6	protein tyrosine phosphatase, non-receptor type 6 [Source:HGNC Symbol;Acc:HGNC:9658]
SDCBP	syndecan binding protein [Source:HGNC Symbol;Acc:HGNC:10662]
1-Dec	deleted in esophageal cancer 1 [Source:HGNC Symbol;Acc:HGNC:23658]
AES	amino-terminal enhancer of split [Source:HGNC Symbol;Acc:HGNC:307]
ANKS1A	ankyrin repeat and sterile alpha motif domain containing 1A [Source:HGNC Symbol;Acc:HGNC:20961]

CLOCK	clock circadian regulator [Source:HGNC Symbol;Acc:HGNC:2082]
NSD1	nuclear receptor binding SET domain protein 1 [Source:HGNC Symbol;Acc:HGNC:14234]
PSMC4	proteasome 26S subunit, ATPase 4 [Source:HGNC Symbol;Acc:HGNC:9551]
RORC	RAR related orphan receptor C [Source:HGNC Symbol;Acc:HGNC:10260]
SCNM1	sodium channel modifier 1 [Source:HGNC Symbol;Acc:HGNC:23136]
SMARCD1	SWI/SNF related, matrix associated, actin dependent regulator of chromatin, subfamily d, member 1 [Source:HGNC Symbol;Acc:HGNC:11106]
UBE2I	ubiquitin conjugating enzyme E2 I [Source:HGNC Symbol;Acc:HGNC:12485]
ZNF587	zinc finger protein 587 [Source:HGNC Symbol;Acc:HGNC:30955]
H3F3A	H3 histone, family 3A [Source:HGNC Symbol;Acc:HGNC:4764]
ADPRH	ADP-ribosylarginine hydrolase [Source:HGNC Symbol;Acc:HGNC:269]
AHCYL1	adenosylhomocysteinase like 1 [Source:HGNC Symbol;Acc:HGNC:344]
ALKBH3	alkB homolog 3, alpha-ketoglutaratedependent dioxygenase [Source:HGNC Symbol;Acc:HGNC:30141]
AMMECR1L	AMMECR1 like [Source:HGNC Symbol;Acc:HGNC:28658]
ANK3	ankyrin 3, node of Ranvier (ankyrin G) [Source:HGNC Symbol;Acc:HGNC:494]
ANKFY1	ankyrin repeat and FYVE domain containing 1 [Source:HGNC Symbol;Acc:HGNC:20763]
ANKRD35	ankyrin repeat domain 35 [Source:HGNC Symbol;Acc:HGNC:26323]
AP1M1	adaptor related protein complex 1 mu 1 subunit [Source:HGNC Symbol;Acc:HGNC:13667]
APOBEC3C	apolipoprotein B mRNA editing enzyme catalytic subunit 3C [Source:HGNC Symbol;Acc:HGNC:17353]
ARGLU1	arginine and glutamate rich 1 [Source:HGNC Symbol;Acc:HGNC:25482]
ARHGAP12	Rho GTPase activating protein 12 [Source:HGNC Symbol;Acc:HGNC:16348]
ARL6IP4	ADP ribosylation factor like GTPase 6 interacting protein 4 [Source:HGNC Symbol;Acc:HGNC:18076]
ASL	argininosuccinate lyase [Source:HGNC Symbol;Acc:HGNC:746]
BAIAP2	BAI1 associated protein 2 [Source:HGNC Symbol;Acc:HGNC:947]
BAZ1A	bromodomain adjacent to zinc finger domain 1A [Source:HGNC Symbol;Acc:HGNC:960]
BCL9L	B-cell CLL/lymphoma 9-like [Source:HGNC Symbol;Acc:HGNC:23688]
BCLAF1	BCL2 associated transcription factor 1 [Source:HGNC Symbol;Acc:HGNC:16863]
BRD3	bromodomain containing 3 [Source:HGNC Symbol;Acc:HGNC:1104]
C18orf25	chromosome 18 open reading frame 25 [Source:HGNC Symbol;Acc:HGNC:28172]
C1orf35	chromosome 1 open reading frame 35 [Source:HGNC Symbol;Acc:HGNC:19032]
C5orf42	chromosome 5 open reading frame 42 [Source:HGNC Symbol;Acc:HGNC:25801]
CACNG2	calcium voltage-gated channel auxiliary subunit gamma 2 [Source:HGNC Symbol;Acc:HGNC:1406]
CALCOCO1	calcium binding and coiled-coil domain 1 [Source:HGNC Symbol;Acc:HGNC:29306]
CAMSAP1	calmodulin regulated spectrin associated protein 1 [Source:HGNC Symbol;Acc:HGNC:19946]
CAND1	cullin associated and neddylation dissociated 1 [Source:HGNC Symbol;Acc:HGNC:30688]

CARD11	caspase recruitment domain family member 11 [Source:HGNC Symbol;Acc:HGNC:16393]
CASK	calcium/calmodulin dependent serine protein kinase [Source:HGNC Symbol;Acc:HGNC:1497]
CCDC28A	coiled-coil domain containing 28A [Source:HGNC Symbol;Acc:HGNC:21098]
CCDC86	coiled-coil domain containing 86 [Source:HGNC Symbol;Acc:HGNC:28359]
CCM2	CCM2 scaffolding protein [Source:HGNC Symbol;Acc:HGNC:21708]
CDC42EP4	CDC42 effector protein 4 [Source:HGNC Symbol;Acc:HGNC:17147]
CDK13	cyclin dependent kinase 13 [Source:HGNC Symbol;Acc:HGNC:1733]
CDK7	cyclin dependent kinase 7 [Source:HGNC Symbol;Acc:HGNC:1778]
CEP170	centrosomal protein 170 [Source:HGNC Symbol;Acc:HGNC:28920]
CHD1L	chromodomain helicase DNA binding protein 1 like [Source:HGNC Symbol;Acc:HGNC:1916]
CHERP	calcium homeostasis endoplasmic reticulum protein [Source:HGNC Symbol;Acc:HGNC:16930]
CHTOP	chromatin target of PRMT1 [Source:HGNC Symbol;Acc:HGNC:24511]
CLASP1	cytoplasmic linker associated protein 1 [Source:HGNC Symbol;Acc:HGNC:17088]
CLASP2	cytoplasmic linker associated protein 2 [Source:HGNC Symbol;Acc:HGNC:17078]
CLASRP	CLK4 associating serine/arginine rich protein [Source:HGNC Symbol;Acc:HGNC:17731]
CLCNKA	chloride voltage-gated channel Ka [Source:HGNC Symbol;Acc:HGNC:2026]
CLK2	CDC like kinase 2 [Source:HGNC Symbol;Acc:HGNC:2069]
CLK3	CDC like kinase 3 [Source:HGNC Symbol;Acc:HGNC:2071]
CPSF7	cleavage and polyadenylation specific factor 7 [Source:HGNC Symbol;Acc:HGNC:30098]
CRTC1	CREB regulated transcription coactivator 1 [Source:HGNC Symbol;Acc:HGNC:16062]
CTNNBL1	catenin beta like 1 [Source:HGNC Symbol;Acc:HGNC:15879]
DBF4B	DBF4 zinc finger B [Source:HGNC Symbol;Acc:HGNC:17883]
DCAF10	DDB1 and CUL4 associated factor 10 [Source:HGNC Symbol;Acc:HGNC:23686]
DDX39B	DEAD-box helicase 39B [Source:HGNC Symbol;Acc:HGNC:13917]
DDX3X	DEAD-box helicase 3, X-linked [Source:HGNC Symbol;Acc:HGNC:2745]
DDX46	DEAD-box helicase 46 [Source:HGNC Symbol;Acc:HGNC:18681]
DDX6	DEAD-box helicase 6 [Source:HGNC Symbol;Acc:HGNC:2747]
DHX8	DEAH-box helicase 8 [Source:HGNC Symbol;Acc:HGNC:2749]
DIDO1	death inducer-obliterator 1 [Source:HGNC Symbol;Acc:HGNC:2680]
DNAH9	dynein axonemal heavy chain 9 [Source:HGNC Symbol;Acc:HGNC:2953]
DNAJC8	DnaJ heat shock protein family (Hsp40) member C8 [Source:HGNC Symbol;Acc:HGNC:15470]
DOCK3	dedicator of cytokinesis 3 [Source:HGNC Symbol;Acc:HGNC:2989]
DRAP1	DR1 associated protein 1 [Source:HGNC Symbol;Acc:HGNC:3019]
DRG1	developmentally regulated GTP binding protein 1 [Source:HGNC Symbol;Acc:HGNC:3029]
EFCAB12	EF-hand calcium binding domain 12 [Source:HGNC Symbol;Acc:HGNC:28061]
EGLN2	egl-9 family hypoxia inducible factor 2 [Source:HGNC Symbol;Acc:HGNC:14660]

EIF1AY	eukaryotic translation initiation factor 1A, Y-linked [Source:HGNC Symbol;Acc:HGNC:3252]
ELAVL3	ELAV like neuron-specific RNA binding protein 3 [Source:HGNC Symbol;Acc:HGNC:3314]
ELP6	elongator acetyltransferase complex subunit 6 [Source:HGNC Symbol;Acc:HGNC:25976]
ENOX2	ecto-NOX disulfide-thiol exchanger 2 [Source:HGNC Symbol;Acc:HGNC:2259]
ENSA	endosulfine alpha [Source:HGNC Symbol;Acc:HGNC:3360]
EPM2A	epilepsy, progressive myoclonus type 2A, Lafora disease (laforin) [Source:HGNC Symbol;Acc:HGNC:3413]
ERC1	ELKS/RAB6-interacting/CAST family member 1 [Source:HGNC Symbol;Acc:HGNC:17072]
EVI5L	ecotropic viral integration site 5 like [Source:HGNC Symbol;Acc:HGNC:30464]
FAM76B	family with sequence similarity 76 member B [Source:HGNC Symbol;Acc:HGNC:28492]
FBXO24	F-box protein 24 [Source:HGNC Symbol;Acc:HGNC:13595]
FCHO1	FCH domain only 1 [Source:HGNC Symbol;Acc:HGNC:29002]
FGF12	fibroblast growth factor 12 [Source:HGNC Symbol;Acc:HGNC:3668]
FIP1L1	factor interacting with PAPOLA and CPSF1 [Source:HGNC Symbol;Acc:HGNC:19124]
FMNL1	formin like 1 [Source:HGNC Symbol;Acc:HGNC:1212]
FXR2	FMR1 autosomal homolog 2 [Source:HGNC Symbol;Acc:HGNC:4024]
GBF1	golgi brefeldin A resistant guanine nucleotide exchange factor 1 [Source:HGNC Symbol;Acc:HGNC:4181]
GCC2	GRIP and coiled-coil domain containing 2 [Source:HGNC Symbol;Acc:HGNC:23218]
GGCX	gamma-glutamyl carboxylase [Source:HGNC Symbol;Acc:HGNC:4247]
GSE1	Gse1 coiled-coil protein [Source:HGNC Symbol;Acc:HGNC:28979]
GTF2E1	general transcription factor IIE subunit 1 [Source:HGNC Symbol;Acc:HGNC:4650]
GTPBP4	GTP binding protein 4 [Source:HGNC Symbol;Acc:HGNC:21535]
GXYLT2	glucoside xylosyltransferase 2 [Source:HGNC Symbol;Acc:HGNC:33383]
HERC3	HECT and RLD domain containing E3 ubiquitin protein ligase 3 [Source:HGNC Symbol;Acc:HGNC:4876]
HIST1H2BK	histone cluster 1, H2bk [Source:HGNC Symbol;Acc:HGNC:13954]
HMGA1	high mobility group AT-hook 1 [Source:HGNC Symbol;Acc:HGNC:5010]
HMGN3	high mobility group nucleosomal binding domain 3 [Source:HGNC Symbol;Acc:HGNC:12312]
HMGXB4	HMG-box containing 4 [Source:HGNC Symbol;Acc:HGNC:5003]
HNRNPC	heterogeneous nuclear ribonucleoprotein C (C1/C2) [Source:HGNC Symbol;Acc:HGNC:5035]
HOMER3	homer scaffolding protein 3 [Source:HGNC Symbol;Acc:HGNC:17514]
HTATSF1	HIV-1 Tat specific factor 1 [Source:HGNC Symbol;Acc:HGNC:5276]
ISLR	immunoglobulin superfamily containing leucine rich repeat [Source:HGNC Symbol;Acc:HGNC:6133]
JMJD1C	jumonji domain containing 1C [Source:HGNC Symbol;Acc:HGNC:12313]
KCNN2	potassium calcium-activated channel subfamily N member 2 [Source:HGNC Symbol;Acc:HGNC:6291]

LRRN4CL	LRRN4 C-terminal like [Source:HGNC Symbol;Acc:HGNC:33724]
LUC7L	LUC7 like [Source:HGNC Symbol;Acc:HGNC:6723]
LUC7L2	LUC7-like 2 pre-mRNA splicing factor [Source:HGNC Symbol;Acc:HGNC:21608]
LUC7L3	LUC7 like 3 pre-mRNA splicing factor [Source:HGNC Symbol;Acc:HGNC:24309]
MAB21L2	mab-21 like 2 [Source:HGNC Symbol;Acc:HGNC:6758]
MACF1	microtubule-actin crosslinking factor 1 [Source:HGNC Symbol;Acc:HGNC:13664]
MAP1S	microtubule associated protein 1S [Source:HGNC Symbol;Acc:HGNC:15715]
MAP7D1	MAP7 domain containing 1 [Source:HGNC Symbol;Acc:HGNC:25514]
MAPKAPK5	mitogen-activated protein kinase-activated protein kinase 5 [Source:HGNC Symbol;Acc:HGNC:6889]
MAPRE1	microtubule associated protein RP/EB family member 1 [Source:HGNC Symbol;Acc:HGNC:6890]
MAST2	microtubule associated serine/threonine kinase 2 [Source:HGNC Symbol;Acc:HGNC:19035]
MCOLN3	mucolipin 3 [Source:HGNC Symbol;Acc:HGNC:13358]
MDC1	mediator of DNA damage checkpoint 1 [Source:HGNC Symbol;Acc:HGNC:21163]
MDK	midkine (neurite growth-promoting factor 2) [Source:HGNC Symbol;Acc:HGNC:6972]
MFSD10	major facilitator superfamily domain containing 10 [Source:HGNC Symbol;Acc:HGNC:16894]
MIB2	mindbomb E3 ubiquitin protein ligase 2 [Source:HGNC Symbol;Acc:HGNC:30577]
MIPOL1	mirror-image polydactyly 1 [Source:HGNC Symbol;Acc:HGNC:21460]
MPP3	membrane palmitoylated protein 3 [Source:HGNC Symbol;Acc:HGNC:7221]
MTCL1	microtubule crosslinking factor 1 [Source:HGNC Symbol;Acc:HGNC:29121]
MYCBP2	MYC binding protein 2, E3 ubiquitin protein ligase [Source:HGNC Symbol;Acc:HGNC:23386]
MYO1E	myosin IE [Source:HGNC Symbol;Acc:HGNC:7599]
MZT2B	mitotic spindle organizing protein 2B [Source:HGNC Symbol;Acc:HGNC:25886]
NADK	NAD kinase [Source:HGNC Symbol;Acc:HGNC:29831]
NAGK	N-acetylglucosamine kinase [Source:HGNC Symbol;Acc:HGNC:17174]
NCBP3	nuclear cap binding subunit 3 [Source:HGNC Symbol;Acc:HGNC:24612]
NCK2	NCK adaptor protein 2 [Source:HGNC Symbol;Acc:HGNC:7665]
NGDN	neuroguidin [Source:HGNC Symbol;Acc:HGNC:20271]
NGLY1	N-glycanase 1 [Source:HGNC Symbol;Acc:HGNC:17646]
NKAP	NFKB activating protein [Source:HGNC Symbol;Acc:HGNC:29873]
NPM2	nucleophosmin/nucleoplasmin 2 [Source:HGNC Symbol;Acc:HGNC:7930]
NSMCE4A	NSE4 homolog A, SMC5-SMC6 complex component [Source:HGNC Symbol;Acc:HGNC:25935]
NTHL1	nth-like DNA glycosylase 1 [Source:HGNC Symbol;Acc:HGNC:8028]
NUP50	nucleoporin 50 [Source:HGNC Symbol;Acc:HGNC:8065]
NXF1	nuclear RNA export factor 1 [Source:HGNC Symbol;Acc:HGNC:8071]
NXT2	nuclear transport factor 2 like export factor 2 [Source:HGNC Symbol;Acc:HGNC:18151]
OCEL1	occludin/ELL domain containing 1 [Source:HGNC Symbol;Acc:HGNC:26221]
PABPC4	poly(A) binding protein cytoplasmic 4 [Source:HGNC Symbol;Acc:HGNC:8557]

PAK4	p21 (RAC1) activated kinase 4 [Source:HGNC Symbol;Acc:HGNC:16059]
PALLD	palladin, cytoskeletal associated protein [Source:HGNC Symbol;Acc:HGNC:17068]
PCBP1	poly(rC) binding protein 1 [Source:HGNC Symbol;Acc:HGNC:8647]
PDCL3	phosducin like 3 [Source:HGNC Symbol;Acc:HGNC:28860]
PDE7B	phosphodiesterase 7B [Source:HGNC Symbol;Acc:HGNC:8792]
PNISR	PNN interacting serine and arginine rich protein [Source:HGNC Symbol;Acc:HGNC:21222]
PPHLN1	periphilin 1 [Source:HGNC Symbol;Acc:HGNC:19369]
PPIL1	peptidylprolyl isomerase like 1 [Source:HGNC Symbol;Acc:HGNC:9260]
PRPF38A	pre-mRNA processing factor 38A [Source:HGNC Symbol;Acc:HGNC:25930]
PRPF38B	pre-mRNA processing factor 38B [Source:HGNC Symbol;Acc:HGNC:25512]
PRRC2B	proline rich coiled-coil 2B [Source:HGNC Symbol;Acc:HGNC:28121]
PRRC2C	proline rich coiled-coil 2C [Source:HGNC Symbol;Acc:HGNC:24903]
PSMA3	proteasome subunit alpha 3 [Source:HGNC Symbol;Acc:HGNC:9532]
PSMD13	proteasome 26S subunit, non-ATPase 13 [Source:HGNC Symbol;Acc:HGNC:9558]
PTBP1	polypyrimidine tract binding protein 1 [Source:HGNC Symbol;Acc:HGNC:9583]
RABEP1	rabaptin, RAB GTPase binding effector protein 1 [Source:HGNC Symbol;Acc:HGNC:17677]
RBM15B	RNA binding motif protein 15B [Source:HGNC Symbol;Acc:HGNC:24303]
RBM23	RNA binding motif protein 23 [Source:HGNC Symbol;Acc:HGNC:20155]
RBMX	RNA binding motif protein, X-linked [Source:HGNC Symbol;Acc:HGNC:9910]
RIT1	Ras like without CAAX 1 [Source:HGNC Symbol;Acc:HGNC:10023]
RIT2	Ras like without CAAX 2 [Source:HGNC Symbol;Acc:HGNC:10017]
RNF219	ring finger protein 219 [Source:HGNC Symbol;Acc:HGNC:20308]
RNPS1	RNA binding protein with serine rich domain 1 [Source:HGNC Symbol;Acc:HGNC:10080]
RPL39L	ribosomal protein L39 like [Source:HGNC Symbol;Acc:HGNC:17094]
RPL41	ribosomal protein L41 [Source:HGNC Symbol;Acc:HGNC:10354]
RPS26	ribosomal protein S26 [Source:HGNC Symbol;Acc:HGNC:10414]
RSL1D1	ribosomal L1 domain containing 1 [Source:HGNC Symbol;Acc:HGNC:24534]
RSRC2	arginine and serine rich coiled-coil 2 [Source:HGNC Symbol;Acc:HGNC:30559]
RWDD2B	RWD domain containing 2B [Source:HGNC Symbol;Acc:HGNC:1302]
RXFP1	relaxin/insulin like family peptide receptor 1 [Source:HGNC Symbol;Acc:HGNC:19718]
SAFB	scaffold attachment factor B [Source:HGNC Symbol;Acc:HGNC:10520]
SART1	squamous cell carcinoma antigen recognized by T-cells 1 [Source:HGNC Symbol;Acc:HGNC:10538]
SBK1	SH3 domain binding kinase 1 [Source:HGNC Symbol;Acc:HGNC:17699]
SDK2	sidekick cell adhesion molecule 2 [Source:HGNC Symbol;Acc:HGNC:19308]
SDR42E1	short chain dehydrogenase/reductase family 42E, member 1 [Source:HGNC Symbol;Acc:HGNC:29834]
SEC16B	SEC16 homolog B, endoplasmic reticulum export factor [Source:HGNC Symbol;Acc:HGNC:30301]
SEC23B	Sec23 homolog B, coat complex II component [Source:HGNC Symbol;Acc:HGNC:10702]

SGPL1	sphingosine-1-phosphate lyase 1 [Source:HGNC Symbol;Acc:HGNC:10817]
SIPA1L3	signal induced proliferation associated 1 like 3 [Source:HGNC Symbol;Acc:HGNC:23801]
SLAIN2	SLAIN motif family member 2 [Source:HGNC Symbol;Acc:HGNC:29282]
SLC13A2	solute carrier family 13 member 2 [Source:HGNC Symbol;Acc:HGNC:10917]
SLC17A6	solute carrier family 17 member 6 [Source:HGNC Symbol;Acc:HGNC:16703]
SLFN5	schlafen family member 5 [Source:HGNC Symbol;Acc:HGNC:28286]
SLTM	SAFB like transcription modulator [Source:HGNC Symbol;Acc:HGNC:20709]
SMCHD1	structural maintenance of chromosomes flexible hinge domain containing 1 [Source:HGNC Symbol;Acc:HGNC:29090]
SNIP1	Smad nuclear interacting protein 1 [Source:HGNC Symbol;Acc:HGNC:30587]
SNRNP27	small nuclear ribonucleoprotein U4/U6.U5 subunit 27 [Source:HGNC Symbol;Acc:HGNC:30240]
SNRNP70	small nuclear ribonucleoprotein U1 subunit 70 [Source:HGNC Symbol;Acc:HGNC:11150]
SNURF	SNRPN upstream reading frame [Source:HGNC Symbol;Acc:HGNC:11171]
SON	SON DNA binding protein [Source:HGNC Symbol;Acc:HGNC:11183]
SRSF12	serine and arginine rich splicing factor 12 [Source:HGNC Symbol;Acc:HGNC:21220]
SRSF3	serine and arginine rich splicing factor 3 [Source:HGNC Symbol;Acc:HGNC:10785]
SRSF6	serine and arginine rich splicing factor 6 [Source:HGNC Symbol;Acc:HGNC:10788]
SRSF8	serine and arginine rich splicing factor 8 [Source:HGNC Symbol;Acc:HGNC:16988]
SRSF9	serine and arginine rich splicing factor 9 [Source:HGNC Symbol;Acc:HGNC:10791]
STON1	stonin 1 [Source:HGNC Symbol;Acc:HGNC:17003]
SUPT16H	SPT16 homolog, facilitates chromatin remodeling subunit [Source:HGNC Symbol;Acc:HGNC:11465]
TCEANC2	transcription elongation factor A N-terminal and central domain containing 2 [Source:HGNC Symbol;Acc:HGNC:26494]
TJP2	tight junction protein 2 [Source:HGNC Symbol;Acc:HGNC:11828]
TLE4	transducin like enhancer of split 4 [Source:HGNC Symbol;Acc:HGNC:11840]
TMEM57	transmembrane protein 57 [Source:HGNC Symbol;Acc:HGNC:25572]
TNIP2	TNFAIP3 interacting protein 2 [Source:HGNC Symbol;Acc:HGNC:19118]
TPD52	tumor protein D52 [Source:HGNC Symbol;Acc:HGNC:12005]
TRIM41	tripartite motif containing 41 [Source:HGNC Symbol;Acc:HGNC:19013]
TRIP12	thyroid hormone receptor interactor 12 [Source:HGNC Symbol;Acc:HGNC:12306]
TUBA1B	tubulin alpha 1b [Source:HGNC Symbol;Acc:HGNC:18809]
U2AF2	U2 small nuclear RNA auxiliary factor 2 [Source:HGNC Symbol;Acc:HGNC:23156]
UBE2E2	ubiquitin conjugating enzyme E2 E2 [Source:HGNC Symbol;Acc:HGNC:12478]
UBR4	ubiquitin protein ligase E3 component n-recognin 4 [Source:HGNC Symbol;Acc:HGNC:30313]
VAV2	vav guanine nucleotide exchange factor 2 [Source:HGNC Symbol;Acc:HGNC:12658]
WDR70	WD repeat domain 70 [Source:HGNC Symbol;Acc:HGNC:25495]
XRCC5	X-ray repair cross complementing 5 [Source:HGNC Symbol;Acc:HGNC:12833]
YTHDC2	YTH domain containing 2 [Source:HGNC Symbol;Acc:HGNC:24721]

ZC3H13	zinc finger CCCH-type containing 13 [Source:HGNC Symbol;Acc:HGNC:20368]
ZC3H18	zinc finger CCCH-type containing 18 [Source:HGNC Symbol;Acc:HGNC:25091]
ZCCHC17	zinc finger CCHC-type containing 17 [Source:HGNC Symbol;Acc:HGNC:30246]
ZDHHC5	zinc finger DHHC-type containing 5 [Source:HGNC Symbol;Acc:HGNC:18472]
ZFC3H1	zinc finger C3H1-type containing [Source:HGNC Symbol;Acc:HGNC:28328]
ZFHX4	zinc finger homeobox 4 [Source:HGNC Symbol;Acc:HGNC:30939]
ZMAT4	zinc finger matrin-type 4 [Source:HGNC Symbol;Acc:HGNC:25844]
ZMYM4	zinc finger MYM-type containing 4 [Source:HGNC Symbol;Acc:HGNC:13055]
ZNF140	zinc finger protein 140 [Source:HGNC Symbol;Acc:HGNC:12925]
ZNF234	zinc finger protein 234 [Source:HGNC Symbol;Acc:HGNC:13027]
ZNF529	zinc finger protein 529 [Source:HGNC Symbol;Acc:HGNC:29328]
ZNF592	zinc finger protein 592 [Source:HGNC Symbol;Acc:HGNC:28986]
ZNF593	zinc finger protein 593 [Source:HGNC Symbol;Acc:HGNC:30943]
ZNF616	zinc finger protein 616 [Source:HGNC Symbol;Acc:HGNC:28062]
ZRSR2	zinc finger CCCH-type, RNA binding motif and serine/arginine rich 2 [Source:HGNC Symbol;Acc:HGNC:23019]
ZSCAN9	zinc finger and SCAN domain containing 9 [Source:HGNC Symbol;Acc:HGNC:12984]
COL4A5	collagen type IV alpha 5 chain [Source:HGNC Symbol;Acc:HGNC:2207]
ATXN10	ataxin 10 [Source:HGNC Symbol;Acc:HGNC:10549]
LNX1	ligand of numb-protein X 1 [Source:HGNC Symbol;Acc:HGNC:6657]
SNTA1	syntrophin alpha 1 [Source:HGNC Symbol;Acc:HGNC:11167]
SNTB1	syntrophin beta 1 [Source:HGNC Symbol;Acc:HGNC:11168]
SNTB2	syntrophin beta 2 [Source:HGNC Symbol;Acc:HGNC:11169]
MESDC2	mesoderm development candidate 2 [Source:HGNC Symbol;Acc:HGNC:13520]
SGTB	small glutamine rich tetratricopeptide repeat containing beta [Source:HGNC Symbol;Acc:HGNC:23567]
UBQLN1	ubiquilin 1 [Source:HGNC Symbol;Acc:HGNC:12508]
ZNF177	zinc finger protein 177 [Source:HGNC Symbol;Acc:HGNC:12966]
FBXW11	F-box and WD repeat domain containing 11 [Source:HGNC Symbol;Acc:HGNC:13607]
ARL13B	ADP ribosylation factor like GTPase 13B [Source:HGNC Symbol;Acc:HGNC:25419]
BCL2L13	BCL2 like 13 [Source:HGNC Symbol;Acc:HGNC:17164]
CLSTN3	calsyntenin 3 [Source:HGNC Symbol;Acc:HGNC:18371]
CPLX4	complexin 4 [Source:HGNC Symbol;Acc:HGNC:24330]
FKBP7	FK506 binding protein 7 [Source:HGNC Symbol;Acc:HGNC:3723]
GPR37L1	G protein-coupled receptor 37 like 1 [Source:HGNC Symbol;Acc:HGNC:14923]
IFNGR2	interferon gamma receptor 2 (interferon gamma transducer 1) [Source:HGNC Symbol;Acc:HGNC:5440]
LIME1	Lck interacting transmembrane adaptor 1 [Source:HGNC Symbol;Acc:HGNC:26016]
LRRC59	leucine rich repeat containing 59 [Source:HGNC Symbol;Acc:HGNC:28817]
MTIF3	mitochondrial translational initiation factor 3 [Source:HGNC Symbol;Acc:HGNC:29788]
NDUFAF1	NADH:ubiquinone oxidoreductase complex assembly factor 1 [Source:HGNC Symbol;Acc:HGNC:18828]

NDUFAF2	NADH:ubiquinone oxidoreductase complex assembly factor 2 [Source:HGNC Symbol;Acc:HGNC:28086]
PDCD1LG2	programmed cell death 1 ligand 2 [Source:HGNC Symbol;Acc:HGNC:18731]
PLP1	proteolipid protein 1 [Source:HGNC Symbol;Acc:HGNC:9086]
RHCG	Rh family C glycoprotein [Source:HGNC Symbol;Acc:HGNC:18140]
RNF170	ring finger protein 170 [Source:HGNC Symbol;Acc:HGNC:25358]
SLC14A1	solute carrier family 14 member 1 (Kidd blood group) [Source:HGNC Symbol;Acc:HGNC:10918]
TMEM14B	transmembrane protein 14B [Source:HGNC Symbol;Acc:HGNC:21384]
ADTRP	androgen dependent TFPI regulating protein [Source:HGNC Symbol;Acc:HGNC:21214]
AMPH	amphiphysin [Source:HGNC Symbol;Acc:HGNC:471]
CD247	CD247 molecule [Source:HGNC Symbol;Acc:HGNC:1677]
COPS5	COP9 signalosome subunit 5 [Source:HGNC Symbol;Acc:HGNC:2240]
CRADD	CASP2 and RIPK1 domain containing adaptor with death domain [Source:HGNC Symbol;Acc:HGNC:2340]
DLG2	discs large MAGUK scaffold protein 2 [Source:HGNC Symbol;Acc:HGNC:2901]
DLG3	discs large MAGUK scaffold protein 3 [Source:HGNC Symbol;Acc:HGNC:2902]
GLUD1	glutamate dehydrogenase 1 [Source:HGNC Symbol;Acc:HGNC:4335]
GOPC	golgi associated PDZ and coiled-coil motif containing [Source:HGNC Symbol;Acc:HGNC:17643]
LNX2	ligand of numb-protein X 2 [Source:HGNC Symbol;Acc:HGNC:20421]
PRDX4	peroxiredoxin 4 [Source:HGNC Symbol;Acc:HGNC:17169]
RHOXF1	Rhox homeobox family member 1 [Source:HGNC Symbol;Acc:HGNC:29993]
SIX1	SIX homeobox 1 [Source:HGNC Symbol;Acc:HGNC:10887]
SNTG1	syntrophin gamma 1 [Source:HGNC Symbol;Acc:HGNC:13740]
COL9A2	collagen type IX alpha 2 [Source:HGNC Symbol;Acc:HGNC:2218]
CALM2	calmodulin 2 (phosphorylase kinase, delta) [Source:HGNC Symbol;Acc:HGNC:1445]
CALM3	calmodulin 3 (phosphorylase kinase, delta) [Source:HGNC Symbol;Acc:HGNC:1449]
CCNH	cyclin H [Source:HGNC Symbol;Acc:HGNC:1594]
CTDP1	CTD phosphatase subunit 1 [Source:HGNC Symbol;Acc:HGNC:2498]
GTF2F1	general transcription factor IIF subunit 1 [Source:HGNC Symbol;Acc:HGNC:4652]
GTF2F2	general transcription factor IIF subunit 2 [Source:HGNC Symbol;Acc:HGNC:4653]
ITCH	itchy E3 ubiquitin protein ligase [Source:HGNC Symbol;Acc:HGNC:13890]
POLR2A	polymerase (RNA) II subunit A [Source:HGNC Symbol;Acc:HGNC:9187]
POLR2C	polymerase (RNA) II subunit C [Source:HGNC Symbol;Acc:HGNC:9189]
POLR2H	polymerase (RNA) II subunit H [Source:HGNC Symbol;Acc:HGNC:9195]
SUB1	SUB1 homolog, transcriptional regulator [Source:HGNC Symbol;Acc:HGNC:19985]
TCEA1	transcription elongation factor A1 [Source:HGNC Symbol;Acc:HGNC:11612]
ZSCAN1	zinc finger and SCAN domain containing 1 [Source:HGNC Symbol;Acc:HGNC:23712]
CHMP3	charged multivesicular body protein 3 [Source:HGNC Symbol;Acc:HGNC:29865]
EGFL7	EGF like domain multiple 7 [Source:HGNC Symbol;Acc:HGNC:20594]
C14orf2	chromosome 14 open reading frame 2 [Source:HGNC Symbol;Acc:HGNC:1188]

HMSD	histocompatibility minor serpin domain containing [Source:HGNC Symbol;Acc:HGNC:23037]
LETMD1	LETM1 domain containing 1 [Source:HGNC Symbol;Acc:HGNC:24241]
MPC2	mitochondrial pyruvate carrier 2 [Source:HGNC Symbol;Acc:HGNC:24515]
MTERF3	mitochondrial transcription termination factor 3 [Source:HGNC Symbol;Acc:HGNC:24258]
NFU1	NFU1 iron-sulfur cluster scaffold [Source:HGNC Symbol;Acc:HGNC:16287]
RADIL	Rap associating with DIL domain [Source:HGNC Symbol;Acc:HGNC:22226]
SIRPB1	signal regulatory protein beta 1 [Source:HGNC Symbol;Acc:HGNC:15928]
SPG21	spastic paraplegia 21 (autosomal recessive, Mast syndrome) [Source:HGNC Symbol;Acc:HGNC:20373]
DVL2	dishevelled segment polarity protein 2 [Source:HGNC Symbol;Acc:HGNC:3086]
RPS15	ribosomal protein S15 [Source:HGNC Symbol;Acc:HGNC:10388]
UMPS	uridine monophosphate synthetase [Source:HGNC Symbol;Acc:HGNC:12563]
ATXN1	ataxin 1 [Source:HGNC Symbol;Acc:HGNC:10548]
KDM1A	lysine demethylase 1A [Source:HGNC Symbol;Acc:HGNC:29079]
PIAS1	protein inhibitor of activated STAT 1 [Source:HGNC Symbol;Acc:HGNC:2752]
TLE1	transducin like enhancer of split 1 [Source:HGNC Symbol;Acc:HGNC:11837]
TLE2	transducin like enhancer of split 2 [Source:HGNC Symbol;Acc:HGNC:11838]
TPRN	taperin [Source:HGNC Symbol;Acc:HGNC:26894]
FLRT3	fibronectin leucine rich transmembrane protein 3 [Source:HGNC Symbol;Acc:HGNC:3762]
SAMM50	SAMM50 sorting and assembly machinery component [Source:HGNC Symbol;Acc:HGNC:24276]
PCOLCE	procollagen C-endopeptidase enhancer [Source:HGNC Symbol;Acc:HGNC:8738]
POU6F2	POU class 6 homeobox 2 [Source:HGNC Symbol;Acc:HGNC:21694]
ANKRD49	ankyrin repeat domain 49 [Source:HGNC Symbol;Acc:HGNC:25970]
CALCOCO2	calcium binding and coiled-coil domain 2 [Source:HGNC Symbol;Acc:HGNC:29912]
RBCK1	RANBP2-type and C3HC4-type zinc finger containing 1 [Source:HGNC Symbol;Acc:HGNC:15864]
UNC119	unc-119 lipid binding chaperone [Source:HGNC Symbol;Acc:HGNC:12565]
SEC22A	SEC22 homolog A, vesicle trafficking protein [Source:HGNC Symbol;Acc:HGNC:20260]
CDYL	chromodomain protein, Y-like [Source:HGNC Symbol;Acc:HGNC:1811]
TBP	TATA-box binding protein [Source:HGNC Symbol;Acc:HGNC:11588]
CAMLG	calcium modulating ligand [Source:HGNC Symbol;Acc:HGNC:1471]
CD79A	CD79a molecule [Source:HGNC Symbol;Acc:HGNC:1698]
PPP2R5B	protein phosphatase 2 regulatory subunit B'beta [Source:HGNC Symbol;Acc:HGNC:9310]
SGTA	small glutamine rich tetratricopeptide repeat containing alpha [Source:HGNC Symbol;Acc:HGNC:10819]
SLC13A4	solute carrier family 13 member 4 [Source:HGNC Symbol;Acc:HGNC:15827]
TMBIM6	transmembrane BAX inhibitor motif containing 6 [Source:HGNC Symbol;Acc:HGNC:11723]

TMX2	thioredoxin related transmembrane protein 2 [Source:HGNC Symbol;Acc:HGNC:30739]
NUDT3	nudix hydrolase 3 [Source:HGNC Symbol;Acc:HGNC:8050]
SNAPIN	SNAP associated protein [Source:HGNC Symbol;Acc:HGNC:17145]
SNRNP40	small nuclear ribonucleoprotein U5 subunit 40 [Source:HGNC Symbol;Acc:HGNC:30857]
ATF5	activating transcription factor 5 [Source:HGNC Symbol;Acc:HGNC:790]
CRKL	v-crk avian sarcoma virus CT10 oncogene homolog-like [Source:HGNC Symbol;Acc:HGNC:2363]
ANKRD46	ankyrin repeat domain 46 [Source:HGNC Symbol;Acc:HGNC:27229]
MGAT5B	mannosyl (alpha-1,6-)-glycoprotein beta-1,6-N-acetyl-glucosaminyltransferase, isozyme B [Source:HGNC Symbol;Acc:HGNC:24140]
SUFU	SUFU negative regulator of hedgehog signaling [Source:HGNC Symbol;Acc:HGNC:16466]
ZIC1	Zic family member 1 [Source:HGNC Symbol;Acc:HGNC:12872]
BTRC	beta-transducin repeat containing E3 ubiquitin protein ligase [Source:HGNC Symbol;Acc:HGNC:1144]
CSNK1A1	casein kinase 1 alpha 1 [Source:HGNC Symbol;Acc:HGNC:2451]
MED12	mediator complex subunit 12 [Source:HGNC Symbol;Acc:HGNC:11957]
SPOP	speckle type BTB/POZ protein [Source:HGNC Symbol;Acc:HGNC:11254]
ZIC2	Zic family member 2 [Source:HGNC Symbol;Acc:HGNC:12873]
ZIC3	Zic family member 3 [Source:HGNC Symbol;Acc:HGNC:12874]
BAG4	BCL2 associated athanogene 4 [Source:HGNC Symbol;Acc:HGNC:940]
DDIT4L	DNA damage inducible transcript 4 like [Source:HGNC Symbol;Acc:HGNC:30555]
FGFR4	fibroblast growth factor receptor 4 [Source:HGNC Symbol;Acc:HGNC:3691]
TAX1BP1	Tax1 binding protein 1 [Source:HGNC Symbol;Acc:HGNC:11575]
XRCC3	X-ray repair cross complementing 3 [Source:HGNC Symbol;Acc:HGNC:12830]
TRAPPC2L	trafficking protein particle complex 2-like [Source:HGNC Symbol;Acc:HGNC:30887]
LRP12	LDL receptor related protein 12 [Source:HGNC Symbol;Acc:HGNC:31708]
AK3	adenylate kinase 3 [Source:HGNC Symbol;Acc:HGNC:17376]
MYL12B	myosin light chain 12B [Source:HGNC Symbol;Acc:HGNC:29827]
RAD21	RAD21 cohesin complex component [Source:HGNC Symbol;Acc:HGNC:9811]
TCP10L	t-complex 10-like [Source:HGNC Symbol;Acc:HGNC:11657]
ATP5J2	ATP synthase, H+ transporting, mitochondrial Fo complex subunit F2 [Source:HGNC Symbol;Acc:HGNC:848]
DUS2	dihydrouridine synthase 2 [Source:HGNC Symbol;Acc:HGNC:26014]
MDFI	MyoD family inhibitor [Source:HGNC Symbol;Acc:HGNC:6967]
NECTIN1	nectin cell adhesion molecule 1 [Source:HGNC Symbol;Acc:HGNC:9706]
USP53	ubiquitin specific peptidase 53 [Source:HGNC Symbol;Acc:HGNC:29255]
APTX	aprataxin [Source:HGNC Symbol;Acc:HGNC:15984]
ATN1	atrophin 1 [Source:HGNC Symbol;Acc:HGNC:3033]
CCNT1	cyclin T1 [Source:HGNC Symbol;Acc:HGNC:1599]
CDK9	cyclin dependent kinase 9 [Source:HGNC Symbol;Acc:HGNC:1780]
CTDSP1	CTD small phosphatase 1 [Source:HGNC Symbol;Acc:HGNC:21614]

CTDSPL	CTD small phosphatase like [Source:HGNC Symbol;Acc:HGNC:16890]
HLA-DRA	major histocompatibility complex, class II, DR alpha [Source:HGNC Symbol;Acc:HGNC:4947]
HLA-DRB5	major histocompatibility complex, class II, DR beta 5 [Source:HGNC Symbol;Acc:HGNC:4953]
IKBKE	inhibitor of kappa light polypeptide gene enhancer in B-cells, kinase epsilon [Source:HGNC Symbol;Acc:HGNC:14552]
IRAK4	interleukin 1 receptor associated kinase 4 [Source:HGNC Symbol;Acc:HGNC:17967]
MAP3K3	mitogen-activated protein kinase kinase kinase 3 [Source:HGNC Symbol;Acc:HGNC:6855]
PKN1	protein kinase N1 [Source:HGNC Symbol;Acc:HGNC:9405]
PPP2R5C	protein phosphatase 2 regulatory subunit B'gamma [Source:HGNC Symbol;Acc:HGNC:9311]
PRKDC	protein kinase, DNA-activated, catalytic polypeptide [Source:HGNC Symbol;Acc:HGNC:9413]
PRMT5	protein arginine methyltransferase 5 [Source:HGNC Symbol;Acc:HGNC:10894]
RPS6KA5	ribosomal protein S6 kinase A5 [Source:HGNC Symbol;Acc:HGNC:10434]
STK3	serine/threonine kinase 3 [Source:HGNC Symbol;Acc:HGNC:11406]
IKZF1	IKAROS family zinc finger 1 [Source:HGNC Symbol;Acc:HGNC:13176]
TAB1	TGF-beta activated kinase 1/MAP3K7 binding protein 1 [Source:HGNC Symbol;Acc:HGNC:18157]
VPS52	VPS52, GARP complex subunit [Source:HGNC Symbol;Acc:HGNC:10518]
ASCC2	activating signal cointegrator 1 complex subunit 2 [Source:HGNC Symbol;Acc:HGNC:24103]
CEP126	centrosomal protein 126 [Source:HGNC Symbol;Acc:HGNC:29264]
COPS6	COP9 signalosome subunit 6 [Source:HGNC Symbol;Acc:HGNC:21749]
EMD	emerin [Source:HGNC Symbol;Acc:HGNC:3331]
PTPN4	protein tyrosine phosphatase, non-receptor type 4 [Source:HGNC Symbol;Acc:HGNC:9656]
UBE2Z	ubiquitin conjugating enzyme E2 Z [Source:HGNC Symbol;Acc:HGNC:25847]
SCN5A	sodium voltage-gated channel alpha subunit 5 [Source:HGNC Symbol;Acc:HGNC:10593]
SCN8A	sodium voltage-gated channel alpha subunit 8 [Source:HGNC Symbol;Acc:HGNC:10596]
FBXO25	F-box protein 25 [Source:HGNC Symbol;Acc:HGNC:13596]
C11orf68	chromosome 11 open reading frame 68 [Source:HGNC Symbol;Acc:HGNC:28801]
CSTF2	cleavage stimulation factor subunit 2 [Source:HGNC Symbol;Acc:HGNC:2484]
PTK6	protein tyrosine kinase 6 [Source:HGNC Symbol;Acc:HGNC:9617]
WDYHV1	WDYHV motif containing 1 [Source:HGNC Symbol;Acc:HGNC:25490]
ZDHHC17	zinc finger DHHC-type containing 17 [Source:HGNC Symbol;Acc:HGNC:18412]
LDB2	LIM domain binding 2 [Source:HGNC Symbol;Acc:HGNC:6533]
BRICD5	BRICHOS domain containing 5 [Source:HGNC Symbol;Acc:HGNC:28309]
MSN	moesin [Source:HGNC Symbol;Acc:HGNC:7373]
NINJ2	ninjurin 2 [Source:HGNC Symbol;Acc:HGNC:7825]

SFTPC	surfactant protein C [Source:HGNC Symbol;Acc:HGNC:10802]
SMCO4	single-pass membrane protein with coiled-coil domains 4 [Source:HGNC Symbol;Acc:HGNC:24810]
SMIM1	small integral membrane protein 1 (Vel blood group) [Source:HGNC Symbol;Acc:HGNC:44204]
CACNA1A	calcium voltage-gated channel subunit alpha1 A [Source:HGNC Symbol;Acc:HGNC:1388]
ZNF441	zinc finger protein 441 [Source:HGNC Symbol;Acc:HGNC:20875]
EXOSC5	exosome component 5 [Source:HGNC Symbol;Acc:HGNC:24662]
LSM7	LSM7 homolog, U6 small nuclear RNA and mRNA degradation associated [Source:HGNC Symbol;Acc:HGNC:20470]
PLK3	polo like kinase 3 [Source:HGNC Symbol;Acc:HGNC:2154]
PCBD2	pterin-4 alpha-carbinolamine dehydratase 2 [Source:HGNC Symbol;Acc:HGNC:24474]
SUOX	sulfite oxidase [Source:HGNC Symbol;Acc:HGNC:11460]
TSNAX	translin associated factor X [Source:HGNC Symbol;Acc:HGNC:12380]
ZGPAT	zinc finger CCCH-type and G-patch domain containing [Source:HGNC Symbol;Acc:HGNC:15948]
APOL2	apolipoprotein L2 [Source:HGNC Symbol;Acc:HGNC:619]
RAB27A	RAB27A, member RAS oncogene family [Source:HGNC Symbol;Acc:HGNC:9766]
CIB1	calcium and integrin binding 1 [Source:HGNC Symbol;Acc:HGNC:16920]
TRIP6	thyroid hormone receptor interactor 6 [Source:HGNC Symbol;Acc:HGNC:12311]
ABI2	abl interactor 2 [Source:HGNC Symbol;Acc:HGNC:24011]
APC	adenomatous polyposis coli [Source:HGNC Symbol;Acc:HGNC:583]
ARFIP2	ADP ribosylation factor interacting protein 2 [Source:HGNC Symbol;Acc:HGNC:17160]
ARL6IP1	ADP ribosylation factor like GTPase 6 interacting protein 1 [Source:HGNC Symbol;Acc:HGNC:697]
ARMC8	armadillo repeat containing 8 [Source:HGNC Symbol;Acc:HGNC:24999]
ATXN1L	ataxin 1 like [Source:HGNC Symbol;Acc:HGNC:33279]
BEGAIN	brain enriched guanylate kinase associated [Source:HGNC Symbol;Acc:HGNC:24163]
CCDC103	coiled-coil domain containing 103 [Source:HGNC Symbol;Acc:HGNC:32700]
CEP57L1	centrosomal protein 57 like 1 [Source:HGNC Symbol;Acc:HGNC:21561]
CRMP1	collapsin response mediator protein 1 [Source:HGNC Symbol;Acc:HGNC:2365]
DCTN2	dynactin subunit 2 [Source:HGNC Symbol;Acc:HGNC:2712]
DGCR6	DiGeorge syndrome critical region gene 6 [Source:HGNC Symbol;Acc:HGNC:2846]
ESRRG	estrogen related receptor gamma [Source:HGNC Symbol;Acc:HGNC:3474]
EXOC7	exocyst complex component 7 [Source:HGNC Symbol;Acc:HGNC:23214]
EXOC8	exocyst complex component 8 [Source:HGNC Symbol;Acc:HGNC:24659]
GGA2	golgi associated, gamma adaptin ear containing, ARF binding protein 2 [Source:HGNC Symbol;Acc:HGNC:16064]
GGA3	golgi associated, gamma adaptin ear containing, ARF binding protein 3 [Source:HGNC Symbol;Acc:HGNC:17079]
GKAP1	G kinase anchoring protein 1 [Source:HGNC Symbol;Acc:HGNC:17496]
IL2RB	interleukin 2 receptor subunit beta [Source:HGNC Symbol;Acc:HGNC:6009]

IL4R	interleukin 4 receptor [Source:HGNC Symbol;Acc:HGNC:6015]
ILKAP	ILK associated serine/threonine phosphatase [Source:HGNC Symbol;Acc:HGNC:15566]
ING5	inhibitor of growth family member 5 [Source:HGNC Symbol;Acc:HGNC:19421]
JAKMIP2	janus kinase and microtubule interacting protein 2 [Source:HGNC Symbol;Acc:HGNC:29067]
LDOC1	leucine zipper, down-regulated in cancer 1 [Source:HGNC Symbol;Acc:HGNC:6548]
LURAP1	leucine rich adaptor protein 1 [Source:HGNC Symbol;Acc:HGNC:32327]
LYST	lysosomal trafficking regulator [Source:HGNC Symbol;Acc:HGNC:1968]
MAP3K10	mitogen-activated protein kinase kinase kinase 10 [Source:HGNC Symbol;Acc:HGNC:6849]
MAPK1IP1L	mitogen-activated protein kinase 1 interacting protein 1-like [Source:HGNC Symbol;Acc:HGNC:19840]
MED7	mediator complex subunit 7 [Source:HGNC Symbol;Acc:HGNC:2378]
MIF4GD	MIF4G domain containing [Source:HGNC Symbol;Acc:HGNC:24030]
MKNK1	MAP kinase interacting serine/threonine kinase 1 [Source:HGNC Symbol;Acc:HGNC:7110]
MRFAP1L1	Morf4 family associated protein 1 like 1 [Source:HGNC Symbol;Acc:HGNC:28796]
NF2	neurofibromin 2 (merlin) [Source:HGNC Symbol;Acc:HGNC:7773]
NMI	N-myc and STAT interactor [Source:HGNC Symbol;Acc:HGNC:7854]
NUP54	nucleoporin 54 [Source:HGNC Symbol;Acc:HGNC:17359]
OSBPL5	oxysterol binding protein like 5 [Source:HGNC Symbol;Acc:HGNC:16392]
P4HA3	prolyl 4-hydroxylase subunit alpha 3 [Source:HGNC Symbol;Acc:HGNC:30135]
PEF1	penta-EF-hand domain containing 1 [Source:HGNC Symbol;Acc:HGNC:30009]
PITX1	paired like homeodomain 1 [Source:HGNC Symbol;Acc:HGNC:9004]
POGZ	pogo transposable element with ZNF domain [Source:HGNC Symbol;Acc:HGNC:18801]
PPP1R16A	protein phosphatase 1 regulatory subunit 16A [Source:HGNC Symbol;Acc:HGNC:14941]
PPP1R7	protein phosphatase 1 regulatory subunit 7 [Source:HGNC Symbol;Acc:HGNC:9295]
PTCD3	pentatricopeptide repeat domain 3 [Source:HGNC Symbol;Acc:HGNC:24717]
RHOBTB3	Rho related BTB domain containing 3 [Source:HGNC Symbol;Acc:HGNC:18757]
RSU1	Ras suppressor protein 1 [Source:HGNC Symbol;Acc:HGNC:10464]
SNX1	sorting nexin 1 [Source:HGNC Symbol;Acc:HGNC:11172]
SNX7	sorting nexin 7 [Source:HGNC Symbol;Acc:HGNC:14971]
STAM	signal transducing adaptor molecule [Source:HGNC Symbol;Acc:HGNC:11357]
STAM2	signal transducing adaptor molecule 2 [Source:HGNC Symbol;Acc:HGNC:11358]
STXBP1	syntaxin binding protein 1 [Source:HGNC Symbol;Acc:HGNC:11444]
SUN2	Sad1 and UNC84 domain containing 2 [Source:HGNC Symbol;Acc:HGNC:14210]
TADA2A	transcriptional adaptor 2A [Source:HGNC Symbol;Acc:HGNC:11531]
TIMMDC1	translocase of inner mitochondrial membrane domain containing 1 [Source:HGNC Symbol;Acc:HGNC:1321]
TMEM189	transmembrane protein 189 [Source:HGNC Symbol;Acc:HGNC:16735]
TOM1L1	target of myb1 like 1 membrane trafficking protein [Source:HGNC Symbol;Acc:HGNC:11983]

UBA1	ubiquitin like modifier activating enzyme 1 [Source:HGNC Symbol;Acc:HGNC:12469]
UBE4B	ubiquitination factor E4B [Source:HGNC Symbol;Acc:HGNC:12500]
USHBP1	USH1 protein network component harmonin binding protein 1 [Source:HGNC Symbol;Acc:HGNC:24058]
VPS37C	VPS37C, ESCRT-I subunit [Source:HGNC Symbol;Acc:HGNC:26097]
ZNF302	zinc finger protein 302 [Source:HGNC Symbol;Acc:HGNC:13848]
BTNL9	butyrophilin like 9 [Source:HGNC Symbol;Acc:HGNC:24176]
CCDC107	coiled-coil domain containing 107 [Source:HGNC Symbol;Acc:HGNC:28465]
CCDC155	coiled-coil domain containing 155 [Source:HGNC Symbol;Acc:HGNC:26520]
CLEC10A	C-type lectin domain family 10 member A [Source:HGNC Symbol;Acc:HGNC:16916]
CLEC3B	C-type lectin domain family 3 member B [Source:HGNC Symbol;Acc:HGNC:11891]
FNDC9	fibronectin type III domain containing 9 [Source:HGNC Symbol;Acc:HGNC:33547]
LYN	LYN proto-oncogene, Src family tyrosine kinase [Source:HGNC Symbol;Acc:HGNC:6735]
MRM3	mitochondrial rRNA methyltransferase 3 [Source:HGNC Symbol;Acc:HGNC:18485]
PANX1	pannexin 1 [Source:HGNC Symbol;Acc:HGNC:8599]
PLEKHO1	pleckstrin homology domain containing O1 [Source:HGNC Symbol;Acc:HGNC:24310]
POU2F1	POU class 2 homeobox 1 [Source:HGNC Symbol;Acc:HGNC:9212]
RC3H1	ring finger and CCCH-type domains 1 [Source:HGNC Symbol;Acc:HGNC:29434]
STX1A	syntaxin 1A [Source:HGNC Symbol;Acc:HGNC:11433]
ZCCHC12	zinc finger CCHC-type containing 12 [Source:HGNC Symbol;Acc:HGNC:27273]
PAAF1	proteasomal ATPase associated factor 1 [Source:HGNC Symbol;Acc:HGNC:25687]
ZNF655	zinc finger protein 655 [Source:HGNC Symbol;Acc:HGNC:30899]
CSTB	cystatin B [Source:HGNC Symbol;Acc:HGNC:2482]
RNF115	ring finger protein 115 [Source:HGNC Symbol;Acc:HGNC:18154]
ERCC5	ERCC excision repair 5, endonuclease [Source:HGNC Symbol;Acc:HGNC:3437]
ERCC8	ERCC excision repair 8, CSA ubiquitin ligase complex subunit [Source:HGNC Symbol;Acc:HGNC:3439]
RNF11	ring finger protein 11 [Source:HGNC Symbol;Acc:HGNC:10056]
RNF216	ring finger protein 216 [Source:HGNC Symbol;Acc:HGNC:21698]
TRIL	TLR4 interactor with leucine rich repeats [Source:HGNC Symbol;Acc:HGNC:22200]
PAXIP1	PAX interacting protein 1 [Source:HGNC Symbol;Acc:HGNC:8624]
GPS2	G protein pathway suppressor 2 [Source:HGNC Symbol;Acc:HGNC:4550]
MAP1LC3C	microtubule associated protein 1 light chain 3 gamma [Source:HGNC Symbol;Acc:HGNC:13353]
UBAC1	UBA domain containing 1 [Source:HGNC Symbol;Acc:HGNC:30221]
UBE2W	ubiquitin conjugating enzyme E2 W (putative) [Source:HGNC Symbol;Acc:HGNC:25616]
TMED7	transmembrane p24 trafficking protein 7 [Source:HGNC Symbol;Acc:HGNC:24253]
TOLLIP	toll interacting protein [Source:HGNC Symbol;Acc:HGNC:16476]
DAB1	DAB1, reelin adaptor protein [Source:HGNC Symbol;Acc:HGNC:2661]
SNX17	sorting nexin 17 [Source:HGNC Symbol;Acc:HGNC:14979]
ZSCAN12	zinc finger and SCAN domain containing 12 [Source:HGNC Symbol;Acc:HGNC:13172]

CYP4F12	cytochrome P450 family 4 subfamily F member 12 [Source:HGNC Symbol;Acc:HGNC:18857]
NUP85	nucleoporin 85 [Source:HGNC Symbol;Acc:HGNC:8734]
COL9A1	collagen type IX alpha 1 [Source:HGNC Symbol;Acc:HGNC:2217]
COL9A3	collagen type IX alpha 3 [Source:HGNC Symbol;Acc:HGNC:2219]
EIF3F	eukaryotic translation initiation factor 3 subunit F [Source:HGNC Symbol;Acc:HGNC:3275]
KCNIP4	potassium voltage-gated channel interacting protein 4 [Source:HGNC Symbol;Acc:HGNC:30083]
LIG4	DNA ligase 4 [Source:HGNC Symbol;Acc:HGNC:6601]
ATP5H	ATP synthase, H+ transporting, mitochondrial Fo complex subunit D [Source:HGNC Symbol;Acc:HGNC:845]
ATP6V0C	ATPase H+ transporting V0 subunit c [Source:HGNC Symbol;Acc:HGNC:855]
ATP6V0E1	ATPase H+ transporting V0 subunit e1 [Source:HGNC Symbol;Acc:HGNC:863]
ATP6V1B2	ATPase H+ transporting V1 subunit B2 [Source:HGNC Symbol;Acc:HGNC:854]
DDB1	damage specific DNA binding protein 1 [Source:HGNC Symbol;Acc:HGNC:2717]
PRDX5	peroxiredoxin 5 [Source:HGNC Symbol;Acc:HGNC:9355]
CTSC	cathepsin C [Source:HGNC Symbol;Acc:HGNC:2528]
ATP5F1	ATP synthase, H+ transporting, mitochondrial Fo complex subunit B1 [Source:HGNC Symbol;Acc:HGNC:840]
ATP5J	ATP synthase, H+ transporting, mitochondrial Fo complex subunit F6 [Source:HGNC Symbol;Acc:HGNC:847]
ATP6AP1	ATPase H+ transporting accessory protein 1 [Source:HGNC Symbol;Acc:HGNC:868]
ATP6V0A1	ATPase H+ transporting V0 subunit a1 [Source:HGNC Symbol;Acc:HGNC:865]
ATP6V1E1	ATPase H+ transporting V1 subunit E1 [Source:HGNC Symbol;Acc:HGNC:857]
COX14	COX14 cytochrome c oxidase assembly factor [Source:HGNC Symbol;Acc:HGNC:28216]
COX7A2L	cytochrome c oxidase subunit 7A2 like [Source:HGNC Symbol;Acc:HGNC:2289]
FBXO7	F-box protein 7 [Source:HGNC Symbol;Acc:HGNC:13586]
GPI	glucose-6-phosphate isomerase [Source:HGNC Symbol;Acc:HGNC:4458]
PFKL	phosphofructokinase, liver type [Source:HGNC Symbol;Acc:HGNC:8876]
RHOT2	ras homolog family member T2 [Source:HGNC Symbol;Acc:HGNC:21169]
SCARB2	scavenger receptor class B member 2 [Source:HGNC Symbol;Acc:HGNC:1665]
UQCC2	ubiquinol-cytochrome c reductase complex assembly factor 2 [Source:HGNC Symbol;Acc:HGNC:21237]
XRCC6	X-ray repair cross complementing 6 [Source:HGNC Symbol;Acc:HGNC:4055]
ATP6V0E2	ATPase H+ transporting V0 subunit e2 [Source:HGNC Symbol;Acc:HGNC:21723]
ATP6V1G1	ATPase H+ transporting V1 subunit G1 [Source:HGNC Symbol;Acc:HGNC:864]
CTSF	cathepsin F [Source:HGNC Symbol;Acc:HGNC:2531]
LAMB1	laminin subunit beta 1 [Source:HGNC Symbol;Acc:HGNC:6486]
PRELP	proline and arginine rich end leucine rich repeat protein [Source:HGNC Symbol;Acc:HGNC:9357]
TRAK1	trafficking kinesin protein 1 [Source:HGNC Symbol;Acc:HGNC:29947]
ULK3	unc-51 like kinase 3 [Source:HGNC Symbol;Acc:HGNC:19703]

ARF3	ADP ribosylation factor 3 [Source:HGNC Symbol;Acc:HGNC:654]
ATP6V1A	ATPase H+ transporting V1 subunit A [Source:HGNC Symbol;Acc:HGNC:851]
BAG5	BCL2 associated athanogene 5 [Source:HGNC Symbol;Acc:HGNC:941]
CTSA	cathepsin A [Source:HGNC Symbol;Acc:HGNC:9251]
IFNGR1	interferon gamma receptor 1 [Source:HGNC Symbol;Acc:HGNC:5439]
LAMB2	laminin subunit beta 2 [Source:HGNC Symbol;Acc:HGNC:6487]
MPC1	mitochondrial pyruvate carrier 1 [Source:HGNC Symbol;Acc:HGNC:21606]
OGN	osteoglycin [Source:HGNC Symbol;Acc:HGNC:8126]
P2RX5	purinergic receptor P2X 5 [Source:HGNC Symbol;Acc:HGNC:8536]
PIN1	peptidylprolyl cis/trans isomerase, NIMA-interacting 1 [Source:HGNC Symbol;Acc:HGNC:8988]
SEC13	SEC13 homolog, nuclear pore and COPII coat complex component [Source:HGNC Symbol;Acc:HGNC:10697]
UQCRFS1	ubiquinol-cytochrome c reductase, Rieske iron-sulfur polypeptide 1 [Source:HGNC Symbol;Acc:HGNC:12587]
UQCRQ	ubiquinol-cytochrome c reductase complex III subunit VII [Source:HGNC Symbol;Acc:HGNC:29594]
SCO2	SCO2 cytochrome c oxidase assembly protein [Source:HGNC Symbol;Acc:HGNC:10604]
VPS26A	VPS26, retromer complex component A [Source:HGNC Symbol;Acc:HGNC:12711]
UQCC1	ubiquinol-cytochrome c reductase complex assembly factor 1 [Source:HGNC Symbol;Acc:HGNC:15891]
ITGA8	integrin subunit alpha 8 [Source:HGNC Symbol;Acc:HGNC:6144]
ATP6V1C1	ATPase H+ transporting V1 subunit C1 [Source:HGNC Symbol;Acc:HGNC:856]
ACP2	acid phosphatase 2, lysosomal [Source:HGNC Symbol;Acc:HGNC:123]
AKAP1	A-kinase anchoring protein 1 [Source:HGNC Symbol;Acc:HGNC:367]
APOL1	apolipoprotein L1 [Source:HGNC Symbol;Acc:HGNC:618]
ARF6	ADP ribosylation factor 6 [Source:HGNC Symbol;Acc:HGNC:659]
ATP5SL	ATP5S like [Source:HGNC Symbol;Acc:HGNC:25496]
ATP6V1D	ATPase H+ transporting V1 subunit D [Source:HGNC Symbol;Acc:HGNC:13527]
ATP6V1H	ATPase H+ transporting V1 subunit H [Source:HGNC Symbol;Acc:HGNC:18303]
CAPN1	calpain 1 [Source:HGNC Symbol;Acc:HGNC:1476]
CLPP	caseinolytic mitochondrial matrix peptidase proteolytic subunit [Source:HGNC Symbol;Acc:HGNC:2084]
COA1	cytochrome c oxidase assembly factor 1 homolog [Source:HGNC Symbol;Acc:HGNC:21868]
COA3	cytochrome c oxidase assembly factor 3 [Source:HGNC Symbol;Acc:HGNC:24990]
COA4	cytochrome c oxidase assembly factor 4 homolog [Source:HGNC Symbol;Acc:HGNC:24604]
COA5	cytochrome c oxidase assembly factor 5 [Source:HGNC Symbol;Acc:HGNC:33848]
COA6	cytochrome c oxidase assembly factor 6 [Source:HGNC Symbol;Acc:HGNC:18025]
COL5A3	collagen type V alpha 3 [Source:HGNC Symbol;Acc:HGNC:14864]
COX16	COX16 cytochrome c oxidase assembly homolog [Source:HGNC Symbol;Acc:HGNC:20213]

CUL1	cullin 1 [Source:HGNC Symbol;Acc:HGNC:2551]
CUL4A	cullin 4A [Source:HGNC Symbol;Acc:HGNC:2554]
DBNL	drebrin like [Source:HGNC Symbol;Acc:HGNC:2696]
EMILIN1	elastin microfibril interfacer 1 [Source:HGNC Symbol;Acc:HGNC:19880]
FABP12	fatty acid binding protein 12 [Source:HGNC Symbol;Acc:HGNC:34524]
FH	fumarate hydratase [Source:HGNC Symbol;Acc:HGNC:3700]
HKDC1	hexokinase domain containing 1 [Source:HGNC Symbol;Acc:HGNC:23302]
IDH3G	isocitrate dehydrogenase 3 (NAD(+)) gamma [Source:HGNC Symbol;Acc:HGNC:5386]
KAT2A	lysine acetyltransferase 2A [Source:HGNC Symbol;Acc:HGNC:4201]
KIF5B	kinesin family member 5B [Source:HGNC Symbol;Acc:HGNC:6324]
LAMA2	laminin subunit alpha 2 [Source:HGNC Symbol;Acc:HGNC:6482]
MAPK8IP3	mitogen-activated protein kinase 8 interacting protein 3 [Source:HGNC Symbol;Acc:HGNC:6884]
MCUR1	mitochondrial calcium uniporter regulator 1 [Source:HGNC Symbol;Acc:HGNC:21097]
MEF2D	myocyte enhancer factor 2D [Source:HGNC Symbol;Acc:HGNC:6997]
MLST8	MTOR associated protein, LST8 homolog [Source:HGNC Symbol;Acc:HGNC:24825]
MPI	mannose phosphate isomerase [Source:HGNC Symbol;Acc:HGNC:7216]
NDUFA10	NADH:ubiquinone oxidoreductase subunit A10 [Source:HGNC Symbol;Acc:HGNC:7684]
NDUFA9	NADH:ubiquinone oxidoreductase subunit A9 [Source:HGNC Symbol;Acc:HGNC:7693]
NMT1	N-myristoyltransferase 1 [Source:HGNC Symbol;Acc:HGNC:7857]
NR1H2	nuclear receptor subfamily 1 group H member 2 [Source:HGNC Symbol;Acc:HGNC:7965]
PARL	presenilin associated rhomboid like [Source:HGNC Symbol;Acc:HGNC:18253]
PDGFD	platelet derived growth factor D [Source:HGNC Symbol;Acc:HGNC:30620]
PDK2	pyruvate dehydrogenase kinase 2 [Source:HGNC Symbol;Acc:HGNC:8810]
PIP4K2A	phosphatidylinositol-5-phosphate 4-kinase type 2 alpha [Source:HGNC Symbol;Acc:HGNC:8997]
PIP4K2B	phosphatidylinositol-5-phosphate 4-kinase type 2 beta [Source:HGNC Symbol;Acc:HGNC:8998]
PIP5K1A	phosphatidylinositol-4-phosphate 5-kinase type 1 alpha [Source:HGNC Symbol;Acc:HGNC:8994]
PIP5K1C	phosphatidylinositol-4-phosphate 5-kinase type 1 gamma [Source:HGNC Symbol;Acc:HGNC:8996]
PPP5C	protein phosphatase 5 catalytic subunit [Source:HGNC Symbol;Acc:HGNC:9322]
PUM1	pumilio RNA binding family member 1 [Source:HGNC Symbol;Acc:HGNC:14957]
RCC2	regulator of chromosome condensation 2 [Source:HGNC Symbol;Acc:HGNC:30297]
RDX	radixin [Source:HGNC Symbol;Acc:HGNC:9944]
RXRG	retinoid X receptor gamma [Source:HGNC Symbol;Acc:HGNC:10479]
S100A12	S100 calcium binding protein A12 [Source:HGNC Symbol;Acc:HGNC:10489]
SLC25A1	solute carrier family 25 member 1 [Source:HGNC Symbol;Acc:HGNC:10979]
SLC25A12	solute carrier family 25 member 12 [Source:HGNC Symbol;Acc:HGNC:10982]
SLC25A23	solute carrier family 25 member 23 [Source:HGNC Symbol;Acc:HGNC:19375]

TIMM17B	translocase of inner mitochondrial membrane 17 homolog B (yeast) [Source:HGNC Symbol;Acc:HGNC:17310]
TPPP	tubulin polymerization promoting protein [Source:HGNC Symbol;Acc:HGNC:24164]
VPS11	VPS11, CORVET/HOPS core subunit [Source:HGNC Symbol;Acc:HGNC:14583]
VPS26B	VPS26, retromer complex component B [Source:HGNC Symbol;Acc:HGNC:28119]
YME1L1	YME1 like 1 ATPase [Source:HGNC Symbol;Acc:HGNC:12843]
YY1	YY1 transcription factor [Source:HGNC Symbol;Acc:HGNC:12856]
IRF5	interferon regulatory factor 5 [Source:HGNC Symbol;Acc:HGNC:6120]
NFKBIE	NFKB inhibitor epsilon [Source:HGNC Symbol;Acc:HGNC:7799]
ACP7	acid phosphatase 7, tartrate resistant (putative) [Source:HGNC Symbol;Acc:HGNC:33781]
NXPH1	neurexophilin 1 [Source:HGNC Symbol;Acc:HGNC:20693]
HEXA	hexosaminidase subunit alpha [Source:HGNC Symbol;Acc:HGNC:4878]
APOF	apolipoprotein F [Source:HGNC Symbol;Acc:HGNC:615]
ATG10	autophagy related 10 [Source:HGNC Symbol;Acc:HGNC:20315]
ATP5L2	ATP synthase, H+ transporting, mitochondrial Fo complex subunit G2 [Source:HGNC Symbol;Acc:HGNC:13213]
ATP5S	ATP synthase, H+ transporting, mitochondrial Fo complex subunit s (factor B) [Source:HGNC Symbol;Acc:HGNC:18799]
ATP6AP1L	ATPase H+ transporting accessory protein 1 like [Source:HGNC Symbol;Acc:HGNC:28091]
ATP6V0A2	ATPase H+ transporting V0 subunit a2 [Source:HGNC Symbol;Acc:HGNC:18481]
ATP6V0A4	ATPase H+ transporting V0 subunit a4 [Source:HGNC Symbol;Acc:HGNC:866]
ATP6V0D2	ATPase H+ transporting V0 subunit d2 [Source:HGNC Symbol;Acc:HGNC:18266]
ATP6V1E2	ATPase H+ transporting V1 subunit E2 [Source:HGNC Symbol;Acc:HGNC:18125]
ATP6V1G3	ATPase H+ transporting V1 subunit G3 [Source:HGNC Symbol;Acc:HGNC:18265]
C1orf56	chromosome 1 open reading frame 56 [Source:HGNC Symbol;Acc:HGNC:26045]
C6	complement component 6 [Source:HGNC Symbol;Acc:HGNC:1339]
C8A	complement component 8 alpha subunit [Source:HGNC Symbol;Acc:HGNC:1352]
CAMKK2	calcium/calmodulin dependent protein kinase kinase 2 [Source:HGNC Symbol;Acc:HGNC:1470]
CLPB	ClpB homolog, mitochondrial AAA ATPase chaperonin [Source:HGNC Symbol;Acc:HGNC:30664]
COA7	cytochrome c oxidase assembly factor 7 (putative) [Source:HGNC Symbol;Acc:HGNC:25716]
COL6A5	collagen type VI alpha 5 [Source:HGNC Symbol;Acc:HGNC:26674]
COL6A6	collagen type VI alpha 6 [Source:HGNC Symbol;Acc:HGNC:27023]
COX18	COX18 cytochrome c oxidase assembly factor [Source:HGNC Symbol;Acc:HGNC:26801]
COX19	COX19 cytochrome c oxidase assembly factor [Source:HGNC Symbol;Acc:HGNC:28074]
COX20	COX20 cytochrome c oxidase assembly factor [Source:HGNC Symbol;Acc:HGNC:26970]
COX6A2	cytochrome c oxidase subunit 6A2 [Source:HGNC Symbol;Acc:HGNC:2279]
COX7B2	cytochrome c oxidase subunit 7B2 [Source:HGNC Symbol;Acc:HGNC:24381]
COX8C	cytochrome c oxidase subunit 8C [Source:HGNC Symbol;Acc:HGNC:24382]

DEPDC5	DEP domain containing 5 [Source:HGNC Symbol;Acc:HGNC:18423]
DIS3L2	DIS3 like 3'-5' exoribonuclease 2 [Source:HGNC Symbol;Acc:HGNC:28648]
DUOX2	dual oxidase 2 [Source:HGNC Symbol;Acc:HGNC:13273]
FABP9	fatty acid binding protein 9 [Source:HGNC Symbol;Acc:HGNC:3563]
FCAR	Fc fragment of IgA receptor [Source:HGNC Symbol;Acc:HGNC:3608]
FCER2	Fc fragment of IgE receptor II [Source:HGNC Symbol;Acc:HGNC:3612]
FCGR1B	Fc fragment of IgG receptor Ib [Source:HGNC Symbol;Acc:HGNC:3614]
GAS2	growth arrest specific 2 [Source:HGNC Symbol;Acc:HGNC:4167]
GPIHBP1	glycosylphosphatidylinositol anchored high density lipoprotein binding protein 1 [Source:HGNC Symbol;Acc:HGNC:24945]
HAPLN1	hyaluronan and proteoglycan link protein 1 [Source:HGNC Symbol;Acc:HGNC:2380]
INHA	inhibin alpha subunit [Source:HGNC Symbol;Acc:HGNC:6065]
INSRR	insulin receptor related receptor [Source:HGNC Symbol;Acc:HGNC:6093]
ITGA11	integrin subunit alpha 11 [Source:HGNC Symbol;Acc:HGNC:6136]
ITGA4	integrin subunit alpha 4 [Source:HGNC Symbol;Acc:HGNC:6140]
ITGA9	integrin subunit alpha 9 [Source:HGNC Symbol;Acc:HGNC:6145]
LAMB4	laminin subunit beta 4 [Source:HGNC Symbol;Acc:HGNC:6491]
LDHAL6A	lactate dehydrogenase A like 6A [Source:HGNC Symbol;Acc:HGNC:28335]
LDHAL6B	lactate dehydrogenase A like 6B [Source:HGNC Symbol;Acc:HGNC:21481]
LNPEP	leucyl/cystinyl aminopeptidase [Source:HGNC Symbol;Acc:HGNC:6656]
MAS1	MAS1 proto-oncogene, G protein-coupled receptor [Source:HGNC Symbol;Acc:HGNC:6899]
MMP24	matrix metallopeptidase 24 [Source:HGNC Symbol;Acc:HGNC:7172]
MMP25	matrix metallopeptidase 25 [Source:HGNC Symbol;Acc:HGNC:14246]
MPC1L	mitochondrial pyruvate carrier 1-like [Source:HGNC Symbol;Acc:HGNC:44205]
MT-CO1	mitochondrially encoded cytochrome c oxidase I [Source:HGNC Symbol;Acc:HGNC:7419]
MT-CO2	mitochondrially encoded cytochrome c oxidase II [Source:HGNC Symbol;Acc:HGNC:7421]
MT-ND1	mitochondrially encoded NADH:ubiquinone oxidoreductase core subunit 1 [Source:HGNC Symbol;Acc:HGNC:7455]
MT-ND3	mitochondrially encoded NADH:ubiquinone oxidoreductase core subunit 3 [Source:HGNC Symbol;Acc:HGNC:7458]
MT-ND4L	mitochondrially encoded NADH:ubiquinone oxidoreductase core subunit 4L [Source:HGNC Symbol;Acc:HGNC:7460]
MT-ND5	mitochondrially encoded NADH:ubiquinone oxidoreductase core subunit 5 [Source:HGNC Symbol;Acc:HGNC:7461]
MT-ND6	mitochondrially encoded NADH:ubiquinone oxidoreductase core subunit 6 [Source:HGNC Symbol;Acc:HGNC:7462]
NEK1	NIMA related kinase 1 [Source:HGNC Symbol;Acc:HGNC:7744]
NFKBID	NFKB inhibitor delta [Source:HGNC Symbol;Acc:HGNC:15671]
NOX5	NADPH oxidase, EF-hand calcium binding domain 5 [Source:HGNC Symbol;Acc:HGNC:14874]
P2RX1	purinergic receptor P2X 1 [Source:HGNC Symbol;Acc:HGNC:8533]

P2RX2	purinergic receptor P2X 2 [Source:HGNC Symbol;Acc:HGNC:15459]
P2RX3	purinergic receptor P2X 3 [Source:HGNC Symbol;Acc:HGNC:8534]
P2RX6	purinergic receptor P2X 6 [Source:HGNC Symbol;Acc:HGNC:8538]
P2RY1	purinergic receptor P2Y1 [Source:HGNC Symbol;Acc:HGNC:8539]
P2RY10	purinergic receptor P2Y10 [Source:HGNC Symbol;Acc:HGNC:19906]
P2RY13	purinergic receptor P2Y13 [Source:HGNC Symbol;Acc:HGNC:4537]
P2RY2	purinergic receptor P2Y2 [Source:HGNC Symbol;Acc:HGNC:8541]
P2RY6	pyrimidinergic receptor P2Y6 [Source:HGNC Symbol;Acc:HGNC:8543]
PDHA2	pyruvate dehydrogenase (lipoamide) alpha 2 [Source:HGNC Symbol;Acc:HGNC:8807]
PDK3	pyruvate dehydrogenase kinase 3 [Source:HGNC Symbol;Acc:HGNC:8811]
PDP2	pyruvate dehyrogenase phosphatase catalytic subunit 2 [Source:HGNC Symbol;Acc:HGNC:30263]
PF4V1	platelet factor 4 variant 1 [Source:HGNC Symbol;Acc:HGNC:8862]
PGAM4	phosphoglycerate mutase family member 4 [Source:HGNC Symbol;Acc:HGNC:21731]
PKLR	pyruvate kinase, liver and RBC [Source:HGNC Symbol;Acc:HGNC:9020]
PPP3R2	protein phosphatase 3 regulatory subunit B, beta [Source:HGNC Symbol;Acc:HGNC:9318]
PRKACG	protein kinase cAMP-activated catalytic subunit gamma [Source:HGNC Symbol;Acc:HGNC:9382]
PRKAR1B	protein kinase cAMP-dependent type I regulatory subunit beta [Source:HGNC Symbol;Acc:HGNC:9390]
PSG11	pregnancy specific beta-1-glycoprotein 11 [Source:HGNC Symbol;Acc:HGNC:9516]
PSG9	pregnancy specific beta-1-glycoprotein 9 [Source:HGNC Symbol;Acc:HGNC:9526]
SFN	stratifin [Source:HGNC Symbol;Acc:HGNC:10773]
SLC25A6	solute carrier family 25 member 6 [Source:HGNC Symbol;Acc:HGNC:10992]
SLC38A9	solute carrier family 38 member 9 [Source:HGNC Symbol;Acc:HGNC:26907]
STAC	SH3 and cysteine rich domain [Source:HGNC Symbol;Acc:HGNC:11353]
STAC3	SH3 and cysteine rich domain 3 [Source:HGNC Symbol;Acc:HGNC:28423]
TNN	tenascin N [Source:HGNC Symbol;Acc:HGNC:22942]
TNR	tenascin R [Source:HGNC Symbol;Acc:HGNC:11953]
ULK4	unc-51 like kinase 4 [Source:HGNC Symbol;Acc:HGNC:15784]
UQCRHL	ubiquinol-cytochrome c reductase hinge protein like [Source:HGNC Symbol;Acc:HGNC:51714]
WDR24	WD repeat domain 24 [Source:HGNC Symbol;Acc:HGNC:20852]
PGM1	phosphoglucomutase 1 [Source:HGNC Symbol;Acc:HGNC:8905]
NFKBIL1	NFKB inhibitor like 1 [Source:HGNC Symbol;Acc:HGNC:7800]
SDHAF4	succinate dehydrogenase complex assembly factor 4 [Source:HGNC Symbol;Acc:HGNC:20957]
NPRL2	NPR2-like, GATOR1 complex subunit [Source:HGNC Symbol;Acc:HGNC:24969]
MAPK10	mitogen-activated protein kinase 10 [Source:HGNC Symbol;Acc:HGNC:6872]
SDHAF3	succinate dehydrogenase complex assembly factor 3 [Source:HGNC Symbol;Acc:HGNC:21752]

PHLPP1	PH domain and leucine rich repeat protein phosphatase 1 [Source:HGNC Symbol;Acc:HGNC:20610]
PLXNA1	plexin A1 [Source:HGNC Symbol;Acc:HGNC:9099]
ATG16L1	autophagy related 16 like 1 [Source:HGNC Symbol;Acc:HGNC:21498]
BCAN	brevican [Source:HGNC Symbol;Acc:HGNC:23059]
BPGM	bisphosphoglycerate mutase [Source:HGNC Symbol;Acc:HGNC:1093]
C7	complement component 7 [Source:HGNC Symbol;Acc:HGNC:1346]
CHCHD4	coiled-coil-helix-coiled-coil-helix domain containing 4 [Source:HGNC Symbol;Acc:HGNC:26467]
CSPG4	chondroitin sulfate proteoglycan 4 [Source:HGNC Symbol;Acc:HGNC:2466]
DIS3L	DIS3 like exosome 3'-5' exoribonuclease [Source:HGNC Symbol;Acc:HGNC:28698]
EXOSC3	exosome component 3 [Source:HGNC Symbol;Acc:HGNC:17944]
EXOSC8	exosome component 8 [Source:HGNC Symbol;Acc:HGNC:17035]
EXOSC9	exosome component 9 [Source:HGNC Symbol;Acc:HGNC:9137]
FCGBP	Fc fragment of IgG binding protein [Source:HGNC Symbol;Acc:HGNC:13572]
GALM	galactose mutarotase [Source:HGNC Symbol;Acc:HGNC:24063]
HAPLN4	hyaluronan and proteoglycan link protein 4 [Source:HGNC Symbol;Acc:HGNC:31357]
IRF2	interferon regulatory factor 2 [Source:HGNC Symbol;Acc:HGNC:6117]
ITGA10	integrin subunit alpha 10 [Source:HGNC Symbol;Acc:HGNC:6135]
KCNJ8	potassium voltage-gated channel subfamily J member 8 [Source:HGNC Symbol;Acc:HGNC:6269]
LDHD	lactate dehydrogenase D [Source:HGNC Symbol;Acc:HGNC:19708]
MAP2K4	mitogen-activated protein kinase kinase 4 [Source:HGNC Symbol;Acc:HGNC:6844]
MAP2K7	mitogen-activated protein kinase kinase 7 [Source:HGNC Symbol;Acc:HGNC:6847]
5-Mar	membrane associated ring-CH-type finger 5 [Source:HGNC Symbol;Acc:HGNC:26025]
MIOS	meiosis regulator for oocyte development [Source:HGNC Symbol;Acc:HGNC:21905]
MMP15	matrix metallopeptidase 15 [Source:HGNC Symbol;Acc:HGNC:7161]
MMP19	matrix metallopeptidase 19 [Source:HGNC Symbol;Acc:HGNC:7165]
MSRB1	methionine sulfoxide reductase B1 [Source:HGNC Symbol;Acc:HGNC:14133]
NCF4	neutrophil cytosolic factor 4 [Source:HGNC Symbol;Acc:HGNC:7662]
NFATC2IP	nuclear factor of activated T-cells 2 interacting protein [Source:HGNC Symbol;Acc:HGNC:25906]
NFATC3	nuclear factor of activated T-cells 3 [Source:HGNC Symbol;Acc:HGNC:7777]
NNT	nicotinamide nucleotide transhydrogenase [Source:HGNC Symbol;Acc:HGNC:7863]
OMA1	OMA1 zinc metallopeptidase [Source:HGNC Symbol;Acc:HGNC:29661]
P2RY11	purinergic receptor P2Y11 [Source:HGNC Symbol;Acc:HGNC:8540]
P2RY14	purinergic receptor P2Y14 [Source:HGNC Symbol;Acc:HGNC:16442]
PDP1	pyruvate dehyrogenase phosphatase catalytic subunit 1 [Source:HGNC Symbol;Acc:HGNC:9279]
PDPR	pyruvate dehydrogenase phosphatase regulatory subunit [Source:HGNC Symbol;Acc:HGNC:30264]
PGAM5	PGAM family member 5, mitochondrial serine/threonine protein phosphatase [Source:HGNC Symbol;Acc:HGNC:28763]

POLRMT	polymerase (RNA) mitochondrial [Source:HGNC Symbol;Acc:HGNC:9200]
PPP3CA	protein phosphatase 3 catalytic subunit alpha [Source:HGNC Symbol;Acc:HGNC:9314]
RCC1	regulator of chromosome condensation 1 [Source:HGNC Symbol;Acc:HGNC:1913]
RCC1L	RCC1 like [Source:HGNC Symbol;Acc:HGNC:14948]
SDHAF1	succinate dehydrogenase complex assembly factor 1 [Source:HGNC Symbol;Acc:HGNC:33867]
SEH1L	SEH1 like nucleoporin [Source:HGNC Symbol;Acc:HGNC:30379]
SENP2	SUMO1/sentrin/SMT3 specific peptidase 2 [Source:HGNC Symbol;Acc:HGNC:23116]
SENP5	SUMO1/sentrin specific peptidase 5 [Source:HGNC Symbol;Acc:HGNC:28407]
SLC25A14	solute carrier family 25 member 14 [Source:HGNC Symbol;Acc:HGNC:10984]
SLC25A27	solute carrier family 25 member 27 [Source:HGNC Symbol;Acc:HGNC:21065]
SLC8B1	solute carrier family 8 member B1 [Source:HGNC Symbol;Acc:HGNC:26175]
SURF2	surfeit 2 [Source:HGNC Symbol;Acc:HGNC:11475]
TXNRD2	thioredoxin reductase 2 [Source:HGNC Symbol;Acc:HGNC:18155]
ULK2	unc-51 like autophagy activating kinase 2 [Source:HGNC Symbol;Acc:HGNC:13480]
UQCC3	ubiquinol-cytochrome c reductase complex assembly factor 3 [Source:HGNC Symbol;Acc:HGNC:34399]
VPS13C	vacuolar protein sorting 13 homolog C [Source:HGNC Symbol;Acc:HGNC:23594]
VPS18	VPS18, CORVET/HOPS core subunit [Source:HGNC Symbol;Acc:HGNC:15972]
WDR59	WD repeat domain 59 [Source:HGNC Symbol;Acc:HGNC:25706]
ADD1	adducin 1 [Source:HGNC Symbol;Acc:HGNC:243]
ATP5B	ATP synthase, H+ transporting, mitochondrial F1 complex, beta polypeptide [Source:HGNC Symbol;Acc:HGNC:830]
ATP5I	ATP synthase, H+ transporting, mitochondrial Fo complex subunit E [Source:HGNC Symbol;Acc:HGNC:846]
COX7C	cytochrome c oxidase subunit 7C [Source:HGNC Symbol;Acc:HGNC:2292]
SAA4	serum amyloid A4, constitutive [Source:HGNC Symbol;Acc:HGNC:10516]

Supplementary Table S3: the third list of genes involved in the anti-VEGF resistance related network (AV-DRN).	
GJA1	gap junction protein alpha 1 [Source:HGNC Symbol;Acc:HGNC:4274]
GJD2	gap junction protein delta 2 [Source:HGNC Symbol;Acc:HGNC:19154]
LPAR1	lysophosphatidic acid receptor 1 [Source:HGNC Symbol;Acc:HGNC:3166]
GNAI1	G protein subunit alpha i1 [Source:HGNC Symbol;Acc:HGNC:4384]
GNAI3	G protein subunit alpha i3 [Source:HGNC Symbol;Acc:HGNC:4387]
GNAI2	G protein subunit alpha i2 [Source:HGNC Symbol;Acc:HGNC:4385]
PDGFA	platelet derived growth factor subunit A [Source:HGNC Symbol;Acc:HGNC:8799]
PDGFB	platelet derived growth factor subunit B [Source:HGNC Symbol;Acc:HGNC:8800]
PDGFC	platelet derived growth factor C [Source:HGNC Symbol;Acc:HGNC:8801]
PDGFD	platelet derived growth factor D [Source:HGNC Symbol;Acc:HGNC:30620]
EGF	epidermal growth factor [Source:HGNC Symbol;Acc:HGNC:3229]
PDGFRA	platelet derived growth factor receptor alpha [Source:HGNC Symbol;Acc:HGNC:8803]
PDGFRB	platelet derived growth factor receptor beta [Source:HGNC Symbol;Acc:HGNC:8804]
EGFR	epidermal growth factor receptor [Source:HGNC Symbol;Acc:HGNC:3236]
GRB2	growth factor receptor bound protein 2 [Source:HGNC Symbol;Acc:HGNC:4566]
SOS1	SOS Ras/Rac guanine nucleotide exchange factor 1 [Source:HGNC Symbol;Acc:HGNC:11187]
SOS2	SOS Ras/Rho guanine nucleotide exchange factor 2 [Source:HGNC Symbol;Acc:HGNC:11188]
HRAS	Harvey rat sarcoma viral oncogene homolog [Source:HGNC Symbol;Acc:HGNC:5173]
KRAS	KRAS proto-oncogene, GTPase [Source:HGNC Symbol;Acc:HGNC:6407]
NRAS	neuroblastoma RAS viral oncogene homolog [Source:HGNC Symbol;Acc:HGNC:7989]
RAF1	Raf-1 proto-oncogene, serine/threonine kinase [Source:HGNC Symbol;Acc:HGNC:9829]
MAP2K1	mitogen-activated protein kinase kinase 1 [Source:HGNC Symbol;Acc:HGNC:6840]
MAP2K2	mitogen-activated protein kinase kinase 2 [Source:HGNC Symbol;Acc:HGNC:6842]
MAPK1	mitogen-activated protein kinase 1 [Source:HGNC Symbol;Acc:HGNC:6871]
MAPK3	mitogen-activated protein kinase 3 [Source:HGNC Symbol;Acc:HGNC:6877]
SRC	SRC proto-oncogene, non-receptor tyrosine kinase [Source:HGNC Symbol;Acc:HGNC:11283]
MAP3K2	mitogen-activated protein kinase kinase kinase 2 [Source:HGNC Symbol;Acc:HGNC:6854]
MAP2K5	mitogen-activated protein kinase kinase 5 [Source:HGNC Symbol;Acc:HGNC:6845]
MAPK7	mitogen-activated protein kinase 7 [Source:HGNC Symbol;Acc:HGNC:6880]
TUBA1B	tubulin alpha 1b [Source:HGNC Symbol;Acc:HGNC:18809]
TUBA4A	tubulin alpha 4a [Source:HGNC Symbol;Acc:HGNC:12407]
TUBA3C	tubulin alpha 3c [Source:HGNC Symbol;Acc:HGNC:12408]
TUBA1A	tubulin alpha 1a [Source:HGNC Symbol;Acc:HGNC:20766]
TUBA1C	tubulin alpha 1c [Source:HGNC Symbol;Acc:HGNC:20768]
TUBA8	tubulin alpha 8 [Source:HGNC Symbol;Acc:HGNC:12410]
TUBA3E	tubulin alpha 3e [Source:HGNC Symbol;Acc:HGNC:20765]
TUBA3D	tubulin alpha 3d [Source:HGNC Symbol;Acc:HGNC:24071]
TUBAL3	tubulin alpha like 3 [Source:HGNC Symbol;Acc:HGNC:23534]
TUBB6	tubulin beta 6 class V [Source:HGNC Symbol;Acc:HGNC:20776]
TUBB	tubulin beta class I [Source:HGNC Symbol;Acc:HGNC:20778]

TUBB1	tubulin beta 1 class VI [Source:HGNC Symbol;Acc:HGNC:16257]
TUBB2A	tubulin beta 2A class IIa [Source:HGNC Symbol;Acc:HGNC:12412]
TUBB3	tubulin beta 3 class III [Source:HGNC Symbol;Acc:HGNC:20772]
TUBB4A	tubulin beta 4A class IVa [Source:HGNC Symbol;Acc:HGNC:20774]
TUBB8	tubulin beta 8 class VIII [Source:HGNC Symbol;Acc:HGNC:20773]
TUBB2B	tubulin beta 2B class IIb [Source:HGNC Symbol;Acc:HGNC:30829]
TUBB4B	tubulin beta 4B class IVb [Source:HGNC Symbol;Acc:HGNC:20771]
CSNK1D	casein kinase 1 delta [Source:HGNC Symbol;Acc:HGNC:2452]
CDK1	cyclin dependent kinase 1 [Source:HGNC Symbol;Acc:HGNC:1722]
TJP1	tight junction protein 1 [Source:HGNC Symbol;Acc:HGNC:11827]
ADRB1	adrenoceptor beta 1 [Source:HGNC Symbol;Acc:HGNC:285]
DRD1	dopamine receptor D1 [Source:HGNC Symbol;Acc:HGNC:3020]
GNAS	GNAS complex locus [Source:HGNC Symbol;Acc:HGNC:4392]
DRD2	dopamine receptor D2 [Source:HGNC Symbol;Acc:HGNC:3023]
ADCY1	adenylate cyclase 1 (brain) [Source:HGNC Symbol;Acc:HGNC:232]
ADCY2	adenylate cyclase 2 (brain) [Source:HGNC Symbol;Acc:HGNC:233]
ADCY3	adenylate cyclase 3 [Source:HGNC Symbol;Acc:HGNC:234]
ADCY4	adenylate cyclase 4 [Source:HGNC Symbol;Acc:HGNC:235]
ADCY5	adenylate cyclase 5 [Source:HGNC Symbol;Acc:HGNC:236]
ADCY6	adenylate cyclase 6 [Source:HGNC Symbol;Acc:HGNC:237]
ADCY7	adenylate cyclase 7 [Source:HGNC Symbol;Acc:HGNC:238]
ADCY8	adenylate cyclase 8 (brain) [Source:HGNC Symbol;Acc:HGNC:239]
ADCY9	adenylate cyclase 9 [Source:HGNC Symbol;Acc:HGNC:240]
PRKACA	protein kinase cAMP-activated catalytic subunit alpha [Source:HGNC Symbol;Acc:HGNC:9380]
PRKACB	protein kinase cAMP-activated catalytic subunit beta [Source:HGNC Symbol;Acc:HGNC:9381]
PRKACG	protein kinase cAMP-activated catalytic subunit gamma [Source:HGNC Symbol;Acc:HGNC:9382]
HTR2A	5-hydroxytryptamine receptor 2A [Source:HGNC Symbol;Acc:HGNC:5293]
HTR2B	5-hydroxytryptamine receptor 2B [Source:HGNC Symbol;Acc:HGNC:5294]
HTR2C	5-hydroxytryptamine receptor 2C [Source:HGNC Symbol;Acc:HGNC:5295]
GRM1	glutamate metabotropic receptor 1 [Source:HGNC Symbol;Acc:HGNC:4593]
GRM5	glutamate metabotropic receptor 5 [Source:HGNC Symbol;Acc:HGNC:4597]
GNA11	G protein subunit alpha 11 [Source:HGNC Symbol;Acc:HGNC:4379]
GNAQ	G protein subunit alpha q [Source:HGNC Symbol;Acc:HGNC:4390]
PLCB1	phospholipase C beta 1 [Source:HGNC Symbol;Acc:HGNC:15917]
PLCB2	phospholipase C beta 2 [Source:HGNC Symbol;Acc:HGNC:9055]
PLCB3	phospholipase C beta 3 [Source:HGNC Symbol;Acc:HGNC:9056]
PLCB4	phospholipase C beta 4 [Source:HGNC Symbol;Acc:HGNC:9059]
ITPR1	inositol 1,4,5-trisphosphate receptor type 1 [Source:HGNC Symbol;Acc:HGNC:6180]
ITPR2	inositol 1,4,5-trisphosphate receptor type 2 [Source:HGNC Symbol;Acc:HGNC:6181]
ITPR3	inositol 1,4,5-trisphosphate receptor type 3 [Source:HGNC Symbol;Acc:HGNC:6182]
PRKCA	protein kinase C alpha [Source:HGNC Symbol;Acc:HGNC:9393]

PRKCB	protein kinase C beta [Source:HGNC Symbol;Acc:HGNC:9395]
PRKCG	protein kinase C gamma [Source:HGNC Symbol;Acc:HGNC:9402]
GUCY1A2	guanylate cyclase 1, soluble, alpha 2 [Source:HGNC Symbol;Acc:HGNC:4684]
PRKG1	protein kinase, cGMP-dependent, type I [Source:HGNC Symbol;Acc:HGNC:9414]
PRKG2	protein kinase, cGMP-dependent, type II [Source:HGNC Symbol;Acc:HGNC:9416]
GUCY1A3	guanylate cyclase 1, soluble, alpha 3 [Source:HGNC Symbol;Acc:HGNC:4685]
CRB3	crumbs 3, cell polarity complex component [Source:HGNC Symbol;Acc:HGNC:20237]
CLDN4	claudin 4 [Source:HGNC Symbol;Acc:HGNC:2046]
CLDN3	claudin 3 [Source:HGNC Symbol;Acc:HGNC:2045]
CLDN7	claudin 7 [Source:HGNC Symbol;Acc:HGNC:2049]
CLDN19	claudin 19 [Source:HGNC Symbol;Acc:HGNC:2040]
CLDN16	claudin 16 [Source:HGNC Symbol;Acc:HGNC:2037]
CLDN14	claudin 14 [Source:HGNC Symbol;Acc:HGNC:2035]
CLDN15	claudin 15 [Source:HGNC Symbol;Acc:HGNC:2036]
CLDN17	claudin 17 [Source:HGNC Symbol;Acc:HGNC:2038]
CLDN20	claudin 20 [Source:HGNC Symbol;Acc:HGNC:2042]
CLDN11	claudin 11 [Source:HGNC Symbol;Acc:HGNC:8514]
CLDN18	claudin 18 [Source:HGNC Symbol;Acc:HGNC:2039]
CLDN22	claudin 22 [Source:HGNC Symbol;Acc:HGNC:2044]
CLDN5	claudin 5 [Source:HGNC Symbol;Acc:HGNC:2047]
CLDN10	claudin 10 [Source:HGNC Symbol;Acc:HGNC:2033]
CLDN8	claudin 8 [Source:HGNC Symbol;Acc:HGNC:2050]
CLDN6	claudin 6 [Source:HGNC Symbol;Acc:HGNC:2048]
CLDN2	claudin 2 [Source:HGNC Symbol;Acc:HGNC:2041]
CLDN1	claudin 1 [Source:HGNC Symbol;Acc:HGNC:2032]
CLDN9	claudin 9 [Source:HGNC Symbol;Acc:HGNC:2051]
CLDN23	claudin 23 [Source:HGNC Symbol;Acc:HGNC:17591]
CLDN34	claudin 34 [Source:HGNC Symbol;Acc:HGNC:51259]
CLDN25	claudin 25 [Source:HGNC Symbol;Acc:HGNC:37218]
CLDN24	claudin 24 [Source:HGNC Symbol;Acc:HGNC:37200]
OCLN	occludin [Source:HGNC Symbol;Acc:HGNC:8104]
F11R	F11 receptor [Source:HGNC Symbol;Acc:HGNC:14685]
JAM2	junctional adhesion molecule 2 [Source:HGNC Symbol;Acc:HGNC:14686]
JAM3	junctional adhesion molecule 3 [Source:HGNC Symbol;Acc:HGNC:15532]
BVES	blood vessel epicardial substance [Source:HGNC Symbol;Acc:HGNC:1152]
CDC42	cell division cycle 42 [Source:HGNC Symbol;Acc:HGNC:1736]
PARD6A	par-6 family cell polarity regulator alpha [Source:HGNC Symbol;Acc:HGNC:15943]
PARD6G	par-6 family cell polarity regulator gamma [Source:HGNC Symbol;Acc:HGNC:16076]
PARD6B	par-6 family cell polarity regulator beta [Source:HGNC Symbol;Acc:HGNC:16245]
MPP5	membrane palmitoylated protein 5 [Source:HGNC Symbol;Acc:HGNC:18669]
MPP4	membrane palmitoylated protein 4 [Source:HGNC Symbol;Acc:HGNC:13680]

TJP3	tight junction protein 3 [Source:HGNC Symbol;Acc:HGNC:11829]
PATJ	PATJ, crumbs cell polarity complex component [Source:HGNC Symbol;Acc:HGNC:28881]
MPDZ	multiple PDZ domain crumbs cell polarity complex component [Source:HGNC Symbol;Acc:HGNC:7208]
PRKCZ	protein kinase C zeta [Source:HGNC Symbol;Acc:HGNC:9412]
PRKCI	protein kinase C iota [Source:HGNC Symbol;Acc:HGNC:9404]
AMOT	angiomotin [Source:HGNC Symbol;Acc:HGNC:17810]
AMOTL1	angiomotin like 1 [Source:HGNC Symbol;Acc:HGNC:17811]
AMOTL2	angiomotin like 2 [Source:HGNC Symbol;Acc:HGNC:17812]
ARHGAP17	Rho GTPase activating protein 17 [Source:HGNC Symbol;Acc:HGNC:18239]
RAC1	ras-related C3 botulinum toxin substrate 1 (rho family, small GTP binding protein Rac1) [Source:HGNC Symbol;Acc:HGNC:9801]
NF2	neurofibromin 2 (merlin) [Source:HGNC Symbol;Acc:HGNC:7773]
LLGL2	LLGL2, scribble cell polarity complex component [Source:HGNC Symbol;Acc:HGNC:6629]
LLGL1	LLGL1, scribble cell polarity complex component [Source:HGNC Symbol;Acc:HGNC:6628]
DLG1	discs large MAGUK scaffold protein 1 [Source:HGNC Symbol;Acc:HGNC:2900]
SCRIB	scribbled planar cell polarity protein [Source:HGNC Symbol;Acc:HGNC:30377]
PPP2CA	protein phosphatase 2 catalytic subunit alpha [Source:HGNC Symbol;Acc:HGNC:9299]
PPP2CB	protein phosphatase 2 catalytic subunit beta [Source:HGNC Symbol;Acc:HGNC:9300]
PPP2R1B	protein phosphatase 2 scaffold subunit Abeta [Source:HGNC Symbol;Acc:HGNC:9303]
PPP2R1A	protein phosphatase 2 scaffold subunit Aalpha [Source:HGNC Symbol;Acc:HGNC:9302]
PPP2R2A	protein phosphatase 2 regulatory subunit Balpha [Source:HGNC Symbol;Acc:HGNC:9304]
PPP2R2B	protein phosphatase 2 regulatory subunit Bbeta [Source:HGNC Symbol;Acc:HGNC:9305]
PPP2R2C	protein phosphatase 2 regulatory subunit Bgamma [Source:HGNC Symbol;Acc:HGNC:9306]
PPP2R2D	protein phosphatase 2 regulatory subunit Bdelta [Source:HGNC Symbol;Acc:HGNC:23732]
PARD3	par-3 family cell polarity regulator [Source:HGNC Symbol;Acc:HGNC:16051]
TIAM1	T-cell lymphoma invasion and metastasis 1 [Source:HGNC Symbol;Acc:HGNC:11805]
TJAP1	tight junction associated protein 1 [Source:HGNC Symbol;Acc:HGNC:17949]
DLG2	discs large MAGUK scaffold protein 2 [Source:HGNC Symbol;Acc:HGNC:2901]
DLG3	discs large MAGUK scaffold protein 3 [Source:HGNC Symbol;Acc:HGNC:2902]
NEDD4	neural precursor cell expressed, developmentally down-regulated 4, E3 ubiquitin protein ligase [Source:HGNC Symbol;Acc:HGNC:7727]
NEDD4L	neural precursor cell expressed, developmentally down-regulated 4-like, E3 ubiquitin protein ligase [Source:HGNC Symbol;Acc:HGNC:7728]
CGN	cingulin [Source:HGNC Symbol;Acc:HGNC:17429]
CGNL1	cingulin-like 1 [Source:HGNC Symbol;Acc:HGNC:25931]
ARHGEF2	Rho/Rac guanine nucleotide exchange factor 2 [Source:HGNC Symbol;Acc:HGNC:682]
RHOA	ras homolog family member A [Source:HGNC Symbol;Acc:HGNC:667]
GATA4	GATA binding protein 4 [Source:HGNC Symbol;Acc:HGNC:4173]
MARVELD3	MARVEL domain containing 3 [Source:HGNC Symbol;Acc:HGNC:30525]
MAP3K1	mitogen-activated protein kinase kinase kinase 1 [Source:HGNC Symbol;Acc:HGNC:6848]
MAPK8	mitogen-activated protein kinase 8 [Source:HGNC Symbol;Acc:HGNC:6881]
MAPK10	mitogen-activated protein kinase 10 [Source:HGNC Symbol;Acc:HGNC:6872]

MAPK9	mitogen-activated protein kinase 9 [Source:HGNC Symbol;Acc:HGNC:6886]
JUN	Jun proto-oncogene, AP-1 transcription factor subunit [Source:HGNC Symbol;Acc:HGNC:6204]
CD1A	CD1a molecule [Source:HGNC Symbol;Acc:HGNC:1634]
CD1B	CD1b molecule [Source:HGNC Symbol;Acc:HGNC:1635]
CD1C	CD1c molecule [Source:HGNC Symbol;Acc:HGNC:1636]
CD1D	CD1d molecule [Source:HGNC Symbol;Acc:HGNC:1637]
CD1E	CD1e molecule [Source:HGNC Symbol;Acc:HGNC:1638]
CFTR	cystic fibrosis transmembrane conductance regulator [Source:HGNC Symbol;Acc:HGNC:1884]
CDK4	cyclin dependent kinase 4 [Source:HGNC Symbol;Acc:HGNC:1773]
YBX3	Y-box binding protein 3 [Source:HGNC Symbol;Acc:HGNC:2428]
SYMPK	symplekin [Source:HGNC Symbol;Acc:HGNC:22935]
PCNA	proliferating cell nuclear antigen [Source:HGNC Symbol;Acc:HGNC:8729]
CCND1	cyclin D1 [Source:HGNC Symbol;Acc:HGNC:1582]
ERBB2	erb-b2 receptor tyrosine kinase 2 [Source:HGNC Symbol;Acc:HGNC:3430]
RUNX1	runt related transcription factor 1 [Source:HGNC Symbol;Acc:HGNC:10471]
HSPA4	heat shock protein family A (Hsp70) member 4 [Source:HGNC Symbol;Acc:HGNC:5237]
SLC9A3R1	SLC9A3 regulator 1 [Source:HGNC Symbol;Acc:HGNC:11075]
EZR	ezrin [Source:HGNC Symbol;Acc:HGNC:12691]
RDX	radixin [Source:HGNC Symbol;Acc:HGNC:9944]
MSN	moesin [Source:HGNC Symbol;Acc:HGNC:7373]
PRKCE	protein kinase C epsilon [Source:HGNC Symbol;Acc:HGNC:9401]
ACTG1	actin gamma 1 [Source:HGNC Symbol;Acc:HGNC:144]
ACTB	actin beta [Source:HGNC Symbol;Acc:HGNC:132]
CACNA1D	calcium voltage-gated channel subunit alpha1 D [Source:HGNC Symbol;Acc:HGNC:1391]
MAP3K5	mitogen-activated protein kinase kinase kinase 5 [Source:HGNC Symbol;Acc:HGNC:6857]
MAP2K7	mitogen-activated protein kinase kinase 7 [Source:HGNC Symbol;Acc:HGNC:6847]
CTTN	cortactin [Source:HGNC Symbol;Acc:HGNC:3338]
HCLS1	hematopoietic cell-specific Lyn substrate 1 [Source:HGNC Symbol;Acc:HGNC:4844]
ACTR2	ARP2 actin-related protein 2 homolog (yeast) [Source:HGNC Symbol;Acc:HGNC:169]
ACTR3B	ARP3 actin-related protein 3 homolog B (yeast) [Source:HGNC Symbol;Acc:HGNC:17256]
ACTR3C	ARP3 actin-related protein 3 homolog C [Source:HGNC Symbol;Acc:HGNC:37282]
ACTR3	ARP3 actin-related protein 3 homolog (yeast) [Source:HGNC Symbol;Acc:HGNC:170]
ARPC1B	actin related protein 2/3 complex subunit 1B [Source:HGNC Symbol;Acc:HGNC:704]
ARPC1A	actin related protein 2/3 complex subunit 1A [Source:HGNC Symbol;Acc:HGNC:703]
ARPC2	actin related protein 2/3 complex subunit 2 [Source:HGNC Symbol;Acc:HGNC:705]
ARPC3	actin related protein 2/3 complex subunit 3 [Source:HGNC Symbol;Acc:HGNC:706]
ARPC4	actin related protein 2/3 complex subunit 4 [Source:HGNC Symbol;Acc:HGNC:707]
ARPC5	actin related protein 2/3 complex subunit 5 [Source:HGNC Symbol;Acc:HGNC:708]
ARPC5L	actin related protein 2/3 complex subunit 5 like [Source:HGNC Symbol;Acc:HGNC:23366]
WHAMM	WAS protein homolog associated with actin, golgi membranes and microtubules [Source:HGNC Symbol;Acc:HGNC:30493]
WAS	Wiskott-Aldrich syndrome [Source:HGNC Symbol;Acc:HGNC:12731]

VASP	vasodilator-stimulated phosphoprotein [Source:HGNC Symbol;Acc:HGNC:12652]
RAB13	RAB13, member RAS oncogene family [Source:HGNC Symbol;Acc:HGNC:9762]
ARHGEF18	Rho/Rac guanine nucleotide exchange factor 18 [Source:HGNC Symbol;Acc:HGNC:17090]
ROCK1	Rho associated coiled-coil containing protein kinase 1 [Source:HGNC Symbol;Acc:HGNC:10251]
ROCK2	Rho associated coiled-coil containing protein kinase 2 [Source:HGNC Symbol;Acc:HGNC:10252]
MYL2	myosin light chain 2 [Source:HGNC Symbol;Acc:HGNC:7583]
EPB41L4B	erythrocyte membrane protein band 4.1 like 4B [Source:HGNC Symbol;Acc:HGNC:19818]
STK11	serine/threonine kinase 11 [Source:HGNC Symbol;Acc:HGNC:11389]
PRKAA1	protein kinase AMP-activated catalytic subunit alpha 1 [Source:HGNC Symbol;Acc:HGNC:9376]
PRKAA2	protein kinase AMP-activated catalytic subunit alpha 2 [Source:HGNC Symbol;Acc:HGNC:9377]
PRKAB1	protein kinase AMP-activated non-catalytic subunit beta 1 [Source:HGNC Symbol;Acc:HGNC:9378]
PRKAB2	protein kinase AMP-activated non-catalytic subunit beta 2 [Source:HGNC Symbol;Acc:HGNC:9379]
PRKAG1	protein kinase AMP-activated non-catalytic subunit gamma 1 [Source:HGNC Symbol;Acc:HGNC:9385]
PRKAG3	protein kinase AMP-activated non-catalytic subunit gamma 3 [Source:HGNC Symbol;Acc:HGNC:9387]
PRKAG2	protein kinase AMP-activated non-catalytic subunit gamma 2 [Source:HGNC Symbol;Acc:HGNC:9386]
MYH9	myosin, heavy chain 9, non-muscle [Source:HGNC Symbol;Acc:HGNC:7579]
MYH10	myosin, heavy chain 10, non-muscle [Source:HGNC Symbol;Acc:HGNC:7568]
MYH11	myosin, heavy chain 11, smooth muscle [Source:HGNC Symbol;Acc:HGNC:7569]
MYH14	myosin, heavy chain 14, non-muscle [Source:HGNC Symbol;Acc:HGNC:23212]
MYL6B	myosin light chain 6B [Source:HGNC Symbol;Acc:HGNC:29823]
MYL6	myosin light chain 6 [Source:HGNC Symbol;Acc:HGNC:7587]
MYL9	myosin light chain 9 [Source:HGNC Symbol;Acc:HGNC:15754]
MYL12B	myosin light chain 12B [Source:HGNC Symbol;Acc:HGNC:29827]
MYL12A	myosin light chain 12A [Source:HGNC Symbol;Acc:HGNC:16701]
IGSF5	immunoglobulin superfamily member 5 [Source:HGNC Symbol;Acc:HGNC:5952]
MAGI1	membrane associated guanylate kinase, WW and PDZ domain containing 1 [Source:HGNC Symbol;Acc:HGNC:946]
SYNPO	synaptopodin [Source:HGNC Symbol;Acc:HGNC:30672]
ACTN1	actinin alpha 1 [Source:HGNC Symbol;Acc:HGNC:163]
ACTN4	actinin alpha 4 [Source:HGNC Symbol;Acc:HGNC:166]
MICALL2	MICAL like 2 [Source:HGNC Symbol;Acc:HGNC:29672]
RAB8A	RAB8A, member RAS oncogene family [Source:HGNC Symbol;Acc:HGNC:7007]
RAB8B	RAB8B, member RAS oncogene family [Source:HGNC Symbol;Acc:HGNC:30273]
RAPGEF6	Rap guanine nucleotide exchange factor 6 [Source:HGNC Symbol;Acc:HGNC:20655]
RAP1A	RAP1A, member of RAS oncogene family [Source:HGNC Symbol;Acc:HGNC:9855]
ITGB1	integrin subunit beta 1 [Source:HGNC Symbol;Acc:HGNC:6153]
TJP2	tight junction protein 2 [Source:HGNC Symbol;Acc:HGNC:11828]
RAPGEF2	Rap guanine nucleotide exchange factor 2 [Source:HGNC Symbol;Acc:HGNC:16854]
RAP2C	RAP2C, member of RAS oncogene family [Source:HGNC Symbol;Acc:HGNC:21165]

MARVELD2	MARVEL domain containing 2 [Source:HGNC Symbol;Acc:HGNC:26401]
MLLT4	myeloid/lymphoid or mixed-lineage leukemia; translocated to, 4 [Source:HGNC Symbol;Acc:HGNC:7137]
CD58	CD58 molecule [Source:HGNC Symbol;Acc:HGNC:1688]
CD2	CD2 molecule [Source:HGNC Symbol;Acc:HGNC:1639]
IGSF11	immunoglobulin superfamily member 11 [Source:HGNC Symbol;Acc:HGNC:16669]
CD80	CD80 molecule [Source:HGNC Symbol;Acc:HGNC:1700]
CD274	CD274 molecule [Source:HGNC Symbol;Acc:HGNC:17635]
CD28	CD28 molecule [Source:HGNC Symbol;Acc:HGNC:1653]
CD86	CD86 molecule [Source:HGNC Symbol;Acc:HGNC:1705]
CTLA4	cytotoxic T-lymphocyte associated protein 4 [Source:HGNC Symbol;Acc:HGNC:2505]
ICOSLG	inducible T-cell costimulator ligand [Source:HGNC Symbol;Acc:HGNC:17087]
ICOS	inducible T-cell costimulator [Source:HGNC Symbol;Acc:HGNC:5351]
HLA-DMA	major histocompatibility complex, class II, DM alpha [Source:HGNC Symbol;Acc:HGNC:4934]
HLA-DMB	major histocompatibility complex, class II, DM beta [Source:HGNC Symbol;Acc:HGNC:4935]
HLA-DOA	major histocompatibility complex, class II, DO alpha [Source:HGNC Symbol;Acc:HGNC:4936]
HLA-DOB	major histocompatibility complex, class II, DO beta [Source:HGNC Symbol;Acc:HGNC:4937]
HLA-DPA1	major histocompatibility complex, class II, DP alpha 1 [Source:HGNC Symbol;Acc:HGNC:4938]
HLA-DPB1	major histocompatibility complex, class II, DP beta 1 [Source:HGNC Symbol;Acc:HGNC:4940]
HLA-DQA1	major histocompatibility complex, class II, DQ alpha 1 [Source:HGNC Symbol;Acc:HGNC:4942]
HLA-DQA2	major histocompatibility complex, class II, DQ alpha 2 [Source:HGNC Symbol;Acc:HGNC:4943]
HLA-DQB1	major histocompatibility complex, class II, DQ beta 1 [Source:HGNC Symbol;Acc:HGNC:4944]
HLA-DRA	major histocompatibility complex, class II, DR alpha [Source:HGNC Symbol;Acc:HGNC:4947]
HLA-DRB1	major histocompatibility complex, class II, DR beta 1 [Source:HGNC Symbol;Acc:HGNC:4948]
HLA-DRB3	major histocompatibility complex, class II, DR beta 3 [Source:HGNC Symbol;Acc:HGNC:4951]
HLA-DRB4	major histocompatibility complex, class II, DR beta 4 [Source:HGNC Symbol;Acc:HGNC:4952]
HLA-DRB5	major histocompatibility complex, class II, DR beta 5 [Source:HGNC Symbol;Acc:HGNC:4953]
CD4	CD4 molecule [Source:HGNC Symbol;Acc:HGNC:1678]
HLA-A	major histocompatibility complex, class I, A [Source:HGNC Symbol;Acc:HGNC:4931]
HLA-B	major histocompatibility complex, class I, B [Source:HGNC Symbol;Acc:HGNC:4932]
HLA-C	major histocompatibility complex, class I, C [Source:HGNC Symbol;Acc:HGNC:4933]
HLA-F	major histocompatibility complex, class I, F [Source:HGNC Symbol;Acc:HGNC:4963]
HLA-G	major histocompatibility complex, class I, G [Source:HGNC Symbol;Acc:HGNC:4964]
HLA-E	major histocompatibility complex, class I, E [Source:HGNC Symbol;Acc:HGNC:4962]
CD8A	CD8a molecule [Source:HGNC Symbol;Acc:HGNC:1706]
CD8B	CD8b molecule [Source:HGNC Symbol;Acc:HGNC:1707]
PDCD1LG2	programmed cell death 1 ligand 2 [Source:HGNC Symbol;Acc:HGNC:18731]
CD276	CD276 molecule [Source:HGNC Symbol;Acc:HGNC:19137]
VTCN1	V-set domain containing T cell activation inhibitor 1 [Source:HGNC Symbol;Acc:HGNC:28873]
PDCD1	programmed cell death 1 [Source:HGNC Symbol;Acc:HGNC:8760]
CD40	CD40 molecule [Source:HGNC Symbol;Acc:HGNC:11919]
CD40LG	CD40 ligand [Source:HGNC Symbol;Acc:HGNC:11935]

ALCAM	activated leukocyte cell adhesion molecule [Source:HGNC Symbol;Acc:HGNC:400]
CD6	CD6 molecule [Source:HGNC Symbol;Acc:HGNC:1691]
PVR	poliovirus receptor [Source:HGNC Symbol;Acc:HGNC:9705]
CD226	CD226 molecule [Source:HGNC Symbol;Acc:HGNC:16961]
NECTIN2	nectin cell adhesion molecule 2 [Source:HGNC Symbol;Acc:HGNC:9707]
TIGIT	T-cell immunoreceptor with Ig and ITIM domains [Source:HGNC Symbol;Acc:HGNC:26838]
ITGAL	integrin subunit alpha L [Source:HGNC Symbol;Acc:HGNC:6148]
ITGB2	integrin subunit beta 2 [Source:HGNC Symbol;Acc:HGNC:6155]
ICAM1	intercellular adhesion molecule 1 [Source:HGNC Symbol;Acc:HGNC:5344]
ICAM2	intercellular adhesion molecule 2 [Source:HGNC Symbol;Acc:HGNC:5345]
ICAM3	intercellular adhesion molecule 3 [Source:HGNC Symbol;Acc:HGNC:5346]
CD22	CD22 molecule [Source:HGNC Symbol;Acc:HGNC:1643]
PTPRC	protein tyrosine phosphatase, receptor type C [Source:HGNC Symbol;Acc:HGNC:9666]
SIGLEC1	sialic acid binding Ig like lectin 1 [Source:HGNC Symbol;Acc:HGNC:11127]
SPN	sialophorin [Source:HGNC Symbol;Acc:HGNC:11249]
NECTIN3	nectin cell adhesion molecule 3 [Source:HGNC Symbol;Acc:HGNC:17664]
ESAM	endothelial cell adhesion molecule [Source:HGNC Symbol;Acc:HGNC:17474]
CDH5	cadherin 5 [Source:HGNC Symbol;Acc:HGNC:1764]
PECAM1	platelet and endothelial cell adhesion molecule 1 [Source:HGNC Symbol;Acc:HGNC:8823]
CD99	CD99 molecule [Source:HGNC Symbol;Acc:HGNC:7082]
CD99L2	CD99 molecule like 2 [Source:HGNC Symbol;Acc:HGNC:18237]
ITGAM	integrin subunit alpha M [Source:HGNC Symbol;Acc:HGNC:6149]
SELPLG	selectin P ligand [Source:HGNC Symbol;Acc:HGNC:10722]
SELP	selectin P [Source:HGNC Symbol;Acc:HGNC:10721]
ITGA4	integrin subunit alpha 4 [Source:HGNC Symbol;Acc:HGNC:6140]
ITGA9	integrin subunit alpha 9 [Source:HGNC Symbol;Acc:HGNC:6145]
VCAM1	vascular cell adhesion molecule 1 [Source:HGNC Symbol;Acc:HGNC:12663]
ITGB7	integrin subunit beta 7 [Source:HGNC Symbol;Acc:HGNC:6162]
MADCAM1	mucosal vascular addressin cell adhesion molecule 1 [Source:HGNC Symbol;Acc:HGNC:6765]
SELL	selectin L [Source:HGNC Symbol;Acc:HGNC:10720]
CD34	CD34 molecule [Source:HGNC Symbol;Acc:HGNC:1662]
GLG1	golgi glycoprotein 1 [Source:HGNC Symbol;Acc:HGNC:4316]
SELE	selectin E [Source:HGNC Symbol;Acc:HGNC:10718]
NECTIN1	nectin cell adhesion molecule 1 [Source:HGNC Symbol;Acc:HGNC:9706]
CDH2	cadherin 2 [Source:HGNC Symbol;Acc:HGNC:1759]
NCAM1	neural cell adhesion molecule 1 [Source:HGNC Symbol;Acc:HGNC:7656]
NCAM2	neural cell adhesion molecule 2 [Source:HGNC Symbol;Acc:HGNC:7657]
L1CAM	L1 cell adhesion molecule [Source:HGNC Symbol;Acc:HGNC:6470]
CADM1	cell adhesion molecule 1 [Source:HGNC Symbol;Acc:HGNC:5951]
NEGR1	neuronal growth regulator 1 [Source:HGNC Symbol;Acc:HGNC:17302]
NTNG1	netrin G1 [Source:HGNC Symbol;Acc:HGNC:23319]

LRRC4C	leucine rich repeat containing 4C [Source:HGNC Symbol;Acc:HGNC:29317]
NTNG2	netrin G2 [Source:HGNC Symbol;Acc:HGNC:14288]
LRRC4	leucine rich repeat containing 4 [Source:HGNC Symbol;Acc:HGNC:15586]
PTPRF	protein tyrosine phosphatase, receptor type F [Source:HGNC Symbol;Acc:HGNC:9670]
LRRC4B	leucine rich repeat containing 4B [Source:HGNC Symbol;Acc:HGNC:25042]
SDC1	syndecan 1 [Source:HGNC Symbol;Acc:HGNC:10658]
SDC2	syndecan 2 [Source:HGNC Symbol;Acc:HGNC:10659]
SDC3	syndecan 3 [Source:HGNC Symbol;Acc:HGNC:10660]
SDC4	syndecan 4 [Source:HGNC Symbol;Acc:HGNC:10661]
ITGAV	integrin subunit alpha V [Source:HGNC Symbol;Acc:HGNC:6150]
ITGB8	integrin subunit beta 8 [Source:HGNC Symbol;Acc:HGNC:6163]
ITGA8	integrin subunit alpha 8 [Source:HGNC Symbol;Acc:HGNC:6144]
NRXN1	neurexin 1 [Source:HGNC Symbol;Acc:HGNC:8008]
NRXN2	neurexin 2 [Source:HGNC Symbol;Acc:HGNC:8009]
NRXN3	neurexin 3 [Source:HGNC Symbol;Acc:HGNC:8010]
NLGN1	neuroligin 1 [Source:HGNC Symbol;Acc:HGNC:14291]
NLGN2	neuroligin 2 [Source:HGNC Symbol;Acc:HGNC:14290]
NLGN3	neuroligin 3 [Source:HGNC Symbol;Acc:HGNC:14289]
NLGN4X	neuroligin 4, X-linked [Source:HGNC Symbol;Acc:HGNC:14287]
NLGN4Y	neuroligin 4, Y-linked [Source:HGNC Symbol;Acc:HGNC:15529]
CADM3	cell adhesion molecule 3 [Source:HGNC Symbol;Acc:HGNC:17601]
NRCAM	neuronal cell adhesion molecule [Source:HGNC Symbol;Acc:HGNC:7994]
CNTN1	contactin 1 [Source:HGNC Symbol;Acc:HGNC:2171]
PTPRM	protein tyrosine phosphatase, receptor type M [Source:HGNC Symbol;Acc:HGNC:9675]
CNTN2	contactin 2 [Source:HGNC Symbol;Acc:HGNC:2172]
NFASC	neurofascin [Source:HGNC Symbol;Acc:HGNC:29866]
CNTNAP1	contactin associated protein 1 [Source:HGNC Symbol;Acc:HGNC:8011]
CNTNAP2	contactin associated protein-like 2 [Source:HGNC Symbol;Acc:HGNC:13830]
MPZ	myelin protein zero [Source:HGNC Symbol;Acc:HGNC:7225]
MPZL1	myelin protein zero like 1 [Source:HGNC Symbol;Acc:HGNC:7226]
MAG	myelin associated glycoprotein [Source:HGNC Symbol;Acc:HGNC:6783]
CDH1	cadherin 1 [Source:HGNC Symbol;Acc:HGNC:1748]
VCAN	versican [Source:HGNC Symbol;Acc:HGNC:2464]
ITGA6	integrin subunit alpha 6 [Source:HGNC Symbol;Acc:HGNC:6142]
CDH3	cadherin 3 [Source:HGNC Symbol;Acc:HGNC:1762]
CDH4	cadherin 4 [Source:HGNC Symbol;Acc:HGNC:1763]
CDH15	cadherin 15 [Source:HGNC Symbol;Acc:HGNC:1754]
NEO1	neogenin 1 [Source:HGNC Symbol;Acc:HGNC:7754]
C10orf54	chromosome 10 open reading frame 54 [Source:HGNC Symbol;Acc:HGNC:30085]
YY1AP1	YY1 associated protein 1 [Source:HGNC Symbol;Acc:HGNC:30935]
TAZ	tafazzin [Source:HGNC Symbol;Acc:HGNC:11577]

A1BG	alpha-1-B glycoprotein [Source:HGNC Symbol;Acc:HGNC:5]
A2M	alpha-2-macroglobulin [Source:HGNC Symbol;Acc:HGNC:7]
SGMS1	sphingomyelin synthase 1 [Source:HGNC Symbol;Acc:HGNC:29799]
SAV1	salvador family WW domain containing protein 1 [Source:HGNC Symbol;Acc:HGNC:17795]
LARGE1	LARGE xylosyl- and glucuronyltransferase 1 [Source:HGNC Symbol;Acc:HGNC:6511]
CRB1	crumbs 1, cell polarity complex component [Source:HGNC Symbol;Acc:HGNC:2343]
SMAD1	SMAD family member 1 [Source:HGNC Symbol;Acc:HGNC:6767]
SMAD7	SMAD family member 7 [Source:HGNC Symbol;Acc:HGNC:6773]
RUNX2	runt related transcription factor 2 [Source:HGNC Symbol;Acc:HGNC:10472]
TP73	tumor protein p73 [Source:HGNC Symbol;Acc:HGNC:12003]
ERBB4	erb-b2 receptor tyrosine kinase 4 [Source:HGNC Symbol;Acc:HGNC:3432]
CTGF	connective tissue growth factor [Source:HGNC Symbol;Acc:HGNC:2500]
CYR61	cysteine rich angiogenic inducer 61 [Source:HGNC Symbol;Acc:HGNC:2654]
AXL	AXL receptor tyrosine kinase [Source:HGNC Symbol;Acc:HGNC:905]
ANKRD1	ankyrin repeat domain 1 [Source:HGNC Symbol;Acc:HGNC:15819]
AREG	amphiregulin [Source:HGNC Symbol;Acc:HGNC:651]
ADAMTS1	ADAM metallopeptidase with thrombospondin type 1 motif 1 [Source:HGNC Symbol;Acc:HGNC:217]
THBS1	thrombospondin 1 [Source:HGNC Symbol;Acc:HGNC:11785]
WWC1	WW and C2 domain containing 1 [Source:HGNC Symbol;Acc:HGNC:29435]
YWHAQ	tyrosine 3-monooxygenase/tryptophan 5-monooxygenase activation protein theta [Source:HGNC Symbol;Acc:HGNC:12854]
SGCG	sarcoglycan gamma [Source:HGNC Symbol;Acc:HGNC:10809]
AAMP	angio associated migratory cell protein [Source:HGNC Symbol;Acc:HGNC:18]
CDH17	cadherin 17 [Source:HGNC Symbol;Acc:HGNC:1756]
NOV	nephroblastoma overexpressed [Source:HGNC Symbol;Acc:HGNC:7885]
STAT3	signal transducer and activator of transcription 3 [Source:HGNC Symbol;Acc:HGNC:11364]
LATS1	large tumor suppressor kinase 1 [Source:HGNC Symbol;Acc:HGNC:6514]
LATS2	large tumor suppressor kinase 2 [Source:HGNC Symbol;Acc:HGNC:6515]
STK3	serine/threonine kinase 3 [Source:HGNC Symbol;Acc:HGNC:11406]
STK4	serine/threonine kinase 4 [Source:HGNC Symbol;Acc:HGNC:11408]
WWTR1	WW domain containing transcription regulator 1 [Source:HGNC Symbol;Acc:HGNC:24042]
FRMD6	FERM domain containing 6 [Source:HGNC Symbol;Acc:HGNC:19839]
MST1	macrophage stimulating 1 [Source:HGNC Symbol;Acc:HGNC:7380]
MYO1C	myosin IC [Source:HGNC Symbol;Acc:HGNC:7597]
PDK1	pyruvate dehydrogenase kinase 1 [Source:HGNC Symbol;Acc:HGNC:8809]
PIK3CA	phosphatidylinositol-4,5-bisphosphate 3-kinase catalytic subunit alpha [Source:HGNC Symbol;Acc:HGNC:8975]
PTPN14	protein tyrosine phosphatase, non-receptor type 14 [Source:HGNC Symbol;Acc:HGNC:9647]
TBX5	T-box 5 [Source:HGNC Symbol;Acc:HGNC:11604]
TIE1	tyrosine kinase with immunoglobulin like and EGF like domains 1 [Source:HGNC Symbol;Acc:HGNC:11809]
MOB1A	MOB kinase activator 1A [Source:HGNC Symbol;Acc:HGNC:16015]

MOB1B	MOB kinase activator 1B [Source:HGNC Symbol;Acc:HGNC:29801]
TEAD1	TEA domain transcription factor 1 [Source:HGNC Symbol;Acc:HGNC:11714]
TEK	TEK receptor tyrosine kinase [Source:HGNC Symbol;Acc:HGNC:11724]
YAP1	Yes associated protein 1 [Source:HGNC Symbol;Acc:HGNC:16262]
MAP4K1	mitogen-activated protein kinase kinase kinase kinase 1 [Source:HGNC Symbol;Acc:HGNC:6863]
MAP4K2	mitogen-activated protein kinase kinase kinase kinase 2 [Source:HGNC Symbol;Acc:HGNC:6864]
MAP4K3	mitogen-activated protein kinase kinase kinase kinase 3 [Source:HGNC Symbol;Acc:HGNC:6865]
MAP4K5	mitogen-activated protein kinase kinase kinase kinase 5 [Source:HGNC Symbol;Acc:HGNC:6867]
SMAD2	SMAD family member 2 [Source:HGNC Symbol;Acc:HGNC:6768]
SMAD3	SMAD family member 3 [Source:HGNC Symbol;Acc:HGNC:6769]
TAOK1	TAO kinase 1 [Source:HGNC Symbol;Acc:HGNC:29259]
TAOK2	TAO kinase 2 [Source:HGNC Symbol;Acc:HGNC:16835]
TAOK3	TAO kinase 3 [Source:HGNC Symbol;Acc:HGNC:18133]
ANGPT1	angiopoietin 1 [Source:HGNC Symbol;Acc:HGNC:484]
ANGPT2	angiopoietin 2 [Source:HGNC Symbol;Acc:HGNC:485]
ANGPTL1	angiopoietin like 1 [Source:HGNC Symbol;Acc:HGNC:489]
ANGPT4	angiopoietin 4 [Source:HGNC Symbol;Acc:HGNC:487]
MAPK4	mitogen-activated protein kinase 4 [Source:HGNC Symbol;Acc:HGNC:6878]
TEAD2	TEA domain transcription factor 2 [Source:HGNC Symbol;Acc:HGNC:11715]
TEAD3	TEA domain transcription factor 3 [Source:HGNC Symbol;Acc:HGNC:11716]
TEAD4	TEA domain transcription factor 4 [Source:HGNC Symbol;Acc:HGNC:11717]
SPTA1	spectrin alpha, erythrocytic 1 [Source:HGNC Symbol;Acc:HGNC:11272]
ITGA5	integrin subunit alpha 5 [Source:HGNC Symbol;Acc:HGNC:6141]
ITGB3	integrin subunit beta 3 [Source:HGNC Symbol;Acc:HGNC:6156]
ITGB5	integrin subunit beta 5 [Source:HGNC Symbol;Acc:HGNC:6160]
ITGA2	integrin subunit alpha 2 [Source:HGNC Symbol;Acc:HGNC:6137]
ITGA2B	integrin subunit alpha 2b [Source:HGNC Symbol;Acc:HGNC:6138]
CTNNB1	catenin beta 1 [Source:HGNC Symbol;Acc:HGNC:2514]
CTNNA1	catenin alpha 1 [Source:HGNC Symbol;Acc:HGNC:2509]
CXCL8	C-X-C motif chemokine ligand 8 [Source:HGNC Symbol;Acc:HGNC:6025]
WNT1	Wnt family member 1 [Source:HGNC Symbol;Acc:HGNC:12774]
VEGFA	vascular endothelial growth factor A [Source:HGNC Symbol;Acc:HGNC:12680]
WDR45	WD repeat domain 45 [Source:HGNC Symbol;Acc:HGNC:28912]
KDR	kinase insert domain receptor [Source:HGNC Symbol;Acc:HGNC:6307]
TGFB1	transforming growth factor beta 1 [Source:HGNC Symbol;Acc:HGNC:11766]
SMAD4	SMAD family member 4 [Source:HGNC Symbol;Acc:HGNC:6770]
SMAD5	SMAD family member 5 [Source:HGNC Symbol;Acc:HGNC:6771]
SMAD6	SMAD family member 6 [Source:HGNC Symbol;Acc:HGNC:6772]
SMAD9	SMAD family member 9 [Source:HGNC Symbol;Acc:HGNC:6774]
PIK3C3	phosphatidylinositol 3-kinase catalytic subunit type 3 [Source:HGNC Symbol;Acc:HGNC:8974]
PIK3R4	phosphoinositide-3-kinase regulatory subunit 4 [Source:HGNC Symbol;Acc:HGNC:8982]

LAMP1	lysosomal associated membrane protein 1 [Source:HGNC Symbol;Acc:HGNC:6499]
LAMP2	lysosomal associated membrane protein 2 [Source:HGNC Symbol;Acc:HGNC:6501]
NFKB1	nuclear factor kappa B subunit 1 [Source:HGNC Symbol;Acc:HGNC:7794]
NFKB2	nuclear factor kappa B subunit 2 [Source:HGNC Symbol;Acc:HGNC:7795]
HMGB1	high mobility group box 1 [Source:HGNC Symbol;Acc:HGNC:4983]
BMP1	bone morphogenetic protein 1 [Source:HGNC Symbol;Acc:HGNC:1067]
BMP2	bone morphogenetic protein 2 [Source:HGNC Symbol;Acc:HGNC:1069]
BMP3	bone morphogenetic protein 3 [Source:HGNC Symbol;Acc:HGNC:1070]
BMP4	bone morphogenetic protein 4 [Source:HGNC Symbol;Acc:HGNC:1071]
BMP5	bone morphogenetic protein 5 [Source:HGNC Symbol;Acc:HGNC:1072]
BMP6	bone morphogenetic protein 6 [Source:HGNC Symbol;Acc:HGNC:1073]
BMP7	bone morphogenetic protein 7 [Source:HGNC Symbol;Acc:HGNC:1074]
BMP8B	bone morphogenetic protein 8b [Source:HGNC Symbol;Acc:HGNC:1075]
GDF2	growth differentiation factor 2 [Source:HGNC Symbol;Acc:HGNC:4217]
BMP10	bone morphogenetic protein 10 [Source:HGNC Symbol;Acc:HGNC:20869]
BECN1	beclin 1 [Source:HGNC Symbol;Acc:HGNC:1034]
CAV1	caveolin 1 [Source:HGNC Symbol;Acc:HGNC:1527]
TSC2	tuberous sclerosis 2 [Source:HGNC Symbol;Acc:HGNC:12363]
AKT1	AKT serine/threonine kinase 1 [Source:HGNC Symbol;Acc:HGNC:391]
PARK2	parkin RBR E3 ubiquitin protein ligase [Source:HGNC Symbol;Acc:HGNC:8607]
PINK1	PTEN induced putative kinase 1 [Source:HGNC Symbol;Acc:HGNC:14581]
MAP1LC3A	microtubule associated protein 1 light chain 3 alpha [Source:HGNC Symbol;Acc:HGNC:6838]
MAP1LC3B	microtubule associated protein 1 light chain 3 beta [Source:HGNC Symbol;Acc:HGNC:13352]
MAP1LC3C	microtubule associated protein 1 light chain 3 gamma [Source:HGNC Symbol;Acc:HGNC:13353]
IFNG	interferon, gamma [Source:HGNC Symbol;Acc:HGNC:5438]
IFNB1	interferon beta 1 [Source:HGNC Symbol;Acc:HGNC:5434]
CDH19	cadherin 19 [Source:HGNC Symbol;Acc:HGNC:1758]
CDH12	cadherin 12 [Source:HGNC Symbol;Acc:HGNC:1751]
HIF1A	hypoxia inducible factor 1 alpha subunit [Source:HGNC Symbol;Acc:HGNC:4910]
TNFSF4	tumor necrosis factor superfamily member 4 [Source:HGNC Symbol;Acc:HGNC:11934]
NOTCH1	notch 1 [Source:HGNC Symbol;Acc:HGNC:7881]
NOTCH2	notch 2 [Source:HGNC Symbol;Acc:HGNC:7882]
NOTCH3	notch 3 [Source:HGNC Symbol;Acc:HGNC:7883]
NOTCH4	notch 4 [Source:HGNC Symbol;Acc:HGNC:7884]
TANK	TRAF family member associated NFKB activator [Source:HGNC Symbol;Acc:HGNC:11562]
PTEN	phosphatase and tensin homolog [Source:HGNC Symbol;Acc:HGNC:9588]
NLRP3	NLR family pyrin domain containing 3 [Source:HGNC Symbol;Acc:HGNC:16400]
SNCA	synuclein alpha [Source:HGNC Symbol;Acc:HGNC:11138]
PTGER2	prostaglandin E receptor 2 [Source:HGNC Symbol;Acc:HGNC:9594]
GABARAPL1	GABA type A receptor associated protein like 1 [Source:HGNC Symbol;Acc:HGNC:4068]
GABARAPL2	GABA type A receptor associated protein like 2 [Source:HGNC Symbol;Acc:HGNC:13291]

ATG16L1	autophagy related 16 like 1 [Source:HGNC Symbol;Acc:HGNC:21498]
BNIP3	BCL2/adenovirus E1B 19kDa interacting protein 3 [Source:HGNC Symbol;Acc:HGNC:1084]
BCL2	B-cell CLL/lymphoma 2 [Source:HGNC Symbol;Acc:HGNC:990]
ULK1	unc-51 like autophagy activating kinase 1 [Source:HGNC Symbol;Acc:HGNC:12558]
SIRT1	sirtuin 1 [Source:HGNC Symbol;Acc:HGNC:14929]
FOXO1	forkhead box O1 [Source:HGNC Symbol;Acc:HGNC:3819]
PFKFB3	6-phosphofructo-2-kinase/fructose-2,6-biphosphatase 3 [Source:HGNC Symbol;Acc:HGNC:8874]
SLC2A1	solute carrier family 2 member 1 [Source:HGNC Symbol;Acc:HGNC:11005]
DLL4	delta like canonical Notch ligand 4 [Source:HGNC Symbol;Acc:HGNC:2910]
IL6	interleukin 6 [Source:HGNC Symbol;Acc:HGNC:6018]
HSPA8	heat shock protein family A (Hsp70) member 8 [Source:HGNC Symbol;Acc:HGNC:5241]
RB1CC1	RB1 inducible coiled-coil 1 [Source:HGNC Symbol;Acc:HGNC:15574]
ATG13	autophagy related 13 [Source:HGNC Symbol;Acc:HGNC:29091]
ATG101	autophagy related 101 [Source:HGNC Symbol;Acc:HGNC:25679]
SERPINA1	serpin family A member 1 [Source:HGNC Symbol;Acc:HGNC:8941]
ATG14	autophagy related 14 [Source:HGNC Symbol;Acc:HGNC:19962]
NRBF2	nuclear receptor binding factor 2 [Source:HGNC Symbol;Acc:HGNC:19692]
ATG2A	autophagy related 2A [Source:HGNC Symbol;Acc:HGNC:29028]
ATG2B	autophagy related 2B [Source:HGNC Symbol;Acc:HGNC:20187]
ATG9A	autophagy related 9A [Source:HGNC Symbol;Acc:HGNC:22408]
ATG12	autophagy related 12 [Source:HGNC Symbol;Acc:HGNC:588]
ATG5	autophagy related 5 [Source:HGNC Symbol;Acc:HGNC:589]
ATG7	autophagy related 7 [Source:HGNC Symbol;Acc:HGNC:16935]
ATG10	autophagy related 10 [Source:HGNC Symbol;Acc:HGNC:20315]
GABARAP	GABA type A receptor-associated protein [Source:HGNC Symbol;Acc:HGNC:4067]
TBK1	TANK binding kinase 1 [Source:HGNC Symbol;Acc:HGNC:11584]
OPTN	optineurin [Source:HGNC Symbol;Acc:HGNC:17142]
IL1B	interleukin 1 beta [Source:HGNC Symbol;Acc:HGNC:5992]
IL18	interleukin 18 [Source:HGNC Symbol;Acc:HGNC:5986]
CALCOCO2	calcium binding and coiled-coil domain 2 [Source:HGNC Symbol;Acc:HGNC:29912]
DDX58	DEXD/H-box helicase 58 [Source:HGNC Symbol;Acc:HGNC:19102]
MAVS	mitochondrial antiviral signaling protein [Source:HGNC Symbol;Acc:HGNC:29233]
TFEB	transcription factor EB [Source:HGNC Symbol;Acc:HGNC:11753]
TNF	tumor necrosis factor [Source:HGNC Symbol;Acc:HGNC:11892]
CXCL10	C-X-C motif chemokine ligand 10 [Source:HGNC Symbol;Acc:HGNC:10637]
CXCL11	C-X-C motif chemokine ligand 11 [Source:HGNC Symbol;Acc:HGNC:10638]
IRF3	interferon regulatory factor 3 [Source:HGNC Symbol;Acc:HGNC:6118]
CCL5	C-C motif chemokine ligand 5 [Source:HGNC Symbol;Acc:HGNC:10632]
CCL2	C-C motif chemokine ligand 2 [Source:HGNC Symbol;Acc:HGNC:10618]
BCL2L1	BCL2 like 1 [Source:HGNC Symbol;Acc:HGNC:992]
ARHGAP1	Rho GTPase activating protein 1 [Source:HGNC Symbol;Acc:HGNC:673]

RHO	rhodopsin [Source:HGNC Symbol;Acc:HGNC:10012]
GPC1	glypican 1 [Source:HGNC Symbol;Acc:HGNC:4449]
RAB7A	RAB7A, member RAS oncogene family [Source:HGNC Symbol;Acc:HGNC:9788]
HMBOX1	homeobox containing 1 [Source:HGNC Symbol;Acc:HGNC:26137]
MT2A	metallothionein 2A [Source:HGNC Symbol;Acc:HGNC:7406]
SOX2	SRY-box 2 [Source:HGNC Symbol;Acc:HGNC:11195]
POU5F1	POU class 5 homeobox 1 [Source:HGNC Symbol;Acc:HGNC:9221]
AMBRA1	autophagy and beclin 1 regulator 1 [Source:HGNC Symbol;Acc:HGNC:25990]
GATA1	GATA binding protein 1 [Source:HGNC Symbol;Acc:HGNC:4170]
FOXO3	forkhead box O3 [Source:HGNC Symbol;Acc:HGNC:3821]
FOXO4	forkhead box O4 [Source:HGNC Symbol;Acc:HGNC:7139]
CDKN2A	cyclin dependent kinase inhibitor 2A [Source:HGNC Symbol;Acc:HGNC:1787]
CDKN2B	cyclin dependent kinase inhibitor 2B [Source:HGNC Symbol;Acc:HGNC:1788]
CDKN1A	cyclin dependent kinase inhibitor 1A [Source:HGNC Symbol;Acc:HGNC:1784]
NOS3	nitric oxide synthase 3 [Source:HGNC Symbol;Acc:HGNC:7876]
VWF	von Willebrand factor [Source:HGNC Symbol;Acc:HGNC:12726]
PEG3	paternally expressed 3 [Source:HGNC Symbol;Acc:HGNC:8826]
GRP	gastrin releasing peptide [Source:HGNC Symbol;Acc:HGNC:4605]
GRPR	gastrin releasing peptide receptor [Source:HGNC Symbol;Acc:HGNC:4609]
AGGF1	angiogenic factor with G-patch and FHA domains 1 [Source:HGNC Symbol;Acc:HGNC:24684]
HSPA5	heat shock protein family A (Hsp70) member 5 [Source:HGNC Symbol;Acc:HGNC:5238]
IDO1	indoleamine 2,3-dioxygenase 1 [Source:HGNC Symbol;Acc:HGNC:6059]
KLF2	Kruppel like factor 2 [Source:HGNC Symbol;Acc:HGNC:6347]
UVRAG	UV radiation resistance associated [Source:HGNC Symbol;Acc:HGNC:12640]
MB21D1	Mab-21 domain containing 1 [Source:HGNC Symbol;Acc:HGNC:21367]
CXCL9	C-X-C motif chemokine ligand 9 [Source:HGNC Symbol;Acc:HGNC:7098]
NBR1	NBR1, autophagy cargo receptor [Source:HGNC Symbol;Acc:HGNC:6746]
HAVCR2	hepatitis A virus cellular receptor 2 [Source:HGNC Symbol;Acc:HGNC:18437]
ATG9B	autophagy related 9B [Source:HGNC Symbol;Acc:HGNC:21899]
ATG16L2	autophagy related 16 like 2 [Source:HGNC Symbol;Acc:HGNC:25464]
ATG4A	autophagy related 4A cysteine peptidase [Source:HGNC Symbol;Acc:HGNC:16489]
ATG4B	autophagy related 4B cysteine peptidase [Source:HGNC Symbol;Acc:HGNC:20790]
ATG4C	autophagy related 4C cysteine peptidase [Source:HGNC Symbol;Acc:HGNC:16040]
ATG4D	autophagy related 4D cysteine peptidase [Source:HGNC Symbol;Acc:HGNC:20789]
ACTC1	actin, alpha, cardiac muscle 1 [Source:HGNC Symbol;Acc:HGNC:143]
MYC	v-myc avian myelocytomatosis viral oncogene homolog [Source:HGNC Symbol;Acc:HGNC:7553]
ATG3	autophagy related 3 [Source:HGNC Symbol;Acc:HGNC:20962]
BEST1	bestrophin 1 [Source:HGNC Symbol;Acc:HGNC:12703]
AADAC	arylacetamide deacetylase [Source:HGNC Symbol;Acc:HGNC:17]
TP53	tumor protein p53 [Source:HGNC Symbol;Acc:HGNC:11998]
P2RY2	purinergic receptor P2Y2 [Source:HGNC Symbol;Acc:HGNC:8541]

P2RX7	purinergic receptor P2X 7 [Source:HGNC Symbol;Acc:HGNC:8537]
BCL10	B-cell CLL/lymphoma 10 [Source:HGNC Symbol;Acc:HGNC:989]
NOD2	nucleotide binding oligomerization domain containing 2 [Source:HGNC Symbol;Acc:HGNC:5331]
RPS13	ribosomal protein S13 [Source:HGNC Symbol;Acc:HGNC:10386]
MDM2	MDM2 proto-oncogene [Source:HGNC Symbol;Acc:HGNC:6973]
FUNDC1	FUN14 domain containing 1 [Source:HGNC Symbol;Acc:HGNC:28746]
SPARC	secreted protein acidic and cysteine rich [Source:HGNC Symbol;Acc:HGNC:11219]
CD44	CD44 molecule (Indian blood group) [Source:HGNC Symbol;Acc:HGNC:1681]
BRAF	B-Raf proto-oncogene, serine/threonine kinase [Source:HGNC Symbol;Acc:HGNC:1097]
SETD2	SET domain containing 2 [Source:HGNC Symbol;Acc:HGNC:18420]
MYCL	v-myc avian myelocytomatosis viral oncogene lung carcinoma derived homolog [Source:HGNC Symbol;Acc:HGNC:7555]
MYCN	v-myc avian myelocytomatosis viral oncogene neuroblastoma derived homolog [Source:HGNC Symbol;Acc:HGNC:7559]
ZBTB24	zinc finger and BTB domain containing 24 [Source:HGNC Symbol;Acc:HGNC:21143]
DAPK1	death associated protein kinase 1 [Source:HGNC Symbol;Acc:HGNC:2674]
DIRAS3	DIRAS family GTPase 3 [Source:HGNC Symbol;Acc:HGNC:687]
DRAM1	DNA damage regulated autophagy modulator 1 [Source:HGNC Symbol;Acc:HGNC:25645]
NF1	neurofibromin 1 [Source:HGNC Symbol;Acc:HGNC:7765]
RB1	RB transcriptional corepressor 1 [Source:HGNC Symbol;Acc:HGNC:9884]
TSC1	tuberous sclerosis 1 [Source:HGNC Symbol;Acc:HGNC:12362]
VHL	von Hippel-Lindau tumor suppressor [Source:HGNC Symbol;Acc:HGNC:12687]
TRPM3	transient receptor potential cation channel subfamily M member 3 [Source:HGNC Symbol;Acc:HGNC:17992]
BNIP3L	BCL2/adenovirus E1B 19kDa interacting protein 3-like [Source:HGNC Symbol;Acc:HGNC:1085]
NAT2	N-acetyltransferase 2 (arylamine N-acetyltransferase) [Source:HGNC Symbol;Acc:HGNC:7646]
BAD	BCL2 associated agonist of cell death [Source:HGNC Symbol;Acc:HGNC:936]
BRCA1	BRCA1, DNA repair associated [Source:HGNC Symbol;Acc:HGNC:1100]
WIPI1	WD repeat domain, phosphoinositide interacting 1 [Source:HGNC Symbol;Acc:HGNC:25471]
INS	insulin [Source:HGNC Symbol;Acc:HGNC:6081]
IGF1R	insulin like growth factor 1 receptor [Source:HGNC Symbol;Acc:HGNC:5465]
DDIT4	DNA damage inducible transcript 4 [Source:HGNC Symbol;Acc:HGNC:24944]
RHEB	Ras homolog enriched in brain [Source:HGNC Symbol;Acc:HGNC:10011]
ERN1	endoplasmic reticulum to nucleus signaling 1 [Source:HGNC Symbol;Acc:HGNC:3449]
EIF2AK3	eukaryotic translation initiation factor 2 alpha kinase 3 [Source:HGNC Symbol;Acc:HGNC:3255]
EIF2AK4	eukaryotic translation initiation factor 2 alpha kinase 4 [Source:HGNC Symbol;Acc:HGNC:19687]
VAMP8	vesicle associated membrane protein 8 [Source:HGNC Symbol;Acc:HGNC:12647]
CTSD	cathepsin D [Source:HGNC Symbol;Acc:HGNC:2529]
CTSB	cathepsin B [Source:HGNC Symbol;Acc:HGNC:2527]
CTSL	cathepsin L [Source:HGNC Symbol;Acc:HGNC:2537]
STX17	syntaxin 17 [Source:HGNC Symbol;Acc:HGNC:11432]
SNAP29	synaptosome associated protein 29 [Source:HGNC Symbol;Acc:HGNC:11133]

RAB33B	RAB33B, member RAS oncogene family [Source:HGNC Symbol;Acc:HGNC:16075]
RPTOR	regulatory associated protein of MTOR complex 1 [Source:HGNC Symbol;Acc:HGNC:30287]
MTOR	mechanistic target of rapamycin [Source:HGNC Symbol;Acc:HGNC:3942]
MLST8	MTOR associated protein, LST8 homolog [Source:HGNC Symbol;Acc:HGNC:24825]
RPS6KB1	ribosomal protein S6 kinase B1 [Source:HGNC Symbol;Acc:HGNC:10436]
TRAF6	TNF receptor associated factor 6 [Source:HGNC Symbol;Acc:HGNC:12036]
VMP1	vacuole membrane protein 1 [Source:HGNC Symbol;Acc:HGNC:29559]
SUPT20H	SPT20 homolog, SAGA complex component [Source:HGNC Symbol;Acc:HGNC:20596]
RUBCN	RUN and cysteine rich domain containing beclin 1 interacting protein [Source:HGNC Symbol;Acc:HGNC:28991]
SH3GLB1	SH3 domain containing GRB2 like endophilin B1 [Source:HGNC Symbol;Acc:HGNC:10833]
ZFYVE1	zinc finger FYVE-type containing 1 [Source:HGNC Symbol;Acc:HGNC:13180]
MTMR3	myotubularin related protein 3 [Source:HGNC Symbol;Acc:HGNC:7451]
CREB1	cAMP responsive element binding protein 1 [Source:HGNC Symbol;Acc:HGNC:2345]
EIF4EBP1	eukaryotic translation initiation factor 4E binding protein 1 [Source:HGNC Symbol;Acc:HGNC:3288]
KEAP1	kelch like ECH associated protein 1 [Source:HGNC Symbol;Acc:HGNC:23177]
NDRG1	N-myc downstream regulated 1 [Source:HGNC Symbol;Acc:HGNC:7679]
NFE2L2	nuclear factor, erythroid 2 like 2 [Source:HGNC Symbol;Acc:HGNC:7782]
PGAM5	PGAM family member 5, mitochondrial serine/threonine protein phosphatase [Source:HGNC Symbol;Acc:HGNC:28763]
SQSTM1	sequestosome 1 [Source:HGNC Symbol;Acc:HGNC:11280]
XIAP	X-linked inhibitor of apoptosis [Source:HGNC Symbol;Acc:HGNC:592]
SESN1	sestrin 1 [Source:HGNC Symbol;Acc:HGNC:21595]
SESN2	sestrin 2 [Source:HGNC Symbol;Acc:HGNC:20746]
PA2G4	proliferation-associated 2G4 [Source:HGNC Symbol;Acc:HGNC:8550]
ABL1	ABL proto-oncogene 1, non-receptor tyrosine kinase [Source:HGNC Symbol;Acc:HGNC:76]
BCR	BCR, RhoGEF and GTPase activating protein [Source:HGNC Symbol;Acc:HGNC:1014]
DIRAS1	DIRAS family GTPase 1 [Source:HGNC Symbol;Acc:HGNC:19127]
DIRAS2	DIRAS family GTPase 2 [Source:HGNC Symbol;Acc:HGNC:19323]
PDPK1	3-phosphoinositide dependent protein kinase 1 [Source:HGNC Symbol;Acc:HGNC:8816]
EIF2A	eukaryotic translation initiation factor 2A [Source:HGNC Symbol;Acc:HGNC:3254]
CAMKK2	calcium/calmodulin dependent protein kinase kinase 2 [Source:HGNC Symbol;Acc:HGNC:1470]
E2F2	E2F transcription factor 2 [Source:HGNC Symbol;Acc:HGNC:3114]
E2F4	E2F transcription factor 4 [Source:HGNC Symbol;Acc:HGNC:3118]
E2F6	E2F transcription factor 6 [Source:HGNC Symbol;Acc:HGNC:3120]
TMEM173	transmembrane protein 173 [Source:HGNC Symbol;Acc:HGNC:27962]
PRAP1	proline rich acidic protein 1 [Source:HGNC Symbol;Acc:HGNC:23304]
ULK2	unc-51 like autophagy activating kinase 2 [Source:HGNC Symbol;Acc:HGNC:13480]
SESN3	sestrin 3 [Source:HGNC Symbol;Acc:HGNC:23060]
NRP2	neuropilin 2 [Source:HGNC Symbol;Acc:HGNC:8005]
FUNDC2	FUN14 domain containing 2 [Source:HGNC Symbol;Acc:HGNC:24925]

RRAGB	Ras related GTP binding B [Source:HGNC Symbol;Acc:HGNC:19901]
RRAGA	Ras related GTP binding A [Source:HGNC Symbol;Acc:HGNC:16963]
RRAGC	Ras related GTP binding C [Source:HGNC Symbol;Acc:HGNC:19902]
RRAGD	Ras related GTP binding D [Source:HGNC Symbol;Acc:HGNC:19903]
LAMP3	lysosomal associated membrane protein 3 [Source:HGNC Symbol;Acc:HGNC:14582]
NR2C2	nuclear receptor subfamily 2 group C member 2 [Source:HGNC Symbol;Acc:HGNC:7972]
PRAM1	PML-RARA regulated adaptor molecule 1 [Source:HGNC Symbol;Acc:HGNC:30091]
SLC16A1	solute carrier family 16 member 1 [Source:HGNC Symbol;Acc:HGNC:10922]
EGLN1	egl-9 family hypoxia inducible factor 1 [Source:HGNC Symbol;Acc:HGNC:1232]
SLC1A5	solute carrier family 1 member 5 [Source:HGNC Symbol;Acc:HGNC:10943]
GFPT1	glutamine--fructose-6-phosphate transaminase 1 [Source:HGNC Symbol;Acc:HGNC:4241]
G6PD	glucose-6-phosphate dehydrogenase [Source:HGNC Symbol;Acc:HGNC:4057]
SLC27A1	solute carrier family 27 member 1 [Source:HGNC Symbol;Acc:HGNC:10995]
LDHA	lactate dehydrogenase A [Source:HGNC Symbol;Acc:HGNC:6535]
SIRT3	sirtuin 3 [Source:HGNC Symbol;Acc:HGNC:14931]
SIRT6	sirtuin 6 [Source:HGNC Symbol;Acc:HGNC:14934]
IDH1	isocitrate dehydrogenase (NADP(+)) 1, cytosolic [Source:HGNC Symbol;Acc:HGNC:5382]
IDH2	isocitrate dehydrogenase (NADP(+)) 2, mitochondrial [Source:HGNC Symbol;Acc:HGNC:5383]
ACADSB	acyl-CoA dehydrogenase, short/branched chain [Source:HGNC Symbol;Acc:HGNC:91]
APLN	apelin [Source:HGNC Symbol;Acc:HGNC:16665]
TOP2A	topoisomerase (DNA) II alpha [Source:HGNC Symbol;Acc:HGNC:11989]
MKI67	marker of proliferation Ki-67 [Source:HGNC Symbol;Acc:HGNC:7107]
KIT	KIT proto-oncogene receptor tyrosine kinase [Source:HGNC Symbol;Acc:HGNC:6342]
SLC2A3	solute carrier family 2 member 3 [Source:HGNC Symbol;Acc:HGNC:11007]
GSK3B	glycogen synthase kinase 3 beta [Source:HGNC Symbol;Acc:HGNC:4617]
ALDH18A1	aldehyde dehydrogenase 18 family member A1 [Source:HGNC Symbol;Acc:HGNC:9722]
SQLE	squalene epoxidase [Source:HGNC Symbol;Acc:HGNC:11279]
ALDOA	aldolase, fructose-bisphosphate A [Source:HGNC Symbol;Acc:HGNC:414]
ALDOC	aldolase, fructose-bisphosphate C [Source:HGNC Symbol;Acc:HGNC:418]
BMPR2	bone morphogenetic protein receptor type 2 [Source:HGNC Symbol;Acc:HGNC:1078]
CD36	CD36 molecule [Source:HGNC Symbol;Acc:HGNC:1663]
CPT1A	carnitine palmitoyltransferase 1A [Source:HGNC Symbol;Acc:HGNC:2328]
DHFR	dihydrofolate reductase [Source:HGNC Symbol;Acc:HGNC:2861]
ENO1	enolase 1 [Source:HGNC Symbol;Acc:HGNC:3350]
FABP4	fatty acid binding protein 4 [Source:HGNC Symbol;Acc:HGNC:3559]
FFAR1	free fatty acid receptor 1 [Source:HGNC Symbol;Acc:HGNC:4498]
FASN	fatty acid synthase [Source:HGNC Symbol;Acc:HGNC:3594]
GAPDH	glyceraldehyde-3-phosphate dehydrogenase [Source:HGNC Symbol;Acc:HGNC:4141]
GLS	glutaminase [Source:HGNC Symbol;Acc:HGNC:4331]
GCH1	GTP cyclohydrolase 1 [Source:HGNC Symbol;Acc:HGNC:4193]
HK1	hexokinase 1 [Source:HGNC Symbol;Acc:HGNC:4922]

HK2	hexokinase 2 [Source:HGNC Symbol;Acc:HGNC:4923]
IRS2	insulin receptor substrate 2 [Source:HGNC Symbol;Acc:HGNC:6126]
LDHB	lactate dehydrogenase B [Source:HGNC Symbol;Acc:HGNC:6541]
PARP1	poly(ADP-ribose) polymerase 1 [Source:HGNC Symbol;Acc:HGNC:270]
PFKM	phosphofructokinase, muscle [Source:HGNC Symbol;Acc:HGNC:8877]
PPARGC1A	PPARG coactivator 1 alpha [Source:HGNC Symbol;Acc:HGNC:9237]
PGK1	phosphoglycerate kinase 1 [Source:HGNC Symbol;Acc:HGNC:8896]
EGLN3	egl-9 family hypoxia inducible factor 3 [Source:HGNC Symbol;Acc:HGNC:14661]
PKM	pyruvate kinase, muscle [Source:HGNC Symbol;Acc:HGNC:9021]
PPARG	peroxisome proliferator activated receptor gamma [Source:HGNC Symbol;Acc:HGNC:9236]
SLC16A3	solute carrier family 16 member 3 [Source:HGNC Symbol;Acc:HGNC:10924]
GPI	glucose-6-phosphate isomerase [Source:HGNC Symbol;Acc:HGNC:4458]
PRRT2	proline rich transmembrane protein 2 [Source:HGNC Symbol;Acc:HGNC:30500]
PPARA	peroxisome proliferator activated receptor alpha [Source:HGNC Symbol;Acc:HGNC:9232]
PGK2	phosphoglycerate kinase 2 [Source:HGNC Symbol;Acc:HGNC:8898]
CPT1B	carnitine palmitoyltransferase 1B [Source:HGNC Symbol;Acc:HGNC:2329]
CPT1C	carnitine palmitoyltransferase 1C [Source:HGNC Symbol;Acc:HGNC:18540]
PDK2	pyruvate dehydrogenase kinase 2 [Source:HGNC Symbol;Acc:HGNC:8810]
PDK3	pyruvate dehydrogenase kinase 3 [Source:HGNC Symbol;Acc:HGNC:8811]
PDK4	pyruvate dehydrogenase kinase 4 [Source:HGNC Symbol;Acc:HGNC:8812]
PRMT1	protein arginine methyltransferase 1 [Source:HGNC Symbol;Acc:HGNC:5187]
PRMT2	protein arginine methyltransferase 2 [Source:HGNC Symbol;Acc:HGNC:5186]
PRMT3	protein arginine methyltransferase 3 [Source:HGNC Symbol;Acc:HGNC:30163]
CARM1	coactivator associated arginine methyltransferase 1 [Source:HGNC Symbol;Acc:HGNC:23393]
PRMT5	protein arginine methyltransferase 5 [Source:HGNC Symbol;Acc:HGNC:10894]
PRMT6	protein arginine methyltransferase 6 [Source:HGNC Symbol;Acc:HGNC:18241]
PRMT7	protein arginine methyltransferase 7 [Source:HGNC Symbol;Acc:HGNC:25557]
PRMT8	protein arginine methyltransferase 8 [Source:HGNC Symbol;Acc:HGNC:5188]
PRMT9	protein arginine methyltransferase 9 [Source:HGNC Symbol;Acc:HGNC:25099]
COQ10A	coenzyme Q10A [Source:HGNC Symbol;Acc:HGNC:26515]
COQ10B	coenzyme Q10B [Source:HGNC Symbol;Acc:HGNC:25819]
HK3	hexokinase 3 [Source:HGNC Symbol;Acc:HGNC:4925]
JMJD6	arginine demethylase and lysine hydroxylase [Source:HGNC Symbol;Acc:HGNC:19355]
MDH2	malate dehydrogenase 2 [Source:HGNC Symbol;Acc:HGNC:6971]
SLC27A2	solute carrier family 27 member 2 [Source:HGNC Symbol;Acc:HGNC:10996]
SLC27A3	solute carrier family 27 member 3 [Source:HGNC Symbol;Acc:HGNC:10997]
SLC27A4	solute carrier family 27 member 4 [Source:HGNC Symbol;Acc:HGNC:10998]
SLC27A5	solute carrier family 27 member 5 [Source:HGNC Symbol;Acc:HGNC:10999]
SLC27A6	solute carrier family 27 member 6 [Source:HGNC Symbol;Acc:HGNC:11000]
FABP1	fatty acid binding protein 1 [Source:HGNC Symbol;Acc:HGNC:3555]
FABP2	fatty acid binding protein 2 [Source:HGNC Symbol;Acc:HGNC:3556]

FABP3	fatty acid binding protein 3 [Source:HGNC Symbol;Acc:HGNC:3557]
FABP5	fatty acid binding protein 5 [Source:HGNC Symbol;Acc:HGNC:3560]
FABP6	fatty acid binding protein 6 [Source:HGNC Symbol;Acc:HGNC:3561]
FABP7	fatty acid binding protein 7 [Source:HGNC Symbol;Acc:HGNC:3562]
PMP2	peripheral myelin protein 2 [Source:HGNC Symbol;Acc:HGNC:9117]
FABP9	fatty acid binding protein 9 [Source:HGNC Symbol;Acc:HGNC:3563]
FABP12	fatty acid binding protein 12 [Source:HGNC Symbol;Acc:HGNC:34524]
SDHB	succinate dehydrogenase complex iron sulfur subunit B [Source:HGNC Symbol;Acc:HGNC:10681]
SDHA	succinate dehydrogenase complex flavoprotein subunit A [Source:HGNC Symbol;Acc:HGNC:10680]
POLG	polymerase (DNA) gamma, catalytic subunit [Source:HGNC Symbol;Acc:HGNC:9179]
SLC16A4	solute carrier family 16 member 4 [Source:HGNC Symbol;Acc:HGNC:10925]
GYS1	glycogen synthase 1 [Source:HGNC Symbol;Acc:HGNC:4706]
PYGB	phosphorylase, glycogen; brain [Source:HGNC Symbol;Acc:HGNC:9723]
PYGL	phosphorylase, glycogen, liver [Source:HGNC Symbol;Acc:HGNC:9725]
PYGM	phosphorylase, glycogen, muscle [Source:HGNC Symbol;Acc:HGNC:9726]
LGALS1	galectin 1 [Source:HGNC Symbol;Acc:HGNC:6561]
CA1	carbonic anhydrase 1 [Source:HGNC Symbol;Acc:HGNC:1368]
EGLN2	egl-9 family hypoxia inducible factor 2 [Source:HGNC Symbol;Acc:HGNC:14660]
LDHC	lactate dehydrogenase C [Source:HGNC Symbol;Acc:HGNC:6544]
LDHD	lactate dehydrogenase D [Source:HGNC Symbol;Acc:HGNC:19708]
ALDH1A1	aldehyde dehydrogenase 1 family member A1 [Source:HGNC Symbol;Acc:HGNC:402]
CA10	carbonic anhydrase 10 [Source:HGNC Symbol;Acc:HGNC:1369]
CA11	carbonic anhydrase 11 [Source:HGNC Symbol;Acc:HGNC:1370]
CA12	carbonic anhydrase 12 [Source:HGNC Symbol;Acc:HGNC:1371]
CA9	carbonic anhydrase 9 [Source:HGNC Symbol;Acc:HGNC:1383]
CA8	carbonic anhydrase 8 [Source:HGNC Symbol;Acc:HGNC:1382]
CA7	carbonic anhydrase 7 [Source:HGNC Symbol;Acc:HGNC:1381]
CA6	carbonic anhydrase 6 [Source:HGNC Symbol;Acc:HGNC:1380]
CA5B	carbonic anhydrase 5B [Source:HGNC Symbol;Acc:HGNC:1378]
CA5A	carbonic anhydrase 5A [Source:HGNC Symbol;Acc:HGNC:1377]
CA13	carbonic anhydrase 13 [Source:HGNC Symbol;Acc:HGNC:14914]
CA14	carbonic anhydrase 14 [Source:HGNC Symbol;Acc:HGNC:1372]
CA2	carbonic anhydrase 2 [Source:HGNC Symbol;Acc:HGNC:1373]
CA3	carbonic anhydrase 3 [Source:HGNC Symbol;Acc:HGNC:1374]
CA4	carbonic anhydrase 4 [Source:HGNC Symbol;Acc:HGNC:1375]
PHGDH	phosphoglycerate dehydrogenase [Source:HGNC Symbol;Acc:HGNC:8923]
FAHD1	fumarylacetoacetate hydrolase domain containing 1 [Source:HGNC Symbol;Acc:HGNC:14169]
PROX1	prospero homeobox 1 [Source:HGNC Symbol;Acc:HGNC:9459]
EP300	E1A binding protein p300 [Source:HGNC Symbol;Acc:HGNC:3373]
RHOJ	ras homolog family member J [Source:HGNC Symbol;Acc:HGNC:688]

UQCRQ	ubiquinol-cytochrome c reductase complex III subunit VII [Source:HGNC Symbol;Acc:HGNC:29594]
P2RY1	purinergic receptor P2Y1 [Source:HGNC Symbol;Acc:HGNC:8539]
DDAH1	dimethylarginine dimethylaminohydrolase 1 [Source:HGNC Symbol;Acc:HGNC:2715]
CYBB	cytochrome b-245 beta chain [Source:HGNC Symbol;Acc:HGNC:2578]
SLC2A4	solute carrier family 2 member 4 [Source:HGNC Symbol;Acc:HGNC:11009]
FLT4	fms related tyrosine kinase 4 [Source:HGNC Symbol;Acc:HGNC:3767]
NRP1	neuropilin 1 [Source:HGNC Symbol;Acc:HGNC:8004]
FOXO6	forkhead box O6 [Source:HGNC Symbol;Acc:HGNC:24814]
GLS2	glutaminase 2 [Source:HGNC Symbol;Acc:HGNC:29570]
IL17A	interleukin 17A [Source:HGNC Symbol;Acc:HGNC:5981]
FXN	frataxin [Source:HGNC Symbol;Acc:HGNC:3951]
MAGEE1	MAGE family member E1 [Source:HGNC Symbol;Acc:HGNC:24934]
CXCL1	C-X-C motif chemokine ligand 1 [Source:HGNC Symbol;Acc:HGNC:4602]
FLT1	fms related tyrosine kinase 1 [Source:HGNC Symbol;Acc:HGNC:3763]
GCNT2	glucosaminyl (N-acetyl) transferase 2, I-branching enzyme (I blood group) [Source:HGNC Symbol;Acc:HGNC:4204]
SLC7A1	solute carrier family 7 member 1 [Source:HGNC Symbol;Acc:HGNC:11057]
ASS1	argininosuccinate synthase 1 [Source:HGNC Symbol;Acc:HGNC:758]
ASL	argininosuccinate lyase [Source:HGNC Symbol;Acc:HGNC:746]
NOX4	NADPH oxidase 4 [Source:HGNC Symbol;Acc:HGNC:7891]
NOX5	NADPH oxidase, EF-hand calcium binding domain 5 [Source:HGNC Symbol;Acc:HGNC:14874]
ACSM3	acyl-CoA synthetase medium-chain family member 3 [Source:HGNC Symbol;Acc:HGNC:10522]
GLO1	glyoxalase I [Source:HGNC Symbol;Acc:HGNC:4323]
GLRX2	glutaredoxin 2 [Source:HGNC Symbol;Acc:HGNC:16065]
ASNS	asparagine synthetase (glutamine-hydrolyzing) [Source:HGNC Symbol;Acc:HGNC:753]
ATF4	activating transcription factor 4 [Source:HGNC Symbol;Acc:HGNC:786]
CAT	catalase [Source:HGNC Symbol;Acc:HGNC:1516]
CBS	cystathionine-beta-synthase [Source:HGNC Symbol;Acc:HGNC:1550]
DHODH	dihydroorotate dehydrogenase (quinone) [Source:HGNC Symbol;Acc:HGNC:2867]
FAS	Fas cell surface death receptor [Source:HGNC Symbol;Acc:HGNC:11920]
GAD1	glutamate decarboxylase 1 [Source:HGNC Symbol;Acc:HGNC:4092]
RNF130	ring finger protein 130 [Source:HGNC Symbol;Acc:HGNC:18280]
GPX3	glutathione peroxidase 3 [Source:HGNC Symbol;Acc:HGNC:4555]
LPL	lipoprotein lipase [Source:HGNC Symbol;Acc:HGNC:6677]
MTHFR	methylenetetrahydrofolate reductase (NAD(P)H) [Source:HGNC Symbol;Acc:HGNC:7436]
NDRG3	NDRG family member 3 [Source:HGNC Symbol;Acc:HGNC:14462]
OXCT1	3-oxoacid CoA-transferase 1 [Source:HGNC Symbol;Acc:HGNC:8527]
PC	pyruvate carboxylase [Source:HGNC Symbol;Acc:HGNC:8636]
PDP1	pyruvate dehyrogenase phosphatase catalytic subunit 1 [Source:HGNC Symbol;Acc:HGNC:9279]
PLPP3	phospholipid phosphatase 3 [Source:HGNC Symbol;Acc:HGNC:9229]
PRDX1	peroxiredoxin 1 [Source:HGNC Symbol;Acc:HGNC:9352]

AHCY	adenosylhomocysteinase [Source:HGNC Symbol;Acc:HGNC:343]
SORD	sorbitol dehydrogenase [Source:HGNC Symbol;Acc:HGNC:11184]
TKT	transketolase [Source:HGNC Symbol;Acc:HGNC:11834]
UBIAD1	UbiA prenyltransferase domain containing 1 [Source:HGNC Symbol;Acc:HGNC:30791]
SLC35A2	solute carrier family 35 member A2 [Source:HGNC Symbol;Acc:HGNC:11022]
ACACA	acetyl-CoA carboxylase alpha [Source:HGNC Symbol;Acc:HGNC:84]
JMJD8	jumonji domain containing 8 [Source:HGNC Symbol;Acc:HGNC:14148]
NOX1	NADPH oxidase 1 [Source:HGNC Symbol;Acc:HGNC:7889]
FGFR1	fibroblast growth factor receptor 1 [Source:HGNC Symbol;Acc:HGNC:3688]
FGFR3	fibroblast growth factor receptor 3 [Source:HGNC Symbol;Acc:HGNC:3690]
VEGFB	vascular endothelial growth factor B [Source:HGNC Symbol;Acc:HGNC:12681]
KLK15	kallikrein related peptidase 15 [Source:HGNC Symbol;Acc:HGNC:20453]
SCD	stearoyl-CoA desaturase [Source:HGNC Symbol;Acc:HGNC:10571]
SLC2A2	solute carrier family 2 member 2 [Source:HGNC Symbol;Acc:HGNC:11006]
SLC2A5	solute carrier family 2 member 5 [Source:HGNC Symbol;Acc:HGNC:11010]
SLC2A6	solute carrier family 2 member 6 [Source:HGNC Symbol;Acc:HGNC:11011]
SLC2A7	solute carrier family 2 member 7 [Source:HGNC Symbol;Acc:HGNC:13445]
SLC2A9	solute carrier family 2 member 9 [Source:HGNC Symbol;Acc:HGNC:13446]
SLC2A8	solute carrier family 2 member 8 [Source:HGNC Symbol;Acc:HGNC:13812]
SLC2A11	solute carrier family 2 member 11 [Source:HGNC Symbol;Acc:HGNC:14239]
SLC2A12	solute carrier family 2 member 12 [Source:HGNC Symbol;Acc:HGNC:18067]
SLC2A14	solute carrier family 2 member 14 [Source:HGNC Symbol;Acc:HGNC:18301]
ACO2	aconitase 2 [Source:HGNC Symbol;Acc:HGNC:118]
RBPJ	recombination signal binding protein for immunoglobulin kappa J region [Source:HGNC Symbol;Acc:HGNC:5724]
ARG1	arginase 1 [Source:HGNC Symbol;Acc:HGNC:663]
APOA1	apolipoprotein A1 [Source:HGNC Symbol;Acc:HGNC:600]
GABPA	GA binding protein transcription factor alpha subunit [Source:HGNC Symbol;Acc:HGNC:4071]
NFE2	nuclear factor, erythroid 2 [Source:HGNC Symbol;Acc:HGNC:7780]
ICAM4	intercellular adhesion molecule 4 (Landsteiner-Wiener blood group) [Source:HGNC Symbol;Acc:HGNC:5347]
ICAM5	intercellular adhesion molecule 5 [Source:HGNC Symbol;Acc:HGNC:5348]
FGF1	fibroblast growth factor 1 [Source:HGNC Symbol;Acc:HGNC:3665]
SLC7A5	solute carrier family 7 member 5 [Source:HGNC Symbol;Acc:HGNC:11063]
XBP1	X-box binding protein 1 [Source:HGNC Symbol;Acc:HGNC:12801]
BCL2L13	BCL2 like 13 [Source:HGNC Symbol;Acc:HGNC:17164]
TOMM7	translocase of outer mitochondrial membrane 7 [Source:HGNC Symbol;Acc:HGNC:21648]
UBA52	ubiquitin A-52 residue ribosomal protein fusion product 1 [Source:HGNC Symbol;Acc:HGNC:12458]
RPS27A	ribosomal protein S27a [Source:HGNC Symbol;Acc:HGNC:10417]
UBB	ubiquitin B [Source:HGNC Symbol;Acc:HGNC:12463]
UBC	ubiquitin C [Source:HGNC Symbol;Acc:HGNC:12468]

MFN1	mitofusin 1 [Source:HGNC Symbol;Acc:HGNC:18262]
MFN2	mitofusin 2 [Source:HGNC Symbol;Acc:HGNC:16877]
RHOT1	ras homolog family member T1 [Source:HGNC Symbol;Acc:HGNC:21168]
RHOT2	ras homolog family member T2 [Source:HGNC Symbol;Acc:HGNC:21169]
USP8	ubiquitin specific peptidase 8 [Source:HGNC Symbol;Acc:HGNC:12631]
USP15	ubiquitin specific peptidase 15 [Source:HGNC Symbol;Acc:HGNC:12613]
USP30	ubiquitin specific peptidase 30 [Source:HGNC Symbol;Acc:HGNC:20065]
TAX1BP1	Tax1 binding protein 1 [Source:HGNC Symbol;Acc:HGNC:11575]
MAP1LC3B2	microtubule associated protein 1 light chain 3 beta 2 [Source:HGNC Symbol;Acc:HGNC:34390]
MITF	melanogenesis associated transcription factor [Source:HGNC Symbol;Acc:HGNC:7105]
TFE3	transcription factor binding to IGHM enhancer 3 [Source:HGNC Symbol;Acc:HGNC:11752]
BECN2	beclin 2 [Source:HGNC Symbol;Acc:HGNC:38606]
CSNK2A1	casein kinase 2 alpha 1 [Source:HGNC Symbol;Acc:HGNC:2457]
CSNK2A2	casein kinase 2 alpha 2 [Source:HGNC Symbol;Acc:HGNC:2459]
CSNK2A3	casein kinase 2 alpha 3 [Source:HGNC Symbol;Acc:HGNC:2458]
CSNK2B	casein kinase 2 beta [Source:HGNC Symbol;Acc:HGNC:2460]
E2F1	E2F transcription factor 1 [Source:HGNC Symbol;Acc:HGNC:3113]
RELA	RELA proto-oncogene, NF-kB subunit [Source:HGNC Symbol;Acc:HGNC:9955]
MRAS	muscle RAS oncogene homolog [Source:HGNC Symbol;Acc:HGNC:7227]
RRAS	related RAS viral (r-ras) oncogene homolog [Source:HGNC Symbol;Acc:HGNC:10447]
RRAS2	related RAS viral (r-ras) oncogene homolog 2 [Source:HGNC Symbol;Acc:HGNC:17271]
CITED2	Cbp/p300 interacting transactivator with Glu/Asp rich carboxy-terminal domain 2 [Source:HGNC Symbol;Acc:HGNC:1987]
SP1	Sp1 transcription factor [Source:HGNC Symbol;Acc:HGNC:11205]
TBC1D15	TBC1 domain family member 15 [Source:HGNC Symbol;Acc:HGNC:25694]
TBC1D17	TBC1 domain family member 17 [Source:HGNC Symbol;Acc:HGNC:25699]
RAB7B	RAB7B, member RAS oncogene family [Source:HGNC Symbol;Acc:HGNC:30513]
FIS1	fission, mitochondrial 1 [Source:HGNC Symbol;Acc:HGNC:21689]
ZEB2	zinc finger E-box binding homeobox 2 [Source:HGNC Symbol;Acc:HGNC:14881]
ILK	integrin linked kinase [Source:HGNC Symbol;Acc:HGNC:6040]
ZEB1	zinc finger E-box binding homeobox 1 [Source:HGNC Symbol;Acc:HGNC:11642]
TWIST1	twist family bHLH transcription factor 1 [Source:HGNC Symbol;Acc:HGNC:12428]
SNAI2	snail family transcriptional repressor 2 [Source:HGNC Symbol;Acc:HGNC:11094]
EPAS1	endothelial PAS domain protein 1 [Source:HGNC Symbol;Acc:HGNC:3374]
SNAI1	snail family transcriptional repressor 1 [Source:HGNC Symbol;Acc:HGNC:11128]
MET	MET proto-oncogene, receptor tyrosine kinase [Source:HGNC Symbol;Acc:HGNC:7029]
IRS1	insulin receptor substrate 1 [Source:HGNC Symbol;Acc:HGNC:6125]
IRS4	insulin receptor substrate 4 [Source:HGNC Symbol;Acc:HGNC:6128]
PIK3CD	phosphatidylinositol-4,5-bisphosphate 3-kinase catalytic subunit delta [Source:HGNC Symbol;Acc:HGNC:8977]
PIK3CB	phosphatidylinositol-4,5-bisphosphate 3-kinase catalytic subunit beta [Source:HGNC Symbol;Acc:HGNC:8976]

PIK3R1	phosphoinositide-3-kinase regulatory subunit 1 [Source:HGNC Symbol;Acc:HGNC:8979]
PIK3R2	phosphoinositide-3-kinase regulatory subunit 2 [Source:HGNC Symbol;Acc:HGNC:8980]
PIK3R3	phosphoinositide-3-kinase regulatory subunit 3 [Source:HGNC Symbol;Acc:HGNC:8981]
AKT2	AKT serine/threonine kinase 2 [Source:HGNC Symbol;Acc:HGNC:392]
AKT3	AKT serine/threonine kinase 3 [Source:HGNC Symbol;Acc:HGNC:393]
DEPTOR	DEP domain containing MTOR-interacting protein [Source:HGNC Symbol;Acc:HGNC:22953]
AKT1S1	AKT1 substrate 1 [Source:HGNC Symbol;Acc:HGNC:28426]
RPS6KB2	ribosomal protein S6 kinase B2 [Source:HGNC Symbol;Acc:HGNC:10437]
IGBP1	immunoglobulin (CD79A) binding protein 1 [Source:HGNC Symbol;Acc:HGNC:5461]
WIPI2	WD repeat domain, phosphoinositide interacting 2 [Source:HGNC Symbol;Acc:HGNC:32225]
SMCR8	Smith-Magenis syndrome chromosome region, candidate 8 [Source:HGNC Symbol;Acc:HGNC:17921]
WDR41	WD repeat domain 41 [Source:HGNC Symbol;Acc:HGNC:25601]
C9orf72	chromosome 9 open reading frame 72 [Source:HGNC Symbol;Acc:HGNC:28337]
RAB39B	RAB39B, member RAS oncogene family [Source:HGNC Symbol;Acc:HGNC:16499]
RAB1A	RAB1A, member RAS oncogene family [Source:HGNC Symbol;Acc:HGNC:9758]
PRKCD	protein kinase C delta [Source:HGNC Symbol;Acc:HGNC:9399]
TP53INP2	tumor protein p53 inducible nuclear protein 2 [Source:HGNC Symbol;Acc:HGNC:16104]
MAP3K7	mitogen-activated protein kinase kinase kinase 7 [Source:HGNC Symbol;Acc:HGNC:6859]
DAPK3	death associated protein kinase 3 [Source:HGNC Symbol;Acc:HGNC:2676]
DAPK2	death associated protein kinase 2 [Source:HGNC Symbol;Acc:HGNC:2675]
EIF2S1	eukaryotic translation initiation factor 2 subunit alpha [Source:HGNC Symbol;Acc:HGNC:3265]
MTMR4	myotubularin related protein 4 [Source:HGNC Symbol;Acc:HGNC:7452]
MTMR14	myotubularin related protein 14 [Source:HGNC Symbol;Acc:HGNC:26190]
CFLAR	CASP8 and FADD like apoptosis regulator [Source:HGNC Symbol;Acc:HGNC:1876]
PRKCQ	protein kinase C theta [Source:HGNC Symbol;Acc:HGNC:9410]
LPAR2	lysophosphatidic acid receptor 2 [Source:HGNC Symbol;Acc:HGNC:3168]
LPAR4	lysophosphatidic acid receptor 4 [Source:HGNC Symbol;Acc:HGNC:4478]
LPAR5	lysophosphatidic acid receptor 5 [Source:HGNC Symbol;Acc:HGNC:13307]
CXCL12	C-X-C motif chemokine ligand 12 [Source:HGNC Symbol;Acc:HGNC:10672]
CXCR4	C-X-C motif chemokine receptor 4 [Source:HGNC Symbol;Acc:HGNC:2561]
F2	coagulation factor II, thrombin [Source:HGNC Symbol;Acc:HGNC:3535]
F2R	coagulation factor II thrombin receptor [Source:HGNC Symbol;Acc:HGNC:3537]
FGF2	fibroblast growth factor 2 [Source:HGNC Symbol;Acc:HGNC:3676]
FGF3	fibroblast growth factor 3 [Source:HGNC Symbol;Acc:HGNC:3681]
FGF4	fibroblast growth factor 4 [Source:HGNC Symbol;Acc:HGNC:3682]
FGF17	fibroblast growth factor 17 [Source:HGNC Symbol;Acc:HGNC:3673]
FGF6	fibroblast growth factor 6 [Source:HGNC Symbol;Acc:HGNC:3684]
FGF7	fibroblast growth factor 7 [Source:HGNC Symbol;Acc:HGNC:3685]
FGF8	fibroblast growth factor 8 [Source:HGNC Symbol;Acc:HGNC:3686]
FGF9	fibroblast growth factor 9 [Source:HGNC Symbol;Acc:HGNC:3687]
FGF10	fibroblast growth factor 10 [Source:HGNC Symbol;Acc:HGNC:3666]

FGF16	fibroblast growth factor 16 [Source:HGNC Symbol;Acc:HGNC:3672]
FGF5	fibroblast growth factor 5 [Source:HGNC Symbol;Acc:HGNC:3683]
FGF18	fibroblast growth factor 18 [Source:HGNC Symbol;Acc:HGNC:3674]
FGF20	fibroblast growth factor 20 [Source:HGNC Symbol;Acc:HGNC:3677]
FGF22	fibroblast growth factor 22 [Source:HGNC Symbol;Acc:HGNC:3679]
FGF19	fibroblast growth factor 19 [Source:HGNC Symbol;Acc:HGNC:3675]
FGF21	fibroblast growth factor 21 [Source:HGNC Symbol;Acc:HGNC:3678]
FGF23	fibroblast growth factor 23 [Source:HGNC Symbol;Acc:HGNC:3680]
INSRR	insulin receptor related receptor [Source:HGNC Symbol;Acc:HGNC:6093]
FGFR2	fibroblast growth factor receptor 2 [Source:HGNC Symbol;Acc:HGNC:3689]
FGFR4	fibroblast growth factor receptor 4 [Source:HGNC Symbol;Acc:HGNC:3691]
FN1	fibronectin 1 [Source:HGNC Symbol;Acc:HGNC:3778]
ITGA1	integrin subunit alpha 1 [Source:HGNC Symbol;Acc:HGNC:6134]
ITGA3	integrin subunit alpha 3 [Source:HGNC Symbol;Acc:HGNC:6139]
ITGA7	integrin subunit alpha 7 [Source:HGNC Symbol;Acc:HGNC:6143]
ITGA10	integrin subunit alpha 10 [Source:HGNC Symbol;Acc:HGNC:6135]
ITGA11	integrin subunit alpha 11 [Source:HGNC Symbol;Acc:HGNC:6136]
ITGAX	integrin subunit alpha X [Source:HGNC Symbol;Acc:HGNC:6152]
ITGAD	integrin subunit alpha D [Source:HGNC Symbol;Acc:HGNC:6146]
ITGAE	integrin subunit alpha E [Source:HGNC Symbol;Acc:HGNC:6147]
ITGB4	integrin subunit beta 4 [Source:HGNC Symbol;Acc:HGNC:6158]
ITGB6	integrin subunit beta 6 [Source:HGNC Symbol;Acc:HGNC:6161]
KNG1	kininogen 1 [Source:HGNC Symbol;Acc:HGNC:6383]
BDKRB1	bradykinin receptor B1 [Source:HGNC Symbol;Acc:HGNC:1029]
BDKRB2	bradykinin receptor B2 [Source:HGNC Symbol;Acc:HGNC:1030]
CHRM1	cholinergic receptor muscarinic 1 [Source:HGNC Symbol;Acc:HGNC:1950]
CHRM2	cholinergic receptor muscarinic 2 [Source:HGNC Symbol;Acc:HGNC:1951]
CHRM3	cholinergic receptor muscarinic 3 [Source:HGNC Symbol;Acc:HGNC:1952]
CHRM4	cholinergic receptor muscarinic 4 [Source:HGNC Symbol;Acc:HGNC:1953]
CHRM5	cholinergic receptor muscarinic 5 [Source:HGNC Symbol;Acc:HGNC:1954]
GNA12	G protein subunit alpha 12 [Source:HGNC Symbol;Acc:HGNC:4380]
GNA13	G protein subunit alpha 13 [Source:HGNC Symbol;Acc:HGNC:4381]
GNG12	G protein subunit gamma 12 [Source:HGNC Symbol;Acc:HGNC:19663]
FGD1	FYVE, RhoGEF and PH domain containing 1 [Source:HGNC Symbol;Acc:HGNC:3663]
FGD3	FYVE, RhoGEF and PH domain containing 3 [Source:HGNC Symbol;Acc:HGNC:16027]
PTK2	protein tyrosine kinase 2 [Source:HGNC Symbol;Acc:HGNC:9611]
BCAR1	BCAR1, Cas family scaffolding protein [Source:HGNC Symbol;Acc:HGNC:971]
CRK	v-crk avian sarcoma virus CT10 oncogene homolog [Source:HGNC Symbol;Acc:HGNC:2362]
CRKL	v-crk avian sarcoma virus CT10 oncogene homolog-like [Source:HGNC Symbol;Acc:HGNC:2363]
DOCK1	dedicator of cytokinesis 1 [Source:HGNC Symbol;Acc:HGNC:2987]
ARHGEF6	Rac/Cdc42 guanine nucleotide exchange factor 6 [Source:HGNC Symbol;Acc:HGNC:685]

VAV3	vav guanine nucleotide exchange factor 3 [Source:HGNC Symbol;Acc:HGNC:12659]
VAV1	vav guanine nucleotide exchange factor 1 [Source:HGNC Symbol;Acc:HGNC:12657]
VAV2	vav guanine nucleotide exchange factor 2 [Source:HGNC Symbol;Acc:HGNC:12658]
ARAF	A-Raf proto-oncogene, serine/threonine kinase [Source:HGNC Symbol;Acc:HGNC:646]
MOS	v-mos Moloney murine sarcoma viral oncogene homolog [Source:HGNC Symbol;Acc:HGNC:7199]
ARHGEF1	Rho guanine nucleotide exchange factor 1 [Source:HGNC Symbol;Acc:HGNC:681]
ARHGEF12	Rho guanine nucleotide exchange factor 12 [Source:HGNC Symbol;Acc:HGNC:14193]
ARHGAP35	Rho GTPase activating protein 35 [Source:HGNC Symbol;Acc:HGNC:4591]
RAC2	ras-related C3 botulinum toxin substrate 2 (rho family, small GTP binding protein Rac2) [Source:HGNC Symbol;Acc:HGNC:9802]
RAC3	ras-related C3 botulinum toxin substrate 3 (rho family, small GTP binding protein Rac3) [Source:HGNC Symbol;Acc:HGNC:9803]
PAK1	p21 (RAC1) activated kinase 1 [Source:HGNC Symbol;Acc:HGNC:8590]
PAK2	p21 (RAC1) activated kinase 2 [Source:HGNC Symbol;Acc:HGNC:8591]
PAK3	p21 (RAC1) activated kinase 3 [Source:HGNC Symbol;Acc:HGNC:8592]
PAK4	p21 (RAC1) activated kinase 4 [Source:HGNC Symbol;Acc:HGNC:16059]
PAK5	p21 (RAC1) activated kinase 5 [Source:HGNC Symbol;Acc:HGNC:15916]
PAK6	p21 (RAC1) activated kinase 6 [Source:HGNC Symbol;Acc:HGNC:16061]
BUB1B-PAK6	BUB1B-PAK6 readthrough [Source:EntrezGene;Acc:106821730]
ARHGEF7	Rho guanine nucleotide exchange factor 7 [Source:HGNC Symbol;Acc:HGNC:15607]
GIT1	GIT ArfGAP 1 [Source:HGNC Symbol;Acc:HGNC:4272]
MYLK	myosin light chain kinase [Source:HGNC Symbol;Acc:HGNC:7590]
MYLK2	myosin light chain kinase 2 [Source:HGNC Symbol;Acc:HGNC:16243]
MYLK3	myosin light chain kinase 3 [Source:HGNC Symbol;Acc:HGNC:29826]
MYLK4	myosin light chain kinase family member 4 [Source:HGNC Symbol;Acc:HGNC:27972]
PPP1CA	protein phosphatase 1 catalytic subunit alpha [Source:HGNC Symbol;Acc:HGNC:9281]
PPP1CB	protein phosphatase 1 catalytic subunit beta [Source:HGNC Symbol;Acc:HGNC:9282]
PPP1CC	protein phosphatase 1 catalytic subunit gamma [Source:HGNC Symbol;Acc:HGNC:9283]
PPP1R12A	protein phosphatase 1 regulatory subunit 12A [Source:HGNC Symbol;Acc:HGNC:7618]
PPP1R12B	protein phosphatase 1 regulatory subunit 12B [Source:HGNC Symbol;Acc:HGNC:7619]
PPP1R12C	protein phosphatase 1 regulatory subunit 12C [Source:HGNC Symbol;Acc:HGNC:14947]
MYL5	myosin light chain 5 [Source:HGNC Symbol;Acc:HGNC:7586]
MYL7	myosin light chain 7 [Source:HGNC Symbol;Acc:HGNC:21719]
MYL10	myosin light chain 10 [Source:HGNC Symbol;Acc:HGNC:29825]
MYLPF	myosin light chain, phosphorylatable, fast skeletal muscle [Source:HGNC Symbol;Acc:HGNC:29824]
DIAPH1	diaphanous related formin 1 [Source:HGNC Symbol;Acc:HGNC:2876]
DIAPH2	diaphanous related formin 2 [Source:HGNC Symbol;Acc:HGNC:2877]
SLC9A1	solute carrier family 9 member A1 [Source:HGNC Symbol;Acc:HGNC:11071]
PIP5K1C	phosphatidylinositol-4-phosphate 5-kinase type 1 gamma [Source:HGNC Symbol;Acc:HGNC:8996]
PIP5K1A	phosphatidylinositol-4-phosphate 5-kinase type 1 alpha [Source:HGNC Symbol;Acc:HGNC:8994]

PIP5K1B	phosphatidylinositol-4-phosphate 5-kinase type 1 beta [Source:HGNC Symbol;Acc:HGNC:8995]
PIP4K2C	phosphatidylinositol-5-phosphate 4-kinase type 2 gamma [Source:HGNC Symbol;Acc:HGNC:23786]
PIP4K2A	phosphatidylinositol-5-phosphate 4-kinase type 2 alpha [Source:HGNC Symbol;Acc:HGNC:8997]
PIP4K2B	phosphatidylinositol-5-phosphate 4-kinase type 2 beta [Source:HGNC Symbol;Acc:HGNC:8998]
PIKFYVE	phosphoinositide kinase, FYVE-type zinc finger containing [Source:HGNC Symbol;Acc:HGNC:23785]
LIMK1	LIM domain kinase 1 [Source:HGNC Symbol;Acc:HGNC:6613]
LIMK2	LIM domain kinase 2 [Source:HGNC Symbol;Acc:HGNC:6614]
DIAPH3	diaphanous related formin 3 [Source:HGNC Symbol;Acc:HGNC:15480]
BAIAP2	BAI1 associated protein 2 [Source:HGNC Symbol;Acc:HGNC:947]
ENAH	enabled homolog (Drosophila) [Source:HGNC Symbol;Acc:HGNC:18271]
WASL	Wiskott-Aldrich syndrome like [Source:HGNC Symbol;Acc:HGNC:12735]
WASF2	WAS protein family member 2 [Source:HGNC Symbol;Acc:HGNC:12733]
CYFIP1	cytoplasmic FMR1 interacting protein 1 [Source:HGNC Symbol;Acc:HGNC:13759]
CYFIP2	cytoplasmic FMR1 interacting protein 2 [Source:HGNC Symbol;Acc:HGNC:13760]
NCKAP1	NCK associated protein 1 [Source:HGNC Symbol;Acc:HGNC:7666]
NCKAP1L	NCK associated protein 1 like [Source:HGNC Symbol;Acc:HGNC:4862]
ABI2	abl interactor 2 [Source:HGNC Symbol;Acc:HGNC:24011]
BRK1	BRICK1, SCAR/WAVE actin nucleating complex subunit [Source:HGNC Symbol;Acc:HGNC:23057]
WASF1	WAS protein family member 1 [Source:HGNC Symbol;Acc:HGNC:12732]
PFN3	profilin 3 [Source:HGNC Symbol;Acc:HGNC:18627]
PFN1	profilin 1 [Source:HGNC Symbol;Acc:HGNC:8881]
PFN2	profilin 2 [Source:HGNC Symbol;Acc:HGNC:8882]
PFN4	profilin family member 4 [Source:HGNC Symbol;Acc:HGNC:31103]
PXN	paxillin [Source:HGNC Symbol;Acc:HGNC:9718]
TMSB4X	thymosin beta 4, X-linked [Source:HGNC Symbol;Acc:HGNC:11881]
TMSB4Y	thymosin beta 4, Y-linked [Source:HGNC Symbol;Acc:HGNC:11882]
CFL1	cofilin 1 [Source:HGNC Symbol;Acc:HGNC:1874]
CFL2	cofilin 2 [Source:HGNC Symbol;Acc:HGNC:1875]
SSH1	slingshot protein phosphatase 1 [Source:HGNC Symbol;Acc:HGNC:30579]
SSH3	slingshot protein phosphatase 3 [Source:HGNC Symbol;Acc:HGNC:30581]
SSH2	slingshot protein phosphatase 2 [Source:HGNC Symbol;Acc:HGNC:30580]
VCL	vinculin [Source:HGNC Symbol;Acc:HGNC:12665]
IQGAP1	IQ motif containing GTPase activating protein 1 [Source:HGNC Symbol;Acc:HGNC:6110]
IQGAP2	IQ motif containing GTPase activating protein 2 [Source:HGNC Symbol;Acc:HGNC:6111]
IQGAP3	IQ motif containing GTPase activating protein 3 [Source:HGNC Symbol;Acc:HGNC:20669]
GSN	gelsolin [Source:HGNC Symbol;Acc:HGNC:4620]
SCIN	scinderin [Source:HGNC Symbol;Acc:HGNC:21695]
APC	adenomatous polyposis coli [Source:HGNC Symbol;Acc:HGNC:583]
APC2	adenomatosis polyposis coli 2 [Source:HGNC Symbol;Acc:HGNC:24036]
ARHGEF4	Rho guanine nucleotide exchange factor 4 [Source:HGNC Symbol;Acc:HGNC:684]

SPATA13	spermatogenesis associated 13 [Source:HGNC Symbol;Acc:HGNC:23222]
COL1A1	collagen type I alpha 1 [Source:HGNC Symbol;Acc:HGNC:2197]
COL1A2	collagen type I alpha 2 chain [Source:HGNC Symbol;Acc:HGNC:2198]
COL2A1	collagen type II alpha 1 chain [Source:HGNC Symbol;Acc:HGNC:2200]
COL4A2	collagen type IV alpha 2 [Source:HGNC Symbol;Acc:HGNC:2203]
COL4A4	collagen type IV alpha 4 chain [Source:HGNC Symbol;Acc:HGNC:2206]
COL4A6	collagen type IV alpha 6 chain [Source:HGNC Symbol;Acc:HGNC:2208]
COL4A1	collagen type IV alpha 1 chain [Source:HGNC Symbol;Acc:HGNC:2202]
COL4A5	collagen type IV alpha 5 chain [Source:HGNC Symbol;Acc:HGNC:2207]
COL4A3	collagen type IV alpha 3 chain [Source:HGNC Symbol;Acc:HGNC:2204]
COL6A1	collagen type VI alpha 1 [Source:HGNC Symbol;Acc:HGNC:2211]
COL6A2	collagen type VI alpha 2 [Source:HGNC Symbol;Acc:HGNC:2212]
COL6A3	collagen type VI alpha 3 chain [Source:HGNC Symbol;Acc:HGNC:2213]
COL6A6	collagen type VI alpha 6 [Source:HGNC Symbol;Acc:HGNC:27023]
COL6A5	collagen type VI alpha 5 [Source:HGNC Symbol;Acc:HGNC:26674]
COL9A1	collagen type IX alpha 1 [Source:HGNC Symbol;Acc:HGNC:2217]
COL9A2	collagen type IX alpha 2 [Source:HGNC Symbol;Acc:HGNC:2218]
COL9A3	collagen type IX alpha 3 [Source:HGNC Symbol;Acc:HGNC:2219]
LAMA1	laminin subunit alpha 1 [Source:HGNC Symbol;Acc:HGNC:6481]
LAMA2	laminin subunit alpha 2 [Source:HGNC Symbol;Acc:HGNC:6482]
LAMA3	laminin subunit alpha 3 [Source:HGNC Symbol;Acc:HGNC:6483]
LAMA5	laminin subunit alpha 5 [Source:HGNC Symbol;Acc:HGNC:6485]
LAMA4	laminin subunit alpha 4 [Source:HGNC Symbol;Acc:HGNC:6484]
LAMB1	laminin subunit beta 1 [Source:HGNC Symbol;Acc:HGNC:6486]
LAMB2	laminin subunit beta 2 [Source:HGNC Symbol;Acc:HGNC:6487]
LAMB3	laminin subunit beta 3 [Source:HGNC Symbol;Acc:HGNC:6490]
LAMB4	laminin subunit beta 4 [Source:HGNC Symbol;Acc:HGNC:6491]
LAMC1	laminin subunit gamma 1 [Source:HGNC Symbol;Acc:HGNC:6492]
LAMC2	laminin subunit gamma 2 [Source:HGNC Symbol;Acc:HGNC:6493]
LAMC3	laminin subunit gamma 3 [Source:HGNC Symbol;Acc:HGNC:6494]
CHAD	chondroadherin [Source:HGNC Symbol;Acc:HGNC:1909]
RELN	reelin [Source:HGNC Symbol;Acc:HGNC:9957]
COMP	cartilage oligomeric matrix protein [Source:HGNC Symbol;Acc:HGNC:2227]
THBS2	thrombospondin 2 [Source:HGNC Symbol;Acc:HGNC:11786]
THBS3	thrombospondin 3 [Source:HGNC Symbol;Acc:HGNC:11787]
THBS4	thrombospondin 4 [Source:HGNC Symbol;Acc:HGNC:11788]
SPP1	secreted phosphoprotein 1 [Source:HGNC Symbol;Acc:HGNC:11255]
VTN	vitronectin [Source:HGNC Symbol;Acc:HGNC:12724]
TNC	tenascin C [Source:HGNC Symbol;Acc:HGNC:5318]
TNN	tenascin N [Source:HGNC Symbol;Acc:HGNC:22942]
TNR	tenascin R [Source:HGNC Symbol;Acc:HGNC:11953]

TNXB	tenascin XB [Source:HGNC Symbol;Acc:HGNC:11976]
IBSP	integrin binding sialoprotein [Source:HGNC Symbol;Acc:HGNC:5341]
IGF1	insulin like growth factor 1 [Source:HGNC Symbol;Acc:HGNC:5464]
PGF	placental growth factor [Source:HGNC Symbol;Acc:HGNC:8893]
VEGFC	vascular endothelial growth factor C [Source:HGNC Symbol;Acc:HGNC:12682]
VEGFD	vascular endothelial growth factor D [Source:HGNC Symbol;Acc:HGNC:3708]
HGF	hepatocyte growth factor [Source:HGNC Symbol;Acc:HGNC:4893]
ARHGAP5	Rho GTPase activating protein 5 [Source:HGNC Symbol;Acc:HGNC:675]
RASGRF1	Ras protein specific guanine nucleotide releasing factor 1 [Source:HGNC Symbol;Acc:HGNC:9875]
CAPN2	calpain 2 [Source:HGNC Symbol;Acc:HGNC:1479]
TLN1	talin 1 [Source:HGNC Symbol;Acc:HGNC:11845]
TLN2	talin 2 [Source:HGNC Symbol;Acc:HGNC:15447]
FLNA	filamin A [Source:HGNC Symbol;Acc:HGNC:3754]
FLNC	filamin C [Source:HGNC Symbol;Acc:HGNC:3756]
FLNB	filamin B [Source:HGNC Symbol;Acc:HGNC:3755]
ZYX	zyxin [Source:HGNC Symbol;Acc:HGNC:13200]
PARVB	parvin beta [Source:HGNC Symbol;Acc:HGNC:14653]
PARVA	parvin alpha [Source:HGNC Symbol;Acc:HGNC:14652]
PARVG	parvin gamma [Source:HGNC Symbol;Acc:HGNC:14654]
RAPGEF1	Rap guanine nucleotide exchange factor 1 [Source:HGNC Symbol;Acc:HGNC:4568]
RAP1B	RAP1B, member of RAS oncogene family [Source:HGNC Symbol;Acc:HGNC:9857]
CAV2	caveolin 2 [Source:HGNC Symbol;Acc:HGNC:1528]
CAV3	caveolin 3 [Source:HGNC Symbol;Acc:HGNC:1529]
FYN	FYN proto-oncogene, Src family tyrosine kinase [Source:HGNC Symbol;Acc:HGNC:4037]
SHC1	SHC adaptor protein 1 [Source:HGNC Symbol;Acc:HGNC:10840]
SHC2	SHC adaptor protein 2 [Source:HGNC Symbol;Acc:HGNC:29869]
SHC3	SHC adaptor protein 3 [Source:HGNC Symbol;Acc:HGNC:18181]
SHC4	SHC adaptor protein 4 [Source:HGNC Symbol;Acc:HGNC:16743]
ELK1	ELK1, ETS transcription factor [Source:HGNC Symbol;Acc:HGNC:3321]
CCND2	cyclin D2 [Source:HGNC Symbol;Acc:HGNC:1583]
CCND3	cyclin D3 [Source:HGNC Symbol;Acc:HGNC:1585]
BIRC2	baculoviral IAP repeat containing 2 [Source:HGNC Symbol;Acc:HGNC:590]
BIRC3	baculoviral IAP repeat containing 3 [Source:HGNC Symbol;Acc:HGNC:591]
NECTIN4	nectin cell adhesion molecule 4 [Source:HGNC Symbol;Acc:HGNC:19688]
FARP2	FERM, ARH/RhoGEF and pleckstrin domain protein 2 [Source:HGNC Symbol;Acc:HGNC:16460]
WASF3	WAS protein family member 3 [Source:HGNC Symbol;Acc:HGNC:12734]
LMO7	LIM domain 7 [Source:HGNC Symbol;Acc:HGNC:6646]
SSX2IP	SSX family member 2 interacting protein [Source:HGNC Symbol;Acc:HGNC:16509]
SORBS1	sorbin and SH3 domain containing 1 [Source:HGNC Symbol;Acc:HGNC:14565]
CTNND1	catenin delta 1 [Source:HGNC Symbol;Acc:HGNC:2515]
CTNNA3	catenin alpha 3 [Source:HGNC Symbol;Acc:HGNC:2511]

CTNNA2	catenin alpha 2 [Source:HGNC Symbol;Acc:HGNC:2510]
ACP1	acid phosphatase 1, soluble [Source:HGNC Symbol;Acc:HGNC:122]
PTPRB	protein tyrosine phosphatase, receptor type B [Source:HGNC Symbol;Acc:HGNC:9665]
PTPN1	protein tyrosine phosphatase, non-receptor type 1 [Source:HGNC Symbol;Acc:HGNC:9642]
PTPN6	protein tyrosine phosphatase, non-receptor type 6 [Source:HGNC Symbol;Acc:HGNC:9658]
PTPRJ	protein tyrosine phosphatase, receptor type J [Source:HGNC Symbol;Acc:HGNC:9673]
TCF7	transcription factor 7 (T-cell specific, HMG-box) [Source:HGNC Symbol;Acc:HGNC:11639]
TCF7L1	transcription factor 7 like 1 [Source:HGNC Symbol;Acc:HGNC:11640]
TCF7L2	transcription factor 7 like 2 [Source:HGNC Symbol;Acc:HGNC:11641]
LEF1	lymphoid enhancer binding factor 1 [Source:HGNC Symbol;Acc:HGNC:6551]
INSR	insulin receptor [Source:HGNC Symbol;Acc:HGNC:6091]
FER	FER tyrosine kinase [Source:HGNC Symbol;Acc:HGNC:3655]
YES1	YES proto-oncogene 1, Src family tyrosine kinase [Source:HGNC Symbol;Acc:HGNC:12841]
TGFBR1	transforming growth factor beta receptor 1 [Source:HGNC Symbol;Acc:HGNC:11772]
TGFBR2	transforming growth factor beta receptor 2 [Source:HGNC Symbol;Acc:HGNC:11773]
CREBBP	CREB binding protein [Source:HGNC Symbol;Acc:HGNC:2348]
NLK	nemo like kinase [Source:HGNC Symbol;Acc:HGNC:29858]
PGLS	6-phosphogluconolactonase [Source:HGNC Symbol;Acc:HGNC:8903]
H6PD	hexose-6-phosphate dehydrogenase/glucose 1-dehydrogenase [Source:HGNC Symbol;Acc:HGNC:4795]
PGD	phosphogluconate dehydrogenase [Source:HGNC Symbol;Acc:HGNC:8891]
RPE	ribulose-5-phosphate-3-epimerase [Source:HGNC Symbol;Acc:HGNC:10293]
RPEL1	ribulose-5-phosphate-3-epimerase-like 1 [Source:HGNC Symbol;Acc:HGNC:45241]
TKTL2	transketolase like 2 [Source:HGNC Symbol;Acc:HGNC:25313]
TKTL1	transketolase like 1 [Source:HGNC Symbol;Acc:HGNC:11835]
TALDO1	transaldolase 1 [Source:HGNC Symbol;Acc:HGNC:11559]
RPIA	ribose 5-phosphate isomerase A [Source:HGNC Symbol;Acc:HGNC:10297]
DERA	deoxyribose-phosphate aldolase [Source:HGNC Symbol;Acc:HGNC:24269]
RBKS	ribokinase [Source:HGNC Symbol;Acc:HGNC:30325]
PGM1	phosphoglucomutase 1 [Source:HGNC Symbol;Acc:HGNC:8905]
PGM2	phosphoglucomutase 2 [Source:HGNC Symbol;Acc:HGNC:8906]
PRPS1L1	phosphoribosyl pyrophosphate synthetase 1-like 1 [Source:HGNC Symbol;Acc:HGNC:9463]
PRPS2	phosphoribosyl pyrophosphate synthetase 2 [Source:HGNC Symbol;Acc:HGNC:9465]
PRPS1	phosphoribosyl pyrophosphate synthetase 1 [Source:HGNC Symbol;Acc:HGNC:9462]
RGN	regucalcin [Source:HGNC Symbol;Acc:HGNC:9989]
IDNK	IDNK, gluconokinase [Source:HGNC Symbol;Acc:HGNC:31367]
GLYCTK	glycerate kinase [Source:HGNC Symbol;Acc:HGNC:24247]
ALDOB	aldolase, fructose-bisphosphate B [Source:HGNC Symbol;Acc:HGNC:417]
FBP1	fructose-bisphosphatase 1 [Source:HGNC Symbol;Acc:HGNC:3606]
FBP2	fructose-bisphosphatase 2 [Source:HGNC Symbol;Acc:HGNC:3607]
PFKP	phosphofructokinase, platelet [Source:HGNC Symbol;Acc:HGNC:8878]
PFKL	phosphofructokinase, liver type [Source:HGNC Symbol;Acc:HGNC:8876]

ACSS1	acyl-CoA synthetase short-chain family member 1 [Source:HGNC Symbol;Acc:HGNC:16091]
ACSS2	acyl-CoA synthetase short-chain family member 2 [Source:HGNC Symbol;Acc:HGNC:15814]
PDHA2	pyruvate dehydrogenase (lipoamide) alpha 2 [Source:HGNC Symbol;Acc:HGNC:8807]
PDHA1	pyruvate dehydrogenase (lipoamide) alpha 1 [Source:HGNC Symbol;Acc:HGNC:8806]
PDHB	pyruvate dehydrogenase (lipoamide) beta [Source:HGNC Symbol;Acc:HGNC:8808]
DLAT	dihydrolipoamide S-acetyltransferase [Source:HGNC Symbol;Acc:HGNC:2896]
DLD	dihydrolipoamide dehydrogenase [Source:HGNC Symbol;Acc:HGNC:2898]
ADH1A	alcohol dehydrogenase 1A (class I), alpha polypeptide [Source:HGNC Symbol;Acc:HGNC:249]
ADH1B	alcohol dehydrogenase 1B (class I), beta polypeptide [Source:HGNC Symbol;Acc:HGNC:250]
ADH1C	alcohol dehydrogenase 1C (class I), gamma polypeptide [Source:HGNC Symbol;Acc:HGNC:251]
ADH7	alcohol dehydrogenase 7 (class IV), mu or sigma polypeptide [Source:HGNC Symbol;Acc:HGNC:256]
ADH4	alcohol dehydrogenase 4 (class II), pi polypeptide [Source:HGNC Symbol;Acc:HGNC:252]
ADH6	alcohol dehydrogenase 6 (class V) [Source:HGNC Symbol;Acc:HGNC:255]
ADH5	alcohol dehydrogenase 5 (class III), chi polypeptide [Source:HGNC Symbol;Acc:HGNC:253]
AKR1A1	aldo-keto reductase family 1 member A1 [Source:HGNC Symbol;Acc:HGNC:380]
PKLR	pyruvate kinase, liver and RBC [Source:HGNC Symbol;Acc:HGNC:9020]
ACACB	acetyl-CoA carboxylase beta [Source:HGNC Symbol;Acc:HGNC:85]
ACYP2	acylphosphatase 2 [Source:HGNC Symbol;Acc:HGNC:180]
ACYP1	acylphosphatase 1 [Source:HGNC Symbol;Acc:HGNC:179]
ALDH2	aldehyde dehydrogenase 2 family (mitochondrial) [Source:HGNC Symbol;Acc:HGNC:404]
ALDH3A2	aldehyde dehydrogenase 3 family member A2 [Source:HGNC Symbol;Acc:HGNC:403]
ALDH1B1	aldehyde dehydrogenase 1 family member B1 [Source:HGNC Symbol;Acc:HGNC:407]
ALDH7A1	aldehyde dehydrogenase 7 family member A1 [Source:HGNC Symbol;Acc:HGNC:877]
ALDH9A1	aldehyde dehydrogenase 9 family member A1 [Source:HGNC Symbol;Acc:HGNC:412]
ACOT12	acyl-CoA thioesterase 12 [Source:HGNC Symbol;Acc:HGNC:24436]
LDHAL6A	lactate dehydrogenase A like 6A [Source:HGNC Symbol;Acc:HGNC:28335]
LDHAL6B	lactate dehydrogenase A like 6B [Source:HGNC Symbol;Acc:HGNC:21481]
HAGH	hydroxyacylglutathione hydrolase [Source:HGNC Symbol;Acc:HGNC:4805]
GRHPR	glyoxylate reductase/hydroxypyruvate reductase [Source:HGNC Symbol;Acc:HGNC:4570]
ME2	malic enzyme 2 [Source:HGNC Symbol;Acc:HGNC:6984]
ME3	malic enzyme 3 [Source:HGNC Symbol;Acc:HGNC:6985]
ME1	malic enzyme 1 [Source:HGNC Symbol;Acc:HGNC:6983]
MDH1	malate dehydrogenase 1 [Source:HGNC Symbol;Acc:HGNC:6970]
FH	fumarate hydratase [Source:HGNC Symbol;Acc:HGNC:3700]
PCK1	phosphoenolpyruvate carboxykinase 1 [Source:HGNC Symbol;Acc:HGNC:8724]
PCK2	phosphoenolpyruvate carboxykinase 2, mitochondrial [Source:HGNC Symbol;Acc:HGNC:8725]
ACAT2	acetyl-CoA acetyltransferase 2 [Source:HGNC Symbol;Acc:HGNC:94]
ACAT1	acetyl-CoA acetyltransferase 1 [Source:HGNC Symbol;Acc:HGNC:93]
GCK	glucokinase [Source:HGNC Symbol;Acc:HGNC:4195]
SCO2	SCO2 cytochrome c oxidase assembly protein [Source:HGNC Symbol;Acc:HGNC:10604]
RET	ret proto-oncogene [Source:HGNC Symbol;Acc:HGNC:9967]

NTRK1	neurotrophic receptor tyrosine kinase 1 [Source:HGNC Symbol;Acc:HGNC:8031]
NTRK3	neurotrophic receptor tyrosine kinase 3 [Source:HGNC Symbol;Acc:HGNC:8033]
FLT3	fms related tyrosine kinase 3 [Source:HGNC Symbol;Acc:HGNC:3765]
HKDC1	hexokinase domain containing 1 [Source:HGNC Symbol;Acc:HGNC:23302]
PGAM1	phosphoglycerate mutase 1 [Source:HGNC Symbol;Acc:HGNC:8888]
PGAM2	phosphoglycerate mutase 2 [Source:HGNC Symbol;Acc:HGNC:8889]
PGAM4	phosphoglycerate mutase family member 4 [Source:HGNC Symbol;Acc:HGNC:21731]
TIGAR	TP53 induced glycolysis regulatory phosphatase [Source:HGNC Symbol;Acc:HGNC:1185]
CS	citrate synthase [Source:HGNC Symbol;Acc:HGNC:2422]
ACLY	ATP citrate lyase [Source:HGNC Symbol;Acc:HGNC:115]
ACO1	aconitase 1 [Source:HGNC Symbol;Acc:HGNC:117]
IDH3B	isocitrate dehydrogenase 3 (NAD(+)) beta [Source:HGNC Symbol;Acc:HGNC:5385]
IDH3G	isocitrate dehydrogenase 3 (NAD(+)) gamma [Source:HGNC Symbol;Acc:HGNC:5386]
IDH3A	isocitrate dehydrogenase 3 (NAD(+)) alpha [Source:HGNC Symbol;Acc:HGNC:5384]
OGDHL	oxoglutarate dehydrogenase-like [Source:HGNC Symbol;Acc:HGNC:25590]
OGDH	oxoglutarate dehydrogenase [Source:HGNC Symbol;Acc:HGNC:8124]
DLST	dihydrolipoamide S-succinyltransferase [Source:HGNC Symbol;Acc:HGNC:2911]
SUCLG1	succinate-CoA ligase alpha subunit [Source:HGNC Symbol;Acc:HGNC:11449]
SUCLG2	succinate-CoA ligase GDP-forming beta subunit [Source:HGNC Symbol;Acc:HGNC:11450]
SUCLA2	succinate-CoA ligase ADP-forming beta subunit [Source:HGNC Symbol;Acc:HGNC:11448]
SDHC	succinate dehydrogenase complex subunit C [Source:HGNC Symbol;Acc:HGNC:10682]
SDHD	succinate dehydrogenase complex subunit D [Source:HGNC Symbol;Acc:HGNC:10683]
TPI1	triosephosphate isomerase 1 [Source:HGNC Symbol;Acc:HGNC:12009]
GAPDHS	glyceraldehyde-3-phosphate dehydrogenase, spermatogenic [Source:HGNC Symbol;Acc:HGNC:24864]
ENO3	enolase 3 [Source:HGNC Symbol;Acc:HGNC:3354]
ENO2	enolase 2 [Source:HGNC Symbol;Acc:HGNC:3353]
ENO4	enolase family member 4 [Source:HGNC Symbol;Acc:HGNC:31670]
ALDH3B1	aldehyde dehydrogenase 3 family member B1 [Source:HGNC Symbol;Acc:HGNC:410]
ALDH3B2	aldehyde dehydrogenase 3 family member B2 [Source:HGNC Symbol;Acc:HGNC:411]
ALDH3A1	aldehyde dehydrogenase 3 family member A1 [Source:HGNC Symbol;Acc:HGNC:405]
GALM	galactose mutarotase [Source:HGNC Symbol;Acc:HGNC:24063]
G6PC2	glucose-6-phosphatase catalytic subunit 2 [Source:HGNC Symbol;Acc:HGNC:28906]
G6PC3	glucose-6-phosphatase catalytic subunit 3 [Source:HGNC Symbol;Acc:HGNC:24861]
ADPGK	ADP dependent glucokinase [Source:HGNC Symbol;Acc:HGNC:25250]
BPGM	bisphosphoglycerate mutase [Source:HGNC Symbol;Acc:HGNC:1093]
MINPP1	multiple inositol-polyphosphate phosphatase 1 [Source:HGNC Symbol;Acc:HGNC:7102]
G6PC	glucose-6-phosphatase catalytic subunit [Source:HGNC Symbol;Acc:HGNC:4056]
MT-ND2	mitochondrially encoded NADH:ubiquinone oxidoreductase core subunit 2 [Source:HGNC Symbol;Acc:HGNC:7456]
MT-ND3	mitochondrially encoded NADH:ubiquinone oxidoreductase core subunit 3 [Source:HGNC Symbol;Acc:HGNC:7458]

MT-ND4	mitochondrially encoded NADH:ubiquinone oxidoreductase core subunit 4 [Source:HGNC Symbol;Acc:HGNC:7459]
MT-ND4L	mitochondrially encoded NADH:ubiquinone oxidoreductase core subunit 4L [Source:HGNC Symbol;Acc:HGNC:7460]
MT-ND5	mitochondrially encoded NADH:ubiquinone oxidoreductase core subunit 5 [Source:HGNC Symbol;Acc:HGNC:7461]
MT-ND6	mitochondrially encoded NADH:ubiquinone oxidoreductase core subunit 6 [Source:HGNC Symbol;Acc:HGNC:7462]
NDUFS1	NADH:ubiquinone oxidoreductase core subunit S1 [Source:HGNC Symbol;Acc:HGNC:7707]
NDUFS2	NADH:ubiquinone oxidoreductase core subunit S2 [Source:HGNC Symbol;Acc:HGNC:7708]
NDUFS3	NADH:ubiquinone oxidoreductase core subunit S3 [Source:HGNC Symbol;Acc:HGNC:7710]
NDUFS4	NADH:ubiquinone oxidoreductase subunit S4 [Source:HGNC Symbol;Acc:HGNC:7711]
NDUFS5	NADH:ubiquinone oxidoreductase subunit S5 [Source:HGNC Symbol;Acc:HGNC:7712]
NDUFS6	NADH:ubiquinone oxidoreductase subunit S6 [Source:HGNC Symbol;Acc:HGNC:7713]
NDUFS7	NADH:ubiquinone oxidoreductase core subunit S7 [Source:HGNC Symbol;Acc:HGNC:7714]
NDUFS8	NADH:ubiquinone oxidoreductase core subunit S8 [Source:HGNC Symbol;Acc:HGNC:7715]
NDUFV1	NADH:ubiquinone oxidoreductase core subunit V1 [Source:HGNC Symbol;Acc:HGNC:7716]
NDUFV2	NADH:ubiquinone oxidoreductase core subunit V2 [Source:HGNC Symbol;Acc:HGNC:7717]
NDUFV3	NADH:ubiquinone oxidoreductase subunit V3 [Source:HGNC Symbol;Acc:HGNC:7719]
NDUFA1	NADH:ubiquinone oxidoreductase subunit A1 [Source:HGNC Symbol;Acc:HGNC:7683]
NDUFA2	NADH:ubiquinone oxidoreductase subunit A2 [Source:HGNC Symbol;Acc:HGNC:7685]
NDUFA3	NADH:ubiquinone oxidoreductase subunit A3 [Source:HGNC Symbol;Acc:HGNC:7686]
NDUFA4	NDUFA4, mitochondrial complex associated [Source:HGNC Symbol;Acc:HGNC:7687]
NDUFA4L2	NADH dehydrogenase (ubiquinone) 1 alpha subcomplex, 4-like 2 [Source:HGNC Symbol;Acc:HGNC:29836]
NDUFA5	NADH:ubiquinone oxidoreductase subunit A5 [Source:HGNC Symbol;Acc:HGNC:7688]
NDUFA6	NADH:ubiquinone oxidoreductase subunit A6 [Source:HGNC Symbol;Acc:HGNC:7690]
NDUFA7	NADH:ubiquinone oxidoreductase subunit A7 [Source:HGNC Symbol;Acc:HGNC:7691]
NDUFA8	NADH:ubiquinone oxidoreductase subunit A8 [Source:HGNC Symbol;Acc:HGNC:7692]
NDUFA9	NADH:ubiquinone oxidoreductase subunit A9 [Source:HGNC Symbol;Acc:HGNC:7693]
NDUFA10	NADH:ubiquinone oxidoreductase subunit A10 [Source:HGNC Symbol;Acc:HGNC:7684]
NDUFAB1	NADH:ubiquinone oxidoreductase subunit AB1 [Source:HGNC Symbol;Acc:HGNC:7694]
NDUFA11	NADH:ubiquinone oxidoreductase subunit A11 [Source:HGNC Symbol;Acc:HGNC:20371]
NDUFA12	NADH:ubiquinone oxidoreductase subunit A12 [Source:HGNC Symbol;Acc:HGNC:23987]
NDUFA13	NADH:ubiquinone oxidoreductase subunit A13 [Source:HGNC Symbol;Acc:HGNC:17194]
NDUFB1	NADH:ubiquinone oxidoreductase subunit B1 [Source:HGNC Symbol;Acc:HGNC:7695]
NDUFB2	NADH:ubiquinone oxidoreductase subunit B2 [Source:HGNC Symbol;Acc:HGNC:7697]
NDUFB3	NADH:ubiquinone oxidoreductase subunit B3 [Source:HGNC Symbol;Acc:HGNC:7698]
NDUFB4	NADH:ubiquinone oxidoreductase subunit B4 [Source:HGNC Symbol;Acc:HGNC:7699]
NDUFB5	NADH:ubiquinone oxidoreductase subunit B5 [Source:HGNC Symbol;Acc:HGNC:7700]
NDUFB6	NADH:ubiquinone oxidoreductase subunit B6 [Source:HGNC Symbol;Acc:HGNC:7701]
NDUFB7	NADH:ubiquinone oxidoreductase subunit B7 [Source:HGNC Symbol;Acc:HGNC:7702]
NDUFB8	NADH:ubiquinone oxidoreductase subunit B8 [Source:HGNC Symbol;Acc:HGNC:7703]

NDUFB9	NADH:ubiquinone oxidoreductase subunit B9 [Source:HGNC Symbol;Acc:HGNC:7704]
NDUFB10	NADH:ubiquinone oxidoreductase subunit B10 [Source:HGNC Symbol;Acc:HGNC:7696]
NDUFB11	NADH:ubiquinone oxidoreductase subunit B11 [Source:HGNC Symbol;Acc:HGNC:20372]
NDUFC1	NADH:ubiquinone oxidoreductase subunit C1 [Source:HGNC Symbol;Acc:HGNC:7705]
NDUFC2	NADH:ubiquinone oxidoreductase subunit C2 [Source:HGNC Symbol;Acc:HGNC:7706]
NDUFC2-KCTD14	NDUFC2-KCTD14 readthrough [Source:HGNC Symbol;Acc:HGNC:42956]
UQCRFS1	ubiquinol-cytochrome c reductase, Rieske iron-sulfur polypeptide 1 [Source:HGNC Symbol;Acc:HGNC:12587]
MT-CYB	mitochondrially encoded cytochrome b [Source:HGNC Symbol;Acc:HGNC:7427]
CYC1	cytochrome c1 [Source:HGNC Symbol;Acc:HGNC:2579]
UQCRC1	ubiquinol-cytochrome c reductase core protein I [Source:HGNC Symbol;Acc:HGNC:12585]
UQCRC2	ubiquinol-cytochrome c reductase core protein II [Source:HGNC Symbol;Acc:HGNC:12586]
UQCRH	ubiquinol-cytochrome c reductase hinge protein [Source:HGNC Symbol;Acc:HGNC:12590]
UQCRHL	ubiquinol-cytochrome c reductase hinge protein like [Source:HGNC Symbol;Acc:HGNC:51714]
UQCRB	ubiquinol-cytochrome c reductase binding protein [Source:HGNC Symbol;Acc:HGNC:12582]
UQCR10	ubiquinol-cytochrome c reductase, complex III subunit X [Source:HGNC Symbol;Acc:HGNC:30863]
UQCR11	ubiquinol-cytochrome c reductase, complex III subunit XI [Source:HGNC Symbol;Acc:HGNC:30862]
COX10	COX10 heme A:farnesyltransferase cytochrome c oxidase assembly factor [Source:HGNC Symbol;Acc:HGNC:2260]
MT-CO3	mitochondrially encoded cytochrome c oxidase III [Source:HGNC Symbol;Acc:HGNC:7422]
COX4I2	cytochrome c oxidase subunit 4I2 [Source:HGNC Symbol;Acc:HGNC:16232]
COX4I1	cytochrome c oxidase subunit 4I1 [Source:HGNC Symbol;Acc:HGNC:2265]
COX5A	cytochrome c oxidase subunit 5A [Source:HGNC Symbol;Acc:HGNC:2267]
COX5B	cytochrome c oxidase subunit 5B [Source:HGNC Symbol;Acc:HGNC:2269]
COX6A1	cytochrome c oxidase subunit 6A1 [Source:HGNC Symbol;Acc:HGNC:2277]
COX6A2	cytochrome c oxidase subunit 6A2 [Source:HGNC Symbol;Acc:HGNC:2279]
COX6B1	cytochrome c oxidase subunit 6B1 [Source:HGNC Symbol;Acc:HGNC:2280]
COX6B2	cytochrome c oxidase subunit 6B2 [Source:HGNC Symbol;Acc:HGNC:24380]
COX6C	cytochrome c oxidase subunit 6C [Source:HGNC Symbol;Acc:HGNC:2285]
COX7A1	cytochrome c oxidase subunit 7A1 [Source:HGNC Symbol;Acc:HGNC:2287]
COX7A2	cytochrome c oxidase subunit 7A2 [Source:HGNC Symbol;Acc:HGNC:2288]
COX7A2L	cytochrome c oxidase subunit 7A2 like [Source:HGNC Symbol;Acc:HGNC:2289]
COX7B	cytochrome c oxidase subunit 7B [Source:HGNC Symbol;Acc:HGNC:2291]
COX7B2	cytochrome c oxidase subunit 7B2 [Source:HGNC Symbol;Acc:HGNC:24381]
COX7C	cytochrome c oxidase subunit 7C [Source:HGNC Symbol;Acc:HGNC:2292]
COX8C	cytochrome c oxidase subunit 8C [Source:HGNC Symbol;Acc:HGNC:24382]
COX8A	cytochrome c oxidase subunit 8A [Source:HGNC Symbol;Acc:HGNC:2294]
COX11	COX11 cytochrome c oxidase copper chaperone [Source:HGNC Symbol;Acc:HGNC:2261]
COX15	COX15 cytochrome c oxidase assembly homolog [Source:HGNC Symbol;Acc:HGNC:2263]
COX17	COX17 cytochrome c oxidase copper chaperone [Source:HGNC Symbol;Acc:HGNC:2264]

CYCS	cytochrome c, somatic [Source:HGNC Symbol;Acc:HGNC:19986]
MT-ATP6	mitochondrially encoded ATP synthase 6 [Source:HGNC Symbol;Acc:HGNC:7414]
MT-ATP8	mitochondrially encoded ATP synthase 8 [Source:HGNC Symbol;Acc:HGNC:7415]
ATP6V1A	ATPase H+ transporting V1 subunit A [Source:HGNC Symbol;Acc:HGNC:851]
ATP6V1B1	ATPase H+ transporting V1 subunit B1 [Source:HGNC Symbol;Acc:HGNC:853]
ATP6V1B2	ATPase H+ transporting V1 subunit B2 [Source:HGNC Symbol;Acc:HGNC:854]
ATP6V1C2	ATPase H+ transporting V1 subunit C2 [Source:HGNC Symbol;Acc:HGNC:18264]
ATP6V1C1	ATPase H+ transporting V1 subunit C1 [Source:HGNC Symbol;Acc:HGNC:856]
ATP6V1D	ATPase H+ transporting V1 subunit D [Source:HGNC Symbol;Acc:HGNC:13527]
ATP6V1E2	ATPase H+ transporting V1 subunit E2 [Source:HGNC Symbol;Acc:HGNC:18125]
ATP6V1E1	ATPase H+ transporting V1 subunit E1 [Source:HGNC Symbol;Acc:HGNC:857]
ATP6V1F	ATPase H+ transporting V1 subunit F [Source:HGNC Symbol;Acc:HGNC:16832]
ATP6V1G1	ATPase H+ transporting V1 subunit G1 [Source:HGNC Symbol;Acc:HGNC:864]
ATP6V1G3	ATPase H+ transporting V1 subunit G3 [Source:HGNC Symbol;Acc:HGNC:18265]
ATP6V1G2	ATPase H+ transporting V1 subunit G2 [Source:HGNC Symbol;Acc:HGNC:862]
ATP6V1H	ATPase H+ transporting V1 subunit H [Source:HGNC Symbol;Acc:HGNC:18303]
TCIRG1	T-cell immune regulator 1, ATPase H+ transporting V0 subunit a3 [Source:HGNC Symbol;Acc:HGNC:11647]
ATP6V0A2	ATPase H+ transporting V0 subunit a2 [Source:HGNC Symbol;Acc:HGNC:18481]
ATP6V0A4	ATPase H+ transporting V0 subunit a4 [Source:HGNC Symbol;Acc:HGNC:866]
ATP6V0A1	ATPase H+ transporting V0 subunit a1 [Source:HGNC Symbol;Acc:HGNC:865]
ATP6V0C	ATPase H+ transporting V0 subunit c [Source:HGNC Symbol;Acc:HGNC:855]
ATP6V0B	ATPase H+ transporting V0 subunit b [Source:HGNC Symbol;Acc:HGNC:861]
ATP6V0D1	ATPase H+ transporting V0 subunit d1 [Source:HGNC Symbol;Acc:HGNC:13724]
ATP6V0D2	ATPase H+ transporting V0 subunit d2 [Source:HGNC Symbol;Acc:HGNC:18266]
ATP6V0E1	ATPase H+ transporting V0 subunit e1 [Source:HGNC Symbol;Acc:HGNC:863]
ATP6V0E2	ATPase H+ transporting V0 subunit e2 [Source:HGNC Symbol;Acc:HGNC:21723]
ATP6AP1	ATPase H+ transporting accessory protein 1 [Source:HGNC Symbol;Acc:HGNC:868]
ATP4A	ATPase H+/K+ transporting alpha subunit [Source:HGNC Symbol;Acc:HGNC:819]
ATP4B	ATPase H+/K+ transporting beta subunit [Source:HGNC Symbol;Acc:HGNC:820]
ATP12A	ATPase H+/K+ transporting non-gastric alpha2 subunit [Source:HGNC Symbol;Acc:HGNC:13816]
PPA2	pyrophosphatase (inorganic) 2 [Source:HGNC Symbol;Acc:HGNC:28883]
PPA1	pyrophosphatase (inorganic) 1 [Source:HGNC Symbol;Acc:HGNC:9226]
LHPP	phospholysine phosphohistidine inorganic pyrophosphate phosphatase [Source:HGNC Symbol;Acc:HGNC:30042]
ATP5A1	ATP synthase, H+ transporting, mitochondrial F1 complex, alpha subunit 1, cardiac muscle [Source:HGNC Symbol;Acc:HGNC:823]
ATP5B	ATP synthase, H+ transporting, mitochondrial F1 complex, beta polypeptide [Source:HGNC Symbol;Acc:HGNC:830]
ATP5C1	ATP synthase, H+ transporting, mitochondrial F1 complex, gamma polypeptide 1 [Source:HGNC Symbol;Acc:HGNC:833]
ATP5D	ATP synthase, H+ transporting, mitochondrial F1 complex, delta subunit [Source:HGNC Symbol;Acc:HGNC:837]

ATP5E	ATP synthase, H+ transporting, mitochondrial F1 complex, epsilon subunit [Source:HGNC Symbol;Acc:HGNC:838]
ATP5G1	ATP synthase, H+ transporting, mitochondrial Fo complex subunit C1 (subunit 9) [Source:HGNC Symbol;Acc:HGNC:841]
ATP5G2	ATP synthase, H+ transporting, mitochondrial Fo complex subunit C2 (subunit 9) [Source:HGNC Symbol;Acc:HGNC:842]
ATP5G3	ATP synthase, H+ transporting, mitochondrial Fo complex subunit C3 (subunit 9) [Source:HGNC Symbol;Acc:HGNC:843]
ATP5I	ATP synthase, H+ transporting, mitochondrial Fo complex subunit E [Source:HGNC Symbol;Acc:HGNC:846]
ATP5J2	ATP synthase, H+ transporting, mitochondrial Fo complex subunit F2 [Source:HGNC Symbol;Acc:HGNC:848]
ATP5L	ATP synthase, H+ transporting, mitochondrial Fo complex subunit G [Source:HGNC Symbol;Acc:HGNC:14247]
ATP5F1	ATP synthase, H+ transporting, mitochondrial Fo complex subunit B1 [Source:HGNC Symbol;Acc:HGNC:840]
ATP5H	ATP synthase, H+ transporting, mitochondrial Fo complex subunit D [Source:HGNC Symbol;Acc:HGNC:845]
ATP5J	ATP synthase, H+ transporting, mitochondrial Fo complex subunit F6 [Source:HGNC Symbol;Acc:HGNC:847]
ATP5O	ATP synthase, H+ transporting, mitochondrial F1 complex, O subunit [Source:HGNC Symbol;Acc:HGNC:850]
MT-CO1	mitochondrially encoded cytochrome c oxidase I [Source:HGNC Symbol;Acc:HGNC:7419]
MT-CO2	mitochondrially encoded cytochrome c oxidase II [Source:HGNC Symbol;Acc:HGNC:7421]
MT-ND1	mitochondrially encoded NADH:ubiquinone oxidoreductase core subunit 1 [Source:HGNC Symbol;Acc:HGNC:7455]
MGAM	maltase-glucoamylase [Source:HGNC Symbol;Acc:HGNC:7043]
MGAM2	maltase-glucoamylase 2 (putative) [Source:HGNC Symbol;Acc:HGNC:28101]
GAA	glucosidase alpha, acid [Source:HGNC Symbol;Acc:HGNC:4065]
GANC	glucosidase alpha, neutral C [Source:HGNC Symbol;Acc:HGNC:4139]
SI	sucrase-isomaltase [Source:HGNC Symbol;Acc:HGNC:10856]
UGP2	UDP-glucose pyrophosphorylase 2 [Source:HGNC Symbol;Acc:HGNC:12527]
ENPP1	ectonucleotide pyrophosphatase/phosphodiesterase 1 [Source:HGNC Symbol;Acc:HGNC:3356]
ENPP3	ectonucleotide pyrophosphatase/phosphodiesterase 3 [Source:HGNC Symbol;Acc:HGNC:3358]
GYS2	glycogen synthase 2 [Source:HGNC Symbol;Acc:HGNC:4707]
GYG1	glycogenin 1 [Source:HGNC Symbol;Acc:HGNC:4699]
GYG2	glycogenin 2 [Source:HGNC Symbol;Acc:HGNC:4700]
GBE1	glucan (1,4-alpha-), branching enzyme 1 [Source:HGNC Symbol;Acc:HGNC:4180]
AGL	amylo-alpha-1, 6-glucosidase, 4-alpha-glucanotransferase [Source:HGNC Symbol;Acc:HGNC:321]
AMY1C	amylase, alpha 1C (salivary) [Source:HGNC Symbol;Acc:HGNC:476]
AMY2A	amylase, alpha 2A (pancreatic) [Source:HGNC Symbol;Acc:HGNC:477]
AMY1A	amylase, alpha 1A (salivary) [Source:HGNC Symbol;Acc:HGNC:474]
AMY2B	amylase, alpha 2B (pancreatic) [Source:HGNC Symbol;Acc:HGNC:478]
AMY1B	amylase, alpha 1B (salivary) [Source:HGNC Symbol;Acc:HGNC:475]
TREH	trehalase [Source:HGNC Symbol;Acc:HGNC:12266]
PGM2L1	phosphoglucomutase 2-like 1 [Source:HGNC Symbol;Acc:HGNC:20898]

GBA	glucosylceramidase beta [Source:HGNC Symbol;Acc:HGNC:4177]
CYP27A1	cytochrome P450 family 27 subfamily A member 1 [Source:HGNC Symbol;Acc:HGNC:2605]
CYP7A1	cytochrome P450 family 7 subfamily A member 1 [Source:HGNC Symbol;Acc:HGNC:2651]
LCAT	lecithin-cholesterol acyltransferase [Source:HGNC Symbol;Acc:HGNC:6522]
LIPA	lipase A, lysosomal acid type [Source:HGNC Symbol;Acc:HGNC:6617]
APOE	apolipoprotein E [Source:HGNC Symbol;Acc:HGNC:613]
LRP1	LDL receptor related protein 1 [Source:HGNC Symbol;Acc:HGNC:6692]
ABCA1	ATP binding cassette subfamily A member 1 [Source:HGNC Symbol;Acc:HGNC:29]
ABCB11	ATP binding cassette subfamily B member 11 [Source:HGNC Symbol;Acc:HGNC:42]
ABCG5	ATP binding cassette subfamily G member 5 [Source:HGNC Symbol;Acc:HGNC:13886]
ABCG8	ATP binding cassette subfamily G member 8 [Source:HGNC Symbol;Acc:HGNC:13887]
TSPO	translocator protein [Source:HGNC Symbol;Acc:HGNC:1158]
VDAC1	voltage dependent anion channel 1 [Source:HGNC Symbol;Acc:HGNC:12669]
VAPA	VAMP associated protein A [Source:HGNC Symbol;Acc:HGNC:12648]
LRP2	LDL receptor related protein 2 [Source:HGNC Symbol;Acc:HGNC:6694]
APOA2	apolipoprotein A2 [Source:HGNC Symbol;Acc:HGNC:601]
APOC3	apolipoprotein C3 [Source:HGNC Symbol;Acc:HGNC:610]
APOA4	apolipoprotein A4 [Source:HGNC Symbol;Acc:HGNC:602]
PLTP	phospholipid transfer protein [Source:HGNC Symbol;Acc:HGNC:9093]
ANGPTL4	angiopoietin like 4 [Source:HGNC Symbol;Acc:HGNC:16039]
LPA	lipoprotein(a) [Source:HGNC Symbol;Acc:HGNC:6667]
VAPB	VAMP (vesicle-associated membrane protein)-associated protein B and C [Source:HGNC Symbol;Acc:HGNC:12649]
NPC1	NPC intracellular cholesterol transporter 1 [Source:HGNC Symbol;Acc:HGNC:7897]
SORT1	sortilin 1 [Source:HGNC Symbol;Acc:HGNC:11186]
LDLR	low density lipoprotein receptor [Source:HGNC Symbol;Acc:HGNC:6547]
LDLRAP1	low density lipoprotein receptor adaptor protein 1 [Source:HGNC Symbol;Acc:HGNC:18640]
PCSK9	proprotein convertase subtilisin/kexin type 9 [Source:HGNC Symbol;Acc:HGNC:20001]
NPC2	NPC intracellular cholesterol transporter 2 [Source:HGNC Symbol;Acc:HGNC:14537]
SCARB1	scavenger receptor class B member 1 [Source:HGNC Symbol;Acc:HGNC:1664]
NCEH1	neutral cholesterol ester hydrolase 1 [Source:HGNC Symbol;Acc:HGNC:29260]
APOB	apolipoprotein B [Source:HGNC Symbol;Acc:HGNC:603]
VDAC2	voltage dependent anion channel 2 [Source:HGNC Symbol;Acc:HGNC:12672]
VDAC3	voltage dependent anion channel 3 [Source:HGNC Symbol;Acc:HGNC:12674]
CETP	cholesteryl ester transfer protein [Source:HGNC Symbol;Acc:HGNC:1869]
STAR	steroidogenic acute regulatory protein [Source:HGNC Symbol;Acc:HGNC:11359]
APOH	apolipoprotein H [Source:HGNC Symbol;Acc:HGNC:616]
OSBPL5	oxysterol binding protein like 5 [Source:HGNC Symbol;Acc:HGNC:16392]
LIPC	lipase C, hepatic type [Source:HGNC Symbol;Acc:HGNC:6619]
LIPG	lipase G, endothelial type [Source:HGNC Symbol;Acc:HGNC:6623]
APOC1	apolipoprotein C1 [Source:HGNC Symbol;Acc:HGNC:607]
APOC2	apolipoprotein C2 [Source:HGNC Symbol;Acc:HGNC:609]

ANGPTL3	angiopoietin like 3 [Source:HGNC Symbol;Acc:HGNC:491]
ANGPTL8	angiopoietin like 8 [Source:HGNC Symbol;Acc:HGNC:24933]
LRPAP1	LDL receptor related protein associated protein 1 [Source:HGNC Symbol;Acc:HGNC:6701]
STARD3	StAR related lipid transfer domain containing 3 [Source:HGNC Symbol;Acc:HGNC:17579]
MYLIP	myosin regulatory light chain interacting protein [Source:HGNC Symbol;Acc:HGNC:21155]
SOAT1	sterol O-acyltransferase 1 [Source:HGNC Symbol;Acc:HGNC:11177]
AKR1C4	aldo-keto reductase family 1 member C4 [Source:HGNC Symbol;Acc:HGNC:387]
HSD17B1	hydroxysteroid 17-beta dehydrogenase 1 [Source:HGNC Symbol;Acc:HGNC:5210]
HSD3B1	hydroxy-delta-5-steroid dehydrogenase, 3 beta- and steroid delta-isomerase 1 [Source:HGNC Symbol;Acc:HGNC:5217]
HSD11B2	hydroxysteroid 11-beta dehydrogenase 2 [Source:HGNC Symbol;Acc:HGNC:5209]
AKR1C2	aldo-keto reductase family 1 member C2 [Source:HGNC Symbol;Acc:HGNC:385]
AKR1C1	aldo-keto reductase family 1 member C1 [Source:HGNC Symbol;Acc:HGNC:384]
AKR1D1	aldo-keto reductase family 1 member D1 [Source:HGNC Symbol;Acc:HGNC:388]
CYP11B1	cytochrome P450 family 11 subfamily B member 1 [Source:HGNC Symbol;Acc:HGNC:2591]
CYP11A1	cytochrome P450 family 11 subfamily A member 1 [Source:HGNC Symbol;Acc:HGNC:2590]
COMT	catechol-O-methyltransferase [Source:HGNC Symbol;Acc:HGNC:2228]
SULT1E1	sulfotransferase family 1E member 1 [Source:HGNC Symbol;Acc:HGNC:11377]
STS	steroid sulfatase (microsomal), isozyme S [Source:HGNC Symbol;Acc:HGNC:11425]
AKR1C3	aldo-keto reductase family 1 member C3 [Source:HGNC Symbol;Acc:HGNC:386]
CYP1A1	cytochrome P450 family 1 subfamily A member 1 [Source:HGNC Symbol;Acc:HGNC:2595]
CYP1A2	cytochrome P450 family 1 subfamily A member 2 [Source:HGNC Symbol;Acc:HGNC:2596]
CYP1B1	cytochrome P450 family 1 subfamily B member 1 [Source:HGNC Symbol;Acc:HGNC:2597]
CYP2B6	cytochrome P450 family 2 subfamily B member 6 [Source:HGNC Symbol;Acc:HGNC:2615]
CYP2D6	cytochrome P450 family 2 subfamily D member 6 [Source:HGNC Symbol;Acc:HGNC:2625]
CYP2E1	cytochrome P450 family 2 subfamily E member 1 [Source:HGNC Symbol;Acc:HGNC:2631]
CYP11B2	cytochrome P450 family 11 subfamily B member 2 [Source:HGNC Symbol;Acc:HGNC:2592]
HSD17B3	hydroxysteroid 17-beta dehydrogenase 3 [Source:HGNC Symbol;Acc:HGNC:5212]
SRD5A1	steroid 5 alpha-reductase 1 [Source:HGNC Symbol;Acc:HGNC:11284]
SRD5A2	steroid 5 alpha-reductase 2 [Source:HGNC Symbol;Acc:HGNC:11285]
SRD5A3	steroid 5 alpha-reductase 3 [Source:HGNC Symbol;Acc:HGNC:25812]
HSD17B2	hydroxysteroid 17-beta dehydrogenase 2 [Source:HGNC Symbol;Acc:HGNC:5211]
HSD17B6	hydroxysteroid 17-beta dehydrogenase 6 [Source:HGNC Symbol;Acc:HGNC:23316]
HSD17B8	hydroxysteroid 17-beta dehydrogenase 8 [Source:HGNC Symbol;Acc:HGNC:3554]
HSD17B7	hydroxysteroid 17-beta dehydrogenase 7 [Source:HGNC Symbol;Acc:HGNC:5215]
HSD11B1	hydroxysteroid 11-beta dehydrogenase 1 [Source:HGNC Symbol;Acc:HGNC:5208]
CYP3A4	cytochrome P450 family 3 subfamily A member 4 [Source:HGNC Symbol;Acc:HGNC:2637]
CYP3A5	cytochrome P450 family 3 subfamily A member 5 [Source:HGNC Symbol;Acc:HGNC:2638]
CYP3A7	cytochrome P450 family 3 subfamily A member 7 [Source:HGNC Symbol;Acc:HGNC:2640]
DHRS11	dehydrogenase/reductase 11 [Source:HGNC Symbol;Acc:HGNC:28639]
HSD17B12	hydroxysteroid 17-beta dehydrogenase 12 [Source:HGNC Symbol;Acc:HGNC:18646]
CYP19A1	cytochrome P450 family 19 subfamily A member 1 [Source:HGNC Symbol;Acc:HGNC:2594]

CYP17A1	cytochrome P450 family 17 subfamily A member 1 [Source:HGNC Symbol;Acc:HGNC:2593]
CYP21A2	cytochrome P450 family 21 subfamily A member 2 [Source:HGNC Symbol;Acc:HGNC:2600]
CYP2C9	cytochrome P450 family 2 subfamily C member 9 [Source:HGNC Symbol;Acc:HGNC:2623]
CYP2C18	cytochrome P450 family 2 subfamily C member 18 [Source:HGNC Symbol;Acc:HGNC:2620]
CYP2C19	cytochrome P450 family 2 subfamily C member 19 [Source:HGNC Symbol;Acc:HGNC:2621]
CYP2C8	cytochrome P450 family 2 subfamily C member 8 [Source:HGNC Symbol;Acc:HGNC:2622]
CYP3A43	cytochrome P450 family 3 subfamily A member 43 [Source:HGNC Symbol;Acc:HGNC:17450]
HADH	hydroxyacyl-CoA dehydrogenase [Source:HGNC Symbol;Acc:HGNC:4799]
ACADS	acyl-CoA dehydrogenase, C-2 to C-3 short chain [Source:HGNC Symbol;Acc:HGNC:90]
ACADM	acyl-CoA dehydrogenase, C-4 to C-12 straight chain [Source:HGNC Symbol;Acc:HGNC:89]
ACADL	acyl-CoA dehydrogenase, long chain [Source:HGNC Symbol;Acc:HGNC:88]
MCAT	malonyl-CoA-acyl carrier protein transacylase [Source:HGNC Symbol;Acc:HGNC:29622]
PPT1	palmitoyl-protein thioesterase 1 [Source:HGNC Symbol;Acc:HGNC:9325]
GPR3	G protein-coupled receptor 3 [Source:HGNC Symbol;Acc:HGNC:4484]
ACAA2	acetyl-CoA acyltransferase 2 [Source:HGNC Symbol;Acc:HGNC:83]
HADHB	hydroxyacyl-CoA dehydrogenase/3-ketoacyl-CoA thiolase/enoyl-CoA hydratase (trifunctional protein), beta subunit [Source:HGNC Symbol;Acc:HGNC:4803]
ECHS1	enoyl-CoA hydratase, short chain, 1, mitochondrial [Source:HGNC Symbol;Acc:HGNC:3151]
MECR	mitochondrial trans-2-enoyl-CoA reductase [Source:HGNC Symbol;Acc:HGNC:19691]
ACAA1	acetyl-CoA acyltransferase 1 [Source:HGNC Symbol;Acc:HGNC:82]
EHHADH	enoyl-CoA, hydratase/3-hydroxyacyl CoA dehydrogenase [Source:HGNC Symbol;Acc:HGNC:3247]
HADHA	hydroxyacyl-CoA dehydrogenase/3-ketoacyl-CoA thiolase/enoyl-CoA hydratase (trifunctional protein), alpha subunit [Source:HGNC Symbol;Acc:HGNC:4801]
SCP2	sterol carrier protein 2 [Source:HGNC Symbol;Acc:HGNC:10606]
CPT2	carnitine palmitoyltransferase 2 [Source:HGNC Symbol;Acc:HGNC:2330]
ACADVL	acyl-CoA dehydrogenase, very long chain [Source:HGNC Symbol;Acc:HGNC:92]
ELOVL6	ELOVL fatty acid elongase 6 [Source:HGNC Symbol;Acc:HGNC:15829]
ELOVL2	ELOVL fatty acid elongase 2 [Source:HGNC Symbol;Acc:HGNC:14416]
FADS1	fatty acid desaturase 1 [Source:HGNC Symbol;Acc:HGNC:3574]
FADS2	fatty acid desaturase 2 [Source:HGNC Symbol;Acc:HGNC:3575]
ELOVL5	ELOVL fatty acid elongase 5 [Source:HGNC Symbol;Acc:HGNC:21308]
ELOVL1	ELOVL fatty acid elongase 1 [Source:HGNC Symbol;Acc:HGNC:14418]
ELOVL3	ELOVL fatty acid elongase 3 [Source:HGNC Symbol;Acc:HGNC:18047]
ELOVL4	ELOVL fatty acid elongase 4 [Source:HGNC Symbol;Acc:HGNC:14415]
ELOVL7	ELOVL fatty acid elongase 7 [Source:HGNC Symbol;Acc:HGNC:26292]
TECR	trans-2,3-enoyl-CoA reductase [Source:HGNC Symbol;Acc:HGNC:4551]
CBR4	carbonyl reductase 4 [Source:HGNC Symbol;Acc:HGNC:25891]
HSD17B4	hydroxysteroid 17-beta dehydrogenase 4 [Source:HGNC Symbol;Acc:HGNC:5213]
ACSF3	acyl-CoA synthetase family member 3 [Source:HGNC Symbol;Acc:HGNC:27288]
ACSL5	acyl-CoA synthetase long-chain family member 5 [Source:HGNC Symbol;Acc:HGNC:16526]
FADS3	fatty acid desaturase 3 [Source:HGNC Symbol;Acc:HGNC:3576]

SCD5	stearoyl-CoA desaturase 5 [Source:HGNC Symbol;Acc:HGNC:21088]
FADS6	fatty acid desaturase 6 [Source:HGNC Symbol;Acc:HGNC:30459]
DEGS1	delta(4)-desaturase, sphingolipid 1 [Source:HGNC Symbol;Acc:HGNC:13709]
DEGS2	delta(4)-desaturase, sphingolipid 2 [Source:HGNC Symbol;Acc:HGNC:20113]
HACD1	3-hydroxyacyl-CoA dehydratase 1 [Source:HGNC Symbol;Acc:HGNC:9639]
HACD2	3-hydroxyacyl-CoA dehydratase 2 [Source:HGNC Symbol;Acc:HGNC:9640]
HACD3	3-hydroxyacyl-CoA dehydratase 3 [Source:HGNC Symbol;Acc:HGNC:24175]
HACD4	3-hydroxyacyl-CoA dehydratase 4 [Source:HGNC Symbol;Acc:HGNC:20920]
PAAF1	proteasomal ATPase associated factor 1 [Source:HGNC Symbol;Acc:HGNC:25687]
TMED10	transmembrane p24 trafficking protein 10 [Source:HGNC Symbol;Acc:HGNC:16998]
SEC22B	SEC22 homolog B, vesicle trafficking protein (gene/pseudogene) [Source:HGNC Symbol;Acc:HGNC:10700]
STX3	syntaxin 3 [Source:HGNC Symbol;Acc:HGNC:11438]
NAPA	NSF attachment protein alpha [Source:HGNC Symbol;Acc:HGNC:7641]
TMED9	transmembrane p24 trafficking protein 9 [Source:HGNC Symbol;Acc:HGNC:24878]
STX4	syntaxin 4 [Source:HGNC Symbol;Acc:HGNC:11439]
GOLGA2	golgin A2 [Source:HGNC Symbol;Acc:HGNC:4425]
VAMP3	vesicle associated membrane protein 3 [Source:HGNC Symbol;Acc:HGNC:12644]
YKT6	YKT6 v-SNARE homolog (S. cerevisiae) [Source:HGNC Symbol;Acc:HGNC:16959]
PSMD4	proteasome 26S subunit, non-ATPase 4 [Source:HGNC Symbol;Acc:HGNC:9561]
HSP90AA1	heat shock protein 90kDa alpha family class A member 1 [Source:HGNC Symbol;Acc:HGNC:5253]
SEC22A	SEC22 homolog A, vesicle trafficking protein [Source:HGNC Symbol;Acc:HGNC:20260]
SNAP23	synaptosome associated protein 23 [Source:HGNC Symbol;Acc:HGNC:11131]
ACBD3	acyl-CoA binding domain containing 3 [Source:HGNC Symbol;Acc:HGNC:15453]
ACBD5	acyl-CoA binding domain containing 5 [Source:HGNC Symbol;Acc:HGNC:23338]
PHB2	prohibitin 2 [Source:HGNC Symbol;Acc:HGNC:30306]
SNX18	sorting nexin 18 [Source:HGNC Symbol;Acc:HGNC:19245]
TGM2	transglutaminase 2 [Source:HGNC Symbol;Acc:HGNC:11778]
TRIM16	tripartite motif containing 16 [Source:HGNC Symbol;Acc:HGNC:17241]
VCP	valosin containing protein [Source:HGNC Symbol;Acc:HGNC:12666]
EI24	EI24, autophagy associated transmembrane protein [Source:HGNC Symbol;Acc:HGNC:13276]
EPG5	ectopic P-granules autophagy protein 5 homolog (C. elegans) [Source:HGNC Symbol;Acc:HGNC:29331]
LGALS3	galectin 3 [Source:HGNC Symbol;Acc:HGNC:6563]
SNX4	sorting nexin 4 [Source:HGNC Symbol;Acc:HGNC:11175]
TOLLIP	toll interacting protein [Source:HGNC Symbol;Acc:HGNC:16476]
RAB11A	RAB11A, member RAS oncogene family [Source:HGNC Symbol;Acc:HGNC:9760]
SNX3	sorting nexin 3 [Source:HGNC Symbol;Acc:HGNC:11174]
WDFY3	WD repeat and FYVE domain containing 3 [Source:HGNC Symbol;Acc:HGNC:20751]
TRIM10	tripartite motif containing 10 [Source:HGNC Symbol;Acc:HGNC:10072]
IL1A	interleukin 1 alpha [Source:HGNC Symbol;Acc:HGNC:5991]

ANXA1	annexin A1 [Source:HGNC Symbol;Acc:HGNC:533]
HSP90AB1	heat shock protein 90kDa alpha family class B member 1 [Source:HGNC Symbol;Acc:HGNC:5258]
GORASP2	golgi reassembly stacking protein 2 [Source:HGNC Symbol;Acc:HGNC:17500]
MGMT	O-6-methylguanine-DNA methyltransferase [Source:HGNC Symbol;Acc:HGNC:7059]
RAB3A	RAB3A, member RAS oncogene family [Source:HGNC Symbol;Acc:HGNC:9777]
CAMK2A	calcium/calmodulin dependent protein kinase II alpha [Source:HGNC Symbol;Acc:HGNC:1460]
GNAO1	G protein subunit alpha o1 [Source:HGNC Symbol;Acc:HGNC:4389]
PACSIN1	protein kinase C and casein kinase substrate in neurons 1 [Source:HGNC Symbol;Acc:HGNC:8570]
STX1B	syntaxin 1B [Source:HGNC Symbol;Acc:HGNC:18539]
SYN1	synapsin I [Source:HGNC Symbol;Acc:HGNC:11494]
NQO1	NAD(P)H quinone dehydrogenase 1 [Source:HGNC Symbol;Acc:HGNC:2874]
SYN2	synapsin II [Source:HGNC Symbol;Acc:HGNC:11495]
ADM	adrenomedullin [Source:HGNC Symbol;Acc:HGNC:259]
LTF	lactotransferrin [Source:HGNC Symbol;Acc:HGNC:6720]
AK5	adenylate kinase 5 [Source:HGNC Symbol;Acc:HGNC:365]
NEFM	neurofilament, medium polypeptide [Source:HGNC Symbol;Acc:HGNC:7734]
GABRA1	gamma-aminobutyric acid type A receptor alpha1 subunit [Source:HGNC Symbol;Acc:HGNC:4075]
CDR1	cerebellar degeneration related protein 1 [Source:HGNC Symbol;Acc:HGNC:1798]
CXCL14	C-X-C motif chemokine ligand 14 [Source:HGNC Symbol;Acc:HGNC:10640]
PCP4	Purkinje cell protein 4 [Source:HGNC Symbol;Acc:HGNC:8742]
SLN	sarcolipin [Source:HGNC Symbol;Acc:HGNC:11089]
POSTN	periostin [Source:HGNC Symbol;Acc:HGNC:16953]
SAA2	serum amyloid A2 [Source:HGNC Symbol;Acc:HGNC:10514]
STC1	stanniocalcin 1 [Source:HGNC Symbol;Acc:HGNC:11373]
SOD2	superoxide dismutase 2, mitochondrial [Source:HGNC Symbol;Acc:HGNC:11180]
STMN1	stathmin 1 [Source:HGNC Symbol;Acc:HGNC:6510]
MAPT	microtubule associated protein tau [Source:HGNC Symbol;Acc:HGNC:6893]
SRSF3	serine and arginine rich splicing factor 3 [Source:HGNC Symbol;Acc:HGNC:10785]
SMARCC2	SWI/SNF related, matrix associated, actin dependent regulator of chromatin subfamily c member 2 [Source:HGNC Symbol;Acc:HGNC:11105]
SAA1	serum amyloid A1 [Source:HGNC Symbol;Acc:HGNC:10513]
GBP2	guanylate binding protein 2 [Source:HGNC Symbol;Acc:HGNC:4183]
CHI3L2	chitinase 3 like 2 [Source:HGNC Symbol;Acc:HGNC:1933]
ENPP5	ectonucleotide pyrophosphatase/phosphodiesterase 5 (putative) [Source:HGNC Symbol;Acc:HGNC:13717]
SLC1A2	solute carrier family 1 member 2 [Source:HGNC Symbol;Acc:HGNC:10940]
CHRNA1	cholinergic receptor nicotinic alpha 1 subunit [Source:HGNC Symbol;Acc:HGNC:1955]
SLC4A4	solute carrier family 4 member 4 [Source:HGNC Symbol;Acc:HGNC:11030]
OLFM1	olfactomedin 1 [Source:HGNC Symbol;Acc:HGNC:17187]
LUM	lumican [Source:HGNC Symbol;Acc:HGNC:6724]
CSMD3	CUB and Sushi multiple domains 3 [Source:HGNC Symbol;Acc:HGNC:19291]

GABRB2	gamma-aminobutyric acid type A receptor beta2 subunit [Source:HGNC Symbol;Acc:HGNC:4082]
SLC17A7	solute carrier family 17 member 7 [Source:HGNC Symbol;Acc:HGNC:16704]
FAM180A	family with sequence similarity 180 member A [Source:HGNC Symbol;Acc:HGNC:33773]
LMO3	LIM domain only 3 [Source:HGNC Symbol;Acc:HGNC:6643]
VSNL1	visinin like 1 [Source:HGNC Symbol;Acc:HGNC:12722]
BRINP1	BMP/retinoic acid inducible neural specific 1 [Source:HGNC Symbol;Acc:HGNC:2687]
SYT1	synaptotagmin 1 [Source:HGNC Symbol;Acc:HGNC:11509]
CRYM	crystallin mu [Source:HGNC Symbol;Acc:HGNC:2418]
MBP	myelin basic protein [Source:HGNC Symbol;Acc:HGNC:6925]
ATP1A3	ATPase Na+/K+ transporting subunit alpha 3 [Source:HGNC Symbol;Acc:HGNC:801]
BASP1	brain abundant membrane attached signal protein 1 [Source:HGNC Symbol;Acc:HGNC:957]
GOT1	glutamic-oxaloacetic transaminase 1 [Source:HGNC Symbol;Acc:HGNC:4432]
HIST1H2BJ	histone cluster 1, H2bj [Source:HGNC Symbol;Acc:HGNC:4761]
MAOB	monoamine oxidase B [Source:HGNC Symbol;Acc:HGNC:6834]
STXBP1	syntaxin binding protein 1 [Source:HGNC Symbol;Acc:HGNC:11444]
TERT	telomerase reverse transcriptase [Source:HGNC Symbol;Acc:HGNC:11730]
CPLX1	complexin 1 [Source:HGNC Symbol;Acc:HGNC:2309]
STX1A	syntaxin 1A [Source:HGNC Symbol;Acc:HGNC:11433]
SNAP25	synaptosome associated protein 25 [Source:HGNC Symbol;Acc:HGNC:11132]
SLC18A2	solute carrier family 18 member A2 [Source:HGNC Symbol;Acc:HGNC:10935]
RIMS1	regulating synaptic membrane exocytosis 1 [Source:HGNC Symbol;Acc:HGNC:17282]
UNC13B	unc-13 homolog B (C. elegans) [Source:HGNC Symbol;Acc:HGNC:12566]
TSPOAP1	TSPO associated protein 1 [Source:HGNC Symbol;Acc:HGNC:16831]
SYN3	synapsin III [Source:HGNC Symbol;Acc:HGNC:11496]
VAMP2	vesicle associated membrane protein 2 [Source:HGNC Symbol;Acc:HGNC:12643]
ADAP1	ArfGAP with dual PH domains 1 [Source:HGNC Symbol;Acc:HGNC:16486]
TAB2	TGF-beta activated kinase 1/MAP3K7 binding protein 2 [Source:HGNC Symbol;Acc:HGNC:17075]
NCOR1	nuclear receptor corepressor 1 [Source:HGNC Symbol;Acc:HGNC:7672]
BCL3	B-cell CLL/lymphoma 3 [Source:HGNC Symbol;Acc:HGNC:998]
TGFBI	transforming growth factor beta induced [Source:HGNC Symbol;Acc:HGNC:11771]
LOX	lysyl oxidase [Source:HGNC Symbol;Acc:HGNC:6664]
VDR	vitamin D (1,25- dihydroxyvitamin D3) receptor [Source:HGNC Symbol;Acc:HGNC:12679]
MMP7	matrix metallopeptidase 7 [Source:HGNC Symbol;Acc:HGNC:7174]
HOXD3	homeobox D3 [Source:HGNC Symbol;Acc:HGNC:5137]
SOX10	SRY-box 10 [Source:HGNC Symbol;Acc:HGNC:11190]
CDKN1C	cyclin dependent kinase inhibitor 1C [Source:HGNC Symbol;Acc:HGNC:1786]
HDAC2	histone deacetylase 2 [Source:HGNC Symbol;Acc:HGNC:4853]
EPHB1	EPH receptor B1 [Source:HGNC Symbol;Acc:HGNC:3392]
PTPRA	protein tyrosine phosphatase, receptor type A [Source:HGNC Symbol;Acc:HGNC:9664]
SOX9	SRY-box 9 [Source:HGNC Symbol;Acc:HGNC:11204]
PAX6	paired box 6 [Source:HGNC Symbol;Acc:HGNC:8620]

MEOX2	mesenchyme homeobox 2 [Source:HGNC Symbol;Acc:HGNC:7014]
SPHK1	sphingosine kinase 1 [Source:HGNC Symbol;Acc:HGNC:11240]
ATM	ATM serine/threonine kinase [Source:HGNC Symbol;Acc:HGNC:795]
EEF2	eukaryotic translation elongation factor 2 [Source:HGNC Symbol;Acc:HGNC:3214]
CEND1	cell cycle exit and neuronal differentiation 1 [Source:HGNC Symbol;Acc:HGNC:24153]
CRYAB	crystallin alpha B [Source:HGNC Symbol;Acc:HGNC:2389]
HSP90B1	heat shock protein 90kDa beta family member 1 [Source:HGNC Symbol;Acc:HGNC:12028]
LGALS8	galectin 8 [Source:HGNC Symbol;Acc:HGNC:6569]
GRASP	GRP1 (general receptor for phosphoinositides 1)-associated scaffold protein [Source:HGNC Symbol;Acc:HGNC:18707]
VTI1A	vesicle transport through interaction with t-SNAREs 1A [Source:HGNC Symbol;Acc:HGNC:17792]
VTI1B	vesicle transport through interaction with t-SNAREs 1B [Source:HGNC Symbol;Acc:HGNC:17793]
MRC1	mannose receptor, C type 1 [Source:HGNC Symbol;Acc:HGNC:7228]
SCFD2	sec1 family domain containing 2 [Source:HGNC Symbol;Acc:HGNC:30676]
VAMP7	vesicle associated membrane protein 7 [Source:HGNC Symbol;Acc:HGNC:11486]
VAMP1	vesicle associated membrane protein 1 [Source:HGNC Symbol;Acc:HGNC:12642]
VAMP4	vesicle associated membrane protein 4 [Source:HGNC Symbol;Acc:HGNC:12645]
VAMP5	vesicle associated membrane protein 5 [Source:HGNC Symbol;Acc:HGNC:12646]
BAAT	bile acid-CoA:amino acid N-acyltransferase [Source:HGNC Symbol;Acc:HGNC:932]
CYP8B1	cytochrome P450 family 8 subfamily B member 1 [Source:HGNC Symbol;Acc:HGNC:2653]
CYP39A1	cytochrome P450 family 39 subfamily A member 1 [Source:HGNC Symbol;Acc:HGNC:17449]
CYP46A1	cytochrome P450 family 46 subfamily A member 1 [Source:HGNC Symbol;Acc:HGNC:2641]
ACOX2	acyl-CoA oxidase 2 [Source:HGNC Symbol;Acc:HGNC:120]
CH25H	cholesterol 25-hydroxylase [Source:HGNC Symbol;Acc:HGNC:1907]
HSD3B7	hydroxy-delta-5-steroid dehydrogenase, 3 beta- and steroid delta-isomerase 7 [Source:HGNC Symbol;Acc:HGNC:18324]
ACOT8	acyl-CoA thioesterase 8 [Source:HGNC Symbol;Acc:HGNC:15919]
AMACR	alpha-methylacyl-CoA racemase [Source:HGNC Symbol;Acc:HGNC:451]
CYP7B1	cytochrome P450 family 7 subfamily B member 1 [Source:HGNC Symbol;Acc:HGNC:2652]
ACOT4	acyl-CoA thioesterase 4 [Source:HGNC Symbol;Acc:HGNC:19748]
NR3C2	nuclear receptor subfamily 3 group C member 2 [Source:HGNC Symbol;Acc:HGNC:7979]
DGAT1	diacylglycerol O-acyltransferase 1 [Source:HGNC Symbol;Acc:HGNC:2843]
DGAT2	diacylglycerol O-acyltransferase 2 [Source:HGNC Symbol;Acc:HGNC:16940]
CEL	carboxyl ester lipase [Source:HGNC Symbol;Acc:HGNC:1848]
PNLIPRP1	pancreatic lipase related protein 1 [Source:HGNC Symbol;Acc:HGNC:9156]
LIPF	lipase F, gastric type [Source:HGNC Symbol;Acc:HGNC:6622]
GOT2	glutamic-oxaloacetic transaminase 2 [Source:HGNC Symbol;Acc:HGNC:4433]
MGAT3	mannosyl (beta-1,4-)-glycoprotein beta-1,4-N-acetylglucosaminyltransferase [Source:HGNC Symbol;Acc:HGNC:7046]
MGAT2	mannosyl (alpha-1,6-)-glycoprotein beta-1,2-N-acetylglucosaminyltransferase [Source:HGNC Symbol;Acc:HGNC:7045]
CLPS	colipase [Source:HGNC Symbol;Acc:HGNC:2085]
NPC1L1	NPC1 like intracellular cholesterol transporter 1 [Source:HGNC Symbol;Acc:HGNC:7898]

MTTP	microsomal triglyceride transfer protein [Source:HGNC Symbol;Acc:HGNC:7467]
AGPAT1	1-acylglycerol-3-phosphate O-acyltransferase 1 [Source:HGNC Symbol;Acc:HGNC:324]
AGPAT2	1-acylglycerol-3-phosphate O-acyltransferase 2 [Source:HGNC Symbol;Acc:HGNC:325]
MOGAT2	monoacylglycerol O-acyltransferase 2 [Source:HGNC Symbol;Acc:HGNC:23248]
MOGAT3	monoacylglycerol O-acyltransferase 3 [Source:HGNC Symbol;Acc:HGNC:23249]
PLPP1	phospholipid phosphatase 1 [Source:HGNC Symbol;Acc:HGNC:9228]
PLPP2	phospholipid phosphatase 2 [Source:HGNC Symbol;Acc:HGNC:9230]
PNLIP	pancreatic lipase [Source:HGNC Symbol;Acc:HGNC:9155]
PLA2G2A	phospholipase A2 group IIA [Source:HGNC Symbol;Acc:HGNC:9031]
PLA2G2C	phospholipase A2 group IIC [Source:HGNC Symbol;Acc:HGNC:9032]
PLA2G2D	phospholipase A2 group IID [Source:HGNC Symbol;Acc:HGNC:9033]
PLA2G2E	phospholipase A2 group IIE [Source:HGNC Symbol;Acc:HGNC:13414]
PLA2G2F	phospholipase A2 group IIF [Source:HGNC Symbol;Acc:HGNC:30040]
PNLIPRP3	pancreatic lipase related protein 3 [Source:HGNC Symbol;Acc:HGNC:23492]
ATP1B1	ATPase Na+/K+ transporting subunit beta 1 [Source:HGNC Symbol;Acc:HGNC:804]
AGTR1	angiotensin II receptor type 1 [Source:HGNC Symbol;Acc:HGNC:336]
MC2R	melanocortin 2 receptor [Source:HGNC Symbol;Acc:HGNC:6930]
OCA2	OCA2 melanosomal transmembrane protein [Source:HGNC Symbol;Acc:HGNC:8101]
ATF2	activating transcription factor 2 [Source:HGNC Symbol;Acc:HGNC:784]
NR4A1	nuclear receptor subfamily 4 group A member 1 [Source:HGNC Symbol;Acc:HGNC:7980]
CAMK2B	calcium/calmodulin dependent protein kinase II beta [Source:HGNC Symbol;Acc:HGNC:1461]
KCNK3	potassium two pore domain channel subfamily K member 3 [Source:HGNC Symbol;Acc:HGNC:6278]
KCNK9	potassium two pore domain channel subfamily K member 9 [Source:HGNC Symbol;Acc:HGNC:6283]
KCNJ5	potassium voltage-gated channel subfamily J member 5 [Source:HGNC Symbol;Acc:HGNC:6266]
POMC	proopiomelanocortin [Source:HGNC Symbol;Acc:HGNC:9201]
ATP2A2	ATPase sarcoplasmic/endoplasmic reticulum Ca2+ transporting 2 [Source:HGNC Symbol;Acc:HGNC:812]
CAMK4	calcium/calmodulin dependent protein kinase IV [Source:HGNC Symbol;Acc:HGNC:1464]
PRKD1	protein kinase D1 [Source:HGNC Symbol;Acc:HGNC:9407]
LIPE	lipase E, hormone sensitive type [Source:HGNC Symbol;Acc:HGNC:6621]
NR4A2	nuclear receptor subfamily 4 group A member 2 [Source:HGNC Symbol;Acc:HGNC:7981]
CAMK1	calcium/calmodulin dependent protein kinase I [Source:HGNC Symbol;Acc:HGNC:1459]
CREB5	cAMP responsive element binding protein 5 [Source:HGNC Symbol;Acc:HGNC:16844]
CREB3	cAMP responsive element binding protein 3 [Source:HGNC Symbol;Acc:HGNC:2347]
ATF6B	activating transcription factor 6 beta [Source:HGNC Symbol;Acc:HGNC:2349]
ATF1	activating transcription factor 1 [Source:HGNC Symbol;Acc:HGNC:783]
AGT	angiotensinogen [Source:HGNC Symbol;Acc:HGNC:333]
NPR1	natriuretic peptide receptor 1 [Source:HGNC Symbol;Acc:HGNC:7943]
NPPA	natriuretic peptide A [Source:HGNC Symbol;Acc:HGNC:7939]
ORAI1	ORAI calcium release-activated calcium modulator 1 [Source:HGNC Symbol;Acc:HGNC:25896]

PDE2A	phosphodiesterase 2A [Source:HGNC Symbol;Acc:HGNC:8777]
ATP1A2	ATPase Na+/K+ transporting subunit alpha 2 [Source:HGNC Symbol;Acc:HGNC:800]
ATP1A1	ATPase Na+/K+ transporting subunit alpha 1 [Source:HGNC Symbol;Acc:HGNC:799]
ATP1A4	ATPase Na+/K+ transporting subunit alpha 4 [Source:HGNC Symbol;Acc:HGNC:14073]
CACNA1A	calcium voltage-gated channel subunit alpha1 A [Source:HGNC Symbol;Acc:HGNC:1388]
CACNA1B	calcium voltage-gated channel subunit alpha1 B [Source:HGNC Symbol;Acc:HGNC:1389]
CACNA1C	calcium voltage-gated channel subunit alpha1 C [Source:HGNC Symbol;Acc:HGNC:1390]
CACNA1E	calcium voltage-gated channel subunit alpha1 E [Source:HGNC Symbol;Acc:HGNC:1392]
CACNA1F	calcium voltage-gated channel subunit alpha1 F [Source:HGNC Symbol;Acc:HGNC:1393]
CACNA1G	calcium voltage-gated channel subunit alpha1 G [Source:HGNC Symbol;Acc:HGNC:1394]
CACNA1H	calcium voltage-gated channel subunit alpha1 H [Source:HGNC Symbol;Acc:HGNC:1395]
CACNA1I	calcium voltage-gated channel subunit alpha1 I [Source:HGNC Symbol;Acc:HGNC:1396]
CACNA1S	calcium voltage-gated channel subunit alpha1 S [Source:HGNC Symbol;Acc:HGNC:1397]
CALM1	calmodulin 1 (phosphorylase kinase, delta) [Source:HGNC Symbol;Acc:HGNC:1442]
CALM2	calmodulin 2 (phosphorylase kinase, delta) [Source:HGNC Symbol;Acc:HGNC:1445]
CALM3	calmodulin 3 (phosphorylase kinase, delta) [Source:HGNC Symbol;Acc:HGNC:1449]
DAGLA	diacylglycerol lipase alpha [Source:HGNC Symbol;Acc:HGNC:1165]
DAGLB	diacylglycerol lipase beta [Source:HGNC Symbol;Acc:HGNC:28923]
LRAT	lecithin retinol acyltransferase (phosphatidylcholine--retinol O-acyltransferase) [Source:HGNC Symbol;Acc:HGNC:6685]
BTD	biotinidase [Source:HGNC Symbol;Acc:HGNC:1122]
ABCC1	ATP binding cassette subfamily C member 1 [Source:HGNC Symbol;Acc:HGNC:51]
AWAT2	acyl-CoA wax alcohol acyltransferase 2 [Source:HGNC Symbol;Acc:HGNC:23251]
SLC5A6	solute carrier family 5 member 6 [Source:HGNC Symbol;Acc:HGNC:11041]
FOLH1	folate hydrolase (prostate-specific membrane antigen) 1 [Source:HGNC Symbol;Acc:HGNC:3788]
SLC19A1	solute carrier family 19 member 1 [Source:HGNC Symbol;Acc:HGNC:10937]
SLC19A2	solute carrier family 19 member 2 [Source:HGNC Symbol;Acc:HGNC:10938]
SLC19A3	solute carrier family 19 member 3 [Source:HGNC Symbol;Acc:HGNC:16266]
SLC23A1	solute carrier family 23 member 1 [Source:HGNC Symbol;Acc:HGNC:10974]
SLC46A1	solute carrier family 46 member 1 [Source:HGNC Symbol;Acc:HGNC:30521]
GIF	gastric intrinsic factor [Source:HGNC Symbol;Acc:HGNC:4268]
CUBN	cubilin [Source:HGNC Symbol;Acc:HGNC:2548]
LMBRD1	LMBR1 domain containing 1 [Source:HGNC Symbol;Acc:HGNC:23038]
MMACHC	methylmalonic aciduria (cobalamin deficiency) cblC type, with homocystinuria [Source:HGNC Symbol;Acc:HGNC:24525]
TCN2	transcobalamin 2 [Source:HGNC Symbol;Acc:HGNC:11653]
SLC52A3	solute carrier family 52 member 3 [Source:HGNC Symbol;Acc:HGNC:16187]
PLB1	phospholipase B1 [Source:HGNC Symbol;Acc:HGNC:30041]
RBP2	retinol binding protein 2 [Source:HGNC Symbol;Acc:HGNC:9920]
DHCR7	7-dehydrocholesterol reductase [Source:HGNC Symbol;Acc:HGNC:2860]
TM7SF2	transmembrane 7 superfamily member 2 [Source:HGNC Symbol;Acc:HGNC:11863]
SC5D	sterol-C5-desaturase [Source:HGNC Symbol;Acc:HGNC:10547]

FDFT1	farnesyl-diphosphate farnesyltransferase 1 [Source:HGNC Symbol;Acc:HGNC:3629]
EBP	emopamil binding protein (sterol isomerase) [Source:HGNC Symbol;Acc:HGNC:3133]
LSS	lanosterol synthase (2,3-oxidosqualene-lanosterol cyclase) [Source:HGNC Symbol;Acc:HGNC:6708]
CYP51A1	cytochrome P450 family 51 subfamily A member 1 [Source:HGNC Symbol;Acc:HGNC:2649]
CYP2R1	cytochrome P450 family 2 subfamily R member 1 [Source:HGNC Symbol;Acc:HGNC:20580]
CYP24A1	cytochrome P450 family 24 subfamily A member 1 [Source:HGNC Symbol;Acc:HGNC:2602]
CYP27B1	cytochrome P450 family 27 subfamily B member 1 [Source:HGNC Symbol;Acc:HGNC:2606]
NSDHL	NAD(P) dependent steroid dehydrogenase-like [Source:HGNC Symbol;Acc:HGNC:13398]
DHCR24	24-dehydrocholesterol reductase [Source:HGNC Symbol;Acc:HGNC:2859]
KCNH6	potassium voltage-gated channel subfamily H member 6 [Source:HGNC Symbol;Acc:HGNC:18862]
LBR	lamin B receptor [Source:HGNC Symbol;Acc:HGNC:6518]
SOAT2	sterol O-acyltransferase 2 [Source:HGNC Symbol;Acc:HGNC:11178]
KCNA4	potassium voltage-gated channel subfamily A member 4 [Source:HGNC Symbol;Acc:HGNC:6222]
KCNK2	potassium two pore domain channel subfamily K member 2 [Source:HGNC Symbol;Acc:HGNC:6277]
NR5A1	nuclear receptor subfamily 5 group A member 1 [Source:HGNC Symbol;Acc:HGNC:7983]
NR0B1	nuclear receptor subfamily 0 group B member 1 [Source:HGNC Symbol;Acc:HGNC:7960]
PBX1	PBX homeobox 1 [Source:HGNC Symbol;Acc:HGNC:8632]
PDE8A	phosphodiesterase 8A [Source:HGNC Symbol;Acc:HGNC:8793]
PDE8B	phosphodiesterase 8B [Source:HGNC Symbol;Acc:HGNC:8794]
ALOX5	arachidonate 5-lipoxygenase [Source:HGNC Symbol;Acc:HGNC:435]
FSHR	follicle stimulating hormone receptor [Source:HGNC Symbol;Acc:HGNC:3969]
LHCGR	luteinizing hormone/choriogonadotropin receptor [Source:HGNC Symbol;Acc:HGNC:6585]
FSHB	follicle stimulating hormone beta subunit [Source:HGNC Symbol;Acc:HGNC:3964]
BMP15	bone morphogenetic protein 15 [Source:HGNC Symbol;Acc:HGNC:1068]
LHB	luteinizing hormone beta polypeptide [Source:HGNC Symbol;Acc:HGNC:6584]
CGA	glycoprotein hormones, alpha polypeptide [Source:HGNC Symbol;Acc:HGNC:1885]
KAT5	lysine acetyltransferase 5 [Source:HGNC Symbol;Acc:HGNC:5275]
ACOT1	acyl-CoA thioesterase 1 [Source:HGNC Symbol;Acc:HGNC:33128]
ACOT2	acyl-CoA thioesterase 2 [Source:HGNC Symbol;Acc:HGNC:18431]
PLA2G4A	phospholipase A2 group IVA [Source:HGNC Symbol;Acc:HGNC:9035]
PTGS2	prostaglandin-endoperoxide synthase 2 [Source:HGNC Symbol;Acc:HGNC:9605]
CYP2J2	cytochrome P450 family 2 subfamily J member 2 [Source:HGNC Symbol;Acc:HGNC:2634]
HMGCR	3-hydroxy-3-methylglutaryl-CoA reductase [Source:HGNC Symbol;Acc:HGNC:5006]
EPHX1	epoxide hydrolase 1 [Source:HGNC Symbol;Acc:HGNC:3401]
FXYD2	FXYD domain containing ion transport regulator 2 [Source:HGNC Symbol;Acc:HGNC:4026]
ATP1B3	ATPase Na+/K+ transporting subunit beta 3 [Source:HGNC Symbol;Acc:HGNC:806]
SCTR	secretin receptor [Source:HGNC Symbol;Acc:HGNC:10608]
KCNN2	potassium calcium-activated channel subfamily N member 2 [Source:HGNC Symbol;Acc:HGNC:6291]
SCT	secretin [Source:HGNC Symbol;Acc:HGNC:10607]

ABCB1	ATP binding cassette subfamily B member 1 [Source:HGNC Symbol;Acc:HGNC:40]
ABCB4	ATP binding cassette subfamily B member 4 [Source:HGNC Symbol;Acc:HGNC:45]
ABCC2	ATP binding cassette subfamily C member 2 [Source:HGNC Symbol;Acc:HGNC:53]
ABCC3	ATP binding cassette subfamily C member 3 [Source:HGNC Symbol;Acc:HGNC:54]
ABCC4	ATP binding cassette subfamily C member 4 [Source:HGNC Symbol;Acc:HGNC:55]
ABCG2	ATP binding cassette subfamily G member 2 (Junior blood group) [Source:HGNC Symbol;Acc:HGNC:74]
SLC22A7	solute carrier family 22 member 7 [Source:HGNC Symbol;Acc:HGNC:10971]
SLC22A8	solute carrier family 22 member 8 [Source:HGNC Symbol;Acc:HGNC:10972]
RXRA	retinoid X receptor alpha [Source:HGNC Symbol;Acc:HGNC:10477]
NR1H4	nuclear receptor subfamily 1 group H member 4 [Source:HGNC Symbol;Acc:HGNC:7967]
NR0B2	nuclear receptor subfamily 0 group B member 2 [Source:HGNC Symbol;Acc:HGNC:7961]
AQP1	aquaporin 1 (Colton blood group) [Source:HGNC Symbol;Acc:HGNC:633]
AQP4	aquaporin 4 [Source:HGNC Symbol;Acc:HGNC:637]
AQP8	aquaporin 8 [Source:HGNC Symbol;Acc:HGNC:642]
AQP9	aquaporin 9 [Source:HGNC Symbol;Acc:HGNC:643]
SLC9A3	solute carrier family 9 member A3 [Source:HGNC Symbol;Acc:HGNC:11073]
SLC4A5	solute carrier family 4 member 5 [Source:HGNC Symbol;Acc:HGNC:18168]
SLC5A1	solute carrier family 5 member 1 [Source:HGNC Symbol;Acc:HGNC:11036]
SLC10A1	solute carrier family 10 member 1 [Source:HGNC Symbol;Acc:HGNC:10905]
SLC10A2	solute carrier family 10 member 2 [Source:HGNC Symbol;Acc:HGNC:10906]
SLC51A	solute carrier family 51 alpha subunit [Source:HGNC Symbol;Acc:HGNC:29955]
SLC51B	solute carrier family 51 beta subunit [Source:HGNC Symbol;Acc:HGNC:29956]
SLC22A1	solute carrier family 22 member 1 [Source:HGNC Symbol;Acc:HGNC:10963]
SLC4A2	solute carrier family 4 member 2 [Source:HGNC Symbol;Acc:HGNC:11028]
SLCO1A2	solute carrier organic anion transporter family member 1A2 [Source:HGNC Symbol;Acc:HGNC:10956]
SLCO1B1	solute carrier organic anion transporter family member 1B1 [Source:HGNC Symbol;Acc:HGNC:10959]
SLCO1B3	solute carrier organic anion transporter family member 1B3 [Source:HGNC Symbol;Acc:HGNC:10961]
SLCO1B7	solute carrier organic anion transporter family member 1B7 (putative) [Source:HGNC Symbol;Acc:HGNC:32934]
SLCO1C1	solute carrier organic anion transporter family member 1C1 [Source:HGNC Symbol;Acc:HGNC:13819]
SULT2A1	sulfotransferase family 2A member 1 [Source:HGNC Symbol;Acc:HGNC:11458]
SULT2B1	sulfotransferase family 2B member 1 [Source:HGNC Symbol;Acc:HGNC:11459]
PLCG1	phospholipase C gamma 1 [Source:HGNC Symbol;Acc:HGNC:9065]
CASP1	caspase 1 [Source:HGNC Symbol;Acc:HGNC:1499]
MMP1	matrix metallopeptidase 1 [Source:HGNC Symbol;Acc:HGNC:7155]
MMP3	matrix metallopeptidase 3 [Source:HGNC Symbol;Acc:HGNC:7173]
MMP9	matrix metallopeptidase 9 [Source:HGNC Symbol;Acc:HGNC:7176]
APAF1	apoptotic peptidase activating factor 1 [Source:HGNC Symbol;Acc:HGNC:576]
BAX	BCL2 associated X protein [Source:HGNC Symbol;Acc:HGNC:959]

CASP3	caspase 3 [Source:HGNC Symbol;Acc:HGNC:1504]
TNFRSF1A	tumor necrosis factor receptor superfamily member 1A [Source:HGNC Symbol;Acc:HGNC:11916]
TRAF2	TNF receptor associated factor 2 [Source:HGNC Symbol;Acc:HGNC:12032]
TRAF3	TNF receptor associated factor 3 [Source:HGNC Symbol;Acc:HGNC:12033]
HSPD1	heat shock protein family D (Hsp60) member 1 [Source:HGNC Symbol;Acc:HGNC:5261]
FOS	Fos proto-oncogene, AP-1 transcription factor subunit [Source:HGNC Symbol;Acc:HGNC:3796]
FASLG	Fas ligand [Source:HGNC Symbol;Acc:HGNC:11936]
CD14	CD14 molecule [Source:HGNC Symbol;Acc:HGNC:1628]
CASP6	caspase 6 [Source:HGNC Symbol;Acc:HGNC:1507]
CASP7	caspase 7 [Source:HGNC Symbol;Acc:HGNC:1508]
CASP8	caspase 8 [Source:HGNC Symbol;Acc:HGNC:1509]
CASP9	caspase 9 [Source:HGNC Symbol;Acc:HGNC:1511]
TAB1	TGF-beta activated kinase 1/MAP3K7 binding protein 1 [Source:HGNC Symbol;Acc:HGNC:18157]
MAP2K4	mitogen-activated protein kinase kinase 4 [Source:HGNC Symbol;Acc:HGNC:6844]
MAP2K3	mitogen-activated protein kinase kinase 3 [Source:HGNC Symbol;Acc:HGNC:6843]
MAP2K6	mitogen-activated protein kinase kinase 6 [Source:HGNC Symbol;Acc:HGNC:6846]
NFATC1	nuclear factor of activated T-cells 1 [Source:HGNC Symbol;Acc:HGNC:7775]
JAK2	Janus kinase 2 [Source:HGNC Symbol;Acc:HGNC:6192]
DDIT3	DNA damage inducible transcript 3 [Source:HGNC Symbol;Acc:HGNC:2726]
CHUK	conserved helix-loop-helix ubiquitous kinase [Source:HGNC Symbol;Acc:HGNC:1974]
TNFSF10	tumor necrosis factor superfamily member 10 [Source:HGNC Symbol;Acc:HGNC:11925]
BID	BH3 interacting domain death agonist [Source:HGNC Symbol;Acc:HGNC:1050]
MYD88	myeloid differentiation primary response 88 [Source:HGNC Symbol;Acc:HGNC:7562]
IRAK1	interleukin 1 receptor associated kinase 1 [Source:HGNC Symbol;Acc:HGNC:6112]
IRAK4	interleukin 1 receptor associated kinase 4 [Source:HGNC Symbol;Acc:HGNC:17967]
NFKBIA	NFKB inhibitor alpha [Source:HGNC Symbol;Acc:HGNC:7797]
LBP	lipopolysaccharide binding protein [Source:HGNC Symbol;Acc:HGNC:6517]
LY96	lymphocyte antigen 96 [Source:HGNC Symbol;Acc:HGNC:17156]
TIRAP	toll-interleukin 1 receptor (TIR) domain containing adaptor protein [Source:HGNC Symbol;Acc:HGNC:17192]
IL12A	interleukin 12A [Source:HGNC Symbol;Acc:HGNC:5969]
CCL3	C-C motif chemokine ligand 3 [Source:HGNC Symbol;Acc:HGNC:10627]
IFNA2	interferon, alpha 2 [Source:HGNC Symbol;Acc:HGNC:5423]
IL12B	interleukin 12B [Source:HGNC Symbol;Acc:HGNC:5970]
ABCG1	ATP binding cassette subfamily G member 1 [Source:HGNC Symbol;Acc:HGNC:73]
LYN	LYN proto-oncogene, Src family tyrosine kinase [Source:HGNC Symbol;Acc:HGNC:6735]
PPP3R1	protein phosphatase 3 regulatory subunit B, alpha [Source:HGNC Symbol;Acc:HGNC:9317]
IKBKB	inhibitor of kappa light polypeptide gene enhancer in B-cells, kinase beta [Source:HGNC Symbol;Acc:HGNC:5960]
IKBKG	inhibitor of kappa light polypeptide gene enhancer in B-cells, kinase gamma [Source:HGNC Symbol;Acc:HGNC:5961]

IKBKE	inhibitor of kappa light polypeptide gene enhancer in B-cells, kinase epsilon [Source:HGNC Symbol;Acc:HGNC:14552]
NCF2	neutrophil cytosolic factor 2 [Source:HGNC Symbol;Acc:HGNC:7661]
NCF1	neutrophil cytosolic factor 1 [Source:HGNC Symbol;Acc:HGNC:7660]
NCF4	neutrophil cytosolic factor 4 [Source:HGNC Symbol;Acc:HGNC:7662]
RXRB	retinoid X receptor beta [Source:HGNC Symbol;Acc:HGNC:10478]
RXRG	retinoid X receptor gamma [Source:HGNC Symbol;Acc:HGNC:10479]
OLR1	oxidized low density lipoprotein receptor 1 [Source:HGNC Symbol;Acc:HGNC:8133]
ATF6	activating transcription factor 6 [Source:HGNC Symbol;Acc:HGNC:791]
IRF7	interferon regulatory factor 7 [Source:HGNC Symbol;Acc:HGNC:6122]
TLR2	toll like receptor 2 [Source:HGNC Symbol;Acc:HGNC:11848]
TLR4	toll like receptor 4 [Source:HGNC Symbol;Acc:HGNC:11850]
TLR6	toll like receptor 6 [Source:HGNC Symbol;Acc:HGNC:16711]
MIB1	mindbomb E3 ubiquitin protein ligase 1 [Source:HGNC Symbol;Acc:HGNC:21086]
ERO1A	endoplasmic reticulum oxidoreductase alpha [Source:HGNC Symbol;Acc:HGNC:13280]
PYCARD	PYD and CARD domain containing [Source:HGNC Symbol;Acc:HGNC:16608]
NFATC2	nuclear factor of activated T-cells 2 [Source:HGNC Symbol;Acc:HGNC:7776]
NFATC3	nuclear factor of activated T-cells 3 [Source:HGNC Symbol;Acc:HGNC:7777]
CYP2A6	cytochrome P450 family 2 subfamily A member 6 [Source:HGNC Symbol;Acc:HGNC:2610]
VLDLR	very low density lipoprotein receptor [Source:HGNC Symbol;Acc:HGNC:12698]
AGER	advanced glycosylation end product-specific receptor [Source:HGNC Symbol;Acc:HGNC:320]
CYBA	cytochrome b-245 alpha chain [Source:HGNC Symbol;Acc:HGNC:2577]
TICAM2	toll like receptor adaptor molecule 2 [Source:HGNC Symbol;Acc:HGNC:21354]
TNFRSF10A	tumor necrosis factor receptor superfamily member 10a [Source:HGNC Symbol;Acc:HGNC:11904]
TNFRSF10B	tumor necrosis factor receptor superfamily member 10b [Source:HGNC Symbol;Acc:HGNC:11905]
CXCL2	C-X-C motif chemokine ligand 2 [Source:HGNC Symbol;Acc:HGNC:4603]
CXCL3	C-X-C motif chemokine ligand 3 [Source:HGNC Symbol;Acc:HGNC:4604]
POU2F1	POU class 2 homeobox 1 [Source:HGNC Symbol;Acc:HGNC:9212]
POU2F2	POU class 2 homeobox 2 [Source:HGNC Symbol;Acc:HGNC:9213]
POU2F3	POU class 2 homeobox 3 [Source:HGNC Symbol;Acc:HGNC:19864]
HSPA1A	heat shock protein family A (Hsp70) member 1A [Source:HGNC Symbol;Acc:HGNC:5232]
HSPA1B	heat shock protein family A (Hsp70) member 1B [Source:HGNC Symbol;Acc:HGNC:5233]
HSPA1L	heat shock protein family A (Hsp70) member 1 like [Source:HGNC Symbol;Acc:HGNC:5234]
MAPK14	mitogen-activated protein kinase 14 [Source:HGNC Symbol;Acc:HGNC:6876]
PPP3CA	protein phosphatase 3 catalytic subunit alpha [Source:HGNC Symbol;Acc:HGNC:9314]
PPP3CB	protein phosphatase 3 catalytic subunit beta [Source:HGNC Symbol;Acc:HGNC:9315]
PPP3CC	protein phosphatase 3 catalytic subunit gamma [Source:HGNC Symbol;Acc:HGNC:9316]
PPP3R2	protein phosphatase 3 regulatory subunit B, beta [Source:HGNC Symbol;Acc:HGNC:9318]
MOK	MOK protein kinase [Source:HGNC Symbol;Acc:HGNC:9833]
TRAM1	translocation associated membrane protein 1 [Source:HGNC Symbol;Acc:HGNC:20568]

TRAM2	translocation associated membrane protein 2 [Source:HGNC Symbol;Acc:HGNC:16855]
NOX3	NADPH oxidase 3 [Source:HGNC Symbol;Acc:HGNC:7890]
POU5F1B	POU class 5 homeobox 1B [Source:HGNC Symbol;Acc:HGNC:9223]
POU3F1	POU class 3 homeobox 1 [Source:HGNC Symbol;Acc:HGNC:9214]
POU3F2	POU class 3 homeobox 2 [Source:HGNC Symbol;Acc:HGNC:9215]
POU3F3	POU class 3 homeobox 3 [Source:HGNC Symbol;Acc:HGNC:9216]
POU3F4	POU class 3 homeobox 4 [Source:HGNC Symbol;Acc:HGNC:9217]
TBCE	tubulin folding cofactor E [Source:HGNC Symbol;Acc:HGNC:11582]
THEM4	thioesterase superfamily member 4 [Source:HGNC Symbol;Acc:HGNC:17947]
ACOT7	acyl-CoA thioesterase 7 [Source:HGNC Symbol;Acc:HGNC:24157]
THEM5	thioesterase superfamily member 5 [Source:HGNC Symbol;Acc:HGNC:26755]
CYP2U1	cytochrome P450 family 2 subfamily U member 1 [Source:HGNC Symbol;Acc:HGNC:20582]
ACOX1	acyl-CoA oxidase 1 [Source:HGNC Symbol;Acc:HGNC:119]
ACOX3	acyl-CoA oxidase 3, pristanoyl [Source:HGNC Symbol;Acc:HGNC:121]
ECI1	enoyl-CoA delta isomerase 1 [Source:HGNC Symbol;Acc:HGNC:2703]
ECI2	enoyl-CoA delta isomerase 2 [Source:HGNC Symbol;Acc:HGNC:14601]
GCDH	glutaryl-CoA dehydrogenase [Source:HGNC Symbol;Acc:HGNC:4189]
ACSBG1	acyl-CoA synthetase bubblegum family member 1 [Source:HGNC Symbol;Acc:HGNC:29567]
ACSBG2	acyl-CoA synthetase bubblegum family member 2 [Source:HGNC Symbol;Acc:HGNC:24174]
ACSL1	acyl-CoA synthetase long-chain family member 1 [Source:HGNC Symbol;Acc:HGNC:3569]
ACSL3	acyl-CoA synthetase long-chain family member 3 [Source:HGNC Symbol;Acc:HGNC:3570]
ACSL4	acyl-CoA synthetase long-chain family member 4 [Source:HGNC Symbol;Acc:HGNC:3571]
ACSL6	acyl-CoA synthetase long-chain family member 6 [Source:HGNC Symbol;Acc:HGNC:16496]
ALDH1L1	aldehyde dehydrogenase 1 family member L1 [Source:HGNC Symbol;Acc:HGNC:3978]
ALDH1L2	aldehyde dehydrogenase 1 family member L2 [Source:HGNC Symbol;Acc:HGNC:26777]
DNMT3A	DNA (cytosine-5-)-methyltransferase 3 alpha [Source:HGNC Symbol;Acc:HGNC:2978]
CYP4A11	cytochrome P450 family 4 subfamily A member 11 [Source:HGNC Symbol;Acc:HGNC:2642]
CYP4Z1	cytochrome P450 family 4 subfamily Z member 1 [Source:HGNC Symbol;Acc:HGNC:20583]
CYP4A22	cytochrome P450 family 4 subfamily A member 22 [Source:HGNC Symbol;Acc:HGNC:20575]
PPID	peptidylprolyl isomerase D [Source:HGNC Symbol;Acc:HGNC:9257]
PPIF	peptidylprolyl isomerase F [Source:HGNC Symbol;Acc:HGNC:9259]
PPIE	peptidylprolyl isomerase E [Source:HGNC Symbol;Acc:HGNC:9258]
SH2D2A	SH2 domain containing 2A [Source:HGNC Symbol;Acc:HGNC:10821]
PLCG2	phospholipase C gamma 2 [Source:HGNC Symbol;Acc:HGNC:9066]
SPHK2	sphingosine kinase 2 [Source:HGNC Symbol;Acc:HGNC:18859]
PLA2G4E	phospholipase A2 group IVE [Source:HGNC Symbol;Acc:HGNC:24791]
JMJD7-PLA2G4B	JMJD7-PLA2G4B readthrough [Source:HGNC Symbol;Acc:HGNC:34449]
PLA2G4B	phospholipase A2 group IVB [Source:HGNC Symbol;Acc:HGNC:9036]
PLA2G4C	phospholipase A2 group IVC [Source:HGNC Symbol;Acc:HGNC:9037]
PLA2G4D	phospholipase A2 group IVD [Source:HGNC Symbol;Acc:HGNC:30038]
PLA2G4F	phospholipase A2 group IVF [Source:HGNC Symbol;Acc:HGNC:27396]

MAPK11	mitogen-activated protein kinase 11 [Source:HGNC Symbol;Acc:HGNC:6873]
MAPK12	mitogen-activated protein kinase 12 [Source:HGNC Symbol;Acc:HGNC:6874]
MAPK13	mitogen-activated protein kinase 13 [Source:HGNC Symbol;Acc:HGNC:6875]
MAPKAPK2	mitogen-activated protein kinase-activated protein kinase 2 [Source:HGNC Symbol;Acc:HGNC:6887]
MAPKAPK3	mitogen-activated protein kinase-activated protein kinase 3 [Source:HGNC Symbol;Acc:HGNC:6888]
HSPB1	heat shock protein family B (small) member 1 [Source:HGNC Symbol;Acc:HGNC:5246]
TGFA	transforming growth factor alpha [Source:HGNC Symbol;Acc:HGNC:11765]
EREG	epiregulin [Source:HGNC Symbol;Acc:HGNC:3443]
NGF	nerve growth factor [Source:HGNC Symbol;Acc:HGNC:7808]
BDNF	brain derived neurotrophic factor [Source:HGNC Symbol;Acc:HGNC:1033]
NTF3	neurotrophin 3 [Source:HGNC Symbol;Acc:HGNC:8023]
NTF4	neurotrophin 4 [Source:HGNC Symbol;Acc:HGNC:8024]
IGF2	insulin like growth factor 2 [Source:HGNC Symbol;Acc:HGNC:5466]
CSF1	colony stimulating factor 1 [Source:HGNC Symbol;Acc:HGNC:2432]
KITLG	KIT ligand [Source:HGNC Symbol;Acc:HGNC:6343]
FLT3LG	fms related tyrosine kinase 3 ligand [Source:HGNC Symbol;Acc:HGNC:3766]
EFNA1	ephrin A1 [Source:HGNC Symbol;Acc:HGNC:3221]
EFNA2	ephrin A2 [Source:HGNC Symbol;Acc:HGNC:3222]
EFNA3	ephrin A3 [Source:HGNC Symbol;Acc:HGNC:3223]
EFNA4	ephrin A4 [Source:HGNC Symbol;Acc:HGNC:3224]
EFNA5	ephrin A5 [Source:HGNC Symbol;Acc:HGNC:3225]
ERBB3	erb-b2 receptor tyrosine kinase 3 [Source:HGNC Symbol;Acc:HGNC:3431]
NGFR	nerve growth factor receptor [Source:HGNC Symbol;Acc:HGNC:7809]
NTRK2	neurotrophic receptor tyrosine kinase 2 [Source:HGNC Symbol;Acc:HGNC:8032]
CSF1R	colony stimulating factor 1 receptor [Source:HGNC Symbol;Acc:HGNC:2433]
EPHA2	EPH receptor A2 [Source:HGNC Symbol;Acc:HGNC:3386]
SYK	spleen tyrosine kinase [Source:HGNC Symbol;Acc:HGNC:11491]
CD19	CD19 molecule [Source:HGNC Symbol;Acc:HGNC:1633]
PIK3AP1	phosphoinositide-3-kinase adaptor protein 1 [Source:HGNC Symbol;Acc:HGNC:30034]
GH1	growth hormone 1 [Source:HGNC Symbol;Acc:HGNC:4261]
GH2	growth hormone 2 [Source:HGNC Symbol;Acc:HGNC:4262]
CSH1	chorionic somatomammotropin hormone 1 [Source:HGNC Symbol;Acc:HGNC:2440]
CSH2	chorionic somatomammotropin hormone 2 [Source:HGNC Symbol;Acc:HGNC:2441]
PRL	prolactin [Source:HGNC Symbol;Acc:HGNC:9445]
OSM	oncostatin M [Source:HGNC Symbol;Acc:HGNC:8506]
IL2	interleukin 2 [Source:HGNC Symbol;Acc:HGNC:6001]
IL3	interleukin 3 [Source:HGNC Symbol;Acc:HGNC:6011]
IL4	interleukin 4 [Source:HGNC Symbol;Acc:HGNC:6014]
IL7	interleukin 7 [Source:HGNC Symbol;Acc:HGNC:6023]
IFNA1	interferon, alpha 1 [Source:HGNC Symbol;Acc:HGNC:5417]

IFNA4	interferon, alpha 4 [Source:HGNC Symbol;Acc:HGNC:5425]
IFNA5	interferon alpha 5 [Source:HGNC Symbol;Acc:HGNC:5426]
IFNA6	interferon alpha 6 [Source:HGNC Symbol;Acc:HGNC:5427]
IFNA7	interferon, alpha 7 [Source:HGNC Symbol;Acc:HGNC:5428]
IFNA8	interferon alpha 8 [Source:HGNC Symbol;Acc:HGNC:5429]
IFNA10	interferon, alpha 10 [Source:HGNC Symbol;Acc:HGNC:5418]
IFNA13	interferon, alpha 13 [Source:HGNC Symbol;Acc:HGNC:5419]
IFNA14	interferon alpha 14 [Source:HGNC Symbol;Acc:HGNC:5420]
IFNA16	interferon, alpha 16 [Source:HGNC Symbol;Acc:HGNC:5421]
IFNA17	interferon, alpha 17 [Source:HGNC Symbol;Acc:HGNC:5422]
IFNA21	interferon, alpha 21 [Source:HGNC Symbol;Acc:HGNC:5424]
EPO	erythropoietin [Source:HGNC Symbol;Acc:HGNC:3415]
CSF3	colony stimulating factor 3 [Source:HGNC Symbol;Acc:HGNC:2438]
GHR	growth hormone receptor [Source:HGNC Symbol;Acc:HGNC:4263]
PRLR	prolactin receptor [Source:HGNC Symbol;Acc:HGNC:9446]
OSMR	oncostatin M receptor [Source:HGNC Symbol;Acc:HGNC:8507]
IL2RA	interleukin 2 receptor subunit alpha [Source:HGNC Symbol;Acc:HGNC:6008]
IL2RB	interleukin 2 receptor subunit beta [Source:HGNC Symbol;Acc:HGNC:6009]
IL2RG	interleukin 2 receptor subunit gamma [Source:HGNC Symbol;Acc:HGNC:6010]
IL3RA	interleukin 3 receptor subunit alpha [Source:HGNC Symbol;Acc:HGNC:6012]
IL6R	interleukin 6 receptor [Source:HGNC Symbol;Acc:HGNC:6019]
IL4R	interleukin 4 receptor [Source:HGNC Symbol;Acc:HGNC:6015]
IL7R	interleukin 7 receptor [Source:HGNC Symbol;Acc:HGNC:6024]
IFNAR1	interferon alpha and beta receptor subunit 1 [Source:HGNC Symbol;Acc:HGNC:5432]
IFNAR2	interferon alpha and beta receptor subunit 2 [Source:HGNC Symbol;Acc:HGNC:5433]
EPOR	erythropoietin receptor [Source:HGNC Symbol;Acc:HGNC:3416]
CSF3R	colony stimulating factor 3 receptor [Source:HGNC Symbol;Acc:HGNC:2439]
JAK1	Janus kinase 1 [Source:HGNC Symbol;Acc:HGNC:6190]
JAK3	Janus kinase 3 [Source:HGNC Symbol;Acc:HGNC:6193]
LPAR3	lysophosphatidic acid receptor 3 [Source:HGNC Symbol;Acc:HGNC:14298]
LPAR6	lysophosphatidic acid receptor 6 [Source:HGNC Symbol;Acc:HGNC:15520]
GNB1	G protein subunit beta 1 [Source:HGNC Symbol;Acc:HGNC:4396]
GNB2	G protein subunit beta 2 [Source:HGNC Symbol;Acc:HGNC:4398]
GNB3	G protein subunit beta 3 [Source:HGNC Symbol;Acc:HGNC:4400]
GNB4	G protein subunit beta 4 [Source:HGNC Symbol;Acc:HGNC:20731]
GNB5	G protein subunit beta 5 [Source:HGNC Symbol;Acc:HGNC:4401]
GNG2	G protein subunit gamma 2 [Source:HGNC Symbol;Acc:HGNC:4404]
GNG3	G protein subunit gamma 3 [Source:HGNC Symbol;Acc:HGNC:4405]
GNG4	G protein subunit gamma 4 [Source:HGNC Symbol;Acc:HGNC:4407]
GNG5	G protein subunit gamma 5 [Source:HGNC Symbol;Acc:HGNC:4408]
GNG7	G protein subunit gamma 7 [Source:HGNC Symbol;Acc:HGNC:4410]

GNG8	G protein subunit gamma 8 [Source:HGNC Symbol;Acc:HGNC:19664]
GNG10	G protein subunit gamma 10 [Source:HGNC Symbol;Acc:HGNC:4402]
GNG11	G protein subunit gamma 11 [Source:HGNC Symbol;Acc:HGNC:4403]
GNG13	G protein subunit gamma 13 [Source:HGNC Symbol;Acc:HGNC:14131]
GNGT1	G protein subunit gamma transducin 1 [Source:HGNC Symbol;Acc:HGNC:4411]
GNGT2	G protein subunit gamma transducin 2 [Source:HGNC Symbol;Acc:HGNC:4412]
PIK3CG	phosphatidylinositol-4,5-bisphosphate 3-kinase catalytic subunit gamma [Source:HGNC Symbol;Acc:HGNC:8978]
PIK3R5	phosphoinositide-3-kinase regulatory subunit 5 [Source:HGNC Symbol;Acc:HGNC:30035]
PIK3R6	phosphoinositide-3-kinase regulatory subunit 6 [Source:HGNC Symbol;Acc:HGNC:27101]
EIF4E	eukaryotic translation initiation factor 4E [Source:HGNC Symbol;Acc:HGNC:3287]
EIF4E2	eukaryotic translation initiation factor 4E family member 2 [Source:HGNC Symbol;Acc:HGNC:3293]
EIF4E1B	eukaryotic translation initiation factor 4E family member 1B [Source:HGNC Symbol;Acc:HGNC:33179]
EIF4B	eukaryotic translation initiation factor 4B [Source:HGNC Symbol;Acc:HGNC:3285]
RPS6	ribosomal protein S6 [Source:HGNC Symbol;Acc:HGNC:10429]
PKN1	protein kinase N1 [Source:HGNC Symbol;Acc:HGNC:9405]
PKN2	protein kinase N2 [Source:HGNC Symbol;Acc:HGNC:9406]
PKN3	protein kinase N3 [Source:HGNC Symbol;Acc:HGNC:17999]
SGK1	serum/glucocorticoid regulated kinase 1 [Source:HGNC Symbol;Acc:HGNC:10810]
SGK2	SGK2, serine/threonine kinase 2 [Source:HGNC Symbol;Acc:HGNC:13900]
SGK3	serum/glucocorticoid regulated kinase family member 3 [Source:HGNC Symbol;Acc:HGNC:10812]
C8orf44-SGK3	C8orf44-SGK3 readthrough [Source:HGNC Symbol;Acc:HGNC:48354]
MAGI2	membrane associated guanylate kinase, WW and PDZ domain containing 2 [Source:HGNC Symbol;Acc:HGNC:18957]
PPP2R3B	protein phosphatase 2 regulatory subunit B''beta [Source:HGNC Symbol;Acc:HGNC:13417]
PPP2R3C	protein phosphatase 2 regulatory subunit B''gamma [Source:HGNC Symbol;Acc:HGNC:17485]
PPP2R3A	protein phosphatase 2 regulatory subunit B''alpha [Source:HGNC Symbol;Acc:HGNC:9307]
PPP2R5B	protein phosphatase 2 regulatory subunit B'beta [Source:HGNC Symbol;Acc:HGNC:9310]
PPP2R5C	protein phosphatase 2 regulatory subunit B'gamma [Source:HGNC Symbol;Acc:HGNC:9311]
PPP2R5D	protein phosphatase 2 regulatory subunit B'delta [Source:HGNC Symbol;Acc:HGNC:9312]
PPP2R5E	protein phosphatase 2 regulatory subunit B'epsilon [Source:HGNC Symbol;Acc:HGNC:9313]
PPP2R5A	protein phosphatase 2 regulatory subunit B'alpha [Source:HGNC Symbol;Acc:HGNC:9309]
CDC37	cell division cycle 37 [Source:HGNC Symbol;Acc:HGNC:1735]
CRTC2	CREB regulated transcription coactivator 2 [Source:HGNC Symbol;Acc:HGNC:27301]
PHLPP1	PH domain and leucine rich repeat protein phosphatase 1 [Source:HGNC Symbol;Acc:HGNC:20610]
PHLPP2	PH domain and leucine rich repeat protein phosphatase 2 [Source:HGNC Symbol;Acc:HGNC:29149]
TCL1A	T-cell leukemia/lymphoma 1A [Source:HGNC Symbol;Acc:HGNC:11648]
TCL1B	T-cell leukemia/lymphoma 1B [Source:HGNC Symbol;Acc:HGNC:11649]

MTCP1	mature T-cell proliferation 1 [Source:HGNC Symbol;Acc:HGNC:7423]
CDKN1B	cyclin dependent kinase inhibitor 1B [Source:HGNC Symbol;Acc:HGNC:1785]
CDK2	cyclin dependent kinase 2 [Source:HGNC Symbol;Acc:HGNC:1771]
CDK6	cyclin dependent kinase 6 [Source:HGNC Symbol;Acc:HGNC:1777]
CCNE1	cyclin E1 [Source:HGNC Symbol;Acc:HGNC:1589]
CCNE2	cyclin E2 [Source:HGNC Symbol;Acc:HGNC:1590]
RBL2	RB transcriptional corepressor like 2 [Source:HGNC Symbol;Acc:HGNC:9894]
BCL2L11	BCL2 like 11 [Source:HGNC Symbol;Acc:HGNC:994]
YWHAZ	tyrosine 3-monooxygenase/tryptophan 5-monooxygenase activation protein zeta [Source:HGNC Symbol;Acc:HGNC:12855]
YWHAB	tyrosine 3-monooxygenase/tryptophan 5-monooxygenase activation protein beta [Source:HGNC Symbol;Acc:HGNC:12849]
YWHAE	tyrosine 3-monooxygenase/tryptophan 5-monooxygenase activation protein epsilon [Source:HGNC Symbol;Acc:HGNC:12851]
YWHAH	tyrosine 3-monooxygenase/tryptophan 5-monooxygenase activation protein eta [Source:HGNC Symbol;Acc:HGNC:12853]
YWHAG	tyrosine 3-monooxygenase/tryptophan 5-monooxygenase activation protein gamma [Source:HGNC Symbol;Acc:HGNC:12852]
CREB3L1	cAMP responsive element binding protein 3 like 1 [Source:HGNC Symbol;Acc:HGNC:18856]
CREB3L2	cAMP responsive element binding protein 3 like 2 [Source:HGNC Symbol;Acc:HGNC:23720]
CREB3L3	cAMP responsive element binding protein 3 like 3 [Source:HGNC Symbol;Acc:HGNC:18855]
CREB3L4	cAMP responsive element binding protein 3 like 4 [Source:HGNC Symbol;Acc:HGNC:18854]
MCL1	myeloid cell leukemia 1 [Source:HGNC Symbol;Acc:HGNC:6943]
MYB	MYB proto-oncogene, transcription factor [Source:HGNC Symbol;Acc:HGNC:7545]
GAB1	GRB2 associated binding protein 1 [Source:HGNC Symbol;Acc:HGNC:4066]
GAB2	GRB2 associated binding protein 2 [Source:HGNC Symbol;Acc:HGNC:14458]
PTPN11	protein tyrosine phosphatase, non-receptor type 11 [Source:HGNC Symbol;Acc:HGNC:9644]
RASGRP1	RAS guanyl releasing protein 1 [Source:HGNC Symbol;Acc:HGNC:9878]
RASGRP2	RAS guanyl releasing protein 2 [Source:HGNC Symbol;Acc:HGNC:9879]
RASGRP3	RAS guanyl releasing protein 3 [Source:HGNC Symbol;Acc:HGNC:14545]
RASGRP4	RAS guanyl releasing protein 4 [Source:HGNC Symbol;Acc:HGNC:18958]
ZAP70	zeta chain of T cell receptor associated protein kinase 70kDa [Source:HGNC Symbol;Acc:HGNC:12858]
LAT	linker for activation of T-cells [Source:HGNC Symbol;Acc:HGNC:18874]
HTR7	5-hydroxytryptamine receptor 7 [Source:HGNC Symbol;Acc:HGNC:5302]
RASGRF2	Ras protein specific guanine nucleotide releasing factor 2 [Source:HGNC Symbol;Acc:HGNC:9876]
GRIN1	glutamate ionotropic receptor NMDA type subunit 1 [Source:HGNC Symbol;Acc:HGNC:4584]
GRIN2A	glutamate ionotropic receptor NMDA type subunit 2A [Source:HGNC Symbol;Acc:HGNC:4585]
GRIN2B	glutamate ionotropic receptor NMDA type subunit 2B [Source:HGNC Symbol;Acc:HGNC:4586]
CALML3	calmodulin like 3 [Source:HGNC Symbol;Acc:HGNC:1452]
CALML6	calmodulin like 6 [Source:HGNC Symbol;Acc:HGNC:24193]
CALML5	calmodulin like 5 [Source:HGNC Symbol;Acc:HGNC:18180]
CALML4	calmodulin like 4 [Source:HGNC Symbol;Acc:HGNC:18445]

RASA1	RAS p21 protein activator 1 [Source:HGNC Symbol;Acc:HGNC:9871]
RASA2	RAS p21 protein activator 2 [Source:HGNC Symbol;Acc:HGNC:9872]
RASA3	RAS p21 protein activator 3 [Source:HGNC Symbol;Acc:HGNC:20331]
RASA4	RAS p21 protein activator 4 [Source:HGNC Symbol;Acc:HGNC:23181]
RASA4B	RAS p21 protein activator 4B [Source:HGNC Symbol;Acc:HGNC:35202]
SYNGAP1	synaptic Ras GTPase activating protein 1 [Source:HGNC Symbol;Acc:HGNC:11497]
RASAL1	RAS protein activator like 1 [Source:HGNC Symbol;Acc:HGNC:9873]
RASAL2	RAS protein activator like 2 [Source:HGNC Symbol;Acc:HGNC:9874]
RASAL3	RAS protein activator like 3 [Source:HGNC Symbol;Acc:HGNC:26129]
RASSF1	Ras association domain family member 1 [Source:HGNC Symbol;Acc:HGNC:9882]
RASSF5	Ras association domain family member 5 [Source:HGNC Symbol;Acc:HGNC:17609]
SHOC2	SHOC2, leucine rich repeat scaffold protein [Source:HGNC Symbol;Acc:HGNC:15454]
PLA1A	phospholipase A1 member A [Source:HGNC Symbol;Acc:HGNC:17661]
PLA2G10	phospholipase A2 group X [Source:HGNC Symbol;Acc:HGNC:9029]
PLA2G3	phospholipase A2 group III [Source:HGNC Symbol;Acc:HGNC:17934]
PLA2G12A	phospholipase A2 group XIIA [Source:HGNC Symbol;Acc:HGNC:18554]
PLA2G12B	phospholipase A2 group XIIB [Source:HGNC Symbol;Acc:HGNC:18555]
PLA2G1B	phospholipase A2 group IB [Source:HGNC Symbol;Acc:HGNC:9030]
PLA2G5	phospholipase A2 group V [Source:HGNC Symbol;Acc:HGNC:9038]
PLA2G6	phospholipase A2 group VI [Source:HGNC Symbol;Acc:HGNC:9039]
ETS1	ETS proto-oncogene 1, transcription factor [Source:HGNC Symbol;Acc:HGNC:3488]
ETS2	ETS proto-oncogene 2, transcription factor [Source:HGNC Symbol;Acc:HGNC:3489]
BRAP	BRCA1 associated protein [Source:HGNC Symbol;Acc:HGNC:1099]
KSR1	kinase suppressor of ras 1 [Source:HGNC Symbol;Acc:HGNC:6465]
KSR2	kinase suppressor of ras 2 [Source:HGNC Symbol;Acc:HGNC:18610]
RAPGEF5	Rap guanine nucleotide exchange factor 5 [Source:HGNC Symbol;Acc:HGNC:16862]
RALGDS	ral guanine nucleotide dissociation stimulator [Source:HGNC Symbol;Acc:HGNC:9842]
RGL1	ral guanine nucleotide dissociation stimulator like 1 [Source:HGNC Symbol;Acc:HGNC:30281]
RGL2	ral guanine nucleotide dissociation stimulator like 2 [Source:HGNC Symbol;Acc:HGNC:9769]
RALA	RALA Ras like proto-oncogene A [Source:HGNC Symbol;Acc:HGNC:9839]
RALB	RALB Ras like proto-oncogene B [Source:HGNC Symbol;Acc:HGNC:9840]
EXOC2	exocyst complex component 2 [Source:HGNC Symbol;Acc:HGNC:24968]
REL	REL proto-oncogene, NF-kB subunit [Source:HGNC Symbol;Acc:HGNC:9954]
PLD1	phospholipase D1 [Source:HGNC Symbol;Acc:HGNC:9067]
PLD2	phospholipase D2 [Source:HGNC Symbol;Acc:HGNC:9068]
RALBP1	ralA binding protein 1 [Source:HGNC Symbol;Acc:HGNC:9841]
PLCE1	phospholipase C epsilon 1 [Source:HGNC Symbol;Acc:HGNC:17175]
RIN1	Ras and Rab interactor 1 [Source:HGNC Symbol;Acc:HGNC:18749]
ABL2	ABL proto-oncogene 2, non-receptor tyrosine kinase [Source:HGNC Symbol;Acc:HGNC:77]
RAB5A	RAB5A, member RAS oncogene family [Source:HGNC Symbol;Acc:HGNC:9783]
RAB5B	RAB5B, member RAS oncogene family [Source:HGNC Symbol;Acc:HGNC:9784]

RAB5C	RAB5C, member RAS oncogene family [Source:HGNC Symbol;Acc:HGNC:9785]
ARF6	ADP ribosylation factor 6 [Source:HGNC Symbol;Acc:HGNC:659]
PLA2G16	phospholipase A2 group XVI [Source:HGNC Symbol;Acc:HGNC:17825]
CHRD	chordin [Source:HGNC Symbol;Acc:HGNC:1949]
NOG	noggin [Source:HGNC Symbol;Acc:HGNC:7866]
NBL1	neuroblastoma 1, DAN family BMP antagonist [Source:HGNC Symbol;Acc:HGNC:7650]
GREM1	gremlin 1, DAN family BMP antagonist [Source:HGNC Symbol;Acc:HGNC:2001]
GREM2	gremlin 2, DAN family BMP antagonist [Source:HGNC Symbol;Acc:HGNC:17655]
DCN	decorin [Source:HGNC Symbol;Acc:HGNC:2705]
FMOD	fibromodulin [Source:HGNC Symbol;Acc:HGNC:3774]
LEFTY1	left-right determination factor 1 [Source:HGNC Symbol;Acc:HGNC:6552]
LEFTY2	left-right determination factor 2 [Source:HGNC Symbol;Acc:HGNC:3122]
FST	follistatin [Source:HGNC Symbol;Acc:HGNC:3971]
INHBB	inhibin beta B subunit [Source:HGNC Symbol;Acc:HGNC:6067]
BMP8A	bone morphogenetic protein 8a [Source:HGNC Symbol;Acc:HGNC:21650]
GDF5	growth differentiation factor 5 [Source:HGNC Symbol;Acc:HGNC:4220]
GDF6	growth differentiation factor 6 [Source:HGNC Symbol;Acc:HGNC:4221]
GDF7	growth differentiation factor 7 [Source:HGNC Symbol;Acc:HGNC:4222]
AMH	anti-Mullerian hormone [Source:HGNC Symbol;Acc:HGNC:464]
THSD4	thrombospondin type 1 domain containing 4 [Source:HGNC Symbol;Acc:HGNC:25835]
FBN1	fibrillin 1 [Source:HGNC Symbol;Acc:HGNC:3603]
LTBP1	latent transforming growth factor beta binding protein 1 [Source:HGNC Symbol;Acc:HGNC:6714]
TGFB2	transforming growth factor beta 2 [Source:HGNC Symbol;Acc:HGNC:11768]
TGFB3	transforming growth factor beta 3 [Source:HGNC Symbol;Acc:HGNC:11769]
INHBA	inhibin beta A subunit [Source:HGNC Symbol;Acc:HGNC:6066]
INHBC	inhibin beta C subunit [Source:HGNC Symbol;Acc:HGNC:6068]
INHBE	inhibin beta E subunit [Source:HGNC Symbol;Acc:HGNC:24029]
NODAL	nodal growth differentiation factor [Source:HGNC Symbol;Acc:HGNC:7865]
HFE2	hemochromatosis type 2 (juvenile) [Source:HGNC Symbol;Acc:HGNC:4887]
BMPR1A	bone morphogenetic protein receptor type 1A [Source:HGNC Symbol;Acc:HGNC:1076]
BMPR1B	bone morphogenetic protein receptor type 1B [Source:HGNC Symbol;Acc:HGNC:1077]
ACVR1	activin A receptor type 1 [Source:HGNC Symbol;Acc:HGNC:171]
ACVR2A	activin A receptor type 2A [Source:HGNC Symbol;Acc:HGNC:173]
RGMA	repulsive guidance molecule family member a [Source:HGNC Symbol;Acc:HGNC:30308]
RGMB	repulsive guidance molecule family member b [Source:HGNC Symbol;Acc:HGNC:26896]
AMHR2	anti-Mullerian hormone receptor type 2 [Source:HGNC Symbol;Acc:HGNC:465]
ACVR1B	activin A receptor type 1B [Source:HGNC Symbol;Acc:HGNC:172]
ACVR2B	activin A receptor type 2B [Source:HGNC Symbol;Acc:HGNC:174]
ACVR1C	activin A receptor type 1C [Source:HGNC Symbol;Acc:HGNC:18123]
BAMBI	BMP and activin membrane bound inhibitor [Source:HGNC Symbol;Acc:HGNC:30251]
SMURF1	SMAD specific E3 ubiquitin protein ligase 1 [Source:HGNC Symbol;Acc:HGNC:16807]

SMURF2	SMAD specific E3 ubiquitin protein ligase 2 [Source:HGNC Symbol;Acc:HGNC:16809]
ZFYVE9	zinc finger FYVE-type containing 9 [Source:HGNC Symbol;Acc:HGNC:6775]
ZFYVE16	zinc finger FYVE-type containing 16 [Source:HGNC Symbol;Acc:HGNC:20756]
HAMP	hepcidin antimicrobial peptide [Source:HGNC Symbol;Acc:HGNC:15598]
ID1	inhibitor of DNA binding 1, HLH protein [Source:HGNC Symbol;Acc:HGNC:5360]
ID2	inhibitor of DNA binding 2, HLH protein [Source:HGNC Symbol;Acc:HGNC:5361]
ID3	inhibitor of DNA binding 3, HLH protein [Source:HGNC Symbol;Acc:HGNC:5362]
ID4	inhibitor of DNA binding 4, HLH protein [Source:HGNC Symbol;Acc:HGNC:5363]
RBL1	RB transcriptional corepressor like 1 [Source:HGNC Symbol;Acc:HGNC:9893]
E2F5	E2F transcription factor 5 [Source:HGNC Symbol;Acc:HGNC:3119]
TFDP1	transcription factor Dp-1 [Source:HGNC Symbol;Acc:HGNC:11749]
TGIF1	TGFB induced factor homeobox 1 [Source:HGNC Symbol;Acc:HGNC:11776]
TGIF2	TGFB induced factor homeobox 2 [Source:HGNC Symbol;Acc:HGNC:15764]
PITX2	paired like homeodomain 2 [Source:HGNC Symbol;Acc:HGNC:9005]
RBX1	ring-box 1 [Source:HGNC Symbol;Acc:HGNC:9928]
CUL1	cullin 1 [Source:HGNC Symbol;Acc:HGNC:2551]
SKP1	S-phase kinase-associated protein 1 [Source:HGNC Symbol;Acc:HGNC:10899]
MINOS1	mitochondrial inner membrane organizing system 1 [Source:HGNC Symbol;Acc:HGNC:32068]
IFNGR1	interferon gamma receptor 1 [Source:HGNC Symbol;Acc:HGNC:5439]
IFNGR2	interferon gamma receptor 2 (interferon gamma transducer 1) [Source:HGNC Symbol;Acc:HGNC:5440]
MKNK1	MAP kinase interacting serine/threonine kinase 1 [Source:HGNC Symbol;Acc:HGNC:7110]
MKNK2	MAP kinase interacting serine/threonine kinase 2 [Source:HGNC Symbol;Acc:HGNC:7111]
CUL2	cullin 2 [Source:HGNC Symbol;Acc:HGNC:2552]
ARNT	aryl hydrocarbon receptor nuclear translocator [Source:HGNC Symbol;Acc:HGNC:700]
CAMK2D	calcium/calmodulin dependent protein kinase II delta [Source:HGNC Symbol;Acc:HGNC:1462]
CAMK2G	calcium/calmodulin dependent protein kinase II gamma [Source:HGNC Symbol;Acc:HGNC:1463]
TIMP1	TIMP metallopeptidase inhibitor 1 [Source:HGNC Symbol;Acc:HGNC:11820]
LTBR	lymphotoxin beta receptor [Source:HGNC Symbol;Acc:HGNC:6718]
TF	transferrin [Source:HGNC Symbol;Acc:HGNC:11740]
TFRC	transferrin receptor [Source:HGNC Symbol;Acc:HGNC:11763]
SERPINE1	serpin family E member 1 [Source:HGNC Symbol;Acc:HGNC:8583]
EDN1	endothelin 1 [Source:HGNC Symbol;Acc:HGNC:3176]
NOS2	nitric oxide synthase 2 [Source:HGNC Symbol;Acc:HGNC:7873]
HMOX1	heme oxygenase 1 [Source:HGNC Symbol;Acc:HGNC:5013]
TCEB2	transcription elongation factor B subunit 2 [Source:HGNC Symbol;Acc:HGNC:11619]
TCEB3	transcription elongation factor B subunit 3 [Source:HGNC Symbol;Acc:HGNC:11620]
CAB39	calcium binding protein 39 [Source:HGNC Symbol;Acc:HGNC:20292]
CAB39L	calcium binding protein 39 like [Source:HGNC Symbol;Acc:HGNC:20290]
STRADA	STE20-related kinase adaptor alpha [Source:HGNC Symbol;Acc:HGNC:30172]
STRADB	STE20-related kinase adaptor beta [Source:HGNC Symbol;Acc:HGNC:13205]
LEP	leptin [Source:HGNC Symbol;Acc:HGNC:6553]

LEPR	leptin receptor [Source:HGNC Symbol;Acc:HGNC:6554]
ADRA1A	adrenoceptor alpha 1A [Source:HGNC Symbol;Acc:HGNC:277]
ADIPOQ	adiponectin, C1Q and collagen domain containing [Source:HGNC Symbol;Acc:HGNC:13633]
ADIPOR1	adiponectin receptor 1 [Source:HGNC Symbol;Acc:HGNC:24040]
ADIPOR2	adiponectin receptor 2 [Source:HGNC Symbol;Acc:HGNC:24041]
PFKFB1	6-phosphofructo-2-kinase/fructose-2,6-biphosphatase 1 [Source:HGNC Symbol;Acc:HGNC:8872]
PFKFB2	6-phosphofructo-2-kinase/fructose-2,6-biphosphatase 2 [Source:HGNC Symbol;Acc:HGNC:8873]
PFKFB4	6-phosphofructo-2-kinase/fructose-2,6-biphosphatase 4 [Source:HGNC Symbol;Acc:HGNC:8875]
HNF4A	hepatocyte nuclear factor 4 alpha [Source:HGNC Symbol;Acc:HGNC:5024]
ELAVL1	ELAV like RNA binding protein 1 [Source:HGNC Symbol;Acc:HGNC:3312]
CCNA2	cyclin A2 [Source:HGNC Symbol;Acc:HGNC:1578]
CCNA1	cyclin A1 [Source:HGNC Symbol;Acc:HGNC:1577]
EEF2K	eukaryotic elongation factor 2 kinase [Source:HGNC Symbol;Acc:HGNC:24615]
SREBF1	sterol regulatory element binding transcription factor 1 [Source:HGNC Symbol;Acc:HGNC:11289]
MLYCD	malonyl-CoA decarboxylase [Source:HGNC Symbol;Acc:HGNC:7150]
TBC1D1	TBC1 domain family member 1 [Source:HGNC Symbol;Acc:HGNC:11578]
RAB2A	RAB2A, member RAS oncogene family [Source:HGNC Symbol;Acc:HGNC:9763]
RAB10	RAB10, member RAS oncogene family [Source:HGNC Symbol;Acc:HGNC:9759]
RAB11B	RAB11B, member RAS oncogene family [Source:HGNC Symbol;Acc:HGNC:9761]
RAB14	RAB14, member RAS oncogene family [Source:HGNC Symbol;Acc:HGNC:16524]
CACNA2D1	calcium voltage-gated channel auxiliary subunit alpha2delta 1 [Source:HGNC Symbol;Acc:HGNC:1399]
CACNA2D2	calcium voltage-gated channel auxiliary subunit alpha2delta 2 [Source:HGNC Symbol;Acc:HGNC:1400]
CACNA2D3	calcium voltage-gated channel auxiliary subunit alpha2delta 3 [Source:HGNC Symbol;Acc:HGNC:15460]
CACNA2D4	calcium voltage-gated channel auxiliary subunit alpha2delta 4 [Source:HGNC Symbol;Acc:HGNC:20202]
CACNB1	calcium voltage-gated channel auxiliary subunit beta 1 [Source:HGNC Symbol;Acc:HGNC:1401]
CACNB2	calcium voltage-gated channel auxiliary subunit beta 2 [Source:HGNC Symbol;Acc:HGNC:1402]
CACNB3	calcium voltage-gated channel auxiliary subunit beta 3 [Source:HGNC Symbol;Acc:HGNC:1403]
CACNB4	calcium voltage-gated channel auxiliary subunit beta 4 [Source:HGNC Symbol;Acc:HGNC:1404]
CACNG1	calcium voltage-gated channel auxiliary subunit gamma 1 [Source:HGNC Symbol;Acc:HGNC:1405]
CACNG2	calcium voltage-gated channel auxiliary subunit gamma 2 [Source:HGNC Symbol;Acc:HGNC:1406]
CACNG3	calcium voltage-gated channel auxiliary subunit gamma 3 [Source:HGNC Symbol;Acc:HGNC:1407]
CACNG4	calcium voltage-gated channel auxiliary subunit gamma 4 [Source:HGNC Symbol;Acc:HGNC:1408]
CACNG5	calcium voltage-gated channel auxiliary subunit gamma 5 [Source:HGNC Symbol;Acc:HGNC:1409]
CACNG6	calcium voltage-gated channel auxiliary subunit gamma 6 [Source:HGNC Symbol;Acc:HGNC:13625]

CACNG7	calcium voltage-gated channel auxiliary subunit gamma 7 [Source:HGNC Symbol;Acc:HGNC:13626]
CACNG8	calcium voltage-gated channel auxiliary subunit gamma 8 [Source:HGNC Symbol;Acc:HGNC:13628]
LAMTOR3	late endosomal/lysosomal adaptor, MAPK and MTOR activator 3 [Source:HGNC Symbol;Acc:HGNC:15606]
RPS6KA3	ribosomal protein S6 kinase A3 [Source:HGNC Symbol;Acc:HGNC:10432]
RPS6KA1	ribosomal protein S6 kinase A1 [Source:HGNC Symbol;Acc:HGNC:10430]
RPS6KA2	ribosomal protein S6 kinase A2 [Source:HGNC Symbol;Acc:HGNC:10431]
RPS6KA6	ribosomal protein S6 kinase A6 [Source:HGNC Symbol;Acc:HGNC:10435]
ELK4	ELK4, ETS transcription factor [Source:HGNC Symbol;Acc:HGNC:3326]
SRF	serum response factor [Source:HGNC Symbol;Acc:HGNC:11291]
IL1R1	interleukin 1 receptor type 1 [Source:HGNC Symbol;Acc:HGNC:5993]
IL1RAP	interleukin 1 receptor accessory protein [Source:HGNC Symbol;Acc:HGNC:5995]
TRADD	TNFRSF1A associated via death domain [Source:HGNC Symbol;Acc:HGNC:12030]
DAXX	death-domain associated protein [Source:HGNC Symbol;Acc:HGNC:2681]
GADD45A	growth arrest and DNA damage inducible alpha [Source:HGNC Symbol;Acc:HGNC:4095]
GADD45B	growth arrest and DNA damage inducible beta [Source:HGNC Symbol;Acc:HGNC:4096]
GADD45G	growth arrest and DNA damage inducible gamma [Source:HGNC Symbol;Acc:HGNC:4097]
ECSIT	ECSIT signalling integrator [Source:HGNC Symbol;Acc:HGNC:29548]
MAP4K4	mitogen-activated protein kinase kinase kinase kinase 4 [Source:HGNC Symbol;Acc:HGNC:6866]
MAP3K8	mitogen-activated protein kinase kinase kinase 8 [Source:HGNC Symbol;Acc:HGNC:6860]
MAP3K11	mitogen-activated protein kinase kinase kinase 11 [Source:HGNC Symbol;Acc:HGNC:6850]
MAP3K3	mitogen-activated protein kinase kinase kinase 3 [Source:HGNC Symbol;Acc:HGNC:6855]
MAP3K13	mitogen-activated protein kinase kinase kinase 13 [Source:HGNC Symbol;Acc:HGNC:6852]
MAP3K12	mitogen-activated protein kinase kinase kinase 12 [Source:HGNC Symbol;Acc:HGNC:6851]
MAP3K6	mitogen-activated protein kinase kinase kinase 6 [Source:HGNC Symbol;Acc:HGNC:6858]
MAP3K4	mitogen-activated protein kinase kinase kinase 4 [Source:HGNC Symbol;Acc:HGNC:6856]
MAPK8IP1	mitogen-activated protein kinase 8 interacting protein 1 [Source:HGNC Symbol;Acc:HGNC:6882]
MAPK8IP2	mitogen-activated protein kinase 8 interacting protein 2 [Source:HGNC Symbol;Acc:HGNC:6883]
MAPK8IP3	mitogen-activated protein kinase 8 interacting protein 3 [Source:HGNC Symbol;Acc:HGNC:6884]
ARRB1	arrestin beta 1 [Source:HGNC Symbol;Acc:HGNC:711]
ARRB2	arrestin beta 2 [Source:HGNC Symbol;Acc:HGNC:712]
MAPKAPK5	mitogen-activated protein kinase-activated protein kinase 5 [Source:HGNC Symbol;Acc:HGNC:6889]
RPS6KA5	ribosomal protein S6 kinase A5 [Source:HGNC Symbol;Acc:HGNC:10434]
RPS6KA4	ribosomal protein S6 kinase A4 [Source:HGNC Symbol;Acc:HGNC:10433]
CDC25B	cell division cycle 25B [Source:HGNC Symbol;Acc:HGNC:1726]
JUND	JunD proto-oncogene, AP-1 transcription factor subunit [Source:HGNC Symbol;Acc:HGNC:6206]
MAX	MYC associated factor X [Source:HGNC Symbol;Acc:HGNC:6913]
MEF2C	myocyte enhancer factor 2C [Source:HGNC Symbol;Acc:HGNC:6996]
PPM1A	protein phosphatase, Mg2+/Mn2+ dependent 1A [Source:HGNC Symbol;Acc:HGNC:9275]
PTPRR	protein tyrosine phosphatase, receptor type R [Source:HGNC Symbol;Acc:HGNC:9680]

PTPN5	protein tyrosine phosphatase, non-receptor type 5 [Source:HGNC Symbol;Acc:HGNC:9657]
PTPN7	protein tyrosine phosphatase, non-receptor type 7 [Source:HGNC Symbol;Acc:HGNC:9659]
DUSP1	dual specificity phosphatase 1 [Source:HGNC Symbol;Acc:HGNC:3064]
DUSP4	dual specificity phosphatase 4 [Source:HGNC Symbol;Acc:HGNC:3070]
DUSP2	dual specificity phosphatase 2 [Source:HGNC Symbol;Acc:HGNC:3068]
DUSP7	dual specificity phosphatase 7 [Source:HGNC Symbol;Acc:HGNC:3073]
DUSP8	dual specificity phosphatase 8 [Source:HGNC Symbol;Acc:HGNC:3074]
DUSP5	dual specificity phosphatase 5 [Source:HGNC Symbol;Acc:HGNC:3071]
DUSP16	dual specificity phosphatase 16 [Source:HGNC Symbol;Acc:HGNC:17909]
DUSP6	dual specificity phosphatase 6 [Source:HGNC Symbol;Acc:HGNC:3072]
DUSP9	dual specificity phosphatase 9 [Source:HGNC Symbol;Acc:HGNC:3076]
DUSP10	dual specificity phosphatase 10 [Source:HGNC Symbol;Acc:HGNC:3065]
DUSP3	dual specificity phosphatase 3 [Source:HGNC Symbol;Acc:HGNC:3069]
PPP5C	protein phosphatase 5 catalytic subunit [Source:HGNC Symbol;Acc:HGNC:9322]
PPM1B	protein phosphatase, Mg2+/Mn2+ dependent 1B [Source:HGNC Symbol;Acc:HGNC:9276]
HSPA2	heat shock protein family A (Hsp70) member 2 [Source:HGNC Symbol;Acc:HGNC:5235]
HSPA6	heat shock protein family A (Hsp70) member 6 [Source:HGNC Symbol;Acc:HGNC:5239]
MECOM	MDS1 and EVI1 complex locus [Source:HGNC Symbol;Acc:HGNC:3498]
MAP3K14	mitogen-activated protein kinase kinase kinase 14 [Source:HGNC Symbol;Acc:HGNC:6853]
RELB	RELB proto-oncogene, NF-kB subunit [Source:HGNC Symbol;Acc:HGNC:9956]
DOCK4	dedicator of cytokinesis 4 [Source:HGNC Symbol;Acc:HGNC:19192]
F2RL3	F2R like thrombin/trypsin receptor 3 [Source:HGNC Symbol;Acc:HGNC:3540]
FPR1	formyl peptide receptor 1 [Source:HGNC Symbol;Acc:HGNC:3826]
ADORA2A	adenosine A2a receptor [Source:HGNC Symbol;Acc:HGNC:263]
ADORA2B	adenosine A2b receptor [Source:HGNC Symbol;Acc:HGNC:264]
RAPGEF3	Rap guanine nucleotide exchange factor 3 [Source:HGNC Symbol;Acc:HGNC:16629]
RAPGEF4	Rap guanine nucleotide exchange factor 4 [Source:HGNC Symbol;Acc:HGNC:16626]
MAGI3	membrane associated guanylate kinase, WW and PDZ domain containing 3 [Source:HGNC Symbol;Acc:HGNC:29647]
LCP2	lymphocyte cytosolic protein 2 [Source:HGNC Symbol;Acc:HGNC:6529]
SKAP1	src kinase associated phosphoprotein 1 [Source:HGNC Symbol;Acc:HGNC:15605]
PRKD3	protein kinase D3 [Source:HGNC Symbol;Acc:HGNC:9408]
PRKD2	protein kinase D2 [Source:HGNC Symbol;Acc:HGNC:17293]
CNR1	cannabinoid receptor 1 (brain) [Source:HGNC Symbol;Acc:HGNC:2159]
RAP1GAP	RAP1 GTPase activating protein [Source:HGNC Symbol;Acc:HGNC:9858]
SIPA1L1	signal induced proliferation associated 1 like 1 [Source:HGNC Symbol;Acc:HGNC:20284]
SIPA1	signal-induced proliferation-associated 1 [Source:HGNC Symbol;Acc:HGNC:10885]
SIPA1L2	signal induced proliferation associated 1 like 2 [Source:HGNC Symbol;Acc:HGNC:23800]
SIPA1L3	signal induced proliferation associated 1 like 3 [Source:HGNC Symbol;Acc:HGNC:23801]
APBB1IP	amyloid beta precursor protein binding family B member 1 interacting protein [Source:HGNC Symbol;Acc:HGNC:17379]
EVL	Enah/Vasp-like [Source:HGNC Symbol;Acc:HGNC:20234]

ARAP3	ArfGAP with RhoGAP domain, ankyrin repeat and PH domain 3 [Source:HGNC Symbol;Acc:HGNC:24097]
KRIT1	KRIT1, ankyrin repeat containing [Source:HGNC Symbol;Acc:HGNC:1573]
RGS14	regulator of G-protein signaling 14 [Source:HGNC Symbol;Acc:HGNC:9996]
FYB	FYN binding protein [Source:HGNC Symbol;Acc:HGNC:4036]
PORCN	porcupine homolog (Drosophila) [Source:HGNC Symbol;Acc:HGNC:17652]
WNT2	Wnt family member 2 [Source:HGNC Symbol;Acc:HGNC:12780]
WNT2B	Wnt family member 2B [Source:HGNC Symbol;Acc:HGNC:12781]
WNT3	Wnt family member 3 [Source:HGNC Symbol;Acc:HGNC:12782]
WNT3A	Wnt family member 3A [Source:HGNC Symbol;Acc:HGNC:15983]
WNT4	Wnt family member 4 [Source:HGNC Symbol;Acc:HGNC:12783]
WNT5A	Wnt family member 5A [Source:HGNC Symbol;Acc:HGNC:12784]
WNT5B	Wnt family member 5B [Source:HGNC Symbol;Acc:HGNC:16265]
WNT6	Wnt family member 6 [Source:HGNC Symbol;Acc:HGNC:12785]
WNT7A	Wnt family member 7A [Source:HGNC Symbol;Acc:HGNC:12786]
WNT7B	Wnt family member 7B [Source:HGNC Symbol;Acc:HGNC:12787]
WNT8A	Wnt family member 8A [Source:HGNC Symbol;Acc:HGNC:12788]
WNT8B	Wnt family member 8B [Source:HGNC Symbol;Acc:HGNC:12789]
WNT9A	Wnt family member 9A [Source:HGNC Symbol;Acc:HGNC:12778]
WNT9B	Wnt family member 9B [Source:HGNC Symbol;Acc:HGNC:12779]
WNT10B	Wnt family member 10B [Source:HGNC Symbol;Acc:HGNC:12775]
WNT10A	Wnt family member 10A [Source:HGNC Symbol;Acc:HGNC:13829]
WNT11	Wnt family member 11 [Source:HGNC Symbol;Acc:HGNC:12776]
WNT16	Wnt family member 16 [Source:HGNC Symbol;Acc:HGNC:16267]
CER1	cerberus 1, DAN family BMP antagonist [Source:HGNC Symbol;Acc:HGNC:1862]
NOTUM	NOTUM, palmitoleoyl-protein carboxylesterase [Source:HGNC Symbol;Acc:HGNC:27106]
WIF1	WNT inhibitory factor 1 [Source:HGNC Symbol;Acc:HGNC:18081]
SERPINF1	serpin family F member 1 [Source:HGNC Symbol;Acc:HGNC:8824]
SOST	sclerostin [Source:HGNC Symbol;Acc:HGNC:13771]
DKK1	dickkopf WNT signaling pathway inhibitor 1 [Source:HGNC Symbol;Acc:HGNC:2891]
DKK2	dickkopf WNT signaling pathway inhibitor 2 [Source:HGNC Symbol;Acc:HGNC:2892]
DKK4	dickkopf WNT signaling pathway inhibitor 4 [Source:HGNC Symbol;Acc:HGNC:2894]
SFRP1	secreted frizzled related protein 1 [Source:HGNC Symbol;Acc:HGNC:10776]
SFRP2	secreted frizzled related protein 2 [Source:HGNC Symbol;Acc:HGNC:10777]
SFRP4	secreted frizzled related protein 4 [Source:HGNC Symbol;Acc:HGNC:10778]
SFRP5	secreted frizzled related protein 5 [Source:HGNC Symbol;Acc:HGNC:10779]
RSPO1	R-spondin 1 [Source:HGNC Symbol;Acc:HGNC:21679]
RSPO2	R-spondin 2 [Source:HGNC Symbol;Acc:HGNC:28583]
RSPO3	R-spondin 3 [Source:HGNC Symbol;Acc:HGNC:20866]
RSPO4	R-spondin 4 [Source:HGNC Symbol;Acc:HGNC:16175]
LGR4	leucine rich repeat containing G protein-coupled receptor 4 [Source:HGNC Symbol;Acc:HGNC:13299]

LGR5	leucine rich repeat containing G protein-coupled receptor 5 [Source:HGNC Symbol;Acc:HGNC:4504]
LGR6	leucine rich repeat containing G protein-coupled receptor 6 [Source:HGNC Symbol;Acc:HGNC:19719]
RNF43	ring finger protein 43 [Source:HGNC Symbol;Acc:HGNC:18505]
ZNRF3	zinc and ring finger 3 [Source:HGNC Symbol;Acc:HGNC:18126]
FZD1	frizzled class receptor 1 [Source:HGNC Symbol;Acc:HGNC:4038]
FZD7	frizzled class receptor 7 [Source:HGNC Symbol;Acc:HGNC:4045]
FZD2	frizzled class receptor 2 [Source:HGNC Symbol;Acc:HGNC:4040]
FZD3	frizzled class receptor 3 [Source:HGNC Symbol;Acc:HGNC:4041]
FZD4	frizzled class receptor 4 [Source:HGNC Symbol;Acc:HGNC:4042]
FZD5	frizzled class receptor 5 [Source:HGNC Symbol;Acc:HGNC:4043]
FZD8	frizzled class receptor 8 [Source:HGNC Symbol;Acc:HGNC:4046]
FZD6	frizzled class receptor 6 [Source:HGNC Symbol;Acc:HGNC:4044]
FZD10	frizzled class receptor 10 [Source:HGNC Symbol;Acc:HGNC:4039]
FZD9	frizzled class receptor 9 [Source:HGNC Symbol;Acc:HGNC:4047]
LRP5	LDL receptor related protein 5 [Source:HGNC Symbol;Acc:HGNC:6697]
LRP6	LDL receptor related protein 6 [Source:HGNC Symbol;Acc:HGNC:6698]
CSNK1E	casein kinase 1 epsilon [Source:HGNC Symbol;Acc:HGNC:2453]
DVL3	dishevelled segment polarity protein 3 [Source:HGNC Symbol;Acc:HGNC:3087]
DVL2	dishevelled segment polarity protein 2 [Source:HGNC Symbol;Acc:HGNC:3086]
DVL1	dishevelled segment polarity protein 1 [Source:HGNC Symbol;Acc:HGNC:3084]
FRAT1	frequently rearranged in advanced T-cell lymphomas 1 [Source:HGNC Symbol;Acc:HGNC:3944]
FRAT2	frequently rearranged in advanced T-cell lymphomas 2 [Source:HGNC Symbol;Acc:HGNC:16048]
NKD1	naked cuticle homolog 1 [Source:HGNC Symbol;Acc:HGNC:17045]
NKD2	naked cuticle homolog 2 [Source:HGNC Symbol;Acc:HGNC:17046]
CXXC4	CXXC finger protein 4 [Source:HGNC Symbol;Acc:HGNC:24593]
SENP2	SUMO1/sentrin/SMT3 specific peptidase 2 [Source:HGNC Symbol;Acc:HGNC:23116]
AXIN1	axin 1 [Source:HGNC Symbol;Acc:HGNC:903]
AXIN2	axin 2 [Source:HGNC Symbol;Acc:HGNC:904]
CSNK1A1L	casein kinase 1 alpha 1 like [Source:HGNC Symbol;Acc:HGNC:20289]
CSNK1A1	casein kinase 1 alpha 1 [Source:HGNC Symbol;Acc:HGNC:2451]
CTNNBIP1	catenin beta interacting protein 1 [Source:HGNC Symbol;Acc:HGNC:16913]
CBY1	chibby family member 1, beta catenin antagonist [Source:HGNC Symbol;Acc:HGNC:1307]
CHD8	chromodomain helicase DNA binding protein 8 [Source:HGNC Symbol;Acc:HGNC:20153]
SOX17	SRY-box 17 [Source:HGNC Symbol;Acc:HGNC:18122]
CTBP1	C-terminal binding protein 1 [Source:HGNC Symbol;Acc:HGNC:2494]
CTBP2	C-terminal binding protein 2 [Source:HGNC Symbol;Acc:HGNC:2495]
TLE1	transducin like enhancer of split 1 [Source:HGNC Symbol;Acc:HGNC:11837]
TLE2	transducin like enhancer of split 2 [Source:HGNC Symbol;Acc:HGNC:11838]
TLE3	transducin like enhancer of split 3 [Source:HGNC Symbol;Acc:HGNC:11839]
TLE4	transducin like enhancer of split 4 [Source:HGNC Symbol;Acc:HGNC:11840]

TLE6	transducin like enhancer of split 6 [Source:HGNC Symbol;Acc:HGNC:30788]
CTNND2	catenin delta 2 [Source:HGNC Symbol;Acc:HGNC:2516]
RUVBL1	RuvB like AAA ATPase 1 [Source:HGNC Symbol;Acc:HGNC:10474]
FOSL1	FOS like 1, AP-1 transcription factor subunit [Source:HGNC Symbol;Acc:HGNC:13718]
WISP1	WNT1 inducible signaling pathway protein 1 [Source:HGNC Symbol;Acc:HGNC:12769]
PPARD	peroxisome proliferator activated receptor delta [Source:HGNC Symbol;Acc:HGNC:9235]
PSEN1	presenilin 1 [Source:HGNC Symbol;Acc:HGNC:9508]
SIAH1	siah E3 ubiquitin protein ligase 1 [Source:HGNC Symbol;Acc:HGNC:10857]
CACYBP	calcyclin binding protein [Source:HGNC Symbol;Acc:HGNC:30423]
TBL1X	transducin (beta)-like 1X-linked [Source:HGNC Symbol;Acc:HGNC:11585]
TBL1Y	transducin (beta)-like 1, Y-linked [Source:HGNC Symbol;Acc:HGNC:18502]
TBL1XR1	transducin (beta)-like 1 X-linked receptor 1 [Source:HGNC Symbol;Acc:HGNC:29529]
BTRC	beta-transducin repeat containing E3 ubiquitin protein ligase [Source:HGNC Symbol;Acc:HGNC:1144]
FBXW11	F-box and WD repeat domain containing 11 [Source:HGNC Symbol;Acc:HGNC:13607]
GPC4	glypican 4 [Source:HGNC Symbol;Acc:HGNC:4452]
ROR1	receptor tyrosine kinase-like orphan receptor 1 [Source:HGNC Symbol;Acc:HGNC:10256]
ROR2	receptor tyrosine kinase like orphan receptor 2 [Source:HGNC Symbol;Acc:HGNC:10257]
RYK	receptor-like tyrosine kinase [Source:HGNC Symbol;Acc:HGNC:10481]
VANGL2	VANGL planar cell polarity protein 2 [Source:HGNC Symbol;Acc:HGNC:15511]
VANGL1	VANGL planar cell polarity protein 1 [Source:HGNC Symbol;Acc:HGNC:15512]
PRICKLE1	prickle planar cell polarity protein 1 [Source:HGNC Symbol;Acc:HGNC:17019]
PRICKLE2	prickle planar cell polarity protein 2 [Source:HGNC Symbol;Acc:HGNC:20340]
PRICKLE4	prickle planar cell polarity protein 4 [Source:HGNC Symbol;Acc:HGNC:16805]
PRICKLE3	prickle planar cell polarity protein 3 [Source:HGNC Symbol;Acc:HGNC:6645]
INVS	inversin [Source:HGNC Symbol;Acc:HGNC:17870]
DAAM1	dishevelled associated activator of morphogenesis 1 [Source:HGNC Symbol;Acc:HGNC:18142]
DAAM2	dishevelled associated activator of morphogenesis 2 [Source:HGNC Symbol;Acc:HGNC:18143]
NFATC4	nuclear factor of activated T-cells 4 [Source:HGNC Symbol;Acc:HGNC:7778]
DLL3	delta like canonical Notch ligand 3 [Source:HGNC Symbol;Acc:HGNC:2909]
DLL1	delta like canonical Notch ligand 1 [Source:HGNC Symbol;Acc:HGNC:2908]
JAG1	jagged 1 [Source:HGNC Symbol;Acc:HGNC:6188]
JAG2	jagged 2 [Source:HGNC Symbol;Acc:HGNC:6189]
MFNG	MFNG O-fucosylpeptide 3-beta-N-acetylglucosaminyltransferase [Source:HGNC Symbol;Acc:HGNC:7038]
LFNG	LFNG O-fucosylpeptide 3-beta-N-acetylglucosaminyltransferase [Source:HGNC Symbol;Acc:HGNC:6560]
RFNG	RFNG O-fucosylpeptide 3-beta-N-acetylglucosaminyltransferase [Source:HGNC Symbol;Acc:HGNC:9974]
RBPJL	recombination signal binding protein for immunoglobulin kappa J region like [Source:HGNC Symbol;Acc:HGNC:13761]
HES1	hes family bHLH transcription factor 1 [Source:HGNC Symbol;Acc:HGNC:5192]
HES5	hes family bHLH transcription factor 5 [Source:HGNC Symbol;Acc:HGNC:19764]

HEYL	hes related family bHLH transcription factor with YRPW motif-like [Source:HGNC Symbol;Acc:HGNC:4882]
HEY1	hes related family bHLH transcription factor with YRPW motif 1 [Source:HGNC Symbol;Acc:HGNC:4880]
HEY2	hes related family bHLH transcription factor with YRPW motif 2 [Source:HGNC Symbol;Acc:HGNC:4881]
PTCRA	pre T-cell antigen receptor alpha [Source:HGNC Symbol;Acc:HGNC:21290]
NUMB	NUMB, endocytic adaptor protein [Source:HGNC Symbol;Acc:HGNC:8060]
NUMBL	NUMB like, endocytic adaptor protein [Source:HGNC Symbol;Acc:HGNC:8061]
DTX2	deltex 2, E3 ubiquitin ligase [Source:HGNC Symbol;Acc:HGNC:15973]
DTX3L	deltex 3 like, E3 ubiquitin ligase [Source:HGNC Symbol;Acc:HGNC:30323]
DTX1	deltex 1 [Source:HGNC Symbol;Acc:HGNC:3060]
DTX3	deltex 3, E3 ubiquitin ligase [Source:HGNC Symbol;Acc:HGNC:24457]
DTX4	deltex 4, E3 ubiquitin ligase [Source:HGNC Symbol;Acc:HGNC:29151]
ADAM17	ADAM metallopeptidase domain 17 [Source:HGNC Symbol;Acc:HGNC:195]
PSEN2	presenilin 2 [Source:HGNC Symbol;Acc:HGNC:9509]
PSENEN	presenilin enhancer gamma-secretase subunit [Source:HGNC Symbol;Acc:HGNC:30100]
NCSTN	nicastrin [Source:HGNC Symbol;Acc:HGNC:17091]
APH1A	aph-1 homolog A, gamma-secretase subunit [Source:HGNC Symbol;Acc:HGNC:29509]
APH1B	aph-1 homolog B, gamma-secretase subunit [Source:HGNC Symbol;Acc:HGNC:24080]
MAML3	mastermind like transcriptional coactivator 3 [Source:HGNC Symbol;Acc:HGNC:16272]
MAML2	mastermind like transcriptional coactivator 2 [Source:HGNC Symbol;Acc:HGNC:16259]
MAML1	mastermind like transcriptional coactivator 1 [Source:HGNC Symbol;Acc:HGNC:13632]
KAT2B	lysine acetyltransferase 2B [Source:HGNC Symbol;Acc:HGNC:8638]
KAT2A	lysine acetyltransferase 2A [Source:HGNC Symbol;Acc:HGNC:4201]
SNW1	SNW domain containing 1 [Source:HGNC Symbol;Acc:HGNC:16696]
NCOR2	nuclear receptor corepressor 2 [Source:HGNC Symbol;Acc:HGNC:7673]
CIR1	corepressor interacting with RBPJ, 1 [Source:HGNC Symbol;Acc:HGNC:24217]
HDAC1	histone deacetylase 1 [Source:HGNC Symbol;Acc:HGNC:4852]
ATXN1L	ataxin 1 like [Source:HGNC Symbol;Acc:HGNC:33279]
ATXN1	ataxin 1 [Source:HGNC Symbol;Acc:HGNC:10548]
PTCH1	patched 1 [Source:HGNC Symbol;Acc:HGNC:9585]
PTCH2	patched 2 [Source:HGNC Symbol;Acc:HGNC:9586]
SMO	smoothened, frizzled class receptor [Source:HGNC Symbol;Acc:HGNC:11119]
GPR161	G protein-coupled receptor 161 [Source:HGNC Symbol;Acc:HGNC:23694]
CSNK1G2	casein kinase 1 gamma 2 [Source:HGNC Symbol;Acc:HGNC:2455]
CSNK1G3	casein kinase 1 gamma 3 [Source:HGNC Symbol;Acc:HGNC:2456]
CSNK1G1	casein kinase 1 gamma 1 [Source:HGNC Symbol;Acc:HGNC:2454]
GLI1	GLI family zinc finger 1 [Source:HGNC Symbol;Acc:HGNC:4317]
GLI2	GLI family zinc finger 2 [Source:HGNC Symbol;Acc:HGNC:4318]
GLI3	GLI family zinc finger 3 [Source:HGNC Symbol;Acc:HGNC:4319]
SUFU	SUFU negative regulator of hedgehog signaling [Source:HGNC Symbol;Acc:HGNC:16466]

KIF7	kinesin family member 7 [Source:HGNC Symbol;Acc:HGNC:30497]
HHIP	hedgehog interacting protein [Source:HGNC Symbol;Acc:HGNC:14866]
HHAT	hedgehog acyltransferase [Source:HGNC Symbol;Acc:HGNC:18270]
HHATL	hedgehog acyltransferase-like [Source:HGNC Symbol;Acc:HGNC:13242]
SHH	sonic hedgehog [Source:HGNC Symbol;Acc:HGNC:10848]
IHH	indian hedgehog [Source:HGNC Symbol;Acc:HGNC:5956]
DHH	desert hedgehog [Source:HGNC Symbol;Acc:HGNC:2865]
DISP1	dispatched RND transporter family member 1 [Source:HGNC Symbol;Acc:HGNC:19711]
SCUBE2	signal peptide, CUB domain and EGF like domain containing 2 [Source:HGNC Symbol;Acc:HGNC:30425]
BOC	BOC cell adhesion associated, oncogene regulated [Source:HGNC Symbol;Acc:HGNC:17173]
CDON	cell adhesion associated, oncogene regulated [Source:HGNC Symbol;Acc:HGNC:17104]
GAS1	growth arrest specific 1 [Source:HGNC Symbol;Acc:HGNC:4165]
MEGF8	multiple EGF like domains 8 [Source:HGNC Symbol;Acc:HGNC:3233]
MGRN1	mahogunin ring finger 1 [Source:HGNC Symbol;Acc:HGNC:20254]
GRK2	G protein-coupled receptor kinase 2 [Source:HGNC Symbol;Acc:HGNC:289]
GRK3	G protein-coupled receptor kinase 3 [Source:HGNC Symbol;Acc:HGNC:290]
EVC	EvC ciliary complex subunit 1 [Source:HGNC Symbol;Acc:HGNC:3497]
EVC2	EvC ciliary complex subunit 2 [Source:HGNC Symbol;Acc:HGNC:19747]
EFCAB7	EF-hand calcium binding domain 7 [Source:HGNC Symbol;Acc:HGNC:29379]
IQCE	IQ motif containing E [Source:HGNC Symbol;Acc:HGNC:29171]
KIF3A	kinesin family member 3A [Source:HGNC Symbol;Acc:HGNC:6319]
CUL3	cullin 3 [Source:HGNC Symbol;Acc:HGNC:2553]
SPOP	speckle type BTB/POZ protein [Source:HGNC Symbol;Acc:HGNC:11254]
SPOPL	speckle type BTB/POZ protein like [Source:HGNC Symbol;Acc:HGNC:27934]
C16orf52	chromosome 16 open reading frame 52 [Source:HGNC Symbol;Acc:HGNC:27087]
CRB2	crumbs 2, cell polarity complex component [Source:HGNC Symbol;Acc:HGNC:18688]
LIMD1	LIM domains containing 1 [Source:HGNC Symbol;Acc:HGNC:6612]
AJUBA	ajuba LIM protein [Source:HGNC Symbol;Acc:HGNC:20250]
WTIP	Wilms tumor 1 interacting protein [Source:HGNC Symbol;Acc:HGNC:20964]
FRMD1	FERM domain containing 1 [Source:HGNC Symbol;Acc:HGNC:21240]
RASSF6	Ras association domain family member 6 [Source:HGNC Symbol;Acc:HGNC:20796]
TP53BP2	tumor protein p53 binding protein 2 [Source:HGNC Symbol;Acc:HGNC:12000]
DLG4	discs large MAGUK scaffold protein 4 [Source:HGNC Symbol;Acc:HGNC:2903]
DLG5	discs large MAGUK scaffold protein 5 [Source:HGNC Symbol;Acc:HGNC:2904]
BBC3	BCL2 binding component 3 [Source:HGNC Symbol;Acc:HGNC:17868]
BIRC5	baculoviral IAP repeat containing 5 [Source:HGNC Symbol;Acc:HGNC:593]
AFP	alpha fetoprotein [Source:HGNC Symbol;Acc:HGNC:317]
IL5	interleukin 5 [Source:HGNC Symbol;Acc:HGNC:6016]
IL9	interleukin 9 [Source:HGNC Symbol;Acc:HGNC:6029]
IL10	interleukin 10 [Source:HGNC Symbol;Acc:HGNC:5962]
IL11	interleukin 11 [Source:HGNC Symbol;Acc:HGNC:5966]

IL13	interleukin 13 [Source:HGNC Symbol;Acc:HGNC:5973]
IL15	interleukin 15 [Source:HGNC Symbol;Acc:HGNC:5977]
IL17D	interleukin 17D [Source:HGNC Symbol;Acc:HGNC:5984]
IL19	interleukin 19 [Source:HGNC Symbol;Acc:HGNC:5990]
IL20	interleukin 20 [Source:HGNC Symbol;Acc:HGNC:6002]
IL21	interleukin 21 [Source:HGNC Symbol;Acc:HGNC:6005]
IL22	interleukin 22 [Source:HGNC Symbol;Acc:HGNC:14900]
IL23A	interleukin 23 subunit alpha [Source:HGNC Symbol;Acc:HGNC:15488]
IL24	interleukin 24 [Source:HGNC Symbol;Acc:HGNC:11346]
IFNE	interferon, epsilon [Source:HGNC Symbol;Acc:HGNC:18163]
IFNK	interferon kappa [Source:HGNC Symbol;Acc:HGNC:21714]
IFNL1	interferon, lambda 1 [Source:HGNC Symbol;Acc:HGNC:18363]
IFNL2	interferon, lambda 2 [Source:HGNC Symbol;Acc:HGNC:18364]
IFNL3	interferon, lambda 3 [Source:HGNC Symbol;Acc:HGNC:18365]
IFNW1	interferon, omega 1 [Source:HGNC Symbol;Acc:HGNC:5448]
LIF	leukemia inhibitory factor [Source:HGNC Symbol;Acc:HGNC:6596]
TSLP	thymic stromal lymphopoietin [Source:HGNC Symbol;Acc:HGNC:30743]
CTF1	cardiotrophin 1 [Source:HGNC Symbol;Acc:HGNC:2499]
CSF2	colony stimulating factor 2 [Source:HGNC Symbol;Acc:HGNC:2434]
CNTF	ciliary neurotrophic factor [Source:HGNC Symbol;Acc:HGNC:2169]
THPO	thrombopoietin [Source:HGNC Symbol;Acc:HGNC:11795]
IL5RA	interleukin 5 receptor subunit alpha [Source:HGNC Symbol;Acc:HGNC:6017]
IL9R	interleukin 9 receptor [Source:HGNC Symbol;Acc:HGNC:6030]
IL10RA	interleukin 10 receptor subunit alpha [Source:HGNC Symbol;Acc:HGNC:5964]
IL10RB	interleukin 10 receptor subunit beta [Source:HGNC Symbol;Acc:HGNC:5965]
IL11RA	interleukin 11 receptor subunit alpha [Source:HGNC Symbol;Acc:HGNC:5967]
IL12RB1	interleukin 12 receptor subunit beta 1 [Source:HGNC Symbol;Acc:HGNC:5971]
IL12RB2	interleukin 12 receptor subunit beta 2 [Source:HGNC Symbol;Acc:HGNC:5972]
IL13RA1	interleukin 13 receptor subunit alpha 1 [Source:HGNC Symbol;Acc:HGNC:5974]
IL13RA2	interleukin 13 receptor subunit alpha 2 [Source:HGNC Symbol;Acc:HGNC:5975]
IL15RA	interleukin 15 receptor subunit alpha [Source:HGNC Symbol;Acc:HGNC:5978]
IL20RA	interleukin 20 receptor subunit alpha [Source:HGNC Symbol;Acc:HGNC:6003]
IL20RB	interleukin 20 receptor subunit beta [Source:HGNC Symbol;Acc:HGNC:6004]
IL21R	interleukin 21 receptor [Source:HGNC Symbol;Acc:HGNC:6006]
IL22RA1	interleukin 22 receptor subunit alpha 1 [Source:HGNC Symbol;Acc:HGNC:13700]
IL22RA2	interleukin 22 receptor subunit alpha 2 [Source:HGNC Symbol;Acc:HGNC:14901]
IL23R	interleukin 23 receptor [Source:HGNC Symbol;Acc:HGNC:19100]
IL27RA	interleukin 27 receptor subunit alpha [Source:HGNC Symbol;Acc:HGNC:17290]
IL6ST	interleukin 6 signal transducer [Source:HGNC Symbol;Acc:HGNC:6021]
IFNLR1	interferon lambda receptor 1 [Source:HGNC Symbol;Acc:HGNC:18584]
LIFR	leukemia inhibitory factor receptor alpha [Source:HGNC Symbol;Acc:HGNC:6597]

CRLF2	cytokine receptor-like factor 2 [Source:HGNC Symbol;Acc:HGNC:14281]
CNTFR	ciliary neurotrophic factor receptor [Source:HGNC Symbol;Acc:HGNC:2170]
CSF2RA	colony stimulating factor 2 receptor alpha subunit [Source:HGNC Symbol;Acc:HGNC:2435]
CSF2RB	colony stimulating factor 2 receptor beta common subunit [Source:HGNC Symbol;Acc:HGNC:2436]
MPL	MPL proto-oncogene, thrombopoietin receptor [Source:HGNC Symbol;Acc:HGNC:7217]
TYK2	tyrosine kinase 2 [Source:HGNC Symbol;Acc:HGNC:12440]
STAT1	signal transducer and activator of transcription 1 [Source:HGNC Symbol;Acc:HGNC:11362]
STAT2	signal transducer and activator of transcription 2 [Source:HGNC Symbol;Acc:HGNC:11363]
STAT4	signal transducer and activator of transcription 4 [Source:HGNC Symbol;Acc:HGNC:11365]
STAT5A	signal transducer and activator of transcription 5A [Source:HGNC Symbol;Acc:HGNC:11366]
STAT5B	signal transducer and activator of transcription 5B [Source:HGNC Symbol;Acc:HGNC:11367]
STAT6	signal transducer and activator of transcription 6 [Source:HGNC Symbol;Acc:HGNC:11368]
CISH	cytokine inducible SH2 containing protein [Source:HGNC Symbol;Acc:HGNC:1984]
SOCS1	suppressor of cytokine signaling 1 [Source:HGNC Symbol;Acc:HGNC:19383]
SOCS2	suppressor of cytokine signaling 2 [Source:HGNC Symbol;Acc:HGNC:19382]
SOCS3	suppressor of cytokine signaling 3 [Source:HGNC Symbol;Acc:HGNC:19391]
SOCS4	suppressor of cytokine signaling 4 [Source:HGNC Symbol;Acc:HGNC:19392]
SOCS5	suppressor of cytokine signaling 5 [Source:HGNC Symbol;Acc:HGNC:16852]
SOCS7	suppressor of cytokine signaling 7 [Source:HGNC Symbol;Acc:HGNC:29846]
SOCS6	suppressor of cytokine signaling 6 [Source:HGNC Symbol;Acc:HGNC:16833]
PIM1	Pim-1 proto-oncogene, serine/threonine kinase [Source:HGNC Symbol;Acc:HGNC:8986]
AOX1	aldehyde oxidase 1 [Source:HGNC Symbol;Acc:HGNC:553]
GFAP	glial fibrillary acidic protein [Source:HGNC Symbol;Acc:HGNC:4235]
STAM2	signal transducing adaptor molecule 2 [Source:HGNC Symbol;Acc:HGNC:11358]
STAM	signal transducing adaptor molecule [Source:HGNC Symbol;Acc:HGNC:11357]
PTPN2	protein tyrosine phosphatase, non-receptor type 2 [Source:HGNC Symbol;Acc:HGNC:9650]
IRF9	interferon regulatory factor 9 [Source:HGNC Symbol;Acc:HGNC:6131]
PIAS1	protein inhibitor of activated STAT 1 [Source:HGNC Symbol;Acc:HGNC:2752]
PIAS2	protein inhibitor of activated STAT 2 [Source:HGNC Symbol;Acc:HGNC:17311]
PIAS3	protein inhibitor of activated STAT 3 [Source:HGNC Symbol;Acc:HGNC:16861]
PIAS4	protein inhibitor of activated STAT 4 [Source:HGNC Symbol;Acc:HGNC:17002]
FHL1	four and a half LIM domains 1 [Source:HGNC Symbol;Acc:HGNC:3702]
ADCY10	adenylate cyclase 10 (soluble) [Source:HGNC Symbol;Acc:HGNC:21285]
APELA	apelin receptor early endogenous ligand [Source:HGNC Symbol;Acc:HGNC:48925]
APLNR	apelin receptor [Source:HGNC Symbol;Acc:HGNC:339]
RYR1	ryanodine receptor 1 [Source:HGNC Symbol;Acc:HGNC:10483]
RYR2	ryanodine receptor 2 [Source:HGNC Symbol;Acc:HGNC:10484]
RYR3	ryanodine receptor 3 [Source:HGNC Symbol;Acc:HGNC:10485]
NOS1	nitric oxide synthase 1 [Source:HGNC Symbol;Acc:HGNC:7872]
SLC8A1	solute carrier family 8 member A1 [Source:HGNC Symbol;Acc:HGNC:11068]
SLC8A2	solute carrier family 8 member A2 [Source:HGNC Symbol;Acc:HGNC:11069]

SLC8A3	solute carrier family 8 member A3 [Source:HGNC Symbol;Acc:HGNC:11070]
MYL3	myosin light chain 3 [Source:HGNC Symbol;Acc:HGNC:7584]
MYL4	myosin light chain 4 [Source:HGNC Symbol;Acc:HGNC:7585]
EGR1	early growth response 1 [Source:HGNC Symbol;Acc:HGNC:3238]
PDE3B	phosphodiesterase 3B [Source:HGNC Symbol;Acc:HGNC:8779]
PLIN1	perilipin 1 [Source:HGNC Symbol;Acc:HGNC:9076]
UCP1	uncoupling protein 1 [Source:HGNC Symbol;Acc:HGNC:12517]
NRF1	nuclear respiratory factor 1 [Source:HGNC Symbol;Acc:HGNC:7996]
TFAM	transcription factor A, mitochondrial [Source:HGNC Symbol;Acc:HGNC:11741]
HDAC4	histone deacetylase 4 [Source:HGNC Symbol;Acc:HGNC:14063]
HDAC5	histone deacetylase 5 [Source:HGNC Symbol;Acc:HGNC:14068]
MEF2A	myocyte enhancer factor 2A [Source:HGNC Symbol;Acc:HGNC:6993]
BORCS8-MEF2B	BORCS8-MEF2B readthrough [Source:HGNC Symbol;Acc:HGNC:39979]
MEF2B	myocyte enhancer factor 2B [Source:HGNC Symbol;Acc:HGNC:6995]
MEF2D	myocyte enhancer factor 2D [Source:HGNC Symbol;Acc:HGNC:6997]
ACTA2	actin, alpha 2, smooth muscle, aorta [Source:HGNC Symbol;Acc:HGNC:130]
PLAT	plasminogen activator, tissue type [Source:HGNC Symbol;Acc:HGNC:9051]
LCK	LCK proto-oncogene, Src family tyrosine kinase [Source:HGNC Symbol;Acc:HGNC:6524]
BLNK	B-cell linker [Source:HGNC Symbol;Acc:HGNC:14211]
BTK	Bruton tyrosine kinase [Source:HGNC Symbol;Acc:HGNC:1133]
CARD10	caspase recruitment domain family member 10 [Source:HGNC Symbol;Acc:HGNC:16422]
CARD11	caspase recruitment domain family member 11 [Source:HGNC Symbol;Acc:HGNC:16393]
CARD14	caspase recruitment domain family member 14 [Source:HGNC Symbol;Acc:HGNC:16446]
MALT1	MALT1 paracaspase [Source:HGNC Symbol;Acc:HGNC:6819]
RIPK1	receptor interacting serine/threonine kinase 1 [Source:HGNC Symbol;Acc:HGNC:10019]
TRAF5	TNF receptor associated factor 5 [Source:HGNC Symbol;Acc:HGNC:12035]
EDA	ectodysplasin A [Source:HGNC Symbol;Acc:HGNC:3157]
EDAR	ectodysplasin A receptor [Source:HGNC Symbol;Acc:HGNC:2895]
EDARADD	EDAR-associated death domain [Source:HGNC Symbol;Acc:HGNC:14341]
CYLD	CYLD lysine 63 deubiquitinase [Source:HGNC Symbol;Acc:HGNC:2584]
EDA2R	ectodysplasin A2 receptor [Source:HGNC Symbol;Acc:HGNC:17756]
TRIM25	tripartite motif containing 25 [Source:HGNC Symbol;Acc:HGNC:12932]
TICAM1	toll like receptor adaptor molecule 1 [Source:HGNC Symbol;Acc:HGNC:18348]
TNFSF11	tumor necrosis factor superfamily member 11 [Source:HGNC Symbol;Acc:HGNC:11926]
TNFRSF11A	tumor necrosis factor receptor superfamily member 11a [Source:HGNC Symbol;Acc:HGNC:11908]
LTA	lymphotoxin alpha [Source:HGNC Symbol;Acc:HGNC:6709]
LTB	lymphotoxin beta [Source:HGNC Symbol;Acc:HGNC:6711]
TNFSF14	tumor necrosis factor superfamily member 14 [Source:HGNC Symbol;Acc:HGNC:11930]
TAB3	TGF-beta activated kinase 1/MAP3K7 binding protein 3 [Source:HGNC Symbol;Acc:HGNC:30681]
TNFSF13B	tumor necrosis factor superfamily member 13b [Source:HGNC Symbol;Acc:HGNC:11929]

TNFRSF13C	tumor necrosis factor receptor superfamily member 13C [Source:HGNC Symbol;Acc:HGNC:17755]
UBE2I	ubiquitin conjugating enzyme E2 I [Source:HGNC Symbol;Acc:HGNC:12485]
PIDD1	p53-induced death domain protein 1 [Source:HGNC Symbol;Acc:HGNC:16491]
ERC1	ELKS/RAB6-interacting/CAST family member 1 [Source:HGNC Symbol;Acc:HGNC:17072]
TRAF1	TNF receptor associated factor 1 [Source:HGNC Symbol;Acc:HGNC:12031]
BCL2A1	BCL2 related protein A1 [Source:HGNC Symbol;Acc:HGNC:991]
TNFAIP3	TNF alpha induced protein 3 [Source:HGNC Symbol;Acc:HGNC:11896]
CCL4	C-C motif chemokine ligand 4 [Source:HGNC Symbol;Acc:HGNC:10630]
CCL4L2	C-C motif chemokine ligand 4 like 2 [Source:HGNC Symbol;Acc:HGNC:24066]
PLAU	plasminogen activator, urokinase [Source:HGNC Symbol;Acc:HGNC:9052]
CCL13	C-C motif chemokine ligand 13 [Source:HGNC Symbol;Acc:HGNC:10611]
CCL19	C-C motif chemokine ligand 19 [Source:HGNC Symbol;Acc:HGNC:10617]
CCL21	C-C motif chemokine ligand 21 [Source:HGNC Symbol;Acc:HGNC:10620]
BAG4	BCL2 associated athanogene 4 [Source:HGNC Symbol;Acc:HGNC:940]
ITCH	itchy E3 ubiquitin protein ligase [Source:HGNC Symbol;Acc:HGNC:13890]
CEBPB	CCAAT/enhancer binding protein beta [Source:HGNC Symbol;Acc:HGNC:1834]
RIPK3	receptor interacting serine/threonine kinase 3 [Source:HGNC Symbol;Acc:HGNC:10021]
MLKL	mixed lineage kinase domain-like [Source:HGNC Symbol;Acc:HGNC:26617]
DNM1L	dynamin 1-like [Source:HGNC Symbol;Acc:HGNC:2973]
FADD	Fas associated via death domain [Source:HGNC Symbol;Acc:HGNC:3573]
CASP10	caspase 10 [Source:HGNC Symbol;Acc:HGNC:1500]
CCL20	C-C motif chemokine ligand 20 [Source:HGNC Symbol;Acc:HGNC:10619]
CXCL5	C-X-C motif chemokine ligand 5 [Source:HGNC Symbol;Acc:HGNC:10642]
CXCL6	C-X-C motif chemokine ligand 6 [Source:HGNC Symbol;Acc:HGNC:10643]
CX3CL1	C-X3-C motif chemokine ligand 1 [Source:HGNC Symbol;Acc:HGNC:10647]
IL18R1	interleukin 18 receptor 1 [Source:HGNC Symbol;Acc:HGNC:5988]
JUNB	JunB proto-oncogene, AP-1 transcription factor subunit [Source:HGNC Symbol;Acc:HGNC:6205]
MMP14	matrix metallopeptidase 14 [Source:HGNC Symbol;Acc:HGNC:7160]
TNFRSF1B	tumor necrosis factor receptor superfamily member 1B [Source:HGNC Symbol;Acc:HGNC:11917]
DAB2IP	DAB2 interacting protein [Source:HGNC Symbol;Acc:HGNC:17294]
IRF1	interferon regulatory factor 1 [Source:HGNC Symbol;Acc:HGNC:6116]
SKP2	S-phase kinase-associated protein 2, E3 ubiquitin protein ligase [Source:HGNC Symbol;Acc:HGNC:10901]
SETD7	SET domain containing lysine methyltransferase 7 [Source:HGNC Symbol;Acc:HGNC:30412]
USP7	ubiquitin specific peptidase 7 (herpes virus-associated) [Source:HGNC Symbol;Acc:HGNC:12630]
HOMER1	homer scaffolding protein 1 [Source:HGNC Symbol;Acc:HGNC:17512]
HOMER2	homer scaffolding protein 2 [Source:HGNC Symbol;Acc:HGNC:17513]
HOMER3	homer scaffolding protein 3 [Source:HGNC Symbol;Acc:HGNC:17514]
AGAP2	ArfGAP with GTPase domain, ankyrin repeat and PH domain 2 [Source:HGNC Symbol;Acc:HGNC:16921]
FOXG1	forkhead box G1 [Source:HGNC Symbol;Acc:HGNC:3811]

CCNB1	cyclin B1 [Source:HGNC Symbol;Acc:HGNC:1579]
CCNB2	cyclin B2 [Source:HGNC Symbol;Acc:HGNC:1580]
CCNB3	cyclin B3 [Source:HGNC Symbol;Acc:HGNC:18709]
CCNG2	cyclin G2 [Source:HGNC Symbol;Acc:HGNC:1593]
CDKN2D	cyclin dependent kinase inhibitor 2D [Source:HGNC Symbol;Acc:HGNC:1790]
PLK1	polo like kinase 1 [Source:HGNC Symbol;Acc:HGNC:9077]
PLK2	polo like kinase 2 [Source:HGNC Symbol;Acc:HGNC:19699]
PLK3	polo like kinase 3 [Source:HGNC Symbol;Acc:HGNC:2154]
PLK4	polo like kinase 4 [Source:HGNC Symbol;Acc:HGNC:11397]
BCL6	B-cell CLL/lymphoma 6 [Source:HGNC Symbol;Acc:HGNC:1001]
S1PR1	sphingosine-1-phosphate receptor 1 [Source:HGNC Symbol;Acc:HGNC:3165]
S1PR4	sphingosine-1-phosphate receptor 4 [Source:HGNC Symbol;Acc:HGNC:3170]
RAG1	recombination activating gene 1 [Source:HGNC Symbol;Acc:HGNC:9831]
RAG2	recombination activating gene 2 [Source:HGNC Symbol;Acc:HGNC:9832]
FBXO25	F-box protein 25 [Source:HGNC Symbol;Acc:HGNC:13596]
FBXO32	F-box protein 32 [Source:HGNC Symbol;Acc:HGNC:16731]
PI4KA	phosphatidylinositol 4-kinase alpha [Source:HGNC Symbol;Acc:HGNC:8983]
PI4KB	phosphatidylinositol 4-kinase beta [Source:HGNC Symbol;Acc:HGNC:8984]
PI4K2A	phosphatidylinositol 4-kinase type 2 alpha [Source:HGNC Symbol;Acc:HGNC:30031]
PI4K2B	phosphatidylinositol 4-kinase type 2 beta [Source:HGNC Symbol;Acc:HGNC:18215]
SACM1L	SAC1 suppressor of actin mutations 1-like (yeast) [Source:HGNC Symbol;Acc:HGNC:17059]
INPP5F	inositol polyphosphate-5-phosphatase F [Source:HGNC Symbol;Acc:HGNC:17054]
OCRL	OCRL, inositol polyphosphate-5-phosphatase [Source:HGNC Symbol;Acc:HGNC:8108]
INPP5B	inositol polyphosphate-5-phosphatase B [Source:HGNC Symbol;Acc:HGNC:6077]
INPP5E	inositol polyphosphate-5-phosphatase E [Source:HGNC Symbol;Acc:HGNC:21474]
SYNJ1	synaptojanin 1 [Source:HGNC Symbol;Acc:HGNC:11503]
SYNJ2	synaptojanin 2 [Source:HGNC Symbol;Acc:HGNC:11504]
INPP5D	inositol polyphosphate-5-phosphatase D [Source:HGNC Symbol;Acc:HGNC:6079]
INPPL1	inositol polyphosphate phosphatase like 1 [Source:HGNC Symbol;Acc:HGNC:6080]
INPP4A	inositol polyphosphate-4-phosphatase type I A [Source:HGNC Symbol;Acc:HGNC:6074]
INPP4B	inositol polyphosphate-4-phosphatase type II B [Source:HGNC Symbol;Acc:HGNC:6075]
MTM1	myotubularin 1 [Source:HGNC Symbol;Acc:HGNC:7448]
MTMR1	myotubularin related protein 1 [Source:HGNC Symbol;Acc:HGNC:7449]
MTMR2	myotubularin related protein 2 [Source:HGNC Symbol;Acc:HGNC:7450]
MTMR8	myotubularin related protein 8 [Source:HGNC Symbol;Acc:HGNC:16825]
MTMR6	myotubularin related protein 6 [Source:HGNC Symbol;Acc:HGNC:7453]
MTMR7	myotubularin related protein 7 [Source:HGNC Symbol;Acc:HGNC:7454]
PIK3C2G	phosphatidylinositol-4-phosphate 3-kinase catalytic subunit type 2 gamma [Source:HGNC Symbol;Acc:HGNC:8973]
PIK3C2A	phosphatidylinositol-4-phosphate 3-kinase catalytic subunit type 2 alpha [Source:HGNC Symbol;Acc:HGNC:8971]

PIK3C2B	phosphatidylinositol-4-phosphate 3-kinase catalytic subunit type 2 beta [Source:HGNC Symbol;Acc:HGNC:8972]
PLCD1	phospholipase C delta 1 [Source:HGNC Symbol;Acc:HGNC:9060]
PLCD3	phospholipase C delta 3 [Source:HGNC Symbol;Acc:HGNC:9061]
PLCD4	phospholipase C delta 4 [Source:HGNC Symbol;Acc:HGNC:9062]
PLCZ1	phospholipase C zeta 1 [Source:HGNC Symbol;Acc:HGNC:19218]
IPMK	inositol polyphosphate multikinase [Source:HGNC Symbol;Acc:HGNC:20739]
ITPKB	inositol-trisphosphate 3-kinase B [Source:HGNC Symbol;Acc:HGNC:6179]
ITPKA	inositol-trisphosphate 3-kinase A [Source:HGNC Symbol;Acc:HGNC:6178]
ITPKC	inositol-trisphosphate 3-kinase C [Source:HGNC Symbol;Acc:HGNC:14897]
INPP5A	inositol polyphosphate-5-phosphatase A [Source:HGNC Symbol;Acc:HGNC:6076]
ITPK1	inositol-tetrakisphosphate 1-kinase [Source:HGNC Symbol;Acc:HGNC:6177]
IPPK	inositol-pentakisphosphate 2-kinase [Source:HGNC Symbol;Acc:HGNC:14645]
IP6K1	inositol hexakisphosphate kinase 1 [Source:HGNC Symbol;Acc:HGNC:18360]
IP6K2	inositol hexakisphosphate kinase 2 [Source:HGNC Symbol;Acc:HGNC:17313]
IP6K3	inositol hexakisphosphate kinase 3 [Source:HGNC Symbol;Acc:HGNC:17269]
PPIP5K1	diphosphoinositol pentakisphosphate kinase 1 [Source:HGNC Symbol;Acc:HGNC:29023]
PPIP5K2	diphosphoinositol pentakisphosphate kinase 2 [Source:HGNC Symbol;Acc:HGNC:29035]
INPP1	inositol polyphosphate-1-phosphatase [Source:HGNC Symbol;Acc:HGNC:6071]
IMPA2	inositol monophosphatase 2 [Source:HGNC Symbol;Acc:HGNC:6051]
IMPA1	inositol monophosphatase 1 [Source:HGNC Symbol;Acc:HGNC:6050]
DGKZ	diacylglycerol kinase zeta [Source:HGNC Symbol;Acc:HGNC:2857]
DGKD	diacylglycerol kinase delta [Source:HGNC Symbol;Acc:HGNC:2851]
DGKI	diacylglycerol kinase iota [Source:HGNC Symbol;Acc:HGNC:2855]
DGKA	diacylglycerol kinase alpha [Source:HGNC Symbol;Acc:HGNC:2849]
DGKE	diacylglycerol kinase epsilon [Source:HGNC Symbol;Acc:HGNC:2852]
DGKB	diacylglycerol kinase beta [Source:HGNC Symbol;Acc:HGNC:2850]
DGKH	diacylglycerol kinase eta [Source:HGNC Symbol;Acc:HGNC:2854]
DGKG	diacylglycerol kinase gamma [Source:HGNC Symbol;Acc:HGNC:2853]
DGKQ	diacylglycerol kinase theta [Source:HGNC Symbol;Acc:HGNC:2856]
DGKK	diacylglycerol kinase kappa [Source:HGNC Symbol;Acc:HGNC:32395]
CDS1	CDP-diacylglycerol synthase 1 [Source:HGNC Symbol;Acc:HGNC:1800]
CDS2	CDP-diacylglycerol synthase 2 [Source:HGNC Symbol;Acc:HGNC:1801]
CDIPT	CDP-diacylglycerol--inositol 3-phosphatidyltransferase [Source:HGNC Symbol;Acc:HGNC:1769]
IMPAD1	inositol monophosphatase domain containing 1 [Source:HGNC Symbol;Acc:HGNC:26019]
TMEM55B	transmembrane protein 55B [Source:HGNC Symbol;Acc:HGNC:19299]
TMEM55A	transmembrane protein 55A [Source:HGNC Symbol;Acc:HGNC:25452]
SLC3A2	solute carrier family 3 member 2 [Source:HGNC Symbol;Acc:HGNC:11026]
SLC38A9	solute carrier family 38 member 9 [Source:HGNC Symbol;Acc:HGNC:26907]
LAMTOR1	late endosomal/lysosomal adaptor, MAPK and MTOR activator 1 [Source:HGNC Symbol;Acc:HGNC:26068]

LAMTOR2	late endosomal/lysosomal adaptor, MAPK and MTOR activator 2 [Source:HGNC Symbol;Acc:HGNC:29796]
LAMTOR4	late endosomal/lysosomal adaptor, MAPK and MTOR activator 4 [Source:HGNC Symbol;Acc:HGNC:33772]
LAMTOR5	late endosomal/lysosomal adaptor, MAPK and MTOR activator 5 [Source:HGNC Symbol;Acc:HGNC:17955]
FLCN	folliculin [Source:HGNC Symbol;Acc:HGNC:27310]
FNIP1	folliculin interacting protein 1 [Source:HGNC Symbol;Acc:HGNC:29418]
FNIP2	folliculin interacting protein 2 [Source:HGNC Symbol;Acc:HGNC:29280]
MIOS	meiosis regulator for oocyte development [Source:HGNC Symbol;Acc:HGNC:21905]
SEH1L	SEH1 like nucleoporin [Source:HGNC Symbol;Acc:HGNC:30379]
WDR24	WD repeat domain 24 [Source:HGNC Symbol;Acc:HGNC:20852]
WDR59	WD repeat domain 59 [Source:HGNC Symbol;Acc:HGNC:25706]
SEC13	SEC13 homolog, nuclear pore and COPII coat complex component [Source:HGNC Symbol;Acc:HGNC:10697]
DEPDC5	DEP domain containing 5 [Source:HGNC Symbol;Acc:HGNC:18423]
NPRL2	NPR2-like, GATOR1 complex subunit [Source:HGNC Symbol;Acc:HGNC:24969]
NPRL3	NPR3 like, GATOR1 complex subunit [Source:HGNC Symbol;Acc:HGNC:14124]
RNF152	ring finger protein 152 [Source:HGNC Symbol;Acc:HGNC:26811]
TELO2	telomere maintenance 2 [Source:HGNC Symbol;Acc:HGNC:29099]
TTI1	TELO2 interacting protein 1 [Source:HGNC Symbol;Acc:HGNC:29029]
CLIP1	CAP-Gly domain containing linker protein 1 [Source:HGNC Symbol;Acc:HGNC:10461]
GRB10	growth factor receptor bound protein 10 [Source:HGNC Symbol;Acc:HGNC:4564]
LPIN1	lipin 1 [Source:HGNC Symbol;Acc:HGNC:13345]
LPIN3	lipin 3 [Source:HGNC Symbol;Acc:HGNC:14451]
LPIN2	lipin 2 [Source:HGNC Symbol;Acc:HGNC:14450]
TBC1D7	TBC1 domain family member 7 [Source:HGNC Symbol;Acc:HGNC:21066]
MAPKAP1	mitogen-activated protein kinase associated protein 1 [Source:HGNC Symbol;Acc:HGNC:18752]
RICTOR	RPTOR independent companion of MTOR complex 2 [Source:HGNC Symbol;Acc:HGNC:28611]
PRR5	proline rich 5 [Source:HGNC Symbol;Acc:HGNC:31682]
GATSL3	GATS protein-like 3 [Source:HGNC Symbol;Acc:HGNC:34423]
GATSL2	GATS protein-like 2 [Source:HGNC Symbol;Acc:HGNC:37073]
CHEK2	checkpoint kinase 2 [Source:HGNC Symbol;Acc:HGNC:16627]
ATR	ATR serine/threonine kinase [Source:HGNC Symbol;Acc:HGNC:882]
CHEK1	checkpoint kinase 1 [Source:HGNC Symbol;Acc:HGNC:1925]
GORAB	golgin, RAB6 interacting [Source:HGNC Symbol;Acc:HGNC:25676]
MDM4	MDM4, p53 regulator [Source:HGNC Symbol;Acc:HGNC:6974]
SFN	stratifin [Source:HGNC Symbol;Acc:HGNC:10773]
RPRM	reprimo, TP53 dependent G2 arrest mediator candidate [Source:HGNC Symbol;Acc:HGNC:24201]
GTSE1	G2 and S-phase expressed 1 [Source:HGNC Symbol;Acc:HGNC:13698]
PMAIP1	phorbol-12-myristate-13-acetate-induced protein 1 [Source:HGNC Symbol;Acc:HGNC:9108]
TP53AIP1	tumor protein p53 regulated apoptosis inducing protein 1 [Source:HGNC Symbol;Acc:HGNC:29984]

SIVA1	SIVA1 apoptosis inducing factor [Source:HGNC Symbol;Acc:HGNC:17712]
TP53I3	tumor protein p53 inducible protein 3 [Source:HGNC Symbol;Acc:HGNC:19373]
SHISA5	shisa family member 5 [Source:HGNC Symbol;Acc:HGNC:30376]
PERP	PERP, TP53 apoptosis effector [Source:HGNC Symbol;Acc:HGNC:17637]
ZMAT3	zinc finger matrin-type 3 [Source:HGNC Symbol;Acc:HGNC:29983]
AIFM2	apoptosis inducing factor, mitochondria associated 2 [Source:HGNC Symbol;Acc:HGNC:21411]
IGFBP3	insulin like growth factor binding protein 3 [Source:HGNC Symbol;Acc:HGNC:5472]
ADGRB1	adhesion G protein-coupled receptor B1 [Source:HGNC Symbol;Acc:HGNC:943]
CD82	CD82 molecule [Source:HGNC Symbol;Acc:HGNC:6210]
SERPINB5	serpin family B member 5 [Source:HGNC Symbol;Acc:HGNC:8949]
DDB2	damage specific DNA binding protein 2 [Source:HGNC Symbol;Acc:HGNC:2718]
RRM2B	ribonucleotide reductase regulatory TP53 inducible subunit M2B [Source:HGNC Symbol;Acc:HGNC:17296]
RRM2	ribonucleotide reductase regulatory subunit M2 [Source:HGNC Symbol;Acc:HGNC:10452]
STEAP3	STEAP3 metalloreductase [Source:HGNC Symbol;Acc:HGNC:24592]
RCHY1	ring finger and CHY zinc finger domain containing 1 [Source:HGNC Symbol;Acc:HGNC:17479]
CCNG1	cyclin G1 [Source:HGNC Symbol;Acc:HGNC:1592]
PPM1D	protein phosphatase, Mg2+/Mn2+ dependent 1D [Source:HGNC Symbol;Acc:HGNC:9277]
RFWD2	ring finger and WD repeat domain 2 [Source:HGNC Symbol;Acc:HGNC:17440]
BAK1	BCL2 antagonist/killer 1 [Source:HGNC Symbol;Acc:HGNC:949]
DIABLO	diablo IAP-binding mitochondrial protein [Source:HGNC Symbol;Acc:HGNC:21528]
HTRA2	HtrA serine peptidase 2 [Source:HGNC Symbol;Acc:HGNC:14348]
PRF1	perforin 1 [Source:HGNC Symbol;Acc:HGNC:9360]
GZMB	granzyme B [Source:HGNC Symbol;Acc:HGNC:4709]
SPTAN1	spectrin alpha, non-erythrocytic 1 [Source:HGNC Symbol;Acc:HGNC:11273]
LMNA	lamin A/C [Source:HGNC Symbol;Acc:HGNC:6636]
LMNB1	lamin B1 [Source:HGNC Symbol;Acc:HGNC:6637]
LMNB2	lamin B2 [Source:HGNC Symbol;Acc:HGNC:6638]
PARP2	poly(ADP-ribose) polymerase 2 [Source:HGNC Symbol;Acc:HGNC:272]
PARP3	poly(ADP-ribose) polymerase family member 3 [Source:HGNC Symbol;Acc:HGNC:273]
PARP4	poly(ADP-ribose) polymerase family member 4 [Source:HGNC Symbol;Acc:HGNC:271]
DFFA	DNA fragmentation factor subunit alpha [Source:HGNC Symbol;Acc:HGNC:2772]
DFFB	DNA fragmentation factor subunit beta [Source:HGNC Symbol;Acc:HGNC:2773]
ENDOG	endonuclease G [Source:HGNC Symbol;Acc:HGNC:3346]
AIFM1	apoptosis inducing factor, mitochondria associated 1 [Source:HGNC Symbol;Acc:HGNC:8768]
CAPN1	calpain 1 [Source:HGNC Symbol;Acc:HGNC:1476]
CTSC	cathepsin C [Source:HGNC Symbol;Acc:HGNC:2528]
CTSF	cathepsin F [Source:HGNC Symbol;Acc:HGNC:2531]
CTSH	cathepsin H [Source:HGNC Symbol;Acc:HGNC:2535]
CTSK	cathepsin K [Source:HGNC Symbol;Acc:HGNC:2536]
CTSO	cathepsin O [Source:HGNC Symbol;Acc:HGNC:2542]
CTSS	cathepsin S [Source:HGNC Symbol;Acc:HGNC:2545]

CTSV	cathepsin V [Source:HGNC Symbol;Acc:HGNC:2538]
CTSW	cathepsin W [Source:HGNC Symbol;Acc:HGNC:2546]
CTSZ	cathepsin Z [Source:HGNC Symbol;Acc:HGNC:2547]
HRK	harakiri, BCL2 interacting protein [Source:HGNC Symbol;Acc:HGNC:5185]
PTPN13	protein tyrosine phosphatase, non-receptor type 13 [Source:HGNC Symbol;Acc:HGNC:9646]
CASP2	caspase 2 [Source:HGNC Symbol;Acc:HGNC:1503]
C17orf47	chromosome 17 open reading frame 47 [Source:HGNC Symbol;Acc:HGNC:26844]
UBA1	ubiquitin like modifier activating enzyme 1 [Source:HGNC Symbol;Acc:HGNC:12469]
SAE1	SUMO1 activating enzyme subunit 1 [Source:HGNC Symbol;Acc:HGNC:30660]
UBA2	ubiquitin like modifier activating enzyme 2 [Source:HGNC Symbol;Acc:HGNC:30661]
UBA3	ubiquitin like modifier activating enzyme 3 [Source:HGNC Symbol;Acc:HGNC:12470]
UBA7	ubiquitin like modifier activating enzyme 7 [Source:HGNC Symbol;Acc:HGNC:12471]
UBA6	ubiquitin like modifier activating enzyme 6 [Source:HGNC Symbol;Acc:HGNC:25581]
UBE2A	ubiquitin conjugating enzyme E2 A [Source:HGNC Symbol;Acc:HGNC:12472]
UBE2B	ubiquitin conjugating enzyme E2 B [Source:HGNC Symbol;Acc:HGNC:12473]
UBE2C	ubiquitin conjugating enzyme E2 C [Source:HGNC Symbol;Acc:HGNC:15937]
UBE2D4	ubiquitin conjugating enzyme E2 D4 (putative) [Source:HGNC Symbol;Acc:HGNC:21647]
UBE2D1	ubiquitin conjugating enzyme E2 D1 [Source:HGNC Symbol;Acc:HGNC:12474]
UBE2D2	ubiquitin conjugating enzyme E2 D2 [Source:HGNC Symbol;Acc:HGNC:12475]
UBE2D3	ubiquitin conjugating enzyme E2 D3 [Source:HGNC Symbol;Acc:HGNC:12476]
UBE2E3	ubiquitin conjugating enzyme E2 E3 [Source:HGNC Symbol;Acc:HGNC:12479]
UBE2E1	ubiquitin conjugating enzyme E2 E1 [Source:HGNC Symbol;Acc:HGNC:12477]
UBE2E2	ubiquitin conjugating enzyme E2 E2 [Source:HGNC Symbol;Acc:HGNC:12478]
UBE2F	ubiquitin conjugating enzyme E2 F (putative) [Source:HGNC Symbol;Acc:HGNC:12480]
UBE2G1	ubiquitin conjugating enzyme E2 G1 [Source:HGNC Symbol;Acc:HGNC:12482]
UBE2G2	ubiquitin conjugating enzyme E2 G2 [Source:HGNC Symbol;Acc:HGNC:12483]
UBE2H	ubiquitin conjugating enzyme E2 H [Source:HGNC Symbol;Acc:HGNC:12484]
UBE2J1	ubiquitin conjugating enzyme E2 J1 [Source:HGNC Symbol;Acc:HGNC:17598]
UBE2J2	ubiquitin conjugating enzyme E2 J2 [Source:HGNC Symbol;Acc:HGNC:19268]
UBE2L3	ubiquitin conjugating enzyme E2 L3 [Source:HGNC Symbol;Acc:HGNC:12488]
UBE2L6	ubiquitin conjugating enzyme E2 L6 [Source:HGNC Symbol;Acc:HGNC:12490]
UBE2M	ubiquitin conjugating enzyme E2 M [Source:HGNC Symbol;Acc:HGNC:12491]
UBE2N	ubiquitin conjugating enzyme E2 N [Source:HGNC Symbol;Acc:HGNC:12492]
UBE2O	ubiquitin conjugating enzyme E2 O [Source:HGNC Symbol;Acc:HGNC:29554]
UBE2Q1	ubiquitin conjugating enzyme E2 Q1 [Source:HGNC Symbol;Acc:HGNC:15698]
UBE2Q2	ubiquitin conjugating enzyme E2 Q2 [Source:HGNC Symbol;Acc:HGNC:19248]
UBE2QL1	ubiquitin conjugating enzyme E2 Q family like 1 [Source:HGNC Symbol;Acc:HGNC:37269]
UBE2R2	ubiquitin conjugating enzyme E2 R2 [Source:HGNC Symbol;Acc:HGNC:19907]
CDC34	cell division cycle 34 [Source:HGNC Symbol;Acc:HGNC:1734]
UBE2S	ubiquitin conjugating enzyme E2 S [Source:HGNC Symbol;Acc:HGNC:17895]
UBE2U	ubiquitin conjugating enzyme E2 U (putative) [Source:HGNC Symbol;Acc:HGNC:28559]

UBE2W	ubiquitin conjugating enzyme E2 W (putative) [Source:HGNC Symbol;Acc:HGNC:25616]
UBE2Z	ubiquitin conjugating enzyme E2 Z [Source:HGNC Symbol;Acc:HGNC:25847]
UBE2K	ubiquitin conjugating enzyme E2 K [Source:HGNC Symbol;Acc:HGNC:4914]
BIRC6	baculoviral IAP repeat containing 6 [Source:HGNC Symbol;Acc:HGNC:13516]
UBE3A	ubiquitin protein ligase E3A [Source:HGNC Symbol;Acc:HGNC:12496]
UBE3B	ubiquitin protein ligase E3B [Source:HGNC Symbol;Acc:HGNC:13478]
UBE3C	ubiquitin protein ligase E3C [Source:HGNC Symbol;Acc:HGNC:16803]
WWP1	WW domain containing E3 ubiquitin protein ligase 1 [Source:HGNC Symbol;Acc:HGNC:17004]
WWP2	WW domain containing E3 ubiquitin protein ligase 2 [Source:HGNC Symbol;Acc:HGNC:16804]
TRIP12	thyroid hormone receptor interactor 12 [Source:HGNC Symbol;Acc:HGNC:12306]
HUWE1	HECT, UBA and WWE domain containing 1, E3 ubiquitin protein ligase [Source:HGNC Symbol;Acc:HGNC:30892]
UBR5	ubiquitin protein ligase E3 component n-recognin 5 [Source:HGNC Symbol;Acc:HGNC:16806]
HERC1	HECT and RLD domain containing E3 ubiquitin protein ligase family member 1 [Source:HGNC Symbol;Acc:HGNC:4867]
HERC2	HECT and RLD domain containing E3 ubiquitin protein ligase 2 [Source:HGNC Symbol;Acc:HGNC:4868]
HERC3	HECT and RLD domain containing E3 ubiquitin protein ligase 3 [Source:HGNC Symbol;Acc:HGNC:4876]
HERC4	HECT and RLD domain containing E3 ubiquitin protein ligase 4 [Source:HGNC Symbol;Acc:HGNC:24521]
UBE4A	ubiquitination factor E4A [Source:HGNC Symbol;Acc:HGNC:12499]
UBE4B	ubiquitination factor E4B [Source:HGNC Symbol;Acc:HGNC:12500]
STUB1	STIP1 homology and U-box containing protein 1 [Source:HGNC Symbol;Acc:HGNC:11427]
PPIL2	peptidylprolyl isomerase like 2 [Source:HGNC Symbol;Acc:HGNC:9261]
PRPF19	pre-mRNA processing factor 19 [Source:HGNC Symbol;Acc:HGNC:17896]
UBOX5	U-box domain containing 5 [Source:HGNC Symbol;Acc:HGNC:17777]
CBL	Cbl proto-oncogene [Source:HGNC Symbol;Acc:HGNC:1541]
CBLB	Cbl proto-oncogene B [Source:HGNC Symbol;Acc:HGNC:1542]
CBLC	Cbl proto-oncogene C [Source:HGNC Symbol;Acc:HGNC:15961]
PML	promyelocytic leukemia [Source:HGNC Symbol;Acc:HGNC:9113]
BIRC7	baculoviral IAP repeat containing 7 [Source:HGNC Symbol;Acc:HGNC:13702]
SYVN1	synoviolin 1 [Source:HGNC Symbol;Acc:HGNC:20738]
NHLRC1	NHL repeat containing E3 ubiquitin protein ligase 1 [Source:HGNC Symbol;Acc:HGNC:21576]
AIRE	autoimmune regulator [Source:HGNC Symbol;Acc:HGNC:360]
FANCL	Fanconi anemia complementation group L [Source:HGNC Symbol;Acc:HGNC:20748]
MID1	midline 1 [Source:HGNC Symbol;Acc:HGNC:7095]
TRIM32	tripartite motif containing 32 [Source:HGNC Symbol;Acc:HGNC:16380]
TRIM37	tripartite motif containing 37 [Source:HGNC Symbol;Acc:HGNC:7523]
FBXW7	F-box and WD repeat domain containing 7 [Source:HGNC Symbol;Acc:HGNC:16712]
FBXO2	F-box protein 2 [Source:HGNC Symbol;Acc:HGNC:13581]
FBXO4	F-box protein 4 [Source:HGNC Symbol;Acc:HGNC:13583]
KLHL9	kelch like family member 9 [Source:HGNC Symbol;Acc:HGNC:18732]

KLHL13	kelch like family member 13 [Source:HGNC Symbol;Acc:HGNC:22931]
RHOBTB2	Rho related BTB domain containing 2 [Source:HGNC Symbol;Acc:HGNC:18756]
RHOBTB1	Rho related BTB domain containing 1 [Source:HGNC Symbol;Acc:HGNC:18738]
CUL4B	cullin 4B [Source:HGNC Symbol;Acc:HGNC:2555]
CUL4A	cullin 4A [Source:HGNC Symbol;Acc:HGNC:2554]
DDB1	damage specific DNA binding protein 1 [Source:HGNC Symbol;Acc:HGNC:2717]
ERCC8	ERCC excision repair 8, CSA ubiquitin ligase complex subunit [Source:HGNC Symbol;Acc:HGNC:3439]
DET1	de-etiolated homolog 1 (Arabidopsis) [Source:HGNC Symbol;Acc:HGNC:25477]
RNF7	ring finger protein 7 [Source:HGNC Symbol;Acc:HGNC:10070]
CUL5	cullin 5 [Source:HGNC Symbol;Acc:HGNC:2556]
CUL7	cullin 7 [Source:HGNC Symbol;Acc:HGNC:21024]
FBXW8	F-box and WD repeat domain containing 8 [Source:HGNC Symbol;Acc:HGNC:13597]
ANAPC11	anaphase promoting complex subunit 11 [Source:HGNC Symbol;Acc:HGNC:14452]
ANAPC2	anaphase promoting complex subunit 2 [Source:HGNC Symbol;Acc:HGNC:19989]
CDC20	cell division cycle 20 [Source:HGNC Symbol;Acc:HGNC:1723]
FZR1	fizzy/cell division cycle 20 related 1 [Source:HGNC Symbol;Acc:HGNC:24824]
ANAPC1	anaphase promoting complex subunit 1 [Source:HGNC Symbol;Acc:HGNC:19988]
CDC27	cell division cycle 27 [Source:HGNC Symbol;Acc:HGNC:1728]
ANAPC4	anaphase promoting complex subunit 4 [Source:HGNC Symbol;Acc:HGNC:19990]
ANAPC5	anaphase promoting complex subunit 5 [Source:HGNC Symbol;Acc:HGNC:15713]
CDC16	cell division cycle 16 [Source:HGNC Symbol;Acc:HGNC:1720]
ANAPC7	anaphase promoting complex subunit 7 [Source:HGNC Symbol;Acc:HGNC:17380]
CDC23	cell division cycle 23 [Source:HGNC Symbol;Acc:HGNC:1724]
ANAPC10	anaphase promoting complex subunit 10 [Source:HGNC Symbol;Acc:HGNC:24077]
CDC26	cell division cycle 26 [Source:HGNC Symbol;Acc:HGNC:17839]
ANAPC13	anaphase promoting complex subunit 13 [Source:HGNC Symbol;Acc:HGNC:24540]
ANAPC15	anaphase promoting complex subunit 15 [Source:HGNC Symbol;Acc:HGNC:24531]
ANAPC16	anaphase promoting complex subunit 16 [Source:HGNC Symbol;Acc:HGNC:26976]
TCEB1	transcription elongation factor B subunit 1 [Source:HGNC Symbol;Acc:HGNC:11617]
E2F3	E2F transcription factor 3 [Source:HGNC Symbol;Acc:HGNC:3115]
TFDP2	transcription factor Dp-2 [Source:HGNC Symbol;Acc:HGNC:11751]
ZBTB17	zinc finger and BTB domain containing 17 [Source:HGNC Symbol;Acc:HGNC:12936]
CDKN2C	cyclin dependent kinase inhibitor 2C [Source:HGNC Symbol;Acc:HGNC:1789]
CDC6	cell division cycle 6 [Source:HGNC Symbol;Acc:HGNC:1744]
CDC45	cell division cycle 45 [Source:HGNC Symbol;Acc:HGNC:1739]
CDC7	cell division cycle 7 [Source:HGNC Symbol;Acc:HGNC:1745]
DBF4	DBF4 zinc finger [Source:HGNC Symbol;Acc:HGNC:17364]
CDC25C	cell division cycle 25C [Source:HGNC Symbol;Acc:HGNC:1727]
WEE1	WEE1 G2 checkpoint kinase [Source:HGNC Symbol;Acc:HGNC:12761]
WEE2	WEE1 homolog 2 (S. pombe) [Source:HGNC Symbol;Acc:HGNC:19684]

PKMYT1	protein kinase, membrane associated tyrosine/threonine 1 [Source:HGNC Symbol;Acc:HGNC:29650]
CCNH	cyclin H [Source:HGNC Symbol;Acc:HGNC:1594]
CDK7	cyclin dependent kinase 7 [Source:HGNC Symbol;Acc:HGNC:1778]
PTTG1	pituitary tumor-transforming 1 [Source:HGNC Symbol;Acc:HGNC:9690]
PTTG2	pituitary tumor-transforming 2 [Source:HGNC Symbol;Acc:HGNC:9691]
ESPL1	extra spindle pole bodies like 1, separase [Source:HGNC Symbol;Acc:HGNC:16856]
SMC1A	structural maintenance of chromosomes 1A [Source:HGNC Symbol;Acc:HGNC:11111]
SMC1B	structural maintenance of chromosomes 1B [Source:HGNC Symbol;Acc:HGNC:11112]
SMC3	structural maintenance of chromosomes 3 [Source:HGNC Symbol;Acc:HGNC:2468]
STAG2	stromal antigen 2 [Source:HGNC Symbol;Acc:HGNC:11355]
STAG1	stromal antigen 1 [Source:HGNC Symbol;Acc:HGNC:11354]
RAD21	RAD21 cohesin complex component [Source:HGNC Symbol;Acc:HGNC:9811]
TTK	TTK protein kinase [Source:HGNC Symbol;Acc:HGNC:12401]
BUB1	BUB1 mitotic checkpoint serine/threonine kinase [Source:HGNC Symbol;Acc:HGNC:1148]
BUB3	BUB3, mitotic checkpoint protein [Source:HGNC Symbol;Acc:HGNC:1151]
BUB1B	BUB1 mitotic checkpoint serine/threonine kinase B [Source:HGNC Symbol;Acc:HGNC:1149]
MAD1L1	MAD1 mitotic arrest deficient like 1 [Source:HGNC Symbol;Acc:HGNC:6762]
MAD2L1	MAD2 mitotic arrest deficient-like 1 (yeast) [Source:HGNC Symbol;Acc:HGNC:6763]
MAD2L2	MAD2 mitotic arrest deficient-like 2 (yeast) [Source:HGNC Symbol;Acc:HGNC:6764]
CDC14B	cell division cycle 14B [Source:HGNC Symbol;Acc:HGNC:1719]
CDC14A	cell division cycle 14A [Source:HGNC Symbol;Acc:HGNC:1718]
PRKDC	protein kinase, DNA-activated, catalytic polypeptide [Source:HGNC Symbol;Acc:HGNC:9413]
CDC25A	cell division cycle 25A [Source:HGNC Symbol;Acc:HGNC:1725]
ORC1	origin recognition complex subunit 1 [Source:HGNC Symbol;Acc:HGNC:8487]
ORC2	origin recognition complex subunit 2 [Source:HGNC Symbol;Acc:HGNC:8488]
ORC3	origin recognition complex subunit 3 [Source:HGNC Symbol;Acc:HGNC:8489]
ORC4	origin recognition complex subunit 4 [Source:HGNC Symbol;Acc:HGNC:8490]
ORC5	origin recognition complex subunit 5 [Source:HGNC Symbol;Acc:HGNC:8491]
ORC6	origin recognition complex subunit 6 [Source:HGNC Symbol;Acc:HGNC:17151]
MCM2	minichromosome maintenance complex component 2 [Source:HGNC Symbol;Acc:HGNC:6944]
MCM3	minichromosome maintenance complex component 3 [Source:HGNC Symbol;Acc:HGNC:6945]
MCM4	minichromosome maintenance complex component 4 [Source:HGNC Symbol;Acc:HGNC:6947]
MCM5	minichromosome maintenance complex component 5 [Source:HGNC Symbol;Acc:HGNC:6948]
MCM6	minichromosome maintenance complex component 6 [Source:HGNC Symbol;Acc:HGNC:6949]
MCM7	minichromosome maintenance complex component 7 [Source:HGNC Symbol;Acc:HGNC:6950]
ATP2B1	ATPase plasma membrane Ca2+ transporting 1 [Source:HGNC Symbol;Acc:HGNC:814]
ATP2B3	ATPase plasma membrane Ca2+ transporting 3 [Source:HGNC Symbol;Acc:HGNC:816]
ATP2B4	ATPase plasma membrane Ca2+ transporting 4 [Source:HGNC Symbol;Acc:HGNC:817]
ATP2B2	ATPase plasma membrane Ca2+ transporting 2 [Source:HGNC Symbol;Acc:HGNC:815]
ADRB2	adrenoceptor beta 2 [Source:HGNC Symbol;Acc:HGNC:286]
ADRB3	adrenoceptor beta 3 [Source:HGNC Symbol;Acc:HGNC:288]

DRD5	dopamine receptor D5 [Source:HGNC Symbol;Acc:HGNC:3026]
HRH2	histamine receptor H2 [Source:HGNC Symbol;Acc:HGNC:5183]
HTR4	5-hydroxytryptamine receptor 4 [Source:HGNC Symbol;Acc:HGNC:5299]
HTR5A	5-hydroxytryptamine receptor 5A [Source:HGNC Symbol;Acc:HGNC:5300]
HTR6	5-hydroxytryptamine receptor 6 [Source:HGNC Symbol;Acc:HGNC:5301]
GNAL	G protein subunit alpha L [Source:HGNC Symbol;Acc:HGNC:4388]
PLN	phospholamban [Source:HGNC Symbol;Acc:HGNC:9080]
ATP2A1	ATPase sarcoplasmic/endoplasmic reticulum Ca2+ transporting 1 [Source:HGNC Symbol;Acc:HGNC:811]
ATP2A3	ATPase sarcoplasmic/endoplasmic reticulum Ca2+ transporting 3 [Source:HGNC Symbol;Acc:HGNC:813]
HRC	histidine rich calcium binding protein [Source:HGNC Symbol;Acc:HGNC:5178]
STIM1	stromal interaction molecule 1 [Source:HGNC Symbol;Acc:HGNC:11386]
STIM2	stromal interaction molecule 2 [Source:HGNC Symbol;Acc:HGNC:19205]
ORAI2	ORAI calcium release-activated calcium modulator 2 [Source:HGNC Symbol;Acc:HGNC:21667]
ORAI3	ORAI calcium release-activated calcium modulator 3 [Source:HGNC Symbol;Acc:HGNC:28185]
CHRNA7	cholinergic receptor nicotinic alpha 7 subunit [Source:HGNC Symbol;Acc:HGNC:1960]
P2RX1	purinergic receptor P2X 1 [Source:HGNC Symbol;Acc:HGNC:8533]
P2RX2	purinergic receptor P2X 2 [Source:HGNC Symbol;Acc:HGNC:15459]
P2RX3	purinergic receptor P2X 3 [Source:HGNC Symbol;Acc:HGNC:8534]
P2RX4	purinergic receptor P2X 4 [Source:HGNC Symbol;Acc:HGNC:8535]
P2RX5	purinergic receptor P2X 5 [Source:HGNC Symbol;Acc:HGNC:8536]
P2RX6	purinergic receptor P2X 6 [Source:HGNC Symbol;Acc:HGNC:8538]
GRIN2C	glutamate ionotropic receptor NMDA type subunit 2C [Source:HGNC Symbol;Acc:HGNC:4587]
GRIN2D	glutamate ionotropic receptor NMDA type subunit 2D [Source:HGNC Symbol;Acc:HGNC:4588]
TRDN	triadin [Source:HGNC Symbol;Acc:HGNC:12261]
CASQ1	calsequestrin 1 [Source:HGNC Symbol;Acc:HGNC:1512]
CASQ2	calsequestrin 2 [Source:HGNC Symbol;Acc:HGNC:1513]
ASPH	aspartate beta-hydroxylase [Source:HGNC Symbol;Acc:HGNC:757]
CYSLTR1	cysteinyl leukotriene receptor 1 [Source:HGNC Symbol;Acc:HGNC:17451]
CYSLTR2	cysteinyl leukotriene receptor 2 [Source:HGNC Symbol;Acc:HGNC:18274]
ADRA1B	adrenoceptor alpha 1B [Source:HGNC Symbol;Acc:HGNC:278]
ADRA1D	adrenoceptor alpha 1D [Source:HGNC Symbol;Acc:HGNC:280]
EDNRA	endothelin receptor type A [Source:HGNC Symbol;Acc:HGNC:3179]
EDNRB	endothelin receptor type B [Source:HGNC Symbol;Acc:HGNC:3180]
HRH1	histamine receptor H1 [Source:HGNC Symbol;Acc:HGNC:5182]
NTSR1	neurotensin receptor 1 (high affinity) [Source:HGNC Symbol;Acc:HGNC:8039]
OXTR	oxytocin receptor [Source:HGNC Symbol;Acc:HGNC:8529]
AVPR1A	arginine vasopressin receptor 1A [Source:HGNC Symbol;Acc:HGNC:895]
AVPR1B	arginine vasopressin receptor 1B [Source:HGNC Symbol;Acc:HGNC:896]
LTB4R2	leukotriene B4 receptor 2 [Source:HGNC Symbol;Acc:HGNC:19260]
PTAFR	platelet activating factor receptor [Source:HGNC Symbol;Acc:HGNC:9582]

PTGER1	prostaglandin E receptor 1 [Source:HGNC Symbol;Acc:HGNC:9593]
PTGER3	prostaglandin E receptor 3 [Source:HGNC Symbol;Acc:HGNC:9595]
PTGFR	prostaglandin F receptor [Source:HGNC Symbol;Acc:HGNC:9600]
TACR1	tachykinin receptor 1 [Source:HGNC Symbol;Acc:HGNC:11526]
TACR2	tachykinin receptor 2 [Source:HGNC Symbol;Acc:HGNC:11527]
TACR3	tachykinin receptor 3 [Source:HGNC Symbol;Acc:HGNC:11528]
TBXA2R	thromboxane A2 receptor [Source:HGNC Symbol;Acc:HGNC:11608]
TRHR	thyrotropin releasing hormone receptor [Source:HGNC Symbol;Acc:HGNC:12299]
CCKAR	cholecystokinin A receptor [Source:HGNC Symbol;Acc:HGNC:1570]
CCKBR	cholecystokinin B receptor [Source:HGNC Symbol;Acc:HGNC:1571]
GNA14	G protein subunit alpha 14 [Source:HGNC Symbol;Acc:HGNC:4382]
GNA15	G protein subunit alpha 15 [Source:HGNC Symbol;Acc:HGNC:4383]
GDNF	glial cell derived neurotrophic factor [Source:HGNC Symbol;Acc:HGNC:4232]
MST1R	macrophage stimulating 1 receptor [Source:HGNC Symbol;Acc:HGNC:7381]
CD38	CD38 molecule [Source:HGNC Symbol;Acc:HGNC:1667]
TPCN1	two pore segment channel 1 [Source:HGNC Symbol;Acc:HGNC:18182]
TPCN2	two pore segment channel 2 [Source:HGNC Symbol;Acc:HGNC:20820]
MCOLN1	mucolipin 1 [Source:HGNC Symbol;Acc:HGNC:13356]
MCOLN2	mucolipin 2 [Source:HGNC Symbol;Acc:HGNC:13357]
MCOLN3	mucolipin 3 [Source:HGNC Symbol;Acc:HGNC:13358]
MCU	mitochondrial calcium uniporter [Source:HGNC Symbol;Acc:HGNC:23526]
SLC25A4	solute carrier family 25 member 4 [Source:HGNC Symbol;Acc:HGNC:10990]
SLC25A5	solute carrier family 25 member 5 [Source:HGNC Symbol;Acc:HGNC:10991]
SLC25A6	solute carrier family 25 member 6 [Source:HGNC Symbol;Acc:HGNC:10992]
SLC25A31	solute carrier family 25 member 31 [Source:HGNC Symbol;Acc:HGNC:25319]
TNNC1	troponin C1, slow skeletal and cardiac type [Source:HGNC Symbol;Acc:HGNC:11943]
TNNC2	troponin C2, fast skeletal type [Source:HGNC Symbol;Acc:HGNC:11944]
PHKG1	phosphorylase kinase catalytic subunit gamma 1 [Source:HGNC Symbol;Acc:HGNC:8930]
PHKG2	phosphorylase kinase catalytic subunit gamma 2 [Source:HGNC Symbol;Acc:HGNC:8931]
PHKB	phosphorylase kinase regulatory subunit beta [Source:HGNC Symbol;Acc:HGNC:8927]
PHKA2	phosphorylase kinase regulatory subunit alpha 2 [Source:HGNC Symbol;Acc:HGNC:8926]
PHKA1	phosphorylase kinase regulatory subunit alpha 1 [Source:HGNC Symbol;Acc:HGNC:8925]
CAMK1D	calcium/calmodulin dependent protein kinase ID [Source:HGNC Symbol;Acc:HGNC:19341]
CAMK1G	calcium/calmodulin dependent protein kinase IG [Source:HGNC Symbol;Acc:HGNC:14585]
PDE1A	phosphodiesterase 1A [Source:HGNC Symbol;Acc:HGNC:8774]
PDE1B	phosphodiesterase 1B [Source:HGNC Symbol;Acc:HGNC:8775]
PDE1C	phosphodiesterase 1C [Source:HGNC Symbol;Acc:HGNC:8776]
PTK2B	protein tyrosine kinase 2 beta [Source:HGNC Symbol;Acc:HGNC:9612]
CD3D	CD3d molecule [Source:HGNC Symbol;Acc:HGNC:1673]
CD3E	CD3e molecule [Source:HGNC Symbol;Acc:HGNC:1674]
CD3G	CD3g molecule [Source:HGNC Symbol;Acc:HGNC:1675]

CD247	CD247 molecule [Source:HGNC Symbol;Acc:HGNC:1677]
ITK	IL2 inducible T-cell kinase [Source:HGNC Symbol;Acc:HGNC:6171]
TEC	tec protein tyrosine kinase [Source:HGNC Symbol;Acc:HGNC:11719]
NCK1	NCK adaptor protein 1 [Source:HGNC Symbol;Acc:HGNC:7664]
NCK2	NCK adaptor protein 2 [Source:HGNC Symbol;Acc:HGNC:7665]
GRAP2	GRB2-related adaptor protein 2 [Source:HGNC Symbol;Acc:HGNC:4563]
NFKBIB	NFKB inhibitor beta [Source:HGNC Symbol;Acc:HGNC:7798]
NFKBIE	NFKB inhibitor epsilon [Source:HGNC Symbol;Acc:HGNC:7799]
GUCY1B3	guanylate cyclase 1, soluble, beta 3 [Source:HGNC Symbol;Acc:HGNC:4687]
PTGS1	prostaglandin-endoperoxide synthase 1 [Source:HGNC Symbol;Acc:HGNC:9604]
PTGES	prostaglandin E synthase [Source:HGNC Symbol;Acc:HGNC:9599]
PTGES2	prostaglandin E synthase 2 [Source:HGNC Symbol;Acc:HGNC:17822]
PTGES3	prostaglandin E synthase 3 [Source:HGNC Symbol;Acc:HGNC:16049]
CBR1	carbonyl reductase 1 [Source:HGNC Symbol;Acc:HGNC:1548]
CBR3	carbonyl reductase 3 [Source:HGNC Symbol;Acc:HGNC:1549]
TBXAS1	thromboxane A synthase 1 [Source:HGNC Symbol;Acc:HGNC:11609]
PTGDS	prostaglandin D2 synthase [Source:HGNC Symbol;Acc:HGNC:9592]
HPGDS	hematopoietic prostaglandin D synthase [Source:HGNC Symbol;Acc:HGNC:17890]
PTGIS	prostaglandin I2 (prostacyclin) synthase [Source:HGNC Symbol;Acc:HGNC:9603]
LTA4H	leukotriene A4 hydrolase [Source:HGNC Symbol;Acc:HGNC:6710]
CYP4F2	cytochrome P450 family 4 subfamily F member 2 [Source:HGNC Symbol;Acc:HGNC:2645]
CYP4F3	cytochrome P450 family 4 subfamily F member 3 [Source:HGNC Symbol;Acc:HGNC:2646]
LTC4S	leukotriene C4 synthase [Source:HGNC Symbol;Acc:HGNC:6719]
GGT1	gamma-glutamyltransferase 1 [Source:HGNC Symbol;Acc:HGNC:4250]
GGT5	gamma-glutamyltransferase 5 [Source:HGNC Symbol;Acc:HGNC:4260]
GPX6	glutathione peroxidase 6 [Source:HGNC Symbol;Acc:HGNC:4558]
GPX7	glutathione peroxidase 7 [Source:HGNC Symbol;Acc:HGNC:4559]
GPX2	glutathione peroxidase 2 [Source:HGNC Symbol;Acc:HGNC:4554]
GPX1	glutathione peroxidase 1 [Source:HGNC Symbol;Acc:HGNC:4553]
GPX5	glutathione peroxidase 5 [Source:HGNC Symbol;Acc:HGNC:4557]
GPX8	glutathione peroxidase 8 (putative) [Source:HGNC Symbol;Acc:HGNC:33100]
CYP4F8	cytochrome P450 family 4 subfamily F member 8 [Source:HGNC Symbol;Acc:HGNC:2648]
ALOX12	arachidonate 12-lipoxygenase, 12S type [Source:HGNC Symbol;Acc:HGNC:429]
ALOX12B	arachidonate 12-lipoxygenase, 12R type [Source:HGNC Symbol;Acc:HGNC:430]
ALOX15B	arachidonate 15-lipoxygenase, type B [Source:HGNC Symbol;Acc:HGNC:434]
EPHX2	epoxide hydrolase 2 [Source:HGNC Symbol;Acc:HGNC:3402]
ALOX15	arachidonate 15-lipoxygenase [Source:HGNC Symbol;Acc:HGNC:433]
FAM213B	family with sequence similarity 213 member B [Source:HGNC Symbol;Acc:HGNC:28390]
TSHB	thyroid stimulating hormone beta [Source:HGNC Symbol;Acc:HGNC:12372]
TSHR	thyroid stimulating hormone receptor [Source:HGNC Symbol;Acc:HGNC:12373]
PNPLA2	patatin like phospholipase domain containing 2 [Source:HGNC Symbol;Acc:HGNC:30802]

ABHD5	abhydrolase domain containing 5 [Source:HGNC Symbol;Acc:HGNC:21396]
MGLL	monoglyceride lipase [Source:HGNC Symbol;Acc:HGNC:17038]
AQP7	aquaporin 7 [Source:HGNC Symbol;Acc:HGNC:640]
ADORA1	adenosine A1 receptor [Source:HGNC Symbol;Acc:HGNC:262]
NPY	neuropeptide Y [Source:HGNC Symbol;Acc:HGNC:7955]
NPY1R	neuropeptide Y receptor Y1 [Source:HGNC Symbol;Acc:HGNC:7956]
CCL1	C-C motif chemokine ligand 1 [Source:HGNC Symbol;Acc:HGNC:10609]
CCL25	C-C motif chemokine ligand 25 [Source:HGNC Symbol;Acc:HGNC:10624]
CCL3L1	C-C motif chemokine ligand 3 like 1 [Source:HGNC Symbol;Acc:HGNC:10628]
CCL3L3	C-C motif chemokine ligand 3 like 3 [Source:HGNC Symbol;Acc:HGNC:30554]
CCL17	C-C motif chemokine ligand 17 [Source:HGNC Symbol;Acc:HGNC:10615]
CCL22	C-C motif chemokine ligand 22 [Source:HGNC Symbol;Acc:HGNC:10621]
CCL8	C-C motif chemokine ligand 8 [Source:HGNC Symbol;Acc:HGNC:10635]
CCL14	C-C motif chemokine ligand 14 [Source:HGNC Symbol;Acc:HGNC:10612]
CCL16	C-C motif chemokine ligand 16 [Source:HGNC Symbol;Acc:HGNC:10614]
CCL15	C-C motif chemokine ligand 15 [Source:HGNC Symbol;Acc:HGNC:10613]
CCL23	C-C motif chemokine ligand 23 [Source:HGNC Symbol;Acc:HGNC:10622]
CCL7	C-C motif chemokine ligand 7 [Source:HGNC Symbol;Acc:HGNC:10634]
CCL11	C-C motif chemokine ligand 11 [Source:HGNC Symbol;Acc:HGNC:10610]
CCL24	C-C motif chemokine ligand 24 [Source:HGNC Symbol;Acc:HGNC:10623]
CCL26	C-C motif chemokine ligand 26 [Source:HGNC Symbol;Acc:HGNC:10625]
CCL27	C-C motif chemokine ligand 27 [Source:HGNC Symbol;Acc:HGNC:10626]
CCL28	C-C motif chemokine ligand 28 [Source:HGNC Symbol;Acc:HGNC:17700]
CCL18	C-C motif chemokine ligand 18 [Source:HGNC Symbol;Acc:HGNC:10616]
PPBP	pro-platelet basic protein [Source:HGNC Symbol;Acc:HGNC:9240]
PF4	platelet factor 4 [Source:HGNC Symbol;Acc:HGNC:8861]
PF4V1	platelet factor 4 variant 1 [Source:HGNC Symbol;Acc:HGNC:8862]
CXCL13	C-X-C motif chemokine ligand 13 [Source:HGNC Symbol;Acc:HGNC:10639]
CXCL16	C-X-C motif chemokine ligand 16 [Source:HGNC Symbol;Acc:HGNC:16642]
CXCL17	C-X-C motif chemokine ligand 17 [Source:HGNC Symbol;Acc:HGNC:19232]
XCL1	X-C motif chemokine ligand 1 [Source:HGNC Symbol;Acc:HGNC:10645]
XCL2	X-C motif chemokine ligand 2 [Source:HGNC Symbol;Acc:HGNC:10646]
IL27	interleukin 27 [Source:HGNC Symbol;Acc:HGNC:19157]
EBI3	Epstein-Barr virus induced 3 [Source:HGNC Symbol;Acc:HGNC:3129]
IL31	interleukin 31 [Source:HGNC Symbol;Acc:HGNC:19372]
CLCF1	cardiotrophin-like cytokine factor 1 [Source:HGNC Symbol;Acc:HGNC:17412]
IL26	interleukin 26 [Source:HGNC Symbol;Acc:HGNC:17119]
IL1RN	interleukin 1 receptor antagonist [Source:HGNC Symbol;Acc:HGNC:6000]
IL36RN	interleukin 36 receptor antagonist [Source:HGNC Symbol;Acc:HGNC:15561]
IL36A	interleukin 36, alpha [Source:HGNC Symbol;Acc:HGNC:15562]
IL36B	interleukin 36, beta [Source:HGNC Symbol;Acc:HGNC:15564]

IL36G	interleukin 36, gamma [Source:HGNC Symbol;Acc:HGNC:15741]
IL1F10	interleukin 1 family member 10 (theta) [Source:HGNC Symbol;Acc:HGNC:15552]
IL37	interleukin 37 [Source:HGNC Symbol;Acc:HGNC:15563]
IL33	interleukin 33 [Source:HGNC Symbol;Acc:HGNC:16028]
IL17F	interleukin 17F [Source:HGNC Symbol;Acc:HGNC:16404]
IL17B	interleukin 17B [Source:HGNC Symbol;Acc:HGNC:5982]
IL17C	interleukin 17C [Source:HGNC Symbol;Acc:HGNC:5983]
IL25	interleukin 25 [Source:HGNC Symbol;Acc:HGNC:13765]
IL16	interleukin 16 [Source:HGNC Symbol;Acc:HGNC:5980]
IL32	interleukin 32 [Source:HGNC Symbol;Acc:HGNC:16830]
IL34	interleukin 34 [Source:HGNC Symbol;Acc:HGNC:28529]
TNFSF15	tumor necrosis factor superfamily member 15 [Source:HGNC Symbol;Acc:HGNC:11931]
TNFSF12	tumor necrosis factor superfamily member 12 [Source:HGNC Symbol;Acc:HGNC:11927]
CD70	CD70 molecule [Source:HGNC Symbol;Acc:HGNC:11937]
TNFSF8	tumor necrosis factor superfamily member 8 [Source:HGNC Symbol;Acc:HGNC:11938]
TNFSF9	tumor necrosis factor superfamily member 9 [Source:HGNC Symbol;Acc:HGNC:11939]
TNFSF18	tumor necrosis factor superfamily member 18 [Source:HGNC Symbol;Acc:HGNC:11932]
TNFSF13	tumor necrosis factor superfamily member 13 [Source:HGNC Symbol;Acc:HGNC:11928]
GDF15	growth differentiation factor 15 [Source:HGNC Symbol;Acc:HGNC:30142]
INHA	inhibin alpha subunit [Source:HGNC Symbol;Acc:HGNC:6065]
GDF10	growth differentiation factor 10 [Source:HGNC Symbol;Acc:HGNC:4215]
GDF11	growth differentiation factor 11 [Source:HGNC Symbol;Acc:HGNC:4216]
MSTN	myostatin [Source:HGNC Symbol;Acc:HGNC:4223]
GDF1	growth differentiation factor 1 [Source:HGNC Symbol;Acc:HGNC:4214]
GDF3	growth differentiation factor 3 [Source:HGNC Symbol;Acc:HGNC:4218]
GDF9	growth differentiation factor 9 [Source:HGNC Symbol;Acc:HGNC:4224]
CCR8	C-C motif chemokine receptor 8 [Source:HGNC Symbol;Acc:HGNC:1609]
CCR9	C-C motif chemokine receptor 9 [Source:HGNC Symbol;Acc:HGNC:1610]
ACKR4	atypical chemokine receptor 4 [Source:HGNC Symbol;Acc:HGNC:1611]
CCR7	C-C motif chemokine receptor 7 [Source:HGNC Symbol;Acc:HGNC:1608]
CCR4	C-C motif chemokine receptor 4 [Source:HGNC Symbol;Acc:HGNC:1605]
CCR5	C-C motif chemokine receptor 5 (gene/pseudogene) [Source:HGNC Symbol;Acc:HGNC:1606]
CCR3	C-C motif chemokine receptor 3 [Source:HGNC Symbol;Acc:HGNC:1604]
CCR2	C-C motif chemokine receptor 2 [Source:HGNC Symbol;Acc:HGNC:1603]
CCR1	C-C motif chemokine receptor 1 [Source:HGNC Symbol;Acc:HGNC:1602]
CCR10	C-C motif chemokine receptor 10 [Source:HGNC Symbol;Acc:HGNC:4474]
CCR6	C-C motif chemokine receptor 6 [Source:HGNC Symbol;Acc:HGNC:1607]
CXCR1	C-X-C motif chemokine receptor 1 [Source:HGNC Symbol;Acc:HGNC:6026]
CXCR2	C-X-C motif chemokine receptor 2 [Source:HGNC Symbol;Acc:HGNC:6027]
CXCR3	C-X-C motif chemokine receptor 3 [Source:HGNC Symbol;Acc:HGNC:4540]
CXCR5	C-X-C motif chemokine receptor 5 [Source:HGNC Symbol;Acc:HGNC:1060]

ACKR3	atypical chemokine receptor 3 [Source:HGNC Symbol;Acc:HGNC:23692]
CXCR6	C-X-C motif chemokine receptor 6 [Source:HGNC Symbol;Acc:HGNC:16647]
XCR1	X-C motif chemokine receptor 1 [Source:HGNC Symbol;Acc:HGNC:1625]
CX3CR1	C-X3-C motif chemokine receptor 1 [Source:HGNC Symbol;Acc:HGNC:2558]
IL31RA	interleukin 31 receptor A [Source:HGNC Symbol;Acc:HGNC:18969]
IL1R2	interleukin 1 receptor type 2 [Source:HGNC Symbol;Acc:HGNC:5994]
IL1RL2	interleukin 1 receptor like 2 [Source:HGNC Symbol;Acc:HGNC:5999]
IL18RAP	interleukin 18 receptor accessory protein [Source:HGNC Symbol;Acc:HGNC:5989]
IL1RL1	interleukin 1 receptor like 1 [Source:HGNC Symbol;Acc:HGNC:5998]
IL17RA	interleukin 17 receptor A [Source:HGNC Symbol;Acc:HGNC:5985]
IL17RC	interleukin 17 receptor C [Source:HGNC Symbol;Acc:HGNC:18358]
IL17RB	interleukin 17 receptor B [Source:HGNC Symbol;Acc:HGNC:18015]
IL17RE	interleukin 17 receptor E [Source:HGNC Symbol;Acc:HGNC:18439]
TNFRSF14	tumor necrosis factor receptor superfamily member 14 [Source:HGNC Symbol;Acc:HGNC:11912]
TNFRSF6B	tumor necrosis factor receptor superfamily member 6b [Source:HGNC Symbol;Acc:HGNC:11921]
TNFRSF25	tumor necrosis factor receptor superfamily member 25 [Source:HGNC Symbol;Acc:HGNC:11910]
TNFRSF10C	tumor necrosis factor receptor superfamily member 10c [Source:HGNC Symbol;Acc:HGNC:11906]
TNFRSF10D	tumor necrosis factor receptor superfamily member 10d [Source:HGNC Symbol;Acc:HGNC:11907]
TNFRSF21	tumor necrosis factor receptor superfamily member 21 [Source:HGNC Symbol;Acc:HGNC:13469]
TNFRSF11B	tumor necrosis factor receptor superfamily member 11b [Source:HGNC Symbol;Acc:HGNC:11909]
TNFRSF12A	tumor necrosis factor receptor superfamily member 12A [Source:HGNC Symbol;Acc:HGNC:18152]
CD27	CD27 molecule [Source:HGNC Symbol;Acc:HGNC:11922]
TNFRSF8	tumor necrosis factor receptor superfamily member 8 [Source:HGNC Symbol;Acc:HGNC:11923]
TNFRSF9	tumor necrosis factor receptor superfamily member 9 [Source:HGNC Symbol;Acc:HGNC:11924]
TNFRSF4	tumor necrosis factor receptor superfamily member 4 [Source:HGNC Symbol;Acc:HGNC:11918]
TNFRSF18	tumor necrosis factor receptor superfamily member 18 [Source:HGNC Symbol;Acc:HGNC:11914]
TNFRSF17	tumor necrosis factor receptor superfamily member 17 [Source:HGNC Symbol;Acc:HGNC:11913]
TNFRSF13B	tumor necrosis factor receptor superfamily member 13B [Source:HGNC Symbol;Acc:HGNC:18153]
TNFRSF19	tumor necrosis factor receptor superfamily member 19 [Source:HGNC Symbol;Acc:HGNC:11915]
RELT	RELT tumor necrosis factor receptor [Source:HGNC Symbol;Acc:HGNC:13764]
ACVRL1	activin A receptor like type 1 [Source:HGNC Symbol;Acc:HGNC:175]
PSMD3	proteasome 26S subunit, non-ATPase 3 [Source:HGNC Symbol;Acc:HGNC:9560]
PSMD9	proteasome 26S subunit, non-ATPase 9 [Source:HGNC Symbol;Acc:HGNC:9567]
PSMD12	proteasome 26S subunit, non-ATPase 12 [Source:HGNC Symbol;Acc:HGNC:9557]
PSMD11	proteasome 26S subunit, non-ATPase 11 [Source:HGNC Symbol;Acc:HGNC:9556]
PSMD6	proteasome 26S subunit, non-ATPase 6 [Source:HGNC Symbol;Acc:HGNC:9564]
PSMD7	proteasome 26S subunit, non-ATPase 7 [Source:HGNC Symbol;Acc:HGNC:9565]
PSMD13	proteasome 26S subunit, non-ATPase 13 [Source:HGNC Symbol;Acc:HGNC:9558]

PSMD14	proteasome 26S subunit, non-ATPase 14 [Source:HGNC Symbol;Acc:HGNC:16889]
PSMD8	proteasome 26S subunit, non-ATPase 8 [Source:HGNC Symbol;Acc:HGNC:9566]
SHFM1	split hand/foot malformation (ectrodactyly) type 1 [Source:HGNC Symbol;Acc:HGNC:10845]
PSMD2	proteasome 26S subunit, non-ATPase 2 [Source:HGNC Symbol;Acc:HGNC:9559]
PSMD1	proteasome 26S subunit, non-ATPase 1 [Source:HGNC Symbol;Acc:HGNC:9554]
ADRM1	adhesion regulating molecule 1 [Source:HGNC Symbol;Acc:HGNC:15759]
PSMC2	proteasome 26S subunit, ATPase 2 [Source:HGNC Symbol;Acc:HGNC:9548]
PSMC1	proteasome 26S subunit, ATPase 1 [Source:HGNC Symbol;Acc:HGNC:9547]
PSMC5	proteasome 26S subunit, ATPase 5 [Source:HGNC Symbol;Acc:HGNC:9552]
PSMC6	proteasome 26S subunit, ATPase 6 [Source:HGNC Symbol;Acc:HGNC:9553]
PSMC3	proteasome 26S subunit, ATPase 3 [Source:HGNC Symbol;Acc:HGNC:9549]
PSMC4	proteasome 26S subunit, ATPase 4 [Source:HGNC Symbol;Acc:HGNC:9551]
PSME1	proteasome activator subunit 1 [Source:HGNC Symbol;Acc:HGNC:9568]
PSME2	proteasome activator subunit 2 [Source:HGNC Symbol;Acc:HGNC:9569]
PSME3	proteasome activator subunit 3 [Source:HGNC Symbol;Acc:HGNC:9570]
PSME4	proteasome activator subunit 4 [Source:HGNC Symbol;Acc:HGNC:20635]
PSMA6	proteasome subunit alpha 6 [Source:HGNC Symbol;Acc:HGNC:9535]
PSMA2	proteasome subunit alpha 2 [Source:HGNC Symbol;Acc:HGNC:9531]
PSMA4	proteasome subunit alpha 4 [Source:HGNC Symbol;Acc:HGNC:9533]
PSMA7	proteasome subunit alpha 7 [Source:HGNC Symbol;Acc:HGNC:9536]
PSMA8	proteasome subunit alpha 8 [Source:HGNC Symbol;Acc:HGNC:22985]
PSMA5	proteasome subunit alpha 5 [Source:HGNC Symbol;Acc:HGNC:9534]
PSMA1	proteasome subunit alpha 1 [Source:HGNC Symbol;Acc:HGNC:9530]
PSMA3	proteasome subunit alpha 3 [Source:HGNC Symbol;Acc:HGNC:9532]
PSMB6	proteasome subunit beta 6 [Source:HGNC Symbol;Acc:HGNC:9543]
PSMB7	proteasome subunit beta 7 [Source:HGNC Symbol;Acc:HGNC:9544]
PSMB3	proteasome subunit beta 3 [Source:HGNC Symbol;Acc:HGNC:9540]
PSMB2	proteasome subunit beta 2 [Source:HGNC Symbol;Acc:HGNC:9539]
PSMB5	proteasome subunit beta 5 [Source:HGNC Symbol;Acc:HGNC:9542]
PSMB1	proteasome subunit beta 1 [Source:HGNC Symbol;Acc:HGNC:9537]
PSMB4	proteasome subunit beta 4 [Source:HGNC Symbol;Acc:HGNC:9541]
PSMB9	proteasome subunit beta 9 [Source:HGNC Symbol;Acc:HGNC:9546]
PSMB10	proteasome subunit beta 10 [Source:HGNC Symbol;Acc:HGNC:9538]
PSMB8	proteasome subunit beta 8 [Source:HGNC Symbol;Acc:HGNC:9545]
PSMB11	proteasome subunit beta 11 [Source:HGNC Symbol;Acc:HGNC:31963]
PSMF1	proteasome inhibitor subunit 1 [Source:HGNC Symbol;Acc:HGNC:9571]
POMP	proteasome maturation protein [Source:HGNC Symbol;Acc:HGNC:20330]
CD79A	CD79a molecule [Source:HGNC Symbol;Acc:HGNC:1698]
CD79B	CD79b molecule [Source:HGNC Symbol;Acc:HGNC:1699]
DAPP1	dual adaptor of phosphotyrosine and 3-phosphoinositides 1 [Source:HGNC Symbol;Acc:HGNC:16500]
IFITM1	interferon induced transmembrane protein 1 [Source:HGNC Symbol;Acc:HGNC:5412]

CD81	CD81 molecule [Source:HGNC Symbol;Acc:HGNC:1701]
CR2	complement component 3d receptor 2 [Source:HGNC Symbol;Acc:HGNC:2336]
FCGR2B	Fc fragment of IgG receptor IIb [Source:HGNC Symbol;Acc:HGNC:3618]
LILRB2	leukocyte immunoglobulin like receptor B2 [Source:HGNC Symbol;Acc:HGNC:6606]
LILRB1	leukocyte immunoglobulin like receptor B1 [Source:HGNC Symbol;Acc:HGNC:6605]
LILRB5	leukocyte immunoglobulin like receptor B5 [Source:HGNC Symbol;Acc:HGNC:6609]
LILRB4	leukocyte immunoglobulin like receptor B4 [Source:HGNC Symbol;Acc:HGNC:6608]
LILRA1	leukocyte immunoglobulin like receptor A1 [Source:HGNC Symbol;Acc:HGNC:6602]
LILRB3	leukocyte immunoglobulin like receptor B3 [Source:HGNC Symbol;Acc:HGNC:6607]
LILRA3	leukocyte immunoglobulin like receptor A3 [Source:HGNC Symbol;Acc:HGNC:6604]
LILRA2	leukocyte immunoglobulin like receptor A2 [Source:HGNC Symbol;Acc:HGNC:6603]
LILRA4	leukocyte immunoglobulin like receptor A4 [Source:HGNC Symbol;Acc:HGNC:15503]
LILRA6	leukocyte immunoglobulin like receptor A6 [Source:HGNC Symbol;Acc:HGNC:15495]
LILRA5	leukocyte immunoglobulin like receptor A5 [Source:HGNC Symbol;Acc:HGNC:16309]
CD72	CD72 molecule [Source:HGNC Symbol;Acc:HGNC:1696]
F3	coagulation factor III, tissue factor [Source:HGNC Symbol;Acc:HGNC:3541]
F7	coagulation factor VII [Source:HGNC Symbol;Acc:HGNC:3544]
F10	coagulation factor X [Source:HGNC Symbol;Acc:HGNC:3528]
F5	coagulation factor V [Source:HGNC Symbol;Acc:HGNC:3542]
F12	coagulation factor XII [Source:HGNC Symbol;Acc:HGNC:3530]
F11	coagulation factor XI [Source:HGNC Symbol;Acc:HGNC:3529]
F9	coagulation factor IX [Source:HGNC Symbol;Acc:HGNC:3551]
F8	coagulation factor VIII [Source:HGNC Symbol;Acc:HGNC:3546]
THBD	thrombomodulin [Source:HGNC Symbol;Acc:HGNC:11784]
PROCR	protein C receptor [Source:HGNC Symbol;Acc:HGNC:9452]
PROC	protein C, inactivator of coagulation factors Va and VIIIa [Source:HGNC Symbol;Acc:HGNC:9451]
F2RL2	coagulation factor II thrombin receptor like 2 [Source:HGNC Symbol;Acc:HGNC:3539]
F13A1	coagulation factor XIII A chain [Source:HGNC Symbol;Acc:HGNC:3531]
F13B	coagulation factor XIII B chain [Source:HGNC Symbol;Acc:HGNC:3534]
CPB2	carboxypeptidase B2 [Source:HGNC Symbol;Acc:HGNC:2300]
FGA	fibrinogen alpha chain [Source:HGNC Symbol;Acc:HGNC:3661]
FGB	fibrinogen beta chain [Source:HGNC Symbol;Acc:HGNC:3662]
FGG	fibrinogen gamma chain [Source:HGNC Symbol;Acc:HGNC:3694]
KLKB1	kallikrein B1 [Source:HGNC Symbol;Acc:HGNC:6371]
PLG	plasminogen [Source:HGNC Symbol;Acc:HGNC:9071]
TFPI	tissue factor pathway inhibitor [Source:HGNC Symbol;Acc:HGNC:11760]
SERPINC1	serpin family C member 1 [Source:HGNC Symbol;Acc:HGNC:775]
SERPIND1	serpin family D member 1 [Source:HGNC Symbol;Acc:HGNC:4838]
SERPINA5	serpin family A member 5 [Source:HGNC Symbol;Acc:HGNC:8723]
PROS1	protein S (alpha) [Source:HGNC Symbol;Acc:HGNC:9456]
SERPINB2	serpin family B member 2 [Source:HGNC Symbol;Acc:HGNC:8584]

PLAUR	plasminogen activator, urokinase receptor [Source:HGNC Symbol;Acc:HGNC:9053]
SERPINF2	serpin family F member 2 [Source:HGNC Symbol;Acc:HGNC:9075]
CFB	complement factor B [Source:HGNC Symbol;Acc:HGNC:1037]
CFD	complement factor D [Source:HGNC Symbol;Acc:HGNC:2771]
C3	complement component 3 [Source:HGNC Symbol;Acc:HGNC:1318]
C5	complement component 5 [Source:HGNC Symbol;Acc:HGNC:1331]
C6	complement component 6 [Source:HGNC Symbol;Acc:HGNC:1339]
C7	complement component 7 [Source:HGNC Symbol;Acc:HGNC:1346]
C8A	complement component 8 alpha subunit [Source:HGNC Symbol;Acc:HGNC:1352]
C8B	complement component 8, beta polypeptide [Source:HGNC Symbol;Acc:HGNC:1353]
C8G	complement component 8, gamma polypeptide [Source:HGNC Symbol;Acc:HGNC:1354]
C9	complement component 9 [Source:HGNC Symbol;Acc:HGNC:1358]
C1QA	complement component 1, q subcomponent, A chain [Source:HGNC Symbol;Acc:HGNC:1241]
C1QB	complement component 1, q subcomponent, B chain [Source:HGNC Symbol;Acc:HGNC:1242]
C1QC	complement component 1, q subcomponent, C chain [Source:HGNC Symbol;Acc:HGNC:1245]
C1R	complement C1r subcomponent [Source:HGNC Symbol;Acc:HGNC:1246]
C1S	complement component 1, s subcomponent [Source:HGNC Symbol;Acc:HGNC:1247]
MBL2	mannose binding lectin 2 [Source:HGNC Symbol;Acc:HGNC:6922]
MASP1	mannan binding lectin serine peptidase 1 [Source:HGNC Symbol;Acc:HGNC:6901]
MASP2	mannan binding lectin serine peptidase 2 [Source:HGNC Symbol;Acc:HGNC:6902]
C2	complement component 2 [Source:HGNC Symbol;Acc:HGNC:1248]
C4A	complement component 4A (Rodgers blood group) [Source:HGNC Symbol;Acc:HGNC:1323]
C4B	complement component 4B (Chido blood group) [Source:HGNC Symbol;Acc:HGNC:1324]
C3AR1	complement component 3a receptor 1 [Source:HGNC Symbol;Acc:HGNC:1319]
VSIG4	V-set and immunoglobulin domain containing 4 [Source:HGNC Symbol;Acc:HGNC:17032]
CR1	complement component 3b/4b receptor 1 (Knops blood group) [Source:HGNC Symbol;Acc:HGNC:2334]
CR1L	complement component 3b/4b receptor 1-like [Source:HGNC Symbol;Acc:HGNC:2335]
C5AR1	complement component 5a receptor 1 [Source:HGNC Symbol;Acc:HGNC:1338]
CFH	complement factor H [Source:HGNC Symbol;Acc:HGNC:4883]
CFI	complement factor I [Source:HGNC Symbol;Acc:HGNC:5394]
SERPING1	serpin family G member 1 [Source:HGNC Symbol;Acc:HGNC:1228]
CD55	CD55 molecule (Cromer blood group) [Source:HGNC Symbol;Acc:HGNC:2665]
CD46	CD46 molecule [Source:HGNC Symbol;Acc:HGNC:6953]
C4BPA	complement component 4 binding protein alpha [Source:HGNC Symbol;Acc:HGNC:1325]
C4BPB	complement component 4 binding protein beta [Source:HGNC Symbol;Acc:HGNC:1328]
CFHR3	complement factor H related 3 [Source:HGNC Symbol;Acc:HGNC:16980]
CFHR1	complement factor H related 1 [Source:HGNC Symbol;Acc:HGNC:4888]
CFHR2	complement factor H related 2 [Source:HGNC Symbol;Acc:HGNC:4890]
CFHR4	complement factor H related 4 [Source:HGNC Symbol;Acc:HGNC:16979]
CFHR5	complement factor H related 5 [Source:HGNC Symbol;Acc:HGNC:24668]
CD59	CD59 molecule [Source:HGNC Symbol;Acc:HGNC:1689]

CLU	clusterin [Source:HGNC Symbol;Acc:HGNC:2095]
TLR1	toll like receptor 1 [Source:HGNC Symbol;Acc:HGNC:11847]
TLR3	toll like receptor 3 [Source:HGNC Symbol;Acc:HGNC:11849]
TLR5	toll like receptor 5 [Source:HGNC Symbol;Acc:HGNC:11851]
TLR7	toll like receptor 7 [Source:HGNC Symbol;Acc:HGNC:15631]
TLR8	toll like receptor 8 [Source:HGNC Symbol;Acc:HGNC:15632]
TLR9	toll like receptor 9 [Source:HGNC Symbol;Acc:HGNC:15633]
IRF5	interferon regulatory factor 5 [Source:HGNC Symbol;Acc:HGNC:6120]
IFIH1	interferon induced with helicase C domain 1 [Source:HGNC Symbol;Acc:HGNC:18873]
DHX58	DEXH-box helicase 58 [Source:HGNC Symbol;Acc:HGNC:29517]
AZI2	5-azacytidine induced 2 [Source:HGNC Symbol;Acc:HGNC:24002]
TBKBP1	TBK1 binding protein 1 [Source:HGNC Symbol;Acc:HGNC:30140]
RNF125	ring finger protein 125 [Source:HGNC Symbol;Acc:HGNC:21150]
ISG15	ISG15 ubiquitin-like modifier [Source:HGNC Symbol;Acc:HGNC:4053]
NLRX1	NLR family member X1 [Source:HGNC Symbol;Acc:HGNC:29890]
OTUD5	OTU deubiquitinase 5 [Source:HGNC Symbol;Acc:HGNC:25402]
SIKE1	suppressor of IKBKE 1 [Source:HGNC Symbol;Acc:HGNC:26119]
DDX3X	DEAD-box helicase 3, X-linked [Source:HGNC Symbol;Acc:HGNC:2745]
PIN1	peptidylprolyl cis/trans isomerase, NIMA-interacting 1 [Source:HGNC Symbol;Acc:HGNC:8988]
TKFC	triokinase and FMN cyclase [Source:HGNC Symbol;Acc:HGNC:24552]
PPP1R3A	protein phosphatase 1 regulatory subunit 3A [Source:HGNC Symbol;Acc:HGNC:9291]
PPP1R3C	protein phosphatase 1 regulatory subunit 3C [Source:HGNC Symbol;Acc:HGNC:9293]
PPP1R3D	protein phosphatase 1 regulatory subunit 3D [Source:HGNC Symbol;Acc:HGNC:9294]
PPP1R3B	protein phosphatase 1 regulatory subunit 3B [Source:HGNC Symbol;Acc:HGNC:14942]
PPP1R3E	protein phosphatase 1 regulatory subunit 3E [Source:HGNC Symbol;Acc:HGNC:14943]
PPP1R3F	protein phosphatase 1 regulatory subunit 3F [Source:HGNC Symbol;Acc:HGNC:14944]
PRKAR1A	protein kinase cAMP-dependent type I regulatory subunit alpha [Source:HGNC Symbol;Acc:HGNC:9388]
PRKAR2A	protein kinase cAMP-dependent type II regulatory subunit alpha [Source:HGNC Symbol;Acc:HGNC:9391]
PRKAR2B	protein kinase cAMP-dependent type II regulatory subunit beta [Source:HGNC Symbol;Acc:HGNC:9392]
PRKAR1B	protein kinase cAMP-dependent type I regulatory subunit beta [Source:HGNC Symbol;Acc:HGNC:9390]
FLOT2	flotillin 2 [Source:HGNC Symbol;Acc:HGNC:3758]
FLOT1	flotillin 1 [Source:HGNC Symbol;Acc:HGNC:3757]
SH2B2	SH2B adaptor protein 2 [Source:HGNC Symbol;Acc:HGNC:17381]
RHOQ	ras homolog family member Q [Source:HGNC Symbol;Acc:HGNC:17736]
EXOC7	exocyst complex component 7 [Source:HGNC Symbol;Acc:HGNC:23214]
TRIP10	thyroid hormone receptor interactor 10 [Source:HGNC Symbol;Acc:HGNC:12304]
CTSA	cathepsin A [Source:HGNC Symbol;Acc:HGNC:9251]
CTSE	cathepsin E [Source:HGNC Symbol;Acc:HGNC:2530]
CTSG	cathepsin G [Source:HGNC Symbol;Acc:HGNC:2532]

NAPSA	napsin A aspartic peptidase [Source:HGNC Symbol;Acc:HGNC:13395]
LGMN	legumain [Source:HGNC Symbol;Acc:HGNC:9472]
TPP1	tripeptidyl peptidase 1 [Source:HGNC Symbol;Acc:HGNC:2073]
GLA	galactosidase alpha [Source:HGNC Symbol;Acc:HGNC:4296]
GLB1	galactosidase beta 1 [Source:HGNC Symbol;Acc:HGNC:4298]
IDUA	iduronidase, alpha-L- [Source:HGNC Symbol;Acc:HGNC:5391]
NAGA	alpha-N-acetylgalactosaminidase [Source:HGNC Symbol;Acc:HGNC:7631]
NAGLU	N-acetyl-alpha-glucosaminidase [Source:HGNC Symbol;Acc:HGNC:7632]
GALC	galactosylceramidase [Source:HGNC Symbol;Acc:HGNC:4115]
GUSB	glucuronidase beta [Source:HGNC Symbol;Acc:HGNC:4696]
FUCA1	fucosidase, alpha-L- 1, tissue [Source:HGNC Symbol;Acc:HGNC:4006]
FUCA2	fucosidase, alpha-L- 2, plasma [Source:HGNC Symbol;Acc:HGNC:4008]
HEXA	hexosaminidase subunit alpha [Source:HGNC Symbol;Acc:HGNC:4878]
HEXB	hexosaminidase subunit beta [Source:HGNC Symbol;Acc:HGNC:4879]
MANBA	mannosidase beta [Source:HGNC Symbol;Acc:HGNC:6831]
MAN2B1	mannosidase alpha class 2B member 1 [Source:HGNC Symbol;Acc:HGNC:6826]
NEU1	neuraminidase 1 (lysosomal sialidase) [Source:HGNC Symbol;Acc:HGNC:7758]
HYAL2	hyaluronoglucosaminidase 2 [Source:HGNC Symbol;Acc:HGNC:5321]
HYAL1	hyaluronoglucosaminidase 1 [Source:HGNC Symbol;Acc:HGNC:5320]
SPAM1	sperm adhesion molecule 1 [Source:HGNC Symbol;Acc:HGNC:11217]
HYAL4	hyaluronoglucosaminidase 4 [Source:HGNC Symbol;Acc:HGNC:5323]
HYAL3	hyaluronoglucosaminidase 3 [Source:HGNC Symbol;Acc:HGNC:5322]
ARSA	arylsulfatase A [Source:HGNC Symbol;Acc:HGNC:713]
ARSB	arylsulfatase B [Source:HGNC Symbol;Acc:HGNC:714]
ARSG	arylsulfatase G [Source:HGNC Symbol;Acc:HGNC:24102]
GALNS	galactosamine (N-acetyl)-6-sulfatase [Source:HGNC Symbol;Acc:HGNC:4122]
GNS	glucosamine (N-acetyl)-6-sulfatase [Source:HGNC Symbol;Acc:HGNC:4422]
IDS	iduronate 2-sulfatase [Source:HGNC Symbol;Acc:HGNC:5389]
SGSH	N-sulfoglucosamine sulfohydrolase [Source:HGNC Symbol;Acc:HGNC:10818]
PLA2G15	phospholipase A2 group XV [Source:HGNC Symbol;Acc:HGNC:17163]
DNASE2	deoxyribonuclease II, lysosomal [Source:HGNC Symbol;Acc:HGNC:2960]
DNASE2B	deoxyribonuclease II beta [Source:HGNC Symbol;Acc:HGNC:28875]
ACP2	acid phosphatase 2, lysosomal [Source:HGNC Symbol;Acc:HGNC:123]
ACP5	acid phosphatase 5, tartrate resistant [Source:HGNC Symbol;Acc:HGNC:124]
SMPD1	sphingomyelin phosphodiesterase 1 [Source:HGNC Symbol;Acc:HGNC:11120]
ASAH1	N-acylsphingosine amidohydrolase (acid ceramidase) 1 [Source:HGNC Symbol;Acc:HGNC:735]
AGA	aspartylglucosaminidase [Source:HGNC Symbol;Acc:HGNC:318]
PSAP	prosaposin [Source:HGNC Symbol;Acc:HGNC:9498]
PSAPL1	prosaposin-like 1 (gene/pseudogene) [Source:HGNC Symbol;Acc:HGNC:33131]
GM2A	GM2 ganglioside activator [Source:HGNC Symbol;Acc:HGNC:4367]
PPT2	palmitoyl-protein thioesterase 2 [Source:HGNC Symbol;Acc:HGNC:9326]

CD68	CD68 molecule [Source:HGNC Symbol;Acc:HGNC:1693]
CD63	CD63 molecule [Source:HGNC Symbol;Acc:HGNC:1692]
SCARB2	scavenger receptor class B member 2 [Source:HGNC Symbol;Acc:HGNC:1665]
CTNS	cystinosin, lysosomal cystine transporter [Source:HGNC Symbol;Acc:HGNC:2518]
SLC17A5	solute carrier family 17 member 5 [Source:HGNC Symbol;Acc:HGNC:10933]
SLC11A1	solute carrier family 11 member 1 [Source:HGNC Symbol;Acc:HGNC:10907]
SLC11A2	solute carrier family 11 member 2 [Source:HGNC Symbol;Acc:HGNC:10908]
LAPTM4B	lysosomal protein transmembrane 4 beta [Source:HGNC Symbol;Acc:HGNC:13646]
LAPTM5	lysosomal protein transmembrane 5 [Source:HGNC Symbol;Acc:HGNC:29612]
LAPTM4A	lysosomal protein transmembrane 4 alpha [Source:HGNC Symbol;Acc:HGNC:6924]
ABCA2	ATP binding cassette subfamily A member 2 [Source:HGNC Symbol;Acc:HGNC:32]
ABCB9	ATP binding cassette subfamily B member 9 [Source:HGNC Symbol;Acc:HGNC:50]
CD164	CD164 molecule [Source:HGNC Symbol;Acc:HGNC:1632]
ENTPD4	ectonucleoside triphosphate diphosphohydrolase 4 [Source:HGNC Symbol;Acc:HGNC:14573]
CLN3	ceroid-lipofuscinosis, neuronal 3 [Source:HGNC Symbol;Acc:HGNC:2074]
CLN5	ceroid-lipofuscinosis, neuronal 5 [Source:HGNC Symbol;Acc:HGNC:2076]
MFSD8	major facilitator superfamily domain containing 8 [Source:HGNC Symbol;Acc:HGNC:28486]
HGSNAT	heparan-alpha-glucosaminide N-acetyltransferase [Source:HGNC Symbol;Acc:HGNC:26527]
SUMF1	sulfatase modifying factor 1 [Source:HGNC Symbol;Acc:HGNC:20376]
GNPTAB	N-acetylglucosamine-1-phosphate transferase alpha and beta subunits [Source:HGNC Symbol;Acc:HGNC:29670]
GNPTG	N-acetylglucosamine-1-phosphate transferase gamma subunit [Source:HGNC Symbol;Acc:HGNC:23026]
NAGPA	N-acetylglucosamine-1-phosphodiester alpha-N-acetylglucosaminidase [Source:HGNC Symbol;Acc:HGNC:17378]
IGF2R	insulin like growth factor 2 receptor [Source:HGNC Symbol;Acc:HGNC:5467]
M6PR	mannose-6-phosphate receptor, cation dependent [Source:HGNC Symbol;Acc:HGNC:6752]
CLTA	clathrin light chain A [Source:HGNC Symbol;Acc:HGNC:2090]
CLTB	clathrin light chain B [Source:HGNC Symbol;Acc:HGNC:2091]
CLTC	clathrin heavy chain [Source:HGNC Symbol;Acc:HGNC:2092]
CLTCL1	clathrin heavy chain like 1 [Source:HGNC Symbol;Acc:HGNC:2093]
AP1G1	adaptor related protein complex 1 gamma 1 subunit [Source:HGNC Symbol;Acc:HGNC:555]
AP1G2	adaptor related protein complex 1 gamma 2 subunit [Source:HGNC Symbol;Acc:HGNC:556]
AP1B1	adaptor related protein complex 1 beta 1 subunit [Source:HGNC Symbol;Acc:HGNC:554]
AP1M1	adaptor related protein complex 1 mu 1 subunit [Source:HGNC Symbol;Acc:HGNC:13667]
AP1M2	adaptor related protein complex 1 mu 2 subunit [Source:HGNC Symbol;Acc:HGNC:558]
AP1S1	adaptor related protein complex 1 sigma 1 subunit [Source:HGNC Symbol;Acc:HGNC:559]
AP1S2	adaptor related protein complex 1 sigma 2 subunit [Source:HGNC Symbol;Acc:HGNC:560]
AP1S3	adaptor related protein complex 1 sigma 3 subunit [Source:HGNC Symbol;Acc:HGNC:18971]
AP3D1	adaptor related protein complex 3 delta 1 subunit [Source:HGNC Symbol;Acc:HGNC:568]
AP3B2	adaptor related protein complex 3 beta 2 subunit [Source:HGNC Symbol;Acc:HGNC:567]
AP3B1	adaptor related protein complex 3 beta 1 subunit [Source:HGNC Symbol;Acc:HGNC:566]

AP3M1	adaptor related protein complex 3 mu 1 subunit [Source:HGNC Symbol;Acc:HGNC:569]
AP3M2	adaptor related protein complex 3 mu 2 subunit [Source:HGNC Symbol;Acc:HGNC:570]
AP3S2	adaptor related protein complex 3 sigma 2 subunit [Source:HGNC Symbol;Acc:HGNC:571]
AP3S1	adaptor related protein complex 3 sigma 1 subunit [Source:HGNC Symbol;Acc:HGNC:2013]
AP4E1	adaptor related protein complex 4 epsilon 1 subunit [Source:HGNC Symbol;Acc:HGNC:573]
AP4B1	adaptor related protein complex 4 beta 1 subunit [Source:HGNC Symbol;Acc:HGNC:572]
AP4M1	adaptor related protein complex 4 mu 1 subunit [Source:HGNC Symbol;Acc:HGNC:574]
AP4S1	adaptor related protein complex 4 sigma 1 subunit [Source:HGNC Symbol;Acc:HGNC:575]
GGA2	golgi associated, gamma adaptin ear containing, ARF binding protein 2 [Source:HGNC Symbol;Acc:HGNC:16064]
GGA3	golgi associated, gamma adaptin ear containing, ARF binding protein 3 [Source:HGNC Symbol;Acc:HGNC:17079]
GGA1	golgi associated, gamma adaptin ear containing, ARF binding protein 1 [Source:HGNC Symbol;Acc:HGNC:17842]
LITAF	lipopolysaccharide induced TNF factor [Source:HGNC Symbol;Acc:HGNC:16841]
ARF1	ADP ribosylation factor 1 [Source:HGNC Symbol;Acc:HGNC:652]
FCER1A	Fc fragment of IgE receptor Ia [Source:HGNC Symbol;Acc:HGNC:3609]
MS4A2	membrane spanning 4-domains A2 [Source:HGNC Symbol;Acc:HGNC:7316]
FCER1G	Fc fragment of IgE receptor Ig [Source:HGNC Symbol;Acc:HGNC:3611]
AGPAT3	1-acylglycerol-3-phosphate O-acyltransferase 3 [Source:HGNC Symbol;Acc:HGNC:326]
AGPAT4	1-acylglycerol-3-phosphate O-acyltransferase 4 [Source:HGNC Symbol;Acc:HGNC:20885]
AGPAT5	1-acylglycerol-3-phosphate O-acyltransferase 5 [Source:HGNC Symbol;Acc:HGNC:20886]
AVP	arginine vasopressin [Source:HGNC Symbol;Acc:HGNC:894]
GRM2	glutamate metabotropic receptor 2 [Source:HGNC Symbol;Acc:HGNC:4594]
GRM3	glutamate metabotropic receptor 3 [Source:HGNC Symbol;Acc:HGNC:4595]
GRM4	glutamate metabotropic receptor 4 [Source:HGNC Symbol;Acc:HGNC:4596]
GRM6	glutamate metabotropic receptor 6 [Source:HGNC Symbol;Acc:HGNC:4598]
GRM7	glutamate metabotropic receptor 7 [Source:HGNC Symbol;Acc:HGNC:4599]
GRM8	glutamate metabotropic receptor 8 [Source:HGNC Symbol;Acc:HGNC:4600]
AVPR2	arginine vasopressin receptor 2 [Source:HGNC Symbol;Acc:HGNC:897]
CYTH3	cytohesin 3 [Source:HGNC Symbol;Acc:HGNC:9504]
CYTH4	cytohesin 4 [Source:HGNC Symbol;Acc:HGNC:9505]
CYTH2	cytohesin 2 [Source:HGNC Symbol;Acc:HGNC:9502]
CYTH1	cytohesin 1 [Source:HGNC Symbol;Acc:HGNC:9501]
DNM1	dynamin 1 [Source:HGNC Symbol;Acc:HGNC:2972]
DNM3	dynamin 3 [Source:HGNC Symbol;Acc:HGNC:29125]
DNM2	dynamin 2 [Source:HGNC Symbol;Acc:HGNC:2974]
KLF4	Kruppel like factor 4 [Source:HGNC Symbol;Acc:HGNC:6348]
TBX3	T-box 3 [Source:HGNC Symbol;Acc:HGNC:11602]
NANOG	Nanog homeobox [Source:HGNC Symbol;Acc:HGNC:20857]
NANOGP8	Nanog homeobox pseudogene 8 [Source:HGNC Symbol;Acc:HGNC:23106]
TCF3	transcription factor 3 [Source:HGNC Symbol;Acc:HGNC:11633]

ESRRB	estrogen related receptor beta [Source:HGNC Symbol;Acc:HGNC:3473]
HESX1	HESX homeobox 1 [Source:HGNC Symbol;Acc:HGNC:4877]
ZIC3	Zic family member 3 [Source:HGNC Symbol;Acc:HGNC:12874]
SKIL	SKI-like proto-oncogene [Source:HGNC Symbol;Acc:HGNC:10897]
SMARCAD1	SWI/SNF-related, matrix-associated actin-dependent regulator of chromatin, subfamily a, containing DEAD/H box 1 [Source:HGNC Symbol;Acc:HGNC:18398]
KAT6A	lysine acetyltransferase 6A [Source:HGNC Symbol;Acc:HGNC:13013]
SETDB1	SET domain bifurcated 1 [Source:HGNC Symbol;Acc:HGNC:10761]
JARID2	jumonji and AT-rich interaction domain containing 2 [Source:HGNC Symbol;Acc:HGNC:6196]
REST	RE1 silencing transcription factor [Source:HGNC Symbol;Acc:HGNC:9966]
RIF1	replication timing regulatory factor 1 [Source:HGNC Symbol;Acc:HGNC:23207]
PCGF1	polycomb group ring finger 1 [Source:HGNC Symbol;Acc:HGNC:17615]
PCGF2	polycomb group ring finger 2 [Source:HGNC Symbol;Acc:HGNC:12929]
PCGF3	polycomb group ring finger 3 [Source:HGNC Symbol;Acc:HGNC:10066]
BMI1	BMI1 proto-oncogene, polycomb ring finger [Source:HGNC Symbol;Acc:HGNC:1066]
COMMD3-BMI1	COMMD3-BMI1 readthrough [Source:HGNC Symbol;Acc:HGNC:48326]
PCGF5	polycomb group ring finger 5 [Source:HGNC Symbol;Acc:HGNC:28264]
PCGF6	polycomb group ring finger 6 [Source:HGNC Symbol;Acc:HGNC:21156]
MEIS1	Meis homeobox 1 [Source:HGNC Symbol;Acc:HGNC:7000]
HOXB1	homeobox B1 [Source:HGNC Symbol;Acc:HGNC:5111]
HOXA1	homeobox A1 [Source:HGNC Symbol;Acc:HGNC:5099]
HOXD1	homeobox D1 [Source:HGNC Symbol;Acc:HGNC:5132]
LHX5	LIM homeobox 5 [Source:HGNC Symbol;Acc:HGNC:14216]
OTX1	orthodenticle homeobox 1 [Source:HGNC Symbol;Acc:HGNC:8521]
NEUROG1	neurogenin 1 [Source:HGNC Symbol;Acc:HGNC:7764]
HAND1	heart and neural crest derivatives expressed 1 [Source:HGNC Symbol;Acc:HGNC:4807]
DLX5	distal-less homeobox 5 [Source:HGNC Symbol;Acc:HGNC:2918]
MYF5	myogenic factor 5 [Source:HGNC Symbol;Acc:HGNC:7565]
ONECUT1	one cut homeobox 1 [Source:HGNC Symbol;Acc:HGNC:8138]
ISL1	ISL LIM homeobox 1 [Source:HGNC Symbol;Acc:HGNC:6132]
ZFHX3	zinc finger homeobox 3 [Source:HGNC Symbol;Acc:HGNC:777]
ESX1	ESX homeobox 1 [Source:HGNC Symbol;Acc:HGNC:14865]
NPNT	nephronectin [Source:HGNC Symbol;Acc:HGNC:27405]
FRAS1	Fraser extracellular matrix complex subunit 1 [Source:HGNC Symbol;Acc:HGNC:19185]
FREM2	FRAS1 related extracellular matrix protein 2 [Source:HGNC Symbol;Acc:HGNC:25396]
FREM1	FRAS1 related extracellular matrix 1 [Source:HGNC Symbol;Acc:HGNC:23399]
DSPP	dentin sialophosphoprotein [Source:HGNC Symbol;Acc:HGNC:3054]
DMP1	dentin matrix acidic phosphoprotein 1 [Source:HGNC Symbol;Acc:HGNC:2932]
AGRN	agrin [Source:HGNC Symbol;Acc:HGNC:329]
HSPG2	heparan sulfate proteoglycan 2 [Source:HGNC Symbol;Acc:HGNC:5273]
SV2C	synaptic vesicle glycoprotein 2C [Source:HGNC Symbol;Acc:HGNC:30670]

SV2B	synaptic vesicle glycoprotein 2B [Source:HGNC Symbol;Acc:HGNC:16874]
SV2A	synaptic vesicle glycoprotein 2A [Source:HGNC Symbol;Acc:HGNC:20566]
GP5	glycoprotein V platelet [Source:HGNC Symbol;Acc:HGNC:4443]
GP1BA	glycoprotein Ib platelet alpha subunit [Source:HGNC Symbol;Acc:HGNC:4439]
GP1BB	glycoprotein Ib platelet beta subunit [Source:HGNC Symbol;Acc:HGNC:4440]
GP9	glycoprotein IX platelet [Source:HGNC Symbol;Acc:HGNC:4444]
GP6	glycoprotein VI platelet [Source:HGNC Symbol;Acc:HGNC:14388]
DAG1	dystroglycan 1 [Source:HGNC Symbol;Acc:HGNC:2666]
CD47	CD47 molecule [Source:HGNC Symbol;Acc:HGNC:1682]
HMMR	hyaluronan mediated motility receptor [Source:HGNC Symbol;Acc:HGNC:5012]
EML4	echinoderm microtubule associated protein like 4 [Source:HGNC Symbol;Acc:HGNC:1316]
ALK	anaplastic lymphoma receptor tyrosine kinase [Source:HGNC Symbol;Acc:HGNC:427]
BATF3	basic leucine zipper ATF-like transcription factor 3 [Source:HGNC Symbol;Acc:HGNC:28915]
BATF	basic leucine zipper ATF-like transcription factor [Source:HGNC Symbol;Acc:HGNC:958]
BATF2	basic leucine zipper ATF-like transcription factor 2 [Source:HGNC Symbol;Acc:HGNC:25163]
DCC	DCC netrin 1 receptor [Source:HGNC Symbol;Acc:HGNC:2701]
APPL1	adaptor protein, phosphotyrosine interacting with PH domain and leucine zipper 1 [Source:HGNC Symbol;Acc:HGNC:24035]
ARHGEF11	Rho guanine nucleotide exchange factor 11 [Source:HGNC Symbol;Acc:HGNC:14580]
PLEKHG5	pleckstrin homology and RhoGEF domain containing G5 [Source:HGNC Symbol;Acc:HGNC:29105]
PTGER4	prostaglandin E receptor 4 [Source:HGNC Symbol;Acc:HGNC:9596]
NKX3-1	NK3 homeobox 1 [Source:HGNC Symbol;Acc:HGNC:7838]
TRAF4	TNF receptor associated factor 4 [Source:HGNC Symbol;Acc:HGNC:12034]
PIM2	Pim-2 proto-oncogene, serine/threonine kinase [Source:HGNC Symbol;Acc:HGNC:8987]
MMP2	matrix metallopeptidase 2 [Source:HGNC Symbol;Acc:HGNC:7166]
CCDC6	coiled-coil domain containing 6 [Source:HGNC Symbol;Acc:HGNC:18782]
NCOA4	nuclear receptor coactivator 4 [Source:HGNC Symbol;Acc:HGNC:7671]
TPM3	tropomyosin 3 [Source:HGNC Symbol;Acc:HGNC:12012]
TPR	translocated promoter region, nuclear basket protein [Source:HGNC Symbol;Acc:HGNC:12017]
TFG	TRK-fused gene [Source:HGNC Symbol;Acc:HGNC:11758]
PAX8	paired box 8 [Source:HGNC Symbol;Acc:HGNC:8622]
RARB	retinoic acid receptor beta [Source:HGNC Symbol;Acc:HGNC:9865]
ESR1	estrogen receptor 1 [Source:HGNC Symbol;Acc:HGNC:3467]
ESR2	estrogen receptor 2 [Source:HGNC Symbol;Acc:HGNC:3468]
NCOA1	nuclear receptor coactivator 1 [Source:HGNC Symbol;Acc:HGNC:7668]
NCOA3	nuclear receptor coactivator 3 [Source:HGNC Symbol;Acc:HGNC:7670]
JUP	junction plakoglobin [Source:HGNC Symbol;Acc:HGNC:6207]
ZBTB16	zinc finger and BTB domain containing 16 [Source:HGNC Symbol;Acc:HGNC:12930]
RARA	retinoic acid receptor alpha [Source:HGNC Symbol;Acc:HGNC:9864]
RUNX1T1	RUNX1 translocation partner 1 [Source:HGNC Symbol;Acc:HGNC:1535]
SPI1	Spi-1 proto-oncogene [Source:HGNC Symbol;Acc:HGNC:11241]

CEBPA	CCAAT/enhancer binding protein alpha [Source:HGNC Symbol;Acc:HGNC:1833]
CKS1B	CDC28 protein kinase regulatory subunit 1B [Source:HGNC Symbol;Acc:HGNC:19083]
CKS2	CDC28 protein kinase regulatory subunit 2 [Source:HGNC Symbol;Acc:HGNC:2000]
POLK	polymerase (DNA) kappa [Source:HGNC Symbol;Acc:HGNC:9183]
MLH1	mutL homolog 1 [Source:HGNC Symbol;Acc:HGNC:7127]
MSH2	mutS homolog 2 [Source:HGNC Symbol;Acc:HGNC:7325]
MSH3	mutS homolog 3 [Source:HGNC Symbol;Acc:HGNC:7326]
MSH6	mutS homolog 6 [Source:HGNC Symbol;Acc:HGNC:7329]
BRCA2	BRCA2, DNA repair associated [Source:HGNC Symbol;Acc:HGNC:1101]
RAD51	RAD51 recombinase [Source:HGNC Symbol;Acc:HGNC:9817]
GSTA5	glutathione S-transferase alpha 5 [Source:HGNC Symbol;Acc:HGNC:19662]
GSTA2	glutathione S-transferase alpha 2 [Source:HGNC Symbol;Acc:HGNC:4627]
GSTA4	glutathione S-transferase alpha 4 [Source:HGNC Symbol;Acc:HGNC:4629]
GSTO2	glutathione S-transferase omega 2 [Source:HGNC Symbol;Acc:HGNC:23064]
GSTM4	glutathione S-transferase mu 4 [Source:HGNC Symbol;Acc:HGNC:4636]
GSTT2	glutathione S-transferase theta 2 (gene/pseudogene) [Source:HGNC Symbol;Acc:HGNC:4642]
GSTT1	glutathione S-transferase theta 1 [Source:HGNC Symbol;Acc:HGNC:4641]
GSTM3	glutathione S-transferase mu 3 (brain) [Source:HGNC Symbol;Acc:HGNC:4635]
MGST1	microsomal glutathione S-transferase 1 [Source:HGNC Symbol;Acc:HGNC:7061]
MGST3	microsomal glutathione S-transferase 3 [Source:HGNC Symbol;Acc:HGNC:7064]
GSTM1	glutathione S-transferase mu 1 [Source:HGNC Symbol;Acc:HGNC:4632]
GSTM5	glutathione S-transferase mu 5 [Source:HGNC Symbol;Acc:HGNC:4637]
MGST2	microsomal glutathione S-transferase 2 [Source:HGNC Symbol;Acc:HGNC:7063]
GSTA1	glutathione S-transferase alpha 1 [Source:HGNC Symbol;Acc:HGNC:4626]
GSTM2	glutathione S-transferase mu 2 (muscle) [Source:HGNC Symbol;Acc:HGNC:4634]
GSTA3	glutathione S-transferase alpha 3 [Source:HGNC Symbol;Acc:HGNC:4628]
GSTO1	glutathione S-transferase omega 1 [Source:HGNC Symbol;Acc:HGNC:13312]
GSTT2B	glutathione S-transferase theta 2B (gene/pseudogene) [Source:HGNC Symbol;Acc:HGNC:33437]
GSTP1	glutathione S-transferase pi 1 [Source:HGNC Symbol;Acc:HGNC:4638]
TXNRD1	thioredoxin reductase 1 [Source:HGNC Symbol;Acc:HGNC:12437]
TXNRD3	thioredoxin reductase 3 [Source:HGNC Symbol;Acc:HGNC:20667]
TXNRD2	thioredoxin reductase 2 [Source:HGNC Symbol;Acc:HGNC:18155]
ARNT2	aryl hydrocarbon receptor nuclear translocator 2 [Source:HGNC Symbol;Acc:HGNC:16876]
AR	androgen receptor [Source:HGNC Symbol;Acc:HGNC:644]
KLK3	kallikrein related peptidase 3 [Source:HGNC Symbol;Acc:HGNC:6364]
MPO	myeloperoxidase [Source:HGNC Symbol;Acc:HGNC:7218]
SIN3A	SIN3 transcription regulator family member A [Source:HGNC Symbol;Acc:HGNC:19353]
PER2	period circadian clock 2 [Source:HGNC Symbol;Acc:HGNC:8846]
FCGR1A	Fc fragment of IgG receptor Ia [Source:HGNC Symbol;Acc:HGNC:3613]
CEBPE	CCAAT/enhancer binding protein epsilon [Source:HGNC Symbol;Acc:HGNC:1836]
ETV6	ETS variant 6 [Source:HGNC Symbol;Acc:HGNC:3495]

ETV7	ETS variant 7 [Source:HGNC Symbol;Acc:HGNC:18160]
DEFA1	defensin alpha 1 [Source:HGNC Symbol;Acc:HGNC:2761]
DEFA3	defensin alpha 3 [Source:HGNC Symbol;Acc:HGNC:2762]
DEFA4	defensin alpha 4 [Source:HGNC Symbol;Acc:HGNC:2763]
DEFA5	defensin alpha 5 [Source:HGNC Symbol;Acc:HGNC:2764]
DEFA6	defensin alpha 6 [Source:HGNC Symbol;Acc:HGNC:2765]
DEFA1B	defensin alpha 1B [Source:HGNC Symbol;Acc:HGNC:33596]
ELANE	elastase, neutrophil expressed [Source:HGNC Symbol;Acc:HGNC:3309]
KMT2A	lysine methyltransferase 2A [Source:HGNC Symbol;Acc:HGNC:7132]
AFF1	AF4/FMR2 family member 1 [Source:HGNC Symbol;Acc:HGNC:7135]
CDK9	cyclin dependent kinase 9 [Source:HGNC Symbol;Acc:HGNC:1780]
CCNT1	cyclin T1 [Source:HGNC Symbol;Acc:HGNC:1599]
CCNT2	cyclin T2 [Source:HGNC Symbol;Acc:HGNC:1600]
MLLT1	myeloid/lymphoid or mixed-lineage leukemia; translocated to, 1 [Source:HGNC Symbol;Acc:HGNC:7134]
MLLT3	myeloid/lymphoid or mixed-lineage leukemia; translocated to, 3 [Source:HGNC Symbol;Acc:HGNC:7136]
DOT1L	DOT1 like histone lysine methyltransferase [Source:HGNC Symbol;Acc:HGNC:24948]
LMO2	LIM domain only 2 [Source:HGNC Symbol;Acc:HGNC:6642]
PBX3	PBX homeobox 3 [Source:HGNC Symbol;Acc:HGNC:8634]
KLF3	Kruppel like factor 3 [Source:HGNC Symbol;Acc:HGNC:16516]
HOXA9	homeobox A9 [Source:HGNC Symbol;Acc:HGNC:5109]
HOXA10	homeobox A10 [Source:HGNC Symbol;Acc:HGNC:5100]
JMJD1C	jumonji domain containing 1C [Source:HGNC Symbol;Acc:HGNC:12313]
HMGA2	high mobility group AT-hook 2 [Source:HGNC Symbol;Acc:HGNC:5009]
KDM6A	lysine demethylase 6A [Source:HGNC Symbol;Acc:HGNC:12637]
SUPT3H	SPT3 homolog, SAGA and STAGA complex component [Source:HGNC Symbol;Acc:HGNC:11466]
PROM1	prominin 1 [Source:HGNC Symbol;Acc:HGNC:9454]
BMP2K	BMP2 inducible kinase [Source:HGNC Symbol;Acc:HGNC:18041]
CDK14	cyclin dependent kinase 14 [Source:HGNC Symbol;Acc:HGNC:8883]
HOXA11	homeobox A11 [Source:HGNC Symbol;Acc:HGNC:5101]
SIX1	SIX homeobox 1 [Source:HGNC Symbol;Acc:HGNC:10887]
SIX4	SIX homeobox 4 [Source:HGNC Symbol;Acc:HGNC:10890]
EYA1	EYA transcriptional coactivator and phosphatase 1 [Source:HGNC Symbol;Acc:HGNC:3519]
HPGD	hydroxyprostaglandin dehydrogenase 15-(NAD) [Source:HGNC Symbol;Acc:HGNC:5154]
GRIA3	glutamate ionotropic receptor AMPA type subunit 3 [Source:HGNC Symbol;Acc:HGNC:4573]
FUT8	fucosyltransferase 8 [Source:HGNC Symbol;Acc:HGNC:4019]
TLX3	T-cell leukemia homeobox 3 [Source:HGNC Symbol;Acc:HGNC:13532]
TLX1	T-cell leukemia homeobox 1 [Source:HGNC Symbol;Acc:HGNC:5056]
BCL11B	B-cell CLL/lymphoma 11B [Source:HGNC Symbol;Acc:HGNC:13222]
LDB1	LIM domain binding 1 [Source:HGNC Symbol;Acc:HGNC:6532]

LYL1	lymphoblastic leukemia associated hematopoiesis regulator 1 [Source:HGNC Symbol;Acc:HGNC:6734]
HHEX	hematopoietically expressed homeobox [Source:HGNC Symbol;Acc:HGNC:4901]
MAF	MAF bZIP transcription factor [Source:HGNC Symbol;Acc:HGNC:6776]
WHSC1	Wolf-Hirschhorn syndrome candidate 1 [Source:HGNC Symbol;Acc:HGNC:12766]
PAX5	paired box 5 [Source:HGNC Symbol;Acc:HGNC:8619]
PRCC	papillary renal cell carcinoma (translocation-associated) [Source:HGNC Symbol;Acc:HGNC:9343]
TMPRSS2	transmembrane protease, serine 2 [Source:HGNC Symbol;Acc:HGNC:11876]
ERG	ERG, ETS transcription factor [Source:HGNC Symbol;Acc:HGNC:3446]
SPINT1	serine peptidase inhibitor, Kunitz type 1 [Source:HGNC Symbol;Acc:HGNC:11246]
ETV1	ETS variant 1 [Source:HGNC Symbol;Acc:HGNC:3490]
ETV4	ETS variant 4 [Source:HGNC Symbol;Acc:HGNC:3493]
ETV5	ETS variant 5 [Source:HGNC Symbol;Acc:HGNC:3494]
SLC45A3	solute carrier family 45 member 3 [Source:HGNC Symbol;Acc:HGNC:8642]
DDX5	DEAD-box helicase 5 [Source:HGNC Symbol;Acc:HGNC:2746]
MEN1	menin 1 [Source:HGNC Symbol;Acc:HGNC:7010]
EWSR1	EWS RNA binding protein 1 [Source:HGNC Symbol;Acc:HGNC:3508]
FLI1	Fli-1 proto-oncogene, ETS transcription factor [Source:HGNC Symbol;Acc:HGNC:3749]
FEV	FEV, ETS transcription factor [Source:HGNC Symbol;Acc:HGNC:18562]
WT1	Wilms tumor 1 [Source:HGNC Symbol;Acc:HGNC:12796]
BAIAP3	BAI1 associated protein 3 [Source:HGNC Symbol;Acc:HGNC:948]
TSPAN7	tetraspanin 7 [Source:HGNC Symbol;Acc:HGNC:11854]
MLF1	myeloid leukemia factor 1 [Source:HGNC Symbol;Acc:HGNC:7125]
NR4A3	nuclear receptor subfamily 4 group A member 3 [Source:HGNC Symbol;Acc:HGNC:7982]
TAF15	TATA-box binding protein associated factor 15 [Source:HGNC Symbol;Acc:HGNC:11547]
FUS	FUS RNA binding protein [Source:HGNC Symbol;Acc:HGNC:4010]
NFKBIZ	NFKB inhibitor zeta [Source:HGNC Symbol;Acc:HGNC:29805]
PAX7	paired box 7 [Source:HGNC Symbol;Acc:HGNC:8621]
PAX3	paired box 3 [Source:HGNC Symbol;Acc:HGNC:8617]
SS18	SS18, nBAF chromatin remodeling complex subunit [Source:HGNC Symbol;Acc:HGNC:11340]
SSX1	SSX family member 1 [Source:HGNC Symbol;Acc:HGNC:11335]
SSX2	SSX family member 2 [Source:HGNC Symbol;Acc:HGNC:11336]
SSX2B	SSX family member 2B [Source:HGNC Symbol;Acc:HGNC:22263]
NUPR1	nuclear protein 1, transcriptional regulator [Source:HGNC Symbol;Acc:HGNC:29990]
ASPSCR1	ASPSCR1, UBX domain containing tether for SLC2A4 [Source:HGNC Symbol;Acc:HGNC:13825]
TRAF3IP2	TRAF3 interacting protein 2 [Source:HGNC Symbol;Acc:HGNC:1343]
FOSB	FosB proto-oncogene, AP-1 transcription factor subunit [Source:HGNC Symbol;Acc:HGNC:3797]
MAPK6	mitogen-activated protein kinase 6 [Source:HGNC Symbol;Acc:HGNC:6879]
MAPK15	mitogen-activated protein kinase 15 [Source:HGNC Symbol;Acc:HGNC:24667]
USP25	ubiquitin specific peptidase 25 [Source:HGNC Symbol;Acc:HGNC:12624]
SRSF1	serine and arginine rich splicing factor 1 [Source:HGNC Symbol;Acc:HGNC:10780]
DEFB4A	defensin beta 4A [Source:HGNC Symbol;Acc:HGNC:2767]

DEFB4B	defensin beta 4B [Source:HGNC Symbol;Acc:HGNC:30193]
MUC5AC	mucin 5AC, oligomeric mucus/gel-forming [Source:HGNC Symbol;Acc:HGNC:7515]
MUC5B	mucin 5B, oligomeric mucus/gel-forming [Source:HGNC Symbol;Acc:HGNC:7516]
S100A7	S100 calcium binding protein A7 [Source:HGNC Symbol;Acc:HGNC:10497]
S100A7A	S100 calcium binding protein A7A [Source:HGNC Symbol;Acc:HGNC:21657]
S100A8	S100 calcium binding protein A8 [Source:HGNC Symbol;Acc:HGNC:10498]
S100A9	S100 calcium binding protein A9 [Source:HGNC Symbol;Acc:HGNC:10499]
LCN2	lipocalin 2 [Source:HGNC Symbol;Acc:HGNC:6526]
MMP13	matrix metallopeptidase 13 [Source:HGNC Symbol;Acc:HGNC:7159]
RBCK1	RANBP2-type and C3HC4-type zinc finger containing 1 [Source:HGNC Symbol;Acc:HGNC:15864]
RNF31	ring finger protein 31 [Source:HGNC Symbol;Acc:HGNC:16031]
SHARPIN	SHANK associated RH domain interactor [Source:HGNC Symbol;Acc:HGNC:25321]
SPATA2L	spermatogenesis associated 2 like [Source:HGNC Symbol;Acc:HGNC:28393]
SPATA2	spermatogenesis associated 2 [Source:HGNC Symbol;Acc:HGNC:14681]
GLUD2	glutamate dehydrogenase 2 [Source:HGNC Symbol;Acc:HGNC:4336]
GLUD1	glutamate dehydrogenase 1 [Source:HGNC Symbol;Acc:HGNC:4335]
GLUL	glutamate-ammonia ligase [Source:HGNC Symbol;Acc:HGNC:4341]
FTH1	ferritin heavy chain 1 [Source:HGNC Symbol;Acc:HGNC:3976]
FTL	ferritin, light polypeptide [Source:HGNC Symbol;Acc:HGNC:3999]
CHMP2A	charged multivesicular body protein 2A [Source:HGNC Symbol;Acc:HGNC:30216]
CHMP2B	charged multivesicular body protein 2B [Source:HGNC Symbol;Acc:HGNC:24537]
CHMP3	charged multivesicular body protein 3 [Source:HGNC Symbol;Acc:HGNC:29865]
RNF103-CHMP3	RNF103-CHMP3 readthrough [Source:HGNC Symbol;Acc:HGNC:38847]
CHMP4B	charged multivesicular body protein 4B [Source:HGNC Symbol;Acc:HGNC:16171]
CHMP4A	charged multivesicular body protein 4A [Source:HGNC Symbol;Acc:HGNC:20274]
CHMP4C	charged multivesicular body protein 4C [Source:HGNC Symbol;Acc:HGNC:30599]
CHMP6	charged multivesicular body protein 6 [Source:HGNC Symbol;Acc:HGNC:25675]
VPS4B	vacuolar protein sorting 4 homolog B [Source:HGNC Symbol;Acc:HGNC:10895]
VPS4A	vacuolar protein sorting 4 homolog A [Source:HGNC Symbol;Acc:HGNC:13488]
CHMP1B	charged multivesicular body protein 1B [Source:HGNC Symbol;Acc:HGNC:24287]
CHMP1A	charged multivesicular body protein 1A [Source:HGNC Symbol;Acc:HGNC:8740]
CHMP5	charged multivesicular body protein 5 [Source:HGNC Symbol;Acc:HGNC:26942]
CHMP7	charged multivesicular body protein 7 [Source:HGNC Symbol;Acc:HGNC:28439]
TRPM7	transient receptor potential cation channel subfamily M member 7 [Source:HGNC Symbol;Acc:HGNC:17994]
FAF1	Fas associated factor 1 [Source:HGNC Symbol;Acc:HGNC:3578]
EIF2AK2	eukaryotic translation initiation factor 2 alpha kinase 2 [Source:HGNC Symbol;Acc:HGNC:9437]
ZBP1	Z-DNA binding protein 1 [Source:HGNC Symbol;Acc:HGNC:16176]
USP21	ubiquitin specific peptidase 21 [Source:HGNC Symbol;Acc:HGNC:12620]
H2AFX	H2A histone family member X [Source:HGNC Symbol;Acc:HGNC:4739]
H2AFY2	H2A histone family member Y2 [Source:HGNC Symbol;Acc:HGNC:14453]

PPIA	peptidylprolyl isomerase A [Source:HGNC Symbol;Acc:HGNC:9253]
KIR2DL4	killer cell immunoglobulin like receptor, two Ig domains and long cytoplasmic tail 4 [Source:HGNC Symbol;Acc:HGNC:6332]
MYBL2	MYB proto-oncogene like 2 [Source:HGNC Symbol;Acc:HGNC:7548]
LIN9	lin-9 DREAM MuvB core complex component [Source:HGNC Symbol;Acc:HGNC:30830]
LIN37	lin-37 DREAM MuvB core complex component [Source:HGNC Symbol;Acc:HGNC:33234]
LIN52	lin-52 DREAM MuvB core complex component [Source:HGNC Symbol;Acc:HGNC:19856]
LIN54	lin-54 DREAM MuvB core complex component [Source:HGNC Symbol;Acc:HGNC:25397]
RBBP4	RB binding protein 4, chromatin remodeling factor [Source:HGNC Symbol;Acc:HGNC:9887]
FOXM1	forkhead box M1 [Source:HGNC Symbol;Acc:HGNC:3818]
HIPK3	homeodomain interacting protein kinase 3 [Source:HGNC Symbol;Acc:HGNC:4915]
HIPK1	homeodomain interacting protein kinase 1 [Source:HGNC Symbol;Acc:HGNC:19006]
HIPK2	homeodomain interacting protein kinase 2 [Source:HGNC Symbol;Acc:HGNC:14402]
HIPK4	homeodomain interacting protein kinase 4 [Source:HGNC Symbol;Acc:HGNC:19007]
MRE11A	MRE11 homolog A, double strand break repair nuclease [Source:HGNC Symbol;Acc:HGNC:7230]
RAD50	RAD50 double strand break repair protein [Source:HGNC Symbol;Acc:HGNC:9816]
NBN	nibrin [Source:HGNC Symbol;Acc:HGNC:7652]
RAD9A	RAD9 checkpoint clamp component A [Source:HGNC Symbol;Acc:HGNC:9827]
RAD9B	RAD9 checkpoint clamp component B [Source:HGNC Symbol;Acc:HGNC:21700]
RAD1	RAD1 checkpoint DNA exonuclease [Source:HGNC Symbol;Acc:HGNC:9806]
HUS1	HUS1 checkpoint clamp component [Source:HGNC Symbol;Acc:HGNC:5309]
ZFP36L1	ZFP36 ring finger protein-like 1 [Source:HGNC Symbol;Acc:HGNC:1107]
ZFP36L2	ZFP36 ring finger protein-like 2 [Source:HGNC Symbol;Acc:HGNC:1108]
TRPV4	transient receptor potential cation channel subfamily V member 4 [Source:HGNC Symbol;Acc:HGNC:18083]
HCK	HCK proto-oncogene, Src family tyrosine kinase [Source:HGNC Symbol;Acc:HGNC:4840]
FGR	FGR proto-oncogene, Src family tyrosine kinase [Source:HGNC Symbol;Acc:HGNC:3697]
GSK3A	glycogen synthase kinase 3 alpha [Source:HGNC Symbol;Acc:HGNC:4616]
PREX1	phosphatidylinositol-3,4,5-trisphosphate dependent Rac exchange factor 1 [Source:HGNC Symbol;Acc:HGNC:32594]
ELMO1	engulfment and cell motility 1 [Source:HGNC Symbol;Acc:HGNC:16286]
DOCK2	dedicator of cytokinesis 2 [Source:HGNC Symbol;Acc:HGNC:2988]
GRK7	G protein-coupled receptor kinase 7 [Source:HGNC Symbol;Acc:HGNC:17031]
GRK1	G protein-coupled receptor kinase 1 [Source:HGNC Symbol;Acc:HGNC:10013]
GRK4	G protein-coupled receptor kinase 4 [Source:HGNC Symbol;Acc:HGNC:4543]
GRK5	G protein-coupled receptor kinase 5 [Source:HGNC Symbol;Acc:HGNC:4544]
GRK6	G protein-coupled receptor kinase 6 [Source:HGNC Symbol;Acc:HGNC:4545]
ANK1	ankyrin 1 [Source:HGNC Symbol;Acc:HGNC:492]
ANK2	ankyrin 2, neuronal [Source:HGNC Symbol;Acc:HGNC:493]
ANK3	ankyrin 3, node of Ranvier (ankyrin G) [Source:HGNC Symbol;Acc:HGNC:494]
DROSHA	drosha ribonuclease III [Source:HGNC Symbol;Acc:HGNC:17904]
TWIST2	twist family bHLH transcription factor 2 [Source:HGNC Symbol;Acc:HGNC:20670]

HOXD10	homeobox D10 [Source:HGNC Symbol;Acc:HGNC:5133]
PDCD4	programmed cell death 4 (neoplastic transformation inhibitor) [Source:HGNC Symbol;Acc:HGNC:8763]
TFAP4	transcription factor AP-4 (activating enhancer binding protein 4) [Source:HGNC Symbol;Acc:HGNC:11745]
TIMP3	TIMP metallopeptidase inhibitor 3 [Source:HGNC Symbol;Acc:HGNC:11822]
HPSE	heparanase [Source:HGNC Symbol;Acc:HGNC:5164]
HPSE2	heparanase 2 (inactive) [Source:HGNC Symbol;Acc:HGNC:18374]
FRS2	fibroblast growth factor receptor substrate 2 [Source:HGNC Symbol;Acc:HGNC:16971]
NUDT16L1	nudix hydrolase 16 like 1 [Source:HGNC Symbol;Acc:HGNC:28154]
HBEGF	heparin binding EGF like growth factor [Source:HGNC Symbol;Acc:HGNC:3059]
GPC3	glypican 3 [Source:HGNC Symbol;Acc:HGNC:4451]
HSPB2	heat shock protein family B (small) member 2 [Source:HGNC Symbol;Acc:HGNC:5247]
SLC5A7	solute carrier family 5 member 7 [Source:HGNC Symbol;Acc:HGNC:14025]
SLC44A1	solute carrier family 44 member 1 [Source:HGNC Symbol;Acc:HGNC:18798]
SLC44A4	solute carrier family 44 member 4 [Source:HGNC Symbol;Acc:HGNC:13941]
SLC44A5	solute carrier family 44 member 5 [Source:HGNC Symbol;Acc:HGNC:28524]
SLC44A2	solute carrier family 44 member 2 [Source:HGNC Symbol;Acc:HGNC:17292]
SLC44A3	solute carrier family 44 member 3 [Source:HGNC Symbol;Acc:HGNC:28689]
SLC22A2	solute carrier family 22 member 2 [Source:HGNC Symbol;Acc:HGNC:10966]
SLC22A3	solute carrier family 22 member 3 [Source:HGNC Symbol;Acc:HGNC:10967]
SLC22A5	solute carrier family 22 member 5 [Source:HGNC Symbol;Acc:HGNC:10969]
SLC22A4	solute carrier family 22 member 4 [Source:HGNC Symbol;Acc:HGNC:10968]
CHKA	choline kinase alpha [Source:HGNC Symbol;Acc:HGNC:1937]
CHKB	choline kinase beta [Source:HGNC Symbol;Acc:HGNC:1938]
PCYT1B	phosphate cytidylyltransferase 1, choline, beta [Source:HGNC Symbol;Acc:HGNC:8755]
PCYT1A	phosphate cytidylyltransferase 1, choline, alpha [Source:HGNC Symbol;Acc:HGNC:8754]
CHPT1	choline phosphotransferase 1 [Source:HGNC Symbol;Acc:HGNC:17852]
LYPLA1	lysophospholipase I [Source:HGNC Symbol;Acc:HGNC:6737]
GPCPD1	glycerophosphocholine phosphodiesterase 1 [Source:HGNC Symbol;Acc:HGNC:26957]
SLC7A11	solute carrier family 7 member 11 [Source:HGNC Symbol;Acc:HGNC:11059]
GCLC	glutamate-cysteine ligase catalytic subunit [Source:HGNC Symbol;Acc:HGNC:4311]
GCLM	glutamate-cysteine ligase modifier subunit [Source:HGNC Symbol;Acc:HGNC:4312]
GSS	glutathione synthetase [Source:HGNC Symbol;Acc:HGNC:4624]
GPX4	glutathione peroxidase 4 [Source:HGNC Symbol;Acc:HGNC:4556]
LPCAT3	lysophosphatidylcholine acyltransferase 3 [Source:HGNC Symbol;Acc:HGNC:30244]
SAT2	spermidine/spermine N1-acetyltransferase family member 2 [Source:HGNC Symbol;Acc:HGNC:23160]
SAT1	spermidine/spermine N1-acetyltransferase 1 [Source:HGNC Symbol;Acc:HGNC:10540]
SLC39A8	solute carrier family 39 member 8 [Source:HGNC Symbol;Acc:HGNC:20862]
SLC39A14	solute carrier family 39 member 14 [Source:HGNC Symbol;Acc:HGNC:20858]
PCBP2	poly(rC) binding protein 2 [Source:HGNC Symbol;Acc:HGNC:8648]

SLC40A1	solute carrier family 40 member 1 [Source:HGNC Symbol;Acc:HGNC:10909]
CP	ceruloplasmin [Source:HGNC Symbol;Acc:HGNC:2295]
PCBP1	poly(rC) binding protein 1 [Source:HGNC Symbol;Acc:HGNC:8647]
PRNP	prion protein [Source:HGNC Symbol;Acc:HGNC:9449]
FTMT	ferritin mitochondrial [Source:HGNC Symbol;Acc:HGNC:17345]
TNFAIP6	TNF alpha induced protein 6 [Source:HGNC Symbol;Acc:HGNC:11898]
ENG	endoglin [Source:HGNC Symbol;Acc:HGNC:3349]
MDK	midkine (neurite growth-promoting factor 2) [Source:HGNC Symbol;Acc:HGNC:6972]
UBD	ubiquitin D [Source:HGNC Symbol;Acc:HGNC:18795]
KLK11	kallikrein related peptidase 11 [Source:HGNC Symbol;Acc:HGNC:6359]
CD69	CD69 molecule [Source:HGNC Symbol;Acc:HGNC:1694]
GBP5	guanylate binding protein 5 [Source:HGNC Symbol;Acc:HGNC:19895]
GNLY	granulysin [Source:HGNC Symbol;Acc:HGNC:4414]
ANPEP	alanyl aminopeptidase, membrane [Source:HGNC Symbol;Acc:HGNC:500]
IL4I1	interleukin 4 induced 1 [Source:HGNC Symbol;Acc:HGNC:19094]
EIF4A1	eukaryotic translation initiation factor 4A1 [Source:HGNC Symbol;Acc:HGNC:3282]
ELF4	E74 like ETS transcription factor 4 [Source:HGNC Symbol;Acc:HGNC:3319]
MAP3K7CL	MAP3K7 C-terminal like [Source:HGNC Symbol;Acc:HGNC:16457]
MINK1	misshapen like kinase 1 [Source:HGNC Symbol;Acc:HGNC:17565]
HAS1	hyaluronan synthase 1 [Source:HGNC Symbol;Acc:HGNC:4818]
TIPARP	TCDD inducible poly(ADP-ribose) polymerase [Source:HGNC Symbol;Acc:HGNC:23696]
DICER1	dicer 1, ribonuclease III [Source:HGNC Symbol;Acc:HGNC:17098]
SP7	Sp7 transcription factor [Source:HGNC Symbol;Acc:HGNC:17321]
SLIT3	slit guidance ligand 3 [Source:HGNC Symbol;Acc:HGNC:11087]
THY1	Thy-1 cell surface antigen [Source:HGNC Symbol;Acc:HGNC:11801]
ADK	adenosine kinase [Source:HGNC Symbol;Acc:HGNC:257]
ROS1	ROS proto-oncogene 1, receptor tyrosine kinase [Source:HGNC Symbol;Acc:HGNC:10261]
DDOST	dolichyl-diphosphooligosaccharide--protein glycosyltransferase non-catalytic subunit [Source:HGNC Symbol;Acc:HGNC:2728]
TNIK	TRAF2 and NCK interacting kinase [Source:HGNC Symbol;Acc:HGNC:30765]
ZIC1	Zic family member 1 [Source:HGNC Symbol;Acc:HGNC:12872]
NR2E1	nuclear receptor subfamily 2 group E member 1 [Source:HGNC Symbol;Acc:HGNC:7973]
LHX2	LIM homeobox 2 [Source:HGNC Symbol;Acc:HGNC:6594]
NR2F1	nuclear receptor subfamily 2 group F member 1 [Source:HGNC Symbol;Acc:HGNC:7975]
MYO10	myosin X [Source:HGNC Symbol;Acc:HGNC:7593]
CLIC5	chloride intracellular channel 5 [Source:HGNC Symbol;Acc:HGNC:13517]
SOX8	SRY-box 8 [Source:HGNC Symbol;Acc:HGNC:11203]
OLIG1	oligodendrocyte transcription factor 1 [Source:HGNC Symbol;Acc:HGNC:16983]
RHOC	ras homolog family member C [Source:HGNC Symbol;Acc:HGNC:669]
NCAN	neurocan [Source:HGNC Symbol;Acc:HGNC:2465]
PTPRZ1	protein tyrosine phosphatase, receptor type Z1 [Source:HGNC Symbol;Acc:HGNC:9685]
CIT	citron rho-interacting serine/threonine kinase [Source:HGNC Symbol;Acc:HGNC:1985]

SLC1A3	solute carrier family 1 member 3 [Source:HGNC Symbol;Acc:HGNC:10941]
OMG	oligodendrocyte myelin glycoprotein [Source:HGNC Symbol;Acc:HGNC:8135]
SLITRK2	SLIT and NTRK like family member 2 [Source:HGNC Symbol;Acc:HGNC:13449]
LGI4	leucine rich repeat LGI family member 4 [Source:HGNC Symbol;Acc:HGNC:18712]
GRIA1	glutamate ionotropic receptor AMPA type subunit 1 [Source:HGNC Symbol;Acc:HGNC:4571]
CALB1	calbindin 1 [Source:HGNC Symbol;Acc:HGNC:1434]
CCK	cholecystokinin [Source:HGNC Symbol;Acc:HGNC:1569]
DBI	diazepam binding inhibitor, acyl-CoA binding protein [Source:HGNC Symbol;Acc:HGNC:2690]
KCNJ10	potassium voltage-gated channel subfamily J member 10 [Source:HGNC Symbol;Acc:HGNC:6256]
KCNJ16	potassium voltage-gated channel subfamily J member 16 [Source:HGNC Symbol;Acc:HGNC:6262]
METRN	meteorin, glial cell differentiation regulator [Source:HGNC Symbol;Acc:HGNC:14151]
METRNL	meteorin, glial cell differentiation regulator-like [Source:HGNC Symbol;Acc:HGNC:27584]
GNAT2	G protein subunit alpha transducin 2 [Source:HGNC Symbol;Acc:HGNC:4394]
IMPG2	interphotoreceptor matrix proteoglycan 2 [Source:HGNC Symbol;Acc:HGNC:18362]
CNGA1	cyclic nucleotide gated channel alpha 1 [Source:HGNC Symbol;Acc:HGNC:2148]
PLEKHB1	pleckstrin homology domain containing B1 [Source:HGNC Symbol;Acc:HGNC:19079]
CRX	cone-rod homeobox [Source:HGNC Symbol;Acc:HGNC:2383]
AIPL1	aryl hydrocarbon receptor interacting protein like 1 [Source:HGNC Symbol;Acc:HGNC:359]
SLC24A1	solute carrier family 24 member 1 [Source:HGNC Symbol;Acc:HGNC:10975]
FSCN2	fascin actin-bundling protein 2, retinal [Source:HGNC Symbol;Acc:HGNC:3960]
ROM1	retinal outer segment membrane protein 1 [Source:HGNC Symbol;Acc:HGNC:10254]
OPN1LW	opsin 1 (cone pigments), long-wave-sensitive [Source:HGNC Symbol;Acc:HGNC:9936]
OPN1MW	opsin 1 (cone pigments), medium-wave-sensitive [Source:HGNC Symbol;Acc:HGNC:4206]
TULP1	tubby like protein 1 [Source:HGNC Symbol;Acc:HGNC:12423]
RP1L1	retinitis pigmentosa 1-like 1 [Source:HGNC Symbol;Acc:HGNC:15946]
GUCA1A	guanylate cyclase activator 1A [Source:HGNC Symbol;Acc:HGNC:4678]
PRPH2	peripherin 2 [Source:HGNC Symbol;Acc:HGNC:9942]
CNGB1	cyclic nucleotide gated channel beta 1 [Source:HGNC Symbol;Acc:HGNC:2151]
CD9	CD9 molecule [Source:HGNC Symbol;Acc:HGNC:1709]
AURKA	aurora kinase A [Source:HGNC Symbol;Acc:HGNC:11393]
ENTPD1	ectonucleoside triphosphate diphosphohydrolase 1 [Source:HGNC Symbol;Acc:HGNC:3363]
ARID1A	AT-rich interaction domain 1A [Source:HGNC Symbol;Acc:HGNC:11110]
HNF1A	HNF1 homeobox A [Source:HGNC Symbol;Acc:HGNC:11621]
TOX	thymocyte selection associated high mobility group box [Source:HGNC Symbol;Acc:HGNC:18988]
LRRC23	leucine rich repeat containing 23 [Source:HGNC Symbol;Acc:HGNC:19138]
CLC	Charcot-Leyden crystal galectin [Source:HGNC Symbol;Acc:HGNC:2014]
MICA	MHC class I polypeptide-related sequence A [Source:HGNC Symbol;Acc:HGNC:7090]
KLRK1	killer cell lectin like receptor K1 [Source:HGNC Symbol;Acc:HGNC:18788]
NCR3	natural cytotoxicity triggering receptor 3 [Source:HGNC Symbol;Acc:HGNC:19077]

LAG3	lymphocyte activating 3 [Source:HGNC Symbol;Acc:HGNC:6476]
PROK2	prokineticin 2 [Source:HGNC Symbol;Acc:HGNC:18455]
ABO	ABO blood group (transferase A, alpha 1-3-N-acetylgalactosaminyltransferase; transferase B, alpha 1-3-galactosyltransferase) [Source:HGNC Symbol;Acc:HGNC:79]
BTLA	B and T lymphocyte associated [Source:HGNC Symbol;Acc:HGNC:21087]
ABCB7	ATP binding cassette subfamily B member 7 [Source:HGNC Symbol;Acc:HGNC:48]
SERPINA3	serpin family A member 3 [Source:HGNC Symbol;Acc:HGNC:16]
FOXP3	forkhead box P3 [Source:HGNC Symbol;Acc:HGNC:6106]
B2M	beta-2-microglobulin [Source:HGNC Symbol;Acc:HGNC:914]
ZFP36	ZFP36 ring finger protein [Source:HGNC Symbol;Acc:HGNC:12862]
SLPI	secretory leukocyte peptidase inhibitor [Source:HGNC Symbol;Acc:HGNC:11092]
IGFBP2	insulin like growth factor binding protein 2 [Source:HGNC Symbol;Acc:HGNC:5471]
FCGBP	Fc fragment of IgG binding protein [Source:HGNC Symbol;Acc:HGNC:13572]
TSPEAR	thrombospondin type laminin G domain and EAR repeats [Source:HGNC Symbol;Acc:HGNC:1268]
MUC1	mucin 1, cell surface associated [Source:HGNC Symbol;Acc:HGNC:7508]
KISS1	KiSS-1 metastasis-suppressor [Source:HGNC Symbol;Acc:HGNC:6341]
ADGRG1	adhesion G protein-coupled receptor G1 [Source:HGNC Symbol;Acc:HGNC:4512]
PTHLH	parathyroid hormone-like hormone [Source:HGNC Symbol;Acc:HGNC:9607]
BRMS1	breast cancer metastasis suppressor 1 [Source:HGNC Symbol;Acc:HGNC:17262]
NME1	NME/NM23 nucleoside diphosphate kinase 1 [Source:HGNC Symbol;Acc:HGNC:7849]
TINAGL1	tubulointerstitial nephritis antigen like 1 [Source:HGNC Symbol;Acc:HGNC:19168]
ESYT3	extended synaptotagmin 3 [Source:HGNC Symbol;Acc:HGNC:24295]
FKBP10	FK506 binding protein 10 [Source:HGNC Symbol;Acc:HGNC:18169]
NEDD9	neural precursor cell expressed, developmentally down-regulated 9 [Source:HGNC Symbol;Acc:HGNC:7733]
TBC1D16	TBC1 domain family member 16 [Source:HGNC Symbol;Acc:HGNC:28356]
AEBP1	AE binding protein 1 [Source:HGNC Symbol;Acc:HGNC:303]
MTA1	metastasis associated 1 [Source:HGNC Symbol;Acc:HGNC:7410]
PNCK	pregnancy up-regulated nonubiquitous CaM kinase [Source:HGNC Symbol;Acc:HGNC:13415]
CENPJ	centromere protein J [Source:HGNC Symbol;Acc:HGNC:17272]
MCAM	melanoma cell adhesion molecule [Source:HGNC Symbol;Acc:HGNC:6934]
BACH1	BTB domain and CNC homolog 1 [Source:HGNC Symbol;Acc:HGNC:935]
MAFF	MAF bZIP transcription factor F [Source:HGNC Symbol;Acc:HGNC:6780]
ACKR1	atypical chemokine receptor 1 (Duffy blood group) [Source:HGNC Symbol;Acc:HGNC:4035]
FRMD4A	FERM domain containing 4A [Source:HGNC Symbol;Acc:HGNC:25491]
CD24	CD24 molecule [Source:HGNC Symbol;Acc:HGNC:1645]
ABCB5	ATP binding cassette subfamily B member 5 [Source:HGNC Symbol;Acc:HGNC:46]
SOX4	SRY-box 4 [Source:HGNC Symbol;Acc:HGNC:11200]
EPCAM	epithelial cell adhesion molecule [Source:HGNC Symbol;Acc:HGNC:11529]
LINGO2	leucine rich repeat and Ig domain containing 2 [Source:HGNC Symbol;Acc:HGNC:21207]
CEACAM8	carcinoembryonic antigen related cell adhesion molecule 8 [Source:HGNC Symbol;Acc:HGNC:1820]

NELFCD	negative elongation factor complex member C/D [Source:HGNC Symbol;Acc:HGNC:15934]
HDAC3	histone deacetylase 3 [Source:HGNC Symbol;Acc:HGNC:4854]
HDAC9	histone deacetylase 9 [Source:HGNC Symbol;Acc:HGNC:14065]
LOXL2	lysyl oxidase like 2 [Source:HGNC Symbol;Acc:HGNC:6666]
MIF	macrophage migration inhibitory factor (glycosylation-inhibiting factor) [Source:HGNC Symbol;Acc:HGNC:7097]
EPHA1	EPH receptor A1 [Source:HGNC Symbol;Acc:HGNC:3385]
CDK5	cyclin dependent kinase 5 [Source:HGNC Symbol;Acc:HGNC:1774]
SEMA4D	semaphorin 4D [Source:HGNC Symbol;Acc:HGNC:10732]
MACC1	metastasis associated in colon cancer 1 [Source:HGNC Symbol;Acc:HGNC:30215]
WWOX	WW domain containing oxidoreductase [Source:HGNC Symbol;Acc:HGNC:12799]
EPHA3	EPH receptor A3 [Source:HGNC Symbol;Acc:HGNC:3387]
NTS	neurotensin [Source:HGNC Symbol;Acc:HGNC:8038]
TM4SF5	transmembrane 4 L six family member 5 [Source:HGNC Symbol;Acc:HGNC:11857]
BCL9	B-cell CLL/lymphoma 9 [Source:HGNC Symbol;Acc:HGNC:1008]
NPY4R	neuropeptide Y receptor Y4 [Source:HGNC Symbol;Acc:HGNC:9329]
LOXL1	lysyl oxidase like 1 [Source:HGNC Symbol;Acc:HGNC:6665]
XAF1	XIAP associated factor 1 [Source:HGNC Symbol;Acc:HGNC:30932]
CAMP	cathelicidin antimicrobial peptide [Source:HGNC Symbol;Acc:HGNC:1472]
EPHB2	EPH receptor B2 [Source:HGNC Symbol;Acc:HGNC:3393]
TIMP2	TIMP metallopeptidase inhibitor 2 [Source:HGNC Symbol;Acc:HGNC:11821]
PPP1R15A	protein phosphatase 1 regulatory subunit 15A [Source:HGNC Symbol;Acc:HGNC:14375]
IRF6	interferon regulatory factor 6 [Source:HGNC Symbol;Acc:HGNC:6121]
HYOU1	hypoxia up-regulated 1 [Source:HGNC Symbol;Acc:HGNC:16931]
MBTPS1	membrane bound transcription factor peptidase, site 1 [Source:HGNC Symbol;Acc:HGNC:15456]
MBTPS2	membrane bound transcription factor peptidase, site 2 [Source:HGNC Symbol;Acc:HGNC:15455]
RPAP2	RNA polymerase II associated protein 2 [Source:HGNC Symbol;Acc:HGNC:25791]
T	T brachyury transcription factor [Source:HGNC Symbol;Acc:HGNC:11515]
DNAJC3	DnaJ heat shock protein family (Hsp40) member C3 [Source:HGNC Symbol;Acc:HGNC:9439]
INSIG1	insulin induced gene 1 [Source:HGNC Symbol;Acc:HGNC:6083]
SCAP	SREBF chaperone [Source:HGNC Symbol;Acc:HGNC:30634]
HMGCS1	3-hydroxy-3-methylglutaryl-CoA synthase 1 [Source:HGNC Symbol;Acc:HGNC:5007]
EZH2	enhancer of zeste 2 polycomb repressive complex 2 subunit [Source:HGNC Symbol;Acc:HGNC:3527]
DNAJA1	DnaJ heat shock protein family (Hsp40) member A1 [Source:HGNC Symbol;Acc:HGNC:5229]
SEC61A1	Sec61 translocon alpha 1 subunit [Source:HGNC Symbol;Acc:HGNC:18276]
EIF2B1	eukaryotic translation initiation factor 2B subunit alpha [Source:HGNC Symbol;Acc:HGNC:3257]
LMOD1	leiomodin 1 [Source:HGNC Symbol;Acc:HGNC:6647]
SEC23A	Sec23 homolog A, coat complex II component [Source:HGNC Symbol;Acc:HGNC:10701]
EIF5	eukaryotic translation initiation factor 5 [Source:HGNC Symbol;Acc:HGNC:3299]
EDEM1	ER degradation enhancing alpha-mannosidase like protein 1 [Source:HGNC Symbol;Acc:HGNC:18967]

EIF2S2	eukaryotic translation initiation factor 2 subunit beta [Source:HGNC Symbol;Acc:HGNC:3266]
EIF3C	eukaryotic translation initiation factor 3 subunit C [Source:HGNC Symbol;Acc:HGNC:3279]
EIF4G2	eukaryotic translation initiation factor 4 gamma 2 [Source:HGNC Symbol;Acc:HGNC:3297]
ERN2	endoplasmic reticulum to nucleus signaling 2 [Source:HGNC Symbol;Acc:HGNC:16942]
DNAJB6	DnaJ heat shock protein family (Hsp40) member B6 [Source:HGNC Symbol;Acc:HGNC:14888]
DNAJA2	DnaJ heat shock protein family (Hsp40) member A2 [Source:HGNC Symbol;Acc:HGNC:14884]
DNAJC14	DnaJ heat shock protein family (Hsp40) member C14 [Source:HGNC Symbol;Acc:HGNC:24581]
SLC6A9	solute carrier family 6 member 9 [Source:HGNC Symbol;Acc:HGNC:11056]
TRIB3	tribbles pseudokinase 3 [Source:HGNC Symbol;Acc:HGNC:16228]
ABCD1	ATP binding cassette subfamily D member 1 [Source:HGNC Symbol;Acc:HGNC:61]
ALPI	alkaline phosphatase, intestinal [Source:HGNC Symbol;Acc:HGNC:437]
BGLAP	bone gamma-carboxyglutamate protein [Source:HGNC Symbol;Acc:HGNC:1043]
CEACAM1	carcinoembryonic antigen related cell adhesion molecule 1 [Source:HGNC Symbol;Acc:HGNC:1814]
PRDM1	PR domain 1 [Source:HGNC Symbol;Acc:HGNC:9346]
S100G	S100 calcium binding protein G [Source:HGNC Symbol;Acc:HGNC:1436]
CASP5	caspase 5 [Source:HGNC Symbol;Acc:HGNC:1506]
CCNC	cyclin C [Source:HGNC Symbol;Acc:HGNC:1581]
ADGRE5	adhesion G protein-coupled receptor E5 [Source:HGNC Symbol;Acc:HGNC:1711]
CDX2	caudal type homeobox 2 [Source:HGNC Symbol;Acc:HGNC:1806]
COL13A1	collagen type XIII alpha 1 chain [Source:HGNC Symbol;Acc:HGNC:2190]
CST1	cystatin SN [Source:HGNC Symbol;Acc:HGNC:2473]
CST6	cystatin E/M [Source:HGNC Symbol;Acc:HGNC:2478]
SERPINB1	serpin family B member 1 [Source:HGNC Symbol;Acc:HGNC:3311]
EPHB4	EPH receptor B4 [Source:HGNC Symbol;Acc:HGNC:3395]
IRF8	interferon regulatory factor 8 [Source:HGNC Symbol;Acc:HGNC:5358]
IGFBP1	insulin like growth factor binding protein 1 [Source:HGNC Symbol;Acc:HGNC:5469]
IGFBP5	insulin like growth factor binding protein 5 [Source:HGNC Symbol;Acc:HGNC:5474]
IRF4	interferon regulatory factor 4 [Source:HGNC Symbol;Acc:HGNC:6119]
KRT13	keratin 13 [Source:HGNC Symbol;Acc:HGNC:6415]
KRT16	keratin 16 [Source:HGNC Symbol;Acc:HGNC:6423]
KRT34	keratin 34 [Source:HGNC Symbol;Acc:HGNC:6452]
LGALS9	galectin 9 [Source:HGNC Symbol;Acc:HGNC:6570]
MXD1	MAX dimerization protein 1 [Source:HGNC Symbol;Acc:HGNC:6761]
CD200	CD200 molecule [Source:HGNC Symbol;Acc:HGNC:7203]
MX2	MX dynamin like GTPase 2 [Source:HGNC Symbol;Acc:HGNC:7533]
MYO9B	myosin IXB [Source:HGNC Symbol;Acc:HGNC:7609]
NINJ1	ninjurin 1 [Source:HGNC Symbol;Acc:HGNC:7824]
ORM1	orosomucoid 1 [Source:HGNC Symbol;Acc:HGNC:8498]
ORM2	orosomucoid 2 [Source:HGNC Symbol;Acc:HGNC:8499]
PNOC	prepronociceptin [Source:HGNC Symbol;Acc:HGNC:9163]
KLK6	kallikrein related peptidase 6 [Source:HGNC Symbol;Acc:HGNC:6367]

PTH	parathyroid hormone [Source:HGNC Symbol;Acc:HGNC:9606]
S100A2	S100 calcium binding protein A2 [Source:HGNC Symbol;Acc:HGNC:10492]
S100A4	S100 calcium binding protein A4 [Source:HGNC Symbol;Acc:HGNC:10494]
S100A6	S100 calcium binding protein A6 [Source:HGNC Symbol;Acc:HGNC:10496]
SATB1	SATB homeobox 1 [Source:HGNC Symbol;Acc:HGNC:10541]
SPRR1B	small proline rich protein 1B [Source:HGNC Symbol;Acc:HGNC:11260]
SULT1C2	sulfotransferase family 1C member 2 [Source:HGNC Symbol;Acc:HGNC:11456]
TPM1	tropomyosin 1 (alpha) [Source:HGNC Symbol;Acc:HGNC:12010]
SEMA3B	semaphorin 3B [Source:HGNC Symbol;Acc:HGNC:10724]
NRIP1	nuclear receptor interacting protein 1 [Source:HGNC Symbol;Acc:HGNC:8001]
KRT38	keratin 38 [Source:HGNC Symbol;Acc:HGNC:6456]
ASAP2	ArfGAP with SH3 domain, ankyrin repeat and PH domain 2 [Source:HGNC Symbol;Acc:HGNC:2721]
KL	klotho [Source:HGNC Symbol;Acc:HGNC:6344]
ATP2C2	ATPase secretory pathway Ca2+ transporting 2 [Source:HGNC Symbol;Acc:HGNC:29103]
LPGAT1	lysophosphatidylglycerol acyltransferase 1 [Source:HGNC Symbol;Acc:HGNC:28985]
SLC34A2	solute carrier family 34 member 2 [Source:HGNC Symbol;Acc:HGNC:11020]
ADAMTS5	ADAM metallopeptidase with thrombospondin type 1 motif 5 [Source:HGNC Symbol;Acc:HGNC:221]
TRAK1	trafficking kinesin protein 1 [Source:HGNC Symbol;Acc:HGNC:29947]
IGSF9B	immunoglobulin superfamily member 9B [Source:HGNC Symbol;Acc:HGNC:32326]
CLEC16A	C-type lectin domain family 16 member A [Source:HGNC Symbol;Acc:HGNC:29013]
CASP14	caspase 14 [Source:HGNC Symbol;Acc:HGNC:1502]
SOSTDC1	sclerostin domain containing 1 [Source:HGNC Symbol;Acc:HGNC:21748]
LCE2B	late cornified envelope 2B [Source:HGNC Symbol;Acc:HGNC:16610]
CYP2S1	cytochrome P450 family 2 subfamily S member 1 [Source:HGNC Symbol;Acc:HGNC:15654]
HILPDA	hypoxia inducible lipid droplet associated [Source:HGNC Symbol;Acc:HGNC:28859]
G0S2	G0/G1 switch 2 [Source:HGNC Symbol;Acc:HGNC:30229]
TREM1	triggering receptor expressed on myeloid cells 1 [Source:HGNC Symbol;Acc:HGNC:17760]
CDKAL1	CDK5 regulatory subunit associated protein 1 like 1 [Source:HGNC Symbol;Acc:HGNC:21050]
MED9	mediator complex subunit 9 [Source:HGNC Symbol;Acc:HGNC:25487]
TRPV6	transient receptor potential cation channel subfamily V member 6 [Source:HGNC Symbol;Acc:HGNC:14006]
LRRC8A	leucine rich repeat containing 8 family member A [Source:HGNC Symbol;Acc:HGNC:19027]
TRPV5	transient receptor potential cation channel subfamily V member 5 [Source:HGNC Symbol;Acc:HGNC:3145]
SALL4	spalt like transcription factor 4 [Source:HGNC Symbol;Acc:HGNC:15924]
COLEC11	collectin subfamily member 11 [Source:HGNC Symbol;Acc:HGNC:17213]
STEAP4	STEAP4 metalloreductase [Source:HGNC Symbol;Acc:HGNC:21923]
CLMN	calmin (calponin-like, transmembrane) [Source:HGNC Symbol;Acc:HGNC:19972]
CLPTM1L	CLPTM1-like [Source:HGNC Symbol;Acc:HGNC:24308]
CRACR2A	calcium release activated channel regulator 2A [Source:HGNC Symbol;Acc:HGNC:28657]
KRTAP4-1	keratin associated protein 4-1 [Source:HGNC Symbol;Acc:HGNC:18907]

DNER	delta/notch like EGF repeat containing [Source:HGNC Symbol;Acc:HGNC:24456]
KRT71	keratin 71 [Source:HGNC Symbol;Acc:HGNC:28927]
ZNF257	zinc finger protein 257 [Source:HGNC Symbol;Acc:HGNC:13498]
LRRC25	leucine rich repeat containing 25 [Source:HGNC Symbol;Acc:HGNC:29806]
DACT2	dishevelled binding antagonist of beta catenin 2 [Source:HGNC Symbol;Acc:HGNC:21231]
CREG2	cellular repressor of E1A stimulated genes 2 [Source:HGNC Symbol;Acc:HGNC:14272]
SLC37A2	solute carrier family 37 member 2 [Source:HGNC Symbol;Acc:HGNC:20644]
CRACR2B	calcium release activated channel regulator 2B [Source:HGNC Symbol;Acc:HGNC:28703]
KRTAP8-1	keratin associated protein 8-1 [Source:HGNC Symbol;Acc:HGNC:18935]
LCE1D	late cornified envelope 1D [Source:HGNC Symbol;Acc:HGNC:29465]
LCE1F	late cornified envelope 1F [Source:HGNC Symbol;Acc:HGNC:29467]
KRTAP12-2	keratin associated protein 12-2 [Source:HGNC Symbol;Acc:HGNC:20530]
DND1	DND microRNA-mediated repression inhibitor 1 [Source:HGNC Symbol;Acc:HGNC:23799]
KRTAP10-4	keratin associated protein 10-4 [Source:HGNC Symbol;Acc:HGNC:20521]
KRTAP10-7	keratin associated protein 10-7 [Source:HGNC Symbol;Acc:HGNC:22970]
KRTAP10-9	keratin associated protein 10-9 [Source:HGNC Symbol;Acc:HGNC:22971]
KRTAP10-2	keratin associated protein 10-2 [Source:HGNC Symbol;Acc:HGNC:22967]
KRTAP5-1	keratin associated protein 5-1 [Source:HGNC Symbol;Acc:HGNC:23596]
KRTAP5-4	keratin associated protein 5-4 [Source:HGNC Symbol;Acc:HGNC:23599]
DEFB132	defensin beta 132 [Source:HGNC Symbol;Acc:HGNC:33806]
GXYLT2	glucoside xylosyltransferase 2 [Source:HGNC Symbol;Acc:HGNC:33383]
ALPPL2	alkaline phosphatase, placental like 2 [Source:HGNC Symbol;Acc:HGNC:441]
GC	GC, vitamin D binding protein [Source:HGNC Symbol;Acc:HGNC:4187]
MED1	mediator complex subunit 1 [Source:HGNC Symbol;Acc:HGNC:9234]
DBP	D-box binding PAR bZIP transcription factor [Source:HGNC Symbol;Acc:HGNC:2697]
PDIA3	protein disulfide isomerase family A member 3 [Source:HGNC Symbol;Acc:HGNC:4606]
PLAA	phospholipase A2 activating protein [Source:HGNC Symbol;Acc:HGNC:9043]
CALR	calreticulin [Source:HGNC Symbol;Acc:HGNC:1455]
CANX	calnexin [Source:HGNC Symbol;Acc:HGNC:1473]
AHR	aryl hydrocarbon receptor [Source:HGNC Symbol;Acc:HGNC:348]
RORA	RAR related orphan receptor A [Source:HGNC Symbol;Acc:HGNC:10258]
PHEX	phosphate regulating endopeptidase homolog, X-linked [Source:HGNC Symbol;Acc:HGNC:8918]
MZF1	myeloid zinc finger 1 [Source:HGNC Symbol;Acc:HGNC:13108]
CASR	calcium sensing receptor [Source:HGNC Symbol;Acc:HGNC:1514]
WNK4	WNK lysine deficient protein kinase 4 [Source:HGNC Symbol;Acc:HGNC:14544]
TRPV2	transient receptor potential cation channel subfamily V member 2 [Source:HGNC Symbol;Acc:HGNC:18082]
POU1F1	POU class 1 homeobox 1 [Source:HGNC Symbol;Acc:HGNC:9210]
REPIN1	replication initiator 1 [Source:HGNC Symbol;Acc:HGNC:17922]
KCNJ1	potassium voltage-gated channel subfamily J member 1 [Source:HGNC Symbol;Acc:HGNC:6255]
BANF1	barrier to autointegration factor 1 [Source:HGNC Symbol;Acc:HGNC:17397]
BRD7	bromodomain containing 7 [Source:HGNC Symbol;Acc:HGNC:14310]

BRD9	bromodomain containing 9 [Source:HGNC Symbol;Acc:HGNC:25818]
SLC34A1	solute carrier family 34 member 1 [Source:HGNC Symbol;Acc:HGNC:11019]
SLC20A1	solute carrier family 20 member 1 [Source:HGNC Symbol;Acc:HGNC:10946]
SLC20A2	solute carrier family 20 member 2 [Source:HGNC Symbol;Acc:HGNC:10947]
TRPC1	transient receptor potential cation channel subfamily C member 1 [Source:HGNC Symbol;Acc:HGNC:12333]
ALPL	alkaline phosphatase, liver/bone/kidney [Source:HGNC Symbol;Acc:HGNC:438]
CD93	CD93 molecule [Source:HGNC Symbol;Acc:HGNC:15855]
SEMA6B	semaphorin 6B [Source:HGNC Symbol;Acc:HGNC:10739]
SRGN	serglycin [Source:HGNC Symbol;Acc:HGNC:9361]
THEMIS2	thymocyte selection associated family member 2 [Source:HGNC Symbol;Acc:HGNC:16839]
PDLIM4	PDZ and LIM domain 4 [Source:HGNC Symbol;Acc:HGNC:16501]
TMEM37	transmembrane protein 37 [Source:HGNC Symbol;Acc:HGNC:18216]
PDZD7	PDZ domain containing 7 [Source:HGNC Symbol;Acc:HGNC:26257]
PPARGC1B	PPARG coactivator 1 beta [Source:HGNC Symbol;Acc:HGNC:30022]
SMPDL3A	sphingomyelin phosphodiesterase acid like 3A [Source:HGNC Symbol;Acc:HGNC:17389]
DPF3	double PHD fingers 3 [Source:HGNC Symbol;Acc:HGNC:17427]
MYBBP1A	MYB binding protein 1a [Source:HGNC Symbol;Acc:HGNC:7546]
YY1	YY1 transcription factor [Source:HGNC Symbol;Acc:HGNC:12856]
NAT1	N-acetyltransferase 1 (arylamine N-acetyltransferase) [Source:HGNC Symbol;Acc:HGNC:7645]
SLC1A1	solute carrier family 1 member 1 [Source:HGNC Symbol;Acc:HGNC:10939]
CCNJ	cyclin J [Source:HGNC Symbol;Acc:HGNC:23434]
MICB	MHC class I polypeptide-related sequence B [Source:HGNC Symbol;Acc:HGNC:7091]
ULBP2	UL16 binding protein 2 [Source:HGNC Symbol;Acc:HGNC:14894]
KDM3A	lysine demethylase 3A [Source:HGNC Symbol;Acc:HGNC:20815]
NCOA2	nuclear receptor coactivator 2 [Source:HGNC Symbol;Acc:HGNC:7669]
PDLIM2	PDZ and LIM domain 2 [Source:HGNC Symbol;Acc:HGNC:13992]
SMARCA1	SWI/SNF related, matrix associated, actin dependent regulator of chromatin, subfamily a, member 1 [Source:HGNC Symbol;Acc:HGNC:11097]
ARNTL	aryl hydrocarbon receptor nuclear translocator like [Source:HGNC Symbol;Acc:HGNC:701]
SOD1	superoxide dismutase 1, soluble [Source:HGNC Symbol;Acc:HGNC:11179]
TP53BP1	tumor protein p53 binding protein 1 [Source:HGNC Symbol;Acc:HGNC:11999]
DAP3	death associated protein 3 [Source:HGNC Symbol;Acc:HGNC:2673]
DNMT1	DNA (cytosine-5-)-methyltransferase 1 [Source:HGNC Symbol;Acc:HGNC:2976]
FOXK1	forkhead box K1 [Source:HGNC Symbol;Acc:HGNC:23480]
GLIS3	GLIS family zinc finger 3 [Source:HGNC Symbol;Acc:HGNC:28510]
KCNF1	potassium voltage-gated channel modifier subfamily F member 1 [Source:HGNC Symbol;Acc:HGNC:6246]
LTK	leukocyte receptor tyrosine kinase [Source:HGNC Symbol;Acc:HGNC:6721]
NFIA	nuclear factor I A [Source:HGNC Symbol;Acc:HGNC:7784]
POU4F2	POU class 4 homeobox 2 [Source:HGNC Symbol;Acc:HGNC:9219]
PPP1R3G	protein phosphatase 1 regulatory subunit 3G [Source:HGNC Symbol;Acc:HGNC:14945]

REM1	RRAD and GEM like GTPase 1 [Source:HGNC Symbol;Acc:HGNC:15922]
SFXN3	sideroflexin 3 [Source:HGNC Symbol;Acc:HGNC:16087]
SP100	SP100 nuclear antigen [Source:HGNC Symbol;Acc:HGNC:11206]
KCTD12	potassium channel tetramerization domain containing 12 [Source:HGNC Symbol;Acc:HGNC:14678]
ITSN1	intersectin 1 [Source:HGNC Symbol;Acc:HGNC:6183]
TRIM38	tripartite motif containing 38 [Source:HGNC Symbol;Acc:HGNC:10059]
ELL2	elongation factor for RNA polymerase II 2 [Source:HGNC Symbol;Acc:HGNC:17064]
MARCKS	myristoylated alanine rich protein kinase C substrate [Source:HGNC Symbol;Acc:HGNC:6759]
EFL1	elongation factor like GTPase 1 [Source:HGNC Symbol;Acc:HGNC:25789]
SEL1L3	SEL1L family member 3 [Source:HGNC Symbol;Acc:HGNC:29108]
RASSF2	Ras association domain family member 2 [Source:HGNC Symbol;Acc:HGNC:9883]
MS4A7	membrane spanning 4-domains A7 [Source:HGNC Symbol;Acc:HGNC:13378]
MFAP4	microfibrillar associated protein 4 [Source:HGNC Symbol;Acc:HGNC:7035]
MTSS1L	metastasis suppressor 1-like [Source:HGNC Symbol;Acc:HGNC:25094]
ST3GAL4	ST3 beta-galactoside alpha-2,3-sialyltransferase 4 [Source:HGNC Symbol;Acc:HGNC:10864]
SCPEP1	serine carboxypeptidase 1 [Source:HGNC Symbol;Acc:HGNC:29507]
ARHGEF40	Rho guanine nucleotide exchange factor 40 [Source:HGNC Symbol;Acc:HGNC:25516]
CST7	cystatin F [Source:HGNC Symbol;Acc:HGNC:2479]
BIN1	bridging integrator 1 [Source:HGNC Symbol;Acc:HGNC:1052]
CKAP4	cytoskeleton-associated protein 4 [Source:HGNC Symbol;Acc:HGNC:16991]
BEX1	brain expressed X-linked 1 [Source:HGNC Symbol;Acc:HGNC:1036]
UGT3A2	UDP glycosyltransferase family 3 member A2 [Source:HGNC Symbol;Acc:HGNC:27266]
DENND6B	DENN domain containing 6B [Source:HGNC Symbol;Acc:HGNC:32690]
SSBP3	single stranded DNA binding protein 3 [Source:HGNC Symbol;Acc:HGNC:15674]
MLC1	megalencephalic leukoencephalopathy with subcortical cysts 1 [Source:HGNC Symbol;Acc:HGNC:17082]
VAT1L	vesicle amine transport 1-like [Source:HGNC Symbol;Acc:HGNC:29315]
ADGRG5	adhesion G protein-coupled receptor G5 [Source:HGNC Symbol;Acc:HGNC:19010]
HSH2D	hematopoietic SH2 domain containing [Source:HGNC Symbol;Acc:HGNC:24920]
DDAH2	dimethylarginine dimethylaminohydrolase 2 [Source:HGNC Symbol;Acc:HGNC:2716]
FAM117A	family with sequence similarity 117 member A [Source:HGNC Symbol;Acc:HGNC:24179]
TFAP2C	transcription factor AP-2 gamma [Source:HGNC Symbol;Acc:HGNC:11744]
TBX15	T-box 15 [Source:HGNC Symbol;Acc:HGNC:11594]
POU4F1	POU class 4 homeobox 1 [Source:HGNC Symbol;Acc:HGNC:9218]
ESRRA	estrogen related receptor alpha [Source:HGNC Symbol;Acc:HGNC:3471]
FOXF2	forkhead box F2 [Source:HGNC Symbol;Acc:HGNC:3810]
MLXIPL	MLX interacting protein like [Source:HGNC Symbol;Acc:HGNC:12744]
EOMES	eomesodermin [Source:HGNC Symbol;Acc:HGNC:3372]
TBX20	T-box 20 [Source:HGNC Symbol;Acc:HGNC:11598]
KLF13	Kruppel like factor 13 [Source:HGNC Symbol;Acc:HGNC:13672]
BARX1	BARX homeobox 1 [Source:HGNC Symbol;Acc:HGNC:955]

OSR2	odd-skipped related transciption factor 2 [Source:HGNC Symbol;Acc:HGNC:15830]
EBF3	early B-cell factor 3 [Source:HGNC Symbol;Acc:HGNC:19087]
NR1I2	nuclear receptor subfamily 1 group I member 2 [Source:HGNC Symbol;Acc:HGNC:7968]
TBX4	T-box 4 [Source:HGNC Symbol;Acc:HGNC:11603]
JDP2	Jun dimerization protein 2 [Source:HGNC Symbol;Acc:HGNC:17546]
ZNF41	zinc finger protein 41 [Source:HGNC Symbol;Acc:HGNC:13107]
LMX1B	LIM homeobox transcription factor 1 beta [Source:HGNC Symbol;Acc:HGNC:6654]
ZBTB7A	zinc finger and BTB domain containing 7A [Source:HGNC Symbol;Acc:HGNC:18078]
VEZF1	vascular endothelial zinc finger 1 [Source:HGNC Symbol;Acc:HGNC:12949]
NR2F6	nuclear receptor subfamily 2 group F member 6 [Source:HGNC Symbol;Acc:HGNC:7977]
TBP	TATA-box binding protein [Source:HGNC Symbol;Acc:HGNC:11588]
CUX1	cut like homeobox 1 [Source:HGNC Symbol;Acc:HGNC:2557]
CENPB	centromere protein B [Source:HGNC Symbol;Acc:HGNC:1852]
MS4A14	membrane spanning 4-domains A14 [Source:HGNC Symbol;Acc:HGNC:30706]
MS4A4A	membrane spanning 4-domains A4A [Source:HGNC Symbol;Acc:HGNC:13371]
TERF2IP	TERF2 interacting protein [Source:HGNC Symbol;Acc:HGNC:19246]
GJA8	gap junction protein alpha 8 [Source:HGNC Symbol;Acc:HGNC:4281]
PTGDR	prostaglandin D2 receptor (DP) [Source:HGNC Symbol;Acc:HGNC:9591]
PTGDR2	prostaglandin D2 receptor 2 [Source:HGNC Symbol;Acc:HGNC:4502]
NTN1	netrin 1 [Source:HGNC Symbol;Acc:HGNC:8029]
NTN3	netrin 3 [Source:HGNC Symbol;Acc:HGNC:8030]
NTN4	netrin 4 [Source:HGNC Symbol;Acc:HGNC:13658]
TRPC3	transient receptor potential cation channel subfamily C member 3 [Source:HGNC Symbol;Acc:HGNC:12335]
TRPC4	transient receptor potential cation channel subfamily C member 4 [Source:HGNC Symbol;Acc:HGNC:12336]
TRPC5	transient receptor potential cation channel subfamily C member 5 [Source:HGNC Symbol;Acc:HGNC:12337]
TRPC6	transient receptor potential cation channel subfamily C member 6 [Source:HGNC Symbol;Acc:HGNC:12338]
ABLIM1	actin binding LIM protein 1 [Source:HGNC Symbol;Acc:HGNC:78]
ABLIM3	actin binding LIM protein family member 3 [Source:HGNC Symbol;Acc:HGNC:29132]
ABLIM2	actin binding LIM protein family member 2 [Source:HGNC Symbol;Acc:HGNC:19195]
UNC5A	unc-5 netrin receptor A [Source:HGNC Symbol;Acc:HGNC:12567]
UNC5B	unc-5 netrin receptor B [Source:HGNC Symbol;Acc:HGNC:12568]
UNC5C	unc-5 netrin receptor C [Source:HGNC Symbol;Acc:HGNC:12569]
UNC5D	unc-5 netrin receptor D [Source:HGNC Symbol;Acc:HGNC:18634]
LRIG2	leucine rich repeats and immunoglobulin like domains 2 [Source:HGNC Symbol;Acc:HGNC:20889]
EFNB1	ephrin B1 [Source:HGNC Symbol;Acc:HGNC:3226]
EFNB2	ephrin B2 [Source:HGNC Symbol;Acc:HGNC:3227]
EFNB3	ephrin B3 [Source:HGNC Symbol;Acc:HGNC:3228]
EPHA4	EPH receptor A4 [Source:HGNC Symbol;Acc:HGNC:3388]

EPHA5	EPH receptor A5 [Source:HGNC Symbol;Acc:HGNC:3389]
EPHA6	EPH receptor A6 [Source:HGNC Symbol;Acc:HGNC:19296]
EPHA7	EPH receptor A7 [Source:HGNC Symbol;Acc:HGNC:3390]
EPHA8	EPH receptor A8 [Source:HGNC Symbol;Acc:HGNC:3391]
EPHB3	EPH receptor B3 [Source:HGNC Symbol;Acc:HGNC:3394]
EPHB6	EPH receptor B6 [Source:HGNC Symbol;Acc:HGNC:3396]
NGEF	neuronal guanine nucleotide exchange factor [Source:HGNC Symbol;Acc:HGNC:7807]
RGS3	regulator of G-protein signaling 3 [Source:HGNC Symbol;Acc:HGNC:9999]
SLIT1	slit guidance ligand 1 [Source:HGNC Symbol;Acc:HGNC:11085]
SLIT2	slit guidance ligand 2 [Source:HGNC Symbol;Acc:HGNC:11086]
ROBO1	roundabout guidance receptor 1 [Source:HGNC Symbol;Acc:HGNC:10249]
ROBO2	roundabout guidance receptor 2 [Source:HGNC Symbol;Acc:HGNC:10250]
ROBO3	roundabout guidance receptor 3 [Source:HGNC Symbol;Acc:HGNC:13433]
SRGAP2	SLIT-ROBO Rho GTPase activating protein 2 [Source:HGNC Symbol;Acc:HGNC:19751]
SRGAP1	SLIT-ROBO Rho GTPase activating protein 1 [Source:HGNC Symbol;Acc:HGNC:17382]
SRGAP3	SLIT-ROBO Rho GTPase activating protein 3 [Source:HGNC Symbol;Acc:HGNC:19744]
SEMA3A	semaphorin 3A [Source:HGNC Symbol;Acc:HGNC:10723]
SEMA3C	semaphorin 3C [Source:HGNC Symbol;Acc:HGNC:10725]
SEMA3D	semaphorin 3D [Source:HGNC Symbol;Acc:HGNC:10726]
SEMA3E	semaphorin 3E [Source:HGNC Symbol;Acc:HGNC:10727]
SEMA3F	semaphorin 3F [Source:HGNC Symbol;Acc:HGNC:10728]
SEMA3G	semaphorin 3G [Source:HGNC Symbol;Acc:HGNC:30400]
PLXNA2	plexin A2 [Source:HGNC Symbol;Acc:HGNC:9100]
PLXNA3	plexin A3 [Source:HGNC Symbol;Acc:HGNC:9101]
PLXNA1	plexin A1 [Source:HGNC Symbol;Acc:HGNC:9099]
PLXNA4	plexin A4 [Source:HGNC Symbol;Acc:HGNC:9102]
RHOD	ras homolog family member D [Source:HGNC Symbol;Acc:HGNC:670]
RND1	Rho family GTPase 1 [Source:HGNC Symbol;Acc:HGNC:18314]
FES	FES proto-oncogene, tyrosine kinase [Source:HGNC Symbol;Acc:HGNC:3657]
DPYSL5	dihydropyrimidinase like 5 [Source:HGNC Symbol;Acc:HGNC:20637]
DPYSL2	dihydropyrimidinase like 2 [Source:HGNC Symbol;Acc:HGNC:3014]
SEMA4F	ssemaphorin 4F [Source:HGNC Symbol;Acc:HGNC:10734]
SEMA4B	semaphorin 4B [Source:HGNC Symbol;Acc:HGNC:10730]
SEMA4C	semaphorin 4C [Source:HGNC Symbol;Acc:HGNC:10731]
SEMA4G	semaphorin 4G [Source:HGNC Symbol;Acc:HGNC:10735]
SEMA4A	semaphorin 4A [Source:HGNC Symbol;Acc:HGNC:10729]
SEMA5A	semaphorin 5A [Source:HGNC Symbol;Acc:HGNC:10736]
SEMA5B	semaphorin 5B [Source:HGNC Symbol;Acc:HGNC:10737]
SEMA6A	semaphorin 6A [Source:HGNC Symbol;Acc:HGNC:10738]
SEMA6C	semaphorin 6C [Source:HGNC Symbol;Acc:HGNC:10740]
SEMA6D	semaphorin 6D [Source:HGNC Symbol;Acc:HGNC:16770]

PLXNB1	plexin B1 [Source:HGNC Symbol;Acc:HGNC:9103]
PLXNB2	plexin B2 [Source:HGNC Symbol;Acc:HGNC:9104]
PLXNB3	plexin B3 [Source:HGNC Symbol;Acc:HGNC:9105]
SEMA7A	semaphorin 7A (John Milton Hagen blood group) [Source:HGNC Symbol;Acc:HGNC:10741]
PLXNC1	plexin C1 [Source:HGNC Symbol;Acc:HGNC:9106]
PTGIR	prostaglandin I2 (prostacyclin) receptor (IP) [Source:HGNC Symbol;Acc:HGNC:9602]
SLCO2A1	solute carrier organic anion transporter family member 2A1 [Source:HGNC Symbol;Acc:HGNC:10955]
KDM6B	lysine demethylase 6B [Source:HGNC Symbol;Acc:HGNC:29012]
KDM1A	lysine demethylase 1A [Source:HGNC Symbol;Acc:HGNC:29079]
KDM1B	lysine demethylase 1B [Source:HGNC Symbol;Acc:HGNC:21577]
NR3C1	nuclear receptor subfamily 3 group C member 1 [Source:HGNC Symbol;Acc:HGNC:7978]
CYP26B1	cytochrome P450 family 26 subfamily B member 1 [Source:HGNC Symbol;Acc:HGNC:20581]
STRA8	stimulated by retinoic acid 8 [Source:HGNC Symbol;Acc:HGNC:30653]
PDE6A	phosphodiesterase 6A [Source:HGNC Symbol;Acc:HGNC:8785]
GUCY2F	guanylate cyclase 2F, retinal [Source:HGNC Symbol;Acc:HGNC:4691]
PDE6B	phosphodiesterase 6B [Source:HGNC Symbol;Acc:HGNC:8786]
PDE6G	phosphodiesterase 6G [Source:HGNC Symbol;Acc:HGNC:8789]
RCVRN	recoverin [Source:HGNC Symbol;Acc:HGNC:9937]
RGS9	regulator of G-protein signaling 9 [Source:HGNC Symbol;Acc:HGNC:10004]
SAG	S-antigen; retina and pineal gland (arrestin) [Source:HGNC Symbol;Acc:HGNC:10521]
GNAT1	G protein subunit alpha transducin 1 [Source:HGNC Symbol;Acc:HGNC:4393]
PICALM	phosphatidylinositol binding clathrin assembly protein [Source:HGNC Symbol;Acc:HGNC:15514]
GUCY2D	guanylate cyclase 2D, retinal [Source:HGNC Symbol;Acc:HGNC:4689]
OPRM1	opioid receptor mu 1 [Source:HGNC Symbol;Acc:HGNC:8156]
GPER1	G protein-coupled estrogen receptor 1 [Source:HGNC Symbol;Acc:HGNC:4485]
KCNJ3	potassium voltage-gated channel subfamily J member 3 [Source:HGNC Symbol;Acc:HGNC:6264]
KCNJ6	potassium voltage-gated channel subfamily J member 6 [Source:HGNC Symbol;Acc:HGNC:6267]
KCNJ9	potassium voltage-gated channel subfamily J member 9 [Source:HGNC Symbol;Acc:HGNC:6270]
KRT1	keratin 1 [Source:HGNC Symbol;Acc:HGNC:6412]
PGR	progesterone receptor [Source:HGNC Symbol;Acc:HGNC:8910]
EBAG9	estrogen receptor binding site associated, antigen, 9 [Source:HGNC Symbol;Acc:HGNC:3123]
TFF1	trefoil factor 1 [Source:HGNC Symbol;Acc:HGNC:11755]
FKBP5	FK506 binding protein 5 [Source:HGNC Symbol;Acc:HGNC:3721]
FKBP4	FK506 binding protein 4 [Source:HGNC Symbol;Acc:HGNC:3720]
GABBR1	gamma-aminobutyric acid type B receptor subunit 1 [Source:HGNC Symbol;Acc:HGNC:4070]
GABBR2	gamma-aminobutyric acid type B receptor subunit 2 [Source:HGNC Symbol;Acc:HGNC:4507]
F2RL1	F2R like trypsin receptor 1 [Source:HGNC Symbol;Acc:HGNC:3538]
ASIC2	acid sensing ion channel subunit 2 [Source:HGNC Symbol;Acc:HGNC:99]
ASIC1	acid sensing ion channel subunit 1 [Source:HGNC Symbol;Acc:HGNC:100]
ASIC3	acid sensing ion channel subunit 3 [Source:HGNC Symbol;Acc:HGNC:101]
ASIC4	acid sensing ion channel subunit family member 4 [Source:HGNC Symbol;Acc:HGNC:21263]

ASIC5	acid sensing ion channel subunit family member 5 [Source:HGNC Symbol;Acc:HGNC:17537]
TRPV3	transient receptor potential cation channel subfamily V member 3 [Source:HGNC Symbol;Acc:HGNC:18084]
TRPM8	transient receptor potential cation channel subfamily M member 8 [Source:HGNC Symbol;Acc:HGNC:17961]
TRPA1	transient receptor potential cation channel subfamily A member 1 [Source:HGNC Symbol;Acc:HGNC:497]
TRPV1	transient receptor potential cation channel subfamily V member 1 [Source:HGNC Symbol;Acc:HGNC:12716]
PRKCH	protein kinase C eta [Source:HGNC Symbol;Acc:HGNC:9403]
ABCF1	ATP binding cassette subfamily F member 1 [Source:HGNC Symbol;Acc:HGNC:70]

Supplementary Table S4: Topological network analysis related to nAMD disease							
Betweenness unDir	Bridging unDir	Centroid unDir	Closeness unDir	Degree unDir	Eccentricity unDir	EigenVector unDir	gene name
0	0	0	0	0	0	0	PLET1
0	0	0	0	0	0	0	NPIPA2
0	0	0	0	0	0	0	ARMS2
0	0	0	0	0	0	0	NPIPA3
0	0	0	0	0	0	0	CCDC175
0	0	0	0	0	0	0	XKR9
0	0	0	0	0	0	0	MTRNR2L13
0	0	0	0	0	0	0	C11orf91
0	0	0	0	0	0	0	ARL14EPL
0	0	0	0	0	0	0	GAGE12B
0	0	-7068	4.84E-05	1	0.25	1.06E-05	ATP5L2
0.422064	3.270977	-7068	5.81E-05	9	0.25	1.15E-04	CBSL
0	0	-7068	4.97E-05	2	0.25	2.22E-05	MPC1L
0	0	-7068	4.83E-05	1	0.25	1.06E-05	UQCRHL
0	0	-7068	5.09E-05	3	0.25	3.23E-05	MAGEB16
0	0	-7068	6.31E-05	21	0.25	2.99E-04	HMSD
0	0	-7068	4.79E-05	1	0.25	7.37E-06	NBPF6
3.89924	2.111259	-7068	6.36E-05	27	0.25	2.44E-04	NKX2-4
0.060427	0.082591	-7068	6.27E-05	18	0.25	2.08E-04	SULT6B1
0	0	-7068	4.78E-05	2	0.25	4.07E-06	KRTAP10-6
2.876746	2.035024	-7068	6.40E-05	25	0.25	2.86E-04	ARGFX
14.19459	3.252732	-7068	6.61E-05	48	0.25	5.05E-04	XXbac-BPG116M5.17
29.85258	9.268707	-7068	6.68E-05	39	0.25	4.13E-04	RP11-330H6.5
51.8231	6.739027	-7068	6.83E-05	62	0.25	6.94E-04	XXbac-BPG246D15.9
0.144661	0.232833	-7068	5.81E-05	11	0.25	1.18E-04	TBC1D3I
0	0	-7068	4.76E-05	3	0.25	3.70E-06	SPDYE5
0	0	-7068	4.64E-05	2	0.25	6.54E-07	PRAMEF5
15.94021	10.87097	-7068	6.45E-05	26	0.25	3.06E-04	RP11-566K11.2
0	0	-7068	6.03E-05	23	0.25	1.75E-04	OPN1MW3
0	0	-7068	5.29E-05	29	0.25	5.45E-05	OR8B3
0	0	-7068	5.29E-05	29	0.25	5.45E-05	OR4F16
0	0	-7068	4.94E-05	3	0.25	1.81E-05	GAGE12G
0	0	-7068	4.94E-05	3	0.25	1.81E-05	GAGE12J
0	0	-1	1	1	1	-5.71E-38	FRG2C
0	0	-1	1	1	1	1.37E-37	FRG2B
0	0	-7068	4.99E-05	5	0.25	2.37E-05	CT45A2
0	0	-7068	4.99E-05	5	0.25	2.37E-05	CT45A6

11.34631	1.284786	-7068	6.86E-05	55	0.25	7.24E-04	HES3
0	0	-7068	6.21E-05	24	0.25	2.30E-04	FABP9
1.711409	0.65972	-7068	6.25E-05	25	0.25	2.36E-04	LCN9
0.558884	1.234766	-7068	6.64E-05	15	0.25	2.34E-04	FER1L6
0.99207	1.728618	-7068	6.73E-05	18	0.25	2.90E-04	SYNJ2BP-COX16
0	0	-7068	6.05E-05	2	0.25	7.48E-05	FAM72D
25.68782	10.39498	-7068	5.22E-05	6	0.25	4.53E-05	CT45A3
3.376956	2.24473	-7068	6.14E-05	3	0.25	8.86E-05	FAM72A
298.1002	5.830237	-7068	7.01E-05	143	0.25	0.001283	ZNF774
0.347207	8.288332	-7068	5.47E-05	5	0.25	6.47E-05	CCDC103
7.372626	0.142835	-7068	6.81E-05	125	0.333333	4.58E-04	ZNF816
2124.667	1350.073	-7068	6.06E-05	3	0.25	7.53E-05	PRAMEF14
3.338812	0.274585	-7068	7.01E-05	79	0.333333	0.001052	ZNF286B
3.361538	3.212917	-7068	6.74E-05	22	0.25	3.15E-04	PPP1R42
0.256778	1.527373	-7068	6.51E-05	11	0.333333	1.93E-04	PGAM4
8.8684	2.832745	-7068	6.57E-05	33	0.25	3.63E-04	AGAP4
7.217815	2.433165	-7068	6.55E-05	32	0.25	3.49E-04	AGAP5
0	0	-7068	5.23E-05	20	0.25	5.39E-05	UGT1A3
0	0	-7068	5.23E-05	20	0.25	5.39E-05	UGT1A9
17.43957	3.057227	-7068	5.51E-05	18	0.25	8.41E-05	UGT1A7
0.033408	5.318025	-7068	5.04E-05	2	0.25	2.62E-05	CDRT4
2.44733	5.117225	-7068	5.95E-05	15	0.25	1.66E-04	HIST2H3PS2
0.015873	2.078055	-7068	5.48E-05	3	0.25	7.26E-05	SLFN12L
1.532176	2.939222	-7068	6.62E-05	16	0.25	2.63E-04	KBTBD13
0.006711	2.346643	-7068	5.25E-05	2	0.25	4.67E-05	NPIPB3
12.92875	1.428447	-7068	6.87E-05	56	0.25	7.48E-04	SCX
2.578124	0.664229	-7068	6.60E-05	34	0.25	3.89E-04	HELT
2.574998	2.449998	-7068	6.65E-05	27	0.25	4.19E-04	KPNA7
1.079224	4.39136	-7068	6.55E-05	12	0.333333	2.38E-04	MZT2A
184.2197	5.619463	-7068	6.97E-05	105	0.333333	0.001098	GPR21
14.0439	7.891421	-7068	6.63E-05	31	0.25	4.33E-04	USP50
16.5118	1.180955	-7068	5.48E-05	30	0.25	7.34E-05	OR4F21
25.56095	1.76106	-7068	5.61E-05	31	0.25	8.71E-05	OR2T34
25.56095	1.76106	-7068	5.61E-05	31	0.25	8.71E-05	OR2AT4
25.56095	1.76106	-7068	5.61E-05	31	0.25	8.71E-05	OR1L8
25.56095	1.76106	-7068	5.61E-05	31	0.25	8.71E-05	OR2T3
9.040928	0.646326	-7068	5.45E-05	30	0.25	6.82E-05	OR6C75
9.040928	0.646326	-7068	5.45E-05	30	0.25	6.82E-05	OR6S1
9.040928	0.646326	-7068	5.45E-05	30	0.25	6.82E-05	OR8J3
206.1147	18.23558	-7068	6.42E-05	30	0.25	2.66E-04	UGT1A10
2.531606	3.625075	-7068	6.07E-05	19	0.25	2.13E-04	MT-CYB

3.612852	2.258594	-7068	6.33E-05	23	0.25	2.97E-04	MT-CO3
24.35464	2.354276	-7068	6.50E-05	50	0.25	4.03E-04	MT-ND5
5.35651	0.964575	-7068	6.21E-05	35	0.25	3.14E-04	MT-ND4L
0	0	-7068	6.13E-05	34	0.25	2.99E-04	MT-ND1
0	0	-7068	6.13E-05	34	0.25	2.99E-04	MT-ND3
53.64532	5.777051	-7068	6.98E-05	78	0.333333	0.001067	GRK1
2076.119	50.28496	-7068	6.98E-05	77	0.333333	9.59E-04	OR5B3
9.200289	5.605351	-7068	6.95E-05	38	0.333333	6.74E-04	GPR179
71.06972	7.154139	-7068	7.05E-05	89	0.333333	0.001518	CERKL
275.0484	9.504399	-7068	7.08E-05	135	0.333333	0.002031	TMIGD1
438.5931	6.832813	-7068	7.16E-05	226	0.333333	0.00378	HRNR
195.0854	8.040193	-7068	7.06E-05	118	0.333333	0.00211	SYT15
386.9249	7.021768	-7068	7.13E-05	202	0.333333	0.003426	GDF1
21.95245	10.59598	-7068	6.80E-05	35	0.25	5.10E-04	INCA1
2378.096	50.31453	-7068	7.01E-05	85	0.333333	0.001008	OR4X1
2494.72	5.976816	-7068	7.37E-05	607	0.333333	0.010706	ITCH
330.5595	6.851175	-7068	7.13E-05	197	0.333333	0.003373	SP110
1196.585	39.27573	-7068	6.75E-05	58	0.333333	4.89E-04	OR4Q3
2612.048	6.796457	-7068	7.34E-05	562	0.333333	0.00942	ATG5
175.3925	8.812308	-7068	7.04E-05	107	0.25	0.001435	KIR3DL1
4970.29	6.533477	-7068	7.49E-05	807	0.333333	0.014277	SHTN1
966.442	6.568944	-7068	7.23E-05	353	0.333333	0.006108	FGF13
15975	48.56064	-7068	7.24E-05	371	0.333333	0.006109	OR2Y1
309.0818	7.896675	-7068	7.12E-05	172	0.333333	0.002907	LGALS7
1968.594	6.452741	-7068	7.31E-05	505	0.333333	0.008368	LIMS1
299.8375	7.042872	-7068	7.13E-05	188	0.333333	0.003265	PI4K2B
54.93278	6.229445	-7068	7.04E-05	88	0.333333	0.001539	TINCR
3705.381	7.08263	-7068	7.39E-05	648	0.333333	0.011102	ASPH
1358.481	6.393515	-7068	7.27E-05	429	0.25	0.007557	ANXA8
770.7453	7.245045	-7068	7.19E-05	293	0.333333	0.004972	PRR20A
4338.352	9.519032	-7068	7.35E-05	562	0.333333	0.009602	TBC1D3
99.15024	8.357576	-7068	7.05E-05	87	0.333333	0.001446	SLC22A17
5772.995	56.78734	-7068	7.10E-05	158	0.333333	0.002299	OR1J1
261.8053	6.23125	-7068	7.12E-05	192	0.333333	0.003432	FAM177B
1412.21	7.913186	-7068	7.23E-05	352	0.333333	0.005875	SYT14
2925.07	7.332279	-7068	7.35E-05	559	0.333333	0.009161	OSTN
39.58778	8.270222	-7068	6.92E-05	57	0.25	8.67E-04	EPGN
3073.492	6.948716	-7068	7.36E-05	597	0.333333	0.010292	OCM
689.093	8.317366	-7068	7.16E-05	245	0.25	0.004048	VSX2
248.6318	8.047341	-7068	7.08E-05	147	0.25	0.002398	SAMD1
17960.33	45.65369	-7068	7.26E-05	415	0.333333	0.006853	OR5AN1

967.5766	6.520672	-7068	7.24E-05	356	0.25	0.006207	LRIT3
507.3977	7.249187	-7068	7.15E-05	235	0.25	0.003924	CD200R1L
166.9119	6.514085	-7068	7.09E-05	146	0.25	0.00242	APOC2
210.6329	6.407555	-7068	7.11E-05	167	0.333333	0.002969	TMEM189
1105.921	6.969024	-7068	7.22E-05	361	0.333333	0.006174	TJP2
618.0642	7.83994	-7068	7.15E-05	239	0.333333	0.003693	HLA-DQA1
157.281	7.779296	-7068	7.06E-05	119	0.25	0.001773	BFSP1
799.1741	7.547658	-7068	7.17E-05	274	0.333333	0.004167	CALM2
2009.595	6.448639	-7068	7.32E-05	517	0.333333	0.008952	ENTPD1
5161.72	6.583788	-7068	7.48E-05	814	0.333333	0.013733	GSN
3291.777	7.394603	-7068	7.35E-05	584	0.25	0.009612	AMY1A
3.561178	6.328823	-7068	6.05E-05	17	0.25	1.67E-04	PRY
241.9272	8.806639	-7068	7.08E-05	133	0.333333	0.001831	AMY2A
1315.899	7.786385	-7068	7.21E-05	353	0.25	0.005512	C4A
828.585	8.040042	-7068	7.18E-05	275	0.333333	0.004343	GANC
5.906222	8.473852	-7068	6.14E-05	18	0.333333	1.66E-04	NUDT17
283.95	13.12915	-7068	6.97E-05	86	0.25	0.001061	MT-ND6
6079.618	7.249118	-7068	7.49E-05	821	0.333333	0.013844	NAALADL2
60.0001	4.70605	-7068	6.92E-05	68	0.25	8.55E-04	TCF24
4187.285	6.765228	-7068	7.44E-05	719	0.333333	0.012611	PTCHD4
5936.553	7.403324	-7068	7.49E-05	803	0.333333	0.013732	ILDR2
15624.14	27.61397	-7068	7.24E-05	376	0.333333	0.006295	NBPF4
1846.695	7.028857	-7068	7.29E-05	463	0.333333	0.007896	KCNT2
853.3065	8.646166	-7068	7.18E-05	262	0.333333	0.004164	SYPL2
281.1494	8.985286	-7068	7.05E-05	123	0.333333	0.001463	SP6
29.78079	13.03601	-7068	6.65E-05	33	0.25	3.42E-04	IRGM
8329.231	57.63604	-7068	7.10E-05	150	0.25	0.001786	KRTAP5-2
208.4232	6.563666	-7068	7.11E-05	159	0.333333	0.002743	VANGL2
204.4172	8.852742	-7068	7.05E-05	121	0.25	0.001705	C8orf82
474.118	17.22841	-7068	6.31E-05	51	0.333333	2.25E-04	OR52D1
5950.017	54.13695	-7068	7.11E-05	163	0.333333	0.002266	OR8B2
387.601	11.33052	-7068	7.10E-05	142	0.333333	0.002319	ATP6V1E2
420.0357	9.849306	-7068	7.08E-05	138	0.333333	0.001905	PPP1R27
216.8794	7.604128	-7068	7.07E-05	137	0.333333	0.001774	TTC36
7.892079	10.38648	-7068	5.83E-05	15	0.25	1.21E-04	C2orf71
81.09107	6.787382	-7068	6.97E-05	84	0.25	0.001114	SRL
5.117996	6.941845	-7068	5.90E-05	17	0.25	1.39E-04	DEFB108B
847.7078	15.01881	-7068	6.89E-05	84	0.25	7.88E-04	UGT1A1
73.86677	13.19224	-7068	6.75E-05	45	0.25	4.13E-04	BTBD17
152.0138	6.35893	-7068	7.01E-05	116	0.25	0.001708	LRRN4CL
88.79086	4.932558	-7068	6.99E-05	97	0.25	0.001103	CYSRT1

126.6058	5.37856	-7068	7.05E-05	112	0.333333	0.001372	KRT78
269.7365	8.822456	-7068	6.96E-05	90	0.25	9.54E-04	MT-ND4
134.154	8.566897	-7068	6.97E-05	72	0.25	9.70E-04	MT-CO2
314.0838	10.42404	-7068	7.00E-05	88	0.25	9.96E-04	MT-ND2
152.47	7.710524	-7068	6.89E-05	63	0.25	7.58E-04	MT-CO1
42.21674	6.261667	-7068	6.92E-05	52	0.333333	6.97E-04	MT-ATP6
31.62677	11.67661	-7068	6.16E-05	22	0.25	1.87E-04	COX8C
38.36342	17.04477	-7068	6.78E-05	26	0.25	3.76E-04	FBXO43
222.704	3.430206	-7068	6.88E-05	144	0.333333	5.92E-04	ZNF316
163.8689	2.278439	-7068	6.85E-05	154	0.25	7.44E-04	ZNF805
1605.254	6.579695	-7068	7.27E-05	450	0.333333	0.007655	RSBN1L
2723.329	5.851032	-7068	7.34E-05	610	0.333333	0.00914	PGBD5
1309.657	8.706293	-7068	7.22E-05	328	0.333333	0.004824	C19orf54
472.2899	9.02444	-7068	7.11E-05	176	0.333333	0.002169	CST4
605.2255	8.698003	-7068	7.11E-05	190	0.333333	0.00211	KIR2DL3
376.8712	6.199996	-7068	7.11E-05	209	0.25	0.002889	MT1HL1
2020.111	8.500328	-7068	7.24E-05	398	0.333333	0.005572	FCGR1A
875.08	7.315356	-7068	7.21E-05	312	0.333333	0.004864	YIF1B
6037.965	7.175458	-7068	7.48E-05	811	0.333333	0.01336	SNX25
1183.048	10.85769	-7068	7.17E-05	258	0.333333	0.003403	KANSL3
1002.493	7.478439	-7068	7.19E-05	314	0.333333	0.004617	NLRP12
3390.059	12.38236	-7068	7.22E-05	347	0.333333	0.004097	GDF2
731.1187	7.348545	-7068	7.18E-05	274	0.333333	0.004179	AKR1C2
916.0544	7.638474	-7068	7.19E-05	302	0.333333	0.004952	RSPRY1
882.5394	18.2641	-7068	7.05E-05	132	0.25	0.001079	SSX3
93.70651	7.622666	-7068	7.00E-05	89	0.333333	0.001198	IFNA13
238.0506	6.320384	-7068	7.11E-05	173	0.333333	0.002824	MSH5
153.0133	10.06966	-7068	7.02E-05	86	0.25	0.001189	SULT1A3
3956.91	9.732289	-7068	7.30E-05	486	0.333333	0.006469	GRM6
1588.072	10.33844	-7068	7.20E-05	299	0.333333	0.004104	RNF32
5348.103	8.916797	-7068	7.38E-05	643	0.333333	0.00936	TRIM16
11403.37	6.867685	-7068	7.65E-05	1150	0.333333	0.01841	BTF3L4
1039.497	7.511026	-7068	7.21E-05	325	0.333333	0.005394	ARHGAP8
76.72704	6.883284	-7068	7.01E-05	91	0.25	0.001318	CKMT1B
0.942151	2.660683	-7068	6.11E-05	16	0.25	2.10E-04	GOLGA6L1
1191.413	5.078885	-7068	7.21E-05	415	0.25	0.004828	PLGLB2
648.9707	13.41843	-7068	7.05E-05	138	0.25	0.00134	SSX4
165.5702	10.16968	-7068	7.05E-05	100	0.333333	0.001359	BAHCC1
505.4897	12.15681	-7068	7.10E-05	150	0.333333	0.001961	GGT1
5182.091	7.088441	-7068	7.43E-05	748	0.333333	0.011792	TICAM2
28.81553	7.215941	-7068	6.83E-05	49	0.25	6.15E-04	OVOS2

3458.795	7.929891	-7068	7.34E-05	568	0.333333	0.009124	MYRFL
1141.696	8.079538	-7068	7.22E-05	332	0.333333	0.005345	FBF1
1743.014	9.484908	-7068	7.23E-05	363	0.333333	0.006076	RAD9B
0.823281	2.484938	-7068	6.11E-05	15	0.25	2.45E-04	PYURF
369.0547	74.19517	-7068	5.56E-05	12	0.25	7.73E-05	SPDYE1
1691.386	12.94332	-7068	7.19E-05	273	0.333333	0.004129	USP17L2
526.7442	9.154079	-7068	7.08E-05	148	0.333333	0.001661	P2RY11
10335.21	7.936009	-7068	7.55E-05	971	0.333333	0.013811	BAG3
7653.198	8.025966	-7068	7.46E-05	818	0.333333	0.011208	SIRT7
16959.09	8.801299	-7068	7.65E-05	1151	0.333333	0.016007	ERCC5
6210.237	10.2044	-7068	7.37E-05	616	0.333333	0.008073	HECTD3
5189.595	8.401008	-7068	7.38E-05	650	0.333333	0.00938	POLI
10421.25	7.079591	-7068	7.58E-05	1044	0.333333	0.015221	ACTR6
6815.701	6.51335	-7068	7.47E-05	898	0.333333	0.012453	NOTCH1
17165.52	7.677642	-7068	7.73E-05	1293	0.333333	0.019503	COMMD3
10955.71	7.721539	-7068	7.53E-05	993	0.333333	0.012939	CPSF7
22030.31	7.708428	-7068	7.79E-05	1409	0.333333	0.020596	ASH1L
8399.704	7.751735	-7068	7.42E-05	801	0.333333	0.010294	ZNF302
15847.2	8.2688	-7068	7.66E-05	1168	0.333333	0.017094	UBQLN4
6765.527	8.252318	-7068	7.44E-05	775	0.333333	0.011359	FBXO6
2997.675	6.465791	-7068	7.34E-05	603	0.333333	0.008746	BMI1
21009.95	8.602895	-7068	7.73E-05	1290	0.333333	0.018521	DRD5
18011.11	9.728257	-7068	7.61E-05	1055	0.333333	0.013181	ALPP
9631.572	8.274409	-7068	7.52E-05	911	0.333333	0.012804	SIRT2
9493.464	9.220713	-7068	7.46E-05	808	0.333333	0.011202	ANKRD49
12342.69	8.122707	-7068	7.59E-05	1045	0.333333	0.015953	ARGLU1
13454.15	7.565606	-7068	7.66E-05	1153	0.333333	0.017704	MYO9A
4157.933	5.983673	-7068	7.36E-05	711	0.333333	0.008452	RPL3
7975.301	6.205265	-7068	7.52E-05	986	0.333333	0.012919	PTBP1
6615.873	7.775453	-7068	7.46E-05	805	0.333333	0.012095	SMC6
12835.05	7.160756	-7068	7.63E-05	1142	0.333333	0.016924	CAAP1
29942.99	7.024737	-7068	8.02E-05	1815	0.333333	0.027327	FBXW7
6879.457	7.670964	-7068	7.46E-05	818	0.333333	0.011957	FAF1
27262.43	6.915393	-7068	7.97E-05	1743	0.333333	0.026103	CAMK2D
2986.211	9.24294	-7068	7.27E-05	448	0.333333	0.005645	NPIPA1
14972.93	6.969615	-7068	7.72E-05	1292	0.333333	0.019821	USP47
930.9118	6.21293	-7068	7.20E-05	340	0.333333	0.005261	HSPA1A
15416.15	6.959159	-7068	7.70E-05	1286	0.333333	0.01817	PDGFD
5264.596	8.175446	-7068	7.40E-05	672	0.333333	0.00943	PUS1
7143.563	7.603131	-7068	7.47E-05	830	0.333333	0.01213	INTS8
7501.491	6.448514	-7068	7.55E-05	968	0.333333	0.014799	ARL8B

8190.242	8.634735	-7068	7.47E-05	817	0.333333	0.011788	TAF1D
4730.018	6.885106	-7068	7.43E-05	736	0.333333	0.011275	YPEL5
9751.494	7.358208	-7068	7.57E-05	1002	0.333333	0.015294	SENP7
4904.443	8.984369	-7068	7.36E-05	618	0.333333	0.009015	ULK4
13029.52	7.236453	-7068	7.65E-05	1163	0.333333	0.016977	SRPRB
20308.13	6.435581	-7068	7.88E-05	1617	0.333333	0.024698	SET
13239.49	7.715319	-7068	7.63E-05	1125	0.333333	0.016115	DCTN1
8104.602	11.12252	-7068	7.39E-05	645	0.333333	0.007932	P4HTM
18624.59	12.37981	-7068	7.49E-05	851	0.333333	0.010632	GPR75
8755.74	7.130952	-7068	7.54E-05	968	0.333333	0.014159	DROSHA
9641.389	6.228956	-7068	7.59E-05	1087	0.333333	0.015797	NOL11
5240.518	7.605711	-7068	7.40E-05	699	0.333333	0.010288	CASP8AP2
12251.41	5.783018	-7068	7.62E-05	1292	0.333333	0.015955	MAPK3
6697.254	9.204547	-7068	7.39E-05	672	0.333333	0.008113	DENND1C
15686.88	6.312503	-7068	7.79E-05	1429	0.333333	0.021891	PELI1
5105.729	8.516304	-7068	7.37E-05	628	0.333333	0.008275	C12orf10
6842.91	6.566708	-7068	7.48E-05	873	0.333333	0.012186	NOC3L
13321.01	7.377758	-7068	7.64E-05	1162	0.333333	0.017402	BACH2
20659.05	6.714179	-7068	7.87E-05	1564	0.333333	0.024036	TBL1XR1
7492.033	6.952537	-7068	7.52E-05	924	0.333333	0.014389	SNX5
10977.66	7.759131	-7068	7.57E-05	1016	0.333333	0.01499	RMI1
16355.29	7.971508	-7068	7.69E-05	1212	0.333333	0.018148	HDAC11
5271.463	6.739289	-7068	7.42E-05	776	0.333333	0.010921	MATR3
4648.827	9.937542	-7068	7.32E-05	537	0.333333	0.007033	ARFGAP1
5222.93	6.508581	-7068	7.43E-05	798	0.333333	0.011623	PDPK1
6542.26	8.034067	-7068	7.42E-05	758	0.333333	0.010642	KLF2
6663.77	7.58307	-7068	7.45E-05	783	0.333333	0.010355	LONP1
5570.108	9.207146	-7068	7.39E-05	635	0.333333	0.008826	MAP1S
7369.988	6.520261	-7068	7.57E-05	974	0.333333	0.016444	CEP170
3063.548	7.436078	-7068	7.31E-05	549	0.333333	0.007885	EIF3L
11205.76	7.40767	-7068	7.61E-05	1075	0.333333	0.016588	PDCD4
9331.067	6.561067	-7068	7.61E-05	1083	0.333333	0.01742	REV1
7657.01	6.443849	-7068	7.53E-05	979	0.333333	0.014417	NCOR1
4900.678	11.203	-7068	7.29E-05	487	0.333333	0.006209	ANKRD36B
9596.886	6.911522	-7068	7.56E-05	1035	0.333333	0.015157	UBQLN1
11912.86	7.528504	-7068	7.64E-05	1116	0.333333	0.017204	NAGK
2172.306	8.139453	-7068	7.27E-05	445	0.333333	0.00671	NCBP3
10204.78	6.615314	-7068	7.58E-05	1061	0.333333	0.014634	MRPL15
11715.17	6.445885	-7068	7.64E-05	1183	0.333333	0.017491	DCAF6
8042.63	6.855992	-7068	7.51E-05	926	0.333333	0.012611	HLA-DRB1
48837.8	8.757599	-7068	8.06E-05	1913	0.333333	0.026454	DAB1

13890.83	7.579911	-7068	7.67E-05	1190	0.333333	0.018257	ANKFY1
10956.5	6.918013	-7068	7.61E-05	1103	0.333333	0.016511	SLTM
1282.14	10.54716	-7068	7.16E-05	248	0.25	0.00331	MESP1
11130.74	6.863587	-7068	7.63E-05	1128	0.333333	0.017298	SLC24A3
12641.09	6.776514	-7068	7.69E-05	1217	0.333333	0.019406	ARMC1
17564.87	7.866987	-7068	7.71E-05	1254	0.333333	0.019239	LRRC49
6383.369	6.99231	-7068	7.50E-05	850	0.333333	0.01323	PTCD3
12852.24	7.665983	-7068	7.60E-05	1082	0.333333	0.014972	ACSF2
14526.25	5.840626	-7068	7.77E-05	1463	0.333333	0.0222	FKBP1A
2775.306	7.89111	-7068	7.31E-05	510	0.333333	0.007777	GCC2
4594.731	7.570142	-7068	7.39E-05	673	0.333333	0.009938	FKBP3
2673.921	8.873957	-7068	7.27E-05	445	0.333333	0.006117	NPDC1
4807.142	7.827995	-7068	7.36E-05	645	0.333333	0.00863	CHCHD2
6739.259	8.468382	-7068	7.43E-05	748	0.333333	0.010793	PLEKHO1
5296.362	8.621645	-7068	7.39E-05	655	0.333333	0.009286	MUL1
2650.695	5.037854	-7068	7.34E-05	639	0.333333	0.008945	PUS7
5156.502	7.229328	-7068	7.42E-05	730	0.333333	0.010848	COPS4
15074.46	6.562925	-7068	7.77E-05	1367	0.333333	0.021715	AK3
8154.159	7.776118	-7068	7.51E-05	887	0.333333	0.013538	JADE1
6221.925	7.050727	-7068	7.49E-05	843	0.333333	0.013154	UBE2Z
4254.14	7.312373	-7068	7.38E-05	642	0.333333	0.009003	IMP3
4794.036	10.55551	-7068	7.32E-05	525	0.333333	0.007039	CDK5RAP3
2476.547	6.844595	-7068	7.33E-05	545	0.333333	0.009178	LSM11
3829.462	7.222561	-7068	7.35E-05	613	0.333333	0.008258	UXT
8123.964	8.495629	-7068	7.45E-05	804	0.333333	0.010925	ARHGAP17
9247.835	7.309181	-7068	7.56E-05	990	0.333333	0.015282	POMP
4597.335	8.320667	-7068	7.36E-05	607	0.333333	0.008609	LRIF1
6893.458	7.514629	-7068	7.49E-05	836	0.333333	0.01299	PLEKHF2
2608.382	8.956938	-7068	7.24E-05	419	0.25	0.005862	LAMP5
3717.7	7.473553	-7068	7.36E-05	618	0.333333	0.009396	AFF4
9849.941	10.21768	-7068	7.38E-05	705	0.333333	0.008043	ZNF587
10819.88	6.913627	-7068	7.61E-05	1116	0.333333	0.016502	YWHAG
14332.31	6.530072	-7068	7.75E-05	1329	0.333333	0.020827	SH3BP4
4842.438	8.293726	-7068	7.36E-05	620	0.333333	0.007968	ECSIT
4285.621	10.52687	-7068	7.29E-05	481	0.333333	0.005643	E4F1
6216.841	7.334508	-7068	7.45E-05	770	0.333333	0.011534	ENOPH1
2995.953	8.020325	-7068	7.31E-05	527	0.333333	0.007638	TINF2
5301.639	6.354373	-7068	7.46E-05	814	0.333333	0.012293	NMD3
4331.212	6.248419	-7068	7.43E-05	749	0.333333	0.01182	FBXO3
10915.05	6.631472	-7068	7.65E-05	1154	0.333333	0.017971	ZRANB2
3927.668	8.124334	-7068	7.36E-05	599	0.25	0.009198	MGAT4A

3168.769	8.50532	-7068	7.31E-05	514	0.333333	0.007951	IFT46
5007.496	6.071166	-7068	7.44E-05	812	0.333333	0.012004	EDN1
2905.171	8.015626	-7068	7.30E-05	516	0.333333	0.007593	EIF4A2
4969.989	7.329002	-7068	7.41E-05	718	0.333333	0.010616	PBRM1
12057.29	8.078526	-7068	7.60E-05	1045	0.333333	0.01593	RPRM
8046.507	9.647954	-7068	7.43E-05	744	0.333333	0.00992	SUV39H1
3116.878	8.935712	-7068	7.30E-05	483	0.333333	0.006993	TAC3
7310.872	7.118452	-7068	7.52E-05	895	0.333333	0.014273	MEAF6
5762.437	6.647424	-7068	7.46E-05	811	0.333333	0.01216	PCNP
7297.767	7.200924	-7068	7.51E-05	884	0.333333	0.013788	ERMP1
5440.876	7.765634	-7068	7.41E-05	713	0.333333	0.010747	RCBTB1
5028.933	8.572996	-7068	7.38E-05	641	0.333333	0.009737	MRPL36
6135.136	8.284754	-7068	7.42E-05	728	0.333333	0.010645	APTX
5803.845	8.457503	-7068	7.39E-05	662	0.333333	0.008427	ATP13A1
10071.52	6.604832	-7068	7.60E-05	1073	0.333333	0.016143	CDC73
6083.531	7.148827	-7068	7.46E-05	807	0.333333	0.01222	ALDH18A1
5046.001	8.92403	-7068	7.37E-05	626	0.333333	0.009159	GEMIN7
5843.134	6.566344	-7068	7.43E-05	799	0.333333	0.010502	GSTA3
12649.28	7.793644	-7068	7.53E-05	1020	0.333333	0.013491	NDUFA8
2923.823	7.604471	-7068	7.30E-05	512	0.333333	0.006949	TIMMDC1
5169.533	7.0351	-7068	7.43E-05	754	0.333333	0.011052	LYRM4
3253.708	6.246621	-7068	7.34E-05	626	0.333333	0.008904	USP39
3748.656	9.657448	-7068	7.30E-05	490	0.333333	0.006422	C11orf68
2354.523	7.602866	-7068	7.29E-05	486	0.333333	0.007228	NOL9
7105.205	7.308316	-7068	7.49E-05	860	0.333333	0.013514	USP16
7446.185	7.946601	-7068	7.48E-05	834	0.333333	0.012736	ATG16L1
6664.527	6.051724	-7068	7.50E-05	939	0.333333	0.013583	SEH1L
9690.281	8.496959	-7068	7.52E-05	901	0.333333	0.013601	L2HGDH
15878.55	6.893852	-7068	7.77E-05	1355	0.333333	0.021195	ZBTB10
6044.367	8.721442	-7068	7.41E-05	689	0.333333	0.009918	MEPCE
3528.352	8.174761	-7068	7.32E-05	544	0.333333	0.007356	FIS1
10808.55	6.5151	-7068	7.64E-05	1153	0.333333	0.018047	AZI2
5598.234	7.403085	-7068	7.42E-05	750	0.333333	0.01087	SIRT1
7095.514	7.432853	-7068	7.48E-05	843	0.333333	0.012604	FANCF
3433.542	6.701267	-7068	7.36E-05	631	0.333333	0.009535	RAB4A
2935.148	7.240946	-7068	7.33E-05	562	0.333333	0.008832	TIFA
7958.995	7.871899	-7068	7.50E-05	866	0.333333	0.012924	PLA2G12A
6077.395	6.597772	-7068	7.45E-05	815	0.333333	0.011823	RBM7
3673.944	7.568369	-7068	7.35E-05	605	0.333333	0.009385	FCRLA
3133.7	10.76189	-7068	7.26E-05	413	0.333333	0.005549	NANOG
7278.58	8.3467	-7068	7.44E-05	770	0.333333	0.011554	ANKRD10

14434.2	8.366519	-7068	7.61E-05	1084	0.333333	0.015862	ZMAT4
4049.935	7.790039	-7068	7.35E-05	612	0.333333	0.008629	GSTM1
4600.444	6.310074	-7068	7.38E-05	727	0.333333	0.008961	SNRNP200
4454.228	7.51174	-7068	7.37E-05	658	0.333333	0.00897	SHMT1
5271.595	7.756149	-7068	7.41E-05	709	0.333333	0.010743	CHRM2
5800.139	8.530154	-7068	7.41E-05	698	0.333333	0.009891	CENPO
6461.83	7.111784	-7068	7.42E-05	798	0.333333	0.010701	GPR37
7350.889	5.108558	-7068	7.53E-05	1050	0.333333	0.01385	NUP107
4450.162	9.634988	-7068	7.33E-05	552	0.333333	0.007649	GTPBP3
4064.52	7.126602	-7068	7.37E-05	640	0.333333	0.008766	TNFSF13
5358.36	7.080172	-7068	7.44E-05	767	0.333333	0.011772	SRP68
6712.716	7.443291	-7068	7.47E-05	813	0.333333	0.012082	SDHAF3
11989.16	7.239835	-7068	7.61E-05	1110	0.333333	0.016272	ISOC1
3632.77	10.04415	-7068	7.27E-05	455	0.333333	0.005692	SOX18
2721.523	7.42322	-7068	7.31E-05	532	0.333333	0.007873	COA7
10220.37	7.065099	-7068	7.61E-05	1078	0.333333	0.016798	THRAP3
2562.777	11.42142	-7068	7.22E-05	346	0.333333	0.004204	SLC16A8
6593.676	7.329655	-7068	7.48E-05	835	0.333333	0.013138	PRPF38B
6839.47	8.652955	-7068	7.43E-05	737	0.333333	0.010587	UCP1
4106.556	6.356114	-7068	7.39E-05	707	0.333333	0.010229	GTF3C3
6751.535	6.61003	-7068	7.50E-05	908	0.333333	0.013724	BRAF
1861.382	7.161071	-7068	7.27E-05	451	0.333333	0.007015	C5orf42
8531.813	6.476068	-7068	7.55E-05	1022	0.333333	0.015052	CORO1C
11512.15	7.308478	-7068	7.62E-05	1088	0.333333	0.01654	DERA
8365.036	7.440026	-7068	7.49E-05	889	0.333333	0.012423	FXYD2
6153.762	6.614651	-7068	7.46E-05	846	0.333333	0.011995	ARF1
2971.353	7.774549	-7068	7.31E-05	531	0.333333	0.007729	ACAD8
8905.394	7.189601	-7068	7.54E-05	961	0.333333	0.013777	TACC3
4177.435	5.563677	-7068	7.40E-05	747	0.333333	0.01029	MRTO4
7625.763	6.602389	-7068	7.56E-05	977	0.333333	0.015575	CCDC88A
21077.94	7.205857	-7068	7.88E-05	1522	0.333333	0.023795	MRPS33
8794.066	6.72775	-7068	7.58E-05	1020	0.333333	0.016046	EXOC2
10156.36	7.048807	-7068	7.59E-05	1045	0.333333	0.015475	PARP12
10229.62	6.386921	-7068	7.62E-05	1139	0.333333	0.017271	MIS18BP1
3975.101	8.63972	-7068	7.33E-05	557	0.333333	0.007759	NOSIP
2318.45	7.111528	-7068	7.30E-05	491	0.333333	0.007565	DCTN4
2444.471	7.491171	-7068	7.30E-05	495	0.333333	0.007777	RBM45
4379.251	7.799444	-7068	7.37E-05	636	0.333333	0.00916	PARL
9845.979	7.230045	-7068	7.55E-05	1000	0.333333	0.014663	MYO5C
14288.55	6.876565	-7068	7.71E-05	1270	0.333333	0.019029	LMCD1
15964.49	11.64799	-7068	7.51E-05	875	0.333333	0.012205	PHLDB1

2414.975	10.31035	-7068	7.24E-05	375	0.333333	0.004733	MRM3
15722.67	6.935705	-7068	7.75E-05	1328	0.333333	0.020796	ANKH
5542.447	12.30037	-7068	7.31E-05	500	0.333333	0.006651	A4GALT
2254.183	9.557003	-7068	7.24E-05	391	0.333333	0.005309	RC3H1
4636.78	9.60004	-7068	7.32E-05	540	0.333333	0.007263	ATRAID
2932.95	9.937576	-7068	7.27E-05	424	0.333333	0.005228	ABCA7
4616.672	7.208302	-7068	7.40E-05	690	0.333333	0.01073	TMEM126B
1790.663	8.212299	-7068	7.23E-05	377	0.333333	0.005161	PSG3
3354.649	9.162279	-7068	7.30E-05	502	0.333333	0.006806	RBM15
6106.701	7.060435	-7068	7.46E-05	803	0.333333	0.012006	TFB2M
9668.471	6.29817	-7068	7.58E-05	1100	0.333333	0.015653	A2M
7203.876	8.191029	-7068	7.46E-05	784	0.333333	0.011899	LRRC40
7774.293	7.874742	-7068	7.50E-05	857	0.333333	0.013197	MYLIP
2628.218	8.54647	-7068	7.27E-05	449	0.333333	0.005883	CTLA4
4376.892	7.448114	-7068	7.39E-05	667	0.333333	0.010378	SNTG1
5387.545	7.980097	-7068	7.41E-05	693	0.333333	0.009761	MLST8
2569.147	8.745946	-7068	7.28E-05	455	0.333333	0.006624	ATP8B2
3713.475	6.335085	-7068	7.38E-05	667	0.333333	0.009497	TOMM22
3626.405	8.102945	-7068	7.33E-05	558	0.333333	0.008138	KCNK5
7458.418	7.398728	-7068	7.50E-05	878	0.333333	0.013554	GREM2
6710.145	7.386534	-7068	7.47E-05	821	0.333333	0.01221	OGFOD1
3734.801	6.759152	-7068	7.41E-05	679	0.333333	0.011583	SNX18
6547.228	7.966582	-7068	7.46E-05	783	0.333333	0.011877	SNX11
5136.345	8.670259	-7068	7.38E-05	640	0.333333	0.009097	SYTL1
9632.233	6.42183	-7068	7.62E-05	1112	0.333333	0.017392	TNS3
1936.261	7.029966	-7068	7.27E-05	443	0.333333	0.006751	DNAJB14
4777.868	7.248	-7068	7.40E-05	691	0.333333	0.010032	MRPL16
6371.913	9.240349	-7068	7.39E-05	683	0.333333	0.010222	TBC1D15
3162.909	7.184182	-7068	7.34E-05	578	0.333333	0.009079	FYTTD1
4099.644	6.782484	-7068	7.41E-05	700	0.333333	0.01123	SLC38A2
9205.22	7.997133	-7068	7.52E-05	905	0.333333	0.013408	GALNT12
5006.542	7.445308	-7068	7.39E-05	686	0.333333	0.009428	SLAMF7
2728.474	4.004958	-7068	7.34E-05	749	0.333333	0.009003	RPS3A
4510.518	6.678241	-7068	7.39E-05	711	0.333333	0.009927	SHCBP1
13425.76	6.173853	-7068	7.70E-05	1318	0.333333	0.01909	RAB7A
4121.434	7.745654	-7068	7.32E-05	596	0.333333	0.00715	MST1
12680.6	7.015059	-7068	7.67E-05	1187	0.333333	0.018213	BCOR
2806.654	8.503455	-7068	7.29E-05	483	0.333333	0.006807	MED25
3375.243	8.861668	-7068	7.32E-05	518	0.333333	0.007693	FCF1
3611.755	10.14733	-7068	7.28E-05	467	0.333333	0.005933	DEDD2
8666.857	7.01157	-7068	7.52E-05	941	0.333333	0.013337	MRPS28

3039.042	8.651097	-7068	7.28E-05	466	0.25	0.006539	SPATA7
3967.808	9.340466	-7068	7.30E-05	514	0.333333	0.00704	DUSP13
2546.763	6.83302	-7068	7.32E-05	541	0.333333	0.008695	ATAD1
4549.586	7.886198	-7068	7.38E-05	647	0.333333	0.010047	SEC22A
8523.466	5.195505	-7068	7.59E-05	1173	0.333333	0.016401	SUPT16H
4813.936	9.308253	-7068	7.35E-05	592	0.333333	0.008629	OXTR
6906.75	7.773415	-7068	7.47E-05	813	0.333333	0.012122	CCDC109B
10743.42	7.830635	-7068	7.59E-05	1016	0.333333	0.015779	WDR59
3282.907	8.854564	-7068	7.31E-05	512	0.333333	0.00727	SLC25A22
4383.095	7.888093	-7068	7.38E-05	625	0.333333	0.009172	CMC2
4575.739	6.527496	-7068	7.40E-05	735	0.333333	0.010643	RAB8B
2472.626	6.30617	-7068	7.31E-05	554	0.333333	0.008274	MORF4L1
4156.454	8.169021	-7068	7.37E-05	614	0.333333	0.009701	STRIP1
6512.96	7.899555	-7068	7.45E-05	777	0.333333	0.011378	ATG101
9230.966	7.170179	-7068	7.57E-05	1001	0.333333	0.014998	NDE1
7522.327	7.125518	-7068	7.50E-05	884	0.333333	0.013251	MAT2B
5142.898	7.550027	-7068	7.40E-05	695	0.333333	0.010101	MED30
12061.21	6.989927	-7068	7.68E-05	1168	0.333333	0.018905	G2E3
4946.004	7.131383	-7068	7.40E-05	709	0.333333	0.009771	MRPL11
4210.035	8.679759	-7068	7.31E-05	558	0.333333	0.007206	LUC7L
7870.057	9.942992	-7068	7.41E-05	712	0.333333	0.009965	SEMA4A
4421.641	7.240504	-7068	7.39E-05	667	0.333333	0.009333	ORMDL2
3898.785	7.387862	-7068	7.34E-05	593	0.333333	0.008182	MUC13
4836.58	9.566729	-7068	7.35E-05	582	0.333333	0.00827	ISYNA1
1740.28	8.513676	-7068	7.25E-05	385	0.333333	0.005864	GJA3
1672.599	8.297124	-7068	7.23E-05	370	0.333333	0.005168	NOL12
3197.974	7.265655	-7068	7.34E-05	588	0.333333	0.009189	WDCP
4492.967	7.71947	-7068	7.39E-05	662	0.333333	0.010114	CRYZL1
6064.789	7.932657	-7068	7.44E-05	754	0.333333	0.011503	FBXO25
7920.415	7.888297	-7068	7.48E-05	852	0.333333	0.012581	RHOU
1850.457	8.360154	-7068	7.24E-05	387	0.333333	0.005396	MRPS15
9112.833	6.780088	-7068	7.50E-05	982	0.333333	0.012938	CENPA
5807.679	7.194811	-7068	7.46E-05	792	0.333333	0.012181	DDX50
11270.08	6.982035	-7068	7.61E-05	1105	0.333333	0.016343	DCUN1D5
3191.312	9.735016	-7068	7.28E-05	459	0.333333	0.006278	PIP4K2C
3857.326	6.861957	-7068	7.37E-05	642	0.333333	0.009436	EMC8
4128.5	7.172532	-7068	7.37E-05	655	0.333333	0.00954	PER3
2025.646	7.277851	-7068	7.28E-05	467	0.333333	0.00741	TTC7A
3040.61	7.064587	-7068	7.35E-05	586	0.333333	0.009591	PLEKHA5
5777.951	7.357717	-7068	7.45E-05	779	0.333333	0.012051	RWDD2B
2724.48	7.842934	-7068	7.30E-05	501	0.333333	0.007315	PLVAP

10746.92	6.869794	-7068	7.63E-05	1112	0.333333	0.017294	FAM46A
3228.705	7.671083	-7068	7.34E-05	569	0.333333	0.008875	OSBPL11
4682.776	7.877794	-7068	7.35E-05	639	0.333333	0.008653	RAD54B
34624.08	6.980397	-7068	8.14E-05	1978	0.333333	0.030523	FOXP1
2622.324	9.473999	-7068	7.26E-05	415	0.333333	0.005824	CYP46A1
5242.932	7.145519	-7068	7.41E-05	734	0.333333	0.010527	NUP54
3362.024	10.94087	-7068	7.27E-05	430	0.333333	0.006141	EN1
2894.232	6.487271	-7068	7.33E-05	597	0.333333	0.008533	PTMA
3955.751	8.961997	-7068	7.32E-05	538	0.333333	0.007386	VPS25
5221.892	7.107476	-7068	7.43E-05	749	0.333333	0.010988	TFB1M
7019.776	6.788246	-7068	7.53E-05	903	0.333333	0.01465	TMEM117
2141.441	10.18939	-7068	7.21E-05	337	0.333333	0.004272	SEMA6B
3905.054	7.941245	-7068	7.33E-05	569	0.333333	0.007492	SLC13A1
2554.276	6.107339	-7068	7.30E-05	544	0.333333	0.007302	SF3B5
4108.539	6.474361	-7068	7.36E-05	639	0.333333	0.007744	MRPL4
7003.089	6.645519	-7068	7.45E-05	869	0.333333	0.011627	RBM28
3259.552	8.090899	-7068	7.32E-05	529	0.333333	0.00784	TCEAL9
3308.807	8.350796	-7068	7.32E-05	542	0.333333	0.008224	UBA6
4332.07	7.695461	-7068	7.36E-05	627	0.333333	0.008944	PALB2
2782.457	7.903766	-7068	7.29E-05	503	0.333333	0.007433	WDYHV1
10907.67	7.49392	-7068	7.62E-05	1051	0.333333	0.016888	ARPP21
4772.168	6.817248	-7068	7.41E-05	728	0.333333	0.010678	EGLN1
3823.18	7.205268	-7068	7.38E-05	651	0.333333	0.010589	PCGF5
11337.65	8.205139	-7068	7.59E-05	1016	0.333333	0.01559	NINJ2
6732.602	7.418192	-7068	7.48E-05	828	0.333333	0.01193	HN1
5271.051	7.5916	-7068	7.41E-05	710	0.333333	0.01029	GLRX2
6161.86	12.26484	-7068	7.32E-05	532	0.333333	0.006679	CPSF1
3019.753	8.20828	-7068	7.31E-05	512	0.333333	0.007762	RNF19A
3965.393	6.28164	-7068	7.38E-05	709	0.333333	0.010003	HSP90B1
2705.622	10.25943	-7068	7.26E-05	406	0.333333	0.005395	BOK
6175.179	7.403015	-7068	7.44E-05	785	0.333333	0.011588	GRHL2
4627.021	8.370214	-7068	7.36E-05	617	0.333333	0.008825	CORIN
8206.232	7.694948	-7068	7.51E-05	887	0.333333	0.013077	CPT2
7498.238	7.168679	-7068	7.51E-05	877	0.333333	0.013047	MRPL13
3336.397	7.360779	-7068	7.33E-05	576	0.333333	0.008754	SLC25A32
2919.253	6.011796	-7068	7.33E-05	617	0.333333	0.008324	RPLP2
5331.408	7.158019	-7068	7.43E-05	760	0.333333	0.011685	RBM22
2874.918	8.857414	-7068	7.29E-05	476	0.333333	0.007255	FAM214A
12606.02	5.483765	-7068	7.72E-05	1388	0.333333	0.020054	DCN
4460.789	7.916413	-7068	7.40E-05	663	0.333333	0.010617	SMUG1
5258.979	8.247236	-7068	7.41E-05	679	0.333333	0.009935	ZDHHC7

11843.14	7.191023	-7068	7.62E-05	1122	0.333333	0.016796	PID1
6957.277	6.900826	-7068	7.48E-05	866	0.333333	0.012491	GAR1
5150.615	8.177914	-7068	7.39E-05	673	0.333333	0.009672	SLC8B1
6404.084	8.053396	-7068	7.45E-05	766	0.333333	0.011154	CNN2
5900.651	9.15404	-7068	7.39E-05	661	0.333333	0.00951	ADRA2B
3878.136	7.647306	-7068	7.37E-05	624	0.333333	0.009635	ELP3
4929.199	7.937158	-7068	7.40E-05	685	0.333333	0.010545	COX15
2672.581	6.819658	-7068	7.30E-05	534	0.333333	0.00754	UTP11
6878.111	11.66862	-7068	7.35E-05	567	0.333333	0.007618	GJD2
13385.7	6.744807	-7068	7.71E-05	1258	0.333333	0.019344	EGLN3
9549.734	12.70351	-7068	7.38E-05	628	0.333333	0.008699	APOL5
3846.108	7.767356	-7068	7.36E-05	609	0.333333	0.009107	ADIPOR1
3332.563	7.112265	-7068	7.36E-05	607	0.333333	0.009278	ARPC5L
3064.807	8.938013	-7068	7.30E-05	493	0.333333	0.00725	FBXL12
2797.2	8.428916	-7068	7.31E-05	493	0.333333	0.007302	PDPR
5184.776	7.152887	-7068	7.45E-05	758	0.333333	0.012328	RMDN1
8855.794	7.637188	-7068	7.53E-05	931	0.333333	0.013994	CTNNBL1
2299.083	10.25401	-7068	7.24E-05	371	0.25	0.004819	SPATA20
10008.77	6.871845	-7068	7.61E-05	1078	0.333333	0.01653	BACE2
6216.902	6.508586	-7068	7.48E-05	858	0.333333	0.012403	GMNN
10877.57	7.042534	-7068	7.63E-05	1102	0.333333	0.017094	PAG1
27306.21	7.151792	-7068	7.94E-05	1691	0.333333	0.024385	ADAMTS9
3189.248	8.532235	-7068	7.30E-05	513	0.333333	0.007351	SPG21
5734.742	7.299231	-7068	7.43E-05	759	0.333333	0.011432	EIF2AK3
9150.198	7.064243	-7068	7.56E-05	998	0.25	0.015058	FLRT3
1816.643	7.694873	-7068	7.25E-05	401	0.333333	0.005412	MRPL20
3025.659	7.667667	-7068	7.34E-05	554	0.333333	0.008697	SENP2
5213.471	8.100047	-7068	7.40E-05	681	0.333333	0.009974	MRPL44
2825.039	6.382372	-7068	7.33E-05	588	0.333333	0.00844	LSM8
6986.507	10.65284	-7068	7.38E-05	625	0.333333	0.008935	IL1RAPL2
5062.436	8.130381	-7068	7.39E-05	669	0.333333	0.010472	GDNF
1760.961	8.323453	-7068	7.25E-05	393	0.333333	0.005704	SLC12A7
26832.29	7.065501	-7068	8.01E-05	1734	0.333333	0.027545	FRMD4A
4006.986	9.07587	-7068	7.32E-05	541	0.333333	0.007487	KLF13
2272.289	10.93234	-7068	7.23E-05	354	0.333333	0.005072	TPTE
7790.886	8.093585	-7068	7.48E-05	825	0.333333	0.012156	FZD4
3532.947	8.181113	-7068	7.33E-05	549	0.333333	0.007757	COQ6
10214.46	8.413754	-7068	7.52E-05	908	0.333333	0.012871	SLC2A9
13477.09	8.1884	-7068	7.63E-05	1089	0.333333	0.016667	HERC1
2091.829	11.51571	-7068	7.21E-05	311	0.333333	0.003651	BIRC7
9358.7	8.611407	-7068	7.50E-05	860	0.333333	0.012194	TP73

3121.576	7.537997	-7068	7.34E-05	565	0.333333	0.008584	CYB5R4
13863.44	7.339632	-7068	7.68E-05	1184	0.333333	0.018298	COMMD10
11323.37	7.032848	-7068	7.62E-05	1116	0.333333	0.01692	FOLH1
6279.08	7.132801	-7068	7.45E-05	786	0.333333	0.010715	MRPS7
264.0626	6.221307	-7068	7.07E-05	174	0.333333	0.002088	MED26
3074.475	10.77385	-7068	7.26E-05	414	0.333333	0.005152	FRS3
10501.22	8.031321	-7068	7.55E-05	964	0.333333	0.013925	FA2H
3246.029	6.6136	-7068	7.32E-05	593	0.333333	0.007939	MYBBP1A
10856.25	7.468453	-7068	7.58E-05	1032	0.333333	0.015561	ST6GALNAC5
4987.816	8.271828	-7068	7.39E-05	664	0.333333	0.00988	PCTP
1953.846	7.462971	-7068	7.26E-05	436	0.333333	0.006561	IMPACT
5077.267	6.711857	-7068	7.44E-05	784	0.25	0.012038	MLYCD
10609.47	8.600122	-7068	7.54E-05	930	0.333333	0.01377	SALL4
10063.36	5.984325	-7068	7.59E-05	1139	0.333333	0.016226	COL5A1
3203.774	6.771921	-7068	7.36E-05	622	0.333333	0.009866	MFF
10674.51	8.589393	-7068	7.54E-05	930	0.333333	0.013488	ADAMTS12
9186.909	8.268255	-7068	7.51E-05	888	0.333333	0.012884	OGDHL
6732.927	6.913965	-7068	7.50E-05	871	0.333333	0.014004	RRM2B
7644.994	7.278056	-7068	7.51E-05	894	0.333333	0.013537	CYBRD1
9127.237	7.392478	-7068	7.55E-05	967	0.333333	0.014427	ANO10
3255.494	10.54342	-7068	7.28E-05	439	0.333333	0.006223	CACNA1A
2591.909	10.06409	-7068	7.25E-05	392	0.333333	0.004928	HCFC1R1
1272.72	7.606214	-7068	7.22E-05	347	0.25	0.005121	CALM3
4794.402	8.056885	-7068	7.39E-05	653	0.333333	0.009691	SCD5
3032.275	5.595371	-7068	7.36E-05	653	0.333333	0.009468	PRMT5
6468.275	4.96163	-7068	7.53E-05	1055	0.333333	0.014885	SRSF2
3642.164	4.034249	-7068	7.40E-05	840	0.333333	0.010149	DKC1
2686.806	9.712316	-7068	7.26E-05	430	0.333333	0.006334	BBS2
7437.553	7.474982	-7068	7.46E-05	836	0.333333	0.011861	SPDL1
8538.131	7.177525	-7068	7.55E-05	958	0.333333	0.014801	MOB3B
3800.113	9.771901	-7068	7.30E-05	493	0.333333	0.007111	SBNO1
3179.302	8.310755	-7068	7.30E-05	509	0.333333	0.006875	GSTM2
3230.237	7.740733	-7068	7.30E-05	523	0.333333	0.007065	PRR15L
4953.277	8.348575	-7068	7.39E-05	652	0.333333	0.009684	SESN1
10479.18	8.039217	-7068	7.57E-05	989	0.333333	0.015321	RUFY1
5681.321	9.649457	-7068	7.31E-05	564	0.333333	0.006786	ZNF286A
7470.143	6.611267	-7068	7.53E-05	957	0.333333	0.014572	MED28
13320.95	7.395571	-7068	7.67E-05	1176	0.333333	0.018077	RASSF4
4806.859	7.382531	-7068	7.41E-05	713	0.333333	0.011046	DNMT3A
18082.2	7.252109	-7068	7.79E-05	1389	0.333333	0.021686	BUD13
14542.65	6.816304	-7068	7.70E-05	1288	0.333333	0.018944	DAB2

2098.397	7.114697	-7068	7.30E-05	485	0.333333	0.007765	ATL2
10031.94	7.045343	-7068	7.61E-05	1060	0.333333	0.016523	MRPL22
6419.458	8.098723	-7068	7.45E-05	761	0.333333	0.011639	PCYOX1L
14163.89	6.54132	-7068	7.72E-05	1309	0.333333	0.019688	TCF7L1
9924.444	13.54388	-7068	7.34E-05	557	0.333333	0.006151	RTBDN
8566.381	7.825368	-7068	7.51E-05	890	0.333333	0.013781	NBEA
4775.345	8.548223	-7068	7.37E-05	625	0.333333	0.009026	HYI
6079.937	7.606433	-7068	7.45E-05	777	0.333333	0.011794	PPTC7
3175.958	7.726922	-7068	7.33E-05	558	0.333333	0.008016	PNPO
5538.984	6.791812	-7068	7.46E-05	815	0.333333	0.012744	BCCIP
4449.935	7.919502	-7068	7.38E-05	644	0.333333	0.009902	HOOK1
3862.001	6.481982	-7068	7.30E-05	605	0.333333	0.007169	NDUFA4
7389.6	7.181833	-7068	7.54E-05	910	0.333333	0.014913	CDKAL1
10137.95	7.025123	-7068	7.60E-05	1056	0.333333	0.016079	CHCHD3
4508.383	7.01681	-7068	7.39E-05	688	0.333333	0.010217	PPIL1
747.1623	7.256941	-7068	7.19E-05	282	0.333333	0.004694	C5orf24
2961.078	10.24602	-7068	7.25E-05	414	0.333333	0.00563	HOXA7
2782.257	7.283672	-7068	7.32E-05	532	0.333333	0.008047	VCPKMT
7150.364	10.09787	-7068	7.37E-05	626	0.333333	0.008043	PITPNM3
6072.347	6.687676	-7068	7.43E-05	801	0.333333	0.010468	CENPM
4043.872	8.434289	-7068	7.34E-05	581	0.333333	0.008354	GALNT6
3110.683	7.761141	-7068	7.32E-05	546	0.333333	0.008321	CLIP4
4455.488	7.574016	-7068	7.38E-05	662	0.333333	0.009895	SMU1
3209.389	8.880021	-7068	7.31E-05	509	0.333333	0.007992	CHST8
7714.969	6.505409	-7068	7.56E-05	989	0.333333	0.01577	PPP1R3B
10650.11	8.543563	-7068	7.50E-05	894	0.333333	0.011978	RAB25
317.6307	7.832024	-7068	7.12E-05	175	0.333333	0.002732	CDR2
7640.433	6.569131	-7068	7.53E-05	960	0.333333	0.014879	INTS7
9581.561	7.415558	-7068	7.57E-05	997	0.333333	0.015436	FAIM
1967.679	6.792734	-7068	7.29E-05	484	0.333333	0.007823	FBLIM1
9162.944	6.709164	-7068	7.56E-05	1034	0.333333	0.015249	LIMA1
6797.275	8.11079	-7068	7.46E-05	784	0.333333	0.011807	NMRK1
3615.312	8.077273	-7068	7.35E-05	584	0.333333	0.008903	VPS37C
3633.964	7.437984	-7068	7.33E-05	582	0.333333	0.008405	TLR7
15705.59	6.917879	-7068	7.75E-05	1333	0.333333	0.020746	PDGFC
8696.464	7.703544	-7068	7.53E-05	918	0.333333	0.014174	DGKI
4725.639	10.43687	-7068	7.31E-05	502	0.333333	0.006273	ATP6V0A4
5564.933	7.32381	-7068	7.39E-05	708	0.333333	0.009211	NCF1
3283.053	8.393155	-7068	7.30E-05	508	0.333333	0.006811	PMF1
3609.027	7.773945	-7068	7.36E-05	594	0.333333	0.009234	WNT5B
2515.748	11.83612	-7068	7.20E-05	322	0.333333	0.003791	DDX4

3590.353	8.292456	-7068	7.32E-05	539	0.333333	0.007373	PTGES2
2615.661	11.59183	-7068	7.23E-05	356	0.333333	0.004371	TRABD
10685.69	6.610269	-7068	7.65E-05	1151	0.333333	0.018249	SYBU
3396.614	7.324508	-7068	7.34E-05	593	0.333333	0.008723	NELFCD
4342.124	10.54696	-7068	7.31E-05	499	0.333333	0.006362	HGH1
3795.897	7.210895	-7068	7.38E-05	637	0.333333	0.010075	USP25
8118.312	7.812042	-7068	7.47E-05	828	0.25	0.011274	SLC7A9
7468.925	7.544291	-7068	7.49E-05	861	0.333333	0.012753	TOLLIP
4646.226	6.828648	-7068	7.41E-05	717	0.333333	0.010863	UBE2W
4683.958	9.793779	-7068	7.33E-05	552	0.333333	0.007484	SLC17A5
3560.833	8.384665	-7068	7.27E-05	498	0.25	0.006746	ZNF655
8958.923	6.495665	-7068	7.57E-05	1041	0.333333	0.015593	ERBIN
3013.908	7.237701	-7068	7.29E-05	515	0.333333	0.005915	CYP4F3
5196.382	7.718675	-7068	7.41E-05	703	0.333333	0.010296	ACSS3
4752.245	7.598704	-7068	7.40E-05	678	0.333333	0.010016	MCCC1
7461.506	7.56112	-7068	7.50E-05	865	0.333333	0.013075	GLRX5
2880.175	7.633185	-7068	7.32E-05	528	0.333333	0.0078	OTUB1
5210.004	7.44785	-7068	7.43E-05	739	0.333333	0.011568	WDR70
6499.504	8.565507	-7068	7.41E-05	703	0.333333	0.009271	FXYD5
11130.04	6.653376	-7068	7.64E-05	1161	0.333333	0.01783	NFATC2
5832.516	7.915314	-7068	7.42E-05	731	0.333333	0.011004	SNCAIP
4222.495	7.616443	-7068	7.37E-05	650	0.333333	0.009442	CISH
4732.667	11.63933	-7068	7.29E-05	460	0.333333	0.005977	EXOC3L2
6765.974	6.80172	-7068	7.47E-05	851	0.333333	0.011884	HLA-C
22644.04	7.437279	-7068	7.88E-05	1520	0.333333	0.024362	RALGPS2
6438.748	7.323094	-7068	7.48E-05	820	0.333333	0.012797	KATNAL1
4108.88	7.627705	-7068	7.39E-05	648	0.333333	0.010196	DNAJC17
5701.173	8.675658	-7068	7.41E-05	686	0.333333	0.01055	RNPC3
3729.935	6.590913	-7068	7.35E-05	626	0.333333	0.008296	EXOSC5
2283.514	11.18764	-7068	7.20E-05	329	0.333333	0.004069	SMR3A
4792.731	8.719481	-7068	7.36E-05	597	0.333333	0.007839	PEF1
1563.138	12.52972	-7068	7.18E-05	255	0.333333	0.003235	FBXO24
2594.526	6.957571	-7068	7.32E-05	539	0.333333	0.008458	HAUS6
8738.723	8.649435	-7068	7.49E-05	837	0.333333	0.011855	MECR
1911.491	6.030475	-7068	7.26E-05	491	0.333333	0.006926	CFL2
4439.458	8.613176	-7068	7.36E-05	604	0.333333	0.008877	GFOD1
5298.459	8.022481	-7068	7.33E-05	618	0.333333	0.007683	ZNF107
4869.371	9.993303	-7068	7.33E-05	537	0.333333	0.007242	PLAC1
2983.714	7.533524	-7068	7.29E-05	508	0.333333	0.006271	TBX21
4542.578	7.309662	-7068	7.38E-05	681	0.333333	0.010196	LUC7L2
6268.411	11.56962	-7068	7.29E-05	504	0.333333	0.004971	TLR9

4725.947	8.621377	-7068	7.37E-05	611	0.333333	0.00886	CPA4
2701.837	7.84675	-7068	7.27E-05	474	0.333333	0.005865	PORCN
20958.1	8.423065	-7068	7.75E-05	1323	0.333333	0.020002	CMSS1
4892.385	5.87397	-7068	7.41E-05	784	0.333333	0.010443	GTPBP4
1571.4	6.138275	-7068	7.23E-05	409	0.333333	0.005132	MRPS2
2358.591	6.513961	-7068	7.31E-05	528	0.333333	0.008196	AP5M1
1454.502	6.763341	-7068	7.22E-05	386	0.333333	0.00527	DERL2
3369.034	8.594963	-7068	7.33E-05	541	0.333333	0.008267	CCAR2
3935.441	7.069594	-7068	7.37E-05	653	0.333333	0.009638	VAV3
10604.78	7.760008	-7068	7.55E-05	981	0.333333	0.014045	TFCP2L1
6165.553	7.371773	-7068	7.46E-05	800	0.333333	0.012545	SGPP1
2196.194	8.556052	-7068	7.27E-05	425	0.333333	0.006709	LRP11
2028.832	8.775555	-7068	7.24E-05	394	0.333333	0.005289	SRCAP
4131.73	6.911803	-7068	7.39E-05	678	0.333333	0.010498	PLEKHA1
6795.138	10.77021	-7068	7.37E-05	621	0.333333	0.008765	LPAR6
6895.571	8.720064	-7068	7.42E-05	719	0.333333	0.009701	RETSAT
7584.239	7.63222	-7068	7.50E-05	877	0.333333	0.013844	RTN3
3169.527	7.670238	-7068	7.31E-05	533	0.333333	0.008029	COMMD8
6514.043	7.595778	-7068	7.46E-05	791	0.333333	0.011986	BACE1
6712.491	8.652106	-7068	7.41E-05	718	0.333333	0.009538	PYCARD
2127.565	12.60549	-7068	7.18E-05	282	0.333333	0.002962	KCNK4
1948.485	9.421247	-7068	7.24E-05	372	0.333333	0.005102	IL37
4037.435	7.48023	-7068	7.38E-05	649	0.333333	0.009357	SOD2
10899.7	7.166155	-7068	7.61E-05	1081	0.333333	0.016758	ANTXR1
3225.594	7.048751	-7068	7.32E-05	568	0.333333	0.008003	PTRH2
5214.582	7.596802	-7068	7.41E-05	710	0.333333	0.010855	BCL11B
5458.665	7.340562	-7068	7.45E-05	771	0.333333	0.012411	PAAF1
6062.228	6.554666	-7068	7.48E-05	871	0.333333	0.013644	SH3KBP1
1041.148	11.14734	-7068	7.16E-05	228	0.333333	0.002902	TMEM164
18977.48	6.585868	-7068	7.87E-05	1526	0.333333	0.024193	ARHGAP21
8787.953	7.628484	-7068	7.54E-05	930	0.333333	0.014778	ALDH1A2
2864.85	10.68121	-7068	7.23E-05	369	0.333333	0.004334	C5AR2
5518.207	8.308866	-7068	7.39E-05	678	0.333333	0.010004	ARAP3
2284.465	9.711706	-7068	7.23E-05	383	0.25	0.005209	GDPD3
11030.53	6.619232	-7068	7.66E-05	1164	0.333333	0.018546	RHOT1
4769.826	8.706064	-7068	7.35E-05	598	0.333333	0.008513	RAB11FIP1
2948.074	6.177096	-7068	7.35E-05	617	0.333333	0.009312	TBK1
23058.49	7.27919	-7068	7.91E-05	1581	0.333333	0.025016	DPF3
3602.406	12.00369	-7068	7.26E-05	397	0.333333	0.004883	CPNE7
2864.251	5.118429	-7068	7.35E-05	689	0.333333	0.009426	ACTG1
3467.266	5.179331	-7068	7.36E-05	699	0.333333	0.009192	NUP85

3257.842	10.96979	-7068	7.27E-05	418	0.333333	0.005359	IFT122
2889.889	8.726087	-7068	7.29E-05	477	0.333333	0.006799	POLR3GL
4665.717	7.65414	-7068	7.39E-05	668	0.333333	0.010191	EPB41L5
9230.858	8.571835	-7068	7.51E-05	877	0.333333	0.01326	LHPP
1921.929	6.597339	-7068	7.29E-05	486	0.333333	0.00739	NECAP2
1438.261	6.950645	-7068	7.24E-05	403	0.333333	0.006254	5-Mar
1711.543	8.275141	-7068	7.24E-05	379	0.333333	0.005113	PGLS
5729.712	9.377954	-7068	7.38E-05	650	0.333333	0.009617	HSD17B12
412.1872	8.268148	-7068	7.12E-05	185	0.333333	0.002651	COMMD7
5562.766	8.18048	-7068	7.41E-05	705	0.333333	0.010215	SLC39A1
2277.918	6.08967	-7068	7.28E-05	519	0.333333	0.006668	UPF3B
674.107	9.567883	-7068	7.16E-05	213	0.333333	0.002989	BAIAP2L2
2649.795	10.41044	-7068	7.25E-05	394	0.333333	0.005167	CLDN15
5748.044	7.286492	-7068	7.43E-05	770	0.333333	0.011216	BRIP1
4926.596	8.450362	-7068	7.39E-05	658	0.333333	0.009993	FAM111A
6031.299	9.113015	-7068	7.41E-05	685	0.333333	0.010646	ATAT1
2535.961	7.301511	-7068	7.27E-05	490	0.333333	0.006657	LYAR
10538.54	5.516624	-7068	7.63E-05	1260	0.333333	0.017402	EIF4A1
4631.485	7.667549	-7068	7.40E-05	681	0.333333	0.010721	CSNK1G1
3505.041	11.35179	-7068	7.27E-05	403	0.333333	0.005056	MZT2B
3413.813	7.194836	-7068	7.30E-05	550	0.25	0.007335	OGN
2837.306	7.452543	-7068	7.30E-05	528	0.333333	0.007722	LY75
6871.834	6.416561	-7068	7.51E-05	925	0.333333	0.01381	TDP1
4592.348	6.991799	-7068	7.40E-05	708	0.333333	0.010281	NEIL3
2428.831	8.845083	-7068	7.25E-05	426	0.333333	0.005678	MRPL34
2995.902	9.352808	-7068	7.26E-05	430	0.333333	0.005775	BEX2
2399.624	7.011175	-7068	7.28E-05	488	0.333333	0.006277	MRPL24
11876.97	7.297368	-7068	7.65E-05	1124	0.333333	0.017365	CCNL1
5331.397	6.418041	-7068	7.42E-05	782	0.333333	0.010356	SUCLG1
6337.463	7.105941	-7068	7.48E-05	840	0.333333	0.012881	BACH1
4387.386	7.910807	-7068	7.36E-05	615	0.333333	0.008548	TNFAIP8L2
3938.491	8.495456	-7068	7.30E-05	522	0.333333	0.006247	SLC22A8
5824.684	8.135258	-7068	7.42E-05	716	0.333333	0.010283	TRIM8
14344.27	8.851034	-7068	7.62E-05	1069	0.333333	0.016896	NKX6-1
7479.439	10.48521	-7068	7.39E-05	631	0.333333	0.008429	HRH2
4421.399	10.39878	-7068	7.29E-05	491	0.333333	0.006086	TMEM40
3779.379	11.08416	-7068	7.29E-05	481	0.333333	0.007149	DHX35
5691.017	7.619139	-7068	7.42E-05	731	0.25	0.010504	KCNJ16
5747.508	7.171792	-7068	7.46E-05	801	0.333333	0.012657	TNIP2
5526.263	6.945322	-7068	7.44E-05	785	0.333333	0.012073	RAB23
5847.397	7.413638	-7068	7.45E-05	781	0.333333	0.012567	FAM135A

4130.372	11.70534	-7068	7.27E-05	434	0.333333	0.005704	FSD1
2009.351	6.70272	-7068	7.29E-05	490	0.333333	0.007646	APPL1
4553.048	5.086586	-7068	7.41E-05	842	0.333333	0.010856	RPL17
10677.88	7.619801	-7068	7.62E-05	1046	0.333333	0.016992	PDE7B
6938.344	7.727817	-7068	7.45E-05	796	0.333333	0.011917	CHPT1
2878.83	6.865026	-7068	7.34E-05	574	0.333333	0.009176	NUFIP2
5174.502	7.458955	-7068	7.41E-05	711	0.333333	0.010477	EGFL7
11483.5	7.278245	-7068	7.61E-05	1085	0.333333	0.016381	AGPAT4
5221.606	8.660553	-7068	7.39E-05	654	0.333333	0.010195	1-Dec
3345.77	9.17968	-7068	7.30E-05	479	0.333333	0.006406	RHOT2
2712.743	6.341412	-7068	7.35E-05	597	0.333333	0.009676	HIGD1A
5209.1	5.246243	-7068	7.43E-05	888	0.333333	0.011954	RPL10
413.1103	9.836153	-7068	7.07E-05	141	0.25	0.001967	SVIP
7161.356	8.164184	-7068	7.46E-05	804	0.333333	0.012465	DNER
6625.275	7.041117	-7068	7.48E-05	848	0.333333	0.012966	SNX10
5894.415	7.43682	-7068	7.45E-05	776	0.333333	0.012093	MRPL48
5820.677	7.867535	-7068	7.44E-05	740	0.333333	0.01158	C11orf1
2260.536	8.712255	-7068	7.25E-05	400	0.333333	0.005287	C1orf116
2228.418	8.788492	-7068	7.25E-05	408	0.333333	0.005769	TPPP3
3380.992	7.868203	-7068	7.26E-05	480	0.333333	0.005251	NDUFA13
2092.538	8.900431	-7068	7.24E-05	394	0.333333	0.005741	PCSK1N
1011.975	7.862574	-7068	7.18E-05	307	0.333333	0.004391	TMEM156
6289.3	8.187882	-7068	7.42E-05	733	0.333333	0.009932	HPR
3748.062	8.529785	-7068	7.32E-05	550	0.333333	0.00762	LXN
3745.287	11.03623	-7068	7.27E-05	435	0.333333	0.005935	ARHGAP28
11045.44	6.684433	-7068	7.62E-05	1138	0.333333	0.017145	RHOJ
2466.998	9.606459	-7068	7.26E-05	411	0.333333	0.005613	PLEKHF1
2217.631	8.105254	-7068	7.25E-05	421	0.333333	0.005251	DVL2
1893.564	6.450719	-7068	7.29E-05	489	0.333333	0.007538	TRMT6
644.1988	9.890611	-7068	7.12E-05	196	0.333333	0.002556	VSTM2L
23964.82	22.73589	-7068	7.41E-05	701	0.333333	0.010745	INTS6
3431.421	6.124799	-7068	7.35E-05	636	0.333333	0.008869	TIPIN
3728.965	8.123732	-7068	7.35E-05	577	0.333333	0.008553	VPS37B
6987.202	7.77855	-7068	7.43E-05	780	0.333333	0.010702	ESRP1
889.0744	10.16002	-7068	7.15E-05	231	0.25	0.003322	EPHX4
2711.493	10.54156	-7068	7.24E-05	381	0.333333	0.005052	SLC17A6
2005.241	10.71879	-7068	7.22E-05	325	0.333333	0.00407	AHSP
8871.955	6.839349	-7068	7.56E-05	1011	0.333333	0.015563	TBX3
2790.645	7.936927	-7068	7.31E-05	518	0.333333	0.007991	MCUR1
16745.79	6.466216	-7068	7.77E-05	1415	0.333333	0.020841	CENPU
7339.92	7.583244	-7068	7.50E-05	854	0.333333	0.013423	INTS2

3373.473	7.69553	-7068	7.30E-05	542	0.333333	0.006953	EXOSC4
2521.05	8.367445	-7068	7.27E-05	455	0.333333	0.006541	SNAPIN
3910.878	8.790523	-7068	7.33E-05	554	0.333333	0.007851	SLC27A3
12714.48	7.045207	-7068	7.68E-05	1199	0.333333	0.019022	IGF2BP2
3028.131	9.88426	-7068	7.27E-05	449	0.25	0.00663	C1QTNF6
9297.055	6.729018	-7068	7.59E-05	1053	0.333333	0.016606	C1GALT1
2275.506	9.663234	-7068	7.24E-05	382	0.333333	0.005433	C9orf40
1560.456	6.828322	-7068	7.23E-05	394	0.333333	0.005635	MTERF3
2296.633	9.074028	-7068	7.27E-05	426	0.333333	0.006468	STAMBPL1
1886.721	8.456277	-7068	7.23E-05	383	0.333333	0.005469	CACNG2
2938.683	9.433155	-7068	7.28E-05	456	0.333333	0.00605	HSD17B7
7408.823	10.16474	-7068	7.39E-05	666	0.333333	0.009473	GRTP1
2508.861	9.915404	-7068	7.25E-05	396	0.333333	0.005349	COA4
11449.3	8.171186	-7068	7.60E-05	1019	0.333333	0.016063	CHODL
6523.06	7.258657	-7068	7.48E-05	833	0.333333	0.013117	GPCPD1
5170.049	7.633808	-7068	7.40E-05	703	0.333333	0.010301	CXCL14
4373.693	8.551416	-7068	7.36E-05	604	0.333333	0.009368	ARID3B
572.2854	10.55896	-7068	7.10E-05	138	0.333333	0.001367	KRTAP13-4
3735.799	7.25937	-7068	7.37E-05	641	0.333333	0.009938	MOCOS
6087.057	9.386784	-7068	7.40E-05	674	0.333333	0.010366	H3F3A
6873.656	8.253093	-7068	7.43E-05	760	0.333333	0.010932	DNAJA4
1820.172	7.909238	-7068	7.23E-05	396	0.333333	0.005387	PPCS
2142.038	11.17124	-7068	7.20E-05	312	0.333333	0.00392	IL19
456.7556	7.536517	-7068	7.12E-05	208	0.333333	0.002826	SYVN1
5693.527	8.535539	-7068	7.41E-05	692	0.333333	0.010133	AP1M1
4235.849	7.105243	-7068	7.38E-05	667	0.333333	0.00982	MANSC1
7117.639	7.732956	-7068	7.49E-05	833	0.333333	0.013276	SH3TC2
11659.23	6.914986	-7068	7.64E-05	1158	0.333333	0.017742	PSAT1
648.7198	8.909828	-7068	7.15E-05	226	0.25	0.003388	ARSG
1908.666	9.062063	-7068	7.24E-05	382	0.333333	0.005326	METRN
8953.746	9.171981	-7068	7.46E-05	794	0.333333	0.011263	HS3ST2
5238.246	7.670685	-7068	7.41E-05	702	0.333333	0.010492	SYTL2
1720.066	8.496369	-7068	7.18E-05	313	0.333333	0.003711	NDUFA11
2615.075	8.041833	-7068	7.27E-05	464	0.333333	0.006136	PRDX5
11540.39	5.87714	-7068	7.68E-05	1266	0.333333	0.019016	ADAMTS1
6061.627	6.467851	-7068	7.50E-05	878	0.333333	0.013571	ECT2
13326.63	7.914084	-7068	7.63E-05	1140	0.333333	0.01744	INCENP
3347.813	8.953009	-7068	7.28E-05	472	0.333333	0.00578	NUBP2
2027.474	6.909919	-7068	7.28E-05	477	0.333333	0.007374	ATG3
1026.472	7.201241	-7068	7.22E-05	340	0.333333	0.005678	AP1S3
4086.332	7.875325	-7068	7.36E-05	612	0.333333	0.009108	ABHD17B

3283.685	6.3598	-7068	7.36E-05	644	0.333333	0.009707	DDR1
8060.071	6.760423	-7068	7.56E-05	972	0.333333	0.015623	GPBP1
2114.519	8.145889	-7068	7.27E-05	430	0.333333	0.006316	FXYD6
7184.681	9.392275	-7068	7.43E-05	719	0.333333	0.010603	OMP
133.7971	7.569973	-7068	7.05E-05	108	0.25	0.001526	SDR42E1
5559.737	7.844195	-7068	7.41E-05	710	0.333333	0.010872	MFSD6
9255.976	7.087843	-7068	7.57E-05	1007	0.333333	0.0158	AGO2
2185.006	7.413279	-7068	7.26E-05	452	0.25	0.006418	CAMK2N1
1528.739	6.18652	-7068	7.24E-05	420	0.333333	0.005411	DDX54
4330.419	10.57457	-7068	7.29E-05	487	0.333333	0.006444	CA14
7297.147	7.682477	-7068	7.49E-05	844	0.333333	0.013344	SDPR
5370.553	11.90885	-7068	7.30E-05	491	0.333333	0.006186	SEMA3B
3198.938	10.96229	-7068	7.25E-05	411	0.333333	0.005214	NT5M
6136.05	7.457419	-7068	7.42E-05	753	0.333333	0.010872	ZWILCH
10195.69	7.634346	-7068	7.58E-05	1005	0.333333	0.015682	NSD1
4050.606	9.062359	-7068	7.32E-05	553	0.333333	0.008185	BANP
3120.976	7.194828	-7068	7.32E-05	552	0.333333	0.007815	TMEM204
5175.45	7.475568	-7068	7.44E-05	739	0.333333	0.011757	DOCK8
99.11542	7.533098	-7068	7.03E-05	94	0.333333	0.001394	POTEF
9386.248	5.657304	-7068	7.60E-05	1164	0.333333	0.016736	SRSF9
9678.934	6.926384	-7068	7.59E-05	1041	0.333333	0.016283	CADPS2
3299.407	9.109633	-7068	7.31E-05	517	0.333333	0.007669	POLR3E
1738.365	6.375546	-7068	7.26E-05	454	0.25	0.006835	TMEM14B
4788.448	9.187971	-7068	7.34E-05	581	0.333333	0.007656	GSTK1
1536.047	9.075199	-7068	7.23E-05	347	0.333333	0.005072	ATG10
6107.905	7.064176	-7068	7.48E-05	824	0.333333	0.013109	XRN1
1553.759	7.012886	-7068	7.26E-05	424	0.333333	0.00707	CDK11A
9353.577	7.302392	-7068	7.55E-05	966	0.333333	0.014651	CDK5RAP2
3339.158	8.824918	-7068	7.32E-05	515	0.333333	0.007717	TRPC5
1844.952	6.142311	-7068	7.28E-05	493	0.333333	0.00764	PRPF38A
3080.573	7.505757	-7068	7.33E-05	560	0.333333	0.008963	PDP1
5132.265	7.019978	-7068	7.42E-05	737	0.333333	0.010592	MS4A6A
3277.136	7.239395	-7068	7.35E-05	591	0.333333	0.009462	PPP1R9A
6061.174	7.125614	-7068	7.46E-05	809	0.333333	0.012139	PDSS1
5137.124	6.109809	-7068	7.44E-05	808	0.333333	0.011517	NCAPG2
12016.93	11.23079	-7068	7.48E-05	810	0.333333	0.012094	GHSR
2681.917	7.813774	-7068	7.30E-05	502	0.333333	0.007329	SDF2L1
1295.363	6.532945	-7068	7.23E-05	390	0.333333	0.005566	QARS
2367.607	9.766313	-7068	7.25E-05	396	0.333333	0.005283	FKBPL
2572.794	10.8707	-7068	7.25E-05	381	0.333333	0.005022	G6PC3
3769.797	9.640687	-7068	7.28E-05	477	0.333333	0.006049	FGF23

4189.637	7.076161	-7068	7.39E-05	674	0.333333	0.009876	PPIA
9450.075	8.870303	-7068	7.50E-05	868	0.333333	0.013008	CLPB
690.5188	8.707122	-7068	7.15E-05	234	0.333333	0.003416	GGNBP2
6230.133	7.308139	-7068	7.44E-05	793	0.333333	0.011616	MRPL46
6180.267	7.995145	-7068	7.44E-05	745	0.333333	0.011001	RNF123
6876.449	7.677891	-7068	7.47E-05	821	0.333333	0.012759	AACS
8691.741	6.449425	-7068	7.56E-05	1042	0.333333	0.015163	TUBA1C
4143.326	8.054334	-7068	7.34E-05	591	0.333333	0.007688	C1RL
5159.921	7.256151	-7068	7.42E-05	739	0.333333	0.011095	UCHL5
12123.29	6.875707	-7068	7.65E-05	1168	0.333333	0.017648	KCNN2
5419.45	7.72313	-7068	7.42E-05	717	0.333333	0.010635	DIABLO
2218.96	7.972157	-7068	7.27E-05	449	0.333333	0.006688	ACSL5
14289.33	6.942746	-7068	7.68E-05	1261	0.333333	0.018095	PPARGC1A
2971.189	7.167176	-7068	7.33E-05	565	0.333333	0.008791	NRN1
3563.645	7.151232	-7068	7.34E-05	602	0.333333	0.008684	EGFL6
2862.933	10.50329	-7068	7.25E-05	398	0.333333	0.004972	TSSC4
8326.553	8.267861	-7068	7.50E-05	860	0.333333	0.013106	SLC35F2
2207.973	6.543058	-7068	7.32E-05	527	0.333333	0.00895	PLEKHH2
3998.382	11.30259	-7068	7.29E-05	455	0.333333	0.00594	CSAD
4662.376	7.157736	-7068	7.40E-05	684	0.333333	0.010217	YIPF5
2535.85	8.801946	-7068	7.27E-05	444	0.333333	0.006798	SLC27A6
1813.778	8.891589	-7068	7.23E-05	371	0.333333	0.005242	FNTB
17123.95	6.861655	-7068	7.81E-05	1416	0.333333	0.022335	CAMK1D
1381.12	8.928138	-7068	7.21E-05	333	0.333333	0.005027	CXXC5
3210.018	8.150229	-7068	7.30E-05	494	0.333333	0.006011	POLDIP2
5193.074	6.916906	-7068	7.43E-05	765	0.333333	0.011632	MCAM
1985.813	7.757073	-7068	7.27E-05	431	0.333333	0.006104	LTBP3
4024.434	8.153182	-7068	7.34E-05	595	0.333333	0.008287	TRIB3
5060.819	11.30105	-7068	7.29E-05	480	0.333333	0.006638	SYT3
2796.785	8.912235	-7068	7.28E-05	451	0.333333	0.005994	CLIC3
4432.807	7.523232	-7068	7.38E-05	657	0.333333	0.00989	PMEPA1
13298.57	6.233089	-7068	7.71E-05	1336	0.333333	0.020058	RUNX1
1898.327	9.734065	-7068	7.22E-05	353	0.333333	0.005027	OLAH
4153.706	8.269067	-7068	7.36E-05	616	0.333333	0.008968	CARHSP1
3005.444	6.975109	-7068	7.33E-05	572	0.333333	0.008238	MTCH2
2510.841	7.330396	-7068	7.31E-05	514	0.333333	0.008209	OTUD6B
4631.786	11.30487	-7068	7.29E-05	469	0.333333	0.006446	LIN28A
2599.776	8.395164	-7068	7.27E-05	460	0.333333	0.006627	CCM2
4066.662	6.319331	-7068	7.35E-05	659	0.333333	0.007591	PECR
2584.466	6.72672	-7068	7.30E-05	512	0.333333	0.006953	MRPS16
2625.032	6.723965	-7068	7.30E-05	546	0.333333	0.007544	ORC6

810.869	5.769507	-7068	7.21E-05	349	0.333333	0.005738	HNRNPLL
11397.1	6.715538	-7068	7.66E-05	1165	0.333333	0.01839	IWS1
2680.335	7.559569	-7068	7.29E-05	491	0.333333	0.007499	TIGAR
6604.639	7.348858	-7068	7.47E-05	822	0.333333	0.012669	GALNT7
7339.328	9.199457	-7068	7.44E-05	729	0.333333	0.010378	PXMP4
10022.59	7.208065	-7068	7.59E-05	1039	0.333333	0.016284	BCAS3
3837.493	8.962433	-7068	7.30E-05	515	0.333333	0.006414	ETNK2
5512.942	8.313185	-7068	7.40E-05	686	0.333333	0.010502	GALNT14
3150.895	10.95021	-7068	7.27E-05	423	0.333333	0.005745	ELP6
2285.674	9.103295	-7068	7.25E-05	398	0.333333	0.005081	TREM2
5559.342	11.50692	-7068	7.30E-05	515	0.333333	0.006355	CERS4
8350.718	7.135241	-7068	7.51E-05	928	0.333333	0.013119	PERP
1286.495	8.721037	-7068	7.19E-05	320	0.333333	0.004736	SCML1
6770.982	5.964594	-7068	7.48E-05	911	0.333333	0.012033	PBK
3170.623	7.863851	-7068	7.31E-05	530	0.333333	0.007467	IL17RB
4194.019	6.779514	-7068	7.40E-05	701	0.333333	0.010625	SUV39H2
4080.374	7.738973	-7068	7.37E-05	625	0.333333	0.00935	TAOK1
3606.461	9.578465	-7068	7.30E-05	490	0.333333	0.00676	C1orf35
1001.336	3.812655	-7068	7.20E-05	446	0.333333	0.004607	RPL13A
1778.137	10.99631	-7068	7.19E-05	298	0.25	0.00383	MS4A12
7711.053	7.082235	-7068	7.52E-05	916	0.333333	0.014387	MEMO1
3243.731	9.311425	-7068	7.30E-05	479	0.333333	0.006814	NKAIN1
3828.112	5.052847	-7068	7.38E-05	763	0.333333	0.009946	FBXO5
4014.639	9.607696	-7068	7.32E-05	519	0.333333	0.006985	TEX264
3152.151	9.798307	-7068	7.29E-05	465	0.333333	0.006547	TRMT2B
1057.489	7.898681	-7068	7.21E-05	320	0.333333	0.005261	PDP2
3185.242	8.505642	-7068	7.29E-05	485	0.333333	0.006093	PREB
5612.295	7.454563	-7068	7.43E-05	752	0.333333	0.012003	ZNF644
4322.499	5.741176	-7068	7.42E-05	773	0.333333	0.010602	EFTUD2
1837.399	8.354685	-7068	7.24E-05	390	0.333333	0.005582	TIMM22
8904.435	7.473353	-7068	7.55E-05	951	0.333333	0.014762	RNLS
4078.01	7.642916	-7068	7.33E-05	606	0.333333	0.007313	VKORC1
633.7327	9.210149	-7068	7.13E-05	204	0.333333	0.002904	NAP1L5
143.1183	2.0151	-7068	6.95E-05	153	0.333333	7.88E-04	ZNF441
3519.653	12.0769	-7068	7.24E-05	389	0.333333	0.005016	NYX
1566.993	8.054725	-7068	7.23E-05	380	0.333333	0.006195	FAM167A
1696.754	7.343522	-7068	7.25E-05	414	0.333333	0.006149	YRDC
4951.319	7.809041	-7068	7.40E-05	684	0.333333	0.01022	VTCN1
1809.001	7.630978	-7068	7.25E-05	411	0.333333	0.005841	C1orf54
7893.096	6.967916	-7068	7.55E-05	955	0.333333	0.01521	SETMAR
10343.71	9.146908	-7068	7.47E-05	827	0.333333	0.010277	HAMP

5029.558	8.387648	-7068	7.38E-05	642	0.333333	0.008705	DCXR
15033.01	7.996007	-7068	7.65E-05	1159	0.333333	0.017256	RAB38
1762.758	8.970852	-7068	7.22E-05	364	0.333333	0.005028	NFS1
2607.197	6.790108	-7068	7.31E-05	541	0.333333	0.007992	DNAJB11
1746.242	7.877299	-7068	7.24E-05	386	0.333333	0.005762	DBR1
2781.945	8.443145	-7068	7.28E-05	473	0.333333	0.006201	EDF1
8173.33	8.954791	-7068	7.46E-05	773	0.333333	0.011234	IL21
4667.939	7.570709	-7068	7.39E-05	677	0.333333	0.010356	TSPAN12
2202.813	7.368885	-7068	7.25E-05	443	0.333333	0.00549	EXOSC1
854.2415	9.593318	-7068	7.14E-05	217	0.333333	0.002878	MXD4
3459.534	8.555183	-7068	7.32E-05	525	0.333333	0.007183	CTDSP1
4800.827	7.408769	-7068	7.42E-05	711	0.333333	0.011183	LGSN
6153.896	7.422539	-7068	7.45E-05	790	0.333333	0.011898	MSRA
2338.649	8.005341	-7068	7.28E-05	462	0.333333	0.006725	NTN1
4360.588	6.760411	-7068	7.38E-05	687	0.333333	0.009636	HLA-B
3427.282	8.088175	-7068	7.32E-05	532	0.333333	0.006865	CORO1B
1208.119	6.795624	-7068	7.22E-05	373	0.333333	0.005666	EEF2K
1893.352	10.78183	-7068	7.21E-05	331	0.25	0.004401	XYLT2
8520.751	7.199967	-7068	7.55E-05	958	0.333333	0.015391	NLGN4X
4439.018	10.72988	-7068	7.31E-05	506	0.333333	0.007333	GP6
12398.95	7.634043	-7068	7.64E-05	1113	0.333333	0.017254	PRSS1
4108.473	7.67613	-7068	7.34E-05	605	0.333333	0.008555	DDX55
2968.744	9.577424	-7068	7.27E-05	445	0.333333	0.00623	BBS1
3506.099	7.587462	-7068	7.33E-05	581	0.333333	0.008445	WDR5
3398.631	7.433177	-7068	7.34E-05	571	0.333333	0.008938	MCTP1
3244.468	9.558172	-7068	7.31E-05	481	0.333333	0.007234	EDA2R
3429.604	8.146499	-7068	7.33E-05	539	0.333333	0.007782	MRPS18C
2436.654	6.355707	-7068	7.33E-05	560	0.333333	0.008807	TMEM33
9751.925	8.084758	-7068	7.55E-05	946	0.333333	0.014923	TM6SF1
4806.019	7.935097	-7068	7.39E-05	668	0.333333	0.010536	SLC39A10
1471.494	7.645336	-7068	7.14E-05	296	0.25	0.002987	RBAK
1170.903	6.98958	-7068	7.22E-05	361	0.333333	0.005448	BRCC3
3828.545	7.350593	-7068	7.36E-05	623	0.333333	0.00949	CDH7
4999.149	9.219187	-7068	7.34E-05	576	0.333333	0.008453	NOTCH2NL
369.5417	9.439337	-7068	7.10E-05	155	0.333333	0.002012	LRRC37A2
1413.572	7.418793	-7068	7.21E-05	354	0.333333	0.004869	CEP83
3614.271	6.906202	-7068	7.37E-05	634	0.333333	0.009909	ARL6
890.8875	7.813865	-7068	7.17E-05	273	0.333333	0.003532	EXOC7
3587.874	6.578148	-7068	7.36E-05	630	0.333333	0.009368	DONSON
2620.114	7.762054	-7068	7.30E-05	495	0.333333	0.007476	LACTB2
3926.506	6.521019	-7068	7.39E-05	689	0.333333	0.010603	RAB6A

6229.232	7.391066	-7068	7.47E-05	815	0.333333	0.01299	ASAP1
871.4045	3.410118	-7068	7.19E-05	439	0.333333	0.004301	RPL7A
3997.571	8.050219	-7068	7.36E-05	611	0.333333	0.009409	SCLY
11149.13	7.837606	-7068	7.59E-05	1034	0.333333	0.015598	NEK6
7836.993	7.587272	-7068	7.49E-05	875	0.333333	0.012952	SPRY4
218.4703	10.64956	-7068	7.06E-05	112	0.333333	0.001555	QSOX2
3775.665	6.681823	-7068	7.35E-05	632	0.333333	0.008328	ASF1B
2232.041	7.248623	-7068	7.26E-05	451	0.333333	0.00592	TIMM50
2209.586	8.130204	-7068	7.26E-05	425	0.333333	0.005788	ROBO4
5728.964	7.040396	-7068	7.44E-05	778	0.333333	0.01166	MRPL1
4047.085	7.577108	-7068	7.38E-05	632	0.333333	0.009849	FBXO34
2152.305	9.120474	-7068	7.24E-05	397	0.333333	0.005564	FKBP11
5610.499	7.863996	-7068	7.43E-05	730	0.333333	0.011038	GSDMD
1795.383	8.203102	-7068	7.24E-05	392	0.333333	0.005734	FGD4
4271.338	7.559816	-7068	7.39E-05	659	0.333333	0.010219	COTL1
2164.732	8.410174	-7068	7.26E-05	427	0.333333	0.006295	LDAH
6459.932	8.143171	-7068	7.43E-05	747	0.333333	0.01122	SMCO4
2504.695	9.7201	-7068	7.26E-05	423	0.333333	0.006105	CYB5R2
4547.536	7.486673	-7068	7.40E-05	683	0.333333	0.010491	ERO1A
1882.215	10.64254	-7068	7.21E-05	327	0.333333	0.004412	IFT22
3347.526	13.07415	-7068	7.24E-05	373	0.333333	0.00503	MTNR1A
8920.418	6.813561	-7068	7.58E-05	1024	0.333333	0.016169	UQCC1
869.8846	9.175988	-7068	7.14E-05	238	0.333333	0.003263	TAF9B
4306.987	7.682142	-7068	7.36E-05	642	0.333333	0.009172	RIPK3
10832.66	9.12917	-7068	7.52E-05	903	0.333333	0.013894	TBC1D7
3154.279	6.966622	-7068	7.36E-05	607	0.333333	0.010113	HDAC8
5363.517	7.615174	-7068	7.42E-05	723	0.333333	0.011384	COA5
1655.199	7.058887	-7068	7.27E-05	431	0.333333	0.007251	TMEM64
2878.359	9.109213	-7068	7.28E-05	461	0.333333	0.006778	LRRC47
3888.546	7.54981	-7068	7.38E-05	630	0.333333	0.009927	ADI1
867.4196	11.48436	-7068	7.14E-05	213	0.333333	0.002997	NUP62CL
1669.056	8.127765	-7068	7.25E-05	394	0.333333	0.005908	BCL2L13
807.2021	9.452835	-7068	7.15E-05	236	0.333333	0.003613	SLC27A1
5893.927	13.77622	-7068	7.30E-05	484	0.333333	0.005913	ZNF358
2862.932	7.456349	-7068	7.33E-05	541	0.333333	0.008404	UBE2R2
5738.002	6.201995	-7068	7.46E-05	851	0.333333	0.012147	PRC1
4657.909	6.369448	-7068	7.45E-05	787	0.333333	0.012868	ERRFI1
5702.044	6.811781	-7068	7.47E-05	819	0.333333	0.01301	FAM107B
4222.404	9.977648	-7068	7.31E-05	530	0.333333	0.007471	DNMT3B
1266.938	10.26579	-7068	7.19E-05	281	0.333333	0.003993	FRMD8
1273.61	7.775005	-7068	7.22E-05	354	0.333333	0.005731	PGM2L1

2518.799	8.192447	-7068	7.26E-05	444	0.333333	0.005507	TIMM13
963.4269	3.654896	-7068	7.21E-05	456	0.333333	0.005352	RPL18A
2036.293	6.091738	-7068	7.28E-05	516	0.333333	0.007088	EEF1B2
3003.453	9.86931	-7068	7.28E-05	451	0.333333	0.006199	TMEM57
2536.866	9.219538	-7068	7.24E-05	403	0.333333	0.005245	ANKRD2
2735.916	5.802249	-7068	7.35E-05	624	0.333333	0.009503	NDC1
726.989	9.331552	-7068	7.17E-05	237	0.333333	0.00374	ORMDL3
2339.372	7.872258	-7068	7.28E-05	465	0.333333	0.0068	MARK4
2286.536	9.634656	-7068	7.24E-05	382	0.333333	0.005282	SLC45A2
2409.585	10.53447	-7068	7.25E-05	378	0.333333	0.005476	PLA2G2E
6900.082	8.182687	-7068	7.45E-05	776	0.333333	0.012311	CFAP36
3624.466	7.842242	-7068	7.35E-05	590	0.333333	0.008892	HK1
2008.819	6.666806	-7068	7.23E-05	436	0.333333	0.00581	CENPQ
2362.662	7.657586	-7068	7.28E-05	477	0.333333	0.007133	ADORA2A
3277.651	8.020821	-7068	7.31E-05	527	0.333333	0.007557	UQCR10
1510.443	9.298753	-7068	7.23E-05	339	0.333333	0.005375	RDH13
2618.565	8.738158	-7068	7.27E-05	448	0.333333	0.005836	NADSYN1
4511.038	7.923526	-7068	7.38E-05	645	0.333333	0.010186	ORMDL1
6147.517	7.485423	-7068	7.44E-05	778	0.333333	0.011446	SGK2
6430.72	7.790011	-7068	7.46E-05	788	0.333333	0.012455	MANEA
603.185	7.081885	-7068	7.12E-05	218	0.333333	0.003347	SLAIN2
731.1344	7.512274	-7068	7.15E-05	259	0.25	0.003693	TBCCD1
3130.954	5.782282	-7068	7.32E-05	628	0.25	0.008267	OLFML3
2693.924	10.29655	-7068	7.26E-05	405	0.333333	0.005775	BEGAIN
1974.711	6.419431	-7068	7.27E-05	469	0.333333	0.006656	MRPS17
5080.623	9.368211	-7068	7.36E-05	605	0.333333	0.009188	CCDC14
2452.573	8.453234	-7068	7.28E-05	455	0.333333	0.007105	ULK3
4941.466	8.224224	-7068	7.38E-05	640	0.333333	0.009961	ACP6
3093.638	8.69669	-7068	7.27E-05	456	0.333333	0.005646	KLK5
3310.042	7.700126	-7068	7.34E-05	568	0.333333	0.008562	RBM47
1418.625	7.040157	-7068	7.25E-05	401	0.333333	0.006448	FGD6
2755.974	7.102046	-7068	7.33E-05	552	0.333333	0.008635	BAG4
3110.166	9.481584	-7068	7.27E-05	458	0.25	0.006556	SULT1E1
930.1714	9.147816	-7068	7.17E-05	263	0.333333	0.003786	SLC38A7
3105.558	6.024349	-7068	7.35E-05	631	0.333333	0.008943	HNRNPH2
3870.094	6.354454	-7068	7.39E-05	695	0.333333	0.010139	BORA
6849.938	7.231908	-7068	7.50E-05	863	0.333333	0.013709	RRP1
8019.268	7.658181	-7068	7.52E-05	906	0.333333	0.014042	DCPS
2805.899	9.395719	-7068	7.27E-05	448	0.333333	0.006286	SLC41A3
1607.964	8.402752	-7068	7.21E-05	346	0.333333	0.004369	INTS5
5040.581	7.131233	-7068	7.40E-05	706	0.25	0.009685	HAO2

3478.558	10.21054	-7068	7.29E-05	475	0.333333	0.006959	ULBP2
6031.466	8.820647	-7068	7.40E-05	687	0.333333	0.010356	ERMN
2478.536	9.462091	-7068	7.26E-05	406	0.333333	0.00571	KCNJ11
1350.486	9.684604	-7068	7.18E-05	278	0.333333	0.003418	IL22
3747.873	9.798199	-7068	7.31E-05	502	0.333333	0.007356	TREML2
247.2737	6.937686	-7068	7.07E-05	158	0.333333	0.002318	PHAX
4682.508	5.911002	-7068	7.40E-05	758	0.333333	0.010406	EXOSC3
1288.819	6.321673	-7068	7.24E-05	412	0.333333	0.006276	HARS
739.6388	12.80506	-7068	7.11E-05	169	0.333333	0.002019	HOXB4
48.40583	8.402391	-7068	6.93E-05	59	0.25	8.87E-04	GALNT4
6414.301	8.97136	-7068	7.40E-05	692	0.333333	0.010623	TSHZ1
5075.071	9.041643	-7068	7.35E-05	606	0.333333	0.009121	ATOH8
3506.833	8.757046	-7068	7.33E-05	540	0.333333	0.008119	IL18BP
9270.009	6.237935	-7068	7.62E-05	1106	0.333333	0.017489	DACT1
2555.611	6.363205	-7068	7.33E-05	573	0.333333	0.009075	CNOT7
1746.825	7.817635	-7068	7.25E-05	406	0.333333	0.006174	WNK2
1523.71	12.4775	-7068	7.16E-05	251	0.333333	0.002881	NPPC
2439.089	7.625373	-7068	7.27E-05	472	0.333333	0.006467	SNRNP25
6859.177	9.068912	-7068	7.42E-05	713	0.333333	0.010269	SIL1
2909.01	7.127322	-7068	7.32E-05	547	0.333333	0.007902	HSPB8
5136.53	7.483211	-7068	7.43E-05	729	0.333333	0.011545	TTC19
2355.409	8.287357	-7068	7.28E-05	453	0.25	0.006928	SLC39A9
2455.16	7.449535	-7068	7.30E-05	499	0.333333	0.007973	TMEM38B
2401.348	8.027923	-7068	7.29E-05	472	0.333333	0.007262	VPS53
8359.566	7.252494	-7068	7.54E-05	942	0.333333	0.014643	SLC40A1
13844.1	9.621101	-7068	7.58E-05	983	0.333333	0.014823	ZNF703
5913.855	11.93091	-7068	7.30E-05	505	0.333333	0.006203	TPSD1
15768.23	6.569452	-7068	7.80E-05	1401	0.333333	0.022482	CREB5
7843.425	6.793594	-7068	7.55E-05	968	0.333333	0.01534	RERG
9345.447	6.677512	-7068	7.60E-05	1063	0.333333	0.016642	XPO5
1061.93	10.90912	-7068	7.15E-05	238	0.333333	0.003078	FAM222B
3682.763	7.504339	-7068	7.36E-05	615	0.333333	0.009416	MID1IP1
5415.363	6.710405	-7068	7.48E-05	819	0.333333	0.013717	CA8
8991.55	7.130679	-7068	7.56E-05	992	0.333333	0.01548	RSRC1
12742.77	6.934692	-7068	7.67E-05	1195	0.333333	0.018287	CYP51A1
5669.553	8.864502	-7068	7.38E-05	637	0.333333	0.009027	LRRC59
1233.854	7.598716	-7068	7.20E-05	339	0.333333	0.004906	NOA1
534.2415	7.456718	-7068	7.14E-05	222	0.333333	0.003441	CCDC117
6400.717	7.536228	-7068	7.46E-05	798	0.333333	0.013153	KLHL12
6879.055	7.328657	-7068	7.47E-05	836	0.333333	0.012168	SLC47A1
5023.225	7.642582	-7068	7.38E-05	678	0.333333	0.009636	TMC5

3738.172	8.190354	-7068	7.34E-05	571	0.333333	0.008609	TMEM132A
9393.859	6.972013	-7068	7.56E-05	1000	0.333333	0.01474	AGXT2
1899.1	10.41261	-7068	7.21E-05	337	0.333333	0.004575	KERA
2415.733	10.09919	-7068	7.19E-05	335	0.333333	0.003248	NDUFA3
4512.728	8.123248	-7068	7.36E-05	615	0.333333	0.007872	G6PD
11293.09	6.039862	-7068	7.65E-05	1224	0.333333	0.018197	PAH
3129.719	6.926855	-7068	7.34E-05	590	0.333333	0.008928	CLDN1
2492.171	7.889444	-7068	7.29E-05	475	0.333333	0.007136	NFU1
1037.109	9.585868	-7068	7.17E-05	264	0.333333	0.003447	ABHD11
1056.399	8.009442	-7068	7.19E-05	298	0.333333	0.004145	SLC35C2
4594.518	7.206361	-7068	7.41E-05	701	0.333333	0.010995	SURF4
5592.296	6.668026	-7068	7.39E-05	761	0.333333	0.009617	C3
699.8032	10.91496	-7068	7.11E-05	182	0.333333	0.00215	SNTG2
969.6827	10.17675	-7068	7.16E-05	242	0.25	0.003371	TEX15
1081.317	11.22086	-7068	7.15E-05	218	0.333333	0.002445	HOXB9
2870.518	8.860821	-7068	7.28E-05	472	0.333333	0.006851	OSBP2
1037.3	13.16386	-7068	7.13E-05	184	0.333333	0.001923	GPX5
9167.186	6.804288	-7068	7.60E-05	1041	0.333333	0.016803	FAR1
1361.688	7.512972	-7068	7.21E-05	365	0.333333	0.005155	SNIP1
3622.925	9.280694	-7068	7.31E-05	506	0.333333	0.006913	AVPI1
3700.874	8.476249	-7068	7.33E-05	546	0.333333	0.007833	SLC34A2
11326.85	8.831982	-7068	7.55E-05	934	0.333333	0.013854	THSD4
3611.042	8.935422	-7068	7.30E-05	498	0.333333	0.00637	RDH5
2227.4	9.021683	-7068	7.25E-05	406	0.333333	0.006084	USP53
733.0088	10.82461	-7068	7.13E-05	194	0.333333	0.002618	SDR9C7
1643.398	30.23898	-7068	6.92E-05	88	0.333333	6.22E-04	OR4C46
2327.735	9.888982	-7068	7.25E-05	388	0.333333	0.00573	FOXP3
7523.408	6.958479	-7068	7.50E-05	897	0.333333	0.013189	NUP35
4326.182	10.47239	-7068	7.31E-05	509	0.333333	0.007389	GSDMB
4836.251	7.783603	-7068	7.36E-05	648	0.333333	0.00826	ECI2
1492.598	9.477913	-7068	7.21E-05	314	0.333333	0.004549	CLDN11
1416.113	8.724393	-7068	7.22E-05	337	0.333333	0.00512	NHLRC2
1643.539	6.52327	-7068	7.25E-05	410	0.333333	0.005223	SLAMF8
9923.279	7.460173	-7068	7.59E-05	1018	0.333333	0.016138	SCO1
4926.15	7.127983	-7068	7.39E-05	699	0.333333	0.009545	C1QA
3582.544	6.945934	-7068	7.35E-05	605	0.333333	0.008433	TREM1
3133.56	7.871257	-7068	7.31E-05	526	0.333333	0.007363	MRPL27
4712.477	7.981862	-7068	7.38E-05	651	0.333333	0.010313	COX16
2987.567	7.735405	-7068	7.32E-05	531	0.333333	0.007689	NPLOC4
3895.992	9.243533	-7068	7.31E-05	512	0.333333	0.007273	BEX3
1631.895	6.538497	-7068	7.24E-05	431	0.333333	0.006181	WDR36

1191.57	8.598618	-7068	7.20E-05	314	0.333333	0.004836	EIF5A2
10119.05	6.287877	-7068	7.60E-05	1114	0.333333	0.01606	NUF2
6564.906	6.859245	-7068	7.42E-05	808	0.333333	0.010494	ORM1
4216.05	10.63941	-7068	7.29E-05	485	0.333333	0.006262	LIME1
1827.048	7.89657	-7068	7.26E-05	418	0.333333	0.006613	GNPNAT1
3189.359	6.191817	-7068	7.34E-05	617	0.333333	0.008519	MS4A7
3365.388	11.01587	-7068	7.28E-05	461	0.333333	0.007477	FBXO11
4871.03	5.904116	-7068	7.40E-05	774	0.333333	0.009839	APOA2
6506.428	8.469073	-7068	7.44E-05	741	0.333333	0.011431	SLC13A4
2843.405	7.067089	-7068	7.33E-05	551	0.333333	0.008088	MRPS18A
5472.465	8.632884	-7068	7.38E-05	647	0.333333	0.008372	CIDEB
3814.82	8.213395	-7068	7.35E-05	574	0.333333	0.008507	PDXP
312.7923	10.82774	-7068	7.05E-05	130	0.333333	0.001612	TMEM107
2931.157	10.14427	-7068	7.29E-05	446	0.333333	0.006312	REEP4
1659.452	8.155716	-7068	7.23E-05	373	0.333333	0.005175	AKT1S1
4433.806	6.025757	-7068	7.41E-05	760	0.333333	0.011041	CKAP2
12577.68	7.082272	-7068	7.65E-05	1166	0.333333	0.017653	NNMT
1972.585	8.396859	-7068	7.25E-05	410	0.333333	0.005933	PRR13
8897.943	6.684971	-7068	7.57E-05	1029	0.333333	0.015563	DEPDC1
8568.883	7.480567	-7068	7.56E-05	942	0.333333	0.015005	PRDM16
8078.864	7.036331	-7068	7.52E-05	924	0.333333	0.014412	SYT7
3328.451	7.130718	-7068	7.36E-05	609	0.333333	0.009553	VMP1
4110.002	7.249201	-7068	7.36E-05	651	0.333333	0.009694	NOD1
4164.395	7.034021	-7068	7.40E-05	685	0.333333	0.010927	ATP8B4
1799.878	4.856345	-7068	7.28E-05	555	0.333333	0.007362	RBMX
3654.962	7.765992	-7068	7.33E-05	579	0.333333	0.008044	TRIP6
2849.629	8.59218	-7068	7.30E-05	489	0.333333	0.007358	SLC25A19
44.96907	8.248968	-7068	6.95E-05	63	0.333333	9.77E-04	SNURF
1325.233	6.779902	-7068	7.24E-05	393	0.333333	0.005998	NSMCE4A
2696.328	9.282103	-7068	7.27E-05	437	0.333333	0.006116	FOXRED2
2582.693	7.09691	-7068	7.32E-05	528	0.333333	0.00829	NKAP
1432.073	8.220432	-7068	7.22E-05	358	0.333333	0.005391	DCAF10
815.2444	13.59926	-7068	7.11E-05	165	0.333333	0.001974	VAX2
2043.095	12.97326	-7068	7.18E-05	267	0.333333	0.003126	ASCL3
5959.966	7.640362	-7068	7.42E-05	739	0.333333	0.010465	RHCG
7849.712	6.923703	-7068	7.55E-05	955	0.333333	0.015469	SMG1
377.342	9.430768	-7068	7.06E-05	146	0.333333	0.001658	DEFB4A
4084.308	8.943429	-7068	7.33E-05	555	0.333333	0.008011	UPK3B
3592.919	7.486977	-7068	7.36E-05	603	0.333333	0.009197	PDCD1LG2
2799.985	7.066693	-7068	7.32E-05	545	0.333333	0.008021	PCOLCE2
4918.018	6.746001	-7068	7.42E-05	756	0.333333	0.011358	RAB10

1078.512	8.772492	-7068	7.20E-05	297	0.333333	0.004492	FBXL20
1429.251	8.421983	-7068	7.19E-05	328	0.333333	0.004891	RNF219
1612.763	6.855359	-7068	7.27E-05	436	0.333333	0.006865	UBE2V1
1620.368	6.555273	-7068	7.27E-05	442	0.333333	0.006923	WBP11
19953.22	7.02068	-7068	7.88E-05	1500	0.333333	0.024289	FMNL2
1910.653	10.72633	-7068	7.20E-05	314	0.25	0.004358	ENPP5
2264.024	7.872431	-7068	7.28E-05	463	0.333333	0.007168	YOD1
1416.673	9.167093	-7068	7.20E-05	325	0.333333	0.004626	NT5DC3
3977.947	8.060926	-7068	7.34E-05	572	0.333333	0.008007	AP1M2
3173.52	9.981102	-7068	7.29E-05	461	0.333333	0.006739	MARS2
1682.264	8.254363	-7068	7.24E-05	385	0.333333	0.005879	RAB2B
2527.194	8.961666	-7068	7.27E-05	433	0.333333	0.006169	BCAN
1417.152	9.382292	-7068	7.19E-05	299	0.333333	0.003905	NTM
7899.961	7.975035	-7068	7.49E-05	849	0.333333	0.013272	IL1RAPL1
2909.476	7.530134	-7068	7.34E-05	547	0.333333	0.009048	WNT10A
387.4317	7.140307	-7068	7.13E-05	199	0.333333	0.002787	EHBP1L1
3448.023	8.343204	-7068	7.33E-05	545	0.333333	0.008138	RTP4
9795.597	8.430985	-7068	7.48E-05	894	0.333333	0.013006	NDUFB4
8065.154	6.948726	-7068	7.53E-05	954	0.333333	0.014556	E2F8
2996.688	7.801964	-7068	7.33E-05	535	0.333333	0.008172	ILKAP
1308.835	9.52176	-7068	7.19E-05	297	0.333333	0.004213	PLPPR2
2359.468	7.715611	-7068	7.28E-05	466	0.333333	0.006573	TNFRSF12A
9832.817	7.616209	-7068	7.58E-05	986	0.333333	0.015828	HRH4
9289.191	7.012216	-7068	7.59E-05	1028	0.333333	0.015946	MGST1
1811.913	6.424867	-7068	7.28E-05	467	0.333333	0.007143	MCOLN3
4329.161	8.050137	-7068	7.37E-05	627	0.25	0.009696	CRB1
630.815	8.097502	-7068	7.13E-05	221	0.333333	0.003177	CISD2
2748.248	7.900109	-7068	7.30E-05	490	0.333333	0.007092	TMX2
1246.317	7.835444	-7068	7.19E-05	315	0.333333	0.003641	XAB2
14597.33	6.921694	-7068	7.76E-05	1295	0.333333	0.020952	VPS13C
2769.043	7.165371	-7068	7.32E-05	543	0.333333	0.008309	PRMT6
3713.689	15.79487	-7068	7.21E-05	345	0.333333	0.004293	ZNF747
1801.394	13.76339	-7068	7.17E-05	244	0.333333	0.00276	TRIM17
1938.931	9.339177	-7068	7.23E-05	363	0.333333	0.005043	NYNRIN
4338.726	12.9705	-7068	7.26E-05	394	0.333333	0.004587	FXYD7
3003.97	9.151126	-7068	7.26E-05	445	0.333333	0.005667	TMPRSS3
6263.469	7.220007	-7068	7.48E-05	833	0.333333	0.013223	UCK2
2604.81	7.859635	-7068	7.30E-05	495	0.333333	0.007453	ESRP2
2798.732	8.023632	-7068	7.31E-05	501	0.333333	0.007475	ABHD6
2942.349	7.905362	-7068	7.29E-05	496	0.333333	0.007451	HEY2
1490.281	7.79978	-7068	7.22E-05	362	0.333333	0.004769	TRAPPC2L

4270.481	7.923145	-7068	7.36E-05	621	0.333333	0.00871	LSM10
1893.678	8.577525	-7068	7.24E-05	395	0.333333	0.005863	SOX17
4393.549	8.530342	-7068	7.35E-05	597	0.333333	0.008779	RIPK4
6077.728	6.533141	-7068	7.48E-05	861	0.333333	0.012912	PABPC4
2523.966	7.049366	-7068	7.33E-05	539	0.333333	0.008736	NGLY1
4183.684	7.552002	-7068	7.38E-05	645	0.333333	0.009808	NT5C3A
4633.416	5.910071	-7068	7.40E-05	763	0.333333	0.009987	CDCA8
1610.11	7.816463	-7068	7.22E-05	360	0.25	0.004375	FTCD
2029.678	7.260654	-7068	7.28E-05	459	0.333333	0.006713	AP2A1
5045.673	7.393049	-7068	7.42E-05	713	0.333333	0.011179	KMT2C
5466.551	7.02821	-7068	7.44E-05	774	0.333333	0.011779	DLL1
4831.031	7.887412	-7068	7.40E-05	667	0.333333	0.010307	AMMECR1L
3782.43	7.376847	-7068	7.36E-05	629	0.333333	0.009481	FIP1L1
2358.621	7.936664	-7068	7.30E-05	478	0.333333	0.007986	POLN
2199.028	15.20178	-7068	7.18E-05	259	0.333333	0.003463	TAF7L
2995.866	6.259312	-7068	7.33E-05	596	0.333333	0.008481	HJURP
3041.948	7.250983	-7068	7.34E-05	575	0.333333	0.009003	GLYR1
2164.721	10.2166	-7068	7.23E-05	361	0.333333	0.005033	TNMD
1483.011	12.3804	-7068	7.14E-05	221	0.25	0.002573	LRRC46
2544.359	7.513343	-7068	7.28E-05	493	0.25	0.007386	SLC44A4
2395.161	10.09525	-7068	7.26E-05	397	0.333333	0.005929	TXNL4B
4337.858	7.603766	-7068	7.37E-05	652	0.333333	0.009583	TUBB6
3709.279	9.52335	-7068	7.30E-05	506	0.333333	0.007113	PSRC1
10899.8	6.723879	-7068	7.63E-05	1128	0.333333	0.017333	RACGAP1
8967.962	8.049528	-7068	7.53E-05	905	0.333333	0.014246	NOX5
7354.152	7.406223	-7068	7.52E-05	888	0.333333	0.014416	EVA1A
6255.028	8.158098	-7068	7.44E-05	747	0.333333	0.011248	STEAP3
3813.952	6.983246	-7068	7.37E-05	639	0.333333	0.00955	CEP55
8301.121	7.169655	-7068	7.55E-05	952	0.333333	0.015227	RSPO3
1825.887	11.05814	-7068	7.20E-05	310	0.333333	0.00378	FBRS
2932.676	7.448666	-7068	7.34E-05	556	0.333333	0.008927	AXIN2
4254.494	6.884479	-7068	7.41E-05	708	0.333333	0.011196	RCC2
3816.197	6.941034	-7068	7.39E-05	661	0.333333	0.010433	PKIB
4883.343	7.802828	-7068	7.40E-05	681	0.333333	0.010182	PAK6
1578.641	7.881212	-7068	7.22E-05	370	0.333333	0.004749	MCOLN1
3213.138	9.788928	-7068	7.28E-05	464	0.333333	0.006922	CHRAC1
3472.203	6.162299	-7068	7.38E-05	677	0.333333	0.010629	TNS1
499.1774	8.324748	-7068	7.11E-05	196	0.333333	0.002478	MMP28
1039.999	11.12018	-7068	7.15E-05	224	0.333333	0.002869	PROK1
15074.05	6.89565	-7068	7.71E-05	1284	0.333333	0.019253	TLR4
1658.926	8.922299	-7068	7.22E-05	356	0.333333	0.005329	SNX24

4199.232	8.459946	-7068	7.34E-05	589	0.333333	0.008424	RBM34
2044.912	8.543375	-7068	7.26E-05	410	0.333333	0.00593	STRA6
3321.575	6.024311	-7068	7.39E-05	679	0.333333	0.010575	ATAD2
8881.365	7.088301	-7068	7.59E-05	1002	0.333333	0.016627	NALCN
3836.703	14.01053	-7068	7.25E-05	380	0.333333	0.005532	NOX3
1267.865	11.15291	-7068	7.18E-05	261	0.333333	0.003505	ZSWIM3
2435.788	6.842715	-7068	7.29E-05	513	0.333333	0.007344	DDX47
1326.593	7.231997	-7068	7.23E-05	376	0.25	0.005937	NTN4
4209.409	8.712003	-7068	7.32E-05	565	0.333333	0.007591	RAB20
1774.014	9.980012	-7068	7.21E-05	334	0.333333	0.004555	ATP13A2
2417.733	12.12659	-7068	7.20E-05	309	0.333333	0.00337	CCL24
3728.775	9.935185	-7068	7.28E-05	478	0.333333	0.006525	CLCF1
1051.972	9.238277	-7068	7.17E-05	260	0.333333	0.003208	USE1
2308.502	8.366725	-7068	7.27E-05	439	0.333333	0.006225	PPP1R13L
2526.005	5.692603	-7068	7.29E-05	558	0.333333	0.007113	KIF18A
5049.316	8.67308	-7068	7.38E-05	623	0.333333	0.009853	ROPN1L
1709.463	9.12341	-7068	7.23E-05	362	0.333333	0.005342	PNPLA3
5760.493	8.28035	-7068	7.40E-05	702	0.333333	0.01062	TRPC4
3995.206	7.198385	-7068	7.39E-05	660	0.333333	0.010259	DEPTOR
1138.893	10.82404	-7068	7.16E-05	240	0.333333	0.003447	SIX5
3213.982	7.04324	-7068	7.35E-05	603	0.333333	0.009714	EPC1
4585.93	8.068429	-7068	7.39E-05	651	0.333333	0.010135	CYP2R1
3525.123	7.920596	-7068	7.35E-05	574	0.333333	0.009089	MAK
2971.034	8.669154	-7068	7.30E-05	487	0.333333	0.007138	PAQR5
1601.254	8.261806	-7068	7.23E-05	378	0.333333	0.006081	ZKSCAN2
1383.145	7.256461	-7068	7.21E-05	365	0.333333	0.005356	BRD7
2400.626	10.09388	-7068	7.26E-05	408	0.333333	0.006029	DBF4B
6949.998	6.726064	-7068	7.53E-05	923	0.333333	0.014904	PPHLN1
1892.501	7.827557	-7068	7.24E-05	402	0.333333	0.005434	SDHAF2
2811.649	7.790856	-7068	7.31E-05	519	0.333333	0.007906	ANO1
3686.289	8.73825	-7068	7.31E-05	526	0.333333	0.007772	HAUS1
995.3308	4.93857	-7068	7.22E-05	403	0.333333	0.005175	RPL28
3191.326	6.165039	-7068	7.32E-05	624	0.333333	0.008389	EEF1G
1851.908	10.43427	-7068	7.20E-05	320	0.333333	0.004076	TBX4
1190.199	13.8221	-7068	7.13E-05	201	0.25	0.002489	ZCWPW1
6799.583	6.900667	-7068	7.50E-05	886	0.333333	0.013935	ANTXR2
1189.44	8.352559	-7068	7.19E-05	305	0.333333	0.004197	PPP1R1B
2937.313	6.730134	-7068	7.34E-05	593	0.333333	0.009038	CD302
7285.41	6.748775	-7068	7.54E-05	937	0.333333	0.015303	STXBP6
195.0881	10.86053	-7068	7.00E-05	93	0.333333	0.001321	GXYLT1
103.3929	11.161	-7068	6.98E-05	64	0.333333	8.88E-04	B3GLCT

2178.774	7.889766	-7068	7.27E-05	453	0.333333	0.006931	RBM23
2589.901	8.103071	-7068	7.29E-05	480	0.333333	0.007515	ALG1
2428.351	8.635999	-7068	7.27E-05	442	0.333333	0.006502	TSPAN13
2180.342	7.641141	-7068	7.24E-05	414	0.333333	0.004717	RETN
2640.556	8.737885	-7068	7.23E-05	401	0.333333	0.004112	PRG3
3718.237	8.260785	-7068	7.36E-05	577	0.333333	0.009132	MAP1LC3C
3675.587	7.493026	-7068	7.34E-05	599	0.333333	0.008711	LIPG
1275.164	8.10194	-7068	7.21E-05	324	0.333333	0.004428	DGAT2
3273.326	7.746855	-7068	7.33E-05	543	0.333333	0.007535	SCPEP1
1786.087	7.540074	-7068	7.26E-05	422	0.333333	0.006444	CAB39L
8996.424	7.176374	-7068	7.57E-05	986	0.333333	0.01605	WDSUB1
3014.72	5.581611	-7068	7.35E-05	644	0.333333	0.008838	DTL
1494.251	7.605488	-7068	7.23E-05	378	0.333333	0.005736	NIPSNAP3B
1359.068	6.977226	-7068	7.11E-05	268	0.25	0.002041	ZNF225
5601.337	7.309746	-7068	7.45E-05	776	0.333333	0.012183	GJB2
4153.393	8.026765	-7068	7.32E-05	592	0.333333	0.007378	PRR11
7252.227	7.809927	-7068	7.48E-05	829	0.333333	0.012117	ATPIF1
3583.255	5.136315	-7068	7.37E-05	726	0.333333	0.00942	NUSAP1
2159.655	8.068607	-7068	7.25E-05	431	0.25	0.006064	RBKS
926.2656	6.14732	-7068	7.18E-05	316	0.25	0.003864	GSTA2
338.051	8.380142	-7068	7.09E-05	166	0.333333	0.002406	PTPMT1
3200.427	8.456363	-7068	7.28E-05	483	0.333333	0.006365	NAA20
1652.387	8.306831	-7068	7.23E-05	368	0.333333	0.005323	DMKN
10359.46	7.225066	-7068	7.61E-05	1058	0.333333	0.01681	STT3B
3905.167	9.726651	-7068	7.31E-05	497	0.333333	0.00722	TRPM4
3547.306	6.749711	-7068	7.36E-05	638	0.333333	0.009746	RGCC
2084.575	7.378883	-7068	7.28E-05	456	0.333333	0.006975	RAB33B
1226.151	7.761807	-7068	7.20E-05	331	0.333333	0.004613	TRUB2
2255.341	8.602144	-7068	7.26E-05	402	0.333333	0.005311	CALML5
2572.128	9.298326	-7068	7.26E-05	419	0.333333	0.006256	CCDC170
2382.392	6.931435	-7068	7.30E-05	517	0.333333	0.007843	ARL5A
770.5503	7.348816	-7068	7.18E-05	284	0.333333	0.004211	ALG9
983.4469	12.61367	-7068	7.13E-05	202	0.333333	0.002285	ESPN
4578.283	8.368774	-7068	7.32E-05	566	0.333333	0.006672	CRYL1
3268.795	7.889515	-7068	7.32E-05	538	0.333333	0.007703	LYVE1
4977.636	9.969278	-7068	7.34E-05	559	0.333333	0.008144	CHRM1
8333.579	7.513334	-7068	7.51E-05	908	0.333333	0.013786	LNX1
652.3467	7.857739	-7068	7.16E-05	247	0.333333	0.00394	STEAP2
560.7469	7.19139	-7068	7.13E-05	222	0.333333	0.002862	ZC3H12A
2669.645	6.835901	-7068	7.28E-05	522	0.333333	0.007072	EHF
7488.161	6.770064	-7068	7.55E-05	947	0.333333	0.015225	SPATS2L

2044.129	7.90341	-7068	7.27E-05	438	0.333333	0.006732	HKDC1
2637.008	7.643028	-7068	7.31E-05	510	0.333333	0.007896	BRI3BP
2981.618	12.36504	-7068	7.23E-05	374	0.25	0.005353	FAXDC2
4635.858	7.164542	-7068	7.42E-05	711	0.333333	0.01128	EPB41L4A
4402.941	8.431297	-7068	7.38E-05	616	0.333333	0.009398	TBC1D2
284.7679	9.048195	-7068	7.08E-05	143	0.25	0.001956	SLC25A27
2947.435	9.532109	-7068	7.20E-05	368	0.333333	0.003905	ZFP14
5282.628	11.22704	-7068	7.24E-05	451	0.333333	0.004203	ZNF611
1969.335	8.537291	-7068	7.26E-05	397	0.333333	0.00595	CTRB1
1555.68	11.00624	-7068	7.17E-05	273	0.333333	0.003052	WDR24
24501.02	7.263183	-7068	7.98E-05	1639	0.333333	0.027113	HS3ST4
1755.969	8.338037	-7068	7.24E-05	389	0.25	0.005644	C3orf52
182.7004	6.732438	-7068	7.08E-05	149	0.25	0.002438	CA13
7106.608	10.45782	-7068	7.38E-05	651	0.333333	0.009627	RBP2
1520.973	8.58307	-7068	7.23E-05	361	0.333333	0.005424	ADCK2
12948.84	7.199854	-7068	7.69E-05	1188	0.333333	0.019163	MTHFD1L
5091.081	9.328934	-7068	7.32E-05	554	0.333333	0.006609	GPR87
1078.137	11.65267	-7068	7.13E-05	202	0.25	0.002124	GSC2
3024.48	10.04783	-7068	7.26E-05	420	0.333333	0.005111	SIGIRR
2063.358	11.38228	-7068	7.21E-05	321	0.333333	0.004381	ADAP1
2080.317	8.339949	-7068	7.24E-05	407	0.333333	0.00548	NOX4
5060.954	7.545074	-7068	7.38E-05	679	0.333333	0.009199	ABCG5
2862.801	9.688825	-7068	7.24E-05	419	0.333333	0.005539	PILRA
13871.49	6.641133	-7068	7.74E-05	1297	0.333333	0.020919	TMEM135
3614.549	7.94569	-7068	7.33E-05	576	0.333333	0.008449	COX17
2681.534	11.18576	-7068	7.22E-05	357	0.333333	0.00466	MAGIX
2902.369	8.522199	-7068	7.28E-05	484	0.333333	0.006408	MSRB1
7436.201	7.290967	-7068	7.52E-05	884	0.333333	0.014521	HERC3
6772.062	7.601966	-7068	7.47E-05	813	0.333333	0.012519	SEMA6D
2930.173	9.368886	-7068	7.27E-05	450	0.333333	0.006561	DCP1B
5271.872	8.063271	-7068	7.41E-05	689	0.333333	0.011298	SPTSSB
1998.164	10.38915	-7068	7.21E-05	335	0.333333	0.004366	PIGZ
591.9717	7.91592	-7068	7.16E-05	236	0.333333	0.003755	ADIG
2020.927	10.99732	-7068	7.21E-05	322	0.333333	0.004447	INSRR
5760.349	9.412423	-7068	7.35E-05	634	0.333333	0.008921	NDUFS8
1275.001	7.984957	-7068	7.18E-05	313	0.333333	0.003818	HSH2D
5296.597	8.275863	-7068	7.41E-05	692	0.333333	0.01078	NABP1
5663.722	7.286759	-7068	7.44E-05	765	0.333333	0.011519	PSTPIP2
3047.12	8.682075	-7068	7.30E-05	492	0.333333	0.007001	ZDHHC5
3562.262	8.120754	-7068	7.25E-05	466	0.333333	0.005255	ZNF675
3315.343	6.672882	-7068	7.35E-05	621	0.25	0.009205	DRAM1

2153.106	10.60058	-7068	7.22E-05	356	0.333333	0.00526	HOXA3
4041.99	7.788224	-7068	7.36E-05	620	0.333333	0.009792	CRISPLD1
9906.201	9.436387	-7068	7.48E-05	855	0.333333	0.012723	NDUFA10
1429.168	12.62365	-7068	7.17E-05	245	0.333333	0.00325	PRSS50
6192.501	6.881419	-7068	7.46E-05	828	0.333333	0.012592	GRHL1
2650.051	9.682939	-7068	7.24E-05	400	0.333333	0.005279	HEMGN
1456.202	8.591576	-7068	7.21E-05	348	0.333333	0.005198	NCOA5
2916.781	10.74498	-7068	7.26E-05	394	0.333333	0.005315	RASAL1
5049.235	7.36169	-7068	7.40E-05	711	0.333333	0.010447	RNF128
6044.17	8.231536	-7068	7.41E-05	710	0.333333	0.010831	TMEM45B
836.395	9.869204	-7068	7.14E-05	232	0.333333	0.003183	ELP5
76.46297	8.143088	-7068	7.02E-05	80	0.333333	0.001192	AMOTL1
5901.533	7.017354	-7068	7.46E-05	802	0.333333	0.012551	COLEC12
4050.028	7.195405	-7068	7.37E-05	654	0.333333	0.009726	F11R
82.23272	6.956552	-7068	7.00E-05	93	0.25	0.001451	SPIN4
6518.049	7.937137	-7068	7.44E-05	763	0.333333	0.011877	LRRC1
1492.585	6.673868	-7068	7.25E-05	428	0.333333	0.006537	STK26
12499.63	34.24511	-7068	7.22E-05	332	0.333333	0.003898	OR10J1
4923.48	5.455817	-7068	7.43E-05	828	0.333333	0.011141	UBE2T
2649.49	7.891384	-7068	7.30E-05	491	0.333333	0.007398	GNPTG
1877.802	8.660895	-7068	7.23E-05	380	0.333333	0.005174	ATP5E
2867.337	10.732	-7068	7.24E-05	386	0.333333	0.004831	KRT24
16498.46	7.85526	-7068	7.73E-05	1258	0.333333	0.019922	TRPM3
2902.135	9.237233	-7068	7.28E-05	452	0.333333	0.006243	MAP1LC3A
1780.659	7.160914	-7068	7.26E-05	429	0.333333	0.006495	CDCA4
1811.704	7.456956	-7068	7.27E-05	433	0.333333	0.006835	ATPAF1
7569.632	6.954469	-7068	7.53E-05	924	0.333333	0.014784	RAB18
643.7224	11.83332	-7068	7.13E-05	169	0.333333	0.001962	FN3K
5996.196	7.391827	-7068	7.46E-05	787	0.333333	0.012767	CCDC113
5638.753	7.577258	-7068	7.43E-05	750	0.333333	0.011322	EMILIN2
1354.413	8.465711	-7068	7.20E-05	331	0.333333	0.004466	IRAK4
1139.433	6.764252	-7068	7.21E-05	349	0.333333	0.005153	DNAAF5
4282.705	7.389514	-7068	7.37E-05	660	0.333333	0.009823	DDX58
1695.605	9.424548	-7068	7.22E-05	338	0.333333	0.004438	PGPEP1
6395.364	5.596002	-7068	7.52E-05	973	0.333333	0.014799	COL12A1
2655.777	10.47478	-7068	7.23E-05	372	0.333333	0.004746	FOXL2
3885.124	8.110559	-7068	7.35E-05	581	0.333333	0.008487	SNAI1
247.3002	10.63524	-7068	7.08E-05	117	0.333333	0.001654	TRABD2A
3119.736	8.861644	-7068	7.29E-05	477	0.333333	0.006733	CES3
81.22014	11.14371	-7068	6.65E-05	53	0.25	4.51E-04	WFDC13
15936.57	30.11043	-7068	7.26E-05	409	0.333333	0.00428	OR3A1

2699.585	6.826404	-7068	7.31E-05	544	0.333333	0.007919	COL5A3
2681.074	5.150601	-7068	7.35E-05	644	0.333333	0.009105	GINS2
3093.269	10.20912	-7068	7.28E-05	444	0.333333	0.006937	GKAP1
2905.706	8.100208	-7068	7.31E-05	516	0.333333	0.00786	GRAMD1A
5248.625	9.741766	-7068	7.36E-05	599	0.333333	0.009377	DCAKD
1319.476	10.24172	-7068	7.18E-05	286	0.333333	0.004087	BHLHB9
9058.123	7.557911	-7068	7.54E-05	947	0.333333	0.014245	HIBADH
3739.285	9.573031	-7068	7.31E-05	505	0.333333	0.007616	IRF2BPL
1697.619	7.837598	-7068	7.25E-05	402	0.333333	0.006401	STRBP
318.1972	9.653771	-7068	7.09E-05	142	0.333333	0.001719	PGP
2020.853	10.25088	-7068	7.21E-05	335	0.333333	0.00423	B4GALT7
1478.957	8.290106	-7068	7.21E-05	344	0.333333	0.004852	TLR10
1648.444	7.340776	-7068	7.25E-05	418	0.333333	0.006627	CHMP3
1314.519	10.81818	-7068	7.19E-05	271	0.333333	0.003417	SLC35F6
1018.755	8.624957	-7068	7.20E-05	292	0.333333	0.004545	ARMC9
11812.03	6.681915	-7068	7.68E-05	1189	0.333333	0.018857	CRIM1
2842.894	8.829537	-7068	7.29E-05	466	0.333333	0.006938	ELMO3
906.9234	9.224699	-7068	7.17E-05	247	0.333333	0.003178	LRAT
3207.643	11.36242	-7068	7.24E-05	381	0.333333	0.004919	GPR27
2665.216	7.572634	-7068	7.31E-05	521	0.333333	0.008007	CCNK
2058.908	6.609145	-7068	7.29E-05	502	0.333333	0.007672	FADS1
3302.237	7.155881	-7068	7.36E-05	604	0.333333	0.009768	STARD4
1871.68	7.716461	-7068	7.26E-05	419	0.333333	0.006462	CCPG1
4459.284	6.448452	-7068	7.43E-05	755	0.333333	0.012269	FBXO30
600.6708	7.33459	-7068	7.16E-05	239	0.333333	0.003471	KRT6C
2355.352	6.125611	-7068	7.28E-05	506	0.333333	0.006196	KRT6B
1194.853	8.665001	-7068	7.20E-05	311	0.333333	0.004608	CCDC97
3653.403	7.626211	-7068	7.37E-05	616	0.333333	0.009764	HPS6
5334.64	8.158787	-7068	7.39E-05	669	0.333333	0.010224	OTX2
855.7083	7.09097	-7068	7.18E-05	300	0.333333	0.004453	KCTD10
1401.738	9.129126	-7068	7.21E-05	330	0.333333	0.005055	ZBTB2
5607.68	8.818958	-7068	7.39E-05	659	0.25	0.009738	ADGRF1
1302.646	10.60613	-7068	7.19E-05	271	0.333333	0.003673	SEC14L4
2888.002	8.864511	-7068	7.30E-05	477	0.333333	0.007561	CHRNA9
97.45671	8.152905	-7068	6.96E-05	81	0.25	9.75E-04	HBG2
721.362	6.498526	-7068	7.16E-05	277	0.25	0.003752	KLRK1
1899.961	6.948117	-7068	7.29E-05	473	0.333333	0.008037	LCLAT1
1615.119	9.086307	-7068	7.21E-05	341	0.333333	0.004536	SIRT6
6063.56	7.689778	-7068	7.45E-05	771	0.333333	0.011518	ATG7
2234.034	7.731155	-7068	7.28E-05	459	0.333333	0.006993	SEC11C
6405.904	6.760638	-7068	7.49E-05	865	0.333333	0.013692	CLEC3B

3329.359	5.912469	-7068	7.37E-05	654	0.333333	0.009324	KIF4A
2684.826	7.890311	-7068	7.30E-05	502	0.333333	0.007736	DNAJC27
2392.856	7.485309	-7068	7.29E-05	475	0.333333	0.006517	MAP7D1
800.0123	7.705124	-7068	7.17E-05	265	0.333333	0.003919	KANK2
1331.227	12.67941	-7068	7.15E-05	224	0.333333	0.002606	VSX1
1756.241	10.72372	-7068	7.21E-05	316	0.333333	0.004088	ACAD10
860.9461	8.362039	-7068	7.14E-05	242	0.333333	0.002925	ARL6IP4
2965.584	7.53389	-7068	7.34E-05	559	0.333333	0.009158	NTPCR
3671.446	8.629958	-7068	7.33E-05	549	0.333333	0.0082	SYAP1
4051.041	6.890631	-7068	7.40E-05	690	0.333333	0.010652	DIAPH3
8017.983	7.689237	-7068	7.52E-05	887	0.333333	0.014039	PALMD
1896.602	7.485995	-7068	7.27E-05	439	0.333333	0.006589	C1orf115
3103.293	7.374475	-7068	7.34E-05	575	0.333333	0.009455	SCN3A
2059.756	7.343047	-7068	7.27E-05	460	0.333333	0.006985	DERL1
1962.511	8.058389	-7068	7.25E-05	407	0.333333	0.005583	STEAP4
1850.656	9.548387	-7068	7.23E-05	363	0.333333	0.005227	HILPDA
7144.9	7.010112	-7068	7.52E-05	898	0.333333	0.014406	ARRDC3
823.3722	7.863437	-7068	7.18E-05	280	0.333333	0.004204	NDUFAF7
1816.742	9.195367	-7068	7.20E-05	316	0.333333	0.003287	NPHS2
2050.428	9.016573	-7068	7.24E-05	388	0.333333	0.005648	UNK
1709.391	6.974625	-7068	7.25E-05	426	0.333333	0.006093	VPS35
2467.438	9.838404	-7068	7.25E-05	398	0.333333	0.005302	DPP7
573.304	9.485885	-7068	7.14E-05	197	0.333333	0.003106	SH3PXD2B
5368.025	6.968601	-7068	7.46E-05	786	0.333333	0.012871	CTTNBP2NL
1602.038	8.575855	-7068	7.23E-05	367	0.25	0.005588	THEM4
263.5334	7.832609	-7068	7.11E-05	160	0.333333	0.002494	FITM2
5280.415	8.884398	-7068	7.39E-05	639	0.333333	0.00912	SLC25A10
373.7227	10.75475	-7068	7.05E-05	122	0.25	0.001189	KIR2DS1
3739.508	9.842989	-7068	7.26E-05	421	0.333333	0.004273	KIR2DS2
3092.559	5.850835	-7068	7.34E-05	652	0.25	0.009122	MCM8
8659.552	6.728845	-7068	7.56E-05	1002	0.333333	0.015618	GPAT3
3069.736	7.894492	-7068	7.31E-05	526	0.333333	0.007828	GDA
1601.978	8.500206	-7068	7.22E-05	350	0.333333	0.004828	ADGRE3
1961.995	12.77908	-7068	7.20E-05	292	0.333333	0.003655	TIGD5
3242.721	13.29748	-7068	7.22E-05	334	0.333333	0.003704	MEFV
1814.512	7.78978	-7068	7.15E-05	304	0.333333	0.002513	ZNF256
5086.295	8.253136	-7068	7.40E-05	673	0.333333	0.01061	ZFP91
11365.38	7.534873	-7068	7.63E-05	1074	0.333333	0.01742	LRRN1
1986.524	9.808483	-7068	7.23E-05	366	0.25	0.005533	TRIM35
1764.783	6.751605	-7068	7.28E-05	458	0.333333	0.007253	PBDC1
331.2128	8.0033	-7068	7.11E-05	171	0.333333	0.002674	ZDHHC2

11117.57	7.789956	-7068	7.61E-05	1041	0.333333	0.016502	ATE1
7946.86	7.037032	-7068	7.55E-05	948	0.333333	0.0151	ARID1B
3477.477	9.123672	-7068	7.29E-05	492	0.333333	0.006508	TAPBPL
1792.389	7.104276	-7068	7.26E-05	432	0.333333	0.00645	AP3M1
319.5726	8.133471	-7068	7.10E-05	164	0.333333	0.002384	KANSL1L
5414.801	9.011372	-7068	7.33E-05	593	0.333333	0.008114	ZNF44
4981.854	6.881805	-7068	7.44E-05	759	0.333333	0.012017	CARD11
4219.997	7.629215	-7068	7.39E-05	649	0.333333	0.010607	GALNT13
3989.007	8.356803	-7068	7.34E-05	577	0.333333	0.008253	ANGPTL4
1774.366	6.541682	-7068	7.25E-05	456	0.333333	0.006461	TNFRSF10A
3287.95	7.874065	-7068	7.34E-05	562	0.333333	0.008905	WWC2
6201.017	6.938886	-7068	7.49E-05	849	0.333333	0.013713	CYYR1
775.777	8.29007	-7068	7.16E-05	254	0.333333	0.003672	PAGR1
45.78407	6.718994	-7068	6.87E-05	61	0.25	6.74E-04	CGB3
1930.968	8.83121	-7068	7.20E-05	336	0.333333	0.003847	LHB
793.6178	10.71879	-7068	7.15E-05	212	0.333333	0.002957	SLC30A6
795.1546	7.383355	-7068	7.18E-05	287	0.333333	0.00429	PCSK6
2430.203	7.793973	-7068	7.28E-05	468	0.333333	0.006676	CYP21A2
749.8226	10.02365	-7068	7.15E-05	206	0.333333	0.002323	SCT
1011.946	10.95071	-7068	7.17E-05	241	0.333333	0.003285	KCNK12
1451.774	6.038787	-7068	7.24E-05	414	0.333333	0.005558	IFI30
1179.178	6.940573	-7068	7.20E-05	339	0.333333	0.004207	DHRS4
1683.623	8.898281	-7068	7.22E-05	358	0.333333	0.004974	COX14
3553.242	9.279977	-7068	7.31E-05	508	0.333333	0.007446	PPP1R16A
849.2936	11.61891	-7068	7.14E-05	196	0.333333	0.002244	EDDM3B
6073.508	7.372539	-7068	7.47E-05	804	0.333333	0.012776	FGD3
16.32049	10.39624	-7068	6.35E-05	26	0.25	2.54E-04	RESP18
81.67584	10.75515	-7068	6.97E-05	66	0.25	8.24E-04	HS6ST2
1309.234	13.92888	-7068	7.15E-05	206	0.333333	0.00242	ULBP1
6396.658	7.422435	-7068	7.48E-05	820	0.333333	0.013284	UACA
343.9415	7.465819	-7068	7.12E-05	183	0.25	0.002924	SYTL5
12137.91	6.441873	-7068	7.69E-05	1231	0.333333	0.019576	FLRT2
1961.522	8.564787	-7068	7.25E-05	401	0.333333	0.005765	RCC1L
574.9582	9.695206	-7068	7.13E-05	195	0.333333	0.002684	VPS18
1484.149	7.491401	-7068	7.21E-05	369	0.333333	0.004594	SLC25A37
1238.982	11.36263	-7068	7.17E-05	259	0.333333	0.003371	PRR7
16305.43	25.59612	-7068	7.31E-05	502	0.333333	0.007724	INTS6L
3947.909	7.773459	-7068	7.37E-05	624	0.333333	0.009915	POMGNT2
653.7402	7.708762	-7068	7.16E-05	251	0.333333	0.003711	TOMM7
394.33	9.282045	-7068	7.10E-05	167	0.25	0.002518	CYGB
3692.694	8.79429	-7068	7.31E-05	520	0.333333	0.007733	VIT

3749.259	9.587787	-7068	7.31E-05	503	0.333333	0.007003	SPHK1
726.2334	7.99359	-7068	7.17E-05	261	0.333333	0.004041	HMGN5
2391.041	8.068802	-7068	7.29E-05	470	0.333333	0.007064	FOXRED1
1657.298	8.811418	-7068	7.24E-05	367	0.333333	0.005538	SLC26A1
1087.474	7.548673	-7068	7.21E-05	326	0.333333	0.004794	UBA7
4821.152	6.309948	-7068	7.46E-05	799	0.333333	0.012857	NR1D1
1696.427	8.512978	-7068	7.23E-05	380	0.333333	0.005647	SRXN1
2016.096	8.791419	-7068	7.25E-05	395	0.333333	0.005653	GKN1
4726.709	6.147635	-7068	7.42E-05	770	0.333333	0.010883	NCAPG
1006.28	5.716422	-7068	7.11E-05	269	0.333333	0.002368	ZNF430
1206.165	9.960467	-7068	7.18E-05	270	0.333333	0.003337	SIRT3
8182.968	7.204784	-7068	7.55E-05	957	0.25	0.015784	CYP26B1
5741.687	6.824597	-7068	7.46E-05	807	0.333333	0.012019	SKA1
3341.989	6.752851	-7068	7.38E-05	640	0.333333	0.010329	RPRD1B
1795.162	10.04413	-7068	7.21E-05	327	0.333333	0.004141	UQCC2
11530.39	7.969859	-7068	7.61E-05	1029	0.333333	0.016515	INTU
1345.638	6.909901	-7068	7.24E-05	396	0.333333	0.006208	MAGOHB
1191.031	7.689693	-7068	7.19E-05	333	0.25	0.004634	COLEC11
8493.304	7.761608	-7068	7.54E-05	919	0.333333	0.01459	FGGY
10529.13	7.194485	-7068	7.61E-05	1063	0.333333	0.016933	GPAM
2762.832	8.296037	-7068	7.30E-05	489	0.333333	0.007885	IL17D
1491.181	7.812911	-7068	7.20E-05	341	0.333333	0.004382	NLRC5
1627.465	8.803748	-7068	7.23E-05	355	0.333333	0.004853	DHDDS
2899.463	7.745405	-7068	7.32E-05	532	0.333333	0.008087	SUFU
3611.868	8.350064	-7068	7.33E-05	550	0.333333	0.008848	KLHL7
810.0434	6.572017	-7068	7.17E-05	288	0.25	0.003887	HBA2
3462.473	8.58673	-7068	7.31E-05	520	0.333333	0.007179	HINT2
1342.939	11.10933	-7068	7.19E-05	286	0.333333	0.004224	INTS4
663.6664	8.942495	-7068	7.15E-05	217	0.333333	0.002782	ETV7
2740.465	7.043307	-7068	7.30E-05	514	0.333333	0.006756	SLC15A3
8130.826	7.628197	-7068	7.50E-05	887	0.333333	0.013414	NFKBIZ
1760.051	6.958866	-7068	7.24E-05	418	0.333333	0.005855	RPF2
2168.177	8.543501	-7068	7.25E-05	418	0.333333	0.005884	DAPK3
953.7988	7.905281	-7068	7.18E-05	291	0.333333	0.004271	MED29
541.0233	10.32064	-7068	7.12E-05	188	0.333333	0.002675	RPAIN
5681.86	14.04419	-7068	7.26E-05	409	0.333333	0.004576	NXNL1
322.2611	9.762125	-7068	7.10E-05	144	0.333333	0.002054	WDR17
934.4806	10.18651	-7068	7.16E-05	237	0.333333	0.003159	NGB
4462.832	7.16666	-7068	7.36E-05	671	0.333333	0.009303	BCL3
3667.754	6.501289	-7068	7.40E-05	685	0.333333	0.011373	XPR1
1600.153	7.349691	-7068	7.24E-05	401	0.333333	0.006053	PITHD1

1085.839	11.49182	-7068	7.15E-05	222	0.25	0.002892	MROH9
1638.531	7.808499	-7068	7.23E-05	381	0.333333	0.005523	NTMT1
747.3537	10.74713	-7068	7.14E-05	204	0.333333	0.002835	ZNHIT2
960.0116	8.317235	-7068	7.17E-05	275	0.333333	0.003746	CHPF2
1858.664	12.45935	-7068	7.18E-05	280	0.333333	0.003636	NUDT16L1
5074.691	7.29057	-7068	7.42E-05	719	0.333333	0.011207	SHPRH
10983.04	6.748789	-7068	7.65E-05	1130	0.333333	0.018118	PPIL4
735.5804	13.37491	-7068	7.10E-05	158	0.333333	0.001976	DMRT1
19121.92	6.995633	-7068	7.85E-05	1467	0.333333	0.023344	GPC6
891.824	9.512951	-7068	7.15E-05	240	0.333333	0.003232	USP36
6968.928	7.83976	-7068	7.49E-05	825	0.333333	0.013458	PNKD
1757.376	8.766425	-7068	7.23E-05	376	0.333333	0.005572	BMF
4815.111	6.822959	-7068	7.43E-05	745	0.333333	0.011665	PRRG4
3085.993	7.666299	-7068	7.31E-05	528	0.333333	0.007508	NOD2
3017.048	6.797387	-7068	7.35E-05	600	0.333333	0.009681	IFT43
1923.886	15.53934	-7068	7.18E-05	242	0.333333	0.003147	BCO1
3962.609	7.688085	-7068	7.38E-05	631	0.333333	0.010164	MTHFD2L
10348.5	8.399008	-7068	7.47E-05	875	0.333333	0.012467	ZNF700
1352.482	8.48305	-7068	7.20E-05	337	0.333333	0.004589	TTPAL
1264.437	6.547451	-7068	7.20E-05	354	0.333333	0.003936	CFHR5
634.8173	10.19824	-7068	7.13E-05	201	0.333333	0.002865	C1orf56
1248.297	9.411215	-7068	7.16E-05	253	0.333333	0.002538	RDH8
1532.941	7.176232	-7068	7.25E-05	411	0.333333	0.006426	TMEM70
8682.549	6.869554	-7068	7.59E-05	1012	0.333333	0.016772	SSH2
1776.287	8.80513	-7068	7.22E-05	360	0.25	0.005199	WHRN
1611.952	9.413228	-7068	7.22E-05	336	0.333333	0.004995	KCNE2
1335.943	8.630849	-7068	7.20E-05	323	0.333333	0.004557	PSMG3
6216.513	7.324433	-7068	7.48E-05	821	0.333333	0.013131	AGTRAP
5391.107	7.273396	-7068	7.45E-05	759	0.333333	0.012181	CNEP1R1
804.6129	8.448016	-7068	7.16E-05	252	0.25	0.003472	RBM48
1076.128	5.837414	-7068	7.19E-05	348	0.333333	0.004351	TIMM10
1507.499	8.956011	-7068	7.21E-05	327	0.333333	0.004196	EMC9
707.9477	7.802395	-7068	7.15E-05	249	0.333333	0.003415	VPS26B
793.9291	7.257401	-7068	7.18E-05	279	0.333333	0.004304	EXOC8
4732.731	6.983341	-7068	7.41E-05	705	0.333333	0.010001	C1QC
529.2212	11.43019	-7068	7.04E-05	125	0.25	0.001222	ADGB
1558.678	7.881138	-7068	7.20E-05	352	0.333333	0.004188	KRI1
1159.461	6.108496	-7068	7.23E-05	393	0.333333	0.005574	TPT1
2205.302	8.147665	-7068	7.27E-05	430	0.333333	0.006267	COA6
1453.389	9.821036	-7068	7.21E-05	310	0.333333	0.004493	SEC14L3
1776.956	7.438174	-7068	7.28E-05	433	0.333333	0.007122	SDK2

4433.868	6.874945	-7068	7.42E-05	725	0.333333	0.011657	ANAPC1
2260.14	7.241906	-7068	7.31E-05	503	0.333333	0.008106	KMT5A
1472.031	10.65269	-7068	7.16E-05	281	0.333333	0.003664	MCEE
2082.169	9.879038	-7068	7.23E-05	363	0.333333	0.00533	UBE2D4
1722.536	9.697333	-7068	7.23E-05	347	0.333333	0.004958	CHAC1
3958.382	8.577124	-7068	7.34E-05	570	0.333333	0.00833	SLCO4A1
1370.175	10.04591	-7068	7.18E-05	276	0.333333	0.003341	RHBG
1233.827	9.626582	-7068	7.17E-05	277	0.333333	0.00332	MAVS
1865.22	7.493094	-7068	7.26E-05	428	0.333333	0.006358	RASIP1
4885.722	7.781391	-7068	7.41E-05	690	0.333333	0.01113	ADAMTSL1
3084.935	10.9939	-7068	7.26E-05	404	0.333333	0.005328	OSGIN1
921.3593	8.30733	-7068	7.17E-05	277	0.333333	0.004088	ZDHHC9
4316.531	8.568362	-7068	7.36E-05	596	0.333333	0.008813	GALM
3661.273	7.132956	-7068	7.36E-05	627	0.333333	0.010029	SLF1
1033.583	5.316821	-7068	7.21E-05	369	0.333333	0.004311	ANGPTL3
1931.499	6.646225	-7068	7.25E-05	444	0.333333	0.005778	MRPL12
3004.133	7.150573	-7068	7.36E-05	586	0.333333	0.010073	GPR89B
2921.395	10.00165	-7068	7.26E-05	424	0.333333	0.005927	USF1
12177.87	7.01985	-7068	7.69E-05	1179	0.333333	0.019459	ZCCHC7
7168.143	12.02205	-7068	7.28E-05	504	0.333333	0.006608	MPC1
8279.868	6.969757	-7068	7.58E-05	986	0.333333	0.016283	SLC41A2
2497.283	10.17296	-7068	7.25E-05	397	0.333333	0.005705	SLC27A4
1793.318	8.718644	-7068	7.23E-05	372	0.333333	0.005734	RGS17
3056.055	8.071489	-7068	7.31E-05	515	0.333333	0.007801	SH3BGRL2
1743.296	9.717049	-7068	7.21E-05	349	0.333333	0.005136	SRD5A3
494.5543	8.849317	-7068	7.12E-05	191	0.25	0.002781	CPT1B
1945.12	9.979391	-7068	7.21E-05	332	0.333333	0.004164	FBXW5
2120.359	11.44743	-7068	7.21E-05	307	0.333333	0.003717	RNF39
3422.827	9.437235	-7068	7.26E-05	446	0.333333	0.00618	AK6
761.4795	7.769168	-7068	7.18E-05	271	0.333333	0.004091	BCL9L
529.3003	8.476667	-7068	7.13E-05	202	0.333333	0.003061	SLC2A14
2140.7	7.784025	-7068	7.27E-05	451	0.333333	0.006822	SEMA4C
853.2804	9.282307	-7068	7.16E-05	240	0.333333	0.0031	SLC25A23
1253.824	8.192867	-7068	7.20E-05	328	0.25	0.004772	TMSB15A
3564.42	6.821792	-7068	7.39E-05	659	0.333333	0.01108	HM13
2945.87	7.257273	-7068	7.32E-05	552	0.333333	0.008245	RPTOR
1145.053	12.55145	-7068	7.13E-05	217	0.25	0.002603	PAOX
2243.362	7.366313	-7068	7.28E-05	468	0.333333	0.007139	TAX1BP3
3822.385	7.17144	-7068	7.38E-05	652	0.333333	0.010319	LEPR
1049.23	8.223852	-7068	7.19E-05	308	0.333333	0.004809	TRPM7
11525.88	7.379055	-7068	7.63E-05	1090	0.333333	0.01712	KMT2E

1767.609	7.167074	-7068	7.27E-05	439	0.333333	0.006831	VPS29
3027.84	8.378183	-7068	7.32E-05	513	0.333333	0.007665	HS1BP3
2820.914	6.644488	-7068	7.32E-05	567	0.333333	0.008228	CASC5
3053.332	7.970141	-7068	7.31E-05	527	0.333333	0.007955	CD209
3875.364	7.893902	-7068	7.36E-05	602	0.333333	0.00931	RELT
760.0174	8.109306	-7068	7.17E-05	259	0.333333	0.003979	ANAPC16
6533.084	6.608322	-7068	7.51E-05	894	0.333333	0.014151	SBF2
2860.435	7.30859	-7068	7.32E-05	548	0.333333	0.00874	SERTAD4
3804.993	7.446303	-7068	7.35E-05	617	0.333333	0.008822	CDCA3
1330.526	5.478916	-7068	7.24E-05	434	0.333333	0.00618	CRISPLD2
2218.082	13.4327	-7068	7.19E-05	282	0.333333	0.003437	ZNF496
1275.752	12.93358	-7068	7.15E-05	220	0.333333	0.002768	ARMC5
3473.138	9.312793	-7068	7.30E-05	497	0.333333	0.006874	ZGPAT
765.4014	14.23063	-7068	7.11E-05	161	0.25	0.001958	NUDT7
15480.13	7.075455	-7068	7.76E-05	1317	0.333333	0.021222	RDH14
3501.398	7.338978	-7068	7.37E-05	613	0.333333	0.009763	NCOA7
1384.194	9.626456	-7068	7.19E-05	292	0.333333	0.003837	HRH3
1388.988	7.380533	-7068	7.23E-05	377	0.333333	0.00584	EXOC6
4927.128	7.114908	-7068	7.45E-05	749	0.333333	0.012619	SLC15A4
1192.913	6.864904	-7068	7.12E-05	271	0.333333	0.002422	ZNF791
1163.946	7.256048	-7068	7.22E-05	357	0.333333	0.006042	CNST
824.2198	7.120474	-7068	7.20E-05	308	0.333333	0.005288	NEIL2
1070.704	7.291124	-7068	7.21E-05	334	0.333333	0.005494	NSRP1
491.1406	8.574868	-7068	7.13E-05	195	0.333333	0.003	SGK3
2177.532	7.921257	-7068	7.28E-05	456	0.333333	0.007241	C18orf8
1339.769	7.618067	-7068	7.24E-05	366	0.333333	0.005889	TSEN15
2033.042	8.486738	-7068	7.25E-05	407	0.333333	0.005813	PLXNA1
1485.932	11.74657	-7068	7.17E-05	268	0.333333	0.003897	C7orf31
834.2886	9.362535	-7068	7.16E-05	243	0.333333	0.003354	AFTPH
7288.588	8.40181	-7068	7.46E-05	776	0.333333	0.012329	PGAM5
1669.685	9.681628	-7068	7.21E-05	324	0.333333	0.004298	TACO1
1529.329	8.661301	-7068	7.23E-05	354	0.25	0.005533	NXPE3
13671.79	7.829748	-7068	7.67E-05	1144	0.25	0.018229	FSTL5
4615.01	7.785759	-7068	7.37E-05	624	0.333333	0.008627	S100A14
11391.82	6.854003	-7068	7.66E-05	1152	0.333333	0.018206	MSI2
991.3938	7.225505	-7068	7.20E-05	323	0.333333	0.004968	ATL3
3635.635	7.915408	-7068	7.35E-05	581	0.333333	0.009618	MAMDC2
13761.65	7.440898	-7068	7.67E-05	1178	0.333333	0.018474	JAZF1
3550.95	7.94879	-7068	7.33E-05	562	0.333333	0.008005	S100A6
2718.329	7.570459	-7068	7.30E-05	525	0.333333	0.007527	SLC25A15
1681.811	8.42123	-7068	7.23E-05	361	0.333333	0.00531	NIPSNAP3A

4862.302	7.245916	-7068	7.45E-05	739	0.333333	0.012444	RHBDD1
1602.092	9.81129	-7068	7.22E-05	322	0.333333	0.004225	ADAMTSL4
543.0526	9.420046	-7068	7.13E-05	191	0.333333	0.002557	TMED4
4096.678	7.307955	-7068	7.39E-05	665	0.333333	0.010247	ATP6V1F
3256.853	9.535799	-7068	7.28E-05	458	0.333333	0.006153	DUOX2
2118.387	8.871933	-7068	7.23E-05	402	0.25	0.005298	C15orf48
4016.288	7.759008	-7068	7.36E-05	618	0.333333	0.009416	OSBPL5
1853.841	6.63928	-7068	7.25E-05	463	0.25	0.006007	VTN
1310.736	6.718705	-7068	7.21E-05	368	0.333333	0.004627	APOC4
1386.214	6.875955	-7068	7.23E-05	382	0.333333	0.005399	RBM4
1991.767	7.616786	-7068	7.26E-05	421	0.333333	0.006032	TLR8
1178.071	8.026473	-7068	7.19E-05	305	0.333333	0.003551	TTC38
3034.381	7.894279	-7068	7.32E-05	536	0.333333	0.008186	IFT80
1337.035	11.06674	-7068	7.18E-05	259	0.333333	0.003028	RNF208
1186.327	5.790728	-7068	7.23E-05	392	0.333333	0.005264	HLA-DRB5
8459.288	7.234774	-7068	7.55E-05	951	0.333333	0.015288	JAML
1649.094	9.952749	-7068	7.19E-05	315	0.333333	0.004132	MIF4GD
2475.684	12.56438	-7068	7.21E-05	325	0.333333	0.004456	TGM7
679.9135	7.168486	-7068	7.16E-05	267	0.333333	0.004124	NUCKS1
3609.374	10.205	-7068	7.22E-05	419	0.333333	0.004773	ZNF552
2101.685	9.813285	-7068	7.23E-05	363	0.25	0.00491	KLF15
1865.113	7.912635	-7068	7.27E-05	427	0.333333	0.006988	IRF2BP2
4802.729	8.440097	-7068	7.38E-05	637	0.333333	0.010034	GNPDA2
948.0629	7.41484	-7068	7.19E-05	301	0.333333	0.004085	NABP2
1418.316	3.893886	-7068	7.26E-05	540	0.333333	0.006418	RPL4
720.3765	9.894938	-7068	7.15E-05	218	0.333333	0.00293	CD200R1
13191.96	7.323995	-7068	7.69E-05	1182	0.333333	0.019138	ARHGAP20
2733.191	14.05025	-7068	7.19E-05	300	0.333333	0.003326	TMEM79
429.0966	11.46959	-7068	7.08E-05	131	0.333333	0.001803	KLHL29
2214.168	9.591142	-7068	7.24E-05	387	0.333333	0.005707	TMEM263
1284.393	8.583906	-7068	7.22E-05	339	0.333333	0.005315	EDARADD
3264.585	8.107291	-7068	7.32E-05	541	0.333333	0.007979	NRM
3283.318	6.773673	-7068	7.34E-05	611	0.333333	0.008946	ECHDC3
1484.568	9.907009	-7068	7.20E-05	309	0.333333	0.004214	TRIM41
3016.704	8.506211	-7068	7.31E-05	516	0.333333	0.00835	H3F3B
3385.623	7.956869	-7068	7.35E-05	566	0.333333	0.009062	PPP1R14C
2705.732	8.853176	-7068	7.28E-05	457	0.333333	0.007014	AJUBA
1938.61	12.16767	-7068	7.19E-05	295	0.25	0.00438	SPZ1
4963.839	10.94338	-7068	7.33E-05	533	0.333333	0.008378	UBE2U
3854.54	7.507745	-7068	7.38E-05	626	0.333333	0.010039	TMEM167A
1633.639	9.883607	-7068	7.21E-05	315	0.333333	0.004369	CASP14

3340.601	7.700329	-7068	7.33E-05	562	0.333333	0.0085	CD274
1568.97	8.132833	-7068	7.23E-05	370	0.333333	0.00531	CERS2
547.5305	9.225589	-7068	7.11E-05	189	0.333333	0.002282	UCK1
2827.672	7.902804	-7068	7.31E-05	511	0.333333	0.007918	GPAT4
698.6109	12.94817	-7068	7.11E-05	169	0.333333	0.002565	BBS5
1862.231	13.29333	-7068	7.17E-05	238	0.333333	0.003245	IFT172
975.7682	8.374044	-7068	7.19E-05	292	0.333333	0.004472	ZCCHC17
1241.599	7.542715	-7068	7.19E-05	324	0.333333	0.004325	PDCD2L
4525.598	7.63118	-7068	7.41E-05	679	0.333333	0.011138	RNF185
1555.174	7.869431	-7068	7.23E-05	370	0.333333	0.005411	WNT3A
3217.858	7.546983	-7068	7.33E-05	552	0.333333	0.008206	FZD10
1694.398	6.635532	-7068	7.19E-05	351	0.333333	0.003965	ZNF493
2098.283	7.456429	-7068	7.29E-05	463	0.333333	0.007358	FAM76B
1335.245	8.234683	-7068	7.20E-05	320	0.25	0.00436	SCIN
2942.99	8.517064	-7068	7.30E-05	497	0.333333	0.007826	OMA1
2168.067	4.520761	-7068	7.29E-05	600	0.333333	0.007186	SF3B6
4000.189	7.183575	-7068	7.39E-05	664	0.333333	0.010953	FRMD6
365.9516	9.00189	-7068	7.12E-05	166	0.333333	0.002486	LOXL4
1624.451	10.13465	-7068	7.19E-05	319	0.333333	0.004646	BTBD6
3320.049	10.50143	-7068	7.27E-05	443	0.333333	0.006295	EGLN2
1399.768	9.815349	-7068	7.19E-05	294	0.333333	0.003593	THEM6
283.9848	3.62145	-7068	7.10E-05	249	0.333333	0.002809	RPS26
1162.821	4.985795	-7068	7.21E-05	396	0.333333	0.00491	TIMM8B
2596.408	7.910847	-7068	7.30E-05	494	0.333333	0.007393	PHF6
211.1904	6.805157	-7068	7.05E-05	141	0.333333	0.001882	COL6A6
7765.569	8.124347	-7068	7.48E-05	828	0.333333	0.012802	LRRK2
5955.688	7.206409	-7068	7.46E-05	800	0.333333	0.012889	ZNRF2
3139.707	7.749505	-7068	7.33E-05	548	0.333333	0.008657	DZANK1
1539.702	6.102255	-7068	7.26E-05	444	0.25	0.00673	COL8A2
1198.574	13.55119	-7068	7.15E-05	212	0.333333	0.002792	SIRT4
1713.795	7.971426	-7068	7.22E-05	369	0.333333	0.004774	ARHGAP9
2863.03	7.172606	-7068	7.31E-05	539	0.333333	0.007842	C1orf112
1778.605	9.220665	-7068	7.21E-05	347	0.25	0.004894	SLC19A3
3522.801	6.849469	-7068	7.37E-05	636	0.333333	0.010068	MATN2
1089.372	7.279064	-7068	7.21E-05	342	0.333333	0.005396	MMGT1
1950.492	8.872483	-7068	7.24E-05	385	0.333333	0.005823	IMMP1L
3139.081	8.428038	-7068	7.32E-05	514	0.333333	0.007786	TNFSF15
4756.473	14.89425	-7068	7.23E-05	365	0.333333	0.004561	ZSCAN1
891.0182	8.268941	-7068	7.18E-05	278	0.333333	0.004404	KCTD14
3569.552	7.922274	-7068	7.34E-05	575	0.333333	0.008673	CSNK1A1L
797.3052	4.29663	-7068	7.20E-05	386	0.333333	0.005454	PHF5A

1521.31	9.437317	-7068	7.18E-05	314	0.333333	0.004332	GMPPB
2470.489	10.31872	-7068	7.24E-05	389	0.333333	0.005229	CRELD2
978.5857	8.077171	-7068	7.17E-05	282	0.333333	0.003785	DUS2
2436.408	7.983418	-7068	7.28E-05	461	0.333333	0.006561	SLC6A14
2741.079	6.831082	-7068	7.30E-05	540	0.333333	0.007384	RAD18
671.4556	12.65757	-7068	7.11E-05	154	0.333333	0.001893	LDHAL6B
2445.066	7.323527	-7068	7.31E-05	515	0.333333	0.008535	RAPH1
2315.985	9.215453	-7068	7.25E-05	406	0.333333	0.005756	SEMA4B
2311.42	8.262375	-7068	7.28E-05	446	0.333333	0.006981	PHLDB2
12207.03	7.503892	-7068	7.62E-05	1093	0.333333	0.016896	TSHZ3
1225.351	8.783173	-7068	7.19E-05	302	0.333333	0.004115	NMRAL1
964.41	8.735508	-7068	7.18E-05	275	0.333333	0.0039	TMEM209
1693.66	8.666697	-7068	7.23E-05	367	0.333333	0.005172	NT5DC2
4153.25	6.13377	-7068	7.39E-05	717	0.333333	0.010242	KIF15
3223.675	7.630112	-7068	7.34E-05	566	0.333333	0.008704	SUSD1
766.5951	8.490789	-7068	7.16E-05	252	0.333333	0.00397	MACC1
2454.172	8.25	-7068	7.27E-05	447	0.333333	0.006482	GRHL3
1461.772	7.141568	-7068	7.25E-05	409	0.25	0.006738	C15orf57
1785.927	8.643307	-7068	7.23E-05	372	0.25	0.005166	PHF11
1142.213	9.657084	-7068	7.18E-05	279	0.333333	0.00396	APLN
2113.975	9.91646	-7068	7.24E-05	362	0.333333	0.004786	REEP6
1470.249	6.225187	-7068	7.24E-05	418	0.333333	0.005844	DSCC1
4871.057	6.837999	-7068	7.45E-05	760	0.333333	0.012579	DDIT4L
3076.921	8.196471	-7068	7.32E-05	523	0.333333	0.008098	AIF1L
1090.677	6.422463	-7068	7.22E-05	370	0.333333	0.00583	GINS3
1357.848	9.968924	-7068	7.17E-05	279	0.25	0.003723	NEIL1
11837.69	10.33129	-7068	7.50E-05	835	0.333333	0.011973	FBLN7
4188.516	7.281978	-7068	7.40E-05	673	0.333333	0.010919	CREB3L4
761.1859	7.633251	-7068	7.18E-05	278	0.333333	0.0044	ITLN1
4663.628	8.966574	-7068	7.35E-05	593	0.333333	0.008851	AGR3
1501.062	8.532765	-7068	7.21E-05	352	0.333333	0.005146	FOXA3
2073.266	9.039981	-7068	7.24E-05	387	0.333333	0.005614	CIB3
1486.241	7.45487	-7068	7.23E-05	378	0.333333	0.005629	MITD1
1093.766	10.16351	-7068	7.17E-05	250	0.333333	0.003128	IL17RC
696.8247	11.21105	-7068	7.14E-05	190	0.333333	0.002634	PLD4
287.9806	8.901862	-7068	7.09E-05	153	0.25	0.002261	ZFAND2A
7176.053	7.432183	-7068	7.51E-05	870	0.333333	0.014191	ALG14
9016.181	7.067185	-7068	7.59E-05	1006	0.333333	0.016309	RAB3C
5118.26	11.09981	-7068	7.34E-05	550	0.333333	0.009173	TMCO5A
2752.347	10.87665	-7068	7.24E-05	384	0.333333	0.00591	ACTRT2
2325.591	7.220059	-7068	7.32E-05	508	0.333333	0.008266	FCHO2

3093.244	11.36736	-7068	7.26E-05	401	0.25	0.005985	OPN4
9322.137	7.056855	-7068	7.56E-05	1008	0.333333	0.015183	ANLN
5493.542	8.71176	-7068	7.39E-05	659	0.333333	0.010093	MTPN
2443.885	8.021509	-7068	7.29E-05	471	0.333333	0.007362	KIAA1524
11591.79	7.994739	-7068	7.56E-05	1014	0.333333	0.014832	PARPBP
880.8094	11.44777	-7068	7.12E-05	190	0.333333	0.002022	KIR2DS4
4019.271	7.070348	-7068	7.39E-05	671	0.333333	0.010646	HELLS
3268.439	7.441025	-7068	7.33E-05	572	0.333333	0.008151	CES1
3044.426	8.329294	-7068	7.31E-05	517	0.333333	0.007848	RNF135
1112.086	7.662791	-7068	7.20E-05	319	0.333333	0.004781	PIM3
2541.5	7.858907	-7068	7.29E-05	487	0.25	0.007681	SMIM3
2392.007	6.275877	-7068	7.31E-05	540	0.333333	0.007856	CLEC7A
424.908	10.90771	-7068	7.07E-05	140	0.333333	0.001549	CRYGB
162.1513	8.473014	-7068	7.06E-05	114	0.333333	0.001552	BBC3
1596.128	10.35137	-7068	7.19E-05	299	0.333333	0.003733	RPUSD1
4741.455	7.152146	-7068	7.44E-05	731	0.333333	0.012141	NUDCD2
477.4222	10.24871	-7068	7.11E-05	166	0.333333	0.001983	WNT7B
1078.613	8.685363	-7068	7.20E-05	300	0.333333	0.004388	PITPNC1
1803.02	10.60183	-7068	7.20E-05	309	0.333333	0.003976	CLEC3A
3538.24	7.220946	-7068	7.35E-05	609	0.333333	0.009156	CDH23
3687.912	6.941915	-7068	7.38E-05	653	0.333333	0.010659	ARFGEF3
2430.922	10.43849	-7068	7.22E-05	360	0.25	0.005142	CHST6
5840.804	6.545613	-7068	7.50E-05	866	0.25	0.014568	GAS2L3
612.5814	8.269201	-7068	7.16E-05	234	0.333333	0.003717	ALKBH2
414.6743	5.068247	-7068	7.13E-05	249	0.333333	0.003725	UHRF1
8211.113	6.929306	-7068	7.56E-05	978	0.333333	0.015866	E2F7
434.6273	11.82257	-7068	7.05E-05	123	0.25	0.001311	CFAP45
1722.885	10.90865	-7068	7.19E-05	295	0.333333	0.004369	LRRTM4
332.3522	7.600661	-7068	7.12E-05	183	0.333333	0.00288	RP9
7052.029	8.218145	-7068	7.48E-05	801	0.333333	0.01276	PDCD7
9651.915	7.24719	-7068	7.61E-05	1032	0.333333	0.017238	PPM1L
5026.901	7.44147	-7068	7.42E-05	724	0.333333	0.011477	RBM33
907.1563	9.389915	-7068	7.16E-05	248	0.333333	0.003125	UNC93B1
4284.128	7.113015	-7068	7.40E-05	686	0.25	0.011363	FMN2
482.993	10.59968	-7068	7.09E-05	167	0.333333	0.002269	FAM111B
5102.634	6.336822	-7068	7.46E-05	811	0.333333	0.01246	MCM10
4626.196	13.76866	-7068	7.24E-05	374	0.333333	0.004259	TBATA
1632.518	7.55095	-7068	7.24E-05	394	0.333333	0.005655	DLL4
1552.887	8.991082	-7068	7.21E-05	346	0.333333	0.005397	TTLL3
966.4453	10.58026	-7068	7.16E-05	238	0.333333	0.003351	TIGD7
1377.31	7.661385	-7068	7.12E-05	269	0.333333	0.002123	ZNF234

9371.354	7.181993	-7068	7.60E-05	1016	0.333333	0.016779	C9orf72
750.3719	7.61026	-7068	7.16E-05	267	0.333333	0.003886	DBNL
1241.338	4.124975	-7068	7.24E-05	483	0.333333	0.005928	NOP56
1997.091	6.032718	-7068	7.19E-05	429	0.333333	0.004342	NDUFB9
1508.86	8.849301	-7068	7.21E-05	337	0.333333	0.004937	NKD2
2184.816	7.287024	-7068	7.29E-05	483	0.333333	0.007204	ATP5SL
663.3907	10.79586	-7068	7.15E-05	203	0.333333	0.002923	MRI1
1183.8	11.07864	-7068	7.15E-05	227	0.333333	0.00262	CELA1
1147.705	12.29272	-7068	7.15E-05	223	0.25	0.003447	LRRC34
1717.343	11.29997	-7068	7.19E-05	295	0.333333	0.004325	ACTRT3
4879.697	10.24249	-7068	7.33E-05	536	0.333333	0.008453	FBXO15
10714.35	7.089194	-7068	7.65E-05	1093	0.333333	0.01809	CPNE8
1276.908	8.898255	-7068	7.20E-05	313	0.25	0.004673	B3GNT5
861.9379	9.192859	-7068	7.16E-05	251	0.333333	0.003641	SNX22
442.6566	11.89651	-7068	7.09E-05	138	0.333333	0.001694	TCP10L
1354.283	8.678433	-7068	7.20E-05	322	0.333333	0.004533	STBD1
3970.294	7.664405	-7068	7.39E-05	638	0.333333	0.010518	ALKBH3
4637.284	7.768423	-7068	7.41E-05	674	0.333333	0.011019	CLRN1
3630.384	7.05479	-7068	7.38E-05	644	0.333333	0.010658	FBXO32
4639.121	7.71454	-7068	7.40E-05	670	0.333333	0.01021	ITGA11
7274.969	7.220418	-7068	7.51E-05	877	0.333333	0.013996	TMCC3
10886.74	7.73897	-7068	7.61E-05	1027	0.333333	0.016368	AGBL4
6436.732	8.517977	-7068	7.44E-05	741	0.333333	0.011672	RBM24
1875.043	10.14291	-7068	7.21E-05	337	0.25	0.004761	ZBTB4
405.7011	7.875817	-7068	7.11E-05	186	0.333333	0.0025	CCDC88B
935.5122	10.82098	-7068	7.15E-05	218	0.25	0.003026	KLHDC7B
1323.096	7.828477	-7068	7.18E-05	307	0.333333	0.004066	RASAL3
786.3036	11.15638	-7068	7.09E-05	173	0.333333	0.001703	KIR2DL2
615.9573	9.183586	-7068	7.12E-05	195	0.333333	0.002704	GOPC
2532.877	8.813742	-7068	7.28E-05	445	0.333333	0.006965	LCA5
1329.271	8.965297	-7068	7.20E-05	318	0.333333	0.004622	DGCR6
1147.749	9.005482	-7068	7.17E-05	276	0.25	0.003578	SEMA3G
1571.839	11.12033	-7068	7.17E-05	273	0.333333	0.003616	RBP7
6294.767	7.39007	-7068	7.49E-05	818	0.333333	0.013323	ST3GAL3
2839.526	8.041857	-7068	7.29E-05	485	0.333333	0.007551	TC2N
705.389	6.949059	-7068	7.15E-05	262	0.333333	0.00364	HAVCR2
4756.478	8.1521	-7068	7.38E-05	648	0.333333	0.010324	MFSD4A
1706.352	6.521802	-7068	7.25E-05	434	0.333333	0.00576	ALYREF
4618.375	12.58943	-7068	7.24E-05	391	0.333333	0.004517	OPTC
893.858	7.73705	-7068	7.19E-05	297	0.333333	0.004721	DENND2C
3301.523	7.282363	-7068	7.35E-05	586	0.333333	0.009573	FGF10

32.00165	4.690494	-7068	6.86E-05	75	0.25	0.001249	SMN2
1401.497	4.944796	-7068	7.27E-05	492	0.333333	0.007102	TCP1
4380.707	7.689733	-7068	7.40E-05	656	0.333333	0.010918	RNF144B
1843.013	7.732566	-7068	7.24E-05	404	0.333333	0.005413	GNGT2
7464.297	6.937326	-7068	7.55E-05	937	0.333333	0.015464	PDCL3
7516.687	7.066675	-7068	7.53E-05	911	0.333333	0.014675	NT5DC1
1586.659	8.067482	-7068	7.24E-05	390	0.333333	0.00623	PELO
1386.974	9.278951	-7068	7.18E-05	299	0.333333	0.003724	FGF21
8090.23	7.91651	-7068	7.53E-05	885	0.333333	0.014836	AGBL1
1297.743	8.143368	-7068	7.22E-05	340	0.333333	0.004897	APOL2
1990.285	10.47584	-7068	7.22E-05	337	0.333333	0.004933	PELI3
3120.648	8.044723	-7068	7.33E-05	539	0.333333	0.008484	SESN2
2673.121	6.984829	-7068	7.30E-05	535	0.333333	0.008145	ACAD11
1514.332	7.109592	-7068	7.24E-05	406	0.333333	0.00592	SGO1
1162.959	7.986205	-7068	7.20E-05	318	0.333333	0.004794	MYO1G
1049.799	11.30382	-7068	7.14E-05	216	0.333333	0.002451	SPSB3
1021.265	7.207831	-7068	7.22E-05	336	0.333333	0.005466	LRP12
2304.112	8.363945	-7068	7.27E-05	439	0.333333	0.006561	CIDEA
1275.09	9.182431	-7068	7.20E-05	302	0.333333	0.004248	LZTS2
2875.17	7.320822	-7068	7.33E-05	554	0.333333	0.00895	NOM1
800.3195	8.36366	-7068	7.17E-05	261	0.333333	0.003917	RNF170
5099.577	7.001052	-7068	7.47E-05	772	0.333333	0.013083	RFFL
1552.548	9.373408	-7068	7.19E-05	310	0.333333	0.003934	COA3
5595.306	8.945265	-7068	7.33E-05	596	0.333333	0.008286	ZNF709
1912.711	8.186191	-7068	7.16E-05	308	0.333333	0.002781	ZNF443
1113.775	9.176783	-7068	7.17E-05	274	0.333333	0.00369	YPEL3
6046.012	6.996344	-7068	7.47E-05	825	0.333333	0.012633	CDCA7
6836.073	9.763166	-7068	7.40E-05	672	0.333333	0.009844	RXFP2
400.0284	8.920428	-7068	7.12E-05	180	0.333333	0.002633	NELFA
1238.276	11.58576	-7068	7.14E-05	234	0.333333	0.003058	ZMYND19
147.5307	5.581085	-7068	7.05E-05	139	0.333333	0.002111	TMED7
3030.961	7.843619	-7068	7.32E-05	535	0.333333	0.008684	NADK2
10385.02	7.205889	-7068	7.62E-05	1065	0.333333	0.017624	PLEKHA7
420.8985	8.143884	-7068	7.14E-05	194	0.25	0.003115	CIART
1934.288	9.563867	-7068	7.22E-05	354	0.333333	0.004903	SEMA5B
1186.498	9.18518	-7068	7.17E-05	286	0.333333	0.003571	PIDD1
1724.435	12.67576	-7068	7.17E-05	284	0.333333	0.003998	OPN3
2591.499	9.203221	-7068	7.28E-05	437	0.333333	0.006874	CRYGN
597.5994	6.671151	-7068	7.14E-05	246	0.333333	0.00362	CTHRC1
5977.799	7.197794	-7068	7.47E-05	804	0.333333	0.012916	TMEM154
345.7515	9.156812	-7068	7.11E-05	161	0.333333	0.002407	C7orf25

458.6843	6.943935	-7068	7.14E-05	226	0.25	0.003802	THAP6
5898.128	7.96662	-7068	7.44E-05	724	0.333333	0.011703	TTC8
822.7993	8.203257	-7068	7.16E-05	253	0.333333	0.003499	ADCY4
410.2877	8.186504	-7068	7.09E-05	174	0.333333	0.002666	ANKIB1
7733.128	8.45368	-7068	7.45E-05	797	0.333333	0.012033	SGO2
1048.452	10.58817	-7068	7.16E-05	239	0.333333	0.003495	ZFHX4
237.7396	8.31417	-7068	7.10E-05	143	0.333333	0.002287	MGAT5B
3963.903	8.845185	-7068	7.33E-05	556	0.333333	0.008729	HES6
4171.047	7.653772	-7068	7.38E-05	640	0.333333	0.010112	SLC4A11
10589.35	6.792858	-7068	7.62E-05	1117	0.333333	0.017808	MYO18A
584.0195	8.300417	-7068	7.11E-05	202	0.333333	0.002393	TNXB
1261.055	7.964389	-7068	7.19E-05	316	0.333333	0.004147	ABCC6
1726.502	8.601083	-7068	7.23E-05	376	0.333333	0.005682	MTFP1
2339.575	9.122135	-7068	7.26E-05	418	0.25	0.006212	SUSD3
1801.9	6.402866	-7068	7.26E-05	464	0.333333	0.006651	SERTAD1
11.9084	2.791106	-7068	6.73E-05	60	0.333333	8.97E-04	PPIAL4A
17485.48	17.90282	-7068	7.34E-05	561	0.333333	0.009093	UQCRH
2817.156	6.140311	-7068	7.34E-05	607	0.333333	0.008911	FANCD2
1872.291	8.096243	-7068	7.26E-05	416	0.333333	0.00644	DGCR8
3503.791	6.934848	-7068	7.38E-05	636	0.333333	0.010336	GLB1
8969.335	6.874674	-7068	7.60E-05	1023	0.333333	0.017156	TMTC2
5455.082	7.904082	-7068	7.43E-05	722	0.333333	0.01119	DEPDC1B
3133.708	10.99026	-7068	7.24E-05	391	0.333333	0.004698	IRAK2
662.1632	5.468468	-7068	7.08E-05	216	0.333333	0.001631	ZNF138
2294.38	8.776935	-7068	7.18E-05	348	0.333333	0.003573	ZNF649
2095.314	8.16678	-7068	7.27E-05	429	0.333333	0.006611	CKAP2L
862.4829	5.213437	-7068	7.20E-05	361	0.333333	0.0049	DDX39B
2807.708	6.282867	-7068	7.33E-05	581	0.333333	0.008388	KIF20A
3340.461	8.217968	-7068	7.33E-05	544	0.333333	0.00833	HFE2
1644.219	8.648608	-7068	7.23E-05	359	0.333333	0.005299	PCDH15
745.0331	8.023467	-7068	7.16E-05	260	0.333333	0.003941	NMNAT1
2120.778	7.764103	-7068	7.26E-05	442	0.333333	0.006667	CENPL
588.2952	7.157921	-7068	7.14E-05	232	0.25	0.003437	HBA1
2559.026	7.760623	-7068	7.32E-05	504	0.333333	0.008498	GRID1
9712.026	7.29402	-7068	7.60E-05	1018	0.333333	0.016448	DSCAML1
1968.687	7.297772	-7068	7.28E-05	448	0.333333	0.007081	EID2
199.7318	7.697072	-7068	7.08E-05	132	0.333333	0.002024	UBE2QL1
75.19834	12.34973	-7068	6.82E-05	49	0.25	5.12E-04	RGS9BP
1124.294	8.629552	-7068	7.17E-05	276	0.333333	0.003447	TRIM63
447.8529	8.377149	-7068	7.07E-05	168	0.333333	0.001831	UQCC3
80.54984	6.770958	-7068	6.90E-05	85	0.25	0.001013	C22orf39

2198.852	7.83313	-7068	7.28E-05	456	0.333333	0.007283	NDUFAF2
1032.239	8.781267	-7068	7.17E-05	263	0.333333	0.003405	GUCA1C
443.8398	12.22908	-7068	7.09E-05	141	0.333333	0.002033	DYNC2H1
100.6008	4.952058	-7068	7.07E-05	103	0.333333	0.001339	ZNF740
1127.864	11.64478	-7068	7.14E-05	205	0.333333	0.002645	HES7
4469.352	8.328716	-7068	7.38E-05	631	0.333333	0.009843	TMEM60
403.9176	11.36254	-7068	7.08E-05	131	0.333333	0.001489	RGSL1
1502.815	8.062857	-7068	7.22E-05	360	0.333333	0.005482	UBE2E2
102.3549	8.896455	-7068	7.00E-05	84	0.25	0.001262	TMEM237
68.36997	10.4161	-7068	6.98E-05	65	0.25	9.44E-04	FAM117B
1818.612	11.02412	-7068	7.20E-05	310	0.25	0.004174	UNC119B
1499.033	10.38015	-7068	7.18E-05	288	0.333333	0.003447	DLK2
15371.48	69.98879	-7068	7.16E-05	245	0.333333	0.002956	SPDYE2
1258.627	9.177349	-7068	7.19E-05	306	0.333333	0.004497	ADHFE1
4053.455	6.590276	-7068	7.42E-05	713	0.333333	0.012023	YPEL2
601.3609	7.621786	-7068	7.16E-05	242	0.333333	0.003874	TMTC3
1066.144	7.99764	-7068	7.20E-05	317	0.333333	0.005105	SARNP
1634.245	7.284085	-7068	7.25E-05	418	0.333333	0.006712	PYROXD2
811.8412	12.09765	-7068	7.13E-05	179	0.333333	0.002146	RAX2
5757.866	7.783506	-7068	7.43E-05	742	0.333333	0.011732	ZNF831
728.2296	8.890427	-7068	7.14E-05	221	0.333333	0.002795	RHBDD2
657.7617	7.482323	-7068	7.15E-05	243	0.333333	0.003234	APOL6
595.5097	11.08883	-7068	7.11E-05	169	0.333333	0.002098	COX4I2
2764.81	11.07114	-7068	7.24E-05	382	0.333333	0.005042	USHBP1
9133.281	7.949985	-7068	7.54E-05	923	0.333333	0.014602	KCNH8
946.5322	7.877571	-7068	7.19E-05	293	0.25	0.004534	MSANTD4
824.8753	8.617514	-7068	7.17E-05	259	0.333333	0.003834	SIRT5
2778.435	7.037666	-7068	7.33E-05	556	0.333333	0.008756	KNSTRN
754.9754	7.7049	-7068	7.15E-05	262	0.333333	0.003815	MASTL
1439.432	4.880818	-7068	7.26E-05	471	0.333333	0.00649	ERCC6L
2128.654	7.536666	-7068	7.29E-05	472	0.25	0.007526	PCBD2
1365.084	8.720885	-7068	7.22E-05	335	0.333333	0.00528	TTC9C
2682.412	8.589942	-7068	7.21E-05	383	0.333333	0.004222	ZNF558
456.3921	8.813654	-7068	7.10E-05	183	0.25	0.002783	GLCCI1
981.2244	7.557911	-7068	7.15E-05	275	0.333333	0.003116	MIOX
2816.843	13.62998	-7068	7.22E-05	339	0.333333	0.005132	ULBP3
2663.603	7.060364	-7068	7.32E-05	546	0.333333	0.008459	RHNO1
1843.833	8.875394	-7068	7.23E-05	373	0.333333	0.00578	ZSWIM6
2407.045	8.681107	-7068	7.24E-05	398	0.25	0.005184	PDZK1
448.9266	11.53587	-7068	7.03E-05	123	0.25	0.001298	C9orf24
507.5262	11.26964	-7068	7.09E-05	156	0.333333	0.002205	C5orf51

941.5217	7.466839	-7068	7.18E-05	310	0.333333	0.004474	DCLRE1B
771.1207	9.582512	-7068	7.16E-05	234	0.333333	0.003593	EMB
2073.125	8.170499	-7068	7.23E-05	396	0.25	0.005086	MAL2
1348.145	7.623828	-7068	7.20E-05	340	0.333333	0.004129	SPC24
367.686	8.420171	-7068	7.10E-05	167	0.333333	0.002213	GHRL
1788.499	7.370207	-7068	7.25E-05	419	0.333333	0.006012	BOP1
437.332	7.348474	-7068	7.12E-05	196	0.333333	0.002656	CRTC2
1125.082	8.745495	-7068	7.19E-05	299	0.333333	0.00453	SSBP4
1168.256	11.31937	-7068	7.16E-05	247	0.333333	0.003293	USP18
2589.726	7.024625	-7068	7.32E-05	537	0.333333	0.008612	LNX2
13745.08	6.896313	-7068	7.75E-05	1273	0.333333	0.021417	KCTD1
10536.9	6.881318	-7068	7.66E-05	1118	0.333333	0.018702	MAML2
1450.294	6.773518	-7068	7.23E-05	390	0.333333	0.005487	RAB34
1826.836	8.725648	-7068	7.24E-05	368	0.333333	0.005304	SDR16C5
1608.498	11.61736	-7068	7.16E-05	253	0.333333	0.002766	P2RX2
882.4322	8.837153	-7068	7.18E-05	267	0.333333	0.004071	ACSS1
154.8521	19.88988	-7068	6.67E-05	43	0.25	3.54E-04	SYCE3
1809.675	10.67203	-7068	7.20E-05	317	0.333333	0.004643	TSSK6
2228.755	8.40582	-7068	7.26E-05	433	0.333333	0.006324	NES
1701.726	7.477509	-7068	7.25E-05	409	0.333333	0.00643	C1orf131
1944.059	8.591886	-7068	7.25E-05	399	0.333333	0.006217	MRPS6
650.4419	7.991742	-7068	7.16E-05	245	0.333333	0.003875	SFR1
3245.766	9.140224	-7068	7.31E-05	500	0.333333	0.008155	MIPOL1
175.6805	9.08263	-7068	7.01E-05	104	0.25	0.001338	HSBP1L1
1183.095	8.544374	-7068	7.20E-05	313	0.333333	0.004408	MAP3K6
892.6693	9.486212	-7068	7.16E-05	250	0.333333	0.003513	PIGU
2223.826	8.213745	-7068	7.27E-05	441	0.333333	0.00704	SAMD11
4627.392	7.982471	-7068	7.39E-05	651	0.333333	0.010587	USP31
2035.996	10.11333	-7068	7.21E-05	343	0.333333	0.004633	TPRG1L
1231.628	7.888916	-7068	7.22E-05	345	0.25	0.005563	TP53INP1
897.6411	16.28177	-7068	7.10E-05	147	0.333333	0.001472	CHAT
13690.88	7.320004	-7068	7.72E-05	1217	0.333333	0.020274	KLRG2
1430.65	5.001636	-7068	7.24E-05	437	0.333333	0.004983	ADH4
20999.85	8.057114	-7068	7.83E-05	1411	0.333333	0.022825	CNTN4
1520.04	9.401623	-7068	7.22E-05	333	0.333333	0.005083	MYOCD
1783.217	8.135459	-7068	7.24E-05	390	0.333333	0.005415	DUS3L
2679.466	8.208128	-7068	7.31E-05	496	0.333333	0.007829	DIS3L
9076.711	7.270576	-7068	7.58E-05	982	0.333333	0.015855	SESN3
3238.004	6.649775	-7068	7.37E-05	628	0.333333	0.010484	LRIG3
9638.817	7.126905	-7068	7.60E-05	1033	0.333333	0.016813	PRICKLE1
2714.533	7.572135	-7068	7.31E-05	513	0.333333	0.007919	SFRP2

5613.133	9.129082	-7068	7.41E-05	657	0.333333	0.010501	TMEM252
2734.571	7.202971	-7068	7.33E-05	550	0.333333	0.009162	GRPEL2
710.7261	9.742969	-7068	7.13E-05	197	0.333333	0.002271	SCAF1
2542.618	9.648472	-7068	7.26E-05	425	0.333333	0.006534	CDK15
399.5951	7.720285	-7068	7.11E-05	192	0.333333	0.00288	MRPL10
571.2945	8.211591	-7068	7.16E-05	226	0.333333	0.003642	ING5
1206.7	8.635922	-7068	7.20E-05	314	0.333333	0.004738	RNF26
3020.874	11.4175	-7068	7.24E-05	374	0.333333	0.005002	NMRK2
747.2589	6.549326	-7068	7.19E-05	306	0.333333	0.004879	GLIPR2
8598.658	7.079564	-7068	7.58E-05	976	0.333333	0.016108	HHIP
1434.442	10.051	-7068	7.19E-05	293	0.333333	0.004171	ZNF341
1980.633	11.47492	-7068	7.18E-05	281	0.333333	0.003268	SLC22A12
2022.064	8.064596	-7068	7.26E-05	422	0.333333	0.006618	ZNF462
2194.531	8.114151	-7068	7.28E-05	450	0.333333	0.006962	ACOT9
1747.043	7.903016	-7068	7.24E-05	407	0.333333	0.006225	PINX1
1033.205	9.20546	-7068	7.19E-05	274	0.333333	0.004156	FKBP7
955.0069	11.22933	-7068	7.14E-05	203	0.333333	0.002803	HES5
5483.652	6.909494	-7068	7.47E-05	795	0.333333	0.01325	NAV1
1658.082	7.184077	-7068	7.26E-05	418	0.25	0.006765	CCDC80
1546.456	10.36334	-7068	7.19E-05	293	0.333333	0.003607	ZNF503
732.4514	78.2501	-7068	5.82E-05	20	0.25	1.25E-04	SPDYE3
1569.431	15.55113	-7068	7.15E-05	201	0.333333	0.002556	NXF5
362.3504	10.37539	-7068	7.09E-05	142	0.333333	0.002015	ABCC11
1280.477	12.0191	-7068	7.16E-05	242	0.25	0.0035	TSPAN16
1510.985	6.668448	-7068	7.25E-05	418	0.25	0.006594	MEDAG
1199.361	9.157624	-7068	7.19E-05	292	0.333333	0.004379	TCEAL7
4470.296	6.643876	-7068	7.43E-05	747	0.333333	0.012284	PIK3AP1
1359.543	7.240228	-7068	7.24E-05	389	0.333333	0.006528	GPR155
8916.421	9.823237	-7068	7.44E-05	761	0.333333	0.011043	SUCNR1
4040.84	8.52463	-7068	7.35E-05	581	0.333333	0.009502	NUDT12
1111.291	7.397439	-7068	7.21E-05	333	0.333333	0.005456	SLC38A9
831.4838	6.364733	-7068	7.19E-05	317	0.333333	0.004802	SPON2
1546.449	9.516048	-7068	7.21E-05	318	0.333333	0.00452	DCD
6199.062	8.203326	-7068	7.45E-05	743	0.333333	0.011795	CPO
928.8418	5.589661	-7068	7.19E-05	341	0.333333	0.004193	KRT17
382.3862	7.954937	-7068	7.10E-05	180	0.25	0.002563	DEFA3
6616.377	8.227298	-7068	7.45E-05	758	0.333333	0.012133	GKN2
3776.703	7.145275	-7068	7.40E-05	660	0.333333	0.011484	ABCA10
464.8628	6.761733	-7068	7.14E-05	226	0.333333	0.003472	ARL13B
811.2582	8.960728	-7068	7.15E-05	239	0.25	0.003421	MICU3
2403.385	8.570422	-7068	7.20E-05	373	0.333333	0.004487	ZNF624

2394.192	8.914225	-7068	7.27E-05	427	0.333333	0.006321	RPS19BP1
1205.08	12.26256	-7068	7.16E-05	226	0.333333	0.003153	PGLYRP3
3596.577	7.850992	-7068	7.34E-05	578	0.333333	0.00892	ACMSD
2102.475	7.991766	-7068	7.27E-05	445	0.333333	0.007046	BGLAP
1055.239	9.72101	-7068	7.16E-05	257	0.333333	0.003198	PYGO2
600.8101	9.503555	-7068	7.13E-05	203	0.25	0.003053	MRAP2
829.0723	7.872561	-7068	7.16E-05	270	0.333333	0.003987	MPLKIP
3392.084	11.97512	-7068	7.25E-05	386	0.333333	0.005252	SP7
6286.362	7.47976	-7068	7.48E-05	808	0.333333	0.013032	TMEM161B
2001.122	8.836194	-7068	7.26E-05	404	0.333333	0.006677	MMD2
1798.573	9.677928	-7068	7.23E-05	341	0.333333	0.005455	LYPD6
1203.12	8.922562	-7068	7.19E-05	292	0.333333	0.003936	CCDC12
1843.702	11.19575	-7068	7.20E-05	313	0.25	0.004761	PHOSPHO1
145.722	5.867514	-7068	6.91E-05	88	0.333333	5.98E-04	ZNF714
3596.607	7.268444	-7068	7.38E-05	622	0.333333	0.010161	COX18
271.4843	8.246977	-7068	7.07E-05	142	0.333333	0.001658	MYH4
9239.918	7.419794	-7068	7.59E-05	988	0.333333	0.01633	RASEF
3154.042	8.784489	-7068	7.23E-05	438	0.333333	0.005507	ZNF382
44.58652	8.080314	-7068	6.95E-05	60	0.333333	7.55E-04	FAM20C
2566.34	7.733977	-7068	7.31E-05	501	0.333333	0.007972	CD109
1085.339	9.925665	-7068	7.17E-05	262	0.333333	0.00409	FAM83A
2188.737	7.093856	-7068	7.30E-05	495	0.333333	0.008268	GDF6
122.0284	10.47669	-7068	6.99E-05	80	0.25	0.001078	PLPP4
4145.756	7.38637	-7068	7.39E-05	658	0.333333	0.010465	SULF2
2946.957	7.183449	-7068	7.35E-05	575	0.333333	0.009453	FIGNL1
1192.753	11.29095	-7068	7.15E-05	230	0.333333	0.002913	HOXC9
2798.372	8.073307	-7068	7.31E-05	506	0.333333	0.008087	HOXA9
8170.792	7.538532	-7068	7.53E-05	915	0.333333	0.014519	SEC16B
662.7344	9.625114	-7068	7.14E-05	211	0.333333	0.003024	AMDHD2
1278.43	10.43972	-7068	7.17E-05	256	0.333333	0.002942	SEMA4G
3285.295	8.00455	-7068	7.32E-05	528	0.333333	0.007494	S100A16
1416.087	8.592836	-7068	7.20E-05	329	0.333333	0.004748	TMEM125
3512.317	7.664026	-7068	7.35E-05	584	0.333333	0.009008	KCNE3
536.293	6.046996	-7068	7.12E-05	233	0.333333	0.002537	SLC22A7
2135.335	8.673402	-7068	7.26E-05	416	0.333333	0.006884	ZCCHC12
172.541	10.80613	-7068	7.06E-05	95	0.333333	0.001366	ARHGAP23
1635.727	10.97827	-7068	7.19E-05	288	0.333333	0.003961	NPM2
6947.884	7.294285	-7068	7.51E-05	865	0.333333	0.014341	FAT3
7379.414	8.376036	-7068	7.42E-05	720	0.333333	0.011758	SLFN5
985.0125	9.139775	-7068	7.18E-05	275	0.333333	0.00424	PIWIL4
9161.86	7.605804	-7068	7.58E-05	961	0.333333	0.015839	MPP7

181.9308	14.62139	-7068	7.00E-05	74	0.333333	8.10E-04	LURAP1
101.2871	5.702999	-7068	7.05E-05	111	0.333333	0.001674	TCEAL6
4095.01	8.0227	-7068	7.36E-05	609	0.333333	0.009475	NMNAT3
1249.958	6.830498	-7068	7.22E-05	358	0.333333	0.004959	CYP8B1
1547.85	8.652889	-7068	7.23E-05	349	0.333333	0.005475	PHOSPHO2
818.8926	7.428881	-7068	7.18E-05	289	0.25	0.004722	C2orf69
3024.489	8.752933	-7068	7.30E-05	496	0.333333	0.007585	COMTD1
513.668	7.784845	-7068	7.12E-05	210	0.333333	0.003331	MESDC2
1758.657	8.215432	-7068	7.24E-05	379	0.333333	0.006024	CENPW
1044.674	9.286489	-7068	7.16E-05	268	0.333333	0.003551	BCL2L12
4796.341	7.839551	-7068	7.39E-05	668	0.333333	0.010459	NIM1K
1025.369	11.25183	-7068	7.14E-05	211	0.333333	0.002672	CERCAM
3558.619	6.688555	-7068	7.39E-05	659	0.333333	0.010682	JDP2
1693.919	7.204717	-7068	7.24E-05	409	0.333333	0.005553	C4B
2602.494	12.46724	-7068	7.22E-05	328	0.333333	0.004538	GRIN3B
6008.709	7.240875	-7068	7.48E-05	815	0.333333	0.013326	CIT
231.2008	8.640865	-7068	7.07E-05	135	0.333333	0.002086	TRNP1
1559.532	8.015556	-7068	7.23E-05	370	0.333333	0.005501	RTN4IP1
1159.667	8.390706	-7068	7.20E-05	313	0.333333	0.004643	BSCL2
953.8348	10.45674	-7068	7.13E-05	216	0.333333	0.002278	TP53I13
1089.385	13.87535	-7068	7.13E-05	193	0.333333	0.002469	MAFA
390.0755	12.36516	-7068	7.04E-05	114	0.333333	0.001127	KISS1R
1628.873	9.065829	-7068	7.21E-05	330	0.333333	0.004769	CAPSL
5767.245	7.187471	-7068	7.47E-05	800	0.333333	0.013285	COL22A1
381.0552	8.215039	-7068	7.10E-05	174	0.25	0.002248	BCO2
5830.58	7.156791	-7068	7.45E-05	798	0.333333	0.012736	COL23A1
728.8178	8.578558	-7068	7.18E-05	256	0.333333	0.004297	STON2
1342.639	9.047751	-7068	7.18E-05	299	0.25	0.00388	GSTA1
137.0159	9.363436	-7068	7.03E-05	95	0.25	0.00125	RBFOX3
17379.33	7.386871	-7068	7.80E-05	1350	0.333333	0.022	NEGR1
409.8961	9.043555	-7068	7.12E-05	172	0.333333	0.002471	U2AF1L4
2888.906	12.78906	-7068	7.22E-05	334	0.333333	0.003941	RAB4B
3636.906	8.211316	-7068	7.34E-05	564	0.333333	0.009067	STAC2
861.042	5.518403	-7068	7.19E-05	339	0.333333	0.004618	NELFE
746.2327	10.46403	-7068	7.13E-05	203	0.25	0.002757	COX7B2
2403.055	8.088406	-7068	7.28E-05	462	0.25	0.007401	NXPE2
215.1817	9.411558	-7068	7.04E-05	115	0.333333	0.001509	ABCA13
2360.696	8.979115	-7068	7.27E-05	425	0.333333	0.006644	C20orf85
4078.169	8.265439	-7068	7.37E-05	599	0.333333	0.00997	BHLHE22
257.3969	21.94583	-7068	6.92E-05	54	0.25	7.22E-04	FAM47E
13251.77	7.300238	-7068	7.69E-05	1189	0.333333	0.019456	ZNF385B

786.5189	7.583938	-7068	7.19E-05	282	0.333333	0.004693	GBA
609.7125	8.71307	-7068	7.14E-05	215	0.333333	0.002975	ELOVL1
4944.122	7.56647	-7068	7.42E-05	710	0.333333	0.011698	CLYBL
412.8953	6.102809	-7068	7.15E-05	237	0.333333	0.003877	HNRNPA1L2
3207.132	8.41806	-7068	7.24E-05	435	0.333333	0.005012	ZNF567
1290.329	8.256479	-7068	7.21E-05	339	0.333333	0.005214	SFXN2
5113.428	8.287206	-7068	7.40E-05	677	0.333333	0.0105	THRSP
3516.97	7.430291	-7068	7.36E-05	605	0.333333	0.00986	TRERF1
2274.492	7.899878	-7068	7.28E-05	466	0.333333	0.007606	TRAM1L1
306.5265	6.910251	-7068	7.10E-05	161	0.333333	0.002013	KRT80
893.2697	9.298422	-7068	7.17E-05	254	0.333333	0.003954	SLC30A8
934.5619	7.786483	-7068	7.17E-05	275	0.25	0.003563	P2RY13
339.9951	6.648524	-7068	7.10E-05	187	0.333333	0.002498	CARD16
1709.842	8.752268	-7068	7.25E-05	378	0.333333	0.006405	TMEM229B
1417.055	8.759899	-7068	7.21E-05	334	0.333333	0.005178	CCNYL1
2029.749	10.01804	-7068	7.23E-05	360	0.333333	0.005438	RHEBL1
1466.395	11.34282	-7068	7.18E-05	277	0.25	0.00407	CRYGS
2510.077	7.915944	-7068	7.30E-05	485	0.333333	0.007778	WBSCR17
6571.684	7.816418	-7068	7.48E-05	801	0.333333	0.013312	B3GNT7
3097.105	8.980986	-7068	7.31E-05	499	0.25	0.007951	CACNA2D4
3772.617	7.999745	-7068	7.35E-05	585	0.333333	0.009152	BTNL9
1944.64	9.486915	-7068	7.21E-05	327	0.333333	0.003798	SULT1A2
711.7685	10.23223	-7068	7.15E-05	207	0.333333	0.002915	HERC6
895.6742	10.87033	-7068	7.13E-05	203	0.333333	0.00219	MIB2
2775.767	8.429533	-7068	7.31E-05	496	0.25	0.008016	KLC3
1412.754	7.491478	-7068	7.24E-05	380	0.333333	0.006198	PYCR2
2991.042	8.383661	-7068	7.31E-05	515	0.333333	0.008261	FOXQ1
276.1481	8.401789	-7068	6.99E-05	114	0.333333	0.001158	ZNF720
846.0055	7.20178	-7068	7.18E-05	291	0.333333	0.004415	CTDSPL2
3742.855	8.617322	-7068	7.34E-05	555	0.333333	0.008423	TIMM23
2065.716	9.0813	-7068	7.26E-05	406	0.333333	0.006681	SNRNP48
729.7522	9.464245	-7068	7.15E-05	224	0.333333	0.003167	PKN3
1521.159	7.463104	-7068	7.22E-05	377	0.25	0.005668	SAMD9L
2559.187	8.879643	-7068	7.28E-05	443	0.333333	0.007421	ATOH7
4942.162	30.05923	-7068	7.09E-05	182	0.25	0.001513	OR1D5
1067.225	12.74929	-7068	7.14E-05	199	0.333333	0.00251	TREML1
2626.54	14.46605	-7068	7.18E-05	278	0.333333	0.003108	ATXN1L
2696.145	7.096521	-7068	7.33E-05	550	0.25	0.00907	SLITRK6
6593.181	7.262871	-7068	7.50E-05	844	0.333333	0.014035	RASGEF1B
1202.7	8.20307	-7068	7.20E-05	318	0.333333	0.004686	ABCG8
1141.186	8.503709	-7068	7.19E-05	295	0.333333	0.004068	ADGRG7

1517.976	8.397358	-7068	7.21E-05	339	0.333333	0.004521	DUOX1
563.9083	8.917428	-7068	7.14E-05	206	0.333333	0.002857	ASPHD2
7266.833	6.97788	-7068	7.55E-05	921	0.333333	0.015172	TMEM199
815.4612	12.42933	-7068	7.13E-05	181	0.333333	0.002491	CFAP161
136.5334	12.1367	-7068	6.97E-05	76	0.25	0.001038	C4orf46
1105.739	3.894841	-7068	7.21E-05	453	0.333333	0.005503	CENPH
2456.761	9.279296	-7068	7.18E-05	344	0.333333	0.003677	ZNF25
3297.57	7.045422	-7068	7.36E-05	609	0.333333	0.009997	CMTM4
1195.459	9.340191	-7068	7.18E-05	288	0.25	0.004356	NXPH1
531.3886	10.62858	-7068	7.13E-05	177	0.333333	0.002497	MEX3D
4440.324	9.185023	-7068	7.34E-05	566	0.25	0.008698	ANKRD22
3427.961	8.52369	-7068	7.33E-05	535	0.333333	0.008331	LRRC25
10799.09	7.467288	-7068	7.62E-05	1051	0.333333	0.017075	GLIS3
3555.423	11.15006	-7068	7.23E-05	388	0.333333	0.004802	ZIM3
744.6425	10.37296	-7068	7.13E-05	201	0.333333	0.00297	PCDHB5
5372.719	7.401509	-7068	7.45E-05	751	0.333333	0.012099	ELOVL7
747.8384	9.241487	-7068	7.16E-05	231	0.333333	0.003558	ANKRD18A
12157.47	327.0743	-7068	6.91E-05	41	0.333333	4.83E-04	PRAMEF1
1613.087	14.93668	-7068	7.14E-05	205	0.333333	0.002369	EVI5L
411.4974	5.283482	-7068	7.12E-05	229	0.25	0.003039	MPEG1
636.5238	6.671209	-7068	7.15E-05	256	0.333333	0.003465	NLRC4
657.2944	9.875845	-7068	7.13E-05	190	0.333333	0.002598	ATP6V1G3
1248.141	12.29005	-7068	7.12E-05	214	0.25	0.002138	ZNF697
4193.506	7.052166	-7068	7.41E-05	688	0.333333	0.011167	SPECC1
2131.368	8.300871	-7068	7.22E-05	374	0.333333	0.003887	PRAM1
1638.458	8.840939	-7068	7.20E-05	341	0.333333	0.00447	TXNDC17
2574.663	11.71261	-7068	7.23E-05	349	0.333333	0.005326	TEX19
1267.746	8.48174	-7068	7.20E-05	322	0.333333	0.004662	AIFM2
459.2125	9.469554	-7068	7.09E-05	167	0.25	0.001981	TRAPPC5
525.917	7.390057	-7068	7.11E-05	209	0.333333	0.002777	MRPL54
2059.439	10.1732	-7068	7.24E-05	365	0.333333	0.005641	OTOS
1972.718	10.65473	-7068	7.19E-05	313	0.25	0.003895	LDHD
1906.778	6.647229	-7068	7.28E-05	473	0.333333	0.007652	SKA2
6534.168	7.189765	-7068	7.50E-05	847	0.333333	0.014263	CPEB2
7189.2	34.93667	-7068	7.15E-05	221	0.333333	0.002145	OR8B8
1196.633	7.211812	-7068	7.20E-05	340	0.333333	0.004722	TIRAP
911.6438	12.12612	-7068	7.13E-05	200	0.25	0.002989	CATSPERD
1456.143	8.107684	-7068	7.23E-05	362	0.333333	0.005502	SGTB
649.6778	8.106289	-7068	7.15E-05	240	0.333333	0.003749	SCNM1
998.7566	7.523083	-7068	7.17E-05	299	0.25	0.004419	GTF3C6
830.5892	10.79013	-7068	7.15E-05	211	0.333333	0.002693	MYH14

66.03063	9.157003	-7068	6.93E-05	63	0.25	8.51E-04	SLC2A13
1173.163	9.808836	-7068	7.18E-05	273	0.333333	0.0042	SLC26A11
2228.13	7.917961	-7068	7.27E-05	451	0.333333	0.00702	GALNT15
596.705	8.712867	-7068	7.11E-05	198	0.333333	0.002647	ZXDA
388.5448	10.84963	-7068	7.09E-05	145	0.25	0.00202	P4HA3
112.3218	11.69623	-7068	6.97E-05	70	0.25	8.43E-04	KCNRG
714.8726	8.682394	-7068	7.14E-05	220	0.333333	0.003094	SYTL4
77.29416	11.22036	-7068	6.64E-05	43	0.25	4.10E-04	FABP12
1350.621	17.83891	-7068	7.08E-05	122	0.25	0.001478	MAGEB18
530.7619	10.17959	-7068	7.10E-05	169	0.333333	0.002216	BMP8B
4468.803	8.773887	-7068	7.36E-05	597	0.333333	0.009617	IRX2
3019.157	8.580554	-7068	7.28E-05	474	0.333333	0.006669	HOGA1
825.2001	8.410618	-7068	7.15E-05	242	0.333333	0.003482	NOG
1715.598	7.514437	-7068	7.25E-05	417	0.333333	0.006731	SLC16A3
757.9245	8.853318	-7068	7.16E-05	239	0.333333	0.003616	ABCA9
957.6127	8.298876	-7068	7.17E-05	275	0.333333	0.003885	HTRA3
1098.928	9.636323	-7068	7.18E-05	270	0.333333	0.004022	TRIM49
1524.389	12.33749	-7068	7.15E-05	236	0.333333	0.002663	HES4
5028.153	9.093125	-7068	7.35E-05	600	0.333333	0.009218	ANKRD37
409.7303	12.19399	-7068	7.05E-05	119	0.25	0.001418	LRRC39
111.089	9.266118	-7068	6.99E-05	85	0.25	0.001147	DOK7
1404.923	11.20896	-7068	7.17E-05	256	0.25	0.003937	KBTBD12
1279.079	10.42702	-7068	7.18E-05	279	0.333333	0.004085	TRIM59
202.1971	7.785529	-7068	7.06E-05	137	0.25	0.002034	DUSP8
3282.547	6.32952	-7068	7.38E-05	652	0.333333	0.01077	DBNDD2
5300.403	8.577276	-7068	7.41E-05	666	0.333333	0.011142	SPEF2
2889.226	12.55757	-7068	7.18E-05	290	0.333333	0.002873	IL17C
305.8051	8.582291	-7068	7.07E-05	155	0.333333	0.002416	FAM102B
1254.825	5.934871	-7068	7.25E-05	410	0.333333	0.006512	MTFR2
866.056	9.244399	-7068	7.16E-05	247	0.333333	0.003322	EXOSC6
619.4014	8.603801	-7068	7.14E-05	224	0.333333	0.003504	MTIF3
321.0856	7.535852	-7068	7.10E-05	172	0.333333	0.002637	COMMD6
4083.291	6.865732	-7068	7.42E-05	700	0.333333	0.011793	CLIC6
4553.409	7.092763	-7068	7.44E-05	723	0.333333	0.012185	DIS3L2
1904.251	7.046818	-7068	7.29E-05	464	0.333333	0.00768	FAM171B
1305.166	8.377948	-7068	7.19E-05	314	0.25	0.00462	VSIG2
484.2714	7.186689	-7068	7.13E-05	217	0.25	0.003088	CD163L1
4456.161	7.168012	-7068	7.41E-05	701	0.333333	0.011289	FAM43A
2186.641	7.745176	-7068	7.29E-05	460	0.333333	0.007332	ARRDC2
452.7894	11.04969	-7068	7.10E-05	149	0.333333	0.002033	IQCD
6258.924	7.553734	-7068	7.48E-05	802	0.333333	0.01305	CLDN2

3540.815	11.88286	-7068	7.22E-05	346	0.333333	0.003964	GPR142
905.7055	3.660893	-7068	7.21E-05	425	0.333333	0.005311	CENPK
6584.683	8.020178	-7068	7.46E-05	775	0.333333	0.01271	WSCD1
330.2083	9.060977	-7068	7.09E-05	153	0.333333	0.002038	GLMP
811.2598	9.018856	-7068	7.15E-05	238	0.333333	0.003115	RAB3D
956.5084	8.092883	-7068	7.19E-05	298	0.333333	0.004839	VKORC1L1
2362.15	7.849523	-7068	7.29E-05	476	0.333333	0.007794	NANOS1
808.0469	11.10071	-7068	7.14E-05	197	0.333333	0.003128	JPH1
613.7533	11.42191	-7068	7.11E-05	165	0.333333	0.002132	STAC3
1664.052	6.338092	-7068	7.26E-05	442	0.333333	0.006742	ASPM
925.1149	6.414059	-7068	7.18E-05	308	0.333333	0.004043	FCGR1B
1473.499	7.779357	-7068	7.24E-05	381	0.333333	0.005939	PCSK9
889.1242	6.465624	-7068	7.20E-05	324	0.333333	0.004772	AKR1C1
1168.376	4.80639	-7068	7.23E-05	425	0.333333	0.00537	DSN1
1516.233	9.540482	-7068	7.21E-05	323	0.333333	0.004642	CCL26
1839.814	7.16692	-7068	7.27E-05	447	0.333333	0.00724	RDH10
932.3642	8.121146	-7068	7.18E-05	291	0.25	0.004781	SLC16A12
1279.353	7.620504	-7068	7.21E-05	342	0.333333	0.004687	NAT8
856.6535	9.790152	-7068	7.14E-05	224	0.333333	0.003138	BEX5
1184.618	8.904017	-7068	7.19E-05	300	0.25	0.004537	RXFP1
5167.523	11.3286	-7068	7.31E-05	506	0.333333	0.007186	GPR26
436.9075	9.179435	-7068	7.10E-05	169	0.333333	0.002381	LEFTY2
380.6493	9.525784	-7068	7.06E-05	144	0.25	0.001676	RAET1E
2274.083	21.35966	-7068	7.11E-05	197	0.333333	0.00167	SSX2
594.72	12.07337	-7068	7.11E-05	160	0.333333	0.002221	CCDC96
735.9267	7.317259	-7068	7.16E-05	264	0.333333	0.003723	EPB41L4B
1411.043	7.859572	-7068	7.23E-05	369	0.25	0.005914	GP1BB
983.5222	7.497931	-7068	7.20E-05	301	0.333333	0.004544	KCNIP3
2538.005	9.204369	-7068	7.29E-05	457	0.333333	0.007684	SYNE3
2335.939	10.57797	-7068	7.22E-05	365	0.333333	0.005374	NFKBID
651.4521	8.174583	-7068	7.15E-05	240	0.333333	0.003853	SDHAF4
771.6766	8.709059	-7068	7.15E-05	238	0.333333	0.003419	NTF4
396.9065	7.780552	-7068	7.11E-05	185	0.333333	0.002576	SURF6
2035.036	6.627245	-7068	7.28E-05	475	0.333333	0.007148	CDCA5
2006.065	15.13486	-7068	7.16E-05	219	0.333333	0.002244	TRIM16L
4016.749	7.621575	-7068	7.38E-05	637	0.333333	0.010296	HMCN1
1887.426	9.580014	-7068	7.23E-05	364	0.333333	0.005946	IRX3
885.7459	8.025352	-7068	7.16E-05	278	0.333333	0.003416	CLSPN
1753.563	11.79175	-7068	7.20E-05	296	0.333333	0.004746	LDHAL6A
598.7132	8.708532	-7068	7.16E-05	223	0.333333	0.003585	TCP11L2
2730.012	7.67168	-7068	7.30E-05	509	0.25	0.007927	METRNL

1017.115	8.204078	-7068	7.19E-05	299	0.333333	0.005011	DACT2
1242.653	8.703621	-7068	7.21E-05	318	0.333333	0.005119	CPLX4
619.8406	11.93729	-7068	7.10E-05	149	0.25	0.001708	GJA10
1143.673	7.941234	-7068	7.20E-05	306	0.333333	0.004162	C5AR1
5784.365	8.014261	-7068	7.45E-05	742	0.333333	0.011984	EYS
953.4374	10.93952	-7068	7.16E-05	220	0.333333	0.002917	MFRP
2544.552	6.852345	-7068	7.33E-05	553	0.333333	0.00911	RMI2
2568.437	7.162105	-7068	7.33E-05	527	0.333333	0.008307	SKA3
1163.94	11.10413	-7068	7.16E-05	245	0.333333	0.003545	SLC30A2
963.1526	10.71391	-7068	7.15E-05	230	0.333333	0.003201	SYNE4
787.8485	11.74091	-7068	7.14E-05	197	0.333333	0.002862	IL23R
592.5009	9.791092	-7068	7.12E-05	191	0.333333	0.00241	TMEM256
2831.547	7.158725	-7068	7.33E-05	560	0.333333	0.009082	TMEM92
386.0698	9.235455	-7068	7.10E-05	162	0.333333	0.00206	FAM86C1
2402.003	8.537653	-7068	7.27E-05	437	0.333333	0.006838	GLIS1
1429.399	8.788348	-7068	7.23E-05	347	0.333333	0.0058	TMEM26
2857.867	7.353653	-7068	7.34E-05	551	0.333333	0.009177	FTMT
2787.092	11.32362	-7068	7.23E-05	361	0.333333	0.004493	CXCL16
3422.821	6.844026	-7068	7.39E-05	645	0.333333	0.010978	CEP128
1110.144	8.060103	-7068	7.19E-05	312	0.333333	0.004641	HAUS8
469.5612	10.29025	-7068	7.05E-05	142	0.333333	0.001424	KLK4
1839.67	8.938564	-7068	7.22E-05	362	0.333333	0.004839	LAMC3
1070.524	10.75241	-7068	7.16E-05	248	0.25	0.003729	NUDT16
142.0223	9.558272	-7068	7.04E-05	94	0.333333	0.001208	BCL2L15
166.3714	11.776	-7068	6.99E-05	80	0.333333	9.49E-04	FNDC9
6184.495	36.74561	-7068	7.12E-05	194	0.333333	0.00214	OR10P1
172.6884	2.396015	-7068	6.96E-05	153	0.333333	7.32E-04	ZNF568
881.3267	6.000873	-7068	7.09E-05	242	0.25	0.001973	ZNF585A
384.0405	7.851193	-7068	7.12E-05	188	0.333333	0.002909	EPN2
483.9706	11.04186	-7068	7.08E-05	154	0.333333	0.001796	SPRYD4
1088.79	9.413111	-7068	7.18E-05	270	0.333333	0.003958	ZDHHC23
2678.747	8.184111	-7068	7.30E-05	490	0.333333	0.007537	ACOT4
384.3251	11.60779	-7068	7.07E-05	136	0.25	0.001736	PILRB
186.1185	13.46777	-7068	7.00E-05	77	0.333333	8.49E-04	TMEM31
640.9602	12.68202	-7068	7.10E-05	157	0.25	0.002158	SLCO6A1
680.5442	15.34878	-7068	7.09E-05	144	0.333333	0.001924	TDRD9
1134.15	7.771938	-7068	7.22E-05	335	0.333333	0.005547	TMEM86A
832.6156	12.11126	-7068	7.11E-05	177	0.333333	0.002035	SLC16A11
598.6395	10.09833	-7068	7.12E-05	187	0.333333	0.002558	PKDCC
3223.341	7.106314	-7068	7.36E-05	598	0.333333	0.009879	SHISA2
351.0474	10.83706	-7068	7.07E-05	133	0.25	0.001698	CEP126

77.39301	10.94784	-7068	6.91E-05	61	0.25	8.05E-04	C15orf65
3.630486	6.545139	-7068	6.16E-05	15	0.25	1.82E-04	RSPH10B
1298.488	10.0738	-7068	7.16E-05	241	0.25	0.003673	MORN2
533.5736	8.125332	-7068	7.13E-05	214	0.25	0.003226	SIAE
2129.728	8.447986	-7068	7.27E-05	420	0.333333	0.006996	NUP210L
1652.646	4.487147	-7068	7.21E-05	472	0.333333	0.004626	ORM2
1205.125	10.9583	-7068	7.15E-05	242	0.333333	0.003175	ABHD15
746.4125	7.052804	-7068	7.17E-05	277	0.333333	0.004092	ACOT2
403.628	11.42877	-7068	7.06E-05	129	0.333333	0.001378	SLC45A1
3452.71	7.421622	-7068	7.36E-05	604	0.333333	0.0103	SAMD12
196.2622	12.32455	-7068	7.00E-05	82	0.333333	8.82E-04	NYAP1
581.7106	11.34466	-7068	7.13E-05	161	0.333333	0.002262	SERPINA12
359.2555	10.61615	-7068	7.09E-05	144	0.333333	0.00206	ELOVL3
363.1737	12.4527	-7068	7.08E-05	115	0.333333	0.001526	LETM2
612.566	7.065551	-7068	7.14E-05	242	0.333333	0.003222	ZC3H18
794.9988	9.947913	-7068	7.15E-05	225	0.333333	0.003032	HNRNPUL2
496.4276	4.484372	-7068	7.02E-05	199	0.25	0.001234	ZNF404
612.8086	5.248737	-7068	7.05E-05	206	0.25	0.0013	ZNF177
781.0505	9.301522	-7068	7.16E-05	235	0.333333	0.003429	CLDN19
1429.064	9.833716	-7068	7.20E-05	310	0.333333	0.004455	RNF183
604.9597	7.86876	-7068	7.14E-05	237	0.25	0.003426	NDUFA4L2
1932.586	9.337426	-7068	7.22E-05	350	0.333333	0.004726	RHOV
860.1896	9.93704	-7068	7.14E-05	231	0.333333	0.003384	CHMP4C
1465.849	7.44581	-7068	7.25E-05	393	0.333333	0.00647	RAB3IP
1379.18	8.558378	-7068	7.22E-05	343	0.333333	0.005455	GPRIN1
913.2377	9.40318	-7068	7.17E-05	254	0.333333	0.003807	HYKK
984.3709	8.98153	-7068	7.18E-05	267	0.333333	0.004015	GRK7
985.0857	9.398856	-7068	7.17E-05	262	0.25	0.004028	PROM2
1840.365	7.1413	-7068	7.29E-05	461	0.333333	0.00785	UBE3D
549.4732	7.7019	-7068	7.14E-05	223	0.333333	0.003479	VEGFD
835.5862	8.75683	-7068	7.16E-05	252	0.333333	0.003632	GPIHBP1
2029.565	7.194745	-7068	7.29E-05	473	0.333333	0.007876	CDCA2
1340.273	10.80598	-7068	7.18E-05	275	0.333333	0.003613	GSG2
1921.54	8.688517	-7068	7.25E-05	395	0.333333	0.006471	IL31RA
383.5721	11.09802	-7068	7.07E-05	129	0.25	0.001455	EFCAB12
1877.936	8.399281	-7068	7.25E-05	395	0.333333	0.006064	SCARA5
644.2526	14.03092	-7068	7.03E-05	110	0.333333	0.001214	TBC1D3F
145.1091	8.831151	-7068	7.00E-05	95	0.25	0.00116	DAPL1
2170.869	9.279344	-7068	7.23E-05	382	0.25	0.005496	IL20RB
1489.991	10.10211	-7068	7.15E-05	259	0.333333	0.003066	GPR34
1110.715	8.011433	-7068	7.17E-05	287	0.333333	0.003524	P2RY12

315.2685	9.813451	-7068	7.10E-05	140	0.333333	0.001943	HAPLN4
320.9556	10.0775	-7068	7.07E-05	133	0.333333	0.001751	SLC35F1
436.5465	11.89245	-7068	7.08E-05	135	0.333333	0.00168	PRRT3
630.069	8.480972	-7068	7.15E-05	222	0.333333	0.003121	EPPK1
1606.848	9.858944	-7068	7.19E-05	302	0.25	0.004205	ANKRD35
528.1886	11.17741	-7068	7.11E-05	160	0.333333	0.002114	RDH12
1397.226	7.777815	-7068	7.23E-05	364	0.333333	0.005783	ZFYVE28
1746.257	7.07501	-7068	7.27E-05	445	0.333333	0.007475	EPDR1
4030.3	7.961145	-7068	7.37E-05	619	0.333333	0.010356	MAB21L3
249.7822	12.86169	-7068	7.05E-05	97	0.333333	0.001198	MAB21L2
1648.35	7.731376	-7068	7.25E-05	405	0.333333	0.006887	ADAMTS18
1445.112	8.552305	-7068	7.22E-05	340	0.333333	0.005254	KANK4
1399.415	14.46673	-7068	7.12E-05	180	0.333333	0.001734	SPSB4
519.8557	13.0654	-7068	7.06E-05	130	0.333333	0.001427	CLEC4F
384.8908	9.582789	-7068	7.03E-05	121	0.333333	0.001072	OPN1LW
235.5962	14.23786	-7068	7.06E-05	88	0.333333	0.001088	SMCR8
229.5998	8.030183	-7068	7.05E-05	135	0.25	0.002076	FNDC1
1167.959	7.388742	-7068	7.21E-05	336	0.333333	0.004892	RASD1
2180.593	12.46597	-7068	7.22E-05	326	0.333333	0.004973	APLF
3064.169	9.870518	-7068	7.22E-05	384	0.333333	0.004403	ZFP62
342.3378	11.79371	-7068	7.08E-05	118	0.333333	0.00147	CERS1
234.628	9.431384	-7068	7.09E-05	124	0.333333	0.001985	MAP7D2
171.7626	10.1854	-7068	7.04E-05	99	0.333333	0.001299	LYPD6B
2253.355	9.005682	-7068	7.26E-05	414	0.333333	0.006632	FAM83B
661.292	7.128404	-7068	7.15E-05	257	0.333333	0.003869	FNDC3B
772.1514	9.86406	-7068	7.14E-05	215	0.333333	0.002909	RAB43
268.5136	8.863857	-7068	7.06E-05	136	0.333333	0.001637	SDHAF1
158.3195	9.77112	-7068	6.97E-05	91	0.25	0.001038	MUC20
31.26654	12.75656	-7068	6.63E-05	35	0.25	3.72E-04	GLOD5
1304.505	9.961748	-7068	7.17E-05	264	0.333333	0.003449	TMC4
1486.716	10.06405	-7068	7.19E-05	296	0.333333	0.004499	LRR1
173.8496	10.89627	-7068	7.04E-05	92	0.333333	9.96E-04	AGBL3
201.7339	14.69405	-7068	6.99E-05	80	0.333333	9.66E-04	GTPBP10
2399.689	8.374165	-7068	7.29E-05	456	0.333333	0.007448	CEP57L1
971.2817	8.544664	-7068	7.17E-05	282	0.333333	0.004105	PHF19
1021.684	4.787028	-7068	7.22E-05	394	0.333333	0.00548	KIF18B
4855.457	8.231558	-7068	7.39E-05	659	0.333333	0.010224	ZNF367
272.5543	10.63627	-7068	7.08E-05	120	0.333333	0.001651	SLC35D3
717.3768	8.398379	-7068	7.12E-05	224	0.333333	0.002757	CCDC137
493.493	5.8005	-7068	7.15E-05	250	0.333333	0.00332	PPAN
624.0233	9.116176	-7068	7.15E-05	208	0.333333	0.002825	ADAMTS4

728.6383	7.752155	-7068	7.13E-05	236	0.25	0.003016	SLC39A5
741.3062	6.060461	-7068	7.19E-05	318	0.333333	0.004954	CYP4V2
75.87516	8.187944	-7068	6.97E-05	78	0.25	0.001111	ACER2
1177.876	7.502483	-7068	7.12E-05	249	0.333333	0.002184	ZNF616
629.1312	7.530069	-7068	7.14E-05	244	0.333333	0.003731	CDCA7L
544.907	8.983575	-7068	7.13E-05	200	0.333333	0.003112	ATP6V1C2
1006.671	18.29038	-7068	7.05E-05	123	0.333333	0.001084	C20orf144
450.7442	9.092077	-7068	7.08E-05	161	0.333333	0.00218	APITD1
257.8555	9.351915	-7068	7.08E-05	134	0.333333	0.002048	LIN54
643.6349	10.62005	-7068	7.11E-05	186	0.333333	0.002516	TSPAN33
2714.197	9.68379	-7068	7.20E-05	362	0.333333	0.003992	ZIK1
317.8743	7.592769	-7068	7.08E-05	159	0.25	0.001993	TMEM37
428.7747	8.891701	-7068	7.09E-05	160	0.25	0.001982	SLC23A3
446.6606	7.389966	-7068	7.12E-05	199	0.25	0.002762	GSTA5
2423.942	8.318323	-7068	7.28E-05	455	0.333333	0.007218	ADGRD1
814.4079	9.449277	-7068	7.15E-05	233	0.333333	0.003491	MEIOB
36.43923	12.28904	-7068	6.81E-05	40	0.25	5.18E-04	CCSAP
453.6809	10.27429	-7068	7.11E-05	163	0.333333	0.00233	HTRA4
598.2087	10.09794	-7068	7.15E-05	195	0.333333	0.002946	TRPV3
1106.233	9.476747	-7068	7.18E-05	267	0.333333	0.003843	COL6A5
973.7681	8.565418	-7068	7.18E-05	276	0.333333	0.004249	MARVELD2
185.778	12.86039	-7068	7.03E-05	79	0.333333	9.24E-04	SLC46A1
857.0718	9.316258	-7068	7.16E-05	245	0.333333	0.003509	METTL7B
557.9474	12.1549	-7068	7.10E-05	151	0.333333	0.001785	CMBL
152.5097	10.94429	-7068	6.98E-05	86	0.25	0.001079	TPRN
650.1159	8.36798	-7068	7.14E-05	225	0.333333	0.003014	FAHD1
997.9857	14.24999	-7068	7.09E-05	161	0.333333	0.001538	PNPLA5
7877.123	50.59077	-7068	7.15E-05	211	0.333333	0.003105	OR4D5
3484.441	33.05491	-7068	7.08E-05	122	0.25	0.00183	PAGE2
1266.726	9.149384	-7068	7.20E-05	304	0.25	0.004774	C1QL2
850.827	8.392421	-7068	7.17E-05	264	0.25	0.004084	C1QL3
395.6848	8.950606	-7068	7.13E-05	174	0.333333	0.002915	LY6K
5979.457	18.60125	-7068	7.22E-05	366	0.333333	0.00556	UGT1A8
302.2863	9.544544	-7068	7.10E-05	147	0.25	0.002316	TCEANC2
15.831	7.706122	-7068	6.56E-05	32	0.25	3.32E-04	C6orf223
393.6987	13.25273	-7068	7.08E-05	112	0.333333	0.001237	TRIM51
32.83326	7.645904	-7068	6.70E-05	43	0.333333	4.20E-04	PRCD
720.0141	11.38862	-7068	7.11E-05	166	0.333333	0.001868	S1PR3
401.1546	12.99695	-7068	7.09E-05	129	0.333333	0.001469	D2HGDH
702.9348	9.163671	-7068	7.08E-05	176	0.333333	0.001685	OPN1MW
7752.343	46.26417	-7068	7.11E-05	176	0.333333	0.001686	KRTAP2-1

419.1705	8.185326	-7068	7.11E-05	178	0.333333	0.002376	PER1
1007.956	9.868928	-7068	7.12E-05	193	0.333333	0.001855	GPR119
408.2379	12.56148	-7068	7.08E-05	129	0.333333	0.001439	ASB16
465.1902	9.323125	-7068	7.11E-05	180	0.333333	0.002578	ATP6V0D2
716.6798	14.87161	-7068	7.10E-05	151	0.25	0.002391	SYCE1
697.8442	8.646471	-7068	7.11E-05	222	0.333333	0.00306	CHAC2
615.9857	8.111181	-7068	7.11E-05	216	0.25	0.002961	CHCHD4
118.8819	6.77302	-7068	7.05E-05	115	0.25	0.001865	FRMD3
232.0305	9.386022	-7068	7.06E-05	119	0.333333	0.001624	TMEM139
848.4986	10.67452	-7068	7.13E-05	204	0.333333	0.002288	FCAMR
47.58327	11.88717	-7068	6.78E-05	44	0.25	5.40E-04	LGALS16
841.3657	9.940762	-7068	7.12E-05	208	0.333333	0.002374	NECTIN4
217.5841	10.40971	-7068	7.06E-05	114	0.25	0.001697	DCANP1
205.7367	14.70092	-7068	6.95E-05	75	0.25	8.50E-04	TMEM99
518.5918	7.250174	-7068	7.08E-05	197	0.333333	0.002125	NAA38
364.6877	11.48762	-7068	7.06E-05	125	0.25	0.001518	KIR3DL2
270.0307	8.367423	-7068	7.05E-05	130	0.25	0.001528	SLC47A2
310.5287	3.647472	-7068	7.02E-05	173	0.333333	0.001084	ZNF845
1129.903	6.964971	-7068	7.12E-05	263	0.333333	0.002444	ZNF416
600.8492	10.49825	-7068	7.12E-05	186	0.333333	0.002558	CEP78
155.9626	8.975836	-7068	7.06E-05	108	0.25	0.001504	MPV17L
325.5967	9.635297	-7068	7.05E-05	138	0.333333	0.001712	RAB42
494.9829	11.77925	-7068	7.12E-05	154	0.333333	0.00216	MB21D1
664.7947	16.70692	-7068	7.07E-05	119	0.25	0.001197	CCDC155
3610.852	33.48099	-7068	7.06E-05	131	0.25	0.001701	CT45A5
256.0434	13.7732	-7068	6.96E-05	81	0.333333	8.19E-04	ADGRF2
646.6363	10.65609	-7068	7.08E-05	166	0.333333	0.001669	IL20
876.0843	12.35986	-7068	7.12E-05	195	0.333333	0.002439	TSTD1
145.3151	12.14262	-7068	7.04E-05	81	0.333333	0.001099	GDPD1
596.2155	8.893747	-7068	7.12E-05	199	0.25	0.002841	SRSF12
423.4637	9.888198	-7068	7.08E-05	154	0.333333	0.002117	HYLS1
1563.166	8.101782	-7068	7.23E-05	374	0.333333	0.005779	DDIAS
712.4945	13.26343	-7068	7.13E-05	178	0.333333	0.00273	PIWIL3
372.5258	12.90843	-7068	7.05E-05	117	0.333333	0.001477	RADIL
100.1202	11.1657	-7068	6.86E-05	62	0.25	6.25E-04	CSTL1
344.4168	15.43565	-7068	6.98E-05	85	0.333333	7.55E-04	COX6B2
602.8106	17.01813	-7068	7.00E-05	99	0.25	9.17E-04	C7orf61
252.5089	10.82595	-7068	7.06E-05	111	0.333333	0.00151	SPANXD
800.5522	13.81375	-7068	7.06E-05	143	0.333333	0.001288	TMEM114
1785.526	16.31044	-7068	7.15E-05	213	0.333333	0.002351	KRT40
2021.353	34.65442	-7068	6.88E-05	91	0.25	6.30E-04	OR51B4

373.6417	3.857488	-7068	7.14E-05	268	0.333333	0.003593	CENPN
378.6446	8.19132	-7068	7.05E-05	160	0.333333	0.001932	C1QTNF5
130.5495	15.0703	-7068	6.88E-05	57	0.25	6.36E-04	CCDC172
156.1957	12.30804	-7068	6.96E-05	72	0.333333	8.27E-04	CASKIN1
517.8602	11.42635	-7068	7.04E-05	125	0.333333	0.00113	NKX1-2
691.4035	3.601854	-7068	7.18E-05	375	0.25	0.004224	CFHR1
494.1365	10.52665	-7068	7.09E-05	156	0.333333	0.001807	VSIG10L
663.7233	15.41977	-7068	7.07E-05	123	0.333333	0.00122	IL27
259.2833	11.24594	-7068	7.01E-05	102	0.333333	0.001127	MUC12
63.7744	11.06517	-7068	6.68E-05	45	0.333333	3.71E-04	C11orf40
264.3151	12.04782	-7068	7.05E-05	97	0.333333	0.001228	ACP7
765.0445	9.130524	-7068	7.15E-05	236	0.333333	0.003446	SFTPA1
103.7342	14.5277	-7068	6.58E-05	35	0.333333	3.06E-04	TBC1D3B
489.5606	16.70066	-7068	7.00E-05	98	0.25	8.94E-04	BRICD5
689.7106	9.703636	-7068	7.13E-05	206	0.333333	0.002663	SBK1
441.1793	7.863027	-7068	7.12E-05	191	0.333333	0.002579	ARHGAP30
384.9787	8.197041	-7068	7.09E-05	172	0.25	0.002209	CLDN23
740.4065	12.01025	-7068	7.10E-05	173	0.333333	0.001855	CRB3
106.6831	13.65018	-7068	6.97E-05	65	0.25	8.89E-04	ALDH1L2
667.2257	7.91604	-7068	7.14E-05	235	0.25	0.003378	PPARGC1B
372.3009	13.09383	-7068	7.03E-05	109	0.333333	0.001197	PRSS38
523.2903	17.25851	-7068	7.03E-05	98	0.333333	9.34E-04	TXNDC2
521.5008	6.1844	-7068	7.12E-05	238	0.25	0.00319	MT1M
289.1374	11.79921	-7068	7.04E-05	112	0.333333	0.001324	HDDC3
496.2435	7.371098	-7068	7.12E-05	214	0.333333	0.003002	TCF19
284.7606	9.766328	-7068	7.07E-05	132	0.333333	0.001761	RCCD1
227.8486	8.057743	-7068	7.06E-05	129	0.333333	0.001634	FAM110C
2687.392	14.46438	-7068	7.10E-05	219	0.25	0.002616	UGT1A6
324.7585	10.42135	-7068	7.05E-05	130	0.333333	0.001783	TMEM181
54.83711	12.77589	-7068	6.86E-05	44	0.25	5.36E-04	FAM161B
973.9043	15.13166	-7068	7.10E-05	159	0.333333	0.001625	SMIM1
683.3066	5.324706	-7068	7.17E-05	303	0.333333	0.004251	MND1
442.6414	6.640248	-7068	7.09E-05	190	0.333333	0.002862	MZT1
756.2685	6.778485	-7068	7.15E-05	274	0.25	0.00399	C11orf96
471.9493	10.21883	-7068	7.07E-05	164	0.25	0.002032	CCDC107
1405.321	11.40474	-7068	7.10E-05	203	0.333333	0.00127	ZNF90
284.21	22.17739	-7068	6.83E-05	54	0.25	4.71E-04	FAM209B
113.9625	6.975899	-7068	7.00E-05	99	0.333333	0.001261	PLXNA4
722.3755	11.34684	-7068	7.13E-05	180	0.333333	0.002145	PLIN4
464.6983	11.45365	-7068	7.05E-05	133	0.333333	0.001415	SNAI3
821.2671	11.80028	-7068	7.10E-05	174	0.333333	0.00186	ZNF296

100.4235	14.10173	-7068	6.80E-05	47	0.25	4.56E-04	ATP6AP1L
170.176	14.27792	-7068	6.98E-05	67	0.333333	7.20E-04	COX19
237.6734	8.589642	-7068	7.04E-05	133	0.25	0.001951	ITPRIPL2
825.3158	9.346071	-7068	7.13E-05	205	0.25	0.002824	TWIST2
146.9101	14.14591	-7068	6.82E-05	59	0.333333	5.53E-04	RHOXF1
506.0456	11.60181	-7068	7.09E-05	147	0.25	0.001883	PPP3R2
281.7295	16.74718	-7068	6.92E-05	70	0.333333	6.15E-04	C17orf64
1007.488	8.925264	-7068	7.15E-05	255	0.25	0.00348	ZNF469
401.5761	8.711648	-7068	7.10E-05	164	0.333333	0.002357	SBK2
92.65055	10.26048	-7068	6.99E-05	60	0.25	6.29E-04	TSPAN10
491.21	8.840668	-7068	7.08E-05	177	0.333333	0.002222	MCEMP1
292.7217	8.015656	-7068	7.10E-05	164	0.333333	0.002692	LRRC8C
446.5704	6.477673	-7068	7.12E-05	212	0.333333	0.002715	A1BG
364.276	5.455511	-7068	7.12E-05	211	0.333333	0.002538	SAA2
711.4025	8.219525	-7068	7.13E-05	232	0.25	0.002992	LRG1
129.7815	16.76875	-7068	6.85E-05	50	0.333333	5.09E-04	B4GALNT4
371.964	11.51331	-7068	7.09E-05	134	0.333333	0.001754	KIF7
510.0533	15.3819	-7068	7.06E-05	117	0.25	0.001297	CATSPER1
160.6487	12.68286	-7068	6.97E-05	79	0.333333	8.96E-04	DERL3
524.7189	10.44818	-7068	7.10E-05	168	0.333333	0.002068	CCL28
223.9924	9.360432	-7068	7.02E-05	106	0.25	0.001468	AHRR
219.4566	12.62915	-7068	7.00E-05	88	0.333333	9.80E-04	ZP1
803.375	16.05318	-7068	7.04E-05	131	0.333333	0.001086	ZNF114
811.8271	16.40956	-7068	7.05E-05	122	0.25	0.001298	ANKRD30B
394.1559	18.31966	-7068	7.07E-05	91	0.333333	0.001007	PLD6
1377.48	27.35362	-7068	6.99E-05	78	0.333333	5.60E-04	OR7G3
937.0885	20.461	-7068	6.65E-05	73	0.333333	3.65E-04	OR1L3
387.2804	8.68048	-7068	7.10E-05	167	0.333333	0.002591	GPX8
160.9029	7.803868	-7068	7.00E-05	108	0.333333	0.00144	ALG11
1746.605	31.89624	-7068	6.88E-05	85	0.333333	6.48E-04	OR4K1
98.59585	6.857726	-7068	6.95E-05	89	0.25	0.00103	SFTA2
183.596	12.01698	-7068	6.96E-05	82	0.25	9.54E-04	SFTA3
185.1818	16.87445	-7068	6.89E-05	60	0.25	5.91E-04	GPX6
96.93267	10.86593	-7068	6.66E-05	52	0.25	4.39E-04	NPSR1
226.4395	9.87587	-7068	7.04E-05	115	0.25	0.001605	SAMD5
319.389	6.985704	-7068	7.09E-05	177	0.333333	0.002668	PRDM6
60.58086	12.06913	-7068	6.88E-05	50	0.25	6.38E-04	TDRP
318.3011	16.93749	-7068	7.05E-05	90	0.333333	0.001085	NLRP7
54.81232	7.205307	-7068	6.94E-05	68	0.25	9.90E-04	C3orf80
480.8115	11.6587	-7068	7.07E-05	138	0.333333	0.002044	GXYLT2
102.1591	10.62698	-7068	7.01E-05	78	0.25	0.001136	FAM84B

113.2269	9.379545	-7068	7.02E-05	87	0.333333	0.00123	SYDE2
19.45059	17.01489	-7068	6.48E-05	24	0.25	2.59E-04	VMAC
392.2094	5.073373	-7068	6.95E-05	148	0.333333	6.23E-04	ZNF554
4.592059	7.514126	-7068	6.40E-05	9	0.25	1.45E-04	GOLGA6L10
74.87883	6.252512	-7068	6.49E-05	37	0.25	3.53E-04	GOLGA6L9
10609.2	5.87825	-7068	7.58E-05	1172	0.333333	0.014432	RPS23
26004.11	7.120217	-7068	7.90E-05	1648	0.333333	0.023609	CDH3
8281.717	6.464636	-7068	7.51E-05	977	0.333333	0.013017	HLA-DMB
6985.8	6.674182	-7068	7.47E-05	887	0.333333	0.011464	ALAS1
20813.57	6.364802	-7068	7.87E-05	1625	0.333333	0.023429	GOT2
18743.72	5.638628	-7068	7.84E-05	1646	0.333333	0.022532	SNW1
15326.45	7.076996	-7068	7.69E-05	1269	0.333333	0.017707	COL17A1
8784.651	6.219603	-7068	7.51E-05	1023	0.333333	0.012636	RPL27
7901.54	5.388437	-7068	7.56E-05	1066	0.333333	0.014335	C1QBP
19767.03	6.309147	-7068	7.81E-05	1551	0.333333	0.0203	PSMD2
12881.79	6.337211	-7068	7.69E-05	1283	0.333333	0.018971	CD9
14718.83	6.549157	-7068	7.64E-05	1279	0.333333	0.016118	RPL11
4182.538	5.274979	-7068	7.42E-05	807	0.333333	0.01145	CYR61
10091.72	6.029288	-7068	7.54E-05	1114	0.333333	0.013378	RPS16
9785.162	6.701306	-7068	7.57E-05	1066	0.333333	0.015063	PPIB
9944.653	6.466542	-7068	7.58E-05	1080	0.333333	0.01489	GBP2
14523.16	5.887521	-7068	7.65E-05	1350	0.333333	0.016356	RPS5
20029.73	6.005674	-7068	7.87E-05	1635	0.333333	0.022602	CCT7
23094.42	6.691056	-7068	7.85E-05	1617	0.333333	0.022683	ADSL
19102.6	5.846409	-7068	7.79E-05	1589	0.333333	0.019961	FBL
522169.9	9.843176	-7068	1.06E-04	5253	0.333333	0.063788	UBC
72974.44	7.665748	-7068	8.35E-05	2564	0.333333	0.033334	APP
29616.14	8.258898	-7068	7.90E-05	1624	0.333333	0.022876	NTRK1
31961.64	5.124484	-7068	8.06E-05	2220	0.333333	0.027587	XPO1
24455.46	6.037019	-7068	7.85E-05	1750	0.333333	0.021506	MCM2
22974.88	6.431118	-7068	7.91E-05	1693	0.333333	0.024359	VCAM1
16459.78	6.8856	-7068	7.69E-05	1322	0.333333	0.018135	CUL3
33208.44	5.405169	-7068	8.09E-05	2249	0.333333	0.028979	EGFR
21323.84	5.41242	-7068	7.87E-05	1786	0.333333	0.023241	SUMO1
23524.7	6.654551	-7068	7.77E-05	1488	0.333333	0.019617	COPS5
7840.669	7.100063	-7068	7.49E-05	909	0.333333	0.013004	CLK1
45121.97	5.645794	-7068	8.31E-05	2564	0.333333	0.03441	FN1
25816.89	5.674051	-7068	8.00E-05	1930	0.333333	0.02744	EPS15
19968.61	6.214179	-7068	7.80E-05	1597	0.333333	0.021349	MYC
9039.161	6.601771	-7068	7.57E-05	1039	0.333333	0.015394	CUL5
24130.45	6.015461	-7068	7.93E-05	1790	0.333333	0.024281	RPA3

10876.28	7.058381	-7068	7.59E-05	1107	0.333333	0.015707	ITGA4
13319.89	6.122345	-7068	7.71E-05	1332	0.333333	0.019512	FBXW11
9288.233	6.183067	-7068	7.57E-05	1074	0.333333	0.01431	NEDD8
12893.68	6.002411	-7068	7.68E-05	1320	0.333333	0.018496	CUL1
12495.58	5.78676	-7068	7.66E-05	1308	0.333333	0.017236	RPA2
40591.37	6.861624	-7068	8.14E-05	2061	0.333333	0.030638	CAND1
12803.09	7.096923	-7068	7.60E-05	1146	0.333333	0.014699	RPL32
11907.08	7.932622	-7068	7.56E-05	1013	0.333333	0.012928	IRF3
12226.46	8.761892	-7068	7.53E-05	963	0.333333	0.013323	TSC1
27698.75	6.946086	-7068	7.84E-05	1626	0.333333	0.023112	MDM2
15039.47	7.669608	-7068	7.65E-05	1219	0.333333	0.018395	KRAS
11462.82	7.159816	-7068	7.57E-05	1076	0.333333	0.014233	RPL37
9462.703	6.334901	-7068	7.54E-05	1060	0.333333	0.013211	EEF2
7980.843	7.066097	-7068	7.45E-05	888	0.333333	0.011064	DLG4
12268.03	5.38332	-7068	7.70E-05	1369	0.333333	0.018993	HDAC2
19019.18	6.970971	-7068	7.77E-05	1413	0.333333	0.020117	CTR9
8520.746	6.775558	-7068	7.49E-05	950	0.333333	0.011516	RPL19
10473.95	8.223945	-7068	7.54E-05	944	0.333333	0.012668	BCAP31
18446.22	8.128996	-7068	7.73E-05	1282	0.333333	0.018436	GBF1
9607.088	4.999587	-7068	7.60E-05	1243	0.333333	0.01585	FUS
21827.55	7.679697	-7068	7.80E-05	1450	0.333333	0.020708	IREB2
24174.74	5.846031	-7068	7.95E-05	1825	0.333333	0.025704	MAP3K7
11294.69	6.525479	-7068	7.63E-05	1164	0.333333	0.016663	TSG101
19037.97	7.302758	-7068	7.76E-05	1399	0.333333	0.019809	HTT
10312.08	6.698106	-7068	7.55E-05	1059	0.333333	0.013284	IL2RG
11966.26	4.82698	-7068	7.65E-05	1430	0.333333	0.017313	CDK2
8242.385	7.09005	-7068	7.50E-05	911	0.333333	0.012135	ERCC3
22477.7	6.43538	-7068	7.84E-05	1637	0.333333	0.022198	ABL1
5336.794	5.980389	-7068	7.42E-05	812	0.333333	0.010198	CD3D
13057.92	5.703088	-7068	7.65E-05	1328	0.333333	0.016123	AKT1
7734.851	7.205895	-7068	7.51E-05	909	0.333333	0.013466	MAML1
13437.11	6.832775	-7068	7.69E-05	1241	0.333333	0.018976	UBE4A
12999.83	8.037562	-7068	7.58E-05	1057	0.333333	0.014366	CLK2
24950.03	6.446833	-7068	7.94E-05	1746	0.333333	0.025269	ACVR1
24931.02	5.894108	-7068	7.96E-05	1858	0.333333	0.026265	PPP2CA
25922.17	5.43507	-7068	8.03E-05	1987	0.333333	0.027938	POLR2B
9022.793	5.617325	-7068	7.58E-05	1132	0.333333	0.01501	APEX1
12301.81	7.050479	-7068	7.62E-05	1148	0.333333	0.016389	PAXIP1
20631.64	6.938218	-7068	7.83E-05	1521	0.333333	0.022193	ZHX2
19302	5.878762	-7068	7.78E-05	1615	0.333333	0.020335	ESR1
19578.74	5.993888	-7068	7.84E-05	1618	0.333333	0.022802	FLI1

13405.18	5.286549	-7068	7.73E-05	1473	0.333333	0.020485	YWHAZ
28613.65	6.820889	-7068	7.97E-05	1786	0.333333	0.024391	TRIM28
10251.62	7.253044	-7068	7.52E-05	980	0.333333	0.012751	CD27
26566.51	6.389029	-7068	7.99E-05	1822	0.333333	0.027028	ATXN1
10366.74	5.001129	-7068	7.61E-05	1296	0.333333	0.01636	RPA1
20369.98	9.453199	-7068	7.65E-05	1159	0.333333	0.014932	MAP3K10
20447.03	5.113505	-7068	7.88E-05	1814	0.333333	0.023942	FYN
31375.54	16.02092	-7068	7.63E-05	1159	0.333333	0.016186	GNB1
20602.45	6.599772	-7068	7.85E-05	1576	0.333333	0.022667	IRF2
17959.54	5.588634	-7068	7.80E-05	1611	0.333333	0.021418	BARD1
14231.72	6.294678	-7068	7.71E-05	1335	0.333333	0.019211	BIRC2
14845.76	5.104033	-7068	7.73E-05	1505	0.333333	0.019267	DHX15
9986.275	5.731355	-7068	7.56E-05	1148	0.333333	0.013647	RPLP0
14041.59	5.938833	-7068	7.73E-05	1404	0.333333	0.020033	KIT
12815.83	6.040166	-7068	7.68E-05	1296	0.333333	0.018055	CHUK
16596.4	6.113044	-7068	7.77E-05	1473	0.333333	0.020651	VCP
10483.04	7.035287	-7068	7.59E-05	1045	0.333333	0.015288	EMC2
8766.156	7.647565	-7068	7.52E-05	918	0.333333	0.013324	OPRK1
11030.44	5.928781	-7068	7.64E-05	1225	0.333333	0.017472	PIK3CA
13257.56	7.257303	-7068	7.65E-05	1191	0.333333	0.017314	PFKM
6724.93	5.655387	-7068	7.52E-05	990	0.333333	0.014431	DARS
8087.121	7.210419	-7068	7.49E-05	899	0.333333	0.011612	MAP4K1
10421.24	6.558682	-7068	7.59E-05	1118	0.333333	0.01532	DDB1
7308.775	5.711372	-7068	7.52E-05	1018	0.333333	0.01404	EED
18609.7	5.762072	-7068	7.82E-05	1599	0.333333	0.021844	CD53
7543.614	4.541415	-7068	7.52E-05	1142	0.333333	0.013272	HNRNPA1
6129.333	5.409369	-7068	7.50E-05	957	0.333333	0.013365	SUZ12
20395.87	5.41731	-7068	7.81E-05	1724	0.333333	0.021143	HDAC1
10486.93	7.420651	-7068	7.55E-05	1010	0.333333	0.013699	RAB5B
7323.586	6.794778	-7068	7.46E-05	886	0.333333	0.011092	RPS17
11067.73	7.035219	-7068	7.59E-05	1071	0.333333	0.01423	CDC37
9329.996	7.103743	-7068	7.53E-05	991	0.333333	0.013055	HDAC3
13800.16	5.934856	-7068	7.72E-05	1360	0.333333	0.018579	PRMT1
7959.401	7.108143	-7068	7.51E-05	925	0.333333	0.012988	HDAC5
7198.523	7.35995	-7068	7.46E-05	834	0.333333	0.010869	DGKA
7588.648	5.424892	-7068	7.51E-05	1043	0.333333	0.01318	RPL6
9135.76	5.951541	-7068	7.56E-05	1070	0.333333	0.013829	EIF3B
17077.94	5.283417	-7068	7.80E-05	1625	0.333333	0.021546	HNRNPA2B1
25445.68	5.652628	-7068	7.99E-05	1912	0.333333	0.026366	PPP1CC
11768.88	8.544456	-7068	7.53E-05	948	0.333333	0.011891	GDI1
8064.644	7.71963	-7068	7.50E-05	869	0.333333	0.012903	RNF139

10666.66	6.752594	-7068	7.58E-05	1086	0.333333	0.014278	EIF3I
4352.307	5.652093	-7068	7.37E-05	758	0.333333	0.00886	RPS15
22765.99	6.466839	-7068	7.88E-05	1652	0.333333	0.023465	CPE
12963.73	6.253702	-7068	7.69E-05	1306	0.333333	0.019197	SMURF2
31727.48	5.273587	-7068	8.08E-05	2213	0.333333	0.02906	CTNNB1
29764.58	6.369688	-7068	8.03E-05	1930	0.333333	0.026824	CLIC1
35579.22	5.983953	-7068	8.16E-05	2180	0.333333	0.031096	PPP3CA
18155.57	5.627967	-7068	7.80E-05	1588	0.333333	0.020058	CDK4
7383.537	6.773933	-7068	7.48E-05	910	0.333333	0.012038	ATF4
8569.05	8.12596	-7068	7.48E-05	845	0.333333	0.011569	CHGB
15263.2	6.641671	-7068	7.68E-05	1324	0.333333	0.017991	CSNK2A2
14091.39	7.394052	-7068	7.68E-05	1212	0.333333	0.018064	CCNG1
14861.72	8.121477	-7068	7.63E-05	1098	0.333333	0.014348	KRT2
22598.55	6.344585	-7068	7.90E-05	1679	0.333333	0.024137	CTNND2
11994	6.833411	-7068	7.61E-05	1117	0.333333	0.016308	DPM1
11865.07	6.907213	-7068	7.66E-05	1181	0.333333	0.017593	HDGF
14946.76	9.615473	-7068	7.57E-05	985	0.333333	0.012602	SMO
7749.825	7.140372	-7068	7.48E-05	883	0.333333	0.011498	APRT
6750.14	9.553082	-7068	7.39E-05	660	0.333333	0.008698	CPA1
10836.97	6.338629	-7068	7.64E-05	1178	0.333333	0.017508	DCK
12898.37	5.497713	-7068	7.73E-05	1398	0.333333	0.020085	NUP153
18211.08	5.602975	-7068	7.80E-05	1619	0.333333	0.021897	HSP90AA1
22986.29	4.963556	-7068	7.97E-05	1994	0.333333	0.026988	CSNK2A1
15328.5	6.162701	-7068	7.72E-05	1407	0.333333	0.018902	CDK7
12371.77	6.9529	-7068	7.64E-05	1174	0.333333	0.017252	HIVEP1
10882.21	8.893915	-7068	7.51E-05	910	0.333333	0.012442	KAT2A
9223.764	7.965436	-7068	7.51E-05	887	0.333333	0.011667	CLPTM1
8929.874	9.055235	-7068	7.46E-05	819	0.333333	0.011975	LIG4
8521.247	5.976154	-7068	7.53E-05	1047	0.333333	0.013606	IL2RB
12543.52	6.516486	-7068	7.63E-05	1217	0.333333	0.016526	KAT2B
13890.89	7.843001	-7068	7.65E-05	1137	0.333333	0.015995	ASCC2
14190.08	5.481732	-7068	7.77E-05	1482	0.333333	0.021661	RAD21
11816.7	7.333004	-7068	7.61E-05	1114	0.333333	0.016299	TERF2
9531.928	7.80879	-7068	7.54E-05	948	0.333333	0.013893	ZCCHC11
11083.5	6.332976	-7068	7.60E-05	1143	0.333333	0.015224	SELL
21976.45	6.571417	-7068	7.88E-05	1624	0.333333	0.023208	ATP5J
12505.76	5.716706	-7068	7.67E-05	1338	0.333333	0.018642	SMAD2
13466.11	5.565631	-7068	7.73E-05	1398	0.333333	0.019589	PSMC6
8837.682	8.056987	-7068	7.50E-05	884	0.333333	0.012032	TBCB
12575.8	5.325897	-7068	7.69E-05	1382	0.333333	0.01841	MSH2
6431.286	7.13641	-7068	7.48E-05	829	0.333333	0.012724	FMR1

10827.32	8.109245	-7068	7.50E-05	939	0.333333	0.012131	PAX6
11052.77	5.895971	-7068	7.60E-05	1207	0.333333	0.015605	EEF1A1
9535.161	5.749269	-7068	7.59E-05	1160	0.333333	0.016326	SMAD4
5761.675	6.841364	-7068	7.47E-05	814	0.333333	0.012458	TSNAX
15767.97	6.643175	-7068	7.71E-05	1327	0.333333	0.018599	USP1
12937.27	7.122108	-7068	7.63E-05	1153	0.333333	0.015057	HRAS
8857.116	5.172697	-7068	7.55E-05	1133	0.333333	0.013346	SNRPD2
10369.32	6.037675	-7068	7.61E-05	1169	0.333333	0.016489	ATR
7313.053	7.040525	-7068	7.45E-05	870	0.333333	0.011634	RPS12
9756.922	7.864916	-7068	7.53E-05	953	0.333333	0.013399	ALOX12
13926.84	8.458187	-7068	7.61E-05	1053	0.333333	0.01416	XDH
11235.51	7.169228	-7068	7.59E-05	1085	0.333333	0.015153	TUBB2A
8201.075	5.641424	-7068	7.54E-05	1072	0.333333	0.014171	TRAF6
12513.93	5.386178	-7068	7.69E-05	1353	0.333333	0.018229	DLD
7465.73	6.316338	-7068	7.52E-05	959	0.333333	0.014123	TOP2B
15150.96	7.593028	-7068	7.66E-05	1205	0.333333	0.016309	RPS6KA1
7141.2	6.897196	-7068	7.47E-05	867	0.333333	0.012357	MS4A1
24711.41	6.416134	-7068	7.92E-05	1743	0.333333	0.023765	ACTN4
7968.205	8.233278	-7068	7.46E-05	828	0.333333	0.011308	POU2F1
16017.45	6.182462	-7068	7.74E-05	1395	0.333333	0.018511	NME1
7701.143	6.447565	-7068	7.53E-05	959	0.333333	0.014012	UBR5
25954.84	7.54595	-7068	7.85E-05	1602	0.333333	0.022579	SNCA
6376.161	8.566284	-7068	7.41E-05	702	0.333333	0.009415	MADD
24450.74	7.286175	-7068	7.87E-05	1580	0.333333	0.023081	BMPR1B
8648.941	6.60394	-7068	7.57E-05	1005	0.333333	0.015455	MORC3
5223.51	6.328043	-7068	7.45E-05	826	0.333333	0.012447	CUL4A
13415.48	10.07443	-7068	7.53E-05	910	0.333333	0.012488	LZTR1
10094.26	6.888044	-7068	7.60E-05	1064	0.333333	0.016339	ZMYM4
11050.63	6.606335	-7068	7.62E-05	1145	0.333333	0.016656	MAP3K4
9534.118	7.668241	-7068	7.54E-05	954	0.333333	0.01331	OS9
18813.99	6.566406	-7068	7.78E-05	1462	0.333333	0.02088	DEK
6741.162	7.265261	-7068	7.48E-05	831	0.333333	0.012576	ZDHHC17
21719.65	6.395005	-7068	7.93E-05	1675	0.333333	0.025685	BZW1
17037.82	6.447475	-7068	7.79E-05	1452	0.333333	0.021534	ADD3
24343.44	7.183971	-7068	7.88E-05	1619	0.333333	0.023475	TUBA1A
14828.46	5.901026	-7068	7.75E-05	1413	0.333333	0.020167	MRPL3
15003.92	6.184305	-7068	7.72E-05	1363	0.333333	0.019052	DDX21
10288.52	7.581766	-7068	7.55E-05	1007	0.333333	0.013943	CD81
38414.18	6.839398	-7068	8.16E-05	2075	0.333333	0.030442	DLG2
12532.15	6.559962	-7068	7.64E-05	1216	0.333333	0.017397	SP3
22934.38	6.867958	-7068	7.82E-05	1558	0.333333	0.021449	SH3GL2

9989.817	7.081027	-7068	7.56E-05	1025	0.333333	0.014709	THOC1
7796.568	7.885349	-7068	7.47E-05	847	0.333333	0.011992	XPA
15262.89	6.769567	-7068	7.69E-05	1308	0.333333	0.01804	RPL34
15139.12	5.51296	-7068	7.69E-05	1482	0.333333	0.01831	GRB2
7385.375	7.678553	-7068	7.49E-05	852	0.333333	0.012642	CAMLG
4594.301	7.358426	-7068	7.38E-05	680	0.333333	0.009898	PMS1
11542.85	6.754887	-7068	7.63E-05	1160	0.333333	0.01725	PCF11
10288.85	6.41176	-7068	7.62E-05	1122	0.333333	0.017002	RB1CC1
20310.63	6.870658	-7068	7.84E-05	1511	0.333333	0.022781	STAM
32048.15	6.002176	-7068	8.08E-05	2085	0.333333	0.028859	HNRNPD
13629.13	6.238042	-7068	7.67E-05	1307	0.333333	0.017478	ILK
15637.56	6.15841	-7068	7.74E-05	1413	0.333333	0.02015	RBM39
24951.62	6.710666	-7068	7.92E-05	1700	0.333333	0.024415	LSM6
21008.86	5.831625	-7068	7.83E-05	1645	0.333333	0.021362	CDC20
10125.03	6.351873	-7068	7.60E-05	1118	0.333333	0.015693	IL4R
18742.22	5.268632	-7068	7.81E-05	1665	0.333333	0.021317	PSMD14
5908.304	8.67017	-7068	7.40E-05	685	0.333333	0.010014	ATXN2
5519.374	6.902491	-7068	7.40E-05	754	0.333333	0.009322	CD3G
12158.42	5.746429	-7068	7.68E-05	1311	0.333333	0.018387	SSB
7817.196	7.763565	-7068	7.50E-05	855	0.333333	0.012042	EDC4
10710.25	8.50387	-7068	7.54E-05	913	0.333333	0.012155	SCAP
12093.45	5.385379	-7068	7.64E-05	1321	0.333333	0.01637	FLNA
8007.942	7.332769	-7068	7.51E-05	920	0.333333	0.013432	NVL
23318.7	7.68672	-7068	7.84E-05	1499	0.333333	0.021716	PTPRU
4208.725	8.235913	-7068	7.36E-05	601	0.333333	0.008801	SPG11
8515.873	6.821102	-7068	7.54E-05	993	0.333333	0.014783	PRKACB
8995.422	6.125499	-7068	7.56E-05	1067	0.333333	0.01525	CDK8
14186.08	7.808505	-7068	7.63E-05	1145	0.333333	0.015758	TRIP10
13173.45	8.970264	-7068	7.57E-05	1000	0.333333	0.013498	MRAS
9452.837	8.532694	-7068	7.50E-05	870	0.333333	0.012335	KCNJ10
11510.69	5.561146	-7068	7.61E-05	1277	0.333333	0.016106	BRCA1
12838.28	8.818423	-7068	7.56E-05	973	0.333333	0.012671	MPP3
10361.76	9.224443	-7068	7.49E-05	833	0.333333	0.010087	PDE6G
12822.4	5.979658	-7068	7.72E-05	1344	0.333333	0.019746	GTF2F2
6375.939	8.181088	-7068	7.41E-05	718	0.333333	0.009346	DHX16
6248.789	7.428798	-7068	7.39E-05	740	0.333333	0.008955	CD79A
8570.534	7.590176	-7068	7.50E-05	886	0.333333	0.011743	SART1
7456.636	8.660652	-7068	7.44E-05	759	0.333333	0.01029	TSHB
12031.5	7.016917	-7068	7.65E-05	1152	0.333333	0.017338	MYCBP2
32573.39	5.69542	-7068	8.11E-05	2161	0.333333	0.029566	SRPK1
6068.781	6.535507	-7068	7.48E-05	853	0.333333	0.012815	RNF11

7236.576	7.858402	-7068	7.45E-05	801	0.333333	0.010122	ACADVL
9708.806	6.460237	-7068	7.59E-05	1083	0.333333	0.016134	CLASP2
18762.02	6.31314	-7068	7.86E-05	1567	0.333333	0.023927	LBR
31593.47	21.12715	-7068	7.50E-05	928	0.333333	0.011717	ARRB2
10058.94	6.701401	-7068	7.58E-05	1072	0.333333	0.014684	TNFRSF1B
9222.323	7.658299	-7068	7.51E-05	913	0.333333	0.012012	BAG6
7447.505	7.073704	-7068	7.50E-05	902	0.333333	0.012613	GYS1
15872.33	7.526193	-7068	7.63E-05	1182	0.333333	0.015215	TUBG1
12737.3	6.715794	-7068	7.62E-05	1179	0.333333	0.015187	IRAK1
12580.65	6.1756	-7068	7.69E-05	1268	0.333333	0.019332	SMNDC1
12609.92	8.406627	-7068	7.56E-05	1014	0.333333	0.013537	ACIN1
17426.82	6.285387	-7068	7.80E-05	1485	0.333333	0.021723	CDC123
8959.424	5.005997	-7068	7.58E-05	1191	0.333333	0.014783	MCM5
13602.45	7.756236	-7068	7.62E-05	1122	0.333333	0.015519	ENO2
7651.799	6.222627	-7068	7.53E-05	994	0.333333	0.014471	CDKN1B
14772.03	7.505517	-7068	7.65E-05	1204	0.333333	0.016714	FOXA1
11164.2	7.146995	-7068	7.57E-05	1055	0.333333	0.013504	ARAF
6820.018	5.840057	-7068	7.51E-05	980	0.333333	0.013976	CREB1
16896.26	6.488819	-7068	7.76E-05	1414	0.333333	0.019561	CD34
10797.5	6.161852	-7068	7.61E-05	1173	0.333333	0.016129	IRF8
12777.8	6.744803	-7068	7.65E-05	1209	0.333333	0.017109	SPTAN1
5845.151	9.464719	-7068	7.38E-05	639	0.333333	0.008215	SH2B2
13279.14	7.448223	-7068	7.63E-05	1142	0.25	0.016614	TSPAN7
10526.53	9.999657	-7068	7.45E-05	772	0.333333	0.009407	WNT1
5690.721	8.493031	-7068	7.38E-05	668	0.333333	0.008723	SST
16130.72	8.152762	-7068	7.69E-05	1204	0.333333	0.018196	SCRN1
15952.01	8.706747	-7068	7.58E-05	1045	0.333333	0.013049	HGS
5401.566	7.113258	-7068	7.38E-05	718	0.333333	0.008919	MYL2
12134.93	5.83841	-7068	7.67E-05	1312	0.333333	0.018349	YWHAE
18533.45	6.51718	-7068	7.84E-05	1524	0.333333	0.022988	SLC16A1
11805.82	6.491719	-7068	7.62E-05	1174	0.333333	0.015068	CSNK2B
8689.214	5.97441	-7068	7.53E-05	1042	0.333333	0.013029	ARHGDIB
11045.45	6.911245	-7068	7.60E-05	1094	0.333333	0.014743	UBA1
10778.65	7.223263	-7068	7.57E-05	1027	0.333333	0.013579	ATP4A
9358.516	5.541349	-7068	7.60E-05	1180	0.333333	0.016472	GSK3B
14318.96	7.099314	-7068	7.70E-05	1258	0.333333	0.018986	SOX2
8475.397	8.771885	-7068	7.46E-05	801	0.333333	0.010469	RING1
4397.435	7.839547	-7068	7.37E-05	647	0.333333	0.009433	TERF1
5991.547	7.275561	-7068	7.45E-05	781	0.333333	0.011043	BAP1
3375.925	7.385447	-7068	7.34E-05	577	0.333333	0.008891	PNISR
7646.281	7.100963	-7068	7.52E-05	914	0.333333	0.013853	CSRNP2

13075.34	5.634452	-7068	7.70E-05	1373	0.333333	0.019216	MAP3K5
19428.32	7.612376	-7068	7.75E-05	1373	0.333333	0.019254	GSK3A
6223.365	6.530826	-7068	7.45E-05	847	0.333333	0.011653	MDC1
14995.02	4.77875	-7068	7.68E-05	1578	0.333333	0.017628	TP53
10211.05	7.99269	-7068	7.55E-05	967	0.333333	0.014386	CPOX
7086.796	6.279378	-7068	7.51E-05	941	0.333333	0.013788	ATF1
8490.634	7.287381	-7068	7.49E-05	890	0.333333	0.01144	CORO1A
6756.239	6.036111	-7068	7.48E-05	920	0.333333	0.011921	ITGAL
8025.531	11.42063	-7068	7.36E-05	603	0.333333	0.007107	CLPS
6889.171	8.601121	-7068	7.41E-05	723	0.333333	0.009257	CSRP3
4348.181	6.882508	-7068	7.38E-05	697	0.333333	0.009654	BMX
19125.36	6.545151	-7068	7.82E-05	1530	0.333333	0.022339	MAPK7
14925.99	6.542997	-7068	7.72E-05	1338	0.333333	0.019575	ATM
9784.033	5.87047	-7068	7.63E-05	1188	0.333333	0.017609	YWHAQ
15592.13	7.615888	-7068	7.65E-05	1217	0.333333	0.016474	PRKCZ
14872.08	6.147563	-7068	7.66E-05	1302	0.333333	0.015996	SLC2A2
7378.845	7.940991	-7068	7.45E-05	807	0.333333	0.011348	DCX
14663.37	6.165283	-7068	7.71E-05	1356	0.333333	0.018576	HLA-DRA
12159.09	7.317232	-7068	7.60E-05	1095	0.333333	0.01545	CDH17
7827.765	6.299324	-7068	7.46E-05	922	0.333333	0.010879	PTPN6
9094.928	7.83788	-7068	7.54E-05	942	0.333333	0.013978	CHD2
8838.529	6.042107	-7068	7.54E-05	1042	0.333333	0.0136	IL10RA
8005.547	6.194927	-7068	7.47E-05	961	0.333333	0.01119	RPS19
6663.761	6.832591	-7068	7.49E-05	845	0.333333	0.011497	CLPP
12462.98	8.422921	-7068	7.56E-05	1026	0.333333	0.014099	CDK9
7090.582	8.380783	-7068	7.46E-05	781	0.333333	0.011509	IQCB1
9625.691	7.049087	-7068	7.58E-05	1040	0.333333	0.014894	HMGN2
17964.47	6.623527	-7068	7.80E-05	1462	0.333333	0.021791	GTF2E1
7808.063	6.794456	-7068	7.53E-05	949	0.333333	0.014168	TBCA
10670.8	9.028444	-7068	7.52E-05	882	0.333333	0.011535	PLOD3
20919.84	6.774286	-7068	7.81E-05	1525	0.333333	0.021688	GAP43
7023.218	10.46606	-7068	7.36E-05	626	0.333333	0.00758	LTA
8901.787	5.868336	-7068	7.54E-05	1060	0.333333	0.013248	PRPF8
11988.77	7.322079	-7068	7.63E-05	1129	0.333333	0.016759	COX10
9798.789	7.738484	-7068	7.56E-05	974	0.333333	0.014394	TOR1B
13671.02	5.912037	-7068	7.70E-05	1356	0.333333	0.018133	TNFRSF1A
18308.99	6.221849	-7068	7.75E-05	1493	0.333333	0.019941	SRSF11
11364.14	6.856443	-7068	7.61E-05	1129	0.333333	0.016709	RPS6KA5
17179.15	6.025134	-7068	7.83E-05	1556	0.333333	0.023445	MAPK6
12664.99	9.46379	-7068	7.54E-05	924	0.333333	0.012065	IPO13
13660.44	5.715154	-7068	7.72E-05	1381	0.333333	0.019822	SMC3

6057.185	7.657147	-7068	7.42E-05	747	0.333333	0.010276	CCK
30730.13	6.59594	-7068	8.00E-05	1894	0.333333	0.026697	APC
8933.839	7.526609	-7068	7.51E-05	926	0.333333	0.012974	SLC4A1
17966.69	5.790383	-7068	7.81E-05	1563	0.333333	0.021509	NCBP1
12006.73	7.127534	-7068	7.56E-05	1083	0.333333	0.01432	PLP1
12801.78	7.605657	-7068	7.60E-05	1088	0.333333	0.014721	FMO4
10055.28	6.149477	-7068	7.61E-05	1143	0.333333	0.016303	HADHB
22231.11	7.583178	-7068	7.79E-05	1455	0.333333	0.020862	ISL1
11801.09	8.452974	-7068	7.55E-05	976	0.333333	0.013417	KRT9
12220.53	8.451643	-7068	7.57E-05	1008	0.333333	0.014046	TAB1
9814.191	6.100921	-7068	7.63E-05	1159	0.333333	0.01771	PPP1R12A
28272.71	6.88317	-7068	7.99E-05	1787	0.333333	0.026145	GATA3
25303.04	6.43662	-7068	7.91E-05	1748	0.333333	0.023436	SOD1
9894.057	6.793839	-7068	7.60E-05	1068	0.333333	0.016545	ATP2C1
5633.917	6.767028	-7068	7.42E-05	792	0.333333	0.011015	USP7
8362.013	6.387327	-7068	7.53E-05	1016	0.333333	0.013929	CRK
10534.22	4.852237	-7068	7.60E-05	1322	0.333333	0.015995	EP300
7319.464	7.710683	-7068	7.45E-05	826	0.333333	0.011368	RBM5
25100.3	7.723673	-7068	7.85E-05	1525	0.333333	0.021609	SOD3
8568.532	7.443594	-7068	7.48E-05	894	0.333333	0.011636	CSF2RB
11682.06	6.95115	-7068	7.60E-05	1098	0.333333	0.015109	CYTIP
9111.687	6.952181	-7068	7.57E-05	1018	0.333333	0.015388	NEDD4
13997.81	6.151292	-7068	7.71E-05	1335	0.333333	0.018477	CTPS1
11086.49	9.493655	-7068	7.51E-05	879	0.333333	0.012041	DRAP1
8666.355	7.98147	-7068	7.47E-05	851	0.333333	0.010895	PRKCG
19653.86	6.739628	-7068	7.82E-05	1503	0.333333	0.022191	EPS8
8237.915	7.095465	-7068	7.52E-05	928	0.333333	0.012552	GRN
6757.292	9.520662	-7068	7.40E-05	684	0.333333	0.009685	CDKN2D
12190.03	5.60603	-7068	7.72E-05	1345	0.333333	0.019298	CCT5
14535.12	5.529044	-7068	7.72E-05	1461	0.333333	0.019782	PSMA3
5831.569	5.98176	-7068	7.48E-05	868	0.333333	0.012699	DDX1
13047.33	5.936028	-7068	7.72E-05	1351	0.333333	0.019961	NRAS
9529.233	6.680787	-7068	7.58E-05	1075	0.333333	0.015987	SMURF1
11305.92	8.749129	-7068	7.53E-05	950	0.333333	0.013365	MED24
11260.31	10.68593	-7068	7.45E-05	774	0.333333	0.009658	CTF1
11063.42	6.083419	-7068	7.65E-05	1224	0.333333	0.017718	PPP2CB
15871.8	7.373807	-7068	7.71E-05	1254	0.333333	0.016912	CAPNS1
15334.11	6.430479	-7068	7.69E-05	1330	0.333333	0.017806	MGP
7806.19	5.430011	-7068	7.57E-05	1092	0.333333	0.015129	GART
16955.25	7.883473	-7068	7.69E-05	1240	0.333333	0.016656	FOSL1
9701.9	7.237654	-7068	7.55E-05	996	0.333333	0.014103	ACADM

20288.59	6.888378	-7068	7.83E-05	1511	0.333333	0.022499	RIT2
6899.49	7.333946	-7068	7.46E-05	830	0.333333	0.011626	GABARAPL2
4963.272	6.735253	-7068	7.38E-05	711	0.333333	0.008745	GZMK
21330.55	6.224291	-7068	7.82E-05	1596	0.333333	0.022392	SYT1
20938.86	7.270937	-7068	7.79E-05	1446	0.333333	0.020086	HCLS1
10463.91	5.894061	-7068	7.63E-05	1206	0.333333	0.017039	POLA1
7219.776	8.092439	-7068	7.44E-05	789	0.333333	0.010317	ICAM3
9712.368	6.811635	-7068	7.57E-05	1032	0.333333	0.014727	GABRA1
15955.45	6.546136	-7068	7.72E-05	1347	0.333333	0.017608	SNRPA
5264.422	8.463221	-7068	7.38E-05	660	0.333333	0.009262	NDP
3644.569	9.366326	-7068	7.32E-05	523	0.333333	0.008045	HIST1H2BK
25631	6.502169	-7068	7.97E-05	1768	0.333333	0.026254	PIK3C3
15522.61	7.044166	-7068	7.69E-05	1280	0.333333	0.018088	PCSK1
13097.55	10.14461	-7068	7.51E-05	887	0.333333	0.011991	MMP17
9360.66	6.274012	-7068	7.55E-05	1078	0.333333	0.014413	HSP90AB1
12177.66	5.497235	-7068	7.69E-05	1344	0.333333	0.018779	CSE1L
17231.03	7.499015	-7068	7.69E-05	1284	0.333333	0.016561	ASGR1
15073.62	6.079462	-7068	7.75E-05	1420	0.333333	0.020109	PARP1
14147.23	5.828973	-7068	7.75E-05	1409	0.333333	0.020633	PTGES3
13575.68	6.7466	-7068	7.66E-05	1236	0.333333	0.017449	AMPH
15429.51	8.033757	-7068	7.65E-05	1161	0.333333	0.016677	ATP12A
11978.35	6.691143	-7068	7.64E-05	1174	0.333333	0.017894	SIAH1
6915.948	5.065471	-7068	7.54E-05	1057	0.333333	0.014429	TOPBP1
8948.978	9.003704	-7068	7.49E-05	834	0.333333	0.012412	MUTYH
11420.55	5.011061	-7068	7.67E-05	1381	0.333333	0.018104	LYN
16818.69	6.318575	-7068	7.77E-05	1452	0.333333	0.020889	SCP2
12794.2	6.367679	-7068	7.66E-05	1266	0.333333	0.017719	PTK2B
8731.802	8.639006	-7068	7.46E-05	824	0.333333	0.010827	S100A1
5845.7	8.480735	-7068	7.41E-05	701	0.333333	0.009693	NOS2
12076.91	5.782207	-7068	7.67E-05	1309	0.333333	0.018508	NR3C1
8149.287	8.392081	-7068	7.44E-05	805	0.333333	0.010167	MYH7
12884.51	8.466215	-7068	7.56E-05	1001	0.333333	0.012656	PIGR
15283.77	6.914903	-7068	7.70E-05	1285	0.333333	0.017523	TOMM40
6589.077	7.013496	-7068	7.46E-05	841	0.333333	0.011885	DYNLL1
10629.25	5.646166	-7068	7.62E-05	1236	0.333333	0.016898	CASP3
6000.396	7.521879	-7068	7.40E-05	753	0.333333	0.009373	WAS
11172.46	6.522794	-7068	7.61E-05	1164	0.333333	0.016735	FOXO1
16420.27	4.910729	-7068	7.79E-05	1683	0.333333	0.021699	MAPK1
4954.363	8.123845	-7068	7.39E-05	663	0.333333	0.009779	METTL18
5737.072	7.57297	-7068	7.42E-05	732	0.333333	0.00986	AES
24594.14	6.01753	-7068	7.94E-05	1806	0.333333	0.025621	AHR

7008.704	7.572915	-7068	7.45E-05	802	0.333333	0.010491	GPX2
18635.24	7.058926	-7068	7.77E-05	1401	0.333333	0.01972	CSF3R
9596.141	7.48012	-7068	7.54E-05	981	0.333333	0.013922	QDPR
13292.45	7.033541	-7068	7.61E-05	1169	0.333333	0.015026	SNRNP70
5468.094	8.500374	-7068	7.37E-05	645	0.333333	0.008319	CD19
34756.39	7.384477	-7068	8.05E-05	1873	0.333333	0.02711	PRL
12824.75	6.2073	-7068	7.66E-05	1261	0.333333	0.016841	HCK
14545.3	8.433792	-7068	7.56E-05	1048	0.333333	0.013031	CYP11A1
7368.891	6.737039	-7068	7.47E-05	898	0.333333	0.01149	PRPF4
9632.952	9.658973	-7068	7.47E-05	806	0.333333	0.010309	CD70
21790.71	6.603518	-7068	7.86E-05	1592	0.333333	0.022558	FMOD
12011.43	7.010879	-7068	7.66E-05	1161	0.333333	0.018133	ZFC3H1
15795.94	7.473152	-7068	7.71E-05	1270	0.333333	0.019416	SORL1
1999.768	6.865165	-7068	7.28E-05	476	0.333333	0.007241	PEX1
14012.11	6.921113	-7068	7.69E-05	1249	0.333333	0.018607	STT3A
6544.739	6.767401	-7068	7.43E-05	837	0.333333	0.010306	SPI1
11304.04	6.061338	-7068	7.67E-05	1241	0.333333	0.018814	SMARCA5
33526.95	6.919294	-7068	8.07E-05	1946	0.333333	0.028739	PARK2
19577.01	5.502003	-7068	7.89E-05	1716	0.333333	0.024151	CCT2
14463.43	7.544392	-7068	7.63E-05	1150	0.333333	0.01509	SLC17A3
10118.39	5.853816	-7068	7.60E-05	1165	0.333333	0.015667	DNMT1
13134.34	5.558652	-7068	7.71E-05	1360	0.333333	0.018264	ILF2
11247.93	5.886114	-7068	7.66E-05	1243	0.333333	0.017767	PTPRC
8403.797	8.125258	-7068	7.49E-05	860	0.333333	0.011756	XRCC1
8640.651	7.128451	-7068	7.51E-05	945	0.333333	0.012442	GSTZ1
6165.43	7.114984	-7068	7.43E-05	783	0.333333	0.010378	NXF1
5638.13	7.27393	-7068	7.43E-05	772	0.333333	0.011544	AKAP9
8186.295	8.746896	-7068	7.45E-05	793	0.333333	0.011093	DRD1
12721.72	6.26416	-7068	7.65E-05	1269	0.333333	0.01738	PAK1
11524.43	10.33969	-7068	7.47E-05	804	0.333333	0.010292	SLC5A5
6234.581	7.465927	-7068	7.43E-05	761	0.333333	0.010161	LSP1
5320.334	6.518063	-7068	7.45E-05	794	0.333333	0.011437	SRP19
6802.648	6.294861	-7068	7.52E-05	944	0.333333	0.014022	ADCY7
12405.54	6.449036	-7068	7.64E-05	1211	0.333333	0.015928	EIF6
8497.986	5.949441	-7068	7.60E-05	1091	0.333333	0.016612	TRIP12
5618.572	8.077999	-7068	7.41E-05	695	0.333333	0.009229	AAMP
18602.98	7.996539	-7068	7.71E-05	1295	0.333333	0.018671	CAMK4
14320.7	9.211096	-7068	7.59E-05	1018	0.333333	0.014158	COX6A2
18371.82	7.164472	-7068	7.74E-05	1388	0.333333	0.018706	MAP3K3
7326.946	9.436787	-7068	7.42E-05	707	0.333333	0.009466	PALM
23995.24	5.498298	-7068	7.94E-05	1916	0.333333	0.02545	MAPK14

7224.028	6.547086	-7068	7.54E-05	960	0.333333	0.015322	RCOR1
7830.784	6.921969	-7068	7.53E-05	960	0.333333	0.013653	DLST
22278.5	6.36129	-7068	7.89E-05	1665	0.333333	0.024264	TGFBR3
5155.529	10.72647	-7068	7.32E-05	532	0.333333	0.007081	CSN2
11630.17	8.020948	-7068	7.48E-05	913	0.25	0.011675	ZNF140
11094.5	7.083072	-7068	7.62E-05	1112	0.333333	0.016642	BTG1
9081.378	6.212812	-7068	7.60E-05	1087	0.333333	0.016315	PUM1
8428.617	8.016721	-7068	7.50E-05	872	0.333333	0.012861	RRAGA
15331.42	8.951285	-7068	7.60E-05	1089	0.333333	0.01568	TFAP4
5792.515	7.911508	-7068	7.42E-05	711	0.333333	0.009347	GPS2
5944.577	5.498296	-7068	7.49E-05	949	0.333333	0.013334	E2F3
6924.889	7.734775	-7068	7.47E-05	815	0.333333	0.012194	CGRRF1
7185.186	5.935501	-7068	7.54E-05	1000	0.333333	0.01517	TRAM1
24732.14	6.392598	-7068	7.94E-05	1751	0.333333	0.02538	PRKCQ
18772.07	7.288313	-7068	7.75E-05	1410	0.333333	0.019633	IKBKE
8634.079	5.840195	-7068	7.54E-05	1072	0.333333	0.013865	BTK
5487.515	7.896486	-7068	7.39E-05	702	0.333333	0.009245	APBB1
6596.907	6.195312	-7068	7.46E-05	886	0.333333	0.011679	TBP
6956.768	8.594554	-7068	7.42E-05	728	0.333333	0.009432	REG1B
25242.12	5.946124	-7068	8.01E-05	1889	0.333333	0.027723	TOP1
17330.23	6.240491	-7068	7.74E-05	1435	0.333333	0.01838	CPB2
10459.07	6.955684	-7068	7.61E-05	1092	0.333333	0.016429	PNRC1
7403.827	6.546854	-7068	7.49E-05	912	0.333333	0.012565	MAK16
6763.085	7.436014	-7068	7.44E-05	792	0.333333	0.010542	ABCC8
7374.323	6.534303	-7068	7.55E-05	954	0.333333	0.015081	VPS26A
10974.18	7.005697	-7068	7.60E-05	1087	0.333333	0.015844	AQP4
10855.74	6.555422	-7068	7.62E-05	1129	0.333333	0.016773	EYA1
20241.33	6.573564	-7068	7.86E-05	1565	0.333333	0.023349	SATB1
6480.171	8.141262	-7068	7.42E-05	733	0.333333	0.009824	CD40LG
16630.99	5.868693	-7068	7.83E-05	1545	0.333333	0.023126	ARPC5
6846.522	7.539333	-7068	7.43E-05	795	0.333333	0.010598	FLNC
9745.138	7.501565	-7068	7.52E-05	964	0.333333	0.013384	MPO
36607.28	6.411216	-7068	8.14E-05	2099	0.333333	0.030526	RDX
10177.92	11.75992	-7068	7.38E-05	646	0.333333	0.007242	ARR3
12084.72	6.689633	-7068	7.61E-05	1161	0.333333	0.015579	C5
6997.392	7.993909	-7068	7.45E-05	790	0.333333	0.011164	CELSR2
9491.04	8.292389	-7068	7.52E-05	904	0.333333	0.013171	ABCA3
6079.23	6.480422	-7068	7.47E-05	845	0.333333	0.011995	TRIM22
9632.227	8.951784	-7068	7.49E-05	847	0.333333	0.011126	FUT1
7894.328	7.043622	-7068	7.49E-05	886	0.333333	0.011724	WDR46
24315.05	6.998062	-7068	7.89E-05	1640	0.333333	0.023257	GSTP1

8270.936	7.180346	-7068	7.51E-05	934	0.333333	0.013593	RAP2A
6186.906	6.186595	-7068	7.50E-05	906	0.333333	0.01371	CNBP
8011.584	6.518575	-7068	7.53E-05	975	0.333333	0.013386	FGR
11049.86	6.82909	-7068	7.61E-05	1102	0.333333	0.015974	NUP155
13211.58	6.93175	-7068	7.66E-05	1209	0.333333	0.017214	CD38
7006.687	6.716098	-7068	7.49E-05	909	0.333333	0.013128	PIK3CG
5720.931	5.488881	-7068	7.43E-05	902	0.333333	0.011221	LCK
12430.88	5.198016	-7068	7.68E-05	1382	0.333333	0.018693	KPNB1
14351.59	6.230784	-7068	7.71E-05	1359	0.333333	0.018981	GAPDH
10058.51	6.068522	-7068	7.62E-05	1167	0.333333	0.01754	ZEB1
8283.353	6.066311	-7068	7.57E-05	1057	0.333333	0.015876	RAP1A
11953.88	7.077744	-7068	7.61E-05	1148	0.333333	0.015919	TAT
13134.56	5.976698	-7068	7.67E-05	1291	0.333333	0.016747	PSMC4
14939.34	6.13904	-7068	7.71E-05	1382	0.333333	0.018876	BIRC3
11851.24	8.102938	-7068	7.58E-05	1027	0.333333	0.015055	FICD
10318.19	5.518851	-7068	7.63E-05	1244	0.333333	0.017187	RHOA
10045.1	7.01275	-7068	7.56E-05	1031	0.333333	0.013985	DHX38
9624.95	6.670449	-7068	7.58E-05	1069	0.333333	0.015866	KMT2A
18998.75	7.372194	-7068	7.72E-05	1358	0.333333	0.018629	SRPK2
10305.71	7.443061	-7068	7.56E-05	1017	0.333333	0.014385	FURIN
7431.336	9.124778	-7068	7.43E-05	738	0.333333	0.009933	POMC
28364.17	5.332935	-7068	8.03E-05	2103	0.333333	0.027915	PIK3R1
6445.631	6.236333	-7068	7.48E-05	886	0.333333	0.012537	CBX1
16728.96	6.49814	-7068	7.77E-05	1421	0.333333	0.020761	F3
7674.867	6.220975	-7068	7.53E-05	973	0.333333	0.014133	SUCLA2
4530.308	7.055446	-7068	7.37E-05	670	0.333333	0.008623	TNNT3
2475.86	5.499593	-7068	7.30E-05	589	0.333333	0.007186	RPS21
10172.26	8.522008	-7068	7.51E-05	891	0.333333	0.012044	HSD11B2
9423.941	6.304899	-7068	7.56E-05	1090	0.333333	0.014821	PLCG2
14921.91	6.414896	-7068	7.74E-05	1357	0.333333	0.019901	SEC61G
6166.468	8.562446	-7068	7.42E-05	711	0.333333	0.010265	CLASRP
11176.17	7.779005	-7068	7.53E-05	995	0.333333	0.013269	STAR
10992.79	8.106034	-7068	7.56E-05	988	0.333333	0.01421	CALCOCO1
10178.12	7.755005	-7068	7.54E-05	978	0.333333	0.013274	MB
10137.72	6.761955	-7068	7.61E-05	1103	0.333333	0.016946	TP53BP2
5899.053	7.940321	-7068	7.41E-05	709	0.333333	0.010198	FANCL
13050.64	10.21039	-7068	7.49E-05	858	0.333333	0.011036	MYOG
20733.9	6.794736	-7068	7.82E-05	1534	0.333333	0.021227	ITGA3
16047.86	5.977581	-7068	7.77E-05	1479	0.333333	0.020559	AHCY
16276.24	6.715483	-7068	7.70E-05	1323	0.333333	0.017556	MTTP
14109.15	6.928318	-7068	7.67E-05	1243	0.333333	0.017464	CD8A

4620.682	6.372993	-7068	7.42E-05	760	0.333333	0.011235	ROCK1
28191.92	6.91307	-7068	7.97E-05	1772	0.333333	0.025598	KDM1A
21000.28	10.22529	-7068	7.62E-05	1067	0.333333	0.012686	CRYBB3
14736.76	5.723751	-7068	7.76E-05	1462	0.333333	0.020833	EIF4E
6774.268	8.580607	-7068	7.42E-05	733	0.333333	0.009037	RPL41
8109.455	4.681017	-7068	7.53E-05	1182	0.333333	0.014264	CREBBP
6271.662	8.741933	-7068	7.40E-05	676	0.333333	0.00821	SKIV2L
20315.52	6.063661	-7068	7.86E-05	1656	0.333333	0.023402	DUT
5970.922	7.392189	-7068	7.42E-05	753	0.333333	0.010045	GFAP
16447.1	6.162077	-7068	7.79E-05	1481	0.333333	0.021921	PRNP
7911.031	8.361426	-7068	7.46E-05	805	0.333333	0.011759	GLRA1
7331.752	7.047981	-7068	7.48E-05	888	0.333333	0.012356	STK11
13969.26	6.280012	-7068	7.76E-05	1357	0.333333	0.021243	DYNLT3
4985.861	7.054324	-7068	7.43E-05	749	0.333333	0.011677	SETD2
19617.62	5.935621	-7068	7.90E-05	1660	0.333333	0.0251	UBE3A
9255.857	6.638712	-7068	7.56E-05	1040	0.333333	0.014909	GPN1
5389.536	7.357446	-7068	7.43E-05	745	0.333333	0.011089	SLC26A2
8605.353	7.449894	-7068	7.49E-05	910	0.333333	0.011608	GTF2F1
8949.426	7.067167	-7068	7.53E-05	980	0.333333	0.013656	ATP6V1B2
6922.373	7.364057	-7068	7.40E-05	748	0.333333	0.009627	ZNF43
15947.2	6.538525	-7068	7.76E-05	1384	0.333333	0.020949	PKN2
12720.02	6.543022	-7068	7.67E-05	1249	0.333333	0.018398	UMPS
9560.694	7.209551	-7068	7.54E-05	965	0.333333	0.01287	POLD2
7286.521	6.394297	-7068	7.51E-05	942	0.333333	0.013463	BLNK
17224.94	6.874235	-7068	7.76E-05	1393	0.333333	0.020426	MME
9465.583	6.349849	-7068	7.56E-05	1070	0.333333	0.014505	IL7R
19199.99	6.109761	-7068	7.81E-05	1583	0.333333	0.021297	CDKN1A
4630.351	4.952555	-7068	7.41E-05	848	0.333333	0.010437	RPL10A
16837.92	8.058019	-7068	7.69E-05	1222	0.333333	0.017593	VPS52
18590.5	5.877185	-7068	7.85E-05	1606	0.333333	0.022883	PSMA5
10213.16	4.49896	-7068	7.62E-05	1350	0.333333	0.016304	PRIM1
11659.96	6.484374	-7068	7.65E-05	1187	0.333333	0.016807	AATF
13613.88	6.755691	-7068	7.69E-05	1255	0.333333	0.01859	TXNL1
13663	6.751508	-7068	7.68E-05	1258	0.333333	0.017872	ICAM2
9488.23	7.238496	-7068	7.54E-05	997	0.333333	0.013631	CDC25B
11208.86	6.506778	-7068	7.64E-05	1178	0.333333	0.018019	SNAP23
13772.98	5.142542	-7068	7.74E-05	1489	0.333333	0.020343	HNRNPR
15607.05	6.636002	-7068	7.76E-05	1376	0.333333	0.021018	RNF4
3332.775	5.871916	-7068	7.34E-05	650	0.333333	0.008431	RPS27A
7735.181	7.001003	-7068	7.50E-05	905	0.333333	0.012389	CD69
15218.4	5.397899	-7068	7.75E-05	1527	0.333333	0.019873	NFKB1

11283.08	6.140392	-7068	7.67E-05	1232	0.333333	0.018371	CLIP1
10524.01	6.407182	-7068	7.61E-05	1142	0.333333	0.016579	PIK3CB
14497.15	6.417289	-7068	7.74E-05	1347	0.333333	0.020308	KIF5B
5702.256	7.547257	-7068	7.41E-05	718	0.333333	0.00929	PKN1
10574.06	7.851315	-7068	7.55E-05	963	0.333333	0.013051	FSHB
13563.68	6.536774	-7068	7.69E-05	1275	0.333333	0.018726	PPP2R3A
5533.35	7.160287	-7068	7.43E-05	769	0.333333	0.010484	PSMB10
10557.08	10.14154	-7068	7.46E-05	796	0.333333	0.010258	MAPK8IP2
9450.817	8.698847	-7068	7.48E-05	851	0.333333	0.011332	SNTA1
9098.193	8.30535	-7068	7.50E-05	869	0.333333	0.012197	GSTM5
11253.69	6.641555	-7068	7.65E-05	1154	0.333333	0.017655	C1D
16699.45	6.60376	-7068	7.76E-05	1403	0.333333	0.020176	LDB2
9205.685	7.672452	-7068	7.51E-05	894	0.333333	0.012811	DRG1
11776.9	10.27693	-7068	7.47E-05	804	0.333333	0.009978	OPN1SW
11325.93	7.418152	-7068	7.55E-05	1039	0.333333	0.013296	CDK5
33902.03	6.244874	-7068	8.07E-05	2025	0.333333	0.028111	UQCRFS1
7950.238	8.652612	-7068	7.47E-05	793	0.333333	0.010781	ARFIP2
7646.482	7.153197	-7068	7.49E-05	897	0.333333	0.012385	DECR1
6077.662	6.344521	-7068	7.46E-05	874	0.333333	0.012443	GAB1
26374.47	7.059712	-7068	7.88E-05	1649	0.333333	0.023012	GLI3
17943.1	6.108965	-7068	7.82E-05	1548	0.333333	0.02219	CCT4
14330.78	5.708887	-7068	7.76E-05	1459	0.333333	0.021792	PTPN12
7387.777	6.318638	-7068	7.51E-05	959	0.333333	0.013219	GTF2B
17205.13	7.028345	-7068	7.77E-05	1395	0.333333	0.020359	FTL
8678.391	6.976709	-7068	7.55E-05	976	0.333333	0.014376	AUH
6473.108	8.861209	-7068	7.38E-05	682	0.333333	0.008576	RXRB
6336.949	7.079746	-7068	7.47E-05	828	0.333333	0.012519	PPIG
14975.78	5.845349	-7068	7.71E-05	1422	0.333333	0.019196	SRSF5
5341.24	6.588316	-7068	7.46E-05	799	0.333333	0.012536	VAMP7
8427.679	6.267637	-7068	7.56E-05	1055	0.333333	0.015646	PTEN
12794.13	7.348091	-7068	7.61E-05	1118	0.333333	0.015366	FOXF2
15025.05	5.657305	-7068	7.75E-05	1487	0.333333	0.020849	PTK2
12158.7	6.893479	-7068	7.64E-05	1181	0.333333	0.017687	TXNIP
14002.23	7.099563	-7068	7.69E-05	1238	0.333333	0.018859	DCTN6
11820.93	5.882993	-7068	7.71E-05	1308	0.333333	0.020239	CAPRIN1
9335.966	7.240411	-7068	7.54E-05	983	0.333333	0.013639	KAT5
8878.829	5.520155	-7068	7.60E-05	1164	0.333333	0.01682	SRRM1
4182.808	8.466681	-7068	7.35E-05	594	0.333333	0.008525	ZC3H13
9673.56	8.175575	-7068	7.49E-05	878	0.333333	0.012309	TSC2
16891.6	7.948961	-7068	7.69E-05	1242	0.333333	0.018134	ERCC6
14698.23	7.772911	-7068	7.63E-05	1146	0.333333	0.015224	EGF

7302.414	5.045714	-7068	7.55E-05	1099	0.333333	0.014658	HNRNPK
15850.69	6.788574	-7068	7.72E-05	1346	0.333333	0.019604	GRIN2B
8407.105	9.485593	-7068	7.43E-05	756	0.333333	0.010333	HSD17B3
15851.8	7.623569	-7068	7.65E-05	1194	0.333333	0.016351	ISLR
4597.638	7.544693	-7068	7.39E-05	673	0.333333	0.0101	TBPL1
4236.695	6.488774	-7068	7.39E-05	701	0.333333	0.009369	CD52
18855.49	6.338766	-7068	7.83E-05	1527	0.333333	0.021721	HNRNPAB
9222.298	7.343008	-7068	7.57E-05	995	0.333333	0.015599	HMGN3
8317.722	6.755168	-7068	7.52E-05	960	0.333333	0.013542	GSTA4
14765.9	6.754592	-7068	7.68E-05	1283	0.333333	0.017848	ABCB1
33451.14	6.813487	-7068	8.03E-05	1912	0.333333	0.027479	TCF4
11772.13	7.364508	-7068	7.62E-05	1114	0.333333	0.015931	DDB2
10556.91	9.85629	-7068	7.47E-05	796	0.333333	0.010478	SLC9A3
17179.91	10.10267	-7068	7.58E-05	1033	0.333333	0.013463	GPER1
7268.134	7.386588	-7068	7.46E-05	828	0.333333	0.010622	NR0B2
8999.734	6.588303	-7068	7.58E-05	1043	0.333333	0.015867	CCNG2
14488.5	6.473204	-7068	7.73E-05	1331	0.333333	0.0203	ATP2B1
4836.277	7.520293	-7068	7.38E-05	674	0.333333	0.00895	ALAS2
6541.203	8.261526	-7068	7.43E-05	738	0.333333	0.010011	CDIPT
6841.466	8.031749	-7068	7.44E-05	771	0.333333	0.010488	ALDH3B2
12537.39	7.083621	-7068	7.65E-05	1168	0.333333	0.017582	SSBP2
5838.58	6.696882	-7068	7.47E-05	824	0.333333	0.012673	RSBN1
7524.009	6.698812	-7068	7.51E-05	923	0.333333	0.01339	EIF1B
11117.26	7.137337	-7068	7.55E-05	1050	0.333333	0.014016	STXBP1
8345.953	7.129144	-7068	7.53E-05	954	0.333333	0.014177	NELL2
5963.826	9.736359	-7068	7.35E-05	591	0.333333	0.007451	CLCN7
8009.156	5.587225	-7068	7.57E-05	1092	0.333333	0.015565	CDC5L
10643.59	6.334226	-7068	7.63E-05	1172	0.333333	0.01752	CANX
8632.405	7.904521	-7068	7.50E-05	888	0.333333	0.012216	SUOX
8746.242	9.253241	-7068	7.47E-05	794	0.333333	0.010732	MYEF2
10578.83	8.19277	-7068	7.52E-05	959	0.333333	0.012768	HSPB1
13861.01	6.239188	-7068	7.71E-05	1343	0.333333	0.019248	ITGA6
17391.02	6.58719	-7068	7.79E-05	1455	0.333333	0.021757	AP2B1
10795.25	6.219848	-7068	7.63E-05	1182	0.333333	0.017393	GTF2E2
7022.469	6.146896	-7068	7.55E-05	981	0.333333	0.01552	STRN3
8908.562	8.452289	-7068	7.47E-05	850	0.333333	0.011432	FMO1
3418.836	9.31339	-7068	7.30E-05	484	0.333333	0.006438	KCNS1
11763.64	7.429207	-7068	7.58E-05	1044	0.333333	0.013655	SLC17A1
8728.248	6.828001	-7068	7.57E-05	1017	0.333333	0.015209	GNAS
10950.76	7.985595	-7068	7.55E-05	986	0.333333	0.014017	NUCB2
14130.89	6.790766	-7068	7.70E-05	1276	0.333333	0.018914	ALCAM

13938.05	6.855975	-7068	7.68E-05	1254	0.333333	0.018339	AMFR
15415.34	8.151782	-7068	7.63E-05	1140	0.333333	0.015545	ITGA10
6739.168	7.649737	-7068	7.47E-05	810	0.333333	0.011731	TMEM11
15121.04	5.653512	-7068	7.75E-05	1468	0.333333	0.019916	KDR
8770.164	6.531799	-7068	7.51E-05	997	0.333333	0.012533	VAV1
16650.2	6.925297	-7068	7.74E-05	1358	0.333333	0.020345	TCF12
20584.8	6.090797	-7068	7.87E-05	1642	0.333333	0.023399	VIM
25268.71	6.577504	-7068	7.94E-05	1734	0.333333	0.025378	ANK3
9031.626	6.95027	-7068	7.56E-05	1015	0.333333	0.015087	IGF2R
5178.74	9.694109	-7068	7.36E-05	592	0.333333	0.008584	ABCA5
6151.542	5.411564	-7068	7.50E-05	980	0.333333	0.014059	VCL
7752.906	6.800148	-7068	7.52E-05	936	0.333333	0.013834	PEX2
9852.336	7.2557	-7068	7.58E-05	1033	0.333333	0.015639	NCOA4
6144.974	6.352524	-7068	7.49E-05	884	0.333333	0.013694	MRPL19
7941.889	6.538097	-7068	7.54E-05	974	0.333333	0.014136	ODC1
8265.4	6.768609	-7068	7.54E-05	981	0.333333	0.014339	DHX8
6980.782	7.047296	-7068	7.50E-05	882	0.333333	0.013059	DCAF8
13326.21	5.685251	-7068	7.74E-05	1407	0.333333	0.020747	RAP1B
6015.758	9.982091	-7068	7.35E-05	601	0.333333	0.007459	CDX1
7386.813	6.890105	-7068	7.49E-05	894	0.333333	0.012062	ACTA1
23545.16	5.876688	-7068	7.96E-05	1815	0.333333	0.026369	ACTR3
12781.98	6.360474	-7068	7.66E-05	1251	0.333333	0.017924	NCOA3
7492.065	7.171455	-7068	7.52E-05	912	0.333333	0.014044	MAD2L1BP
5226.458	8.068047	-7068	7.38E-05	649	0.333333	0.008331	CNGA3
4525.545	7.597568	-7068	7.37E-05	660	0.333333	0.008595	MAP3K14
7248.749	7.931821	-7068	7.47E-05	810	0.333333	0.011862	PNLIP
10344.73	7.083316	-7068	7.60E-05	1079	0.333333	0.015853	GLRX
7173.836	6.84938	-7068	7.49E-05	897	0.333333	0.01288	SF1
5799.967	6.997765	-7068	7.43E-05	778	0.333333	0.010407	DDX49
11389.29	7.469059	-7068	7.60E-05	1071	0.333333	0.015557	WWOX
16448.69	5.846624	-7068	7.79E-05	1520	0.333333	0.022024	SMAD1
18278.4	5.837133	-7068	7.83E-05	1602	0.333333	0.022451	SYK
5374.041	5.395045	-7068	7.43E-05	878	0.333333	0.011124	RPL7
3281.597	5.319312	-7068	7.36E-05	694	0.333333	0.00933	WRN
22755.06	5.595581	-7068	7.94E-05	1824	0.333333	0.025465	SNRPB2
7591.478	6.460995	-7068	7.50E-05	945	0.333333	0.013463	TTN
19522.49	7.022892	-7068	7.82E-05	1487	0.333333	0.021821	ADRB2
8148.557	6.233676	-7068	7.54E-05	1024	0.333333	0.013931	HMGA1
10901.72	7.045422	-7068	7.60E-05	1097	0.333333	0.015305	TUBA4A
16022.09	6.989988	-7068	7.77E-05	1349	0.333333	0.021029	AFF3
9397.507	6.328007	-7068	7.60E-05	1092	0.333333	0.016701	PIK3C2A

10179.92	8.396977	-7068	7.51E-05	899	0.333333	0.0133	TSPYL4
5148.647	6.338145	-7068	7.45E-05	793	0.333333	0.011657	PDHX
13493.52	6.41419	-7068	7.63E-05	1235	0.333333	0.015774	ITIH2
12433.28	6.652814	-7068	7.67E-05	1217	0.333333	0.018127	PRKAR2B
16290.37	7.146132	-7068	7.72E-05	1318	0.333333	0.019303	TSPAN8
7463.481	8.244515	-7068	7.45E-05	796	0.333333	0.010739	ABCF1
27277.5	7.037321	-7068	7.94E-05	1715	0.333333	0.024228	NCOR2
8541.922	6.811956	-7068	7.54E-05	978	0.333333	0.014283	CA3
5902.935	6.615262	-7068	7.46E-05	831	0.333333	0.012386	MPHOSPH10
3899.977	6.789748	-7068	7.38E-05	670	0.333333	0.010278	SH3GLB1
17505.38	6.413183	-7068	7.77E-05	1458	0.333333	0.020559	CXCR4
5171.347	6.935106	-7068	7.39E-05	711	0.333333	0.008953	KRT14
5974.362	7.513589	-7068	7.43E-05	770	0.333333	0.010859	MTA1
8375.535	7.026285	-7068	7.52E-05	953	0.333333	0.01375	BIN1
9690.531	6.047066	-7068	7.62E-05	1128	0.333333	0.016857	SLBP
13001.36	7.04631	-7068	7.64E-05	1161	0.333333	0.016972	TOMM70
5693.773	8.654199	-7068	7.39E-05	679	0.333333	0.010027	FAN1
10585.47	7.151927	-7068	7.58E-05	1063	0.333333	0.015129	CASP6
11321.86	4.745233	-7068	7.67E-05	1393	0.333333	0.017757	MCM3
9863.112	6.767944	-7068	7.58E-05	1077	0.333333	0.01594	STK3
12201.53	6.905067	-7068	7.66E-05	1181	0.333333	0.017613	DOCK2
10168.89	6.803547	-7068	7.62E-05	1100	0.333333	0.01676	IFNGR2
24518.53	6.454329	-7068	7.96E-05	1744	0.333333	0.026272	PAPSS1
5595.803	7.762691	-7068	7.43E-05	741	0.333333	0.010983	SRRM2
10111.53	6.347525	-7068	7.65E-05	1147	0.333333	0.017899	SNRNP27
11953.52	7.070581	-7068	7.62E-05	1134	0.333333	0.016151	TRIP13
14517.12	7.364128	-7068	7.69E-05	1223	0.333333	0.018011	FOXC1
15176.53	9.299994	-7068	7.57E-05	1024	0.333333	0.013444	KLK6
10303.77	6.677803	-7068	7.63E-05	1126	0.333333	0.017308	IK
12284.6	7.118856	-7068	7.65E-05	1169	0.333333	0.01723	CALCOCO2
9915.249	8.906428	-7068	7.51E-05	866	0.333333	0.012072	T
3889.741	8.937955	-7068	7.31E-05	520	0.333333	0.006376	AMPD1
16162.94	7.708172	-7068	7.67E-05	1225	0.333333	0.017286	TPO
12271.11	6.314914	-7068	7.69E-05	1261	0.333333	0.019145	MAN2A1
11822.05	5.511961	-7068	7.70E-05	1351	0.333333	0.019593	CBFB
10418.8	6.849725	-7068	7.60E-05	1090	0.333333	0.015715	PCBP1
11810.14	6.772319	-7068	7.66E-05	1186	0.333333	0.017964	ADD1
5642.665	7.703924	-7068	7.41E-05	721	0.333333	0.01066	COX7A2L
10054.52	5.695252	-7068	7.62E-05	1208	0.333333	0.017509	CDC27
9571.748	8.259347	-7068	7.53E-05	928	0.333333	0.013751	ADORA2B
17699.08	6.70634	-7068	7.79E-05	1443	0.333333	0.021839	AMOTL2

12716.28	5.711472	-7068	7.64E-05	1324	0.333333	0.016438	RELA
12433.38	9.068947	-7068	7.54E-05	942	0.333333	0.013245	SLC10A2
7984.352	7.203539	-7068	7.52E-05	918	0.333333	0.013239	FMNL1
5339.192	9.086261	-7068	7.37E-05	622	0.333333	0.008254	MFSD10
9019.032	8.47966	-7068	7.50E-05	858	0.333333	0.011798	ALPI
13803.55	6.978935	-7068	7.65E-05	1215	0.333333	0.017087	ABCC2
9626.065	7.56767	-7068	7.52E-05	943	0.333333	0.012466	PIN1
15340.04	5.890911	-7068	7.73E-05	1441	0.333333	0.019542	MYH9
6487.695	7.113383	-7068	7.47E-05	833	0.333333	0.012353	SEC23B
8132.476	9.865371	-7068	7.42E-05	723	0.333333	0.008963	RAC3
6983.807	6.749413	-7068	7.45E-05	871	0.333333	0.011629	ACTN2
5827.662	6.246702	-7068	7.45E-05	823	0.333333	0.010909	BYSL
6101.231	6.987681	-7068	7.45E-05	778	0.333333	0.011442	BCAS2
6183.17	6.902949	-7068	7.47E-05	831	0.333333	0.01264	UBE2J1
6573.311	7.682437	-7068	7.45E-05	768	0.333333	0.010482	PRRC2A
17523.25	6.35356	-7068	7.79E-05	1476	0.333333	0.02154	PMP22
10320.86	6.674804	-7068	7.58E-05	1094	0.333333	0.015133	HSD17B4
15933.26	5.633437	-7068	7.82E-05	1549	0.333333	0.023175	NOLC1
7421.117	7.861241	-7068	7.45E-05	823	0.333333	0.010455	RPL39
9118.267	6.748879	-7068	7.56E-05	1019	0.333333	0.014259	PPP2R5D
16814.42	6.640072	-7068	7.76E-05	1404	0.333333	0.020774	MAP1B
5446.113	8.917385	-7068	7.39E-05	648	0.333333	0.008841	PLCB3
5508.364	7.419491	-7068	7.42E-05	738	0.333333	0.010534	BDNF
22109.95	5.893488	-7068	7.88E-05	1719	0.333333	0.02314	CDH1
8741.795	5.843034	-7068	7.55E-05	1073	0.333333	0.014162	ACADSB
11718.01	6.323429	-7068	7.64E-05	1218	0.333333	0.017731	NCOA1
28930.16	6.911741	-7068	8.04E-05	1816	0.333333	0.027958	CADPS
18252.18	7.386721	-7068	7.76E-05	1355	0.333333	0.019737	TYRP1
5172.991	10.01921	-7068	7.32E-05	537	0.333333	0.006356	LY6G6C
19969.17	7.917957	-7068	7.77E-05	1374	0.333333	0.02109	TBC1D5
17320.27	9.167993	-7068	7.62E-05	1102	0.333333	0.015092	OPRD1
6486.634	7.755512	-7068	7.44E-05	775	0.333333	0.010865	CYP24A1
16428.25	7.223151	-7068	7.71E-05	1296	0.333333	0.017344	CYP27A1
11000.55	7.17658	-7068	7.60E-05	1080	0.333333	0.015209	TSPO
7987.785	8.324648	-7068	7.46E-05	823	0.333333	0.011474	ROS1
7137.087	12.3992	-7068	7.34E-05	584	0.333333	0.007495	ZNF35
10258.5	6.545213	-7068	7.58E-05	1075	0.333333	0.01481	EEF1E1
6018.82	7.714666	-7068	7.41E-05	728	0.333333	0.009824	TCN1
11328.72	6.334089	-7068	7.63E-05	1189	0.333333	0.017533	ARF4
5954.047	8.267661	-7068	7.40E-05	698	0.333333	0.009193	CRYAA
7659.83	7.232513	-7068	7.50E-05	904	0.333333	0.013129	PSEN2

18723.88	6.896671	-7068	7.77E-05	1429	0.333333	0.020594	ID4
6269.486	8.078001	-7068	7.37E-05	702	0.25	0.008489	FABP1
6945.934	10.13462	-7068	7.38E-05	655	0.333333	0.008847	RFX2
9493.709	6.446886	-7068	7.61E-05	1084	0.333333	0.01595	COPS3
7989.464	7.239231	-7068	7.50E-05	906	0.333333	0.01279	HAX1
8967.684	9.245278	-7068	7.45E-05	786	0.333333	0.010991	CRYBA1
7504.086	6.440863	-7068	7.50E-05	935	0.333333	0.012259	CAD
12746.35	7.615841	-7068	7.63E-05	1119	0.333333	0.016685	DGKG
6799.992	6.591092	-7068	7.52E-05	909	0.333333	0.014118	BBX
9280.998	8.169764	-7068	7.51E-05	900	0.333333	0.012718	PPP2R5B
4994.259	8.474905	-7068	7.36E-05	626	0.333333	0.008379	CHGA
4257.956	8.511346	-7068	7.33E-05	571	0.333333	0.007348	GABRD
6522.995	6.338863	-7068	7.51E-05	913	0.333333	0.014259	BNIP2
20903.03	7.050972	-7068	7.85E-05	1520	0.333333	0.023145	STAC
8098.843	7.987918	-7068	7.50E-05	850	0.333333	0.012301	EVI2A
8781.652	8.882033	-7068	7.46E-05	813	0.333333	0.011643	ZNF146
20209.62	5.928254	-7068	7.86E-05	1673	0.333333	0.02412	TGFBR2
6025.591	5.619442	-7068	7.48E-05	933	0.333333	0.012672	PSMB7
7973.543	7.686204	-7068	7.49E-05	875	0.333333	0.011972	GPX1
5349.648	9.871654	-7068	7.34E-05	577	0.333333	0.007707	IL5
6695.701	5.265301	-7068	7.50E-05	997	0.333333	0.01268	PSMB5
7909.661	5.152324	-7068	7.55E-05	1118	0.333333	0.014547	RNPS1
11296.72	9.651595	-7068	7.47E-05	830	0.333333	0.010282	SLC5A2
7879.987	5.191397	-7068	7.58E-05	1118	0.333333	0.016104	DDX18
5707.977	10.99768	-7068	7.33E-05	530	0.333333	0.00642	TULP1
8193.888	7.26929	-7068	7.54E-05	941	0.333333	0.014176	SLC7A6
6305.138	6.737381	-7068	7.45E-05	840	0.333333	0.011399	FANCG
10278.44	6.742752	-7068	7.60E-05	1101	0.333333	0.016451	UNG
7240.876	5.230463	-7068	7.51E-05	1013	0.333333	0.012927	CASP1
12537.09	6.605157	-7068	7.69E-05	1239	0.333333	0.018941	MORF4L2
16399.51	6.582405	-7068	7.70E-05	1354	0.333333	0.017492	PROC
10337.19	6.681912	-7068	7.61E-05	1116	0.333333	0.016401	CEBPG
8089.425	6.206203	-7068	7.54E-05	1014	0.333333	0.014773	BMPR2
6866.527	8.208756	-7068	7.43E-05	761	0.333333	0.009966	EMD
14851.21	6.392197	-7068	7.74E-05	1370	0.333333	0.020399	SOX9
14266.74	5.733813	-7068	7.72E-05	1409	0.333333	0.018958	CDC7
8681.303	6.934581	-7068	7.55E-05	989	0.333333	0.014757	NEO1
10928.78	6.021408	-7068	7.63E-05	1215	0.333333	0.017314	ITGAV
7053.049	6.340238	-7068	7.49E-05	901	0.333333	0.011899	HLA-DPB1
7300.681	6.912279	-7068	7.49E-05	885	0.333333	0.012408	KNTC1
11879.51	7.889497	-7068	7.55E-05	1046	0.333333	0.014486	CCNH

5851.751	8.634928	-7068	7.37E-05	644	0.333333	0.0079	LGALS4
6780.027	6.949862	-7068	7.45E-05	842	0.333333	0.010698	TRAF1
16717.16	7.15244	-7068	7.72E-05	1335	0.333333	0.019516	GRK5
9063.843	5.832841	-7068	7.56E-05	1109	0.333333	0.014567	BLM
5866.257	6.42219	-7068	7.43E-05	819	0.333333	0.010817	PRPF3
8147.89	5.376706	-7068	7.59E-05	1132	0.333333	0.016631	PSMD12
12709.31	5.516792	-7068	7.71E-05	1387	0.333333	0.019459	ACTN1
12343.39	6.104208	-7068	7.68E-05	1291	0.333333	0.018623	YWHAH
12975.48	7.583617	-7068	7.59E-05	1101	0.333333	0.015783	PSMD10
8732.368	8.28118	-7068	7.49E-05	851	0.333333	0.012319	IMPDH2
6641.435	7.847553	-7068	7.44E-05	749	0.333333	0.009702	NELFB
3902.324	5.575027	-7068	7.42E-05	761	0.333333	0.011428	GSPT1
24672.92	6.291128	-7068	7.97E-05	1761	0.333333	0.026297	XYLT1
13223.79	6.893859	-7068	7.68E-05	1225	0.333333	0.01814	RELN
10394.04	6.478308	-7068	7.58E-05	1086	0.333333	0.014188	POLR2E
8016.249	6.827836	-7068	7.50E-05	950	0.333333	0.012616	TNF
8031.116	8.498995	-7068	7.45E-05	789	0.333333	0.010126	GABARAP
7059.945	6.842841	-7068	7.45E-05	866	0.333333	0.011008	CD3E
13177.83	5.401417	-7068	7.71E-05	1429	0.333333	0.019379	ITGB1
21071.05	6.167218	-7068	7.86E-05	1638	0.333333	0.022469	SNRPB
11281.83	5.873777	-7068	7.65E-05	1260	0.333333	0.018224	IRS1
5114.414	7.017713	-7068	7.42E-05	752	0.333333	0.01089	UROD
11171.29	6.814613	-7068	7.64E-05	1140	0.333333	0.017377	LNPEP
5015.055	7.566795	-7068	7.39E-05	677	0.333333	0.008621	ARF5
7705.102	7.322489	-7068	7.52E-05	902	0.333333	0.01311	LASP1
5665.77	5.894588	-7068	7.45E-05	848	0.333333	0.011352	CCR1
10806.58	9.751606	-7068	7.45E-05	789	0.333333	0.009545	CRYBB1
6968.762	7.153816	-7068	7.49E-05	864	0.333333	0.012988	TRIP4
3099.024	7.115244	-7068	7.34E-05	588	0.333333	0.008765	SLC25A12
17528.82	7.13717	-7068	7.77E-05	1377	0.333333	0.020357	TFAP2C
8723.097	8.214149	-7068	7.51E-05	882	0.333333	0.012803	SUN2
15059.45	6.733289	-7068	7.68E-05	1290	0.333333	0.017684	CAMK2A
8052.743	11.93793	-7068	7.35E-05	582	0.333333	0.006873	CRYGC
5888.012	8.670495	-7068	7.41E-05	695	0.333333	0.009941	PRKAB2
34067.61	6.380749	-7068	8.10E-05	2056	0.333333	0.029188	DAPK1
10021.33	8.505043	-7068	7.51E-05	898	0.333333	0.01235	IMPDH1
13346.57	6.026062	-7068	7.74E-05	1357	0.333333	0.02053	TLK2
10860.47	9.103486	-7068	7.50E-05	877	0.333333	0.011925	PDE6A
9920.209	6.876113	-7068	7.59E-05	1065	0.333333	0.016284	TSC22D1
10494.88	6.449489	-7068	7.64E-05	1153	0.333333	0.017447	TXNRD1
19249.74	6.536625	-7068	7.82E-05	1523	0.333333	0.021587	YARS

9777.271	7.063624	-7068	7.57E-05	1031	0.333333	0.014753	HINT1
5522.328	7.291489	-7068	7.45E-05	769	0.333333	0.01198	SACS
4097.202	7.068233	-7068	7.37E-05	646	0.333333	0.008762	ZNF593
4915.649	7.833867	-7068	7.40E-05	684	0.333333	0.010446	MFN1
8138.537	6.619185	-7068	7.51E-05	967	0.333333	0.012985	EIF5A
13767.96	6.255419	-7068	7.72E-05	1352	0.333333	0.0199	ACLY
23348.82	6.871935	-7068	7.89E-05	1620	0.333333	0.024136	SGMS1
7477.556	5.883763	-7068	7.53E-05	1003	0.333333	0.013338	IRF1
12673.8	6.301198	-7068	7.69E-05	1282	0.333333	0.019211	MEF2C
10165.53	6.916137	-7068	7.58E-05	1073	0.333333	0.015422	NFATC1
10857.78	6.088668	-7068	7.65E-05	1201	0.333333	0.017089	LAPTM5
10371.65	10.03459	-7068	7.47E-05	805	0.333333	0.011501	POU4F2
11018.29	7.518669	-7068	7.59E-05	1060	0.333333	0.015766	SIAH2
12528.17	6.571872	-7068	7.68E-05	1238	0.333333	0.018818	GNS
11177.03	6.994646	-7068	7.59E-05	1096	0.333333	0.015576	MNAT1
15114.49	6.487518	-7068	7.74E-05	1358	0.333333	0.020528	DYRK1A
7521.49	7.06274	-7068	7.48E-05	872	0.333333	0.011313	CTSA
28994.68	7.519309	-7068	7.95E-05	1689	0.333333	0.02505	MAML3
25146.83	7.569477	-7068	7.87E-05	1563	0.333333	0.02343	ANKS1A
8867.151	5.670205	-7068	7.54E-05	1077	0.333333	0.013473	SNRNP40
7330.128	6.746708	-7068	7.49E-05	878	0.333333	0.011686	PUF60
13592.06	6.478495	-7068	7.70E-05	1297	0.333333	0.019461	IL7
7744.831	8.818151	-7068	7.45E-05	775	0.333333	0.010928	PTH
5177.566	8.509428	-7068	7.40E-05	670	0.333333	0.009853	EYA3
8552.175	6.561939	-7068	7.54E-05	1004	0.333333	0.01433	HSPE1
5249.171	6.762906	-7068	7.43E-05	777	0.333333	0.011576	ATRX
3062.068	6.642845	-7068	7.32E-05	588	0.333333	0.008524	SMAD5
5572.806	8.625478	-7068	7.39E-05	660	0.333333	0.009024	CRMP1
8458.789	7.965525	-7068	7.51E-05	883	0.333333	0.013007	CFTR
4415.471	5.406973	-7068	7.42E-05	785	0.333333	0.010279	CD74
8067.625	6.270292	-7068	7.55E-05	1011	0.333333	0.015205	WWP1
15284.59	7.684141	-7068	7.66E-05	1189	0.333333	0.016875	CACNG3
5468.375	7.466313	-7068	7.34E-05	640	0.333333	0.008002	ZNF84
13552.92	6.279062	-7068	7.68E-05	1305	0.333333	0.018732	MAP2K4
9546.953	8.275223	-7068	7.51E-05	902	0.333333	0.01211	NR1H3
5978.412	6.555976	-7068	7.49E-05	867	0.333333	0.013374	ELF1
6189.619	7.701084	-7068	7.44E-05	757	0.333333	0.010386	REN
6948.895	8.775531	-7068	7.42E-05	725	0.333333	0.009635	PIK3R2
16863.57	6.546008	-7068	7.79E-05	1440	0.333333	0.022079	ROBO1
19399.8	7.231711	-7068	7.79E-05	1430	0.333333	0.0206	PINK1
13354.46	8.228211	-7068	7.61E-05	1060	0.333333	0.014781	SPINT2

6864.525	8.551817	-7068	7.42E-05	712	0.333333	0.008714	CYP4F12
19380.56	7.434387	-7068	7.78E-05	1398	0.333333	0.02061	TIMP4
3762.361	4.953497	-7068	7.38E-05	780	0.333333	0.010177	RPS6
4180.485	7.010561	-7068	7.39E-05	673	0.333333	0.010056	BPTF
8440.808	8.303421	-7068	7.47E-05	835	0.333333	0.011713	GABRA6
10658.59	6.401913	-7068	7.61E-05	1153	0.333333	0.015917	FES
7598.914	7.303666	-7068	7.51E-05	898	0.333333	0.013534	P2RX5
18147.11	6.692857	-7068	7.79E-05	1440	0.333333	0.020996	BMP4
7595.445	7.457347	-7068	7.48E-05	861	0.333333	0.011896	ATP6V0A1
5740.769	7.947148	-7068	7.40E-05	701	0.333333	0.009146	ARL2
11077.87	6.357406	-7068	7.66E-05	1195	0.333333	0.018731	CHMP2B
10649.78	7.620443	-7068	7.55E-05	1003	0.333333	0.01382	SERPINA5
5825.526	5.997698	-7068	7.45E-05	885	0.333333	0.011734	ERBB2
18068.3	6.783421	-7068	7.78E-05	1432	0.333333	0.02039	NGFR
8933.481	7.553421	-7068	7.52E-05	942	0.333333	0.013045	FGFR4
8490.183	7.715942	-7068	7.48E-05	880	0.333333	0.012427	SCG2
8223.86	7.58617	-7068	7.50E-05	892	0.333333	0.012347	GSS
3061.507	7.140691	-7068	7.33E-05	567	0.333333	0.007976	TCF7
19436.33	6.592527	-7068	7.83E-05	1514	0.333333	0.022318	GPM6A
6356.927	9.829064	-7068	7.35E-05	588	0.333333	0.006465	CACNA1F
12915.71	6.810526	-7068	7.62E-05	1181	0.333333	0.015458	HSD17B10
6435.902	8.713026	-7068	7.41E-05	719	0.333333	0.010897	WSB1
9340.84	6.110539	-7068	7.57E-05	1108	0.333333	0.015062	STAT5A
20943.25	6.454786	-7068	7.84E-05	1590	0.333333	0.022328	NNT
26771.99	6.096664	-7068	7.98E-05	1884	0.333333	0.02607	HMGB1
8451.524	7.014433	-7068	7.54E-05	957	0.333333	0.014335	LUC7L3
4838.759	7.517152	-7068	7.41E-05	703	0.333333	0.010159	ALDH5A1
4553.365	6.8726	-7068	7.40E-05	713	0.333333	0.010322	IFIT1
24794.42	6.264514	-7068	7.94E-05	1765	0.333333	0.025364	SPARC
8135.333	7.863845	-7068	7.48E-05	855	0.333333	0.01177	CCKAR
3938.096	8.33653	-7068	7.34E-05	566	0.333333	0.007522	NUMA1
8221.562	7.782828	-7068	7.48E-05	861	0.333333	0.011438	MAG
9893.085	6.138674	-7068	7.64E-05	1153	0.333333	0.017695	RAB21
12278.73	6.14945	-7068	7.63E-05	1251	0.333333	0.016712	CASP8
3544.572	11.21677	-7068	7.27E-05	435	0.333333	0.005632	MC1R
8982.469	6.788628	-7068	7.57E-05	1023	0.333333	0.01516	RTN4
19061.19	6.792225	-7068	7.75E-05	1474	0.333333	0.020087	SP1
10009	7.150594	-7068	7.51E-05	956	0.333333	0.010974	APOC3
5671.14	6.331831	-7068	7.45E-05	835	0.333333	0.011694	POLR2K
14789.4	5.937599	-7068	7.72E-05	1431	0.333333	0.020023	YWHAB
5344.766	7.292547	-7068	7.43E-05	737	0.333333	0.011248	ZMYM2

4378.985	7.433142	-7068	7.36E-05	645	0.333333	0.008473	COPS6
23061.35	8.412039	-7068	7.78E-05	1369	0.333333	0.019082	NOS1AP
9921.917	5.820187	-7068	7.56E-05	1160	0.333333	0.014709	AR
10864.08	6.496002	-7068	7.59E-05	1110	0.333333	0.01507	MNDA
12029.16	5.06584	-7068	7.67E-05	1380	0.333333	0.01778	RRM1
5237.538	6.637484	-7068	7.44E-05	790	0.333333	0.01194	RABEP1
9459.88	7.965677	-7068	7.51E-05	917	0.333333	0.012991	HSD3B1
6008.433	6.284043	-7068	7.48E-05	889	0.333333	0.012382	CCND3
5965.162	5.36346	-7068	7.50E-05	960	0.333333	0.013791	EIF2S1
6878.276	7.885728	-7068	7.44E-05	790	0.333333	0.010776	IL12B
18484.3	7.380961	-7068	7.73E-05	1348	0.333333	0.017934	BLVRB
15578.17	6.856245	-7068	7.75E-05	1345	0.333333	0.020444	ANXA4
6837.009	9.053178	-7068	7.41E-05	705	0.333333	0.009234	ACTR1B
10100.3	6.773033	-7068	7.58E-05	1080	0.333333	0.015581	SMAD7
6138.755	6.756134	-7068	7.45E-05	828	0.333333	0.011846	ISG15
8655.405	7.979046	-7068	7.51E-05	900	0.333333	0.012904	SLC3A2
7570.535	6.631262	-7068	7.51E-05	938	0.333333	0.013226	ADGRE5
13380.79	7.64592	-7068	7.65E-05	1145	0.333333	0.017368	UAP1
8144.729	7.253839	-7068	7.49E-05	898	0.333333	0.01228	TAF12
23869.57	6.516404	-7068	7.86E-05	1648	0.333333	0.022161	C8A
7311.783	6.477851	-7068	7.54E-05	958	0.333333	0.014987	TRIB2
18636.54	6.884751	-7068	7.81E-05	1447	0.333333	0.021809	FGF12
10454.61	8.649682	-7068	7.51E-05	897	0.333333	0.012237	PRPH
13580.55	6.059645	-7068	7.70E-05	1345	0.333333	0.019236	CLTC
10461.97	6.534525	-7068	7.63E-05	1139	0.333333	0.017731	LPGAT1
14624.59	7.559475	-7068	7.67E-05	1199	0.333333	0.017989	EZH1
5267.673	5.79597	-7068	7.48E-05	869	0.333333	0.013055	DDX46
7982.702	8.013613	-7068	7.48E-05	844	0.333333	0.012246	USP4
7498.443	5.099023	-7068	7.54E-05	1081	0.333333	0.01358	ITGB2
19609.44	6.713988	-7068	7.84E-05	1533	0.333333	0.022442	GCH1
10788.19	7.918828	-7068	7.55E-05	976	0.333333	0.012886	ATP6V0D1
25124.3	6.72067	-7068	7.92E-05	1702	0.333333	0.024622	VEGFC
5900.861	5.986875	-7068	7.43E-05	847	0.333333	0.010569	G6PC
16137.01	7.617764	-7068	7.69E-05	1249	0.333333	0.018196	MGLL
12201.36	7.460066	-7068	7.62E-05	1121	0.333333	0.016663	LDHB
3816.304	6.585607	-7068	7.37E-05	665	0.333333	0.009451	DDX42
5048.654	6.22986	-7068	7.44E-05	812	0.333333	0.011875	GTF2H1
8823.408	6.768808	-7068	7.48E-05	922	0.333333	0.011608	COX6B1
9539.571	7.230582	-7068	7.55E-05	999	0.333333	0.01434	FHL1
5562.441	7.349976	-7068	7.42E-05	746	0.333333	0.010155	GTF2H4
5533.635	7.370448	-7068	7.41E-05	725	0.333333	0.009439	ATP6V0B

7453.865	7.646434	-7068	7.49E-05	853	0.333333	0.012776	SNAPC1
13030.75	8.028774	-7068	7.61E-05	1088	0.333333	0.015816	EDN2
15035.89	6.08148	-7068	7.71E-05	1396	0.333333	0.019156	ATP5O
4698.8	7.59662	-7068	7.38E-05	659	0.333333	0.009674	BET1
6273.744	8.032078	-7068	7.41E-05	730	0.333333	0.009637	PLXNB1
4892.533	8.546675	-7068	7.37E-05	624	0.333333	0.008296	ELAVL3
9350.311	7.281368	-7068	7.55E-05	970	0.333333	0.013539	SELP
13789.73	6.28013	-7068	7.73E-05	1338	0.333333	0.020045	IPO7
16396.24	5.5376	-7068	7.73E-05	1511	0.333333	0.018486	PLK1
16626.3	6.990822	-7068	7.74E-05	1351	0.333333	0.019529	TGFA
11286.76	6.328583	-7068	7.66E-05	1199	0.333333	0.017906	IFNGR1
7151.044	7.787858	-7068	7.45E-05	792	0.333333	0.010448	KRT1
16976.82	6.57326	-7068	7.79E-05	1447	0.333333	0.022276	SEL1L
12416.2	7.123756	-7068	7.65E-05	1165	0.333333	0.017446	NGF
6829.001	10.61493	-7068	7.34E-05	603	0.333333	0.008028	FABP2
9990.081	6.973172	-7068	7.59E-05	1044	0.333333	0.015932	DNAJB9
16512.21	6.630582	-7068	7.75E-05	1400	0.333333	0.019679	CTH
17856.44	6.259563	-7068	7.82E-05	1525	0.333333	0.022551	ITGA2
2882.316	6.833126	-7068	7.33E-05	572	0.333333	0.0086	CCP110
7060.958	9.466633	-7068	7.42E-05	701	0.333333	0.009688	CD1A
9330.67	8.852212	-7068	7.49E-05	832	0.333333	0.010864	RABGGTA
12675	7.184889	-7068	7.65E-05	1157	0.333333	0.017359	MED7
9740.799	8.621144	-7068	7.50E-05	879	0.333333	0.012547	SAG
6698.096	6.169765	-7068	7.48E-05	907	0.333333	0.012213	DDX23
5254.195	7.067355	-7068	7.43E-05	751	0.333333	0.010731	WNT2
7452.674	9.243626	-7068	7.40E-05	708	0.333333	0.009273	DUSP9
9934.622	8.716457	-7068	7.50E-05	869	0.333333	0.011776	ADH7
11647.61	7.218303	-7068	7.61E-05	1111	0.333333	0.016014	SEC13
9055.374	8.858783	-7068	7.48E-05	832	0.333333	0.011681	ZNF507
9590.531	7.072378	-7068	7.55E-05	1023	0.333333	0.014035	SMARCD1
4742.924	6.698877	-7068	7.43E-05	747	0.333333	0.011597	ATP6V1G1
5409.065	6.094915	-7068	7.42E-05	841	0.333333	0.010601	U2AF2
8406.263	6.823374	-7068	7.53E-05	976	0.333333	0.014069	CRYZ
15233.55	8.42961	-7068	7.64E-05	1121	0.333333	0.015554	EMP3
13911.45	7.00999	-7068	7.67E-05	1233	0.333333	0.017329	PTTG1
4095.709	5.193301	-7068	7.42E-05	813	0.333333	0.011692	EIF3A
6745.166	6.648105	-7068	7.50E-05	896	0.333333	0.013345	APBA2
10914.58	7.852106	-7068	7.56E-05	996	0.333333	0.014089	INHBB
10693.43	6.591882	-7068	7.63E-05	1143	0.333333	0.017445	MARCKS
8044.445	6.6857	-7068	7.49E-05	896	0.333333	0.011343	TYROBP
8265.098	7.201572	-7068	7.52E-05	938	0.333333	0.013674	HEXA

10025.48	7.226197	-7068	7.50E-05	981	0.333333	0.012298	CD4
7608.853	8.379337	-7068	7.45E-05	787	0.333333	0.010967	SLC9A3R2
28769.22	6.582903	-7068	7.97E-05	1839	0.333333	0.02587	ID2
13671.22	6.514981	-7068	7.69E-05	1281	0.333333	0.018664	MAPK9
7716.777	7.304777	-7068	7.48E-05	878	0.333333	0.011301	ECH1
6263.992	7.856787	-7068	7.41E-05	744	0.333333	0.009932	PWP2
8149.569	7.006133	-7068	7.54E-05	956	0.333333	0.014346	PTPRA
27631.82	7.012318	-7068	7.97E-05	1744	0.333333	0.025452	CYP19A1
7795.911	7.471571	-7068	7.48E-05	874	0.333333	0.012416	NLRP1
17278.25	7.886532	-7068	7.69E-05	1251	0.333333	0.017261	FCGRT
3791.244	6.256316	-7068	7.36E-05	684	0.333333	0.008862	CASP9
6504.984	6.818153	-7068	7.44E-05	820	0.333333	0.010206	ATP5D
13496.9	6.341377	-7068	7.61E-05	1219	0.333333	0.014804	SERPINC1
18924.55	10.08354	-7068	7.59E-05	1052	0.333333	0.014217	ADRA1D
16410.69	4.804452	-7068	7.79E-05	1694	0.333333	0.021832	STAT3
9163.971	6.213471	-7068	7.57E-05	1076	0.333333	0.015517	SRP72
8230.846	6.521164	-7068	7.53E-05	975	0.333333	0.013619	CXCL9
16023.3	6.874641	-7068	7.68E-05	1296	0.333333	0.016512	APCS
7530.019	6.778674	-7068	7.43E-05	843	0.333333	0.00956	SERPINA7
20859.08	7.704413	-7068	7.78E-05	1416	0.333333	0.021655	RABGAP1L
6145.252	7.903559	-7068	7.44E-05	755	0.333333	0.010954	RAMP1
11540.86	6.716427	-7068	7.66E-05	1169	0.333333	0.017588	GABRB3
9471.696	6.729391	-7068	7.59E-05	1069	0.333333	0.016199	PIP5K1A
11489.58	6.84566	-7068	7.64E-05	1149	0.333333	0.01716	LIPA
9525.311	6.451777	-7068	7.60E-05	1089	0.333333	0.015866	CSRP1
6249.603	6.091147	-7068	7.48E-05	872	0.333333	0.012673	EXOSC8
11241.71	8.202224	-7068	7.56E-05	963	0.333333	0.013068	SLC25A1
6800.74	6.043618	-7068	7.54E-05	959	0.333333	0.014807	CNIH1
7326.863	9.633347	-7068	7.40E-05	677	0.333333	0.008994	SIX6
8652.67	7.453762	-7068	7.50E-05	925	0.333333	0.012925	CALM1
8211.952	6.813415	-7068	7.52E-05	951	0.333333	0.013047	RANGAP1
19822.4	6.279811	-7068	7.89E-05	1609	0.333333	0.024569	FOXN3
8565.944	6.775436	-7068	7.51E-05	958	0.333333	0.012705	POLR2H
11094.29	7.519915	-7068	7.59E-05	1046	0.333333	0.015368	RYR1
9464.79	6.389439	-7068	7.58E-05	1087	0.333333	0.015032	PRKCD
10329.27	8.495489	-7068	7.50E-05	883	0.333333	0.011405	PAX7
8705.325	9.020503	-7068	7.46E-05	789	0.333333	0.010856	RPE65
8916.437	6.511046	-7068	7.57E-05	1043	0.333333	0.015917	ADAM10
7883.506	5.932266	-7068	7.59E-05	1062	0.333333	0.016549	PRKAR1A
5515.627	5.568177	-7068	7.45E-05	850	0.333333	0.011172	ATP5F1
13374.14	6.491558	-7068	7.65E-05	1258	0.333333	0.017585	NCOA2

10027.46	10.46863	-7068	7.45E-05	774	0.333333	0.010509	MAST1
9810.29	9.170971	-7068	7.47E-05	794	0.333333	0.009403	FOXN1
15395	6.338398	-7068	7.75E-05	1412	0.333333	0.020073	MAPKAPK3
4691.689	7.609093	-7068	7.39E-05	668	0.333333	0.009215	PIP5K1C
14462.1	6.746301	-7068	7.72E-05	1304	0.333333	0.019678	WASF2
18448.51	6.586003	-7068	7.76E-05	1450	0.333333	0.019611	ATP5B
5286.553	6.845363	-7068	7.41E-05	736	0.333333	0.009334	GYS2
12820.12	6.865709	-7068	7.68E-05	1221	0.333333	0.018374	CD58
7956.523	7.389344	-7068	7.52E-05	909	0.333333	0.013929	DEGS1
9199.534	7.703491	-7068	7.53E-05	933	0.333333	0.012819	ALPL
10633.41	11.4304	-7068	7.41E-05	688	0.333333	0.007805	CCL1
7701.384	7.687078	-7068	7.49E-05	855	0.333333	0.01156	NR1H2
9901.66	6.913465	-7068	7.56E-05	1039	0.333333	0.015562	ENC1
4707.452	9.287869	-7068	7.35E-05	571	0.333333	0.007671	GAL3ST1
29028.01	6.567059	-7068	8.04E-05	1875	0.333333	0.028135	PRKG1
8836.09	5.914102	-7068	7.57E-05	1100	0.333333	0.015634	IL6ST
11424.06	7.1887	-7068	7.60E-05	1076	0.333333	0.01474	SRM
7374.478	8.501866	-7068	7.37E-05	707	0.333333	0.007638	ELANE
4332.277	6.505144	-7068	7.42E-05	742	0.333333	0.011731	ST13
10936.07	6.309836	-7068	7.60E-05	1157	0.333333	0.015635	ITGA5
8605.303	6.048405	-7068	7.57E-05	1055	0.333333	0.015481	TMED10
5234.303	8.53239	-7068	7.36E-05	628	0.333333	0.008061	GPR37L1
7870.614	7.598761	-7068	7.51E-05	878	0.333333	0.012724	PLD3
17278.28	6.682012	-7068	7.73E-05	1397	0.333333	0.018691	LIPC
6735.134	6.39686	-7068	7.48E-05	888	0.333333	0.012199	FYB
14289.06	6.611262	-7068	7.70E-05	1306	0.333333	0.018909	ALOX5
5502.139	6.688522	-7068	7.45E-05	804	0.333333	0.012478	IBTK
12958.71	9.785697	-7068	7.53E-05	925	0.333333	0.012417	XRCC3
5941.953	8.433155	-7068	7.40E-05	694	0.333333	0.009524	ARHGEF16
7455.287	6.73131	-7068	7.52E-05	927	0.333333	0.014146	RNF6
13110.56	6.113148	-7068	7.71E-05	1336	0.333333	0.019793	PAK2
6624.735	7.574071	-7068	7.46E-05	807	0.333333	0.012196	TDP2
8421.472	7.251388	-7068	7.52E-05	941	0.333333	0.013158	CTSD
17866.45	6.336287	-7068	7.83E-05	1504	0.333333	0.022841	RAPGEF2
26106.03	8.069003	-7068	7.86E-05	1519	0.333333	0.02292	PDHA2
13694.26	6.601508	-7068	7.64E-05	1231	0.333333	0.016073	ITGAM
15579.6	6.224511	-7068	7.79E-05	1434	0.333333	0.022028	PRKD1
6717.481	9.080893	-7068	7.41E-05	685	0.333333	0.009204	KRT20
5453.822	7.123813	-7068	7.42E-05	733	0.333333	0.00923	MAN2B1
5224.684	7.434382	-7068	7.40E-05	714	0.333333	0.009675	LIG1
11222.68	10.67499	-7068	7.42E-05	729	0.333333	0.008529	RCVRN

7840.255	8.229543	-7068	7.47E-05	813	0.333333	0.011003	CST3
6229.774	8.152567	-7068	7.42E-05	732	0.333333	0.009938	ERP29
9023.19	7.198628	-7068	7.56E-05	984	0.333333	0.014077	ARPC1B
1375.181	4.505423	-7068	7.26E-05	499	0.333333	0.006662	NACA
13842.52	6.357189	-7068	7.72E-05	1331	0.333333	0.019344	RBBP8
14567.06	7.581631	-7068	7.67E-05	1205	0.333333	0.017486	MCC
8007.215	6.191757	-7068	7.49E-05	952	0.333333	0.011468	CYP2C9
5758.116	5.739719	-7068	7.46E-05	904	0.333333	0.011785	RAF1
7185.821	7.573038	-7068	7.46E-05	828	0.333333	0.011408	MYO1C
9450.466	7.825052	-7068	7.53E-05	939	0.333333	0.01385	PRKAG1
10272.47	6.223813	-7068	7.58E-05	1128	0.333333	0.015112	H2AFX
11078.37	7.147697	-7068	7.63E-05	1108	0.333333	0.017056	CCDC86
4176.366	6.053847	-7068	7.37E-05	713	0.333333	0.00904	ITK
6182.652	6.850039	-7068	7.48E-05	853	0.333333	0.013219	FNTA
10315.68	5.76243	-7068	7.61E-05	1181	0.333333	0.015994	CDH5
15070.29	6.73031	-7068	7.73E-05	1323	0.333333	0.019648	AIMP2
6847.306	8.249369	-7068	7.44E-05	765	0.333333	0.011468	SERPINI1
8450.006	6.477944	-7068	7.54E-05	1015	0.333333	0.014676	RANBP2
7906.363	6.194774	-7068	7.51E-05	982	0.333333	0.012903	PSMC5
6929.146	6.460265	-7068	7.50E-05	912	0.333333	0.013781	ARL1
7795.124	7.899235	-7068	7.46E-05	815	0.333333	0.010238	ATP6V1B1
4840.099	8.718537	-7068	7.36E-05	607	0.333333	0.008189	PDYN
13210.44	7.573186	-7068	7.67E-05	1165	0.333333	0.018389	CACNA1E
5335.08	7.852294	-7068	7.41E-05	703	0.333333	0.010412	KCNH2
30266.19	6.093969	-7068	8.08E-05	2009	0.333333	0.029324	IQGAP2
8984.214	7.274391	-7068	7.54E-05	968	0.333333	0.014228	NMI
23405.9	6.946602	-7068	7.84E-05	1577	0.333333	0.021687	CTSK
6768.157	7.616245	-7068	7.48E-05	831	0.333333	0.012918	OXCT1
4107.195	9.415604	-7068	7.32E-05	535	0.333333	0.00764	ZSCAN26
6531.188	6.964027	-7068	7.48E-05	848	0.333333	0.013	KDM5B
6747.058	7.697796	-7068	7.43E-05	789	0.333333	0.010607	CYP4B1
3617.574	7.860721	-7068	7.33E-05	571	0.333333	0.007558	DFFA
4020.394	8.06942	-7068	7.34E-05	579	0.333333	0.007438	CDK2AP2
5580.706	6.440062	-7068	7.45E-05	830	0.333333	0.012286	ATP6V1A
14661.11	6.839351	-7068	7.71E-05	1293	0.333333	0.019189	BMP7
7279.514	8.37515	-7068	7.43E-05	754	0.333333	0.00996	TGM3
19205.34	7.237246	-7068	7.78E-05	1427	0.333333	0.020407	CRADD
13790.04	6.782217	-7068	7.68E-05	1261	0.333333	0.018251	VLDLR
8996.574	7.671614	-7068	7.52E-05	926	0.333333	0.013355	PDGFRL
7219.852	9.722353	-7068	7.40E-05	692	0.333333	0.009077	CCR9
8491.423	7.935038	-7068	7.50E-05	875	0.333333	0.012441	BHMT

4929.915	7.387491	-7068	7.41E-05	721	0.333333	0.010716	ACE
8741.735	6.90762	-7068	7.56E-05	1004	0.333333	0.015272	F2R
4848.203	6.858792	-7068	7.42E-05	736	0.333333	0.010922	ALG8
16261.22	6.442413	-7068	7.76E-05	1428	0.333333	0.020879	ANXA5
12139.75	8.098136	-7068	7.58E-05	1033	0.333333	0.01485	THY1
3328.651	6.307008	-7068	7.38E-05	650	0.333333	0.010396	IMPA1
12619.27	8.198343	-7068	7.59E-05	1044	0.333333	0.014541	ONECUT1
22967.51	6.685597	-7068	7.90E-05	1655	0.333333	0.024313	TXN
6926.882	6.624699	-7068	7.51E-05	921	0.333333	0.013878	IDE
7446.74	10.24659	-7068	7.38E-05	634	0.333333	0.008137	FGF6
6032.292	7.892689	-7068	7.39E-05	724	0.333333	0.008824	ENO3
15049.51	7.763918	-7068	7.63E-05	1166	0.333333	0.015617	JUP
3853.358	8.644975	-7068	7.33E-05	567	0.333333	0.008231	GNRH1
8989.95	7.211194	-7068	7.55E-05	980	0.333333	0.0142	VASP
6811.038	7.66703	-7068	7.44E-05	790	0.333333	0.010707	DSG1
4103.221	7.555787	-7068	7.36E-05	629	0.333333	0.008918	GSTM3
9742.701	6.495807	-7068	7.59E-05	1094	0.333333	0.016095	CALU
11009.22	7.233708	-7068	7.63E-05	1099	0.333333	0.016745	OSTF1
4198.347	8.575397	-7068	7.36E-05	605	0.333333	0.00933	PPP3CC
33216.13	6.511006	-7068	8.07E-05	1988	0.333333	0.028945	PBX1
2989.677	6.719275	-7068	7.34E-05	588	0.333333	0.008424	ACYP1
13336.13	6.515533	-7068	7.66E-05	1266	0.333333	0.018398	HIF1A
10698.69	6.55478	-7068	7.58E-05	1119	0.333333	0.014858	PSMB8
10217.49	6.936874	-7068	7.57E-05	1056	0.333333	0.014474	CD63
8096.128	6.89621	-7068	7.52E-05	940	0.333333	0.01382	GBAS
5006.011	5.362536	-7068	7.48E-05	896	0.333333	0.013688	BCLAF1
4353.479	7.548532	-7068	7.37E-05	649	0.25	0.009416	ENPP4
5473.8	7.342571	-7068	7.44E-05	751	0.333333	0.01102	UBE2G2
9022.724	6.672629	-7068	7.50E-05	977	0.333333	0.012854	NDUFS4
8359.914	9.545179	-7068	7.40E-05	716	0.333333	0.00852	AGER
5529.094	8.028284	-7068	7.40E-05	692	0.333333	0.009099	DIO1
9805.441	7.31767	-7068	7.58E-05	1022	0.333333	0.015206	EDEM1
5236.863	9.40087	-7068	7.35E-05	598	0.333333	0.007906	POU1F1
5669.167	8.055753	-7068	7.40E-05	702	0.333333	0.009443	EHMT2
6575.368	7.114133	-7068	7.44E-05	805	0.333333	0.010161	CPN1
6203.577	9.920775	-7068	7.36E-05	616	0.333333	0.008059	NINL
23073.54	6.709706	-7068	7.90E-05	1660	0.333333	0.024356	ADK
3562.672	7.787351	-7068	7.34E-05	573	0.333333	0.008139	PAF1
7933.534	6.965042	-7068	7.49E-05	913	0.333333	0.012086	COX7A1
6641.35	8.664521	-7068	7.41E-05	716	0.333333	0.009474	SLC13A2
10307.39	8.005975	-7068	7.55E-05	959	0.333333	0.013777	ADTRP

8727.451	8.490639	-7068	7.49E-05	847	0.333333	0.012475	MLLT11
3892.713	6.345818	-7068	7.41E-05	720	0.333333	0.011241	MAT2A
4229.612	7.170128	-7068	7.38E-05	675	0.333333	0.009863	ARHGEF2
5660.218	7.44055	-7068	7.44E-05	761	0.25	0.011722	CD200
9848.848	6.821221	-7068	7.57E-05	1051	0.333333	0.015004	SGCG
11567.79	9.090695	-7068	7.52E-05	909	0.333333	0.012013	SLC9A3R1
5180.954	6.293383	-7068	7.43E-05	789	0.333333	0.010564	DDOST
9103.4	6.439625	-7068	7.58E-05	1066	0.333333	0.015971	SPEN
10867.04	6.16782	-7068	7.63E-05	1160	0.333333	0.016086	FCER1G
6264.733	6.386694	-7068	7.50E-05	904	0.333333	0.01337	BTF3
8287.927	5.850909	-7068	7.58E-05	1075	0.333333	0.015422	SLC25A5
12626.82	6.492365	-7068	7.63E-05	1203	0.333333	0.016267	MYD88
9203.012	7.799372	-7068	7.52E-05	927	0.333333	0.013454	TSHR
10218.73	5.310747	-7068	7.61E-05	1268	0.333333	0.017023	RB1
6886.591	7.120404	-7068	7.44E-05	807	0.333333	0.010209	SERPINA6
8901.344	9.126983	-7068	7.47E-05	819	0.333333	0.011333	XRCC2
6160.401	7.320362	-7068	7.45E-05	779	0.333333	0.011617	TOPORS
8450.308	12.36128	-7068	7.34E-05	556	0.333333	0.006211	CHRND
24636.67	7.00618	-7068	7.92E-05	1656	0.333333	0.024401	RREB1
6281.935	6.280962	-7068	7.46E-05	875	0.333333	0.011924	DES
4234.937	8.880731	-7068	7.33E-05	552	0.333333	0.006783	GCK
7750.977	5.765732	-7068	7.53E-05	1033	0.333333	0.01419	HSPA5
7921.763	7.682273	-7068	7.45E-05	804	0.333333	0.009578	KRT13
3882.875	7.584722	-7068	7.34E-05	586	0.333333	0.007796	EIF2B4
9632.61	6.204744	-7068	7.61E-05	1124	0.333333	0.017238	DICER1
5999.998	7.895032	-7068	7.43E-05	748	0.333333	0.01072	GPKOW
4779.392	9.597654	-7068	7.33E-05	552	0.333333	0.006996	PMEL
9660.396	6.618619	-7068	7.54E-05	1034	0.333333	0.013483	ARG1
4481.985	8.319563	-7068	7.36E-05	614	0.333333	0.00826	GAA
6462.956	8.32688	-7068	7.42E-05	723	0.333333	0.009673	GPR143
3768.815	7.075786	-7068	7.36E-05	612	0.333333	0.009061	GABPA
8842.958	7.998832	-7068	7.49E-05	893	0.333333	0.012956	ID3
11352.45	6.986192	-7068	7.63E-05	1135	0.333333	0.016947	VAV2
13855.62	5.649014	-7068	7.73E-05	1416	0.333333	0.020154	SF3B1
10255.87	6.580109	-7068	7.60E-05	1099	0.333333	0.015668	CSF1R
11854.93	10.70658	-7068	7.50E-05	872	0.333333	0.012753	ASH2L
6569.218	7.02727	-7068	7.48E-05	852	0.333333	0.012596	PIK3C2B
22392.48	7.28767	-7068	7.87E-05	1552	0.333333	0.023829	CUX1
6616.2	8.947921	-7068	7.40E-05	671	0.333333	0.008704	KRT76
4101.478	7.249616	-7068	7.38E-05	666	0.333333	0.009706	BCAT2
26259.47	6.925699	-7068	7.96E-05	1710	0.333333	0.0254	DCLK1

20300.54	9.252657	-7068	7.68E-05	1234	0.333333	0.018423	SDCBP
7754.621	5.672309	-7068	7.55E-05	1044	0.333333	0.014989	CNN1
2666.983	6.840989	-7068	7.32E-05	543	0.333333	0.0081	WDR43
15772.42	5.983963	-7068	7.80E-05	1486	0.333333	0.022656	PPP2R5C
9377.171	8.458964	-7068	7.50E-05	880	0.333333	0.012075	MVP
6952.303	3.415585	-7068	7.55E-05	1268	0.333333	0.014167	RFC4
4438.954	9.841634	-7068	7.32E-05	524	0.333333	0.007061	RRH
9589.413	9.024582	-7068	7.45E-05	792	0.333333	0.010816	NDUFB5
4664.518	7.060462	-7068	7.41E-05	722	0.333333	0.01117	IFRD1
12140.34	7.405495	-7068	7.61E-05	1099	0.333333	0.015738	CA2
4120.123	8.361183	-7068	7.32E-05	556	0.333333	0.007246	TFF1
12838.78	7.093352	-7068	7.67E-05	1197	0.333333	0.018154	CKAP4
8652.973	5.59993	-7068	7.57E-05	1129	0.333333	0.01549	JAK1
5882.031	5.77339	-7068	7.50E-05	930	0.333333	0.014098	G3BP1
6731.661	8.55738	-7068	7.42E-05	742	0.333333	0.011194	RSRP1
15470.03	7.248227	-7068	7.73E-05	1288	0.333333	0.019619	GPD1L
7680.276	8.301889	-7068	7.46E-05	807	0.333333	0.011398	ZNF592
6323.297	6.800548	-7068	7.45E-05	836	0.333333	0.011292	PLA2G2A
6918.254	6.804552	-7068	7.44E-05	848	0.333333	0.010882	B2M
13696.07	7.529843	-7068	7.63E-05	1144	0.333333	0.015866	IRF6
5828.349	7.140718	-7068	7.44E-05	792	0.333333	0.011401	CCNT1
5022.498	7.308607	-7068	7.41E-05	709	0.333333	0.010165	TRAF3IP3
3111.708	8.261743	-7068	7.32E-05	531	0.333333	0.008035	PCNX4
6674.021	7.144816	-7068	7.49E-05	850	0.333333	0.012537	TNPO3
4263.601	8.544215	-7068	7.35E-05	579	0.333333	0.007575	NDUFAF3
6851.505	9.070104	-7068	7.43E-05	722	0.333333	0.010159	LETMD1
7106.96	7.648966	-7068	7.46E-05	816	0.333333	0.011479	ATP7B
4312.099	7.589717	-7068	7.37E-05	634	0.333333	0.008797	DCTN3
6820.158	5.714223	-7068	7.51E-05	986	0.333333	0.013506	PRKCB
10689.17	8.434129	-7068	7.52E-05	904	0.333333	0.011406	TM4SF5
10432.91	9.601557	-7068	7.49E-05	846	0.333333	0.012424	KCNH1
7893.24	6.016316	-7068	7.50E-05	979	0.333333	0.011663	ITIH1
8700.46	7.320065	-7068	7.54E-05	968	0.333333	0.014025	PNP
10152.53	7.620229	-7068	7.56E-05	989	0.333333	0.014682	POGZ
9881.116	7.177896	-7068	7.57E-05	1023	0.333333	0.014839	COX7A2
6286.656	12.08444	-7068	7.32E-05	498	0.333333	0.005439	GNRH2
12679.02	11.75984	-7068	7.43E-05	750	0.333333	0.009186	CELA2B
22760.23	7.833242	-7068	7.82E-05	1460	0.333333	0.021555	OCA2
14296.74	6.564987	-7068	7.72E-05	1318	0.333333	0.019851	PIP5K1B
15493.2	8.185633	-7068	7.66E-05	1153	0.333333	0.016674	CALB2
5899.041	9.787211	-7068	7.37E-05	624	0.333333	0.008515	SCRIB

3532.07	6.023004	-7068	7.35E-05	658	0.333333	0.008305	PRPF6
4423.167	5.990098	-7068	7.41E-05	758	0.333333	0.010629	RPN1
7071.285	6.22304	-7068	7.50E-05	936	0.333333	0.01266	ICAM1
9277.09	8.354131	-7068	7.50E-05	866	0.333333	0.012368	IGHMBP2
8340.458	5.260617	-7068	7.61E-05	1165	0.333333	0.016814	NCL
6861.523	6.663815	-7068	7.45E-05	852	0.333333	0.010121	F10
15199.45	4.594779	-7068	7.77E-05	1652	0.333333	0.020793	PCNA
19704.05	7.722731	-7068	7.77E-05	1372	0.333333	0.020544	FSTL4
3399.411	8.15433	-7068	7.33E-05	553	0.333333	0.007856	PPP1R1A
8611.223	7.611819	-7068	7.53E-05	928	0.333333	0.013817	UBAC1
4791.677	7.453982	-7068	7.42E-05	707	0.333333	0.011187	ARFGEF1
3730.667	8.716901	-7068	7.33E-05	547	0.333333	0.007622	TRH
8013.951	7.805015	-7068	7.50E-05	876	0.333333	0.012728	CBR1
13450.94	7.291101	-7068	7.66E-05	1202	0.333333	0.018435	UGP2
5524.29	6.018399	-7068	7.47E-05	870	0.333333	0.012957	PPP1CB
11346.59	7.307852	-7068	7.61E-05	1081	0.333333	0.015726	HYOU1
6944.043	6.919954	-7068	7.49E-05	887	0.333333	0.012995	XRCC4
7006.09	5.651162	-7068	7.53E-05	1028	0.333333	0.014741	INSR
14845.19	6.65011	-7068	7.72E-05	1328	0.333333	0.019356	CD55
5561.877	7.012281	-7068	7.44E-05	781	0.333333	0.010991	BAK1
9059.089	6.498524	-7068	7.56E-05	1045	0.333333	0.015017	TRAF3
6434.048	7.460354	-7068	7.46E-05	804	0.333333	0.011658	PRDX4
8336.202	7.464824	-7068	7.50E-05	924	0.333333	0.012945	VHL
5998.322	10.02922	-7068	7.36E-05	605	0.333333	0.007988	PRKACG
4533.583	7.803848	-7068	7.38E-05	652	0.333333	0.009134	CIRBP
8389.464	8.625763	-7068	7.48E-05	828	0.333333	0.012015	COL9A2
6203.883	7.585586	-7068	7.44E-05	764	0.333333	0.010577	CNGA1
5995.175	8.08257	-7068	7.39E-05	696	0.333333	0.008808	CSF3
16796.27	7.064782	-7068	7.75E-05	1358	0.333333	0.020281	MAP2K5
7673.026	7.900889	-7068	7.46E-05	816	0.333333	0.010859	NPY
6360.559	7.167733	-7068	7.45E-05	815	0.333333	0.010849	ALDOA
6684.071	6.231415	-7068	7.49E-05	919	0.333333	0.013513	PAM
12483.15	5.747609	-7068	7.67E-05	1327	0.333333	0.01803	HMGB2
4971.418	6.713534	-7068	7.42E-05	760	0.333333	0.010744	MAP3K1
5635.721	6.992514	-7068	7.44E-05	765	0.333333	0.010322	TRAP1
26172.71	6.307555	-7068	7.99E-05	1831	0.333333	0.027095	MAF
7929.56	6.723603	-7068	7.51E-05	954	0.333333	0.013518	CASP7
8911.076	7.518395	-7068	7.53E-05	937	0.333333	0.013215	ATP6V0C
17000.69	6.513721	-7068	7.79E-05	1446	0.333333	0.021762	PLPP3
7838.215	10.86592	-7068	7.38E-05	645	0.333333	0.007918	NKX2-5
6076.577	7.01686	-7068	7.46E-05	798	0.333333	0.011972	ZMPSTE24

11401.78	6.177304	-7068	7.66E-05	1243	0.333333	0.018379	CCND2
6282.917	8.457058	-7068	7.42E-05	720	0.333333	0.010056	TNK2
9576.201	8.551935	-7068	7.49E-05	881	0.333333	0.01143	SLC2A4
10907.77	7.149929	-7068	7.58E-05	1065	0.333333	0.015237	SCN2A
9970.59	7.717567	-7068	7.55E-05	978	0.333333	0.013868	MMP15
8314.862	7.130785	-7068	7.51E-05	936	0.333333	0.013291	PRG4
14198.8	8.122316	-7068	7.64E-05	1140	0.333333	0.017023	CLASP1
6460.337	7.85283	-7068	7.43E-05	749	0.333333	0.009991	MALL
6184.988	7.664881	-7068	7.44E-05	762	0.333333	0.010603	PARK7
7220.986	7.536109	-7068	7.46E-05	839	0.333333	0.012082	MAX
9408.871	6.646657	-7068	7.60E-05	1073	0.333333	0.016407	PON2
6526.526	8.239633	-7068	7.42E-05	731	0.333333	0.009771	FGF4
6072.002	7.459147	-7068	7.44E-05	759	0.333333	0.010596	SNX17
4714.473	6.415609	-7068	7.44E-05	783	0.333333	0.012303	PPP3CB
3361.549	7.864667	-7068	7.31E-05	543	0.333333	0.006864	VAMP2
9615.63	9.544884	-7068	7.46E-05	797	0.333333	0.010404	DNASE1
8235.45	8.280296	-7068	7.49E-05	853	0.25	0.012276	SPOCK2
5836.274	6.282067	-7068	7.46E-05	853	0.333333	0.01189	EIF4B
9130.752	8.065644	-7068	7.46E-05	851	0.333333	0.0105	IKBKG
10414.79	7.564429	-7068	7.57E-05	1009	0.333333	0.014623	CD226
6110.724	9.533122	-7068	7.36E-05	633	0.333333	0.008527	MYF5
4147.152	7.588951	-7068	7.39E-05	653	0.333333	0.009993	PPT1
8256.858	7.433593	-7068	7.52E-05	920	0.333333	0.013728	ABCC5
11314.14	7.282235	-7068	7.59E-05	1074	0.333333	0.014998	GRHPR
9309.798	8.019857	-7068	7.51E-05	912	0.333333	0.012439	ZRSR2
8746.224	8.230217	-7068	7.49E-05	852	0.333333	0.012165	KEAP1
11614.72	6.064407	-7068	7.66E-05	1253	0.333333	0.01786	PSMA1
5437.15	9.383351	-7068	7.36E-05	606	0.333333	0.008017	S1PR2
8843.245	6.324574	-7068	7.53E-05	1026	0.333333	0.013674	UQCRC2
2481.004	6.9875	-7068	7.29E-05	504	0.333333	0.007003	EXOSC10
10587.64	6.934756	-7068	7.59E-05	1082	0.333333	0.015242	MAOA
7630.685	8.359119	-7068	7.46E-05	803	0.333333	0.011056	KCNN4
10311.66	7.345295	-7068	7.57E-05	1027	0.333333	0.015671	UGCG
8184.32	7.296276	-7068	7.50E-05	910	0.333333	0.012606	PPP5C
6004.547	6.108938	-7068	7.46E-05	873	0.333333	0.011824	PDHA1
7582.693	6.495112	-7068	7.52E-05	947	0.333333	0.01293	EIF4G1
8964.95	7.314691	-7068	7.52E-05	942	0.333333	0.012427	FBP1
17131.55	7.74045	-7068	7.72E-05	1276	0.333333	0.018422	SELENBP1
14723.66	7.139825	-7068	7.69E-05	1255	0.333333	0.018524	SLC12A2
6738.217	8.598541	-7068	7.43E-05	742	0.333333	0.010272	CKB
6494.58	7.311076	-7068	7.43E-05	794	0.333333	0.01013	RELB

10943.02	7.408495	-7068	7.59E-05	1067	0.333333	0.015402	DDX6
10007.66	8.838748	-7068	7.49E-05	869	0.333333	0.012054	SIM1
10996.03	7.24522	-7068	7.60E-05	1096	0.333333	0.016079	NF2
21059.06	31.47015	-7068	7.31E-05	495	0.333333	0.005714	OR10H3
2795.992	6.397064	-7068	7.33E-05	582	0.333333	0.008852	MED13
8523.119	6.90224	-7068	7.54E-05	982	0.333333	0.014919	TFCP2
5152.006	6.956036	-7068	7.43E-05	761	0.333333	0.011447	ZFP36L2
6816.563	8.683803	-7068	7.40E-05	712	0.333333	0.008719	GPT
13371.76	6.665054	-7068	7.67E-05	1256	0.333333	0.017992	IL1RAP
5680.402	8.337018	-7068	7.40E-05	692	0.333333	0.00921	XPC
13052.85	6.125293	-7068	7.68E-05	1284	0.333333	0.017981	F13A1
7183.803	6.816572	-7068	7.51E-05	920	0.333333	0.013551	GSR
8423.142	7.337927	-7068	7.54E-05	943	0.333333	0.014468	CTNNAL1
10297.15	7.204487	-7068	7.57E-05	1029	0.333333	0.014663	MMP7
11455.04	9.201863	-7068	7.51E-05	900	0.333333	0.01196	HSPB2
3615.616	7.398856	-7068	7.36E-05	612	0.333333	0.009471	MTMR4
9021.495	6.872747	-7068	7.56E-05	1012	0.333333	0.014788	SDC2
8978.864	6.353034	-7068	7.54E-05	1027	0.333333	0.013591	LSM2
9721.92	10.30017	-7068	7.45E-05	776	0.333333	0.010993	HOXA1
10560.52	7.905075	-7068	7.56E-05	988	0.333333	0.01387	LTBR
7734.355	5.474728	-7068	7.53E-05	1070	0.333333	0.014213	MAPK8
11019.62	7.049978	-7068	7.60E-05	1098	0.333333	0.015452	EPHA2
8395.189	7.403333	-7068	7.51E-05	925	0.333333	0.012917	IDH2
6886.764	7.369043	-7068	7.47E-05	829	0.333333	0.011674	YIF1A
8349.337	4.857092	-7068	7.57E-05	1167	0.333333	0.01474	EZH2
16854.3	6.251023	-7068	7.77E-05	1459	0.333333	0.020866	GHR
10070.56	7.098945	-7068	7.59E-05	1043	0.333333	0.015736	PTPRR
7958.944	7.410466	-7068	7.49E-05	887	0.333333	0.012567	TNFRSF25
8136.823	6.090092	-7068	7.59E-05	1056	0.333333	0.016586	SPAG9
5601.52	6.304139	-7068	7.47E-05	849	0.333333	0.012917	PRKCI
11645.35	8.224474	-7068	7.56E-05	990	0.333333	0.014017	SPINK2
9435.395	6.557266	-7068	7.61E-05	1084	0.333333	0.016791	UBE2H
7467.376	7.624732	-7068	7.47E-05	834	0.333333	0.011416	ALDH3A1
12464.09	6.503779	-7068	7.64E-05	1197	0.333333	0.016032	FOXM1
4039.269	9.257763	-7068	7.32E-05	524	0.333333	0.00665	SMG5
5008.265	6.322388	-7068	7.43E-05	780	0.333333	0.011415	RAB11A
10520.56	6.606306	-7068	7.62E-05	1122	0.333333	0.016792	MAPK10
6140.574	8.742347	-7068	7.40E-05	670	0.333333	0.009249	ACP2
6974.116	7.766639	-7068	7.46E-05	805	0.333333	0.01161	PDE2A
8207.015	6.019703	-7068	7.56E-05	1072	0.333333	0.01542	MAP2K1
7340.068	7.156666	-7068	7.49E-05	889	0.333333	0.01299	AKT2

7805.703	6.855738	-7068	7.49E-05	934	0.333333	0.012585	PLCG1
5906.382	5.320439	-7068	7.50E-05	948	0.333333	0.013233	PSMD6
15771.94	6.885304	-7068	7.68E-05	1299	0.333333	0.017529	APOB
12766.06	7.063858	-7068	7.66E-05	1195	0.333333	0.01793	KRT10
5519.144	7.745759	-7068	7.42E-05	726	0.333333	0.010976	NAP1L3
14387.65	5.993038	-7068	7.73E-05	1392	0.333333	0.020324	PALLD
12581.74	6.554642	-7068	7.68E-05	1249	0.333333	0.019009	SEC31A
7432.091	8.277412	-7068	7.45E-05	787	0.333333	0.011739	FEM1B
5921.505	7.418658	-7068	7.44E-05	765	0.333333	0.010486	GPI
5424.838	7.774419	-7068	7.39E-05	705	0.333333	0.009875	NCOA6
8097.155	7.073214	-7068	7.53E-05	944	0.333333	0.014108	CENPC
7768.711	6.477108	-7068	7.53E-05	984	0.333333	0.014802	PDIA3
8223.59	5.304037	-7068	7.56E-05	1134	0.333333	0.014575	SHC1
6700.779	6.253281	-7068	7.46E-05	870	0.333333	0.011922	GNL2
16371.34	6.372408	-7068	7.76E-05	1417	0.333333	0.020826	MAP7
6241.114	6.976897	-7068	7.45E-05	818	0.333333	0.011407	CEACAM1
14104.62	7.859124	-7068	7.62E-05	1137	0.333333	0.015728	SFN
12491.97	6.872479	-7068	7.67E-05	1206	0.333333	0.018424	CTBP2
6705.126	8.018425	-7068	7.37E-05	687	0.333333	0.008585	ZNF529
8332.083	8.042578	-7068	7.49E-05	860	0.333333	0.012086	SCN8A
11019.43	8.063664	-7068	7.55E-05	978	0.333333	0.013975	EPHX2
12021.96	4.967172	-7068	7.66E-05	1406	0.333333	0.018222	CDC6
9009.301	7.110929	-7068	7.54E-05	975	0.333333	0.014249	SRPX
7778.933	6.499592	-7068	7.50E-05	967	0.333333	0.013218	SQSTM1
8025.559	8.387374	-7068	7.47E-05	803	0.333333	0.011034	CFP
14232.23	7.083838	-7068	7.68E-05	1239	0.333333	0.017272	CAPN1
6975.807	4.510004	-7068	7.55E-05	1143	0.333333	0.01515	DHX9
8198.003	6.685604	-7068	7.51E-05	958	0.333333	0.01275	IGFBP1
12181.87	7.08916	-7068	7.60E-05	1121	0.333333	0.015133	C4BPB
7762.417	5.42466	-7068	7.57E-05	1089	0.333333	0.015622	RAN
7502.563	6.587976	-7068	7.53E-05	951	0.333333	0.014827	MAP4K5
16035.86	5.288672	-7068	7.77E-05	1568	0.333333	0.020316	CKS2
466.911	12.08988	-7068	7.07E-05	136	0.25	0.001626	FAM161A
6699.5	7.71584	-7068	7.46E-05	793	0.333333	0.011297	KCNA5
2364.342	8.245875	-7068	7.27E-05	446	0.333333	0.006033	STARD3
15037.97	5.75458	-7068	7.75E-05	1456	0.333333	0.020694	COL15A1
6292.13	5.842773	-7068	7.50E-05	939	0.333333	0.013206	EWSR1
11848.83	7.161702	-7068	7.63E-05	1129	0.333333	0.016379	ECE1
16450.1	7.324354	-7068	7.70E-05	1297	0.333333	0.017651	ASS1
7714.889	6.956079	-7068	7.49E-05	910	0.333333	0.012356	LAMA5
7724.659	7.949672	-7068	7.44E-05	805	0.333333	0.010206	CCL7

3324.538	7.712331	-7068	7.33E-05	574	0.333333	0.008182	UROS
12137.12	8.474937	-7068	7.56E-05	971	0.333333	0.012614	PRSS8
7198.334	6.742629	-7068	7.48E-05	895	0.333333	0.012148	CRYAB
17730.22	6.880083	-7068	7.80E-05	1440	0.333333	0.021737	SLC20A1
9065.97	6.585056	-7068	7.57E-05	1046	0.333333	0.015441	PTPRF
6987.706	7.960018	-7068	7.43E-05	782	0.333333	0.010863	CAV3
2738.195	6.453774	-7068	7.30E-05	553	0.333333	0.00697	IRF7
4872.642	7.328986	-7068	7.42E-05	718	0.333333	0.010897	TAF1B
5851.388	8.507277	-7068	7.39E-05	665	0.333333	0.008163	SHBG
7731.8	7.821543	-7068	7.50E-05	852	0.333333	0.012837	PPP1R7
15719.71	6.495584	-7068	7.71E-05	1338	0.333333	0.018376	LRRC32
17225.42	7.094137	-7068	7.77E-05	1376	0.333333	0.020774	SYN3
8372.951	8.128599	-7068	7.47E-05	823	0.333333	0.010957	GPX4
30638.09	5.76643	-7068	8.03E-05	2054	0.333333	0.027375	RFC3
11521.55	7.441495	-7068	7.60E-05	1063	0.333333	0.015832	BPGM
5536.041	7.253287	-7068	7.43E-05	763	0.333333	0.011168	FECH
4722.424	8.032999	-7068	7.36E-05	631	0.333333	0.008216	FCMR
8466.241	6.787518	-7068	7.52E-05	973	0.333333	0.013759	ALDH3A2
9073.404	5.661957	-7068	7.57E-05	1098	0.333333	0.014499	AHSA1
8664.628	9.124997	-7068	7.46E-05	790	0.333333	0.011059	POU6F2
6914.374	7.609719	-7068	7.47E-05	830	0.333333	0.012613	SH3YL1
6186.624	9.389635	-7068	7.31E-05	598	0.333333	0.007105	ZNF415
13553.13	6.503508	-7068	7.71E-05	1295	0.333333	0.019222	TGFBI
10156.16	6.663814	-7068	7.59E-05	1102	0.333333	0.015585	PTPN1
4986.157	7.536487	-7068	7.40E-05	674	0.333333	0.009137	IFRD2
8374.136	6.196627	-7068	7.55E-05	1025	0.333333	0.013955	BGN
3155.744	9.174243	-7068	7.30E-05	466	0.333333	0.006151	KRT12
4504.587	8.338138	-7068	7.37E-05	617	0.333333	0.008373	NME4
7249.383	7.079525	-7068	7.51E-05	893	0.333333	0.013697	ZFYVE26
20738.73	9.914919	-7068	7.64E-05	1153	0.333333	0.017713	NDN
5567.683	7.069268	-7068	7.44E-05	779	0.333333	0.01148	MLC1
10421.16	7.136968	-7068	7.54E-05	1011	0.333333	0.012779	F11
30372.31	6.423645	-7068	8.09E-05	1946	0.333333	0.029331	PLXDC2
5639.123	6.663969	-7068	7.45E-05	808	0.333333	0.011524	CDO1
8680.507	4.395018	-7068	7.58E-05	1247	0.333333	0.015183	SNRPE
3653.205	10.30508	-7068	7.29E-05	461	0.333333	0.005796	SNPH
3175.063	9.324959	-7068	7.30E-05	482	0.333333	0.006519	RNF113A
5847.909	7.320735	-7068	7.46E-05	791	0.333333	0.012078	ARHGEF6
5800.302	7.502193	-7068	7.44E-05	767	0.333333	0.0115	RGS13
8783.634	6.995919	-7068	7.53E-05	964	0.333333	0.012736	POLD1
19801.11	7.439159	-7068	7.78E-05	1415	0.333333	0.020196	RBM15B

8440.912	9.052503	-7068	7.47E-05	803	0.333333	0.011232	LSR
7877.477	6.681068	-7068	7.51E-05	940	0.333333	0.012922	FCGR2B
13020.56	6.671176	-7068	7.69E-05	1254	0.333333	0.018525	OGDH
2866.826	9.458335	-7068	7.27E-05	442	0.333333	0.00604	NPHP1
7003.035	5.47463	-7068	7.52E-05	1035	0.333333	0.014116	PXN
5337.607	8.190264	-7068	7.39E-05	662	0.333333	0.008707	ECI1
8497.736	9.223638	-7068	7.45E-05	774	0.333333	0.010497	BCS1L
10130.72	7.570418	-7068	7.57E-05	1005	0.333333	0.015136	ZMYND8
13132.46	6.26194	-7068	7.71E-05	1307	0.333333	0.019426	CD59
12632.69	6.650836	-7068	7.67E-05	1228	0.333333	0.018367	SGCD
8519.043	6.995493	-7068	7.51E-05	951	0.333333	0.012839	TTR
6174.814	4.730784	-7068	7.54E-05	1071	0.333333	0.014868	HNRNPC
10668.03	7.062719	-7068	7.60E-05	1086	0.333333	0.016038	TOX
15124.5	6.766006	-7068	7.70E-05	1318	0.333333	0.018813	TGFBR1
11580.42	5.544376	-7068	7.65E-05	1283	0.333333	0.017264	PDGFRB
6107.496	7.43572	-7068	7.46E-05	797	0.333333	0.012103	DGUOK
6434.104	9.316033	-7068	7.40E-05	686	0.333333	0.009555	CLSTN3
5548.788	8.764412	-7068	7.40E-05	665	0.333333	0.009197	TNFRSF10D
6837.966	5.778185	-7068	7.50E-05	959	0.333333	0.013061	SREK1
5399.646	6.259245	-7068	7.47E-05	843	0.333333	0.0133	TLK1
17941.22	6.711184	-7068	7.81E-05	1452	0.333333	0.021924	ABLIM1
8281.649	6.76993	-7068	7.56E-05	992	0.333333	0.015708	STK38L
9202.59	6.584085	-7068	7.58E-05	1054	0.333333	0.015536	DDX10
13123.16	7.010911	-7068	7.68E-05	1217	0.333333	0.017879	WARS
9179.719	7.275526	-7068	7.56E-05	986	0.333333	0.014976	ITM2B
13994.06	6.279474	-7068	7.70E-05	1325	0.333333	0.018875	NFIL3
17269.99	4.647724	-7068	7.80E-05	1740	0.333333	0.021559	CHEK1
4439.643	6.253463	-7068	7.37E-05	729	0.333333	0.008813	POLR2A
7577.492	7.472245	-7068	7.50E-05	868	0.333333	0.01288	HAPLN1
12862.43	6.709143	-7068	7.67E-05	1231	0.333333	0.018176	NCK2
7081.291	6.875908	-7068	7.47E-05	857	0.333333	0.011495	LST1
6497.908	6.668905	-7068	7.48E-05	883	0.333333	0.013148	BCR
4842.766	7.415021	-7068	7.40E-05	696	0.333333	0.010395	NEK1
3638.199	5.337847	-7068	7.41E-05	743	0.333333	0.010707	PWP1
29332.95	6.596454	-7068	8.02E-05	1865	0.333333	0.027105	SPOCK1
3693.75	7.337961	-7068	7.34E-05	588	0.333333	0.007677	PMPCA
7932.454	8.447444	-7068	7.47E-05	812	0.333333	0.01142	SPTBN2
5543.587	9.416216	-7068	7.36E-05	618	0.333333	0.00781	UNC119
5461.694	7.870002	-7068	7.41E-05	705	0.333333	0.010232	PHF14
5159.95	7.433736	-7068	7.39E-05	700	0.333333	0.009322	WFDC2
13787.38	5.830641	-7068	7.70E-05	1384	0.333333	0.019275	FAS

6923.586	6.591885	-7068	7.52E-05	930	0.333333	0.014171	PPP2R5A
4647.958	8.289868	-7068	7.36E-05	626	0.333333	0.008389	ADGRE1
11496.64	6.714185	-7068	7.61E-05	1146	0.333333	0.016707	ARNT
12803.45	7.249405	-7068	7.63E-05	1150	0.333333	0.016501	KCNJ3
16465.11	7.338599	-7068	7.73E-05	1318	0.333333	0.019482	PIM1
7015.856	6.251832	-7068	7.52E-05	955	0.333333	0.01422	FGF2
10491.86	7.720114	-7068	7.58E-05	1011	0.333333	0.015752	CCDC28A
4578.599	9.061288	-7068	7.35E-05	585	0.333333	0.007877	ERN1
7959.947	5.144171	-7068	7.54E-05	1097	0.333333	0.014083	SNRPF
12540.01	6.750033	-7068	7.68E-05	1223	0.333333	0.018937	ATXN10
8560.926	6.655785	-7068	7.56E-05	1010	0.333333	0.015092	HEXB
9528.386	5.620303	-7068	7.59E-05	1165	0.333333	0.015912	TEK
10850.92	5.784225	-7068	7.65E-05	1238	0.333333	0.017663	HSPA9
4055.989	5.388096	-7068	7.40E-05	775	0.333333	0.010901	RAB5A
4975.324	7.36531	-7068	7.41E-05	719	0.333333	0.011179	TNRC6B
8903.123	7.87886	-7068	7.50E-05	895	0.333333	0.012293	RHOD
8027.582	6.82781	-7068	7.55E-05	974	0.333333	0.014754	RASA2
12177.56	6.447775	-7068	7.61E-05	1177	0.333333	0.01479	C4BPA
10329.02	5.772866	-7068	7.64E-05	1223	0.333333	0.017837	SF3A1
5636.295	6.573503	-7068	7.43E-05	781	0.333333	0.010314	HLA-A
8142.066	6.989087	-7068	7.54E-05	961	0.333333	0.014676	PFN2
10333.73	6.818899	-7068	7.56E-05	1023	0.333333	0.012547	F7
8247.131	6.729636	-7068	7.53E-05	975	0.333333	0.013838	BCL2A1
4799.222	8.01341	-7068	7.40E-05	675	0.333333	0.009802	PPP1R11
6121.787	8.121814	-7068	7.42E-05	727	0.333333	0.00959	ZNHIT1
4831.869	7.099661	-7068	7.42E-05	731	0.333333	0.011108	TAX1BP1
6503.645	8.975927	-7068	7.42E-05	705	0.333333	0.009676	NPPA
9993.106	7.433052	-7068	7.57E-05	1011	0.333333	0.015232	SNAI2
4227.746	8.185595	-7068	7.34E-05	598	0.333333	0.008465	COL4A4
9694.556	9.247078	-7068	7.45E-05	794	0.333333	0.01066	SLC22A2
5142.364	8.290142	-7068	7.36E-05	638	0.333333	0.007744	FCER2
7161.209	6.972031	-7068	7.50E-05	897	0.333333	0.01307	SLC31A1
5348.057	7.164299	-7068	7.43E-05	743	0.333333	0.010173	ARHGDIA
9513.284	7.127812	-7068	7.57E-05	1027	0.333333	0.015267	RRBP1
28615.21	6.422098	-7068	8.05E-05	1888	0.333333	0.028387	ZEB2
9498.937	5.855608	-7068	7.63E-05	1164	0.333333	0.017455	HPRT1
13128.97	6.961372	-7068	7.66E-05	1221	0.333333	0.017656	PPIF
10104.07	7.031924	-7068	7.58E-05	1057	0.333333	0.015219	XBP1
16944.85	6.935032	-7068	7.77E-05	1385	0.333333	0.021229	IQSEC1
9047.587	7.445986	-7068	7.50E-05	921	0.333333	0.011496	HMGCL
8862.518	9.227013	-7068	7.48E-05	841	0.333333	0.01279	UBE4B

6523.349	8.186489	-7068	7.42E-05	737	0.333333	0.009936	GNB3
18823.96	6.939206	-7068	7.80E-05	1450	0.333333	0.022239	NPAS3
7820.049	6.277016	-7068	7.55E-05	1003	0.333333	0.015066	ITGA8
11277.66	7.307075	-7068	7.60E-05	1084	0.333333	0.015523	BAD
6473.581	8.102868	-7068	7.43E-05	737	0.333333	0.009638	NUCB1
4417.924	7.588191	-7068	7.38E-05	661	0.333333	0.009456	DCTN2
4736.942	6.43997	-7068	7.42E-05	745	0.333333	0.01035	KCNMB1
9691.268	7.376778	-7068	7.55E-05	997	0.333333	0.014843	EGR3
6063.488	6.523353	-7068	7.46E-05	866	0.333333	0.01199	ERBB3
11237.42	6.24188	-7068	7.61E-05	1170	0.333333	0.01566	MMP9
7183.61	5.642954	-7068	7.51E-05	1008	0.333333	0.012998	BRCA2
5852.829	6.845454	-7068	7.45E-05	806	0.333333	0.011756	OPTN
5193.396	7.148786	-7068	7.42E-05	741	0.333333	0.01074	ALDH9A1
6640.18	7.83043	-7068	7.45E-05	782	0.333333	0.011138	NRGN
4615.659	6.841262	-7068	7.42E-05	733	0.333333	0.011471	PRRC2C
9400.092	7.268154	-7068	7.54E-05	975	0.333333	0.013538	SLC25A11
5139.019	7.153836	-7068	7.43E-05	745	0.333333	0.011233	MRPS27
8333.001	11.11537	-7068	7.38E-05	626	0.333333	0.007526	INS
6114.742	8.451667	-7068	7.42E-05	705	0.333333	0.009731	TELO2
10984.9	7.492609	-7068	7.55E-05	1012	0.333333	0.013114	CD14
6044.741	5.917435	-7068	7.49E-05	919	0.333333	0.013486	RAD23B
13230.38	7.64059	-7068	7.57E-05	1085	0.333333	0.014163	CYP17A1
12915.83	7.049574	-7068	7.65E-05	1193	0.333333	0.017495	EEF1A2
9578.909	6.459715	-7068	7.60E-05	1092	0.333333	0.016497	GATA6
2614.834	7.059338	-7068	7.30E-05	510	0.333333	0.006391	CCL21
6488.769	6.983777	-7068	7.45E-05	826	0.25	0.011436	IL18R1
8164.578	6.136687	-7068	7.58E-05	1049	0.333333	0.015991	UBE2E1
6934.279	6.280188	-7068	7.50E-05	935	0.333333	0.013506	PLAT
7903.988	4.315609	-7068	7.56E-05	1206	0.333333	0.014037	MCM7
7296.117	6.148939	-7068	7.47E-05	916	0.333333	0.010893	HRG
5788.675	7.592157	-7068	7.41E-05	739	0.333333	0.009803	FOLR3
3663.702	7.424834	-7068	7.35E-05	608	0.333333	0.008584	AKAP8
14096.68	7.776646	-7068	7.65E-05	1156	0.333333	0.016819	ABCG2
8288.264	7.257593	-7068	7.52E-05	926	0.333333	0.013953	TDRD3
6417.254	8.458878	-7068	7.41E-05	727	0.333333	0.010145	SH2D2A
8245.093	6.150431	-7068	7.48E-05	904	0.333333	0.011226	CYC1
9104.851	5.786229	-7068	7.58E-05	1109	0.333333	0.014846	PSMC3
10552.71	6.106438	-7068	7.66E-05	1201	0.333333	0.018541	MYLK
3983.031	8.441581	-7068	7.35E-05	576	0.333333	0.008539	ERC2
7693.519	7.832909	-7068	7.48E-05	855	0.333333	0.012598	REPS1
3017.316	6.659516	-7068	7.35E-05	607	0.333333	0.009502	EP400

4443.593	9.164621	-7068	7.33E-05	548	0.333333	0.007191	PDC
12445.81	6.260295	-7068	7.66E-05	1248	0.333333	0.017786	PTGS2
3573.257	7.87669	-7068	7.34E-05	569	0.333333	0.007782	SLC39A7
2657.883	7.60712	-7068	7.31E-05	512	0.333333	0.007744	PRPSAP1
15331.1	6.429003	-7068	7.77E-05	1389	0.333333	0.021348	FAM13A
21581.46	5.938447	-7068	7.87E-05	1680	0.333333	0.02317	FBN1
4819.84	8.263992	-7068	7.37E-05	641	0.333333	0.008995	IFIH1
8812.943	7.772373	-7068	7.54E-05	927	0.333333	0.014459	PGRMC1
21325.06	6.412346	-7068	7.87E-05	1629	0.333333	0.023605	BCL6
15988.26	5.903856	-7068	7.81E-05	1509	0.333333	0.022362	DUSP3
14037.36	6.432487	-7068	7.68E-05	1287	0.333333	0.017947	ALOX5AP
5920.717	6.15138	-7068	7.49E-05	887	0.333333	0.013557	YTHDC2
19024.4	5.574835	-7068	7.88E-05	1689	0.333333	0.024246	CXCL12
5099.28	10.74864	-7068	7.32E-05	518	0.333333	0.006812	GUCY2D
3665.781	9.217444	-7068	7.32E-05	518	0.333333	0.007273	PTGES
4851.461	8.156912	-7068	7.38E-05	650	0.333333	0.009388	AMELX
12923.89	6.826461	-7068	7.68E-05	1225	0.333333	0.018404	BNIP3
8594.084	6.895997	-7068	7.57E-05	1008	0.333333	0.015712	SKI
15034.39	6.770177	-7068	7.72E-05	1313	0.333333	0.019592	ITPR1
9552.257	5.617981	-7068	7.63E-05	1196	0.333333	0.017061	PSMA4
4802.193	4.955998	-7068	7.47E-05	913	0.333333	0.012958	SRSF7
5597.187	6.309973	-7068	7.45E-05	815	0.333333	0.011046	NCF4
10331.49	6.741597	-7068	7.61E-05	1097	0.333333	0.016853	NRCAM
5739.95	9.486585	-7068	7.36E-05	605	0.333333	0.007481	SULT2B1
18323.16	6.669425	-7068	7.82E-05	1468	0.333333	0.022289	INSIG2
13688.94	6.324438	-7068	7.69E-05	1324	0.333333	0.018909	CBL
7703.171	8.506996	-7068	7.42E-05	751	0.333333	0.00874	GCGR
4643.067	6.028687	-7068	7.44E-05	792	0.333333	0.012295	ACBD3
10969.06	7.144625	-7068	7.60E-05	1085	0.333333	0.015897	RYR2
10816.5	7.614579	-7068	7.56E-05	1016	0.333333	0.015125	TYR
9137.694	7.343701	-7068	7.53E-05	953	0.333333	0.014033	GABBR2
13621.71	6.539838	-7068	7.70E-05	1299	0.333333	0.018817	RCC1
6477.497	8.518849	-7068	7.41E-05	711	0.333333	0.009601	BPHL
6369.824	6.909692	-7068	7.48E-05	852	0.333333	0.012979	RPE
4233.349	7.518778	-7068	7.37E-05	642	0.333333	0.009194	SSTR2
10031.32	7.892248	-7068	7.53E-05	946	0.333333	0.01255	ACADS
4571.119	7.964426	-7068	7.37E-05	635	0.333333	0.008632	GPSM3
12957.51	7.708973	-7068	7.55E-05	1049	0.333333	0.013374	FPR1
5250.78	8.129572	-7068	7.38E-05	634	0.333333	0.007939	GPAA1
16084.02	6.44333	-7068	7.78E-05	1423	0.333333	0.021627	PDE4D
6535.648	7.091376	-7068	7.46E-05	830	0.333333	0.011523	TNFRSF17

3075.852	5.94541	-7068	7.32E-05	609	0.333333	0.008015	GEMIN2
7817.839	6.997625	-7068	7.50E-05	929	0.333333	0.012985	DDIT3
14395.24	7.548309	-7068	7.66E-05	1194	0.333333	0.017787	PTPRN2
4742.965	6.62744	-7068	7.40E-05	720	0.333333	0.009272	AFM
9892.477	7.046494	-7068	7.59E-05	1048	0.333333	0.016094	PKD2
21099.08	7.715633	-7068	7.79E-05	1418	0.333333	0.020505	SPR
5725.833	11.22159	-7068	7.33E-05	542	0.333333	0.006709	NFKBIL1
12603.85	6.888739	-7068	7.66E-05	1197	0.333333	0.017733	PTGS1
11094.48	11.3144	-7068	7.44E-05	735	0.333333	0.009662	MTNR1B
10342.49	6.313251	-7068	7.57E-05	1094	0.333333	0.014433	KRT5
5069.223	7.051726	-7068	7.44E-05	748	0.333333	0.011808	DIDO1
17781.77	7.553535	-7068	7.65E-05	1256	0.333333	0.016548	NDUFA9
10393.53	6.870485	-7068	7.59E-05	1095	0.333333	0.016184	IFNAR2
5608.902	6.721308	-7068	7.39E-05	767	0.333333	0.008967	TRAF2
9031.737	7.460892	-7068	7.54E-05	957	0.333333	0.014263	PTPN4
14809.35	5.955994	-7068	7.77E-05	1446	0.333333	0.021621	CTNNA1
6577.49	11.2745	-7068	7.35E-05	588	0.333333	0.008267	DAZL
6451.075	6.928347	-7068	7.47E-05	864	0.333333	0.012614	GADD45A
9370.621	6.629702	-7068	7.58E-05	1049	0.333333	0.015319	TNFAIP2
3216.048	6.517874	-7068	7.35E-05	629	0.333333	0.0094	DDX17
10720.35	7.400687	-7068	7.56E-05	1034	0.333333	0.014931	CYP7A1
7395.7	5.935174	-7068	7.49E-05	983	0.333333	0.012731	ACTB
12110.79	6.420857	-7068	7.67E-05	1235	0.333333	0.018684	NRIP1
12405.73	7.256188	-7068	7.64E-05	1142	0.333333	0.01695	TFAP2B
18720.69	6.469342	-7068	7.82E-05	1517	0.333333	0.022162	ATP6V1E1
6496.116	7.346592	-7068	7.46E-05	813	0.333333	0.011063	MIF
3579.267	8.11799	-7068	7.32E-05	541	0.333333	0.007028	ERGIC3
6667.884	6.413293	-7068	7.50E-05	914	0.333333	0.013795	SGCB
7252.54	6.971247	-7068	7.50E-05	895	0.333333	0.013158	NFE2L1
6901.463	8.209802	-7068	7.44E-05	754	0.333333	0.009993	IGFALS
7477.365	8.364288	-7068	7.48E-05	800	0.25	0.01193	IPCEF1
15942.85	6.521024	-7068	7.76E-05	1386	0.333333	0.020651	RND3
10807	6.195974	-7068	7.64E-05	1183	0.333333	0.017535	PIAS1
15943.94	6.454737	-7068	7.75E-05	1403	0.333333	0.020596	LEF1
23375.67	7.033174	-7068	7.86E-05	1573	0.333333	0.022161	SLC2A1
9332.388	6.158383	-7068	7.55E-05	1083	0.333333	0.014936	TRA2A
1329.776	7.613507	-7068	7.22E-05	361	0.333333	0.005231	MECP2
4473.682	7.09534	-7068	7.39E-05	688	0.333333	0.010343	SLC35A3
4890.423	10.03469	-7068	7.33E-05	542	0.333333	0.007039	SEMA6C
5669.106	6.944779	-7068	7.43E-05	782	0.333333	0.010804	STAT4
6077.24	9.505344	-7068	7.39E-05	656	0.333333	0.009273	SHH

7490.597	7.211703	-7068	7.51E-05	903	0.333333	0.013436	TMEM109
7900.141	7.972352	-7068	7.47E-05	842	0.333333	0.011613	BSG
9366.995	7.210225	-7068	7.55E-05	990	0.333333	0.014587	FZD2
10099.84	5.819752	-7068	7.62E-05	1173	0.333333	0.016273	SSRP1
10106.59	7.277294	-7068	7.58E-05	1034	0.333333	0.015771	SH3BGRL
11987.1	6.725871	-7068	7.62E-05	1168	0.333333	0.016514	GLUD1
5395.422	9.081241	-7068	7.36E-05	600	0.333333	0.007585	MC2R
32225.73	5.171303	-7068	8.11E-05	2268	0.333333	0.029489	JUN
11298.29	7.260442	-7068	7.63E-05	1104	0.333333	0.016988	BTC
3888.853	7.431485	-7068	7.38E-05	638	0.333333	0.009903	TRIM13
4243.987	7.72651	-7068	7.38E-05	642	0.333333	0.009223	ANPEP
5144.381	7.741345	-7068	7.40E-05	681	0.333333	0.009016	ATP6AP1
14316.41	7.591432	-7068	7.65E-05	1170	0.333333	0.016416	DOK5
11564.09	7.815882	-7068	7.57E-05	1013	0.333333	0.013908	DMBT1
7738.789	6.143745	-7068	7.51E-05	992	0.333333	0.01341	CDC42
5895.865	8.144824	-7068	7.40E-05	714	0.333333	0.010123	MMP13
6627.242	8.488946	-7068	7.43E-05	742	0.333333	0.010508	ZBTB17
4239.091	5.943322	-7068	7.44E-05	769	0.333333	0.012059	LRPPRC
3188.013	6.767056	-7068	7.35E-05	610	0.333333	0.009468	MTMR9
2329.04	7.550499	-7068	7.27E-05	463	0.333333	0.00582	TNNT2
8623.98	8.073394	-7068	7.50E-05	868	0.333333	0.012497	EIF2B5
9769.987	6.799419	-7068	7.61E-05	1090	0.333333	0.016882	MYO1E
12515.7	7.71297	-7068	7.61E-05	1101	0.333333	0.015747	MKNK1
2806.313	8.785256	-7068	7.28E-05	470	0.333333	0.006681	BCL2L2
4286.586	8.35928	-7068	7.35E-05	586	0.333333	0.007892	SYP
7374.52	7.761144	-7068	7.48E-05	842	0.333333	0.012409	PPP1R16B
7650.482	8.405186	-7068	7.45E-05	796	0.333333	0.011164	MAPK8IP3
4625.144	6.257327	-7068	7.42E-05	765	0.333333	0.011063	XCL1
9097.136	9.102948	-7068	7.47E-05	822	0.333333	0.011659	LHCGR
8953.453	8.801048	-7068	7.46E-05	810	0.333333	0.012475	SPAG6
7597.047	7.060457	-7068	7.50E-05	897	0.333333	0.012749	CXCL6
9346.771	7.222769	-7068	7.56E-05	990	0.333333	0.014681	DHRS7
6042.033	7.395699	-7068	7.46E-05	804	0.333333	0.011956	ETV4
5245.731	8.502924	-7068	7.34E-05	608	0.333333	0.00695	PRTN3
8009.484	7.112402	-7068	7.50E-05	919	0.333333	0.012934	IFIT3
4153.815	6.766857	-7068	7.35E-05	651	0.25	0.008084	AKR1D1
5243.7	6.793544	-7068	7.41E-05	765	0.333333	0.010133	TGFB1
20794.82	6.515044	-7068	7.89E-05	1632	0.333333	0.024398	PGD
10800.61	6.211125	-7068	7.64E-05	1186	0.333333	0.017822	TNPO1
8474.133	6.895956	-7068	7.54E-05	970	0.333333	0.013868	PHYH
6667.724	5.768464	-7068	7.51E-05	957	0.333333	0.013353	ETFA

2624.405	7.122622	-7068	7.30E-05	515	0.333333	0.007374	MRPS31
6802.13	7.099484	-7068	7.45E-05	811	0.333333	0.010473	ATP5G1
11677.84	7.348107	-7068	7.61E-05	1111	0.333333	0.016825	AKAP1
7370.379	5.683841	-7068	7.50E-05	1001	0.333333	0.013062	RPL14
8205.114	6.704224	-7068	7.54E-05	981	0.333333	0.014182	BMP6
14702.9	6.496317	-7068	7.72E-05	1328	0.333333	0.018669	RAB5C
1395.345	8.187075	-7068	7.20E-05	342	0.25	0.005057	KRIT1
9418.023	6.104118	-7068	7.57E-05	1122	0.333333	0.015564	CDK6
12091.8	7.003996	-7068	7.64E-05	1163	0.333333	0.016566	ATP1A1
10877.23	6.676196	-7068	7.63E-05	1140	0.333333	0.017788	ACSL3
7615.124	7.451583	-7068	7.49E-05	869	0.333333	0.01277	HSPA4L
10767.46	7.839857	-7068	7.55E-05	983	0.333333	0.013931	OSR2
7313.137	8.304428	-7068	7.44E-05	783	0.333333	0.010813	NFKBIE
3550.621	9.625161	-7068	7.28E-05	465	0.333333	0.005748	PICK1
4400.485	5.711921	-7068	7.45E-05	813	0.333333	0.012763	NFE2L2
14040.33	7.080093	-7068	7.67E-05	1222	0.333333	0.017686	NEFH
4101.712	7.461683	-7068	7.34E-05	609	0.333333	0.00779	JAG2
10803.14	6.441094	-7068	7.65E-05	1188	0.333333	0.018286	TFRC
6812.651	6.018398	-7068	7.42E-05	868	0.333333	0.009511	APOA1
9692.567	7.080243	-7068	7.58E-05	1043	0.333333	0.015542	ANXA6
11347.17	5.946671	-7068	7.65E-05	1246	0.333333	0.018267	ATF2
4740.277	6.003927	-7068	7.41E-05	790	0.333333	0.011065	PSEN1
6372.637	9.78029	-7068	7.37E-05	645	0.333333	0.008726	FABP3
2099.604	5.961134	-7068	7.28E-05	520	0.333333	0.007339	DIS3
6210.192	6.685001	-7068	7.49E-05	869	0.333333	0.013336	MBD2
13932.32	7.288377	-7068	7.68E-05	1216	0.333333	0.018094	RFTN1
2415.792	5.342555	-7068	7.34E-05	610	0.333333	0.009048	PRPF4B
9887.525	6.901929	-7068	7.55E-05	1033	0.333333	0.013803	PGM1
20282.92	5.821165	-7068	7.85E-05	1664	0.333333	0.023423	LAMC1
8710.377	10.63514	-7068	7.40E-05	700	0.333333	0.0091	ZNF134
11246.64	7.988218	-7068	7.55E-05	1002	0.333333	0.014434	PTCH1
7012.093	6.398669	-7068	7.53E-05	953	0.333333	0.014983	HBS1L
8916.615	6.69982	-7068	7.57E-05	1028	0.333333	0.015025	MYL12A
8875.348	8.289687	-7068	7.49E-05	873	0.333333	0.01256	ZNF165
5766.202	6.296265	-7068	7.46E-05	822	0.333333	0.010952	TUFM
5961.645	7.291762	-7068	7.42E-05	769	0.333333	0.009978	ANG
8576.948	7.380388	-7068	7.50E-05	898	0.333333	0.011333	F2
12828.52	6.287176	-7068	7.68E-05	1285	0.333333	0.018427	CD36
6133.396	8.161256	-7068	7.42E-05	746	0.333333	0.010668	CHRNA5
3476.499	6.870992	-7068	7.36E-05	623	0.333333	0.00972	LYPLA1
15209.09	6.31139	-7068	7.69E-05	1340	0.333333	0.017645	GC

4408.922	8.924846	-7068	7.34E-05	581	0.333333	0.008151	CTSF
7444.968	6.772954	-7068	7.49E-05	910	0.333333	0.013408	MYO6
4983.33	7.183514	-7068	7.42E-05	737	0.333333	0.010845	ATF7
6101.843	7.559817	-7068	7.46E-05	777	0.333333	0.011279	RNH1
10239.96	6.422892	-7068	7.63E-05	1146	0.333333	0.017378	CAP1
8739.806	6.285347	-7068	7.57E-05	1062	0.333333	0.015902	DUSP6
8773.771	5.820676	-7068	7.61E-05	1132	0.333333	0.017104	CNN3
2396.463	7.007699	-7068	7.30E-05	508	0.333333	0.00778	ARHGAP5
5705.605	10.11907	-7068	7.34E-05	563	0.333333	0.006662	DPEP1
3527.504	5.251061	-7068	7.38E-05	735	0.333333	0.010175	HNRNPH1
6980.921	6.458536	-7068	7.52E-05	935	0.333333	0.014283	BAZ1A
11491.46	6.664474	-7068	7.63E-05	1165	0.333333	0.017227	LPP
8389.992	5.705356	-7068	7.53E-05	1084	0.333333	0.014326	RAC1
7312.355	6.707775	-7068	7.51E-05	926	0.333333	0.013942	DSG2
4685.825	9.538762	-7068	7.35E-05	579	0.333333	0.008759	NUDT3
7744.586	8.204711	-7068	7.46E-05	805	0.333333	0.011078	DNM2
5199.679	7.184562	-7068	7.42E-05	752	0.333333	0.01151	EIF4G2
8974.619	6.49836	-7068	7.56E-05	1057	0.333333	0.014962	STAT5B
7498.265	7.103607	-7068	7.48E-05	878	0.333333	0.011909	SELE
5294.106	7.37036	-7068	7.43E-05	751	0.333333	0.010847	TCOF1
4305.236	7.142497	-7068	7.38E-05	661	0.333333	0.009993	UBQLN2
9095.206	6.45247	-7068	7.59E-05	1074	0.333333	0.016277	RIT1
9000.592	7.856748	-7068	7.51E-05	927	0.333333	0.013525	TEC
12600.11	6.745635	-7068	7.67E-05	1219	0.333333	0.018099	PRDM1
14257.01	6.511325	-7068	7.72E-05	1317	0.333333	0.01975	HSPH1
16719.71	7.677711	-7068	7.72E-05	1282	0.333333	0.019008	CTNNBIP1
25118.75	7.215496	-7068	7.92E-05	1640	0.333333	0.024799	RAPGEF1
18080.45	6.743831	-7068	7.81E-05	1446	0.333333	0.022162	GRIA1
8261.862	6.774763	-7068	7.54E-05	985	0.333333	0.014307	F5
1987.815	6.387254	-7068	7.26E-05	472	0.333333	0.005885	CD72
3772.662	5.614282	-7068	7.40E-05	732	0.333333	0.010581	CSTF1
6904.543	6.911887	-7068	7.49E-05	873	0.333333	0.012607	CYP3A5
6000.033	7.339915	-7068	7.43E-05	768	0.333333	0.01058	SLC6A4
6338.91	7.470985	-7068	7.44E-05	784	0.333333	0.010523	HMOX1
5349.419	6.888019	-7068	7.41E-05	745	0.333333	0.009837	CCL11
6221.836	7.390998	-7068	7.44E-05	781	0.333333	0.010421	IDO1
6241.014	8.425742	-7068	7.42E-05	727	0.333333	0.01066	FEZ1
10186.66	7.976196	-7068	7.52E-05	939	0.333333	0.013346	TMPRSS2
6890.628	6.53024	-7068	7.51E-05	906	0.333333	0.013929	TFAM
14202.26	7.010243	-7068	7.70E-05	1251	0.333333	0.018562	EFHD1
9205.503	8.000121	-7068	7.51E-05	904	0.333333	0.012997	COL9A1

9902.646	6.419743	-7068	7.64E-05	1146	0.333333	0.01818	LRRFIP1
4223.184	7.838864	-7068	7.37E-05	629	0.333333	0.009078	VPS39
27042.55	6.423567	-7068	8.01E-05	1838	0.333333	0.027363	BMP2
7733.958	8.868004	-7068	7.43E-05	755	0.333333	0.010307	ANKRD1
10919.8	7.271602	-7068	7.58E-05	1062	0.333333	0.01542	ATP1B1
2681.3	9.539104	-7068	7.27E-05	437	0.333333	0.006355	MLH3
6563.959	6.82478	-7068	7.45E-05	833	0.333333	0.010792	C9
17658.8	7.434824	-7068	7.75E-05	1339	0.333333	0.019962	MTCL1
10640.75	8.024125	-7068	7.56E-05	983	0.333333	0.013725	PCK2
7268.048	7.718695	-7068	7.47E-05	840	0.333333	0.012245	CCNA1
31256.89	7.119482	-7068	8.03E-05	1834	0.333333	0.027366	GFRA1
8706.566	7.444642	-7068	7.54E-05	954	0.333333	0.014235	SLC7A5
12449.89	5.889316	-7068	7.64E-05	1261	0.333333	0.016656	ATP5A1
5997.057	6.487214	-7068	7.49E-05	866	0.333333	0.013513	TMEM47
7039.144	7.821302	-7068	7.47E-05	827	0.333333	0.011973	AFP
3360.331	9.622236	-7068	7.30E-05	483	0.333333	0.006425	BRF1
2985.226	7.179429	-7068	7.31E-05	541	0.333333	0.00705	RAB11B
10251.59	8.46859	-7068	7.46E-05	862	0.333333	0.010328	NDUFV1
5755.491	7.549823	-7068	7.45E-05	760	0.333333	0.010964	CRIP2
17443.88	7.456143	-7068	7.73E-05	1317	0.333333	0.018957	TG
9395.422	6.225087	-7068	7.60E-05	1113	0.333333	0.016912	RBM25
2828.706	9.974937	-7068	7.25E-05	406	0.25	0.005429	DNALI1
5859.313	9.874131	-7068	7.36E-05	612	0.333333	0.007907	DNASE2
6641.314	5.832087	-7068	7.48E-05	909	0.333333	0.011696	NCF2
3117.131	7.474663	-7068	7.32E-05	542	0.333333	0.007464	CRH
4905.142	7.815578	-7068	7.41E-05	685	0.333333	0.009893	LRPAP1
4647.899	7.943183	-7068	7.37E-05	648	0.333333	0.009015	P2RX7
6712.225	6.610609	-7068	7.49E-05	893	0.333333	0.013062	RAD51C
4242.047	9.195035	-7068	7.32E-05	530	0.333333	0.00675	RLBP1
13155.88	6.582076	-7068	7.68E-05	1256	0.333333	0.018225	ACO1
6294.484	7.833637	-7068	7.46E-05	784	0.333333	0.011957	SCML2
8979.523	6.168654	-7068	7.56E-05	1073	0.333333	0.014871	CAT
7831.509	6.235974	-7068	7.53E-05	984	0.333333	0.014176	GEM
5920.122	8.628054	-7068	7.42E-05	695	0.333333	0.010345	NAT1
40550.08	7.085244	-7068	8.17E-05	2078	0.333333	0.030558	MUC1
5372.4	7.441295	-7068	7.42E-05	738	0.333333	0.0104	PAX5
14155.49	5.091291	-7068	7.73E-05	1492	0.333333	0.019653	MCM6
12569.19	6.092408	-7068	7.71E-05	1315	0.333333	0.019804	PAICS
5504.371	6.44283	-7068	7.42E-05	791	0.333333	0.010333	ALB
6213.4	7.832324	-7068	7.45E-05	783	0.333333	0.011809	OAZ2
2817.235	6.928601	-7068	7.32E-05	552	0.333333	0.0074	ERCC1

9307.043	8.123101	-7068	7.53E-05	915	0.333333	0.013425	ACSM3
10973.83	7.581588	-7068	7.58E-05	1032	0.333333	0.014859	CACNA2D1
4731.137	8.807864	-7068	7.35E-05	599	0.333333	0.00807	PRSS16
24805.72	6.557055	-7068	7.96E-05	1735	0.333333	0.025899	SUCLG2
8349.044	7.167291	-7068	7.51E-05	949	0.333333	0.013105	MAPK13
6061.487	7.736337	-7068	7.44E-05	771	0.333333	0.011384	ATP6V0A2
7187.58	5.652867	-7068	7.54E-05	1019	0.333333	0.014422	VWF
3191.725	5.74305	-7068	7.37E-05	674	0.333333	0.009974	PNN
8081.053	4.949369	-7068	7.56E-05	1145	0.333333	0.01499	SRSF3
6328.76	6.24112	-7068	7.46E-05	862	0.333333	0.011703	AEBP1
5902.176	7.254243	-7068	7.44E-05	794	0.333333	0.011578	EEF1D
5230.773	6.232973	-7068	7.44E-05	820	0.333333	0.012081	COL4A5
16333.43	6.557072	-7068	7.77E-05	1412	0.333333	0.021072	TRIO
5198.875	5.45487	-7068	7.41E-05	817	0.333333	0.009867	CCL5
7784.367	6.396944	-7068	7.53E-05	987	0.333333	0.013687	ACAT1
6273.256	8.855252	-7068	7.38E-05	665	0.333333	0.008327	APOA4
7346.405	7.839878	-7068	7.48E-05	847	0.333333	0.012071	FXN
5425.168	7.502043	-7068	7.39E-05	695	0.333333	0.008951	SPRR1B
4809.189	9.030404	-7068	7.36E-05	586	0.333333	0.007689	PNPLA2
12045.86	6.457592	-7068	7.66E-05	1217	0.333333	0.018216	CDK14
6609.769	6.536518	-7068	7.48E-05	876	0.333333	0.012542	LSM3
5829.015	8.787993	-7068	7.39E-05	679	0.333333	0.010034	PCP4
8124.371	6.721582	-7068	7.51E-05	958	0.333333	0.012912	GPX3
13607.52	7.333065	-7068	7.68E-05	1202	0.333333	0.017519	LMNB2
6671.676	8.553798	-7068	7.39E-05	699	0.333333	0.008878	NDUFA7
5702.418	6.452052	-7068	7.49E-05	856	0.333333	0.013735	NAB1
3989.005	8.098051	-7068	7.36E-05	600	0.333333	0.008559	MGAT3
5215.84	7.141582	-7068	7.42E-05	749	0.333333	0.010755	CDK13
4539.675	6.669417	-7068	7.42E-05	723	0.333333	0.010538	LYZ
4829.03	7.440788	-7068	7.39E-05	693	0.333333	0.010063	L1CAM
9213.565	9.452858	-7068	7.44E-05	778	0.333333	0.010518	PITX2
6844.545	6.392215	-7068	7.54E-05	941	0.333333	0.015032	WAPL
6912.648	7.794788	-7068	7.46E-05	795	0.333333	0.01122	ISG20
10466.91	6.947432	-7068	7.59E-05	1064	0.333333	0.01532	TGFB3
17248.43	5.626165	-7068	7.78E-05	1547	0.333333	0.021064	MAD2L1
8046.374	8.066681	-7068	7.48E-05	846	0.333333	0.012303	SH3GL3
10303.53	5.76049	-7068	7.63E-05	1212	0.333333	0.017922	CSNK1A1
13348.22	6.232759	-7068	7.65E-05	1269	0.333333	0.016451	F9
3875.105	10.04282	-7068	7.30E-05	488	0.333333	0.00675	HAS1
5457.916	7.18599	-7068	7.45E-05	770	0.333333	0.011752	DCTN5
6945.643	7.980398	-7068	7.45E-05	779	0.333333	0.010959	REG3A

8055.273	7.732059	-7068	7.46E-05	821	0.333333	0.010612	SLC22A6
10190.17	5.564876	-7068	7.64E-05	1234	0.333333	0.01787	TNFAIP3
9964.831	7.31858	-7068	7.55E-05	1011	0.333333	0.014367	CYP26A1
5149.146	10.55236	-7068	7.33E-05	535	0.333333	0.006807	DRD4
5595.55	6.98086	-7068	7.42E-05	781	0.333333	0.011192	CR1
6929.604	8.776843	-7068	7.41E-05	705	0.333333	0.008994	NPPB
14514.22	6.037829	-7068	7.75E-05	1412	0.333333	0.02154	CDH2
3133.139	6.225996	-7068	7.31E-05	581	0.333333	0.006893	TTPA
4149.754	7.486457	-7068	7.38E-05	645	0.333333	0.009387	BTG2
9090.521	7.307536	-7068	7.56E-05	984	0.333333	0.01492	CHRM3
7556.155	6.970854	-7068	7.50E-05	905	0.333333	0.01314	PDK4
11169.29	6.051854	-7068	7.67E-05	1241	0.333333	0.019281	PICALM
8484.278	5.680127	-7068	7.57E-05	1093	0.333333	0.015688	LAMB1
4054.461	7.267379	-7068	7.38E-05	658	0.333333	0.009994	PHTF1
8318.766	6.584731	-7068	7.54E-05	995	0.333333	0.014528	STK17B
6887.608	6.024826	-7068	7.54E-05	972	0.333333	0.014944	DLAT
11359.59	7.510181	-7068	7.59E-05	1055	0.333333	0.014383	CDA
10771.96	6.305709	-7068	7.62E-05	1178	0.333333	0.016922	MET
14888.08	5.854383	-7068	7.75E-05	1450	0.333333	0.020792	IQGAP1
4897.59	6.467751	-7068	7.43E-05	787	0.333333	0.011813	ADH5
5491.164	6.034608	-7068	7.49E-05	879	0.333333	0.013556	SON
2718.115	8.916042	-7068	7.27E-05	441	0.333333	0.005611	GTPBP6
3539.973	8.8551	-7068	7.30E-05	501	0.333333	0.006036	NPHS1
5076.075	6.282663	-7068	7.47E-05	827	0.333333	0.013232	ACP1
13843.73	7.074781	-7068	7.67E-05	1223	0.333333	0.018189	IL15
7008.105	12.2501	-7068	7.32E-05	528	0.333333	0.006107	VGF
8784.023	7.291303	-7068	7.53E-05	956	0.333333	0.01389	DUSP4
9884.435	7.839361	-7068	7.55E-05	961	0.333333	0.013947	TMED3
17533.74	6.124411	-7068	7.85E-05	1547	0.333333	0.024068	MAN1A1
3839.699	6.351002	-7068	7.34E-05	633	0.333333	0.007681	COX7C
7772.224	7.123719	-7068	7.44E-05	785	0.333333	0.011829	MPPED2
8048.685	7.109802	-7068	7.52E-05	929	0.333333	0.013211	ENG
4274.651	6.29658	-7068	7.44E-05	754	0.333333	0.011918	TOMM20
3551.023	8.7081	-7068	7.33E-05	539	0.333333	0.00781	ATN1
5343.252	6.506578	-7068	7.46E-05	814	0.333333	0.01192	SHMT2
4132.357	9.839227	-7068	7.32E-05	521	0.333333	0.007054	SLC6A8
3578.12	6.431098	-7068	7.35E-05	625	0.333333	0.008035	IDH3G
5473.041	7.342584	-7068	7.41E-05	738	0.333333	0.010301	MYOC
7689.762	7.062973	-7068	7.47E-05	868	0.333333	0.011429	DSG3
6983.586	5.792903	-7068	7.47E-05	942	0.333333	0.011605	CYP2C8
7305.09	7.467522	-7068	7.50E-05	876	0.333333	0.012496	GLA

8736.823	8.027084	-7068	7.50E-05	883	0.333333	0.012832	CARTPT
16639.93	6.042285	-7068	7.79E-05	1508	0.333333	0.021672	FLNB
4396.583	7.423789	-7068	7.39E-05	676	0.333333	0.010176	SLC11A2
6578.47	7.190232	-7068	7.48E-05	850	0.333333	0.012997	LIG3
20851.99	6.257288	-7068	7.83E-05	1603	0.333333	0.021916	SERPINA1
44526.54	6.933492	-7068	8.30E-05	2241	0.333333	0.034412	AUTS2
10255.09	6.779907	-7068	7.63E-05	1110	0.333333	0.017136	RASGRP1
7094.71	7.986742	-7068	7.46E-05	800	0.333333	0.01127	SEMA4D
6103.921	7.268652	-7068	7.44E-05	786	0.333333	0.011106	C14orf2
16035.28	6.692499	-7068	7.76E-05	1386	0.333333	0.020574	DNAJA1
12863.05	6.230971	-7068	7.64E-05	1250	0.333333	0.016814	MBL2
3876.139	6.277896	-7068	7.40E-05	712	0.333333	0.010695	PTPN22
5674.612	7.454053	-7068	7.43E-05	760	0.333333	0.011861	ZKSCAN1
10349.62	5.984765	-7068	7.64E-05	1198	0.333333	0.01775	MAPRE1
7457.005	8.573889	-7068	7.45E-05	768	0.333333	0.010901	DRD2
7211.916	7.84547	-7068	7.47E-05	821	0.333333	0.012218	MRPS14
6262.688	8.436231	-7068	7.41E-05	705	0.25	0.009735	SLC5A1
3603.327	7.808394	-7068	7.32E-05	551	0.333333	0.007313	BSN
3928.048	7.943279	-7068	7.36E-05	605	0.333333	0.009176	RPGR
15343.19	7.116809	-7068	7.71E-05	1274	0.333333	0.018681	LOXL1
3582.949	6.43772	-7068	7.36E-05	661	0.333333	0.009266	CTBP1
4943.661	9.391658	-7068	7.35E-05	576	0.333333	0.00768	RUNDC3A
10571.89	6.183059	-7068	7.64E-05	1177	0.333333	0.017698	CAPZA2
4795.216	6.916692	-7068	7.42E-05	732	0.333333	0.010976	OAS1
9112.033	7.017695	-7068	7.56E-05	1010	0.333333	0.014947	CDKL5
10850.61	7.012021	-7068	7.56E-05	1042	0.333333	0.013585	UQCRC1
12137.1	6.918635	-7068	7.66E-05	1172	0.333333	0.018096	ATP8A2
7070.616	10.94598	-7068	7.37E-05	613	0.333333	0.00806	PLA2G6
7607.826	8.456822	-7068	7.43E-05	779	0.333333	0.010047	FDXR
5482.885	9.427899	-7068	7.37E-05	610	0.333333	0.008656	MAS1
11860.02	6.162806	-7068	7.70E-05	1261	0.333333	0.019424	SCARB2
13781.8	6.931247	-7068	7.70E-05	1255	0.333333	0.019557	OTUD4
4038.328	8.514542	-7068	7.35E-05	593	0.333333	0.008536	PATZ1
3495.605	7.568286	-7068	7.34E-05	595	0.333333	0.008512	BCL2L11
12438.66	5.020071	-7068	7.73E-05	1458	0.333333	0.020143	HNRNPM
6659.103	6.995482	-7068	7.49E-05	856	0.333333	0.013449	REEP1
7994.934	8.298193	-7068	7.45E-05	806	0.333333	0.01051	LEP
7103.183	7.119541	-7068	7.50E-05	887	0.333333	0.013347	PPP3R1
2900.655	5.731048	-7068	7.34E-05	626	0.333333	0.008983	ASF1A
7397.914	6.787689	-7068	7.52E-05	924	0.333333	0.013511	MAOB
2485.869	6.258055	-7068	7.31E-05	563	0.333333	0.008401	KRR1

6359.023	6.054159	-7068	7.46E-05	878	0.333333	0.011703	TAGLN
2492.886	9.109665	-7068	7.27E-05	432	0.333333	0.005935	ACHE
8592.01	7.514484	-7068	7.53E-05	943	0.333333	0.013808	AMMECR1
11923.09	7.397767	-7068	7.55E-05	1056	0.333333	0.013432	TK1
23761.51	6.976844	-7068	7.92E-05	1631	0.333333	0.025013	ADGRL3
9280.032	6.445937	-7068	7.58E-05	1066	0.333333	0.015013	TUBA1B
4097.692	8.980657	-7068	7.33E-05	548	0.25	0.007066	DGAT1
6271.164	7.112698	-7068	7.46E-05	823	0.333333	0.012096	PTK7
5699.573	8.734217	-7068	7.39E-05	655	0.333333	0.00922	DNAH9
7900.334	5.832021	-7068	7.50E-05	1030	0.333333	0.012973	STAT1
15610.93	8.444462	-7068	7.60E-05	1113	0.333333	0.014238	COX8A
17055.24	7.711075	-7068	7.71E-05	1266	0.333333	0.018647	GREM1
7614.041	6.869795	-7068	7.52E-05	926	0.333333	0.014217	ZFR
6720.723	8.140745	-7068	7.46E-05	789	0.333333	0.012026	GGH
7966.415	7.463823	-7068	7.50E-05	885	0.333333	0.01233	CIB1
9981.203	7.15791	-7068	7.55E-05	1016	0.333333	0.013416	ETFB
5857.019	8.46724	-7068	7.41E-05	712	0.333333	0.010574	DCLRE1A
3140.083	9.464079	-7068	7.29E-05	469	0.333333	0.006579	ZNF142
5048.736	7.12949	-7068	7.41E-05	725	0.333333	0.01041	LAMTOR5
3080.827	8.592538	-7068	7.31E-05	505	0.333333	0.006874	ZMYM3
6586.901	5.763916	-7068	7.52E-05	971	0.333333	0.014175	TMX1
4513.144	7.766509	-7068	7.40E-05	664	0.333333	0.010117	JADE3
3858.327	9.076251	-7068	7.32E-05	531	0.333333	0.007022	SNRNP35
5470.866	7.146312	-7068	7.42E-05	779	0.333333	0.010644	CDKN2A
5730.176	9.251087	-7068	7.36E-05	620	0.333333	0.007585	NR5A1
8417.912	6.41823	-7068	7.56E-05	1027	0.333333	0.015045	AXL
8747.173	6.195259	-7068	7.58E-05	1061	0.333333	0.015561	LTBP2
9923.549	9.980723	-7068	7.46E-05	775	0.333333	0.01003	MDFI
8202.762	7.349755	-7068	7.50E-05	900	0.333333	0.012015	MAP2K2
2661.523	8.252301	-7068	7.26E-05	446	0.333333	0.005199	TGM1
7091.688	6.842146	-7068	7.49E-05	892	0.333333	0.012735	PRKAA2
7309.129	6.632308	-7068	7.52E-05	954	0.333333	0.013798	CCND1
5218.181	6.829035	-7068	7.42E-05	770	0.333333	0.010929	FGFR3
5471.069	8.69038	-7068	7.40E-05	674	0.333333	0.010117	PADI2
3028.552	6.812027	-7068	7.32E-05	565	0.333333	0.007584	AIM2
3720.722	10.34521	-7068	7.30E-05	470	0.333333	0.005784	SNAPC2
4897.12	8.823405	-7068	7.38E-05	625	0.333333	0.008444	TERT
2837.153	9.030104	-7068	7.29E-05	461	0.333333	0.006221	COG7
23822.89	5.971585	-7068	7.95E-05	1797	0.333333	0.025874	TPM1
12175.95	7.144494	-7068	7.60E-05	1110	0.333333	0.015337	CSTA
10751.23	6.213941	-7068	7.65E-05	1199	0.333333	0.018184	APLP2

6076.4	7.772781	-7068	7.44E-05	764	0.333333	0.010895	IGFBP2
7274.084	5.979684	-7068	7.54E-05	1004	0.333333	0.014569	AK2
5485.092	9.583386	-7068	7.36E-05	606	0.333333	0.007758	SAC3D1
5088.69	6.219389	-7068	7.40E-05	793	0.333333	0.010625	RIPK1
2903.746	9.599145	-7068	7.28E-05	441	0.333333	0.006332	B4GALNT1
10462.79	7.816895	-7068	7.56E-05	988	0.333333	0.014558	NTF3
15529.11	7.03111	-7068	7.73E-05	1320	0.333333	0.019657	ANXA1
4611.865	7.605595	-7068	7.38E-05	667	0.333333	0.009352	PDCD11
8495.4	7.686354	-7068	7.53E-05	919	0.333333	0.01421	CREG1
7342.681	8.929626	-7068	7.43E-05	743	0.333333	0.010052	LLGL2
11291.04	7.491507	-7068	7.61E-05	1065	0.333333	0.016384	EPCAM
5838.165	6.760273	-7068	7.45E-05	814	0.333333	0.011648	HIBCH
8823.772	7.625605	-7068	7.54E-05	939	0.333333	0.014042	P2RX4
2958.309	8.170959	-7068	7.28E-05	481	0.333333	0.006021	CLCA1
13744.01	6.289464	-7068	7.73E-05	1344	0.333333	0.020338	NREP
4187.669	7.335157	-7068	7.39E-05	668	0.333333	0.010224	MBTPS1
11023.09	5.691007	-7068	7.65E-05	1254	0.333333	0.017856	CTGF
8442.077	8.590733	-7068	7.48E-05	831	0.333333	0.011959	NTHL1
10397.86	7.686483	-7068	7.58E-05	1006	0.333333	0.014795	NPC2
3406.394	7.180998	-7068	7.34E-05	594	0.333333	0.008617	CHM
4180.431	6.672489	-7068	7.41E-05	710	0.333333	0.01125	DNAJB6
9219.425	7.048658	-7068	7.51E-05	950	0.333333	0.011763	ECHS1
6994.502	8.304734	-7068	7.45E-05	773	0.333333	0.012007	ARMCX2
2608.86	8.642137	-7068	7.27E-05	445	0.333333	0.005665	SDR39U1
4886.151	6.87858	-7068	7.41E-05	740	0.333333	0.010577	XIAP
10482.03	5.747991	-7068	7.58E-05	1176	0.333333	0.014663	BUB1B
3855.521	9.231809	-7068	7.31E-05	520	0.333333	0.006992	SLC6A1
5368.198	8.176382	-7068	7.41E-05	694	0.333333	0.010186	CBFA2T3
5178.434	6.652338	-7068	7.45E-05	798	0.333333	0.012065	SOCS2
9740.14	5.799389	-7068	7.58E-05	1149	0.333333	0.015473	SRSF6
4000.28	7.284245	-7068	7.35E-05	623	0.333333	0.008812	DAP3
4836.218	6.74838	-7068	7.39E-05	716	0.333333	0.009432	MRPL23
14148.69	8.788252	-7068	7.57E-05	1032	0.333333	0.014202	PGF
8036.252	7.039089	-7068	7.52E-05	935	0.333333	0.013922	NOTCH2
5363.665	8.487048	-7068	7.38E-05	663	0.333333	0.009406	HOXB2
26911.1	6.867392	-7068	7.93E-05	1729	0.333333	0.024794	BHLHE40
15320.71	6.088356	-7068	7.78E-05	1436	0.333333	0.021834	DLG1
7160.88	9.753317	-7068	7.37E-05	664	0.25	0.008367	LCN2
12589.41	7.562574	-7068	7.62E-05	1108	0.333333	0.015871	FGL1
30989.14	7.741705	-7068	7.95E-05	1761	0.333333	0.025808	PRKCE
5301.7	6.679595	-7068	7.45E-05	795	0.333333	0.012174	PSIP1

7382.014	6.921401	-7068	7.50E-05	895	0.333333	0.012921	GABARAPL1
7318.381	6.848535	-7068	7.50E-05	895	0.333333	0.012281	RGN
3011.725	7.86016	-7068	7.31E-05	530	0.333333	0.007609	CACNB3
5143.7	7.867179	-7068	7.40E-05	683	0.333333	0.010442	MRFAP1L1
12927.44	7.375854	-7068	7.63E-05	1146	0.333333	0.016945	VSNL1
5704.004	6.753543	-7068	7.44E-05	793	0.333333	0.010796	AIFM1
6513.687	7.631637	-7068	7.46E-05	796	0.333333	0.011329	CCR6
5482.75	8.530769	-7068	7.40E-05	672	0.333333	0.009407	TST
4937.765	6.522433	-7068	7.39E-05	744	0.333333	0.009542	BCL2L1
11752.26	7.841965	-7068	7.60E-05	1067	0.333333	0.015754	UBB
5240.933	7.5667	-7068	7.36E-05	655	0.333333	0.007482	S100A12
7491.54	6.769628	-7068	7.53E-05	942	0.333333	0.014447	GLS
12709.25	6.682937	-7068	7.68E-05	1228	0.333333	0.018411	PDHB
6614.835	9.720085	-7068	7.38E-05	638	0.333333	0.008303	FUT5
14373.98	6.406584	-7068	7.70E-05	1327	0.333333	0.019223	SNRPA1
5273.467	6.831572	-7068	7.42E-05	769	0.333333	0.011099	BCL10
4567.931	7.915425	-7068	7.38E-05	654	0.333333	0.009462	POLB
16942.78	6.029611	-7068	7.77E-05	1505	0.333333	0.020908	PRKCA
8807.46	6.825792	-7068	7.53E-05	1000	0.333333	0.014219	FHL2
4361.606	8.560522	-7068	7.36E-05	593	0.333333	0.008124	TSC22D4
4030.752	6.286901	-7068	7.39E-05	693	0.333333	0.010035	UFD1L
8198.635	8.109854	-7068	7.48E-05	849	0.333333	0.011715	TRIM21
14836.66	6.669422	-7068	7.69E-05	1300	0.333333	0.018627	COL11A1
4600.777	7.09558	-7068	7.34E-05	645	0.333333	0.007406	CTSG
11103.15	6.078089	-7068	7.62E-05	1191	0.333333	0.016136	STIP1
6188.899	10.26957	-7068	7.33E-05	576	0.333333	0.006697	MYOD1
5676.309	6.519815	-7068	7.44E-05	837	0.333333	0.01196	CRKL
7870.981	6.774992	-7068	7.49E-05	919	0.333333	0.011985	PPP1CA
7744.482	6.239825	-7068	7.55E-05	1011	0.333333	0.01569	CDC42BPA
6819.083	7.466609	-7068	7.46E-05	807	0.333333	0.010962	TIMM44
22947.98	7.017548	-7068	7.88E-05	1584	0.333333	0.023436	PAPPA
5902.743	9.251854	-7068	7.36E-05	625	0.333333	0.008464	APOD
10364.7	7.713686	-7068	7.54E-05	971	0.333333	0.013691	BBOX1
15746.43	7.764205	-7068	7.67E-05	1213	0.333333	0.017381	GRB14
5933.23	8.671261	-7068	7.39E-05	682	0.333333	0.009085	SGTA
2302.163	7.403851	-7068	7.30E-05	489	0.333333	0.007352	RIOK3
6453.886	6.399802	-7068	7.50E-05	905	0.333333	0.013835	HMGCR
36930.8	6.696533	-7068	8.17E-05	2084	0.333333	0.030854	NTRK2
3883.595	7.823091	-7068	7.35E-05	598	0.333333	0.008127	UCP2
8565.275	6.990407	-7068	7.54E-05	980	0.333333	0.014499	BAG2
10530.4	7.290663	-7068	7.58E-05	1041	0.333333	0.015187	DUSP5

4711.814	7.393791	-7068	7.39E-05	676	0.333333	0.009482	ZPR1
8582.267	6.974437	-7068	7.53E-05	947	0.333333	0.013109	TIMP1
10704.32	6.745717	-7068	7.63E-05	1122	0.333333	0.017334	TMEM87A
5247.986	7.543797	-7068	7.40E-05	714	0.333333	0.01039	CSPG4
7559.844	7.862241	-7068	7.49E-05	855	0.333333	0.011979	GSTO1
2484.896	9.747878	-7068	7.24E-05	386	0.333333	0.005222	AHSA2
24450.49	6.478266	-7068	7.97E-05	1752	0.333333	0.02676	NT5E
7744.303	7.480378	-7068	7.50E-05	879	0.333333	0.012968	PAX2
7099.495	4.690414	-7068	7.55E-05	1127	0.333333	0.015226	TRA2B
15876.64	7.167342	-7068	7.73E-05	1314	0.333333	0.019257	PYGL
5940.686	6.937388	-7068	7.45E-05	808	0.333333	0.011803	SEC11A
4907.673	5.969847	-7068	7.41E-05	783	0.333333	0.010153	PON1
13001.07	6.7363	-7068	7.69E-05	1238	0.333333	0.018782	STK39
5093.664	5.45241	-7068	7.43E-05	845	0.333333	0.010899	POLR2G
7729.928	7.098545	-7068	7.48E-05	875	0.333333	0.012235	COL16A1
2836.023	6.035416	-7068	7.36E-05	628	0.333333	0.010016	RTCA
5810.541	7.53086	-7068	7.45E-05	776	0.333333	0.012107	NPY1R
4495.36	10.80185	-7068	7.29E-05	480	0.333333	0.006159	IL9
6098.52	7.758697	-7068	7.44E-05	759	0.333333	0.010538	CLN3
13444.57	5.199248	-7068	7.71E-05	1459	0.333333	0.019548	CAV1
4034.843	8.3809	-7068	7.34E-05	576	0.333333	0.007917	CLC
7273.597	6.252264	-7068	7.53E-05	960	0.333333	0.014419	NID2
3440.547	9.666242	-7068	7.28E-05	463	0.333333	0.005832	ROM1
2282.794	5.561749	-7068	7.32E-05	579	0.333333	0.008773	ABI1
8503.346	7.814533	-7068	7.49E-05	864	0.333333	0.01169	FXYD3
9463.546	7.354913	-7068	7.56E-05	991	0.333333	0.015533	CFDP1
8971.502	8.242676	-7068	7.50E-05	869	0.333333	0.01173	KRT19
3188.433	8.506732	-7068	7.30E-05	504	0.333333	0.007063	GGA3
9482.359	7.763728	-7068	7.54E-05	948	0.333333	0.013801	WFS1
5819.292	8.47728	-7068	7.37E-05	666	0.333333	0.00905	NDUFC1
6940.459	8.351887	-7068	7.45E-05	768	0.333333	0.011035	SLC1A5
3748.238	10.26731	-7068	7.30E-05	477	0.333333	0.00614	INPP5K
4204.138	6.798159	-7068	7.36E-05	642	0.25	0.007905	SERPINA4
4898.789	11.05233	-7068	7.31E-05	514	0.333333	0.007	IFNB1
5481.735	6.265491	-7068	7.47E-05	837	0.333333	0.012934	CASK
16969.65	7.661634	-7068	7.72E-05	1282	0.333333	0.018743	KIFC3
15933.71	6.713736	-7068	7.73E-05	1376	0.333333	0.020254	SOS1
23680.23	6.290101	-7068	7.96E-05	1745	0.333333	0.026137	TCF7L2
5214.842	8.144032	-7068	7.38E-05	673	0.333333	0.009052	MKNK2
9328.459	6.427662	-7068	7.57E-05	1067	0.333333	0.0152	MMP3
11526.75	7.740627	-7068	7.60E-05	1064	0.333333	0.016385	POU4F1

2414.063	9.184046	-7068	7.26E-05	403	0.333333	0.004831	PIGQ
9166.693	7.708745	-7068	7.53E-05	932	0.333333	0.013562	INSM1
5205.324	7.672046	-7068	7.36E-05	665	0.333333	0.007964	TNNC1
7013.936	6.504981	-7068	7.49E-05	925	0.333333	0.013312	SMARCE1
12114.92	7.608036	-7068	7.63E-05	1110	0.333333	0.016611	TMSB10
5231.262	7.342197	-7068	7.40E-05	725	0.333333	0.009847	BATF
17894.37	5.465507	-7068	7.81E-05	1622	0.333333	0.021965	VRK1
7876.95	7.369742	-7068	7.49E-05	872	0.333333	0.012027	LAMB2
7711.491	6.775255	-7068	7.53E-05	948	0.333333	0.014201	SRI
7814.503	6.198112	-7068	7.57E-05	1035	0.333333	0.015885	PPP2R2A
2963.109	8.351398	-7068	7.30E-05	499	0.333333	0.006786	DEXI
11358.6	6.36593	-7068	7.62E-05	1172	0.333333	0.016656	INHBA
6988.316	6.903708	-7068	7.48E-05	859	0.333333	0.013207	SCAF11
8154.751	5.852487	-7068	7.59E-05	1089	0.333333	0.016643	ACTR2
4845.147	10.05203	-7068	7.34E-05	559	0.333333	0.007182	GALK1
5847.507	8.37748	-7068	7.39E-05	693	0.333333	0.009519	ZIC1
9893.959	6.405985	-7068	7.62E-05	1121	0.333333	0.017475	GLIPR1
3618.28	7.042328	-7068	7.37E-05	643	0.333333	0.00993	NFATC3
7721.78	7.582215	-7068	7.46E-05	839	0.333333	0.011467	CCR3
7887.555	7.201625	-7068	7.51E-05	923	0.333333	0.013298	PDK1
8045.207	7.847666	-7068	7.50E-05	861	0.333333	0.012071	AZGP1
7956.448	5.603743	-7068	7.58E-05	1105	0.333333	0.016569	RPS6KB1
6033.879	6.484475	-7068	7.42E-05	799	0.333333	0.009666	SERPIND1
6990.537	7.562931	-7068	7.47E-05	836	0.333333	0.012231	TFAP2A
10322.26	6.760567	-7068	7.61E-05	1097	0.333333	0.016513	PECAM1
3868.759	8.00526	-7068	7.35E-05	595	0.333333	0.009026	ZC3H14
10740.7	6.910771	-7068	7.60E-05	1090	0.333333	0.015984	SERPINB1
6114.866	8.027556	-7068	7.43E-05	741	0.333333	0.010473	ATP2A3
9831.717	7.584356	-7068	7.57E-05	997	0.333333	0.015185	CXCR5
12060.14	6.428683	-7068	7.67E-05	1224	0.333333	0.017995	ABAT
16075.21	7.518194	-7068	7.71E-05	1271	0.333333	0.018995	MSX1
14918.43	7.596622	-7068	7.66E-05	1217	0.333333	0.017773	CBS
5664.377	5.89608	-7068	7.43E-05	846	0.333333	0.011069	EXOSC9
8453.615	7.784311	-7068	7.52E-05	907	0.333333	0.013949	CNR1
2702.359	9.509438	-7068	7.27E-05	424	0.333333	0.005442	WNT6
8514.828	6.420852	-7068	7.57E-05	1039	0.333333	0.015866	EFNB2
8038.42	6.911512	-7068	7.54E-05	958	0.333333	0.014803	PRDM2
2851.119	5.424352	-7068	7.36E-05	664	0.333333	0.009886	TPR
4552.18	10.29389	-7068	7.32E-05	512	0.25	0.006233	OCEL1
5622.856	5.941729	-7068	7.42E-05	830	0.333333	0.010026	ITIH3
7554.268	9.276371	-7068	7.43E-05	730	0.333333	0.010338	SP4

17578.82	7.587445	-7068	7.73E-05	1294	0.333333	0.019379	DZIP1
9121.378	6.602911	-7068	7.58E-05	1049	0.333333	0.015328	FBN2
5333.788	7.530509	-7068	7.43E-05	735	0.333333	0.0109	PLA2G5
10015.9	7.041449	-7068	7.60E-05	1051	0.333333	0.015962	PGRMC2
11961.13	6.459463	-7068	7.68E-05	1233	0.333333	0.019278	ADAM9
3164.588	7.108262	-7068	7.33E-05	577	0.333333	0.008382	PMPCB
6175.56	8.469343	-7068	7.41E-05	709	0.333333	0.009263	GCHFR
7984.281	7.442468	-7068	7.51E-05	919	0.333333	0.013477	ABL2
9595.738	6.64965	-7068	7.53E-05	1026	0.333333	0.013148	NFKBIA
3137.445	6.421622	-7068	7.36E-05	629	0.333333	0.009405	EIF2A
5456.07	7.565046	-7068	7.41E-05	721	0.333333	0.010016	IL18
7637.404	6.91625	-7068	7.50E-05	919	0.333333	0.012676	MYBL2
3846.165	7.976583	-7068	7.36E-05	599	0.333333	0.009066	RASGRF1
716.0178	9.15342	-7068	7.14E-05	215	0.333333	0.00261	MUC5AC
3949.374	10.13871	-7068	7.30E-05	470	0.333333	0.005877	UPK2
17173.15	7.053397	-7068	7.75E-05	1371	0.333333	0.021151	IVNS1ABP
10662.58	6.230028	-7068	7.65E-05	1189	0.333333	0.018657	PTPRG
13657.38	6.728656	-7068	7.67E-05	1239	0.333333	0.018	GNAO1
4651.799	8.679014	-7068	7.35E-05	599	0.333333	0.007957	VEGFB
5507.362	7.893854	-7068	7.40E-05	717	0.333333	0.009833	HDAC6
22656.13	6.69912	-7068	7.87E-05	1617	0.333333	0.023232	FGFR2
9844.175	6.372522	-7068	7.59E-05	1104	0.333333	0.016176	CAPN2
6003.634	6.869185	-7068	7.44E-05	804	0.333333	0.010432	LBP
16295.35	6.524813	-7068	7.79E-05	1427	0.333333	0.022229	PIP4K2A
5516.374	7.309717	-7068	7.42E-05	763	0.333333	0.010864	SMARCB1
17908.82	6.980299	-7068	7.78E-05	1404	0.333333	0.021054	MYRIP
6288.922	6.725985	-7068	7.46E-05	853	0.333333	0.011535	P4HB
4856.524	8.238242	-7068	7.39E-05	646	0.333333	0.009384	WNT11
6456.175	6.955667	-7068	7.48E-05	853	0.333333	0.013123	ROCK2
5640.848	6.303184	-7068	7.46E-05	848	0.333333	0.012042	TIE1
6879.856	6.686478	-7068	7.51E-05	909	0.333333	0.01387	LCOR
9233.481	7.783071	-7068	7.53E-05	940	0.333333	0.013716	PEX5
6222.58	7.942631	-7068	7.44E-05	755	0.333333	0.011111	PPP1R10
7020.806	7.720013	-7068	7.44E-05	805	0.333333	0.011259	ID1
7907.236	5.422846	-7068	7.55E-05	1115	0.333333	0.01578	PTPN11
3908.139	6.273289	-7068	7.36E-05	695	0.333333	0.008951	DAXX
11625.71	6.345585	-7068	7.63E-05	1179	0.25	0.016359	TNFSF10
11853.35	6.413395	-7068	7.66E-05	1223	0.333333	0.017592	CD40
3358.156	12.10327	-7068	7.24E-05	368	0.333333	0.004065	AGRP
7063.449	6.79455	-7068	7.42E-05	831	0.333333	0.009406	KLKB1
9715.776	6.013889	-7068	7.60E-05	1143	0.333333	0.016102	CTSB

5720.087	6.77099	-7068	7.47E-05	826	0.333333	0.012548	ATF6
6077.451	5.847868	-7068	7.49E-05	910	0.333333	0.012615	VDAC2
10679.76	6.941558	-7068	7.62E-05	1098	0.333333	0.017088	DCT
9393.918	7.033873	-7068	7.57E-05	1023	0.333333	0.014975	PDLIM1
9355.844	6.217443	-7068	7.57E-05	1091	0.333333	0.01517	RFC2
5343.376	7.829855	-7068	7.40E-05	701	0.333333	0.010213	RAB9A
5984.076	9.989431	-7068	7.36E-05	616	0.333333	0.008381	PTGDS
5482.892	8.402507	-7068	7.39E-05	666	0.333333	0.009368	RBCK1
4103.796	8.009532	-7068	7.35E-05	585	0.333333	0.007537	PRKCSH
4106.507	8.175978	-7068	7.37E-05	610	0.333333	0.00926	FZD6
11792.86	6.7715	-7068	7.64E-05	1169	0.333333	0.017705	POSTN
9098.058	7.018697	-7068	7.56E-05	1012	0.333333	0.015517	UST
7171.987	8.643866	-7068	7.42E-05	740	0.333333	0.010138	MXD1
4010.995	7.27677	-7068	7.37E-05	641	0.333333	0.009799	ATMIN
5305.365	6.583238	-7068	7.42E-05	761	0.333333	0.010139	TAP1
3171.018	7.381495	-7068	7.31E-05	545	0.333333	0.006731	STX1A
7617.091	7.0829	-7068	7.48E-05	891	0.333333	0.012174	AGT
5582.673	6.243494	-7068	7.43E-05	805	0.333333	0.010692	COX7B
3844.123	8.33854	-7068	7.33E-05	562	0.333333	0.007757	ADORA1
8379.087	7.852822	-7068	7.49E-05	876	0.333333	0.01265	IBSP
8137.706	6.541449	-7068	7.54E-05	984	0.333333	0.014007	LGALS1
9645.476	6.250445	-7068	7.56E-05	1094	0.333333	0.015123	BCL2
9896.198	6.23386	-7068	7.62E-05	1142	0.333333	0.01684	MAP3K8
6366.667	8.090351	-7068	7.39E-05	713	0.333333	0.008783	CXCR3
14566.88	6.495074	-7068	7.74E-05	1346	0.333333	0.020277	BMP2K
17133.82	6.833465	-7068	7.81E-05	1434	0.333333	0.022332	CLTA
7263.307	6.80477	-7068	7.51E-05	916	0.333333	0.013107	PCCB
3382.816	6.806497	-7068	7.33E-05	580	0.333333	0.007443	MRPL40
6898.737	7.518632	-7068	7.47E-05	816	0.333333	0.011393	PLXNB2
13661.47	7.152859	-7068	7.69E-05	1200	0.333333	0.01872	PDZD2
6192.571	7.866201	-7068	7.45E-05	763	0.333333	0.011942	BEX4
4395.336	7.329703	-7068	7.38E-05	670	0.333333	0.009285	F8
4507.158	8.944142	-7068	7.36E-05	592	0.333333	0.008417	FCHO1
5326.591	8.458245	-7068	7.40E-05	670	0.333333	0.009237	TIMM17B
9764.292	7.871975	-7068	7.53E-05	934	0.333333	0.013152	MEP1A
6737.092	5.967649	-7068	7.54E-05	968	0.333333	0.014825	CYP1B1
9017.291	7.505934	-7068	7.55E-05	956	0.333333	0.014234	MBOAT7
3264.031	8.342663	-7068	7.31E-05	514	0.333333	0.00678	IL3
14987.55	7.002773	-7068	7.72E-05	1290	0.333333	0.019672	EPB41L3
4999.02	8.710452	-7068	7.37E-05	630	0.333333	0.008961	ABCD2
11999.13	7.083555	-7068	7.65E-05	1153	0.333333	0.017852	BCAR3

7768.259	6.125034	-7068	7.56E-05	1029	0.333333	0.015863	DNM1L
3644.765	8.209553	-7068	7.33E-05	557	0.333333	0.007799	LY9
20666.77	6.700362	-7068	7.86E-05	1562	0.333333	0.023066	PROX1
6201.806	5.706084	-7068	7.43E-05	880	0.333333	0.010568	KNG1
3438.362	6.368673	-7068	7.36E-05	636	0.333333	0.008643	SDHC
7399.398	6.062537	-7068	7.54E-05	1005	0.333333	0.014968	PSME4
6225.496	8.257679	-7068	7.40E-05	707	0.333333	0.009845	BCAS1
8045.691	6.14024	-7068	7.56E-05	1030	0.333333	0.015519	EDNRA
12311.6	7.373034	-7068	7.63E-05	1131	0.333333	0.016118	SDC1
6590.875	8.696492	-7068	7.42E-05	721	0.333333	0.009883	IL24
5238.483	7.963991	-7068	7.38E-05	675	0.333333	0.008977	LIF
7324.933	7.052507	-7068	7.49E-05	891	0.333333	0.012976	HERPUD1
5407.446	7.318718	-7068	7.42E-05	741	0.333333	0.01041	FUCA1
3751.049	7.063609	-7068	7.32E-05	607	0.333333	0.007602	LRP2
5264.752	5.71226	-7068	7.45E-05	839	0.333333	0.011543	GBP1
3944.412	6.991971	-7068	7.35E-05	628	0.333333	0.007768	PKLR
4616.341	6.277632	-7068	7.45E-05	791	0.333333	0.012688	WTAP
8011.049	9.098097	-7068	7.44E-05	781	0.333333	0.010982	CDKN2C
6694.793	6.463614	-7068	7.50E-05	916	0.333333	0.014188	AKT3
13379.17	6.803926	-7068	7.67E-05	1237	0.333333	0.017831	ACVRL1
8274.334	7.102096	-7068	7.54E-05	957	0.333333	0.014275	LGMN
18855.66	6.830536	-7068	7.82E-05	1476	0.333333	0.022219	KANK1
8590.045	9.238275	-7068	7.45E-05	793	0.333333	0.010944	SCGB1A1
12455.72	6.337166	-7068	7.69E-05	1258	0.333333	0.019124	SYNE2
4993.613	6.528803	-7068	7.42E-05	737	0.333333	0.00988	STOML2
6240.46	7.705577	-7068	7.43E-05	778	0.333333	0.011167	IL1A
16026.47	7.02005	-7068	7.74E-05	1334	0.333333	0.020159	LMO7
3544.29	9.629263	-7068	7.29E-05	482	0.333333	0.006591	DLX5
13181.92	6.548861	-7068	7.69E-05	1260	0.333333	0.018819	PLSCR1
7897.44	7.506164	-7068	7.51E-05	897	0.333333	0.013723	PMAIP1
9753.506	6.502968	-7068	7.61E-05	1129	0.333333	0.017051	PPP2R1B
5217.242	6.446236	-7068	7.42E-05	794	0.333333	0.010753	FADD
2749.379	7.623435	-7068	7.31E-05	510	0.333333	0.007448	APPBP2
9953.407	6.281559	-7068	7.60E-05	1133	0.333333	0.01628	REL
4208.143	6.995146	-7068	7.39E-05	677	0.333333	0.009884	MAP1LC3B
3890.859	9.284616	-7068	7.31E-05	518	0.333333	0.007152	CLUL1
10002.48	6.49731	-7068	7.63E-05	1122	0.333333	0.017727	CDYL
5629.547	6.298679	-7068	7.41E-05	787	0.333333	0.009489	CCR5
8927.857	6.441096	-7068	7.54E-05	1032	0.333333	0.01406	EGR1
4503.946	8.789108	-7068	7.35E-05	599	0.333333	0.008356	PIAS3
3438.782	7.176007	-7068	7.35E-05	604	0.333333	0.009003	ENTPD3

3721.598	7.561209	-7068	7.36E-05	606	0.333333	0.008866	DISC1
16469.44	6.636848	-7068	7.71E-05	1377	0.333333	0.019215	TTK
5720.764	6.894082	-7068	7.44E-05	786	0.333333	0.011222	NPR3
5195.894	6.662035	-7068	7.45E-05	792	0.333333	0.011975	LYST
12738.62	6.839731	-7068	7.70E-05	1227	0.333333	0.019264	OSBPL3
7536.136	6.710899	-7068	7.53E-05	960	0.333333	0.014889	VAPA
5396.877	7.096173	-7068	7.45E-05	772	0.333333	0.011911	BAMBI
7360.692	5.803169	-7068	7.54E-05	1026	0.333333	0.014683	SFPQ
8841.842	7.786586	-7068	7.51E-05	910	0.333333	0.012842	EPHA1
7877.266	5.169617	-7068	7.55E-05	1102	0.333333	0.014433	AURKB
6122.261	8.771649	-7068	7.40E-05	686	0.25	0.009727	CALB1
5957.404	6.91096	-7068	7.48E-05	829	0.333333	0.013316	GAPVD1
4323.345	6.865781	-7068	7.40E-05	717	0.333333	0.010612	PRKAR2A
5740.382	6.616618	-7068	7.48E-05	840	0.333333	0.012841	AGPS
3998.883	7.702012	-7068	7.36E-05	630	0.333333	0.008783	ELK1
3225.607	7.327575	-7068	7.34E-05	570	0.333333	0.008332	EXOC3
16531.99	6.818977	-7068	7.75E-05	1368	0.333333	0.02011	PDE5A
14695.36	7.423131	-7068	7.64E-05	1221	0.333333	0.017181	NDUFS1
11718.63	10.03789	-7068	7.50E-05	859	0.333333	0.012174	CRABP2
7718.794	7.500128	-7068	7.50E-05	884	0.333333	0.013379	APAF1
8884.391	6.571281	-7068	7.53E-05	1027	0.333333	0.013641	RARA
7156.975	9.01473	-7068	7.43E-05	722	0.333333	0.009802	SCN5A
16745.41	6.379275	-7068	7.74E-05	1408	0.333333	0.018834	SERPING1
7041.791	6.770959	-7068	7.50E-05	912	0.333333	0.013853	CREM
15678.61	6.312299	-7068	7.77E-05	1418	0.333333	0.021267	NEDD4L
8417.207	7.362965	-7068	7.54E-05	942	0.333333	0.013699	TPMT
9269.296	6.500199	-7068	7.60E-05	1079	0.333333	0.016705	EPB41L2
4559.432	8.971764	-7068	7.36E-05	595	0.333333	0.008566	INTS3
6090.739	6.360441	-7068	7.49E-05	872	0.333333	0.012397	PRDX1
12990.67	5.913265	-7068	7.73E-05	1361	0.333333	0.020947	SC5D
6669.648	5.100647	-7068	7.50E-05	999	0.333333	0.012967	ITGB3BP
5153.679	6.539819	-7068	7.43E-05	787	0.333333	0.01136	STK17A
2849.06	8.390701	-7068	7.28E-05	474	0.333333	0.006826	ZNF148
6575.002	9.146518	-7068	7.41E-05	676	0.333333	0.008933	P2RX6
6555.171	5.736073	-7068	7.47E-05	936	0.25	0.012574	CFH
5509.552	9.184758	-7068	7.35E-05	592	0.333333	0.006846	SLURP1
10599.05	6.839269	-7068	7.57E-05	1068	0.333333	0.014184	C6
1801.466	6.596279	-7068	7.28E-05	469	0.333333	0.007313	RNMT
4688.6	6.144767	-7068	7.40E-05	760	0.333333	0.009908	CD247
7667.504	7.845283	-7068	7.48E-05	853	0.333333	0.012072	MDK
6518.627	8.562383	-7068	7.41E-05	722	0.333333	0.010547	NDUFA5

5983.217	6.291123	-7068	7.51E-05	893	0.333333	0.014424	TMED5
7952.878	8.995892	-7068	7.44E-05	763	0.333333	0.010897	CADM3
6511.894	7.948972	-7068	7.44E-05	771	0.333333	0.01105	FCER1A
3622.183	7.442166	-7068	7.35E-05	577	0.333333	0.007748	WDR18
6306.593	8.540942	-7068	7.43E-05	721	0.333333	0.009681	POR
6168.856	6.190696	-7068	7.51E-05	915	0.333333	0.014158	RRP1B
11863.19	6.28159	-7068	7.67E-05	1246	0.333333	0.018331	CTSC
5379.039	6.35514	-7068	7.41E-05	783	0.333333	0.010177	HLA-G
5347.154	6.918612	-7068	7.44E-05	784	0.333333	0.011651	HIRA
4401.226	7.253254	-7068	7.40E-05	692	0.333333	0.010412	ARHGAP35
9485.046	7.691601	-7068	7.53E-05	930	0.333333	0.013776	TUBGCP4
3139.445	5.834163	-7068	7.36E-05	637	0.333333	0.008558	FARSA
5154.505	8.793665	-7068	7.36E-05	613	0.333333	0.008127	PIP
7743.112	6.781962	-7068	7.55E-05	958	0.333333	0.015112	OPA1
8719.608	7.111969	-7068	7.53E-05	964	0.333333	0.013793	ABCB4
4649.155	9.701364	-7068	7.33E-05	553	0.333333	0.007276	OVGP1
11225.57	6.744254	-7068	7.66E-05	1165	0.333333	0.018137	MPRIP
12814.76	5.740179	-7068	7.72E-05	1364	0.333333	0.020046	CALD1
3376.437	8.69513	-7068	7.31E-05	497	0.333333	0.006501	GHRH
3674.891	10.07007	-7068	7.30E-05	473	0.333333	0.005905	SLC6A3
5230.172	7.986727	-7068	7.40E-05	694	0.333333	0.010016	EFNB1
6536.686	7.34422	-7068	7.47E-05	830	0.333333	0.012127	BAG1
3609.674	11.93399	-7068	7.24E-05	391	0.333333	0.004913	KCNV2
2562.47	4.524671	-7068	7.35E-05	674	0.333333	0.009359	SRSF10
9007.788	4.013698	-7068	7.62E-05	1355	0.333333	0.016331	CCNB1
4882.291	7.94126	-7068	7.39E-05	677	0.333333	0.009431	GPC1
11177.15	6.261611	-7068	7.68E-05	1219	0.333333	0.01887	TSN
6379.868	10.72045	-7068	7.36E-05	595	0.333333	0.008293	SGSM3
3490.37	6.591183	-7068	7.35E-05	647	0.333333	0.008664	STAT6
4021.339	9.316803	-7068	7.31E-05	526	0.333333	0.006962	TSPAN1
9789.056	6.524052	-7068	7.60E-05	1102	0.333333	0.016691	USP10
6921.717	8.628693	-7068	7.41E-05	725	0.333333	0.010005	ZIC3
11411.58	6.48246	-7068	7.66E-05	1194	0.333333	0.018448	ACSL4
3635.347	7.456216	-7068	7.36E-05	586	0.333333	0.008615	YARS2
4793.152	10.66198	-7068	7.31E-05	498	0.333333	0.007047	RPGRIP1
5378.509	8.015763	-7068	7.41E-05	708	0.333333	0.010605	MFN2
7014.858	7.90016	-7068	7.45E-05	791	0.333333	0.011339	RXRG
4729.9	9.213608	-7068	7.35E-05	596	0.333333	0.008149	KYAT1
13150.55	7.901838	-7068	7.65E-05	1122	0.333333	0.016232	TALDO1
3208.398	7.733096	-7068	7.33E-05	560	0.333333	0.008505	PIP4K2B
4630.366	8.990435	-7068	7.35E-05	585	0.333333	0.008314	DEFA6

11176.8	7.305096	-7068	7.59E-05	1062	0.333333	0.01544	HNF1B
15902.12	6.809059	-7068	7.75E-05	1346	0.333333	0.02074	RNF144A
4890.419	9.951629	-7068	7.34E-05	551	0.333333	0.007233	PTMS
6541.573	6.919081	-7068	7.49E-05	876	0.333333	0.013509	GLO1
5375.609	9.670539	-7068	7.35E-05	581	0.333333	0.007282	MYLPF
7679.029	7.029577	-7068	7.52E-05	935	0.333333	0.014062	PIR
9938.648	6.535122	-7068	7.61E-05	1111	0.333333	0.016653	SGK1
7371.733	6.81254	-7068	7.51E-05	923	0.333333	0.013356	CTSL
9741.47	6.805072	-7068	7.59E-05	1070	0.333333	0.015854	CLU
10011.56	7.143094	-7068	7.56E-05	1020	0.333333	0.013877	FAH
8295.737	5.83309	-7068	7.57E-05	1079	0.333333	0.015458	ATP5G3
6309.015	6.030355	-7068	7.48E-05	896	0.333333	0.012369	PRELP
6679.66	6.13059	-7068	7.48E-05	910	0.333333	0.012699	AOC3
4520.152	8.487386	-7068	7.36E-05	614	0.333333	0.00863	FADS3
4972.724	10.24716	-7068	7.33E-05	555	0.333333	0.007024	LTB4R
4143.782	8.666449	-7068	7.33E-05	567	0.333333	0.008041	ULK1
7754.222	6.300065	-7068	7.51E-05	991	0.333333	0.013345	UBE2I
2837.507	7.70558	-7068	7.31E-05	521	0.333333	0.007689	IFIT5
5243.498	7.008351	-7068	7.44E-05	757	0.333333	0.011739	FBXL5
4739.041	6.720768	-7068	7.42E-05	730	0.333333	0.010481	TIMP2
9722.271	7.097788	-7068	7.56E-05	1024	0.333333	0.015377	CAP2
10836.29	6.138195	-7068	7.59E-05	1154	0.333333	0.015231	APOH
1814.295	7.191242	-7068	7.26E-05	431	0.333333	0.006163	ATG13
4083.355	7.622292	-7068	7.37E-05	619	0.333333	0.008284	PFKL
2786.119	6.775976	-7068	7.30E-05	552	0.333333	0.007872	RAD50
11487.98	6.264611	-7068	7.66E-05	1230	0.333333	0.018213	TFDP1
5821.623	6.263685	-7068	7.48E-05	881	0.333333	0.013286	EIF5
10911.31	7.167106	-7068	7.58E-05	1069	0.333333	0.01503	TRIM27
10177.78	6.609589	-7068	7.61E-05	1109	0.333333	0.016962	LIFR
6749.01	7.74062	-7068	7.44E-05	802	0.333333	0.011564	PPARD
7443.649	5.180883	-7068	7.52E-05	1058	0.333333	0.013561	MMP2
36210.73	20.05036	-7068	7.53E-05	905	0.333333	0.01362	PIWIL1
3324.042	8.244256	-7068	7.32E-05	535	0.333333	0.007433	ALOX15
17325.46	11.82626	-7068	7.50E-05	865	0.333333	0.012386	MPC2
14055.62	7.125743	-7068	7.69E-05	1235	0.333333	0.018686	DCBLD2
3155.524	9.240444	-7068	7.30E-05	477	0.333333	0.006379	CAMP
20654.87	6.011194	-7068	7.89E-05	1669	0.333333	0.023982	JAG1
9971.871	6.782996	-7068	7.59E-05	1066	0.333333	0.016049	PCSK5
9633.858	6.589158	-7068	7.61E-05	1099	0.333333	0.017088	CD47
4134.165	9.413728	-7068	7.31E-05	518	0.333333	0.006538	NRTN
3084.565	7.825924	-7068	7.30E-05	510	0.333333	0.00629	MYBPC2

7525.316	7.026295	-7068	7.50E-05	899	0.333333	0.012657	ALDH2
13236.31	6.771113	-7068	7.71E-05	1263	0.333333	0.019763	MMD
13102.99	6.715901	-7068	7.70E-05	1252	0.333333	0.019095	TNFRSF21
2982.432	10.30367	-7068	7.25E-05	391	0.333333	0.00468	PDE6H
9289.646	6.260113	-7068	7.61E-05	1107	0.333333	0.016708	GCLC
5196.777	7.812063	-7068	7.38E-05	682	0.333333	0.009244	PAX8
7467.804	6.649109	-7068	7.51E-05	934	0.333333	0.013643	MTHFD2
4233.544	9.905651	-7068	7.30E-05	498	0.333333	0.006587	FABP7
6092.398	5.949449	-7068	7.47E-05	899	0.333333	0.012908	VDAC3
5287.693	7.470941	-7068	7.43E-05	739	0.333333	0.011106	SRD5A1
6971.742	8.007064	-7068	7.46E-05	810	0.333333	0.011722	PC
7231.481	6.670069	-7068	7.51E-05	929	0.333333	0.013433	LMNA
13956.12	7.709005	-7068	7.67E-05	1178	0.333333	0.017675	MGMT
9267.746	6.403963	-7068	7.60E-05	1088	0.333333	0.016715	PRPS2
4623.033	6.946536	-7068	7.40E-05	713	0.333333	0.010536	AHCTF1
3539.973	7.228929	-7068	7.35E-05	614	0.333333	0.0093	SPOP
2311.784	6.435939	-7068	7.32E-05	541	0.333333	0.008557	RYBP
1979.661	7.206486	-7068	7.26E-05	444	0.333333	0.005825	CD22
7423.648	8.367678	-7068	7.44E-05	774	0.333333	0.011244	CFAP20
7434.296	8.228888	-7068	7.48E-05	826	0.333333	0.012302	TMSB4X
10156.9	7.225327	-7068	7.59E-05	1048	0.333333	0.01644	LPIN1
3850.368	11.77582	-7068	7.26E-05	419	0.333333	0.005498	PDE6C
2869.504	8.76507	-7068	7.29E-05	468	0.333333	0.006355	TPPP
2931.65	8.17462	-7068	7.31E-05	508	0.333333	0.00684	HMOX2
11626.59	6.983249	-7068	7.63E-05	1135	0.25	0.016935	BCHE
21558.19	6.967857	-7068	7.81E-05	1525	0.333333	0.021096	PPARG
12203.26	7.848887	-7068	7.60E-05	1068	0.333333	0.016223	COX6C
4235.513	7.961086	-7068	7.37E-05	624	0.333333	0.009218	P2RY1
7492.002	8.245536	-7068	7.46E-05	799	0.333333	0.011451	ADIPOQ
3446.848	7.451156	-7068	7.35E-05	599	0.333333	0.00895	SLC23A2
4289.476	6.321056	-7068	7.37E-05	692	0.333333	0.008527	POLR2I
8336.249	7.654101	-7068	7.51E-05	911	0.333333	0.013639	NOS1
14357.84	7.066954	-7068	7.72E-05	1274	0.333333	0.01966	UCHL1
10969.55	6.302496	-7068	7.60E-05	1146	0.333333	0.016028	COL6A3
5625.994	5.950156	-7068	7.45E-05	852	0.333333	0.011578	CYP2A6
3459.92	7.796805	-7068	7.31E-05	557	0.333333	0.007105	GRAP2
4778.265	7.267121	-7068	7.41E-05	709	0.333333	0.010467	ADIPOR2
16641.59	7.378181	-7068	7.71E-05	1295	0.333333	0.018204	PCK1
8952.578	7.653861	-7068	7.53E-05	932	0.333333	0.013583	RORB
10573.71	7.279313	-7068	7.60E-05	1064	0.333333	0.015893	PLP2
7135.229	7.275338	-7068	7.48E-05	865	0.333333	0.012468	CAMKK2

9763.012	8.403122	-7068	7.54E-05	921	0.333333	0.013842	UPP1
6690.22	6.49266	-7068	7.52E-05	918	0.333333	0.014396	PPM1A
4698.073	7.000496	-7068	7.42E-05	731	0.333333	0.011136	SEC14L1
6406.963	8.089489	-7068	7.41E-05	723	0.333333	0.009449	DDC
8404.441	8.064936	-7068	7.50E-05	862	0.333333	0.01273	SCTR
6439.392	7.247083	-7068	7.44E-05	812	0.333333	0.010899	MAPK11
9349.833	5.202356	-7068	7.60E-05	1216	0.333333	0.016279	TOP2A
8017.566	6.823627	-7068	7.52E-05	946	0.333333	0.013444	SDHB
5601.854	10.58835	-7068	7.34E-05	554	0.333333	0.008327	SPAG1
3291.468	8.077691	-7068	7.32E-05	546	0.333333	0.007699	LIMK1
5374.531	6.4082	-7068	7.44E-05	806	0.333333	0.011943	UBE2N
3728.078	10.4097	-7068	7.27E-05	438	0.333333	0.004758	CTRL
2606.992	7.646318	-7068	7.28E-05	490	0.333333	0.006697	IL23A
5169.978	7.605129	-7068	7.41E-05	698	0.333333	0.009807	CCR7
2367.979	9.192179	-7068	7.25E-05	405	0.333333	0.005272	SLC30A4
5779.89	8.327479	-7068	7.37E-05	660	0.333333	0.00833	SULT1A1
12330.06	6.974402	-7068	7.64E-05	1156	0.333333	0.016474	SPP2
4075.346	8.827244	-7068	7.34E-05	571	0.333333	0.008361	SLC14A1
9458.108	6.253072	-7068	7.59E-05	1104	0.333333	0.016362	ATP2A2
4262.4	7.5259	-7068	7.38E-05	653	0.333333	0.009684	REST
8278.933	5.424689	-7068	7.55E-05	1102	0.333333	0.014923	IL1R1
2058.315	8.753182	-7068	7.24E-05	393	0.333333	0.005035	FLT3LG
2336.397	5.882151	-7068	7.33E-05	548	0.333333	0.008747	YTHDF3
5852.578	6.688184	-7068	7.45E-05	830	0.333333	0.01204	FOXO3
3571.462	7.377976	-7068	7.36E-05	601	0.333333	0.008755	RNASE6
6022.766	7.695527	-7068	7.44E-05	754	0.333333	0.010687	C3AR1
8294.887	7.17179	-7068	7.53E-05	949	0.333333	0.013986	MEF2D
3241.277	8.252217	-7068	7.30E-05	511	0.333333	0.006661	CUBN
6363.145	7.910127	-7068	7.44E-05	763	0.333333	0.010665	VAT1
4906.787	8.134427	-7068	7.38E-05	654	0.333333	0.008811	CETP
4356.335	7.495761	-7068	7.38E-05	662	0.333333	0.009659	BRAP
6434.336	7.676141	-7068	7.44E-05	785	0.333333	0.010976	GSTT1
8221.048	6.69093	-7068	7.57E-05	1001	0.333333	0.015444	TPD52
6100.687	6.922697	-7068	7.40E-05	761	0.333333	0.009009	UQCRQ
3843.172	7.687789	-7068	7.36E-05	608	0.333333	0.009316	ARID4A
7038.741	7.248262	-7068	7.48E-05	848	0.333333	0.01221	ALDH1A1
4017.587	6.852137	-7068	7.36E-05	659	0.333333	0.008743	SLC25A6
14553.43	6.999501	-7068	7.70E-05	1271	0.333333	0.019482	TUSC3
6751.085	9.611161	-7068	7.40E-05	666	0.333333	0.008695	PLCD1
6344.732	7.205977	-7068	7.41E-05	774	0.333333	0.009861	PF4
6058.449	6.028138	-7068	7.48E-05	885	0.333333	0.012281	CASP4

17813.57	6.786718	-7068	7.77E-05	1442	0.333333	0.020403	CEBPA
3565.007	7.809894	-7068	7.34E-05	578	0.333333	0.008246	TLE2
11862.59	5.943068	-7068	7.68E-05	1278	0.333333	0.018646	PTGIS
9021.364	9.18927	-7068	7.46E-05	803	0.333333	0.011033	NKX2-2
3776.858	8.915106	-7068	7.31E-05	529	0.333333	0.006752	MVD
5970.707	7.819988	-7068	7.42E-05	745	0.333333	0.010478	CD80
9912.258	7.002742	-7068	7.55E-05	1044	0.333333	0.014102	ORC1
10971.81	6.305272	-7068	7.66E-05	1204	0.333333	0.018627	GJA1
4227.659	6.329324	-7068	7.36E-05	673	0.333333	0.008189	SLC22A1
3876.363	7.757768	-7068	7.33E-05	584	0.333333	0.008156	RHAG
19666.2	6.740057	-7068	7.82E-05	1516	0.333333	0.02236	IRS2
10951.16	6.717355	-7068	7.58E-05	1100	0.333333	0.014558	AHSG
6670.929	7.031982	-7068	7.46E-05	830	0.333333	0.011297	S100B
6343.589	6.187959	-7068	7.50E-05	919	0.333333	0.013378	MSN
5431.795	8.160742	-7068	7.39E-05	699	0.333333	0.009676	GADD45G
7046.689	7.375986	-7068	7.46E-05	833	0.333333	0.012088	RAB27A
3687.39	8.324164	-7068	7.31E-05	529	0.333333	0.00661	HOXD10
15249.55	6.229551	-7068	7.74E-05	1393	0.333333	0.01984	FEN1
4130.8	9.711262	-7068	7.30E-05	501	0.333333	0.006234	IL13
9202.613	5.718028	-7068	7.61E-05	1157	0.333333	0.016919	CD44
8109.288	6.412966	-7068	7.53E-05	994	0.333333	0.013953	VDR
6505.671	8.044305	-7068	7.42E-05	736	0.333333	0.010067	PRF1
9879.416	6.653636	-7068	7.60E-05	1089	0.333333	0.016645	CCNT2
2483.699	9.603792	-7068	7.26E-05	411	0.333333	0.005726	ADPRH
5251.017	7.844742	-7068	7.41E-05	706	0.333333	0.010073	CRIP1
4397.221	7.161133	-7068	7.40E-05	696	0.333333	0.010696	ERC1
3501.213	8.998004	-7068	7.32E-05	519	0.333333	0.007431	MTMR11
4887.556	8.555711	-7068	7.36E-05	617	0.333333	0.008384	CA4
4272.273	7.970601	-7068	7.36E-05	619	0.333333	0.008403	TNIP1
7916.768	7.071818	-7068	7.53E-05	944	0.333333	0.014793	HDAC9
6076.199	6.622314	-7068	7.43E-05	793	0.333333	0.01074	CGA
14035.1	7.905571	-7068	7.65E-05	1142	0.333333	0.017385	CHST2
11178.24	6.789512	-7068	7.65E-05	1157	0.333333	0.018112	ZMIZ1
4448.492	7.297518	-7068	7.36E-05	645	0.333333	0.00841	FCN1
12123.32	8.843917	-7068	7.57E-05	974	0.333333	0.013565	ATP6V0E2
3754.694	8.168803	-7068	7.35E-05	582	0.333333	0.008353	ERF
10886.45	6.406435	-7068	7.64E-05	1158	0.333333	0.017083	KPNA2
4264.737	11.60461	-7068	7.28E-05	447	0.333333	0.005441	IL1RL2
13588.86	6.544806	-7068	7.71E-05	1297	0.333333	0.019499	SCD
5488.174	7.485356	-7068	7.42E-05	741	0.333333	0.010979	FGF5
4303.007	7.270673	-7068	7.39E-05	679	0.333333	0.010664	CD46

18780.16	6.37462	-7068	7.86E-05	1562	0.333333	0.024192	SLC1A1
6725.534	8.066075	-7068	7.45E-05	791	0.333333	0.011794	MEST
6387.09	7.20579	-7068	7.45E-05	806	0.333333	0.010884	EPHX1
7938.353	6.699031	-7068	7.55E-05	984	0.333333	0.01547	PDE4B
2431.884	8.534926	-7068	7.26E-05	440	0.333333	0.006374	BAG5
17446.24	6.876472	-7068	7.76E-05	1403	0.333333	0.020196	SMAD6
8507.105	5.831417	-7068	7.57E-05	1088	0.333333	0.015941	FGF7
3118.817	7.29529	-7068	7.34E-05	577	0.333333	0.008479	FBXO7
13748.53	6.454042	-7068	7.73E-05	1313	0.333333	0.020191	RAB31
7956.324	6.462403	-7068	7.55E-05	995	0.25	0.015311	RGS5
5141.738	7.841529	-7068	7.41E-05	700	0.333333	0.010497	RBM19
5850.493	7.424495	-7068	7.46E-05	780	0.333333	0.012275	COX20
7347.61	7.175989	-7068	7.51E-05	899	0.333333	0.013449	DBT
4739.837	9.16892	-7068	7.35E-05	578	0.333333	0.007866	MYBPH
5292.584	8.02167	-7068	7.39E-05	691	0.333333	0.009383	CYP1A1
6523.961	7.103533	-7068	7.49E-05	849	0.333333	0.012873	PEA15
13149.59	6.90253	-7068	7.68E-05	1231	0.333333	0.019051	RAD51B
3434.779	8.408954	-7068	7.31E-05	524	0.333333	0.006969	METTL1
7718.366	7.323806	-7068	7.49E-05	889	0.333333	0.012438	KRT18
14509.71	7.688445	-7068	7.66E-05	1182	0.333333	0.017463	MARCKSL1
2518.674	7.962515	-7068	7.28E-05	480	0.333333	0.006733	WASL
22269.22	6.684516	-7068	7.91E-05	1626	0.333333	0.024471	ERBB4
11216.65	7.450387	-7068	7.59E-05	1047	0.333333	0.014875	CD93
16162.27	6.377069	-7068	7.81E-05	1445	0.333333	0.02284	ESRRG
3470.918	5.997405	-7068	7.39E-05	683	0.333333	0.010599	PCMT1
10788.62	6.128728	-7068	7.63E-05	1180	0.333333	0.016736	KIF23
6246.386	7.529749	-7068	7.45E-05	783	0.333333	0.011279	PLK3
29305.42	6.439395	-7068	8.03E-05	1892	0.333333	0.027438	ANXA3
4896.813	7.475419	-7068	7.41E-05	715	0.333333	0.010936	RPP38
7641.823	12.52245	-7068	7.34E-05	553	0.333333	0.006283	LMF2
5632.504	9.280877	-7068	7.38E-05	641	0.333333	0.008989	GDPD5
7250.426	7.504361	-7068	7.49E-05	855	0.333333	0.012811	DYNC1I1
11693.81	6.930932	-7068	7.62E-05	1136	0.333333	0.016385	SLCO2A1
6033.572	6.311403	-7068	7.47E-05	868	0.333333	0.012656	UBE2D3
2644.222	9.063926	-7068	7.27E-05	443	0.333333	0.005867	TH
3503.241	7.786962	-7068	7.34E-05	579	0.333333	0.008711	GREB1
8782.998	7.461287	-7068	7.52E-05	933	0.333333	0.013257	SERPINA3
4233.511	9.124785	-7068	7.31E-05	539	0.333333	0.007302	NKX2-1
2775.835	7.629544	-7068	7.30E-05	508	0.333333	0.007993	MGAT2
5333.611	6.137911	-7068	7.42E-05	823	0.333333	0.011044	IKBKB
8945.572	6.731957	-7068	7.58E-05	1024	0.333333	0.015711	SFRP1

2818.53	8.773983	-7068	7.28E-05	470	0.333333	0.006335	FCGBP
6902.73	8.097681	-7068	7.44E-05	783	0.333333	0.010902	ITPR3
9339.047	7.489834	-7068	7.53E-05	955	0.333333	0.01393	IL33
6580.732	7.341593	-7068	7.45E-05	791	0.333333	0.010216	HMGCS2
15684.91	6.584257	-7068	7.77E-05	1384	0.333333	0.021744	DLC1
6819.238	9.383057	-7068	7.40E-05	667	0.333333	0.008453	UPK1A
13009.94	6.463165	-7068	7.68E-05	1259	0.333333	0.018592	CALCRL
3913.705	7.214531	-7068	7.36E-05	648	0.333333	0.009485	TADA2A
5807.379	5.775928	-7068	7.47E-05	892	0.333333	0.012519	PSMA2
7623.675	5.942896	-7068	7.53E-05	1003	0.333333	0.013697	NCAPD2
5787.802	6.683107	-7068	7.44E-05	801	0.25	0.010817	C7
7298.786	10.5866	-7068	7.37E-05	617	0.333333	0.00757	PMVK
6962.96	7.226857	-7068	7.48E-05	855	0.333333	0.012191	STX4
3846.592	8.053699	-7068	7.35E-05	580	0.333333	0.007843	MRPL49
14083.43	5.512711	-7068	7.76E-05	1458	0.333333	0.020802	MCM4
6507.426	5.191948	-7068	7.48E-05	975	0.333333	0.012355	COL6A2
5362.61	8.691821	-7068	7.39E-05	645	0.333333	0.008763	GJB3
3983.939	5.76339	-7068	7.36E-05	711	0.333333	0.008152	CYP2E1
4016.922	6.977023	-7068	7.37E-05	653	0.333333	0.009603	PAK3
4276.805	5.103373	-7068	7.40E-05	815	0.333333	0.010325	RACK1
15383.43	6.660924	-7068	7.76E-05	1370	0.333333	0.020795	WDR1
4950.188	7.558908	-7068	7.42E-05	705	0.333333	0.010151	PLEKHG3
4957.366	6.992782	-7068	7.40E-05	733	0.333333	0.009785	JUND
10966.05	6.866274	-7068	7.58E-05	1096	0.333333	0.015249	MTOR
9944.679	6.825959	-7068	7.60E-05	1094	0.333333	0.016432	IGF2BP3
2615.036	6.255337	-7068	7.31E-05	574	0.333333	0.007744	SRF
12542.33	5.870434	-7068	7.63E-05	1270	0.333333	0.015493	PLG
6269.987	6.364284	-7068	7.50E-05	892	0.333333	0.013382	PTPRE
9711.932	6.460873	-7068	7.60E-05	1105	0.333333	0.016855	TRIM24
6258.385	11.57678	-7068	7.33E-05	568	0.333333	0.007546	ZNF239
5942.247	7.847158	-7068	7.44E-05	751	0.333333	0.011432	TRPA1
3461.832	9.078808	-7068	7.30E-05	502	0.333333	0.006634	RORC
9940.508	6.655817	-7068	7.60E-05	1087	0.333333	0.016643	SEMA3C
9182.528	7.040437	-7068	7.56E-05	1009	0.333333	0.015481	TSC22D2
6156.409	6.671603	-7068	7.49E-05	870	0.333333	0.013636	API5
16373.48	5.737689	-7068	7.81E-05	1548	0.333333	0.022541	PDGFRA
5156.848	6.908242	-7068	7.41E-05	746	0.333333	0.010514	NDUFAF1
4014.936	6.625588	-7068	7.39E-05	695	0.333333	0.010253	EIF2AK2
3137.691	7.387803	-7068	7.35E-05	578	0.333333	0.009006	TNFAIP1
2806.87	6.867549	-7068	7.31E-05	545	0.333333	0.007088	CYP2D6
5942.567	9.171516	-7068	7.39E-05	655	0.333333	0.009311	SLC18A2

8595.905	7.070746	-7068	7.53E-05	955	0.333333	0.013818	ZFP36
5461.831	6.791187	-7068	7.40E-05	755	0.333333	0.009291	RAC2
5459.619	9.243758	-7068	7.37E-05	625	0.333333	0.008307	TRAPPC6A
10267.66	6.578229	-7068	7.60E-05	1102	0.333333	0.01644	IDS
15288.46	8.082387	-7068	7.66E-05	1173	0.333333	0.017447	LRIG1
14862.43	6.642066	-7068	7.71E-05	1325	0.333333	0.019165	IGF1R
2905.534	8.740747	-7068	7.28E-05	459	0.333333	0.006316	SYT17
12720.96	6.310795	-7068	7.70E-05	1276	0.333333	0.018995	CEBPD
8536.36	7.15415	-7068	7.52E-05	946	0.333333	0.013656	ZBTB16
8587.308	8.154809	-7068	7.50E-05	866	0.333333	0.011993	H6PD
4534.545	4.728641	-7068	7.44E-05	887	0.333333	0.011659	U2AF1
4919.584	7.610626	-7068	7.39E-05	684	0.333333	0.009859	EGR2
5383.1	6.703746	-7068	7.45E-05	813	0.333333	0.012795	MPHOSPH9
7213.409	7.379722	-7068	7.49E-05	858	0.333333	0.012273	ACP5
3059.266	8.016596	-7068	7.29E-05	507	0.333333	0.006685	KLK3
7580.317	5.21902	-7068	7.53E-05	1085	0.333333	0.014166	LMNB1
8337.795	11.66533	-7068	7.36E-05	606	0.333333	0.007265	MOS
4561.845	7.064765	-7068	7.40E-05	705	0.333333	0.010257	C21orf33
6170.16	6.743058	-7068	7.47E-05	850	0.25	0.01279	FRK
5306.616	6.733027	-7068	7.40E-05	748	0.333333	0.008995	ITIH4
6260.025	7.498059	-7068	7.46E-05	806	0.25	0.012313	VGLL4
5899.043	6.680207	-7068	7.46E-05	832	0.333333	0.012309	TNFRSF11A
11766.41	6.774112	-7068	7.64E-05	1161	0.333333	0.017212	NEBL
4757.088	7.113419	-7068	7.39E-05	677	0.333333	0.008979	VARS
6303.057	8.571424	-7068	7.39E-05	691	0.333333	0.009559	LGALS3BP
7902.117	8.4993	-7068	7.46E-05	797	0.333333	0.010545	KRT8
2985.425	8.011613	-7068	7.31E-05	511	0.333333	0.007632	KCNC1
5207.033	6.570274	-7068	7.43E-05	791	0.333333	0.011064	FKBP4
6726.818	7.783325	-7068	7.46E-05	807	0.333333	0.012156	STX8
13432.27	6.445372	-7068	7.69E-05	1285	0.333333	0.018955	TGFB2
5418.622	9.616707	-7068	7.37E-05	596	0.333333	0.009069	ANKRD46
2218.94	7.298674	-7068	7.28E-05	468	0.333333	0.007185	TM2D1
6531.586	8.666346	-7068	7.43E-05	736	0.333333	0.009993	EIF4EBP1
8637.291	6.755364	-7068	7.57E-05	1019	0.333333	0.016099	GSE1
4415.803	7.14025	-7068	7.41E-05	702	0.333333	0.010556	EI24
3769.301	6.216173	-7068	7.39E-05	697	0.333333	0.010308	ACTG2
13776.81	6.268259	-7068	7.77E-05	1362	0.333333	0.02185	QKI
2361.411	5.915153	-7068	7.31E-05	548	0.333333	0.007571	SLA
5262.203	6.576907	-7068	7.43E-05	778	0.333333	0.011619	RGS4
6193.998	6.36627	-7068	7.45E-05	857	0.333333	0.011644	CD28
8877.205	6.698491	-7068	7.54E-05	991	0.333333	0.013231	LAMB3

3588.989	7.724889	-7068	7.33E-05	573	0.333333	0.008054	TUSC2
4029.128	6.020089	-7068	7.41E-05	740	0.333333	0.011037	METAP2
17694.22	6.566149	-7068	7.77E-05	1443	0.333333	0.020357	KLF5
5877.219	6.982878	-7068	7.44E-05	802	0.333333	0.011247	FANCA
9054.322	7.566193	-7068	7.51E-05	918	0.333333	0.012984	EMILIN1
5722.572	9.005042	-7068	7.40E-05	661	0.333333	0.009006	MPST
7562.041	6.980015	-7068	7.52E-05	920	0.333333	0.014074	GGA2
12138.05	6.646767	-7068	7.64E-05	1180	0.333333	0.017042	CLEC2B
7743.093	6.818732	-7068	7.50E-05	904	0.333333	0.012078	LY96
4614.488	4.77313	-7068	7.43E-05	863	0.333333	0.010954	SNRPD3
16008.73	7.159382	-7068	7.74E-05	1311	0.333333	0.020024	ICOS
10753.88	6.862257	-7068	7.60E-05	1100	0.333333	0.016319	ENPP1
10637.67	6.814176	-7068	7.64E-05	1125	0.333333	0.017194	RNASE4
8170.856	6.327608	-7068	7.56E-05	1020	0.333333	0.015005	PTP4A1
3409.779	8.160048	-7068	7.32E-05	540	0.333333	0.007616	TRAPPC3
5736.487	7.004007	-7068	7.46E-05	812	0.333333	0.012743	ARHGAP19
3332.796	7.423355	-7068	7.34E-05	581	0.333333	0.008731	ATP7A
9102.574	6.806007	-7068	7.53E-05	998	0.25	0.01349	AOX1
8092.065	6.482106	-7068	7.51E-05	973	0.333333	0.013106	IGFBP4
4904.569	9.199459	-7068	7.35E-05	579	0.333333	0.00779	PFKFB4
1338.095	7.530675	-7068	7.22E-05	354	0.333333	0.005157	NGDN
7135.776	5.98046	-7068	7.54E-05	1008	0.333333	0.014883	ELAVL1
1760.3	10.66521	-7068	7.20E-05	311	0.333333	0.004048	ZSCAN9
5766.76	6.581242	-7068	7.45E-05	824	0.333333	0.011576	FOSB
11400.56	5.999109	-7068	7.63E-05	1220	0.333333	0.016897	ADH1B
9791.862	7.111	-7068	7.58E-05	1042	0.333333	0.015398	FKBP5
3978.289	7.087963	-7068	7.38E-05	657	0.333333	0.009356	PQBP1
10427.69	6.972157	-7068	7.60E-05	1078	0.333333	0.015597	CXCL13
2012.527	6.232252	-7068	7.30E-05	514	0.333333	0.008264	AMD1
5149.624	7.062073	-7068	7.43E-05	751	0.333333	0.011351	PLOD2
4382.2	8.49137	-7068	7.35E-05	598	0.333333	0.008599	CROT
3296.436	5.986982	-7068	7.32E-05	606	0.333333	0.00787	UQCRB
3685.888	8.410517	-7068	7.32E-05	549	0.333333	0.007284	DNTT
2260.584	7.687785	-7068	7.27E-05	448	0.333333	0.006646	ZNF292
2521.545	7.155314	-7068	7.30E-05	506	0.333333	0.007108	MX1
20572.9	6.818914	-7068	7.85E-05	1529	0.333333	0.022809	RORA
5667.777	7.956669	-7068	7.42E-05	729	0.333333	0.011062	GCNT2
2250.308	7.11316	-7068	7.28E-05	479	0.333333	0.006424	MED12
4117.223	7.397552	-7068	7.36E-05	647	0.333333	0.008727	GCDH
13987.22	7.540842	-7068	7.67E-05	1187	0.333333	0.017448	NDRG1
3811.692	9.497939	-7068	7.29E-05	491	0.333333	0.006105	CREB3L1

5007.617	5.563284	-7068	7.43E-05	837	0.333333	0.011242	CTSS
15101.87	6.962267	-7068	7.71E-05	1281	0.333333	0.019599	PATJ
3271.825	10.06926	-7068	7.28E-05	451	0.333333	0.006285	PBX2
6395.414	8.434154	-7068	7.41E-05	717	0.333333	0.010505	TWIST1
7569.237	7.421957	-7068	7.52E-05	899	0.333333	0.014153	VPS45
7936.197	6.860547	-7068	7.51E-05	942	0.333333	0.013336	SLC25A4
12479.27	7.462232	-7068	7.63E-05	1096	0.333333	0.01524	S100A11
12907.27	6.909394	-7068	7.63E-05	1171	0.333333	0.016097	HSD17B2
2405.762	8.302245	-7068	7.28E-05	451	0.333333	0.006503	UBR4
7917.116	7.5642	-7068	7.52E-05	909	0.333333	0.014156	HMGA2
5597.369	8.173209	-7068	7.42E-05	704	0.333333	0.010075	SOX3
11745.82	6.944216	-7068	7.65E-05	1153	0.333333	0.017514	EYA2
7857.742	6.698342	-7068	7.52E-05	949	0.333333	0.013554	CD86
7982.427	7.226883	-7068	7.53E-05	930	0.333333	0.013883	WWP2
1118.413	11.21535	-7068	7.14E-05	212	0.333333	0.002615	LRRC37A3
4452.671	8.622867	-7068	7.35E-05	592	0.333333	0.007951	VILL
3180.24	9.80056	-7068	7.26E-05	438	0.333333	0.005522	CRYBB2
5185.924	6.386194	-7068	7.43E-05	801	0.333333	0.010727	CSTF2
11379.19	8.615667	-7068	7.54E-05	946	0.333333	0.013357	GUCA1A
13730.38	9.012926	-7068	7.59E-05	1062	0.333333	0.015872	FABP5
6639.687	5.893079	-7068	7.51E-05	963	0.333333	0.013848	IGF1
2021.481	8.823908	-7068	7.24E-05	388	0.333333	0.005004	NPRL2
5103.248	6.839731	-7068	7.41E-05	740	0.25	0.010054	NR1H4
8792.467	6.759494	-7068	7.55E-05	1013	0.333333	0.014511	MAP2K6
6522.972	7.029102	-7068	7.48E-05	849	0.333333	0.013042	COL21A1
4396.274	5.281101	-7068	7.43E-05	824	0.333333	0.01164	HNRNPA0
2553.561	8.901611	-7068	7.27E-05	450	0.333333	0.006423	BTN2A1
5335.95	9.541723	-7068	7.34E-05	581	0.333333	0.007605	NEUROD1
6815.755	5.997232	-7068	7.53E-05	971	0.333333	0.015018	SDHD
2349.652	11.75291	-7068	7.21E-05	318	0.333333	0.003975	MSH4
4487.825	7.706304	-7068	7.37E-05	661	0.333333	0.009434	ASNS
15170.26	6.919252	-7068	7.71E-05	1291	0.333333	0.018961	TRIM29
6595.845	6.920664	-7068	7.49E-05	865	0.333333	0.012744	FDFT1
4649.4	5.643862	-7068	7.43E-05	811	0.333333	0.011406	RAB1A
15341.51	6.57976	-7068	7.75E-05	1366	0.333333	0.020743	MITF
5308.224	7.504716	-7068	7.38E-05	706	0.333333	0.009376	LSM7
4125.291	7.731218	-7068	7.35E-05	603	0.333333	0.008008	PGC
11598.11	7.308689	-7068	7.56E-05	1050	0.333333	0.014094	KRT15
2752.556	8.163951	-7068	7.30E-05	492	0.333333	0.006886	PYGB
5587.648	8.307059	-7068	7.41E-05	702	0.333333	0.00988	TP53I3
6053.978	6.924201	-7068	7.45E-05	819	0.333333	0.011922	IL1R2

7499.368	6.77977	-7068	7.48E-05	892	0.333333	0.011727	IL1B
4782.549	7.999333	-7068	7.31E-05	567	0.333333	0.006989	ZNF273
3918.118	7.853273	-7068	7.35E-05	602	0.333333	0.008904	SEMA3E
4554.27	8.64258	-7068	7.34E-05	597	0.333333	0.007903	MAFK
5206.606	9.132433	-7068	7.37E-05	631	0.333333	0.009009	NRL
3728.016	8.764383	-7068	7.31E-05	533	0.333333	0.007137	CHRNA4
11054.49	6.409639	-7068	7.65E-05	1182	0.333333	0.018341	TLE4
9633.052	6.085954	-7068	7.61E-05	1127	0.333333	0.016841	TJP1
13392.46	6.640032	-7068	7.71E-05	1267	0.333333	0.019645	CDH12
2611.916	8.039795	-7068	7.28E-05	473	0.333333	0.006618	PCDH8
3459	8.002004	-7068	7.33E-05	564	0.333333	0.007977	CAMK2G
5650.48	6.333516	-7068	7.43E-05	821	0.333333	0.010837	POLR2C
5505.731	9.552808	-7068	7.36E-05	605	0.333333	0.007973	NOS3
12733.51	6.25603	-7068	7.71E-05	1303	0.333333	0.019953	NUP98
3485.755	8.058316	-7068	7.33E-05	548	0.333333	0.007478	FXR2
2082.271	7.495714	-7068	7.27E-05	450	0.333333	0.006464	CDS1
7321.844	7.001587	-7068	7.50E-05	899	0.333333	0.013498	STX3
7410.296	9.129998	-7068	7.44E-05	734	0.333333	0.010122	CASP5
4965.703	7.348956	-7068	7.42E-05	727	0.333333	0.011147	SLC30A1
4357.462	10.01472	-7068	7.33E-05	530	0.333333	0.007135	P2RY2
6940.52	6.420985	-7068	7.51E-05	913	0.333333	0.013201	CDKN3
11582.78	7.113157	-7068	7.61E-05	1123	0.333333	0.016428	PRDX6
4819.628	7.451427	-7068	7.40E-05	695	0.333333	0.00999	CLK3
4619.745	8.469464	-7068	7.36E-05	612	0.333333	0.008701	ELAVL2
13122.96	6.277841	-7068	7.70E-05	1298	0.25	0.019066	SPP1
1659.916	8.020893	-7068	7.23E-05	388	0.333333	0.005666	BRD3
7654.482	6.120567	-7068	7.54E-05	1011	0.333333	0.015055	NR2F2
10733.64	8.417317	-7068	7.53E-05	936	0.333333	0.014302	RHOBTB1
14628.23	6.884675	-7068	7.74E-05	1297	0.333333	0.020337	LSAMP
6960.059	8.274638	-7068	7.45E-05	783	0.333333	0.011769	ADRB1
7092.352	7.08378	-7068	7.50E-05	882	0.333333	0.013365	WNT5A
5596.981	8.527279	-7068	7.39E-05	667	0.333333	0.009145	FSTL3
3376.131	10.19854	-7068	7.28E-05	459	0.333333	0.006714	CLGN
13010.63	7.137222	-7068	7.66E-05	1186	0.333333	0.017666	TYRO3
7328.789	6.321156	-7068	7.54E-05	976	0.333333	0.015208	AKAP12
9223.878	12.68313	-7068	7.37E-05	605	0.333333	0.007888	ATP6V1G2
2683.125	6.58249	-7068	7.34E-05	577	0.333333	0.009385	RSRC2
13504.41	6.64778	-7068	7.71E-05	1284	0.333333	0.019684	GAS7
4322.823	8.684603	-7068	7.34E-05	565	0.333333	0.007182	MTX1
2890.555	8.233376	-7068	7.31E-05	495	0.333333	0.006635	MBD3
9093.285	5.917025	-7068	7.61E-05	1137	0.333333	0.01727	RAB2A

6751.145	6.918691	-7068	7.48E-05	861	0.333333	0.012533	LPL
5798.231	9.061006	-7068	7.38E-05	647	0.333333	0.008637	CRAT
7323.495	6.710672	-7068	7.50E-05	926	0.333333	0.013713	PDIA4
3010.564	9.151027	-7068	7.28E-05	455	0.333333	0.006195	MYL1
8434.515	5.696034	-7068	7.58E-05	1102	0.333333	0.016174	LTBP1
3612.722	5.477545	-7068	7.35E-05	687	0.333333	0.00824	C1QB
4559.484	7.362493	-7068	7.39E-05	699	0.333333	0.010308	PRKX
6135.265	7.310796	-7068	7.45E-05	792	0.333333	0.01118	SORD
4681.541	6.177486	-7068	7.41E-05	762	0.333333	0.010645	SMTN
2558.548	8.876547	-7068	7.26E-05	424	0.333333	0.005413	PSG11
3854.737	7.717957	-7068	7.36E-05	604	0.333333	0.009166	ARHGAP12
12656.63	6.097525	-7068	7.71E-05	1304	0.333333	0.019829	EPHA4
7265.272	6.52977	-7068	7.49E-05	908	0.333333	0.012163	IFITM2
3804.658	6.491298	-7068	7.34E-05	641	0.25	0.007897	F13B
7161.364	5.813389	-7068	7.54E-05	1014	0.333333	0.014432	XRCC6
1929.202	9.434311	-7068	7.23E-05	363	0.333333	0.00478	FAM155B
7339.162	8.071099	-7068	7.49E-05	820	0.333333	0.012206	SBF1
9556.612	7.240543	-7068	7.56E-05	1007	0.333333	0.015135	NECTIN3
3880.93	7.439056	-7068	7.38E-05	636	0.333333	0.009621	TRAK1
6273.736	6.435768	-7068	7.45E-05	837	0.333333	0.01091	ADH1A
13207.18	7.35186	-7068	7.65E-05	1156	0.333333	0.017048	STEAP1
2685.712	7.646738	-7068	7.31E-05	512	0.25	0.007665	PPM1E
6155.516	7.496354	-7068	7.44E-05	779	0.333333	0.011302	PRLR
6817.605	7.963238	-7068	7.45E-05	784	0.333333	0.011783	PDE10A
4353.826	6.492299	-7068	7.39E-05	727	0.333333	0.009975	CCNE1
12675.54	6.853649	-7068	7.67E-05	1210	0.333333	0.018072	PIK3CD
1814.38	9.409368	-7068	7.23E-05	356	0.25	0.004832	SLC9A8
13630.86	6.808619	-7068	7.70E-05	1254	0.333333	0.019325	CXADR
5142.26	6.252354	-7068	7.44E-05	810	0.333333	0.011048	FDPS
4296.527	6.803609	-7068	7.40E-05	717	0.333333	0.010381	BID
12757.09	6.878221	-7068	7.67E-05	1205	0.333333	0.018151	THRB
7171.237	5.106631	-7068	7.53E-05	1082	0.333333	0.014434	FOS
17561.7	6.854298	-7068	7.80E-05	1427	0.333333	0.0217	SEMA3A
5103.515	9.405521	-7068	7.37E-05	615	0.333333	0.008906	DOT1L
5500.264	8.602605	-7068	7.39E-05	654	0.333333	0.009016	ECM1
6271.753	6.763119	-7068	7.50E-05	874	0.333333	0.013339	ATP1B3
7911.39	6.351795	-7068	7.55E-05	1017	0.333333	0.015262	ALDH7A1
2551.864	8.169241	-7068	7.28E-05	464	0.333333	0.005969	EPO
3476.938	7.179098	-7068	7.33E-05	586	0.333333	0.007995	FOLR2
10432.58	7.616706	-7068	7.58E-05	1013	0.333333	0.015368	KCNK2
4829.652	7.176976	-7068	7.40E-05	724	0.333333	0.010583	TAF1

3969.383	8.123313	-7068	7.34E-05	588	0.333333	0.008001	BIK
7382.597	6.252939	-7068	7.52E-05	981	0.333333	0.014565	DMD
4227.732	8.152831	-7068	7.36E-05	610	0.333333	0.008547	GALNS
3505.996	7.503794	-7068	7.34E-05	593	0.333333	0.008488	RNF8
5140.319	7.202168	-7068	7.43E-05	755	0.25	0.012072	CDC14A
3939.583	7.508971	-7068	7.29E-05	547	0.333333	0.006276	NDUFA1
6040.559	11.80507	-7068	7.31E-05	500	0.333333	0.006061	PROP1
3436.445	6.720201	-7068	7.33E-05	607	0.333333	0.007882	IRF9
6813.504	6.855812	-7068	7.46E-05	852	0.333333	0.011339	FDX1
4799.876	7.636303	-7068	7.39E-05	688	0.333333	0.009856	TNFSF11
6482.258	7.540109	-7068	7.46E-05	816	0.333333	0.012009	TXNRD2
3252.632	8.511048	-7068	7.30E-05	480	0.333333	0.005676	PRODH2
4383.439	6.91761	-7068	7.40E-05	697	0.333333	0.009845	ARF3
4576.347	5.857193	-7068	7.44E-05	807	0.333333	0.012387	TCEA1
6847.045	7.600914	-7068	7.46E-05	811	0.333333	0.010745	ASL
4668.573	6.69646	-7068	7.42E-05	733	0.333333	0.011088	TNFAIP6
7670.879	8.078046	-7068	7.48E-05	838	0.333333	0.012502	SLC2A5
9561.792	9.582631	-7068	7.48E-05	824	0.333333	0.012188	CRABP1
8179.321	4.126456	-7068	7.57E-05	1263	0.333333	0.015222	CCNA2
4162.841	6.756286	-7068	7.41E-05	695	0.333333	0.011057	ARL6IP1
9299.832	7.72378	-7068	7.54E-05	953	0.333333	0.014637	PAX3
1578.579	8.55834	-7068	7.22E-05	345	0.333333	0.004659	SFTPC
1314.24	9.203894	-7068	7.20E-05	304	0.333333	0.004318	DPYSL4
5979.457	9.364294	-7068	7.37E-05	627	0.333333	0.008121	TSSK2
10908.75	7.204351	-7068	7.59E-05	1053	0.333333	0.015679	INPP4B
6377.678	6.112351	-7068	7.52E-05	943	0.333333	0.015018	GCLM
5180.656	6.668336	-7068	7.42E-05	765	0.333333	0.010932	DSC2
5832.324	6.769774	-7068	7.47E-05	825	0.333333	0.012688	RAB14
5414.067	7.390492	-7068	7.42E-05	737	0.333333	0.010738	GAS6
19688.61	6.64766	-7068	7.84E-05	1542	0.333333	0.023159	VEGFA
2040.106	8.996693	-7068	7.23E-05	379	0.333333	0.004743	VPS11
7488.525	7.493788	-7068	7.49E-05	866	0.333333	0.013078	NFAT5
7688.107	6.241709	-7068	7.53E-05	989	0.333333	0.014395	UBE2D1
3509.951	9.733533	-7068	7.29E-05	464	0.333333	0.005721	ABCA2
7500.478	10.47477	-7068	7.39E-05	664	0.333333	0.009381	FABP6
14700.5	7.061851	-7068	7.70E-05	1266	0.333333	0.018656	MBP
9813.962	8.366085	-7068	7.52E-05	915	0.333333	0.013272	SREBF2
2875.537	7.562786	-7068	7.31E-05	528	0.333333	0.007509	AP3D1
4607.045	8.284952	-7068	7.38E-05	637	0.333333	0.009098	BNIP1
2523.032	7.839047	-7068	7.30E-05	493	0.333333	0.007465	GPR107
5020.184	7.175739	-7068	7.40E-05	724	0.333333	0.010449	EPOR

4846.131	8.322404	-7068	7.38E-05	633	0.333333	0.008861	BCAM
1680.257	6.941777	-7068	7.26E-05	432	0.333333	0.006621	CREBZF
6975.121	7.626266	-7068	7.46E-05	806	0.333333	0.011257	SLC7A7
4247.841	6.79887	-7068	7.40E-05	701	0.333333	0.010071	ACADL
9147.668	7.334767	-7068	7.55E-05	985	0.333333	0.01536	GIT2
6874.78	7.358446	-7068	7.49E-05	852	0.333333	0.012944	AKIRIN2
5121.104	12.17115	-7068	7.28E-05	453	0.333333	0.005313	WNT10B
13359.79	6.379533	-7068	7.71E-05	1318	0.333333	0.019853	MYO1B
4100.023	6.726495	-7068	7.40E-05	689	0.333333	0.010188	MMP1
6580.167	14.05421	-7068	7.32E-05	518	0.333333	0.006659	ZNF282
5600.772	6.266781	-7068	7.44E-05	825	0.333333	0.011341	TPM2
4747.778	8.416102	-7068	7.38E-05	642	0.333333	0.009749	CX3CR1
3070.276	7.549281	-7068	7.33E-05	565	0.333333	0.008873	OSBP
13864.01	6.975154	-7068	7.68E-05	1236	0.333333	0.018288	MYOM2
7214.96	8.331475	-7068	7.44E-05	787	0.333333	0.011328	PDCD6
7392.208	8.002815	-7068	7.46E-05	804	0.333333	0.01167	SERPINB2
1380.325	9.720974	-7068	7.20E-05	292	0.333333	0.003566	GIPR
2379.178	8.890978	-7068	7.27E-05	424	0.333333	0.005814	ARC
4549.297	6.900289	-7068	7.42E-05	707	0.333333	0.010181	IFITM1
13921.65	6.463839	-7068	7.69E-05	1296	0.333333	0.018956	KLF9
4710.997	8.551536	-7068	7.29E-05	546	0.333333	0.006559	ZNF91
17493.69	6.618454	-7068	7.80E-05	1447	0.333333	0.022017	SNX7
6672.088	7.2988	-7068	7.46E-05	809	0.333333	0.011711	HBB
5224.353	7.355018	-7068	7.42E-05	725	0.333333	0.010718	JAKMIP2
3258.554	8.902887	-7068	7.31E-05	495	0.333333	0.006716	TRPV6
3059.678	5.782026	-7068	7.34E-05	629	0.333333	0.008175	POLR2J
7699.1	7.980078	-7068	7.48E-05	842	0.333333	0.012516	TACR1
6263.157	6.852489	-7068	7.46E-05	829	0.333333	0.011975	IL1RL1
5452.753	8.256373	-7068	7.40E-05	681	0.333333	0.009701	IL2
13252.86	6.091397	-7068	7.72E-05	1344	0.333333	0.019697	COL18A1
7393.01	7.215896	-7068	7.48E-05	869	0.333333	0.011907	IL1RN
3944.973	6.362769	-7068	7.38E-05	698	0.333333	0.009865	RAB8A
5641.352	6.90205	-7068	7.46E-05	809	0.333333	0.012258	ZC3HAV1
4997.956	8.505788	-7068	7.37E-05	626	0.333333	0.008832	SULT1C2
10421.51	6.451269	-7068	7.62E-05	1133	0.333333	0.01686	TFPI2
5474.804	8.069267	-7068	7.41E-05	711	0.333333	0.010219	ALDH1B1
2608.881	7.034042	-7068	7.30E-05	520	0.333333	0.007409	SNX1
8169.506	8.573257	-7068	7.47E-05	825	0.333333	0.011953	CDK5R1
6532.936	7.698284	-7068	7.46E-05	801	0.333333	0.01207	DEPDC5
7258.859	7.17138	-7068	7.50E-05	882	0.333333	0.013602	RAB28
7859.925	6.519642	-7068	7.55E-05	990	0.333333	0.014977	MCCC2

6343.015	9.47417	-7068	7.39E-05	669	0.333333	0.009772	LDHC
6233.91	8.700635	-7068	7.39E-05	694	0.333333	0.009481	IL10
11884.19	7.19352	-7068	7.62E-05	1128	0.333333	0.016878	NR5A2
6486.515	7.199185	-7068	7.46E-05	820	0.333333	0.010964	AKR1B1
5753.114	9.148036	-7068	7.38E-05	647	0.333333	0.009089	FGF8
5400.625	7.081514	-7068	7.41E-05	733	0.333333	0.009714	KRT6A
3021.199	6.908034	-7068	7.34E-05	590	0.333333	0.008914	NMT1
15914.38	6.458056	-7068	7.77E-05	1409	0.333333	0.021583	EDNRB
6567.174	6.561545	-7068	7.50E-05	906	0.333333	0.013848	TES
11053.33	6.691928	-7068	7.65E-05	1158	0.333333	0.017886	ST3GAL1
3935.718	7.899502	-7068	7.36E-05	601	0.333333	0.008996	LHX2
6132.792	7.658059	-7068	7.45E-05	780	0.333333	0.011608	UGDH
8762.538	5.615237	-7068	7.56E-05	1104	0.333333	0.014899	CENPE
14698.92	7.259003	-7068	7.68E-05	1233	0.333333	0.017937	MRC2
3293.195	6.81589	-7068	7.34E-05	615	0.333333	0.009051	TBL1X
1327.551	6.658476	-7068	7.24E-05	392	0.333333	0.00598	KDM6A
3739.339	7.751564	-7068	7.36E-05	606	0.333333	0.008907	NRF1
4874.316	5.968123	-7068	7.42E-05	788	0.333333	0.010516	MDH2
20914.19	7.453234	-7068	7.82E-05	1455	0.333333	0.021946	NTRK3
10529.87	6.840044	-7068	7.60E-05	1095	0.333333	0.015933	FGF1
2167.451	7.91975	-7068	7.26E-05	439	0.333333	0.005982	MAST2
3600.051	7.759434	-7068	7.33E-05	573	0.333333	0.007314	LCAT
6825.074	6.791555	-7068	7.47E-05	865	0.333333	0.011856	EXO1
9947.873	7.016103	-7068	7.56E-05	1026	0.333333	0.014441	IDH1
7266.395	5.636336	-7068	7.50E-05	1011	0.333333	0.013232	JAK2
20507.02	6.598095	-7068	7.89E-05	1588	0.333333	0.024575	TNIK
4895.208	6.815061	-7068	7.44E-05	767	0.333333	0.012244	CDV3
6153.175	8.528915	-7068	7.39E-05	674	0.333333	0.008627	CLDN4
4405.741	7.494268	-7068	7.39E-05	670	0.333333	0.010161	ERLIN2
8217.078	6.130584	-7068	7.52E-05	1028	0.333333	0.013272	E2F1
7785.045	7.163207	-7068	7.47E-05	884	0.333333	0.011612	ITGB4
10357.02	7.333831	-7068	7.58E-05	1042	0.333333	0.015065	GFPT2
7538.724	7.168264	-7068	7.49E-05	890	0.333333	0.012282	NQO2
7253.834	6.512039	-7068	7.54E-05	953	0.333333	0.015251	KIDINS220
3388.97	9.226194	-7068	7.30E-05	494	0.333333	0.007073	AIPL1
3951.477	7.504121	-7068	7.37E-05	626	0.333333	0.009112	DDAH2
3487.878	7.348894	-7068	7.35E-05	596	0.333333	0.008178	CYBA
5621.926	7.371756	-7068	7.44E-05	767	0.333333	0.011465	NFIX
8985.272	7.057164	-7068	7.56E-05	996	0.333333	0.015323	PIAS2
640.7708	8.634086	-7068	7.15E-05	228	0.25	0.003178	BBS9
2179.156	7.220849	-7068	7.30E-05	482	0.333333	0.007452	NEMF

6074.498	6.388894	-7068	7.51E-05	886	0.333333	0.014138	NEMP1
15374.5	6.721223	-7068	7.68E-05	1289	0.333333	0.017703	C1S
7254.16	8.236033	-7068	7.46E-05	784	0.333333	0.011015	CLEC11A
3863.407	6.578771	-7068	7.35E-05	635	0.333333	0.008103	CYP2C19
5888.243	6.223196	-7068	7.49E-05	887	0.333333	0.013685	BNIP3L
9782.98	7.254201	-7068	7.56E-05	1014	0.333333	0.014617	RHOB
4267.9	8.177825	-7068	7.36E-05	618	0.333333	0.009214	DYRK3
4094.988	6.44046	-7068	7.40E-05	710	0.333333	0.01074	PDCD2
12347.36	6.505208	-7068	7.64E-05	1213	0.333333	0.017271	IFNG
3001.337	9.647508	-7068	7.26E-05	425	0.333333	0.005179	PLA2G1B
5665.081	5.934135	-7068	7.46E-05	856	0.333333	0.011764	IL6
9556.033	6.742424	-7068	7.58E-05	1058	0.333333	0.015292	EPHB2
4065.966	8.244653	-7068	7.36E-05	602	0.333333	0.008641	ARL3
10197.27	6.846008	-7068	7.62E-05	1101	0.333333	0.017017	CREB3L2
14280.96	6.962402	-7068	7.68E-05	1246	0.333333	0.017857	LOX
5126.771	7.780396	-7068	7.42E-05	712	0.333333	0.010791	THOC5
3924.89	7.311813	-7068	7.35E-05	634	0.333333	0.008851	MR1
2911.229	9.556894	-7068	7.26E-05	420	0.333333	0.005175	SEMA7A
6466.934	9.212862	-7068	7.40E-05	674	0.333333	0.009631	ARAP1
7587.341	6.062508	-7068	7.54E-05	1025	0.333333	0.015073	EZR
3700.963	7.375974	-7068	7.35E-05	618	0.333333	0.009029	IPO8
3466.754	8.703927	-7068	7.30E-05	509	0.333333	0.006569	PDK2
4198.096	6.219965	-7068	7.43E-05	741	0.333333	0.011551	NASP
4302.984	7.515855	-7068	7.31E-05	585	0.333333	0.006772	NDUFB7
6053.618	6.752698	-7068	7.48E-05	846	0.333333	0.013003	SLC1A2
3638.776	10.14577	-7068	7.29E-05	470	0.333333	0.0061	TMSB4Y
4109.576	7.897834	-7068	7.37E-05	630	0.333333	0.009226	MGST3
5576.133	11.26079	-7068	7.32E-05	538	0.333333	0.00721	MSI1
6721.079	7.269315	-7068	7.46E-05	800	0.333333	0.011151	DTYMK
5063.123	7.915721	-7068	7.37E-05	667	0.333333	0.009652	CLOCK
2721.21	7.355055	-7068	7.32E-05	545	0.333333	0.008599	MTMR1
5822.493	6.705646	-7068	7.47E-05	832	0.333333	0.012461	ALAD
8976.263	7.287389	-7068	7.55E-05	979	0.333333	0.014688	LRP8
2382.828	8.073703	-7068	7.26E-05	428	0.333333	0.005196	SURF1
13703.12	6.58958	-7068	7.72E-05	1297	0.333333	0.019703	TPD52L1
8380.813	6.686415	-7068	7.57E-05	1005	0.333333	0.015452	ADCY9
5759.028	6.505319	-7068	7.47E-05	848	0.333333	0.012614	LDHA
5914.708	8.482831	-7068	7.41E-05	696	0.333333	0.009608	CD5L
1086.226	11.39786	-7068	7.16E-05	225	0.333333	0.002749	FSCN2
3082.291	6.354626	-7068	7.33E-05	595	0.333333	0.007618	CYP3A4
4575.965	6.382525	-7068	7.39E-05	729	0.333333	0.009442	CYP1A2

3109.775	6.358793	-7068	7.35E-05	633	0.333333	0.009182	EIF3F
3111.331	5.782804	-7068	7.37E-05	663	0.333333	0.00957	KHSRP
3370.49	6.529206	-7068	7.37E-05	646	0.333333	0.00989	PPP1R2
8164.914	7.773243	-7068	7.50E-05	884	0.333333	0.012515	GLDC
2950.159	6.089056	-7068	7.32E-05	588	0.333333	0.007786	MRPS18B
9127.007	5.905616	-7068	7.61E-05	1141	0.333333	0.016322	ENO1
7452.236	6.462137	-7068	7.52E-05	960	0.333333	0.014646	PLS3
3544.185	7.449739	-7068	7.37E-05	613	0.333333	0.009485	IER2
9520.796	7.635535	-7068	7.56E-05	972	0.333333	0.014849	WNT7A
7176.397	7.72686	-7068	7.45E-05	805	0.333333	0.010665	NAT2
3708.115	7.995091	-7068	7.33E-05	561	0.333333	0.007688	GJA4
5883.188	5.868386	-7068	7.50E-05	903	0.333333	0.01331	CKS1B
3341.017	9.369673	-7068	7.28E-05	484	0.333333	0.006626	HSPA1L
4135.935	8.464059	-7068	7.35E-05	602	0.333333	0.008581	GTF3A
6799.537	8.352674	-7068	7.43E-05	749	0.333333	0.010848	ASCL1
4729.758	6.862385	-7068	7.43E-05	739	0.333333	0.011624	NMT2
11513.32	6.605665	-7068	7.61E-05	1132	0.333333	0.015858	COL4A2
7689.427	6.997048	-7068	7.52E-05	928	0.333333	0.013723	PTPN21
6356.86	6.971846	-7068	7.47E-05	836	0.333333	0.012289	ADAM19
3656.341	8.015113	-7068	7.34E-05	580	0.333333	0.008295	IL17RA
5786.537	6.85486	-7068	7.44E-05	792	0.333333	0.010628	CYP4F2
6900.956	7.75182	-7068	7.46E-05	814	0.333333	0.011664	NECTIN2
7577.668	6.70414	-7068	7.54E-05	966	0.333333	0.015029	ETV5
4943.972	7.022527	-7068	7.40E-05	717	0.333333	0.01023	TLR1
6638.603	6.859354	-7068	7.50E-05	876	0.333333	0.013473	SLC25A24
7318.253	7.16894	-7068	7.42E-05	828	0.333333	0.01011	AMBP
19980.35	6.602454	-7068	7.87E-05	1565	0.333333	0.023999	RBMS1
4320.99	5.510558	-7068	7.39E-05	750	0.333333	0.009152	POLR2F
7877.247	7.348576	-7068	7.51E-05	892	0.333333	0.012614	NECTIN1
2618.043	9.458221	-7068	7.25E-05	394	0.333333	0.004523	GNAT1
22208.63	6.709225	-7068	7.90E-05	1624	0.333333	0.024465	NRG1
18042.51	5.78093	-7068	7.82E-05	1578	0.333333	0.022439	COL4A1
8139.874	6.730175	-7068	7.54E-05	993	0.333333	0.014771	PFKP
5839.99	6.80229	-7068	7.44E-05	816	0.333333	0.010861	PRKACA
7109.928	9.235995	-7068	7.40E-05	696	0.333333	0.009444	CCR4
3615.662	7.525969	-7068	7.36E-05	608	0.333333	0.008842	SIGMAR1
3871.239	6.309454	-7068	7.38E-05	698	0.333333	0.010503	DDR2
4625.766	7.099446	-7068	7.41E-05	712	0.333333	0.010461	MARK2
4686.927	7.953189	-7068	7.37E-05	637	0.333333	0.008561	GPD1
3512.793	7.782779	-7068	7.35E-05	583	0.333333	0.008691	ULK2
4292.167	6.791015	-7068	7.41E-05	705	0.333333	0.010621	SP100

7966.484	7.571449	-7068	7.51E-05	889	0.333333	0.013553	SLC7A2
3046.87	7.594272	-7068	7.32E-05	551	0.333333	0.00759	IRF5
3142.643	8.955317	-7068	7.30E-05	492	0.333333	0.006618	P2RX1
5475.586	9.334278	-7068	7.38E-05	629	0.333333	0.008848	GRINA
4322.472	9.349447	-7068	7.33E-05	549	0.333333	0.007703	DRD3
4713.089	7.187441	-7068	7.40E-05	705	0.333333	0.010761	EDRF1
7200.765	5.929326	-7068	7.50E-05	962	0.333333	0.013026	POLR2D
7642.606	6.983144	-7068	7.50E-05	905	0.333333	0.013078	FAM107A
10018.29	6.889361	-7068	7.61E-05	1083	0.333333	0.016677	HEBP2
3905.779	8.163828	-7068	7.36E-05	595	0.333333	0.008669	RANBP3
14666.9	7.089933	-7068	7.70E-05	1269	0.333333	0.018521	FGA
11646.03	6.408087	-7068	7.69E-05	1228	0.333333	0.01966	HACD2
1951.453	5.170885	-7068	7.28E-05	541	0.333333	0.007457	HNRNPDL
7477.484	7.785453	-7068	7.50E-05	865	0.333333	0.013395	TOMM34
13864.81	6.491325	-7068	7.74E-05	1332	0.333333	0.020418	ERG
1322.616	6.712291	-7068	7.20E-05	361	0.333333	0.004157	MASP2
904.596	10.14826	-7068	7.17E-05	238	0.333333	0.003311	RBMXL2
3571.189	9.487981	-7068	7.30E-05	484	0.333333	0.006301	MUC3A
7207.436	7.800713	-7068	7.46E-05	816	0.333333	0.011787	DIO2
17326.65	7.226279	-7068	7.77E-05	1357	0.333333	0.02057	IL12A
8134.491	6.959059	-7068	7.55E-05	963	0.333333	0.014801	RAB30
8181.227	7.686116	-7068	7.52E-05	910	0.333333	0.013771	HLCS
11212.86	6.976637	-7068	7.62E-05	1116	0.333333	0.016719	PKP2
6161.219	6.70305	-7068	7.46E-05	839	0.333333	0.01202	GNG11
1903.22	7.224208	-7068	7.27E-05	458	0.333333	0.006889	NBR1
4350.714	6.231159	-7068	7.42E-05	741	0.333333	0.011281	CYBB
2481.011	8.283921	-7068	7.29E-05	471	0.333333	0.006959	TSPAN31
3731.261	7.635813	-7068	7.34E-05	583	0.333333	0.007969	ADGRE2
3227.576	6.779413	-7068	7.36E-05	615	0.333333	0.008885	ARPC4
11449.15	7.992173	-7068	7.59E-05	1027	0.333333	0.015639	USH2A
3678.367	5.437003	-7068	7.38E-05	740	0.333333	0.009819	JUNB
2538.35	7.384243	-7068	7.31E-05	521	0.333333	0.007812	EMC1
3864.413	9.489253	-7068	7.32E-05	514	0.333333	0.007595	FTO
4792.734	5.900578	-7068	7.38E-05	757	0.333333	0.008805	C2
7252.186	5.424015	-7068	7.53E-05	1029	0.333333	0.014033	TPX2
17373.98	6.789161	-7068	7.64E-05	1227	0.333333	0.016803	KIF2C
3212.658	6.829607	-7068	7.37E-05	620	0.333333	0.010268	TMX4
3587.648	8.089673	-7068	7.33E-05	564	0.333333	0.007946	COL9A3
7341.38	7.70048	-7068	7.46E-05	812	0.333333	0.011178	PI3
4099.95	6.770427	-7068	7.37E-05	670	0.333333	0.008607	NFKB2
11052.8	6.952788	-7068	7.63E-05	1123	0.333333	0.017339	KCNK1

2639.684	7.210251	-7068	7.30E-05	523	0.333333	0.00732	SAFB
5918.823	7.610263	-7068	7.43E-05	758	0.333333	0.010715	TROAP
9665.848	6.979648	-7068	7.57E-05	1040	0.333333	0.014878	MTHFD1
16886.11	7.458345	-7068	7.75E-05	1317	0.333333	0.020708	LARGE1
12230.18	6.107694	-7068	7.71E-05	1289	0.333333	0.020155	HIPK1
4507.973	8.789843	-7068	7.26E-05	501	0.25	0.005569	ZNF33B
6018.94	5.995509	-7068	7.45E-05	868	0.333333	0.01154	MMP14
7032.236	7.716372	-7068	7.46E-05	810	0.333333	0.011834	SLC22A4
1083.584	7.703701	-7068	7.20E-05	324	0.333333	0.004546	EIF1AY
3898.422	6.258768	-7068	7.37E-05	683	0.333333	0.008809	CP
7200.195	7.033255	-7068	7.49E-05	884	0.333333	0.013094	ABI2
5308.955	7.009621	-7068	7.41E-05	736	0.333333	0.009656	LPA
7315.818	7.429575	-7068	7.50E-05	864	0.333333	0.013208	GPD2
13029.07	6.726202	-7068	7.66E-05	1216	0.25	0.017564	ENPP2
4439.226	6.976394	-7068	7.39E-05	688	0.333333	0.009957	LSM5
8647.921	5.784325	-7068	7.55E-05	1098	0.333333	0.014617	SMAD3
4150.115	7.053479	-7068	7.39E-05	674	0.333333	0.01018	YTHDC1
2509.376	7.203489	-7068	7.30E-05	508	0.333333	0.007953	SNX4
3972.312	6.851397	-7068	7.40E-05	685	0.333333	0.010937	KDM3A
13239.52	6.337782	-7068	7.72E-05	1313	0.333333	0.020286	SPTBN1
2960.097	8.444536	-7068	7.30E-05	498	0.333333	0.007283	SARM1
17906.25	7.406997	-7068	7.77E-05	1355	0.333333	0.020992	SRGAP3
3108.306	9.786202	-7068	7.27E-05	437	0.333333	0.005948	SULT4A1
5781.963	6.032543	-7068	7.42E-05	824	0.333333	0.010343	HLA-F
5644.579	7.364453	-7068	7.45E-05	778	0.333333	0.011785	CYB561
8626.183	6.866557	-7068	7.54E-05	995	0.333333	0.014676	PIK3R3
2725.634	8.138545	-7068	7.29E-05	488	0.333333	0.006677	CORO2A
4901.805	12.1258	-7068	7.28E-05	449	0.333333	0.005367	CNTF
17384.01	7.316941	-7068	7.74E-05	1336	0.333333	0.01908	DHRS3
14192.44	5.873813	-7068	7.63E-05	1357	0.333333	0.01618	SRC
4405.906	9.139777	-7068	7.34E-05	559	0.333333	0.007808	TGM5
4574.393	9.693535	-7068	7.32E-05	538	0.333333	0.007402	ARNT2
4262.05	7.700433	-7068	7.39E-05	654	0.333333	0.0099	NFATC2IP
9956.863	7.0236	-7068	7.55E-05	1039	0.333333	0.014107	PML
3678.074	8.930725	-7068	7.30E-05	517	0.333333	0.006669	NFKBIB
9976.276	7.033314	-7068	7.60E-05	1063	0.333333	0.016108	POLD3
2743.419	7.031785	-7068	7.32E-05	552	0.333333	0.008309	ZFYVE9
3325.4	8.846681	-7068	7.30E-05	504	0.333333	0.006503	DNPH1
6140.833	7.278477	-7068	7.46E-05	799	0.333333	0.011557	MMP19
5148.688	4.617238	-7068	7.47E-05	960	0.333333	0.012868	COL3A1
2447.262	8.209303	-7068	7.28E-05	467	0.333333	0.00685	WIPI1

5775.738	7.625041	-7068	7.43E-05	743	0.333333	0.010957	FKBP9
6453.793	6.863471	-7068	7.48E-05	853	0.333333	0.012479	IER3
4007.446	8.333733	-7068	7.35E-05	577	0.333333	0.008187	REG1A
5033.028	7.35178	-7068	7.42E-05	731	0.333333	0.011101	ZNF207
7348.143	7.608636	-7068	7.48E-05	838	0.333333	0.012164	ELF3
5158.968	6.582746	-7068	7.45E-05	805	0.333333	0.012063	YBX3
10633.74	8.320031	-7068	7.55E-05	968	0.333333	0.014448	RAB32
11700.25	6.673167	-7068	7.68E-05	1201	0.333333	0.018556	CTSH
5102.945	7.516952	-7068	7.37E-05	672	0.333333	0.008515	SLPI
13804.1	6.823181	-7068	7.70E-05	1265	0.333333	0.018596	SCARB1
4833.603	8.21495	-7068	7.31E-05	574	0.333333	0.006385	NDUFA2
15255.64	6.907127	-7068	7.71E-05	1312	0.333333	0.019153	HADH
3351.356	5.295166	-7068	7.37E-05	705	0.333333	0.009269	PSMD13
6936.56	6.77587	-7068	7.52E-05	911	0.333333	0.014255	CSRP2
6945.096	6.59933	-7068	7.52E-05	934	0.333333	0.014396	MAP4
8406.808	5.374204	-7068	7.58E-05	1133	0.333333	0.01619	RPL13
13458.29	6.876321	-7068	7.72E-05	1259	0.333333	0.019811	TP53I11
10929.03	7.502318	-7068	7.58E-05	1041	0.333333	0.015112	WNT4
6447.974	6.59874	-7068	7.51E-05	895	0.333333	0.014069	PDK3
5225.138	5.252686	-7068	7.45E-05	886	0.333333	0.011832	ACTA2
6796.844	6.294931	-7068	7.48E-05	909	0.333333	0.012766	NOV
7669.951	5.718821	-7068	7.54E-05	1049	0.333333	0.014679	LAMA3
4921.431	7.081823	-7068	7.40E-05	711	0.333333	0.009826	ACKR1
4480.785	6.527696	-7068	7.41E-05	738	0.333333	0.0103	IVD
6207.194	9.256168	-7068	7.36E-05	645	0.333333	0.00833	NKX3-1
11957.71	7.186761	-7068	7.61E-05	1125	0.333333	0.016057	KLF4
10194.18	8.341886	-7068	7.53E-05	925	0.333333	0.013443	SCEL
2295.597	9.15037	-7068	7.25E-05	402	0.333333	0.005607	UPK1B
9533.775	9.396839	-7068	7.45E-05	788	0.333333	0.010176	PPL
2714.889	10.49666	-7068	7.26E-05	404	0.333333	0.00524	TOP3B
5128.427	5.889848	-7068	7.44E-05	834	0.333333	0.011386	LRP1
18025.04	6.686494	-7068	7.84E-05	1480	0.333333	0.023319	SYNE1
2876.517	6.366521	-7068	7.35E-05	617	0.333333	0.009577	ADAM17
1748.934	6.823379	-7068	7.26E-05	440	0.333333	0.006631	ATG12
5388.532	6.824189	-7068	7.43E-05	780	0.333333	0.011114	RARB
7239.475	6.813042	-7068	7.46E-05	871	0.333333	0.011277	UQCR11
4520.029	8.745477	-7068	7.34E-05	585	0.333333	0.008646	CLCN5
7811.614	7.591834	-7068	7.52E-05	887	0.333333	0.013811	ATP9B
6293.493	8.252176	-7068	7.38E-05	704	0.333333	0.009134	MMP8
4706.993	7.971556	-7068	7.36E-05	624	0.333333	0.008048	MRPS12
3840.735	8.078849	-7068	7.36E-05	595	0.333333	0.008907	C1orf216

6544.261	6.716692	-7068	7.49E-05	878	0.333333	0.012797	GAS2
4879.844	6.335524	-7068	7.45E-05	799	0.333333	0.012387	ANGPT1
5326.547	6.975446	-7068	7.44E-05	778	0.333333	0.011723	BST2
8787.162	6.727218	-7068	7.58E-05	1035	0.333333	0.016165	MTSS1
4391.515	6.679923	-7068	7.43E-05	735	0.333333	0.011732	DAZAP2
6124.124	7.137646	-7068	7.47E-05	819	0.333333	0.012514	GPM6B
7771.691	6.945671	-7068	7.55E-05	955	0.333333	0.01539	MDM1
3722.487	9.649459	-7068	7.28E-05	481	0.333333	0.006124	CCL22
4605.635	6.282124	-7068	7.42E-05	763	0.333333	0.011121	IL13RA1
4545.623	7.756024	-7068	7.36E-05	632	0.333333	0.008593	PPBP
5799.392	6.42058	-7068	7.48E-05	855	0.333333	0.01319	TIMM17A
7606.954	5.354335	-7068	7.56E-05	1093	0.333333	0.015262	FH
12911.82	7.819899	-7068	7.62E-05	1093	0.333333	0.016617	TRPM1
3135.905	7.653397	-7068	7.33E-05	553	0.333333	0.007558	AP2A2
5883.879	5.899825	-7068	7.48E-05	901	0.333333	0.012778	ILF3
8877.909	7.308833	-7068	7.54E-05	963	0.333333	0.01451	ITGA9
2887.814	7.548438	-7068	7.32E-05	540	0.333333	0.008093	DBN1
15220.84	6.860322	-7068	7.74E-05	1319	0.333333	0.019929	SLC4A4
2234.429	9.831919	-7068	7.25E-05	391	0.333333	0.005353	CHRNB1
7663.103	6.795014	-7068	7.54E-05	958	0.333333	0.014726	ERCC4
3919.433	5.112538	-7068	7.41E-05	803	0.333333	0.010763	PSMB2
4801.11	7.649285	-7068	7.38E-05	685	0.333333	0.009217	MAPK12
13158.11	6.819792	-7068	7.69E-05	1234	0.333333	0.018925	DLGAP1
7208.511	6.54954	-7068	7.48E-05	904	0.333333	0.01175	APOE
1457.004	6.940028	-7068	7.25E-05	405	0.333333	0.006249	ZNF24
3895.504	10.92494	-7068	7.28E-05	439	0.333333	0.005473	IL17A
5748.3	7.377406	-7068	7.44E-05	763	0.333333	0.011596	PDE8B
6620.031	6.343825	-7068	7.45E-05	866	0.333333	0.011969	CYCS
5552.31	8.418785	-7068	7.40E-05	684	0.333333	0.009752	AQP3
5112.19	7.667634	-7068	7.41E-05	692	0.333333	0.010479	SYT11
6737.833	7.608603	-7068	7.46E-05	808	0.333333	0.011258	HYAL1
4517.63	6.875618	-7068	7.39E-05	698	0.333333	0.00959	CCL13
6700.294	6.177226	-7068	7.47E-05	888	0.333333	0.011199	CFI
5625.945	7.306481	-7068	7.44E-05	765	0.25	0.011747	ABCA6
16451.37	6.340227	-7068	7.77E-05	1437	0.333333	0.021153	NEDD9
5655.977	6.09637	-7068	7.47E-05	871	0.333333	0.012872	HSPA4
3514.839	6.821249	-7068	7.36E-05	638	0.333333	0.009451	MSH3
8386.132	7.425912	-7068	7.49E-05	893	0.333333	0.01166	PEBP1
8793.887	6.600362	-7068	7.57E-05	1039	0.333333	0.015719	BTRC
6276.541	6.390242	-7068	7.46E-05	865	0.25	0.012515	FSTL1
6181.682	7.715767	-7068	7.46E-05	789	0.333333	0.011672	DHCR24

2687.837	8.811845	-7068	7.28E-05	455	0.333333	0.006075	FOXO4
10307.56	7.405899	-7068	7.56E-05	1016	0.333333	0.014745	TLR3
2470.794	7.469646	-7068	7.31E-05	513	0.333333	0.007928	ZBTB1
5787.319	7.658583	-7068	7.41E-05	731	0.333333	0.009561	PKMYT1
6042.208	8.711563	-7068	7.41E-05	698	0.333333	0.009902	LRP5
4887.494	8.099212	-7068	7.39E-05	667	0.333333	0.009785	LRP6
6915.268	7.702721	-7068	7.46E-05	818	0.333333	0.011994	HHEX
3782.253	6.011303	-7068	7.38E-05	689	0.333333	0.009186	CCL4
5285.644	6.500193	-7068	7.45E-05	810	0.333333	0.011893	ANXA11
9733.991	6.607423	-7068	7.61E-05	1105	0.333333	0.016945	INSIG1
4236.014	8.738609	-7068	7.35E-05	582	0.333333	0.0082	APOBEC3C
3051.244	7.627069	-7068	7.33E-05	550	0.333333	0.008164	SFXN3
16431.44	7.090202	-7068	7.75E-05	1338	0.333333	0.020436	CTDSPL
5434.708	5.770038	-7068	7.44E-05	865	0.333333	0.011011	POLA2
9382.007	7.268163	-7068	7.56E-05	991	0.333333	0.014659	ENPEP
10848.49	6.466318	-7068	7.64E-05	1186	0.333333	0.018313	CBX5
10557.11	6.831374	-7068	7.61E-05	1105	0.333333	0.016108	ACSL1
4275.211	7.665438	-7068	7.39E-05	654	0.333333	0.009732	COA1
4057.637	7.05692	-7068	7.38E-05	662	0.333333	0.009781	PRKAA1
5470.07	6.350475	-7068	7.43E-05	801	0.333333	0.010285	SDHA
8843.891	6.151813	-7068	7.57E-05	1066	0.333333	0.015519	VCAN
4614.213	7.758635	-7068	7.39E-05	670	0.333333	0.009631	NQO1
7003.219	6.230107	-7068	7.52E-05	958	0.333333	0.014343	PTPRB
3034.287	8.131759	-7068	7.30E-05	505	0.333333	0.006802	CNGB1
5015.962	5.10775	-7068	7.47E-05	894	0.333333	0.012582	SMC4
3892.921	8.061332	-7068	7.35E-05	590	0.333333	0.008376	PYCR1
6560.808	5.448123	-7068	7.52E-05	1002	0.333333	0.014135	BUB3
4698.695	6.294538	-7068	7.46E-05	802	0.333333	0.013178	MAPK1IP1L
9553.867	7.285029	-7068	7.55E-05	991	0.333333	0.014431	LGALS3
7337.224	8.476657	-7068	7.46E-05	773	0.333333	0.012077	ABTB2
2302.582	7.179845	-7068	7.30E-05	491	0.333333	0.007625	NXT2
7961.866	7.848911	-7068	7.50E-05	870	0.333333	0.013231	LPCAT3
8722.122	6.377379	-7068	7.55E-05	1036	0.333333	0.014268	GOT1
6315.862	7.158699	-7068	7.43E-05	806	0.333333	0.010315	F12
10469.76	6.498525	-7068	7.63E-05	1141	0.333333	0.01765	MOXD1
6069.451	6.837076	-7068	7.47E-05	834	0.333333	0.012167	TMBIM6
6563.731	7.866572	-7068	7.44E-05	768	0.333333	0.011343	SIX1
3613.639	6.587323	-7068	7.34E-05	637	0.333333	0.008265	TRADD
5619.756	8.570333	-7068	7.39E-05	663	0.333333	0.009037	CRTC1
7377.257	6.919836	-7068	7.51E-05	916	0.333333	0.01377	MYO5A
4788.904	6.182081	-7068	7.44E-05	795	0.333333	0.011716	UBE2L3

7991.409	6.114777	-7068	7.57E-05	1042	0.333333	0.015886	ATP6V1H
5672.421	9.203996	-7068	7.37E-05	630	0.333333	0.008379	OVOL2
2709.607	8.165259	-7068	7.31E-05	506	0.333333	0.007556	SGPL1
19821.58	7.039847	-7068	7.83E-05	1481	0.333333	0.022589	GALNT10
5773.528	7.030398	-7068	7.44E-05	784	0.333333	0.011213	LTF
5622	8.47614	-7068	7.39E-05	672	0.333333	0.009486	INHA
2553.381	8.5529	-7068	7.28E-05	457	0.333333	0.006672	RAB40B
3356.351	7.659331	-7068	7.31E-05	528	0.333333	0.006541	S100A7
4126.666	6.733503	-7068	7.39E-05	677	0.333333	0.009666	MRPS11
3473.524	8.128232	-7068	7.32E-05	547	0.333333	0.007235	ATOX1
9082.365	7.499268	-7068	7.54E-05	967	0.333333	0.014534	POLDIP3
8462.464	7.066334	-7068	7.54E-05	969	0.333333	0.014267	KMO
5477.74	8.240214	-7068	7.40E-05	692	0.333333	0.010004	SPINK1
5439.422	7.324417	-7068	7.44E-05	757	0.333333	0.011708	ERP44
2072.288	8.741436	-7068	7.21E-05	358	0.333333	0.004079	CRX
3184.159	7.419427	-7068	7.32E-05	557	0.333333	0.007734	ELN
14505.47	7.965891	-7068	7.57E-05	1091	0.333333	0.014279	NDUFV2
6050.867	6.656932	-7068	7.47E-05	834	0.333333	0.012269	SLC2A3
6591.145	8.019543	-7068	7.45E-05	783	0.333333	0.01191	LPIN2
7733.279	8.874912	-7068	7.44E-05	760	0.333333	0.010412	CEL
7595.203	7.668046	-7068	7.48E-05	848	0.333333	0.012122	PTAFR
5856.013	6.908592	-7068	7.48E-05	827	0.333333	0.013111	DIP2C
4550.062	6.978337	-7068	7.42E-05	731	0.333333	0.010982	ST6GAL1
6853.101	6.679818	-7068	7.47E-05	871	0.333333	0.011469	CRP
5176.654	8.389856	-7068	7.39E-05	660	0.333333	0.009704	ABCA12
2659.522	9.725112	-7068	7.26E-05	410	0.333333	0.00541	GNAT2
2340.448	6.242967	-7068	7.31E-05	542	0.333333	0.008127	CEP57
5077.604	7.576556	-7068	7.41E-05	700	0.333333	0.010472	MXRA5
4282.749	8.247522	-7068	7.35E-05	585	0.333333	0.007381	TECR
3429.447	6.679327	-7068	7.36E-05	641	0.333333	0.009668	TGIF1
7654.538	7.694188	-7068	7.49E-05	855	0.333333	0.012316	ADGRG1
9714.583	6.47246	-7068	7.62E-05	1104	0.333333	0.016934	ZFAND5
6568.353	6.218536	-7068	7.51E-05	931	0.333333	0.013851	ETS2
8270.894	7.800672	-7068	7.48E-05	857	0.333333	0.011995	LIN7A
6128.145	8.033473	-7068	7.38E-05	686	0.333333	0.008935	NDUFS6
5465.425	12.86956	-7068	7.27E-05	425	0.333333	0.004474	P2RX3
3098.107	6.156287	-7068	7.36E-05	655	0.333333	0.009981	GRB10
3849.845	6.511774	-7068	7.40E-05	698	0.333333	0.011301	RTF1
2048.963	6.582848	-7068	7.29E-05	496	0.333333	0.00758	DNAJB4
4180.063	7.373282	-7068	7.38E-05	658	0.333333	0.009572	CSF1
4752.989	7.886278	-7068	7.37E-05	649	0.333333	0.008583	CSTB

7473.793	7.061784	-7068	7.52E-05	908	0.333333	0.013969	PLEKHA6
4986.318	5.54074	-7068	7.45E-05	844	0.333333	0.011673	HMMR
7111.996	5.806287	-7068	7.54E-05	1015	0.333333	0.014812	SMARCA4
5591.575	6.739116	-7068	7.47E-05	823	0.333333	0.012809	AHCYL1
3855.677	7.797082	-7068	7.30E-05	550	0.333333	0.006477	APOM
3437.421	6.923066	-7068	7.36E-05	623	0.333333	0.009643	ABCA1
2707.062	10.21737	-7068	7.24E-05	392	0.333333	0.005404	GPR161
23880.64	6.638367	-7068	7.94E-05	1693	0.333333	0.025319	SLC7A11
3830.275	6.170721	-7068	7.38E-05	710	0.333333	0.01041	YY1
16260.82	6.121325	-7068	7.78E-05	1484	0.333333	0.021838	XRCC5
4101.54	6.904339	-7068	7.39E-05	687	0.333333	0.010104	PLIN2
5850.405	6.978048	-7068	7.45E-05	799	0.333333	0.011852	PARP2
13842.29	7.046658	-7068	7.66E-05	1217	0.333333	0.018053	HES1
12133.44	7.868852	-7068	7.59E-05	1058	0.333333	0.015178	BDH1
3040.112	7.815433	-7068	7.31E-05	531	0.333333	0.007732	BECN1
1494.034	8.106812	-7068	7.22E-05	361	0.333333	0.004946	NAIP
4658.097	8.027925	-7068	7.38E-05	653	0.333333	0.009388	CHMP7
6002.881	6.882325	-7068	7.43E-05	796	0.333333	0.011369	KL
13764.88	7.458806	-7068	7.63E-05	1163	0.333333	0.016757	SERPINH1
4238.498	9.569827	-7068	7.32E-05	508	0.333333	0.0062	GAST
9102.818	6.07448	-7068	7.59E-05	1100	0.333333	0.01604	NAMPT
2645.089	7.193875	-7068	7.30E-05	523	0.333333	0.007667	DSTN
5082.367	7.623838	-7068	7.41E-05	701	0.333333	0.009947	ACOT7
2944.107	9.342242	-7068	7.28E-05	456	0.333333	0.006208	HR
2415.145	7.829551	-7068	7.27E-05	466	0.333333	0.006141	CTDP1
8783.926	7.453396	-7068	7.54E-05	942	0.333333	0.014464	ZNF711
3031.493	8.276397	-7068	7.29E-05	475	0.333333	0.005962	KRT4
4617.025	8.006102	-7068	7.37E-05	642	0.333333	0.008813	LY6E
4915.91	7.235363	-7068	7.41E-05	723	0.333333	0.010593	P4HA1
2691.427	7.898681	-7068	7.28E-05	480	0.333333	0.006097	THPO
6564.375	8.82298	-7068	7.41E-05	712	0.333333	0.010811	KCTD20
7871.222	5.009965	-7068	7.53E-05	1107	0.333333	0.013984	SMC2
3757.165	6.920065	-7068	7.38E-05	654	0.25	0.010117	ADAMTS3
9057.48	7.052708	-7068	7.57E-05	1009	0.333333	0.015993	PANX1
5443.301	8.861871	-7068	7.39E-05	654	0.333333	0.009491	SBNO2
42695.56	7.112076	-7068	8.26E-05	2153	0.333333	0.032829	KCNIP4
6209.588	6.400736	-7068	7.48E-05	880	0.333333	0.012787	FOSL2
4320.088	8.7491	-7068	7.36E-05	596	0.333333	0.00849	MICB
10705.81	7.648916	-7068	7.58E-05	1039	0.333333	0.015693	IDI1
11149.35	6.942231	-7068	7.61E-05	1109	0.333333	0.015747	CYB5A
16537.33	7.185328	-7068	7.71E-05	1304	0.333333	0.018932	COX5A

8199.903	6.702928	-7068	7.56E-05	1000	0.333333	0.015492	PPID
8213.554	5.637526	-7068	7.57E-05	1101	0.333333	0.015497	ITGB3
3152.64	7.29956	-7068	7.32E-05	576	0.333333	0.008084	TAF15
5086.981	9.379036	-7068	7.34E-05	580	0.333333	0.007614	GLI1
4295.787	8.242656	-7068	7.36E-05	606	0.333333	0.008376	CTSV
1575.77	7.453442	-7068	7.23E-05	386	0.333333	0.00514	NCAM1
3770.79	6.960263	-7068	7.37E-05	652	0.333333	0.009717	SLC25A40
4364.658	7.431143	-7068	7.38E-05	662	0.333333	0.008984	DHCR7
9882.02	7.178415	-7068	7.59E-05	1042	0.333333	0.015737	AGTR1
5177.759	7.328442	-7068	7.42E-05	732	0.333333	0.010907	MAP2
1980.258	8.403425	-7068	7.24E-05	378	0.333333	0.00448	MMP24
2660.467	9.410972	-7068	7.26E-05	423	0.333333	0.005612	SIX3
5190.468	7.525014	-7068	7.41E-05	719	0.333333	0.010958	NOVA1
4870.871	7.491027	-7068	7.40E-05	704	0.333333	0.010571	STX2
4453.24	7.886342	-7068	7.38E-05	652	0.333333	0.009551	HK2
4116.971	9.70686	-7068	7.32E-05	526	0.333333	0.00708	TNFSF9
11486.74	8.634412	-7068	7.57E-05	986	0.333333	0.014564	NMU
6537.549	5.379205	-7068	7.52E-05	1009	0.333333	0.014195	VDAC1
6076.477	7.011962	-7068	7.46E-05	820	0.333333	0.012252	MFGE8
3436.828	11.32293	-7068	7.24E-05	394	0.333333	0.004754	IFNA2
5210.682	7.172946	-7068	7.43E-05	757	0.333333	0.01141	NFE2L3
2355.474	9.504293	-7068	7.25E-05	405	0.333333	0.005234	RENBP
9419.316	6.482937	-7068	7.61E-05	1086	0.333333	0.016733	DNAJC9
9376.302	6.43152	-7068	7.60E-05	1089	0.333333	0.016231	TLE1
5525.28	6.729591	-7068	7.39E-05	746	0.333333	0.008809	CFB
4592.068	7.166736	-7068	7.39E-05	693	0.333333	0.009996	RGS16
2757.342	5.417042	-7068	7.34E-05	630	0.25	0.008756	MFAP5
4140.434	6.017615	-7068	7.41E-05	734	0.333333	0.010935	HTATSF1
2860.588	7.502524	-7068	7.31E-05	531	0.333333	0.008093	GRM8
2039.75	5.777424	-7068	7.30E-05	526	0.333333	0.00768	GHITM
3503.307	6.282882	-7068	7.38E-05	667	0.333333	0.009717	CAPZB
4726.768	6.656782	-7068	7.41E-05	745	0.333333	0.010498	NOTCH3
3407.344	6.066297	-7068	7.35E-05	669	0.333333	0.009069	ETS1
4076.71	6.466223	-7068	7.37E-05	676	0.333333	0.009218	TAF5
14836.27	6.489612	-7068	7.73E-05	1349	0.333333	0.019989	RET
6829.705	8.216782	-7068	7.45E-05	779	0.333333	0.011952	VWA5A
8557.801	13.24403	-7068	7.33E-05	537	0.333333	0.006117	OCM2
4328.375	8.063505	-7068	7.35E-05	602	0.25	0.008111	PZP
6074.924	5.584031	-7068	7.49E-05	945	0.333333	0.013452	NID1
8129.354	7.368573	-7068	7.50E-05	896	0.333333	0.012824	TRPC6
1400.166	9.138209	-7068	7.19E-05	299	0.333333	0.003465	PNLIPRP1

5115.542	8.369253	-7068	7.38E-05	651	0.333333	0.009288	UCP3
10167.93	8.35525	-7068	7.51E-05	926	0.333333	0.013494	FABP4
6556.35	6.250007	-7068	7.49E-05	915	0.333333	0.013107	FGFR1
5511.298	7.292598	-7068	7.42E-05	738	0.333333	0.010208	RHOC
5625.706	6.6236	-7068	7.47E-05	821	0.333333	0.012608	STAM2
4034.263	6.258898	-7068	7.42E-05	734	0.333333	0.011573	LAMP2
3414.731	5.362884	-7068	7.39E-05	742	0.333333	0.010514	PGK1
1898.222	8.8273	-7068	7.23E-05	380	0.333333	0.005347	RPRD2
2382.193	6.452755	-7068	7.30E-05	528	0.333333	0.007227	LSM4
4557.72	8.11736	-7068	7.33E-05	596	0.333333	0.007004	NR1I3
6847.586	7.558776	-7068	7.48E-05	849	0.333333	0.012989	GLUL
1722.587	6.266495	-7068	7.27E-05	460	0.333333	0.00625	HSF1
3791.586	8.291827	-7068	7.32E-05	543	0.333333	0.006831	GH1
2843.283	8.230729	-7068	7.28E-05	485	0.333333	0.006479	SMAD9
4652.138	7.16836	-7068	7.35E-05	658	0.333333	0.008259	GZMB
3972.679	5.370006	-7068	7.35E-05	712	0.333333	0.007421	C8B
5573.622	7.209941	-7068	7.45E-05	779	0.333333	0.012178	ACKR3
9207.763	7.314695	-7068	7.58E-05	1004	0.333333	0.015513	SLC29A1
6008.282	8.756287	-7068	7.41E-05	684	0.333333	0.009564	HSPB6
2014.738	6.985122	-7068	7.26E-05	459	0.333333	0.005759	MAP2K7
5901.856	5.916881	-7068	7.47E-05	885	0.333333	0.012222	NEK2
5645.827	8.839541	-7068	7.35E-05	623	0.333333	0.00801	AGR2
7731.049	6.958397	-7068	7.54E-05	941	0.333333	0.014686	RGS2
12060.09	7.461403	-7068	7.60E-05	1101	0.333333	0.016134	SLC39A8
6898.165	8.555225	-7068	7.41E-05	717	0.333333	0.009139	ST14
2961.98	10.33279	-7068	7.25E-05	405	0.333333	0.005183	RGR
16801.65	5.300572	-7068	7.79E-05	1588	0.333333	0.02148	ZWINT
3737.382	11.5235	-7068	7.28E-05	432	0.333333	0.005054	RTEL1
6889.243	7.018735	-7068	7.49E-05	874	0.333333	0.012812	GM2A
10211.78	5.993759	-7068	7.59E-05	1146	0.333333	0.01608	ATP5C1
2944.548	6.723769	-7068	7.34E-05	587	0.333333	0.008854	ATP6V1C1
5433.416	9.030498	-7068	7.39E-05	633	0.333333	0.00878	SGSH
5804.941	7.465056	-7068	7.43E-05	754	0.333333	0.011378	ZCCHC24
9286.577	6.352234	-7068	7.60E-05	1092	0.333333	0.016521	WHSC1
4498.761	6.346264	-7068	7.41E-05	758	0.333333	0.011225	ACAA2
5941.122	6.126185	-7068	7.49E-05	894	0.333333	0.013296	ARF6
6276.567	8.802758	-7068	7.41E-05	694	0.333333	0.009772	SLC25A20
6362.1	7.125835	-7068	7.49E-05	843	0.333333	0.012723	SLC9A1
2344.231	7.861305	-7068	7.28E-05	463	0.333333	0.006539	SEMA3F
3907.301	12.53001	-7068	7.26E-05	407	0.333333	0.005087	RIBC2
2249.249	7.416454	-7068	7.27E-05	474	0.333333	0.006547	IRF4

9959.387	6.705546	-7068	7.60E-05	1073	0.333333	0.015903	TBX5
3568.162	7.715918	-7068	7.32E-05	567	0.333333	0.007535	COL2A1
3059.121	9.777092	-7068	7.28E-05	446	0.333333	0.005847	GUCA1B
13604.85	6.236569	-7068	7.71E-05	1326	0.333333	0.019608	FLT1
3055.393	7.68566	-7068	7.32E-05	539	0.333333	0.008006	SNTB2
9484.577	6.358926	-7068	7.60E-05	1112	0.333333	0.01667	SKP2
8615.337	7.306936	-7068	7.56E-05	957	0.333333	0.015134	SCAPER
6051.348	7.546857	-7068	7.46E-05	781	0.333333	0.012181	DOCK10
1343.998	10.64806	-7068	7.17E-05	257	0.333333	0.003413	CLCNKA
3565.966	9.415749	-7068	7.31E-05	494	0.333333	0.006338	ZKSCAN3
3276.512	11.03149	-7068	7.26E-05	416	0.333333	0.005667	DOCK3
5526.974	7.262573	-7068	7.44E-05	756	0.333333	0.011721	SNX3
8429.583	8.183625	-7068	7.50E-05	860	0.333333	0.012808	LAD1
4305.542	8.768395	-7068	7.31E-05	545	0.333333	0.006788	LY6D
6804.86	6.14035	-7068	7.52E-05	945	0.333333	0.013897	KIF14
6958.694	6.885926	-7068	7.49E-05	877	0.333333	0.012638	IFI27
4488.426	6.030139	-7068	7.38E-05	722	0.333333	0.008807	CFHR2
4678.94	9.871704	-7068	7.35E-05	566	0.333333	0.007918	SYNGR3
4390.432	5.799421	-7068	7.39E-05	744	0.25	0.009884	FBLN2
5746.741	6.348927	-7068	7.46E-05	847	0.333333	0.012027	TTF2
4062.62	7.541809	-7068	7.36E-05	621	0.333333	0.008735	OLR1
2889.49	6.882229	-7068	7.31E-05	549	0.333333	0.006861	CASP10
9917.593	6.665232	-7068	7.56E-05	1035	0.333333	0.014215	TLR2
9840.955	8.395105	-7068	7.52E-05	913	0.333333	0.013526	RGS10
6972.781	7.552767	-7068	7.47E-05	837	0.333333	0.012414	GDF15
9769.875	5.751081	-7068	7.60E-05	1155	0.333333	0.015857	CXCL8
3019.383	9.192031	-7068	7.28E-05	459	0.333333	0.005634	GCAT
2538.475	8.602766	-7068	7.28E-05	468	0.333333	0.00669	DHODH
4010.644	6.502597	-7068	7.35E-05	653	0.333333	0.008246	COX4I1
1072.049	7.752478	-7068	7.19E-05	320	0.333333	0.004797	PIGC
5608.957	7.40079	-7068	7.43E-05	744	0.333333	0.010355	TUBB3
3025.286	6.865433	-7068	7.34E-05	588	0.333333	0.008787	KCNB1
4143.412	10.02315	-7068	7.32E-05	518	0.333333	0.007208	PTP4A3
6909.262	7.522411	-7068	7.47E-05	836	0.333333	0.012261	TRIM14
10826.41	6.565343	-7068	7.63E-05	1141	0.333333	0.017636	ST3GAL6
4213.411	5.210574	-7068	7.41E-05	800	0.333333	0.010666	FBLN1
8231.981	8.372624	-7068	7.49E-05	840	0.333333	0.012178	RASA3
5301.271	8.406428	-7068	7.39E-05	663	0.333333	0.009763	HRH1
2064.825	9.692293	-7068	7.24E-05	376	0.333333	0.005558	MGA
5034.54	7.668027	-7068	7.40E-05	689	0.333333	0.010316	INPP4A
7894.361	9.859403	-7068	7.42E-05	725	0.333333	0.010229	PITX1

5742.999	7.516651	-7068	7.44E-05	769	0.333333	0.011169	LIMK2
6393.896	6.292605	-7068	7.47E-05	886	0.333333	0.011723	ACO2
4109.134	7.699587	-7068	7.37E-05	635	0.333333	0.009704	ABHD2
792.8647	10.51558	-7068	7.15E-05	210	0.333333	0.002475	CACTIN
4330.87	9.384746	-7068	7.34E-05	546	0.333333	0.006986	ARID3A
10366.07	6.978367	-7068	7.61E-05	1086	0.333333	0.01682	SLC1A3
8680.588	6.902903	-7068	7.56E-05	1002	0.333333	0.015355	STOM
2729.859	8.435356	-7068	7.30E-05	484	0.333333	0.006988	CAPN15
6241.09	7.144357	-7068	7.48E-05	829	0.333333	0.013054	SLC20A2
7344.401	5.923712	-7068	7.53E-05	1015	0.333333	0.014297	SF3B3
7581.007	7.521561	-7068	7.47E-05	842	0.333333	0.011182	PCOLCE
9141.679	6.884124	-7068	7.56E-05	1017	0.333333	0.015123	SORBS1
3742.876	6.080865	-7068	7.38E-05	672	0.333333	0.009153	PLEK
1538.665	8.55782	-7068	7.23E-05	364	0.333333	0.0053	ATXN7
4580.907	8.090025	-7068	7.35E-05	606	0.333333	0.00782	CLDN7
8944.429	7.83258	-7068	7.53E-05	929	0.333333	0.014001	ST3GAL4
12250.31	6.409475	-7068	7.69E-05	1249	0.333333	0.019452	MYH10
4269.759	7.40704	-7068	7.38E-05	663	0.333333	0.009664	IFI6
4111.261	5.914721	-7068	7.39E-05	740	0.333333	0.01004	ITGAX
4685.799	7.227733	-7068	7.42E-05	716	0.333333	0.010985	CDC42EP4
2564.618	9.787909	-7068	7.26E-05	407	0.333333	0.005172	SLC29A2
4526.957	6.879216	-7068	7.42E-05	727	0.333333	0.011329	BICD1
3030.362	7.538239	-7068	7.27E-05	489	0.25	0.005537	C8G
4341.973	7.025288	-7068	7.40E-05	697	0.333333	0.010149	CX3CL1
9173.216	7.187704	-7068	7.56E-05	991	0.333333	0.015104	NEFM
5171.361	8.551948	-7068	7.39E-05	664	0.333333	0.009727	HSD17B1
5072.195	10.93445	-7068	7.30E-05	488	0.333333	0.005579	CTRB2
5178.143	8.029576	-7068	7.39E-05	676	0.333333	0.00952	TFF3
2582.399	9.208197	-7068	7.27E-05	432	0.333333	0.005885	IFT27
3006.045	8.334861	-7068	7.30E-05	504	0.333333	0.006769	RASSF1
7132.289	7.026769	-7068	7.51E-05	902	0.333333	0.014151	ARIH2
2522.737	9.318855	-7068	7.25E-05	417	0.333333	0.005492	CST6
7436.909	7.064892	-7068	7.49E-05	896	0.333333	0.01266	IL2RA
1898.233	6.973153	-7068	7.27E-05	453	0.333333	0.006609	CHERP
6211.2	6.884118	-7068	7.48E-05	844	0.333333	0.012821	PLXNA2
2393.233	6.062587	-7068	7.31E-05	569	0.333333	0.008685	CTNND1
5027.313	6.939948	-7068	7.43E-05	757	0.333333	0.01165	CPEB3
6277.405	8.126765	-7068	7.44E-05	753	0.333333	0.011053	LUZP1
23796.53	7.02538	-7068	7.92E-05	1627	0.333333	0.024837	CACNA1C
3174.727	4.579033	-7068	7.36E-05	748	0.333333	0.009065	MRE11A
2492.995	4.235077	-7068	7.33E-05	651	0.333333	0.007872	SPC25

16632.35	6.093743	-7068	7.79E-05	1494	0.333333	0.021808	MELK
5075.663	6.462304	-7068	7.44E-05	799	0.333333	0.011614	CTTN
3948.023	7.642823	-7068	7.35E-05	614	0.333333	0.008309	AKR1A1
5784.978	7.407806	-7068	7.44E-05	769	0.333333	0.011558	SERPINB8
5770.195	7.669493	-7068	7.43E-05	743	0.333333	0.010598	PPP1R15A
6188.941	6.968432	-7068	7.45E-05	811	0.333333	0.011653	COL8A1
3335.849	8.079245	-7068	7.34E-05	555	0.333333	0.008006	OGG1
5637.147	4.969408	-7068	7.46E-05	960	0.333333	0.012006	AURKA
15604.53	6.534234	-7068	7.77E-05	1393	0.333333	0.021093	KLF6
9606.706	7.164942	-7068	7.58E-05	1028	0.333333	0.015899	PLA2G4A
1760.975	6.780186	-7068	7.25E-05	432	0.333333	0.005741	NOTCH4
4052.51	8.107553	-7068	7.34E-05	581	0.333333	0.007938	BUD31
2455.935	8.108158	-7068	7.28E-05	459	0.333333	0.005985	CDC34
6947.794	7.165007	-7068	7.49E-05	867	0.333333	0.013453	SEMA5A
2467.174	9.053243	-7068	7.27E-05	420	0.333333	0.005732	MYH6
6451.332	7.359989	-7068	7.45E-05	811	0.333333	0.012037	CLIC5
2029.843	8.276433	-7068	7.23E-05	398	0.333333	0.004967	HNF1A
14442.3	6.808283	-7068	7.72E-05	1289	0.333333	0.019232	SLC8A1
5077.396	6.337723	-7068	7.47E-05	824	0.333333	0.01335	N4BP1
5988.576	7.346873	-7068	7.44E-05	788	0.333333	0.011723	EDN3
5551.454	6.075719	-7068	7.44E-05	820	0.333333	0.010893	CXCL1
11125.02	6.479514	-7068	7.64E-05	1175	0.333333	0.017267	TF
8132.598	6.470783	-7068	7.55E-05	1003	0.333333	0.015065	DST
3517.574	8.826527	-7068	7.32E-05	522	0.333333	0.007205	INPP5J
7635.245	8.375354	-7068	7.46E-05	797	0.333333	0.011693	SLC16A4
3874.954	6.975719	-7068	7.38E-05	671	0.333333	0.009945	TMEM106C
2792.194	6.399993	-7068	7.35E-05	604	0.333333	0.009738	VAMP4
1842.055	9.25667	-7068	7.22E-05	351	0.333333	0.005339	KLHL20
3533.195	9.916634	-7068	7.30E-05	475	0.333333	0.006452	LIPE
16745.25	6.089392	-7068	7.83E-05	1530	0.333333	0.023549	TMPO
8900.789	6.370044	-7068	7.59E-05	1073	0.333333	0.016394	MYO10
4890.173	7.460454	-7068	7.42E-05	713	0.333333	0.010969	MRPL33
4870.748	7.425319	-7068	7.41E-05	713	0.333333	0.010772	GATM
7583.578	6.854851	-7068	7.50E-05	922	0.333333	0.013538	HPGD
7781.159	9.075296	-7068	7.46E-05	779	0.333333	0.010934	SCO2
7787.41	6.921695	-7068	7.52E-05	939	0.333333	0.013453	PKM
6130.622	7.459074	-7068	7.45E-05	798	0.333333	0.011696	ELL
4637.311	7.550734	-7068	7.40E-05	683	0.333333	0.010327	INTS10
6655.836	7.457069	-7068	7.44E-05	799	0.333333	0.010575	HSD11B1
2860.821	7.277136	-7068	7.30E-05	517	0.333333	0.006475	APOF
4308.561	6.775722	-7068	7.41E-05	708	0.333333	0.010412	HCCS

3589.014	5.122504	-7068	7.41E-05	766	0.333333	0.010521	YBX1
9276.224	3.636165	-7068	7.62E-05	1463	0.333333	0.016435	CDK1
2782.938	9.483628	-7068	7.27E-05	428	0.333333	0.005397	HAVCR1
2219.606	8.600227	-7068	7.26E-05	426	0.333333	0.006113	TMCC2
3158.359	7.54432	-7068	7.31E-05	545	0.333333	0.00721	ATP5G2
10657.57	7.215129	-7068	7.60E-05	1065	0.333333	0.016115	B3GALNT1
13475.94	6.378741	-7068	7.72E-05	1314	0.333333	0.019888	SPRY1
2496.293	8.092039	-7068	7.26E-05	447	0.333333	0.005747	VGLL1
6679.693	6.822146	-7068	7.49E-05	883	0.333333	0.013129	CACYBP
3936.231	8.218212	-7068	7.36E-05	600	0.333333	0.008892	DKK1
3130.161	4.946745	-7068	7.37E-05	715	0.333333	0.009886	TIMP3
15932.13	5.472963	-7068	7.78E-05	1538	0.333333	0.021407	COL1A2
2040.06	8.278304	-7068	7.26E-05	398	0.333333	0.004808	POLRMT
4489.62	9.572663	-7068	7.34E-05	555	0.333333	0.007732	MPI
3268.504	6.553879	-7068	7.35E-05	622	0.333333	0.008718	PRDX2
7384.238	8.839651	-7068	7.44E-05	747	0.333333	0.010556	SERPINF1
1912.959	7.49666	-7068	7.25E-05	418	0.333333	0.005231	SLC11A1
4143.304	8.674254	-7068	7.36E-05	605	0.333333	0.009412	YME1L1
3320.112	6.92862	-7068	7.33E-05	580	0.333333	0.00757	CXCL3
4808.873	9.956273	-7068	7.31E-05	512	0.333333	0.006055	KIR3DL3
8001.99	5.826457	-7068	7.56E-05	1060	0.333333	0.015527	MSH6
2284.57	7.90649	-7068	7.26E-05	439	0.333333	0.005552	ATP5I
1257.656	7.221568	-7068	7.22E-05	352	0.333333	0.004711	ARHGAP45
3665.301	7.024336	-7068	7.36E-05	623	0.333333	0.008678	MMP11
5570.769	9.267484	-7068	7.38E-05	641	0.333333	0.0091	MTF1
9856.167	8.645549	-7068	7.52E-05	899	0.333333	0.013128	NUDT1
5600.519	5.584757	-7068	7.47E-05	887	0.333333	0.012445	LUM
9283.292	6.783655	-7068	7.60E-05	1052	0.333333	0.016263	CHD1L
2291.134	7.076568	-7068	7.29E-05	493	0.333333	0.007333	SEC22B
6355.896	7.671715	-7068	7.44E-05	763	0.333333	0.011318	PFKFB3
5362.688	8.028269	-7068	7.38E-05	666	0.333333	0.008832	KLK8
3428.856	5.688595	-7068	7.38E-05	699	0.333333	0.009585	TPI1
1700.328	9.662983	-7068	7.21E-05	324	0.333333	0.004383	HOXC6
1851.642	7.340011	-7068	7.27E-05	439	0.333333	0.006326	MYDGF
3446.62	7.258121	-7068	7.36E-05	605	0.333333	0.009272	ALDH1A3
4052.269	4.91088	-7068	7.41E-05	806	0.333333	0.010245	COL1A1
7315.639	8.077232	-7068	7.47E-05	820	0.333333	0.012262	DBH
4974.019	7.915912	-7068	7.41E-05	684	0.333333	0.010584	SMPDL3A
4595.929	6.524433	-7068	7.42E-05	747	0.333333	0.011135	NR4A2
5697.094	7.88547	-7068	7.42E-05	720	0.333333	0.010164	MARCO
7315.692	7.53301	-7068	7.47E-05	819	0.333333	0.011163	S100A2

6969.198	9.260402	-7068	7.41E-05	717	0.333333	0.009795	TUBB4A
3234.108	5.566068	-7068	7.29E-05	595	0.333333	0.006923	NDUFB3
3581.973	9.415839	-7068	7.30E-05	491	0.333333	0.00649	NFATC4
5248.724	5.591172	-7068	7.45E-05	847	0.333333	0.011236	CCL2
6437.201	7.18916	-7068	7.46E-05	806	0.333333	0.011356	S100A4
2788.149	7.304562	-7068	7.30E-05	527	0.333333	0.007577	LPAR1
5591.33	7.168623	-7068	7.45E-05	772	0.333333	0.011928	NMNAT2
5951.17	7.562507	-7068	7.44E-05	759	0.333333	0.011102	GPRC5B
4408.163	7.680912	-7068	7.37E-05	648	0.333333	0.009145	ESR2
1697.478	9.594314	-7068	7.22E-05	338	0.333333	0.004804	MTMR7
7723.347	7.356417	-7068	7.52E-05	904	0.333333	0.013724	ARG2
6569.269	6.807136	-7068	7.46E-05	857	0.333333	0.012199	FASLG
2611.078	6.00529	-7068	7.34E-05	603	0.333333	0.009149	DDX3X
3400.635	6.99593	-7068	7.36E-05	621	0.333333	0.009398	UTRN
4615.248	7.927524	-7068	7.37E-05	640	0.333333	0.008884	CAPN6
4702.296	6.804452	-7068	7.40E-05	727	0.333333	0.010761	GALNT3
2719.783	7.854952	-7068	7.30E-05	505	0.333333	0.007449	IKZF1
1160.905	6.098106	-7068	7.13E-05	290	0.333333	0.002843	ZNF92
3885.282	7.015787	-7068	7.38E-05	650	0.333333	0.009894	TSPYL1
15674.36	6.778109	-7068	7.75E-05	1349	0.333333	0.020853	DTNA
3756.944	6.586122	-7068	7.38E-05	678	0.333333	0.00983	HMGCS1
1209.874	5.531521	-7068	7.25E-05	431	0.333333	0.00681	SUB1
10637.95	5.197666	-7068	7.65E-05	1306	0.333333	0.018342	LAMA4
6296.88	4.099255	-7068	7.52E-05	1138	0.333333	0.014165	SRSF1
8577.25	7.813371	-7068	7.50E-05	888	0.333333	0.01297	GLI2
6997.019	9.507972	-7068	7.40E-05	681	0.333333	0.00917	P2RY6
2211.1	4.58485	-7068	7.31E-05	639	0.333333	0.008247	PRIM2
2733.178	8.214731	-7068	7.29E-05	478	0.333333	0.006841	FLG
8097.096	6.78222	-7068	7.55E-05	985	0.333333	0.015012	DUSP10
2692.882	7.469986	-7068	7.30E-05	509	0.333333	0.007128	MMP10
5960.828	6.892033	-7068	7.46E-05	816	0.333333	0.012243	MT1X
2682.982	6.016655	-7068	7.33E-05	595	0.333333	0.008561	COL4A6
17973.79	6.498487	-7068	7.83E-05	1496	0.333333	0.023075	PTHLH
2020.098	7.844335	-7068	7.24E-05	418	0.333333	0.005183	GRIN1
1963.712	6.977021	-7068	7.28E-05	466	0.333333	0.007341	TMCO1
7576.944	7.512749	-7068	7.51E-05	879	0.333333	0.013209	GPLD1
4055.724	6.601414	-7068	7.38E-05	691	0.333333	0.010074	RBM3
9581.598	6.35495	-7068	7.60E-05	1103	0.333333	0.016763	KITLG
5709.344	7.581336	-7068	7.43E-05	760	0.333333	0.011516	ENOX2
1932.769	9.590609	-7068	7.23E-05	359	0.25	0.005083	RUNDC3B
2099.06	8.884428	-7068	7.25E-05	390	0.333333	0.005374	ADGRG2

6954.044	6.58404	-7068	7.51E-05	909	0.333333	0.013278	DUSP1
9373.306	6.959496	-7068	7.58E-05	1026	0.333333	0.015651	PHLDA1
2831.721	8.110728	-7068	7.27E-05	462	0.333333	0.005529	GZMH
5175.711	8.412456	-7068	7.38E-05	641	0.333333	0.008655	CFD
5544.624	7.367491	-7068	7.42E-05	738	0.333333	0.0108	BMP5
7192.82	7.313678	-7068	7.47E-05	858	0.333333	0.012198	MASP1
2861.814	8.210227	-7068	7.30E-05	498	0.333333	0.006649	ESRRA
6711.811	6.777304	-7068	7.49E-05	887	0.333333	0.012974	DBI
9629.771	5.767922	-7068	7.62E-05	1173	0.333333	0.01698	MCL1
18812.39	6.430323	-7068	7.85E-05	1534	0.333333	0.023389	NRP2
7318.194	6.460378	-7068	7.46E-05	886	0.333333	0.011392	COX5B
1501.703	6.64093	-7068	7.25E-05	423	0.333333	0.006446	MIOS
5295.386	6.653114	-7068	7.44E-05	786	0.25	0.011924	HTRA1
5047.703	6.712198	-7068	7.41E-05	755	0.333333	0.010501	PGR
4728.753	7.682527	-7068	7.40E-05	688	0.333333	0.010032	ELL2
3541.991	8.236113	-7068	7.30E-05	533	0.333333	0.007012	CCL17
7434.526	6.031568	-7068	7.51E-05	979	0.333333	0.012733	UBE2C
2313.49	8.705309	-7068	7.26E-05	432	0.333333	0.005933	TEP1
6709.329	8.230985	-7068	7.44E-05	760	0.333333	0.011489	TNR
2697.624	6.200314	-7068	7.29E-05	546	0.25	0.006528	CFHR4
3865.043	5.513094	-7068	7.39E-05	735	0.333333	0.009763	STIL
5149.226	7.88742	-7068	7.41E-05	704	0.333333	0.010801	MBD1
2113.657	7.104532	-7068	7.27E-05	467	0.333333	0.006527	IFI44L
7048.269	6.927134	-7068	7.47E-05	865	0.333333	0.011213	CFL1
5404.081	7.847268	-7068	7.42E-05	717	0.333333	0.010965	PROCR
4275.578	7.498341	-7068	7.37E-05	635	0.333333	0.008784	SCNN1A
3673.368	6.101169	-7068	7.40E-05	695	0.333333	0.010425	STMN1
1917.682	6.178892	-7068	7.29E-05	497	0.333333	0.007502	RSL1D1
3907.93	7.687372	-7068	7.34E-05	589	0.333333	0.007687	ALDH6A1
8978.019	8.591966	-7068	7.49E-05	863	0.333333	0.012618	MTUS2
8418.008	6.866318	-7068	7.54E-05	974	0.333333	0.014552	CA12
3293.41	6.317572	-7068	7.38E-05	650	0.333333	0.009814	PROS1
3787.263	8.713321	-7068	7.32E-05	535	0.333333	0.007731	CASR
2275.113	6.835555	-7068	7.28E-05	487	0.333333	0.006453	PSG9
9554.062	7.175065	-7068	7.57E-05	1011	0.333333	0.015718	AHNAK
1610.472	7.813334	-7068	7.24E-05	384	0.333333	0.005234	TADA3
7060.633	6.378797	-7068	7.49E-05	922	0.333333	0.012718	SERPINE1
2316.928	6.055935	-7068	7.33E-05	567	0.333333	0.008739	VAMP3
4646.76	8.082713	-7068	7.38E-05	643	0.333333	0.009368	GOLIM4
5874.546	5.867146	-7068	7.47E-05	891	0.333333	0.012608	THBD
6368.405	6.46635	-7068	7.50E-05	894	0.333333	0.014027	OLFM1

4980.462	5.867445	-7068	7.42E-05	796	0.333333	0.010664	POLE2
2415.93	6.927288	-7068	7.30E-05	510	0.333333	0.007368	TSFM
3982.009	10.51571	-7068	7.29E-05	481	0.333333	0.006705	MMP20
3930.799	8.05422	-7068	7.36E-05	601	0.25	0.009229	CCKBR
1492.583	9.353417	-7068	7.21E-05	321	0.333333	0.004337	SLC26A10
2063.775	8.821458	-7068	7.24E-05	401	0.333333	0.005898	B4GALT6
7429.941	7.389302	-7068	7.51E-05	881	0.333333	0.013468	CEP152
5834.659	6.670757	-7068	7.43E-05	796	0.333333	0.010258	HPX
5155.463	6.914382	-7068	7.42E-05	761	0.333333	0.010832	DNAJB1
8766.39	6.892195	-7068	7.57E-05	1017	0.333333	0.015866	RCAN1
4147.265	6.954051	-7068	7.37E-05	674	0.333333	0.009463	ACOX1
5086.303	8.927493	-7068	7.37E-05	620	0.333333	0.008876	HOMER2
5226.278	7.908823	-7068	7.40E-05	701	0.333333	0.010791	MYCN
5098.545	6.526879	-7068	7.42E-05	755	0.333333	0.010746	MAPKAPK5
3935.323	7.176336	-7068	7.38E-05	644	0.333333	0.009025	PLEC
3363.234	9.108528	-7068	7.30E-05	487	0.333333	0.006476	CRLF1
5408.665	7.043346	-7068	7.45E-05	778	0.333333	0.012331	SATB2
10174.6	6.587078	-7068	7.59E-05	1089	0.333333	0.016065	THBS2
3857.459	8.956585	-7068	7.33E-05	539	0.333333	0.008203	RHOBTB3
9469.3	6.536177	-7068	7.62E-05	1087	0.333333	0.017096	ABCC4
3761.659	9.251323	-7068	7.32E-05	512	0.333333	0.006787	HIC1
3271.276	6.5694	-7068	7.35E-05	617	0.333333	0.008734	CXCL10
10255.7	7.810949	-7068	7.49E-05	928	0.333333	0.01197	RBP4
4654.199	8.553588	-7068	7.35E-05	605	0.333333	0.008647	PTX3
5677.83	7.195166	-7068	7.45E-05	780	0.333333	0.012023	SSH1
16533.83	6.388601	-7068	7.81E-05	1450	0.333333	0.022461	SLIT3
5472.989	7.304852	-7068	7.41E-05	737	0.333333	0.010123	MSMO1
8959.323	7.001168	-7068	7.57E-05	1008	0.333333	0.015307	AQP1
3231.786	4.886704	-7068	7.40E-05	744	0.333333	0.010888	FILIP1L
5754.972	7.729828	-7068	7.44E-05	748	0.333333	0.011026	FLOT1
2931.627	5.973552	-7068	7.35E-05	628	0.333333	0.008873	MAP2K3
4227.283	6.917988	-7068	7.38E-05	682	0.333333	0.009334	ITGA7
3283.645	11.39426	-7068	7.24E-05	377	0.333333	0.004308	LCN1
7047.587	7.32966	-7068	7.50E-05	862	0.333333	0.013213	PARN
4890.193	7.689961	-7068	7.39E-05	670	0.333333	0.009463	LAMP3
13400.5	6.913987	-7068	7.68E-05	1227	0.333333	0.018157	RAB3B
3431.848	8.206864	-7068	7.34E-05	555	0.333333	0.008549	THAP3
2169.381	7.819022	-7068	7.26E-05	432	0.333333	0.005612	CLEC10A
1821.501	9.466024	-7068	7.22E-05	355	0.333333	0.004713	AMH
4789.716	9.718278	-7068	7.34E-05	562	0.333333	0.007631	PAK4
4102.32	8.001595	-7068	7.37E-05	616	0.333333	0.008863	NADK

5562.537	7.727393	-7068	7.42E-05	715	0.333333	0.009974	RNASEH2A
2180.74	9.817573	-7068	7.23E-05	378	0.333333	0.00513	MICA
5036.383	6.551791	-7068	7.43E-05	756	0.333333	0.010366	KIF22
2570.666	7.269764	-7068	7.32E-05	526	0.333333	0.007979	TLE3
7998.801	7.274498	-7068	7.51E-05	919	0.333333	0.013861	CELSR1
3194.505	8.64701	-7068	7.31E-05	508	0.333333	0.007461	RNF216
3670.085	11.07785	-7068	7.26E-05	427	0.333333	0.005546	TFEB
6405.652	21.93054	-7068	7.24E-05	391	0.333333	0.005385	UGT2B17
6582.21	18.53424	-7068	7.25E-05	439	0.333333	0.005594	UGT2B15
3962.832	5.829715	-7068	7.41E-05	747	0.25	0.011071	ITGA1
12968.21	7.055701	-7068	7.68E-05	1203	0.333333	0.018975	EFNA5
3251.848	7.836807	-7068	7.33E-05	550	0.333333	0.008095	VIP
7014.89	6.968605	-7068	7.51E-05	895	0.333333	0.013986	NET1
4672.929	5.406213	-7068	7.45E-05	854	0.333333	0.012574	HNRNPA3
3283.556	4.63705	-7068	7.41E-05	794	0.333333	0.011531	KHDRBS1
7102.175	8.05255	-7068	7.46E-05	783	0.333333	0.01153	CAPN9
6095.82	6.532891	-7068	7.48E-05	874	0.333333	0.012966	WEE1
6272.492	7.831893	-7068	7.43E-05	750	0.333333	0.010633	PDLIM4
19995.56	7.053701	-7068	7.85E-05	1490	0.333333	0.023104	ELOVL6
2949.094	9.162051	-7068	7.30E-05	476	0.333333	0.006713	GFER
6319.352	6.530112	-7068	7.49E-05	882	0.25	0.013247	ATP6V0E1
8096.537	7.817241	-7068	7.50E-05	878	0.333333	0.012938	UNC5B
5751.699	5.253453	-7068	7.46E-05	942	0.333333	0.012781	HSPA8
4558.613	5.882296	-7068	7.45E-05	814	0.333333	0.012429	NUP50
8361.895	6.033454	-7068	7.58E-05	1081	0.333333	0.016585	TPM4
10319.14	6.369694	-7068	7.61E-05	1124	0.333333	0.016268	LAMC2
4750.073	7.957583	-7068	7.36E-05	646	0.333333	0.009047	PF4V1
6403.608	5.640893	-7068	7.52E-05	980	0.333333	0.014386	IGFBP3
8791.469	6.709273	-7068	7.55E-05	1010	0.333333	0.014742	BAX
3451.448	11.06868	-7068	7.25E-05	411	0.333333	0.004885	B3GAT3
5278.204	6.612711	-7068	7.43E-05	796	0.333333	0.011295	NUP62
1301.52	11.01502	-7068	7.18E-05	264	0.333333	0.003076	CCDC57
3080.513	11.22755	-7068	7.24E-05	382	0.333333	0.004264	UCN
3488.489	6.940705	-7068	7.36E-05	618	0.333333	0.009174	PLXND1
6484.395	6.693691	-7068	7.48E-05	868	0.333333	0.012842	SKAP2
5974.511	8.79541	-7068	7.40E-05	691	0.333333	0.010246	LITAF
1585.305	5.353368	-7068	7.27E-05	491	0.333333	0.007308	PPAT
3133.347	6.724796	-7068	7.35E-05	606	0.333333	0.009018	CS
11064.56	7.260561	-7068	7.61E-05	1075	0.333333	0.015693	OLFM4
4155.88	6.116055	-7068	7.39E-05	726	0.333333	0.010524	UBE2D2
6161.054	7.31902	-7068	7.46E-05	810	0.333333	0.012832	CNIH3

8472.938	12.24337	-7068	7.35E-05	581	0.333333	0.008362	TRO
6147.889	7.091937	-7068	7.48E-05	835	0.333333	0.013108	DIAPH2
8484.744	7.354057	-7068	7.53E-05	940	0.333333	0.013319	EBP
4771.482	4.757236	-7068	7.43E-05	866	0.333333	0.01076	DBF4
5005.615	10.77984	-7068	7.32E-05	528	0.333333	0.007002	CALCR
19330.21	6.789872	-7068	7.82E-05	1496	0.333333	0.022717	RSU1
2970.214	9.103383	-7068	7.29E-05	470	0.333333	0.006458	PTGER1
8385.26	7.99018	-7068	7.47E-05	843	0.333333	0.011665	SERPINB5
8795.413	6.432276	-7068	7.58E-05	1053	0.333333	0.016381	ETV1
2437.443	5.718567	-7068	7.29E-05	547	0.25	0.006482	ADH6
4215.04	8.199634	-7068	7.35E-05	603	0.333333	0.008714	HSPB3
2941.783	9.336696	-7068	7.28E-05	462	0.333333	0.006634	LDOC1
6278.492	6.421422	-7068	7.47E-05	860	0.333333	0.011746	CXCL2
4079.588	5.756477	-7068	7.41E-05	759	0.333333	0.010758	CHAF1A
11856.1	6.420428	-7068	7.67E-05	1237	0.333333	0.018249	ANXA2
5786.905	7.336907	-7068	7.45E-05	792	0.333333	0.012055	ABCC1
4038.37	9.762727	-7068	7.30E-05	496	0.333333	0.006247	RBP3
5160.331	7.7217	-7068	7.42E-05	718	0.333333	0.010889	SLC39A14
7471.127	6.685146	-7068	7.53E-05	947	0.25	0.01473	ABCA8
3686.469	6.613212	-7068	7.37E-05	672	0.333333	0.0099	CALR
11498.11	8.440075	-7068	7.55E-05	970	0.333333	0.013643	SDS
8977.598	6.080281	-7068	7.59E-05	1101	0.333333	0.016396	NRP1
1841.902	8.668849	-7068	7.24E-05	380	0.333333	0.004907	STK19
2731.337	7.991262	-7068	7.31E-05	505	0.333333	0.007531	CYB5R1
2165.258	7.388478	-7068	7.27E-05	458	0.333333	0.006084	SRRT
16438.87	7.644331	-7068	7.71E-05	1264	0.333333	0.018935	DHRS2
5212.755	8.44405	-7068	7.34E-05	613	0.333333	0.008187	DNA2
4099.054	9.627207	-7068	7.30E-05	488	0.333333	0.006104	RHO
3412.946	6.631214	-7068	7.37E-05	640	0.333333	0.009931	GPNMB
5383.224	6.482875	-7068	7.48E-05	830	0.333333	0.013643	MTUS1
2123.643	7.741262	-7068	7.29E-05	456	0.333333	0.007216	TUB
2940.932	8.106945	-7068	7.29E-05	499	0.333333	0.006846	SPDEF
3643.854	7.365633	-7068	7.36E-05	624	0.333333	0.00947	ENSA
983.1272	6.420334	-7068	7.22E-05	356	0.333333	0.005633	PLAA
4030.532	7.070969	-7068	7.38E-05	668	0.333333	0.010119	HUS1
13884.3	7.072089	-7068	7.71E-05	1238	0.333333	0.019533	PCLO
1713.342	5.132446	-7068	7.27E-05	497	0.333333	0.006292	NOP16
12584.11	6.387301	-7068	7.67E-05	1266	0.333333	0.018568	CHEK2
8721.656	7.36815	-7068	7.53E-05	952	0.333333	0.014085	PPP1R3C
9263.995	7.745406	-7068	7.54E-05	948	0.333333	0.013137	TKT
4555.376	7.276632	-7068	7.42E-05	712	0.333333	0.011224	PTGER4

5678.408	7.129961	-7068	7.43E-05	767	0.333333	0.010802	NR1I2
3965.472	7.480058	-7068	7.38E-05	636	0.333333	0.009868	ZC2HC1A
1101.121	9.84231	-7068	7.18E-05	264	0.333333	0.003379	GFRA2
3434.52	10.95685	-7068	7.25E-05	405	0.333333	0.004741	FOXC2
5021.28	7.689839	-7068	7.42E-05	706	0.333333	0.011206	SLITRK5
2729.718	9.731334	-7068	7.27E-05	422	0.333333	0.006067	MT4
3440.132	6.73477	-7068	7.36E-05	633	0.333333	0.009301	GK
2939.852	6.602926	-7068	7.31E-05	585	0.333333	0.007764	CASP2
966.8374	8.31431	-7068	7.17E-05	276	0.333333	0.003638	TLR6
1271.699	7.800399	-7068	7.22E-05	348	0.333333	0.005257	EIF2AK1
3615.095	7.314985	-7068	7.35E-05	594	0.333333	0.007942	CXCL5
3430.224	6.966257	-7068	7.36E-05	627	0.333333	0.009162	ITGA2B
4215.656	8.960375	-7068	7.34E-05	568	0.333333	0.007767	ALDOC
8559.622	7.680559	-7068	7.54E-05	929	0.333333	0.014026	BLVRA
6630.789	6.828163	-7068	7.51E-05	887	0.333333	0.014012	SBSPON
2473.61	8.554312	-7068	7.28E-05	448	0.333333	0.0062	RHBDF1
2526.547	5.76098	-7068	7.30E-05	556	0.333333	0.006818	NOC2L
3300.346	8.064557	-7068	7.31E-05	533	0.333333	0.006907	TNFRSF9
1559	8.771182	-7068	7.22E-05	357	0.333333	0.005142	PIN4
5314.46	9.345041	-7068	7.37E-05	614	0.333333	0.008821	SSX2IP
1969.412	8.378959	-7068	7.24E-05	406	0.333333	0.005447	RUNX2
3141.452	7.483946	-7068	7.33E-05	561	0.333333	0.008465	LAMB4
576.8927	9.410915	-7068	7.13E-05	184	0.333333	0.002192	POM121L2
1179.47	10.63795	-7068	7.17E-05	254	0.333333	0.003055	PDIA2
1335.547	6.006212	-7068	7.26E-05	441	0.25	0.007437	SLC24A5
6161.647	6.724064	-7068	7.45E-05	834	0.333333	0.011589	HNF4A
978.892	5.441593	-7068	7.22E-05	383	0.333333	0.005976	COX11
5273.615	8.879768	-7068	7.37E-05	627	0.333333	0.008034	ADAM15
3355.628	7.57141	-7068	7.34E-05	577	0.333333	0.008711	ELOVL2
2286.642	6.017121	-7068	7.30E-05	537	0.333333	0.007401	IDH3B
1626.596	8.403784	-7068	7.23E-05	367	0.333333	0.004991	CALCA
6074.551	6.744272	-7068	7.48E-05	852	0.333333	0.012934	ST8SIA4
1953.255	5.161916	-7068	7.29E-05	551	0.333333	0.007386	RPL22
6640.516	7.448517	-7068	7.46E-05	833	0.333333	0.01212	GGCX
5831.261	6.982606	-7068	7.40E-05	744	0.333333	0.009776	EFEMP2
1340.861	10.50161	-7068	7.18E-05	271	0.333333	0.00337	ASPHD1
2884.095	7.813493	-7068	7.32E-05	524	0.333333	0.007715	NTS
5866.149	8.329442	-7068	7.38E-05	663	0.333333	0.008525	KLK7
3308.797	8.324138	-7068	7.31E-05	525	0.333333	0.007435	MPZL2
5420.148	7.08103	-7068	7.46E-05	780	0.333333	0.012183	EPB41L1
8719.631	7.38218	-7068	7.54E-05	949	0.333333	0.014671	SERPINE2

8990.292	7.853346	-7068	7.53E-05	923	0.333333	0.013842	JCHAIN
5902.538	7.600091	-7068	7.44E-05	770	0.333333	0.011812	SLC1A4
4078.975	5.659069	-7068	7.41E-05	766	0.333333	0.010714	CFLAR
7464.985	5.766638	-7068	7.53E-05	1008	0.333333	0.014496	FBLN5
6821.993	5.578057	-7068	7.50E-05	976	0.333333	0.013173	EXOSC2
7022.367	6.523576	-7068	7.52E-05	929	0.333333	0.014107	OSMR
1599.238	8.118197	-7068	7.22E-05	346	0.333333	0.00406	SLC12A3
3646.503	7.695215	-7068	7.35E-05	593	0.333333	0.008439	CBR3
3677.875	6.422182	-7068	7.39E-05	679	0.333333	0.010403	AREG
7519.68	7.015642	-7068	7.52E-05	918	0.333333	0.013874	CYP7B1
2980.965	8.625765	-7068	7.29E-05	481	0.333333	0.00619	TWF2
2224.496	9.026734	-7068	7.24E-05	389	0.333333	0.005121	IL11
2118.868	6.800734	-7068	7.27E-05	473	0.333333	0.006238	PRPF31
10301.62	5.63273	-7068	7.64E-05	1232	0.333333	0.017624	CENPF
2241.921	6.317363	-7068	7.29E-05	517	0.25	0.007223	PLN
2945.797	7.45059	-7068	7.33E-05	550	0.333333	0.008242	ERCC8
1855.422	7.624955	-7068	7.25E-05	415	0.333333	0.006215	KYAT3
883.8501	7.422438	-7068	7.18E-05	284	0.333333	0.003702	NPRL3
3348.653	7.284482	-7068	7.34E-05	589	0.333333	0.008173	IPO9
7218.645	6.456543	-7068	7.52E-05	946	0.333333	0.013806	SAT1
2600.843	7.427026	-7068	7.31E-05	519	0.333333	0.007962	ACSBG1
4005.472	9.199079	-7068	7.33E-05	544	0.333333	0.008394	SRR
9830.296	7.009347	-7068	7.60E-05	1056	0.333333	0.016541	HIP1
10196.7	22.20196	-7068	7.32E-05	523	0.333333	0.007977	UGT8
9509.587	7.588253	-7068	7.50E-05	946	0.333333	0.013123	RBPMS
7800.864	6.606498	-7068	7.55E-05	973	0.333333	0.015295	FAM110B
292.8149	10.29821	-7068	7.09E-05	138	0.333333	0.001914	FAM149B1
2397.652	6.653585	-7068	7.27E-05	492	0.333333	0.00584	SRD5A2
20756.25	6.749291	-7068	7.86E-05	1549	0.333333	0.02356	MSR1
6522.291	7.324109	-7068	7.49E-05	841	0.333333	0.013349	C11orf80
3717.631	6.626027	-7068	7.39E-05	673	0.333333	0.010022	TSC22D3
11730.97	6.782156	-7068	7.65E-05	1183	0.333333	0.017508	ACACA
3839.643	6.706605	-7068	7.38E-05	659	0.333333	0.009449	AIF1
3489.301	4.555476	-7068	7.41E-05	817	0.333333	0.011385	PRKDC
2485.085	10.98753	-7068	7.22E-05	351	0.333333	0.004298	OGFR
17547.72	6.653121	-7068	7.79E-05	1449	0.333333	0.021488	MKI67
3950.773	5.829084	-7068	7.40E-05	756	0.333333	0.010928	SMC1A
2366.686	7.020491	-7068	7.32E-05	522	0.25	0.008336	LGR5
3059.536	6.845867	-7068	7.36E-05	609	0.333333	0.009978	PHTF2
5929.028	7.054203	-7068	7.45E-05	806	0.333333	0.012147	LOXL2
3612.414	5.953635	-7068	7.33E-05	668	0.333333	0.007793	RXRA

2096.806	8.698794	-7068	7.26E-05	415	0.333333	0.005623	ZBTB7A
2171.912	8.087532	-7068	7.28E-05	448	0.333333	0.006658	HIRIP3
8576.977	9.113887	-7068	7.46E-05	800	0.333333	0.011588	PDE6B
5057.648	9.555303	-7068	7.34E-05	591	0.333333	0.008502	RBP1
2140.151	6.539842	-7068	7.30E-05	510	0.333333	0.008019	CHTOP
4074.716	8.540028	-7068	7.32E-05	554	0.333333	0.007201	MT3
5038.124	6.523011	-7068	7.44E-05	786	0.333333	0.011863	IFI44
4478.415	4.886934	-7068	7.43E-05	847	0.333333	0.010839	GINS1
6335.02	7.384314	-7068	7.47E-05	817	0.333333	0.012519	RANBP9
3867.247	8.668735	-7068	7.34E-05	565	0.333333	0.008224	NPC1
16827.29	6.906079	-7068	7.76E-05	1366	0.333333	0.020949	ATP6AP2
2986.087	6.887252	-7068	7.34E-05	587	0.333333	0.008789	CDKN1C
4799.837	7.398413	-7068	7.42E-05	706	0.333333	0.010758	SLC12A4
2449.405	9.263035	-7068	7.26E-05	417	0.333333	0.006289	ARMCX4
1874.09	9.111224	-7068	7.21E-05	330	0.333333	0.003771	RS1
4759.357	7.904653	-7068	7.38E-05	656	0.333333	0.00952	CDHR1
6413.569	8.004843	-7068	7.42E-05	727	0.333333	0.009766	CCL20
2601.722	8.439872	-7068	7.29E-05	462	0.333333	0.006107	AATK
3736.332	7.906672	-7068	7.34E-05	585	0.333333	0.008465	SEMA4F
2323.586	8.922711	-7068	7.27E-05	432	0.25	0.006582	TRAF3IP1
11432.2	7.105818	-7068	7.63E-05	1121	0.333333	0.017078	PLD1
3573.35	7.173573	-7068	7.35E-05	613	0.333333	0.008528	LDLR
3439.407	8.453242	-7068	7.31E-05	526	0.333333	0.007463	SLC5A12
3442.271	6.187823	-7068	7.35E-05	644	0.333333	0.008379	POLR2L
1944.221	11.16398	-7068	7.22E-05	336	0.25	0.004801	ZSCAN12
3509.625	8.419048	-7068	7.32E-05	546	0.333333	0.007474	ABCF2
6252.093	6.904578	-7068	7.44E-05	808	0.333333	0.010753	MT1G
4628.13	8.974503	-7068	7.36E-05	596	0.333333	0.008499	SEPHS2
5551.271	7.644317	-7068	7.41E-05	720	0.333333	0.010323	KCNJ8
5672.408	7.343814	-7068	7.42E-05	757	0.333333	0.010264	CDT1
5226.65	6.990057	-7068	7.45E-05	773	0.333333	0.011856	COL4A3
15173.2	7.758911	-7068	7.68E-05	1211	0.333333	0.017864	GLRX3
89.14807	7.134292	-7068	7.01E-05	96	0.25	0.001371	HSPA1B
3559.08	7.705914	-7068	7.33E-05	559	0.333333	0.007515	COX6A1
5072.648	6.522445	-7068	7.42E-05	778	0.333333	0.010994	RIPK2
3867.902	6.682047	-7068	7.38E-05	670	0.333333	0.009858	RGS1
12940.51	9.531923	-7068	7.53E-05	932	0.333333	0.014299	MEOX2
1754.468	8.557981	-7068	7.24E-05	376	0.333333	0.005016	RASL10A
1081.126	6.329548	-7068	7.20E-05	354	0.333333	0.005077	KDM5A
1228.96	7.614206	-7068	7.21E-05	351	0.25	0.005098	TOB2
5750.702	8.327994	-7068	7.42E-05	714	0.333333	0.010846	ABCD4

3187.819	7.975729	-7068	7.33E-05	546	0.333333	0.008311	CLMN
4149.198	6.485455	-7068	7.40E-05	701	0.333333	0.01016	SAMM50
2178.221	6.61577	-7068	7.29E-05	510	0.333333	0.007265	DAG1
7488.207	6.576652	-7068	7.51E-05	938	0.333333	0.01313	PLAUR
4860.009	8.816359	-7068	7.36E-05	614	0.333333	0.008462	LMAN2
1833.404	9.923641	-7068	7.21E-05	324	0.333333	0.003765	PDX1
6012.438	7.755413	-7068	7.44E-05	763	0.333333	0.011109	FADS2
2646.229	7.475317	-7068	7.29E-05	501	0.333333	0.006419	ERCC2
4509.578	7.350116	-7068	7.39E-05	660	0.333333	0.009728	PGAM1
10479.69	5.787067	-7068	7.65E-05	1223	0.333333	0.018065	KIF11
4064.473	8.257978	-7068	7.35E-05	580	0.333333	0.008437	CHRDL1
12469.68	8.118096	-7068	7.60E-05	1039	0.333333	0.014984	PDZK1IP1
3863.134	8.07846	-7068	7.36E-05	603	0.333333	0.009201	RPL39L
3731.611	9.653754	-7068	7.30E-05	502	0.333333	0.006261	HTRA2
7912.983	7.419778	-7068	7.51E-05	909	0.333333	0.013672	ACACB
3581.647	7.508844	-7068	7.35E-05	599	0.333333	0.008655	CDC42EP2
4508.373	6.417169	-7068	7.40E-05	718	0.333333	0.009673	RAD54L
5262.091	8.204961	-7068	7.39E-05	672	0.333333	0.009514	TXN2
11150.22	6.773998	-7068	7.63E-05	1132	0.333333	0.016544	SLC27A2
8245.549	7.031004	-7068	7.50E-05	908	0.333333	0.012216	S100A8
8169.587	6.912426	-7068	7.50E-05	930	0.333333	0.013155	DSP
2667.11	7.737079	-7068	7.29E-05	496	0.333333	0.006864	HFE
12785.64	6.804803	-7068	7.69E-05	1222	0.333333	0.018982	WWC1
1675.283	7.755132	-7068	7.25E-05	398	0.333333	0.006014	RSAD2
12095.5	6.855542	-7068	7.64E-05	1172	0.333333	0.017293	LAMA1
5997.048	7.888517	-7068	7.42E-05	716	0.333333	0.009357	FCN3
3770.601	8.442099	-7068	7.33E-05	567	0.333333	0.008338	HRK
5834.362	6.090736	-7068	7.49E-05	892	0.333333	0.013278	RBM8A
4099.95	8.105421	-7068	7.35E-05	592	0.333333	0.008158	RRAD
13511.69	7.126091	-7068	7.70E-05	1229	0.333333	0.019486	SOX4
6700.396	6.893636	-7068	7.49E-05	874	0.333333	0.013355	TFPI
2149.42	8.009901	-7068	7.26E-05	433	0.333333	0.006262	PIGH
4251.94	7.218933	-7068	7.40E-05	676	0.333333	0.009976	MYL12B
6155.44	7.058561	-7068	7.48E-05	836	0.333333	0.013227	ABCG1
6981.413	8.114449	-7068	7.45E-05	777	0.333333	0.011349	SLC15A1
9881.433	7.05626	-7068	7.59E-05	1062	0.333333	0.016067	HGF
4446.058	6.055363	-7068	7.44E-05	761	0.333333	0.01128	NCAPH
6600.167	8.017948	-7068	7.43E-05	748	0.333333	0.009913	SOX10
2223.476	9.262213	-7068	7.25E-05	410	0.333333	0.005945	TGIF2
3676.972	9.775874	-7068	7.23E-05	436	0.333333	0.004786	ZNF135
4745.5	8.153356	-7068	7.33E-05	598	0.333333	0.007274	NDUFS7

4033.456	8.47736	-7068	7.34E-05	578	0.333333	0.008022	PLPP2
899.8988	8.994193	-7068	7.16E-05	261	0.25	0.003622	TTC9
3310.243	8.47847	-7068	7.32E-05	526	0.333333	0.007324	ST6GALNAC2
2946.122	7.548807	-7068	7.33E-05	546	0.333333	0.008449	PRSS23
1512.219	6.651661	-7068	7.27E-05	432	0.333333	0.006931	ASAH1
3702.395	8.083017	-7068	7.35E-05	584	0.333333	0.008465	CPT1A
10452.02	6.727001	-7068	7.61E-05	1111	0.333333	0.017051	AKAP13
4543.735	4.892678	-7068	7.44E-05	866	0.333333	0.011688	THBS1
3399.312	9.486529	-7068	7.30E-05	483	0.333333	0.006714	RAB27B
13045.13	6.799203	-7068	7.67E-05	1218	0.333333	0.017756	TRIB1
2680.315	6.810848	-7068	7.29E-05	519	0.333333	0.006791	NEFL
3556.729	7.16024	-7068	7.34E-05	598	0.333333	0.008401	IL4
18185.29	6.418967	-7068	7.88E-05	1536	0.333333	0.024558	MECOM
2021.636	7.686793	-7068	7.24E-05	400	0.333333	0.004755	FCAR
2707.134	7.175923	-7068	7.31E-05	537	0.333333	0.007696	CDK12
3367.037	7.564663	-7068	7.34E-05	580	0.333333	0.008625	DDX52
1634.01	11.13915	-7068	7.20E-05	290	0.333333	0.003882	USP2
11059.77	7.078341	-7068	7.63E-05	1113	0.333333	0.017557	KPNA4
1338.261	7.930652	-7068	7.21E-05	347	0.333333	0.004756	BAIAP2
4323.883	8.410275	-7068	7.37E-05	609	0.333333	0.00904	MCF2L
6002.101	5.753556	-7068	7.49E-05	930	0.333333	0.013184	CDC25A
4687.851	11.98969	-7068	7.27E-05	450	0.333333	0.005755	P2RY10
3138.598	6.229985	-7068	7.36E-05	635	0.333333	0.009634	RAD1
9779.17	6.018886	-7068	7.64E-05	1166	0.333333	0.017972	MERTK
7936.429	7.499584	-7068	7.49E-05	863	0.333333	0.011941	VSIG4
9428.426	7.303427	-7068	7.55E-05	993	0.333333	0.014613	ABCC3
4655.013	9.006739	-7068	7.35E-05	578	0.333333	0.007528	DHRS1
6306.762	8.494589	-7068	7.39E-05	707	0.333333	0.009618	SREBF1
8373.018	5.692433	-7068	7.58E-05	1107	0.333333	0.016326	CPSF6
5363.253	7.752348	-7068	7.41E-05	712	0.333333	0.010356	EFNA1
5600.562	7.611592	-7068	7.41E-05	723	0.333333	0.01003	OASL
5881.256	6.921978	-7068	7.47E-05	819	0.333333	0.012712	SLC16A7
9235.768	6.674922	-7068	7.54E-05	1036	0.333333	0.014213	CEBPB
5463.363	6.216699	-7068	7.44E-05	848	0.333333	0.011791	ATF3
8151.113	7.889139	-7068	7.47E-05	835	0.333333	0.011433	CLCA2
1540.189	6.11884	-7068	7.27E-05	455	0.333333	0.007465	SMCHD1
4444.085	8.467828	-7068	7.37E-05	625	0.333333	0.009565	KIF1C
4163.488	9.194968	-7068	7.34E-05	554	0.333333	0.007742	CDKN2B
781.0953	8.856152	-7068	7.17E-05	241	0.333333	0.003146	BICDL1
3392.107	8.538796	-7068	7.31E-05	522	0.333333	0.006955	TNFRSF10C
2470.71	7.952602	-7068	7.29E-05	482	0.333333	0.007322	ATP5S

4615.802	8.890544	-7068	7.36E-05	598	0.333333	0.008355	ENDOG
7871.824	6.31716	-7068	7.52E-05	959	0.333333	0.013391	C1R
3721.474	7.74451	-7068	7.35E-05	590	0.333333	0.008208	CYB5R3
3828.407	6.897393	-7068	7.36E-05	654	0.333333	0.009228	TNFRSF10B
1137.228	7.957092	-7068	7.20E-05	321	0.25	0.004804	FRMD4B
4951.771	7.36855	-7068	7.41E-05	713	0.25	0.010701	COBLL1
1618.293	6.506518	-7068	7.26E-05	439	0.333333	0.006571	CD300A
4962.036	6.585447	-7068	7.40E-05	737	0.333333	0.010144	FCGR3A
2004.342	8.349462	-7068	7.27E-05	415	0.333333	0.00625	RASGRP2
2494.985	7.151828	-7068	7.28E-05	503	0.333333	0.006262	TFR2
2663.212	7.860865	-7068	7.27E-05	476	0.333333	0.005921	ATP5J2
3490.079	6.387715	-7068	7.37E-05	660	0.333333	0.009977	CAV2
2100.839	8.737864	-7068	7.26E-05	408	0.333333	0.006165	DMXL2
3735.934	9.143429	-7068	7.32E-05	528	0.333333	0.007688	MPL
4489.325	7.293038	-7068	7.38E-05	674	0.333333	0.009407	TAPBP
3303.924	8.910238	-7068	7.30E-05	508	0.333333	0.006953	TAP2
5645.817	6.838636	-7068	7.45E-05	804	0.333333	0.011743	TAGLN2
1578.3	9.170658	-7068	7.21E-05	336	0.333333	0.004538	RAPGEF3
2959.285	7.193261	-7068	7.31E-05	543	0.333333	0.007485	ACOT13
3583.117	7.263437	-7068	7.35E-05	613	0.333333	0.008899	ABLIM3
2832.864	7.608916	-7068	7.31E-05	521	0.333333	0.007541	ARHGEF5
1884.713	8.913168	-7068	7.25E-05	388	0.333333	0.005822	PNMA2
19030.99	6.857749	-7068	7.84E-05	1487	0.333333	0.023312	SH3PXD2A
2862.794	9.492744	-7068	7.28E-05	439	0.333333	0.005599	CAPN5
1613.001	9.411685	-7068	7.21E-05	327	0.333333	0.004223	SURF2
11400.89	6.809941	-7068	7.65E-05	1156	0.333333	0.018112	TIPARP
5611.097	7.154376	-7068	7.42E-05	764	0.333333	0.010991	PDGFB
2632.966	9.483925	-7068	7.24E-05	410	0.333333	0.004975	ACAN
5457.715	6.837909	-7068	7.42E-05	773	0.333333	0.011413	ANP32E
2552.042	9.625697	-7068	7.26E-05	412	0.25	0.005922	PEG3
3824.118	8.736686	-7068	7.30E-05	520	0.333333	0.006529	PKP3
3071.783	8.886238	-7068	7.30E-05	486	0.333333	0.006524	SLC37A4
3514.859	10.49798	-7068	7.28E-05	452	0.333333	0.006024	OVOL1
4979.242	7.864132	-7068	7.39E-05	669	0.333333	0.009395	AKR1B10
2827.789	8.508378	-7068	7.29E-05	469	0.333333	0.006167	TMPRSS6
3678.016	7.819744	-7068	7.37E-05	606	0.333333	0.009252	FSCN1
4372.322	8.112898	-7068	7.36E-05	614	0.333333	0.00885	NPR2
6594.832	6.914055	-7068	7.51E-05	878	0.333333	0.014035	CPNE3
1726.162	7.773801	-7068	7.26E-05	420	0.333333	0.006437	ATXN3
1782.515	7.628832	-7068	7.25E-05	419	0.333333	0.006402	MACF1
2567.148	7.611934	-7068	7.28E-05	484	0.333333	0.006836	PDGFA

7062.492	7.370942	-7068	7.48E-05	852	0.333333	0.012723	TP63
3463.062	6.945312	-7068	7.34E-05	607	0.333333	0.00845	CHI3L1
3408.536	6.413715	-7068	7.36E-05	648	0.333333	0.00932	ATP6V1D
535.3883	11.80169	-7068	7.09E-05	151	0.333333	0.001655	CCL15
4671.521	7.583291	-7068	7.40E-05	687	0.333333	0.010337	TPM3
2942.357	7.757625	-7068	7.30E-05	522	0.333333	0.006765	SHB
4766.566	6.906831	-7068	7.43E-05	747	0.333333	0.012009	RNF115
1369.721	7.162704	-7068	7.24E-05	394	0.333333	0.006227	GTF2H5
2620.369	8.191367	-7068	7.30E-05	491	0.333333	0.007664	AVIL
2531.793	7.411198	-7068	7.31E-05	520	0.333333	0.007905	SYNJ2
1192.536	7.779435	-7068	7.21E-05	323	0.333333	0.00447	PRRC2B
1952.285	6.627899	-7068	7.27E-05	476	0.333333	0.006607	LAMP1
6972.416	6.161212	-7068	7.49E-05	915	0.333333	0.012177	TUBB4B
2485.6	7.43044	-7068	7.31E-05	508	0.333333	0.007681	SLC7A8
4886.97	7.531265	-7068	7.40E-05	697	0.333333	0.010503	HSPA2
1658.038	6.790118	-7068	7.27E-05	447	0.333333	0.007192	EIF1
2302.849	8.196953	-7068	7.26E-05	444	0.25	0.006174	MATN3
3382.551	8.376673	-7068	7.31E-05	532	0.333333	0.007271	NUPR1
6530.257	6.059139	-7068	7.49E-05	922	0.333333	0.013221	IGFBP5
3916.155	9.125962	-7068	7.31E-05	527	0.333333	0.007557	MIA
4059.489	6.533766	-7068	7.42E-05	717	0.333333	0.011355	TM4SF1
4912.539	7.296002	-7068	7.41E-05	728	0.333333	0.011055	BCAT1
2392.316	8.251461	-7068	7.27E-05	447	0.333333	0.006062	FAM89B
6314.919	5.56777	-7068	7.49E-05	942	0.333333	0.012962	COL6A1
5354.144	6.542919	-7068	7.42E-05	793	0.333333	0.010904	SOCS1
1195.681	8.928397	-7068	7.19E-05	289	0.333333	0.003735	TMEM259
5992.43	7.573639	-7068	7.46E-05	787	0.333333	0.012324	SLC6A6
4368.116	7.648515	-7068	7.36E-05	634	0.333333	0.008646	DDX27
8296.554	7.149051	-7068	7.54E-05	953	0.333333	0.014283	STC1
1945.682	9.880504	-7068	7.22E-05	355	0.333333	0.004657	SCAMP5
1236.91	7.588173	-7068	7.20E-05	335	0.25	0.004644	SEMA6A
4071.245	7.242223	-7068	7.39E-05	661	0.333333	0.010256	CPM
2506.073	8.249477	-7068	7.26E-05	433	0.333333	0.005276	KLK2
13212.94	11.55153	-7068	7.49E-05	847	0.333333	0.011359	ZNF467
2923.49	8.75525	-7068	7.29E-05	469	0.25	0.006709	TNN
11701.23	6.905317	-7068	7.62E-05	1142	0.333333	0.016748	EPAS1
8337.894	6.40354	-7068	7.56E-05	1016	0.333333	0.015373	RHOQ
3155.546	7.429006	-7068	7.34E-05	566	0.333333	0.008536	TMEM97
1796.646	5.410782	-7068	7.29E-05	529	0.333333	0.007765	NBN
1964.782	6.373782	-7068	7.28E-05	459	0.333333	0.006161	HLA-DQB1
2368.579	7.493818	-7068	7.28E-05	476	0.333333	0.006905	P2RY14

3531.297	6.642847	-7068	7.35E-05	627	0.333333	0.00893	IRAK3
3437.044	7.575042	-7068	7.36E-05	605	0.333333	0.009803	PLPP1
2893.871	4.905827	-7068	7.35E-05	676	0.333333	0.00876	CD68
3602.009	7.695086	-7068	7.35E-05	587	0.333333	0.008211	CAPG
4399.924	8.410963	-7068	7.33E-05	585	0.333333	0.007521	CALML3
2295.216	9.347619	-7068	7.24E-05	391	0.333333	0.004836	CYB561D2
7484.032	8.505727	-7068	7.47E-05	812	0.333333	0.011932	GUSB
2587.506	7.547095	-7068	7.29E-05	499	0.333333	0.00724	HEPH
3352.403	8.389883	-7068	7.31E-05	517	0.333333	0.006906	HPGDS
10304.58	7.143532	-7068	7.58E-05	1063	0.333333	0.015344	E2F2
4985.729	7.009212	-7068	7.42E-05	749	0.333333	0.010988	TGM2
17332.85	15.79693	-7068	7.35E-05	614	0.333333	0.009091	ATP5L
2974.7	8.224252	-7068	7.30E-05	500	0.333333	0.007294	PER2
7205.374	6.350473	-7068	7.55E-05	965	0.333333	0.01535	PENK
1457.088	9.90269	-7068	7.21E-05	309	0.333333	0.004286	RASSF8
13471.44	8.891403	-7068	7.60E-05	1039	0.333333	0.015961	GPR135
3826.433	8.751961	-7068	7.34E-05	552	0.333333	0.008138	RGS20
7836.85	6.719862	-7068	7.53E-05	958	0.333333	0.014548	EMP1
10604.54	6.176734	-7068	7.63E-05	1176	0.333333	0.017569	TNC
8442.049	7.743865	-7068	7.50E-05	875	0.333333	0.012419	SPINT1
1684.952	7.22805	-7068	7.25E-05	401	0.333333	0.005288	ILVBL
2498.961	7.315141	-7068	7.31E-05	512	0.333333	0.0073	COMT
7692.713	7.680832	-7068	7.51E-05	874	0.333333	0.013708	OLFML2A
3631.657	7.482524	-7068	7.37E-05	605	0.333333	0.009118	AKR1C3
2007.316	9.330668	-7068	7.22E-05	377	0.333333	0.00504	POU5F1
4603.86	8.549874	-7068	7.38E-05	627	0.333333	0.009595	TCF20
3144.726	8.765303	-7068	7.28E-05	474	0.333333	0.005975	ATP2C2
4124.378	8.427829	-7068	7.35E-05	590	0.333333	0.0083	QPRT
3621.857	6.710496	-7068	7.36E-05	631	0.333333	0.009293	SRSF8
1837.545	6.756042	-7068	7.28E-05	454	0.333333	0.007066	JMJD1C
3461.535	7.15793	-7068	7.37E-05	629	0.333333	0.009963	PAPSS2
6037.044	6.833759	-7068	7.45E-05	817	0.333333	0.011281	TDO2
4942.844	7.70567	-7068	7.40E-05	696	0.333333	0.010346	DDIT4
5044.251	7.337925	-7068	7.43E-05	728	0.333333	0.011209	EPM2A
2561.811	7.925131	-7068	7.30E-05	488	0.333333	0.007231	MYO7A
2731.794	5.848809	-7068	7.31E-05	587	0.333333	0.007101	SLCO1B1
3882.844	6.410681	-7068	7.37E-05	689	0.333333	0.009958	HBEGF
4887.475	6.965196	-7068	7.40E-05	728	0.333333	0.010195	PPARA
2905.309	7.268389	-7068	7.31E-05	541	0.333333	0.007548	THRA
3547.027	8.578051	-7068	7.33E-05	542	0.333333	0.007614	CHP1
2915.308	8.179649	-7068	7.29E-05	494	0.333333	0.006893	TIMM8A

3996.389	5.883399	-7068	7.42E-05	762	0.333333	0.011494	IDH3A
2231.521	8.197451	-7068	7.25E-05	423	0.25	0.005575	SFTPB
3130.518	7.090209	-7068	7.32E-05	561	0.333333	0.007645	EXOSC7
2144.029	8.705064	-7068	7.26E-05	417	0.333333	0.006042	GAL
2340.284	9.32922	-7068	7.25E-05	403	0.333333	0.005661	HOXA5
6109.734	7.833377	-7068	7.45E-05	769	0.333333	0.011951	PHLPP1
1667.176	8.923947	-7068	7.22E-05	370	0.333333	0.005616	PSPH
1316.274	6.528932	-7068	7.24E-05	401	0.333333	0.00626	SAR1A
5164.473	4.898687	-7068	7.48E-05	940	0.333333	0.012684	CCNB2
5418.037	5.357501	-7068	7.46E-05	900	0.333333	0.012099	PLK4
2282.09	10.14718	-7068	7.24E-05	378	0.333333	0.004919	ATPAF2
2068.545	7.705597	-7068	7.27E-05	432	0.333333	0.006114	CLDN5
1589.896	8.24453	-7068	7.23E-05	376	0.333333	0.005406	SLC35A2
3697.24	4.463684	-7068	7.33E-05	710	0.333333	0.007646	NDUFS3
6046	8.266583	-7068	7.40E-05	711	0.333333	0.010774	NPAS2
2684.923	10.12037	-7068	7.25E-05	393	0.333333	0.005439	MUC7
3067.452	8.244338	-7068	7.30E-05	497	0.333333	0.006743	BDKRB2
5176.502	6.113791	-7068	7.47E-05	816	0.333333	0.012555	DLGAP5
5609.132	6.18063	-7068	7.48E-05	862	0.333333	0.013212	FANCI
2684.205	6.212885	-7068	7.32E-05	588	0.333333	0.00824	NR4A1
2406.794	4.501557	-7068	7.35E-05	670	0.333333	0.00935	DHFR
2868.855	5.96931	-7068	7.35E-05	621	0.333333	0.009309	CCNE2
3649.241	6.753808	-7068	7.38E-05	664	0.333333	0.009988	PARVB
5133.72	6.651637	-7068	7.47E-05	812	0.333333	0.013563	MDFIC
4157.189	6.983015	-7068	7.40E-05	686	0.333333	0.010663	MMP16
3033.953	7.294013	-7068	7.32E-05	554	0.333333	0.007635	HIPK2
1773.844	8.908163	-7068	7.23E-05	373	0.25	0.005465	CDR1
1453.611	9.483856	-7068	7.22E-05	327	0.333333	0.004674	RNF24
6513.031	5.536302	-7068	7.49E-05	954	0.333333	0.012458	NDC80
7369.394	5.971944	-7068	7.54E-05	1001	0.333333	0.01454	RRM2
24544.33	11.56645	-7068	7.59E-05	1029	0.333333	0.015629	VAC14
4018.65	9.193656	-7068	7.30E-05	499	0.333333	0.006156	PFKFB1
6316.52	6.677496	-7068	7.47E-05	842	0.333333	0.012341	MRC1
1720.998	9.389834	-7068	7.22E-05	353	0.333333	0.005018	NEK3
3241.569	7.989808	-7068	7.33E-05	546	0.333333	0.008002	HOMER3
9472.694	6.346874	-7068	7.60E-05	1105	0.333333	0.016634	MAPT
2029.871	12.1883	-7068	7.18E-05	280	0.333333	0.003973	R3HCC1L
6435.208	6.988228	-7068	7.49E-05	850	0.333333	0.013063	SNTB1
18143.49	6.812065	-7068	7.83E-05	1454	0.333333	0.022841	MAGI1
3597.858	7.520852	-7068	7.32E-05	564	0.333333	0.007169	S100P
4912.446	7.623317	-7068	7.41E-05	702	0.333333	0.010602	PCYT1A

4300.64	9.27057	-7068	7.33E-05	550	0.333333	0.007763	ZIC2
4887.151	7.669614	-7068	7.40E-05	690	0.333333	0.010249	CCR2
1728.993	8.177375	-7068	7.25E-05	387	0.333333	0.005287	VAMP5
3269.851	9.086272	-7068	7.29E-05	482	0.333333	0.00612	TNFSF14
2969.408	9.755104	-7068	7.27E-05	444	0.333333	0.006265	FOXD1
6732.534	6.341937	-7068	7.50E-05	920	0.333333	0.013495	RFC5
4825.897	9.471119	-7068	7.36E-05	594	0.333333	0.008339	ESPL1
3840.97	6.930179	-7068	7.30E-05	586	0.333333	0.00702	NDUFA6
2453.473	6.154738	-7068	7.32E-05	565	0.333333	0.008734	HMGXB4
714.17	9.689637	-7068	7.15E-05	203	0.333333	0.002485	TBX6
1532.979	9.835759	-7068	7.20E-05	302	0.333333	0.003695	KCNJ13
4591.844	5.247596	-7068	7.39E-05	820	0.333333	0.009942	RAD51
2295.02	9.481377	-7068	7.26E-05	410	0.333333	0.005797	DCAF4
9491.086	7.099098	-7068	7.57E-05	1021	0.333333	0.015727	PLXDC1
2400.534	6.869596	-7068	7.28E-05	506	0.333333	0.006634	SOCS3
5502.631	7.718837	-7068	7.42E-05	732	0.333333	0.010758	PVR
3785.713	7.20558	-7068	7.37E-05	640	0.333333	0.009772	COBL
7463.775	8.038778	-7068	7.48E-05	827	0.333333	0.012438	RNF19B
2402.468	8.375893	-7068	7.25E-05	436	0.333333	0.006238	GABPB1
15307.07	6.688148	-7068	7.76E-05	1349	0.333333	0.020677	OIP5
2123.997	6.274319	-7068	7.30E-05	511	0.333333	0.007842	SPON1
1868.426	7.242843	-7068	7.28E-05	446	0.333333	0.007373	ARMC8
838.0199	8.886252	-7068	7.16E-05	252	0.333333	0.003682	MIA3
1316.518	6.061935	-7068	7.23E-05	400	0.333333	0.006097	CSTF2T
1395.455	6.080363	-7068	7.24E-05	421	0.333333	0.00595	DNAJC8
1062.419	8.010348	-7068	7.11E-05	224	0.333333	0.001656	ZNF548
1318.616	8.703261	-7068	7.20E-05	326	0.25	0.004668	SLC25A14
3193.816	8.29486	-7068	7.29E-05	495	0.333333	0.006283	RECQL4
1359.48	9.070087	-7068	7.20E-05	313	0.333333	0.00398	CYHR1
13045.24	7.049476	-7068	7.70E-05	1212	0.333333	0.019219	PHACTR1
2256.903	6.48701	-7068	7.29E-05	516	0.333333	0.007052	CCNF
1817.35	9.68804	-7068	7.22E-05	346	0.333333	0.004561	ABCB9
2875.9	8.236789	-7068	7.29E-05	491	0.333333	0.00683	CSF2
1396.226	7.819976	-7068	7.22E-05	345	0.333333	0.004385	SIRPB1
9242.153	6.986796	-7068	7.58E-05	1019	0.333333	0.015525	SIRPA
4517.858	6.401727	-7068	7.42E-05	753	0.333333	0.011164	TYMS
1331.563	8.335548	-7068	7.20E-05	324	0.333333	0.004358	ANGPTL7
5294.917	6.248581	-7068	7.44E-05	803	0.333333	0.011779	IGFBP7
4136.048	5.26263	-7068	7.40E-05	774	0.333333	0.009703	CDC45
3893.245	8.660455	-7068	7.31E-05	528	0.333333	0.006527	PTK6
5142.383	7.352617	-7068	7.43E-05	741	0.333333	0.011925	CAMSAP1

2600.177	7.036472	-7068	7.30E-05	528	0.333333	0.007793	RARRES1
6118.751	6.866138	-7068	7.47E-05	833	0.333333	0.01287	GAS1
5862.63	7.439146	-7068	7.46E-05	788	0.333333	0.012757	C18orf25
3360.208	8.2012	-7068	7.33E-05	547	0.333333	0.007613	FASN
2905.957	6.689161	-7068	7.33E-05	583	0.333333	0.008559	ACAT2
2660.216	5.478866	-7068	7.34E-05	612	0.333333	0.008456	SPAG5
1675.348	5.045135	-7068	7.29E-05	522	0.333333	0.007392	TIMELESS
5677.732	5.181423	-7068	7.47E-05	934	0.333333	0.012346	BUB1
6618.514	12.96647	-7068	7.29E-05	461	0.333333	0.006782	SORT1
2089.304	7.108019	-7068	7.27E-05	453	0.333333	0.005778	FLT4
2824.081	7.954292	-7068	7.31E-05	515	0.333333	0.007447	NUP210
1374.671	7.411342	-7068	7.19E-05	331	0.333333	0.00392	SERPINB13
4803.623	7.745313	-7068	7.39E-05	664	0.333333	0.009346	FCN2
2343.163	6.707408	-7068	7.23E-05	452	0.333333	0.005624	NDUFS5
3243.86	7.361129	-7068	7.27E-05	510	0.333333	0.006012	NDUFB8
10243.23	6.676767	-7068	7.64E-05	1123	0.333333	0.018068	TOM1L1
4823.34	8.921526	-7068	7.36E-05	605	0.333333	0.008339	ETHE1
2180.452	7.015755	-7068	7.30E-05	491	0.333333	0.007713	YAF2
4341.715	6.470566	-7068	7.40E-05	729	0.333333	0.01057	FST
5398.789	7.306149	-7068	7.43E-05	761	0.333333	0.011622	COQ2
2074.947	9.408554	-7068	7.25E-05	393	0.333333	0.005549	RBMS2
3401.397	7.668941	-7068	7.33E-05	571	0.333333	0.008178	RDH11
1047.441	8.512066	-7068	7.19E-05	299	0.333333	0.004301	MAST4
1978.482	6.704039	-7068	7.28E-05	467	0.333333	0.006237	CYP4F11
2192.659	6.480059	-7068	7.27E-05	488	0.333333	0.006371	FCGR3B
10340.94	6.66844	-7068	7.61E-05	1117	0.333333	0.016817	KYNU
3093.341	7.995169	-7068	7.32E-05	527	0.333333	0.00774	TNFAIP8
13230	6.968779	-7068	7.71E-05	1241	0.333333	0.019738	PARM1
5309.3	7.16095	-7068	7.43E-05	749	0.333333	0.010943	ADM
2831.59	6.779346	-7068	7.32E-05	543	0.333333	0.007338	KRT16
2839.497	7.215523	-7068	7.33E-05	556	0.333333	0.008302	GPSM2
5413.947	6.940026	-7068	7.44E-05	771	0.333333	0.011452	MT2A
2357.048	8.799445	-7068	7.27E-05	428	0.333333	0.005982	GPR4
2205.448	8.339754	-7068	7.28E-05	444	0.333333	0.006721	RABL3
4931.808	10.12241	-7068	7.32E-05	545	0.333333	0.007594	ASCL2
3046.373	8.689777	-7068	7.28E-05	481	0.333333	0.005914	GLS2
802.0966	8.462099	-7068	7.17E-05	259	0.333333	0.003765	SUSD5
1439.52	9.290767	-7068	7.18E-05	299	0.333333	0.003605	NOX1
2688.436	7.087836	-7068	7.31E-05	529	0.333333	0.008034	IL27RA
2902.491	7.652837	-7068	7.30E-05	519	0.333333	0.007368	TNFRSF11B
2517.075	7.892386	-7068	7.27E-05	468	0.333333	0.006686	PKP4

1516.391	8.424565	-7068	7.21E-05	349	0.333333	0.004647	TLR5
1629.846	5.352318	-7068	7.27E-05	490	0.333333	0.006807	MYH11
3919.982	7.495823	-7068	7.35E-05	613	0.333333	0.008682	TJP3
1256.445	8.983969	-7068	7.21E-05	299	0.333333	0.003913	PSCA
6766.111	8.106779	-7068	7.45E-05	777	0.333333	0.011024	ALDH4A1
2664.384	5.405412	-7068	7.32E-05	598	0.333333	0.008176	RAD51AP1
3965.985	7.014565	-7068	7.39E-05	671	0.333333	0.009857	FTH1
3705.511	7.312538	-7068	7.34E-05	618	0.333333	0.008421	ATF5
2259.588	8.636893	-7068	7.26E-05	419	0.333333	0.005293	PRKAR1B
3820.672	7.486078	-7068	7.35E-05	609	0.333333	0.008893	TACSTD2
3631.621	8.169457	-7068	7.33E-05	554	0.333333	0.007781	NLRP3
1509.971	9.782307	-7068	7.20E-05	310	0.333333	0.004003	GYPA
2562.11	6.668987	-7068	7.30E-05	538	0.333333	0.007455	IGF2
3161.336	7.290193	-7068	7.33E-05	575	0.333333	0.008302	ME1
6253.004	7.521726	-7068	7.41E-05	746	0.333333	0.009376	S100A9
1936.64	8.46352	-7068	7.24E-05	386	0.333333	0.005199	MINK1
2715.294	9.315399	-7068	7.26E-05	429	0.333333	0.005393	PELP1
3472.014	7.33152	-7068	7.34E-05	598	0.333333	0.008703	ANGPT2
2952.908	7.461285	-7068	7.33E-05	540	0.333333	0.008479	STON1
2015.65	7.062488	-7068	7.27E-05	458	0.333333	0.006783	MED20
4400.432	8.243708	-7068	7.36E-05	621	0.333333	0.008843	HSDL2
3507.644	6.513865	-7068	7.32E-05	606	0.333333	0.007156	ACAA1
2488.543	6.340546	-7068	7.30E-05	528	0.333333	0.007039	ATP5H
3458.917	6.167649	-7068	7.36E-05	648	0.333333	0.009001	MT1H
4559.053	6.785776	-7068	7.41E-05	723	0.333333	0.010298	GADD45B
3277.455	9.78953	-7068	7.28E-05	450	0.333333	0.005491	ACOT8
938.2778	6.979629	-7068	7.21E-05	325	0.333333	0.005321	VPS13B
3154.62	6.094708	-7068	7.32E-05	612	0.25	0.007774	HSPG2
2656.305	6.270016	-7068	7.32E-05	559	0.333333	0.007882	CD163
1930.349	8.885903	-7068	7.23E-05	374	0.333333	0.004802	MAP1A
2065.549	7.251576	-7068	7.27E-05	449	0.333333	0.006056	VNN1
1927.005	9.322665	-7068	7.23E-05	368	0.333333	0.004899	PAQR4
4715.196	7.546391	-7068	7.39E-05	687	0.333333	0.010265	EREG
4393.611	6.814213	-7068	7.40E-05	692	0.333333	0.010051	IFITM3
3142.428	7.702069	-7068	7.34E-05	555	0.333333	0.008374	IHH
1793.326	8.886077	-7068	7.23E-05	371	0.333333	0.005209	JMJD6
2778.769	8.075009	-7068	7.30E-05	492	0.333333	0.006707	RARG
4938.937	6.122209	-7068	7.38E-05	754	0.333333	0.009352	HP
2121.513	7.487455	-7068	7.28E-05	461	0.333333	0.007008	TRIL
6692.084	7.734952	-7068	7.46E-05	797	0.333333	0.011888	KRT7
2139.709	8.786042	-7068	7.23E-05	388	0.333333	0.005237	FUT3

2433.309	7.50494	-7068	7.26E-05	480	0.333333	0.00657	PSMC3IP
1383.134	8.62472	-7068	7.20E-05	328	0.333333	0.004707	CEP290
1063.553	6.18945	-7068	7.11E-05	269	0.25	0.002449	ZNF780A
5167.829	7.477074	-7068	7.42E-05	726	0.333333	0.011147	PLRG1
1133.983	12.43937	-7068	7.14E-05	215	0.333333	0.002544	B3GAT2
978.7173	9.361557	-7068	7.17E-05	251	0.333333	0.003129	SLC1A7
1445.126	5.783282	-7068	7.24E-05	430	0.333333	0.005604	GTSE1
4764.608	6.22657	-7068	7.43E-05	790	0.333333	0.011514	TUBB
2391.333	9.354793	-7068	7.26E-05	415	0.333333	0.006129	ABCB6
2244.059	8.684119	-7068	7.26E-05	418	0.333333	0.005688	MCAT
2623.859	8.321562	-7068	7.28E-05	468	0.333333	0.0068	FOXA2
1894.333	7.429795	-7068	7.27E-05	437	0.333333	0.006558	OCLN
5788.51	6.95526	-7068	7.46E-05	809	0.333333	0.012629	CERS6
2226.303	8.257307	-7068	7.28E-05	445	0.333333	0.006395	MTHFR
2546.389	8.745113	-7068	7.28E-05	449	0.333333	0.006554	FOXE1
1916.025	9.186837	-7068	7.23E-05	362	0.333333	0.004683	LTC4S
1628.447	9.646092	-7068	7.21E-05	334	0.25	0.004673	ATXN7L3B
1046.696	8.729176	-7068	7.18E-05	281	0.25	0.003857	TBX1
745.6736	7.106919	-7068	7.16E-05	258	0.333333	0.002987	NR2E3
1418.14	8.275843	-7068	7.22E-05	337	0.25	0.004372	MMP25
1292.797	7.40802	-7068	7.22E-05	353	0.333333	0.004943	OAS2
2577.587	8.343447	-7068	7.25E-05	434	0.333333	0.005519	KLK10
4573.592	7.21638	-7068	7.41E-05	697	0.333333	0.010992	EXPH5
2214.203	7.352859	-7068	7.30E-05	485	0.333333	0.007893	ENDOD1
4182.432	7.082741	-7068	7.39E-05	683	0.333333	0.010502	RECK
2078.101	8.640724	-7068	7.24E-05	391	0.333333	0.005231	ADORA3
3773.505	7.763886	-7068	7.35E-05	598	0.333333	0.00883	WISP1
16035.62	7.192329	-7068	7.76E-05	1315	0.333333	0.021274	IQCK
6007.102	7.566105	-7068	7.46E-05	789	0.333333	0.012594	COQ7
1389.627	9.243349	-7068	7.20E-05	312	0.333333	0.004421	MYCL
874.591	11.10447	-7068	7.14E-05	214	0.333333	0.002669	LYRM9
1689.581	7.74897	-7068	7.25E-05	394	0.333333	0.005459	IFNA1
1024.123	10.74867	-7068	7.16E-05	240	0.333333	0.003283	TMEM30B
3630.063	7.277123	-7068	7.37E-05	631	0.333333	0.00982	PAWR
3264.011	5.030088	-7068	7.39E-05	742	0.333333	0.01057	HSPD1
1871.055	12.08142	-7068	7.20E-05	297	0.333333	0.004345	SLC5A3
3206.413	7.7581	-7068	7.31E-05	530	0.333333	0.00729	HSPA6
2962.737	7.091947	-7068	7.31E-05	548	0.333333	0.007389	METTL7A
1952.519	8.622548	-7068	7.24E-05	387	0.333333	0.004997	TICAM1
5105.379	7.169754	-7068	7.41E-05	722	0.333333	0.010285	MAFF
3217.22	6.350582	-7068	7.36E-05	632	0.333333	0.009498	COL14A1

1031.101	6.192325	-7068	7.22E-05	363	0.333333	0.005878	TMEM41B
6028.662	7.281545	-7068	7.47E-05	806	0.333333	0.013108	SAMD4A
4508.986	6.693054	-7068	7.43E-05	745	0.333333	0.011876	SIK1
3410.708	7.096417	-7068	7.37E-05	617	0.333333	0.009578	CH25H
2345.741	7.080856	-7068	7.29E-05	497	0.333333	0.00709	APOL1
2610.207	7.8994	-7068	7.31E-05	505	0.333333	0.007726	STC2
1364.012	6.893877	-7068	7.23E-05	386	0.333333	0.005521	SVEP1
643.2079	8.486972	-7068	7.16E-05	241	0.333333	0.003715	SENP5
3377.967	4.617759	-7068	7.38E-05	758	0.333333	0.009588	BIRC5
5746.214	8.007757	-7068	7.41E-05	724	0.333333	0.010812	KLF11
1377.462	5.531158	-7068	7.23E-05	427	0.333333	0.00563	CCL3
3001.419	5.622566	-7068	7.33E-05	621	0.333333	0.007466	APOC1
1891.944	10.18808	-7068	7.21E-05	324	0.333333	0.003806	SOX15
2028.152	7.566671	-7068	7.27E-05	447	0.333333	0.006694	REEP5
1566.937	7.618911	-7068	7.24E-05	391	0.333333	0.006059	YIPF6
4786.981	7.533374	-7068	7.41E-05	691	0.333333	0.010316	CD24
3147.658	5.946884	-7068	7.37E-05	665	0.333333	0.010224	RHEB
1375.876	14.63023	-7068	7.14E-05	201	0.333333	0.0027	USP6
1942.882	6.745843	-7068	7.28E-05	474	0.333333	0.007013	SQLE
1817.458	8.156744	-7068	7.25E-05	396	0.333333	0.005381	RNF187
1664.584	9.616015	-7068	7.21E-05	328	0.333333	0.00416	SNCG
3279.52	8.032712	-7068	7.34E-05	562	0.333333	0.008314	APOBEC3B
6089.205	7.493454	-7068	7.46E-05	788	0.333333	0.011983	G0S2
1726.313	8.2611	-7068	7.22E-05	369	0.333333	0.004863	WISP2
8111.288	6.633943	-7068	7.52E-05	953	0.333333	0.013816	GPRC5A
5488.221	7.636004	-7068	7.43E-05	749	0.333333	0.011614	RNFT2
5531.811	6.478011	-7068	7.46E-05	814	0.333333	0.011639	PLAU
2248.294	7.587149	-7068	7.29E-05	476	0.333333	0.00714	DLG3
1807.622	7.827987	-7068	7.25E-05	412	0.333333	0.006016	SOX13
2557.793	8.022048	-7068	7.28E-05	464	0.333333	0.006418	COL10A1
1109.073	9.589365	-7068	7.18E-05	262	0.333333	0.003507	SIPA1L3
2131.862	6.550197	-7068	7.29E-05	502	0.25	0.007254	PLTP
2688.934	7.659103	-7068	7.32E-05	515	0.333333	0.007931	SAMHD1
2462.598	5.30423	-7068	7.33E-05	606	0.333333	0.009013	COL5A2
2818.349	9.249466	-7068	7.27E-05	440	0.333333	0.006407	TEX30
1889.354	7.22794	-7068	7.25E-05	430	0.333333	0.005753	CXCL11
1422.519	10.19432	-7068	7.20E-05	306	0.333333	0.004213	IAH1
3364.055	8.196794	-7068	7.33E-05	550	0.333333	0.008297	F2RL1
1769.874	5.863179	-7068	7.26E-05	453	0.333333	0.005768	IGSF6
3143.393	6.551691	-7068	7.35E-05	598	0.333333	0.008579	FCGR2A
2759.99	6.922286	-7068	7.32E-05	548	0.333333	0.008444	PXDN

2169.581	8.613511	-7068	7.26E-05	423	0.333333	0.006086	SLC46A3
3772.246	5.549152	-7068	7.37E-05	733	0.333333	0.00947	WDHD1
3151.257	6.494543	-7068	7.32E-05	603	0.333333	0.007824	CDC25C
3723.59	9.391628	-7068	7.32E-05	520	0.333333	0.007981	OPRM1
2052.455	6.38922	-7068	7.28E-05	495	0.333333	0.006838	BMP1
1938.143	6.094012	-7068	7.28E-05	489	0.333333	0.006684	CCL8
2118.431	7.194108	-7068	7.28E-05	472	0.333333	0.00688	IFIT2
1630.563	8.071866	-7068	7.23E-05	372	0.333333	0.005048	DOK4
1061.756	8.803999	-7068	7.17E-05	275	0.333333	0.003383	FUT6
1218.306	9.019557	-7068	7.20E-05	309	0.333333	0.004505	MOCS1
2132.946	7.276204	-7068	7.28E-05	473	0.333333	0.007073	ELOVL5
1787.386	7.808937	-7068	7.26E-05	414	0.333333	0.006344	PDE1C
9029.184	8.031327	-7068	7.53E-05	912	0.333333	0.013935	SEMA3D
978.5977	10.67979	-7068	7.16E-05	231	0.333333	0.002807	ZSWIM8
807.6921	7.127558	-7068	7.19E-05	298	0.333333	0.004594	FEZ2
4509.306	6.586556	-7068	7.42E-05	738	0.333333	0.011418	MT1E
1027.219	7.718594	-7068	7.18E-05	293	0.333333	0.003813	ACSM2A
1617.083	7.268393	-7068	7.24E-05	404	0.25	0.005916	ABCC9
1859.353	6.697027	-7068	7.27E-05	452	0.25	0.006979	SULF1
2544.343	7.259865	-7068	7.29E-05	489	0.25	0.007086	ANGPTL2
1825.215	8.288773	-7068	7.23E-05	381	0.333333	0.005205	HOPX
3056.972	6.200967	-7068	7.31E-05	590	0.333333	0.007434	CFHR3
2034.122	6.40669	-7068	7.28E-05	485	0.333333	0.006789	EFEMP1
1973.198	5.038578	-7068	7.30E-05	555	0.333333	0.007913	LAMA2
3801.164	22.10573	-7068	7.17E-05	296	0.25	0.004044	UGT2B11
18158.25	16.18881	-7068	7.49E-05	875	0.333333	0.013662	UGT2B7
2205.052	6.361298	-7068	7.28E-05	505	0.333333	0.006813	SAA1
1389.193	6.36371	-7068	7.21E-05	392	0.333333	0.005013	SAA4
326.9074	8.570343	-7068	7.10E-05	151	0.333333	0.001863	PCDHGA12
635.514	8.292693	-7068	7.15E-05	233	0.333333	0.003393	KNOP1
1387.678	7.02676	-7068	7.23E-05	376	0.333333	0.005349	MT1F
184.2382	7.21481	-7068	7.06E-05	130	0.333333	0.001676	MT1A
361.0604	6.76535	-7068	7.11E-05	194	0.25	0.002691	MT1B

Supplementary Table S5: Topological network analysis related to anti-VEGF resistance							
Betweenness unDir	Centroid unDir	Closeness unDir	Degree unDir	Eccentricity unDir	Radiality unDir	Stress unDir	gene name
11515.9	-4354	1.67E-04	3225	0.333333	3.620436	4584184	BMP4
10219.47	-4354	1.65E-04	3096	0.333333	3.609185	4319870	FMOD
12475.34	-4354	1.65E-04	2805	0.333333	3.60597	4575472	TMSB4X
10656.26	-4354	1.64E-04	3032	0.333333	3.598852	4259758	CDH3
9338.793	-4354	1.61E-04	2978	0.333333	3.575201	3888830	TCF7L1
9901.275	-4354	1.61E-04	3042	0.333333	3.574053	3881566	DRD5
12107.06	-4354	1.61E-04	2983	0.333333	3.573364	4120140	UBB
8739.395	-4354	1.60E-04	2577	0.333333	3.560276	3680034	ITPKC
9515.262	-4354	1.59E-04	2720	0.333333	3.558668	3732452	ADCY6
10149.08	-4354	1.59E-04	2541	0.333333	3.550402	3963814	PLA2G2D
7981.938	-4354	1.58E-04	2690	0.333333	3.549024	3436928	FOXF2
9241.452	-4354	1.58E-04	2538	0.333333	3.546498	3587744	CLDN10
10027.91	-4354	1.58E-04	3399	0.333333	3.543972	3624774	UBC
9238.92	-4354	1.58E-04	2751	0.333333	3.543972	3520770	TUBA1A
8406.069	-4354	1.58E-04	2904	0.333333	3.542824	3455684	PPARG
8738.279	-4354	1.58E-04	2596	0.333333	3.541447	3428588	EDAR
9502.391	-4354	1.58E-04	2788	0.333333	3.540987	3451088	CLIC1
8838.422	-4354	1.57E-04	2644	0.333333	3.538462	3443884	PLD2
8300.262	-4354	1.57E-04	2657	0.333333	3.538232	3358972	ATP12A
7830.647	-4354	1.57E-04	2399	0.333333	3.53318	3275670	TINAGL1
8046.941	-4354	1.57E-04	2748	0.333333	3.532721	3335690	PIM1
7432.052	-4354	1.57E-04	2770	0.333333	3.532032	3221672	VEGFC
7217.785	-4354	1.57E-04	2646	0.333333	3.532032	3215426	CD34
8205.219	-4354	1.56E-04	2917	0.333333	3.531573	3332962	NFKB1
8512.423	-4354	1.56E-04	2386	0.333333	3.529277	3359134	SH2B2
7649.614	-4354	1.56E-04	2465	0.333333	3.526521	3198124	CD63
8441.225	-4354	1.56E-04	2859	0.333333	3.525144	3267290	PRL
7701.616	-4354	1.56E-04	2507	0.333333	3.523307	3201004	TRIP10
7164.489	-4354	1.56E-04	2475	0.333333	3.523077	3168558	SDC4
7523.204	-4354	1.56E-04	2556	0.333333	3.522618	3165880	GNA15
7676.734	-4354	1.55E-04	2563	0.333333	3.522158	3148590	ITGA10
7198.178	-4354	1.55E-04	2446	0.333333	3.519173	3095450	CYP27A1
7712.566	-4354	1.55E-04	2395	0.333333	3.519173	3072546	GSTM5
7977.192	-4354	1.55E-04	2766	0.333333	3.517336	3103646	GRM4
7633.816	-4354	1.55E-04	2555	0.333333	3.517336	3154538	FTL
8406.12	-4354	1.55E-04	2925	0.333333	3.517107	3195668	MAPK3
7297.843	-4354	1.55E-04	2722	0.333333	3.517107	3110146	GAB2
7027.675	-4354	1.55E-04	2691	0.333333	3.51527	3068386	CEBPA
7239.319	-4354	1.55E-04	2305	0.333333	3.51504	3008196	PDE6G

6947.126	-4354	1.55E-04	2555	0.333333	3.513433	3001230	PYGL
8073.24	-4354	1.55E-04	2449	0.333333	3.512974	3071978	POU4F2
8163.97	-4354	1.55E-04	2349	0.333333	3.512974	3055882	IFNA16
7704.215	-4354	1.54E-04	2647	0.333333	3.512055	3025588	OCA2
6830.364	-4354	1.54E-04	2342	0.333333	3.511137	2985836	TNFRSF8
7793.448	-4354	1.54E-04	2412	0.333333	3.510907	3034778	CD81
7053.071	-4354	1.54E-04	2626	0.333333	3.509529	2947482	AGT
8033.616	-4354	1.54E-04	2550	0.333333	3.5093	3067982	RPS6KA1
7437.419	-4354	1.54E-04	2318	0.333333	3.50884	3001552	HAMP
7143.593	-4354	1.54E-04	2495	0.333333	3.508381	2998732	DPYSL2
7147.614	-4354	1.54E-04	2467	0.333333	3.508152	3001406	TSPAN7
6812.172	-4354	1.54E-04	2539	0.333333	3.507692	2958538	ITGA5
8223.808	-4354	1.54E-04	2402	0.333333	3.507463	3132444	EPCAM
7184.282	-4354	1.54E-04	2621	0.333333	3.506774	2986576	AMOTL2
7168.511	-4354	1.54E-04	2575	0.333333	3.506544	3016100	ITGA3
7017.272	-4354	1.54E-04	2741	0.333333	3.506315	2960572	ADRB2
7646.91	-4354	1.54E-04	2730	0.333333	3.506315	3007464	RYR3
6829.611	-4354	1.54E-04	2259	0.333333	3.506085	2912102	S100A11
7114.442	-4354	1.54E-04	2385	0.333333	3.505855	2990690	EPHB6
8109.486	-4354	1.54E-04	2376	0.333333	3.504707	2941066	WNT11
7640.169	-4354	1.54E-04	2286	0.333333	3.50333	2969704	PLCD1
7047.815	-4354	1.53E-04	2419	0.333333	3.50287	2882652	SLC2A9
6562.308	-4354	1.53E-04	2393	0.333333	3.50287	2872292	MGLL
7516.761	-4354	1.53E-04	2604	0.333333	3.502411	2999304	NCK2
6995.259	-4354	1.53E-04	2487	0.333333	3.502411	2920258	HCLS1
7248.526	-4354	1.53E-04	2322	0.333333	3.502411	2950214	SMO
6933.292	-4354	1.53E-04	2532	0.333333	3.501722	2903270	CTSK
7362.822	-4354	1.53E-04	2330	0.333333	3.501722	2987960	SLC9A3R1
6871.089	-4354	1.53E-04	2571	0.333333	3.501033	2862668	ANXA5
7008.023	-4354	1.53E-04	2388	0.333333	3.500574	2928346	FBP1
6611.671	-4354	1.53E-04	2759	0.333333	3.499656	2859032	CDKN1A
6644.647	-4354	1.53E-04	2526	0.333333	3.499656	2859978	MYH9
8073.109	-4354	1.53E-04	2712	0.333333	3.499426	2935880	MUC1
6682.139	-4354	1.53E-04	2628	0.333333	3.498278	2824622	VCAM1
7401.388	-4354	1.53E-04	2630	0.333333	3.497589	2922526	CTSO
7124.514	-4354	1.53E-04	2412	0.333333	3.497359	2903774	MST1R
7530.919	-4354	1.53E-04	2388	0.333333	3.495982	2949730	RAB5B
6416.793	-4354	1.53E-04	2576	0.333333	3.495522	2821526	STAT5A
7951.495	-4354	1.53E-04	2808	0.333333	3.494374	2950048	PRKCQ
6222.498	-4354	1.53E-04	2278	0.333333	3.494374	2761560	DAAM2
6753.006	-4354	1.53E-04	2669	0.333333	3.493685	2825750	GNAI2

7058.452	-4354	1.53E-04	2420	0.333333	3.493226	2860296	ASS1
6907.222	-4354	1.52E-04	2372	0.333333	3.492537	2853156	JUP
6654.283	-4354	1.52E-04	2208	0.333333	3.492537	2756946	NPPA
7098.195	-4354	1.52E-04	2315	0.333333	3.492078	2849276	S1PR4
7314.629	-4354	1.52E-04	2304	0.333333	3.492078	2887494	MDK
6335.078	-4354	1.52E-04	2399	0.333333	3.491848	2772322	LAMB3
7553.685	-4354	1.52E-04	2486	0.333333	3.491389	2888716	CACNG3
6802.261	-4354	1.52E-04	2487	0.333333	3.49093	2770212	PRKCD
6885.069	-4354	1.52E-04	2326	0.333333	3.4907	2724014	CER1
6812.06	-4354	1.52E-04	2206	0.333333	3.4907	2801574	NUPR1
6295.689	-4354	1.52E-04	2396	0.333333	3.490471	2718346	IFNGR2
7587.883	-4354	1.52E-04	2699	0.333333	3.490241	2863048	HDAC1
7042.02	-4354	1.52E-04	2626	0.333333	3.490241	2810612	GHR
6378.446	-4354	1.52E-04	2409	0.333333	3.489552	2746312	S1PR1
6939.808	-4354	1.52E-04	2446	0.333333	3.489093	2814474	IL2RG
6506.717	-4354	1.52E-04	2309	0.333333	3.488175	2723340	COX7A1
7100.696	-4354	1.52E-04	2630	0.333333	3.487945	2810736	EGLN3
6111.27	-4354	1.52E-04	2242	0.333333	3.487945	2716720	EFNB1
9568.737	-4354	1.52E-04	2503	0.333333	3.487715	3497026	UVRAG
6030.262	-4354	1.52E-04	2536	0.333333	3.487026	2680350	F3
6470.696	-4354	1.52E-04	2281	0.333333	3.487026	2748416	FOSL1
7296.213	-4354	1.52E-04	2585	0.333333	3.486797	2793308	DPF3
6444.257	-4354	1.52E-04	2174	0.333333	3.486567	2687322	FBP2
6274.811	-4354	1.52E-04	2390	0.333333	3.486108	2699008	RPRM
5968.001	-4354	1.52E-04	2313	0.333333	3.485649	2660348	TCN2
6148.049	-4354	1.52E-04	2300	0.333333	3.485649	2676750	KLK6
7814.423	-4354	1.52E-04	2591	0.333333	3.48496	3193632	TLR4
6513.498	-4354	1.52E-04	2451	0.333333	3.48496	2656592	AVPR1B
6783.592	-4354	1.52E-04	2626	0.333333	3.48473	2731124	ACVR1
6973.639	-4354	1.52E-04	2487	0.333333	3.48473	2713962	ISL1
7165.387	-4354	1.52E-04	2645	0.333333	3.484501	2775032	CTNNA2
7319.46	-4354	1.52E-04	2567	0.333333	3.484501	2812884	PRKCZ
6677.991	-4354	1.52E-04	2365	0.333333	3.484501	2714476	CDH17
7241.925	-4354	1.52E-04	2583	0.333333	3.484271	2782826	PINK1
6204.59	-4354	1.52E-04	2766	0.333333	3.484041	2645334	BMP2
5811.944	-4354	1.52E-04	2463	0.333333	3.483582	2595714	TNFRSF1A
7193.379	-4354	1.52E-04	2412	0.333333	3.483582	2811116	PLCB3
6705.112	-4354	1.52E-04	2289	0.333333	3.483352	2699808	CNTFR
6843.285	-4354	1.52E-04	2183	0.333333	3.483352	2673176	IFNA4
5949.132	-4354	1.51E-04	2209	0.333333	3.482664	2588652	GGT5
5855.002	-4354	1.51E-04	2220	0.333333	3.482204	2607718	GP1BA

6855.473	-4354	1.51E-04	2577	0.333333	3.481975	2773168	SKAP1
5947.62	-4354	1.51E-04	2356	0.333333	3.481975	2596340	RASSF2
8405.252	-4354	1.51E-04	2534	0.333333	3.481745	2783636	GSTP1
11086.15	-4354	1.51E-04	2349	0.333333	3.481515	4324896	ENO2
6438.383	-4354	1.51E-04	2674	0.333333	3.481286	2675602	PTPRM
6916.774	-4354	1.51E-04	2226	0.333333	3.481286	2703518	COX6A2
6603.512	-4354	1.51E-04	2232	0.333333	3.480597	2710450	HLA-A
6709.913	-4354	1.51E-04	2698	0.333333	3.480367	2724592	ABL1
5575.243	-4354	1.51E-04	2193	0.333333	3.480367	2543568	C1R
5924.511	-4354	1.51E-04	2385	0.333333	3.480138	2600534	INHBB
5828.161	-4354	1.51E-04	2373	0.333333	3.480138	2558096	C3
5730.137	-4354	1.51E-04	2338	0.333333	3.479679	2547444	SERPING1
6145.915	-4354	1.51E-04	2285	0.333333	3.479679	2609076	SERPINA5
5833.518	-4354	1.51E-04	2463	0.333333	3.479219	2609074	PDGFRB
6865.294	-4354	1.51E-04	2305	0.333333	3.479219	2694412	OGDHL
6896.287	-4354	1.51E-04	2439	0.333333	3.47899	2719126	NR3C2
6252.726	-4354	1.51E-04	2261	0.333333	3.47899	2609864	SERPINA3
6183.098	-4354	1.51E-04	2210	0.333333	3.47899	2582038	HSPB2
6624.539	-4354	1.51E-04	2320	0.333333	3.47876	2607452	BMP15
6789.075	-4354	1.51E-04	2580	0.333333	3.478071	2639610	C8A
6353.056	-4354	1.51E-04	2286	0.333333	3.477842	2602214	CA9
5712.603	-4354	1.51E-04	2320	0.333333	3.477612	2552750	IL4R
6025.562	-4354	1.51E-04	2239	0.333333	3.477612	2567390	ARPC1B
5998.723	-4354	1.51E-04	2289	0.333333	3.477153	2586024	ALDH2
5655.426	-4354	1.51E-04	2346	0.333333	3.476923	2555530	TGFBI
6040.376	-4354	1.51E-04	2414	0.333333	3.476464	2564412	RELN
5789.684	-4354	1.51E-04	2267	0.333333	3.476464	2559458	FLNC
6239.062	-4354	1.51E-04	2365	0.333333	3.476234	2621262	CXCR1
7120.879	-4354	1.51E-04	2312	0.333333	3.475545	2595944	SDCBP
6271.559	-4354	1.51E-04	2226	0.333333	3.475316	2571298	LTA
5546.761	-4354	1.51E-04	2397	0.333333	3.475086	2519034	PLPP3
6401.486	-4354	1.51E-04	2119	0.333333	3.475086	2563576	CEND1
5782.797	-4354	1.51E-04	2181	0.333333	3.474856	2543516	CTSE
6039.734	-4354	1.51E-04	2195	0.333333	3.474627	2544164	CD1A
6006.239	-4354	1.51E-04	2542	0.333333	3.474168	2552328	KCTD12
5606.393	-4354	1.51E-04	2179	0.333333	3.473708	2459374	AMHR2
6064.587	-4354	1.51E-04	2346	0.333333	3.473479	2586744	EPHA2
5847.403	-4354	1.51E-04	2167	0.333333	3.473479	2532662	FKBP10
6754.189	-4354	1.51E-04	2923	0.333333	3.473249	2679420	MYC
6045.947	-4354	1.51E-04	2107	0.333333	3.473249	2565420	SULT2B1
6457.838	-4354	1.50E-04	2438	0.333333	3.47302	2596412	PTPN6

5771.974	-4354	1.50E-04	2412	0.333333	3.47302	2544970	PIK3R5
5787.13	-4354	1.50E-04	2276	0.333333	3.47302	2515050	RGL1
6407.033	-4354	1.50E-04	2198	0.333333	3.47279	2559818	MLXIPL
5816.438	-4354	1.50E-04	2369	0.333333	3.47256	2535804	LOXL1
6605.176	-4354	1.50E-04	2332	0.333333	3.472331	2612164	SPTA1
5711.981	-4354	1.50E-04	2321	0.333333	3.472331	2522144	NDRG1
6379.308	-4354	1.50E-04	2347	0.333333	3.471642	2610180	UNC13B
6016.963	-4354	1.50E-04	2280	0.333333	3.471412	2527084	RHOG
6136.328	-4354	1.50E-04	2210	0.333333	3.471183	2477184	HAND1
5972.438	-4354	1.50E-04	2459	0.333333	3.470953	2531318	CSF3R
6690.207	-4354	1.50E-04	2424	0.333333	3.470494	2632614	TERF2IP
6043.392	-4354	1.50E-04	2228	0.333333	3.470494	2474702	NR0B2
5709.605	-4354	1.50E-04	2121	0.333333	3.470264	2460922	MADCAM1
5623.161	-4354	1.50E-04	2339	0.333333	3.469805	2465248	CSF1R
5955.283	-4354	1.50E-04	2129	0.333333	3.469805	2504822	SERPINF1
6588.38	-4354	1.50E-04	2433	0.333333	3.469346	2553120	IL10RB
6430.08	-4354	1.50E-04	2443	0.333333	3.469116	2523390	MAP2K1
6949.446	-4354	1.50E-04	2412	0.333333	3.468886	2634152	NTRK1
6069.71	-4354	1.50E-04	2157	0.333333	3.468886	2468852	NCAN
5748.887	-4354	1.50E-04	2223	0.333333	3.468657	2509974	CRYM
5930.131	-4354	1.50E-04	2384	0.333333	3.468427	2536390	VLDLR
7090.596	-4354	1.50E-04	2475	0.333333	3.468197	2625274	GAPDH
5954.234	-4354	1.50E-04	2263	0.333333	3.467968	2484778	ATP6V1B2
5850.236	-4354	1.50E-04	2256	0.333333	3.467738	2470178	REM1
5829.406	-4354	1.50E-04	2180	0.333333	3.467738	2505872	LILRA4
6346.104	-4354	1.50E-04	2108	0.333333	3.467738	2477066	IFNA10
6421.64	-4354	1.50E-04	2423	0.333333	3.467509	2542312	PBX3
6083.904	-4354	1.50E-04	2335	0.333333	3.467509	2540428	ABCG2
5629.377	-4354	1.50E-04	2147	0.333333	3.467509	2439778	PLIN1
5700.384	-4354	1.50E-04	2362	0.333333	3.467049	2465112	MYD88
5739.239	-4354	1.50E-04	2246	0.333333	3.467049	2500470	RAB13
6903.435	-4354	1.50E-04	2241	0.333333	3.467049	2840158	PLA2G2A
5461.315	-4354	1.50E-04	2561	0.333333	3.46682	2464834	CAV1
5770.896	-4354	1.50E-04	2224	0.333333	3.46682	2490558	INPP1
7772.632	-4354	1.50E-04	2076	0.333333	3.46682	2660308	BIRC7
5887.365	-4354	1.50E-04	2058	0.333333	3.466361	2517878	DTX4
6024.802	-4354	1.50E-04	2212	0.333333	3.466131	2494830	SLC22A4
5904.269	-4354	1.50E-04	2314	0.333333	3.465901	2478694	C4BPB
5898.308	-4354	1.50E-04	2262	0.333333	3.465901	2507826	STAT4
6377.759	-4354	1.50E-04	2312	0.333333	3.465672	2547788	AP1B1
5163.854	-4354	1.50E-04	2177	0.333333	3.465672	2399756	SELP

6152.74	-4354	1.50E-04	2424	0.333333	3.465442	2501778	PTPRZ1
5192.168	-4354	1.50E-04	2055	0.333333	3.465442	2390256	MFAP4
5987.848	-4354	1.50E-04	2380	0.333333	3.464753	2485014	PRKAR2B
5653.584	-4354	1.50E-04	2383	0.333333	3.464294	2422020	HCK
6117.158	-4354	1.50E-04	2179	0.333333	3.464294	2521236	ACADS
5402.987	-4354	1.50E-04	2377	0.333333	3.463835	2388030	NGFR
6132.181	-4354	1.50E-04	2318	0.333333	3.463146	2464856	GCH1
6021.397	-4354	1.50E-04	2138	0.333333	3.463146	2447100	GP9
11672.08	-4354	1.49E-04	2934	0.333333	3.462916	4357904	EGFR
6533.299	-4354	1.49E-04	2196	0.333333	3.462687	2534280	TNFRSF14
6268.366	-4354	1.49E-04	2363	0.333333	3.462227	2458154	ILK
5984.217	-4354	1.49E-04	2356	0.333333	3.462227	2450372	ADORA2B
5837.333	-4354	1.49E-04	2062	0.333333	3.462227	2431142	TFF1
6021.137	-4354	1.49E-04	2370	0.333333	3.461998	2423516	GNG3
6432.338	-4354	1.49E-04	2078	0.333333	3.461768	2540804	ADIPOR1
5385.509	-4354	1.49E-04	2236	0.333333	3.461309	2381282	CRYAB
5699.134	-4354	1.49E-04	2154	0.333333	3.461079	2416390	ALDH1A1
5364.132	-4354	1.49E-04	2249	0.333333	3.46039	2350796	TNFRSF1B
7056.643	-4354	1.49E-04	2184	0.333333	3.46039	2830414	MMP7
5605.589	-4354	1.49E-04	2119	0.333333	3.46039	2372752	IL22RA1
5851.821	-4354	1.49E-04	2116	0.333333	3.46039	2468864	CD70
5934.443	-4354	1.49E-04	2226	0.333333	3.460161	2504820	FRAT1
5828.532	-4354	1.49E-04	2123	0.333333	3.460161	2400002	IFNA14
5564.142	-4354	1.49E-04	2485	0.333333	3.459931	2384352	AHR
5639.018	-4354	1.49E-04	2061	0.333333	3.459701	2354770	TM4SF5
6321.497	-4354	1.49E-04	2539	0.333333	3.459472	2447306	IQGAP2
5800.702	-4354	1.49E-04	2479	0.333333	3.459472	2436484	KIT
5989.608	-4354	1.49E-04	2427	0.333333	3.459472	2462952	ACTN4
6008.859	-4354	1.49E-04	2188	0.333333	3.459242	2448260	IMPA2
5645.264	-4354	1.49E-04	2123	0.333333	3.459013	2352222	TUBB6
6641.196	-4354	1.49E-04	2229	0.333333	3.458783	2521528	PFN2
5541.478	-4354	1.49E-04	2184	0.333333	3.458553	2381370	VASP
5998.754	-4354	1.49E-04	2182	0.333333	3.458553	2431700	ARHGEF6
5363.124	-4354	1.49E-04	2138	0.333333	3.458553	2339988	PDE1B
5922.168	-4354	1.49E-04	2449	0.333333	3.458324	2446246	ABLIM1
5200.439	-4354	1.49E-04	2260	0.333333	3.458324	2338444	CEBPB
5921.601	-4354	1.49E-04	2351	0.333333	3.458094	2456060	STXBP1
5690.345	-4354	1.49E-04	2187	0.333333	3.458094	2405410	CA2
6061.406	-4354	1.49E-04	2343	0.333333	3.457865	2369648	NGF
5539.532	-4354	1.49E-04	2155	0.333333	3.457635	2377426	CNTNAP1
6052.148	-4354	1.49E-04	2629	0.333333	3.457405	2445048	PRKCE

5582.014	-4354	1.49E-04	2218	0.333333	3.457405	2350372	CSF2RB
5815.06	-4354	1.49E-04	2155	0.333333	3.457405	2407334	LAMB2
6271.427	-4354	1.49E-04	2958	0.333333	3.457176	2453548	PIK3R1
6772.728	-4354	1.49E-04	2484	0.333333	3.457176	2465864	SNCA
6318.013	-4354	1.49E-04	2250	0.333333	3.457176	2347722	HPSE2
6045.365	-4354	1.49E-04	2498	0.333333	3.456716	2391090	ID2
6201.323	-4354	1.49E-04	2308	0.333333	3.456487	2477538	CD3E
5440.763	-4354	1.49E-04	2167	0.333333	3.456257	2391016	NFE2
6950.656	-4354	1.49E-04	2408	0.333333	3.456028	2469920	HADHB
6095.119	-4354	1.49E-04	2302	0.333333	3.456028	2399228	SGCG
5591.664	-4354	1.49E-04	2273	0.333333	3.456028	2347354	HSD17B2
5654.064	-4354	1.49E-04	2042	0.333333	3.455568	2345256	G6PC2
5865.667	-4354	1.49E-04	2108	0.333333	3.455339	2403694	FGD1
5798.7	-4354	1.49E-04	2355	0.333333	3.455109	2333044	TACR2
4689.095	-4354	1.49E-04	2293	0.333333	3.45442	2196210	APLNR
6026.866	-4354	1.49E-04	2167	0.333333	3.45442	2379818	TUBA4A
5709.856	-4354	1.49E-04	2397	0.333333	3.454191	2387516	ROBO1
6341.471	-4354	1.49E-04	2292	0.333333	3.454191	2467312	CTNNBIP1
5496.154	-4354	1.49E-04	2496	0.333333	3.453961	2375972	IRS2
6078.934	-4354	1.49E-04	2355	0.333333	3.453961	2347568	GJA1
5208.954	-4354	1.49E-04	2269	0.333333	3.453961	2326848	FES
6020.294	-4354	1.49E-04	2155	0.333333	3.453961	2345564	NPC2
4948.9	-4354	1.49E-04	2127	0.333333	3.453961	2256558	HSD11B1
5895.044	-4354	1.49E-04	2373	0.333333	3.453731	2368292	LIPC
5436.153	-4354	1.49E-04	2317	0.333333	3.453731	2282750	FSHR
5750.723	-4354	1.49E-04	2224	0.333333	3.453731	2310712	GRPR
5352.331	-4354	1.49E-04	2113	0.333333	3.453731	2272864	CSF3
5559.986	-4354	1.49E-04	2060	0.333333	3.453731	2353262	RIN1
6201.85	-4354	1.49E-04	2094	0.333333	3.453272	2367648	HYAL2
5390.125	-4354	1.49E-04	2034	0.333333	3.453272	2283906	KLK11
6771.834	-4354	1.49E-04	2296	0.333333	3.453042	2401622	GBE1
4929.404	-4354	1.49E-04	2259	0.333333	3.453042	2259022	SLCO2A1
6004.864	-4354	1.49E-04	2257	0.333333	3.453042	2411676	LAMA5
5303.859	-4354	1.49E-04	2178	0.333333	3.453042	2335490	ELMO1
5800.818	-4354	1.49E-04	2156	0.333333	3.453042	2406160	SLC7A5
5474.036	-4354	1.49E-04	2163	0.333333	3.452583	2369424	P2RY2
5671.145	-4354	1.49E-04	2162	0.333333	3.452583	2362208	ITPKA
6259.541	-4354	1.49E-04	2143	0.333333	3.452583	2438716	RNF43
6093.943	-4354	1.48E-04	2260	0.333333	3.452354	2437046	PHGDH
5787.872	-4354	1.48E-04	2159	0.333333	3.452354	2347184	TALDO1
5279.011	-4354	1.48E-04	2085	0.333333	3.452354	2307536	LIPG

6268.659	-4354	1.48E-04	2105	0.333333	3.452124	2360558	LAPTM4A
5989.165	-4354	1.48E-04	2569	0.333333	3.451894	2368128	DLG2
5284.586	-4354	1.48E-04	2067	0.333333	3.451894	2276226	KISS1
5869.997	-4354	1.48E-04	2288	0.333333	3.451665	2358948	BAAT
5556.393	-4354	1.48E-04	2245	0.333333	3.451665	2334362	SPI1
5532.132	-4354	1.48E-04	2140	0.333333	3.451665	2330612	CYP4F2
5475.813	-4354	1.48E-04	2081	0.333333	3.451665	2344662	S100A2
5697.112	-4354	1.48E-04	2143	0.333333	3.451435	2346808	LDHA
5412.155	-4354	1.48E-04	2457	0.333333	3.451206	2318060	GLI3
5192.454	-4354	1.48E-04	2263	0.333333	3.450976	2298850	TNFRSF21
6004.651	-4354	1.48E-04	2518	0.333333	3.450746	2354890	BIRC3
7020.97	-4354	1.48E-04	2472	0.333333	3.450746	2463738	SOD1
6327.158	-4354	1.48E-04	2290	0.333333	3.450746	2317184	SLC2A2
7247.366	-4354	1.48E-04	2976	0.333333	3.450057	2453812	YWHAE
4940.525	-4354	1.48E-04	2164	0.333333	3.450057	2227800	GPX3
6021.162	-4354	1.48E-04	2145	0.333333	3.450057	2396784	CDC25B
5421.653	-4354	1.48E-04	2194	0.333333	3.449828	2282296	C4BPA
5331.759	-4354	1.48E-04	2301	0.333333	3.449598	2276060	HIF1A
7763.966	-4354	1.48E-04	2408	0.333333	3.449369	2451456	ATP5F1B
5883.988	-4354	1.48E-04	2214	0.333333	3.449369	2348690	MGST2
5147.786	-4354	1.48E-04	2176	0.333333	3.449369	2270794	SNAI2
5659.349	-4354	1.48E-04	2105	0.333333	3.449369	2293666	ATP6V0D1
5558.236	-4354	1.48E-04	2272	0.333333	3.449139	2305404	ARRB2
6394.372	-4354	1.48E-04	2485	0.333333	3.448909	2386300	DAPK1
5635.555	-4354	1.48E-04	2502	0.333333	3.44868	2332784	ACVR2A
6338.321	-4354	1.48E-04	2452	0.333333	3.44868	2323778	TACR3
5109.726	-4354	1.48E-04	2141	0.333333	3.44845	2249810	PDE2A
5740.138	-4354	1.48E-04	2053	0.333333	3.44845	2316814	CCL22
6017.307	-4354	1.48E-04	2602	0.333333	3.44822	2354734	PPP3CA
6032.578	-4354	1.48E-04	2129	0.333333	3.44822	2266232	HYAL1
5448.545	-4354	1.48E-04	2312	0.333333	3.447991	2314170	PROC
5348.751	-4354	1.48E-04	2209	0.333333	3.447991	2285516	SMAD7
5551.423	-4354	1.48E-04	2509	0.333333	3.447532	2302640	GNAI1
4985.154	-4354	1.48E-04	2455	0.333333	3.447532	2211424	KDR
5354.72	-4354	1.48E-04	2010	0.333333	3.447532	2222200	ADGRB1
4826.896	-4354	1.48E-04	2279	0.333333	3.447072	2209324	CDH5
6529.301	-4354	1.48E-04	2154	0.333333	3.447072	2411528	HAGH
6101.806	-4354	1.48E-04	2520	0.333333	3.446843	2353802	PBX1
5220.807	-4354	1.48E-04	2131	0.333333	3.446843	2227018	CYFIP1
4789.519	-4354	1.48E-04	2094	0.333333	3.446843	2148724	WNT2
5444.609	-4354	1.48E-04	2001	0.333333	3.446843	2280740	ACOX2

6160.221	-4354	1.48E-04	1990	0.333333	3.446843	2381094	ATG2A
5959.325	-4354	1.48E-04	2060	0.333333	3.446383	2293354	VAMP8
5181.836	-4354	1.48E-04	2091	0.333333	3.446154	2208544	SLN
5730.772	-4354	1.48E-04	2324	0.333333	3.445924	2299018	SGMS1
5092.561	-4354	1.48E-04	2225	0.333333	3.445924	2240130	CTSH
6136.032	-4354	1.48E-04	2203	0.333333	3.445924	2395388	PFKM
5031.549	-4354	1.48E-04	2047	0.333333	3.445924	2182240	TIMP1
5628.834	-4354	1.48E-04	2016	0.333333	3.445924	2316232	PLPP2
5888.988	-4354	1.48E-04	2297	0.333333	3.445695	2315822	LBR
7674.968	-4354	1.48E-04	2527	0.333333	3.445465	2402956	UQCRFS1
5587.659	-4354	1.48E-04	2351	0.333333	3.445465	2249228	HLA-DRA
4869.64	-4354	1.48E-04	2328	0.333333	3.445235	2201580	FLNA
5074.88	-4354	1.48E-04	2300	0.333333	3.445235	2209778	ALOX5AP
5868.427	-4354	1.48E-04	2135	0.333333	3.445235	2223510	HEXB
5978.41	-4354	1.48E-04	2301	0.333333	3.445006	2343076	ICOS
5567.762	-4354	1.48E-04	2242	0.333333	3.445006	2285710	HSPB1
5410.346	-4354	1.48E-04	2088	0.333333	3.445006	2213616	RGN
4796.269	-4354	1.48E-04	2183	0.333333	3.444776	2190898	FGR
5446.779	-4354	1.48E-04	2160	0.333333	3.444776	2287160	STAR
5656.296	-4354	1.48E-04	2105	0.333333	3.444776	2274880	NCOA4
5441.234	-4354	1.48E-04	2035	0.333333	3.444546	2263906	RELB
4856.651	-4354	1.48E-04	2173	0.333333	3.444317	2157286	C1S
5433.181	-4354	1.48E-04	2049	0.333333	3.444317	2185902	IFNA5
5779.817	-4354	1.48E-04	2271	0.333333	3.444087	2250638	ASIC2
5923.149	-4354	1.48E-04	2249	0.333333	3.443858	2256798	PDHA2
5637.429	-4354	1.48E-04	2110	0.333333	3.443858	2261016	GNG5
6330.641	-4354	1.48E-04	1985	0.333333	3.443858	2368356	CLDN8
5280.684	-4354	1.48E-04	1987	0.333333	3.443628	2179978	SLC10A1
5306.193	-4354	1.48E-04	2231	0.333333	3.443398	2233226	GNB3
6287.703	-4354	1.48E-04	2451	0.333333	3.443169	2331472	PSMD2
5638.499	-4354	1.48E-04	1991	0.333333	3.443169	2284522	CLDN5
4889.736	-4354	1.48E-04	2064	0.333333	3.442939	2190358	MRAS
5706.085	-4354	1.48E-04	2218	0.333333	3.44271	2243032	MTTP
5594.018	-4354	1.48E-04	2152	0.333333	3.44271	2194732	CCL21
6211.971	-4354	1.48E-04	2352	0.333333	3.44248	2353666	KDM1A
7165.338	-4354	1.48E-04	2187	0.333333	3.44248	2669038	CHMP2A
4809.859	-4354	1.48E-04	2007	0.333333	3.44248	2147194	S100A4
5389.772	-4354	1.48E-04	2085	0.333333	3.44225	2223934	CASQ1
5121.735	-4354	1.47E-04	2114	0.333333	3.442021	2161398	F10
5538.484	-4354	1.47E-04	2102	0.333333	3.442021	2271682	ITGB7
5691.922	-4354	1.47E-04	2108	0.333333	3.441791	2266516	DUSP4

4987.932	-4354	1.47E-04	2415	0.333333	3.441561	2141942	LYN
5272.083	-4354	1.47E-04	2250	0.333333	3.441332	2243172	ABCC2
4709.454	-4354	1.47E-04	1949	0.333333	3.441332	2173478	NBL1
5066.103	-4354	1.47E-04	2300	0.333333	3.441102	2204842	SOX9
5484.732	-4354	1.47E-04	2094	0.333333	3.441102	2241016	IL24
4918.165	-4354	1.47E-04	2414	0.333333	3.440873	2169102	COL4A1
5625.209	-4354	1.47E-04	2168	0.333333	3.440873	2269508	TP53BP2
5568.783	-4354	1.47E-04	2251	0.333333	3.440643	2258244	KAT2B
5463.478	-4354	1.47E-04	2146	0.333333	3.440643	2161530	PRKACG
5144.389	-4354	1.47E-04	2359	0.333333	3.440184	2235732	SLC7A11
5314.248	-4354	1.47E-04	2291	0.333333	3.440184	2207588	IRF8
5504.475	-4354	1.47E-04	2208	0.333333	3.439954	2221990	CCR9
4883.522	-4354	1.47E-04	1990	0.333333	3.439954	2176472	P2RX1
6618.546	-4354	1.47E-04	1981	0.333333	3.439954	2432944	CLDN7
5287.536	-4354	1.47E-04	2067	0.333333	3.439724	2225118	TNNC1
4765.442	-4354	1.47E-04	2111	0.333333	3.439495	2104936	ADGRE5
4829.218	-4354	1.47E-04	2025	0.333333	3.439265	2171988	AEBP1
5847.234	-4354	1.47E-04	2206	0.333333	3.439036	2263238	TEC
5010.17	-4354	1.47E-04	2154	0.333333	3.439036	2129004	CASQ2
5155.915	-4354	1.47E-04	1969	0.333333	3.439036	2183372	HK3
5603.252	-4354	1.47E-04	2419	0.333333	3.438806	2249282	MAPK7
5555.369	-4354	1.47E-04	2288	0.333333	3.438806	2252054	CAMK4
5437.451	-4354	1.47E-04	2205	0.333333	3.438576	2247350	PF4
5362.09	-4354	1.47E-04	2078	0.333333	3.438576	2174554	DUSP9
5388.024	-4354	1.47E-04	2074	0.333333	3.438576	2121840	GDF5
5060.561	-4354	1.47E-04	2013	0.333333	3.438576	2183706	CLCF1
6155.685	-4354	1.47E-04	1951	0.333333	3.438576	2502654	PLA2G3
5659.249	-4354	1.47E-04	2446	0.333333	3.438347	2252354	CNTNAP2
6074.209	-4354	1.47E-04	2266	0.333333	3.438347	2319674	CALM1
4960.165	-4354	1.47E-04	2051	0.333333	3.438347	2171282	P2RX4
5433.241	-4354	1.47E-04	2030	0.333333	3.438347	2232880	GIT1
5048.727	-4354	1.47E-04	2036	0.333333	3.438117	2131184	CTSL
5229.862	-4354	1.47E-04	2006	0.333333	3.438117	2145086	IFNA7
5421.061	-4354	1.47E-04	2192	0.333333	3.437887	2192984	PCK1
5866.64	-4354	1.47E-04	2140	0.333333	3.437887	2292804	DGKD
4890.975	-4354	1.47E-04	2115	0.333333	3.437887	2164524	ASAP2
4429.366	-4354	1.47E-04	2024	0.333333	3.437887	2077890	DRAM1
5329.344	-4354	1.47E-04	2081	0.333333	3.437658	2164084	ADH7
4908.811	-4354	1.47E-04	2122	0.333333	3.437428	2112662	SGK1
5959.283	-4354	1.47E-04	2073	0.333333	3.437428	2218992	CLDN17
5487.601	-4354	1.47E-04	2630	0.333333	3.437199	2197988	FN1

4526.738	-4354	1.47E-04	2195	0.333333	3.437199	2088606	BDKRB2
5597.458	-4354	1.47E-04	2163	0.333333	3.437199	2222490	CXCL6
5257.716	-4354	1.47E-04	2153	0.333333	3.437199	2175282	PLA2G4A
5094.143	-4354	1.47E-04	2117	0.333333	3.437199	2150362	GRM6
5093.47	-4354	1.47E-04	2064	0.333333	3.437199	2175048	IGFBP2
6165.984	-4354	1.47E-04	1985	0.333333	3.437199	2242834	PGAM1
6086.137	-4354	1.47E-04	2335	0.333333	3.436969	2297098	GOT2
5148.57	-4354	1.47E-04	2276	0.333333	3.436969	2195282	ITGA6
5457.929	-4354	1.47E-04	2220	0.333333	3.436969	2186482	VAV1
4798.445	-4354	1.47E-04	2148	0.333333	3.436969	2082012	F2
5374.616	-4354	1.47E-04	2360	0.333333	3.43651	2198744	MEIS1
5147.968	-4354	1.47E-04	2329	0.333333	3.43651	2148146	MAP3K5
4810.68	-4354	1.47E-04	2093	0.333333	3.43651	2121966	CD80
4623.381	-4354	1.47E-04	1992	0.333333	3.43628	2081048	SEMA7A
5669.679	-4354	1.47E-04	2245	0.333333	3.436051	2142148	GC
4491.852	-4354	1.47E-04	2101	0.333333	3.436051	2072196	THBS2
4918.482	-4354	1.47E-04	2311	0.333333	3.435821	2171262	FLNB
4934.369	-4354	1.47E-04	2092	0.333333	3.435591	2138198	AQP9
5195.291	-4354	1.47E-04	2085	0.333333	3.435591	2125614	SLC10A2
5341.241	-4354	1.47E-04	2437	0.333333	3.435362	2166150	CXCR4
4525.255	-4354	1.47E-04	2133	0.333333	3.435362	2068516	PLAT
5556.394	-4354	1.47E-04	2121	0.333333	3.435362	2182408	GNB2
5627.781	-4354	1.47E-04	1976	0.333333	3.435362	2163382	CA14
4992.957	-4354	1.47E-04	2147	0.333333	3.435132	2139264	AOAH
5667.617	-4354	1.47E-04	2088	0.333333	3.434902	2221072	CCL20
5613.298	-4354	1.47E-04	2057	0.333333	3.434902	2147978	NAT2
5262.94	-4354	1.47E-04	1993	0.333333	3.434902	2203590	SLC1A5
4984.143	-4354	1.47E-04	2440	0.333333	3.434673	2114366	TGFBR2
5502.974	-4354	1.47E-04	2325	0.333333	3.434673	2214328	IL12A
5724.493	-4354	1.47E-04	2261	0.333333	3.434673	2196274	ATP6V1E1
5316.276	-4354	1.47E-04	2223	0.333333	3.434673	2113786	LHCGR
5131.389	-4354	1.47E-04	2052	0.333333	3.434673	2149730	HSD11B2
5218.974	-4354	1.47E-04	2122	0.333333	3.434214	2110432	AADAC
4846.707	-4354	1.47E-04	2377	0.333333	3.433984	2145492	ITGA2
4916.781	-4354	1.47E-04	2086	0.333333	3.433984	2093058	NPY
5034.871	-4354	1.47E-04	2001	0.333333	3.433984	2141072	ICAM3
5078.483	-4354	1.47E-04	2126	0.333333	3.433754	2069920	NTF3
4718.669	-4354	1.47E-04	2073	0.333333	3.433754	2073190	LGALS1
5023.946	-4354	1.47E-04	1982	0.333333	3.433754	2106944	TRPV4
6175.95	-4354	1.47E-04	2325	0.333333	3.433525	2257230	EHHADH
6335.349	-4354	1.47E-04	2324	0.333333	3.433525	2296560	PDPK1

5826.533	-4354	1.47E-04	2207	0.333333	3.433295	2215840	PPP2R1A
4631.524	-4354	1.47E-04	2186	0.333333	3.433295	2074716	LAPTM5
5539.192	-4354	1.47E-04	2056	0.333333	3.433295	2212448	ATG9A
4553.434	-4354	1.47E-04	2210	0.333333	3.433065	2052140	ANXA1
5201.098	-4354	1.47E-04	1989	0.333333	3.433065	2190342	DHCR24
5527.168	-4354	1.47E-04	1944	0.333333	3.433065	2195588	ORAI3
5105.402	-4354	1.47E-04	2323	0.333333	3.432836	2117898	BCL6
4941.417	-4354	1.47E-04	2035	0.333333	3.432836	2103398	HK1
5039.622	-4354	1.47E-04	2033	0.333333	3.432836	2037018	CFHR2
4591.426	-4354	1.47E-04	1966	0.333333	3.432836	2050634	CHAD
4855.756	-4354	1.47E-04	1947	0.333333	3.432606	2106930	IL32
5770.193	-4354	1.47E-04	2916	0.333333	3.432377	2197342	CTNNB1
5251.593	-4354	1.47E-04	2393	0.333333	3.432377	2155470	BMPR1B
5243.64	-4354	1.47E-04	2316	0.333333	3.432377	2144456	CACNA2D3
4916.3	-4354	1.47E-04	2054	0.333333	3.432377	2083960	CD1B
4740.19	-4354	1.47E-04	2172	0.333333	3.432147	2077136	IL10RA
4745.945	-4354	1.47E-04	2299	0.333333	3.431917	2052504	VIM
4608.978	-4354	1.47E-04	1944	0.333333	3.431917	2083646	SPHK1
5216.33	-4354	1.47E-04	1982	0.333333	3.431688	2114138	GABARAP
5091.404	-4354	1.46E-04	1993	0.333333	3.431458	2073384	TBXT
4345.375	-4354	1.46E-04	1943	0.333333	3.431458	2022146	ACKR1
4714.006	-4354	1.46E-04	1926	0.333333	3.431458	2009892	IL36A
4145.59	-4354	1.46E-04	2174	0.333333	3.431228	1984784	MMP2
5244.128	-4354	1.46E-04	2060	0.333333	3.430999	2117702	ALDH3A1
4794.139	-4354	1.46E-04	2040	0.333333	3.430999	2112216	MLC1
5678.406	-4354	1.46E-04	2027	0.333333	3.430999	2089816	GRN
4987.923	-4354	1.46E-04	1990	0.333333	3.430999	2062490	SEMA6C
5717.883	-4354	1.46E-04	2338	0.333333	3.430769	2188306	TIAM1
5607.253	-4354	1.46E-04	2298	0.333333	3.430769	2176224	MAML3
4933.594	-4354	1.46E-04	2070	0.333333	3.430769	2067514	ACSL1
4891.324	-4354	1.46E-04	2069	0.333333	3.430769	2115694	PYGM
4811.717	-4354	1.46E-04	2016	0.333333	3.430769	2097926	GADD45A
5331.003	-4354	1.46E-04	2116	0.333333	3.43054	2139674	PGM1
4664.342	-4354	1.46E-04	1991	0.333333	3.43054	2069758	EBI3
5061.048	-4354	1.46E-04	1951	0.333333	3.43054	2113046	LYL1
4589.303	-4354	1.46E-04	1919	0.333333	3.43054	2016658	CRLF2
5023.543	-4354	1.46E-04	2184	0.333333	3.43031	2089030	FOLH1
5424.87	-4354	1.46E-04	2145	0.333333	3.43031	2137184	ARPC1A
6869.147	-4354	1.46E-04	2138	0.333333	3.43031	2323676	UBA52
4942.896	-4354	1.46E-04	2088	0.333333	3.43031	2077064	CTSD
5603.654	-4354	1.46E-04	2465	0.333333	3.43008	2160378	MAGI2

5358.053	-4354	1.46E-04	2090	0.333333	3.43008	2104128	PCBP1
4933.374	-4354	1.46E-04	2036	0.333333	3.43008	2126260	IRF6
4400.251	-4354	1.46E-04	2022	0.333333	3.429851	2012548	COL4A4
5021.89	-4354	1.46E-04	2081	0.333333	3.429621	2106430	EDEM1
4872.249	-4354	1.46E-04	2043	0.333333	3.429621	2058828	CTSA
5583.503	-4354	1.46E-04	2014	0.333333	3.429621	2159750	PKMYT1
4391.65	-4354	1.46E-04	1963	0.333333	3.429621	2038126	IL3RA
5061.712	-4354	1.46E-04	2327	0.333333	3.429392	2114042	YWHAH
5073.056	-4354	1.46E-04	2043	0.333333	3.429392	2060050	LGMN
4928.404	-4354	1.46E-04	1979	0.333333	3.429392	2103508	BATF
5035.598	-4354	1.46E-04	2060	0.333333	3.429162	2070668	TSPO
5109.72	-4354	1.46E-04	1965	0.333333	3.429162	2089556	PYCARD
4820.046	-4354	1.46E-04	1948	0.333333	3.429162	2061980	SHC2
4359.378	-4354	1.46E-04	1948	0.333333	3.429162	2013760	TNFSF13
6095.231	-4354	1.46E-04	2030	0.333333	3.428932	2225202	EPHX2
4558.364	-4354	1.46E-04	1991	0.333333	3.428932	2030444	GPX2
5181.914	-4354	1.46E-04	1894	0.333333	3.428932	2163966	FAM117A
5177.405	-4354	1.46E-04	2186	0.333333	3.428703	2116206	ATP1A1
5090.806	-4354	1.46E-04	2017	0.333333	3.428703	2099732	EPHX1
4599.725	-4354	1.46E-04	2103	0.333333	3.428243	2006758	C6
5461.865	-4354	1.46E-04	2088	0.333333	3.428243	2178722	GOT1
4981.008	-4354	1.46E-04	2287	0.333333	3.428014	2139782	RAPGEF1
5085.572	-4354	1.46E-04	2124	0.333333	3.428014	2091096	EGF
4868.719	-4354	1.46E-04	2035	0.333333	3.428014	2081750	ID3
5253.803	-4354	1.46E-04	2482	0.333333	3.427784	2123480	CXCL12
4664.763	-4354	1.46E-04	2232	0.333333	3.427784	2040868	SPARC
4842.274	-4354	1.46E-04	2143	0.333333	3.427784	2025036	IL21
5589.017	-4354	1.46E-04	2105	0.333333	3.427784	2165048	LDHB
4687.468	-4354	1.46E-04	2062	0.333333	3.427784	2016872	LEP
4558.111	-4354	1.46E-04	1907	0.333333	3.427784	1974068	CA7
4629.238	-4354	1.46E-04	2307	0.333333	3.427555	2035438	EDNRB
4816.815	-4354	1.46E-04	2187	0.333333	3.427555	2039488	CCR4
5232.046	-4354	1.46E-04	2151	0.333333	3.427555	2118482	BLNK
5893.669	-4354	1.46E-04	2051	0.333333	3.427555	2176086	CACNG1
4910.904	-4354	1.46E-04	2024	0.333333	3.427555	2088132	CEBPE
5025.387	-4354	1.46E-04	2047	0.333333	3.427325	2084582	NEU1
4950.135	-4354	1.46E-04	2357	0.333333	3.427095	2124522	CDH1
5034.621	-4354	1.46E-04	2191	0.333333	3.427095	2104784	KCNN2
4713.617	-4354	1.46E-04	2075	0.333333	3.427095	2028810	IL33
5123.764	-4354	1.46E-04	2653	0.333333	3.426866	2102688	JUN
4883.872	-4354	1.46E-04	2416	0.333333	3.426636	2055784	NTRK2

5299.211	-4354	1.46E-04	2059	0.333333	3.426636	2169600	DDIT3
4486.136	-4354	1.46E-04	1972	0.333333	3.426636	2002236	CETP
5879.985	-4354	1.46E-04	2201	0.333333	3.426406	2143704	GSK3A
5293.57	-4354	1.46E-04	1916	0.333333	3.426406	2151772	PIP4K2C
5010.629	-4354	1.46E-04	2249	0.333333	3.426177	2031024	RAP1B
5235.704	-4354	1.46E-04	2129	0.333333	3.426177	2121458	RASGRP1
4610.464	-4354	1.46E-04	2072	0.333333	3.426177	1987120	CYP11A1
4432.157	-4354	1.46E-04	1887	0.333333	3.426177	1935166	FEV
5311.674	-4354	1.46E-04	2232	0.333333	3.425947	2069414	MBL2
4821.86	-4354	1.46E-04	2132	0.333333	3.425947	2012960	CPB2
4930.685	-4354	1.46E-04	1985	0.333333	3.425947	2034542	CTF1
5013.495	-4354	1.46E-04	1918	0.333333	3.425947	2038580	PDCD1
5376.703	-4354	1.46E-04	2407	0.333333	3.425718	2105568	PLCB4
6673.391	-4354	1.46E-04	2316	0.333333	3.425718	2376196	TEAD1
4719.657	-4354	1.46E-04	2244	0.333333	3.425718	2063454	GNA12
5367.165	-4354	1.46E-04	2216	0.333333	3.425718	2040142	F9
5642.27	-4354	1.46E-04	1983	0.333333	3.425718	2157558	PCK2
5379.38	-4354	1.46E-04	2463	0.333333	3.425488	2142412	FBXW7
5479.879	-4354	1.46E-04	2084	0.333333	3.425488	2142122	PIK3C2B
4571.549	-4354	1.46E-04	2074	0.333333	3.425488	2015112	PTGER2
5107.366	-4354	1.46E-04	2028	0.333333	3.425488	2033728	HHEX
4626.216	-4354	1.46E-04	1899	0.333333	3.425488	2053830	TLR5
4974.558	-4354	1.46E-04	2139	0.333333	3.425258	2058962	ICAM2
5340.044	-4354	1.46E-04	2086	0.333333	3.425258	2092828	POMC
4982.191	-4354	1.46E-04	1992	0.333333	3.425258	2036336	KCNF1
4968.987	-4354	1.46E-04	1940	0.333333	3.425258	2038310	IFNA17
4562.002	-4354	1.46E-04	1906	0.333333	3.425258	2046802	EFNA1
5667.035	-4354	1.46E-04	2101	0.333333	3.425029	2135056	TFAP4
4764.122	-4354	1.46E-04	2037	0.333333	3.425029	2001116	TSHB
5157.137	-4354	1.46E-04	1883	0.333333	3.425029	2057340	CLDN9
4811.901	-4354	1.46E-04	2213	0.333333	3.424799	2018394	GPC3
5332.695	-4354	1.46E-04	2193	0.333333	3.424799	2098450	MAPKAPK3
4933.814	-4354	1.46E-04	1944	0.333333	3.424799	2073480	ACSL5
4899.879	-4354	1.46E-04	1994	0.333333	3.424569	2031156	SIPA1
5210.508	-4354	1.46E-04	1961	0.333333	3.424569	2062742	SLC27A3
4785.142	-4354	1.46E-04	1961	0.333333	3.424569	2009458	TYROBP
5027.677	-4354	1.46E-04	2349	0.333333	3.42434	2058622	TCF7L2
4684.111	-4354	1.46E-04	2092	0.333333	3.42434	2060566	ZBTB16
6026.194	-4354	1.46E-04	1983	0.333333	3.42434	2148470	PARD6A
5161.429	-4354	1.46E-04	1947	0.333333	3.42434	2039850	CHI3L2
4555.167	-4354	1.46E-04	2179	0.333333	3.42411	2024826	ABCB1

5449.894	-4354	1.46E-04	2419	0.333333	3.423881	2072514	AKT1
5157.597	-4354	1.46E-04	2068	0.333333	3.423881	2012162	PDE6A
5475.55	-4354	1.46E-04	2048	0.333333	3.423881	2047130	TLX1
4952.255	-4354	1.46E-04	1912	0.333333	3.423881	2028174	TCIRG1
6051.604	-4354	1.46E-04	2146	0.333333	3.423651	2193156	BCL9
4341.325	-4354	1.46E-04	1970	0.333333	3.423651	1981904	NPR1
4734.807	-4354	1.46E-04	2245	0.333333	3.423421	2057452	ITPKB
5136.458	-4354	1.46E-04	2241	0.333333	3.423421	2049696	BTK
4975.054	-4354	1.46E-04	1967	0.333333	3.423421	2081476	CACNA2D2
6016.178	-4354	1.46E-04	1955	0.333333	3.423421	2144288	RGL2
4737.703	-4354	1.46E-04	1930	0.333333	3.423421	1966730	MYL7
4780.348	-4354	1.46E-04	2237	0.333333	3.423192	2036998	PGD
4544.721	-4354	1.46E-04	2023	0.333333	3.423192	2009510	ID1
4947.523	-4354	1.46E-04	1954	0.333333	3.423192	2018226	CORO1A
4851.321	-4354	1.46E-04	1897	0.333333	3.423192	2055770	TNFRSF12A
4408.436	-4354	1.46E-04	1999	0.333333	3.422962	2000466	SERPINB2
4717.872	-4354	1.46E-04	2074	0.333333	3.422732	1995594	MNDA
4737.122	-4354	1.46E-04	2008	0.333333	3.422732	1995348	FGG
4867.025	-4354	1.46E-04	2000	0.333333	3.422732	2038076	NOS2
5397.254	-4354	1.46E-04	1964	0.333333	3.422732	2069386	TUBB4A
4518.539	-4354	1.46E-04	1998	0.333333	3.422503	2007548	GABARAPL1
4673.708	-4354	1.46E-04	1972	0.333333	3.422503	2006992	SNAI1
5662.71	-4354	1.46E-04	2324	0.333333	3.422273	2099978	PRKN
4574.885	-4354	1.46E-04	2218	0.333333	3.422273	2001754	SEMA3A
5014.179	-4354	1.46E-04	2006	0.333333	3.422273	2074706	PC
5153.196	-4354	1.46E-04	1955	0.333333	3.422273	2050736	CYP4F3
4866.131	-4354	1.46E-04	2158	0.333333	3.421814	2014886	SELL
4825.597	-4354	1.46E-04	1989	0.333333	3.421814	1990212	SLC22A1
4395.903	-4354	1.46E-04	1946	0.333333	3.421814	1961358	LIF
8056.364	-4354	1.46E-04	1926	0.333333	3.421814	2298740	MT-ND4
4633.483	-4354	1.46E-04	1886	0.333333	3.421814	2014584	KRT16
4995.129	-4354	1.46E-04	2424	0.333333	3.421584	2077394	CBL
5291.147	-4354	1.46E-04	2189	0.333333	3.421584	2076750	STAM
4597.115	-4354	1.46E-04	2006	0.333333	3.421584	1922556	FGF23
5018.248	-4354	1.46E-04	1961	0.333333	3.421584	2053284	RAB3A
4291.812	-4354	1.46E-04	1944	0.333333	3.421584	1972892	ABLIM3
4713.583	-4354	1.46E-04	2289	0.333333	3.421355	1981522	GRIN2A
5020.437	-4354	1.46E-04	2244	0.333333	3.421355	2075526	CREB5
4268.252	-4354	1.46E-04	2046	0.333333	3.421355	1930970	GNG4
4892.478	-4354	1.46E-04	2037	0.333333	3.421125	2050344	CYP2J2
4503.645	-4354	1.46E-04	2037	0.333333	3.421125	1974464	GDF7

4322.186	-4354	1.46E-04	1952	0.333333	3.421125	1913470	F7
6389.039	-4354	1.46E-04	2604	0.333333	3.420896	2169142	MAPK14
4556.903	-4354	1.46E-04	2028	0.333333	3.420896	1918362	ADH1A
5226.02	-4354	1.46E-04	1944	0.333333	3.420896	2069364	AP1M2
4529.795	-4354	1.45E-04	2006	0.333333	3.420666	1990398	OSR2
4862.696	-4354	1.45E-04	2083	0.333333	3.420436	1983438	HRH2
4941.137	-4354	1.45E-04	1939	0.333333	3.419977	1976916	MOS
4724.117	-4354	1.45E-04	1897	0.333333	3.419977	2024416	TEAD3
4482.355	-4354	1.45E-04	2323	0.333333	3.419747	1972786	LAMC1
4573.563	-4354	1.45E-04	2089	0.333333	3.419747	1940368	FCER1G
5437	-4354	1.45E-04	2152	0.333333	3.419518	2069268	PARP1
4609.981	-4354	1.45E-04	2090	0.333333	3.419518	2000352	CD226
4144.264	-4354	1.45E-04	1959	0.333333	3.419518	1902120	GDF10
4883.419	-4354	1.45E-04	2268	0.333333	3.419288	2022780	VEGFA
4986.337	-4354	1.45E-04	2231	0.333333	3.419059	2047828	CSNK1E
4937.547	-4354	1.45E-04	2185	0.333333	3.419059	1985130	CDH12
5284.025	-4354	1.45E-04	1960	0.333333	3.419059	2024614	GPI
4976.235	-4354	1.45E-04	1870	0.333333	3.419059	2040186	FRAT2
5355.983	-4354	1.45E-04	2107	0.333333	3.418829	2027762	CCL19
5461.771	-4354	1.45E-04	2105	0.333333	3.418829	2025048	CYP7A1
4331.025	-4354	1.45E-04	2095	0.333333	3.418829	1906474	TLR2
5110.64	-4354	1.45E-04	2038	0.333333	3.418829	1960210	ONECUT1
4091.717	-4354	1.45E-04	1878	0.333333	3.418829	1906368	PLTP
4580.813	-4354	1.45E-04	2335	0.333333	3.418599	1952432	ZEB2
4979.518	-4354	1.45E-04	2059	0.333333	3.418599	2039594	GRAP2
4272.051	-4354	1.45E-04	1960	0.333333	3.418599	1940478	CD82
4580.561	-4354	1.45E-04	2141	0.333333	3.41814	1979860	CXCR2
4229.48	-4354	1.45E-04	2045	0.333333	3.41814	1921776	MMP9
4479.749	-4354	1.45E-04	2003	0.333333	3.41814	1931262	HLA-DRB1
5083.685	-4354	1.45E-04	2150	0.333333	3.41791	2046364	CXCL13
4809.791	-4354	1.45E-04	1912	0.333333	3.417681	1961194	WNT10B
4599.422	-4354	1.45E-04	2014	0.333333	3.417451	1937900	SERPINF2
5492.108	-4354	1.45E-04	1976	0.333333	3.417451	2014796	SLC3A2
4623.432	-4354	1.45E-04	1957	0.333333	3.417451	2021526	TRIM16
4208.915	-4354	1.45E-04	2123	0.333333	3.417222	1919060	ACTC1
4964.506	-4354	1.45E-04	2047	0.333333	3.417222	2007422	GATA1
4903.885	-4354	1.45E-04	1951	0.333333	3.417222	1992800	ATP6V0C
4151.017	-4354	1.45E-04	2119	0.333333	3.416992	1890474	BDKRB1
4869.31	-4354	1.45E-04	2015	0.333333	3.416992	1962394	CAPNS1
4346.432	-4354	1.45E-04	1971	0.333333	3.416992	1886074	FGF6
4224.954	-4354	1.45E-04	1929	0.333333	3.416992	1906658	CXCL16

4304.605	-4354	1.45E-04	1919	0.333333	3.416992	1934300	OLR1
4349.803	-4354	1.45E-04	1895	0.333333	3.416992	1910976	NINJ1
4617.42	-4354	1.45E-04	1945	0.333333	3.416762	1884122	IL3
5043.495	-4354	1.45E-04	2026	0.333333	3.416533	1990584	RAG2
4942.641	-4354	1.45E-04	1929	0.333333	3.416533	1978094	CCL17
5004.517	-4354	1.45E-04	2120	0.333333	3.416303	1974232	CD9
4669.694	-4354	1.45E-04	2103	0.333333	3.416303	1990224	NEDD4
5892.588	-4354	1.45E-04	2025	0.333333	3.416303	2150518	FABP5
4930.872	-4354	1.45E-04	2170	0.333333	3.416073	2020504	C5
5508.927	-4354	1.45E-04	2244	0.333333	3.415844	2026358	PPP1CC
5294.854	-4354	1.45E-04	2171	0.333333	3.415844	2029942	SLC2A1
5131.846	-4354	1.45E-04	2140	0.333333	3.415844	1943470	SYN3
4558.872	-4354	1.45E-04	2017	0.333333	3.415844	1921048	RXRG
4755.936	-4354	1.45E-04	1971	0.333333	3.415844	1968246	CLTCL1
4368.132	-4354	1.45E-04	1951	0.333333	3.415844	1926292	PLK2
4256.828	-4354	1.45E-04	1877	0.333333	3.415844	1873134	ADH1C
4911.59	-4354	1.45E-04	1924	0.333333	3.415614	2007686	ADIPOR2
4156.456	-4354	1.45E-04	2168	0.333333	3.415385	1912440	CCN2
4562.266	-4354	1.45E-04	2080	0.333333	3.415385	1930026	RALB
4855.689	-4354	1.45E-04	2000	0.333333	3.415385	1922050	ADIPOQ
4030.472	-4354	1.45E-04	1976	0.333333	3.415385	1854276	NCF2
5078.324	-4354	1.45E-04	2052	0.333333	3.415155	1971574	VSNL1
4134.815	-4354	1.45E-04	1961	0.333333	3.415155	1901790	TREM1
4161.796	-4354	1.45E-04	1875	0.333333	3.415155	1913766	SLPI
4443.958	-4354	1.45E-04	2074	0.333333	3.414925	1928096	OXTR
5092.14	-4354	1.45E-04	2480	0.333333	3.414696	1940572	STAT3
4517.6	-4354	1.45E-04	2101	0.333333	3.414696	1958264	DAPK2
5440.263	-4354	1.45E-04	2060	0.333333	3.414696	2037054	DDB1
4723.203	-4354	1.45E-04	1864	0.333333	3.414696	1956626	CDR1
5326.956	-4354	1.45E-04	1963	0.333333	3.414466	1982086	MGAM
4889.966	-4354	1.45E-04	1870	0.333333	3.414466	2009682	ALDOC
4286.056	-4354	1.45E-04	2077	0.333333	3.414237	1936140	SDC1
5292.45	-4354	1.45E-04	2028	0.333333	3.414237	1961500	SGK2
4602.617	-4354	1.45E-04	2179	0.333333	3.414007	1951774	DNM3
5246.026	-4354	1.45E-04	2010	0.333333	3.414007	2029316	LHPP
4057.382	-4354	1.45E-04	2035	0.333333	3.413777	1880232	BASP1
4820.828	-4354	1.45E-04	2004	0.333333	3.413777	1983038	PIM2
4585.113	-4354	1.45E-04	1985	0.333333	3.413777	1967046	SH2D2A
4703.746	-4354	1.45E-04	1872	0.333333	3.413777	1976794	CYP27B1
4556.006	-4354	1.45E-04	2202	0.333333	3.413548	1935442	MAP3K3
4426.117	-4354	1.45E-04	2091	0.333333	3.413548	1895642	NPY1R

4546.621	-4354	1.45E-04	2038	0.333333	3.413548	1946090	PROM1
4906.321	-4354	1.45E-04	2027	0.333333	3.413548	1987512	CXCL1
4221.31	-4354	1.45E-04	1928	0.333333	3.413548	1901978	CA4
4987.529	-4354	1.45E-04	2305	0.333333	3.413088	1960934	RDX
4620.786	-4354	1.45E-04	2108	0.333333	3.413088	1886046	DRD1
4714.816	-4354	1.45E-04	1936	0.333333	3.413088	1915712	PFKFB3
5019.972	-4354	1.45E-04	1971	0.333333	3.412859	1983798	CLDN14
4215.594	-4354	1.45E-04	1878	0.333333	3.412859	1889756	AKR1C3
4300.043	-4354	1.45E-04	2134	0.333333	3.412629	1914716	PRDM1
5620.227	-4354	1.45E-04	2156	0.333333	3.4124	2013636	CDK4
3913.496	-4354	1.45E-04	2095	0.333333	3.4124	1826692	COL4A2
3875.229	-4354	1.45E-04	2010	0.333333	3.4124	1838106	SDC2
4801.094	-4354	1.45E-04	1814	0.333333	3.41194	1919080	DSPP
4464.407	-4354	1.45E-04	2237	0.333333	3.411711	1914992	CDH2
4707.786	-4354	1.45E-04	2049	0.333333	3.411711	1898110	SAG
4671.195	-4354	1.45E-04	1945	0.333333	3.411711	1891438	MPZ
4374.474	-4354	1.45E-04	1931	0.333333	3.411711	1870938	IL15RA
4526.909	-4354	1.45E-04	1871	0.333333	3.411481	1904842	ABCD1
4192.122	-4354	1.45E-04	1860	0.333333	3.411481	1811954	PTCH2
4519.885	-4354	1.45E-04	1972	0.333333	3.411251	1866656	CCL11
5001.678	-4354	1.45E-04	1907	0.333333	3.411251	1986016	MGRN1
4120.339	-4354	1.45E-04	1861	0.333333	3.411251	1806534	SRD5A2
4380.92	-4354	1.45E-04	1848	0.333333	3.411251	1875986	ATP6AP1
4393.226	-4354	1.45E-04	2118	0.333333	3.410792	1912038	IL2RB
4914.436	-4354	1.45E-04	2022	0.333333	3.410792	1987218	WASF1
4418.508	-4354	1.45E-04	1871	0.333333	3.410792	1854850	GJD2
4096.915	-4354	1.45E-04	2161	0.333333	3.410563	1851252	GSN
4046.122	-4354	1.45E-04	1936	0.333333	3.410563	1895502	GADD45G
4868.884	-4354	1.45E-04	2013	0.333333	3.410333	1979354	ELOVL4
4654.836	-4354	1.45E-04	1998	0.333333	3.410333	1930878	MAP3K14
4375.678	-4354	1.45E-04	1996	0.333333	3.410333	1843526	INHA
3979.686	-4354	1.45E-04	1865	0.333333	3.410333	1826790	PROS1
5163.68	-4354	1.45E-04	2015	0.333333	3.410103	1997878	HDAC3
3953.095	-4354	1.45E-04	1986	0.333333	3.410103	1806744	CD14
4278.517	-4354	1.45E-04	1969	0.333333	3.410103	1889062	CCNA1
4726.997	-4354	1.45E-04	2306	0.333333	3.409874	1907748	PRKG1
4311.406	-4354	1.45E-04	1969	0.333333	3.409874	1883216	IL18
3810.131	-4354	1.45E-04	1943	0.333333	3.409874	1813794	ADM
4970.084	-4354	1.45E-04	1864	0.333333	3.409874	1904434	CCL1
4398.104	-4354	1.44E-04	2092	0.333333	3.409644	1908622	CCND1
4567.002	-4354	1.44E-04	1889	0.333333	3.409644	1868280	ATP6V0B

4054.465	-4354	1.44E-04	1854	0.333333	3.409644	1851290	PROCR
5125.901	-4354	1.44E-04	2140	0.333333	3.409414	1970864	YWHAG
4336.572	-4354	1.44E-04	2080	0.333333	3.409414	1892862	TNF
4801.219	-4354	1.44E-04	1981	0.333333	3.409414	1932350	GYS1
4356.747	-4354	1.44E-04	1902	0.333333	3.409414	1832692	SLC8A2
4016.947	-4354	1.44E-04	1854	0.333333	3.409414	1802502	TIMP2
4461.169	-4354	1.44E-04	2005	0.333333	3.409185	1846136	CACNA2D1
4199.457	-4354	1.44E-04	1846	0.333333	3.409185	1862070	DKK1
3936.774	-4354	1.44E-04	1842	0.333333	3.409185	1814736	PLA1A
4213.238	-4354	1.44E-04	1990	0.333333	3.408955	1821492	LY96
4909.768	-4354	1.44E-04	1911	0.333333	3.408955	1954448	SLC19A2
4503.801	-4354	1.44E-04	2124	0.333333	3.408726	1835812	TRHR
4217.425	-4354	1.44E-04	1986	0.333333	3.408726	1873852	BATF3
5030.941	-4354	1.44E-04	2103	0.333333	3.408496	1961236	CXCL9
5065.723	-4354	1.44E-04	1927	0.333333	3.408496	1899862	GLA
5286.397	-4354	1.44E-04	1852	0.333333	3.408496	2005776	CHD8
4064.483	-4354	1.44E-04	1843	0.333333	3.408496	1817596	CFD
4527.449	-4354	1.44E-04	2180	0.333333	3.408266	1867652	PROX1
4548.469	-4354	1.44E-04	2204	0.333333	3.408037	1919148	AFF1
4437.774	-4354	1.44E-04	2082	0.333333	3.408037	1868002	LAMB1
4459.504	-4354	1.44E-04	1970	0.333333	3.408037	1868784	AFP
4802.242	-4354	1.44E-04	1993	0.333333	3.407807	1847042	GSTA3
4551.696	-4354	1.44E-04	2002	0.333333	3.407577	1897218	SFN
4024.804	-4354	1.44E-04	2134	0.333333	3.407348	1816348	FBN1
4372.795	-4354	1.44E-04	2007	0.333333	3.407348	1849144	LIPA
4464.482	-4354	1.44E-04	2069	0.333333	3.407118	1862990	NTNG1
4084.46	-4354	1.44E-04	1877	0.333333	3.407118	1819372	ANGPTL4
3730.724	-4354	1.44E-04	1813	0.333333	3.406889	1776998	SEMA3G
4573.548	-4354	1.44E-04	2395	0.333333	3.406659	1884496	PLCB1
4630.975	-4354	1.44E-04	2147	0.333333	3.406659	1900840	RRAS2
4440.09	-4354	1.44E-04	2020	0.333333	3.406659	1873654	ARHGAP17
4615.845	-4354	1.44E-04	1877	0.333333	3.406659	1883188	AGPAT2
4168.69	-4354	1.44E-04	1845	0.333333	3.406659	1819152	IL17B
4485.712	-4354	1.44E-04	2094	0.333333	3.406429	1849096	GREM1
4220.711	-4354	1.44E-04	2119	0.333333	3.4062	1852814	ETV6
4411.794	-4354	1.44E-04	1989	0.333333	3.4062	1838160	CCK
4343.868	-4354	1.44E-04	1980	0.333333	3.4062	1823852	CAV3
4505.365	-4354	1.44E-04	1883	0.333333	3.40597	1819650	INS
3937.583	-4354	1.44E-04	2118	0.333333	3.405741	1799126	ACVRL1
4705.324	-4354	1.44E-04	2102	0.333333	3.405741	1902406	ZFHX3
4206.048	-4354	1.44E-04	1901	0.333333	3.405741	1769338	F13B

4127.767	-4354	1.44E-04	2042	0.333333	3.405511	1802818	CD55
4527.797	-4354	1.44E-04	1878	0.333333	3.405511	1810244	LHX5
4005.302	-4354	1.44E-04	1861	0.333333	3.405511	1787134	ZYX
4624.121	-4354	1.44E-04	2260	0.333333	3.405281	1883222	ATXN1
4273.667	-4354	1.44E-04	2056	0.333333	3.405052	1827886	HTR2B
5016.948	-4354	1.44E-04	2009	0.333333	3.405052	1947914	CD27
4547.092	-4354	1.44E-04	1919	0.333333	3.405052	1816172	CYP1A1
4906.71	-4354	1.44E-04	2061	0.333333	3.404822	1879556	TUBA1C
4681.192	-4354	1.44E-04	2011	0.333333	3.404592	1914752	CD38
5122.072	-4354	1.44E-04	1893	0.333333	3.404592	1839886	CYP11B2
4425.656	-4354	1.44E-04	2173	0.333333	3.404363	1873528	FRMD4A
4539.693	-4354	1.44E-04	2155	0.333333	3.404363	1869102	FLI1
4345.237	-4354	1.44E-04	2136	0.333333	3.404363	1853318	SLC1A1
4120.94	-4354	1.44E-04	1927	0.333333	3.404363	1830198	ACP5
3972.868	-4354	1.44E-04	1905	0.333333	3.404363	1788352	G0S2
4077.747	-4354	1.44E-04	1899	0.333333	3.404363	1805166	NFKBIE
4664.568	-4354	1.44E-04	1891	0.333333	3.404363	1830954	PFN1
5472.737	-4354	1.44E-04	2375	0.333333	3.404133	1957370	PSMD7
4163.81	-4354	1.44E-04	1917	0.333333	3.404133	1847144	HSPA2
4557.77	-4354	1.44E-04	1862	0.333333	3.404133	1798630	NEUROG1
4731.829	-4354	1.44E-04	2184	0.333333	3.403904	1861762	YAP1
4281.599	-4354	1.44E-04	2058	0.333333	3.403904	1826306	AMOT
4425.272	-4354	1.44E-04	1983	0.333333	3.403674	1853724	MAPK13
4267.381	-4354	1.44E-04	1834	0.333333	3.403674	1844148	WASF3
4228.76	-4354	1.44E-04	2027	0.333333	3.403444	1808052	GNG12
4685.449	-4354	1.44E-04	1990	0.333333	3.403444	1879586	TUBB2A
4251.726	-4354	1.44E-04	1944	0.333333	3.403444	1819668	VTCN1
4117.193	-4354	1.44E-04	1900	0.333333	3.403444	1784376	BMP3
5274.991	-4354	1.44E-04	1862	0.333333	3.403444	1935106	CDIPT
4297.826	-4354	1.44E-04	2198	0.333333	3.403215	1800800	SLIT2
5042.681	-4354	1.44E-04	1949	0.333333	3.403215	1857150	CCL25
4527.272	-4354	1.44E-04	1948	0.333333	3.403215	1854612	CASP7
4751.955	-4354	1.44E-04	1899	0.333333	3.403215	1923150	ATP6V0E2
4003.818	-4354	1.44E-04	1867	0.333333	3.403215	1768042	LEFTY1
4754.808	-4354	1.44E-04	1862	0.333333	3.403215	1888812	CS
4347.762	-4354	1.44E-04	1843	0.333333	3.403215	1844908	TNFRSF13B
4106.44	-4354	1.44E-04	1809	0.333333	3.403215	1747136	CACNA1S
5777.402	-4354	1.44E-04	1784	0.333333	3.403215	1986912	MT-CO2
4136.939	-4354	1.44E-04	1833	0.333333	3.402985	1755354	TBX4
5279.656	-4354	1.44E-04	1965	0.333333	3.402755	1935850	DNMT1
4483.644	-4354	1.44E-04	1805	0.333333	3.402755	1798754	FRMD1

4389.008	-4354	1.44E-04	2010	0.333333	3.402526	1807254	SOSTDC1
4382.798	-4354	1.44E-04	1823	0.333333	3.402526	1848594	CA11
3968.708	-4354	1.44E-04	2004	0.333333	3.402296	1779214	DGKI
4198.306	-4354	1.44E-04	1990	0.333333	3.402296	1796656	NR2F1
3993.278	-4354	1.44E-04	1979	0.333333	3.402296	1800748	ROR2
4880.529	-4354	1.44E-04	2004	0.333333	3.402067	1904100	SLC27A2
4433.876	-4354	1.44E-04	2319	0.333333	3.401837	1826244	SYK
4397.391	-4354	1.44E-04	2125	0.333333	3.401837	1797356	LMO3
4541.909	-4354	1.44E-04	1860	0.333333	3.401837	1860684	CTSV
4655.923	-4354	1.44E-04	2150	0.333333	3.401607	1831602	CTNNA3
3832.934	-4354	1.44E-04	2016	0.333333	3.401607	1762196	COL6A3
4244.406	-4354	1.44E-04	1952	0.333333	3.401378	1768014	C9
4566.394	-4354	1.44E-04	1932	0.333333	3.401378	1829530	AP3S1
4074.788	-4354	1.44E-04	1824	0.333333	3.401378	1766426	HLA-DMA
3966.414	-4354	1.44E-04	2044	0.333333	3.401148	1765628	FZD4
4347.508	-4354	1.44E-04	2088	0.333333	3.400918	1794076	ITGAV
5399.707	-4354	1.44E-04	2011	0.333333	3.400918	1946642	BPGM
3931.896	-4354	1.44E-04	1985	0.333333	3.400918	1757072	KLF4
3751.13	-4354	1.44E-04	1952	0.333333	3.400918	1735800	LMO2
4357.175	-4354	1.44E-04	1876	0.333333	3.400918	1804852	MAPK8IP1
4385.118	-4354	1.44E-04	2060	0.333333	3.400689	1798824	GYG1
3719.198	-4354	1.44E-04	1996	0.333333	3.400689	1722828	CFH
4655.023	-4354	1.44E-04	2195	0.333333	3.400459	1835576	PAK1
4456.781	-4354	1.44E-04	2180	0.333333	3.400459	1827172	FGF9
3603.967	-4354	1.44E-04	2009	0.333333	3.400459	1699776	F13A1
4217	-4354	1.44E-04	2116	0.333333	3.40023	1779656	PTHLH
4266.261	-4354	1.44E-04	1862	0.333333	3.40023	1779512	PCP4
4137.894	-4354	1.44E-04	1806	0.333333	3.40023	1807676	FCGBP
3875.743	-4354	1.44E-04	2186	0.333333	3.4	1766160	MITF
4015.794	-4354	1.44E-04	1975	0.333333	3.4	1777436	FZD7
4067.453	-4354	1.44E-04	1902	0.333333	3.4	1796016	BAMBI
4592.357	-4354	1.44E-04	1884	0.333333	3.4	1823794	ACADVL
3777.711	-4354	1.44E-04	1809	0.333333	3.4	1731740	LILRA3
3813.394	-4354	1.44E-04	1875	0.333333	3.39977	1737516	HMOX1
4060.686	-4354	1.44E-04	1854	0.333333	3.39977	1729018	IL13
4229.852	-4354	1.44E-04	2231	0.333333	3.399541	1821928	CAMK1D
4254.944	-4354	1.44E-04	2158	0.333333	3.399541	1829712	TNIK
4376.242	-4354	1.44E-04	2015	0.333333	3.399541	1834024	RHOBTB1
4204.341	-4354	1.44E-04	1949	0.333333	3.399541	1773456	CD40LG
4239.378	-4354	1.44E-04	1917	0.333333	3.399541	1784828	RAB8A
4008.505	-4354	1.44E-04	1910	0.333333	3.399541	1754522	TWIST1

3829.638	-4354	1.44E-04	1906	0.333333	3.399541	1713072	HEYL
4597.258	-4354	1.44E-04	1892	0.333333	3.399541	1830452	SV2A
3793.537	-4354	1.44E-04	1873	0.333333	3.399541	1716940	IL13RA2
4137.255	-4354	1.44E-04	1862	0.333333	3.399541	1782066	ACKR4
4249.812	-4354	1.44E-04	2139	0.333333	3.399311	1794196	PDGFC
4326.638	-4354	1.44E-04	1931	0.333333	3.399311	1815450	CCL2
4118.081	-4354	1.44E-04	1855	0.333333	3.399311	1781862	KRT1
3934.727	-4354	1.44E-04	1861	0.333333	3.399082	1752082	S100A9
5435.448	-4354	1.44E-04	2058	0.333333	3.398852	1903932	DLST
3803.804	-4354	1.44E-04	1938	0.333333	3.398852	1704478	CNTN2
3700.761	-4354	1.44E-04	1892	0.333333	3.398852	1731132	IL18R1
3985.251	-4354	1.44E-04	1860	0.333333	3.398852	1718382	EDA2R
4802.082	-4354	1.43E-04	1958	0.333333	3.398622	1839826	DERA
4448.014	-4354	1.43E-04	1869	0.333333	3.398622	1786600	PRDX1
4073.694	-4354	1.43E-04	2096	0.333333	3.398163	1791642	LMO7
5000.588	-4354	1.43E-04	2083	0.333333	3.398163	1825692	BIRC2
4161.502	-4354	1.43E-04	1977	0.333333	3.397933	1796824	EPHB3
4634.643	-4354	1.43E-04	1955	0.333333	3.397933	1850082	SALL4
4255.411	-4354	1.43E-04	1940	0.333333	3.397933	1822140	FKBP5
3937.455	-4354	1.43E-04	1878	0.333333	3.397933	1736348	BCL2A1
4476.975	-4354	1.43E-04	2184	0.333333	3.397704	1792352	CYP19A1
3930.924	-4354	1.43E-04	2101	0.333333	3.397704	1789992	TBC1D1
3958.346	-4354	1.43E-04	1869	0.333333	3.397704	1768316	ETV4
4497.726	-4354	1.43E-04	1831	0.333333	3.397704	1795672	DAGLA
4435.215	-4354	1.43E-04	1983	0.333333	3.397474	1837254	PNOC
3961.558	-4354	1.43E-04	1901	0.333333	3.397474	1682600	C8B
4092.335	-4354	1.43E-04	2462	0.333333	3.397245	1766948	FYN
4403.004	-4354	1.43E-04	2120	0.333333	3.397245	1835354	CYFIP2
4079.667	-4354	1.43E-04	2114	0.333333	3.397245	1762990	DKK2
4246.747	-4354	1.43E-04	2005	0.333333	3.397245	1829878	RHOBTB2
4311.997	-4354	1.43E-04	2104	0.333333	3.397015	1818164	CXXC4
3693.895	-4354	1.43E-04	1870	0.333333	3.397015	1712214	LGALS3
3607.318	-4354	1.43E-04	1857	0.333333	3.397015	1698382	RRAS
3857.983	-4354	1.43E-04	1787	0.333333	3.397015	1764046	ARNT2
3944.993	-4354	1.43E-04	2216	0.333333	3.396785	1732046	CNTN1
3973.152	-4354	1.43E-04	1994	0.333333	3.396785	1706562	ADCY8
4366.897	-4354	1.43E-04	1910	0.333333	3.396785	1733768	CCL16
4013.849	-4354	1.43E-04	1791	0.333333	3.396785	1770886	TP53I3
4564.199	-4354	1.43E-04	1777	0.333333	3.396785	1792508	ACP2
4123.296	-4354	1.43E-04	2052	0.333333	3.396326	1779852	IL7
4195.786	-4354	1.43E-04	1950	0.333333	3.396326	1787928	CCR7

3767.907	-4354	1.43E-04	1874	0.333333	3.396326	1674886	ROS1
4218.166	-4354	1.43E-04	2158	0.333333	3.396096	1773992	PTK2B
3786.342	-4354	1.43E-04	2023	0.333333	3.396096	1680142	A2M
3988.405	-4354	1.43E-04	1952	0.333333	3.396096	1748228	ATP1B1
3818.05	-4354	1.43E-04	1916	0.333333	3.396096	1712028	HEY1
3993.207	-4354	1.43E-04	1814	0.333333	3.396096	1706704	CDX2
3708.512	-4354	1.43E-04	1990	0.333333	3.395867	1688564	DUSP6
3982.858	-4354	1.43E-04	1836	0.333333	3.395867	1736988	DUSP5
3987.528	-4354	1.43E-04	1826	0.333333	3.395867	1685832	FZD9
4093.09	-4354	1.43E-04	1773	0.333333	3.395867	1733720	SCT
4240.554	-4354	1.43E-04	1949	0.333333	3.395637	1786928	PPIF
3617.811	-4354	1.43E-04	1909	0.333333	3.395637	1667456	VSIG4
3663.918	-4354	1.43E-04	1884	0.333333	3.395637	1701482	ALPL
4040.794	-4354	1.43E-04	1782	0.333333	3.395637	1759026	BEX1
4206.97	-4354	1.43E-04	2116	0.333333	3.395408	1736868	CCR3
4188.085	-4354	1.43E-04	2071	0.333333	3.395408	1734872	ARPC5
4269	-4354	1.43E-04	2011	0.333333	3.395178	1757022	WNT7A
4801.98	-4354	1.43E-04	1866	0.333333	3.395178	1812752	ECHS1
3732.298	-4354	1.43E-04	1861	0.333333	3.395178	1659144	SERPIND1
4175.17	-4354	1.43E-04	2114	0.333333	3.394948	1788620	PTPN13
4771.64	-4354	1.43E-04	1954	0.333333	3.394948	1791170	PTGES3
4890.519	-4354	1.43E-04	1734	0.333333	3.394948	1845346	SPATA2L
4687.371	-4354	1.43E-04	2105	0.333333	3.394489	1799864	CASP3
4691.847	-4354	1.43E-04	1870	0.333333	3.394489	1795808	GABARAPL2
4023.286	-4354	1.43E-04	2018	0.333333	3.394259	1745378	NECTIN3
4380.164	-4354	1.43E-04	1991	0.333333	3.394259	1789374	CA3
6193.019	-4354	1.43E-04	2284	0.333333	3.39403	1979002	ESR1
4632.181	-4354	1.43E-04	1980	0.333333	3.39403	1828948	EVL
4108.347	-4354	1.43E-04	1960	0.333333	3.39403	1746452	DUSP10
4236.094	-4354	1.43E-04	1802	0.333333	3.39403	1752400	CASP5
4094.031	-4354	1.43E-04	2009	0.333333	3.3938	1778304	RYR1
4032.261	-4354	1.43E-04	1852	0.333333	3.3938	1740158	G6PD
4191.164	-4354	1.43E-04	1833	0.333333	3.3938	1684624	GRK1
3723.533	-4354	1.43E-04	1742	0.333333	3.3938	1705684	LTC4S
4089.061	-4354	1.43E-04	2201	0.333333	3.393571	1746338	MAP4K4
3803.474	-4354	1.43E-04	1993	0.333333	3.393571	1717520	GPER1
4635.519	-4354	1.43E-04	1891	0.333333	3.393571	1750880	MYLK2
3956.762	-4354	1.43E-04	1883	0.333333	3.393571	1665324	ATP4A
4163.294	-4354	1.43E-04	2076	0.333333	3.393341	1766174	NOTCH1
4321.114	-4354	1.43E-04	1931	0.333333	3.393341	1735066	UGP2
5088.69	-4354	1.43E-04	1926	0.333333	3.393341	1837868	ITK

4501.228	-4354	1.43E-04	1920	0.333333	3.393341	1785294	CCNG1
3763.321	-4354	1.43E-04	2087	0.333333	3.393111	1666036	CHRM2
4299.111	-4354	1.43E-04	1998	0.333333	3.393111	1785464	HACD1
3931.945	-4354	1.43E-04	1789	0.333333	3.393111	1663584	TULP1
4081.162	-4354	1.43E-04	1783	0.333333	3.393111	1714982	MYLPF
3861.919	-4354	1.43E-04	1860	0.333333	3.392882	1670292	NCF4
4428.732	-4354	1.43E-04	1787	0.333333	3.392882	1776848	CTSF
4273.292	-4354	1.43E-04	1779	0.333333	3.392882	1721760	KRTAP8-1
4337.843	-4354	1.43E-04	1863	0.333333	3.392652	1763644	CCL7
4265.603	-4354	1.43E-04	1773	0.333333	3.392652	1736166	TUBB2B
4360.812	-4354	1.43E-04	2008	0.333333	3.392423	1691466	SERPINC1
3800.336	-4354	1.43E-04	1860	0.333333	3.392193	1702512	FXYD2
4110.972	-4354	1.43E-04	1855	0.333333	3.392193	1755110	ICAM4
5257.7	-4354	1.43E-04	2041	0.333333	3.391963	1957880	MEF2C
3809.917	-4354	1.43E-04	1849	0.333333	3.391963	1694594	NOD2
4367.841	-4354	1.43E-04	2207	0.333333	3.391734	1745842	GNAQ
3759.578	-4354	1.43E-04	2203	0.333333	3.391734	1686836	JAG1
4104.911	-4354	1.43E-04	2041	0.333333	3.391734	1747270	WASF2
4105.569	-4354	1.43E-04	1975	0.333333	3.391734	1679732	WIF1
3578.844	-4354	1.43E-04	1938	0.333333	3.391734	1651038	VCAN
4522.087	-4354	1.43E-04	1815	0.333333	3.391734	1779654	ABCF1
3683.843	-4354	1.43E-04	2152	0.333333	3.391504	1652932	AGTR1
4199.948	-4354	1.43E-04	2033	0.333333	3.391504	1689514	APOB
4091.161	-4354	1.43E-04	1955	0.333333	3.391504	1734126	TIPARP
4439.925	-4354	1.43E-04	1903	0.333333	3.391504	1734050	FGF3
4108.627	-4354	1.43E-04	1840	0.333333	3.391504	1746166	RND1
3878.409	-4354	1.43E-04	1768	0.333333	3.391504	1709454	TNFRSF10D
3868.367	-4354	1.43E-04	2150	0.333333	3.391274	1728422	EPHA4
4256.451	-4354	1.43E-04	1861	0.333333	3.391274	1700366	PPT1
3739.539	-4354	1.43E-04	1784	0.333333	3.391274	1692620	FADS3
3800.055	-4354	1.43E-04	1995	0.333333	3.391045	1657782	GABRA1
4271.11	-4354	1.43E-04	1949	0.333333	3.391045	1684466	C3AR1
3881.536	-4354	1.43E-04	1919	0.333333	3.391045	1684148	CYP7B1
4413.702	-4354	1.43E-04	1843	0.333333	3.391045	1761294	INPPL1
4027.391	-4354	1.43E-04	2012	0.333333	3.390815	1739306	FUT8
3790.849	-4354	1.43E-04	2092	0.333333	3.390586	1713418	CADM1
3934.916	-4354	1.43E-04	1921	0.333333	3.390586	1656446	FGF20
4993.042	-4354	1.43E-04	2231	0.333333	3.390356	1792666	SYT1
4082.913	-4354	1.43E-04	1984	0.333333	3.390356	1740034	SLC20A1
5463.544	-4354	1.43E-04	1970	0.333333	3.390356	1767540	UQCRC1
3758.396	-4354	1.43E-04	1884	0.333333	3.390356	1698248	FHL1

4259.159	-4354	1.43E-04	1868	0.333333	3.390356	1693998	ARPC3
4283.773	-4354	1.43E-04	1817	0.333333	3.390356	1696154	APOA4
4847.283	-4354	1.43E-04	2115	0.333333	3.389897	1759184	CDK7
4651.962	-4354	1.43E-04	2044	0.333333	3.389897	1774094	IKBKE
3462.833	-4354	1.43E-04	1981	0.333333	3.389897	1647458	PTGS1
4163.369	-4354	1.43E-04	1894	0.333333	3.389897	1746058	DGKA
3734.946	-4354	1.43E-04	2242	0.333333	3.389667	1666140	COL1A2
4608.578	-4354	1.43E-04	1917	0.333333	3.389667	1731322	SLC2A4
4513.304	-4354	1.43E-04	1800	0.333333	3.389667	1794968	IP6K1
4332.183	-4354	1.43E-04	1751	0.333333	3.389667	1693786	NTN3
4534.675	-4354	1.43E-04	2070	0.333333	3.389437	1727616	GABRB2
4484.862	-4354	1.43E-04	1962	0.333333	3.389437	1744650	GLUD1
3892.513	-4354	1.43E-04	1838	0.333333	3.389437	1652752	ARHGEF4
3840.041	-4354	1.43E-04	1786	0.333333	3.389437	1657208	ALOX12B
4074.145	-4354	1.43E-04	2114	0.333333	3.389208	1663098	CD53
4013.073	-4354	1.43E-04	2094	0.333333	3.389208	1727260	RORA
3886.818	-4354	1.43E-04	2087	0.333333	3.389208	1712034	MAF
3399.319	-4354	1.43E-04	1796	0.333333	3.389208	1610136	PLAU
4041.594	-4354	1.43E-04	1945	0.333333	3.388978	1697262	ARSB
3856.91	-4354	1.43E-04	1824	0.333333	3.388978	1706778	STX1A
3599.704	-4354	1.43E-04	1796	0.333333	3.388978	1624110	NR2E1
3677.01	-4354	1.43E-04	1765	0.333333	3.388978	1667236	SCUBE2
4348.453	-4354	1.43E-04	2001	0.333333	3.388749	1731940	VAT1L
4434.779	-4354	1.43E-04	1894	0.333333	3.388749	1673470	PRKG2
4263.798	-4354	1.43E-04	1835	0.333333	3.388749	1705170	SHOC2
4174.095	-4354	1.43E-04	2122	0.333333	3.388519	1703802	CTNND2
4015.835	-4354	1.43E-04	2013	0.333333	3.388519	1709024	ACO1
4029.903	-4354	1.43E-04	1943	0.333333	3.388519	1683388	HLA-B
4363.303	-4354	1.43E-04	1941	0.333333	3.388519	1747644	PAK6
4728.719	-4354	1.43E-04	1930	0.333333	3.388519	1748876	MDH1
4632.919	-4354	1.43E-04	1853	0.333333	3.388519	1794480	CLDN1
3517.793	-4354	1.43E-04	1811	0.333333	3.388519	1629434	LILRB3
4052.868	-4354	1.43E-04	1800	0.333333	3.388519	1650582	AQP8
4324.672	-4354	1.43E-04	1905	0.333333	3.388289	1710480	UBE2E3
3993.588	-4354	1.43E-04	1979	0.333333	3.38806	1691006	MKNK1
3743.499	-4354	1.43E-04	1918	0.333333	3.38806	1698888	IL12RB2
4094.418	-4354	1.43E-04	1812	0.333333	3.38806	1670808	HRC
3371.466	-4354	1.43E-04	1797	0.333333	3.38806	1603452	LILRB5
3448.41	-4354	1.43E-04	1885	0.333333	3.38783	1601924	C5AR1
3557.628	-4354	1.43E-04	1833	0.333333	3.38783	1639314	IL1R2
4268.821	-4354	1.43E-04	2119	0.333333	3.3876	1712900	ACTB

4252.204	-4354	1.43E-04	2103	0.333333	3.3876	1705230	CACNA1C
4481.721	-4354	1.43E-04	1930	0.333333	3.3876	1682540	KL
4172.813	-4354	1.43E-04	1871	0.333333	3.3876	1687830	ELANE
4479.526	-4354	1.42E-04	2150	0.333333	3.387371	1720788	MAPK6
4150.385	-4354	1.42E-04	2070	0.333333	3.387371	1737524	JARID2
3906.656	-4354	1.42E-04	1969	0.333333	3.387371	1658038	ATP1A2
3552.44	-4354	1.42E-04	1875	0.333333	3.387371	1598478	SELE
3827.659	-4354	1.42E-04	2192	0.333333	3.387141	1689264	PRKCA
3987.189	-4354	1.42E-04	2098	0.333333	3.387141	1692074	GRK5
3314.152	-4354	1.42E-04	1789	0.333333	3.387141	1600394	THBS4
5130.179	-4354	1.42E-04	2130	0.333333	3.386912	1815354	PLK1
4800.789	-4354	1.42E-04	2060	0.333333	3.386912	1764008	AXIN1
3830.599	-4354	1.42E-04	2035	0.333333	3.386912	1674174	DUSP3
3730.241	-4354	1.42E-04	1988	0.333333	3.386912	1611748	TNFAIP3
3824.957	-4354	1.42E-04	2018	0.333333	3.386452	1655566	PDGFD
6410.436	-4354	1.42E-04	1952	0.333333	3.386452	2159700	PPP1CA
3895.002	-4354	1.42E-04	1834	0.333333	3.386452	1691300	ERN1
5388.55	-4354	1.42E-04	1702	0.333333	3.386452	1833892	MT-CO1
3677.774	-4354	1.42E-04	2022	0.333333	3.386223	1661202	CCND2
5454.463	-4354	1.42E-04	1970	0.333333	3.385993	1782698	AGL
4237.272	-4354	1.42E-04	1840	0.333333	3.385993	1705740	SLC16A4
4552.28	-4354	1.42E-04	1788	0.333333	3.385993	1633902	HYAL4
3502.302	-4354	1.42E-04	1759	0.333333	3.385993	1629710	MT2A
4849.865	-4354	1.42E-04	1904	0.333333	3.385763	1755302	ACADM
3940.215	-4354	1.42E-04	1733	0.333333	3.385763	1653946	SLC2A6
3829.94	-4354	1.42E-04	2115	0.333333	3.385534	1657692	GNA14
4232.895	-4354	1.42E-04	1861	0.333333	3.385534	1618312	SLC22A2
3805.698	-4354	1.42E-04	2084	0.333333	3.385304	1632694	NRP2
3521.121	-4354	1.42E-04	2000	0.333333	3.385304	1628224	SPP1
3525.907	-4354	1.42E-04	1837	0.333333	3.385304	1625376	CD1D
4478.432	-4354	1.42E-04	1836	0.333333	3.385075	1653726	MANBA
3850.241	-4354	1.42E-04	2020	0.333333	3.384845	1658598	NCOA1
3767.665	-4354	1.42E-04	1935	0.333333	3.384845	1671326	PTPN14
4566.429	-4354	1.42E-04	1828	0.333333	3.384845	1743656	RAC3
3727.804	-4354	1.42E-04	1814	0.333333	3.384845	1670948	COL9A3
3739.886	-4354	1.42E-04	1726	0.333333	3.384845	1635758	KRT13
4279.516	-4354	1.42E-04	1856	0.333333	3.384615	1718788	ULK1
3827.596	-4354	1.42E-04	1817	0.333333	3.384615	1597132	IFNB1
4247.294	-4354	1.42E-04	2431	0.333333	3.384386	1696646	MAPK1
3903.402	-4354	1.42E-04	2102	0.333333	3.384386	1667446	ITPR1
4166.206	-4354	1.42E-04	1866	0.333333	3.384386	1711244	CBR1

4048.625	-4354	1.42E-04	1859	0.333333	3.384386	1672518	ALOX12
4749.585	-4354	1.42E-04	1855	0.333333	3.384386	1728894	IRF3
4079.571	-4354	1.42E-04	1745	0.333333	3.384386	1593672	CLPS
3842.326	-4354	1.42E-04	1735	0.333333	3.384386	1670540	TNFSF9
3657.412	-4354	1.42E-04	1733	0.333333	3.384386	1564028	CACNA1F
4141.992	-4354	1.42E-04	2071	0.333333	3.384156	1692126	SIPA1L1
3606.279	-4354	1.42E-04	1933	0.333333	3.384156	1574072	GRM3
5735.634	-4354	1.42E-04	1904	0.333333	3.384156	1881828	CDC37
3434.248	-4354	1.42E-04	1848	0.333333	3.384156	1581486	CTSG
3601.898	-4354	1.42E-04	1785	0.333333	3.384156	1578418	RCVRN
3391.119	-4354	1.42E-04	1846	0.333333	3.383927	1597836	AQP1
4083.264	-4354	1.42E-04	1752	0.333333	3.383927	1665212	ROM1
3632.015	-4354	1.42E-04	2093	0.333333	3.383697	1638000	NRXN3
3671.818	-4354	1.42E-04	2014	0.333333	3.383697	1613156	PRNP
3760.525	-4354	1.42E-04	1848	0.333333	3.383697	1655514	SCD5
3606.857	-4354	1.42E-04	1752	0.333333	3.383697	1624984	DDAH2
4520.808	-4354	1.42E-04	2011	0.333333	3.383467	1721466	HRAS
3696.392	-4354	1.42E-04	1972	0.333333	3.383467	1617474	SEMA3C
4090.006	-4354	1.42E-04	1938	0.333333	3.383467	1682768	STIM1
3480.413	-4354	1.42E-04	1894	0.333333	3.383467	1577982	IL6
4988.969	-4354	1.42E-04	1886	0.333333	3.383467	1798360	ARHGEF1
4208.732	-4354	1.42E-04	1870	0.333333	3.383467	1707782	SOD2
4237.846	-4354	1.42E-04	1843	0.333333	3.383467	1691712	KEAP1
4157.883	-4354	1.42E-04	1883	0.333333	3.383238	1674070	TYK2
4173.54	-4354	1.42E-04	1879	0.333333	3.383238	1693846	HYOU1
3832.979	-4354	1.42E-04	1829	0.333333	3.383238	1610046	NCF1
3508.474	-4354	1.42E-04	2051	0.333333	3.383008	1595528	NEDD9
3956.919	-4354	1.42E-04	1925	0.333333	3.383008	1639806	RPS6KA2
3652.118	-4354	1.42E-04	1822	0.333333	3.383008	1597184	MAP1LC3C
3163.443	-4354	1.42E-04	1738	0.333333	3.383008	1510312	THEMIS2
5133.305	-4354	1.42E-04	2103	0.333333	3.382778	1783518	CDC20
4129.557	-4354	1.42E-04	1995	0.333333	3.382778	1669694	PIK3R2
4239.192	-4354	1.42E-04	1830	0.333333	3.382778	1705408	ACSM3
3675.98	-4354	1.42E-04	1727	0.333333	3.382778	1592334	DLX5
4533.508	-4354	1.42E-04	2092	0.333333	3.382549	1708426	MAP2K4
3929.99	-4354	1.42E-04	1968	0.333333	3.382549	1637486	NRIP1
3612.93	-4354	1.42E-04	1944	0.333333	3.382549	1603286	GNS
3339.154	-4354	1.42E-04	1884	0.333333	3.382549	1561906	SHC3
3722.423	-4354	1.42E-04	1870	0.333333	3.382549	1619446	GUCY1B1
3634.056	-4354	1.42E-04	1773	0.333333	3.382549	1587800	TLR1
4305.419	-4354	1.42E-04	1715	0.333333	3.382549	1706296	NAT1

3650.091	-4354	1.42E-04	2011	0.333333	3.382319	1629398	ZFP36L1
4178.196	-4354	1.42E-04	2156	0.333333	3.38209	1680216	ERBB4
4507.03	-4354	1.42E-04	1914	0.333333	3.38209	1723226	CD3D
3671.878	-4354	1.42E-04	1983	0.333333	3.38186	1645980	AGPAT4
3921.07	-4354	1.42E-04	1893	0.333333	3.38186	1680562	GABBR1
4189.622	-4354	1.42E-04	1743	0.333333	3.38186	1594222	LMX1B
9743.669	-4354	1.42E-04	1703	0.333333	3.38186	4012822	DEFA5
4085.447	-4354	1.42E-04	2090	0.333333	3.38163	1659682	IFNG
4685.376	-4354	1.42E-04	2073	0.333333	3.38163	1731504	CXCL8
3970.382	-4354	1.42E-04	1906	0.333333	3.38163	1672606	FZD2
3740.289	-4354	1.42E-04	1882	0.333333	3.38163	1615028	APOE
4053.277	-4354	1.42E-04	1823	0.333333	3.38163	1598714	AVP
3670.852	-4354	1.42E-04	1793	0.333333	3.38163	1619416	SEMA4A
4106.477	-4354	1.42E-04	1854	0.333333	3.381401	1627194	PTH
3731.194	-4354	1.42E-04	1777	0.333333	3.381401	1559192	AKR1D1
4239.495	-4354	1.42E-04	2016	0.333333	3.381171	1709430	ELOVL6
4186.039	-4354	1.42E-04	1893	0.333333	3.381171	1632492	CSNK2B
3547.15	-4354	1.42E-04	1890	0.333333	3.381171	1606858	ABCC3
3154.005	-4354	1.42E-04	1871	0.333333	3.381171	1517336	MSN
3245.552	-4354	1.42E-04	1866	0.333333	3.381171	1546372	LPL
3523.377	-4354	1.42E-04	1758	0.333333	3.381171	1540012	CA5A
3659.998	-4354	1.42E-04	1936	0.333333	3.380941	1605234	HLA-DMB
3524.59	-4354	1.42E-04	1868	0.333333	3.380941	1589994	VDR
4174.297	-4354	1.42E-04	2345	0.333333	3.380712	1635938	GRB2
3596.618	-4354	1.42E-04	2057	0.333333	3.380712	1618770	MET
3662.671	-4354	1.42E-04	1862	0.333333	3.380712	1639346	CREB3L2
3501.706	-4354	1.42E-04	1791	0.333333	3.380712	1585422	SLC34A2
3388.966	-4354	1.42E-04	1737	0.333333	3.380712	1550700	HAS1
3915.763	-4354	1.42E-04	2195	0.333333	3.380482	1645726	ITGB1
4155.211	-4354	1.42E-04	2054	0.333333	3.380482	1587908	IQGAP1
3746.451	-4354	1.42E-04	1908	0.333333	3.380482	1606262	ACSL4
4871.436	-4354	1.42E-04	1843	0.333333	3.380482	1774094	KAT2A
3426.107	-4354	1.42E-04	1830	0.333333	3.380482	1562416	ZFP36
4451.821	-4354	1.42E-04	2112	0.333333	3.380253	1673224	PPP2CA
4494.537	-4354	1.42E-04	2049	0.333333	3.380253	1647098	PSMB3
3999.897	-4354	1.42E-04	1798	0.333333	3.380253	1608424	PITX2
3999.976	-4354	1.42E-04	1759	0.333333	3.380253	1669052	MOK
4456.164	-4354	1.42E-04	1687	0.333333	3.380253	1721360	CIR1
3884.918	-4354	1.42E-04	1972	0.333333	3.380023	1635652	DGKG
3289.17	-4354	1.42E-04	1936	0.333333	3.380023	1564224	EDN1
5143.73	-4354	1.42E-04	1775	0.333333	3.380023	1711904	PGAM2

4816.604	-4354	1.42E-04	1738	0.333333	3.380023	1693404	GPX4
3720.102	-4354	1.42E-04	1722	0.333333	3.380023	1581690	ATP6V0A4
4631.41	-4354	1.42E-04	1864	0.333333	3.379793	1683636	NF1
3207.544	-4354	1.42E-04	1846	0.333333	3.379793	1547820	MCAM
4615.965	-4354	1.42E-04	1772	0.333333	3.379793	1724326	MYBL2
3512.039	-4354	1.42E-04	2056	0.333333	3.379564	1570204	ACTN1
3340.525	-4354	1.42E-04	2035	0.333333	3.379564	1571834	ADAMTS1
3748.131	-4354	1.42E-04	1971	0.333333	3.379564	1592288	ADRA1B
3807.428	-4354	1.42E-04	1953	0.333333	3.379564	1625992	TGFA
3442.844	-4354	1.42E-04	1731	0.333333	3.379564	1511310	GJA8
4472.074	-4354	1.42E-04	1964	0.333333	3.379334	1683418	CDK5
3362.311	-4354	1.42E-04	1953	0.333333	3.379334	1555710	PTGS2
3390.114	-4354	1.42E-04	1943	0.333333	3.379334	1549006	FGF2
4300.276	-4354	1.42E-04	1935	0.333333	3.379334	1649286	SCP2
3412.588	-4354	1.42E-04	1923	0.333333	3.379334	1560854	MMP3
3438.08	-4354	1.42E-04	1834	0.333333	3.379334	1553690	JAM2
3580.596	-4354	1.42E-04	1826	0.333333	3.379334	1576314	JUNB
3819.613	-4354	1.42E-04	1740	0.333333	3.379334	1568344	PIK3C2G
4118.224	-4354	1.42E-04	2114	0.333333	3.379104	1661284	TGFBR1
4075.58	-4354	1.42E-04	1867	0.333333	3.379104	1648306	PPBP
4475.755	-4354	1.42E-04	1817	0.333333	3.379104	1712254	YY1AP1
3828.519	-4354	1.42E-04	1810	0.333333	3.379104	1605544	SLC5A1
4130.002	-4354	1.42E-04	1844	0.333333	3.378875	1667948	DDB2
3848.697	-4354	1.42E-04	1817	0.333333	3.378875	1608962	LPIN2
3184.944	-4354	1.42E-04	1728	0.333333	3.378875	1486770	TNN
4203.55	-4354	1.42E-04	1692	0.333333	3.378875	1655522	ATP6V1G2
3323.86	-4354	1.42E-04	1900	0.333333	3.378645	1535360	NTSR1
4137.922	-4354	1.42E-04	1828	0.333333	3.378645	1618908	CCL27
4620.157	-4354	1.42E-04	1892	0.333333	3.378416	1715998	ABCB7
3542.229	-4354	1.42E-04	1838	0.333333	3.378416	1561594	IL10
3753.079	-4354	1.42E-04	1928	0.333333	3.378186	1583274	MAP3K8
3948.653	-4354	1.42E-04	1828	0.333333	3.378186	1634360	MAD1L1
4207.204	-4354	1.42E-04	1752	0.333333	3.378186	1604660	RRAGA
4419.879	-4354	1.42E-04	1729	0.333333	3.378186	1683134	MYL12B
4462.662	-4354	1.42E-04	1890	0.333333	3.377956	1667660	UBE4A
4150.583	-4354	1.42E-04	2148	0.333333	3.377727	1670424	SP1
5667.295	-4354	1.42E-04	1806	0.333333	3.377727	1632782	UCP1
3499.339	-4354	1.42E-04	1789	0.333333	3.377727	1593678	CISH
4253.204	-4354	1.42E-04	1762	0.333333	3.377727	1648312	OXCT1
3291.116	-4354	1.42E-04	1812	0.333333	3.377497	1502662	CFI
4182.16	-4354	1.42E-04	2044	0.333333	3.377268	1645186	SATB1

4466.774	-4354	1.42E-04	2042	0.333333	3.377268	1698080	AR
3234.444	-4354	1.42E-04	1895	0.333333	3.377268	1524956	IGFBP3
3990.124	-4354	1.42E-04	1867	0.333333	3.377268	1591700	PAX6
4762.055	-4354	1.42E-04	1855	0.333333	3.377268	1689906	EEF2
4329.69	-4354	1.42E-04	1713	0.333333	3.377268	1720910	LCN2
4448.267	-4354	1.42E-04	1695	0.333333	3.377268	1650382	BANF1
3116.037	-4354	1.42E-04	1826	0.333333	3.377038	1483188	CD93
3583.787	-4354	1.42E-04	1765	0.333333	3.377038	1512090	SLC9A3
4362.112	-4354	1.42E-04	1707	0.333333	3.377038	1650780	ECI2
4197.663	-4354	1.42E-04	1840	0.333333	3.376808	1641424	PRKAG1
3248.605	-4354	1.42E-04	1836	0.333333	3.376808	1470368	HTR6
3636.109	-4354	1.42E-04	1918	0.333333	3.376579	1534528	LPAR4
3449.255	-4354	1.42E-04	1899	0.333333	3.376579	1555320	P2RY1
3652.773	-4354	1.42E-04	1819	0.333333	3.376579	1574880	STX3
3855.495	-4354	1.42E-04	1707	0.333333	3.376579	1567162	IL34
4576.757	-4354	1.42E-04	2200	0.333333	3.376349	1653412	GSK3B
3380.943	-4354	1.42E-04	1893	0.333333	3.376349	1536394	SLC1A3
4510.098	-4354	1.42E-04	2007	0.333333	3.376119	1623304	PSMC4
3856.89	-4354	1.42E-04	1932	0.333333	3.376119	1598054	RAB5C
4294.911	-4354	1.42E-04	1866	0.333333	3.376119	1654418	BNIP3
3976.966	-4354	1.42E-04	1860	0.333333	3.376119	1595302	CCR10
3988.535	-4354	1.42E-04	1814	0.333333	3.376119	1615604	LEPR
3853.82	-4354	1.42E-04	1777	0.333333	3.376119	1593032	CX3CL1
3617.819	-4354	1.42E-04	1769	0.333333	3.376119	1581438	MXD1
3375.99	-4354	1.42E-04	1719	0.333333	3.376119	1467142	BMP10
3588.062	-4354	1.41E-04	2097	0.333333	3.37566	1594492	SMAD1
3402.714	-4354	1.41E-04	1884	0.333333	3.37566	1552034	EFNB2
3534.371	-4354	1.41E-04	1807	0.333333	3.37566	1526470	CNGA1
4333.951	-4354	1.41E-04	1892	0.333333	3.375431	1669366	CD3G
4086.558	-4354	1.41E-04	1765	0.333333	3.375431	1613794	RELT
3413.275	-4354	1.41E-04	1711	0.333333	3.375431	1532624	LGALS9
4317.927	-4354	1.41E-04	2028	0.333333	3.375201	1646440	LCK
4336.537	-4354	1.41E-04	1973	0.333333	3.375201	1605288	POU3F3
3964.573	-4354	1.41E-04	1865	0.333333	3.375201	1622064	CXCL3
3646.513	-4354	1.41E-04	1832	0.333333	3.375201	1536998	AKR1C4
4103.706	-4354	1.41E-04	2007	0.333333	3.374971	1589066	CA10
3561.738	-4354	1.41E-04	1948	0.333333	3.374971	1557692	IL7R
4358.028	-4354	1.41E-04	1871	0.333333	3.374971	1717588	ORM1
3750.975	-4354	1.41E-04	1755	0.333333	3.374971	1513606	IFNA21
3571.437	-4354	1.41E-04	1731	0.333333	3.374971	1514380	FGF17
3915.81	-4354	1.41E-04	1985	0.333333	3.374742	1593026	MPDZ

4282.414	-4354	1.41E-04	1952	0.333333	3.374742	1681254	STRADB
3447.751	-4354	1.41E-04	1937	0.333333	3.374742	1504896	RIMS1
4291.665	-4354	1.41E-04	1893	0.333333	3.374742	1595848	PSMB10
3326.749	-4354	1.41E-04	1855	0.333333	3.374742	1535998	F5
3917.456	-4354	1.41E-04	1790	0.333333	3.374742	1603072	HOXA1
3589.329	-4354	1.41E-04	1777	0.333333	3.374742	1572328	TNFSF4
3674.972	-4354	1.41E-04	1746	0.333333	3.374742	1583856	F8
3855.868	-4354	1.41E-04	1912	0.333333	3.374512	1609778	SCD
4052.335	-4354	1.41E-04	1798	0.333333	3.374512	1630744	CARM1
3433.307	-4354	1.41E-04	1766	0.333333	3.374512	1538980	NECTIN2
4906.174	-4354	1.41E-04	1663	0.333333	3.374512	1720226	ATG4D
3681.073	-4354	1.41E-04	2038	0.333333	3.374282	1584532	IRS1
3831.333	-4354	1.41E-04	2025	0.333333	3.374282	1610016	RAB7A
4016.981	-4354	1.41E-04	2011	0.333333	3.374282	1589000	DCC
3829.635	-4354	1.41E-04	1854	0.333333	3.374282	1568262	KLKB1
3417.52	-4354	1.41E-04	1765	0.333333	3.374282	1520534	GLI1
3559.358	-4354	1.41E-04	1756	0.333333	3.374282	1516418	WNT1
3500.88	-4354	1.41E-04	1645	0.333333	3.374282	1527994	NDUFA4L2
3815.771	-4354	1.41E-04	1859	0.333333	3.374053	1555410	KCNK2
3822.67	-4354	1.41E-04	1788	0.333333	3.374053	1604378	CYP2S1
3931.045	-4354	1.41E-04	2040	0.333333	3.373823	1616326	PIK3CB
3537.869	-4354	1.41E-04	1852	0.333333	3.373823	1558636	ARNTL
3737.448	-4354	1.41E-04	1789	0.333333	3.373823	1520650	ANKRD1
3764.784	-4354	1.41E-04	1754	0.333333	3.373823	1553322	MYL3
4053.096	-4354	1.41E-04	1708	0.333333	3.373823	1616478	FZD6
3960.088	-4354	1.41E-04	1935	0.333333	3.373594	1562062	IL15
3418	-4354	1.41E-04	1788	0.333333	3.373594	1500254	SFRP5
4593.289	-4354	1.41E-04	1855	0.333333	3.373364	1613934	COX8A
3755.134	-4354	1.41E-04	1814	0.333333	3.373364	1530148	FGF8
4130.89	-4354	1.41E-04	1766	0.333333	3.373364	1604270	PRICKLE3
4029.779	-4354	1.41E-04	1694	0.333333	3.373364	1580454	TMSB4Y
3348.213	-4354	1.41E-04	2091	0.333333	3.373134	1510772	JAK1
3643.677	-4354	1.41E-04	2051	0.333333	3.373134	1560434	PIP4K2A
3906.479	-4354	1.41E-04	1955	0.333333	3.373134	1587366	PPARGC1A
3818.007	-4354	1.41E-04	1847	0.333333	3.373134	1540242	IL12B
3818.965	-4354	1.41E-04	1733	0.333333	3.373134	1588782	PORCN
3813.063	-4354	1.41E-04	1704	0.333333	3.373134	1599380	SEMA4B
3732.067	-4354	1.41E-04	1662	0.333333	3.373134	1573586	CAMP
3426.828	-4354	1.41E-04	2014	0.333333	3.372905	1500052	ID4
3736.972	-4354	1.41E-04	1941	0.333333	3.372905	1587208	TFAP2C
3657.391	-4354	1.41E-04	1743	0.333333	3.372905	1533430	DNAJB6

3195.987	-4354	1.41E-04	1722	0.333333	3.372905	1500878	MMP1
4258.873	-4354	1.41E-04	1698	0.333333	3.372905	1602012	CCL24
3264.527	-4354	1.41E-04	1825	0.333333	3.372675	1513776	SYNPO
3188.267	-4354	1.41E-04	1780	0.333333	3.372675	1504140	ITGA1
3220.674	-4354	1.41E-04	1675	0.333333	3.372675	1493532	STEAP4
4524.728	-4354	1.41E-04	1866	0.333333	3.372445	1624054	EIF2B1
3814.313	-4354	1.41E-04	1701	0.333333	3.372445	1561588	PLAAT3
3523.188	-4354	1.41E-04	1699	0.333333	3.372445	1491954	PLXNB3
3327.275	-4354	1.41E-04	1902	0.333333	3.372216	1490552	CTSC
3611.601	-4354	1.41E-04	1873	0.333333	3.372216	1482964	HTR5A
3593.521	-4354	1.41E-04	1741	0.333333	3.372216	1533018	ICAM5
3915.456	-4354	1.41E-04	1721	0.333333	3.372216	1583164	TAMALIN
3422.28	-4354	1.41E-04	1717	0.333333	3.372216	1457690	RBPJL
3185.033	-4354	1.41E-04	1647	0.333333	3.372216	1428932	ASIC4
3696.078	-4354	1.41E-04	1918	0.333333	3.371986	1546102	NRCAM
3403.597	-4354	1.41E-04	1844	0.333333	3.371986	1476458	IFNGR1
3843.078	-4354	1.41E-04	1824	0.333333	3.371986	1503074	PNLIP
3677.229	-4354	1.41E-04	1726	0.333333	3.371986	1491206	GCK
3722.132	-4354	1.41E-04	1703	0.333333	3.371986	1598196	FOXO4
3620.848	-4354	1.41E-04	1847	0.333333	3.371757	1508412	MARCKS
3644.495	-4354	1.41E-04	1818	0.333333	3.371757	1568944	INSIG1
3476.687	-4354	1.41E-04	1734	0.333333	3.371757	1560022	CBR3
3576.846	-4354	1.41E-04	1645	0.333333	3.371757	1559648	SLC6A9
4018.554	-4354	1.41E-04	1988	0.333333	3.371527	1544020	PSMB8
4386.378	-4354	1.41E-04	2152	0.333333	3.371297	1575288	PSMD14
3946.288	-4354	1.41E-04	1977	0.333333	3.371068	1551318	RASAL2
3554.586	-4354	1.41E-04	1804	0.333333	3.371068	1539916	FLT3
3264.171	-4354	1.41E-04	1780	0.333333	3.371068	1502686	IL6R
3274.019	-4354	1.41E-04	1726	0.333333	3.371068	1442296	CFHR4
3665.87	-4354	1.41E-04	1703	0.333333	3.371068	1488424	GYS2
3670.24	-4354	1.41E-04	1685	0.333333	3.371068	1498904	HPGDS
3552.069	-4354	1.41E-04	1986	0.333333	3.370838	1510590	CCR5
3733.625	-4354	1.41E-04	1962	0.333333	3.370838	1567784	LARGE1
3700.722	-4354	1.41E-04	1741	0.333333	3.370838	1502452	CYP46A1
3985.982	-4354	1.41E-04	2022	0.333333	3.370608	1610836	CDK6
3703.887	-4354	1.41E-04	1970	0.333333	3.370608	1548694	ATP2B1
3334.862	-4354	1.41E-04	1795	0.333333	3.370608	1513388	F2RL1
3453.21	-4354	1.41E-04	1759	0.333333	3.370608	1494102	CYP24A1
3264.742	-4354	1.41E-04	1753	0.333333	3.370608	1503814	ADCY3
3466.344	-4354	1.41E-04	1727	0.333333	3.370608	1505880	PARP4
3715.407	-4354	1.41E-04	1975	0.333333	3.370379	1547398	MECOM

3931.476	-4354	1.41E-04	1894	0.333333	3.370379	1594664	SLC16A1
3159.866	-4354	1.41E-04	1773	0.333333	3.370379	1498788	TNFRSF11A
3275.034	-4354	1.41E-04	1699	0.333333	3.370379	1447118	PSAP
3835.647	-4354	1.41E-04	2032	0.333333	3.370149	1590128	NCOR2
3817.296	-4354	1.41E-04	1856	0.333333	3.370149	1527824	RPS6KA3
3580.589	-4354	1.41E-04	1756	0.333333	3.370149	1545446	SPINT1
3026.29	-4354	1.41E-04	1727	0.333333	3.370149	1436168	CP
3622.095	-4354	1.41E-04	2002	0.333333	3.36992	1563960	PARD3
3189.842	-4354	1.41E-04	1842	0.333333	3.36992	1471656	TGFB3
3336.943	-4354	1.41E-04	1803	0.333333	3.36992	1480390	FCGR2B
3662.878	-4354	1.41E-04	1993	0.333333	3.36969	1509898	GRIA1
5446.524	-4354	1.41E-04	1729	0.333333	3.36969	1979268	CHMP7
3287.401	-4354	1.41E-04	1677	0.333333	3.36969	1502406	RGS9
4250.432	-4354	1.41E-04	1898	0.333333	3.36946	1600792	MLH1
3640.926	-4354	1.41E-04	1866	0.333333	3.36946	1469608	F11
3867.987	-4354	1.41E-04	1792	0.333333	3.36946	1596276	VMP1
3462.498	-4354	1.41E-04	1675	0.333333	3.36946	1458368	SEMA6B
4102.929	-4354	1.41E-04	2011	0.333333	3.369231	1614716	NLK
3065.065	-4354	1.41E-04	1818	0.333333	3.369231	1458604	OSMR
3647.698	-4354	1.41E-04	1812	0.333333	3.369231	1541008	RAPGEF4
3274.607	-4354	1.41E-04	1826	0.333333	3.369001	1459558	LOX
3112.796	-4354	1.41E-04	1782	0.333333	3.369001	1488124	SDC3
4619.331	-4354	1.41E-04	1759	0.333333	3.369001	1592532	AIFM1
3944.214	-4354	1.41E-04	1757	0.333333	3.369001	1586988	GPX7
4016.213	-4354	1.41E-04	1796	0.333333	3.368772	1561810	GNG10
3760.29	-4354	1.41E-04	1753	0.333333	3.368772	1529736	ADPGK
3294.984	-4354	1.41E-04	1782	0.333333	3.368542	1512004	STEAP3
3446.557	-4354	1.41E-04	1716	0.333333	3.368542	1475296	GUCY2D
3251.755	-4354	1.41E-04	1676	0.333333	3.368542	1440044	CFHR5
3504.079	-4354	1.41E-04	1939	0.333333	3.368312	1518022	PLXNC1
4016.569	-4354	1.41E-04	1884	0.333333	3.368312	1575628	AHCY
4414.203	-4354	1.41E-04	1769	0.333333	3.368312	1597796	PLA2G4D
3899.909	-4354	1.41E-04	1769	0.333333	3.368312	1517112	SLC25A5
2967.466	-4354	1.41E-04	1766	0.333333	3.368312	1401824	MRC1
2927.824	-4354	1.41E-04	1675	0.333333	3.368312	1412848	CH25H
4653.05	-4354	1.41E-04	1673	0.333333	3.368312	1619986	PTGDS
3400.708	-4354	1.41E-04	1949	0.333333	3.368083	1515584	MBP
3640.557	-4354	1.41E-04	1911	0.333333	3.368083	1562010	MAP2K6
3378.75	-4354	1.41E-04	1850	0.333333	3.368083	1506322	COL9A2
3447.842	-4354	1.41E-04	1846	0.333333	3.368083	1489766	WNT5A
3704.674	-4354	1.41E-04	1819	0.333333	3.368083	1511264	TP73

3725.27	-4354	1.41E-04	1779	0.333333	3.368083	1464850	FSHB
6415.645	-4354	1.41E-04	1761	0.333333	3.368083	2716128	MKNK2
3473.667	-4354	1.41E-04	1688	0.333333	3.368083	1493714	CST7
3249.923	-4354	1.41E-04	1679	0.333333	3.368083	1436510	GDF2
3825.344	-4354	1.41E-04	1951	0.333333	3.367853	1533922	SMAD6
4459.153	-4354	1.41E-04	1656	0.333333	3.367853	1570930	SEMA3B
3612.788	-4354	1.41E-04	1951	0.333333	3.367623	1512256	TJP1
3345.715	-4354	1.41E-04	1829	0.333333	3.367623	1520536	ABCB4
3517.941	-4354	1.41E-04	1668	0.333333	3.367623	1478858	IL1RL2
4361.258	-4354	1.41E-04	1780	0.333333	3.367394	1577594	PHB2
3643.638	-4354	1.41E-04	1758	0.333333	3.367394	1483328	THY1
3503.927	-4354	1.41E-04	1715	0.333333	3.367394	1456052	GDF3
3212.961	-4354	1.41E-04	1705	0.333333	3.367394	1441142	LILRB1
3180.59	-4354	1.41E-04	1685	0.333333	3.367394	1469272	DDIT4
3933.789	-4354	1.41E-04	2037	0.333333	3.367164	1553994	NFIA
3313.927	-4354	1.41E-04	1851	0.333333	3.367164	1485972	CKAP4
3030.544	-4354	1.41E-04	1808	0.333333	3.367164	1419684	GBP2
3625.508	-4354	1.41E-04	1726	0.333333	3.367164	1481552	ALDOA
3499.847	-4354	1.41E-04	2058	0.333333	3.366935	1509812	FGFR2
3526.658	-4354	1.41E-04	1989	0.333333	3.366935	1509556	PRKD1
3661.201	-4354	1.41E-04	1816	0.333333	3.366935	1543496	ELOVL7
3773.972	-4354	1.41E-04	1773	0.333333	3.366935	1530978	CD79A
3125.906	-4354	1.41E-04	1881	0.333333	3.366705	1436512	ITGA8
3216.672	-4354	1.41E-04	1761	0.333333	3.366705	1475774	LTF
3654.971	-4354	1.41E-04	1720	0.333333	3.366705	1500072	TNNC2
3082.687	-4354	1.41E-04	1640	0.333333	3.366705	1447216	LILRA6
3560.664	-4354	1.41E-04	1737	0.333333	3.366475	1511204	PIP5K1C
3243.269	-4354	1.41E-04	1734	0.333333	3.366475	1498442	CDKN2B
3521.609	-4354	1.41E-04	1701	0.333333	3.366475	1500136	TBKBP1
4386.999	-4354	1.41E-04	1696	0.333333	3.366475	1606816	DHRS11
4054.583	-4354	1.41E-04	1979	0.333333	3.366246	1582316	SUCLG2
3736.493	-4354	1.41E-04	1894	0.333333	3.366016	1513484	VDAC2
3052.779	-4354	1.41E-04	1756	0.333333	3.366016	1418524	SAT1
3648.403	-4354	1.41E-04	1704	0.333333	3.366016	1525434	ALDH9A1
3229.623	-4354	1.41E-04	1682	0.333333	3.366016	1425746	MAN2B1
4840.514	-4354	1.41E-04	1665	0.333333	3.366016	1578610	PTGES2
3428.35	-4354	1.41E-04	1834	0.333333	3.365786	1483836	SEL1L3
3183.718	-4354	1.41E-04	1806	0.333333	3.365786	1449200	CACNB4
3022.142	-4354	1.41E-04	1743	0.333333	3.365786	1418388	PLAUR
3198.065	-4354	1.41E-04	1666	0.333333	3.365786	1436346	PRPH2
3538.148	-4354	1.41E-04	1907	0.333333	3.365557	1509576	ALCAM

3844.603	-4354	1.41E-04	1855	0.333333	3.365557	1505296	UBE2M
3189.672	-4354	1.41E-04	1816	0.333333	3.365557	1464068	JAM3
3819.224	-4354	1.41E-04	2038	0.333333	3.365327	1528786	ANK3
3200.504	-4354	1.41E-04	1809	0.333333	3.365327	1477068	HPGD
3466.256	-4354	1.41E-04	1660	0.333333	3.365327	1454976	ANGPT4
4306.958	-4354	1.41E-04	2129	0.333333	3.365098	1581648	APC
3764.804	-4354	1.41E-04	1977	0.333333	3.365098	1547848	FER
3693.217	-4354	1.41E-04	1797	0.333333	3.364868	1455558	PRKCG
3391.748	-4354	1.41E-04	1759	0.333333	3.364868	1460294	PREX1
3237.386	-4354	1.41E-04	1738	0.333333	3.364868	1448478	GLUL
3254.417	-4354	1.41E-04	1607	0.333333	3.364868	1397464	KRT38
4113.922	-4354	1.41E-04	1792	0.333333	3.364638	1534842	SDHA
3231.324	-4354	1.41E-04	1753	0.333333	3.364638	1425846	C1QA
3474.987	-4354	1.40E-04	1956	0.333333	3.364409	1501758	CDK14
3110.508	-4354	1.40E-04	1743	0.333333	3.364409	1368058	GRP
3675.875	-4354	1.40E-04	1723	0.333333	3.364409	1543184	MCU
3114.48	-4354	1.40E-04	1861	0.333333	3.364179	1415958	PTAFR
4784.359	-4354	1.40E-04	1824	0.333333	3.364179	1594682	COX6C
3359.619	-4354	1.40E-04	1730	0.333333	3.364179	1436686	GPR3
4006.949	-4354	1.40E-04	1653	0.333333	3.364179	1532636	AAMP
3520.596	-4354	1.40E-04	1781	0.333333	3.363949	1448594	TNFSF18
2994.808	-4354	1.40E-04	1726	0.333333	3.363949	1411686	COMP
3928.275	-4354	1.40E-04	2072	0.333333	3.36372	1521388	PIK3CA
3919.629	-4354	1.40E-04	1940	0.333333	3.36372	1536956	CUX1
3419.049	-4354	1.40E-04	1906	0.333333	3.36372	1473042	NLGN1
4361.224	-4354	1.40E-04	1779	0.333333	3.36372	1547458	RPS6KB2
3541.895	-4354	1.40E-04	1749	0.333333	3.36372	1479680	IDO1
3725.971	-4354	1.40E-04	1720	0.333333	3.36372	1503758	CREB3
4231.417	-4354	1.40E-04	1699	0.333333	3.36372	1497314	FUCA2
3052.811	-4354	1.40E-04	1694	0.333333	3.36372	1345128	HOXD10
3448.417	-4354	1.40E-04	1914	0.333333	3.36349	1500606	MYH10
3571.156	-4354	1.40E-04	1813	0.333333	3.36349	1476478	IRAK1
3245.343	-4354	1.40E-04	1783	0.333333	3.36349	1461554	TJP2
3536.744	-4354	1.40E-04	1681	0.333333	3.36349	1478908	ASL
3446.755	-4354	1.40E-04	1675	0.333333	3.36349	1500786	TP53INP2
3165.577	-4354	1.40E-04	1658	0.333333	3.36349	1425854	METRNL
3222.571	-4354	1.40E-04	1610	0.333333	3.36349	1389892	TREH
3736.196	-4354	1.40E-04	2051	0.333333	3.363261	1517682	IGF1R
3445.562	-4354	1.40E-04	1860	0.333333	3.363261	1420612	PTGFR
3254.617	-4354	1.40E-04	1796	0.333333	3.363261	1454502	HMGA2
4025.092	-4354	1.40E-04	1674	0.333333	3.363261	1555098	MYL6B

3426.421	-4354	1.40E-04	1800	0.333333	3.362801	1450906	RSPO3
3070.619	-4354	1.40E-04	1730	0.333333	3.362801	1392770	FZD10
4050.634	-4354	1.40E-04	1661	0.333333	3.362801	1458528	GSTO1
3753.056	-4354	1.40E-04	1628	0.333333	3.362801	1498136	DGAT1
3371.16	-4354	1.40E-04	1877	0.333333	3.362572	1446716	RYR2
3336.126	-4354	1.40E-04	1846	0.333333	3.362572	1412284	COL9A1
3220.07	-4354	1.40E-04	1846	0.333333	3.362572	1439612	PLD1
3647.981	-4354	1.40E-04	1838	0.333333	3.362572	1486944	ITGA4
3763.909	-4354	1.40E-04	2051	0.333333	3.362342	1503770	GNB1
4169.87	-4354	1.40E-04	1913	0.333333	3.362342	1495260	GALC
3799.759	-4354	1.40E-04	1879	0.333333	3.362342	1520696	ZAP70
4126.67	-4354	1.40E-04	1832	0.333333	3.362342	1559256	PTTG1
3996.964	-4354	1.40E-04	1775	0.333333	3.362342	1529994	MAP4K1
3539.522	-4354	1.40E-04	1772	0.333333	3.362342	1487112	CD2
3818.514	-4354	1.40E-04	1749	0.333333	3.362342	1519166	FABP1
3842.015	-4354	1.40E-04	1733	0.333333	3.362342	1530192	MAML1
3442.051	-4354	1.40E-04	1774	0.333333	3.362113	1483080	TRIM25
3701.391	-4354	1.40E-04	1734	0.333333	3.362113	1490670	NAGPA
3265.191	-4354	1.40E-04	1697	0.333333	3.362113	1451702	CD1C
3127.685	-4354	1.40E-04	1676	0.333333	3.362113	1433340	LAMP3
3826.723	-4354	1.40E-04	1657	0.333333	3.362113	1429986	GAA
3641.922	-4354	1.40E-04	1652	0.333333	3.362113	1421962	IL17C
3126.096	-4354	1.40E-04	1599	0.333333	3.362113	1413822	PLPP1
5747.605	-4354	1.40E-04	1891	0.333333	3.361883	1615050	NDUFV2
3258.146	-4354	1.40E-04	1847	0.333333	3.361883	1408470	FRAS1
3750.857	-4354	1.40E-04	1665	0.333333	3.361883	1490796	PFKFB4
5019.524	-4354	1.40E-04	1943	0.333333	3.361653	1584414	NDUFS2
3595.439	-4354	1.40E-04	1884	0.333333	3.361653	1457872	F2R
3900.606	-4354	1.40E-04	1749	0.333333	3.361653	1547636	DEPDC5
3205.471	-4354	1.40E-04	1699	0.333333	3.361653	1395412	NTS
3266.558	-4354	1.40E-04	1803	0.333333	3.361424	1460064	APH1B
3389.308	-4354	1.40E-04	1896	0.333333	3.361194	1436564	UNC5C
4766.13	-4354	1.40E-04	1876	0.333333	3.361194	1843440	CCR6
3300.739	-4354	1.40E-04	1847	0.333333	3.361194	1388476	CYP17A1
3891.909	-4354	1.40E-04	1800	0.333333	3.361194	1492338	PPA1
3344.896	-4354	1.40E-04	1685	0.333333	3.361194	1409748	SLC27A6
3015.068	-4354	1.40E-04	1680	0.333333	3.361194	1392328	ELF4
3672.457	-4354	1.40E-04	1951	0.333333	3.360964	1453536	PLCG2
4019.929	-4354	1.40E-04	1849	0.333333	3.360964	1552386	COX10
4443.457	-4354	1.40E-04	1792	0.333333	3.360964	1631368	PERP
3241.517	-4354	1.40E-04	1728	0.333333	3.360964	1378824	APOC3

3073.255	-4354	1.40E-04	1677	0.333333	3.360964	1371922	LBP
3953.742	-4354	1.40E-04	1891	0.333333	3.360735	1560600	CHEK2
3742.055	-4354	1.40E-04	1891	0.333333	3.360735	1485396	CD8A
3175.914	-4354	1.40E-04	1765	0.333333	3.360735	1449008	SLC9A1
3318.925	-4354	1.40E-04	1727	0.333333	3.360735	1453448	MYLIP
3643.404	-4354	1.40E-04	1968	0.333333	3.360505	1450628	NR3C1
3482.671	-4354	1.40E-04	1851	0.333333	3.360505	1447446	ADCY7
3371.485	-4354	1.40E-04	1817	0.333333	3.360505	1404458	ARHGDIB
3284.531	-4354	1.40E-04	1750	0.333333	3.360505	1471914	CYTH3
3144.008	-4354	1.40E-04	1678	0.333333	3.360505	1435280	DOCK1
3707.97	-4354	1.40E-04	1677	0.333333	3.360505	1531112	SLC5A6
3606.787	-4354	1.40E-04	1624	0.333333	3.360505	1503078	TM7SF2
3178.999	-4354	1.40E-04	1969	0.333333	3.360276	1398980	FAS
3281.192	-4354	1.40E-04	1919	0.333333	3.360276	1452090	INSR
5224.754	-4354	1.40E-04	1831	0.333333	3.360276	2032480	PPP1R3C
3816.21	-4354	1.40E-04	1800	0.333333	3.360276	1521826	TAB1
3863.494	-4354	1.40E-04	1662	0.333333	3.360276	1489006	SCAP
3215.637	-4354	1.40E-04	1866	0.333333	3.360046	1423644	TBX3
3127.827	-4354	1.40E-04	1857	0.333333	3.360046	1411744	MYLK
3088.297	-4354	1.40E-04	1757	0.333333	3.360046	1430472	BIN1
3403.727	-4354	1.40E-04	1708	0.333333	3.360046	1418146	ABCG5
3426.76	-4354	1.40E-04	1677	0.333333	3.360046	1469794	SLC44A4
3451.194	-4354	1.40E-04	1625	0.333333	3.360046	1482506	CD24
5357.011	-4354	1.40E-04	1931	0.333333	3.359816	1574676	ATP5PF
3240.094	-4354	1.40E-04	1876	0.333333	3.359816	1439454	TLN2
3177.239	-4354	1.40E-04	1731	0.333333	3.359816	1425446	CAMK1
3319.584	-4354	1.40E-04	1659	0.333333	3.359816	1438906	CLC
3529.641	-4354	1.40E-04	1601	0.333333	3.359816	1477078	EFNA3
3497.529	-4354	1.40E-04	1876	0.333333	3.359587	1469264	PPP2R3A
3210.226	-4354	1.40E-04	1796	0.333333	3.359587	1375362	CCR8
3849.942	-4354	1.40E-04	1772	0.333333	3.359587	1528528	HHAT
3726.821	-4354	1.40E-04	1727	0.333333	3.359587	1496854	GCNT2
3609.889	-4354	1.40E-04	1706	0.333333	3.359587	1483852	ACSS2
4043.743	-4354	1.40E-04	1667	0.333333	3.359587	1548168	CLDN4
3277.218	-4354	1.40E-04	1633	0.333333	3.359587	1395948	SOAT2
4449.378	-4354	1.40E-04	1977	0.333333	3.359357	1599808	MDM2
3780.033	-4354	1.40E-04	1957	0.333333	3.359357	1469540	RAPGEF2
3236.476	-4354	1.40E-04	1877	0.333333	3.359357	1433272	TLE4
4205.929	-4354	1.40E-04	1662	0.333333	3.359357	1511716	MIF
2845.7	-4354	1.40E-04	1855	0.333333	3.359127	1377246	ITGA9
3649.669	-4354	1.40E-04	1723	0.333333	3.359127	1465028	CEL

3430.332	-4354	1.40E-04	1940	0.333333	3.358898	1441614	PIK3CG
3928.329	-4354	1.40E-04	1906	0.333333	3.358898	1472094	PSMC1
3304.097	-4354	1.40E-04	1654	0.333333	3.358898	1442572	CD19
3788.722	-4354	1.40E-04	1639	0.333333	3.358898	1426602	TUBA8
4030.735	-4354	1.40E-04	2262	0.333333	3.358668	1513624	TP53
3149.594	-4354	1.40E-04	1754	0.333333	3.358668	1409508	WNT5B
3699.968	-4354	1.40E-04	1677	0.333333	3.358668	1438742	ADRM1
3825.917	-4354	1.40E-04	1918	0.333333	3.358439	1459006	NRAS
2913.516	-4354	1.40E-04	1756	0.333333	3.358439	1309316	CHRM5
3425.099	-4354	1.40E-04	1727	0.333333	3.358439	1434916	TXNRD1
3459.495	-4354	1.40E-04	1664	0.333333	3.358439	1445366	TLE2
3707.88	-4354	1.40E-04	1645	0.333333	3.358439	1475938	SLC17A5
4208.238	-4354	1.40E-04	1907	0.333333	3.358209	1486644	RASA1
3243.824	-4354	1.40E-04	1889	0.333333	3.358209	1425896	EPHB1
3149.631	-4354	1.40E-04	1768	0.333333	3.358209	1405550	SOCS2
4125.186	-4354	1.40E-04	1996	0.333333	3.357979	1523630	CAMK2D
3397.845	-4354	1.40E-04	1911	0.333333	3.357979	1427012	FOXG1
3298.913	-4354	1.40E-04	1857	0.333333	3.357979	1419242	IL1RAP
3250.859	-4354	1.40E-04	1813	0.333333	3.357979	1423014	NLGN4X
3043.871	-4354	1.40E-04	1766	0.333333	3.357979	1379358	GNG11
3928.725	-4354	1.40E-04	1704	0.333333	3.357979	1515412	ATG4A
3694.843	-4354	1.40E-04	1670	0.333333	3.357979	1503146	IRAK4
3224.646	-4354	1.40E-04	1745	0.333333	3.35775	1382738	ARAP3
3469.657	-4354	1.40E-04	1624	0.333333	3.35775	1429674	PLA2G15
2854.455	-4354	1.40E-04	1654	0.333333	3.35752	1315844	INSRR
2942.223	-4354	1.40E-04	1635	0.333333	3.35752	1320316	FGF21
3604.286	-4354	1.40E-04	2167	0.333333	3.35729	1452114	YWHAZ
3228.707	-4354	1.40E-04	1886	0.333333	3.35729	1389424	LAMA1
3641.11	-4354	1.40E-04	1873	0.333333	3.35729	1464468	MAP3K1
3007.118	-4354	1.40E-04	1706	0.333333	3.35729	1358506	SEMA3E
3405.674	-4354	1.40E-04	1671	0.333333	3.35729	1443510	TEAD4
3098.401	-4354	1.40E-04	1657	0.333333	3.35729	1399544	RRAGD
3441.576	-4354	1.40E-04	1604	0.333333	3.35729	1415874	M6PR
3114.756	-4354	1.40E-04	1585	0.333333	3.35729	1346302	ANGPTL8
3757.651	-4354	1.40E-04	1935	0.333333	3.357061	1467526	CDKN1B
3165.995	-4354	1.40E-04	1870	0.333333	3.357061	1372648	EYA1
3235.572	-4354	1.40E-04	1855	0.333333	3.357061	1450792	PIK3R3
3094.876	-4354	1.40E-04	1784	0.333333	3.357061	1379148	IGF2R
4304.493	-4354	1.40E-04	1744	0.333333	3.357061	1551632	DROSHA
3584.018	-4354	1.40E-04	1717	0.333333	3.357061	1480756	P2RX5
3535.359	-4354	1.40E-04	1671	0.333333	3.357061	1427956	PMP2

2757.643	-4354	1.40E-04	1618	0.333333	3.357061	1359240	PTGES
3893.943	-4354	1.40E-04	1704	0.333333	3.356831	1519490	DVL1
3416.915	-4354	1.40E-04	1688	0.333333	3.356831	1452394	SESN1
3234.6	-4354	1.40E-04	1696	0.333333	3.356602	1350858	CYP2C19
4904.218	-4354	1.40E-04	1939	0.333333	3.356372	1577930	NDUFA9
3611.417	-4354	1.40E-04	1896	0.333333	3.356372	1415584	CACNA1E
3530.595	-4354	1.40E-04	1780	0.333333	3.356372	1438212	PTPRR
3058.004	-4354	1.40E-04	1755	0.333333	3.356372	1359256	IBSP
5318.954	-4354	1.40E-04	1696	0.333333	3.356372	1945672	ATG14
4262.656	-4354	1.40E-04	1929	0.333333	3.356142	1553776	NR2C2
3052.776	-4354	1.40E-04	1902	0.333333	3.356142	1361378	FOS
3457.077	-4354	1.40E-04	1748	0.333333	3.356142	1424366	SLC22A3
3361.584	-4354	1.40E-04	1665	0.333333	3.356142	1396052	NCEH1
3500.888	-4354	1.40E-04	1651	0.333333	3.355913	1473518	MACROH2A2
3039.762	-4354	1.40E-04	1622	0.333333	3.355913	1371644	SPRR1B
2986.902	-4354	1.40E-04	1807	0.333333	3.355683	1354070	CD59
2896.035	-4354	1.40E-04	1759	0.333333	3.355683	1327312	AOX1
3348.775	-4354	1.40E-04	1960	0.333333	3.355454	1394018	CHRM3
3539.53	-4354	1.40E-04	1917	0.333333	3.355454	1454984	NEDD4L
3721.107	-4354	1.40E-04	1706	0.333333	3.355454	1455268	DBI
3014.682	-4354	1.40E-04	1640	0.333333	3.355454	1319802	CACNA1H
3117.712	-4354	1.40E-04	1622	0.333333	3.355454	1360002	TRPV2
3683.372	-4354	1.40E-04	1975	0.333333	3.355224	1417702	ACTR3
2834.328	-4354	1.40E-04	1924	0.333333	3.355224	1353000	EPAS1
3532.092	-4354	1.40E-04	1879	0.333333	3.355224	1448606	PIP5K1B
3728.811	-4354	1.40E-04	1760	0.333333	3.355224	1488690	FABP4
3827.099	-4354	1.40E-04	1692	0.333333	3.355224	1488512	FABP6
3184.565	-4354	1.40E-04	1851	0.333333	3.354994	1369934	CDON
4562.848	-4354	1.40E-04	1826	0.333333	3.354994	1515666	ESR2
2858.546	-4354	1.40E-04	1795	0.333333	3.354994	1335628	NOTCH2
3031.25	-4354	1.40E-04	1944	0.333333	3.354765	1360368	PDGFRA
3336.758	-4354	1.40E-04	1773	0.333333	3.354765	1442402	NEO1
2700.902	-4354	1.40E-04	1739	0.333333	3.354765	1309446	IL13RA1
2914.529	-4354	1.40E-04	1722	0.333333	3.354765	1338400	LUM
2988.721	-4354	1.40E-04	1941	0.333333	3.354535	1394874	SMAD3
4311.84	-4354	1.40E-04	1842	0.333333	3.354535	1534082	ORC1
3060.843	-4354	1.40E-04	1738	0.333333	3.354535	1346938	IL1B
3188.994	-4354	1.40E-04	1744	0.333333	3.354305	1415066	CBS
3380.823	-4354	1.40E-04	1728	0.333333	3.354305	1375182	PDCD1LG2
3162.061	-4354	1.40E-04	1589	0.333333	3.354305	1371008	TFEB
3164.116	-4354	1.40E-04	1826	0.333333	3.354076	1418834	TLE1

2867.216	-4354	1.40E-04	1774	0.333333	3.354076	1336706	DOCK4
3222.922	-4354	1.40E-04	1659	0.333333	3.354076	1367014	SELPLG
2873.916	-4354	1.40E-04	1587	0.333333	3.354076	1334222	WIPI1
3309.676	-4354	1.40E-04	1794	0.333333	3.353846	1384086	GRK2
3831.132	-4354	1.40E-04	1762	0.333333	3.353846	1443540	MAP4K3
3292.417	-4354	1.40E-04	1655	0.333333	3.353846	1413846	NQO1
3245.225	-4354	1.40E-04	1648	0.333333	3.353846	1377002	CUBN
3005.12	-4354	1.40E-04	1862	0.333333	3.353617	1350644	LAMA3
3207.467	-4354	1.40E-04	1788	0.333333	3.353617	1405488	SCARB1
3480.972	-4354	1.40E-04	1667	0.333333	3.353617	1406936	BAK1
3418.214	-4354	1.40E-04	2168	0.333333	3.353387	1400834	SRC
3012.006	-4354	1.40E-04	1858	0.333333	3.353387	1379468	EPHB2
3579.241	-4354	1.40E-04	1834	0.333333	3.353387	1422694	TAOK3
3587.069	-4354	1.40E-04	1768	0.333333	3.353387	1389694	TBK1
3254.383	-4354	1.40E-04	1630	0.333333	3.353387	1403106	THBS3
2937.796	-4354	1.40E-04	1771	0.333333	3.353157	1335970	CAPN2
3184.179	-4354	1.40E-04	1919	0.333333	3.352928	1362476	SERPINA1
3002.02	-4354	1.40E-04	1913	0.333333	3.352928	1358452	CTNNA1
3294.715	-4354	1.40E-04	1805	0.333333	3.352928	1390222	SLC8A1
3999.792	-4354	1.40E-04	1653	0.333333	3.352928	1459686	FABP2
3521.984	-4354	1.40E-04	1616	0.333333	3.352928	1325456	TLX3
3422.948	-4354	1.40E-04	1587	0.333333	3.352928	1380094	PNPLA2
3046.649	-4354	1.40E-04	1570	0.333333	3.352928	1332670	POU5F1B
3509.906	-4354	1.39E-04	1886	0.333333	3.352698	1403834	RAF1
3161.746	-4354	1.39E-04	1676	0.333333	3.352698	1340462	LTB4R2
2954.449	-4354	1.39E-04	1860	0.333333	3.352468	1301774	CCR1
3250.83	-4354	1.39E-04	1834	0.333333	3.352468	1419140	ME3
3339.975	-4354	1.39E-04	1724	0.333333	3.352468	1405062	RHOD
2871.405	-4354	1.39E-04	1701	0.333333	3.352468	1288378	ICAM1
3279.033	-4354	1.39E-04	1670	0.333333	3.352468	1340890	CAB39
3239.545	-4354	1.39E-04	1656	0.333333	3.352468	1298400	POU3F4
3147.572	-4354	1.39E-04	1770	0.333333	3.352239	1367300	ITGAL
3544.438	-4354	1.39E-04	1729	0.333333	3.352239	1420504	XBP1
2902.91	-4354	1.39E-04	1750	0.333333	3.352009	1349226	ATP2B4
3930.001	-4354	1.39E-04	1724	0.333333	3.352009	1356398	SI
3678.914	-4354	1.39E-04	1723	0.333333	3.352009	1418268	GSTA4
3011.917	-4354	1.39E-04	1699	0.333333	3.352009	1354248	UNC5B
3517.304	-4354	1.39E-04	1615	0.333333	3.352009	1412348	PRCC
2953.747	-4354	1.39E-04	1804	0.333333	3.35178	1358010	FZD1
3162.139	-4354	1.39E-04	1788	0.333333	3.35178	1368608	ARG1
2762.407	-4354	1.39E-04	1738	0.333333	3.35178	1291230	PTGIR

3122.595	-4354	1.39E-04	1674	0.333333	3.35178	1340224	ITGA7
3106.327	-4354	1.39E-04	1612	0.333333	3.35178	1338890	IL36RN
3202.079	-4354	1.39E-04	1600	0.333333	3.35178	1350990	NRBF2
3318.643	-4354	1.39E-04	1585	0.333333	3.35178	1413702	HILPDA
2894.243	-4354	1.39E-04	1824	0.333333	3.35155	1286392	CCKBR
3014.858	-4354	1.39E-04	1802	0.333333	3.35155	1330354	KCNJ6
3923.077	-4354	1.39E-04	1778	0.333333	3.35155	1426928	MSH2
3831.459	-4354	1.39E-04	1623	0.333333	3.35155	1443178	UBE2S
3889.745	-4354	1.39E-04	1874	0.333333	3.35132	1418532	CHUK
2815.572	-4354	1.39E-04	1731	0.333333	3.35132	1308404	SERPINB1
3206.765	-4354	1.39E-04	1683	0.333333	3.35132	1326848	SLC2A3
3553.981	-4354	1.39E-04	2044	0.333333	3.351091	1404806	YWHAB
2637.29	-4354	1.39E-04	1788	0.333333	3.351091	1265634	IL1R1
2747.97	-4354	1.39E-04	1770	0.333333	3.351091	1289886	PECAM1
2903.247	-4354	1.39E-04	1730	0.333333	3.351091	1311398	MAOB
2937.05	-4354	1.39E-04	1625	0.333333	3.351091	1342906	CD200
3524.741	-4354	1.39E-04	1826	0.333333	3.350861	1435054	FARP2
2884.626	-4354	1.39E-04	1761	0.333333	3.350861	1326350	HGF
3550.459	-4354	1.39E-04	1708	0.333333	3.350861	1374750	PRICKLE2
3258.681	-4354	1.39E-04	1814	0.333333	3.350631	1394356	ITPR2
3308.293	-4354	1.39E-04	1792	0.333333	3.350631	1380846	GRK3
3020.509	-4354	1.39E-04	1782	0.333333	3.350631	1303298	CASP1
3070.933	-4354	1.39E-04	1742	0.333333	3.350631	1372962	PFKP
3574.875	-4354	1.39E-04	1692	0.333333	3.350631	1399656	RIPK3
3151.37	-4354	1.39E-04	1616	0.333333	3.350631	1266774	WNT8B
3786.472	-4354	1.39E-04	1892	0.333333	3.350402	1402272	PSMC5
2838.127	-4354	1.39E-04	1864	0.333333	3.350402	1329492	FOXO1
2932.921	-4354	1.39E-04	1849	0.333333	3.350402	1330774	ROR1
2935.974	-4354	1.39E-04	1765	0.333333	3.350402	1307592	SCTR
2939.018	-4354	1.39E-04	1735	0.333333	3.350402	1324078	EPHA7
3696.903	-4354	1.39E-04	1618	0.333333	3.350402	1422626	MEN1
3319.274	-4354	1.39E-04	1840	0.333333	3.350172	1408378	PTPN1
3806.216	-4354	1.39E-04	1805	0.333333	3.350172	1425956	MCM3
2716.239	-4354	1.39E-04	1609	0.333333	3.350172	1282148	TNFAIP6
3427.297	-4354	1.39E-04	1601	0.333333	3.350172	1391842	SLC4A2
3157.952	-4354	1.39E-04	1713	0.333333	3.349943	1336794	MYL2
3140.835	-4354	1.39E-04	1616	0.333333	3.349943	1369984	TICAM1
6055.105	-4354	1.39E-04	1602	0.333333	3.349943	2486938	EIF4EBP1
4014.938	-4354	1.39E-04	1756	0.333333	3.349713	1495386	TSC1
3559.705	-4354	1.39E-04	1733	0.333333	3.349713	1392974	HSD17B4
3621.324	-4354	1.39E-04	1576	0.333333	3.349713	1432968	TSPOAP1

3809.253	-4354	1.39E-04	1923	0.333333	3.349483	1392452	PSMC3
2929.434	-4354	1.39E-04	1810	0.333333	3.349483	1306250	NFKBIA
3431.373	-4354	1.39E-04	1733	0.333333	3.349483	1370720	SIX1
3339.197	-4354	1.39E-04	1687	0.333333	3.349483	1352334	MAP3K11
3296.975	-4354	1.39E-04	1622	0.333333	3.349483	1355544	INHBE
3179.95	-4354	1.39E-04	2005	0.333333	3.349254	1319602	RHOA
3187.07	-4354	1.39E-04	1908	0.333333	3.349254	1396744	LEF1
2833.091	-4354	1.39E-04	1813	0.333333	3.349254	1308076	INHBA
2824.633	-4354	1.39E-04	1766	0.333333	3.349254	1306142	LPAR3
2880.009	-4354	1.39E-04	1707	0.333333	3.349254	1298644	ENG
3263.422	-4354	1.39E-04	1789	0.333333	3.349024	1351872	SLC4A4
3565.387	-4354	1.39E-04	1741	0.333333	3.349024	1434804	MGMT
3161.022	-4354	1.39E-04	1673	0.333333	3.349024	1348826	GPC1
3215.512	-4354	1.39E-04	1631	0.333333	3.349024	1364398	MYL4
8739.427	-4354	1.39E-04	1625	0.333333	3.349024	3760708	DEFA6
3089.328	-4354	1.39E-04	1893	0.333333	3.348794	1354790	RET
3587.425	-4354	1.39E-04	1770	0.333333	3.348794	1376524	SEC13
3321.703	-4354	1.39E-04	1756	0.333333	3.348794	1358440	CYP39A1
3224.69	-4354	1.39E-04	1728	0.333333	3.348794	1368706	SMPD1
3080.16	-4354	1.39E-04	1587	0.333333	3.348794	1328428	IFITM1
3079.124	-4354	1.39E-04	1951	0.333333	3.348565	1362076	TPM1
3956.47	-4354	1.39E-04	1940	0.333333	3.348565	1432548	TBL1XR1
3109.18	-4354	1.39E-04	1870	0.333333	3.348565	1363970	STAT5B
3468.132	-4354	1.39E-04	1696	0.333333	3.348565	1416262	PDK1
3481.779	-4354	1.39E-04	1653	0.333333	3.348565	1347180	RPS6KA4
3123.545	-4354	1.39E-04	1650	0.333333	3.348565	1290420	GNG13
3400.142	-4354	1.39E-04	1621	0.333333	3.348565	1331322	FUCA1
3388.479	-4354	1.39E-04	1896	0.333333	3.348335	1352776	MAPK10
3006.724	-4354	1.39E-04	1757	0.333333	3.348335	1307324	TNFSF10
2741.36	-4354	1.39E-04	1709	0.333333	3.348335	1273892	ADAMTS5
2894.386	-4354	1.39E-04	1693	0.333333	3.348335	1307972	OPTN
3031.544	-4354	1.39E-04	1706	0.333333	3.348106	1300552	CYSLTR2
2486.591	-4354	1.39E-04	1689	0.333333	3.348106	1242506	THBD
3238.725	-4354	1.39E-04	1636	0.333333	3.348106	1338830	SCO2
2919.875	-4354	1.39E-04	1577	0.333333	3.348106	1298140	TNFSF14
2641.975	-4354	1.39E-04	1812	0.333333	3.347876	1261938	CD44
3478.779	-4354	1.39E-04	1712	0.333333	3.347876	1412362	HOMER1
3224.41	-4354	1.39E-04	1647	0.333333	3.347876	1346546	NAPA
3134.435	-4354	1.39E-04	1645	0.333333	3.347876	1290418	MYF5
2958.737	-4354	1.39E-04	1698	0.333333	3.347646	1287664	HSD3B1
3671.093	-4354	1.39E-04	1684	0.333333	3.347646	1370214	GSTT2

2897.426	-4354	1.39E-04	1607	0.333333	3.347646	1321470	TGIF1
3661.39	-4354	1.39E-04	1527	0.333333	3.347646	1400542	CRB3
3311.422	-4354	1.39E-04	1855	0.333333	3.347417	1338120	ANK2
3680.891	-4354	1.39E-04	1759	0.333333	3.347417	1385142	POU4F1
3634.487	-4354	1.39E-04	1751	0.333333	3.347417	1384020	KAT5
3140.511	-4354	1.39E-04	1645	0.333333	3.347417	1342392	RASSF1
3957.58	-4354	1.39E-04	1591	0.333333	3.347417	1466922	SCRIB
3030.791	-4354	1.39E-04	1675	0.333333	3.347187	1274298	LIPF
2986.556	-4354	1.39E-04	1639	0.333333	3.347187	1321244	ALOX15
3054.078	-4354	1.39E-04	1619	0.333333	3.347187	1334576	PMAIP1
2842.716	-4354	1.39E-04	1597	0.333333	3.347187	1330958	IL17RB
3759.808	-4354	1.39E-04	1826	0.333333	3.346958	1429300	MAP3K4
3200.055	-4354	1.39E-04	1612	0.333333	3.346958	1315112	CSH1
2800.794	-4354	1.39E-04	1902	0.333333	3.346728	1280610	DCN
2813.079	-4354	1.39E-04	1785	0.333333	3.346728	1288564	AXL
3283.764	-4354	1.39E-04	1726	0.333333	3.346728	1378178	ABCC4
3087.093	-4354	1.39E-04	1719	0.333333	3.346728	1353088	ALDH3A2
3333.026	-4354	1.39E-04	1664	0.333333	3.346728	1365942	PI4KA
3973.17	-4354	1.39E-04	1658	0.333333	3.346728	1346206	ACADL
3008.945	-4354	1.39E-04	1607	0.333333	3.346728	1354088	PDE3B
3185.569	-4354	1.39E-04	1586	0.333333	3.346728	1274564	IFNA2
3258.202	-4354	1.39E-04	1726	0.333333	3.346498	1360006	DNER
2796.856	-4354	1.39E-04	1558	0.333333	3.346498	1231022	DKK4
3300.365	-4354	1.39E-04	1788	0.333333	3.346039	1302176	APOH
3306.762	-4354	1.39E-04	1734	0.333333	3.346039	1370390	SLC44A1
3048.698	-4354	1.39E-04	1608	0.333333	3.346039	1314016	PPP1R15A
3686.095	-4354	1.39E-04	1568	0.333333	3.346039	1411406	RPIA
3322.866	-4354	1.39E-04	1791	0.333333	3.345809	1343218	RAP1A
2709.434	-4354	1.39E-04	1538	0.333333	3.345809	1264332	CD99
3222.157	-4354	1.39E-04	1836	0.333333	3.34558	1360540	IFNAR1
3235.691	-4354	1.39E-04	1753	0.333333	3.34558	1326632	CAPN1
3546.97	-4354	1.39E-04	1679	0.333333	3.34558	1400678	PF4V1
3024.24	-4354	1.39E-04	1753	0.333333	3.34535	1321772	EZR
3001.311	-4354	1.39E-04	1551	0.333333	3.34535	1304700	MCOLN1
3306.069	-4354	1.39E-04	1714	0.333333	3.345121	1364116	EML4
2973.494	-4354	1.39E-04	1846	0.333333	3.344891	1292164	ALOX5
3298.228	-4354	1.39E-04	1697	0.333333	3.344891	1360732	LMNB1
3499.665	-4354	1.39E-04	1687	0.333333	3.344891	1323874	TUBB4B
3356.415	-4354	1.39E-04	1826	0.333333	3.344661	1350186	MAPKAP1
3090.083	-4354	1.39E-04	1743	0.333333	3.344661	1338896	VAV3
2805.77	-4354	1.39E-04	1616	0.333333	3.344661	1268072	KLF2

2825.445	-4354	1.39E-04	1798	0.333333	3.344432	1273084	TGFB2
2998.882	-4354	1.39E-04	1691	0.333333	3.344432	1333242	PTPN7
3424.84	-4354	1.39E-04	1860	0.333333	3.344202	1337340	ITSN1
3102.589	-4354	1.39E-04	1860	0.333333	3.344202	1292696	PXN
2834.989	-4354	1.39E-04	1690	0.333333	3.344202	1281004	TIE1
2939.099	-4354	1.39E-04	1678	0.333333	3.344202	1302984	CALCOCO2
2908.722	-4354	1.39E-04	1739	0.333333	3.343972	1333778	ABL2
2713.593	-4354	1.39E-04	1684	0.333333	3.343972	1211190	F2RL3
3322.276	-4354	1.39E-04	1673	0.333333	3.343972	1344834	ATF4
2788.149	-4354	1.39E-04	1667	0.333333	3.343972	1267666	TBXAS1
3456.702	-4354	1.39E-04	1632	0.333333	3.343972	1341488	GLO1
3079.896	-4354	1.39E-04	1587	0.333333	3.343972	1313726	LRRC25
2918.846	-4354	1.39E-04	1714	0.333333	3.343743	1315786	PTPRJ
3551.466	-4354	1.39E-04	1599	0.333333	3.343513	1376210	MST1
3356.501	-4354	1.39E-04	1520	0.333333	3.343513	1298962	PLA2G4F
3102.653	-4354	1.39E-04	1795	0.333333	3.343284	1316834	RIPK1
2991.921	-4354	1.39E-04	1774	0.333333	3.343284	1309636	TLR3
2981.491	-4354	1.39E-04	1765	0.333333	3.343284	1335370	ANK1
2702.295	-4354	1.39E-04	1736	0.333333	3.343284	1267436	COL4A5
3312.279	-4354	1.39E-04	1734	0.333333	3.343284	1281150	APOA1
3910.81	-4354	1.39E-04	1707	0.333333	3.343284	1434956	MMACHC
2985.479	-4354	1.39E-04	1690	0.333333	3.343284	1243422	GUCY2F
2660.171	-4354	1.39E-04	1661	0.333333	3.343284	1238148	MMP13
2880.57	-4354	1.39E-04	1609	0.333333	3.343284	1293402	ROBO3
3025.639	-4354	1.39E-04	1839	0.333333	3.343054	1317908	PIK3CD
3303.684	-4354	1.39E-04	2056	0.333333	3.342824	1366934	SOS1
2800.367	-4354	1.39E-04	1760	0.333333	3.342824	1279204	TLN1
3321.786	-4354	1.39E-04	1647	0.333333	3.342824	1306280	CD69
2807.492	-4354	1.39E-04	1784	0.333333	3.342595	1251572	ITGAM
2821.632	-4354	1.39E-04	1692	0.333333	3.342595	1295142	TRAF1
3000.613	-4354	1.39E-04	1652	0.333333	3.342595	1284552	MYL12A
3177.547	-4354	1.39E-04	1519	0.333333	3.342595	1237218	PGK2
3675.819	-4354	1.39E-04	1904	0.333333	3.342365	1354052	PLCG1
3130.193	-4354	1.39E-04	1724	0.333333	3.342365	1306118	CXCL2
3634.883	-4354	1.39E-04	1695	0.333333	3.342365	1377516	NUMB
4243.064	-4354	1.39E-04	1792	0.333333	3.342135	1410030	HADH
2778.956	-4354	1.39E-04	1628	0.333333	3.342135	1267642	LTBR
2510.624	-4354	1.39E-04	1721	0.333333	3.341906	1208996	ENTPD1
2851.017	-4354	1.39E-04	1692	0.333333	3.341906	1301412	ETS1
3027.695	-4354	1.39E-04	1688	0.333333	3.341676	1328528	SSBP3
2946.58	-4354	1.39E-04	1663	0.333333	3.341676	1309112	ST3GAL4

3958.357	-4354	1.39E-04	1874	0.333333	3.341447	1389050	MCM2
2974.69	-4354	1.39E-04	1777	0.333333	3.341447	1272472	CD40
2929.498	-4354	1.39E-04	1733	0.333333	3.341447	1307728	MYO10
3465.502	-4354	1.39E-04	1728	0.333333	3.341447	1335452	CCNH
3478.919	-4354	1.39E-04	1629	0.333333	3.341447	1365586	IGBP1
2703.212	-4354	1.39E-04	1629	0.333333	3.341447	1227914	BDNF
2617.697	-4354	1.39E-04	1624	0.333333	3.341447	1247220	GPC4
3406.612	-4354	1.39E-04	1846	0.333333	3.341217	1302710	PSMD3
3091.393	-4354	1.39E-04	1738	0.333333	3.341217	1274042	LMBRD1
3726.978	-4354	1.39E-04	1947	0.333333	3.340987	1327448	PSMC6
2574.613	-4354	1.39E-04	1771	0.333333	3.340987	1224742	VWF
2625.302	-4354	1.38E-04	1736	0.333333	3.340758	1236442	SEMA5A
2830.08	-4354	1.38E-04	1702	0.333333	3.340758	1249266	FGB
3391.505	-4354	1.38E-04	1656	0.333333	3.340758	1286874	PKN1
2962.417	-4354	1.38E-04	1628	0.333333	3.340758	1286960	TBX15
2981.244	-4354	1.38E-04	1625	0.333333	3.340758	1286944	TNFSF8
2952.689	-4354	1.38E-04	1836	0.333333	3.340528	1279006	PRKCB
3723.16	-4354	1.38E-04	1769	0.333333	3.340528	1341218	LGR4
3015.009	-4354	1.38E-04	1730	0.333333	3.340528	1310804	INPP4B
3216.449	-4354	1.38E-04	1726	0.333333	3.340528	1311394	IKBKG
3574.987	-4354	1.38E-04	1703	0.333333	3.340528	1349110	DDX5
3487.67	-4354	1.38E-04	1605	0.333333	3.340528	1340484	LMNB2
8286.707	-4354	1.38E-04	1590	0.333333	3.340528	3662752	DEFA4
2550.811	-4354	1.38E-04	1556	0.333333	3.340528	1207712	SCPEP1
2593.406	-4354	1.38E-04	1749	0.333333	3.340299	1239354	LAMC2
3133.374	-4354	1.38E-04	1723	0.333333	3.340299	1311452	ATP6V0A1
2889.424	-4354	1.38E-04	1701	0.333333	3.340299	1266198	DOCK2
3176.674	-4354	1.38E-04	1774	0.333333	3.340069	1305634	ERG
2865.971	-4354	1.38E-04	1742	0.333333	3.340069	1241876	NCAM2
2981.514	-4354	1.38E-04	1706	0.333333	3.340069	1290922	ABHD5
3556.02	-4354	1.38E-04	1696	0.333333	3.340069	1320822	SACM1L
3338.863	-4354	1.38E-04	1650	0.333333	3.340069	1314650	HSD3B7
2958.804	-4354	1.38E-04	1821	0.333333	3.339839	1304520	VAV2
2893.95	-4354	1.38E-04	1795	0.333333	3.339839	1234426	GRM2
2961.274	-4354	1.38E-04	1748	0.333333	3.339839	1289204	REL
2886.643	-4354	1.38E-04	1669	0.333333	3.339839	1242912	BID
3352.727	-4354	1.38E-04	1561	0.333333	3.339839	1339130	LDB1
2763.424	-4354	1.38E-04	1798	0.333333	3.33961	1275132	ADCY9
3466.491	-4354	1.38E-04	1777	0.333333	3.33961	1389540	ATG16L1
3636.277	-4354	1.38E-04	1663	0.333333	3.33961	1381966	FOXM1
2419.362	-4354	1.38E-04	1535	0.333333	3.33961	1183442	TNFRSF11B

3856.326	-4354	1.38E-04	1789	0.333333	3.33938	1336706	OGDH
2539.523	-4354	1.38E-04	1731	0.333333	3.33938	1218296	CLU
3009.407	-4354	1.38E-04	1691	0.333333	3.33938	1301662	SLC39A8
3017.514	-4354	1.38E-04	1664	0.333333	3.33938	1268176	HSD17B6
3044.895	-4354	1.38E-04	1796	0.333333	3.33915	1283246	ARRB1
2548.537	-4354	1.38E-04	1720	0.333333	3.33915	1217878	ACKR3
3607.871	-4354	1.38E-04	1663	0.333333	3.33915	1313448	RASA3
2669.599	-4354	1.38E-04	1646	0.333333	3.33915	1218000	KCNJ16
3097.375	-4354	1.38E-04	1762	0.333333	3.338921	1293070	ACLY
3169.198	-4354	1.38E-04	1682	0.333333	3.338921	1319268	LPIN1
2827.969	-4354	1.38E-04	1679	0.333333	3.338921	1264286	CITED2
2698.528	-4354	1.38E-04	1579	0.333333	3.338921	1242154	CX3CR1
2694.144	-4354	1.38E-04	1760	0.333333	3.338691	1207092	CTSB
2895.857	-4354	1.38E-04	1738	0.333333	3.338691	1278548	WNT4
2615.768	-4354	1.38E-04	1639	0.333333	3.338691	1232230	PDK4
2666.681	-4354	1.38E-04	1610	0.333333	3.338691	1183736	CYP11B1
2993.302	-4354	1.38E-04	1598	0.333333	3.338691	1311018	CAB39L
2590.988	-4354	1.38E-04	1804	0.333333	3.338462	1206812	TNC
3530.312	-4354	1.38E-04	1787	0.333333	3.338462	1342274	TRAF2
2877.302	-4354	1.38E-04	1634	0.333333	3.338462	1291006	PPP2R5A
2794.957	-4354	1.38E-04	1577	0.333333	3.338462	1254138	JAG2
3038.301	-4354	1.38E-04	1786	0.333333	3.338232	1291176	CACNB2
2574.253	-4354	1.38E-04	1728	0.333333	3.338232	1219582	LTBP1
3248.357	-4354	1.38E-04	1613	0.333333	3.338232	1332552	CHKA
2850.506	-4354	1.38E-04	1611	0.333333	3.338232	1246554	ATP1B3
2814.341	-4354	1.38E-04	1592	0.333333	3.338232	1196708	PAX7
2714.594	-4354	1.38E-04	1586	0.333333	3.338232	1233722	DIRAS3
3292.538	-4354	1.38E-04	1507	0.333333	3.338232	1284080	BRMS1
3564.566	-4354	1.38E-04	1735	0.333333	3.338002	1342152	SEM1
2885.392	-4354	1.38E-04	1703	0.333333	3.338002	1232622	CYP26B1
2675.336	-4354	1.38E-04	1695	0.333333	3.338002	1204704	CDH19
3010.453	-4354	1.38E-04	1540	0.333333	3.338002	1241280	ERN2
2684.612	-4354	1.38E-04	1847	0.333333	3.337773	1218450	FLT1
2940.511	-4354	1.38E-04	1784	0.333333	3.337773	1256760	PRKAA2
2775.143	-4354	1.38E-04	1779	0.333333	3.337773	1250754	SLIT3
2924.366	-4354	1.38E-04	1770	0.333333	3.337773	1195428	LPAR1
2915.791	-4354	1.38E-04	1700	0.333333	3.337773	1233862	ESRRB
3174.61	-4354	1.38E-04	1694	0.333333	3.337773	1252976	PRMT8
2612.027	-4354	1.38E-04	1686	0.333333	3.337773	1194290	GREM2
3495.698	-4354	1.38E-04	1665	0.333333	3.337773	1318610	PIN1
3065.506	-4354	1.38E-04	1568	0.333333	3.337773	1236264	VAMP7

3131.169	-4354	1.38E-04	1561	0.333333	3.337773	1220172	ACSBG2
3323.039	-4354	1.38E-04	1555	0.333333	3.337773	1265472	MSTN
4916.597	-4354	1.38E-04	1893	0.333333	3.337543	1858970	PIK3C3
2477.784	-4354	1.38E-04	1829	0.333333	3.337543	1188176	FGF7
3051.344	-4354	1.38E-04	1753	0.333333	3.337543	1275068	BMP2K
3017.862	-4354	1.38E-04	1726	0.333333	3.337543	1296474	FOXO3
3130.043	-4354	1.38E-04	1607	0.333333	3.337543	1282380	PPP2R5B
3406.746	-4354	1.38E-04	1739	0.333333	3.337313	1282924	POU3F1
2533.609	-4354	1.38E-04	1665	0.333333	3.337313	1187538	C2
2581.075	-4354	1.38E-04	1656	0.333333	3.337313	1188040	CD74
2766.607	-4354	1.38E-04	1650	0.333333	3.337313	1199248	IGFBP1
3102.33	-4354	1.38E-04	1620	0.333333	3.337313	1265180	AGER
2792.646	-4354	1.38E-04	1603	0.333333	3.337313	1253398	SEMA4D
2720.975	-4354	1.38E-04	1560	0.333333	3.337313	1250918	CTSW
3531.87	-4354	1.38E-04	1538	0.333333	3.337313	1348678	C8G
2690.555	-4354	1.38E-04	1684	0.333333	3.337084	1211766	MMP14
2609.743	-4354	1.38E-04	1625	0.333333	3.337084	1232628	RASGRP3
2644.755	-4354	1.38E-04	1596	0.333333	3.337084	1244898	STS
3101.316	-4354	1.38E-04	1508	0.333333	3.337084	1257384	BAIAP3
3242.296	-4354	1.38E-04	1695	0.333333	3.336854	1249228	PSME1
2548.86	-4354	1.38E-04	1690	0.333333	3.336854	1192964	LRP1
12712.3	-4354	1.38E-04	1584	0.333333	3.336854	4961996	WT1
2855.139	-4354	1.38E-04	1564	0.333333	3.336854	1195502	SLCO1C1
2841.474	-4354	1.38E-04	1529	0.333333	3.336854	1188338	BARX1
3161.903	-4354	1.38E-04	1970	0.333333	3.336625	1272008	RELA
2733.11	-4354	1.38E-04	1757	0.333333	3.336625	1208178	ADRB1
2865.642	-4354	1.38E-04	1624	0.333333	3.336625	1225850	DEGS1
2679.125	-4354	1.38E-04	1600	0.333333	3.336625	1190806	ARPC2
3452.238	-4354	1.38E-04	1781	0.333333	3.336395	1298018	PRKACB
2762.982	-4354	1.38E-04	1721	0.333333	3.336395	1235044	PRKCH
2731.862	-4354	1.38E-04	1691	0.333333	3.336395	1189916	SFRP1
2593.411	-4354	1.38E-04	1643	0.333333	3.336395	1219988	GAD1
3008.255	-4354	1.38E-04	1613	0.333333	3.336395	1257628	HSD17B3
2946.174	-4354	1.38E-04	1594	0.333333	3.336395	1255088	NTN4
2931.095	-4354	1.38E-04	1537	0.333333	3.336395	1230256	TERT
2480.102	-4354	1.38E-04	1507	0.333333	3.336395	1221500	INPP5A
2786.789	-4354	1.38E-04	1704	0.333333	3.336165	1203648	KCNJ10
2651.399	-4354	1.38E-04	1679	0.333333	3.336165	1206906	SEMA6D
2896.853	-4354	1.38E-04	1655	0.333333	3.336165	1251932	LPAR2
3180.492	-4354	1.38E-04	1580	0.333333	3.336165	1275404	MAP4K2
2653.812	-4354	1.38E-04	1535	0.333333	3.336165	1185356	S100A7

2458.321	-4354	1.38E-04	1520	0.333333	3.336165	1183856	SLC19A3
2903.531	-4354	1.38E-04	1571	0.333333	3.335936	1158614	SLC22A8
3559.627	-4354	1.38E-04	1882	0.333333	3.335706	1300294	PCNA
3413.615	-4354	1.38E-04	1709	0.333333	3.335706	1308114	AP3B1
2647.488	-4354	1.38E-04	1631	0.333333	3.335706	1226752	ABCG1
2754.438	-4354	1.38E-04	1589	0.333333	3.335706	1238834	HKDC1
2744.853	-4354	1.38E-04	1558	0.333333	3.335706	1206012	OSM
3154.885	-4354	1.38E-04	1554	0.333333	3.335706	1234948	CNTF
2906.147	-4354	1.38E-04	1522	0.333333	3.335706	1207996	CYP4F8
3031.368	-4354	1.38E-04	1690	0.333333	3.335476	1267722	TFRC
2624.027	-4354	1.38E-04	1562	0.333333	3.335476	1203874	FCGR1A
2725.605	-4354	1.38E-04	1490	0.333333	3.335476	1236816	LFNG
2855.488	-4354	1.38E-04	1745	0.333333	3.335247	1225942	ALK
2526.33	-4354	1.38E-04	1668	0.333333	3.335247	1180848	LMOD1
3100.691	-4354	1.38E-04	1620	0.333333	3.335247	1263698	CXCL14
2654.971	-4354	1.38E-04	1612	0.333333	3.335247	1180466	LRP2
3209.6	-4354	1.38E-04	1585	0.333333	3.335247	1248814	TUBB3
3389.807	-4354	1.38E-04	1528	0.333333	3.335247	1330966	MED9
2700.213	-4354	1.38E-04	1617	0.333333	3.335017	1209162	RRAGC
3550.753	-4354	1.38E-04	1575	0.333333	3.335017	1331370	ANAPC15
3011.959	-4354	1.38E-04	1837	0.333333	3.334788	1266456	ERBB2
3437.415	-4354	1.38E-04	1882	0.333333	3.334558	1262680	PSMA4
2966.59	-4354	1.38E-04	1742	0.333333	3.334558	1241706	MAPKAPK2
2763.644	-4354	1.38E-04	1738	0.333333	3.334558	1248038	HES1
2883.687	-4354	1.38E-04	1685	0.333333	3.334558	1233392	LPGAT1
2825.966	-4354	1.38E-04	1609	0.333333	3.334558	1216990	SLCO1B3
3852.02	-4354	1.38E-04	1566	0.333333	3.334558	1312560	MINPP1
2976.526	-4354	1.38E-04	1556	0.333333	3.334558	1269186	SRD5A1
3118.464	-4354	1.38E-04	1524	0.333333	3.334558	1206144	TUBAL3
2829.665	-4354	1.38E-04	1467	0.333333	3.334558	1194508	PSAPL1
3513.384	-4354	1.38E-04	1854	0.333333	3.334328	1303462	MAPK9
3252.394	-4354	1.38E-04	1826	0.333333	3.334328	1272242	SMURF2
3052.329	-4354	1.38E-04	1790	0.333333	3.334328	1246096	TRDN
3074.675	-4354	1.38E-04	1722	0.333333	3.334328	1246862	CAT
2830.669	-4354	1.38E-04	1661	0.333333	3.334328	1195524	CD58
3098.451	-4354	1.38E-04	1570	0.333333	3.334328	1294040	LLGL2
2829.922	-4354	1.38E-04	1557	0.333333	3.334328	1222076	CALML5
3707.664	-4354	1.38E-04	1525	0.333333	3.334328	1369974	CLDN3
2662.355	-4354	1.38E-04	1712	0.333333	3.334099	1168056	CGA
2776.392	-4354	1.38E-04	1668	0.333333	3.334099	1239742	MPO
2975.1	-4354	1.38E-04	1535	0.333333	3.334099	1237118	VEGFB

2687.598	-4354	1.38E-04	1779	0.333333	3.333869	1220402	IFNAR2
2379.336	-4354	1.38E-04	1626	0.333333	3.333869	1189216	ETV5
2696.043	-4354	1.38E-04	1575	0.333333	3.333869	1191562	AP3B2
2782.233	-4354	1.38E-04	1525	0.333333	3.333639	1204012	CST1
2840.053	-4354	1.38E-04	1689	0.333333	3.33341	1186990	NOX5
2729.306	-4354	1.38E-04	1623	0.333333	3.33341	1194794	HLA-C
2960.411	-4354	1.38E-04	1570	0.333333	3.33341	1249892	HOMER2
3329.169	-4354	1.38E-04	1533	0.333333	3.33341	1274752	VPS4A
3093.962	-4354	1.38E-04	1762	0.333333	3.33318	1264990	MAGI1
3056.28	-4354	1.38E-04	1735	0.333333	3.33318	1255474	RAPGEF5
2868.442	-4354	1.38E-04	1723	0.333333	3.33318	1215562	FGA
2735.643	-4354	1.38E-04	1621	0.333333	3.33318	1202674	S100A8
3110.322	-4354	1.38E-04	1559	0.333333	3.33318	1234854	UBE2L6
2865.217	-4354	1.38E-04	1831	0.333333	3.332951	1214210	SNAP25
2481.09	-4354	1.38E-04	1793	0.333333	3.332951	1164088	EDNRA
3231.193	-4354	1.38E-04	1731	0.333333	3.332951	1256478	SOX2
3111.47	-4354	1.38E-04	1726	0.333333	3.332951	1231556	ARAF
2474.993	-4354	1.38E-04	1610	0.333333	3.332721	1163100	KCNK3
2675.87	-4354	1.38E-04	1565	0.333333	3.332721	1184194	C1QC
2516.097	-4354	1.38E-04	1486	0.333333	3.332721	1189224	MAP3K7CL
2663.611	-4354	1.38E-04	1720	0.333333	3.332491	1238208	WWC1
3835.289	-4354	1.38E-04	1708	0.333333	3.332491	1446382	HDAC9
4217.147	-4354	1.38E-04	1592	0.333333	3.332491	1327794	NDUFB5
2591.273	-4354	1.38E-04	1516	0.333333	3.332491	1173366	SMPDL3A
2618.988	-4354	1.38E-04	1784	0.333333	3.332262	1192534	CD36
3250.975	-4354	1.38E-04	1742	0.333333	3.332262	1281314	ADK
2784.186	-4354	1.38E-04	1616	0.333333	3.332262	1249696	ERC1
3224.78	-4354	1.38E-04	1525	0.333333	3.332262	1217572	TIGAR
2670.028	-4354	1.38E-04	1470	0.333333	3.332262	1144342	PDZD7
3313.501	-4354	1.38E-04	1704	0.333333	3.332032	1291040	HERC1
2763.04	-4354	1.38E-04	1647	0.333333	3.332032	1163964	GNGT1
3148.667	-4354	1.38E-04	1632	0.333333	3.332032	1245610	GCLM
3576.894	-4354	1.38E-04	1623	0.333333	3.332032	1318614	MECR
2867.095	-4354	1.38E-04	1757	0.333333	3.331803	1243592	DAAM1
3028.872	-4354	1.38E-04	1637	0.333333	3.331803	1257764	DTX1
3270.353	-4354	1.38E-04	1583	0.333333	3.331803	1203336	TRPC5
2644.598	-4354	1.38E-04	1590	0.333333	3.331573	1212188	MYO1C
3049.404	-4354	1.38E-04	1545	0.333333	3.331573	1256558	DHCR7
3452.348	-4354	1.38E-04	1537	0.333333	3.331573	1303142	RRAGB
2452.264	-4354	1.38E-04	1498	0.333333	3.331573	1079504	IL25
3221.408	-4354	1.38E-04	1553	0.333333	3.331343	1244208	PLA2G6

2570.994	-4354	1.38E-04	1538	0.333333	3.331343	1139936	VSIR
3022.364	-4354	1.38E-04	1463	0.333333	3.331343	1194124	PRPS1L1
2558.081	-4354	1.38E-04	1680	0.333333	3.331114	1196528	ASPH
3115.39	-4354	1.38E-04	1580	0.333333	3.331114	1232232	CHPT1
3358.925	-4354	1.38E-04	1544	0.333333	3.331114	1261758	GSTM2
2729.368	-4354	1.38E-04	1469	0.333333	3.331114	1155016	P2RX3
2499.734	-4354	1.38E-04	1723	0.333333	3.330884	1163560	COL6A2
2975.322	-4354	1.38E-04	1680	0.333333	3.330884	1227746	HACD2
2419.748	-4354	1.38E-04	1664	0.333333	3.330884	1159684	FCER1A
2552.269	-4354	1.38E-04	1586	0.333333	3.330884	1168202	IL18RAP
3080.124	-4354	1.38E-04	1482	0.333333	3.330884	1263500	NECTIN4
2749.803	-4354	1.38E-04	1477	0.333333	3.330884	1208554	ULBP2
3206.477	-4354	1.38E-04	1903	0.333333	3.330654	1233196	CUL1
3065.63	-4354	1.38E-04	1745	0.333333	3.330654	1217596	TSHR
2631.431	-4354	1.38E-04	1613	0.333333	3.330654	1190936	STC1
2508.769	-4354	1.38E-04	1554	0.333333	3.330654	1156900	SMAD9
2666.687	-4354	1.38E-04	1541	0.333333	3.330654	1202164	SEMA5B
2495.114	-4354	1.38E-04	1734	0.333333	3.330425	1138994	HRH1
3037.39	-4354	1.38E-04	1581	0.333333	3.330425	1246580	CCDC6
2710.322	-4354	1.38E-04	1580	0.333333	3.330425	1166668	ALPI
2708.133	-4354	1.38E-04	1764	0.333333	3.330195	1211224	CTBP2
2527.573	-4354	1.38E-04	1517	0.333333	3.330195	1134782	FTH1
2482.359	-4354	1.38E-04	1477	0.333333	3.330195	1134782	ALOX15B
2522.937	-4354	1.38E-04	1644	0.333333	3.329966	1185796	CEACAM1
3086.897	-4354	1.38E-04	1643	0.333333	3.329966	1229752	HMGCR
2590.806	-4354	1.38E-04	1571	0.333333	3.329966	1197686	ADGRG1
2772.944	-4354	1.38E-04	1554	0.333333	3.329966	1195810	ADCY4
2300.282	-4354	1.38E-04	1523	0.333333	3.329966	1138442	GUCY1A1
2558.833	-4354	1.38E-04	1508	0.333333	3.329966	1134052	S100A6
2661.12	-4354	1.38E-04	1473	0.333333	3.329966	1097984	S100G
3470.981	-4354	1.38E-04	1722	0.333333	3.329736	1260090	DNAJA1
3091.291	-4354	1.38E-04	1666	0.333333	3.329506	1260032	CLEC16A
2758.849	-4354	1.38E-04	1597	0.333333	3.329506	1195424	TMPRSS2
2830.297	-4354	1.38E-04	1591	0.333333	3.329506	1214494	HOXA10
2558.776	-4354	1.38E-04	1540	0.333333	3.329506	1129960	ZIC3
2722.883	-4354	1.38E-04	1707	0.333333	3.329277	1159210	RAC2
2538.942	-4354	1.38E-04	1619	0.333333	3.329277	1139512	BMP5
2645.497	-4354	1.38E-04	1521	0.333333	3.329277	1174216	MAFF
2456.275	-4354	1.38E-04	1507	0.333333	3.329277	1135674	COLEC11
2616.129	-4354	1.38E-04	1446	0.333333	3.329277	1149096	PEG3
2534.161	-4354	1.38E-04	1587	0.333333	3.329047	1133680	NR1H4

2998.207	-4354	1.38E-04	1475	0.333333	3.329047	1258960	EFNA4
2461.001	-4354	1.37E-04	1579	0.333333	3.328817	1117240	C1QB
3065.633	-4354	1.37E-04	1577	0.333333	3.328817	1209612	GP6
2614.683	-4354	1.37E-04	1546	0.333333	3.328817	1161612	APOC1
2572.395	-4354	1.37E-04	1489	0.333333	3.328817	1165566	ADAP1
2990.14	-4354	1.37E-04	1530	0.333333	3.328588	1239184	TCF7
2633.013	-4354	1.37E-04	1518	0.333333	3.328588	1109142	NOX3
2749.659	-4354	1.37E-04	1507	0.333333	3.328588	1096028	IFNW1
2532.874	-4354	1.37E-04	1608	0.333333	3.328358	1181760	FZD5
2661.227	-4354	1.37E-04	1536	0.333333	3.328358	1190770	PDP1
2852.664	-4354	1.37E-04	1502	0.333333	3.328358	1192400	AGPAT1
3091.629	-4354	1.37E-04	1477	0.333333	3.328358	1220106	TBC1D17
2713.521	-4354	1.37E-04	1545	0.333333	3.328129	1183486	CEACAM8
3317.041	-4354	1.37E-04	1520	0.333333	3.328129	1244938	CLDN16
2814.693	-4354	1.37E-04	1937	0.333333	3.327899	1216696	PTK2
3182.543	-4354	1.37E-04	1626	0.333333	3.327899	1240766	EIF2AK3
2735.329	-4354	1.37E-04	1601	0.333333	3.327899	1182606	IDH2
2725.054	-4354	1.37E-04	1565	0.333333	3.327899	1201294	CDKN2D
2600.35	-4354	1.37E-04	1679	0.333333	3.327669	1152368	CDH4
2772.134	-4354	1.37E-04	1700	0.333333	3.32744	1190228	SCARB2
2636.985	-4354	1.37E-04	1694	0.333333	3.32744	1184972	IDS
2435.382	-4354	1.37E-04	1660	0.333333	3.32744	1143704	PLXNA2
2961.699	-4354	1.37E-04	1899	0.333333	3.32721	1208132	VCP
2573.201	-4354	1.37E-04	1847	0.333333	3.32721	1153214	SHC1
2484.207	-4354	1.37E-04	1753	0.333333	3.32721	1135360	NRP1
2979.709	-4354	1.37E-04	1702	0.333333	3.32721	1187402	NTRK3
2160.857	-4354	1.37E-04	1615	0.333333	3.32721	1106126	SERPINE1
2171.111	-4354	1.37E-04	1591	0.333333	3.32721	1069634	CYP1B1
3049.923	-4354	1.37E-04	1551	0.333333	3.32721	1180220	SLC18A2
2679.035	-4354	1.37E-04	1543	0.333333	3.32721	1131418	SLCO1B1
2460.395	-4354	1.37E-04	1526	0.333333	3.32721	1104078	SOX10
3158.841	-4354	1.37E-04	1476	0.333333	3.32721	1170698	SLC25A31
2660.862	-4354	1.37E-04	1707	0.333333	3.32698	1178488	CXCR5
2688.296	-4354	1.37E-04	1667	0.333333	3.32698	1179100	AK5
2590.122	-4354	1.37E-04	1563	0.333333	3.32698	1167046	PLA2G4C
2259.855	-4354	1.37E-04	1554	0.333333	3.32698	1092242	TIMP3
2668.723	-4354	1.37E-04	1707	0.333333	3.326751	1170636	RBPJ
2700.989	-4354	1.37E-04	1698	0.333333	3.326751	1161010	LIFR
2915.514	-4354	1.37E-04	1683	0.333333	3.326751	1184878	FGFR4
2906.777	-4354	1.37E-04	1680	0.333333	3.326751	1218046	SRGAP3
2875.649	-4354	1.37E-04	1659	0.333333	3.326751	1207644	UBE2H

3399.335	-4354	1.37E-04	1656	0.333333	3.326751	1247660	CKS2
2580.145	-4354	1.37E-04	1619	0.333333	3.326751	1177094	DDAH1
2658.639	-4354	1.37E-04	1607	0.333333	3.326751	1101936	FGF4
2409.486	-4354	1.37E-04	1554	0.333333	3.326751	1124354	ANPEP
2541.064	-4354	1.37E-04	1738	0.333333	3.326521	1123312	PTGIS
3611.106	-4354	1.37E-04	1708	0.333333	3.326521	1288982	TTK
2862.567	-4354	1.37E-04	1658	0.333333	3.326521	1182340	WDFY3
2507.404	-4354	1.37E-04	1646	0.333333	3.326521	1125202	RAB8B
2566.904	-4354	1.37E-04	1558	0.333333	3.326521	1096662	KCNA4
3198.397	-4354	1.37E-04	1506	0.333333	3.326521	1265416	IPPK
3209.173	-4354	1.37E-04	1752	0.333333	3.326292	1233880	RPS6KA5
2649.157	-4354	1.37E-04	1687	0.333333	3.326292	1147270	TF
2764.131	-4354	1.37E-04	1532	0.333333	3.326292	1190654	DUSP2
2516.729	-4354	1.37E-04	1497	0.333333	3.326292	1122246	AIRE
3372.767	-4354	1.37E-04	1474	0.333333	3.326292	1346308	PLA2G1B
2624.108	-4354	1.37E-04	1704	0.333333	3.326062	1165956	IL2RA
2787.64	-4354	1.37E-04	1638	0.333333	3.326062	1197056	TOX
2473.536	-4354	1.37E-04	1633	0.333333	3.326062	1118776	CD86
2538.165	-4354	1.37E-04	1601	0.333333	3.326062	1163090	APBB1IP
3033.77	-4354	1.37E-04	1575	0.333333	3.326062	1163464	TAP1
2522.051	-4354	1.37E-04	1743	0.333333	3.325832	1116008	PTGER3
2689.002	-4354	1.37E-04	1676	0.333333	3.325832	1177852	NFATC1
2506.047	-4354	1.37E-04	1658	0.333333	3.325832	1112564	IRF1
3401.569	-4354	1.37E-04	1584	0.333333	3.325832	1251342	DIABLO
3119.252	-4354	1.37E-04	1493	0.333333	3.325832	1208030	PSENEN
3380.446	-4354	1.37E-04	1851	0.333333	3.325603	1265520	HMGB1
3149.25	-4354	1.37E-04	1783	0.333333	3.325603	1217090	ARHGEF12
3192.957	-4354	1.37E-04	1779	0.333333	3.325603	1227752	CSNK2A2
3041.365	-4354	1.37E-04	1517	0.333333	3.325373	1234724	PIAS3
2682.222	-4354	1.37E-04	1639	0.333333	3.325144	1171382	GNA11
2702.93	-4354	1.37E-04	1633	0.333333	3.325144	1183344	SOX4
2694.711	-4354	1.37E-04	1601	0.333333	3.325144	1159868	BNIP3L
2788.151	-4354	1.37E-04	1746	0.333333	3.324914	1170910	KITLG
2628.291	-4354	1.37E-04	1614	0.333333	3.324914	1133778	CYP3A5
2579.996	-4354	1.37E-04	1569	0.333333	3.324914	1162506	CTLA4
5527.744	-4354	1.37E-04	1489	0.333333	3.324914	1441238	MT-ND6
2497.272	-4354	1.37E-04	1642	0.333333	3.324684	1148670	THSD4
3278.039	-4354	1.37E-04	1568	0.333333	3.324684	1234652	MAD2L2
3091.234	-4354	1.37E-04	1561	0.333333	3.324684	1239848	MYL5
2513.507	-4354	1.37E-04	1544	0.333333	3.324684	1149192	ABCC1
2273.512	-4354	1.37E-04	1459	0.333333	3.324684	1102506	BEST1

2354.829	-4354	1.37E-04	1696	0.333333	3.324455	1107510	FGF1
2951.341	-4354	1.37E-04	1622	0.333333	3.324455	1170490	DIRAS2
2692.187	-4354	1.37E-04	1503	0.333333	3.324455	1145682	IL26
3183.441	-4354	1.37E-04	1628	0.333333	3.324225	1209536	NME1
2430.383	-4354	1.37E-04	1763	0.333333	3.323995	1084956	LCP2
2231.784	-4354	1.37E-04	1671	0.333333	3.323995	1107896	VCL
3390.546	-4354	1.37E-04	1476	0.333333	3.323995	1230450	ECI1
2466.853	-4354	1.37E-04	1455	0.333333	3.323995	1104472	DNASE2B
3869.263	-4354	1.37E-04	1745	0.333333	3.323766	1247142	HSP90AB1
2603.124	-4354	1.37E-04	1613	0.333333	3.323766	1156814	TRAM2
2860.157	-4354	1.37E-04	1578	0.333333	3.323766	1199544	TRIB3
2646.095	-4354	1.37E-04	1544	0.333333	3.323766	1130632	TKT
2931.135	-4354	1.37E-04	1686	0.333333	3.323536	1188254	MAML2
2383.773	-4354	1.37E-04	1669	0.333333	3.323536	1086132	GUCY1A2
3168.723	-4354	1.37E-04	1577	0.333333	3.323536	1262328	LRIG2
2568.297	-4354	1.37E-04	1660	0.333333	3.323307	1126814	ITGB8
2242.131	-4354	1.37E-04	1489	0.333333	3.323307	1063690	LITAF
2801.498	-4354	1.37E-04	1428	0.333333	3.323307	1055664	MGAM2
2862.562	-4354	1.37E-04	1714	0.333333	3.323077	1182356	MAPT
2208.693	-4354	1.37E-04	1578	0.333333	3.323077	1070646	ETS2
2508.682	-4354	1.37E-04	1463	0.333333	3.323077	1103862	MLKL
2584.847	-4354	1.37E-04	1630	0.333333	3.322847	1171270	PRKAG2
2376.92	-4354	1.37E-04	1573	0.333333	3.322847	1108814	ARHGEF40
2305.405	-4354	1.37E-04	1502	0.333333	3.322847	1077072	IHH
2728.76	-4354	1.37E-04	1471	0.333333	3.322847	1080320	AIPL1
3161.337	-4354	1.37E-04	1878	0.333333	3.322618	1222098	MAPK8
2656.298	-4354	1.37E-04	1669	0.333333	3.322618	1143456	PPP2R2B
2819.918	-4354	1.37E-04	1566	0.333333	3.322618	1164330	LRP5
2795.241	-4354	1.37E-04	1565	0.333333	3.322618	1191302	PRKAB2
3342.269	-4354	1.37E-04	1672	0.333333	3.322388	1243762	EZH2
3642.203	-4354	1.37E-04	1610	0.333333	3.322388	1171812	CYC1
2355.018	-4354	1.37E-04	1587	0.333333	3.322388	1077422	F2RL2
2342.042	-4354	1.37E-04	1445	0.333333	3.322388	1076210	STING1
3066.344	-4354	1.37E-04	1845	0.333333	3.322158	1195588	MAP3K7
3311.943	-4354	1.37E-04	1772	0.333333	3.322158	1168400	CD4
3151.351	-4354	1.37E-04	1702	0.333333	3.322158	1204326	AGPAT5
3051.789	-4354	1.37E-04	1615	0.333333	3.322158	1195960	IP6K3
3087.582	-4354	1.37E-04	1602	0.333333	3.322158	1189398	PRPS2
2899.533	-4354	1.37E-04	1590	0.333333	3.321929	1171570	FADD
2842.044	-4354	1.37E-04	1575	0.333333	3.321929	1199606	BCL11B
2973.851	-4354	1.37E-04	1542	0.333333	3.321929	1191810	BTD

2616.623	-4354	1.37E-04	1503	0.333333	3.321929	1121088	CLN3
2801.512	-4354	1.37E-04	1443	0.333333	3.321929	1098698	HPSE
3544.637	-4354	1.37E-04	1714	0.333333	3.321699	1252934	CDC7
2697.973	-4354	1.37E-04	1682	0.333333	3.321699	1155732	CLIP1
2641.785	-4354	1.37E-04	1630	0.333333	3.321699	1133234	ENPP1
2885.777	-4354	1.37E-04	1543	0.333333	3.321699	1172684	ALDH18A1
3260.699	-4354	1.37E-04	1624	0.333333	3.32147	1202950	WWP1
2562.52	-4354	1.37E-04	1611	0.333333	3.32147	1151644	PTPRF
2818.199	-4354	1.37E-04	1603	0.333333	3.32147	1143748	IDH1
2640.403	-4354	1.37E-04	1454	0.333333	3.32147	1112774	CYTH1
2846.456	-4354	1.37E-04	1730	0.333333	3.32124	1201404	MAP2K5
2347.938	-4354	1.37E-04	1668	0.333333	3.32124	1105754	EFNA5
3075.34	-4354	1.37E-04	1639	0.333333	3.32124	1137042	PSMB9
2845.451	-4354	1.37E-04	1630	0.333333	3.32124	1177800	MTMR3
2569.88	-4354	1.37E-04	1545	0.333333	3.32124	1122822	TNFSF15
2607.744	-4354	1.37E-04	1606	0.333333	3.32101	1124364	SOAT1
2594.617	-4354	1.37E-04	1582	0.333333	3.32101	1093786	APOA2
2498.033	-4354	1.37E-04	1548	0.333333	3.32101	1085100	SV2B
3893.977	-4354	1.37E-04	1547	0.333333	3.32101	1265718	NANOG
4919.589	-4354	1.37E-04	1487	0.333333	3.32101	1346592	MT-ND2
3116.921	-4354	1.37E-04	1676	0.333333	3.320781	1169616	ROCK2
3547.357	-4354	1.37E-04	1643	0.333333	3.320781	1341460	HDAC4
2946.703	-4354	1.37E-04	1600	0.333333	3.320781	1139720	VPS4B
2530.632	-4354	1.37E-04	1582	0.333333	3.320781	1152880	DEPTOR
2341.225	-4354	1.37E-04	1511	0.333333	3.320781	1076000	HSPA6
2454.976	-4354	1.37E-04	1461	0.333333	3.320781	1098590	TRPV6
3183.084	-4354	1.37E-04	1823	0.333333	3.320551	1160894	PSMA1
2979.157	-4354	1.37E-04	1603	0.333333	3.320551	1189234	SLC25A4
2600.614	-4354	1.37E-04	1563	0.333333	3.320551	1157470	TPM3
2139.849	-4354	1.37E-04	1553	0.333333	3.320551	1046682	FST
3469.278	-4354	1.37E-04	1511	0.333333	3.320551	1260506	COQ10A
2619.872	-4354	1.37E-04	1660	0.333333	3.320321	1163212	GNG7
2861.845	-4354	1.37E-04	1459	0.333333	3.320321	1140892	RASAL1
2567.68	-4354	1.37E-04	1729	0.333333	3.320092	1099912	PLG
2514.736	-4354	1.37E-04	1532	0.333333	3.320092	1128382	GDF15
2430.741	-4354	1.37E-04	1526	0.333333	3.320092	1086492	MAP1LC3B
2689.86	-4354	1.37E-04	1507	0.333333	3.320092	1121958	CFL1
2966.784	-4354	1.37E-04	1733	0.333333	3.319862	1131378	ADRA1D
2703.53	-4354	1.37E-04	1653	0.333333	3.319862	1167028	WWP2
3188.104	-4354	1.37E-04	1629	0.333333	3.319862	1220764	UBE2C
2940.097	-4354	1.37E-04	1611	0.333333	3.319862	1191482	SOCS7

2873.587	-4354	1.37E-04	1581	0.333333	3.319862	1150370	MTM1
2551.419	-4354	1.37E-04	1573	0.333333	3.319862	1134294	EGLN1
2657.207	-4354	1.37E-04	1448	0.333333	3.319862	1156808	PARP3
2472.734	-4354	1.37E-04	1694	0.333333	3.319633	1103690	RUNX1T1
2559.935	-4354	1.37E-04	1682	0.333333	3.319633	1117458	SQSTM1
2551.005	-4354	1.37E-04	1596	0.333333	3.319633	1097398	CCND3
3161.071	-4354	1.37E-04	1553	0.333333	3.319633	1172336	TLR9
2381.195	-4354	1.37E-04	1474	0.333333	3.319633	1118312	SORT1
2231.138	-4354	1.37E-04	1450	0.333333	3.319633	1080452	HOMER3
3418.241	-4354	1.37E-04	1620	0.333333	3.319403	1217528	VHL
2585.953	-4354	1.37E-04	1598	0.333333	3.319403	1101558	WAS
2724.551	-4354	1.37E-04	1581	0.333333	3.319403	1136070	MAPK12
2764.869	-4354	1.37E-04	1668	0.333333	3.319173	1141144	ENAH
2968.117	-4354	1.37E-04	1597	0.333333	3.319173	1119292	STUB1
2852.384	-4354	1.37E-04	1521	0.333333	3.319173	1140284	ATG101
2653.267	-4354	1.37E-04	1520	0.333333	3.319173	1133388	LAPTM4B
2409.506	-4354	1.37E-04	1507	0.333333	3.319173	1105320	LIMK2
2445.435	-4354	1.37E-04	1444	0.333333	3.319173	1094034	IL36G
2640.09	-4354	1.37E-04	1652	0.333333	3.318944	1112080	KCNJ3
2040.25	-4354	1.37E-04	1570	0.333333	3.318944	1037476	CCN1
2841.892	-4354	1.37E-04	1510	0.333333	3.318944	1118456	MAPK8IP2
2967.339	-4354	1.37E-04	1708	0.333333	3.318714	1197692	MTOR
2812.995	-4354	1.37E-04	1548	0.333333	3.318714	1138006	PLXNB1
2834.634	-4354	1.37E-04	1502	0.333333	3.318714	1166752	LDHC
2469.382	-4354	1.37E-04	1494	0.333333	3.318714	1070510	NR5A1
2796.986	-4354	1.37E-04	1814	0.333333	3.318485	1152124	CASP8
2277.561	-4354	1.37E-04	1770	0.333333	3.318485	1068608	TEK
2468.178	-4354	1.37E-04	1647	0.333333	3.318485	1059842	ADH1B
2292.757	-4354	1.37E-04	1478	0.333333	3.318485	1063506	LILRB4
2304.196	-4354	1.37E-04	1715	0.333333	3.318255	1053810	FPR1
2930.531	-4354	1.37E-04	1638	0.333333	3.318255	1166146	SS18
3025.357	-4354	1.37E-04	1503	0.333333	3.318255	1213440	FABP3
2753.051	-4354	1.37E-04	1856	0.333333	3.318025	1148340	TRAF6
2729.845	-4354	1.37E-04	1659	0.333333	3.318025	1100432	GABBR2
2978.649	-4354	1.37E-04	1532	0.333333	3.318025	1155688	GSTT1
2742.073	-4354	1.37E-04	1656	0.333333	3.317796	1139952	HIPK1
2756.003	-4354	1.37E-04	1633	0.333333	3.317796	1102180	AQP4
2476.498	-4354	1.37E-04	1651	0.333333	3.317566	1111478	GNAL
3402.985	-4354	1.37E-04	1638	0.333333	3.317336	1275138	MEF2A
2839.797	-4354	1.37E-04	1539	0.333333	3.317336	1147698	INPP5F
2210.6	-4354	1.37E-04	1477	0.333333	3.317336	1027784	LILRB2

2331.386	-4354	1.37E-04	1427	0.333333	3.317336	1099474	KLF3
2981.813	-4354	1.37E-04	1904	0.333333	3.317107	1157210	EP300
2687.655	-4354	1.37E-04	1751	0.333333	3.316877	1109204	PTEN
3125.397	-4354	1.37E-04	1691	0.333333	3.316877	1162198	CLTA
3169.029	-4354	1.37E-04	1679	0.333333	3.316877	1145250	BMP7
2722.231	-4354	1.37E-04	1660	0.333333	3.316877	1136706	HIPK3
3000.221	-4354	1.37E-04	1644	0.333333	3.316877	1146574	CSNK1G2
2595.569	-4354	1.37E-04	1398	0.333333	3.316877	1078722	FSCN2
3208.405	-4354	1.37E-04	1525	0.333333	3.316648	1177084	LATS1
2572.341	-4354	1.37E-04	1510	0.333333	3.316648	1120592	FADS2
2933.671	-4354	1.36E-04	1697	0.333333	3.316418	1153332	CDK9
2511.673	-4354	1.36E-04	1607	0.333333	3.316418	1088100	FGFR3
2292.222	-4354	1.36E-04	1562	0.333333	3.316418	1090014	FOSB
2097.629	-4354	1.36E-04	1548	0.333333	3.316418	1030480	TFPI
2641.145	-4354	1.36E-04	1542	0.333333	3.316418	1099972	CLIC5
3065.883	-4354	1.36E-04	1524	0.333333	3.316418	1180560	HESX1
2617.858	-4354	1.36E-04	1571	0.333333	3.316188	1103188	CA8
2566.67	-4354	1.36E-04	1529	0.333333	3.316188	1024338	HOXB1
2961.566	-4354	1.36E-04	1482	0.333333	3.316188	1146940	ITGAE
3743.976	-4354	1.36E-04	1753	0.333333	3.315959	1164130	ATP5F1A
3537.381	-4354	1.36E-04	1662	0.333333	3.315959	1174148	YWHAQ
2900.267	-4354	1.36E-04	1862	0.333333	3.315729	1146168	CSNK2A1
3046.156	-4354	1.36E-04	1566	0.333333	3.315729	1144906	RUVBL1
2426.918	-4354	1.36E-04	1531	0.333333	3.315729	1076644	FLOT2
2417.022	-4354	1.36E-04	1482	0.333333	3.315729	1098924	P2RX7
2785.126	-4354	1.36E-04	1703	0.333333	3.315499	1109670	PRKACA
3396.56	-4354	1.36E-04	1638	0.333333	3.31527	1153340	COX6B1
2080.194	-4354	1.36E-04	1623	0.333333	3.31527	987642	HLA-E
2816.849	-4354	1.36E-04	1433	0.333333	3.31527	1117084	CENPB
2268.808	-4354	1.36E-04	1662	0.333333	3.31504	1066048	EGR1
2456.78	-4354	1.36E-04	1521	0.333333	3.31504	1080860	IRF7
2630.678	-4354	1.36E-04	1483	0.333333	3.31504	1122112	TJP3
2758.142	-4354	1.36E-04	1657	0.333333	3.314811	1127780	SC5D
2273.576	-4354	1.36E-04	1719	0.333333	3.314581	1030436	AVPR1A
3041.675	-4354	1.36E-04	1658	0.333333	3.314581	1141544	GFPT1
2217.289	-4354	1.36E-04	1633	0.333333	3.314581	1032930	RHOQ
2838.306	-4354	1.36E-04	1566	0.333333	3.314581	1148460	TRAF5
2357.488	-4354	1.36E-04	1543	0.333333	3.314581	1066882	CA12
2488.594	-4354	1.36E-04	1535	0.333333	3.314581	1080872	FRMD6
2188.029	-4354	1.36E-04	1490	0.333333	3.314581	1045174	H6PD
2556.166	-4354	1.36E-04	1467	0.333333	3.314581	1095818	SLC39A14

2799.782	-4354	1.36E-04	1662	0.333333	3.314351	1105418	B2M
2601.987	-4354	1.36E-04	1617	0.333333	3.314351	1110112	GNA13
2587.97	-4354	1.36E-04	1478	0.333333	3.314351	1080396	ACAA1
2678.252	-4354	1.36E-04	1473	0.333333	3.314351	1039170	NAGA
2958.684	-4354	1.36E-04	1632	0.333333	3.314122	1131094	ACAT1
2520.597	-4354	1.36E-04	1592	0.333333	3.314122	1080434	ZEB1
2596.986	-4354	1.36E-04	1537	0.333333	3.314122	1105424	NR4A2
2133.392	-4354	1.36E-04	1527	0.333333	3.314122	1029310	TGM2
3079.522	-4354	1.36E-04	1761	0.333333	3.313892	1153536	CLTC
2445.479	-4354	1.36E-04	1584	0.333333	3.313892	1066200	PDIA3
2591.585	-4354	1.36E-04	1567	0.333333	3.313892	1077072	IL1A
2174.708	-4354	1.36E-04	1503	0.333333	3.313892	1041102	GAS1
2470.513	-4354	1.36E-04	1473	0.333333	3.313892	1068698	RALGDS
2148.222	-4354	1.36E-04	1468	0.333333	3.313892	1023578	CSF2RA
2164.731	-4354	1.36E-04	1580	0.333333	3.313662	1046150	MYL9
2598.258	-4354	1.36E-04	1579	0.333333	3.313662	1036952	CHRM4
2686.399	-4354	1.36E-04	1472	0.333333	3.313662	1119454	LGR6
2811.499	-4354	1.36E-04	1426	0.333333	3.313662	1142720	RBKS
2521.32	-4354	1.36E-04	1590	0.333333	3.313433	1092446	SIPA1L2
2308.193	-4354	1.36E-04	1420	0.333333	3.313433	1027544	GAPDHS
2277.786	-4354	1.36E-04	1725	0.333333	3.313203	1033248	LAMA4
2262.688	-4354	1.36E-04	1523	0.333333	3.313203	1052288	SSH1
2473.644	-4354	1.36E-04	1511	0.333333	3.313203	1056824	ME2
2752.816	-4354	1.36E-04	1491	0.333333	3.313203	1104264	CCL4
3059.191	-4354	1.36E-04	1520	0.333333	3.312974	1108470	MAPKAPK5
2489.377	-4354	1.36E-04	1506	0.333333	3.312974	1079028	CD79B
2348.954	-4354	1.36E-04	1425	0.333333	3.312974	1070538	NPC1
2569.604	-4354	1.36E-04	1605	0.333333	3.312744	1072266	EPHA5
2504.569	-4354	1.36E-04	1509	0.333333	3.312744	1021780	IL5
3108.302	-4354	1.36E-04	1499	0.333333	3.312744	1121934	ANAPC2
2281.904	-4354	1.36E-04	1497	0.333333	3.312744	1053984	PDGFA
2307.41	-4354	1.36E-04	1471	0.333333	3.312744	1036688	SYN1
2757.797	-4354	1.36E-04	1460	0.333333	3.312744	1050084	PFKFB1
2722.027	-4354	1.36E-04	1736	0.333333	3.312514	1099824	PAK2
3102.679	-4354	1.36E-04	1675	0.333333	3.312514	1087576	PSMB5
3162.538	-4354	1.36E-04	1808	0.333333	3.312285	1114592	PSMA5
2347.291	-4354	1.36E-04	1574	0.333333	3.312285	1070156	PPP1R12B
2629.161	-4354	1.36E-04	1552	0.333333	3.312285	1060490	UBA1
2039.598	-4354	1.36E-04	1493	0.333333	3.312285	987996	LAMC3
2635.6	-4354	1.36E-04	1531	0.333333	3.312055	1095564	UBE2Z
2653.23	-4354	1.36E-04	1477	0.333333	3.312055	1109582	SLC44A3

2408.143	-4354	1.36E-04	1430	0.333333	3.312055	1077698	LAG3
2641.791	-4354	1.36E-04	1420	0.333333	3.312055	1035582	CYP8B1
2420.428	-4354	1.36E-04	1416	0.333333	3.312055	1048732	SGSH
2508.655	-4354	1.36E-04	1527	0.333333	3.311825	1102368	TNFRSF17
2403.887	-4354	1.36E-04	1492	0.333333	3.311825	1032678	LPAR6
2091.159	-4354	1.36E-04	1432	0.333333	3.311825	982532	SULT1E1
2246.67	-4354	1.36E-04	1422	0.333333	3.311825	1012308	ATP2A1
2611.389	-4354	1.36E-04	1601	0.333333	3.311596	1080762	SNAP23
2906.726	-4354	1.36E-04	1568	0.333333	3.311596	1114148	SIRT1
2203.296	-4354	1.36E-04	1511	0.333333	3.311596	1020960	ZFP36L2
2156.194	-4354	1.36E-04	1426	0.333333	3.311596	1034664	TLR6
2441.209	-4354	1.36E-04	1811	0.333333	3.311366	1044466	JAK2
2771.03	-4354	1.36E-04	1658	0.333333	3.311366	1128884	PATJ
2301.598	-4354	1.36E-04	1526	0.333333	3.311366	1024662	CFB
1985.686	-4354	1.36E-04	1520	0.333333	3.311366	976008	C7
2344.763	-4354	1.36E-04	1474	0.333333	3.311366	1068556	PCYT1A
2739.168	-4354	1.36E-04	1721	0.333333	3.311137	1054838	CAMK2A
2112.632	-4354	1.36E-04	1559	0.333333	3.311137	999126	PDE1A
2946.034	-4354	1.36E-04	1487	0.333333	3.311137	1116302	MAP1LC3A
2403.618	-4354	1.36E-04	1576	0.333333	3.310907	1070884	IL16
2396.663	-4354	1.36E-04	1493	0.333333	3.310907	1059388	MTMR14
2281.549	-4354	1.36E-04	1454	0.333333	3.310907	1038072	EREG
2717.01	-4354	1.36E-04	1682	0.333333	3.310677	1049800	PSMD8
2325.541	-4354	1.36E-04	1572	0.333333	3.310677	1028688	POSTN
2355.047	-4354	1.36E-04	1541	0.333333	3.310677	1039650	PIK3AP1
2068.319	-4354	1.36E-04	1531	0.333333	3.310677	1018980	GRB10
2616.592	-4354	1.36E-04	1453	0.333333	3.310677	1023826	IL19
2514.793	-4354	1.36E-04	1446	0.333333	3.310677	1056824	DGKQ
3993.35	-4354	1.36E-04	1659	0.333333	3.310448	1641448	BCL2
2851.447	-4354	1.36E-04	1608	0.333333	3.310448	1103480	SPTAN1
2229.931	-4354	1.36E-04	1546	0.333333	3.310448	1021780	PTGER4
2694.96	-4354	1.36E-04	1542	0.333333	3.310448	1059122	SEC61A1
2485.137	-4354	1.36E-04	1528	0.333333	3.310448	1029108	INPP5D
2266.745	-4354	1.36E-04	1513	0.333333	3.310448	1038566	SEPTIN4
2613.616	-4354	1.36E-04	1622	0.333333	3.310218	1086262	TRAF3
2356.099	-4354	1.36E-04	1520	0.333333	3.310218	1011946	PGR
4010.432	-4354	1.36E-04	1672	0.333333	3.309989	1188092	NDUFA8
2694.661	-4354	1.36E-04	1594	0.333333	3.309759	1123976	SUPT3H
2669.581	-4354	1.36E-04	1567	0.333333	3.309759	1090168	GCLC
2319.202	-4354	1.36E-04	1525	0.333333	3.309759	1017590	PICALM
2656.05	-4354	1.36E-04	1514	0.333333	3.309759	1090116	GNGT2

2415.134	-4354	1.36E-04	1509	0.333333	3.309759	1047302	GZMK
2014.92	-4354	1.36E-04	1457	0.333333	3.309759	988704	NOTCH4
2105.25	-4354	1.36E-04	1452	0.333333	3.309759	1009042	TLE3
2674.55	-4354	1.36E-04	1573	0.333333	3.309529	1087164	ACSL3
2320.748	-4354	1.36E-04	1455	0.333333	3.309529	1037054	PRF1
2120.182	-4354	1.36E-04	1418	0.333333	3.309529	985270	NLGN3
3549.256	-4354	1.36E-04	1576	0.333333	3.3093	1138472	CFTR
2683.191	-4354	1.36E-04	1546	0.333333	3.3093	1096346	SLC20A2
2541.899	-4354	1.36E-04	1450	0.333333	3.3093	1026582	LHX2
2745.18	-4354	1.36E-04	1407	0.333333	3.3093	1089714	BRD9
2743.239	-4354	1.36E-04	1690	0.333333	3.30907	1097238	ARNT
2877.722	-4354	1.36E-04	1616	0.333333	3.30907	1047422	PSMB6
2366.649	-4354	1.36E-04	1541	0.333333	3.30907	1080526	TRAF3IP2
2410.393	-4354	1.36E-04	1485	0.333333	3.30907	1028400	PLXNB2
2451.355	-4354	1.36E-04	1398	0.333333	3.30907	1052024	STARD3
2514.218	-4354	1.36E-04	1387	0.333333	3.30907	1088352	LRRC4
2466.937	-4354	1.36E-04	1712	0.333333	3.30884	1027194	GNAO1
2327.889	-4354	1.36E-04	1488	0.333333	3.30884	1014668	CYP2C18
2189.363	-4354	1.36E-04	1478	0.333333	3.30884	1029372	YBX3
2407.915	-4354	1.36E-04	1360	0.333333	3.30884	1049344	TCL1B
2403.423	-4354	1.36E-04	1651	0.333333	3.308611	1035370	IL6ST
2426.675	-4354	1.36E-04	1640	0.333333	3.308611	1055768	GAB1
2463.517	-4354	1.36E-04	1476	0.333333	3.308611	1002670	POU1F1
1965.709	-4354	1.36E-04	1427	0.333333	3.308611	974412	LILRA2
2379.245	-4354	1.36E-04	1421	0.333333	3.308611	1029382	DNASE2
2462.618	-4354	1.36E-04	1575	0.333333	3.308381	1049744	PTCH1
2421.047	-4354	1.36E-04	1537	0.333333	3.308381	1026886	CACNA1D
2325.841	-4354	1.36E-04	1480	0.333333	3.308381	1020652	GZMB
2218.769	-4354	1.36E-04	1476	0.333333	3.308381	1028240	EPHB4
2663.587	-4354	1.36E-04	1550	0.333333	3.308152	1103740	ELOA
2484.301	-4354	1.36E-04	1525	0.333333	3.308152	1017272	TGFB1
2672.564	-4354	1.36E-04	1498	0.333333	3.308152	1059362	ITPR3
2421.764	-4354	1.36E-04	1453	0.333333	3.308152	1039040	GALNS
2093.344	-4354	1.36E-04	1595	0.333333	3.307922	1004500	ITGB5
2359.865	-4354	1.36E-04	1574	0.333333	3.307922	1002442	MEOX2
2300.086	-4354	1.36E-04	1486	0.333333	3.307922	1006848	PLK3
2252.113	-4354	1.36E-04	1458	0.333333	3.307922	976436	PHKG1
1984.231	-4354	1.36E-04	1417	0.333333	3.307922	971966	SOX17
2065.779	-4354	1.36E-04	1404	0.333333	3.307922	991604	PHEX
2721.134	-4354	1.36E-04	1627	0.333333	3.307692	1088944	NEGR1
3201.03	-4354	1.36E-04	1612	0.333333	3.307692	1133474	ARF1

2571.884	-4354	1.36E-04	1576	0.333333	3.307692	1063882	PPP2CB
2344.539	-4354	1.36E-04	1393	0.333333	3.307692	1027656	SLC44A2
2128.243	-4354	1.36E-04	1672	0.333333	3.307463	996892	ITGB3
2359.635	-4354	1.36E-04	1659	0.333333	3.307463	1002934	CRK
2216.456	-4354	1.36E-04	1605	0.333333	3.307463	1016218	BMP6
2294.842	-4354	1.36E-04	1531	0.333333	3.307463	1041972	TOLLIP
2989.382	-4354	1.36E-04	1463	0.333333	3.307463	1071558	GUSB
2385.199	-4354	1.36E-04	1440	0.333333	3.307463	1020080	GNAT2
2739.112	-4354	1.36E-04	1579	0.333333	3.307233	1098746	WWOX
2457.925	-4354	1.36E-04	1538	0.333333	3.307233	1034918	RALA
2034.771	-4354	1.36E-04	1452	0.333333	3.307003	939276	SFRP4
2224.889	-4354	1.36E-04	1445	0.333333	3.307003	981864	TNFSF13B
2677.853	-4354	1.36E-04	1556	0.333333	3.306774	1075796	ATP6V1H
2306.957	-4354	1.36E-04	1537	0.333333	3.306774	1046778	PSEN2
2094.829	-4354	1.36E-04	1399	0.333333	3.306774	981278	ALDH3B1
2386.352	-4354	1.36E-04	1394	0.333333	3.306774	1047228	TBL1Y
2472.516	-4354	1.36E-04	1543	0.333333	3.306315	1029128	BACH1
2451.986	-4354	1.36E-04	1487	0.333333	3.306315	1033972	CCL18
2431.114	-4354	1.36E-04	1477	0.333333	3.306315	977914	POU3F2
2452.971	-4354	1.36E-04	1348	0.333333	3.306315	993266	NOTUM
3435.88	-4354	1.36E-04	1453	0.333333	3.306085	1163968	TTI1
2862.215	-4354	1.36E-04	1422	0.333333	3.306085	1056912	GLRX2
2922.965	-4354	1.36E-04	1381	0.333333	3.306085	1111162	HSD17B8
2679.46	-4354	1.36E-04	1620	0.333333	3.305855	1076024	SMURF1
2730.519	-4354	1.36E-04	1568	0.333333	3.305855	1037530	CD47
2614.674	-4354	1.36E-04	1497	0.333333	3.305855	1041228	PGF
2503.284	-4354	1.36E-04	1474	0.333333	3.305855	1040152	SLC2A5
2120.954	-4354	1.36E-04	1463	0.333333	3.305855	994848	NRXN2
2881.615	-4354	1.36E-04	1601	0.333333	3.305626	1074010	PKM
3215.503	-4354	1.36E-04	1539	0.333333	3.305626	1133406	PPIP5K1
2045.814	-4354	1.36E-04	1483	0.333333	3.305626	950786	COL2A1
2273.297	-4354	1.36E-04	1465	0.333333	3.305626	991184	ERO1A
2160.351	-4354	1.36E-04	1430	0.333333	3.305626	961238	SH3GLB1
2361.574	-4354	1.36E-04	1623	0.333333	3.305396	992894	PTPRC
2161.732	-4354	1.36E-04	1568	0.333333	3.305396	975096	TRPC6
2902.857	-4354	1.36E-04	1559	0.333333	3.305396	1122272	STX17
2774.793	-4354	1.36E-04	1487	0.333333	3.305396	1039302	CSMD3
2699.62	-4354	1.36E-04	1588	0.333333	3.305166	1089724	HUWE1
2436.504	-4354	1.36E-04	1568	0.333333	3.305166	985354	ATP6V1A
2637.717	-4354	1.36E-04	1514	0.333333	3.305166	1059980	GRHPR
2651.17	-4354	1.36E-04	1496	0.333333	3.305166	1041968	MLST8

2454.451	-4354	1.36E-04	1413	0.333333	3.305166	1011516	SHISA5
2152.798	-4354	1.36E-04	1398	0.333333	3.305166	997698	PYGB
2105.031	-4354	1.36E-04	1598	0.333333	3.304937	1002278	FGFR1
2500.795	-4354	1.36E-04	1545	0.333333	3.304937	1050310	ALDH7A1
2374.838	-4354	1.36E-04	1534	0.333333	3.304937	1005608	LMNA
2215.704	-4354	1.36E-04	1513	0.333333	3.304937	982088	STX4
2236.414	-4354	1.36E-04	1509	0.333333	3.304937	1005744	NR4A3
2463.312	-4354	1.36E-04	1494	0.333333	3.304937	1045354	OLIG1
2070.728	-4354	1.36E-04	1478	0.333333	3.304937	955232	NFE2L2
2157.422	-4354	1.36E-04	1639	0.333333	3.304707	983346	CNR1
4075.333	-4354	1.36E-04	1570	0.333333	3.304707	1578122	AMBRA1
2519.92	-4354	1.36E-04	1648	0.333333	3.304478	1023694	GRIN2B
2325.169	-4354	1.36E-04	1489	0.333333	3.304478	955016	KCNJ9
2061.779	-4354	1.36E-04	1442	0.333333	3.304478	963638	IL17RA
2281.408	-4354	1.36E-04	1431	0.333333	3.304478	993838	LGR5
2478.911	-4354	1.36E-04	1424	0.333333	3.304478	1047124	PPP3CC
3249.631	-4354	1.36E-04	1730	0.333333	3.304248	1050006	DLD
2718.388	-4354	1.36E-04	1573	0.333333	3.304248	1049470	BAX
2577.145	-4354	1.36E-04	1573	0.333333	3.304248	1010996	MAPK11
2364.398	-4354	1.36E-04	1541	0.333333	3.304248	1023268	CAMKK2
2751.379	-4354	1.36E-04	1407	0.333333	3.304248	1067788	PRXL2B
2319.279	-4354	1.35E-04	1602	0.333333	3.304018	988116	HSPA5
2240.654	-4354	1.35E-04	1440	0.333333	3.304018	987572	CTSZ
2410.247	-4354	1.35E-04	1426	0.333333	3.304018	1017224	DAPP1
2420.589	-4354	1.35E-04	1683	0.333333	3.303789	1003676	PARVA
2226.162	-4354	1.35E-04	1617	0.333333	3.303789	983834	ADCY2
2283.492	-4354	1.35E-04	1468	0.333333	3.303789	981734	NOS3
3306.352	-4354	1.35E-04	1443	0.333333	3.303789	1104592	RBP2
2666.733	-4354	1.35E-04	1559	0.333333	3.303559	1051836	EXOC2
2445.613	-4354	1.35E-04	1544	0.333333	3.303559	1029630	E2F3
2031.293	-4354	1.35E-04	1524	0.333333	3.303559	979120	OLFM1
1996.226	-4354	1.35E-04	1520	0.333333	3.303559	945500	IGF1
2147.831	-4354	1.35E-04	1449	0.333333	3.303559	981048	SERPINB5
2485.542	-4354	1.35E-04	1438	0.333333	3.303559	1033752	MGST3
2366.162	-4354	1.35E-04	1430	0.333333	3.303559	1018472	DOT1L
2263.57	-4354	1.35E-04	1469	0.333333	3.30333	1024078	HIPK2
2973.896	-4354	1.35E-04	1385	0.333333	3.30333	1131360	HSD17B7
2668.93	-4354	1.35E-04	1582	0.333333	3.3031	1063086	WEE1
2669.458	-4354	1.35E-04	1579	0.333333	3.3031	1056092	PHKB
2693.222	-4354	1.35E-04	1536	0.333333	3.3031	1044264	STK3
2346.631	-4354	1.35E-04	1436	0.333333	3.3031	1023702	SLC37A2

2236.218	-4354	1.35E-04	1412	0.333333	3.3031	976494	ELOVL2
2570.053	-4354	1.35E-04	1621	0.333333	3.30287	1023718	CCL5
2730.569	-4354	1.35E-04	1557	0.333333	3.30287	1073528	PDCD4
2455.064	-4354	1.35E-04	1402	0.333333	3.30287	1019570	WDR45
2613.445	-4354	1.35E-04	1387	0.333333	3.30287	1026928	PIP4P2
2631.147	-4354	1.35E-04	1376	0.333333	3.30287	1026176	SIRT6
2501.256	-4354	1.35E-04	1605	0.333333	3.302641	1028140	BMPR2
3222.934	-4354	1.35E-04	1571	0.333333	3.302641	1064226	COX5A
2073.25	-4354	1.35E-04	1473	0.333333	3.302641	989082	RGS3
2128.701	-4354	1.35E-04	1450	0.333333	3.302641	927260	CACNA1B
2701.428	-4354	1.35E-04	1438	0.333333	3.302641	1052152	SPHK2
2228.117	-4354	1.35E-04	1424	0.333333	3.302641	992922	DLL4
2256.461	-4354	1.35E-04	1391	0.333333	3.302641	1021430	IL11RA
3022.112	-4354	1.35E-04	1336	0.333333	3.302641	1041198	RASAL3
2776.951	-4354	1.35E-04	1623	0.333333	3.302411	1089596	MDM4
3573.657	-4354	1.35E-04	1593	0.333333	3.302411	1104156	NDUFAB1
2496.873	-4354	1.35E-04	1592	0.333333	3.302411	1035442	PIAS1
2053.621	-4354	1.35E-04	1397	0.333333	3.302411	941366	TLR8
2326.277	-4354	1.35E-04	1492	0.333333	3.302181	954144	XCR1
2669.922	-4354	1.35E-04	1652	0.333333	3.301952	1048366	FBXW11
2736.08	-4354	1.35E-04	1383	0.333333	3.301952	1061484	ACAT2
2751.778	-4354	1.35E-04	1511	0.333333	3.301722	1056104	PPP3R1
2345.528	-4354	1.35E-04	1481	0.333333	3.301722	980078	SHH
2527.65	-4354	1.35E-04	1457	0.333333	3.301722	991754	CACNG2
2092.589	-4354	1.35E-04	1418	0.333333	3.301722	948766	PDLIM4
2194.173	-4354	1.35E-04	1397	0.333333	3.301722	944804	GRIN2D
2747.654	-4354	1.35E-04	1356	0.333333	3.301722	1047254	SAT2
2391.784	-4354	1.35E-04	1339	0.333333	3.301722	929616	LDHAL6B
2701.589	-4354	1.35E-04	1646	0.333333	3.301493	1067676	BTRC
2809.866	-4354	1.35E-04	1636	0.333333	3.301493	1042974	SNW1
2165.94	-4354	1.35E-04	1617	0.333333	3.301493	992622	RARA
2055.392	-4354	1.35E-04	1504	0.333333	3.301493	944320	ANGPT1
2340.747	-4354	1.35E-04	1492	0.333333	3.301493	939920	SLC17A7
2439.96	-4354	1.35E-04	1451	0.333333	3.301493	1008258	GSTM1
2548.27	-4354	1.35E-04	1410	0.333333	3.301493	1041854	CDKN2C
2291.342	-4354	1.35E-04	1399	0.333333	3.301493	911580	ANGPTL3
2150.418	-4354	1.35E-04	1389	0.333333	3.301493	934530	GDF9
2543.218	-4354	1.35E-04	1573	0.333333	3.301263	1044060	TRIM32
2211.241	-4354	1.35E-04	1492	0.333333	3.301263	981210	PRLR
2640.962	-4354	1.35E-04	1372	0.333333	3.301263	1007696	DGAT2
2055.579	-4354	1.35E-04	1512	0.333333	3.301033	925988	G6PC

2074.208	-4354	1.35E-04	1442	0.333333	3.301033	962782	HEY2
2267.624	-4354	1.35E-04	1409	0.333333	3.301033	981802	MICALL2
2613.77	-4354	1.35E-04	1394	0.333333	3.301033	995204	UBA3
2642.301	-4354	1.35E-04	1557	0.333333	3.300804	1052770	PRMT2
2167.437	-4354	1.35E-04	1537	0.333333	3.300804	982910	ETV1
2338.441	-4354	1.35E-04	1468	0.333333	3.300804	1030552	PHLPP1
2230.738	-4354	1.35E-04	1446	0.333333	3.300804	975844	RNF130
2148.335	-4354	1.35E-04	1494	0.333333	3.300574	961962	NEFM
2500.512	-4354	1.35E-04	1480	0.333333	3.300574	1029678	CXCL10
2222	-4354	1.35E-04	1414	0.333333	3.300574	987462	ELL2
2589.026	-4354	1.35E-04	1381	0.333333	3.300574	940062	MOGAT2
2582.738	-4354	1.35E-04	1373	0.333333	3.300574	1019964	SHARPIN
2570.072	-4354	1.35E-04	1607	0.333333	3.300344	994796	GNAI3
3202.026	-4354	1.35E-04	1559	0.333333	3.300344	1274940	CCR2
2521.349	-4354	1.35E-04	1484	0.333333	3.300344	1016696	ACACB
3062.267	-4354	1.35E-04	1469	0.333333	3.300344	1025904	COX7A2L
2760.62	-4354	1.35E-04	1405	0.333333	3.300344	1025504	HYAL3
2397.26	-4354	1.35E-04	1591	0.333333	3.300115	1020942	GNAS
1840.14	-4354	1.35E-04	1590	0.333333	3.300115	887120	ITGB2
2332.424	-4354	1.35E-04	1505	0.333333	3.300115	985956	NFKBIZ
2652.239	-4354	1.35E-04	1536	0.333333	3.299885	1048244	ACVR2B
2282.194	-4354	1.35E-04	1523	0.333333	3.299885	1000272	ARHGEF11
10739.08	-4354	1.35E-04	1762	0.333333	3.299656	3358814	RAC1
2234.729	-4354	1.35E-04	1650	0.333333	3.299656	945186	ADRA1A
2695.732	-4354	1.35E-04	1583	0.333333	3.299656	1052030	E2F1
1970.847	-4354	1.35E-04	1466	0.333333	3.299656	919284	RHOC
2403.402	-4354	1.35E-04	1398	0.333333	3.299656	939794	IFNA8
2433.37	-4354	1.35E-04	1548	0.333333	3.299426	1008920	MAP3K2
2557.732	-4354	1.35E-04	1514	0.333333	3.299426	1048650	MYB
2378.645	-4354	1.35E-04	1467	0.333333	3.299426	988156	EPHA1
2686.516	-4354	1.35E-04	1465	0.333333	3.299426	1045376	PHKA2
2399.263	-4354	1.35E-04	1385	0.333333	3.299426	994788	ARPC5L
3251.658	-4354	1.35E-04	1367	0.333333	3.299426	1109332	CLDN15
2487.945	-4354	1.35E-04	1333	0.333333	3.299426	966932	GGT1
2102.823	-4354	1.35E-04	1512	0.333333	3.299196	939940	NFASC
2119.628	-4354	1.35E-04	1489	0.333333	3.299196	917996	HLA-G
1956.197	-4354	1.35E-04	1368	0.333333	3.299196	936764	TNFRSF10C
2481.778	-4354	1.35E-04	1580	0.333333	3.298967	1008386	E2F4
2343.476	-4354	1.35E-04	1533	0.333333	3.298967	995950	GLIS3
3138.894	-4354	1.35E-04	1458	0.333333	3.298967	1102656	CREB3L4
2197.171	-4354	1.35E-04	1454	0.333333	3.298967	956704	ZIC1

2407.675	-4354	1.35E-04	1433	0.333333	3.298967	1027822	DNMT3A
2490.151	-4354	1.35E-04	1380	0.333333	3.298967	996328	ORAI1
2293.589	-4354	1.35E-04	1588	0.333333	3.298737	990306	ATP2A2
2459.744	-4354	1.35E-04	1518	0.333333	3.298737	995218	AP1G1
2457.71	-4354	1.35E-04	1431	0.333333	3.298737	1015436	TRAF4
2555.434	-4354	1.35E-04	1419	0.333333	3.298737	969632	WNT16
1846.195	-4354	1.35E-04	1360	0.333333	3.298737	871928	SLC5A7
2190.302	-4354	1.35E-04	1335	0.333333	3.298737	985350	NTNG2
2313.561	-4354	1.35E-04	1329	0.333333	3.298737	1013490	TGIF2
1971.408	-4354	1.35E-04	1558	0.333333	3.298507	927974	IGFBP5
2341.522	-4354	1.35E-04	1432	0.333333	3.298507	950256	CALB1
1966.563	-4354	1.35E-04	1425	0.333333	3.298507	891936	LPA
2096.365	-4354	1.35E-04	1401	0.333333	3.298507	939462	MALT1
2376.104	-4354	1.35E-04	1488	0.333333	3.298278	1010482	ACOX3
1972.999	-4354	1.35E-04	1467	0.333333	3.298278	914172	PAK3
2141.204	-4354	1.35E-04	1464	0.333333	3.298278	937244	AP1S2
2343.391	-4354	1.35E-04	1448	0.333333	3.298278	988598	PDE8A
2095.046	-4354	1.35E-04	1415	0.333333	3.298278	920436	IFNA1
2241.299	-4354	1.35E-04	1461	0.333333	3.298048	972110	ATG7
2689.193	-4354	1.35E-04	1458	0.333333	3.298048	1001778	CKS1B
2842.904	-4354	1.35E-04	1366	0.333333	3.298048	1005602	CACNG7
1933.201	-4354	1.35E-04	1329	0.333333	3.298048	890216	LCE2B
2170.28	-4354	1.35E-04	1622	0.333333	3.297819	951094	CXCR3
2162.576	-4354	1.35E-04	1551	0.333333	3.297819	953514	SOCS1
2096.29	-4354	1.35E-04	1494	0.333333	3.297819	953182	IL12RB1
2677.014	-4354	1.35E-04	1458	0.333333	3.297819	1049176	FBXO25
3007.649	-4354	1.35E-04	1506	0.333333	3.297589	1056410	COX7A2
2589.947	-4354	1.35E-04	1471	0.333333	3.297589	1004616	TNFRSF25
2636.134	-4354	1.35E-04	1425	0.333333	3.297589	988902	TUBB1
2652.142	-4354	1.35E-04	1403	0.333333	3.297589	1011344	NELFCD
2288.775	-4354	1.35E-04	1373	0.333333	3.297589	969044	CCL8
2248.19	-4354	1.35E-04	1334	0.333333	3.297589	973342	KDM6B
2291.283	-4354	1.35E-04	1519	0.333333	3.297359	972906	HDAC5
2612.067	-4354	1.35E-04	1493	0.333333	3.297359	1032946	CASP6
2191.087	-4354	1.35E-04	1404	0.333333	3.297359	986206	SESN2
2621.7	-4354	1.35E-04	1611	0.333333	3.29713	995036	UBE2E1
2280.482	-4354	1.35E-04	1504	0.333333	3.29713	998854	NF2
2214.433	-4354	1.35E-04	1459	0.333333	3.29713	983936	TBL1X
2509.142	-4354	1.35E-04	1454	0.333333	3.29713	967912	SUCLA2
2440.522	-4354	1.35E-04	1401	0.333333	3.29713	983484	GNPTG
2825.156	-4354	1.35E-04	1670	0.333333	3.2969	1037982	UBE3A

1959.591	-4354	1.35E-04	1439	0.333333	3.2969	916496	HSPG2
2188.226	-4354	1.35E-04	1410	0.333333	3.2969	918776	CA6
2455.165	-4354	1.35E-04	1495	0.333333	3.29667	996522	PHKG2
2204.236	-4354	1.35E-04	1438	0.333333	3.29667	960672	CYSLTR1
3006.251	-4354	1.35E-04	1710	0.333333	3.296441	1029748	KRAS
2218.984	-4354	1.35E-04	1529	0.333333	3.296441	977532	SYNJ1
2172.804	-4354	1.35E-04	1413	0.333333	3.296441	987584	BCL2L11
3061.488	-4354	1.35E-04	1388	0.333333	3.296441	1209486	CHMP1B
2777.332	-4354	1.35E-04	1366	0.333333	3.296441	1045832	INPP5E
2198.464	-4354	1.35E-04	1355	0.333333	3.296441	975042	TCL1A
2664.793	-4354	1.35E-04	1626	0.333333	3.296211	1022470	DLG1
2832.492	-4354	1.35E-04	1478	0.333333	3.296211	1012512	RASA2
2296.387	-4354	1.35E-04	1421	0.333333	3.296211	988064	ALDH1B1
2667.527	-4354	1.35E-04	1595	0.333333	3.295982	1016216	PPP2R5C
2465.36	-4354	1.35E-04	1562	0.333333	3.295982	965692	BCR
2487.438	-4354	1.35E-04	1484	0.333333	3.295982	991584	RRM2B
2132.235	-4354	1.35E-04	1461	0.333333	3.295982	910800	IMPG2
2296.389	-4354	1.35E-04	1449	0.333333	3.295982	975366	SOCS6
2241.109	-4354	1.35E-04	1423	0.333333	3.295982	966360	AGAP2
2226.26	-4354	1.35E-04	1419	0.333333	3.295982	954372	ASIC1
2143.089	-4354	1.35E-04	1413	0.333333	3.295982	968984	ATP2A3
2346.688	-4354	1.35E-04	1398	0.333333	3.295982	984534	SEMA4C
2245.22	-4354	1.35E-04	1361	0.333333	3.295982	911270	CACNG5
2594.114	-4354	1.35E-04	1329	0.333333	3.295982	989252	PIP4P1
2047.502	-4354	1.35E-04	1321	0.333333	3.295982	938422	MLLT1
2442.403	-4354	1.35E-04	1591	0.333333	3.295752	992222	NCOA3
2014.663	-4354	1.35E-04	1531	0.333333	3.295752	918490	PLCB2
1884.24	-4354	1.35E-04	1517	0.333333	3.295752	910496	ACTA2
3108.65	-4354	1.35E-04	1482	0.333333	3.295752	1084084	CLDN2
2887.218	-4354	1.35E-04	1447	0.333333	3.295752	1021876	PLCD4
2245.265	-4354	1.35E-04	1349	0.333333	3.295752	955302	ACOT7
2592.778	-4354	1.35E-04	1498	0.333333	3.295522	1030472	PIP5K1A
2187.316	-4354	1.35E-04	1477	0.333333	3.295293	966074	MLLT3
2160.172	-4354	1.35E-04	1376	0.333333	3.295293	946060	IL21R
3134.098	-4354	1.35E-04	1322	0.333333	3.295293	1188248	CHMP1A
2528.953	-4354	1.35E-04	1493	0.333333	3.295063	958324	GRK6
2573.922	-4354	1.35E-04	1456	0.333333	3.295063	952522	TUBA1B
2831.108	-4354	1.35E-04	1447	0.333333	3.295063	1036762	HERC2
2588.2	-4354	1.35E-04	1368	0.333333	3.295063	975140	FIS1
2370.962	-4354	1.35E-04	1367	0.333333	3.295063	945902	CLDN11
2696.023	-4354	1.35E-04	1300	0.333333	3.295063	995898	JMJD8

3226.746	-4354	1.35E-04	1500	0.333333	3.294834	1197734	CHMP2B
2843.167	-4354	1.35E-04	1476	0.333333	3.294834	1030304	TBP
2111.096	-4354	1.35E-04	1443	0.333333	3.294834	926608	DIAPH2
2880.377	-4354	1.35E-04	1384	0.333333	3.294834	1032034	CBLC
2845.921	-4354	1.35E-04	1517	0.333333	3.294604	1021088	SMARCAD1
2192.399	-4354	1.35E-04	1486	0.333333	3.294604	918916	DGKB
2230.683	-4354	1.35E-04	1450	0.333333	3.294604	980150	INPP4A
1770.812	-4354	1.35E-04	1395	0.333333	3.294604	888960	WWTR1
2198.753	-4354	1.35E-04	1474	0.333333	3.294374	960414	LRP6
2263.068	-4354	1.35E-04	1456	0.333333	3.294374	927836	ARHGEF2
2124.11	-4354	1.35E-04	1367	0.333333	3.294374	955864	ELOVL5
2236.458	-4354	1.35E-04	1365	0.333333	3.294374	954176	CD8B
2722.164	-4354	1.35E-04	1500	0.333333	3.294145	1007434	ACADSB
3684.808	-4354	1.35E-04	1474	0.333333	3.294145	1074338	NDUFB11
2349.929	-4354	1.35E-04	1446	0.333333	3.294145	984892	IGSF11
2055.501	-4354	1.35E-04	1566	0.333333	3.293915	904094	ADORA1
2127.401	-4354	1.35E-04	1435	0.333333	3.293915	971718	PDK3
2165.613	-4354	1.35E-04	1417	0.333333	3.293915	965228	CPT1A
2857.211	-4354	1.35E-04	1687	0.333333	3.293685	989348	HSP90AA1
2695.679	-4354	1.35E-04	1515	0.333333	3.293685	1028858	WDR59
2278.842	-4354	1.35E-04	1458	0.333333	3.293685	904392	PAK5
1991.257	-4354	1.35E-04	1453	0.333333	3.293685	931436	F11R
1933.602	-4354	1.35E-04	1407	0.333333	3.293685	916404	PARVB
2329.26	-4354	1.35E-04	1399	0.333333	3.293685	923232	SNX3
2309.605	-4354	1.35E-04	1366	0.333333	3.293685	933156	LAMTOR1
2694.797	-4354	1.35E-04	1568	0.333333	3.293456	979896	PDHB
2415.777	-4354	1.35E-04	1387	0.333333	3.293456	968580	LAT
1967.812	-4354	1.35E-04	1387	0.333333	3.293456	904840	ANGPT2
3416.013	-4354	1.35E-04	1668	0.333333	3.293226	1052656	NDUFS1
2005.708	-4354	1.35E-04	1496	0.333333	3.293226	923774	SORBS1
2290.953	-4354	1.35E-04	1483	0.333333	3.293226	979000	OCRL
2460.057	-4354	1.35E-04	1453	0.333333	3.293226	988522	PDE6B
2017.775	-4354	1.35E-04	1417	0.333333	3.293226	913588	TNFSF11
2340.763	-4354	1.35E-04	1451	0.333333	3.292997	975758	PRKAB1
2220.447	-4354	1.35E-04	1447	0.333333	3.292997	922740	TRIP12
1986.85	-4354	1.35E-04	1446	0.333333	3.292997	907152	PTGDR
2478.309	-4354	1.35E-04	1735	0.333333	3.292767	929944	PSMA3
1969.482	-4354	1.35E-04	1488	0.333333	3.292767	891248	PDGFB
3069.413	-4354	1.35E-04	1492	0.333333	3.292537	1133266	MEF2D
2212.361	-4354	1.35E-04	1367	0.333333	3.292537	943510	ALDH3B2
2698.826	-4354	1.35E-04	1356	0.333333	3.292537	1103020	PLA2G2E

3407.035	-4354	1.35E-04	1332	0.333333	3.292537	1238506	CHMP6
1912.5	-4354	1.35E-04	1469	0.333333	3.292308	848154	CTSS
1950.937	-4354	1.35E-04	1439	0.333333	3.292308	888562	CSF1
3067.711	-4354	1.35E-04	1373	0.333333	3.292308	1076554	RNF125
2194.895	-4354	1.35E-04	1335	0.333333	3.292308	912072	CREB3L1
2138.711	-4354	1.35E-04	1419	0.333333	3.292078	911504	GM2A
1916.984	-4354	1.35E-04	1404	0.333333	3.292078	894616	LOXL2
2285.381	-4354	1.35E-04	1309	0.333333	3.292078	938012	EFL1
2032.132	-4354	1.35E-04	1360	0.333333	3.291848	892424	ATP6V1B1
2112.94	-4354	1.34E-04	1501	0.333333	3.291619	956198	GNB5
2001.614	-4354	1.34E-04	1435	0.333333	3.291619	927324	ZMAT3
2064.128	-4354	1.34E-04	1349	0.333333	3.291619	884798	IL37
2483.748	-4354	1.34E-04	1317	0.333333	3.291619	960214	ASPSCR1
2054.294	-4354	1.34E-04	1482	0.333333	3.291389	947532	PPARD
1912.38	-4354	1.34E-04	1477	0.333333	3.291389	884954	RXRA
2361.238	-4354	1.34E-04	1396	0.333333	3.291389	940352	GSTM3
2047.33	-4354	1.34E-04	1363	0.333333	3.291389	919064	CD72
2247.298	-4354	1.34E-04	1361	0.333333	3.291389	968736	PHKA1
2276.225	-4354	1.34E-04	1551	0.333333	3.29116	902790	DRD2
2568.111	-4354	1.34E-04	1429	0.333333	3.29093	946146	UNC5D
1881.1	-4354	1.34E-04	1301	0.333333	3.29093	891520	ETV7
2238.028	-4354	1.34E-04	1719	0.333333	3.2907	963332	CREBBP
2272.948	-4354	1.34E-04	1456	0.333333	3.2907	933662	F12
2578.205	-4354	1.34E-04	1545	0.333333	3.290471	934096	RBX1
2185.214	-4354	1.34E-04	1409	0.333333	3.290471	898342	UBE2J1
3106.865	-4354	1.34E-04	1387	0.333333	3.290471	1057738	ORC6
1775.632	-4354	1.34E-04	1369	0.333333	3.290471	875024	TNFRSF10B
2417.853	-4354	1.34E-04	1350	0.333333	3.290471	958732	KLHL13
1995.107	-4354	1.34E-04	1334	0.333333	3.290471	886258	TLE6
2624.913	-4354	1.34E-04	1465	0.333333	3.290241	1008556	TAOK1
2155.008	-4354	1.34E-04	1450	0.333333	3.290241	945086	CDKN2A
2569.493	-4354	1.34E-04	1333	0.333333	3.290241	978792	RAD9A
1678.499	-4354	1.34E-04	1405	0.333333	3.290011	853312	CAV2
1870.165	-4354	1.34E-04	1404	0.333333	3.290011	852408	EFNA2
2087.383	-4354	1.34E-04	1385	0.333333	3.290011	889360	PRKD2
2288.984	-4354	1.34E-04	1377	0.333333	3.290011	969602	VANGL1
2643.657	-4354	1.34E-04	1292	0.333333	3.290011	967472	PRMT3
1892.901	-4354	1.34E-04	1467	0.333333	3.289782	874192	GLI2
2594.847	-4354	1.34E-04	1407	0.333333	3.289782	1117390	PLA2G5
2534.503	-4354	1.34E-04	1348	0.333333	3.289782	978764	SLC22A5
2757.921	-4354	1.34E-04	1299	0.333333	3.289782	985402	FANCL

1701.123	-4354	1.34E-04	1443	0.333333	3.289552	853022	COL13A1
1904.289	-4354	1.34E-04	1354	0.333333	3.289552	872804	CYP2U1
2218.096	-4354	1.34E-04	1448	0.333333	3.289323	896678	TRPC3
3254.691	-4354	1.34E-04	1425	0.333333	3.289323	996168	ATP5MC1
2446.808	-4354	1.34E-04	1379	0.333333	3.289323	956160	SLC2A8
1780.451	-4354	1.34E-04	1417	0.333333	3.289093	843358	GATA4
2086.724	-4354	1.34E-04	1390	0.333333	3.289093	906404	SREBF1
1728.137	-4354	1.34E-04	1270	0.333333	3.289093	849734	DENND6B
2476.27	-4354	1.34E-04	1503	0.333333	3.288863	947612	RB1CC1
1779.879	-4354	1.34E-04	1469	0.333333	3.288863	849952	ITGAX
2380.095	-4354	1.34E-04	1502	0.333333	3.288634	954170	STK4
2215.984	-4354	1.34E-04	1413	0.333333	3.288634	894922	NR1I2
1907.858	-4354	1.34E-04	1313	0.333333	3.288634	813606	IL9
1877.164	-4354	1.34E-04	1461	0.333333	3.288404	866050	DUSP1
2386.599	-4354	1.34E-04	1510	0.333333	3.288175	952480	AKT2
2434.829	-4354	1.34E-04	1475	0.333333	3.288175	912762	TRPM3
2028.972	-4354	1.34E-04	1325	0.333333	3.288175	880592	PRAM1
1985.684	-4354	1.34E-04	1304	0.333333	3.288175	903942	SCIN
1714.787	-4354	1.34E-04	1279	0.333333	3.288175	822850	MS4A4A
2123.676	-4354	1.34E-04	1500	0.333333	3.287945	882476	MCL1
2488.976	-4354	1.34E-04	1496	0.333333	3.287945	932482	UBA2
2014.142	-4354	1.34E-04	1443	0.333333	3.287945	881938	NOTCH3
2166.727	-4354	1.34E-04	1441	0.333333	3.287945	901186	CXCL5
2630.484	-4354	1.34E-04	1377	0.333333	3.287945	953896	ECSIT
2064.168	-4354	1.34E-04	1392	0.333333	3.287715	923868	PLEKHB1
1959.125	-4354	1.34E-04	1342	0.333333	3.287715	822756	IL22
1718.586	-4354	1.34E-04	1339	0.333333	3.287715	851416	PDLIM2
2222.001	-4354	1.34E-04	1297	0.333333	3.287715	919154	CHKB
2488.995	-4354	1.34E-04	1628	0.333333	3.287486	967122	ATM
2154.045	-4354	1.34E-04	1507	0.333333	3.287486	926468	PPARA
2145.501	-4354	1.34E-04	1424	0.333333	3.287256	877624	NFKB2
1982.597	-4354	1.34E-04	1424	0.333333	3.287026	913582	CASP9
1883.682	-4354	1.34E-04	1390	0.333333	3.287026	858002	GADD45B
2371.924	-4354	1.34E-04	1379	0.333333	3.287026	903046	SLC25A6
2186.977	-4354	1.34E-04	1311	0.333333	3.287026	920196	SLC27A1
2143.337	-4354	1.34E-04	1547	0.333333	3.286797	890416	CAMK2B
2574.696	-4354	1.34E-04	1523	0.333333	3.286797	943998	PIK3C2A
2042.139	-4354	1.34E-04	1438	0.333333	3.286797	875128	CAMK1G
1968.152	-4354	1.34E-04	1537	0.333333	3.286567	880484	JAK3
2189.495	-4354	1.34E-04	1467	0.333333	3.286567	934516	PPP2R1B
1915.143	-4354	1.34E-04	1463	0.333333	3.286567	863708	ITGB4

2417.994	-4354	1.34E-04	1430	0.333333	3.286567	928172	PPP2R5D
2032.249	-4354	1.34E-04	1373	0.333333	3.286567	867668	DNM1
1916.755	-4354	1.34E-04	1367	0.333333	3.286567	882528	MCOLN3
2099.622	-4354	1.34E-04	1363	0.333333	3.286567	920928	CACNB3
1781.149	-4354	1.34E-04	1333	0.333333	3.286567	840644	NLRP3
1796.924	-4354	1.34E-04	1317	0.333333	3.286567	831232	ENPP3
2280.7	-4354	1.34E-04	1626	0.333333	3.286338	914286	RB1
1841.548	-4354	1.34E-04	1337	0.333333	3.286338	866956	LTK
1900.009	-4354	1.34E-04	1389	0.333333	3.286108	879428	PI4K2A
2084.345	-4354	1.34E-04	1322	0.333333	3.286108	890294	MICB
2085.636	-4354	1.34E-04	1489	0.333333	3.285878	884522	CHRM1
2492.506	-4354	1.34E-04	1415	0.333333	3.285878	983048	CDKAL1
2256.777	-4354	1.34E-04	1413	0.333333	3.285878	921082	ACAA2
2023.493	-4354	1.34E-04	1393	0.333333	3.285878	872870	IRF9
2128.827	-4354	1.34E-04	1497	0.333333	3.285649	895298	AKT3
1756.921	-4354	1.34E-04	1415	0.333333	3.285649	830160	STAT6
2036.424	-4354	1.34E-04	1412	0.333333	3.285649	838836	IL4
2147.093	-4354	1.34E-04	1397	0.333333	3.285649	920502	PROK2
2539.49	-4354	1.34E-04	1330	0.333333	3.285649	879398	PFKL
1749.192	-4354	1.34E-04	1328	0.333333	3.285649	844478	LILRA1
2243.809	-4354	1.34E-04	1300	0.333333	3.285649	907720	MICA
2059.303	-4354	1.34E-04	1449	0.333333	3.285419	910860	SKIL
2171.07	-4354	1.34E-04	1427	0.333333	3.285419	925582	GOLGA2
1861.366	-4354	1.34E-04	1381	0.333333	3.285419	847770	CRB1
1780.555	-4354	1.34E-04	1522	0.333333	3.285189	805198	HTR2A
1591.471	-4354	1.34E-04	1441	0.333333	3.285189	805002	THBS1
2175.266	-4354	1.34E-04	1393	0.333333	3.285189	924968	LPCAT3
2600.097	-4354	1.34E-04	1376	0.333333	3.285189	908106	GSS
1708.905	-4354	1.34E-04	1363	0.333333	3.285189	853698	MPZL1
1947.504	-4354	1.34E-04	1326	0.333333	3.285189	885248	PRR5
2061.701	-4354	1.34E-04	1470	0.333333	3.28496	874000	NCK1
2164.835	-4354	1.34E-04	1428	0.333333	3.28496	922096	PTPRA
1991.355	-4354	1.34E-04	1423	0.333333	3.28496	895598	GLS
2387.816	-4354	1.34E-04	1334	0.333333	3.28496	906730	HJV
1812.319	-4354	1.34E-04	1301	0.333333	3.28496	864704	KLF13
2205.674	-4354	1.34E-04	1276	0.333333	3.28496	875266	LAMTOR2
2057.766	-4354	1.34E-04	1263	0.333333	3.28496	868034	RFNG
2670.651	-4354	1.34E-04	1256	0.333333	3.28496	973604	WHAMM
2524.284	-4354	1.34E-04	1829	0.333333	3.28473	941452	CDK2
2004.182	-4354	1.34E-04	1457	0.333333	3.28473	878536	PSEN1
2055.932	-4354	1.34E-04	1420	0.333333	3.28473	891036	SMARCA1

2072.207	-4354	1.34E-04	1367	0.333333	3.28473	849092	CADM3
1733.485	-4354	1.34E-04	1363	0.333333	3.28473	841822	SULT1C2
2082.757	-4354	1.34E-04	1341	0.333333	3.28473	911356	AGPAT3
1927.667	-4354	1.34E-04	1337	0.333333	3.28473	851756	NCKAP1L
1762.575	-4354	1.34E-04	1307	0.333333	3.28473	797100	LRAT
1894.202	-4354	1.34E-04	1388	0.333333	3.284501	849492	IL1RN
2311.885	-4354	1.34E-04	1277	0.333333	3.284501	896680	CLDN6
1948.096	-4354	1.34E-04	1264	0.333333	3.284501	850302	IL4I1
2287.764	-4354	1.34E-04	1520	0.333333	3.284271	892018	CANX
2389.405	-4354	1.34E-04	1471	0.333333	3.284271	913602	PSME4
1976.317	-4354	1.34E-04	1270	0.333333	3.284271	879042	TKTL1
2065.694	-4354	1.34E-04	1250	0.333333	3.284271	850454	PRAP1
2547.127	-4354	1.34E-04	1600	0.333333	3.284041	936320	MAD2L1
1746.519	-4354	1.34E-04	1416	0.333333	3.284041	824372	IL1RL1
2142.914	-4354	1.34E-04	1395	0.333333	3.284041	913840	MLF1
2299.389	-4354	1.34E-04	1316	0.333333	3.284041	872386	HIPK4
2426.107	-4354	1.34E-04	1268	0.333333	3.284041	922390	CRTC2
2039.19	-4354	1.34E-04	1289	0.333333	3.283812	873990	UBE2E2
2085.847	-4354	1.34E-04	1491	0.333333	3.283582	893088	ERBB3
2293.325	-4354	1.34E-04	1464	0.333333	3.283582	910966	SIAH1
1807.32	-4354	1.34E-04	1448	0.333333	3.283582	840416	PTPRB
2247.73	-4354	1.34E-04	1426	0.333333	3.283582	909180	MPP5
1948.466	-4354	1.34E-04	1404	0.333333	3.283582	853280	CCL23
2105.827	-4354	1.34E-04	1392	0.333333	3.283582	885640	GDF11
2074.073	-4354	1.34E-04	1259	0.333333	3.283352	886254	LDLRAP1
2326.694	-4354	1.34E-04	1250	0.333333	3.283352	904474	LDHD
2266.93	-4354	1.34E-04	1370	0.333333	3.283123	919782	FZD3
2089.429	-4354	1.34E-04	1363	0.333333	3.283123	890316	FDFT1
2014.77	-4354	1.34E-04	1327	0.333333	3.283123	873250	TPCN1
2159.872	-4354	1.34E-04	1445	0.333333	3.282893	870254	MCM5
1773.708	-4354	1.34E-04	1441	0.333333	3.282893	846306	ATF3
2405.47	-4354	1.34E-04	1391	0.333333	3.282893	903818	CPT2
2187.126	-4354	1.34E-04	1363	0.333333	3.282893	890920	MFN2
2214.82	-4354	1.34E-04	1499	0.333333	3.282664	820774	HTR2C
2499.683	-4354	1.34E-04	1424	0.333333	3.282664	923610	UBE4B
2454.274	-4354	1.34E-04	1637	0.333333	3.282434	901678	CSNK1A1
2645.753	-4354	1.34E-04	1543	0.333333	3.282434	958436	SKP2
2108.127	-4354	1.34E-04	1445	0.333333	3.282434	871160	MAX
2546.747	-4354	1.34E-04	1500	0.333333	3.282204	945948	BUB1B
2054.136	-4354	1.34E-04	1438	0.333333	3.282204	870842	MGST1
2345.53	-4354	1.34E-04	1312	0.333333	3.282204	919346	AP4M1

2122.93	-4354	1.34E-04	1301	0.333333	3.282204	908928	VANGL2
1828.77	-4354	1.34E-04	1332	0.333333	3.281975	844270	LDLR
2455.482	-4354	1.34E-04	1318	0.333333	3.281975	941262	KIF3A
2063.069	-4354	1.34E-04	1263	0.333333	3.281975	829342	IFNL1
1977.84	-4354	1.34E-04	1458	0.333333	3.281745	870828	CD28
1807.342	-4354	1.34E-04	1438	0.333333	3.281745	817218	TBX5
2109.68	-4354	1.34E-04	1362	0.333333	3.281745	860062	MTMR6
2286.409	-4354	1.34E-04	1248	0.333333	3.281745	905192	CRACR2B
2106.796	-4354	1.34E-04	1360	0.333333	3.281515	868642	RAP2C
1880.699	-4354	1.34E-04	1334	0.333333	3.281515	873868	MAP3K12
2128.191	-4354	1.34E-04	1458	0.333333	3.281286	862052	TAB2
2079.945	-4354	1.34E-04	1441	0.333333	3.281286	902670	E2F2
1870.475	-4354	1.34E-04	1407	0.333333	3.281286	857474	EPOR
2203.188	-4354	1.34E-04	1402	0.333333	3.281286	855652	AP1M1
2174.405	-4354	1.34E-04	1590	0.333333	3.281056	855662	PSMD1
2636.268	-4354	1.34E-04	1559	0.333333	3.281056	934432	CDC42
2235.349	-4354	1.34E-04	1524	0.333333	3.281056	856864	ACTR2
1672.065	-4354	1.34E-04	1414	0.333333	3.281056	795816	CFLAR
2120.61	-4354	1.34E-04	1394	0.333333	3.281056	900750	APAF1
1807.901	-4354	1.34E-04	1516	0.333333	3.280827	787616	KNG1
1952.507	-4354	1.34E-04	1471	0.333333	3.280827	866454	CBLB
2332.152	-4354	1.34E-04	1309	0.333333	3.280827	924082	ACOT4
1677.224	-4354	1.34E-04	1307	0.333333	3.280827	763496	FGF16
1697.37	-4354	1.34E-04	1299	0.333333	3.280827	802716	PLCE1
2531.71	-4354	1.34E-04	1502	0.333333	3.280597	909366	PDHA1
2191.563	-4354	1.34E-04	1416	0.333333	3.280597	874086	LAMTOR3
1723.198	-4354	1.34E-04	1394	0.333333	3.280597	790754	EPHA3
1717.303	-4354	1.34E-04	1266	0.333333	3.280597	837760	CST6
1799.511	-4354	1.34E-04	1360	0.333333	3.280367	827360	ARSA
2230.752	-4354	1.34E-04	1344	0.333333	3.280367	888112	MTMR4
1741.097	-4354	1.34E-04	1311	0.333333	3.280367	834314	GYG2
2299.091	-4354	1.34E-04	1301	0.333333	3.280367	901552	ATG10
4253.069	-4354	1.34E-04	1475	0.333333	3.280138	1747058	ENO1
2163.339	-4354	1.34E-04	1402	0.333333	3.280138	884130	HSP90B1
2106.079	-4354	1.34E-04	1363	0.333333	3.280138	892282	DGKE
2285.221	-4354	1.34E-04	1508	0.333333	3.279908	892524	MCM6
1999.783	-4354	1.34E-04	1432	0.333333	3.279908	877154	CTTN
1939.032	-4354	1.34E-04	1423	0.333333	3.279908	829042	TANK
2041.476	-4354	1.34E-04	1345	0.333333	3.279908	847376	IL20RA
2238.352	-4354	1.34E-04	1322	0.333333	3.279908	871884	MOB1B
2302.678	-4354	1.34E-04	1392	0.333333	3.279679	874372	POMP

2024.783	-4354	1.34E-04	1335	0.333333	3.279679	842238	LLGL1
2050.219	-4354	1.34E-04	1464	0.333333	3.279449	858746	FASLG
1650.043	-4354	1.34E-04	1371	0.333333	3.279449	800892	L1CAM
2561.498	-4354	1.34E-04	1335	0.333333	3.279449	908344	DAPK3
2398.816	-4354	1.34E-04	1552	0.333333	3.279219	894838	PKN2
2067.365	-4354	1.34E-04	1438	0.333333	3.279219	870514	PPP2R2A
2444.264	-4354	1.34E-04	1363	0.333333	3.279219	880140	PVR
1869.206	-4354	1.34E-04	1387	0.333333	3.27899	817470	SLC1A2
1828.703	-4354	1.34E-04	1369	0.333333	3.27899	828238	SAV1
1961.759	-4354	1.33E-04	1447	0.333333	3.27876	817056	CYP4A11
1871.34	-4354	1.33E-04	1424	0.333333	3.27876	811062	FYB1
2059.808	-4354	1.33E-04	1468	0.333333	3.27853	870182	BMPR1A
1846.154	-4354	1.33E-04	1414	0.333333	3.27853	800928	TRPC4
1895.363	-4354	1.33E-04	1364	0.333333	3.27853	839620	TLR7
2266.91	-4354	1.33E-04	1437	0.333333	3.278301	877472	PRICKLE1
4280.722	-4354	1.33E-04	1380	0.333333	3.278301	1692352	FBXO4
1606.795	-4354	1.33E-04	1352	0.333333	3.278301	791390	ABCA1
1986.687	-4354	1.33E-04	1353	0.333333	3.278071	855006	CAMK2G
2495.694	-4354	1.33E-04	1305	0.333333	3.278071	909706	LIMK1
2317.187	-4354	1.33E-04	1285	0.333333	3.278071	918502	LIPE
2400.615	-4354	1.33E-04	1281	0.333333	3.278071	897110	EGLN2
1803.636	-4354	1.33E-04	1259	0.333333	3.278071	808312	VAMP5
2366.741	-4354	1.33E-04	1551	0.333333	3.277842	885594	IKBKB
2377.988	-4354	1.33E-04	1366	0.333333	3.277842	845364	CHRD
2183.265	-4354	1.33E-04	1273	0.333333	3.277842	833948	OTX1
2188.926	-4354	1.33E-04	1438	0.333333	3.277612	825088	TUBB
1667.379	-4354	1.33E-04	1430	0.333333	3.277612	778702	HLA-DPB1
2027.554	-4354	1.33E-04	1332	0.333333	3.277612	855358	ZBTB17
1693.918	-4354	1.33E-04	1304	0.333333	3.277612	805632	SFXN3
1762.31	-4354	1.33E-04	1298	0.333333	3.277612	831018	CLMN
1644.762	-4354	1.33E-04	1308	0.333333	3.277382	763194	VAMP3
1841.326	-4354	1.33E-04	1275	0.333333	3.277382	804332	NFATC4
1973.706	-4354	1.33E-04	1273	0.333333	3.277382	846078	GLS2
1959.287	-4354	1.33E-04	1432	0.333333	3.277153	803824	IL2
2894.536	-4354	1.33E-04	1414	0.333333	3.277153	950948	BRCA2
1981.836	-4354	1.33E-04	1277	0.333333	3.277153	834558	TAZ
2022.128	-4354	1.33E-04	1252	0.333333	3.277153	855246	GLUD2
1542.507	-4354	1.33E-04	1249	0.333333	3.277153	763522	NOX4
1848.777	-4354	1.33E-04	1444	0.333333	3.276923	828506	RHOJ
1954.003	-4354	1.33E-04	1413	0.333333	3.276923	850726	STAT2
2324.6	-4354	1.33E-04	1355	0.333333	3.276923	861260	PGM2

1720.936	-4354	1.33E-04	1330	0.333333	3.276923	791618	ADAM17
2029.864	-4354	1.33E-04	1319	0.333333	3.276923	872822	HSPA1L
2056.334	-4354	1.33E-04	1284	0.333333	3.276923	850658	FREM2
2052.94	-4354	1.33E-04	1241	0.333333	3.276923	863020	LRRC23
2186.473	-4354	1.33E-04	1474	0.333333	3.276693	870058	TFDP1
2241.247	-4354	1.33E-04	1388	0.333333	3.276693	858898	CUL7
2383.174	-4354	1.33E-04	1342	0.333333	3.276693	840844	NAGLU
1842.949	-4354	1.33E-04	1305	0.333333	3.276693	838406	MEGF8
2010.088	-4354	1.33E-04	1284	0.333333	3.276693	864488	CALML4
2284.79	-4354	1.33E-04	1483	0.333333	3.276464	872728	RHOT1
1906.829	-4354	1.33E-04	1374	0.333333	3.276464	841720	ACSL6
2173.568	-4354	1.33E-04	1302	0.333333	3.276464	865250	E2F6
2451.332	-4354	1.33E-04	1650	0.333333	3.276234	909984	CHEK1
1976.285	-4354	1.33E-04	1425	0.333333	3.276234	849418	PML
2627.479	-4354	1.33E-04	1301	0.333333	3.276234	863958	PPIP5K2
1622.954	-4354	1.33E-04	1280	0.333333	3.276234	778470	HLA-DRB4
1682.479	-4354	1.33E-04	1260	0.333333	3.276234	732766	TRPV5
2301.787	-4354	1.33E-04	1255	0.333333	3.276234	966032	PLA2G2F
1918.734	-4354	1.33E-04	1478	0.333333	3.276005	833064	CD247
2439.8	-4354	1.33E-04	1421	0.333333	3.276005	899732	VAPB
2018.88	-4354	1.33E-04	1343	0.333333	3.276005	848582	IL17D
2173.135	-4354	1.33E-04	1304	0.333333	3.276005	842728	EIF4G2
1885.88	-4354	1.33E-04	1283	0.333333	3.276005	823702	LTB
2499.085	-4354	1.33E-04	1556	0.333333	3.275775	908554	CDC6
1716.526	-4354	1.33E-04	1356	0.333333	3.275775	811116	JDP2
1922.81	-4354	1.33E-04	1302	0.333333	3.275545	806844	CD164
1980.47	-4354	1.33E-04	1247	0.333333	3.275545	795008	IFNL2
3380.52	-4354	1.33E-04	1397	0.333333	3.275316	1276806	PPP1R3B
1744.87	-4354	1.33E-04	1359	0.333333	3.275316	814550	EVC
1570.608	-4354	1.33E-04	1289	0.333333	3.275316	782174	CDKN1C
2058.139	-4354	1.33E-04	1276	0.333333	3.275316	842090	PLXNA3
2161.141	-4354	1.33E-04	1457	0.333333	3.275086	842482	DNM1L
1698.14	-4354	1.33E-04	1407	0.333333	3.275086	780964	ADCY1
2125.632	-4354	1.33E-04	1391	0.333333	3.275086	840110	CCNC
1850.324	-4354	1.33E-04	1291	0.333333	3.275086	804144	NCSTN
2068.015	-4354	1.33E-04	1263	0.333333	3.275086	813946	DHH
1910.463	-4354	1.33E-04	1363	0.333333	3.274856	809250	FREM1
2082.371	-4354	1.33E-04	1312	0.333333	3.274856	838912	PRPS1
2114.231	-4354	1.33E-04	1411	0.333333	3.274627	830730	PRMT1
2104.82	-4354	1.33E-04	1402	0.333333	3.274627	848834	PIKFYVE
1863.037	-4354	1.33E-04	1363	0.333333	3.274627	788836	GDNF

2109.99	-4354	1.33E-04	1346	0.333333	3.274627	875818	MLYCD
1794.361	-4354	1.33E-04	1292	0.333333	3.274627	725682	CRX
1948.371	-4354	1.33E-04	1293	0.333333	3.274397	813470	CDC34
1969.513	-4354	1.33E-04	1269	0.333333	3.274397	846126	SORD
1705.487	-4354	1.33E-04	1392	0.333333	3.274168	780696	SEMA3D
1803.372	-4354	1.33E-04	1349	0.333333	3.274168	817834	RAP1GAP
2027.841	-4354	1.33E-04	1290	0.333333	3.274168	802344	IMPA1
2135.469	-4354	1.33E-04	1426	0.333333	3.273938	844050	AZI2
1862.297	-4354	1.33E-04	1356	0.333333	3.273938	836152	FGD3
1832.611	-4354	1.33E-04	1354	0.333333	3.273938	780818	UBE2A
1922.7	-4354	1.33E-04	1258	0.333333	3.273938	844498	OCLN
1713.092	-4354	1.33E-04	1372	0.333333	3.273708	770550	RARB
1806.566	-4354	1.33E-04	1355	0.333333	3.273708	785630	SLIT1
2082.962	-4354	1.33E-04	1275	0.333333	3.273708	824634	PRPF19
1685.466	-4354	1.33E-04	1408	0.333333	3.273479	772428	CR1
2238.992	-4354	1.33E-04	1393	0.333333	3.273479	865150	STRADA
2488.471	-4354	1.33E-04	1382	0.333333	3.273479	911214	RPAP2
2261.413	-4354	1.33E-04	1451	0.333333	3.273249	847642	STAG1
1799.059	-4354	1.33E-04	1392	0.333333	3.273249	805428	SLC40A1
2137.061	-4354	1.33E-04	1367	0.333333	3.273249	859630	TFDP2
1873.782	-4354	1.33E-04	1308	0.333333	3.273249	809796	PIK3R6
1859.522	-4354	1.33E-04	1273	0.333333	3.273249	807076	DACT2
1744.903	-4354	1.33E-04	1257	0.333333	3.273249	789630	FBXO2
2338.692	-4354	1.33E-04	1247	0.333333	3.273249	859536	LIN37
2244.396	-4354	1.33E-04	1371	0.333333	3.27302	835046	HEXA
1849.837	-4354	1.33E-04	1343	0.333333	3.27302	783828	MAG
1868	-4354	1.33E-04	1252	0.333333	3.27302	796478	CD46
1741.821	-4354	1.33E-04	1323	0.333333	3.27279	781190	RASSF5
1954.809	-4354	1.33E-04	1304	0.333333	3.27279	808988	ATP6V1G1
1694.346	-4354	1.33E-04	1350	0.333333	3.27256	793264	CDS2
1791.564	-4354	1.33E-04	1316	0.333333	3.27256	788112	DNM2
1749.05	-4354	1.33E-04	1274	0.333333	3.27256	813214	TBC1D16
2577.227	-4354	1.33E-04	1423	0.333333	3.272331	850020	NDUFS3
2101.282	-4354	1.33E-04	1382	0.333333	3.272331	819456	SUCLG1
2086.761	-4354	1.33E-04	1428	0.333333	3.272101	866398	ACACA
2887.749	-4354	1.33E-04	1405	0.333333	3.272101	884686	NDUFB3
2224.399	-4354	1.33E-04	1373	0.333333	3.272101	879046	CCNJ
1517.493	-4354	1.33E-04	1321	0.333333	3.272101	725910	HLA-DPA1
1590.453	-4354	1.33E-04	1281	0.333333	3.272101	761836	GNLY
2308.976	-4354	1.33E-04	1269	0.333333	3.272101	892826	EDARADD
2033.296	-4354	1.33E-04	1365	0.333333	3.271871	820440	CD274

1805.799	-4354	1.33E-04	1340	0.333333	3.271871	790996	BCL10
1380.379	-4354	1.33E-04	1229	0.333333	3.271871	681068	ATP4B
1727.006	-4354	1.33E-04	1443	0.333333	3.271642	744212	CCKAR
2111.622	-4354	1.33E-04	1376	0.333333	3.271642	836986	PI4KB
1845.402	-4354	1.33E-04	1373	0.333333	3.271642	823464	GGA2
1891.525	-4354	1.33E-04	1339	0.333333	3.271642	833028	GNPTAB
1794.237	-4354	1.33E-04	1318	0.333333	3.271642	820196	ACOX1
1831.237	-4354	1.33E-04	1305	0.333333	3.271642	806896	SOS2
2133.515	-4354	1.33E-04	1564	0.333333	3.271412	813928	UBE2D1
2105.854	-4354	1.33E-04	1492	0.333333	3.271412	834870	NCOA2
2072.945	-4354	1.33E-04	1349	0.333333	3.271412	828038	AGA
1832.765	-4354	1.33E-04	1309	0.333333	3.271412	826138	SLC11A2
2318.199	-4354	1.33E-04	1300	0.333333	3.271412	857886	CACNG4
1933.504	-4354	1.33E-04	1286	0.333333	3.271412	792270	DNAJC3
2332.487	-4354	1.33E-04	1265	0.333333	3.271412	837612	ENDOG
2154.848	-4354	1.33E-04	1256	0.333333	3.271412	819814	SLC27A4
1849.251	-4354	1.33E-04	1305	0.333333	3.271183	784694	POU2F2
1605.182	-4354	1.33E-04	1275	0.333333	3.271183	764968	LRPAP1
2088.851	-4354	1.33E-04	1274	0.333333	3.271183	841858	SENP2
1691.15	-4354	1.33E-04	1274	0.333333	3.270953	774724	ITGAD
2123.471	-4354	1.33E-04	1224	0.333333	3.270953	846672	SRD5A3
2217.878	-4354	1.33E-04	1360	0.333333	3.270723	856600	POU2F1
4321.819	-4354	1.33E-04	1348	0.333333	3.270723	1762748	ENO3
1799.581	-4354	1.33E-04	1346	0.333333	3.270723	787026	TRPC1
1832.254	-4354	1.33E-04	1316	0.333333	3.270723	760046	NTN1
1795.303	-4354	1.33E-04	1287	0.333333	3.270723	810236	HK2
1978.051	-4354	1.33E-04	1195	0.333333	3.270723	827462	PTTG2
1755.812	-4354	1.33E-04	1354	0.333333	3.270494	710442	ADRB3
3042.584	-4354	1.33E-04	1431	0.333333	3.270264	900764	NDUFA4
1643.163	-4354	1.33E-04	1319	0.333333	3.270264	757260	NECTIN1
1671.668	-4354	1.33E-04	1307	0.333333	3.270264	773664	CD1E
1887.762	-4354	1.33E-04	1271	0.333333	3.270264	774002	STX1B
1781.389	-4354	1.33E-04	1203	0.333333	3.270264	761074	PTCRA
2210.129	-4354	1.33E-04	1461	0.333333	3.270034	808474	HADHA
1879.847	-4354	1.33E-04	1388	0.333333	3.270034	783482	VAPA
2248.491	-4354	1.33E-04	1377	0.333333	3.270034	820586	FAF1
1792.419	-4354	1.33E-04	1366	0.333333	3.270034	788356	CCNG2
2042.419	-4354	1.33E-04	1342	0.333333	3.270034	832120	CXCL11
1684.353	-4354	1.33E-04	1232	0.333333	3.270034	747266	MX2
2264.009	-4354	1.33E-04	1419	0.333333	3.269805	837250	CSNK1G3
1632.72	-4354	1.33E-04	1301	0.333333	3.269805	760530	FLOT1

2073.959	-4354	1.33E-04	1283	0.333333	3.269805	811568	GSTM4
1933.549	-4354	1.33E-04	1311	0.333333	3.269575	810304	AMACR
1649.141	-4354	1.33E-04	1262	0.333333	3.269575	797654	SYNJ2
1600.662	-4354	1.33E-04	1224	0.333333	3.269575	770284	NLRX1
2048.902	-4354	1.33E-04	1408	0.333333	3.269346	832276	XCL1
1879.203	-4354	1.33E-04	1370	0.333333	3.269346	799708	NCKAP1
1957.87	-4354	1.33E-04	1265	0.333333	3.269346	812408	NKD2
1958.032	-4354	1.33E-04	1234	0.333333	3.269346	824698	SOX8
2207.843	-4354	1.33E-04	1595	0.333333	3.269116	830846	PTPN11
1945.288	-4354	1.33E-04	1355	0.333333	3.269116	755616	GUCA1A
1512.125	-4354	1.33E-04	1298	0.333333	3.269116	738278	TNFRSF9
1962.086	-4354	1.33E-04	1239	0.333333	3.269116	800860	HSH2D
1559.177	-4354	1.33E-04	1231	0.333333	3.268886	764734	ESAM
1699.645	-4354	1.33E-04	1395	0.333333	3.268657	743376	GRIA3
2128.787	-4354	1.33E-04	1286	0.333333	3.268657	856122	ATP6V0A2
1653.642	-4354	1.33E-04	1240	0.333333	3.268657	731738	CYBA
2002.924	-4354	1.33E-04	1218	0.333333	3.268657	802794	TNFRSF13C
2865.062	-4354	1.33E-04	1475	0.333333	3.268427	898082	NDUFA10
2081.135	-4354	1.33E-04	1415	0.333333	3.268427	747704	TACR1
2122.764	-4354	1.33E-04	1315	0.333333	3.268427	815812	CREB3L3
1504.662	-4354	1.33E-04	1259	0.333333	3.268427	722688	PLN
2785.506	-4354	1.33E-04	1353	0.333333	3.268197	836166	UQCRQ
1874.634	-4354	1.33E-04	1210	0.333333	3.268197	769876	CXCL17
2159.083	-4354	1.33E-04	1427	0.333333	3.267968	818090	ITCH
1905.568	-4354	1.33E-04	1355	0.333333	3.267968	756342	PAX3
1351.774	-4354	1.33E-04	1341	0.333333	3.267968	719202	HBEGF
2112.246	-4354	1.33E-04	1506	0.333333	3.267738	819110	RBBP4
2252.231	-4354	1.33E-04	1404	0.333333	3.267738	814804	BAD
1890.804	-4354	1.33E-04	1205	0.333333	3.267738	790656	NAPSA
1886.312	-4354	1.33E-04	1364	0.333333	3.267509	779592	PRKAR1A
1740.082	-4354	1.33E-04	1284	0.333333	3.267509	762452	LGALS8
1595.978	-4354	1.33E-04	1257	0.333333	3.267509	746452	TNFRSF4
1791.527	-4354	1.33E-04	1359	0.333333	3.267279	760838	COL4A3
2013.167	-4354	1.33E-04	1268	0.333333	3.267279	828234	PHLPP2
1618.788	-4354	1.33E-04	1246	0.333333	3.267279	762272	ME1
1863.501	-4354	1.33E-04	1242	0.333333	3.267279	789066	TSLP
2047.595	-4354	1.33E-04	1406	0.333333	3.267049	798752	MCM7
1924.317	-4354	1.33E-04	1325	0.333333	3.267049	809844	HMGCS1
1978.506	-4354	1.33E-04	1324	0.333333	3.267049	827394	ELK4
1623.645	-4354	1.33E-04	1267	0.333333	3.267049	711840	MAPK4
1636.633	-4354	1.33E-04	1261	0.333333	3.267049	757144	DAG1

1999.418	-4354	1.33E-04	1212	0.333333	3.267049	830884	H2BC11
1514.443	-4354	1.33E-04	1177	0.333333	3.267049	724822	MTSS2
1985.791	-4354	1.33E-04	1434	0.333333	3.26682	758258	PSMD6
1997.921	-4354	1.33E-04	1357	0.333333	3.26682	823554	SSH2
1722.318	-4354	1.33E-04	1245	0.333333	3.26682	740158	LCAT
1626.558	-4354	1.33E-04	1246	0.333333	3.26659	758198	DIAPH1
1495.923	-4354	1.33E-04	1238	0.333333	3.26659	712392	TFE3
1826.687	-4354	1.33E-04	1230	0.333333	3.26659	785176	PARD6B
2858.078	-4354	1.33E-04	1459	0.333333	3.266361	847466	NDUFB4
2013.635	-4354	1.33E-04	1400	0.333333	3.266361	829936	CCNE1
1649.572	-4354	1.33E-04	1397	0.333333	3.266361	720448	HLA-F
1730.941	-4354	1.33E-04	1363	0.333333	3.266361	768448	BCL2L1
1805.99	-4354	1.33E-04	1273	0.333333	3.266361	757108	MYL6
1826.43	-4354	1.33E-04	1427	0.333333	3.266131	778214	DLG4
1866.926	-4354	1.33E-04	1335	0.333333	3.266131	760554	RAB10
1808.652	-4354	1.33E-04	1319	0.333333	3.266131	761348	ACVR1B
2832.349	-4354	1.33E-04	1306	0.333333	3.266131	861700	NDUFS6
2028.974	-4354	1.33E-04	1285	0.333333	3.266131	773282	PAK4
1613.684	-4354	1.33E-04	1249	0.333333	3.265901	755390	AREG
2125.083	-4354	1.33E-04	1236	0.333333	3.265901	779334	LAMTOR5
1633.163	-4354	1.32E-04	1278	0.333333	3.265672	741974	TRIM38
1881.737	-4354	1.32E-04	1174	0.333333	3.265672	775762	KIF7
1569.065	-4354	1.32E-04	1229	0.333333	3.265442	744458	SEMA3F
1823.535	-4354	1.32E-04	1344	0.333333	3.265212	774820	HHIP
2055.304	-4354	1.32E-04	1298	0.333333	3.265212	828848	LIMD1
1718.62	-4354	1.32E-04	1248	0.333333	3.265212	743572	MAP3K6
1728.982	-4354	1.32E-04	1206	0.333333	3.265212	775886	SLC51A
2046.894	-4354	1.32E-04	1464	0.333333	3.264983	784136	HDAC2
1792.573	-4354	1.32E-04	1399	0.333333	3.264983	765940	YES1
1941.411	-4354	1.32E-04	1225	0.333333	3.264983	757690	FLT3LG
1298.243	-4354	1.32E-04	1222	0.333333	3.264983	649194	BRINP1
1911.799	-4354	1.32E-04	1392	0.333333	3.264753	804464	NFATC2
1836.044	-4354	1.32E-04	1302	0.333333	3.264753	748250	UBE2G1
1705.561	-4354	1.32E-04	1260	0.333333	3.264753	720442	ARPC4
2316.49	-4354	1.32E-04	1466	0.333333	3.264524	810588	RPS6KB1
2008.816	-4354	1.32E-04	1320	0.333333	3.264524	809386	CSNK1G1
1705.533	-4354	1.32E-04	1314	0.333333	3.264524	742958	CCL13
1591.26	-4354	1.32E-04	1301	0.333333	3.264524	731788	MYO9B
2130.212	-4354	1.32E-04	1288	0.333333	3.264524	801142	PPIA
1854.566	-4354	1.32E-04	1275	0.333333	3.264524	747914	PRMT5
2065.209	-4354	1.32E-04	1273	0.333333	3.264524	816754	CDC45

1762.391	-4354	1.32E-04	1398	0.333333	3.264294	753974	CSNK1D
1902.968	-4354	1.32E-04	1370	0.333333	3.264294	794308	DICER1
1814.235	-4354	1.32E-04	1198	0.333333	3.264294	747310	AKR1A1
2082.415	-4354	1.32E-04	1506	0.333333	3.264064	781606	CCNB1
1955.551	-4354	1.32E-04	1418	0.333333	3.264064	770046	RAD21
1697.866	-4354	1.32E-04	1335	0.333333	3.264064	711262	CYP2A6
2008.293	-4354	1.32E-04	1221	0.333333	3.264064	766354	ATG16L2
1796.491	-4354	1.32E-04	1244	0.333333	3.263835	760738	NR2F6
1822.013	-4354	1.32E-04	1223	0.333333	3.263835	762116	HTRA2
2512.096	-4354	1.32E-04	1534	0.333333	3.263605	852426	BRCA1
2000.278	-4354	1.32E-04	1467	0.333333	3.263605	770934	CDC27
1863.154	-4354	1.32E-04	1313	0.333333	3.263605	741518	FGF10
2139.205	-4354	1.32E-04	1249	0.333333	3.263605	812778	TJAP1
2626.548	-4354	1.32E-04	1383	0.333333	3.263375	786630	ATP5F1D
1855.53	-4354	1.32E-04	1367	0.333333	3.263375	758348	PPP1R12A
1968.16	-4354	1.32E-04	1313	0.333333	3.263375	786380	C9orf72
1703.343	-4354	1.32E-04	1288	0.333333	3.263146	703928	PKLR
1661.426	-4354	1.32E-04	1243	0.333333	3.263146	738076	MFNG
1959.262	-4354	1.32E-04	1342	0.333333	3.262916	756812	ROCK1
1615.855	-4354	1.32E-04	1258	0.333333	3.262916	737940	RAG1
1697.724	-4354	1.32E-04	1314	0.333333	3.262687	728172	ATP6V0E1
1762.642	-4354	1.32E-04	1302	0.333333	3.262687	712020	DNAJA2
1750.952	-4354	1.32E-04	1293	0.333333	3.262687	725294	SEC23A
2131.214	-4354	1.32E-04	1267	0.333333	3.262687	795224	ORC3
1569.617	-4354	1.32E-04	1262	0.333333	3.262687	739060	ICOSLG
1539.898	-4354	1.32E-04	1223	0.333333	3.262687	708298	MGAT3
1623.176	-4354	1.32E-04	1239	0.333333	3.262457	670270	NODAL
1797.708	-4354	1.32E-04	1193	0.333333	3.262457	740914	TKFC
2053.601	-4354	1.32E-04	1352	0.333333	3.262227	819468	MKI67
2041.817	-4354	1.32E-04	1272	0.333333	3.262227	790592	FRS2
1289.322	-4354	1.32E-04	1233	0.333333	3.262227	639292	SRGN
1982.426	-4354	1.32E-04	1317	0.333333	3.261998	815876	CCNT1
1898.23	-4354	1.32E-04	1293	0.333333	3.261998	774278	CDC14A
1868.311	-4354	1.32E-04	1236	0.333333	3.261998	745522	TMED9
1841.942	-4354	1.32E-04	1220	0.333333	3.261998	731178	TECR
2665.571	-4354	1.32E-04	1386	0.333333	3.261768	814394	NDUFV1
1684.498	-4354	1.32E-04	1326	0.333333	3.261768	715548	CYP2E1
1691.217	-4354	1.32E-04	1321	0.333333	3.261768	745744	GNB4
2072.386	-4354	1.32E-04	1315	0.333333	3.261768	757524	SDHB
1579.673	-4354	1.32E-04	1314	0.333333	3.261768	710644	PTGDR2
1847.709	-4354	1.32E-04	1263	0.333333	3.261768	756444	TBC1D15

1784.24	-4354	1.32E-04	1211	0.333333	3.261768	762630	DHX58
1589.111	-4354	1.32E-04	1188	0.333333	3.261768	726842	SSH3
1717.424	-4354	1.32E-04	1185	0.333333	3.261768	739808	MS4A14
2592.721	-4354	1.32E-04	1318	0.333333	3.261538	816466	NDUFA1
2073.05	-4354	1.32E-04	1235	0.333333	3.261538	786062	SLC27A5
1466.563	-4354	1.32E-04	1235	0.333333	3.261538	675434	MS4A7
1763.863	-4354	1.32E-04	1214	0.333333	3.261538	728622	FABP7
1978.658	-4354	1.32E-04	1199	0.333333	3.261538	763110	USP21
1735.052	-4354	1.32E-04	1342	0.333333	3.261309	743982	HLA-DOB
2074.912	-4354	1.32E-04	1285	0.333333	3.261309	810348	CYP2R1
1814.017	-4354	1.32E-04	1279	0.333333	3.261309	736168	GPCPD1
2012.398	-4354	1.32E-04	1252	0.333333	3.261309	802364	ACTR3B
1655.659	-4354	1.32E-04	1297	0.333333	3.261079	759212	PRKAR2A
1677.55	-4354	1.32E-04	1238	0.333333	3.261079	752964	TRAK1
1724.964	-4354	1.32E-04	1318	0.333333	3.26085	760460	AFDN
1883.301	-4354	1.32E-04	1310	0.333333	3.26085	765568	ISG15
1761.459	-4354	1.32E-04	1299	0.333333	3.26085	719322	TRAM1
1844.022	-4354	1.32E-04	1240	0.333333	3.26085	745132	KDM3A
1377.702	-4354	1.32E-04	1222	0.333333	3.26085	653298	NPC1L1
1751.69	-4354	1.32E-04	1206	0.333333	3.26085	743998	PDK2
1641.975	-4354	1.32E-04	1182	0.333333	3.26062	734936	METRN
1525.735	-4354	1.32E-04	1272	0.333333	3.26039	669010	GBP1
1774.641	-4354	1.32E-04	1251	0.333333	3.26039	702468	DDOST
1856.585	-4354	1.32E-04	1205	0.333333	3.26039	747162	PPM1D
1882.94	-4354	1.32E-04	1194	0.333333	3.26039	754664	CASP14
1888.303	-4354	1.32E-04	1381	0.333333	3.260161	752020	MAP4K5
2060.265	-4354	1.32E-04	1317	0.333333	3.260161	796594	E2F5
1949.435	-4354	1.32E-04	1271	0.333333	3.260161	746020	ATF1
1815.297	-4354	1.32E-04	1270	0.333333	3.260161	748734	VAMP2
1733.434	-4354	1.32E-04	1257	0.333333	3.260161	719076	SOCS5
1962.155	-4354	1.32E-04	1206	0.333333	3.260161	774610	DEGS2
1597.7	-4354	1.32E-04	1504	0.333333	3.259931	724090	GNG2
1738.132	-4354	1.32E-04	1270	0.333333	3.259931	723126	USP25
1924.018	-4354	1.32E-04	1259	0.333333	3.259931	747072	DBF4
1858.878	-4354	1.32E-04	1348	0.333333	3.259701	744346	PRKCI
1787.044	-4354	1.32E-04	1301	0.333333	3.259701	740284	VEZF1
1470.176	-4354	1.32E-04	1291	0.333333	3.259701	684290	WNT2B
1686.45	-4354	1.32E-04	1259	0.333333	3.259701	705372	NLGN4Y
2521.333	-4354	1.32E-04	1468	0.333333	3.259472	756648	UQCRC2
1838.443	-4354	1.32E-04	1351	0.333333	3.259472	757782	DVL3
1675.008	-4354	1.32E-04	1297	0.333333	3.259472	733540	ATF6

1845.749	-4354	1.32E-04	1258	0.333333	3.259472	768670	PPP2R2D
1671.414	-4354	1.32E-04	1187	0.333333	3.259472	719294	ESRRA
1874.878	-4354	1.32E-04	1172	0.333333	3.259472	751210	PRMT7
1503.566	-4354	1.32E-04	1379	0.333333	3.259242	689964	COL6A1
1834.772	-4354	1.32E-04	1271	0.333333	3.259242	718700	MYL10
1956.839	-4354	1.32E-04	1229	0.333333	3.259242	794720	DGKH
2079.367	-4354	1.32E-04	1437	0.333333	3.259013	794888	BRAF
2765.994	-4354	1.32E-04	1527	0.333333	3.258783	810830	ATP5PO
1620.825	-4354	1.32E-04	1277	0.333333	3.258783	683128	CACNA1G
1435.601	-4354	1.32E-04	1210	0.333333	3.258783	707898	DUSP7
1913.933	-4354	1.32E-04	1305	0.333333	3.258553	730330	PPP2R5E
1853.329	-4354	1.32E-04	1357	0.333333	3.258324	702432	CYP2C8
1838.565	-4354	1.32E-04	1257	0.333333	3.258324	742568	UBE3C
1401.504	-4354	1.32E-04	1237	0.333333	3.258324	683022	IRF5
1497.97	-4354	1.32E-04	1206	0.333333	3.258324	646936	P2RX6
1830.895	-4354	1.32E-04	1305	0.333333	3.258094	744186	RBL2
1584.516	-4354	1.32E-04	1259	0.333333	3.258094	687316	LAMP2
1681.936	-4354	1.32E-04	1225	0.333333	3.258094	694624	POU2F3
1472.86	-4354	1.32E-04	1200	0.333333	3.258094	701556	ITPK1
1403.248	-4354	1.32E-04	1394	0.333333	3.257865	670890	COL1A1
2103.049	-4354	1.32E-04	1275	0.333333	3.257865	784072	PIK3R4
1913.14	-4354	1.32E-04	1208	0.333333	3.257865	715668	PNCK
1746.969	-4354	1.32E-04	1321	0.333333	3.257635	748918	ARHGEF7
1747.416	-4354	1.32E-04	1296	0.333333	3.257635	718272	PPP1CB
1676.623	-4354	1.32E-04	1279	0.333333	3.257635	707036	RASGRF1
1305.003	-4354	1.32E-04	1189	0.333333	3.257635	667634	SEMA6A
3035.321	-4354	1.32E-04	1160	0.333333	3.257635	1130722	PPP1R3D
1713.267	-4354	1.32E-04	1158	0.333333	3.257635	723130	CPT1C
1889.196	-4354	1.32E-04	1350	0.333333	3.257405	752066	FUS
1948.403	-4354	1.32E-04	1311	0.333333	3.257405	734734	DLAT
1633.58	-4354	1.32E-04	1294	0.333333	3.257405	688436	LATS2
1673.036	-4354	1.32E-04	1243	0.333333	3.257405	700782	GRK4
1773.634	-4354	1.32E-04	1232	0.333333	3.257405	715912	TAX1BP1
1509.46	-4354	1.32E-04	1269	0.333333	3.257176	673808	TRPA1
1740.358	-4354	1.32E-04	1215	0.333333	3.257176	745156	DLG3
2233.842	-4354	1.32E-04	1174	0.333333	3.257176	712226	VTI1B
1630.891	-4354	1.32E-04	1156	0.333333	3.257176	694862	COX4I2
1912.569	-4354	1.32E-04	1289	0.333333	3.256946	743470	PTPN2
1498.273	-4354	1.32E-04	1228	0.333333	3.256946	691596	KSR1
1760.039	-4354	1.32E-04	1480	0.333333	3.256716	730892	SMAD2
2409.129	-4354	1.32E-04	1278	0.333333	3.256487	759824	NDUFA13

1740.992	-4354	1.32E-04	1274	0.333333	3.256487	723478	MTMR2
1637.729	-4354	1.32E-04	1264	0.333333	3.256487	678736	TNR
1524.209	-4354	1.32E-04	1195	0.333333	3.256487	683328	ALDH1L1
1403.819	-4354	1.32E-04	1222	0.333333	3.256257	654148	CCN4
1422.27	-4354	1.32E-04	1220	0.333333	3.256257	659952	CYTH4
1761.817	-4354	1.32E-04	1346	0.333333	3.256028	709158	ACO2
1979.823	-4354	1.32E-04	1306	0.333333	3.256028	766628	AP4S1
2216.205	-4354	1.32E-04	1229	0.333333	3.256028	855816	SRF
1737.047	-4354	1.32E-04	1207	0.333333	3.256028	696202	CA1
1802.565	-4354	1.32E-04	1159	0.333333	3.256028	714894	HACD3
1831.631	-4354	1.32E-04	1317	0.333333	3.255798	705422	MAP2K2
1558.571	-4354	1.32E-04	1178	0.333333	3.255798	714188	CDS1
1615.455	-4354	1.32E-04	1329	0.333333	3.255568	672000	MASP1
1639.513	-4354	1.32E-04	1258	0.333333	3.255568	693842	TRADD
2015.276	-4354	1.32E-04	1312	0.333333	3.255339	711284	TMED10
1633.083	-4354	1.32E-04	1234	0.333333	3.255339	726874	HSD17B1
1958.528	-4354	1.32E-04	1314	0.333333	3.255109	747186	TCF3
1718.752	-4354	1.32E-04	1260	0.333333	3.255109	703932	LRRC4C
1640.519	-4354	1.32E-04	1183	0.333333	3.255109	718058	BCL2L13
1992.582	-4354	1.32E-04	1224	0.333333	3.254879	741830	GORAB
2117.141	-4354	1.32E-04	1189	0.333333	3.254879	686010	IDH3G
1919.196	-4354	1.32E-04	1352	0.333333	3.25465	748046	ORC2
1731.835	-4354	1.32E-04	1261	0.333333	3.25465	712270	CFL2
1314.792	-4354	1.32E-04	1249	0.333333	3.25465	648366	FGF18
1839.489	-4354	1.32E-04	1232	0.333333	3.25442	702372	USP8
1499.048	-4354	1.32E-04	1222	0.333333	3.25442	713622	ENTPD4
2084.656	-4354	1.32E-04	1188	0.333333	3.25442	765374	ESPL1
1856.032	-4354	1.32E-04	1304	0.333333	3.254191	748180	XIAP
1603.597	-4354	1.32E-04	1305	0.333333	3.253961	728868	ABI2
1423.046	-4354	1.32E-04	1273	0.333333	3.253961	625864	CASR
1651.716	-4354	1.32E-04	1351	0.333333	3.253731	701188	RAB2A
1404.915	-4354	1.32E-04	1246	0.333333	3.253731	674998	CASP10
1540.687	-4354	1.32E-04	1175	0.333333	3.253731	640724	HOXA11
1973.443	-4354	1.32E-04	1469	0.333333	3.253502	701910	PSMD12
1764.952	-4354	1.32E-04	1298	0.333333	3.253502	692506	ATP5MC3
1636.97	-4354	1.32E-04	1228	0.333333	3.253502	667594	GH1
1385.113	-4354	1.32E-04	1326	0.333333	3.253272	640344	GRM5
1462.6	-4354	1.32E-04	1280	0.333333	3.253272	663168	ARF6
2368.37	-4354	1.32E-04	1168	0.333333	3.253272	785926	PCGF2
1456.965	-4354	1.32E-04	1168	0.333333	3.253272	647384	CYTH2
1792.349	-4354	1.32E-04	1252	0.333333	3.253042	721442	RRM2

1263.073	-4354	1.32E-04	1232	0.333333	3.253042	633016	MS4A2
1673.094	-4354	1.32E-04	1198	0.333333	3.253042	714596	GALM
1947.524	-4354	1.32E-04	1169	0.333333	3.253042	757464	MBTPS2
1721.779	-4354	1.32E-04	1166	0.333333	3.253042	659514	IL17F
1608.883	-4354	1.32E-04	1391	0.333333	3.252813	668148	VDAC1
1771.722	-4354	1.32E-04	1362	0.333333	3.252813	730034	NCOR1
1870.454	-4354	1.32E-04	1297	0.333333	3.252813	713386	PPP3CB
1470.071	-4354	1.32E-04	1244	0.333333	3.252813	668058	SFRP2
1605.916	-4354	1.32E-04	1194	0.333333	3.252813	691862	FASN
1795.54	-4354	1.31E-04	1380	0.333333	3.252583	676726	PSMA7
1602.745	-4354	1.31E-04	1176	0.333333	3.252583	721402	IL20RB
1524.42	-4354	1.31E-04	1320	0.333333	3.252354	638970	CYP2C9
1534.411	-4354	1.31E-04	1285	0.333333	3.252354	700704	SRGAP1
2107.159	-4354	1.31E-04	1269	0.333333	3.252354	723712	CUL2
1624.73	-4354	1.31E-04	1161	0.333333	3.252354	669820	CYP4Z1
2116.391	-4354	1.31E-04	1246	0.333333	3.252124	744760	ATG4C
1497.846	-4354	1.31E-04	1285	0.333333	3.251894	637610	PTGER1
1957.068	-4354	1.31E-04	1253	0.333333	3.251894	742634	FXN
1734.767	-4354	1.31E-04	1224	0.333333	3.251894	720624	SLC7A1
1261.054	-4354	1.31E-04	1259	0.333333	3.251665	623044	SOCS3
1534.393	-4354	1.31E-04	1207	0.333333	3.251665	690750	MBTPS1
1537.94	-4354	1.31E-04	1204	0.333333	3.251665	643156	DMP1
1991.106	-4354	1.31E-04	1194	0.333333	3.251665	730292	TELO2
1531.733	-4354	1.31E-04	1288	0.333333	3.251435	641290	GRM8
1595.728	-4354	1.31E-04	1246	0.333333	3.251435	706238	ARHGAP35
1584.313	-4354	1.31E-04	1207	0.333333	3.251435	656844	APC2
1607.523	-4354	1.31E-04	1195	0.333333	3.251435	720478	WASL
1782.907	-4354	1.31E-04	1266	0.333333	3.251206	702434	SLC8A3
1566.027	-4354	1.31E-04	1214	0.333333	3.251206	693732	IQCE
1791.011	-4354	1.31E-04	1213	0.333333	3.251206	686410	RNF7
1964.749	-4354	1.31E-04	1193	0.333333	3.251206	736680	ASNS
1942.066	-4354	1.31E-04	1174	0.333333	3.251206	729638	PKN3
1297.773	-4354	1.31E-04	1147	0.333333	3.251206	601182	TRPM8
1444.476	-4354	1.31E-04	1139	0.333333	3.251206	664800	RGS14
1714.337	-4354	1.31E-04	1252	0.333333	3.250746	668786	COP1
1212.253	-4354	1.31E-04	1141	0.333333	3.250746	615610	SIGLEC1
1326.094	-4354	1.31E-04	1244	0.333333	3.250517	632720	TBXA2R
1856.211	-4354	1.31E-04	1192	0.333333	3.250517	702224	AP1G2
1720.027	-4354	1.31E-04	1116	0.333333	3.250517	685618	G6PC3
1292.274	-4354	1.31E-04	1204	0.333333	3.250287	619666	TPP1
1546.235	-4354	1.31E-04	1186	0.333333	3.250287	656036	DGKZ

1702.061	-4354	1.31E-04	1160	0.333333	3.250287	698924	SLC19A1
1722.16	-4354	1.31E-04	1252	0.333333	3.250057	692470	PPP5C
1868.423	-4354	1.31E-04	1241	0.333333	3.250057	723080	CARD11
1419.654	-4354	1.31E-04	1176	0.333333	3.250057	625776	HOXD1
1303.224	-4354	1.31E-04	1156	0.333333	3.250057	618530	WNT7B
2180.385	-4354	1.31E-04	1352	0.333333	3.249828	734318	RPS27A
1516.4	-4354	1.31E-04	1270	0.333333	3.249828	639546	ATP2B2
1488.758	-4354	1.31E-04	1203	0.333333	3.249828	629140	CDH15
1404.057	-4354	1.31E-04	1163	0.333333	3.249828	648420	GLG1
1550.077	-4354	1.31E-04	1234	0.333333	3.249598	633802	MOB1A
1693.44	-4354	1.31E-04	1221	0.333333	3.249598	683372	WDR41
1379.891	-4354	1.31E-04	1221	0.333333	3.249598	650338	PAX8
1723.341	-4354	1.31E-04	1210	0.333333	3.249598	691684	NDRG3
1363.31	-4354	1.31E-04	1183	0.333333	3.249598	640986	ACSBG1
1859.199	-4354	1.31E-04	1276	0.333333	3.249369	679570	IDH3A
2005.763	-4354	1.31E-04	1161	0.333333	3.249369	729858	ACYP1
1592.01	-4354	1.31E-04	1161	0.333333	3.249369	698624	PER2
1483.792	-4354	1.31E-04	1157	0.333333	3.249369	650724	COMT
2370.427	-4354	1.31E-04	1349	0.333333	3.249139	759328	ANAPC4
1545.11	-4354	1.31E-04	1282	0.333333	3.249139	684702	DLL1
2563.843	-4354	1.31E-04	1257	0.333333	3.249139	749608	NDUFA3
1771.436	-4354	1.31E-04	1230	0.333333	3.249139	676514	ADH5
1835.385	-4354	1.31E-04	1520	0.333333	3.24868	684334	STAT1
1777.223	-4354	1.31E-04	1312	0.333333	3.24868	692864	STK11
1496.643	-4354	1.31E-04	1198	0.333333	3.24822	638182	CALML3
1384.478	-4354	1.31E-04	1139	0.333333	3.24822	636138	PRKAR1B
2500.109	-4354	1.31E-04	1131	0.333333	3.247761	774046	PCGF1
1723.62	-4354	1.31E-04	1427	0.333333	3.247532	691432	SMAD4
1662.318	-4354	1.31E-04	1277	0.333333	3.247532	706288	PIAS2
1292.372	-4354	1.31E-04	1275	0.333333	3.247532	614318	CXCR6
1513.733	-4354	1.31E-04	1245	0.333333	3.247532	622444	CYP1A2
1840.397	-4354	1.31E-04	1175	0.333333	3.247532	700328	NOG
1576.163	-4354	1.31E-04	1312	0.333333	3.247302	684448	CRKL
1859.555	-4354	1.31E-04	1277	0.333333	3.247302	711126	KMT2A
1963.741	-4354	1.31E-04	1231	0.333333	3.247072	693326	BIRC6
1900.208	-4354	1.31E-04	1220	0.333333	3.247072	691884	EBP
1925.418	-4354	1.31E-04	1198	0.333333	3.247072	719330	UBIAD1
1703.995	-4354	1.31E-04	1113	0.333333	3.247072	658462	UBE2Q2
1946.412	-4354	1.31E-04	1263	0.333333	3.246843	722416	RPTOR
1559.091	-4354	1.31E-04	1221	0.333333	3.246843	685256	DUSP16
1480.047	-4354	1.31E-04	1196	0.333333	3.246843	627426	ATP2B3

1434.182	-4354	1.31E-04	1169	0.333333	3.246843	651952	SPN
1680.26	-4354	1.31E-04	1166	0.333333	3.246613	652212	CLDN18
1344.906	-4354	1.31E-04	1261	0.333333	3.246383	602760	FGF5
1525.962	-4354	1.31E-04	1248	0.333333	3.246383	648412	CALR
1863.47	-4354	1.31E-04	1152	0.333333	3.246383	717612	CENPJ
1921.891	-4354	1.31E-04	1657	0.333333	3.246154	697002	CDK1
1613.648	-4354	1.31E-04	1233	0.333333	3.246154	657688	SHC4
1440.985	-4354	1.31E-04	1217	0.333333	3.246154	654498	MID1
1659.95	-4354	1.31E-04	1204	0.333333	3.246154	677016	ELK1
1623.388	-4354	1.31E-04	1164	0.333333	3.246154	647290	SEC22B
1302.901	-4354	1.31E-04	1147	0.333333	3.246154	593308	IL11
1471.361	-4354	1.31E-04	1122	0.333333	3.246154	672960	DLG5
1631.895	-4354	1.31E-04	1192	0.333333	3.245924	670852	TXNRD2
1661.231	-4354	1.31E-04	1132	0.333333	3.245924	649654	PRKAG3
1789.833	-4354	1.31E-04	1109	0.333333	3.245924	682548	SLC2A11
1835.853	-4354	1.31E-04	1261	0.333333	3.245695	685298	SDHD
1371.76	-4354	1.31E-04	1248	0.333333	3.245695	610074	HTR7
1719.108	-4354	1.31E-04	1193	0.333333	3.245695	674304	AP1S1
1556.319	-4354	1.31E-04	1190	0.333333	3.245695	666898	ULK2
1404.247	-4354	1.31E-04	1130	0.333333	3.245695	640384	ZBP1
1253.397	-4354	1.31E-04	1127	0.333333	3.245235	624966	CDC14B
1582.518	-4354	1.31E-04	1122	0.333333	3.245235	658250	GGA3
1763.032	-4354	1.31E-04	1258	0.333333	3.245006	657114	SEH1L
1382.292	-4354	1.31E-04	1170	0.333333	3.245006	637362	IL27RA
1780.049	-4354	1.31E-04	1155	0.333333	3.245006	693604	CCNB3
1261.051	-4354	1.31E-04	1107	0.333333	3.245006	559794	SV2C
1707.284	-4354	1.31E-04	1278	0.333333	3.244776	698622	TP53BP1
1240.848	-4354	1.31E-04	1212	0.333333	3.244776	563210	MC2R
2680.247	-4354	1.31E-04	1121	0.333333	3.244776	972480	CHMP4C
1443.991	-4354	1.31E-04	1189	0.333333	3.244546	652322	CR2
1201.847	-4354	1.31E-04	1160	0.333333	3.244546	596124	FLT4
1448.824	-4354	1.31E-04	1189	0.333333	3.244317	636274	UBE2R2
1455.458	-4354	1.31E-04	1155	0.333333	3.244317	633036	RYK
1309.423	-4354	1.31E-04	1077	0.333333	3.244317	579992	COX6B2
1331.536	-4354	1.31E-04	1183	0.333333	3.244087	579162	ADH6
1723.591	-4354	1.31E-04	1151	0.333333	3.244087	657196	EIF2A
1704.86	-4354	1.31E-04	1119	0.333333	3.244087	646304	EI24
1590.783	-4354	1.31E-04	1236	0.333333	3.243858	642724	DAXX
1458.436	-4354	1.31E-04	1197	0.333333	3.243858	645008	TNFRSF10A
1562.442	-4354	1.31E-04	1185	0.333333	3.243858	633572	NKX3-1
1603.822	-4354	1.31E-04	1388	0.333333	3.243628	662298	CREB1

1602.659	-4354	1.31E-04	1327	0.333333	3.243628	625958	PSME3
1769.302	-4354	1.31E-04	1215	0.333333	3.243628	665880	TSC2
1902.599	-4354	1.31E-04	1187	0.333333	3.243628	690312	CBR4
1174.11	-4354	1.31E-04	1120	0.333333	3.243398	581034	RAPGEF3
1779.858	-4354	1.31E-04	1086	0.333333	3.243398	693440	ZNF41
1423.876	-4354	1.31E-04	1311	0.333333	3.243169	610734	HNF4A
1653.622	-4354	1.31E-04	1172	0.333333	3.243169	648786	NSDHL
1427.78	-4354	1.31E-04	1212	0.333333	3.242939	631220	PAX5
1468.52	-4354	1.31E-04	1203	0.333333	3.242939	618234	IL23A
1480.365	-4354	1.31E-04	1187	0.333333	3.242939	628598	PRKD3
1474.833	-4354	1.31E-04	1108	0.333333	3.242939	653334	FLCN
1377.025	-4354	1.31E-04	1198	0.333333	3.24271	626614	AGRN
1717.202	-4354	1.31E-04	1188	0.333333	3.24271	643252	RALBP1
1533.262	-4354	1.31E-04	1121	0.333333	3.24271	587378	MYLK3
1668.242	-4354	1.31E-04	1165	0.333333	3.24248	659728	RAB33B
1536.07	-4354	1.31E-04	1128	0.333333	3.24248	662906	MAP3K13
1418.433	-4354	1.31E-04	1126	0.333333	3.24248	623844	COQ10B
1530.384	-4354	1.31E-04	1166	0.333333	3.24225	659200	ZFYVE9
1155.954	-4354	1.31E-04	1082	0.333333	3.24225	580932	AKR1C1
1543.709	-4354	1.31E-04	1185	0.333333	3.242021	647690	FKBP4
1602.218	-4354	1.31E-04	1174	0.333333	3.242021	628506	CUL4B
1098.987	-4354	1.31E-04	1159	0.333333	3.242021	579294	MYH11
1552.879	-4354	1.31E-04	1116	0.333333	3.242021	654994	PCSK9
1781.83	-4354	1.31E-04	1095	0.333333	3.242021	651466	MCAT
1480.116	-4354	1.31E-04	1164	0.333333	3.241791	655268	CGN
1662.692	-4354	1.31E-04	1155	0.333333	3.241791	647620	MFN1
1410.668	-4354	1.31E-04	1151	0.333333	3.241791	627708	ZFYVE16
1253.987	-4354	1.31E-04	1188	0.333333	3.241561	574744	CSF2
1372.702	-4354	1.31E-04	1103	0.333333	3.241561	593154	ABCA2
1832.974	-4354	1.31E-04	1195	0.333333	3.241332	691228	VTI1A
1376.357	-4354	1.31E-04	1141	0.333333	3.241332	619674	RASGRP2
1136.838	-4354	1.31E-04	1120	0.333333	3.241332	538294	NR0B1
1405.209	-4354	1.31E-04	1242	0.333333	3.241102	598822	NOS1
1502.866	-4354	1.31E-04	1165	0.333333	3.241102	629312	JUND
1494.118	-4354	1.31E-04	1194	0.333333	3.240873	636186	CLN5
1618.338	-4354	1.31E-04	1171	0.333333	3.240873	645622	RXRB
1478.733	-4354	1.31E-04	1141	0.333333	3.240873	620656	SOST
1618.7	-4354	1.31E-04	1140	0.333333	3.240873	656258	SSX2IP
2138.727	-4354	1.31E-04	1296	0.333333	3.240643	673180	NDUFB9
1622.237	-4354	1.31E-04	1233	0.333333	3.240643	644798	PRKAA1
1445.08	-4354	1.31E-04	1148	0.333333	3.240643	636246	EOMES

1533.074	-4354	1.31E-04	1071	0.333333	3.240643	624030	ACOT8
1474.691	-4354	1.31E-04	1186	0.333333	3.240413	623580	MYCN
1627.857	-4354	1.31E-04	1371	0.333333	3.240184	618232	PSMB7
1708.615	-4354	1.31E-04	1304	0.333333	3.240184	650264	SMC3
1048.985	-4354	1.31E-04	1067	0.333333	3.240184	521354	ALPG
1762.478	-4354	1.31E-04	1330	0.333333	3.239954	662374	ATF2
1443.899	-4354	1.31E-04	1181	0.333333	3.239954	627298	PCBP2
1622.958	-4354	1.31E-04	1171	0.333333	3.239954	650332	OSBPL5
3136.447	-4354	1.31E-04	1147	0.333333	3.239954	1338872	EIF4E2
1666.802	-4354	1.30E-04	1209	0.333333	3.239265	654404	TRIM37
1156.156	-4354	1.30E-04	1195	0.333333	3.239265	564194	COL4A6
1199.574	-4354	1.30E-04	1174	0.333333	3.239036	594866	NR4A1
1429.712	-4354	1.30E-04	1154	0.333333	3.239036	585174	CYP2B6
1517.124	-4354	1.30E-04	1076	0.333333	3.239036	624214	SLC51B
1785.972	-4354	1.30E-04	1294	0.333333	3.238806	624616	MDH2
1564.163	-4354	1.30E-04	1181	0.333333	3.238806	634336	HSD17B12
1714.152	-4354	1.30E-04	1163	0.333333	3.238576	634164	PLCZ1
1481.889	-4354	1.30E-04	1133	0.333333	3.238576	613674	RAB11B
1397.194	-4354	1.30E-04	1111	0.333333	3.238576	598846	IL22RA2
1158.766	-4354	1.30E-04	1132	0.333333	3.238347	545908	LAMP1
1293.346	-4354	1.30E-04	1172	0.333333	3.238117	553840	CYP2D6
1429.591	-4354	1.30E-04	1144	0.333333	3.238117	611276	PLXNA1
1876.956	-4354	1.30E-04	1193	0.333333	3.237887	669148	PAAF1
1423.692	-4354	1.30E-04	1165	0.333333	3.237887	595128	CYLD
1356.506	-4354	1.30E-04	1159	0.333333	3.237887	597826	ATP6V1D
2234.586	-4354	1.30E-04	1145	0.333333	3.237887	857110	BECN1
1322.765	-4354	1.30E-04	1084	0.333333	3.237658	594532	CTNS
1555.691	-4354	1.30E-04	1131	0.333333	3.237428	647818	CBY1
1167.608	-4354	1.30E-04	1119	0.333333	3.237428	565100	GPR161
1451.893	-4354	1.30E-04	1054	0.333333	3.237428	580900	IL27
1589.916	-4354	1.30E-04	1208	0.333333	3.237199	628244	ROBO2
1525.808	-4354	1.30E-04	1197	0.333333	3.237199	646072	SESN3
1285.269	-4354	1.30E-04	1100	0.333333	3.237199	590772	ATP2C2
1638.191	-4354	1.30E-04	1089	0.333333	3.237199	644876	PFKFB2
2041.895	-4354	1.30E-04	1244	0.333333	3.236969	612740	ATP5PB
1795.832	-4354	1.30E-04	1120	0.333333	3.236739	643300	PIAS4
1681.873	-4354	1.30E-04	1061	0.333333	3.236739	648508	ZNRF3
2231.656	-4354	1.30E-04	1345	0.333333	3.23628	640296	ATP5F1C
1541.986	-4354	1.30E-04	1176	0.333333	3.23628	634592	SETDB1
1629.773	-4354	1.30E-04	1164	0.333333	3.23628	633042	IP6K2
1364.07	-4354	1.30E-04	1187	0.333333	3.236051	599222	SNX18

1215.238	-4354	1.30E-04	1110	0.333333	3.236051	534802	SULT2A1
1470.346	-4354	1.30E-04	1126	0.333333	3.235821	598290	NFKBIB
1538.57	-4354	1.30E-04	1188	0.333333	3.235591	627844	RAPGEF6
1120.309	-4354	1.30E-04	1154	0.333333	3.235362	538650	NCAM1
2733.955	-4354	1.30E-04	1141	0.333333	3.235362	946834	PLA2G12A
1378.221	-4354	1.30E-04	1106	0.333333	3.235362	601292	HHATL
1502.874	-4354	1.30E-04	1226	0.333333	3.235132	602144	UBE2B
1590.029	-4354	1.30E-04	1212	0.333333	3.235132	633384	H2AX
1510.751	-4354	1.30E-04	1096	0.333333	3.235132	624548	ZNF257
1369.036	-4354	1.30E-04	1163	0.333333	3.234902	542420	CYP3A4
1650.683	-4354	1.30E-04	1259	0.333333	3.234673	640588	MCM4
1393.189	-4354	1.30E-04	1142	0.333333	3.234673	584950	RHEB
1462.176	-4354	1.30E-04	1067	0.333333	3.234673	608662	SQLE
1675.936	-4354	1.30E-04	1045	0.333333	3.234673	575900	MOGAT3
2039.057	-4354	1.30E-04	1305	0.333333	3.234443	611832	CYCS
1219.233	-4354	1.30E-04	1103	0.333333	3.234443	523288	ADCY10
1213.104	-4354	1.30E-04	1064	0.333333	3.234443	587322	MYCL
1336.255	-4354	1.30E-04	1038	0.333333	3.234443	605224	RUBCN
1541.937	-4354	1.30E-04	1151	0.333333	3.234214	620618	ACVR1C
1442.188	-4354	1.30E-04	1150	0.333333	3.233984	615946	NFATC3
1208.282	-4354	1.30E-04	1133	0.333333	3.233984	553182	EFNB3
1577.966	-4354	1.30E-04	1098	0.333333	3.233984	631810	UBE3B
1297.801	-4354	1.30E-04	1095	0.333333	3.233984	561430	YKT6
1497.598	-4354	1.30E-04	1148	0.333333	3.233754	609664	KAT6A
1478.203	-4354	1.30E-04	1129	0.333333	3.233754	609496	BAG4
1557.25	-4354	1.30E-04	1125	0.333333	3.233754	624506	CCL26
1511.584	-4354	1.30E-04	1064	0.333333	3.233754	576518	IL1F10
1330.275	-4354	1.30E-04	1107	0.333333	3.233525	580172	CD6
1249.843	-4354	1.30E-04	1084	0.333333	3.233525	535228	IL20
1732.548	-4354	1.30E-04	1239	0.333333	3.233065	608124	EIF2S2
1729.071	-4354	1.30E-04	1137	0.333333	3.233065	634766	CCNE2
1043.48	-4354	1.30E-04	1135	0.333333	3.233065	529316	KCNJ5
1173.294	-4354	1.30E-04	1065	0.333333	3.233065	558322	NCR3
1546.794	-4354	1.30E-04	1042	0.333333	3.233065	617938	A1BG
1206.497	-4354	1.30E-04	1118	0.333333	3.232836	576880	CD22
1668.202	-4354	1.30E-04	1044	0.333333	3.232836	635940	DFFB
1502.026	-4354	1.30E-04	1252	0.333333	3.232377	565296	UBE2D3
1380.743	-4354	1.30E-04	1125	0.333333	3.232377	573194	RBCK1
1477.648	-4354	1.30E-04	1123	0.333333	3.232377	597778	GCDH
1279.899	-4354	1.30E-04	1228	0.333333	3.232147	552084	RAB5A
1426.691	-4354	1.30E-04	1098	0.333333	3.232147	599430	ATF6B

1777.841	-4354	1.30E-04	1233	0.333333	3.231917	617990	CDC16
1276.792	-4354	1.30E-04	1059	0.333333	3.231917	577700	LSS
1430.314	-4354	1.30E-04	1176	0.333333	3.231688	554916	RAB11A
1208.638	-4354	1.30E-04	1157	0.333333	3.231688	528884	GFAP
1509.672	-4354	1.30E-04	1168	0.333333	3.231458	603668	UBE2W
1388.426	-4354	1.30E-04	1053	0.333333	3.231458	545512	IFNE
1144.928	-4354	1.30E-04	1030	0.333333	3.231458	527866	FNIP2
1396.127	-4354	1.30E-04	1265	0.333333	3.231228	541018	PSMD13
1480.163	-4354	1.30E-04	1143	0.333333	3.231228	593798	PPM1A
1440.944	-4354	1.30E-04	1137	0.333333	3.230999	604828	CPLX1
1438.817	-4354	1.30E-04	1133	0.333333	3.230999	598206	PPIL2
1067.949	-4354	1.30E-04	1124	0.333333	3.230999	512992	CYBB
986.572	-4354	1.30E-04	1019	0.333333	3.230999	472106	SSX2B
1135.846	-4354	1.30E-04	1220	0.333333	3.230769	534294	GRM1
1576.179	-4354	1.30E-04	1017	0.333333	3.230769	603262	SLC23A1
1550.089	-4354	1.30E-04	1261	0.333333	3.23054	607676	CCNA2
1578.857	-4354	1.30E-04	1244	0.333333	3.23054	604208	UBE2I
1480.022	-4354	1.30E-04	1187	0.333333	3.23054	578728	UBR5
1391.789	-4354	1.30E-04	1171	0.333333	3.23031	583074	LPAR5
1494.642	-4354	1.30E-04	1122	0.333333	3.23031	615770	REST
1559.233	-4354	1.30E-04	1102	0.333333	3.23031	606128	HUS1
1368.918	-4354	1.30E-04	1054	0.333333	3.23008	541228	LYPLA1
1459.506	-4354	1.30E-04	1148	0.333333	3.229851	603350	DIAPH3
1179.871	-4354	1.30E-04	1137	0.333333	3.229851	535506	EDA
1340.552	-4354	1.30E-04	1121	0.333333	3.229851	594788	SPATA13
1510.607	-4354	1.30E-04	1074	0.333333	3.229851	609824	DISP1
989.9496	-4354	1.30E-04	1063	0.333333	3.229851	496402	IGSF9B
1366.824	-4354	1.30E-04	1156	0.333333	3.229621	596754	RASGRF2
1186.511	-4354	1.30E-04	1125	0.333333	3.229621	534304	PARVG
1294.236	-4354	1.30E-04	1100	0.333333	3.229621	585212	SEMA4F
1769.417	-4354	1.30E-04	1258	0.333333	3.229392	624082	CDC25A
1490.684	-4354	1.30E-04	1184	0.333333	3.229392	586674	ELAVL1
1403.037	-4354	1.30E-04	1093	0.333333	3.229392	568074	GORASP2
1631.717	-4354	1.30E-04	1197	0.333333	3.229162	614644	MED1
1515.1	-4354	1.30E-04	1074	0.333333	3.229162	581424	PRMT6
1183.029	-4354	1.30E-04	1012	0.333333	3.229162	546724	HGSNAT
1555.013	-4354	1.30E-04	1092	0.333333	3.228703	582022	FUNDC1
1246.539	-4354	1.30E-04	1028	0.333333	3.228703	519244	ATP6V1G3
1190.13	-4354	1.30E-04	1142	0.333333	3.228473	546760	ITGA2B
985.9227	-4354	1.30E-04	1070	0.333333	3.228473	502484	CHRNA1
2163.113	-4354	1.30E-04	1188	0.333333	3.228243	639506	NDUFA7

1104.53	-4354	1.30E-04	1180	0.333333	3.228243	525322	HLA-DOA
1323.855	-4354	1.30E-04	1077	0.333333	3.228243	558640	ZBTB7A
1190.861	-4354	1.30E-04	1120	0.333333	3.228014	539368	CACNB1
935.031	-4354	1.30E-04	1126	0.333333	3.227784	497068	LAMA2
1014.426	-4354	1.30E-04	1079	0.333333	3.227784	516770	RUNX2
1113.269	-4354	1.30E-04	1071	0.333333	3.227784	528322	BAIAP2
2036.437	-4354	1.30E-04	1289	0.333333	3.227555	621532	NDUFS4
1525.136	-4354	1.30E-04	1111	0.333333	3.227555	565020	RHOT2
1497.378	-4354	1.30E-04	1075	0.333333	3.227555	590948	UBE2O
1066.21	-4354	1.30E-04	1055	0.333333	3.227555	529674	ARHGAP1
1402.002	-4354	1.30E-04	1054	0.333333	3.227555	593848	AP3M2
1094.049	-4354	1.30E-04	1030	0.333333	3.227555	499342	SEMA4G
1621.464	-4354	1.30E-04	1158	0.333333	3.227325	578554	PPID
10241.73	-4354	1.30E-04	1099	0.333333	3.227325	2500892	RPE
1417.511	-4354	1.30E-04	1099	0.333333	3.227095	576178	RNF152
1249.606	-4354	1.30E-04	1058	0.333333	3.227095	555800	TNFRSF18
1514.312	-4354	1.30E-04	1267	0.333333	3.226866	568264	FH
1450.025	-4354	1.30E-04	1211	0.333333	3.226866	573158	PSMF1
1057.749	-4354	1.30E-04	1042	0.333333	3.226866	507702	ASAH1
1136.814	-4354	1.30E-04	1145	0.333333	3.226636	494552	RHO
2590.878	-4354	1.30E-04	1126	0.333333	3.226636	655150	ATP5MF
1246.593	-4354	1.30E-04	1080	0.333333	3.226636	538208	ARHGAP5
1002.128	-4354	1.30E-04	1000	0.333333	3.226636	511526	GXYLT2
1457.609	-4354	1.30E-04	1169	0.333333	3.226406	586154	CCNB2
1146.627	-4354	1.30E-04	1097	0.333333	3.226406	520188	MPL
1394.306	-4354	1.30E-04	1043	0.333333	3.226406	530796	ABCG8
1128.538	-4354	1.30E-04	1034	0.333333	3.226406	502130	RSPO1
1661.903	-4354	1.30E-04	1243	0.333333	3.226177	586410	ATR
1311.761	-4354	1.30E-04	1138	0.333333	3.225947	542018	RAB14
1398.431	-4354	1.30E-04	1114	0.333333	3.225947	558758	MAPK8IP3
1315.723	-4354	1.30E-04	1057	0.333333	3.225947	551792	TFG
1640.533	-4354	1.30E-04	996	0.333333	3.225947	606186	DND1
1304.976	-4354	1.30E-04	1131	0.333333	3.225718	563976	FBXO32
1128.16	-4354	1.30E-04	1112	0.333333	3.225718	486616	ALDOB
1309.499	-4354	1.30E-04	1046	0.333333	3.225718	557174	BATF2
1167.472	-4354	1.30E-04	1034	0.333333	3.225718	527218	ATG13
1323.391	-4354	1.30E-04	1022	0.333333	3.225718	528892	PPP2R3C
1333.135	-4354	1.29E-04	1025	0.333333	3.225488	557360	PPP2R3B
1045.296	-4354	1.29E-04	1012	0.333333	3.225258	497034	TWIST2
1495.326	-4354	1.29E-04	1108	0.333333	3.225029	589726	TBC1D7
1414.341	-4354	1.29E-04	1067	0.333333	3.225029	564276	MYBBP1A

1869.672	-4354	1.29E-04	1104	0.333333	3.224799	623284	COX17
1030.337	-4354	1.29E-04	1059	0.333333	3.224799	469930	IL17A
1961.598	-4354	1.29E-04	1016	0.333333	3.224799	612544	DTX3L
1419.35	-4354	1.29E-04	1105	0.333333	3.22434	590244	INPP5B
1356.481	-4354	1.29E-04	1227	0.333333	3.22411	540732	PSMD9
1509.784	-4354	1.29E-04	1160	0.333333	3.22411	553838	HSPA4
1045.388	-4354	1.29E-04	1156	0.333333	3.22411	474484	AVPR2
1366.034	-4354	1.29E-04	1140	0.333333	3.22411	528884	UBE2L3
1641.977	-4354	1.29E-04	1033	0.333333	3.223881	598530	MAGEE1
1496.233	-4354	1.29E-04	1220	0.333333	3.223651	597148	RBL1
1323.517	-4354	1.29E-04	1024	0.333333	3.223651	495228	WNT8A
1031.526	-4354	1.29E-04	1037	0.333333	3.223421	457828	ABCB11
1216.278	-4354	1.29E-04	1045	0.333333	3.223192	510840	MINK1
1565.925	-4354	1.29E-04	992	0.333333	3.223192	569104	PRRT2
1389.69	-4354	1.29E-04	1066	0.333333	3.222962	525098	FOXP3
1035.399	-4354	1.29E-04	1049	0.333333	3.222732	480636	BMP8A
1081.257	-4354	1.29E-04	1148	0.333333	3.222503	485074	PGK1
1153.455	-4354	1.29E-04	1079	0.333333	3.222503	488176	EPO
1174.803	-4354	1.29E-04	1041	0.333333	3.222503	493682	SLITRK2
1540.273	-4354	1.29E-04	1228	0.333333	3.222273	556516	CUL3
1296.571	-4354	1.29E-04	1081	0.333333	3.221814	559036	PIP4K2B
1348.798	-4354	1.29E-04	1076	0.333333	3.221584	536582	MTA1
1139.113	-4354	1.29E-04	996	0.333333	3.221584	492970	JMJD1C
1366.101	-4354	1.29E-04	1157	0.333333	3.221355	521484	VDAC3
1098.201	-4354	1.29E-04	1164	0.333333	3.221125	473256	RAB1A
1021.309	-4354	1.29E-04	1023	0.333333	3.221125	489856	CARD10
1446.954	-4354	1.29E-04	1106	0.333333	3.220896	532686	EIF5
1002.697	-4354	1.29E-04	1030	0.333333	3.220896	477816	SLC11A1
1906.908	-4354	1.29E-04	1175	0.333333	3.220666	562722	NDUFA12
1287.284	-4354	1.29E-04	1089	0.333333	3.220666	524820	PA2G4
1098.87	-4354	1.29E-04	1061	0.333333	3.220666	513416	IRF4
1465.942	-4354	1.29E-04	1062	0.333333	3.220436	549450	INHBC
3213.134	-4354	1.29E-04	1154	0.333333	3.220207	1241024	EIF4E
1444.036	-4354	1.29E-04	1061	0.333333	3.220207	551780	BRAP
1110.728	-4354	1.29E-04	1012	0.333333	3.220207	510138	LRRC8A
1014.856	-4354	1.29E-04	985	0.333333	3.220207	487006	ORAI2
1250.73	-4354	1.29E-04	1016	0.333333	3.219977	535678	REPIN1
1261.054	-4354	1.29E-04	1027	0.333333	3.219747	514476	UBE2F
1107.371	-4354	1.29E-04	1049	0.333333	3.219518	499648	HRK
1332.855	-4354	1.29E-04	1042	0.333333	3.219518	536198	RASSF6
1259.876	-4354	1.29E-04	968	0.333333	3.219518	481474	RP1L1

1180.866	-4354	1.29E-04	1052	0.333333	3.219288	504212	SNAP29
986.7891	-4354	1.29E-04	1045	0.333333	3.219288	462570	PDE1C
1398.686	-4354	1.29E-04	1179	0.333333	3.219059	536268	FZR1
1826.34	-4354	1.29E-04	1045	0.333333	3.219059	561910	PSMA8
1925.374	-4354	1.29E-04	1032	0.333333	3.219059	605778	UBOX5
1099.477	-4354	1.29E-04	1008	0.333333	3.219059	506890	MTHFR
890.5803	-4354	1.29E-04	1005	0.333333	3.219059	448498	DLL3
1193.451	-4354	1.29E-04	999	0.333333	3.219059	516158	ABCB9
1237.485	-4354	1.29E-04	1103	0.333333	3.218599	504164	CTBP1
1234.716	-4354	1.29E-04	1078	0.333333	3.218599	532056	SPOP
1152.72	-4354	1.29E-04	975	0.333333	3.218599	459626	SSX1
1247.082	-4354	1.29E-04	1060	0.333333	3.21837	509776	APPL1
1290.547	-4354	1.29E-04	1055	0.333333	3.21837	500684	RPS6KA6
1287.797	-4354	1.29E-04	987	0.333333	3.21837	482882	KRT71
1277.142	-4354	1.29E-04	1112	0.333333	3.21814	509348	PPM1B
1430.234	-4354	1.29E-04	1144	0.333333	3.21791	505332	ELOC
1423.393	-4354	1.29E-04	1014	0.333333	3.21791	527902	SYMPK
1424.615	-4354	1.29E-04	1043	0.333333	3.217681	506292	DAP3
1196.123	-4354	1.29E-04	1043	0.333333	3.217681	518392	AJUBA
1513.708	-4354	1.29E-04	1105	0.333333	3.217451	495506	IDH3B
1512.068	-4354	1.29E-04	1088	0.333333	3.217451	551244	DAB2IP
1441.301	-4354	1.29E-04	1082	0.333333	3.217451	534996	BRK1
1013.09	-4354	1.29E-04	1045	0.333333	3.217451	459528	LAMB4
966.4853	-4354	1.29E-04	996	0.333333	3.217451	449952	SYN2
1056.475	-4354	1.29E-04	963	0.333333	3.217451	469366	LPIN3
1209.718	-4354	1.29E-04	1088	0.333333	3.217222	509238	EWSR1
1107.696	-4354	1.29E-04	996	0.333333	3.217222	481856	CLTB
1537.357	-4354	1.29E-04	1051	0.333333	3.216992	550076	PARP2
875.4907	-4354	1.29E-04	1027	0.333333	3.216992	441584	KCNJ1
1473.752	-4354	1.29E-04	997	0.333333	3.216992	490366	IDUA
1778.184	-4354	1.29E-04	1062	0.333333	3.216533	582226	PGAM5
1333.33	-4354	1.29E-04	1002	0.333333	3.216533	480344	SNX4
1824.161	-4354	1.29E-04	1142	0.333333	3.216073	546564	NDUFB6
1082.811	-4354	1.29E-04	971	0.333333	3.216073	464508	MGAT2
1194.069	-4354	1.29E-04	957	0.333333	3.216073	492120	LAMTOR4
1162.482	-4354	1.29E-04	1057	0.333333	3.215844	490624	ACBD3
1396.718	-4354	1.29E-04	1204	0.333333	3.215385	510276	SKP1
1399.043	-4354	1.29E-04	1073	0.333333	3.215155	535696	AXIN2
1484.43	-4354	1.29E-04	1024	0.333333	3.215155	490826	GRK7
1289.668	-4354	1.29E-04	1021	0.333333	3.214925	542910	NRF1
1407.497	-4354	1.29E-04	1017	0.333333	3.214696	528960	HMBOX1

1637.6	-4354	1.29E-04	1059	0.333333	3.214466	567620	EIF3C
1294.975	-4354	1.29E-04	1031	0.333333	3.214466	521274	UBE2G2
1476.18	-4354	1.29E-04	1130	0.333333	3.214237	504154	ANAPC10
1167.066	-4354	1.29E-04	1053	0.333333	3.214237	510554	WNT10A
1522.523	-4354	1.29E-04	1188	0.333333	3.214007	557294	RUNX1
1138.212	-4354	1.29E-04	1066	0.333333	3.214007	480040	IL5RA
908.9684	-4354	1.29E-04	1034	0.333333	3.214007	415718	THPO
1117.969	-4354	1.29E-04	1030	0.333333	3.214007	479158	ATP6V1C1
1047.808	-4354	1.29E-04	985	0.333333	3.213777	471096	DBP
1074.561	-4354	1.29E-04	946	0.333333	3.213777	453648	ACOT12
1014.917	-4354	1.29E-04	985	0.333333	3.213548	441004	ABO
1495.283	-4354	1.29E-04	1247	0.333333	3.213318	529708	AURKA
1262.433	-4354	1.29E-04	1085	0.333333	3.213318	499518	SMARCC2
2017.939	-4354	1.29E-04	1031	0.333333	3.213318	554098	ATP5ME
1216.536	-4354	1.29E-04	1099	0.333333	3.213088	519518	CASP2
1177.774	-4354	1.29E-04	1036	0.333333	3.213088	512770	STIM2
1230.969	-4354	1.29E-04	1000	0.333333	3.213088	513802	SRGAP2
982.4498	-4354	1.29E-04	971	0.333333	3.213088	466256	SLC35A2
1202.523	-4354	1.29E-04	959	0.333333	3.213088	504064	POLG
1176.688	-4354	1.29E-04	1097	0.333333	3.212859	501444	BCAR1
1416.035	-4354	1.29E-04	1073	0.333333	3.212859	504250	DVL2
985.2076	-4354	1.29E-04	1026	0.333333	3.212629	433648	KLK3
1202.966	-4354	1.29E-04	1217	0.333333	3.2124	471948	PSMB2
1329.234	-4354	1.29E-04	1019	0.333333	3.2124	521892	DET1
1191.363	-4354	1.29E-04	995	0.333333	3.2124	505404	MTMR1
1016.267	-4354	1.29E-04	973	0.333333	3.21217	488770	VAMP1
1473.17	-4354	1.29E-04	1039	0.333333	3.21194	529248	SEC22A
1355.955	-4354	1.28E-04	1127	0.333333	3.211711	527008	TOP2A
945.98	-4354	1.28E-04	1006	0.333333	3.211481	400412	CYP3A43
2126.502	-4354	1.28E-04	972	0.333333	3.211481	572220	PCGF6
933.4382	-4354	1.28E-04	969	0.333333	3.211481	433428	IL9R
1048.797	-4354	1.28E-04	952	0.333333	3.211481	488636	SIPA1L3
1187.545	-4354	1.28E-04	1049	0.333333	3.211251	507496	RGMB
1326.486	-4354	1.28E-04	1037	0.333333	3.211251	512956	IPMK
1139.652	-4354	1.28E-04	1027	0.333333	3.211251	463160	STAG2
1210.581	-4354	1.28E-04	989	0.333333	3.211251	471940	CALML6
1017.782	-4354	1.28E-04	965	0.333333	3.211022	444574	IL17RC
1137.313	-4354	1.28E-04	1229	0.333333	3.210792	455818	PSMD11
1198.442	-4354	1.28E-04	1036	0.333333	3.210792	488606	IFIH1
1182.893	-4354	1.28E-04	1015	0.333333	3.210792	488578	ABCB5
2243.948	-4354	1.28E-04	967	0.333333	3.210792	540034	MT-ATP6

960.4188	-4354	1.28E-04	1118	0.333333	3.210563	417096	OPRM1
1312.151	-4354	1.28E-04	995	0.333333	3.210563	510682	HMMR
1368.017	-4354	1.28E-04	980	0.333333	3.210563	494730	THEM5
1237.996	-4354	1.28E-04	943	0.333333	3.210563	485860	GLYCTK
1006.936	-4354	1.28E-04	938	0.333333	3.210333	430844	SPOPL
889.0138	-4354	1.28E-04	1013	0.333333	3.210103	415866	CNGB1
1128.494	-4354	1.28E-04	1008	0.333333	3.210103	490346	CACNA2D4
1130.261	-4354	1.28E-04	1043	0.333333	3.209874	477286	ARID1A
967.7624	-4354	1.28E-04	1030	0.333333	3.209874	439718	CFHR3
1394.824	-4354	1.28E-04	1040	0.333333	3.209644	527294	SCFD2
1555.122	-4354	1.28E-04	1025	0.333333	3.209644	622468	CHMP4B
1100.07	-4354	1.28E-04	979	0.333333	3.209644	469652	LGI4
893.5096	-4354	1.28E-04	944	0.333333	3.209644	398262	P2RX2
1204.707	-4354	1.28E-04	940	0.333333	3.209644	471196	ELOVL3
1332.306	-4354	1.28E-04	1007	0.333333	3.209414	496684	UNC5A
1226.813	-4354	1.28E-04	1090	0.333333	3.209185	484218	USP7
890.6911	-4354	1.28E-04	997	0.333333	3.209185	428170	BMP1
1140.95	-4354	1.28E-04	932	0.333333	3.209185	485304	FOXO6
1221.372	-4354	1.28E-04	1020	0.333333	3.208726	464970	SRSF3
1207.72	-4354	1.28E-04	966	0.333333	3.208726	484350	AP4E1
1204.439	-4354	1.28E-04	966	0.333333	3.208496	458870	LDHAL6A
1176.669	-4354	1.28E-04	963	0.333333	3.208496	467716	GABPA
1086.876	-4354	1.28E-04	950	0.333333	3.208496	457164	SLC4A5
1005.082	-4354	1.28E-04	927	0.333333	3.208496	445742	PNLIPRP3
1015.659	-4354	1.28E-04	1023	0.333333	3.208266	418100	SLCO1A2
919.0618	-4354	1.28E-04	959	0.333333	3.208266	424700	UBE2Q1
1108.837	-4354	1.28E-04	1056	0.333333	3.208037	473440	STAM2
1784.771	-4354	1.28E-04	1012	0.333333	3.208037	576014	PCGF3
1166.192	-4354	1.28E-04	966	0.333333	3.208037	434974	SP7
1107.416	-4354	1.28E-04	951	0.333333	3.207807	475816	DFFA
1191.948	-4354	1.28E-04	1012	0.333333	3.207577	459584	BRD7
1254.481	-4354	1.28E-04	1003	0.333333	3.207577	504022	INVS
1216.159	-4354	1.28E-04	957	0.333333	3.207577	445788	ESX1
1190.897	-4354	1.28E-04	942	0.333333	3.207577	466878	BTLA
947.0242	-4354	1.28E-04	932	0.333333	3.207577	437922	WTIP
1313.342	-4354	1.28E-04	928	0.333333	3.207577	477720	PRMT9
1310.22	-4354	1.28E-04	1011	0.333333	3.207348	500224	FNIP1
1188.74	-4354	1.28E-04	1000	0.333333	3.207348	476974	SMAD5
1083.013	-4354	1.28E-04	1071	0.333333	3.207118	436230	UBE2K
1089.77	-4354	1.28E-04	953	0.333333	3.207118	449384	HES5
1167.762	-4354	1.28E-04	1037	0.333333	3.206659	471122	COX15

1127.515	-4354	1.28E-04	962	0.333333	3.206659	453552	AKT1S1
958.6775	-4354	1.28E-04	1028	0.333333	3.206429	416128	GBP5
999.7107	-4354	1.28E-04	977	0.333333	3.206429	445314	BVES
1046.999	-4354	1.28E-04	941	0.333333	3.206429	445638	JMJD6
1414.366	-4354	1.28E-04	1089	0.333333	3.2062	464486	COX7B
1997.858	-4354	1.28E-04	991	0.333333	3.2062	806176	PPP1R3A
929.1762	-4354	1.28E-04	955	0.333333	3.20597	439156	NBR1
1449.136	-4354	1.28E-04	1132	0.333333	3.205741	485784	CUL5
858.2505	-4354	1.28E-04	1056	0.333333	3.205741	399060	HTR4
1102.056	-4354	1.28E-04	1049	0.333333	3.205741	454424	ITGA11
1271.379	-4354	1.28E-04	1084	0.333333	3.205511	476966	CUL4A
1206.84	-4354	1.28E-04	1010	0.333333	3.205511	482170	HERC4
1041.548	-4354	1.28E-04	1002	0.333333	3.205511	436834	BOC
1033.138	-4354	1.28E-04	963	0.333333	3.205511	425670	GP5
1206.626	-4354	1.28E-04	1002	0.333333	3.205281	467462	KCNK9
1046.896	-4354	1.28E-04	919	0.333333	3.205281	468470	UBE2QL1
1192.407	-4354	1.28E-04	1244	0.333333	3.205052	436824	PSMC2
1111.661	-4354	1.28E-04	1069	0.333333	3.205052	442410	MAP2K7
1429.903	-4354	1.28E-04	1029	0.333333	3.205052	442314	ATP5PD
1054.34	-4354	1.28E-04	907	0.333333	3.205052	448666	CASTOR2
1203.835	-4354	1.28E-04	1061	0.333333	3.204822	486814	NSD2
1158.317	-4354	1.28E-04	1043	0.333333	3.204822	469094	EIF2AK2
1722.751	-4354	1.28E-04	974	0.333333	3.204822	585516	CHMP5
732.5507	-4354	1.28E-04	932	0.333333	3.204822	388582	LILRA5
1281.631	-4354	1.28E-04	1065	0.333333	3.204363	478792	DDX58
1498.184	-4354	1.28E-04	918	0.333333	3.204363	527584	CLDN23
998.8242	-4354	1.28E-04	907	0.333333	3.204133	423476	TKTL2
1260.88	-4354	1.28E-04	1236	0.333333	3.203904	444522	PSMB1
1104.842	-4354	1.28E-04	1043	0.333333	3.203904	451582	ATG5
1210.171	-4354	1.28E-04	945	0.333333	3.203904	484606	MARVELD2
1048.077	-4354	1.28E-04	1036	0.333333	3.203674	424658	PGLS
1192.864	-4354	1.28E-04	1007	0.333333	3.203674	467750	ABLIM2
1069.549	-4354	1.28E-04	931	0.333333	3.203674	463618	MACC1
1429.671	-4354	1.28E-04	927	0.333333	3.203674	498860	DTX3
1248.843	-4354	1.28E-04	960	0.333333	3.203444	448818	TBX20
1229.053	-4354	1.28E-04	1193	0.333333	3.203215	424840	PSMA2
977.5011	-4354	1.28E-04	1035	0.333333	3.203215	425846	APOC2
891.9865	-4354	1.28E-04	962	0.333333	3.203215	410988	APH1A
1058.405	-4354	1.28E-04	951	0.333333	3.203215	411834	WNT9B
1231.023	-4354	1.28E-04	963	0.333333	3.202755	476326	MSH3
930.4489	-4354	1.28E-04	1036	0.333333	3.202526	401330	TPI1

1290.924	-4354	1.28E-04	956	0.333333	3.202526	494220	TAF15
1487.519	-4354	1.28E-04	1076	0.333333	3.202067	457304	COX7C
1039.949	-4354	1.28E-04	1034	0.333333	3.202067	422348	HSPD1
1114.512	-4354	1.28E-04	976	0.333333	3.202067	458162	NGEF
829.9231	-4354	1.28E-04	1002	0.333333	3.201837	369116	GNAT1
928.6195	-4354	1.28E-04	967	0.333333	3.201837	425194	SP100
937.0527	-4354	1.28E-04	979	0.333333	3.201607	430102	SETD7
1600.694	-4354	1.28E-04	1119	0.333333	3.201378	472596	UQCR11
1455.496	-4354	1.28E-04	950	0.333333	3.201378	436956	COX6A1
988.2544	-4354	1.28E-04	932	0.333333	3.201378	454546	SLC45A3
1018.025	-4354	1.28E-04	919	0.333333	3.201378	448160	SPATA2
1065.693	-4354	1.28E-04	1013	0.333333	3.200918	448194	EIF4B
1120.112	-4354	1.28E-04	937	0.333333	3.200918	458190	UBE2D4
1045.043	-4354	1.28E-04	1046	0.333333	3.200689	398810	UBE2N
1044	-4354	1.28E-04	929	0.333333	3.200689	438518	MCOLN2
880.2398	-4354	1.28E-04	891	0.333333	3.200689	400628	GPX8
1040.548	-4354	1.28E-04	1004	0.333333	3.200459	413992	SAE1
1430.131	-4354	1.28E-04	972	0.333333	3.200459	503248	CCL28
1122.893	-4354	1.28E-04	915	0.333333	3.20023	448288	ENPP5
960.4448	-4354	1.28E-04	930	0.333333	3.2	386730	MTMR7
958.2208	-4354	1.28E-04	947	0.333333	3.19977	404056	ATG3
846.9064	-4354	1.28E-04	891	0.333333	3.19977	388074	DGKK
1217.682	-4354	1.28E-04	1029	0.333333	3.199541	424500	CDC26
983.64	-4354	1.28E-04	952	0.333333	3.199541	413094	AP3D1
1309.612	-4354	1.28E-04	1004	0.333333	3.199311	459440	AGGF1
750.8377	-4354	1.28E-04	944	0.333333	3.199311	372418	HLA-DQB1
789.4607	-4354	1.28E-04	892	0.333333	3.199311	402004	RAB7B
1256.906	-4354	1.28E-04	949	0.333333	3.199082	449398	RNF31
1390.445	-4354	1.28E-04	1045	0.333333	3.198852	467244	ORC5
1446.58	-4354	1.28E-04	1035	0.333333	3.198852	454468	NDUFC1
1456.395	-4354	1.28E-04	886	0.333333	3.198852	483884	ATXN1L
1241.073	-4354	1.28E-04	939	0.333333	3.198393	372598	SPAM1
1108.796	-4354	1.28E-04	939	0.333333	3.198393	442904	ATG2B
884.4316	-4354	1.28E-04	995	0.333333	3.198163	385376	DDX3X
1172.286	-4354	1.28E-04	936	0.333333	3.197933	429184	TBCE
968.9985	-4354	1.27E-04	891	0.333333	3.197704	419284	CA5B
1156.202	-4354	1.27E-04	998	0.333333	3.197474	427020	MSH6
1098.977	-4354	1.27E-04	1030	0.333333	3.197245	411884	UBE2D2
1202.778	-4354	1.27E-04	1029	0.333333	3.197245	455298	CCNT2
910.707	-4354	1.27E-04	925	0.333333	3.197245	369418	KCNH6
1411.495	-4354	1.27E-04	1055	0.333333	3.197015	441208	NDUFS7

1009.632	-4354	1.27E-04	970	0.333333	3.197015	437202	PPP2R2C
937.8205	-4354	1.27E-04	974	0.333333	3.196785	426936	MAGI3
1047.842	-4354	1.27E-04	954	0.333333	3.196785	403104	PPA2
1044.531	-4354	1.27E-04	953	0.333333	3.196785	388124	SDHC
1021.82	-4354	1.27E-04	954	0.333333	3.196556	430478	SLC44A5
1143.67	-4354	1.27E-04	1009	0.333333	3.196326	445828	BUB1
1087.942	-4354	1.27E-04	967	0.333333	3.196096	446050	IFNLR1
773.6425	-4354	1.27E-04	897	0.333333	3.195867	369822	CARD14
937.7475	-4354	1.27E-04	896	0.333333	3.195867	408296	AIFM2
793.7529	-4354	1.27E-04	953	0.333333	3.195637	358152	FGF19
1071.815	-4354	1.27E-04	916	0.333333	3.195637	415840	MAVS
757.6923	-4354	1.27E-04	884	0.333333	3.195408	364722	KIR2DL4
1123.716	-4354	1.27E-04	1122	0.333333	3.195178	387958	PSMB4
614.6468	-4354	1.27E-04	876	0.333333	3.194948	332680	PCYT1B
1589.452	-4354	1.27E-04	988	0.333333	3.194719	442766	ATP5F1E
828.0153	-4354	1.27E-04	906	0.333333	3.194719	353192	SLC34A1
935.7537	-4354	1.27E-04	873	0.333333	3.194719	373730	LRRC4B
1208.515	-4354	1.27E-04	891	0.333333	3.194489	431982	NUDT16L1
818.2985	-4354	1.27E-04	888	0.333333	3.194489	380614	ANGPTL1
1080.565	-4354	1.27E-04	1107	0.333333	3.194259	404200	BUB3
1123.718	-4354	1.27E-04	1002	0.333333	3.194259	411294	ADCY5
1130.833	-4354	1.27E-04	880	0.333333	3.194259	425370	ANAPC13
1022.109	-4354	1.27E-04	936	0.333333	3.19403	421826	SETD2
1208.439	-4354	1.27E-04	873	0.333333	3.1938	425050	CLDN20
789.562	-4354	1.27E-04	870	0.333333	3.1938	366470	BPNT2
775.3399	-4354	1.27E-04	893	0.333333	3.193571	356558	COL6A6
1138.101	-4354	1.27E-04	892	0.333333	3.193571	414134	MZF1
961.4784	-4354	1.27E-04	881	0.333333	3.193571	393194	COX7B2
1040.101	-4354	1.27E-04	1052	0.333333	3.193341	408116	ANAPC5
939.3189	-4354	1.27E-04	933	0.333333	3.193341	404514	TNFRSF19
1545.426	-4354	1.27E-04	1071	0.333333	3.193111	462534	NDUFB2
865.2316	-4354	1.27E-04	940	0.333333	3.193111	368892	NBN
971.5713	-4354	1.27E-04	977	0.333333	3.192882	404538	YY1
786.7012	-4354	1.27E-04	919	0.333333	3.192882	348838	HNF1A
1232.37	-4354	1.27E-04	931	0.333333	3.192652	403514	UBE2U
562.4795	-4354	1.27E-04	907	0.333333	3.192652	308548	NRXN1
1134.985	-4354	1.27E-04	947	0.333333	3.192423	420668	SLC2A12
1172.772	-4354	1.27E-04	894	0.333333	3.192193	430002	NUMBL
985.718	-4354	1.27E-04	925	0.333333	3.191504	399676	ATG4B
1375.419	-4354	1.27E-04	922	0.333333	3.191504	532410	PLA2G12B
1036.256	-4354	1.27E-04	946	0.333333	3.191274	436794	FBXW8

867.3064	-4354	1.27E-04	921	0.333333	3.191274	364374	TAOK2
948.0511	-4354	1.27E-04	875	0.333333	3.191274	357312	GSTA1
632.5456	-4354	1.27E-04	860	0.333333	3.190815	321026	MUC5AC
983.0871	-4354	1.27E-04	1148	0.333333	3.190356	362294	PSMD4
667.1656	-4354	1.27E-04	860	0.333333	3.190356	348856	EPB41L4B
1375.219	-4354	1.27E-04	1051	0.333333	3.190126	408686	COX5B
1086.378	-4354	1.27E-04	959	0.333333	3.190126	407900	MIB1
787.5181	-4354	1.27E-04	871	0.333333	3.190126	330390	TP53AIP1
1221.231	-4354	1.27E-04	925	0.333333	3.188978	430362	MOSMO
972.0709	-4354	1.27E-04	920	0.333333	3.188978	397768	UBA6
723.6106	-4354	1.27E-04	918	0.333333	3.188978	321148	GH2
984.1251	-4354	1.27E-04	904	0.333333	3.188978	400876	TPCN2
1349.82	-4354	1.27E-04	951	0.333333	3.188749	390198	UQCR10
889.7951	-4354	1.27E-04	870	0.333333	3.188749	371442	TIGIT
625.145	-4354	1.27E-04	869	0.333333	3.188749	304882	TNXB
714.2942	-4354	1.27E-04	851	0.333333	3.188749	304460	GPX5
901.2814	-4354	1.27E-04	931	0.333333	3.188289	363522	GNG8
661.4994	-4354	1.27E-04	846	0.333333	3.18806	323614	MUC5B
1473.913	-4354	1.27E-04	994	0.333333	3.187371	423320	NDUFB1
1010.058	-4354	1.27E-04	903	0.333333	3.186682	376924	TFAM
618.4755	-4354	1.27E-04	885	0.333333	3.186682	305522	GRIN2C
990.2275	-4354	1.27E-04	850	0.333333	3.186682	391736	DHODH
1147.238	-4354	1.27E-04	985	0.333333	3.186452	427900	CYP51A1
913.9214	-4354	1.27E-04	905	0.333333	3.186452	363998	ACP1
1103.844	-4354	1.27E-04	890	0.333333	3.186223	405774	ERCC8
928.7853	-4354	1.27E-04	878	0.333333	3.186223	391192	STMN1
588.0449	-4354	1.27E-04	839	0.333333	3.185993	299158	KLK15
859.9999	-4354	1.27E-04	996	0.333333	3.185763	367432	PRKDC
918.0208	-4354	1.27E-04	917	0.333333	3.185763	379160	VAMP4
890.4791	-4354	1.27E-04	855	0.333333	3.185763	388366	ZBTB24
1120.997	-4354	1.27E-04	848	0.333333	3.185763	393364	EFCAB7
960.8654	-4354	1.27E-04	855	0.333333	3.185304	355956	IFNK
872.1681	-4354	1.27E-04	855	0.333333	3.184845	363462	OTUD5
764.12	-4354	1.27E-04	932	0.333333	3.184615	326712	GRIN1
1101.316	-4354	1.27E-04	862	0.333333	3.184615	375852	WDR24
766.1851	-4354	1.27E-04	865	0.333333	3.184386	352702	WIPI2
743.4929	-4354	1.27E-04	857	0.333333	3.184156	336740	BMP8B
1101.824	-4354	1.27E-04	938	0.333333	3.183927	395940	RPS6
1041.536	-4354	1.27E-04	867	0.333333	3.183927	375968	ATP5MC2
801.5083	-4354	1.27E-04	864	0.333333	3.183927	362658	PGM2L1
707.5551	-4354	1.27E-04	819	0.333333	3.183927	334690	TMEM37

599.9927	-4354	1.27E-04	833	0.333333	3.183697	305676	NOX1
950.5305	-4354	1.26E-04	941	0.333333	3.183238	379696	SMC1A
933.9309	-4354	1.26E-04	997	0.333333	3.182549	339974	CDC23
689.031	-4354	1.26E-04	878	0.333333	3.182549	308514	WNT6
772.3	-4354	1.26E-04	865	0.333333	3.18186	335756	TRIM10
927.0373	-4354	1.26E-04	834	0.333333	3.18186	353202	EBAG9
950.9662	-4354	1.26E-04	889	0.333333	3.18163	372654	PACSIN1
768.6742	-4354	1.26E-04	828	0.333333	3.181401	351456	SLC24A1
765.549	-4354	1.26E-04	905	0.333333	3.180941	327330	FFAR1
894.1215	-4354	1.26E-04	857	0.333333	3.180712	372732	DIRAS1
744.5166	-4354	1.26E-04	856	0.333333	3.180712	337532	CREG2
831.3914	-4354	1.26E-04	834	0.333333	3.180482	346180	SIRT3
932.3768	-4354	1.26E-04	912	0.333333	3.180253	361740	CDC25C
952.4302	-4354	1.26E-04	869	0.333333	3.180253	338778	MPP4
778.8513	-4354	1.26E-04	821	0.333333	3.180253	338990	KDM1B
610.8322	-4354	1.26E-04	853	0.333333	3.179793	293912	HAVCR2
675.7611	-4354	1.26E-04	820	0.333333	3.179564	312474	CRB2
936.7113	-4354	1.26E-04	836	0.333333	3.178875	339308	PLCD3
860.4159	-4354	1.26E-04	827	0.333333	3.178875	348298	NPRL2
652.9943	-4354	1.26E-04	821	0.333333	3.178875	300850	MYH14
866.5622	-4354	1.26E-04	928	0.333333	3.178645	329502	EIF2S1
467.6991	-4354	1.26E-04	820	0.333333	3.178645	243542	PNLIPRP1
871.4438	-4354	1.26E-04	951	0.333333	3.178416	362568	SIN3A
830.9739	-4354	1.26E-04	844	0.333333	3.178416	328138	KDM6A
708.8629	-4354	1.26E-04	816	0.333333	3.178416	323694	NPNT
577.1634	-4354	1.26E-04	868	0.333333	3.178186	269380	MASP2
984.6848	-4354	1.26E-04	909	0.333333	3.177727	360086	CACYBP
712.1077	-4354	1.26E-04	831	0.333333	3.177727	341272	ARSG
738.1586	-4354	1.26E-04	830	0.333333	3.177038	328672	GGA1
920.2588	-4354	1.26E-04	807	0.333333	3.176808	363988	SUPT20H
954.6037	-4354	1.26E-04	862	0.333333	3.176579	358314	SUFU
655.7724	-4354	1.26E-04	931	0.333333	3.176119	283436	CD68
1365.503	-4354	1.26E-04	865	0.333333	3.176119	414092	PCGF5
763.0488	-4354	1.26E-04	833	0.333333	3.17566	321126	XAF1
666.8146	-4354	1.26E-04	842	0.333333	3.175431	286368	FGF22
847.8315	-4354	1.26E-04	823	0.333333	3.175431	338458	IL17RE
733.3242	-4354	1.26E-04	788	0.333333	3.175431	308856	DAGLB
771.7026	-4354	1.26E-04	796	0.333333	3.174742	326216	ZFYVE1
997.6363	-4354	1.26E-04	936	0.333333	3.174512	362706	MRE11
634.6539	-4354	1.26E-04	811	0.333333	3.174282	305218	RGMA
874.3209	-4354	1.26E-04	881	0.333333	3.173823	320442	ORC4

1632.169	-4354	1.26E-04	792	0.333333	3.173364	808100	PLB1
1147.672	-4354	1.26E-04	811	0.333333	3.173134	351012	IQGAP3
892.8463	-4354	1.26E-04	806	0.333333	3.173134	334044	PIDD1
722.9531	-4354	1.26E-04	780	0.333333	3.172905	325042	CR1L
1000.767	-4354	1.26E-04	1020	0.333333	3.172675	348728	HSPA8
737.4955	-4354	1.26E-04	831	0.333333	3.172675	321490	APLN
879.5773	-4354	1.26E-04	829	0.333333	3.171986	345948	AP1S3
618.6818	-4354	1.26E-04	789	0.333333	3.171527	272438	CACNA1I
959.1701	-4354	1.26E-04	818	0.333333	3.170838	343188	IDNK
879.5141	-4354	1.26E-04	775	0.333333	3.170379	336132	RAB39B
1322.776	-4354	1.26E-04	948	0.333333	3.170149	354336	NDUFA5
1145.398	-4354	1.26E-04	901	0.333333	3.170149	321342	ATP5MG
845.5337	-4354	1.26E-04	840	0.333333	3.170149	326980	SIKE1
697.4737	-4354	1.26E-04	820	0.333333	3.16969	298760	TPR
871.838	-4354	1.26E-04	804	0.333333	3.16969	303636	EPHA8
804.4454	-4354	1.26E-04	787	0.333333	3.16969	302012	WNK4
939.3295	-4354	1.26E-04	931	0.333333	3.16946	344342	PLK4
692.8546	-4354	1.26E-04	837	0.333333	3.16946	276854	ADH4
911.6705	-4354	1.25E-04	905	0.333333	3.169001	321226	ELOB
807.9157	-4354	1.25E-04	821	0.333333	3.169001	324922	RIF1
716.5353	-4354	1.25E-04	782	0.333333	3.168542	308232	SUMF1
574.2289	-4354	1.25E-04	747	0.333333	3.168542	279198	APELA
683.309	-4354	1.25E-04	745	0.333333	3.168542	294408	COX8C
1107.478	-4354	1.25E-04	950	0.333333	3.168312	322184	NDUFB10
563.5417	-4354	1.25E-04	803	0.333333	3.168083	280104	EBF3
925.8804	-4354	1.25E-04	906	0.333333	3.167853	340642	EIF4A1
634.5669	-4354	1.25E-04	804	0.333333	3.167623	282312	NKD1
658.5813	-4354	1.25E-04	749	0.333333	3.166935	270326	DEFB132
567.2831	-4354	1.25E-04	785	0.333333	3.166705	270282	COL6A5
622.0653	-4354	1.25E-04	764	0.333333	3.166705	282566	RASGRP4
606.2087	-4354	1.25E-04	751	0.333333	3.166705	267948	PPP1R12C
1161.923	-4354	1.25E-04	922	0.333333	3.166475	344962	NDUFV3
682.3537	-4354	1.25E-04	809	0.333333	3.166475	295228	PLEKHG5
673.8103	-4354	1.25E-04	799	0.333333	3.166016	301590	TEAD2
903.3619	-4354	1.25E-04	906	0.333333	3.165557	291532	COX4I1
579.9765	-4354	1.25E-04	751	0.333333	3.165557	259412	IGSF5
851.5032	-4354	1.25E-04	784	0.333333	3.165098	301156	PPIE
794.5677	-4354	1.25E-04	857	0.333333	3.164868	319000	RAD51
1215.537	-4354	1.25E-04	884	0.333333	3.16372	329964	NDUFA2
799.6902	-4354	1.25E-04	872	0.333333	3.163031	292940	SRSF1
588.786	-4354	1.25E-04	758	0.333333	3.162801	271532	CD276

596.7103	-4354	1.25E-04	751	0.333333	3.162801	272120	ATP6V0D2
475.5005	-4354	1.25E-04	750	0.333333	3.162801	214608	SLC22A7
786.9994	-4354	1.25E-04	727	0.333333	3.162801	308680	ACTR3C
685.7397	-4354	1.25E-04	757	0.333333	3.162572	289362	CGAS
771.7028	-4354	1.25E-04	775	0.333333	3.162113	279896	IL31RA
775.9974	-4354	1.25E-04	902	0.333333	3.161883	306008	ITGB6
624.4838	-4354	1.25E-04	755	0.333333	3.161194	272808	SLC52A3
550.8678	-4354	1.25E-04	746	0.333333	3.159587	252232	CD99L2
644.1753	-4354	1.25E-04	724	0.333333	3.159127	281732	PPARGC1B
648.7685	-4354	1.25E-04	765	0.333333	3.158439	263532	WNT9A
573.0955	-4354	1.25E-04	712	0.333333	3.158209	235868	SMC1B
700.5877	-4354	1.25E-04	792	0.333333	3.15775	272280	RICTOR
833.4828	-4354	1.25E-04	833	0.333333	3.157061	293366	GRM7
635.6519	-4354	1.25E-04	745	0.333333	3.157061	260808	TSPEAR
647.4869	-4354	1.25E-04	887	0.333333	3.156602	259150	MAP2K3
617.9786	-4354	1.25E-04	732	0.333333	3.156602	240572	MAPK15
720.3382	-4354	1.25E-04	765	0.333333	3.156372	257318	RCHY1
583.4282	-4354	1.25E-04	733	0.333333	3.156372	266418	ACSS1
1006.446	-4354	1.25E-04	857	0.333333	3.156142	287620	NDUFB7
552.454	-4354	1.25E-04	745	0.333333	3.155683	248314	IL23R
583.0845	-4354	1.25E-04	758	0.333333	3.155454	249360	PLAA
976.3883	-4354	1.25E-04	899	0.333333	3.155224	280604	NDUFA6
646.9183	-4354	1.25E-04	748	0.333333	3.154535	254230	RAD1
786.528	-4354	1.25E-04	712	0.333333	3.154535	286848	LIN54
587.1428	-4354	1.25E-04	705	0.333333	3.154535	236474	S100A7A
605.8466	-4354	1.24E-04	711	0.333333	3.153846	252104	MIOS
526.4699	-4354	1.24E-04	695	0.333333	3.153846	234424	NPRL3
733.2704	-4354	1.24E-04	715	0.333333	3.152928	240790	RSPO2
435.0255	-4354	1.24E-04	692	0.333333	3.152468	232436	AMOTL1
710.5949	-4354	1.24E-04	744	0.333333	3.152239	269028	POLK
621.0636	-4354	1.24E-04	738	0.333333	3.152239	242176	DPYSL5
479.6097	-4354	1.24E-04	717	0.333333	3.15178	229762	RSPO4
809.9697	-4354	1.24E-04	707	0.333333	3.15132	261422	CACNG6
596.4388	-4354	1.24E-04	685	0.333333	3.150631	233974	FAHD1
606.1855	-4354	1.24E-04	751	0.333333	3.150402	261416	TIRAP
697.4238	-4354	1.24E-04	750	0.333333	3.150172	265974	CGNL1
705.9335	-4354	1.24E-04	796	0.333333	3.149943	272388	USP15
643.0335	-4354	1.24E-04	765	0.333333	3.149943	253276	BIRC5
601.476	-4354	1.24E-04	743	0.333333	3.149943	238868	RAD50
562.9989	-4354	1.24E-04	745	0.333333	3.149483	231352	DHFR
623.314	-4354	1.24E-04	723	0.333333	3.148794	248104	PARD6G

920.8476	-4354	1.24E-04	837	0.333333	3.148565	261606	NDUFB8
634.4577	-4354	1.24E-04	778	0.333333	3.148565	246596	BCL3
797.703	-4354	1.24E-04	679	0.333333	3.148335	276440	MTCP1
558.5811	-4354	1.24E-04	677	0.333333	3.148335	240596	ATP6V1C2
515.9741	-4354	1.24E-04	676	0.333333	3.148106	218920	NLGN2
481.6904	-4354	1.24E-04	674	0.333333	3.148106	228040	EVC2
713.9476	-4354	1.24E-04	728	0.333333	3.147876	258182	MFSD8
548.1906	-4354	1.24E-04	671	0.333333	3.147876	225212	DNAJC14
639.475	-4354	1.24E-04	696	0.333333	3.147187	233064	MARVELD3
559.1456	-4354	1.24E-04	696	0.333333	3.146728	243138	CRACR2A
611.8726	-4354	1.24E-04	673	0.333333	3.146498	239550	USP30
612.1954	-4354	1.24E-04	696	0.333333	3.145809	250234	THEM4
578.146	-4354	1.24E-04	704	0.333333	3.14535	223108	EIF2AK4
405.7675	-4354	1.24E-04	658	0.333333	3.145121	195018	LINGO2
1199.371	-4354	1.24E-04	819	0.333333	3.144661	284510	NDUFS8
602.6991	-4354	1.24E-04	707	0.333333	3.144432	236652	TAB3
423.5909	-4354	1.24E-04	646	0.333333	3.144432	194364	MAP1LC3B2
414.6394	-4354	1.24E-04	656	0.333333	3.143972	193266	EXOC7
622.3287	-4354	1.24E-04	762	0.333333	3.143743	222622	SAA1
559.7396	-4354	1.24E-04	670	0.333333	3.142135	222080	ATP1A4
695.2393	-4354	1.24E-04	663	0.333333	3.141447	222198	CLDN19
671.3844	-4354	1.24E-04	719	0.333333	3.141217	239362	ATP6V1F
645.515	-4354	1.24E-04	800	0.333333	3.139839	220228	ACTG1
20877.4	-4354	1.24E-04	621	0.333333	3.13961	4531766	KRTAP4-1
493.7201	-4354	1.23E-04	646	0.333333	3.13915	205544	KLHL9
544.7251	-4354	1.23E-04	806	0.333333	3.137773	216278	ADORA2A
470.2128	-4354	1.23E-04	622	0.333333	3.136625	182766	SIVA1
503.2469	-4354	1.23E-04	660	0.333333	3.135936	192698	AP3M1
363.3079	-4354	1.23E-04	628	0.333333	3.135936	172826	CLPTM1L
793.7911	-4354	1.23E-04	788	0.333333	3.135706	203320	UQCRB
468.6169	-4354	1.23E-04	626	0.333333	3.135017	186844	ACBD5
503.6927	-4354	1.23E-04	647	0.333333	3.134788	204792	GTSE1
421.2071	-4354	1.23E-04	629	0.333333	3.134788	174756	PTPN5
345.2055	-4354	1.23E-04	604	0.333333	3.134788	170518	HACD4
423.8194	-4354	1.23E-04	618	0.333333	3.134558	191236	ADGRG5
678.8663	-4354	1.23E-04	663	0.333333	3.134328	224838	CIT
379.8793	-4354	1.23E-04	607	0.333333	3.134328	189320	ALDH1L2
597.7437	-4354	1.23E-04	618	0.333333	3.133869	200500	GSTO2
492.2105	-4354	1.23E-04	722	0.333333	3.13318	206530	FZD8
509.8258	-4354	1.23E-04	631	0.333333	3.13318	196998	SLC38A9
491.5946	-4354	1.23E-04	698	0.333333	3.132721	196378	IGF2

538.5962	-4354	1.23E-04	701	0.333333	3.132032	206378	LTA4H
472.4357	-4354	1.23E-04	636	0.333333	3.132032	179590	ATG12
559.3266	-4354	1.23E-04	622	0.333333	3.132032	209768	ATP6V1E2
744.5637	-4354	1.23E-04	682	0.333333	3.131803	214062	GLB1
402.3774	-4354	1.23E-04	628	0.333333	3.131573	182262	SLC16A3
526.6773	-4354	1.23E-04	596	0.333333	3.130195	196644	SMCR8
288.5467	-4354	1.23E-04	604	0.333333	3.128358	154626	PLXNA4
379.539	-4354	1.23E-04	605	0.333333	3.127899	158186	EPHA6
512.0679	-4354	1.23E-04	594	0.333333	3.127669	167506	SYNGAP1
616.4472	-4354	1.23E-04	650	0.333333	3.125373	197966	CSNK1A1L
467.2614	-4354	1.22E-04	582	0.333333	3.123766	171016	SIX4
462.3287	-4354	1.22E-04	614	0.333333	3.123536	177150	FADS1
340.2681	-4354	1.22E-04	574	0.333333	3.123536	136024	IL36B
438.8506	-4354	1.22E-04	570	0.333333	3.122847	159808	ANAPC16
297.087	-4354	1.22E-04	575	0.333333	3.122618	141294	KSR2
416.5062	-4354	1.22E-04	587	0.333333	3.120781	156970	SOCS4
482.0289	-4354	1.22E-04	788	0.333333	3.120321	162532	PSMA6
350.747	-4354	1.22E-04	559	0.333333	3.120321	139914	ESYT3
450.3062	-4354	1.22E-04	566	0.333333	3.120092	170780	RAD9B
448.3298	-4354	1.22E-04	677	0.333333	3.119862	155188	ANAPC7
602.8224	-4354	1.22E-04	706	0.333333	3.119173	181342	ANAPC1
521.9512	-4354	1.22E-04	608	0.333333	3.118944	168456	CACNA1A
320.7075	-4354	1.22E-04	549	0.333333	3.118944	133072	COX11
465.9653	-4354	1.22E-04	605	0.333333	3.118025	174138	TICAM2
961.4844	-4354	1.22E-04	571	0.333333	3.118025	247088	MEF2B
350.8267	-4354	1.22E-04	548	0.333333	3.116418	148516	FOXK1
469.7197	-4354	1.22E-04	531	0.333333	3.116418	166870	HERC3
420.5922	-4354	1.22E-04	549	0.333333	3.116188	160382	AP4B1
532.3588	-4354	1.22E-04	581	0.333333	3.114811	155902	CALM2
482.3708	-4354	1.22E-04	621	0.333333	3.114351	139858	NPY4R
395.5338	-4354	1.22E-04	572	0.333333	3.114351	155628	ATP1A3
266.382	-4354	1.22E-04	548	0.333333	3.114122	121098	UBE2J2
407.4604	-4354	1.22E-04	547	0.333333	3.113892	150792	TRPM7
345.7988	-4354	1.22E-04	539	0.333333	3.113662	137708	KRIT1
447.0181	-4354	1.22E-04	555	0.333333	3.113433	162376	LIN9
428.4811	-4354	1.22E-04	645	0.333333	3.112974	128106	ANAPC11
343.5894	-4354	1.22E-04	519	0.333333	3.112514	134190	SLC46A1
492.8667	-4354	1.22E-04	544	0.333333	3.112055	148818	GANC
359.8965	-4354	1.22E-04	513	0.333333	3.111825	140152	FUNDC2
781.4765	-4354	1.22E-04	548	0.333333	3.111366	271862	PLA2G10
520.9224	-4354	1.22E-04	573	0.333333	3.110907	156608	CYP21A2

429.9426	-4354	1.22E-04	591	0.333333	3.110677	142590	CYP3A7
422.0518	-4354	1.22E-04	577	0.333333	3.110218	139552	LHB
242.0323	-4354	1.22E-04	519	0.333333	3.110218	106316	PPP3R2
300.0485	-4354	1.21E-04	507	0.333333	3.108611	133984	EPG5
246.2956	-4354	1.21E-04	503	0.333333	3.108381	97596	TRPV3
405.212	-4354	1.21E-04	487	0.333333	3.107463	141016	PLA2G4B
291.0424	-4354	1.21E-04	584	0.333333	3.106085	132254	CTNND1
9357.116	-4354	1.21E-04	582	0.333333	3.105396	1258222	UQCRH
313.4284	-4354	1.21E-04	543	0.333333	3.103559	110348	UBD
249.3126	-4354	1.21E-04	580	0.333333	3.10333	99614	HLA-DRB5
193.7395	-4354	1.21E-04	466	0.333333	3.10287	101390	CASTOR1
392.3564	-4354	1.21E-04	516	0.333333	3.101952	123226	GDF1
337.488	-4354	1.21E-04	510	0.333333	3.101722	116680	PI4K2B
305.0857	-4354	1.21E-04	514	0.333333	3.100344	106340	CCL3
281.7365	-4354	1.21E-04	508	0.333333	3.100115	112560	C4B
748.8652	-4354	1.21E-04	456	0.333333	3.100115	292828	PPP1R3F
240.3427	-4354	1.21E-04	467	0.333333	3.099885	99780	MYLK4
279.5363	-4354	1.21E-04	521	0.333333	3.099656	112840	HSPA1A
187.0801	-4354	1.21E-04	450	0.333333	3.099656	92720	ATG9B
283.7918	-4354	1.21E-04	467	0.333333	3.099426	105282	ELOVL1
358.9843	-4354	1.21E-04	596	0.333333	3.098737	111394	PSME2
333.9915	-4354	1.21E-04	491	0.333333	3.098048	121430	PRICKLE4
262.1485	-4354	1.21E-04	467	0.333333	3.09713	104172	UBA7
264.7116	-4354	1.21E-04	445	0.333333	3.095522	88306	CACNG8
243.3922	-4354	1.21E-04	460	0.333333	3.095293	96498	TNFSF12
245.2623	-4354	1.21E-04	433	0.333333	3.094374	91234	PFN4
281.6965	-4354	1.21E-04	459	0.333333	3.093915	106886	GDF6
547.4712	-4354	1.20E-04	487	0.333333	3.092767	132900	BMI1
257.7692	-4354	1.20E-04	436	0.333333	3.092308	87300	SLC2A14
392.0322	-4354	1.20E-04	500	0.333333	3.091848	110744	POU5F1
333.8556	-4354	1.20E-04	453	0.333333	3.091848	108054	AMH
410.2867	-4354	1.20E-04	520	0.333333	3.091389	95360	NDUFA11
195.6089	-4354	1.20E-04	423	0.333333	3.091389	85742	ACSF3
259.5008	-4354	1.20E-04	515	0.333333	3.089323	91980	VTN
238.8501	-4354	1.20E-04	468	0.333333	3.089093	96924	GP1BB
195.7147	-4354	1.20E-04	421	0.333333	3.089093	72186	MICOS10
293.1612	-4354	1.20E-04	470	0.333333	3.085419	92788	XCL2
341.8944	-4354	1.20E-04	394	0.333333	3.085419	93880	AMY1A
335.9035	-4354	1.20E-04	502	0.333333	3.084501	100152	ORM2
141.6911	-4354	1.20E-04	460	0.333333	3.084041	59278	HLA-DRB3
184.9744	-4354	1.20E-04	430	0.333333	3.083582	74550	VEGFD

254.52	-4354	1.20E-04	458	0.333333	3.082893	84992	WNT3A
213.7574	-4354	1.20E-04	393	0.333333	3.081515	84886	FTMT
318.2475	-4354	1.20E-04	439	0.333333	3.081056	86458	HOXA9
282.7323	-4354	1.20E-04	429	0.333333	3.080367	84040	TUBA3C
349.0941	-4354	1.20E-04	529	0.333333	3.079908	82960	NDUFS5
206.109	-4354	1.20E-04	451	0.333333	3.079449	68036	CFHR1
434.0244	-4354	1.20E-04	425	0.333333	3.07899	149804	CHMP3
284.229	-4354	1.20E-04	419	0.333333	3.07899	83322	TUBB8
196.5112	-4354	1.20E-04	383	0.333333	3.07899	79496	ARHGEF18
176.8472	-4354	1.20E-04	369	0.333333	3.07853	67056	LIN52
226.3631	-4354	1.20E-04	387	0.333333	3.078301	79524	BGLAP
201.621	-4354	1.20E-04	413	0.333333	3.078071	65886	OPN1MW
257.3544	-4354	1.20E-04	401	0.333333	3.078071	80006	CCL14
570.0422	-4354	1.20E-04	360	0.333333	3.077842	185028	PPP1R3E
251.1988	-4354	1.20E-04	410	0.333333	3.077382	76520	CALM3
190.1524	-4354	1.19E-04	399	0.333333	3.076923	68256	NTF4
190.9846	-4354	1.19E-04	410	0.333333	3.076464	72786	WNT3
224.3192	-4354	1.19E-04	371	0.333333	3.075775	71320	LCE1F
152.1766	-4354	1.19E-04	385	0.333333	3.073938	60260	LEFTY2
429.9582	-4354	1.19E-04	384	0.333333	3.073708	139312	CHMP4A
169.4495	-4354	1.19E-04	365	0.333333	3.071871	61004	AQP7
154.7654	-4354	1.19E-04	370	0.333333	3.071183	61194	ACOT2
192.6347	-4354	1.19E-04	378	0.333333	3.070034	64046	CSH2
228.6874	-4354	1.19E-04	381	0.333333	3.069805	58684	GSTA2
238.2416	-4354	1.19E-04	351	0.333333	3.069346	62052	GBA
223.6753	-4354	1.19E-04	370	0.333333	3.067509	58094	IFNA6
121.7817	-4354	1.19E-04	358	0.333333	3.067049	47512	KLRK1
140.3636	-4354	1.19E-04	350	0.333333	3.065672	57492	EEF2K
142.7637	-4354	1.19E-04	342	0.333333	3.065672	54116	AP3S2
131.6313	-4354	1.19E-04	330	0.333333	3.064524	42994	OPN1LW
165.7845	-4354	1.19E-04	299	0.333333	3.063835	52342	CHRNA7
170.5572	-4354	1.19E-04	310	0.333333	3.063605	52328	CPT1B
182.6743	-4354	1.19E-04	300	0.333333	3.062916	49092	FAM180A
226.9197	-4354	1.19E-04	306	0.333333	3.062227	57392	CCL15
186.6165	-4354	1.18E-04	304	0.333333	3.061079	52906	ACYP2
148.5495	-4354	1.18E-04	316	0.333333	3.06062	45762	SGK3
202.8879	-4354	1.18E-04	303	0.333333	3.06062	50824	JMJD7-PLA2G4B
101.6319	-4354	1.18E-04	302	0.333333	3.059013	42840	DTX2
115.1938	-4354	1.18E-04	327	0.333333	3.056028	41938	RPS13
108.1137	-4354	1.18E-04	266	0.333333	3.055109	42266	PDE8B
204.5066	-4354	1.18E-04	281	0.333333	3.05465	37610	GSTA5

109.7946	-4354	1.18E-04	264	0.333333	3.052813	37224	PPT2
120.764	-4354	1.18E-04	302	0.333333	3.052583	38492	OMG
99.00881	-4354	1.18E-04	269	0.333333	3.051894	37120	C4A
121.5806	-4354	1.18E-04	272	0.333333	3.051435	35000	SYVN1
115.9759	-4354	1.18E-04	269	0.333333	3.051435	36158	ASIC3
125.593	-4354	1.18E-04	279	0.333333	3.050746	34652	HOXD3
883.4602	-4354	1.18E-04	253	0.333333	3.049828	438658	DEFA3
112.4015	-4354	1.18E-04	252	0.333333	3.049369	35352	FADS6
84.85072	-4354	1.18E-04	276	0.333333	3.04822	28762	SAA2
77.65664	-4354	1.18E-04	241	0.333333	3.047532	29822	IRS4
99.85861	-4354	1.18E-04	242	0.333333	3.046154	29430	TNFRSF6B
107.4526	-4354	1.18E-04	239	0.333333	3.045695	30694	KRT34
75.28789	-4354	1.18E-04	224	0.333333	3.045695	23046	GPX6
100.0114	-4354	1.18E-04	244	0.333333	3.044776	32188	MTMR8
62.65887	-4354	1.17E-04	223	0.333333	3.044317	24822	AKR1C2
37.53756	-4354	1.17E-04	316	0.333333	3.043858	15254	HLA-DQA2
98.51789	-4354	1.17E-04	210	0.333333	3.043628	26744	LCE1D
68.20383	-4354	1.17E-04	229	0.333333	3.04248	24940	DUSP8
78.0021	-4354	1.17E-04	223	0.333333	3.041791	23980	TOMM7
63.1959	-4354	1.17E-04	217	0.333333	3.041791	21918	ACOT1
62.56566	-4354	1.17E-04	215	0.333333	3.040873	23888	CA13
1068.2	-4354	1.17E-04	296	0.333333	3.040643	311462	NDUFC2
82.01907	-4354	1.17E-04	208	0.333333	3.038576	21612	SSX2
55.15253	-4354	1.17E-04	229	0.333333	3.036739	20722	HLA-DQA1
120.3402	-4354	1.17E-04	192	0.333333	3.03628	20344	AMY1C
73.00305	-4354	1.17E-04	189	0.333333	3.034673	19716	TRPV1
18.77997	-4354	1.17E-04	200	0.333333	3.033525	7798	CSNK2A3
43.27549	-4354	1.17E-04	167	0.333333	3.032147	16336	ASIC5
89.4447	-4354	1.17E-04	170	0.333333	3.03008	19662	CLDN22
41.49822	-4354	1.17E-04	169	0.333333	3.029392	14152	BBC3
7.617443	-4354	1.16E-04	169	0.333333	3.027784	3326	WEE2
31.18919	-4354	1.16E-04	153	0.333333	3.026406	12072	PLA2G4E
30.14405	-4354	1.16E-04	147	0.333333	3.025488	11424	TXNRD3
33.92325	-4354	1.16E-04	142	0.333333	3.025258	11602	CYP4A22
67.47185	-4354	1.16E-04	143	0.333333	3.024569	14098	FABP12
50.25091	-4354	1.16E-04	143	0.333333	3.023881	11212	RASA4
20.755	-4354	1.16E-04	120	0.333333	3.021125	6934	UGT3A2
25.02132	-4354	1.16E-04	129	0.333333	3.020896	8522	HSPA1B
41.1111	-4354	1.16E-04	128	0.333333	3.019977	8106	AMY2A
39.49987	-4354	1.16E-04	123	0.333333	3.017222	6512	TUBA3D
9.452272	-4354	1.16E-04	106	0.333333	3.015385	3940	IL31

69.98963	-4354	1.16E-04	100	0.333333	3.014696	13002	NHLRC1
15.8928	-4354	1.16E-04	95	0.333333	3.014237	3702	KRTAP12-2
16.78796	-4354	1.16E-04	92	0.333333	3.0124	4318	PFN3
4.423044	-4354	1.16E-04	139	0.333333	3.011251	1540	PSMB11
24.27498	-4354	1.16E-04	89	0.333333	3.011251	6290	CCL3L1
10.21889	-4354	1.16E-04	98	0.333333	3.010792	3646	IFNA13
10.55128	-4354	1.15E-04	87	0.333333	3.009644	3510	IFNL3
16.77168	-4354	1.15E-04	84	0.333333	3.008496	3072	AMY2B
15.96527	-4354	1.15E-04	107	0.333333	3.008266	2116	MT-ATP8
7.989549	-4354	1.15E-04	76	0.333333	3.008266	2622	STRA8
5.628847	-4354	1.15E-04	67	0.333333	3.007807	2536	KRTAP5-1
9.107413	-4354	1.15E-04	136	0.333333	3.006659	2422	MT-ND5
253.7091	-4354	1.15E-04	78	0.333333	3.006659	128784	DEFA1
2.935122	-4354	1.15E-04	65	0.333333	3.004133	1192	C8orf44-SGK3
3.782234	-4354	1.15E-04	126	0.333333	3.003904	1136	MT-ND3
5.96986	-4354	1.15E-04	99	0.333333	3.003674	832	MT-CYB
4.661375	-4354	1.15E-04	126	0.333333	3.003215	1162	MT-ND4L
5.170664	-4354	1.15E-04	50	0.333333	3.002526	1524	AWAT2
1.710757	-4354	1.15E-04	50	0.333333	2.997704	446	NANOGP8
2.85626	-4354	1.14E-04	60	0.333333	2.992882	542	MT-CO3
0.04045	-4354	1.14E-04	34	0.333333	2.992882	16	AMY1B
0.309977	-4354	1.14E-04	31	0.333333	2.990815	162	SLC2A7
0.423996	-4354	1.14E-04	27	0.333333	2.988519	176	RASA4B
0.880203	-4354	1.14E-04	48	0.333333	2.9876	212	TUBA3E
0.037448	-4354	1.14E-04	22	0.333333	2.982549	6	GSTT2B
530.5325	-4354	1.14E-04	18	0.333333	2.978645	102536	KRTAP10-7
0	-4354	1.14E-04	50	0.25	2.977268	0	CCL4L2
0	-4354	1.14E-04	44	0.25	2.977268	0	CCL3L3
0	-4354	1.13E-04	44	0.333333	2.973823	0	MT-ND1
0.07912	-4354	1.13E-04	16	0.25	2.968542	38	PGAM4
0	-4354	1.13E-04	30	0.333333	2.967394	0	CLDN25
0	-4354	1.13E-04	30	0.333333	2.967394	0	CLDN24
0.107245	-4354	1.13E-04	12	0.25	2.960046	52	BUB1B-PAK6
0.221899	-4354	1.12E-04	14	0.333333	2.947876	26	COMMD3-BMI1
0.095296	-4354	1.12E-04	16	0.25	2.942135	20	FABP9
0.034058	-4354	1.10E-04	10	0.25	2.918025	14	BORCS8-MEF2B
0	-4354	1.09E-04	13	0.333333	2.883812	0	RNF103-CHMP3
0	-4354	1.08E-04	10	0.25	2.877842	0	PLA2G2C
12.95918	-4354	1.08E-04	11	0.25	2.871183	7124	DEFB4A

0.013245	-4354	1.06E-04	7	0.25	2.837887	2	PPP1R3G
0.007341	-4354	1.06E-04	6	0.25	2.834902	4	BECN2
3.770769	-4354	1.01E-04	10	0.25	2.735017	1506	DEFA1B
0	-4354	9.94E-05	3	0.25	2.688634	0	ENO4
0	-4354	9.91E-05	4	0.25	2.681056	0	EIF4E1B
71.22253	-4354	9.74E-05	7	0.25	2.641102	13380	KRTAP10-9
0	-4354	9.60E-05	7	0.25	2.607807	0	DEFB4B
22.05214	-4354	8.84E-05	6	0.25	2.400459	4486	KRTAP10-4
0	-4354	8.59E-05	2	0.25	2.325832	0	NDUFC2-KCTD14
0	-4354	8.29E-05	1	0.25	2.227784	0	RPEL1
0	-4354	8.04E-05	5	0.25	2.143972	0	KRTAP5-4
0	-4354	8.04E-05	5	0.25	2.143972	0	KRTAP10-2
0	-4354	7.94E-05	1	0.25	2.105855	0	UQCRHL
0	4354	0	0	0	0	0	CLDN34

Supplementary Table S6: a total of 39 genes were generated through the processing of 4340 genes associated with anti-VEGF resistance	
UBC	ubiquitin C [Source:HGNC Symbol;Acc:HGNC:12468]
BMP4	bone morphogenetic protein 4 [Source:HGNC Symbol;Acc:HGNC:1071]
FMOD	fibromodulin [Source:HGNC Symbol;Acc:HGNC:3774]
DRD5	dopamine receptor D5 [Source:HGNC Symbol;Acc:HGNC:3026]
CDH3	cadherin 3 [Source:HGNC Symbol;Acc:HGNC:1762]
UBB	ubiquitin B [Source:HGNC Symbol;Acc:HGNC:12463]
TCF7L1	transcription factor 7 like 1 [Source:HGNC Symbol;Acc:HGNC:11640]
YWHAE	tyrosine 3-monooxygenase/tryptophan 5-monooxygenase activation protein epsilon [Source:HGNC Symbol;Acc:HGNC:12851]
PIK3R1	phosphoinositide-3-kinase regulatory subunit 1 [Source:HGNC Symbol;Acc:HGNC:8979]
EGFR	epidermal growth factor receptor [Source:HGNC Symbol;Acc:HGNC:3236]
MAPK3	mitogen-activated protein kinase 3 [Source:HGNC Symbol;Acc:HGNC:6877]
MYC	v-myc avian myelocytomatosis viral oncogene homolog [Source:HGNC Symbol;Acc:HGNC:7553]
NFKB1	nuclear factor kappa B subunit 1 [Source:HGNC Symbol;Acc:HGNC:7794]
CTNNB1	catenin beta 1 [Source:HGNC Symbol;Acc:HGNC:2514]
PPARG	peroxisome proliferator activated receptor gamma [Source:HGNC Symbol;Acc:HGNC:9236]
PRL	prolactin [Source:HGNC Symbol;Acc:HGNC:9445]
PRKCQ	protein kinase C theta [Source:HGNC Symbol;Acc:HGNC:9410]
TMSB4X	thymosin beta 4, X-linked [Source:HGNC Symbol;Acc:HGNC:11881]
CLIC1	chloride intracellular channel 1 [Source:HGNC Symbol;Acc:HGNC:2062]
VEGFC	vascular endothelial growth factor C [Source:HGNC Symbol;Acc:HGNC:12682]
KRTAP4-1	keratin associated protein 4-1 [Source:HGNC Symbol;Acc:HGNC:18907]
WT1	Wilms tumor 1 [Source:HGNC Symbol;Acc:HGNC:12796]
ENO2	enolase 2 [Source:HGNC Symbol;Acc:HGNC:3353]
RAC1	ras-related C3 botulinum toxin substrate 1 (rho family, small GTP binding protein Rac1) [Source:HGNC Symbol;Acc:HGNC:9801]
RPE	ribulose-5-phosphate-3-epimerase [Source:HGNC Symbol;Acc:HGNC:10293]
PLA2G2D	phospholipase A2 group IID [Source:HGNC Symbol;Acc:HGNC:9033]
DEFA5	defensin alpha 5 [Source:HGNC Symbol;Acc:HGNC:2764]
UVRAG	UV radiation resistance associated [Source:HGNC Symbol;Acc:HGNC:12640]
ADCY6	adenylate cyclase 6 [Source:HGNC Symbol;Acc:HGNC:237]
UQCRH	ubiquinol-cytochrome c reductase hinge protein [Source:HGNC Symbol;Acc:HGNC:12590]
ITPKC	inositol-trisphosphate 3-kinase C [Source:HGNC Symbol;Acc:HGNC:14897]
FOXF2	forkhead box F2 [Source:HGNC Symbol;Acc:HGNC:3810]
CLDN10	claudin 10 [Source:HGNC Symbol;Acc:HGNC:2033]
TUBA1A	tubulin alpha 1a [Source:HGNC Symbol;Acc:HGNC:20766]
EDAR	ectodysplasin A receptor [Source:HGNC Symbol;Acc:HGNC:2895]
PLD2	phospholipase D2 [Source:HGNC Symbol;Acc:HGNC:9068]
ATP12A	ATPase H+/K+ transporting non-gastric alpha2 subunit [Source:HGNC Symbol;Acc:HGNC:13816]
TINAGL1	tubulointerstitial nephritis antigen like 1 [Source:HGNC Symbol;Acc:HGNC:19168]

CLDN34	claudin 34 [Source:HGNC Symbol;Acc:HGNC:51259]

Supplementary Table S7: a total of 52 genes were generated through the processing of 7061 genes associated with nAMD disease	
UBC	ubiquitin C [Source:HGNC Symbol;Acc:HGNC:12468]
APP	amyloid beta precursor protein [Source:HGNC Symbol;Acc:HGNC:620]
FN1	fibronectin 1 [Source:HGNC Symbol;Acc:HGNC:3778]
JUN	Jun proto-oncogene, AP-1 transcription factor subunit [Source:HGNC Symbol;Acc:HGNC:6204]
EGFR	epidermal growth factor receptor [Source:HGNC Symbol;Acc:HGNC:3236]
AUTS2	autism susceptibility candidate 2 [Source:HGNC Symbol;Acc:HGNC:14262]
XPO1	exportin 1 [Source:HGNC Symbol;Acc:HGNC:12825]
CTNNB1	catenin beta 1 [Source:HGNC Symbol;Acc:HGNC:2514]
PPP3CA	protein phosphatase 3 catalytic subunit alpha [Source:HGNC Symbol;Acc:HGNC:9314]
SRPK1	SRSF protein kinase 1 [Source:HGNC Symbol;Acc:HGNC:11305]
KCNIP4	potassium voltage-gated channel interacting protein 4 [Source:HGNC Symbol;Acc:HGNC:30083]
PIK3R1	phosphoinositide-3-kinase regulatory subunit 1 [Source:HGNC Symbol;Acc:HGNC:8979]
RDX	radixin [Source:HGNC Symbol;Acc:HGNC:9944]
HNRNPD	heterogeneous nuclear ribonucleoprotein D [Source:HGNC Symbol;Acc:HGNC:5036]
NTRK2	neurotrophic receptor tyrosine kinase 2 [Source:HGNC Symbol;Acc:HGNC:8032]
MUC1	mucin 1, cell surface associated [Source:HGNC Symbol;Acc:HGNC:7508]
DLG2	discs large MAGUK scaffold protein 2 [Source:HGNC Symbol;Acc:HGNC:2901]
CAND1	cullin associated and neddylation dissociated 1 [Source:HGNC Symbol;Acc:HGNC:30688]
DAPK1	death associated protein kinase 1 [Source:HGNC Symbol;Acc:HGNC:2674]
RFC3	replication factor C subunit 3 [Source:HGNC Symbol;Acc:HGNC:9971]
DAB1	DAB1, reelin adaptor protein [Source:HGNC Symbol;Acc:HGNC:2661]
PIWIL1	piwi like RNA-mediated gene silencing 1 [Source:HGNC Symbol;Acc:HGNC:9007]
PRL	prolactin [Source:HGNC Symbol;Acc:HGNC:9445]
FOXP1	forkhead box P1 [Source:HGNC Symbol;Acc:HGNC:3823]
UQCRFS1	ubiquinol-cytochrome c reductase, Rieske iron-sulfur polypeptide 1 [Source:HGNC Symbol;Acc:HGNC:12587]
PARK2	parkin RBR E3 ubiquitin protein ligase [Source:HGNC Symbol;Acc:HGNC:8607]
TCF4	transcription factor 4 [Source:HGNC Symbol;Acc:HGNC:11634]
PBX1	PBX homeobox 1 [Source:HGNC Symbol;Acc:HGNC:8632]
PRAMEF14	PRAME family member 14 [Source:HGNC Symbol;Acc:HGNC:13576]
PRAMEF1	PRAME family member 1 [Source:HGNC Symbol;Acc:HGNC:28840]
SPDYE3	speedy/RINGO cell cycle regulator family member E3 [Source:HGNC Symbol;Acc:HGNC:35462]
SPDYE1	speedy/RINGO cell cycle regulator family member E1 [Source:HGNC Symbol;Acc:HGNC:16408]
SPDYE2	speedy/RINGO cell cycle regulator family member E2 [Source:HGNC Symbol;Acc:HGNC:33841]
KRTAP5-2	keratin associated protein 5-2 [Source:HGNC Symbol;Acc:HGNC:23597]
OR1J1	olfactory receptor family 1 subfamily J member 1 [Source:HGNC Symbol;Acc:HGNC:8208]
OR8B2	olfactory receptor family 8 subfamily B member 2 [Source:HGNC Symbol;Acc:HGNC:8471]
OR4D5	olfactory receptor family 4 subfamily D member 5 [Source:HGNC Symbol;Acc:HGNC:14852]
OR4X1	olfactory receptor family 4 subfamily X member 1 (gene/pseudogene) [Source:HGNC Symbol;Acc:HGNC:14854]

OR5B3	olfactory receptor family 5 subfamily B member 3 [Source:HGNC Symbol;Acc:HGNC:8324]
OR2Y1	olfactory receptor family 2 subfamily Y member 1 [Source:HGNC Symbol;Acc:HGNC:14837]
KRTAP2-1	keratin associated protein 2-1 [Source:HGNC Symbol;Acc:HGNC:16775]
OR5AN1	olfactory receptor family 5 subfamily AN member 1 [Source:HGNC Symbol;Acc:HGNC:15255]
OR4Q3	olfactory receptor family 4 subfamily Q member 3 [Source:HGNC Symbol;Acc:HGNC:15426]
OR10P1	olfactory receptor family 10 subfamily P member 1 [Source:HGNC Symbol;Acc:HGNC:15378]
OR8B8	olfactory receptor family 8 subfamily B member 8 [Source:HGNC Symbol;Acc:HGNC:8477]
OR51B4	olfactory receptor family 51 subfamily B member 4 [Source:HGNC Symbol;Acc:HGNC:14708]
OR10J1	olfactory receptor family 10 subfamily J member 1 [Source:HGNC Symbol;Acc:HGNC:8175]
CT45A5	cancer/testis antigen family 45, member A5 [Source:HGNC Symbol;Acc:HGNC:33270]
FRG2C	FSHD region gene 2 family member C [Source:HGNC Symbol;Acc:HGNC:33626]
FRG2B	FSHD region gene 2 family member B [Source:HGNC Symbol;Acc:HGNC:33518]
PLXDC2	plexin domain containing 2 [Source:HGNC Symbol;Acc:HGNC:21013]
IQGAP2	IQ motif containing GTPase activating protein 2 [Source:HGNC Symbol;Acc:HGNC:6111]

Supplementary Table S8: The integration of the MuST and DIAMOnD results, anti-VEGF resistance and nAMD related Hub genes	
ABCA4	ATP binding cassette subfamily A member 4 [Source:HGNC Symbol;Acc:HGNC:34]
VEGFA	vascular endothelial growth factor A [Source:HGNC Symbol;Acc:HGNC:12680]
RAB1A	RAB1A, member RAS oncogene family [Source:HGNC Symbol;Acc:HGNC:9758]
TNFRSF10A	tumor necrosis factor receptor superfamily member 10a [Source:HGNC Symbol;Acc:HGNC:11904]
ERCC6	ERCC excision repair 6, chromatin remodeling factor [Source:HGNC Symbol;Acc:HGNC:3438]
C1QTNF5	C1q and tumor necrosis factor related protein 5 [Source:HGNC Symbol;Acc:HGNC:14344]
MYC	v-myc avian myelocytomatosis viral oncogene homolog [Source:HGNC Symbol;Acc:HGNC:7553]
NEFM	neurofilament, medium polypeptide [Source:HGNC Symbol;Acc:HGNC:7734]
C9	complement component 9 [Source:HGNC Symbol;Acc:HGNC:1358]
IRAK1	interleukin 1 receptor associated kinase 1 [Source:HGNC Symbol;Acc:HGNC:6112]
C2	complement component 2 [Source:HGNC Symbol;Acc:HGNC:1248]
C3	complement component 3 [Source:HGNC Symbol;Acc:HGNC:1318]
NPM1	nucleophosmin (nucleolar phosphoprotein B23, numatrin) [Source:HGNC Symbol;Acc:HGNC:7910]
TLR4	toll like receptor 4 [Source:HGNC Symbol;Acc:HGNC:11850]
SLC16A8	solute carrier family 16 member 8 [Source:HGNC Symbol;Acc:HGNC:16270]
HIF1A	hypoxia inducible factor 1 alpha subunit [Source:HGNC Symbol;Acc:HGNC:4910]
TIMP3	TIMP metallopeptidase inhibitor 3 [Source:HGNC Symbol;Acc:HGNC:11822]
IQCB1	IQ motif containing B1 [Source:HGNC Symbol;Acc:HGNC:28949]
UBA52	ubiquitin A-52 residue ribosomal protein fusion product 1 [Source:HGNC Symbol;Acc:HGNC:12458]
RPGR	retinitis pigmentosa GTPase regulator [Source:HGNC Symbol;Acc:HGNC:10295]
VWCE	von Willebrand factor C and EGF domains [Source:HGNC Symbol;Acc:HGNC:26487]
CFH	complement factor H [Source:HGNC Symbol;Acc:HGNC:4883]
NMNAT1	nicotinamide nucleotide adenylyltransferase 1 [Source:HGNC Symbol;Acc:HGNC:17877]
TGM2	transglutaminase 2 [Source:HGNC Symbol;Acc:HGNC:11778]
CFI	complement factor I [Source:HGNC Symbol;Acc:HGNC:5394]
APOE	apolipoprotein E [Source:HGNC Symbol;Acc:HGNC:613]
COPS6	COP9 signalosome subunit 6 [Source:HGNC Symbol;Acc:HGNC:21749]
SQSTM1	sequestosome 1 [Source:HGNC Symbol;Acc:HGNC:11280]
SLC2A4	solute carrier family 2 member 4 [Source:HGNC Symbol;Acc:HGNC:11009]
CFB	complement factor B [Source:HGNC Symbol;Acc:HGNC:1037]
KDM5B	lysine demethylase 5B [Source:HGNC Symbol;Acc:HGNC:18039]
LTBP1	latent transforming growth factor beta binding protein 1 [Source:HGNC Symbol;Acc:HGNC:6714]
SERPINA1	serpin family A member 1 [Source:HGNC Symbol;Acc:HGNC:8941]
CD46	CD46 molecule [Source:HGNC Symbol;Acc:HGNC:6953]
VCAN	versican [Source:HGNC Symbol;Acc:HGNC:2464]

ANTXR2	anthrax toxin receptor 2 [Source:HGNC Symbol;Acc:HGNC:21732]
SNX27	sorting nexin family member 27 [Source:HGNC Symbol;Acc:HGNC:20073]
APP	amyloid beta precursor protein [Source:HGNC Symbol;Acc:HGNC:620]
P3H3	prolyl 3-hydroxylase 3 [Source:HGNC Symbol;Acc:HGNC:19318]
KLK3	kallikrein related peptidase 3 [Source:HGNC Symbol;Acc:HGNC:6364]
LAMA1	laminin subunit alpha 1 [Source:HGNC Symbol;Acc:HGNC:6481]
ADAMTS1	ADAM metallopeptidase with thrombospondin type 1 motif 1 [Source:HGNC Symbol;Acc:HGNC:217]
SERPINA3	serpin family A member 3 [Source:HGNC Symbol;Acc:HGNC:16]
VWF	von Willebrand factor [Source:HGNC Symbol;Acc:HGNC:12726]
PDGFA	platelet derived growth factor subunit A [Source:HGNC Symbol;Acc:HGNC:8799]
PDGFB	platelet derived growth factor subunit B [Source:HGNC Symbol;Acc:HGNC:8800]
CST3	cystatin C [Source:HGNC Symbol;Acc:HGNC:2475]
SERPINE2	serpin family E member 2 [Source:HGNC Symbol;Acc:HGNC:8951]
A2M	alpha-2-macroglobulin [Source:HGNC Symbol;Acc:HGNC:7]
PXN	paxillin [Source:HGNC Symbol;Acc:HGNC:9718]
VTN	vitronectin [Source:HGNC Symbol;Acc:HGNC:12724]
SERPINB6	serpin family B member 6 [Source:HGNC Symbol;Acc:HGNC:8950]
CX3CL1	C-X3-C motif chemokine ligand 1 [Source:HGNC Symbol;Acc:HGNC:10647]
CPB2	carboxypeptidase B2 [Source:HGNC Symbol;Acc:HGNC:2300]
COL18A1	collagen type XVIII alpha 1 chain [Source:HGNC Symbol;Acc:HGNC:2195]
CP	ceruloplasmin [Source:HGNC Symbol;Acc:HGNC:2295]
AMBP	alpha-1-microglobulin/bikunin precursor [Source:HGNC Symbol;Acc:HGNC:453]
FBN2	fibrillin 2 [Source:HGNC Symbol;Acc:HGNC:3604]
FBN1	fibrillin 1 [Source:HGNC Symbol;Acc:HGNC:3603]
CALR	calreticulin [Source:HGNC Symbol;Acc:HGNC:1455]
SERPINE1	serpin family E member 1 [Source:HGNC Symbol;Acc:HGNC:8583]
CXCL12	C-X-C motif chemokine ligand 12 [Source:HGNC Symbol;Acc:HGNC:10672]
ITGA2	integrin subunit alpha 2 [Source:HGNC Symbol;Acc:HGNC:6137]
FCN2	ficolin 2 [Source:HGNC Symbol;Acc:HGNC:3624]
CTGF (CCN2)	connective tissue growth factor [Source:HGNC Symbol;Acc:HGNC:2500]
FN1	fibronectin 1 [Source:HGNC Symbol;Acc:HGNC:3778]
DPT	dermatopontin [Source:HGNC Symbol;Acc:HGNC:3011]
CD36	CD36 molecule [Source:HGNC Symbol;Acc:HGNC:1663]
FCN1	ficolin 1 [Source:HGNC Symbol;Acc:HGNC:3623]
ACHE	acetylcholinesterase (Cartwright blood group) [Source:HGNC Symbol;Acc:HGNC:108]
MAG	myelin associated glycoprotein [Source:HGNC Symbol;Acc:HGNC:6783]
ITGAM	integrin subunit alpha M [Source:HGNC Symbol;Acc:HGNC:6149]
DEFA1B	defensin alpha 1B [Source:HGNC Symbol;Acc:HGNC:33596]
COLGALT2	collagen beta(1-O)galactosyltransferase 2 [Source:HGNC Symbol;Acc:HGNC:16790]

CR1	complement component 3b/4b receptor 1 (Knops blood group) [Source:HGNC Symbol;Acc:HGNC:2334]
SERPING1	serpin family G member 1 [Source:HGNC Symbol;Acc:HGNC:1228]
C1QA	complement component 1, q subcomponent, A chain [Source:HGNC Symbol;Acc:HGNC:1241]
ITGA2B	integrin subunit alpha 2b [Source:HGNC Symbol;Acc:HGNC:6138]
C1QB	complement component 1, q subcomponent, B chain [Source:HGNC Symbol;Acc:HGNC:1242]
IGLL5	immunoglobulin lambda like polypeptide 5 [Source:HGNC Symbol;Acc:HGNC:38476]
C1QC	complement component 1, q subcomponent, C chain [Source:HGNC Symbol;Acc:HGNC:1245]
C1R	complement C1r subcomponent [Source:HGNC Symbol;Acc:HGNC:1246]
DDX19B	DEAD-box helicase 19B [Source:HGNC Symbol;Acc:HGNC:2742]
C1S	complement component 1, s subcomponent [Source:HGNC Symbol;Acc:HGNC:1247]
ITGA5	integrin subunit alpha 5 [Source:HGNC Symbol;Acc:HGNC:6141]
COL14A1	collagen type XIV alpha 1 chain [Source:HGNC Symbol;Acc:HGNC:2191]
PF4	platelet factor 4 [Source:HGNC Symbol;Acc:HGNC:8861]
ITGB3	integrin subunit beta 3 [Source:HGNC Symbol;Acc:HGNC:6156]
CFP	complement factor properdin [Source:HGNC Symbol;Acc:HGNC:8864]
SAA1	serum amyloid A1 [Source:HGNC Symbol;Acc:HGNC:10513]
FGA	fibrinogen alpha chain [Source:HGNC Symbol;Acc:HGNC:3661]
C1QTNF2	C1q and tumor necrosis factor related protein 2 [Source:HGNC Symbol;Acc:HGNC:14325]
TNFAIP6	TNF alpha induced protein 6 [Source:HGNC Symbol;Acc:HGNC:11898]
KLK13	kallikrein related peptidase 13 [Source:HGNC Symbol;Acc:HGNC:6361]
MASP2	mannan binding lectin serine peptidase 2 [Source:HGNC Symbol;Acc:HGNC:6902]
CD44	CD44 molecule (Indian blood group) [Source:HGNC Symbol;Acc:HGNC:1681]
C4A	complement component 4A (Rodgers blood group) [Source:HGNC Symbol;Acc:HGNC:1323]
C4B	complement component 4B (Chido blood group) [Source:HGNC Symbol;Acc:HGNC:1324]
C4BPA	complement component 4 binding protein alpha [Source:HGNC Symbol;Acc:HGNC:1325]
MYOC	myocilin [Source:HGNC Symbol;Acc:HGNC:7610]
ITGAV	integrin subunit alpha V [Source:HGNC Symbol;Acc:HGNC:6150]
PROC	protein C, inactivator of coagulation factors Va and VIIIa [Source:HGNC Symbol;Acc:HGNC:9451]
ITGB1	integrin subunit beta 1 [Source:HGNC Symbol;Acc:HGNC:6153]
C5	complement component 5 [Source:HGNC Symbol;Acc:HGNC:1331]
PROS1	protein S (alpha) [Source:HGNC Symbol;Acc:HGNC:9456]
TFAP2C	transcription factor AP-2 gamma [Source:HGNC Symbol;Acc:HGNC:11744]
COL4A4	collagen type IV alpha 4 chain [Source:HGNC Symbol;Acc:HGNC:2206]
COL4A3	collagen type IV alpha 3 chain [Source:HGNC Symbol;Acc:HGNC:2204]
COL4A2	collagen type IV alpha 2 [Source:HGNC Symbol;Acc:HGNC:2203]

FGF7	fibroblast growth factor 7 [Source:HGNC Symbol;Acc:HGNC:3685]
COL4A1	collagen type IV alpha 1 chain [Source:HGNC Symbol;Acc:HGNC:2202]
COL3A1	collagen type III alpha 1 chain [Source:HGNC Symbol;Acc:HGNC:2201]
COL2A1	collagen type II alpha 1 chain [Source:HGNC Symbol;Acc:HGNC:2200]
HSPG2	heparan sulfate proteoglycan 2 [Source:HGNC Symbol;Acc:HGNC:5273]
ELN	elastin [Source:HGNC Symbol;Acc:HGNC:3327]
FGF2	fibroblast growth factor 2 [Source:HGNC Symbol;Acc:HGNC:3676]
COL1A2	collagen type I alpha 2 chain [Source:HGNC Symbol;Acc:HGNC:2198]
COL1A1	collagen type I alpha 1 [Source:HGNC Symbol;Acc:HGNC:2197]
FGB	fibrinogen beta chain [Source:HGNC Symbol;Acc:HGNC:3662]
TF	transferrin [Source:HGNC Symbol;Acc:HGNC:11740]
TFPI	tissue factor pathway inhibitor [Source:HGNC Symbol;Acc:HGNC:11760]
TTR	transthyretin [Source:HGNC Symbol;Acc:HGNC:12405]
KLK6	kallikrein related peptidase 6 [Source:HGNC Symbol;Acc:HGNC:6367]
COL9A1	collagen type IX alpha 1 [Source:HGNC Symbol;Acc:HGNC:2217]
COL8A2	collagen type VIII alpha 2 [Source:HGNC Symbol;Acc:HGNC:2216]
COL7A1	collagen type VII alpha 1 [Source:HGNC Symbol;Acc:HGNC:2214]
COL6A2	collagen type VI alpha 2 [Source:HGNC Symbol;Acc:HGNC:2212]
COL6A1	collagen type VI alpha 1 [Source:HGNC Symbol;Acc:HGNC:2211]
COL5A1	collagen type V alpha 1 [Source:HGNC Symbol;Acc:HGNC:2209]
COL4A6	collagen type IV alpha 6 chain [Source:HGNC Symbol;Acc:HGNC:2208]
COL4A5	collagen type IV alpha 5 chain [Source:HGNC Symbol;Acc:HGNC:2207]
LAMA3	laminin subunit alpha 3 [Source:HGNC Symbol;Acc:HGNC:6483]
MMP1	matrix metallopeptidase 1 [Source:HGNC Symbol;Acc:HGNC:7155]
MMP2	matrix metallopeptidase 2 [Source:HGNC Symbol;Acc:HGNC:7166]
MMP3	matrix metallopeptidase 3 [Source:HGNC Symbol;Acc:HGNC:7173]
MMP7	matrix metallopeptidase 7 [Source:HGNC Symbol;Acc:HGNC:7174]
MASP1	mannan binding lectin serine peptidase 1 [Source:HGNC Symbol;Acc:HGNC:6901]
MMP9	matrix metallopeptidase 9 [Source:HGNC Symbol;Acc:HGNC:7176]
MMP10	matrix metallopeptidase 10 [Source:HGNC Symbol;Acc:HGNC:7156]
TGFBI	transforming growth factor beta induced [Source:HGNC Symbol;Acc:HGNC:11771]
DDX31	DEAD-box helicase 31 [Source:HGNC Symbol;Acc:HGNC:16715]
IGFBP3	insulin like growth factor binding protein 3 [Source:HGNC Symbol;Acc:HGNC:5472]
IGFBP2	insulin like growth factor binding protein 2 [Source:HGNC Symbol;Acc:HGNC:5471]
IGFBP1	insulin like growth factor binding protein 1 [Source:HGNC Symbol;Acc:HGNC:5469]
F5	coagulation factor V [Source:HGNC Symbol;Acc:HGNC:3542]
HP	haptoglobin [Source:HGNC Symbol;Acc:HGNC:5141]
TGFB1	transforming growth factor beta 1 [Source:HGNC Symbol;Acc:HGNC:11766]
IGF2	insulin like growth factor 2 [Source:HGNC Symbol;Acc:HGNC:5466]
AHSG	alpha 2-HS glycoprotein [Source:HGNC Symbol;Acc:HGNC:349]
LAMC1	laminin subunit gamma 1 [Source:HGNC Symbol;Acc:HGNC:6492]

F2	coagulation factor II, thrombin [Source:HGNC Symbol;Acc:HGNC:3535]
BGN	biglycan [Source:HGNC Symbol;Acc:HGNC:1044]
FGG	fibrinogen gamma chain [Source:HGNC Symbol;Acc:HGNC:3694]
LAMA5	laminin subunit alpha 5 [Source:HGNC Symbol;Acc:HGNC:6485]
LAMB1	laminin subunit beta 1 [Source:HGNC Symbol;Acc:HGNC:6486]
THBS1	thrombospondin 1 [Source:HGNC Symbol;Acc:HGNC:11785]
THBD	thrombomodulin [Source:HGNC Symbol;Acc:HGNC:11784]
PTPRK	protein tyrosine phosphatase, receptor type K [Source:HGNC Symbol;Acc:HGNC:9674]
F13A1	coagulation factor XIII A chain [Source:HGNC Symbol;Acc:HGNC:3531]
VKORC1	vitamin K epoxide reductase complex subunit 1 [Source:HGNC Symbol;Acc:HGNC:23663]
F11	coagulation factor XI [Source:HGNC Symbol;Acc:HGNC:3529]
MIS12	MIS12, kinetochore complex component [Source:HGNC Symbol;Acc:HGNC:24967]
C4B_2	complement component 4B (Chido blood group), copy 2 [Source:HGNC Symbol;Acc:HGNC:42398]
F10	coagulation factor X [Source:HGNC Symbol;Acc:HGNC:3528]
IGFBP5	insulin like growth factor binding protein 5 [Source:HGNC Symbol;Acc:HGNC:5474]
F8	coagulation factor VIII [Source:HGNC Symbol;Acc:HGNC:3546]
CA8	carbonic anhydrase 8 [Source:HGNC Symbol;Acc:HGNC:1382]
CD93	CD93 molecule [Source:HGNC Symbol;Acc:HGNC:15855]
BMP1	bone morphogenetic protein 1 [Source:HGNC Symbol;Acc:HGNC:1067]
PRELP	proline and arginine rich end leucine rich repeat protein [Source:HGNC Symbol;Acc:HGNC:9357]
PITX3	paired like homeodomain 3 [Source:HGNC Symbol;Acc:HGNC:9006]
CTRB1	chymotrypsinogen B1 [Source:HGNC Symbol;Acc:HGNC:2521]
GDPD1	glycerophosphodiester phosphodiesterase domain containing 1 [Source:HGNC Symbol;Acc:HGNC:20883]
PLA2G2A	phospholipase A2 group IIA [Source:HGNC Symbol;Acc:HGNC:9031]
HPX	hemopexin [Source:HGNC Symbol;Acc:HGNC:5171]
CTSL	cathepsin L [Source:HGNC Symbol;Acc:HGNC:2537]
DCN	decorin [Source:HGNC Symbol;Acc:HGNC:2705]
KLK1	kallikrein 1 [Source:HGNC Symbol;Acc:HGNC:6357]
CTSG	cathepsin G [Source:HGNC Symbol;Acc:HGNC:2532]
KLK2	kallikrein related peptidase 2 [Source:HGNC Symbol;Acc:HGNC:6363]
ELANE	elastase, neutrophil expressed [Source:HGNC Symbol;Acc:HGNC:3309]
MEP1A	meprin A subunit alpha [Source:HGNC Symbol;Acc:HGNC:7015]
LCAT	lecithin-cholesterol acyltransferase [Source:HGNC Symbol;Acc:HGNC:6522]
TIMP1	TIMP metallopeptidase inhibitor 1 [Source:HGNC Symbol;Acc:HGNC:11820]
ZNF558	zinc finger protein 558 [Source:HGNC Symbol;Acc:HGNC:26422]
HRG	histidine rich glycoprotein [Source:HGNC Symbol;Acc:HGNC:5181]
HAPLN1	hyaluronan and proteoglycan link protein 1 [Source:HGNC Symbol;Acc:HGNC:2380]
KNG1	kininogen 1 [Source:HGNC Symbol;Acc:HGNC:6383]

CRP	C-reactive protein, pentraxin-related [Source:HGNC Symbol;Acc:HGNC:2367]
HABP2	hyaluronan binding protein 2 [Source:HGNC Symbol;Acc:HGNC:4798]
MFAP2	microfibrillar associated protein 2 [Source:HGNC Symbol;Acc:HGNC:7033]
PLAT	plasminogen activator, tissue type [Source:HGNC Symbol;Acc:HGNC:9051]
PLAU	plasminogen activator, urokinase [Source:HGNC Symbol;Acc:HGNC:9052]
ECE1	endothelin converting enzyme 1 [Source:HGNC Symbol;Acc:HGNC:3146]
PLG	plasminogen [Source:HGNC Symbol;Acc:HGNC:9071]
SERPINF2	serpin family F member 2 [Source:HGNC Symbol;Acc:HGNC:9075]
FBLN2	fibulin 2 [Source:HGNC Symbol;Acc:HGNC:3601]
COLQ	collagen-like tail subunit (single strand of homotrimer) of asymmetric acetylcholinesterase [Source:HGNC Symbol;Acc:HGNC:2226]
NID2	nidogen 2 [Source:HGNC Symbol;Acc:HGNC:13389]
FBLN1	fibulin 1 [Source:HGNC Symbol;Acc:HGNC:3600]
LGALS3	galectin 3 [Source:HGNC Symbol;Acc:HGNC:6563]
FAM20C	family with sequence similarity 20 member C [Source:HGNC Symbol;Acc:HGNC:22140]
PLOD1	procollagen-lysine,2-oxoglutarate 5-dioxygenase 1 [Source:HGNC Symbol;Acc:HGNC:9081]
MAPK6	mitogen-activated protein kinase 6 [Source:HGNC Symbol;Acc:HGNC:6879]
SERPIND1	serpin family D member 1 [Source:HGNC Symbol;Acc:HGNC:4838]
BCAN	brevican [Source:HGNC Symbol;Acc:HGNC:23059]
DEFA1	defensin alpha 1 [Source:HGNC Symbol;Acc:HGNC:2761]
APCS	amyloid P component, serum [Source:HGNC Symbol;Acc:HGNC:584]
SPARC	secreted protein acidic and cysteine rich [Source:HGNC Symbol;Acc:HGNC:11219]
SERPINA5	serpin family A member 5 [Source:HGNC Symbol;Acc:HGNC:8723]
LOX	lysyl oxidase [Source:HGNC Symbol;Acc:HGNC:6664]
NID1	nidogen 1 [Source:HGNC Symbol;Acc:HGNC:7821]
LGALS8	galectin 8 [Source:HGNC Symbol;Acc:HGNC:6569]
LGALS9	galectin 9 [Source:HGNC Symbol;Acc:HGNC:6570]
MBL2	mannose binding lectin 2 [Source:HGNC Symbol;Acc:HGNC:6922]
SPP1	secreted phosphoprotein 1 [Source:HGNC Symbol;Acc:HGNC:11255]
LRP1	LDL receptor related protein 1 [Source:HGNC Symbol;Acc:HGNC:6692]
ORM1	orosomucoid 1 [Source:HGNC Symbol;Acc:HGNC:8498]
C1QTNF9	C1q and tumor necrosis factor related protein 9 [Source:HGNC Symbol;Acc:HGNC:28732]
ALB	albumin [Source:HGNC Symbol;Acc:HGNC:399]
APOA1	apolipoprotein A1 [Source:HGNC Symbol;Acc:HGNC:600]
COLEC12	collectin subfamily member 12 [Source:HGNC Symbol;Acc:HGNC:16016]
APOA2	apolipoprotein A2 [Source:HGNC Symbol;Acc:HGNC:601]
ALDH2	aldehyde dehydrogenase 2 family (mitochondrial) [Source:HGNC Symbol;Acc:HGNC:404]
MATN2	matrilin 2 [Source:HGNC Symbol;Acc:HGNC:6908]
PCOLCE	procollagen C-endopeptidase enhancer [Source:HGNC Symbol;Acc:HGNC:8738]

APOC1	apolipoprotein C1 [Source:HGNC Symbol;Acc:HGNC:607]
SERPINC1	serpin family C member 1 [Source:HGNC Symbol;Acc:HGNC:775]
PTX3	pentraxin 3 [Source:HGNC Symbol;Acc:HGNC:9692]
OSM	oncostatin M [Source:HGNC Symbol;Acc:HGNC:8506]
UBC	ubiquitin C [Source:HGNC Symbol;Acc:HGNC:12468]
JUN	Jun proto-oncogene, AP-1 transcription factor subunit [Source:HGNC Symbol;Acc:HGNC:6204]
EGFR	epidermal growth factor receptor [Source:HGNC Symbol;Acc:HGNC:3236]
AUTS2	autism susceptibility candidate 2 [Source:HGNC Symbol;Acc:HGNC:14262]
XPO1	exportin 1 [Source:HGNC Symbol;Acc:HGNC:12825]
CTNNB1	catenin beta 1 [Source:HGNC Symbol;Acc:HGNC:2514]
PPP3CA	protein phosphatase 3 catalytic subunit alpha [Source:HGNC Symbol;Acc:HGNC:9314]
SRPK1	SRSF protein kinase 1 [Source:HGNC Symbol;Acc:HGNC:11305]
KCNIP4	potassium voltage-gated channel interacting protein 4 [Source:HGNC Symbol;Acc:HGNC:30083]
PIK3R1	phosphoinositide-3-kinase regulatory subunit 1 [Source:HGNC Symbol;Acc:HGNC:8979]
RDX	radixin [Source:HGNC Symbol;Acc:HGNC:9944]
HNRNPD	heterogeneous nuclear ribonucleoprotein D [Source:HGNC Symbol;Acc:HGNC:5036]
NTRK2	neurotrophic receptor tyrosine kinase 2 [Source:HGNC Symbol;Acc:HGNC:8032]
MUC1	mucin 1, cell surface associated [Source:HGNC Symbol;Acc:HGNC:7508]
DLG2	discs large MAGUK scaffold protein 2 [Source:HGNC Symbol;Acc:HGNC:2901]
CAND1	cullin associated and neddylation dissociated 1 [Source:HGNC Symbol;Acc:HGNC:30688]
DAPK1	death associated protein kinase 1 [Source:HGNC Symbol;Acc:HGNC:2674]
RFC3	replication factor C subunit 3 [Source:HGNC Symbol;Acc:HGNC:9971]
DAB1	DAB1, reelin adaptor protein [Source:HGNC Symbol;Acc:HGNC:2661]
PIWIL1	piwi like RNA-mediated gene silencing 1 [Source:HGNC Symbol;Acc:HGNC:9007]
PRL	prolactin [Source:HGNC Symbol;Acc:HGNC:9445]
FOXP1	forkhead box P1 [Source:HGNC Symbol;Acc:HGNC:3823]
UQCRFS1	ubiquinol-cytochrome c reductase, Rieske iron-sulfur polypeptide 1 [Source:HGNC Symbol;Acc:HGNC:12587]
PARK2	parkin RBR E3 ubiquitin protein ligase [Source:HGNC Symbol;Acc:HGNC:8607]
TCF4	transcription factor 4 [Source:HGNC Symbol;Acc:HGNC:11634]
PBX1	PBX homeobox 1 [Source:HGNC Symbol;Acc:HGNC:8632]
PRAMEF14	PRAME family member 14 [Source:HGNC Symbol;Acc:HGNC:13576]
PRAMEF1	PRAME family member 1 [Source:HGNC Symbol;Acc:HGNC:28840]
SPDYE3	speedy/RINGO cell cycle regulator family member E3 [Source:HGNC Symbol;Acc:HGNC:35462]
SPDYE1	speedy/RINGO cell cycle regulator family member E1 [Source:HGNC Symbol;Acc:HGNC:16408]
SPDYE2	speedy/RINGO cell cycle regulator family member E2 [Source:HGNC Symbol;Acc:HGNC:33841]

KRTAP5-2	keratin associated protein 5-2 [Source:HGNC Symbol;Acc:HGNC:23597]
OR1J1	olfactory receptor family 1 subfamily J member 1 [Source:HGNC Symbol;Acc:HGNC:8208]
OR8B2	olfactory receptor family 8 subfamily B member 2 [Source:HGNC Symbol;Acc:HGNC:8471]
OR4D5	olfactory receptor family 4 subfamily D member 5 [Source:HGNC Symbol;Acc:HGNC:14852]
OR4X1	olfactory receptor family 4 subfamily X member 1 (gene/pseudogene) [Source:HGNC Symbol;Acc:HGNC:14854]
OR5B3	olfactory receptor family 5 subfamily B member 3 [Source:HGNC Symbol;Acc:HGNC:8324]
OR2Y1	olfactory receptor family 2 subfamily Y member 1 [Source:HGNC Symbol;Acc:HGNC:14837]
KRTAP2-1	keratin associated protein 2-1 [Source:HGNC Symbol;Acc:HGNC:16775]
OR5AN1	olfactory receptor family 5 subfamily AN member 1 [Source:HGNC Symbol;Acc:HGNC:15255]
OR4Q3	olfactory receptor family 4 subfamily Q member 3 [Source:HGNC Symbol;Acc:HGNC:15426]
OR10P1	olfactory receptor family 10 subfamily P member 1 [Source:HGNC Symbol;Acc:HGNC:15378]
OR8B8	olfactory receptor family 8 subfamily B member 8 [Source:HGNC Symbol;Acc:HGNC:8477]
OR51B4	olfactory receptor family 51 subfamily B member 4 [Source:HGNC Symbol;Acc:HGNC:14708]
OR10J1	olfactory receptor family 10 subfamily J member 1 [Source:HGNC Symbol;Acc:HGNC:8175]
CT45A5	cancer/testis antigen family 45, member A5 [Source:HGNC Symbol;Acc:HGNC:33270]
FRG2C	FSHD region gene 2 family member C [Source:HGNC Symbol;Acc:HGNC:33626]
FRG2B	FSHD region gene 2 family member B [Source:HGNC Symbol;Acc:HGNC:33518]
PLXDC2	plexin domain containing 2 [Source:HGNC Symbol;Acc:HGNC:21013]
IQGAP2	IQ motif containing GTPase activating protein 2 [Source:HGNC Symbol;Acc:HGNC:6111]
BMP4	bone morphogenetic protein 4 [Source:HGNC Symbol;Acc:HGNC:1071]
FMOD	fibromodulin [Source:HGNC Symbol;Acc:HGNC:3774]
DRD5	dopamine receptor D5 [Source:HGNC Symbol;Acc:HGNC:3026]
CDH3	cadherin 3 [Source:HGNC Symbol;Acc:HGNC:1762]
UBB	ubiquitin B [Source:HGNC Symbol;Acc:HGNC:12463]
TCF7L1	transcription factor 7 like 1 [Source:HGNC Symbol;Acc:HGNC:11640]
YWHAE	tyrosine 3-monooxygenase/tryptophan 5-monooxygenase activation protein epsilon [Source:HGNC Symbol;Acc:HGNC:12851]
MAPK3	mitogen-activated protein kinase 3 [Source:HGNC Symbol;Acc:HGNC:6877]
NFKB1	nuclear factor kappa B subunit 1 [Source:HGNC Symbol;Acc:HGNC:7794]
PPARG	peroxisome proliferator activated receptor gamma [Source:HGNC Symbol;Acc:HGNC:9236]
PRKCQ	protein kinase C theta [Source:HGNC Symbol;Acc:HGNC:9410]
TMSB4X	thymosin beta 4, X-linked [Source:HGNC Symbol;Acc:HGNC:11881]
CLIC1	chloride intracellular channel 1 [Source:HGNC Symbol;Acc:HGNC:2062]

VEGFC	vascular endothelial growth factor C [Source:HGNC Symbol;Acc:HGNC:12682]
KRTAP4-1	keratin associated protein 4-1 [Source:HGNC Symbol;Acc:HGNC:18907]
WT1	Wilms tumor 1 [Source:HGNC Symbol;Acc:HGNC:12796]
ENO2	enolase 2 [Source:HGNC Symbol;Acc:HGNC:3353]
RAC1	ras-related C3 botulinum toxin substrate 1 (rho family, small GTP binding protein Rac1) [Source:HGNC Symbol;Acc:HGNC:9801]
RPE	ribulose-5-phosphate-3-epimerase [Source:HGNC Symbol;Acc:HGNC:10293]
PLA2G2D	phospholipase A2 group IID [Source:HGNC Symbol;Acc:HGNC:9033]
DEFA5	defensin alpha 5 [Source:HGNC Symbol;Acc:HGNC:2764]
UVRAG	UV radiation resistance associated [Source:HGNC Symbol;Acc:HGNC:12640]
ADCY6	adenylate cyclase 6 [Source:HGNC Symbol;Acc:HGNC:237]
UQCRH	ubiquinol-cytochrome c reductase hinge protein [Source:HGNC Symbol;Acc:HGNC:12590]
ITPKC	inositol-trisphosphate 3-kinase C [Source:HGNC Symbol;Acc:HGNC:14897]
FOXF2	forkhead box F2 [Source:HGNC Symbol;Acc:HGNC:3810]
CLDN10	claudin 10 [Source:HGNC Symbol;Acc:HGNC:2033]
TUBA1A	tubulin alpha 1a [Source:HGNC Symbol;Acc:HGNC:20766]
EDAR	ectodysplasin A receptor [Source:HGNC Symbol;Acc:HGNC:2895]
PLD2	phospholipase D2 [Source:HGNC Symbol;Acc:HGNC:9068]
ATP12A	ATPase H+/K+ transporting non-gastric alpha2 subunit [Source:HGNC Symbol;Acc:HGNC:13816]
TINAGL1	tubulointerstitial nephritis antigen like 1 [Source:HGNC Symbol;Acc:HGNC:19168]
CLDN34	claudin 34 [Source:HGNC Symbol;Acc:HGNC:51259]

Supplementary Table S9: The top genes were selected from various centralities.					
**Degree unDir**	**Bridging unDir**	**Closeness unDir**	**EigenVector unDir**	**Stress unDir**	**TopologicalCoefficient**
FN1	SPDYE2	FRG2B	FN1	UBC	SPDYE3
COL1A2	DEFA1	FRG2C	SERPING1	FN1	SPDYE1
COL4A1	PITX3	FN1	COL18A1	HABP2	DEFA1B
PLG	DEFA5	SERPING1	CTSK	DAB1	PRAMEF14
COL1A1	KRTAP5-2	UBC	C1S	MUC1	C4B_2
**Betweenness unDir**	**Centroid unDir**	**Eccentricity unDir**	**Radiality unDir**	**ClusteringCoefficient**	**NeighborhoodConnectivity**
UBC	CLDN34	FRG2B	FN1	PRAMEF14	BMP1
FN1	FRG2B	FRG2C	SERPING1	DEFA1B	P3H3
SPDYE2	FRG2C		COL1A2	SPDYE3	PDGFA
HABP2			UBC	SPDYE1	LUM
MUC1			CTSK	C4B_2	COL14A1

Supplementary Table S10: The final gene list that contained 31 essential genes.	
FN1	fibronectin 1 [Source:HGNC Symbol;Acc:HGNC:3778]
COL1A2	collagen type I alpha 2 chain [Source:HGNC Symbol;Acc:HGNC:2198]
COL4A1	collagen type IV alpha 1 chain [Source:HGNC Symbol;Acc:HGNC:2202]
PLG	plasminogen [Source:HGNC Symbol;Acc:HGNC:9071]
COL1A1	collagen type I alpha 1 [Source:HGNC Symbol;Acc:HGNC:2197]
UBC	ubiquitin C [Source:HGNC Symbol;Acc:HGNC:12468]
SPDYE2	speedy/RINGO cell cycle regulator family member E2 [Source:HGNC Symbol;Acc:HGNC:33841]
HABP2	hyaluronan binding protein 2 [Source:HGNC Symbol;Acc:HGNC:4798]
MUC1	mucin 1, cell surface associated [Source:HGNC Symbol;Acc:HGNC:7508]
DEFA1	defensin alpha 1 [Source:HGNC Symbol;Acc:HGNC:2761]
PITX3	paired like homeodomain 3 [Source:HGNC Symbol;Acc:HGNC:9006]
DEFA5	defensin alpha 5 [Source:HGNC Symbol;Acc:HGNC:2764]
KRTAP5-2	keratin associated protein 5-2 [Source:HGNC Symbol;Acc:HGNC:23597]
CLDN34	claudin 34 [Source:HGNC Symbol;Acc:HGNC:51259]
FRG2B	FSHD region gene 2 family member B [Source:HGNC Symbol;Acc:HGNC:33518]
FRG2C	FSHD region gene 2 family member C [Source:HGNC Symbol;Acc:HGNC:33626]
SERPING1	serpin family G member 1 [Source:HGNC Symbol;Acc:HGNC:1228]
COL18A1	collagen type XVIII alpha 1 chain [Source:HGNC Symbol;Acc:HGNC:2195]
CTSK	cathepsin K [Source:HGNC Symbol;Acc:HGNC:2536]
C1S	complement component 1, s subcomponent [Source:HGNC Symbol;Acc:HGNC:1247]
DAB1	DAB1, reelin adaptor protein [Source:HGNC Symbol;Acc:HGNC:2661]
PRAMEF14	PRAME family member 14 [Source:HGNC Symbol;Acc:HGNC:13576]
DEFA1B	defensin alpha 1B [Source:HGNC Symbol;Acc:HGNC:33596]
SPDYE3	speedy/RINGO cell cycle regulator family member E3 [Source:HGNC Symbol;Acc:HGNC:35462]
SPDYE1	speedy/RINGO cell cycle regulator family member E1 [Source:HGNC Symbol;Acc:HGNC:16408]
C4B_2	complement component 4B (Chido blood group), copy 2 [Source:HGNC Symbol;Acc:HGNC:42398]
BMP1	bone morphogenetic protein 1 [Source:HGNC Symbol;Acc:HGNC:1067]
P3H3	prolyl 3-hydroxylase 3 [Source:HGNC Symbol;Acc:HGNC:19318]
PDGFA	platelet derived growth factor subunit A [Source:HGNC Symbol;Acc:HGNC:8799]
LUM	lumican [Source:HGNC Symbol;Acc:HGNC:6724]
COL14A1	collagen type XIV alpha 1 chain [Source:HGNC Symbol;Acc:HGNC:2191]

Supplementary Table S11: The final nAMD-related SNPs		
rs10490924/T	rs2070296	rs243865
rs10737680/A	rs4910623	rs2287074
rs429608/G	rs12366035	rs3918241
rs2230199/C	rs55732851	rs3918242
rs5749482/G	rs1800775	rs4810482
rs4420638/A	rs12603486	rs17576
rs1864163/G	rs13900	rs17577
rs943080/T	rs323085	rs1999930
rs13278062/T	rs7412	rs493258
rs13081855/T	rs148553336	rs11755724
rs8017304/A	rs187328863	rs509859
rs4698775/G	rs61818925	rs12637095
rs920915/C	rs35292876	rs13095226
rs334353/T	rs191281603	rs3748391
rs8135665/T	rs141853578	rs3764261
rs3130783/A	rs570618	rs12678919
rs6795735/T	rs10922109	rs3025033
rs3812111/T	rs62247658	rs1570360A
rs9542236/C	rs943080	rs699947A
rs10801555	rs2043085	rs3025033G
10:124213143:CAA:C	rs11884770	rs2146323A
rs11120691	rs114092250	rs1048661
rs259842	rs7803454	rs1049331
rs2011092	rs1142	rs2293870
rs1005819	rs12357257	rs9973159
rs17421410	rs3138141	rs200437673
rs6899205	rs61941274	rs151214675
rs7896471	rs72802342	rs140250387
rs1926564	rs11080055	rs115333865
rs1378940	rs6565597	rs570523689
rs3825991	rs67538026	rs1329424
rs2232613	rs3750846	rs13278062
rs7405453	rs62358361	rs17073641
rs138650617	rs116503776	rs10490924
rs7678123	rs9564692	rs800292
rs10036789	rs429358	rs1329428
rs7744813	rs8135665	rs1883025
rs9667489	rs10033900	rs3025039
rs12570944	rs4151667	rs3025000
rs2738265	rs140647181	rs10158937

rs62181740	rs5754227	rs12138564
rs10887262	rs5817082	rs3753394
rs12950511	rs2230199	rs1065489
rs113941606	rs2284665	rs17793056
2:146913702:CCTCT:C	rs147859257	rs6828477
rs36090025	rs34882957	rs4576072
rs13118211	rs641153	rs2071559
1:164194417:TA:T	rs17231506	rs4073
rs9330814	rs1626340	rs699946
rs112115087	rs61985136	rs699947
rs10824539	rs71507014	rs2069845
rs41393947	rs10781182	rs25681
rs12193446	rs201459901	rs181705462
rs3138142	rs79037040	rs144629244
rs7184522	rs2740488	rs114254831
rs113721756	rs142450006	rs704
rs111260777	rs429608	rs760070
rs117468955	rs1061170	rs10922153
rs4790395	rs11080055	rs3812111
rs117115124	rs1706902	rs572515
rs78606172	rs117886541	rs10737680
rs200404865	rs12520223	rs7690921
rs61820755	rs78996920	rs522162
rs149369377	rs150308036	rs174547
rs4085749	rs8064132	rs471364
rs70620	rs73036519	rs2967605
rs12047098	rs1837253	rs4939883
rs72732232	rs848	rs2338104
rs79351096	rs911263	rs964184
rs16840522	rs6677089	rs7679
rs34538561	rs226251	rs1800961
rs11711512	rs143255652	rs4846914
rs7439493	rs143606602	rs2736911
rs1017242313	rs4988183	rs577360216
rs752163277	rs374945368	rs6075340
rs587779635	rs1358927744	rs4702
rs1536304	rs561508452	rs2043085
rs2414577	rs116216433	rs4243218
rs8067305	rs760342906	rs5756908
rs34559944	rs34811207	rs7398705
rs7289865	rs1555106201	rs241777

rs79069165	rs59690758	rs157580
rs74320127	rs669953	rs2070780
rs56072732	rs10740759	rs6945359
rs58978565	rs56366109	rs35064805
rs28368872	rs1733807	rs4790874
rs121913059	rs12145913	rs8076897
rs10468017	rs55722898	rs16851585
rs547154	rs375015898	rs1967689
rs11200638	rs1375561443	rs6721654
rs1410996	rs61251108	rs17586843
rs1410966	rs201405032	rs7750345
rs915370426	rs6707761	rs2049622
rs1047286	rs865775509	rs11986011
rs117793540	rs77968014	rs6480975
rs2230210	rs11402250	rs3793917
rs2230205	rs12019136	rs4293143
rs2250656	rs67538023	rs621313
rs9332739	rs9708919	rs9646096
rs541862	rs3750864	rs10406174
rs3826945	rs4351242	rs6857
rs713685	rs17855739	rs2075650
rs743751	rs204993	rs380390
rs5749482	rs8017304	rs1713985
rs9621532	rs10422209	rs2070895
rs8179090	rs9380272	

Supplementary Table S12: The final nAMD-related metabolites		
L-Alanine	Urea	3-Hydroxybutyric acid
L-Arginine	Acetoacetic acid	D-Glucose
L-Aspartic acid	Dimethyl sulfone	Wax ester
Glycine	N-Acetyl-L-alanine	Glutathione
L-Histidine	N1-Methyl-2-pyridone-5-carboxamide	Dopaquinone
L-Isoleucine	Isomaltose	Phytosphingosine
L-Leucine	Hydrocortisone	Zinc
L-Lysine	Biliverdin	Calcium
L-Methionine	Adenosine	Iron
L-Phenylalanine	DG(14:0/14:0/0:0)	Prostaglandin G2
L-Tyrosine	1-Lyso-2-arachidonoyl-phosphatidate	Arachidonic acid
L-Valine	9-Hexadecenoylcarnitine	L-Serine
Glycerophosphocholine	Heptadecanoyl carnitine	L-Proline
1-Acyl-sn-glycerol 3-phosphate	11Z-Octadecenylcarnitine	Docosahexaenoic acid
LysoPC(0:0/18:0)	Stearoylcarnitine	L-Cystine
2-Acetyl-1-alkyl-sn-glycero-3-phosphocholine	N-Ornithyl-L-taurine	N-Acetyl-L-phenylalanine
Phosphatidylserine	Serotonin	L-Cysteine
SM(d18:1/18:0)	N-Acetyl-L-methionine	N-acetyltryptophan
Glycocholic acid	Hypotaurine	Deoxycholic acid glycine conjugate
Deoxycholic acid glycine conjugate	N-Acetylasparagine	Cholesterol
Glycoursodeoxycholic acid	S-Adenosylhomocysteine	Isopentyl beta-D-glucoside
12-HETE	Maltotriose	LysoPC(P-18:0)
L-Lactic acid	Maltose	LysoPC(P-18:1(9Z))
Acetic acid	Guanine	LysoPC(16:1(9Z))
L-Carnitine	Isoleucyl-Glycine	Darunavir
Creatine	Lipoprotein	Bepridil
Cytidine	L-Glutamine	Corchoroside A
Hypoxanthine	p-Hydroxyphenylacetic acid	Oleoylcarnitine
Inosine	Formic acid	Glycerophosphocholine
Adenine	Sucrose	Sphingosine
Betaine	cis-Aconitic acid	L-Cysteine
Niacinamide	Valerylcarnitine	L-Cystine
L-Palmitoylcarnitine	Citric acid	LysoPC(0:0/18:0)
Taurine	4-Trimethylammoniobutanoic acid	Glycerol
Cytidine monophosphate	Mesaconic acid	L-Carnitine
Pyruvic acid	Streptomycin	17-HDoHE
L-Glutamic acid	3-Deoxyarabinohexonic acid	Sphinganine
L-Tryptophan	Oleic acid	Purine
Taurine		

Supplementary Table S13: A list of 30 critical SNPs was created by merging results		
rs17576	rs174547	rs3918242
rs1061170	rs2071559	rs800292
rs699947	rs1800961	rs3025039
rs429358	rs6857	rs7412
rs2043085	rs1837253	rs2070895
rs3764261	rs114254831	rs1800775
rs4073	rs116503776	rs17577
rs243865	rs2740488	rs1065489
rs964184	rs12678919	rs10468017
rs2075650	rs7679	rs17231506

Supplementary Table S14: A total of 33 crucial pathways were chosen based on FDR <0.05			
Pathway	FDR	pathway	FDR
ABC transporters	4.34E-27	Purine metabolism	0.010746
Protein digestion and absorption	5.17E-26	Pantothenate and CoA biosynthesis	0.010746
Central carbon metabolism in cancer	5.26E-20	Thiamine metabolism	0.010746
Aminoacyl-tRNA biosynthesis	3.07E-16	Arginine and proline metabolism	0.01168
Mineral absorption	7.81E-13	Staphylococcus aureus infection	0.021438
Glyoxylate and dicarboxylate metabolism	2.62E-07	Gap junction	0.021454
Taurine and hypotaurine metabolism	4.19E-07	Neuroactive ligand-receptor interaction	0.023787
Cysteine and methionine metabolism	1.68E-05	Primary bile acid biosynthesis	0.027448
Glycine, serine and threonine metabolism	3.15E-05	AGE-RAGE signaling pathway in diabetic complications	0.029015
Alanine, aspartate and glutamate metabolism	0.000523	Tyrosine metabolism	0.032855
Ferroptosis	0.000803	Butanoate metabolism	0.032855
Sulfur metabolism	0.000803	Pyruvate metabolism	0.032855
Arginine biosynthesis	0.000831	Taste transduction	0.032961
Amoebiasis	0.001224	Nitrogen metabolism	0.036419
Valine, leucine and isoleucine biosynthesis	0.001604	Carbohydrate digestion and absorption	0.036419
Sphingolipid metabolism	0.007217	Phenylalanine, tyrosine and tryptophan biosynthesis	0.046353
Phenylalanine metabolism	0.009279		

Supplementary Table S17: the results of two distinct networks were reconstructed to evaluate the multiple centralities of every metabolite.				
	Metabolite-metabolite interaction network		Metabolite-gene-disease interaction network	
	Degree	Betweenness	Degree	Betweenness
Maltotriose	19	984.14	nd	nd
L-Glutamic acid	306	68219.62	5	413.99
Pyruvic acid	249	42673.65	15	845.51
L-Tryptophan	95	8225.5	6	1044.12
Calcium	nd	nd	18	3031.95
Citric acid	144	13038.04	10	1305.28
L-Alanine	126	8628.74	12	893.03
L-Serine	118	6488.68	7	430.09
Betaine	50	2091.47	5	459.91
Dimethyl sulfone	7	15.04	nd	nd
L-Arginine	119	10059.28	16	1398.93
Sphinganine	22	1201.34	nd	nd
L-Glutamine	119	5780.66	6	456.29
L-Tyrosine	80	5265.51	5	359.72
1-Acyl-sn-glycerol 3-phosphate	nd	nd	nd	nd
Cholesterol sulfate	1	0	23	4525.31
Acetoacetic acid	nd	nd	7	615.17
Formic acid	nd	nd	1	0
Sucrose	57	4954.12	1	0
L-Phenylalanine	90	3770.24	6	872.92
L-Cysteine	132	10977.63	5	768.43
L-Aspartate-semialdehyde	13	3.87	nd	nd
Fe3+	nd	nd	nd	nd
Formate	nd	nd	nd	nd
Pyruvate	nd	nd	nd	nd
L-Methionine	107	5789.4	10	1984.29
Creatine	32	1294.85	5	668.71
L-Cystine	2	0	7	393.36
L-Lysine	129	10419.41	15	1406.1
L-Isoleucine	64	755.32	7	443.17
Phytosphingosine	11	398.48	nd	nd
N-Acetyl-L-phenylalanine	nd	nd	nd	nd
Adenosine	111	9108.39	3	267.53
L-Valine	76	1550	8	656.72
2-Acetyl-1-alkyl-sn-glycero-3-phosphocholine	nd	nd	nd	nd
Glycine	156	16594.88	20	2469.18
4-Hydroxyphenylacetate	nd	nd	nd	nd

Acetoacetate	nd	nd	nd	nd
L-Leucine	90	2991.38	8	762.72
Acetate	nd	nd	nd	nd
Zinc (II) ion	nd	nd	4	239.63
cis-Aconitic acid	39	1424.21	3	119.06
Hypotaurine	10	27.43	nd	nd
S-Adenosylhomocysteine	183	43943.55	2	47.9
L-Aspartic acid	115	8786.02	3	126.28
Glutathione	95	8058.05	4	818.93
Acetic acid	nd	nd	3	120.52
Arachidonic acid	73	12822.34	2	263.8
Adenine	96	9588.04	nd	nd
Urea	49	4557.1	5	112.89
Serotonin	108	17274.62	4	593.14
Cortisol	nd	nd	23	6286.31
Taurine	49	3658.42	5	743
3R-hydroxy-butanoic acid	nd	nd	nd	nd
L-Lactic acid	32	1020.11	55	9526.37
Sphingosine	nd	nd	nd	nd
p-Hydroxyphenylacetic acid	9	36.63	3	334.45
Inosine	49	1656.88	3	283.06
Glycocholic acid	nd	nd	nd	nd
SM(d18:1/18:0)	13	1096.7	nd	nd
Hypoxanthine	56	2240.9	5	1032.66
Dopaquinone	6	17.92	nd	nd
L-Proline	68	3215.12	11	1004.54
L-Histidine	74	2692.98	7	460.73
Guanine	53	1765.56	1	0
2-Methylfumarate	nd	nd	nd	nd
4-Trimethylammoniobutanoate	nd	nd	nd	nd
Cytidine	43	2274.23	1	0
Lipoprotein	nd	nd	nd	nd
Glycerol	320	76408.57	6	1613.74

Supplementary Table S18: the normalized results within the metabolite-metabolite interaction network (MMIN)						
Metabolites	Degree	MAX Degree	Normalized Weight 1	Betweenness	MAX Betweenness	Normalized Weight 2
Maltotriose	19	320	0.059375	984.14	76408.57	0.012879969
L-Glutamic acid	306	320	0.95625	68219.62	76408.57	0.892826812
Pyruvic acid	249	320	0.778125	42673.65	76408.57	0.558492981
L-Tryptophan	95	320	0.296875	8225.5	76408.57	0.107651537
Citric acid	144	320	0.45	13038.04	76408.57	0.170635833
L-Alanine	126	320	0.39375	8628.74	76408.57	0.112928955
L-Serine	118	320	0.36875	6488.68	76408.57	0.084920841
Betaine	50	320	0.15625	2091.47	76408.57	0.027372191
Dimethyl sulfone	7	320	0.021875	15.04	76408.57	0.000196837
L-Arginine	119	320	0.371875	10059.28	76408.57	0.131651201
Sphinganine	22	320	0.06875	1201.34	76408.57	0.015722582
L-Glutamine	119	320	0.371875	5780.66	76408.57	0.075654603
L-Tyrosine	80	320	0.25	5265.51	76408.57	0.068912558
Cholesterol sulfate	1	320	0.003125	0	76408.57	0
Sucrose	57	320	0.178125	4954.12	76408.57	0.06483723
L-Phenylalanine	90	320	0.28125	3770.24	76408.57	0.049343156
L-Cysteine	132	320	0.4125	10977.63	76408.57	0.143670141
L-Aspartate-semialdehyde	13	320	0.040625	3.87	76408.57	5.06488E-05
L-Methionine	107	320	0.334375	5789.4	76408.57	0.075768988
Creatine	32	320	0.1	1294.85	76408.57	0.016946398
L-Cystine	2	320	0.00625	0	76408.57	0
L-Lysine	129	320	0.403125	10419.41	76408.57	0.136364416
L-Isoleucine	64	320	0.2	755.32	76408.57	0.009885279
Phytosphingosine	11	320	0.034375	398.48	76408.57	0.005215122
Adenosine	111	320	0.346875	9108.39	76408.57	0.119206393
L-Valine	76	320	0.2375	1550	76408.57	0.020285683
Glycine	156	320	0.4875	16594.88	76408.57	0.217186109
L-Leucine	90	320	0.28125	2991.38	76408.57	0.039149797
cis-Aconitic acid	39	320	0.121875	1424.21	76408.57	0.018639401
Hypotaurine	10	320	0.03125	27.43	76408.57	0.000358991
S-Adenosylhomocysteine	183	320	0.571875	43943.55	76408.57	0.575112844
L-Aspartic acid	115	320	0.359375	8786.02	76408.57	0.114987363
Glutathione	95	320	0.296875	8058.05	76408.57	0.105460029
Arachidonic acid	73	320	0.228125	12822.34	76408.57	0.167812851
Adenine	96	320	0.3	9588.04	76408.57	0.12548383
Urea	49	320	0.153125	4557.1	76408.57	0.059641216
Serotonin	108	320	0.3375	17274.62	76408.57	0.226082231
Taurine	49	320	0.153125	3658.42	76408.57	0.047879708
L-Lactic acid	32	320	0.1	1020.11	76408.57	0.013350728

p-Hydroxyphenylacetic acid	9	320	0.028125	36.63	76408.57	0.000479396
Inosine	49	320	0.153125	1656.88	76408.57	0.021684479
SM(d18:1/18:0)	13	320	0.040625	1096.7	76408.57	0.014353102
Hypoxanthine	56	320	0.175	2240.9	76408.57	0.029327862
Dopaquinone	6	320	0.01875	17.92	76408.57	0.000234529
L-Proline	68	320	0.2125	3215.12	76408.57	0.042078003
L-Histidine	74	320	0.23125	2692.98	76408.57	0.035244476
Guanine	53	320	0.165625	1765.56	76408.57	0.023106832
Cytidine	43	320	0.134375	2274.23	76408.57	0.02976407
Glycerol	320	320	1	76408.57	76408.57	1

Supplementary Table S19: the normalized results within the Metabolite-gene-disease interaction network (MGDIN)						
	Degree	MAX Degree	Normalized Weight 3	Betweenness	MAX Betweenness	Normalized Weight 4
L-Glutamic acid	5	55	0.090909091	413.99	9526.37	0.043457267
Pyruvic acid	15	55	0.272727273	845.51	9526.37	0.088754688
L-Tryptophan	6	55	0.109090909	1044.12	9526.37	0.109603133
Calcium	18	55	0.327272727	3031.95	9526.37	0.318269183
Citric acid	10	55	0.181818182	1305.28	9526.37	0.137017563
L-Alanine	12	55	0.218181818	893.03	9526.37	0.093742947
L-Serine	7	55	0.127272727	430.09	9526.37	0.045147312
Betaine	5	55	0.090909091	459.91	9526.37	0.048277571
L-Arginine	16	55	0.290909091	1398.93	9526.37	0.14684817
L-Glutamine	6	55	0.109090909	456.29	9526.37	0.047897573
L-Tyrosine	5	55	0.090909091	359.72	9526.37	0.037760448
Cholesterol sulfate	23	55	0.418181818	4525.31	9526.37	0.475029838
Acetoacetic acid	7	55	0.127272727	615.17	9526.37	0.064575489
Formic acid	1	55	0.018181818	0	9526.37	0
Sucrose	1	55	0.018181818	0	9526.37	0
L-Phenylalanine	6	55	0.109090909	872.92	9526.37	0.091631965
L-Cysteine	5	55	0.090909091	768.43	9526.37	0.080663464
L-Methionine	10	55	0.181818182	1984.29	9526.37	0.20829445
Creatine	5	55	0.090909091	668.71	9526.37	0.070195678
L-Cystine	7	55	0.127272727	393.36	9526.37	0.041291699
L-Lysine	15	55	0.272727273	1406.1	9526.37	0.147600818
L-Isoleucine	7	55	0.127272727	443.17	9526.37	0.046520343
Adenosine	3	55	0.054545455	267.53	9526.37	0.0280831
L-Valine	8	55	0.145454545	656.72	9526.37	0.068937066
Glycine	20	55	0.363636364	2469.18	9526.37	0.259194216
L-Leucine	8	55	0.145454545	762.72	9526.37	0.080064075
Zinc (II) ion	4	55	0.072727273	239.63	9526.37	0.025154387
cis-Aconitic acid	3	55	0.054545455	119.06	9526.37	0.01249794
S-Adenosylhomocysteine	2	55	0.036363636	47.9	9526.37	0.005028148
L-Aspartic acid	3	55	0.054545455	126.28	9526.37	0.013255836
Glutathione	4	55	0.072727273	818.93	9526.37	0.085964538
Acetic acid	3	55	0.054545455	120.52	9526.37	0.012651199
Arachidonic acid	2	55	0.036363636	263.8	9526.37	0.027691555
Urea	5	55	0.090909091	112.89	9526.37	0.011850264
Serotonin	4	55	0.072727273	593.14	9526.37	0.062262961
Cortisol	23	55	0.418181818	6286.31	9526.37	0.65988514
Taurine	5	55	0.090909091	743	9526.37	0.077994031
L-Lactic acid	55	55	1	9526.37	9526.37	1

p-Hydroxyphenylacetic acid	3	55	0.054545455	334.45	9526.37	0.035107811
Inosine	3	55	0.054545455	283.06	9526.37	0.029713312
Hypoxanthine	5	55	0.090909091	1032.66	9526.37	0.108400157
L-Proline	11	55	0.2	1004.54	9526.37	0.10544835
L-Histidine	7	55	0.127272727	460.73	9526.37	0.048363647
Guanine	1	55	0.018181818	0	9526.37	0
Cytidine	1	55	0.018181818	0	9526.37	0
Glycerol	6	55	0.109090909	1613.74	9526.37	0.169397158

Supplementary Table S20: 256 rule bases were established and decisions were made on the consequences				
Output				Final Decision
Normalized Weight 1	Normalized Weight 2	Normalized Weight 3	Normalized Weight 4	
w	w	w	w	w
w	w	w	m	w
w	w	w	g	m
w	w	w	e	m
w	w	m	w	w
w	w	m	m	m
w	w	m	g	g
w	w	m	e	g
w	w	g	w	m
w	w	g	m	g
w	w	g	g	g
w	w	g	e	g
w	w	e	w	m
w	w	e	m	g
w	w	e	g	g
w	w	e	e	e
w	m	w	w	w
w	m	w	m	w
w	m	w	g	m
w	m	w	e	m
w	m	m	w	w
w	m	m	m	m
w	m	m	g	g
w	m	m	e	g
w	m	g	w	m
w	m	g	m	m
w	m	g	g	g
w	m	g	e	g
w	m	e	w	m
w	m	e	m	g
w	m	e	g	g
w	m	e	e	e
w	g	w	w	w
w	g	w	m	m
w	g	w	g	g
w	g	w	e	m
w	g	m	w	m
w	g	m	m	m

w	g	m	g	g
w	g	m	e	g
w	g	g	w	g
w	g	g	m	m
w	g	g	g	g
w	g	g	e	g
w	g	e	w	m
w	g	e	m	g
w	g	e	g	g
w	g	e	e	e
w	e	w	w	w
w	e	w	m	w
w	e	w	g	m
w	e	w	e	g
w	e	m	w	w
w	e	m	m	m
w	e	m	g	m
w	e	m	e	g
w	e	g	w	m
w	e	g	m	m
w	e	g	g	g
w	e	g	e	g
w	e	e	w	g
w	e	e	m	g
w	e	e	g	g
w	e	e	e	e
m	w	w	w	w
m	w	w	m	m
m	w	w	g	m
m	w	w	e	m
m	w	m	w	m
m	w	m	m	m
m	w	m	g	m
m	w	m	e	g
m	w	g	w	m
m	w	g	m	m
m	w	g	g	g
m	w	g	e	g
m	w	e	w	m
m	w	e	m	g
m	w	e	g	g

m	w	e	e	e
m	m	w	w	w
m	m	w	m	m
m	m	w	g	m
m	m	w	e	m
m	m	m	w	m
m	m	m	m	m
m	m	m	g	m
m	m	m	e	g
m	m	g	w	m
m	m	g	m	g
m	m	g	g	g
m	m	g	e	g
m	m	e	w	m
m	m	e	m	g
m	m	e	g	g
m	m	e	e	e
m	g	w	w	w
m	g	w	m	m
m	g	w	g	g
m	g	w	e	m
m	g	m	w	m
m	g	m	m	m
m	g	m	g	g
m	g	m	e	g
m	g	g	w	m
m	g	g	m	g
m	g	g	g	g
m	g	g	e	g
m	g	e	w	m
m	g	e	m	g
m	g	e	g	g
m	g	e	e	e
m	e	w	w	w
m	e	w	m	m
m	e	w	g	m
m	e	w	e	g
m	e	m	w	m
m	e	m	m	m
m	e	m	g	g
m	e	m	e	g

m	e	g	w	m
m	e	g	m	m
m	e	g	g	g
m	e	g	e	e
m	e	e	w	g
m	e	e	m	g
m	e	e	g	e
m	e	e	e	e
g	w	w	w	w
g	w	w	m	w
g	w	w	g	w
g	w	w	e	m
g	w	m	w	w
g	w	m	m	m
g	w	m	g	m
g	w	m	e	g
g	w	g	w	m
g	w	g	m	g
g	w	g	g	g
g	w	g	e	g
g	w	e	w	m
g	w	e	m	g
g	w	e	g	g
g	w	e	e	e
g	m	w	w	w
g	m	w	m	w
g	m	w	g	w
g	m	w	e	m
g	m	m	w	m
g	m	m	m	m
g	m	m	g	m
g	m	m	e	g
g	m	g	w	m
g	m	g	m	m
g	m	g	g	g
g	m	g	e	g
g	m	e	w	m
g	m	e	m	g
g	m	e	g	g
g	m	e	e	e
g	g	w	w	w

g	g	w	m	w
g	g	w	g	m
g	g	w	e	m
g	g	m	w	m
g	g	m	m	m
g	g	m	g	g
g	g	m	e	g
g	g	g	w	g
g	g	g	m	g
g	g	g	g	g
g	g	g	e	g
g	g	e	w	m
g	g	e	m	g
g	g	e	g	g
g	g	e	e	e
g	e	w	w	w
g	e	w	m	m
g	e	w	g	m
g	e	w	e	g
g	e	m	w	m
g	e	m	m	m
g	e	m	g	m
g	e	m	e	g
g	e	g	w	m
g	e	g	m	m
g	e	g	g	g
g	e	g	e	e
g	e	e	w	g
g	e	e	m	g
g	e	e	g	e
g	e	e	e	e
e	w	w	w	w
e	w	w	m	w
e	w	w	g	m
e	w	w	e	m
e	w	m	w	w
e	w	m	m	m
e	w	m	g	m
e	w	m	e	g
e	w	g	w	m
e	w	g	m	m

e	w	g	g	g
e	w	g	e	g
e	w	e	w	m
e	w	e	m	g
e	w	e	g	g
e	w	e	e	e
e	m	w	w	w
e	m	w	m	w
e	m	w	g	w
e	m	w	e	m
e	m	m	w	m
e	m	m	m	m
e	m	m	g	m
e	m	m	e	g
e	m	g	w	m
e	m	g	m	m
e	m	g	g	g
e	m	g	e	g
e	m	e	w	g
e	m	e	m	g
e	m	e	g	g
e	m	e	e	e
e	g	w	w	w
e	g	w	m	m
e	g	w	g	m
e	g	w	e	g
e	g	m	w	m
e	g	m	m	m
e	g	m	g	g
e	g	m	e	g
e	g	g	w	m
e	g	g	m	g
e	g	g	g	g
e	g	g	e	g
e	g	e	w	g
e	g	e	m	g
e	g	e	g	e
e	g	e	e	e
e	e	w	w	w
e	e	w	m	m
e	e	w	g	g

e	e	w	e	g
e	e	m	w	m
e	e	m	m	m
e	e	m	g	g
e	e	m	e	g
e	e	g	w	g
e	e	g	m	g
e	e	g	g	g
e	e	g	e	e
e	e	e	w	g
e	e	e	m	g
e	e	e	g	e
e	e	e	e	e

Supplementary Table S22: a total of 52 rule bases were established and decisions were made on their consequences				
Degree Impact (DI)	betweenness Impact (BI)	Closeness Impact (CI)	FDR	**FINAL**
D	D	D	D	D
D	D	D	C	D
D	D	D	B	C
D	D	D	A	B
D	D	C	D	D
D	D	B	D	C
D	D	B	B	C
D	D	A	D	B
D	D	A	A	A
D	B	D	D	C
D	B	D	B	C
D	B	B	D	C
D	B	B	B	B
D	A	D	D	B
D	A	D	A	A
D	A	A	D	A
D	A	A	A	A
C	D	D	D	D
C	D	C	C	C
C	B	C	C	C
C	B	B	B	B
C	A	C	C	B
C	A	C	A	A
C	A	A	C	A
C	A	A	A	A
B	D	D	D	C
B	D	D	B	C
B	D	B	D	C
B	B	D	D	C
B	B	D	B	B
B	B	C	B	B
B	B	B	D	B
B	B	B	C	B
B	B	B	B	B
B	B	B	A	B
B	B	A	B	B
B	A	B	B	B
B	A	A	A	A

A	D	D	D	B
A	D	D	A	A
A	D	A	D	A
A	D	A	A	A
A	B	B	B	B
A	B	A	A	A
A	A	D	D	A
A	A	D	A	A
A	A	C	C	A
A	A	C	A	A
A	A	A	D	A
A	A	A	C	A
A	A	A	B	A
A	A	A	A	A
